# Simultaneous Presentation of Multiple Myeloma and Lung Cancer: Case Report and Gene Bioinformatics Analysis

**DOI:** 10.3389/fonc.2022.859735

**Published:** 2022-06-13

**Authors:** Ping-Ping Xiao, Bing-Qing Luo, Wei Fan, Xu-Yan Chen, Zhi-Gao Dong, Jin-Mei Huang, Yi Zhang, Yong-Quan Chen

**Affiliations:** ^1^Department of Hematology and Rheumatology, The Second Affiliated Hospital of Xiamen Medical College, Xiamen, China; ^2^Department of Respiratory Oncology, The Second Affiliated Hospital of Xiamen Medical College, Xiamen, China

**Keywords:** multiple myeloma, lung cancer, multiple primary cancer, bioinformatics analysis, mitochondrial trans-2-enoyl-CoA reductase, survival

## Abstract

Patients diagnosed with more than one cancer generally develop the individual tumors sequentially. There are a few cases of co-occurring multiple myeloma and lung cancer reported in the literature. Here, we report two cases of co-occurring multiple myeloma and lung cancer in patients who presented with the chief complaint of pain. The diagnoses of multiple myeloma and lung cancer were supported by hematologic and biochemical investigations, as well as bone marrow and lung histopathologic examination. We provided suitable interventions for both two patients. The patients are still currently undergoing treatment and followed up closely. We first performed a bioinformatic analysis to determine commonly shared genes and pathways in the two types of cancer types. Fortunately, we identified the hub gene mitochondrial trans-2-enoyl-CoA reductase (MECR), which was overexpressed in both tumors. Survival analysis correlated higher MECR expression with poorer overall survival. Signaling pathway analysis suggested possible transduction pathways implicated in the co-occurrence of both tumors. The clinical cases combined with bioinformatic analysis may provide insight for the pathogenesis of synchronous tumors.

## Introduction

Multiple primary cancer (MPC) refer to two or more malignant tumors that occur simultaneously and independently of each other. They may co-exist within the same or in different organs. MPCs can be further classified based on the duration between the tumor diagnoses such that MPCs are termed ‘synchronous’ when diagnoses are made within 6 months of each other and ’metachronous’ when diagnosis are made over a span of more than 6 months (1). Tumors in patients with MPC most commonly arise sequentially, separated by months or even years. The mechanisms behind the development of a second cancer in a patient with an existing primary cancer are not entirely understood, but specific antitumor drugs may play a contributing role in the pathoenesis of MPC.

Although multiple myeloma (MM) is the second most common hematologic tumor, its prevalence in the general population is far less common than that of lung cancer, the most commonly diagnosed malignancy in patients with MPC. Patients receiving immunomodulators like lenalidomide for the treatment of MM have an increased risk of developing additional solid tumors (2). Patients who have never received treatment for MM, however, are rarely diagnosed with simultaneously occurring lung cancer. In this report, we present two cases of simultaneous MM and lung cancer diagnoses and perform bioinformatic analyses to propose mechanisms for cancer co-occurrence and identify novel therapeutic targets.

## Case Presentation

### Case 1

A 65-year-old male farmer presented with chest pain of over 6 months duration. Vital signs upon admission to the hospital included a temperature of 37°C, blood pressure of 115/80 mmHg, and oxygen saturation of 95%. On physical exam, he was found to have rib tenderness in the absence of organomegaly. Familial and past medical histories were unremarkable. The patient reported a 30 pack-year history of smoking (30 pack-years) and a 40-year history of alcohol consumption.

**Figure 1:**
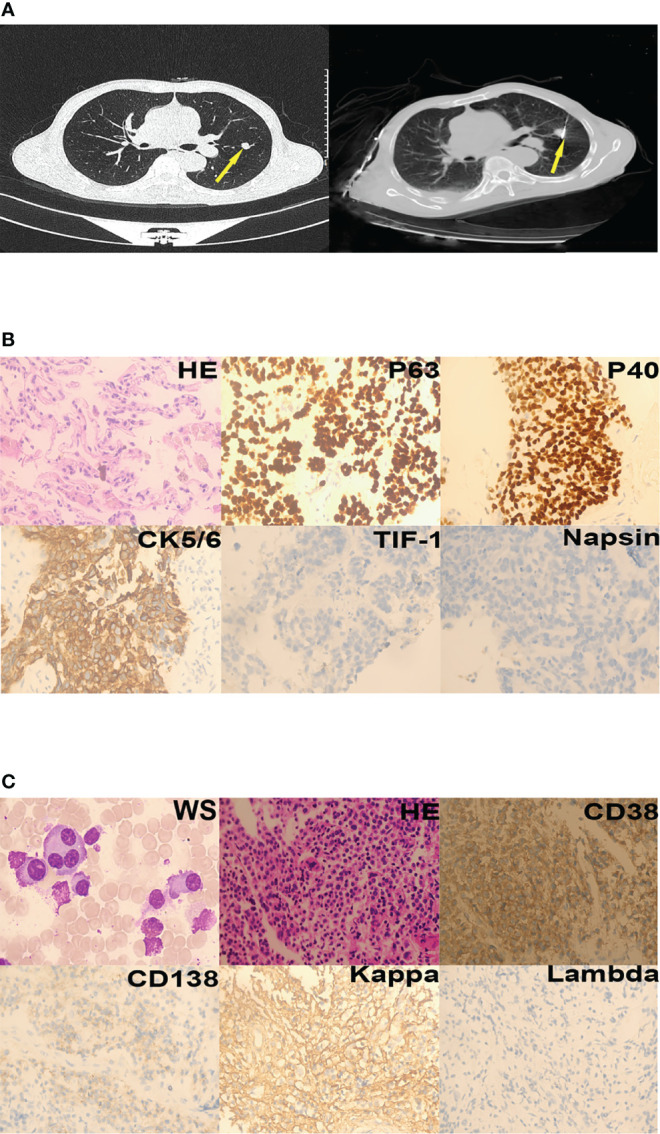
Case 1. **(A)** Lung nodules (yellow arrows) on chest computed tomography; left - cross section, right - post-treatment by percutaneous microwave ablation. **(B)** Squamous cells could be seen in the nodules (hematoxylin-eosin staining, 400×). Immunohistochemistry revealed that the tumor was P63-positive, P40-positive, CK5/6-positive, TIF-1-negative, and Napsin-negative. **(C)** Plasma cells were seen in the bone marrow aspirate and biopsy specimens (Wright staining [WS], 1000×; hematoxylin-eosin staining, 200×). Immunohistochemistry revealed that the plasma cells were CD38-positive, CD138-partly positive, kappa-positive, and lambda-negative.

Laboratory investigations revealed a hemoglobin level of 14.1 g/dL, a total leukocyte count of 11.13 × 10^9^/L, and a platelet count of 352 × 10^9^/L. Erythrocyte sedimentation rate was 41 mm/hour. Further tests showed serum alkaline phosphatase level of 155 U/L, a total serum protein level of 70.70 g/L, serum globulin level of 19.50 g/L, and an albumin/globulin ratio of 2.63. Urine Bence-Jones protein was negative. His serum calcium was elevated at 2.87 mmol/L;blood urea nitrogen was elevated at 9.10 mmol/L, serum creatinine levels was elevated at 138.00 umol/L. Pulmonary nodules were found in the left lung on chest computed tomography (**Figure 1A**, left). The patient was diagnosed with lung squamous cell carcinoma (LUSC) based on the results of pathological and immunohistochemical tests (**Figure 1B**). He underwent thoracentesis *via* percutaneous microwave ablation (**Figure 1A**, right) at 40W for 9 min. Bone marrow aspirate was cellular and showed hematopoietic cells in all series, series and many plasma cells (40%). Bone marrow pathology revealed the presence of myeloma cells (**Figure 1C**), and MM was also diagnosed. Due to financial constraints, the patient refused treatment as well as further investigations to classify the type of MM he was diagnosed with.

### Case 2

A 68-year-old female farmer presented with lower back pain and lower limb numbness for 2 months duration. Physical examination showed only pallor, a blood pressure of 173/73 mmHg, without enlarged spleen, liver and lymphnodes. There was no related familial or past history. The patient denied having a history of smoking or alcohol consumption.

**Figure 2:**
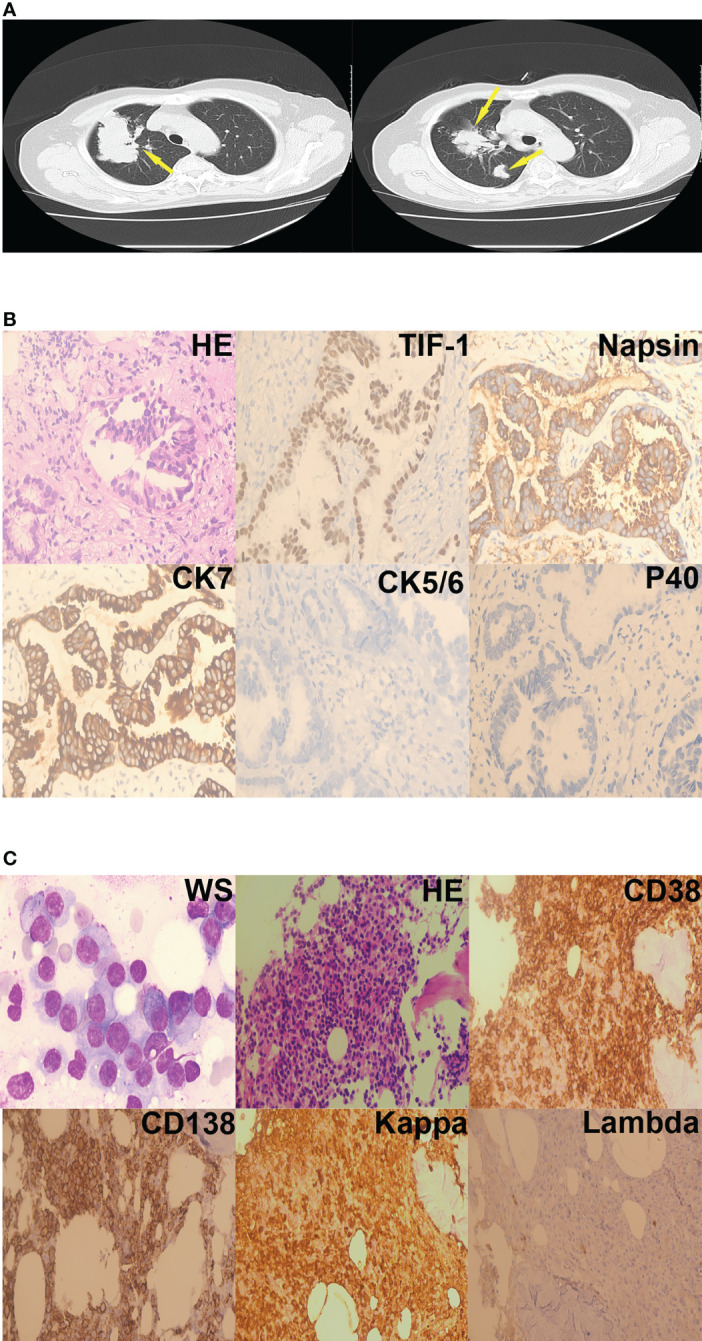
Case 2. **(A)** Lung mass (yellow arrows) on chest computed mass, cross-section. **(B)** Adenocarcinoma cells were present in the right lung nodule (hematoxylin-eosin staining, 400×). Immunohistochemistry revealed that the tumor cells were TIF-1-positive, Napsin-positive, CK7-positive, CK5/6-negative, and P40-negative. **(C)** Plasma cells were present in the bone marrow aspirates and biopsy specimens (Wright staining, 1000×; hematoxylin-eosin staining, 200×). Immunohistochemistry revealed that the plasma cells were CD38-positive, CD138-positive, kappa-positive, and lambda-negative.

Laboratory investigations revealed a hemoglobin level of 7.8 g/dL, a total leukocyte count of 5.59 × 10^9^/L, and a platelet count of 172 × 10^9^/L. Erythrocyte sedimentation rate was 182 mm/hour. Further laboratory testing showed serum alkaline phosphatase level of 55 IU/L, elevated total serum protein (107.52 g/L), elevated serum globulin (79.67g/L), and reduced albumin/globulin ratio (0.35). The urine Bence-Jones protein was positive. Serum calcium was 2.15 mmol/L, blood urea nitrogen was 6.01mmol/L, serum creatinine was 54.70 umol/L. A pulmonary obstruction mass was found in the right lung on chest computed tomography (**Figure 2A**). A diagnosis of lung adenocarcinoma (LUAD) was made based on pathology results and immunohistochemistry findings (**Figure 2B**). Bone marrow aspirate was cellular and showed hematopoietic cells in all series, increased plasma cells (45%). Immunoglobulin G and the kappa light chain were detected by serum protein immunofixation electrophoresis. Serum protein immunofixation electrophoresis helped assess serum values of immunoglobulin G(82.10 g/L), λ free light chain(234.72 mg/L), λ free light chain (2.35 mg/L), and calculated the κ/λ ratio (99.8809). Urine protein immunofixation electrophoresis aided to assess urine values of κ free light chain level (11.70 mg/L) λ free light chain level (2.35 mg/L) and calculate the κ/λ ratio (2.2500). Flow cytometry indicated that monoclonal cells accounted for 5.80% of plasma cells; the immunophenotype was CD38-positive, CD138-positive, CD19-negative, CD20-negative, CD27-partially positive, CD28-negative, CD56-positive, CD81-negative, and CD200-positive. κ light chain expression was restricted to the intracellular globulin. The diagnosis of MM was further supported by the presence of myeloma cells on bone marrow biopsy. (**Figure 2C**). Fluorescence *in situ* hybridization detected 1q21 amplification, immunoglobulin heavy chain (IGH) rearrangement, *RB1* deletion, and no p53 deletion. The chromosome karyotype was 46, XX. The patient diagnosed MM IG-G κ type at D-S IIIA. Hybridization capture-based next-generation sequencing of a sample of the lung tumor revealed mutations in ERBB2 exon 20 (29.82%), KRAS exon 3 (1.04%), and KRAS (1.71%). The patient was administered oral afatinib (40mg/d) that was not tolerated by the patient who experienced repeated episodes of vomiting. Afatinib was, thus, discontinued after two days of administration. The patient was then administered 500 mg/m^2^ of intravenous pemetrexed (500 mg/m^2^, intravenously, day 1) combined with 200 mg intravenous camrelizumab, a monoclonal antibody against PD-1,) every 2 weeks. The patient was discharged from the hospital a month later.

### Analysis of Microarray Data and Identification of Differently Expressed Genes

A database search was performed to identify differently expressed genes (DEGs) in lung cancer and MM. Data from 1325 patients with MM and 58 healthy donors were obtained from seven MM gene expression microarray datasets(GSE2658, GSE5900, GSE6477, GSE39754, GSE16558, GSE13591, and GSE47552) from the Gene Expression Omnibus (http://www.ncbi.nlm.nih.gov/geo/) and analyzed using R software (Version 4.0.5, R Foundation for Statistical Computing, Vienna, Austria). The Cancer Genome Atlas MM RNA sequencing data set (MMRF-CoMMpass), Lung Adenocarcinoma RNA sequencing data set (TCGA-LUAD), Lung Squamous Cell Carcinoma RNA sequencing data set (TCGA-LUSC) were obtained from the Genomic Data Commons Data Portal (GDC, https://portal.gdc.cancer.gov/). Survival data were obtained from UCSC Xena (https://xenabrowser.net/datapages/). Data analysis was performed using the “EdgeR” package in R (version 3.30.3).

**Figure 3:**
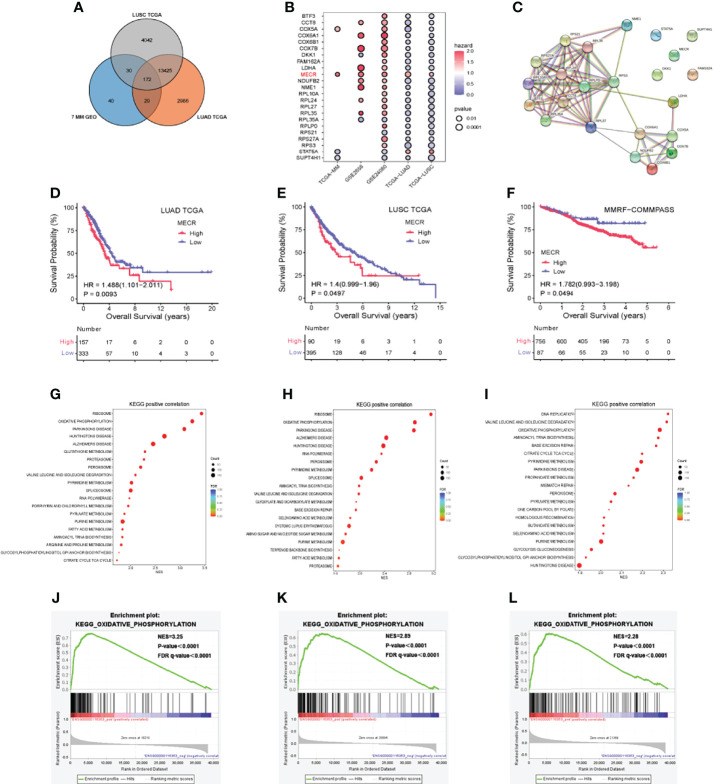
MM and lung cancer bioinformatics analysis **(A)** Venn diagram indicating the number of differentially expressed genes **(B)** Correlation of differentially expressed genes with patient over survival **(C)** Protein-protein interaction network **(D–F)** Overall survival (OS) analysis based on *MECR* expression **(G–I)** Kyoto encyclopedia of genes and genomes (KEGG) pathway enrichment analysis **(J–L)** High expression of *MECR* positive correlated with oxidative phosphorylation pathway.

For lung cancer, data from 510 patients with LUAD were compared to those of 58 control patients, and data from 496 patients with LUSC were compared to those of 49 control patients. We identified 172 genes differentially expressed among all of the lung tumor datasets and the seven MM datasets (**Figure 3A**), including 135 DEGs of the 172 identified ones exhibiting similar expression trends.

23 DEGs of 135 DEGs were significantly associated with overall survival in datasets including GSE24080, TCGA-LUAD, TCGA-LUSC. Most of them in the protein-protein interaction network (**Figure 3C**). Univariate Cox regression analysis showed that one DEG (*MECR*) was a risk factor (hazard ratio > 1.0) in five datasets (**Figure 3B**). A p value < 0.05 was used to identify DEGs in each data set. p < 0.05 was set as the threshold.

### Overall Survival Analysis For Mecr

The correlations between *MECR* expression and overall survival were analyzed by Kaplan-Meier survival plot. The overall surival datases including GSE2658 and GSE24080 dataset and TCGA cohort were analysed. The differences in survival percent between patients with high and low MECR expression levels divided by median value. Statistical significance was set at P < 0.05. High MECR expression was associated with a worse prognosis in LUAD, LUSC, and MM (P < 0.05; **Figures 3D–F**).

### Pathway Enrichment Analysis

We explored Kyoto Encyclopedia of Genes and Genomes (KEGG) pathways associated with the MECR upregulation in LUAD, LUSC and MM, the Gene Set Enrichment Analysis was performed using the TCGA-LUAD, TCGA-LUSC, TCGA-MM datasets. The reference genesets in this study was the c2.cp.kegg.v7.5.1.symbols.gmt, and the NES (normalized enrichment score) was calculated. If the normal p value and FDR (false discovery rate) q value were both less than 0.05, the gene set was significantly enriched. In LUAD, the “ribosome,” “oxidative phosphorylation,” and “parkinsons disease” pathways were significantly enriched (all *P* < 0.05) (**Figure 3G**). In LUSC, the “ribosome,” “oxidative phosphorylation,” and “parkinsons disease” pathways were significantly enriched (all *P* < 0.05) (**Figure 3H**). In MM, the “DNA replication,” “valine leucine and isoleucine degradation” and “oxidative phosphorylation” pathways were significantly enriched (all *P* < 0.05) (**Figure 3I**).”oxidative phosphorylation “ pathways were significantly enriched in TCGA-LUAD, TCGA-LUSC, TCGA-MM (all *P* < 0.05) (**Figures 3J–L**).

## Discussion

The co-occurrence of two simultaneous tumors has great implications on treatment modalities and warrants important considerations. Our first patient was treated with local surgery because of the small focus of his lung cancer. While writing this, our second patient has undergone targeted lung tumors treatment because of the large focus of her lung cancer. The development of additional primary cancers in patients with MM negatively impacts long-term survival. A study of 2,732 patients with MM treated with lenalidomide found no gastric cancers, five (0.18%) lung cancers, and only three multiple primary solid cancers (3). There are some reports of the successive occurrence of MM and solid tumors. Kaiser et al. (4) described their experience treating a 68-year-old man who was diagnosed with non-small cell lung cancer by image-guided biopsy 10 years after being treated for MM. Marinopoulos et al. (5) reported a case of LUAD with MM development after the completion of chemotherapy. Hasskarl et al. (6) reported 59 patients had coexisting primary neoplasms. In patients with MM and solid neoplasms, 53% solid tumors were diagnosed prior to the MM diagnosis, 20% developed after the myeloma, and only three patients (5%) were simultaneously diagnosed with MM and solid tumors. The simultaneous occurrence of lung cancer in patients who have not yet received treatment for MM is relatively rare. Based on the data extracted from our experience with these two patients and the data reported in the literature reports, we further analyzed gene expression in MM and lung cancer in order to identify potential genes promoting the development of a second primary tumor.

Bioinformatics analysis showed that mitochondrial trans-2-enoyl-CoA reductase (*MECR*) was highly expressed in both tumor types. In patients with lung cancer, high *MECR* expression was associated with poorer overall survival compared that in those with low MECR expression. *MECR* is a component of the mitochondrial FAS II pathway and is responsible for catalyzing the final step of the fatty acid elongation cycle: the NADPH-dependent reduction of the enoyl-acyl carrier protein substrate. The role of *MECR* as a novel therapeutic target in cancer has been previously reported, specifically in the context of hepatocellular carcinoma (7). *MECR* is involved in the mitochondrial fatty acid synthesis. MECR overexpression increases peroxisome proliferator-activated receptor-α (PPARα) activity (8). Activated PPARα inhibits or promotes tumorigenesis depending on the specific tissues in which it acts, but the molecular mechanism remains poorly understood (9). Cytosolic *MECR* can act as a coactivator of PPARα in the nucleus of HeLa cells (10). PPARα activation promotes partial restoration of endothelial function by attenuating lipotoxicity, potentially inhibiting ROS generation, and upregulating proinflammatory cytokines and adhesion molecules. (11). Accordingly, we hypothesize that activated PPARα results in the overexpression of MECR and adhesion molecules like junctional adhesion molecule A (JAM-A) found in endothelial cells, promoting neovascularization in tumors. (12). Moschetta et al (13) also previously described the interdependency of endothelial and MM-cells where endothelial progenitor cell trafficking enhances MM progression, particularly at early disease stages. Our results suggest that *MECR* is a potential driver gene for MM and lung cancer co-occurrence.

Our GSEA results identified a positive correlation between the oxidative phosphorylation (OXPHOS) pathway and high MECR expression in patients with MM and lung cancer. Overactive OXPHOS provides sufficient bioenergy to fuel the progression of several tumors. Marlein et al(14). demonstrated that upregulation of glycolysis and OXPHOS are necessary for the survival of MM cells, and that the enhancement of OXPHOS upregulation is facilitated by the transfer of mitochondria from neighboring non-malignant cells to MM cells. Moreover, several recent studies have suggested that unlike many other cancers, lung tumors are highly oxidative, requiring OXPHOS development (15). Previous studies have shown that carfilzomib could provide metabolic stability and has antitumor effects in human MM and lung cancer cell lines (16). Therefore, drugs that target both *MECR* and components of the OXPHOS pathway should be explored as potential treatment for patients with simultaneous MPCs. Unfortunately, we could not proceed further with the research of *MECR* in these two cases. *MECR* may serve as a biomarker in cases of MM and lung cancer co-occurrence. Currently, large-scale data exploring MM and lung cancer co-occurrence are lacking, and only case reports exist. Thus, our report and bioinformatic analysis provides starting points for much needed basic science and clinical investigations.

## Conclusion

The occurrence of lung cancer in patients with untreated MM is rare and interesting. High expression of *MECR* is associated with a worse prognosis in both lung cancer and MM. *MECR* and OXPHOS pathways may be implicated in the development of co-occurring MM and lung cancer.

## Data Availability Statement

The datasets presented in this study can be found in online repositories. The names of the repository/repositories and accession number(s) can be found in the article/**Supplementary Material**.

## Ethics Statement

This study was approved by the second affiliated hospital of xiamen medical college ethics committee, Approval number: (2021068). Written informed consent was obtained from the individual(s) for the publication of any potentially identifiable images or data included in this article.

## Author Contributions

P-PX: writing - original draft and formal analysis. Z-GD and X-YC: supervision. YZ and Y-QC: case tracking. B-QL, WF, and J-MH: data collection. All authors contributed to the article and approved the submitted version.

## Funding

This work was supported by the National Natural Science Foundation of China for youth project (Grant Number 81901669), Xiamen Science and Technology (Medical and Health) Project (No. 3502Z20184061), Xiamen Medical and Health Guidance Project (No. 3502Z20199137), Fujian Medical and Health Training Project for young and middle-aged backbone talents (No. 2020GGB068), and Educational and scientific research program for young and middle-aged teachers of Fujian Province (No. JAT190838).

## Conflict of Interest

The authors declare that the research was conducted in the absence of any commercial or financial relationships that could be construed as a potential conflict of interest.

## Publisher’s Note

All claims expressed in this article are solely those of the authors and do not necessarily represent those of their affiliated organizations, or those of the publisher, the editors and the reviewers. Any product that may be evaluated in this article, or claim that may be made by its manufacturer, is not guaranteed or endorsed by the publisher.
