# Supplementary material for: Simultaneous Presentation of Multiple Myeloma and Lung Cancer: Case Report and Gene Bioinformatics Analysis
Source: Front Oncol. 2022 Jun 13;12:859735. doi: 10.3389/fonc.2022.859735 (PMC9235397; doi:10.3389/fonc.2022.859735)
Supplement: Supplementary file 1 [file DataSheet_1.zip › The bioinformatic analysis of MM and lung cancer supplementary materials/Enrichment analysis/23╕÷DEG/metascape/AnalysisReport.pptx]

## Slide 1
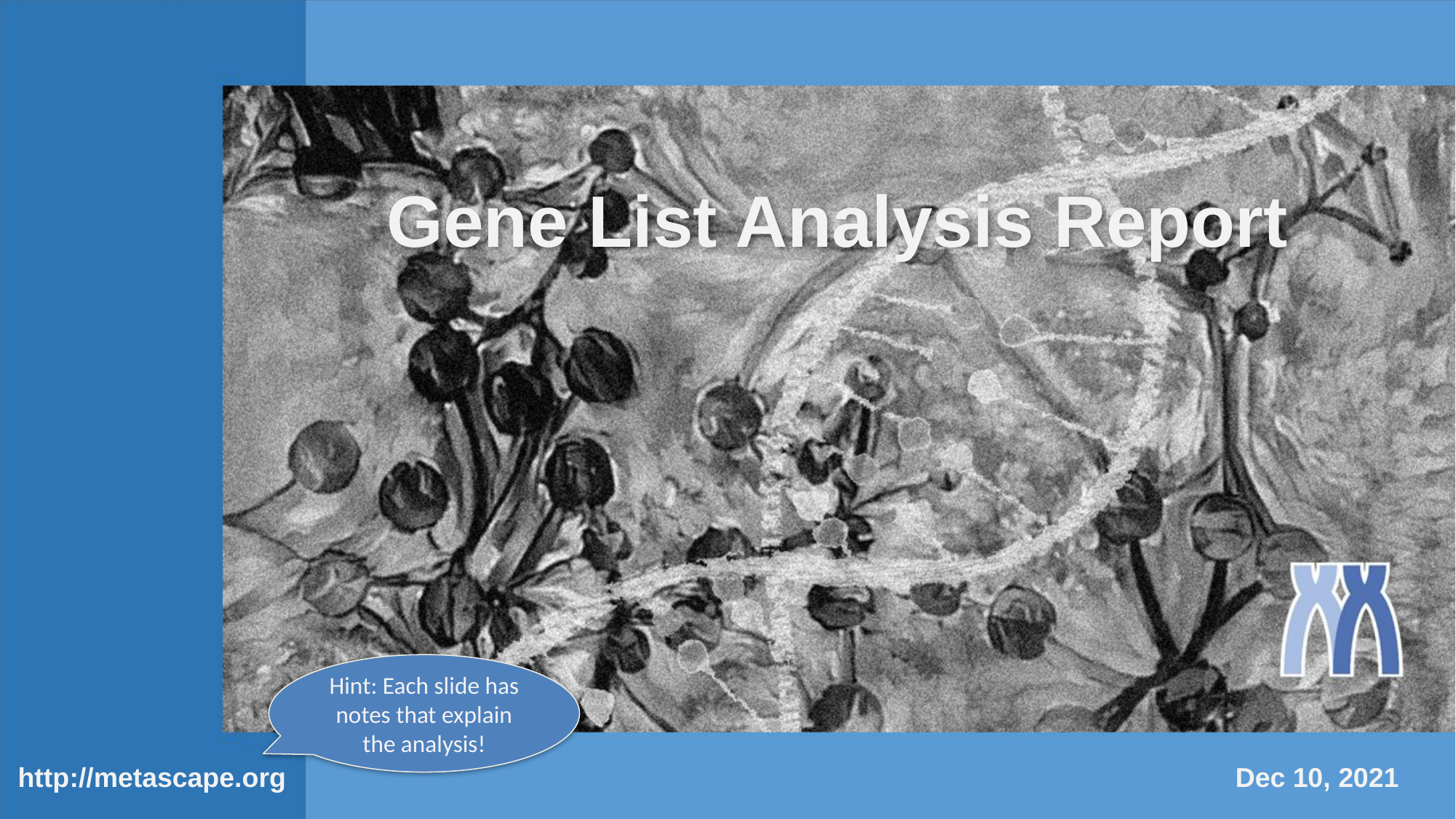

# Gene List Analysis Report
Hint: Each slide has notes that explain the analysis!
http://metascape.org
Dec 10, 2021

## Slide 2
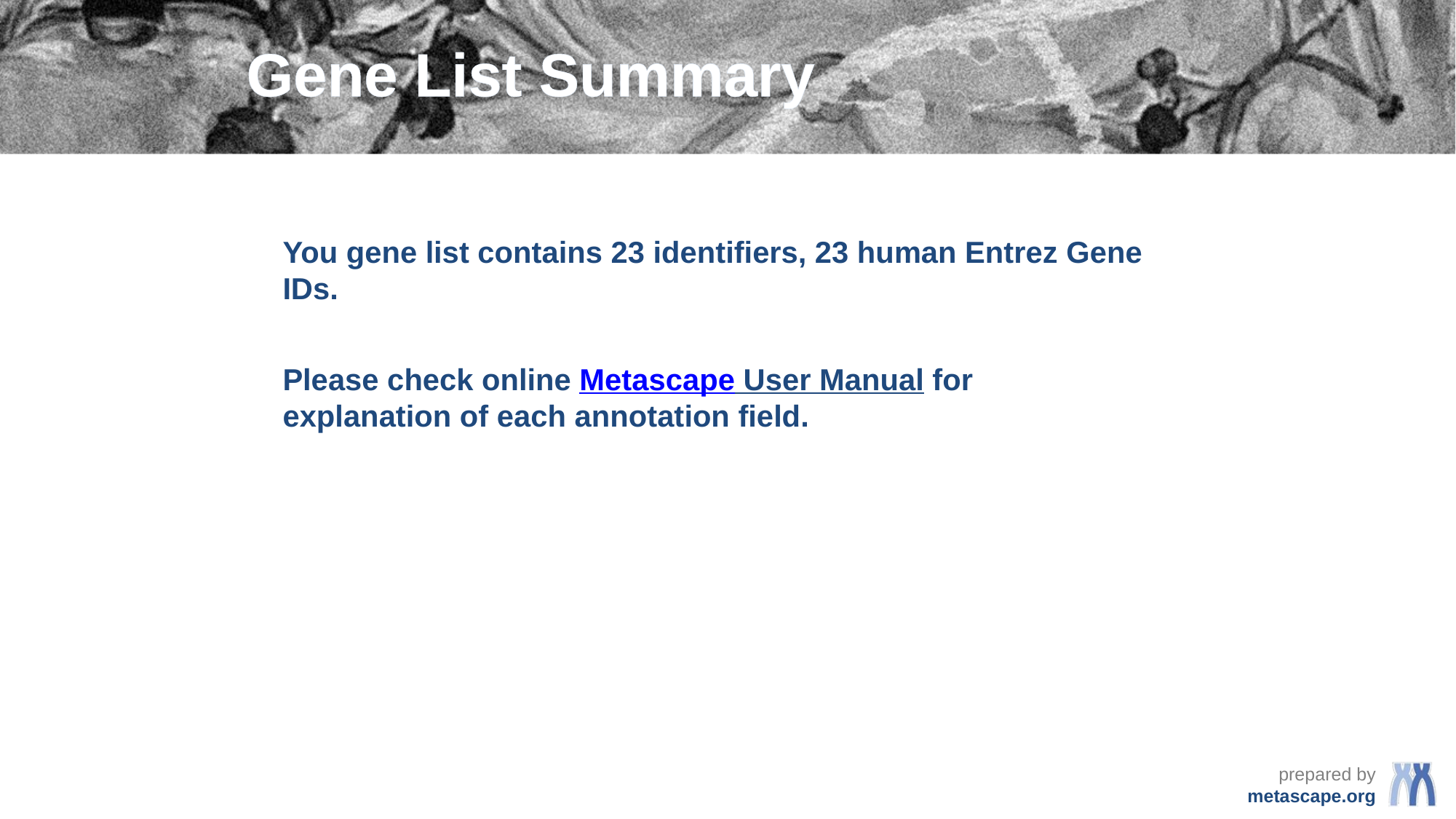

# Gene List Summary
You gene list contains 23 identifiers, 23 human Entrez Gene IDs.
Please check online Metascape User Manual for explanation of each annotation field.

## Slide 3
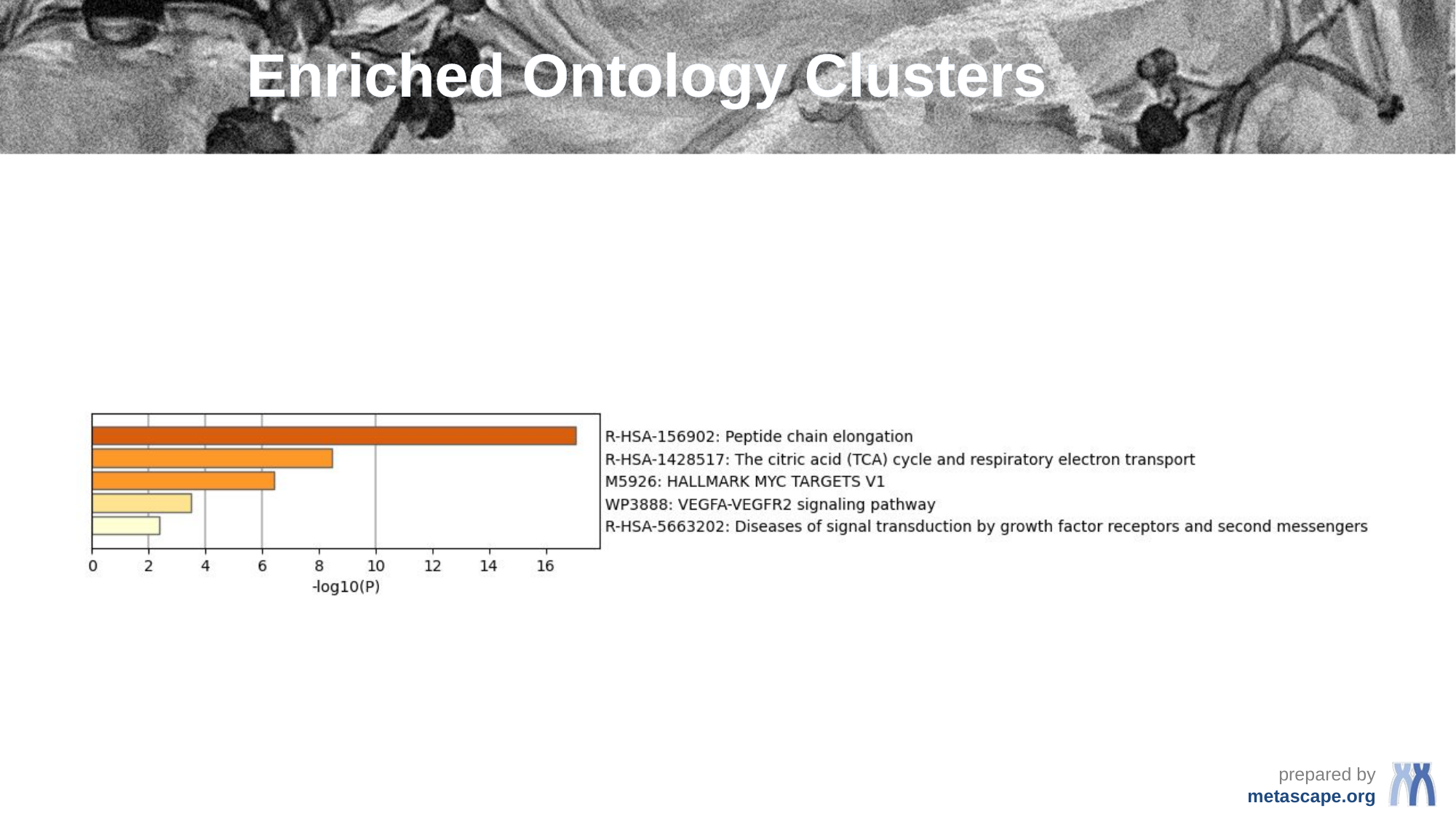

# Enriched Ontology Clusters

## Slide 4
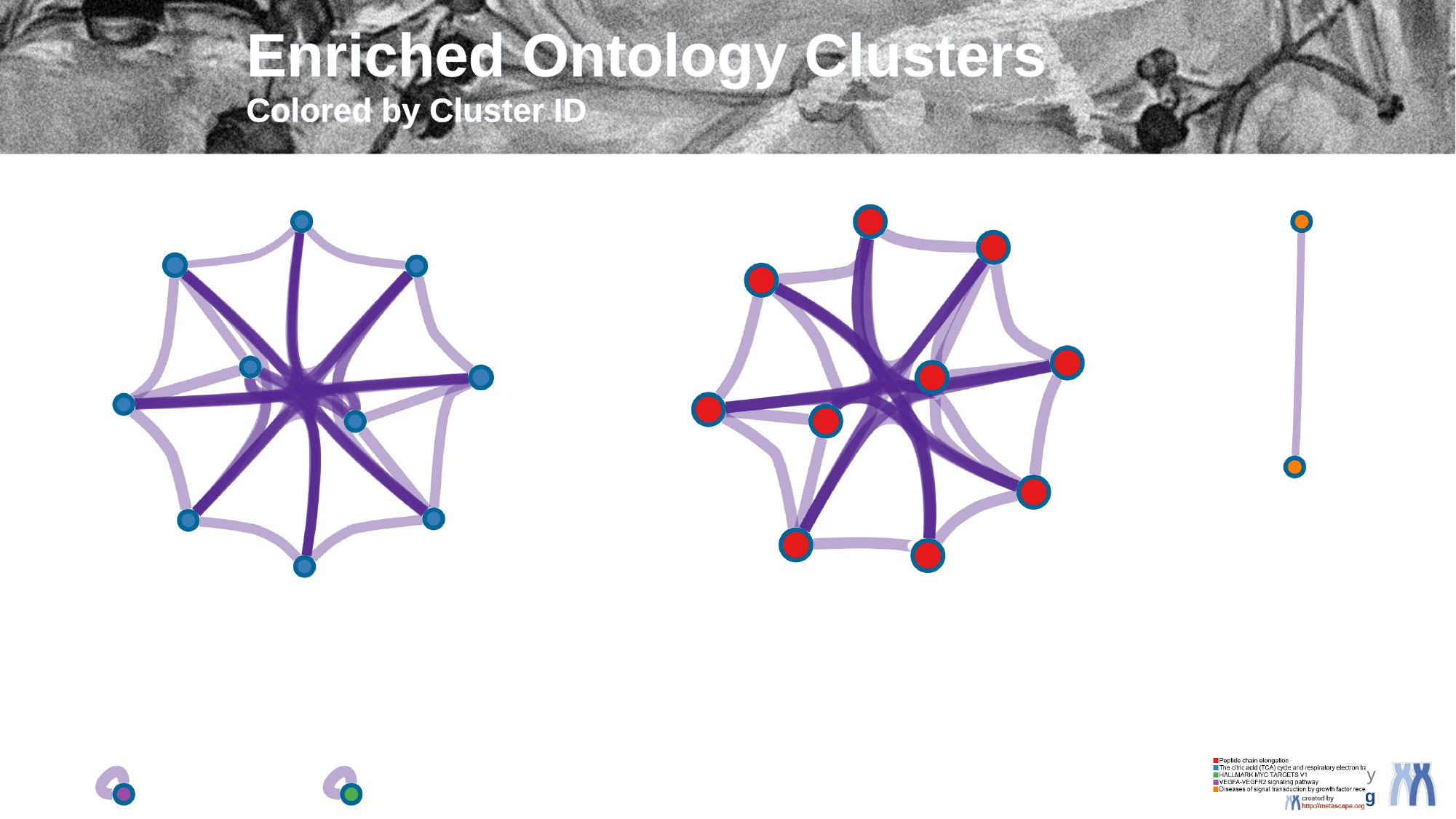

# Enriched Ontology Clusters Colored by Cluster ID

## Slide 5
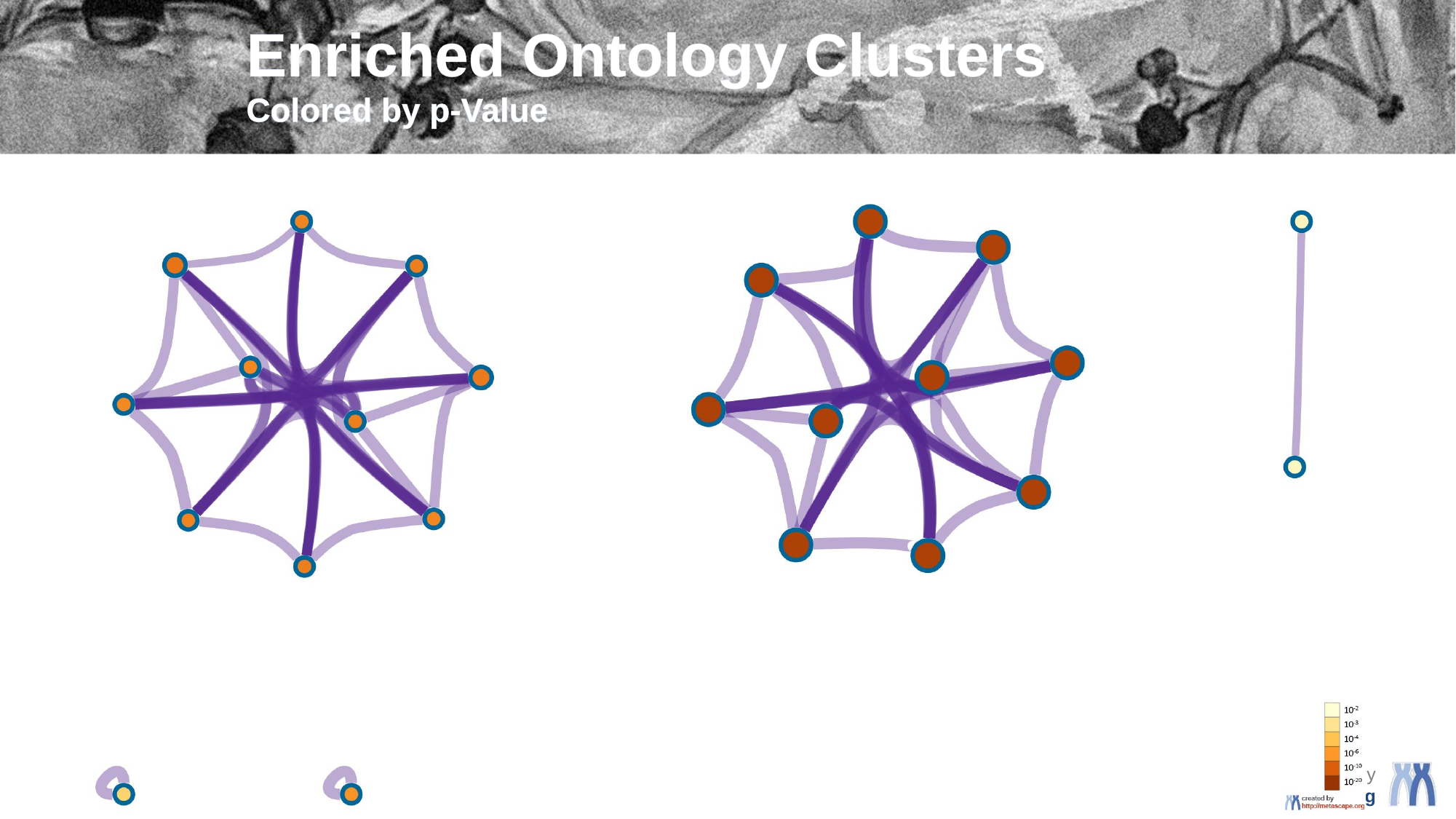

# Enriched Ontology Clusters Colored by p-Value

## Slide 6
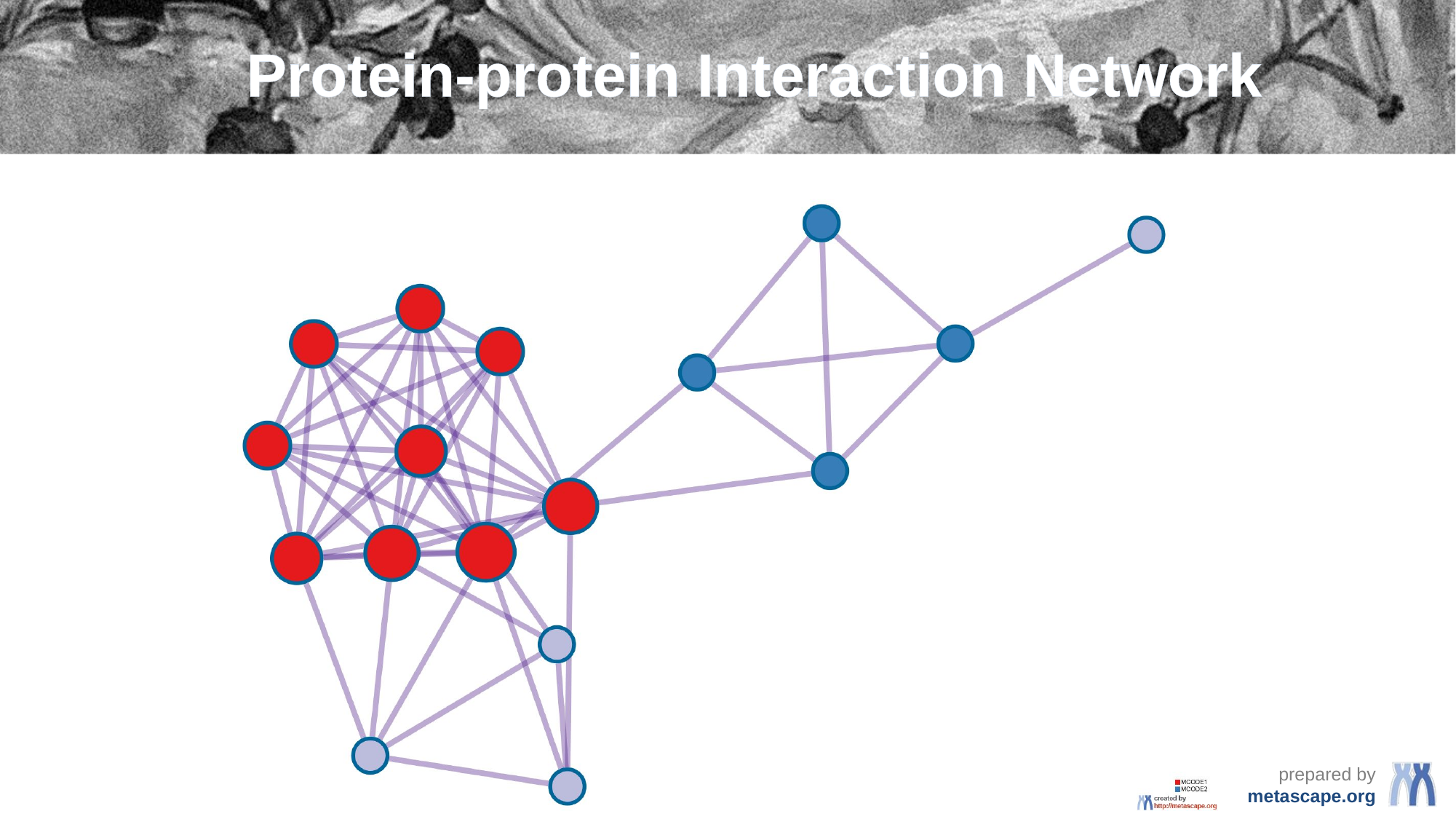

# Protein-protein Interaction Network

## Slide 7
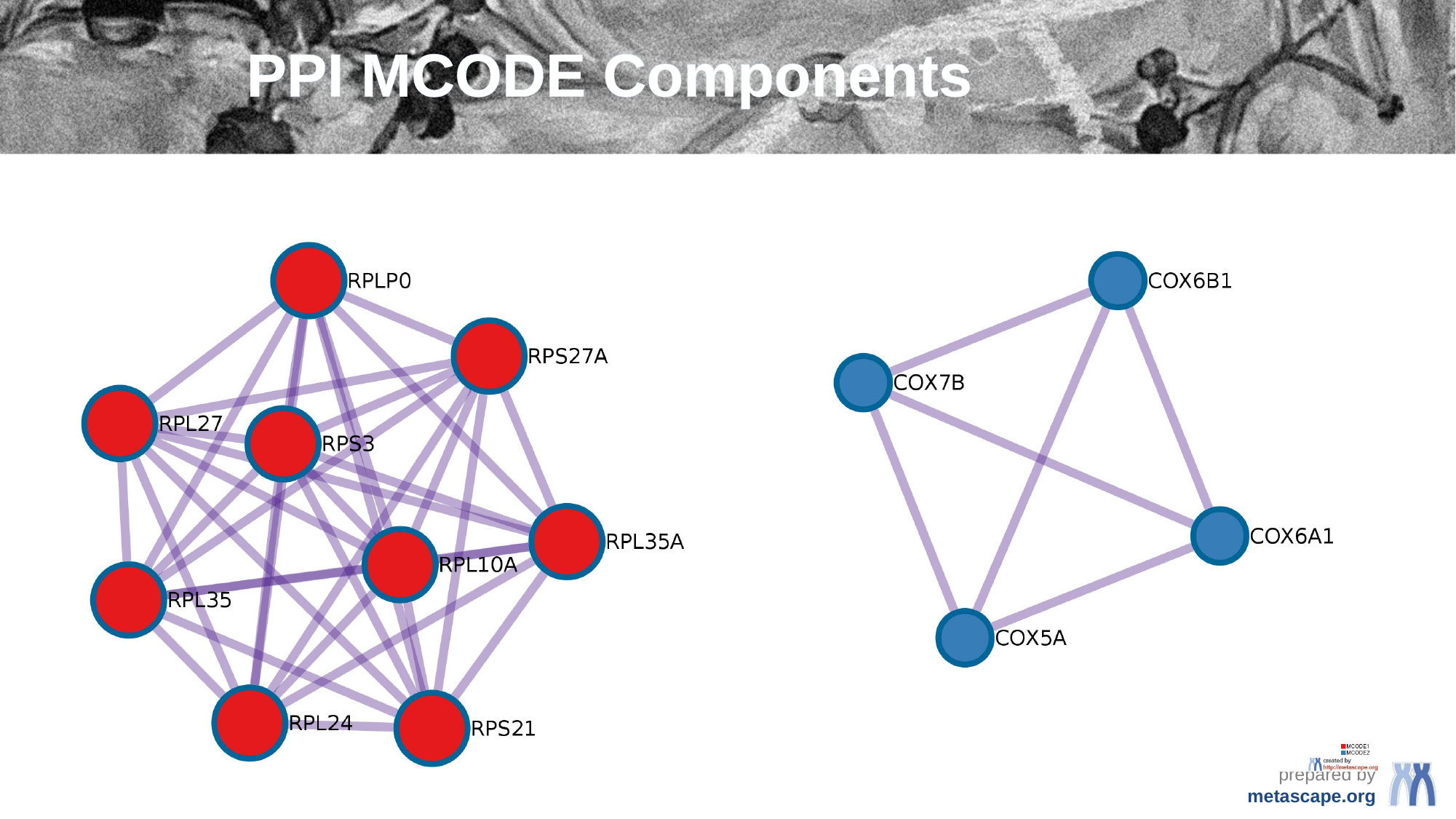

# PPI MCODE Components

## Slide 8
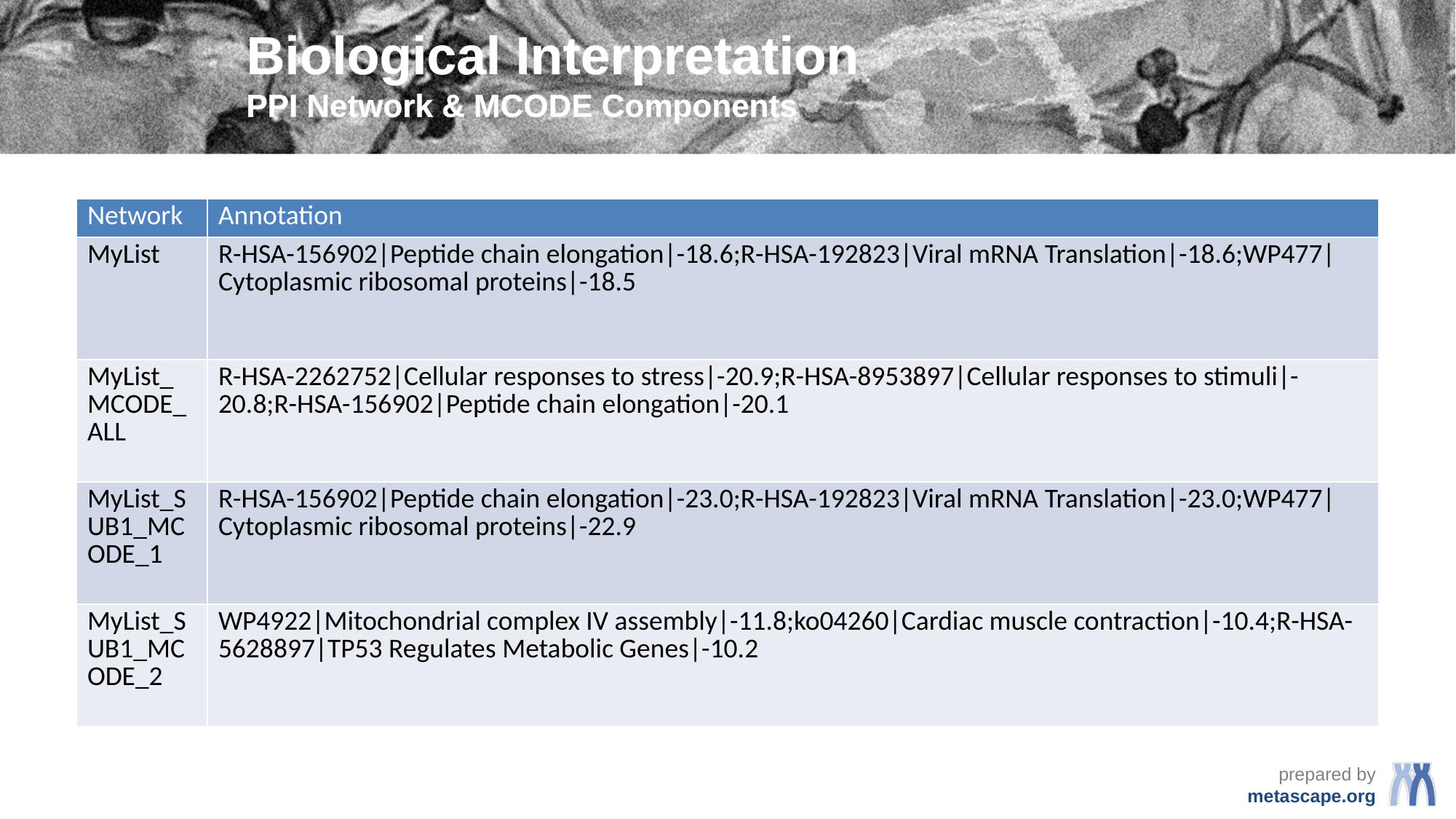

# Biological Interpretation PPI Network & MCODE Components
| Network | Annotation |
| --- | --- |
| MyList | R-HSA-156902|Peptide chain elongation|-18.6;R-HSA-192823|Viral mRNA Translation|-18.6;WP477|Cytoplasmic ribosomal proteins|-18.5 |
| MyList\_MCODE\_ALL | R-HSA-2262752|Cellular responses to stress|-20.9;R-HSA-8953897|Cellular responses to stimuli|-20.8;R-HSA-156902|Peptide chain elongation|-20.1 |
| MyList\_SUB1\_MCODE\_1 | R-HSA-156902|Peptide chain elongation|-23.0;R-HSA-192823|Viral mRNA Translation|-23.0;WP477|Cytoplasmic ribosomal proteins|-22.9 |
| MyList\_SUB1\_MCODE\_2 | WP4922|Mitochondrial complex IV assembly|-11.8;ko04260|Cardiac muscle contraction|-10.4;R-HSA-5628897|TP53 Regulates Metabolic Genes|-10.2 |

## Slide 9
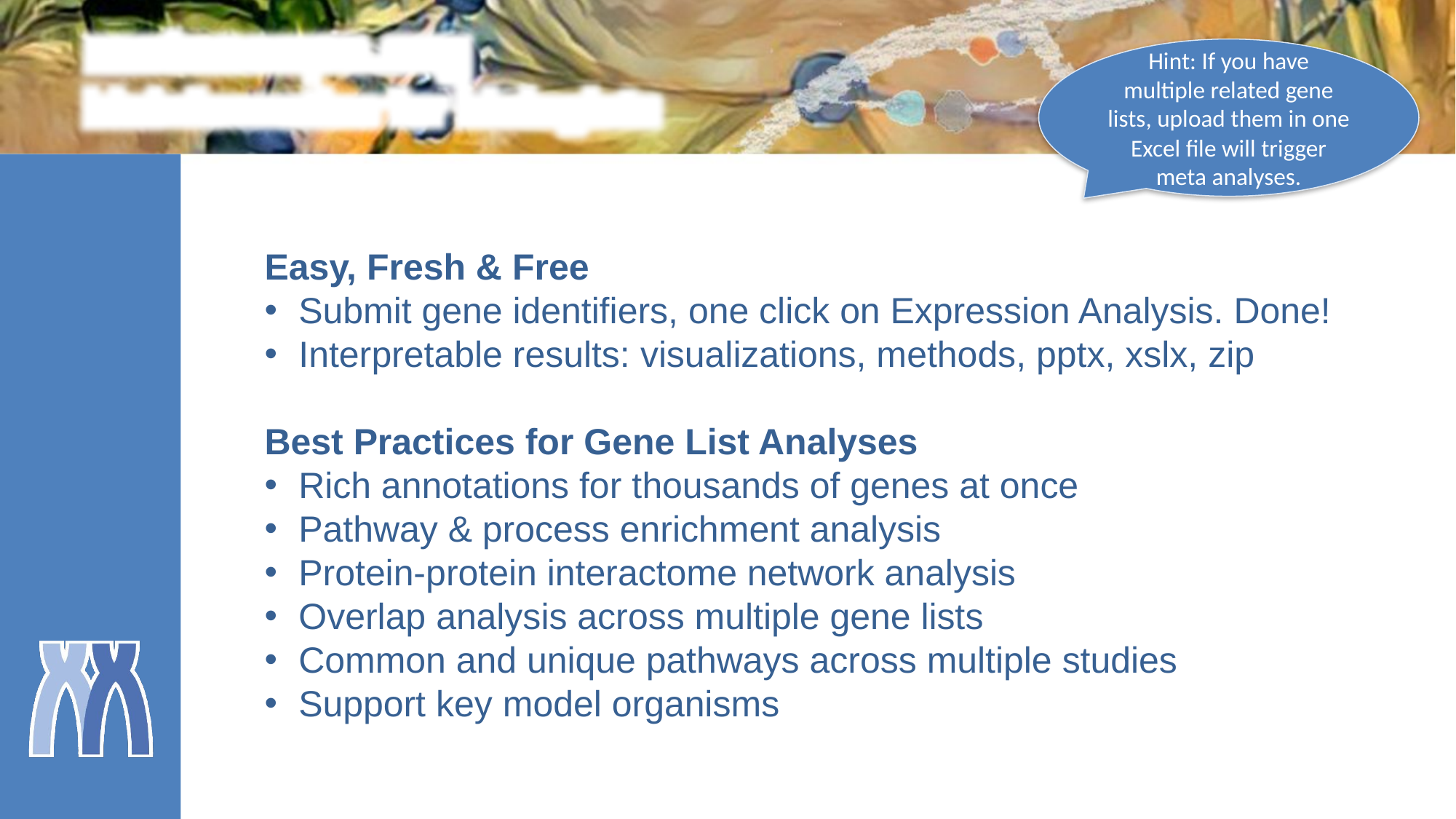

metascape.org
bioinformatics for biologists
Hint: If you have multiple related gene lists, upload them in one Excel file will trigger meta analyses.
Easy, Fresh & Free
Submit gene identifiers, one click on Expression Analysis. Done!
Interpretable results: visualizations, methods, pptx, xslx, zip
Best Practices for Gene List Analyses
Rich annotations for thousands of genes at once
Pathway & process enrichment analysis
Protein-protein interactome network analysis
Overlap analysis across multiple gene lists
Common and unique pathways across multiple studies
Support key model organisms
