# Supplementary material for: Simultaneous Presentation of Multiple Myeloma and Lung Cancer: Case Report and Gene Bioinformatics Analysis
Source: Front Oncol. 2022 Jun 13;12:859735. doi: 10.3389/fonc.2022.859735 (PMC9235397; doi:10.3389/fonc.2022.859735)
Supplement: Supplementary file 1 [file DataSheet_1.zip › The bioinformatic analysis of MM and lung cancer supplementary materials/Enrichment analysis/23╕÷DEG/metascape/Enrichment_GO/ColorByCluster.pdf]

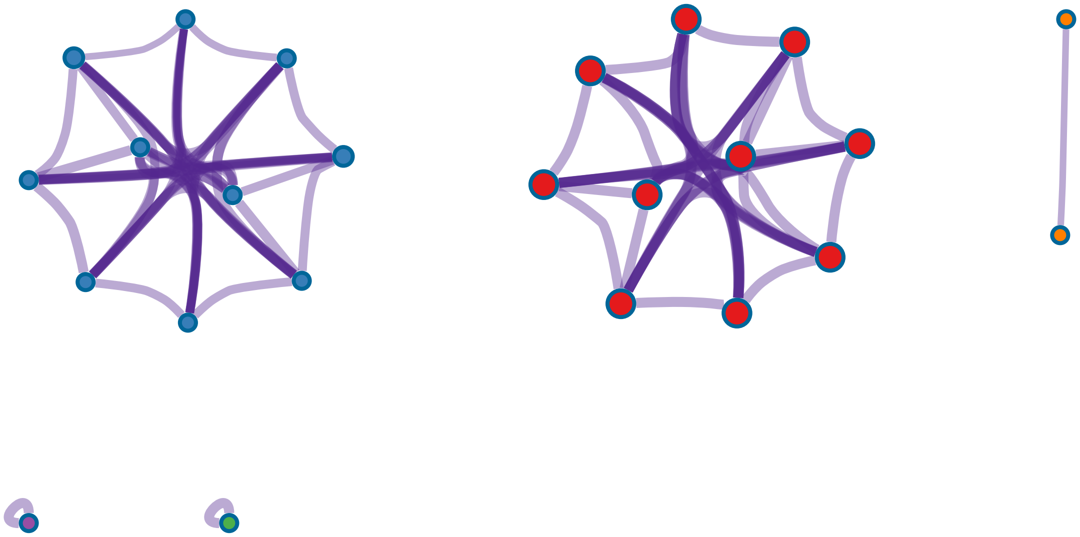

Peptide chain elongation
The citric acid (TCA) cycle and respiratory electron tra
HALLMARK MYC TARGETS V1
VEGFA-VEGFR2 signaling pathway
Diseases of signal transduction by growth factor rece

created by
http://metascape.org
