# Supplementary material for: Simultaneous Presentation of Multiple Myeloma and Lung Cancer: Case Report and Gene Bioinformatics Analysis
Source: Front Oncol. 2022 Jun 13;12:859735. doi: 10.3389/fonc.2022.859735 (PMC9235397; doi:10.3389/fonc.2022.859735)
Supplement: Supplementary file 1 [file DataSheet_1.zip › The bioinformatic analysis of MM and lung cancer supplementary materials/Enrichment analysis/MECR/GSEA_4.1.0/LUAD TCGA/KEGG.Gsea.1639041756227/KEGG_ALZHEIMERS_DISEASE.html]

Details for gene set KEGG\_ALZHEIMERS\_DISEASE[GSEA]

|  || Dataset | ExpData\_collapsed\_to\_symbols.ENSG00000116353\_profile\_in\_ExpData.cls #ENSG00000116353 |
| Phenotype | ENSG00000116353\_profile\_in\_ExpData.cls#ENSG00000116353 |
| Upregulated in class | ENSG00000116353\_pos |
| GeneSet | KEGG\_ALZHEIMERS\_DISEASE |
| Enrichment Score (ES) | 0.5544762 |
| Normalized Enrichment Score (NES) | 2.4552758 |
| Nominal p-value | 0.0 |
| FDR q-value | 0.0 |
| FWER p-Value | 0.0 |
Table: GSEA Results Summary

  

Fig 1: Enrichment plot: KEGG\_ALZHEIMERS\_DISEASE      
 Profile of the Running ES Score & Positions of GeneSet Members on the Rank Ordered List

  

| SYMBOL | TITLE | RANK IN GENE LIST | RANK METRIC SCORE | RUNNING ES | CORE ENRICHMENT || 1 | NDUFS8 | NADH:ubiquinone oxidoreductase core subunit S8 [Source:HGNC Symbol;Acc:HGNC:7715] | 34 | 0.446 | 0.0142 | Yes |
| 2 | NDUFS5 | NADH:ubiquinone oxidoreductase subunit S5 [Source:HGNC Symbol;Acc:HGNC:7712] | 78 | 0.411 | 0.0270 | Yes |
| 3 | CDK5 | cyclin dependent kinase 5 [Source:HGNC Symbol;Acc:HGNC:1774] | 106 | 0.401 | 0.0399 | Yes |
| 4 | NDUFA2 | NADH:ubiquinone oxidoreductase subunit A2 [Source:HGNC Symbol;Acc:HGNC:7685] | 110 | 0.398 | 0.0533 | Yes |
| 5 | NDUFB10 | NADH:ubiquinone oxidoreductase subunit B10 [Source:HGNC Symbol;Acc:HGNC:7696] | 142 | 0.385 | 0.0655 | Yes |
| 6 | BAD | BCL2 associated agonist of cell death [Source:HGNC Symbol;Acc:HGNC:936] | 174 | 0.379 | 0.0775 | Yes |
| 7 | ATP5PO | ATP synthase peripheral stalk subunit OSCP [Source:HGNC Symbol;Acc:HGNC:850] | 231 | 0.366 | 0.0884 | Yes |
| 8 | NDUFB7 | NADH:ubiquinone oxidoreductase subunit B7 [Source:HGNC Symbol;Acc:HGNC:7702] | 278 | 0.355 | 0.0993 | Yes |
| 9 | NDUFV1 | NADH:ubiquinone oxidoreductase core subunit V1 [Source:HGNC Symbol;Acc:HGNC:7716] | 308 | 0.349 | 0.1103 | Yes |
| 10 | NDUFV3 | NADH:ubiquinone oxidoreductase subunit V3 [Source:HGNC Symbol;Acc:HGNC:7719] | 321 | 0.346 | 0.1217 | Yes |
| 11 | ATP5PD | ATP synthase peripheral stalk subunit d [Source:HGNC Symbol;Acc:HGNC:845] | 341 | 0.343 | 0.1328 | Yes |
| 12 | NDUFB2 | NADH:ubiquinone oxidoreductase subunit B2 [Source:HGNC Symbol;Acc:HGNC:7697] | 440 | 0.327 | 0.1414 | Yes |
| 13 | SDHB | succinate dehydrogenase complex iron sulfur subunit B [Source:HGNC Symbol;Acc:HGNC:10681] | 443 | 0.327 | 0.1524 | Yes |
| 14 | NDUFA6 | NADH:ubiquinone oxidoreductase subunit A6 [Source:HGNC Symbol;Acc:HGNC:7690] | 471 | 0.324 | 0.1626 | Yes |
| 15 | ATP5F1D | ATP synthase F1 subunit delta [Source:HGNC Symbol;Acc:HGNC:837] | 480 | 0.321 | 0.1733 | Yes |
| 16 | NDUFS7 | NADH:ubiquinone oxidoreductase core subunit S7 [Source:HGNC Symbol;Acc:HGNC:7714] | 511 | 0.317 | 0.1833 | Yes |
| 17 | UQCR10 | "ubiquinol-cytochrome c reductase, complex III subunit X [Source:HGNC Symbol;Acc:HGNC:30863]" | 623 | 0.305 | 0.1907 | Yes |
| 18 | NDUFC1 | NADH:ubiquinone oxidoreductase subunit C1 [Source:HGNC Symbol;Acc:HGNC:7705] | 648 | 0.303 | 0.2004 | Yes |
| 19 | UQCRC1 | ubiquinol-cytochrome c reductase core protein 1 [Source:HGNC Symbol;Acc:HGNC:12585] | 653 | 0.302 | 0.2105 | Yes |
| 20 | NDUFS6 | NADH:ubiquinone oxidoreductase subunit S6 [Source:HGNC Symbol;Acc:HGNC:7713] | 687 | 0.298 | 0.2197 | Yes |
| 21 | ATP5PF | ATP synthase peripheral stalk subunit F6 [Source:HGNC Symbol;Acc:HGNC:847] | 702 | 0.297 | 0.2294 | Yes |
| 22 | APH1A | "aph-1 homolog A, gamma-secretase subunit [Source:HGNC Symbol;Acc:HGNC:29509]" | 703 | 0.297 | 0.2395 | Yes |
| 23 | CYC1 | cytochrome c1 [Source:HGNC Symbol;Acc:HGNC:2579] | 712 | 0.296 | 0.2493 | Yes |
| 24 | NDUFA4 | NDUFA4 mitochondrial complex associated [Source:HGNC Symbol;Acc:HGNC:7687] | 738 | 0.293 | 0.2585 | Yes |
| 25 | NDUFB8 | NADH:ubiquinone oxidoreductase subunit B8 [Source:HGNC Symbol;Acc:HGNC:7703] | 748 | 0.293 | 0.2682 | Yes |
| 26 | ATP5MC1 | ATP synthase membrane subunit c locus 1 [Source:HGNC Symbol;Acc:HGNC:841] | 755 | 0.293 | 0.2779 | Yes |
| 27 | UQCR11 | "ubiquinol-cytochrome c reductase, complex III subunit XI [Source:HGNC Symbol;Acc:HGNC:30862]" | 764 | 0.292 | 0.2876 | Yes |
| 28 | UQCRQ | ubiquinol-cytochrome c reductase complex III subunit VII [Source:HGNC Symbol;Acc:HGNC:29594] | 818 | 0.286 | 0.2959 | Yes |
| 29 | NDUFS3 | NADH:ubiquinone oxidoreductase core subunit S3 [Source:HGNC Symbol;Acc:HGNC:7710] | 842 | 0.284 | 0.3049 | Yes |
| 30 | NDUFA3 | NADH:ubiquinone oxidoreductase subunit A3 [Source:HGNC Symbol;Acc:HGNC:7686] | 864 | 0.282 | 0.3139 | Yes |
| 31 | NDUFA7 | NADH:ubiquinone oxidoreductase subunit A7 [Source:HGNC Symbol;Acc:HGNC:7691] | 878 | 0.281 | 0.3231 | Yes |
| 32 | NDUFB9 | NADH:ubiquinone oxidoreductase subunit B9 [Source:HGNC Symbol;Acc:HGNC:7704] | 915 | 0.278 | 0.3316 | Yes |
| 33 | UQCRH | ubiquinol-cytochrome c reductase hinge protein [Source:HGNC Symbol;Acc:HGNC:12590] | 937 | 0.275 | 0.3403 | Yes |
| 34 | COX5B | cytochrome c oxidase subunit 5B [Source:HGNC Symbol;Acc:HGNC:2269] | 1019 | 0.268 | 0.3473 | Yes |
| 35 | COX4I1 | cytochrome c oxidase subunit 4I1 [Source:HGNC Symbol;Acc:HGNC:2265] | 1051 | 0.266 | 0.3555 | Yes |
| 36 | NDUFB1 | NADH:ubiquinone oxidoreductase subunit B1 [Source:HGNC Symbol;Acc:HGNC:7695] | 1087 | 0.263 | 0.3635 | Yes |
| 37 | ATP5MC2 | ATP synthase membrane subunit c locus 2 [Source:HGNC Symbol;Acc:HGNC:842] | 1094 | 0.263 | 0.3723 | Yes |
| 38 | COX8A | cytochrome c oxidase subunit 8A [Source:HGNC Symbol;Acc:HGNC:2294] | 1130 | 0.261 | 0.3802 | Yes |
| 39 | NDUFA8 | NADH:ubiquinone oxidoreductase subunit A8 [Source:HGNC Symbol;Acc:HGNC:7692] | 1138 | 0.261 | 0.3889 | Yes |
| 40 | NDUFB4 | NADH:ubiquinone oxidoreductase subunit B4 [Source:HGNC Symbol;Acc:HGNC:7699] | 1212 | 0.254 | 0.3956 | Yes |
| 41 | COX7C | cytochrome c oxidase subunit 7C [Source:HGNC Symbol;Acc:HGNC:2292] | 1244 | 0.252 | 0.4033 | Yes |
| 42 | PSENEN | "presenilin enhancer, gamma-secretase subunit [Source:HGNC Symbol;Acc:HGNC:30100]" | 1409 | 0.241 | 0.4073 | Yes |
| 43 | UQCRHL | ubiquinol-cytochrome c reductase hinge protein like [Source:HGNC Symbol;Acc:HGNC:51714] | 1439 | 0.239 | 0.4146 | Yes |
| 44 | ATP5F1E | ATP synthase F1 subunit epsilon [Source:HGNC Symbol;Acc:HGNC:838] | 1468 | 0.237 | 0.4219 | Yes |
| 45 | NDUFAB1 | NADH:ubiquinone oxidoreductase subunit AB1 [Source:HGNC Symbol;Acc:HGNC:7694] | 1579 | 0.230 | 0.4269 | Yes |
| 46 | MAPK3 | mitogen-activated protein kinase 3 [Source:HGNC Symbol;Acc:HGNC:6877] | 1645 | 0.226 | 0.4328 | Yes |
| 47 | COX6B1 | cytochrome c oxidase subunit 6B1 [Source:HGNC Symbol;Acc:HGNC:2280] | 1782 | 0.218 | 0.4367 | Yes |
| 48 | COX6A1 | cytochrome c oxidase subunit 6A1 [Source:HGNC Symbol;Acc:HGNC:2277] | 1827 | 0.216 | 0.4429 | Yes |
| 49 | SDHC | succinate dehydrogenase complex subunit C [Source:HGNC Symbol;Acc:HGNC:10682] | 2041 | 0.204 | 0.4444 | Yes |
| 50 | NDUFC2 | NADH:ubiquinone oxidoreductase subunit C2 [Source:HGNC Symbol;Acc:HGNC:7706] | 2151 | 0.199 | 0.4483 | Yes |
| 51 | NDUFA1 | NADH:ubiquinone oxidoreductase subunit A1 [Source:HGNC Symbol;Acc:HGNC:7683] | 2163 | 0.198 | 0.4547 | Yes |
| 52 | HSD17B10 | hydroxysteroid 17-beta dehydrogenase 10 [Source:HGNC Symbol;Acc:HGNC:4800] | 2183 | 0.197 | 0.4609 | Yes |
| 53 | CAPN1 | calpain 1 [Source:HGNC Symbol;Acc:HGNC:1476] | 2260 | 0.194 | 0.4655 | Yes |
| 54 | ATP5F1C | ATP synthase F1 subunit gamma [Source:HGNC Symbol;Acc:HGNC:833] | 2375 | 0.190 | 0.4691 | Yes |
| 55 | NDUFV2 | NADH:ubiquinone oxidoreductase core subunit V2 [Source:HGNC Symbol;Acc:HGNC:7717] | 2413 | 0.188 | 0.4745 | Yes |
| 56 | NDUFS2 | NADH:ubiquinone oxidoreductase core subunit S2 [Source:HGNC Symbol;Acc:HGNC:7708] | 2516 | 0.184 | 0.4781 | Yes |
| 57 | COX7B | cytochrome c oxidase subunit 7B [Source:HGNC Symbol;Acc:HGNC:2291] | 2582 | 0.181 | 0.4825 | Yes |
| 58 | MT-CO2 | mitochondrially encoded cytochrome c oxidase II [Source:HGNC Symbol;Acc:HGNC:7421] | 2703 | 0.176 | 0.4854 | Yes |
| 59 | PSEN2 | presenilin 2 [Source:HGNC Symbol;Acc:HGNC:9509] | 2723 | 0.175 | 0.4908 | Yes |
| 60 | COX7A2L | cytochrome c oxidase subunit 7A2 like [Source:HGNC Symbol;Acc:HGNC:2289] | 2825 | 0.171 | 0.4940 | Yes |
| 61 | UQCRB | ubiquinol-cytochrome c reductase binding protein [Source:HGNC Symbol;Acc:HGNC:12582] | 2840 | 0.170 | 0.4994 | Yes |
| 62 | COX6C | cytochrome c oxidase subunit 6C [Source:HGNC Symbol;Acc:HGNC:2285] | 2863 | 0.169 | 0.5046 | Yes |
| 63 | FADD | Fas associated via death domain [Source:HGNC Symbol;Acc:HGNC:3573] | 2883 | 0.169 | 0.5098 | Yes |
| 64 | MT-CO3 | mitochondrially encoded cytochrome c oxidase III [Source:HGNC Symbol;Acc:HGNC:7422] | 2913 | 0.167 | 0.5147 | Yes |
| 65 | NDUFA5 | NADH:ubiquinone oxidoreductase subunit A5 [Source:HGNC Symbol;Acc:HGNC:7688] | 3044 | 0.163 | 0.5169 | Yes |
| 66 | NDUFS4 | NADH:ubiquinone oxidoreductase subunit S4 [Source:HGNC Symbol;Acc:HGNC:7711] | 3070 | 0.162 | 0.5217 | Yes |
| 67 | NDUFB5 | NADH:ubiquinone oxidoreductase subunit B5 [Source:HGNC Symbol;Acc:HGNC:7700] | 3101 | 0.161 | 0.5264 | Yes |
| 68 | CASP9 | caspase 9 [Source:HGNC Symbol;Acc:HGNC:1511] | 3419 | 0.151 | 0.5234 | Yes |
| 69 | NDUFB3 | NADH:ubiquinone oxidoreductase subunit B3 [Source:HGNC Symbol;Acc:HGNC:7698] | 3463 | 0.150 | 0.5273 | Yes |
| 70 | CALML6 | calmodulin like 6 [Source:HGNC Symbol;Acc:HGNC:24193] | 3556 | 0.147 | 0.5299 | Yes |
| 71 | COX5A | cytochrome c oxidase subunit 5A [Source:HGNC Symbol;Acc:HGNC:2267] | 3688 | 0.142 | 0.5314 | Yes |
| 72 | COX7A2 | cytochrome c oxidase subunit 7A2 [Source:HGNC Symbol;Acc:HGNC:2288] | 3752 | 0.141 | 0.5345 | Yes |
| 73 | MT-CO1 | mitochondrially encoded cytochrome c oxidase I [Source:HGNC Symbol;Acc:HGNC:7419] | 3805 | 0.139 | 0.5379 | Yes |
| 74 | NDUFB6 | NADH:ubiquinone oxidoreductase subunit B6 [Source:HGNC Symbol;Acc:HGNC:7701] | 3968 | 0.134 | 0.5383 | Yes |
| 75 | SDHA | succinate dehydrogenase complex flavoprotein subunit A [Source:HGNC Symbol;Acc:HGNC:10680] | 4074 | 0.132 | 0.5401 | Yes |
| 76 | ATP5PB | ATP synthase peripheral stalk-membrane subunit b [Source:HGNC Symbol;Acc:HGNC:840] | 4227 | 0.128 | 0.5406 | Yes |
| 77 | NDUFA10 | NADH:ubiquinone oxidoreductase subunit A10 [Source:HGNC Symbol;Acc:HGNC:7684] | 4232 | 0.128 | 0.5448 | Yes |
| 78 | MT-CYB | mitochondrially encoded cytochrome b [Source:HGNC Symbol;Acc:HGNC:7427] | 4594 | 0.119 | 0.5396 | Yes |
| 79 | ATP5MC3 | ATP synthase membrane subunit c locus 3 [Source:HGNC Symbol;Acc:HGNC:843] | 4619 | 0.119 | 0.5430 | Yes |
| 80 | MT-ATP6 | mitochondrially encoded ATP synthase membrane subunit 6 [Source:HGNC Symbol;Acc:HGNC:7414] | 4718 | 0.116 | 0.5444 | Yes |
| 81 | NCSTN | nicastrin [Source:HGNC Symbol;Acc:HGNC:17091] | 4720 | 0.116 | 0.5484 | Yes |
| 82 | COX7A1 | cytochrome c oxidase subunit 7A1 [Source:HGNC Symbol;Acc:HGNC:2287] | 4947 | 0.112 | 0.5464 | Yes |
| 83 | PPP3CA | protein phosphatase 3 catalytic subunit alpha [Source:HGNC Symbol;Acc:HGNC:9314] | 5070 | 0.110 | 0.5470 | Yes |
| 84 | COX4I2 | cytochrome c oxidase subunit 4I2 [Source:HGNC Symbol;Acc:HGNC:16232] | 5116 | 0.109 | 0.5495 | Yes |
| 85 | UQCR10P1 | UQCR10 pseudogene 1 [Source:HGNC Symbol;Acc:HGNC:54960] | 5130 | 0.109 | 0.5529 | Yes |
| 86 | TNFRSF1A | TNF receptor superfamily member 1A [Source:HGNC Symbol;Acc:HGNC:11916] | 5380 | 0.104 | 0.5500 | Yes |
| 87 | CALM2 | calmodulin 2 [Source:HGNC Symbol;Acc:HGNC:1445] | 5478 | 0.102 | 0.5510 | Yes |
| 88 | COX6A2 | cytochrome c oxidase subunit 6A2 [Source:HGNC Symbol;Acc:HGNC:2279] | 5599 | 0.100 | 0.5513 | Yes |
| 89 | ATP5F1A | ATP synthase F1 subunit alpha [Source:HGNC Symbol;Acc:HGNC:823] | 5690 | 0.099 | 0.5524 | Yes |
| 90 | MAPT | microtubule associated protein tau [Source:HGNC Symbol;Acc:HGNC:6893] | 5834 | 0.096 | 0.5520 | Yes |
| 91 | SDHD | succinate dehydrogenase complex subunit D [Source:HGNC Symbol;Acc:HGNC:10683] | 5864 | 0.096 | 0.5545 | Yes |
| 92 | PLCB3 | phospholipase C beta 3 [Source:HGNC Symbol;Acc:HGNC:9056] | 6249 | 0.089 | 0.5477 | No |
| 93 | BID | BH3 interacting domain death agonist [Source:HGNC Symbol;Acc:HGNC:1050] | 6499 | 0.086 | 0.5442 | No |
| 94 | GAPDH | glyceraldehyde-3-phosphate dehydrogenase [Source:HGNC Symbol;Acc:HGNC:4141] | 6896 | 0.081 | 0.5369 | No |
| 95 | CHP1 | calcineurin like EF-hand protein 1 [Source:HGNC Symbol;Acc:HGNC:17433] | 7211 | 0.077 | 0.5314 | No |
| 96 | UQCRC2 | ubiquinol-cytochrome c reductase core protein 2 [Source:HGNC Symbol;Acc:HGNC:12586] | 7737 | 0.070 | 0.5204 | No |
| 97 | NDUFA9 | NADH:ubiquinone oxidoreductase subunit A9 [Source:HGNC Symbol;Acc:HGNC:7693] | 7750 | 0.070 | 0.5225 | No |
| 98 | CYCS | "cytochrome c, somatic [Source:HGNC Symbol;Acc:HGNC:19986]" | 8363 | 0.064 | 0.5090 | No |
| 99 | CAPN2 | calpain 2 [Source:HGNC Symbol;Acc:HGNC:1479] | 8442 | 0.063 | 0.5091 | No |
| 100 | CACNA1F | calcium voltage-gated channel subunit alpha1 F [Source:HGNC Symbol;Acc:HGNC:1393] | 8784 | 0.059 | 0.5024 | No |
| 101 | UQCRFS1 | "ubiquinol-cytochrome c reductase, Rieske iron-sulfur polypeptide 1 [Source:HGNC Symbol;Acc:HGNC:12587]" | 9310 | 0.055 | 0.4908 | No |
| 102 | MT-ATP8 | mitochondrially encoded ATP synthase membrane subunit 8 [Source:HGNC Symbol;Acc:HGNC:7415] | 9337 | 0.054 | 0.4920 | No |
| 103 | PPP3R1 | "protein phosphatase 3 regulatory subunit B, alpha [Source:HGNC Symbol;Acc:HGNC:9317]" | 10562 | 0.044 | 0.4623 | No |
| 104 | CHP2 | calcineurin like EF-hand protein 2 [Source:HGNC Symbol;Acc:HGNC:24927] | 13971 | 0.020 | 0.3759 | No |
| 105 | ATP2A1 | ATPase sarcoplasmic/endoplasmic reticulum Ca2+ transporting 1 [Source:HGNC Symbol;Acc:HGNC:811] | 14531 | 0.016 | 0.3622 | No |
| 106 | GRIN2A | glutamate ionotropic receptor NMDA type subunit 2A [Source:HGNC Symbol;Acc:HGNC:4585] | 15647 | 0.009 | 0.3340 | No |
| 107 | NDUFA4L2 | NDUFA4 mitochondrial complex associated like 2 [Source:HGNC Symbol;Acc:HGNC:29836] | 15776 | 0.008 | 0.3310 | No |
| 108 | GRIN1 | glutamate ionotropic receptor NMDA type subunit 1 [Source:HGNC Symbol;Acc:HGNC:4584] | 15818 | 0.008 | 0.3302 | No |
| 109 | CALML5 | calmodulin like 5 [Source:HGNC Symbol;Acc:HGNC:18180] | 16902 | 0.002 | 0.3026 | No |
| 110 | APBB1 | amyloid beta precursor protein binding family B member 1 [Source:HGNC Symbol;Acc:HGNC:581] | 17066 | 0.001 | 0.2985 | No |
| 111 | GRIN2C | glutamate ionotropic receptor NMDA type subunit 2C [Source:HGNC Symbol;Acc:HGNC:4587] | 17175 | 0.000 | 0.2958 | No |
| 112 | APP | amyloid beta precursor protein [Source:HGNC Symbol;Acc:HGNC:620] | 18528 | -0.008 | 0.2615 | No |
| 113 | COX8C | cytochrome c oxidase subunit 8C [Source:HGNC Symbol;Acc:HGNC:24382] | 20162 | -0.018 | 0.2204 | No |
| 114 | ATP5MC1P5 | ATP synthase membrane subunit c locus 1 pseudogene 5 [Source:HGNC Symbol;Acc:HGNC:39508] | 20955 | -0.022 | 0.2009 | No |
| 115 | COX6B2 | cytochrome c oxidase subunit 6B2 [Source:HGNC Symbol;Acc:HGNC:24380] | 21295 | -0.025 | 0.1931 | No |
| 116 | COX6CP3 | cytochrome c oxidase subunit 6C pseudogene 3 [Source:HGNC Symbol;Acc:HGNC:31721] | 21611 | -0.027 | 0.1859 | No |
| 117 | NAE1 | NEDD8 activating enzyme E1 subunit 1 [Source:HGNC Symbol;Acc:HGNC:621] | 22256 | -0.030 | 0.1705 | No |
| 118 | COX7B2 | cytochrome c oxidase subunit 7B2 [Source:HGNC Symbol;Acc:HGNC:24381] | 22662 | -0.033 | 0.1613 | No |
| 119 | FAS | Fas cell surface death receptor [Source:HGNC Symbol;Acc:HGNC:11920] | 23994 | -0.042 | 0.1287 | No |
| 120 | LPL | lipoprotein lipase [Source:HGNC Symbol;Acc:HGNC:6677] | 25782 | -0.054 | 0.0849 | No |
| 121 | CALM1 | calmodulin 1 [Source:HGNC Symbol;Acc:HGNC:1442] | 25939 | -0.055 | 0.0828 | No |
| 122 | BACE2 | beta-secretase 2 [Source:HGNC Symbol;Acc:HGNC:934] | 26441 | -0.059 | 0.0720 | No |
| 123 | GRIN2D | glutamate ionotropic receptor NMDA type subunit 2D [Source:HGNC Symbol;Acc:HGNC:4588] | 26595 | -0.060 | 0.0701 | No |
| 124 | CASP8 | caspase 8 [Source:HGNC Symbol;Acc:HGNC:1509] | 27243 | -0.065 | 0.0558 | No |
| 125 | PLCB4 | phospholipase C beta 4 [Source:HGNC Symbol;Acc:HGNC:9059] | 27781 | -0.069 | 0.0444 | No |
| 126 | APOE | apolipoprotein E [Source:HGNC Symbol;Acc:HGNC:613] | 27818 | -0.070 | 0.0459 | No |
| 127 | ATP5F1B | ATP synthase F1 subunit beta [Source:HGNC Symbol;Acc:HGNC:830] | 28627 | -0.077 | 0.0278 | No |
| 128 | CALML3 | calmodulin like 3 [Source:HGNC Symbol;Acc:HGNC:1452] | 29009 | -0.080 | 0.0208 | No |
| 129 | MME | membrane metalloendopeptidase [Source:HGNC Symbol;Acc:HGNC:7154] | 29524 | -0.085 | 0.0105 | No |
| 130 | GRIN2B | glutamate ionotropic receptor NMDA type subunit 2B [Source:HGNC Symbol;Acc:HGNC:4586] | 29542 | -0.085 | 0.0130 | No |
| 131 | PSEN1 | presenilin 1 [Source:HGNC Symbol;Acc:HGNC:9508] | 29819 | -0.088 | 0.0089 | No |
| 132 | CACNA1S | calcium voltage-gated channel subunit alpha1 S [Source:HGNC Symbol;Acc:HGNC:1397] | 30104 | -0.091 | 0.0047 | No |
| 133 | NDUFS1 | NADH:ubiquinone oxidoreductase core subunit S1 [Source:HGNC Symbol;Acc:HGNC:7707] | 30490 | -0.095 | -0.0019 | No |
| 134 | ATF6 | activating transcription factor 6 [Source:HGNC Symbol;Acc:HGNC:791] | 30612 | -0.096 | -0.0017 | No |
| 135 | CASP3 | caspase 3 [Source:HGNC Symbol;Acc:HGNC:1504] | 31110 | -0.102 | -0.0110 | No |
| 136 | ITPR3 | "inositol 1,4,5-trisphosphate receptor type 3 [Source:HGNC Symbol;Acc:HGNC:6182]" | 31781 | -0.110 | -0.0244 | No |
| 137 | CDK5R1 | cyclin dependent kinase 5 regulatory subunit 1 [Source:HGNC Symbol;Acc:HGNC:1775] | 32546 | -0.121 | -0.0398 | No |
| 138 | PPP3R2 | "protein phosphatase 3 regulatory subunit B, beta [Source:HGNC Symbol;Acc:HGNC:9318]" | 32695 | -0.123 | -0.0394 | No |
| 139 | PPP3CB | protein phosphatase 3 catalytic subunit beta [Source:HGNC Symbol;Acc:HGNC:9315] | 33217 | -0.131 | -0.0483 | No |
| 140 | NOS1 | nitric oxide synthase 1 [Source:HGNC Symbol;Acc:HGNC:7872] | 33519 | -0.137 | -0.0514 | No |
| 141 | CACNA1D | calcium voltage-gated channel subunit alpha1 D [Source:HGNC Symbol;Acc:HGNC:1391] | 33627 | -0.138 | -0.0494 | No |
| 142 | MAPK1 | mitogen-activated protein kinase 1 [Source:HGNC Symbol;Acc:HGNC:6871] | 33636 | -0.139 | -0.0450 | No |
| 143 | GNAQ | G protein subunit alpha q [Source:HGNC Symbol;Acc:HGNC:4390] | 33694 | -0.139 | -0.0417 | No |
| 144 | TNF | tumor necrosis factor [Source:HGNC Symbol;Acc:HGNC:11892] | 33732 | -0.140 | -0.0379 | No |
| 145 | RYR3 | ryanodine receptor 3 [Source:HGNC Symbol;Acc:HGNC:10485] | 33737 | -0.140 | -0.0333 | No |
| 146 | PLCB2 | phospholipase C beta 2 [Source:HGNC Symbol;Acc:HGNC:9055] | 33768 | -0.141 | -0.0293 | No |
| 147 | ITPR1 | "inositol 1,4,5-trisphosphate receptor type 1 [Source:HGNC Symbol;Acc:HGNC:6180]" | 33914 | -0.144 | -0.0281 | No |
| 148 | CASP7 | caspase 7 [Source:HGNC Symbol;Acc:HGNC:1508] | 34277 | -0.150 | -0.0323 | No |
| 149 | CALM3 | calmodulin 3 [Source:HGNC Symbol;Acc:HGNC:1449] | 34875 | -0.164 | -0.0420 | No |
| 150 | PPP3CC | protein phosphatase 3 catalytic subunit gamma [Source:HGNC Symbol;Acc:HGNC:9316] | 35754 | -0.186 | -0.0581 | No |
| 151 | BACE1 | beta-secretase 1 [Source:HGNC Symbol;Acc:HGNC:933] | 35864 | -0.190 | -0.0545 | No |
| 152 | ADAM17 | ADAM metallopeptidase domain 17 [Source:HGNC Symbol;Acc:HGNC:195] | 36082 | -0.196 | -0.0534 | No |
| 153 | PLCB1 | phospholipase C beta 1 [Source:HGNC Symbol;Acc:HGNC:15917] | 36397 | -0.208 | -0.0544 | No |
| 154 | IDE | insulin degrading enzyme [Source:HGNC Symbol;Acc:HGNC:5381] | 36437 | -0.209 | -0.0483 | No |
| 155 | SNCA | synuclein alpha [Source:HGNC Symbol;Acc:HGNC:11138] | 36532 | -0.212 | -0.0435 | No |
| 156 | ITPR2 | "inositol 1,4,5-trisphosphate receptor type 2 [Source:HGNC Symbol;Acc:HGNC:6181]" | 37066 | -0.239 | -0.0490 | No |
| 157 | IL1B | interleukin 1 beta [Source:HGNC Symbol;Acc:HGNC:5992] | 37201 | -0.248 | -0.0441 | No |
| 158 | ATP2A2 | ATPase sarcoplasmic/endoplasmic reticulum Ca2+ transporting 2 [Source:HGNC Symbol;Acc:HGNC:812] | 37541 | -0.269 | -0.0436 | No |
| 159 | ERN1 | endoplasmic reticulum to nucleus signaling 1 [Source:HGNC Symbol;Acc:HGNC:3449] | 37544 | -0.270 | -0.0346 | No |
| 160 | GSK3B | glycogen synthase kinase 3 beta [Source:HGNC Symbol;Acc:HGNC:4617] | 37592 | -0.274 | -0.0265 | No |
| 161 | CACNA1C | calcium voltage-gated channel subunit alpha1 C [Source:HGNC Symbol;Acc:HGNC:1390] | 37818 | -0.293 | -0.0224 | No |
| 162 | ATP2A3 | ATPase sarcoplasmic/endoplasmic reticulum Ca2+ transporting 3 [Source:HGNC Symbol;Acc:HGNC:813] | 37862 | -0.298 | -0.0134 | No |
| 163 | LRP1 | LDL receptor related protein 1 [Source:HGNC Symbol;Acc:HGNC:6692] | 38123 | -0.338 | -0.0086 | No |
| 164 | ADAM10 | ADAM metallopeptidase domain 10 [Source:HGNC Symbol;Acc:HGNC:188] | 38234 | -0.377 | 0.0013 | No |
| 165 | APAF1 | apoptotic peptidase activating factor 1 [Source:HGNC Symbol;Acc:HGNC:576] | 38254 | -0.386 | 0.0139 | No |
| 166 | EIF2AK3 | eukaryotic translation initiation factor 2 alpha kinase 3 [Source:HGNC Symbol;Acc:HGNC:3255] | 38278 | -0.396 | 0.0267 | No |
Table: GSEA details [plain text format]

  

Fig 2: KEGG\_ALZHEIMERS\_DISEASE      
 Blue-Pink O' Gram in the Space of the Analyzed GeneSet

  

Fig 3: KEGG\_ALZHEIMERS\_DISEASE: Random ES distribution      
 Gene set null distribution of ES for **KEGG\_ALZHEIMERS\_DISEASE**

  
