# Supplementary material for: Simultaneous Presentation of Multiple Myeloma and Lung Cancer: Case Report and Gene Bioinformatics Analysis
Source: Front Oncol. 2022 Jun 13;12:859735. doi: 10.3389/fonc.2022.859735 (PMC9235397; doi:10.3389/fonc.2022.859735)
Supplement: Supplementary file 1 [file DataSheet_1.zip › The bioinformatic analysis of MM and lung cancer supplementary materials/Enrichment analysis/MECR/GSEA_4.1.0/LUAD TCGA/KEGG.Gsea.1639041756227/KEGG_AMINOACYL_TRNA_BIOSYNTHESIS.html]

Details for gene set KEGG\_AMINOACYL\_TRNA\_BIOSYNTHESIS[GSEA]

|  || Dataset | ExpData\_collapsed\_to\_symbols.ENSG00000116353\_profile\_in\_ExpData.cls #ENSG00000116353 |
| Phenotype | ENSG00000116353\_profile\_in\_ExpData.cls#ENSG00000116353 |
| Upregulated in class | ENSG00000116353\_pos |
| GeneSet | KEGG\_AMINOACYL\_TRNA\_BIOSYNTHESIS |
| Enrichment Score (ES) | 0.52645665 |
| Normalized Enrichment Score (NES) | 1.8306179 |
| Nominal p-value | 0.002008032 |
| FDR q-value | 0.005976401 |
| FWER p-Value | 0.113 |
Table: GSEA Results Summary

  

Fig 1: Enrichment plot: KEGG\_AMINOACYL\_TRNA\_BIOSYNTHESIS      
 Profile of the Running ES Score & Positions of GeneSet Members on the Rank Ordered List

  

| SYMBOL | TITLE | RANK IN GENE LIST | RANK METRIC SCORE | RUNNING ES | CORE ENRICHMENT || 1 | TARS2 | "threonyl-tRNA synthetase 2, mitochondrial [Source:HGNC Symbol;Acc:HGNC:30740]" | 495 | 0.319 | 0.0486 | Yes |
| 2 | FARS2 | "phenylalanyl-tRNA synthetase 2, mitochondrial [Source:HGNC Symbol;Acc:HGNC:21062]" | 760 | 0.292 | 0.0979 | Yes |
| 3 | YARS1 | tyrosyl-tRNA synthetase 1 [Source:HGNC Symbol;Acc:HGNC:12840] | 770 | 0.291 | 0.1535 | Yes |
| 4 | QARS1 | glutaminyl-tRNA synthetase 1 [Source:HGNC Symbol;Acc:HGNC:9751] | 860 | 0.282 | 0.2054 | Yes |
| 5 | VARS2 | "valyl-tRNA synthetase 2, mitochondrial [Source:HGNC Symbol;Acc:HGNC:21642]" | 1131 | 0.261 | 0.2486 | Yes |
| 6 | PARS2 | "prolyl-tRNA synthetase 2, mitochondrial [Source:HGNC Symbol;Acc:HGNC:30563]" | 1152 | 0.259 | 0.2978 | Yes |
| 7 | CARS2 | "cysteinyl-tRNA synthetase 2, mitochondrial [Source:HGNC Symbol;Acc:HGNC:25695]" | 1934 | 0.210 | 0.3182 | Yes |
| 8 | FARSA | phenylalanyl-tRNA synthetase subunit alpha [Source:HGNC Symbol;Acc:HGNC:3592] | 2010 | 0.205 | 0.3556 | Yes |
| 9 | VARS1 | valyl-tRNA synthetase 1 [Source:HGNC Symbol;Acc:HGNC:12651] | 2637 | 0.179 | 0.3739 | Yes |
| 10 | FARSB | phenylalanyl-tRNA synthetase subunit beta [Source:HGNC Symbol;Acc:HGNC:17800] | 2669 | 0.177 | 0.4071 | Yes |
| 11 | EARS2 | "glutamyl-tRNA synthetase 2, mitochondrial [Source:HGNC Symbol;Acc:HGNC:29419]" | 3261 | 0.156 | 0.4219 | Yes |
| 12 | SARS2 | "seryl-tRNA synthetase 2, mitochondrial [Source:HGNC Symbol;Acc:HGNC:17697]" | 3278 | 0.155 | 0.4513 | Yes |
| 13 | HARS1 | histidyl-tRNA synthetase 1 [Source:HGNC Symbol;Acc:HGNC:4816] | 3478 | 0.149 | 0.4748 | Yes |
| 14 | PSTK | phosphoseryl-tRNA kinase [Source:HGNC Symbol;Acc:HGNC:28578] | 3505 | 0.148 | 0.5026 | Yes |
| 15 | WARS2 | "tryptophanyl tRNA synthetase 2, mitochondrial [Source:HGNC Symbol;Acc:HGNC:12730]" | 3955 | 0.135 | 0.5170 | Yes |
| 16 | KARS1 | lysyl-tRNA synthetase 1 [Source:HGNC Symbol;Acc:HGNC:6215] | 4745 | 0.116 | 0.5191 | Yes |
| 17 | HARS2 | "histidyl-tRNA synthetase 2, mitochondrial [Source:HGNC Symbol;Acc:HGNC:4817]" | 5624 | 0.100 | 0.5159 | Yes |
| 18 | AARS2 | "alanyl-tRNA synthetase 2, mitochondrial [Source:HGNC Symbol;Acc:HGNC:21022]" | 6152 | 0.091 | 0.5200 | Yes |
| 19 | SARS1 | seryl-tRNA synthetase 1 [Source:HGNC Symbol;Acc:HGNC:10537] | 7168 | 0.077 | 0.5090 | Yes |
| 20 | RARS1 | arginyl-tRNA synthetase 1 [Source:HGNC Symbol;Acc:HGNC:9870] | 7282 | 0.076 | 0.5206 | Yes |
| 21 | DARS1 | aspartyl-tRNA synthetase 1 [Source:HGNC Symbol;Acc:HGNC:2678] | 7593 | 0.072 | 0.5265 | Yes |
| 22 | NARS2 | "asparaginyl-tRNA synthetase 2, mitochondrial [Source:HGNC Symbol;Acc:HGNC:26274]" | 11476 | 0.037 | 0.4347 | No |
| 23 | GARS1 | glycyl-tRNA synthetase 1 [Source:HGNC Symbol;Acc:HGNC:4162] | 13192 | 0.024 | 0.3958 | No |
| 24 | LARS1 | leucyl-tRNA synthetase 1 [Source:HGNC Symbol;Acc:HGNC:6512] | 14952 | 0.013 | 0.3535 | No |
| 25 | MTFMT | mitochondrial methionyl-tRNA formyltransferase [Source:HGNC Symbol;Acc:HGNC:29666] | 15656 | 0.009 | 0.3374 | No |
| 26 | RARS2 | "arginyl-tRNA synthetase 2, mitochondrial [Source:HGNC Symbol;Acc:HGNC:21406]" | 18236 | -0.006 | 0.2729 | No |
| 27 | MARS2 | "methionyl-tRNA synthetase 2, mitochondrial [Source:HGNC Symbol;Acc:HGNC:25133]" | 19418 | -0.013 | 0.2454 | No |
| 28 | IARS2 | "isoleucyl-tRNA synthetase 2, mitochondrial [Source:HGNC Symbol;Acc:HGNC:29685]" | 19963 | -0.016 | 0.2347 | No |
| 29 | CARS1 | cysteinyl-tRNA synthetase 1 [Source:HGNC Symbol;Acc:HGNC:1493] | 20690 | -0.021 | 0.2202 | No |
| 30 | DARS2 | "aspartyl-tRNA synthetase 2, mitochondrial [Source:HGNC Symbol;Acc:HGNC:25538]" | 22314 | -0.031 | 0.1848 | No |
| 31 | SEPSECS | Sep (O-phosphoserine) tRNA:Sec (selenocysteine) tRNA synthase [Source:HGNC Symbol;Acc:HGNC:30605] | 25506 | -0.052 | 0.1136 | No |
| 32 | AARS1 | alanyl-tRNA synthetase 1 [Source:HGNC Symbol;Acc:HGNC:20] | 26200 | -0.057 | 0.1069 | No |
| 33 | YARS2 | tyrosyl-tRNA synthetase 2 [Source:HGNC Symbol;Acc:HGNC:24249] | 26243 | -0.057 | 0.1168 | No |
| 34 | EPRS1 | glutamyl-prolyl-tRNA synthetase 1 [Source:HGNC Symbol;Acc:HGNC:3418] | 31328 | -0.104 | 0.0074 | No |
| 35 | LARS2 | "leucyl-tRNA synthetase 2, mitochondrial [Source:HGNC Symbol;Acc:HGNC:17095]" | 31738 | -0.110 | 0.0180 | No |
| 36 | TARS1 | threonyl-tRNA synthetase 1 [Source:HGNC Symbol;Acc:HGNC:11572] | 33067 | -0.129 | 0.0089 | No |
| 37 | NARS1 | asparaginyl-tRNA synthetase 1 [Source:HGNC Symbol;Acc:HGNC:7643] | 33410 | -0.135 | 0.0261 | No |
| 38 | MARS1 | methionyl-tRNA synthetase 1 [Source:HGNC Symbol;Acc:HGNC:6898] | 33418 | -0.135 | 0.0518 | No |
| 39 | TARS3 | threonyl-tRNA synthetase 3 [Source:HGNC Symbol;Acc:HGNC:24728] | 33959 | -0.145 | 0.0658 | No |
| 40 | IARS1 | isoleucyl-tRNA synthetase 1 [Source:HGNC Symbol;Acc:HGNC:5330] | 35523 | -0.180 | 0.0604 | No |
| 41 | WARS1 | tryptophanyl-tRNA synthetase 1 [Source:HGNC Symbol;Acc:HGNC:12729] | 35856 | -0.189 | 0.0883 | No |
Table: GSEA details [plain text format]

  

Fig 2: KEGG\_AMINOACYL\_TRNA\_BIOSYNTHESIS      
 Blue-Pink O' Gram in the Space of the Analyzed GeneSet

  

Fig 3: KEGG\_AMINOACYL\_TRNA\_BIOSYNTHESIS: Random ES distribution      
 Gene set null distribution of ES for **KEGG\_AMINOACYL\_TRNA\_BIOSYNTHESIS**

  
