# Supplementary material for: Simultaneous Presentation of Multiple Myeloma and Lung Cancer: Case Report and Gene Bioinformatics Analysis
Source: Front Oncol. 2022 Jun 13;12:859735. doi: 10.3389/fonc.2022.859735 (PMC9235397; doi:10.3389/fonc.2022.859735)
Supplement: Supplementary file 1 [file DataSheet_1.zip › The bioinformatic analysis of MM and lung cancer supplementary materials/Enrichment analysis/MECR/GSEA_4.1.0/LUAD TCGA/KEGG.Gsea.1639041756227/KEGG_ARGININE_AND_PROLINE_METABOLISM.html]

Details for gene set KEGG\_ARGININE\_AND\_PROLINE\_METABOLISM[GSEA]

|  || Dataset | ExpData\_collapsed\_to\_symbols.ENSG00000116353\_profile\_in\_ExpData.cls #ENSG00000116353 |
| Phenotype | ENSG00000116353\_profile\_in\_ExpData.cls#ENSG00000116353 |
| Upregulated in class | ENSG00000116353\_pos |
| GeneSet | KEGG\_ARGININE\_AND\_PROLINE\_METABOLISM |
| Enrichment Score (ES) | 0.49666607 |
| Normalized Enrichment Score (NES) | 1.8076338 |
| Nominal p-value | 0.0 |
| FDR q-value | 0.0069216015 |
| FWER p-Value | 0.137 |
Table: GSEA Results Summary

  

Fig 1: Enrichment plot: KEGG\_ARGININE\_AND\_PROLINE\_METABOLISM      
 Profile of the Running ES Score & Positions of GeneSet Members on the Rank Ordered List

  

| SYMBOL | TITLE | RANK IN GENE LIST | RANK METRIC SCORE | RUNNING ES | CORE ENRICHMENT || 1 | ALDH4A1 | aldehyde dehydrogenase 4 family member A1 [Source:HGNC Symbol;Acc:HGNC:406] | 222 | 0.368 | 0.0517 | Yes |
| 2 | PYCR3 | pyrroline-5-carboxylate reductase 3 [Source:HGNC Symbol;Acc:HGNC:25846] | 241 | 0.362 | 0.1076 | Yes |
| 3 | SRM | spermidine synthase [Source:HGNC Symbol;Acc:HGNC:11296] | 367 | 0.338 | 0.1570 | Yes |
| 4 | GAMT | guanidinoacetate N-methyltransferase [Source:HGNC Symbol;Acc:HGNC:4136] | 593 | 0.308 | 0.1993 | Yes |
| 5 | CKB | creatine kinase B [Source:HGNC Symbol;Acc:HGNC:1991] | 663 | 0.301 | 0.2444 | Yes |
| 6 | PYCR2 | pyrroline-5-carboxylate reductase 2 [Source:HGNC Symbol;Acc:HGNC:30262] | 732 | 0.294 | 0.2884 | Yes |
| 7 | ACY1 | aminoacylase 1 [Source:HGNC Symbol;Acc:HGNC:177] | 868 | 0.282 | 0.3289 | Yes |
| 8 | ASL | argininosuccinate lyase [Source:HGNC Symbol;Acc:HGNC:746] | 1185 | 0.256 | 0.3607 | Yes |
| 9 | PYCR1 | pyrroline-5-carboxylate reductase 1 [Source:HGNC Symbol;Acc:HGNC:9721] | 1477 | 0.236 | 0.3900 | Yes |
| 10 | ASS1 | argininosuccinate synthase 1 [Source:HGNC Symbol;Acc:HGNC:758] | 1488 | 0.235 | 0.4264 | Yes |
| 11 | NAGS | N-acetylglutamate synthase [Source:HGNC Symbol;Acc:HGNC:17996] | 2007 | 0.205 | 0.4451 | Yes |
| 12 | ALDH7A1 | aldehyde dehydrogenase 7 family member A1 [Source:HGNC Symbol;Acc:HGNC:877] | 2018 | 0.205 | 0.4768 | Yes |
| 13 | AZIN2 | antizyme inhibitor 2 [Source:HGNC Symbol;Acc:HGNC:29957] | 2397 | 0.189 | 0.4966 | Yes |
| 14 | SAT2 | spermidine/spermine N1-acetyltransferase family member 2 [Source:HGNC Symbol;Acc:HGNC:23160] | 4297 | 0.127 | 0.4680 | Yes |
| 15 | ALDH2 | aldehyde dehydrogenase 2 family member [Source:HGNC Symbol;Acc:HGNC:404] | 4910 | 0.113 | 0.4699 | Yes |
| 16 | GOT2 | glutamic-oxaloacetic transaminase 2 [Source:HGNC Symbol;Acc:HGNC:4433] | 4966 | 0.112 | 0.4859 | Yes |
| 17 | ALDH3A2 | aldehyde dehydrogenase 3 family member A2 [Source:HGNC Symbol;Acc:HGNC:403] | 5202 | 0.108 | 0.4967 | Yes |
| 18 | GLUD1 | glutamate dehydrogenase 1 [Source:HGNC Symbol;Acc:HGNC:4335] | 7345 | 0.075 | 0.4538 | No |
| 19 | ALDH9A1 | aldehyde dehydrogenase 9 family member A1 [Source:HGNC Symbol;Acc:HGNC:412] | 7785 | 0.070 | 0.4535 | No |
| 20 | PRODH | proline dehydrogenase 1 [Source:HGNC Symbol;Acc:HGNC:9453] | 7979 | 0.068 | 0.4591 | No |
| 21 | MAOA | monoamine oxidase A [Source:HGNC Symbol;Acc:HGNC:6833] | 8946 | 0.058 | 0.4435 | No |
| 22 | P4HA2 | prolyl 4-hydroxylase subunit alpha 2 [Source:HGNC Symbol;Acc:HGNC:8547] | 9401 | 0.054 | 0.4403 | No |
| 23 | ODC1 | ornithine decarboxylase 1 [Source:HGNC Symbol;Acc:HGNC:8109] | 10765 | 0.043 | 0.4122 | No |
| 24 | CKMT1B | "creatine kinase, mitochondrial 1B [Source:HGNC Symbol;Acc:HGNC:1995]" | 10797 | 0.043 | 0.4181 | No |
| 25 | GLS2 | glutaminase 2 [Source:HGNC Symbol;Acc:HGNC:29570] | 11419 | 0.038 | 0.4081 | No |
| 26 | OTC | ornithine carbamoyltransferase [Source:HGNC Symbol;Acc:HGNC:8512] | 11631 | 0.036 | 0.4084 | No |
| 27 | CKM | "creatine kinase, M-type [Source:HGNC Symbol;Acc:HGNC:1994]" | 12252 | 0.031 | 0.3975 | No |
| 28 | ARG1 | arginase 1 [Source:HGNC Symbol;Acc:HGNC:663] | 13532 | 0.022 | 0.3683 | No |
| 29 | CKMT1A | "creatine kinase, mitochondrial 1A [Source:HGNC Symbol;Acc:HGNC:31736]" | 16270 | 0.005 | 0.2995 | No |
| 30 | NOS3 | nitric oxide synthase 3 [Source:HGNC Symbol;Acc:HGNC:7876] | 18404 | -0.007 | 0.2463 | No |
| 31 | SMS | spermine synthase [Source:HGNC Symbol;Acc:HGNC:11123] | 20548 | -0.020 | 0.1948 | No |
| 32 | P4HA3 | prolyl 4-hydroxylase subunit alpha 3 [Source:HGNC Symbol;Acc:HGNC:30135] | 21181 | -0.024 | 0.1825 | No |
| 33 | ALDH1B1 | aldehyde dehydrogenase 1 family member B1 [Source:HGNC Symbol;Acc:HGNC:407] | 21379 | -0.025 | 0.1814 | No |
| 34 | ARG2 | arginase 2 [Source:HGNC Symbol;Acc:HGNC:664] | 22043 | -0.029 | 0.1690 | No |
| 35 | AOC1 | amine oxidase copper containing 1 [Source:HGNC Symbol;Acc:HGNC:80] | 22193 | -0.030 | 0.1699 | No |
| 36 | GOT1 | glutamic-oxaloacetic transaminase 1 [Source:HGNC Symbol;Acc:HGNC:4432] | 22397 | -0.031 | 0.1696 | No |
| 37 | MAOB | monoamine oxidase B [Source:HGNC Symbol;Acc:HGNC:6834] | 22680 | -0.033 | 0.1675 | No |
| 38 | P4HA1 | prolyl 4-hydroxylase subunit alpha 1 [Source:HGNC Symbol;Acc:HGNC:8546] | 23505 | -0.039 | 0.1525 | No |
| 39 | PRODH2 | proline dehydrogenase 2 [Source:HGNC Symbol;Acc:HGNC:17325] | 23686 | -0.040 | 0.1541 | No |
| 40 | CKMT2 | "creatine kinase, mitochondrial 2 [Source:HGNC Symbol;Acc:HGNC:1996]" | 25511 | -0.052 | 0.1158 | No |
| 41 | OAT | ornithine aminotransferase [Source:HGNC Symbol;Acc:HGNC:8091] | 26534 | -0.060 | 0.0991 | No |
| 42 | ALDH18A1 | aldehyde dehydrogenase 18 family member A1 [Source:HGNC Symbol;Acc:HGNC:9722] | 26564 | -0.060 | 0.1077 | No |
| 43 | SAT1 | spermidine/spermine N1-acetyltransferase 1 [Source:HGNC Symbol;Acc:HGNC:10540] | 27557 | -0.068 | 0.0929 | No |
| 44 | GLS | glutaminase [Source:HGNC Symbol;Acc:HGNC:4331] | 28914 | -0.079 | 0.0707 | No |
| 45 | DAO | D-amino acid oxidase [Source:HGNC Symbol;Acc:HGNC:2671] | 29678 | -0.086 | 0.0647 | No |
| 46 | AMD1 | adenosylmethionine decarboxylase 1 [Source:HGNC Symbol;Acc:HGNC:457] | 30376 | -0.094 | 0.0615 | No |
| 47 | GLUL | glutamate-ammonia ligase [Source:HGNC Symbol;Acc:HGNC:4341] | 30845 | -0.099 | 0.0650 | No |
| 48 | GLUD2 | glutamate dehydrogenase 2 [Source:HGNC Symbol;Acc:HGNC:4336] | 31402 | -0.105 | 0.0672 | No |
| 49 | AGMAT | agmatinase [Source:HGNC Symbol;Acc:HGNC:18407] | 31595 | -0.108 | 0.0791 | No |
| 50 | GATM | glycine amidinotransferase [Source:HGNC Symbol;Acc:HGNC:4175] | 31994 | -0.113 | 0.0865 | No |
| 51 | NOS1 | nitric oxide synthase 1 [Source:HGNC Symbol;Acc:HGNC:7872] | 33519 | -0.137 | 0.0690 | No |
| 52 | NOS2 | nitric oxide synthase 2 [Source:HGNC Symbol;Acc:HGNC:7873] | 33997 | -0.145 | 0.0795 | No |
| 53 | CPS1 | carbamoyl-phosphate synthase 1 [Source:HGNC Symbol;Acc:HGNC:2323] | 34009 | -0.145 | 0.1018 | No |
| 54 | LAP3 | leucine aminopeptidase 3 [Source:HGNC Symbol;Acc:HGNC:18449] | 36597 | -0.215 | 0.0694 | No |
Table: GSEA details [plain text format]

  

Fig 2: KEGG\_ARGININE\_AND\_PROLINE\_METABOLISM      
 Blue-Pink O' Gram in the Space of the Analyzed GeneSet

  

Fig 3: KEGG\_ARGININE\_AND\_PROLINE\_METABOLISM: Random ES distribution      
 Gene set null distribution of ES for **KEGG\_ARGININE\_AND\_PROLINE\_METABOLISM**

  
