# Supplementary material for: Simultaneous Presentation of Multiple Myeloma and Lung Cancer: Case Report and Gene Bioinformatics Analysis
Source: Front Oncol. 2022 Jun 13;12:859735. doi: 10.3389/fonc.2022.859735 (PMC9235397; doi:10.3389/fonc.2022.859735)
Supplement: Supplementary file 1 [file DataSheet_1.zip › The bioinformatic analysis of MM and lung cancer supplementary materials/Enrichment analysis/MECR/GSEA_4.1.0/LUAD TCGA/KEGG.Gsea.1639041756227/KEGG_ARRHYTHMOGENIC_RIGHT_VENTRICULAR_CARDIOMYOPATHY_ARVC.html]

Details for gene set KEGG\_ARRHYTHMOGENIC\_RIGHT\_VENTRICULAR\_CARDIOMYOPATHY\_ARVC[GSEA]

|  || Dataset | ExpData\_collapsed\_to\_symbols.ENSG00000116353\_profile\_in\_ExpData.cls #ENSG00000116353 |
| Phenotype | ENSG00000116353\_profile\_in\_ExpData.cls#ENSG00000116353 |
| Upregulated in class | ENSG00000116353\_neg |
| GeneSet | KEGG\_ARRHYTHMOGENIC\_RIGHT\_VENTRICULAR\_CARDIOMYOPATHY\_ARVC |
| Enrichment Score (ES) | -0.5121624 |
| Normalized Enrichment Score (NES) | -2.0369885 |
| Nominal p-value | 0.0 |
| FDR q-value | 3.9498424E-4 |
| FWER p-Value | 0.006 |
Table: GSEA Results Summary

  

Fig 1: Enrichment plot: KEGG\_ARRHYTHMOGENIC\_RIGHT\_VENTRICULAR\_CARDIOMYOPATHY\_ARVC      
 Profile of the Running ES Score & Positions of GeneSet Members on the Rank Ordered List

  

| SYMBOL | TITLE | RANK IN GENE LIST | RANK METRIC SCORE | RUNNING ES | CORE ENRICHMENT || 1 | LMNA | lamin A/C [Source:HGNC Symbol;Acc:HGNC:6636] | 426 | 0.329 | 0.0226 | No |
| 2 | EMD | emerin [Source:HGNC Symbol;Acc:HGNC:3331] | 632 | 0.305 | 0.0483 | No |
| 3 | CACNB1 | calcium voltage-gated channel auxiliary subunit beta 1 [Source:HGNC Symbol;Acc:HGNC:1401] | 1361 | 0.244 | 0.0545 | No |
| 4 | ITGA3 | integrin subunit alpha 3 [Source:HGNC Symbol;Acc:HGNC:6139] | 3614 | 0.145 | 0.0118 | No |
| 5 | ACTB | actin beta [Source:HGNC Symbol;Acc:HGNC:132] | 4499 | 0.122 | 0.0016 | No |
| 6 | ACTG1 | actin gamma 1 [Source:HGNC Symbol;Acc:HGNC:144] | 5516 | 0.102 | -0.0139 | No |
| 7 | CACNB3 | calcium voltage-gated channel auxiliary subunit beta 3 [Source:HGNC Symbol;Acc:HGNC:1403] | 6872 | 0.081 | -0.0402 | No |
| 8 | CACNG1 | calcium voltage-gated channel auxiliary subunit gamma 1 [Source:HGNC Symbol;Acc:HGNC:1405] | 6964 | 0.080 | -0.0344 | No |
| 9 | JUP | junction plakoglobin [Source:HGNC Symbol;Acc:HGNC:6207] | 7299 | 0.076 | -0.0353 | No |
| 10 | TCF7L1 | transcription factor 7 like 1 [Source:HGNC Symbol;Acc:HGNC:11640] | 8097 | 0.066 | -0.0488 | No |
| 11 | ITGB4 | integrin subunit beta 4 [Source:HGNC Symbol;Acc:HGNC:6158] | 8518 | 0.062 | -0.0532 | No |
| 12 | CACNA1F | calcium voltage-gated channel subunit alpha1 F [Source:HGNC Symbol;Acc:HGNC:1393] | 8784 | 0.059 | -0.0539 | No |
| 13 | CTNNA1 | catenin alpha 1 [Source:HGNC Symbol;Acc:HGNC:2509] | 8967 | 0.058 | -0.0527 | No |
| 14 | ITGA2B | integrin subunit alpha 2b [Source:HGNC Symbol;Acc:HGNC:6138] | 9230 | 0.055 | -0.0538 | No |
| 15 | CACNG2 | calcium voltage-gated channel auxiliary subunit gamma 2 [Source:HGNC Symbol;Acc:HGNC:1406] | 9979 | 0.049 | -0.0678 | No |
| 16 | CACNG3 | calcium voltage-gated channel auxiliary subunit gamma 3 [Source:HGNC Symbol;Acc:HGNC:1407] | 10034 | 0.049 | -0.0643 | No |
| 17 | CACNG4 | calcium voltage-gated channel auxiliary subunit gamma 4 [Source:HGNC Symbol;Acc:HGNC:1408] | 10903 | 0.042 | -0.0822 | No |
| 18 | SGCA | sarcoglycan alpha [Source:HGNC Symbol;Acc:HGNC:10805] | 15330 | 0.011 | -0.1938 | No |
| 19 | DAG1 | dystroglycan 1 [Source:HGNC Symbol;Acc:HGNC:2666] | 16322 | 0.005 | -0.2185 | No |
| 20 | CACNA2D3 | calcium voltage-gated channel auxiliary subunit alpha2delta 3 [Source:HGNC Symbol;Acc:HGNC:15460] | 16587 | 0.004 | -0.2249 | No |
| 21 | ITGA7 | integrin subunit alpha 7 [Source:HGNC Symbol;Acc:HGNC:6143] | 18409 | -0.007 | -0.2706 | No |
| 22 | CTNNA2 | catenin alpha 2 [Source:HGNC Symbol;Acc:HGNC:2510] | 18724 | -0.009 | -0.2776 | No |
| 23 | ITGB6 | integrin subunit beta 6 [Source:HGNC Symbol;Acc:HGNC:6161] | 19027 | -0.011 | -0.2842 | No |
| 24 | ACTN4 | actinin alpha 4 [Source:HGNC Symbol;Acc:HGNC:166] | 19317 | -0.013 | -0.2903 | No |
| 25 | ITGA10 | integrin subunit alpha 10 [Source:HGNC Symbol;Acc:HGNC:6135] | 20447 | -0.019 | -0.3171 | No |
| 26 | CTNNA3 | catenin alpha 3 [Source:HGNC Symbol;Acc:HGNC:2511] | 20543 | -0.020 | -0.3175 | No |
| 27 | CACNA2D2 | calcium voltage-gated channel auxiliary subunit alpha2delta 2 [Source:HGNC Symbol;Acc:HGNC:1400] | 20662 | -0.021 | -0.3184 | No |
| 28 | ITGB5 | integrin subunit beta 5 [Source:HGNC Symbol;Acc:HGNC:6160] | 21391 | -0.025 | -0.3344 | No |
| 29 | CACNA2D4 | calcium voltage-gated channel auxiliary subunit alpha2delta 4 [Source:HGNC Symbol;Acc:HGNC:20202] | 22048 | -0.029 | -0.3481 | No |
| 30 | DSG2 | desmoglein 2 [Source:HGNC Symbol;Acc:HGNC:3049] | 24021 | -0.042 | -0.3941 | No |
| 31 | CACNG7 | calcium voltage-gated channel auxiliary subunit gamma 7 [Source:HGNC Symbol;Acc:HGNC:13626] | 24427 | -0.045 | -0.3999 | No |
| 32 | ITGA9 | integrin subunit alpha 9 [Source:HGNC Symbol;Acc:HGNC:6145] | 25285 | -0.051 | -0.4166 | No |
| 33 | DMD | dystrophin [Source:HGNC Symbol;Acc:HGNC:2928] | 26223 | -0.057 | -0.4346 | No |
| 34 | TCF7L2 | transcription factor 7 like 2 [Source:HGNC Symbol;Acc:HGNC:11641] | 26743 | -0.061 | -0.4416 | No |
| 35 | DES | desmin [Source:HGNC Symbol;Acc:HGNC:2770] | 27746 | -0.069 | -0.4601 | No |
| 36 | CACNG6 | calcium voltage-gated channel auxiliary subunit gamma 6 [Source:HGNC Symbol;Acc:HGNC:13625] | 28612 | -0.077 | -0.4744 | No |
| 37 | CTNNB1 | catenin beta 1 [Source:HGNC Symbol;Acc:HGNC:2514] | 29356 | -0.083 | -0.4849 | No |
| 38 | CACNA1S | calcium voltage-gated channel subunit alpha1 S [Source:HGNC Symbol;Acc:HGNC:1397] | 30104 | -0.091 | -0.4947 | No |
| 39 | DSC2 | desmocollin 2 [Source:HGNC Symbol;Acc:HGNC:3036] | 30406 | -0.094 | -0.4928 | No |
| 40 | ITGB8 | integrin subunit beta 8 [Source:HGNC Symbol;Acc:HGNC:6163] | 31035 | -0.101 | -0.4986 | No |
| 41 | ITGA11 | integrin subunit alpha 11 [Source:HGNC Symbol;Acc:HGNC:6136] | 31569 | -0.107 | -0.5013 | Yes |
| 42 | ACTN3 | actinin alpha 3 [Source:HGNC Symbol;Acc:HGNC:165] | 31627 | -0.108 | -0.4917 | Yes |
| 43 | SGCG | sarcoglycan gamma [Source:HGNC Symbol;Acc:HGNC:10809] | 31782 | -0.110 | -0.4844 | Yes |
| 44 | CACNG8 | calcium voltage-gated channel auxiliary subunit gamma 8 [Source:HGNC Symbol;Acc:HGNC:13628] | 31918 | -0.112 | -0.4765 | Yes |
| 45 | ITGB3 | integrin subunit beta 3 [Source:HGNC Symbol;Acc:HGNC:6156] | 32139 | -0.115 | -0.4705 | Yes |
| 46 | ACTN2 | actinin alpha 2 [Source:HGNC Symbol;Acc:HGNC:164] | 32475 | -0.120 | -0.4668 | Yes |
| 47 | ITGA8 | integrin subunit alpha 8 [Source:HGNC Symbol;Acc:HGNC:6144] | 32742 | -0.124 | -0.4611 | Yes |
| 48 | TCF7 | transcription factor 7 [Source:HGNC Symbol;Acc:HGNC:11639] | 32990 | -0.128 | -0.4544 | Yes |
| 49 | CACNG5 | calcium voltage-gated channel auxiliary subunit gamma 5 [Source:HGNC Symbol;Acc:HGNC:1409] | 33224 | -0.131 | -0.4470 | Yes |
| 50 | CACNB2 | calcium voltage-gated channel auxiliary subunit beta 2 [Source:HGNC Symbol;Acc:HGNC:1402] | 33228 | -0.131 | -0.4337 | Yes |
| 51 | CACNA1D | calcium voltage-gated channel subunit alpha1 D [Source:HGNC Symbol;Acc:HGNC:1391] | 33627 | -0.138 | -0.4298 | Yes |
| 52 | CACNB4 | calcium voltage-gated channel auxiliary subunit beta 4 [Source:HGNC Symbol;Acc:HGNC:1404] | 33661 | -0.139 | -0.4165 | Yes |
| 53 | SGCD | sarcoglycan delta [Source:HGNC Symbol;Acc:HGNC:10807] | 34456 | -0.154 | -0.4211 | Yes |
| 54 | DSP | desmoplakin [Source:HGNC Symbol;Acc:HGNC:3052] | 34749 | -0.161 | -0.4122 | Yes |
| 55 | ITGA5 | integrin subunit alpha 5 [Source:HGNC Symbol;Acc:HGNC:6141] | 34763 | -0.161 | -0.3961 | Yes |
| 56 | PKP2 | plakophilin 2 [Source:HGNC Symbol;Acc:HGNC:9024] | 35352 | -0.175 | -0.3933 | Yes |
| 57 | GJA1 | gap junction protein alpha 1 [Source:HGNC Symbol;Acc:HGNC:4274] | 35425 | -0.177 | -0.3771 | Yes |
| 58 | SGCB | sarcoglycan beta [Source:HGNC Symbol;Acc:HGNC:10806] | 35459 | -0.178 | -0.3599 | Yes |
| 59 | ITGA2 | integrin subunit alpha 2 [Source:HGNC Symbol;Acc:HGNC:6137] | 35833 | -0.189 | -0.3502 | Yes |
| 60 | ACTN1 | actinin alpha 1 [Source:HGNC Symbol;Acc:HGNC:163] | 36070 | -0.196 | -0.3363 | Yes |
| 61 | CDH2 | cadherin 2 [Source:HGNC Symbol;Acc:HGNC:1759] | 36615 | -0.216 | -0.3283 | Yes |
| 62 | ITGA6 | integrin subunit alpha 6 [Source:HGNC Symbol;Acc:HGNC:6142] | 37064 | -0.239 | -0.3154 | Yes |
| 63 | ITGA1 | integrin subunit alpha 1 [Source:HGNC Symbol;Acc:HGNC:6134] | 37309 | -0.254 | -0.2959 | Yes |
| 64 | LAMA2 | laminin subunit alpha 2 [Source:HGNC Symbol;Acc:HGNC:6482] | 37457 | -0.263 | -0.2729 | Yes |
| 65 | ATP2A2 | ATPase sarcoplasmic/endoplasmic reticulum Ca2+ transporting 2 [Source:HGNC Symbol;Acc:HGNC:812] | 37541 | -0.269 | -0.2477 | Yes |
| 66 | ITGAV | integrin subunit alpha V [Source:HGNC Symbol;Acc:HGNC:6150] | 37606 | -0.275 | -0.2213 | Yes |
| 67 | LEF1 | lymphoid enhancer binding factor 1 [Source:HGNC Symbol;Acc:HGNC:6551] | 37710 | -0.284 | -0.1951 | Yes |
| 68 | CACNA1C | calcium voltage-gated channel subunit alpha1 C [Source:HGNC Symbol;Acc:HGNC:1390] | 37818 | -0.293 | -0.1681 | Yes |
| 69 | ITGB1 | integrin subunit beta 1 [Source:HGNC Symbol;Acc:HGNC:6153] | 37850 | -0.297 | -0.1388 | Yes |
| 70 | RYR2 | ryanodine receptor 2 [Source:HGNC Symbol;Acc:HGNC:10484] | 37888 | -0.301 | -0.1092 | Yes |
| 71 | CACNA2D1 | calcium voltage-gated channel auxiliary subunit alpha2delta 1 [Source:HGNC Symbol;Acc:HGNC:1399] | 37977 | -0.313 | -0.0796 | Yes |
| 72 | ITGB7 | integrin subunit beta 7 [Source:HGNC Symbol;Acc:HGNC:6162] | 38154 | -0.347 | -0.0488 | Yes |
| 73 | SLC8A1 | solute carrier family 8 member A1 [Source:HGNC Symbol;Acc:HGNC:11068] | 38241 | -0.379 | -0.0125 | Yes |
| 74 | ITGA4 | integrin subunit alpha 4 [Source:HGNC Symbol;Acc:HGNC:6140] | 38275 | -0.394 | 0.0267 | Yes |
Table: GSEA details [plain text format]

  

Fig 2: KEGG\_ARRHYTHMOGENIC\_RIGHT\_VENTRICULAR\_CARDIOMYOPATHY\_ARVC      
 Blue-Pink O' Gram in the Space of the Analyzed GeneSet

  

Fig 3: KEGG\_ARRHYTHMOGENIC\_RIGHT\_VENTRICULAR\_CARDIOMYOPATHY\_ARVC: Random ES distribution      
 Gene set null distribution of ES for **KEGG\_ARRHYTHMOGENIC\_RIGHT\_VENTRICULAR\_CARDIOMYOPATHY\_ARVC**

  
