# Supplementary material for: Simultaneous Presentation of Multiple Myeloma and Lung Cancer: Case Report and Gene Bioinformatics Analysis
Source: Front Oncol. 2022 Jun 13;12:859735. doi: 10.3389/fonc.2022.859735 (PMC9235397; doi:10.3389/fonc.2022.859735)
Supplement: Supplementary file 1 [file DataSheet_1.zip › The bioinformatic analysis of MM and lung cancer supplementary materials/Enrichment analysis/MECR/GSEA_4.1.0/LUAD TCGA/KEGG.Gsea.1639041756227/KEGG_B_CELL_RECEPTOR_SIGNALING_PATHWAY.html]

Details for gene set KEGG\_B\_CELL\_RECEPTOR\_SIGNALING\_PATHWAY[GSEA]

|  || Dataset | ExpData\_collapsed\_to\_symbols.ENSG00000116353\_profile\_in\_ExpData.cls #ENSG00000116353 |
| Phenotype | ENSG00000116353\_profile\_in\_ExpData.cls#ENSG00000116353 |
| Upregulated in class | ENSG00000116353\_neg |
| GeneSet | KEGG\_B\_CELL\_RECEPTOR\_SIGNALING\_PATHWAY |
| Enrichment Score (ES) | -0.5312679 |
| Normalized Enrichment Score (NES) | -2.0979218 |
| Nominal p-value | 0.0 |
| FDR q-value | 2.1168761E-4 |
| FWER p-Value | 0.003 |
Table: GSEA Results Summary

  

Fig 1: Enrichment plot: KEGG\_B\_CELL\_RECEPTOR\_SIGNALING\_PATHWAY      
 Profile of the Running ES Score & Positions of GeneSet Members on the Rank Ordered List

  

| SYMBOL | TITLE | RANK IN GENE LIST | RANK METRIC SCORE | RUNNING ES | CORE ENRICHMENT || 1 | RAC3 | Rac family small GTPase 3 [Source:HGNC Symbol;Acc:HGNC:9803] | 528 | 0.316 | 0.0111 | No |
| 2 | PIK3R2 | phosphoinositide-3-kinase regulatory subunit 2 [Source:HGNC Symbol;Acc:HGNC:8980] | 545 | 0.314 | 0.0351 | No |
| 3 | HRAS | "HRas proto-oncogene, GTPase [Source:HGNC Symbol;Acc:HGNC:5173]" | 1513 | 0.234 | 0.0286 | No |
| 4 | MAPK3 | mitogen-activated protein kinase 3 [Source:HGNC Symbol;Acc:HGNC:6877] | 1645 | 0.226 | 0.0428 | No |
| 5 | MAP2K2 | mitogen-activated protein kinase kinase 2 [Source:HGNC Symbol;Acc:HGNC:6842] | 1791 | 0.218 | 0.0561 | No |
| 6 | CD81 | CD81 molecule [Source:HGNC Symbol;Acc:HGNC:1701] | 1808 | 0.217 | 0.0726 | No |
| 7 | RAC1 | Rac family small GTPase 1 [Source:HGNC Symbol;Acc:HGNC:9801] | 2566 | 0.182 | 0.0674 | No |
| 8 | NFATC4 | nuclear factor of activated T cells 4 [Source:HGNC Symbol;Acc:HGNC:7778] | 2871 | 0.169 | 0.0728 | No |
| 9 | AKT1 | AKT serine/threonine kinase 1 [Source:HGNC Symbol;Acc:HGNC:391] | 3179 | 0.158 | 0.0772 | No |
| 10 | JUN | "Jun proto-oncogene, AP-1 transcription factor subunit [Source:HGNC Symbol;Acc:HGNC:6204]" | 5045 | 0.110 | 0.0383 | No |
| 11 | PPP3CA | protein phosphatase 3 catalytic subunit alpha [Source:HGNC Symbol;Acc:HGNC:9314] | 5070 | 0.110 | 0.0462 | No |
| 12 | NFKBIE | NFKB inhibitor epsilon [Source:HGNC Symbol;Acc:HGNC:7799] | 5113 | 0.109 | 0.0536 | No |
| 13 | NFKBIB | NFKB inhibitor beta [Source:HGNC Symbol;Acc:HGNC:7798] | 5484 | 0.102 | 0.0522 | No |
| 14 | RELA | "RELA proto-oncogene, NF-kB subunit [Source:HGNC Symbol;Acc:HGNC:9955]" | 6022 | 0.093 | 0.0457 | No |
| 15 | CHP1 | calcineurin like EF-hand protein 1 [Source:HGNC Symbol;Acc:HGNC:17433] | 7211 | 0.077 | 0.0214 | No |
| 16 | NFKBIA | NFKB inhibitor alpha [Source:HGNC Symbol;Acc:HGNC:7797] | 7696 | 0.071 | 0.0146 | No |
| 17 | RAF1 | "Raf-1 proto-oncogene, serine/threonine kinase [Source:HGNC Symbol;Acc:HGNC:9829]" | 8635 | 0.061 | -0.0046 | No |
| 18 | PIK3R3 | phosphoinositide-3-kinase regulatory subunit 3 [Source:HGNC Symbol;Acc:HGNC:8981] | 9028 | 0.057 | -0.0102 | No |
| 19 | NFATC1 | nuclear factor of activated T cells 1 [Source:HGNC Symbol;Acc:HGNC:7775] | 9184 | 0.056 | -0.0098 | No |
| 20 | IKBKG | inhibitor of nuclear factor kappa B kinase regulatory subunit gamma [Source:HGNC Symbol;Acc:HGNC:5961] | 9304 | 0.055 | -0.0086 | No |
| 21 | PPP3R1 | "protein phosphatase 3 regulatory subunit B, alpha [Source:HGNC Symbol;Acc:HGNC:9317]" | 10562 | 0.044 | -0.0371 | No |
| 22 | AKT2 | AKT serine/threonine kinase 2 [Source:HGNC Symbol;Acc:HGNC:392] | 10579 | 0.044 | -0.0341 | No |
| 23 | CHP2 | calcineurin like EF-hand protein 2 [Source:HGNC Symbol;Acc:HGNC:24927] | 13971 | 0.020 | -0.1190 | No |
| 24 | BCL10 | BCL10 immune signaling adaptor [Source:HGNC Symbol;Acc:HGNC:989] | 18926 | -0.010 | -0.2444 | No |
| 25 | IKBKB | inhibitor of nuclear factor kappa B kinase subunit beta [Source:HGNC Symbol;Acc:HGNC:5960] | 21393 | -0.025 | -0.3053 | No |
| 26 | IFITM1 | interferon induced transmembrane protein 1 [Source:HGNC Symbol;Acc:HGNC:5412] | 22223 | -0.030 | -0.3240 | No |
| 27 | FOS | "Fos proto-oncogene, AP-1 transcription factor subunit [Source:HGNC Symbol;Acc:HGNC:3796]" | 23563 | -0.039 | -0.3551 | No |
| 28 | PTPN6 | protein tyrosine phosphatase non-receptor type 6 [Source:HGNC Symbol;Acc:HGNC:9658] | 23581 | -0.039 | -0.3525 | No |
| 29 | RAC2 | Rac family small GTPase 2 [Source:HGNC Symbol;Acc:HGNC:9802] | 24228 | -0.043 | -0.3656 | No |
| 30 | VAV2 | vav guanine nucleotide exchange factor 2 [Source:HGNC Symbol;Acc:HGNC:12658] | 24578 | -0.046 | -0.3710 | No |
| 31 | CARD11 | caspase recruitment domain family member 11 [Source:HGNC Symbol;Acc:HGNC:16393] | 27575 | -0.068 | -0.4420 | No |
| 32 | LYN | "LYN proto-oncogene, Src family tyrosine kinase [Source:HGNC Symbol;Acc:HGNC:6735]" | 28456 | -0.075 | -0.4586 | No |
| 33 | VAV3 | vav guanine nucleotide exchange factor 3 [Source:HGNC Symbol;Acc:HGNC:12659] | 29000 | -0.080 | -0.4663 | No |
| 34 | CR2 | complement C3d receptor 2 [Source:HGNC Symbol;Acc:HGNC:2336] | 30834 | -0.099 | -0.5053 | No |
| 35 | CD79B | CD79b molecule [Source:HGNC Symbol;Acc:HGNC:1699] | 31854 | -0.111 | -0.5226 | Yes |
| 36 | PIK3CD | "phosphatidylinositol-4,5-bisphosphate 3-kinase catalytic subunit delta [Source:HGNC Symbol;Acc:HGNC:8977]" | 31859 | -0.111 | -0.5141 | Yes |
| 37 | BLNK | B cell linker [Source:HGNC Symbol;Acc:HGNC:14211] | 31998 | -0.113 | -0.5088 | Yes |
| 38 | PPP3R2 | "protein phosphatase 3 regulatory subunit B, beta [Source:HGNC Symbol;Acc:HGNC:9318]" | 32695 | -0.123 | -0.5170 | Yes |
| 39 | DAPP1 | dual adaptor of phosphotyrosine and 3-phosphoinositides 1 [Source:HGNC Symbol;Acc:HGNC:16500] | 32824 | -0.125 | -0.5105 | Yes |
| 40 | PPP3CB | protein phosphatase 3 catalytic subunit beta [Source:HGNC Symbol;Acc:HGNC:9315] | 33217 | -0.131 | -0.5103 | Yes |
| 41 | MAPK1 | mitogen-activated protein kinase 1 [Source:HGNC Symbol;Acc:HGNC:6871] | 33636 | -0.139 | -0.5102 | Yes |
| 42 | CD22 | CD22 molecule [Source:HGNC Symbol;Acc:HGNC:1643] | 33891 | -0.143 | -0.5055 | Yes |
| 43 | NRAS | "NRAS proto-oncogene, GTPase [Source:HGNC Symbol;Acc:HGNC:7989]" | 34553 | -0.156 | -0.5102 | Yes |
| 44 | SOS2 | SOS Ras/Rho guanine nucleotide exchange factor 2 [Source:HGNC Symbol;Acc:HGNC:11188] | 34598 | -0.157 | -0.4992 | Yes |
| 45 | PIK3R1 | phosphoinositide-3-kinase regulatory subunit 1 [Source:HGNC Symbol;Acc:HGNC:8979] | 34788 | -0.162 | -0.4914 | Yes |
| 46 | VAV1 | vav guanine nucleotide exchange factor 1 [Source:HGNC Symbol;Acc:HGNC:12657] | 34853 | -0.163 | -0.4804 | Yes |
| 47 | PIK3CB | "phosphatidylinositol-4,5-bisphosphate 3-kinase catalytic subunit beta [Source:HGNC Symbol;Acc:HGNC:8976]" | 34871 | -0.164 | -0.4681 | Yes |
| 48 | MAP2K1 | mitogen-activated protein kinase kinase 1 [Source:HGNC Symbol;Acc:HGNC:6840] | 35210 | -0.171 | -0.4634 | Yes |
| 49 | CD72 | CD72 molecule [Source:HGNC Symbol;Acc:HGNC:1696] | 35740 | -0.186 | -0.4624 | Yes |
| 50 | PPP3CC | protein phosphatase 3 catalytic subunit gamma [Source:HGNC Symbol;Acc:HGNC:9316] | 35754 | -0.186 | -0.4483 | Yes |
| 51 | MALT1 | MALT1 paracaspase [Source:HGNC Symbol;Acc:HGNC:6819] | 35793 | -0.188 | -0.4347 | Yes |
| 52 | CD19 | CD19 molecule [Source:HGNC Symbol;Acc:HGNC:1633] | 35881 | -0.190 | -0.4221 | Yes |
| 53 | CD79A | CD79a molecule [Source:HGNC Symbol;Acc:HGNC:1698] | 35992 | -0.194 | -0.4099 | Yes |
| 54 | FCGR2B | Fc fragment of IgG receptor IIb [Source:HGNC Symbol;Acc:HGNC:3618] | 36001 | -0.194 | -0.3950 | Yes |
| 55 | NFKB1 | nuclear factor kappa B subunit 1 [Source:HGNC Symbol;Acc:HGNC:7794] | 36367 | -0.207 | -0.3882 | Yes |
| 56 | GRB2 | growth factor receptor bound protein 2 [Source:HGNC Symbol;Acc:HGNC:4566] | 36516 | -0.212 | -0.3755 | Yes |
| 57 | NFATC2 | nuclear factor of activated T cells 2 [Source:HGNC Symbol;Acc:HGNC:7776] | 36732 | -0.221 | -0.3638 | Yes |
| 58 | BTK | Bruton tyrosine kinase [Source:HGNC Symbol;Acc:HGNC:1133] | 37120 | -0.243 | -0.3548 | Yes |
| 59 | INPP5D | inositol polyphosphate-5-phosphatase D [Source:HGNC Symbol;Acc:HGNC:6079] | 37464 | -0.264 | -0.3431 | Yes |
| 60 | PIK3CA | "phosphatidylinositol-4,5-bisphosphate 3-kinase catalytic subunit alpha [Source:HGNC Symbol;Acc:HGNC:8975]" | 37556 | -0.270 | -0.3244 | Yes |
| 61 | GSK3B | glycogen synthase kinase 3 beta [Source:HGNC Symbol;Acc:HGNC:4617] | 37592 | -0.274 | -0.3040 | Yes |
| 62 | KRAS | "KRAS proto-oncogene, GTPase [Source:HGNC Symbol;Acc:HGNC:6407]" | 37629 | -0.277 | -0.2834 | Yes |
| 63 | NFAT5 | nuclear factor of activated T cells 5 [Source:HGNC Symbol;Acc:HGNC:7774] | 37631 | -0.277 | -0.2619 | Yes |
| 64 | NFATC3 | nuclear factor of activated T cells 3 [Source:HGNC Symbol;Acc:HGNC:7777] | 37672 | -0.281 | -0.2411 | Yes |
| 65 | SYK | spleen associated tyrosine kinase [Source:HGNC Symbol;Acc:HGNC:11491] | 37729 | -0.285 | -0.2203 | Yes |
| 66 | CHUK | component of inhibitor of nuclear factor kappa B kinase complex [Source:HGNC Symbol;Acc:HGNC:1974] | 37762 | -0.287 | -0.1989 | Yes |
| 67 | PRKCB | protein kinase C beta [Source:HGNC Symbol;Acc:HGNC:9395] | 37822 | -0.293 | -0.1776 | Yes |
| 68 | LILRB3 | leukocyte immunoglobulin like receptor B3 [Source:HGNC Symbol;Acc:HGNC:6607] | 37852 | -0.297 | -0.1553 | Yes |
| 69 | PIK3R5 | phosphoinositide-3-kinase regulatory subunit 5 [Source:HGNC Symbol;Acc:HGNC:30035] | 37956 | -0.310 | -0.1338 | Yes |
| 70 | RASGRP3 | RAS guanyl releasing protein 3 [Source:HGNC Symbol;Acc:HGNC:14545] | 38091 | -0.331 | -0.1115 | Yes |
| 71 | PLCG2 | phospholipase C gamma 2 [Source:HGNC Symbol;Acc:HGNC:9066] | 38119 | -0.338 | -0.0859 | Yes |
| 72 | SOS1 | SOS Ras/Rac guanine nucleotide exchange factor 1 [Source:HGNC Symbol;Acc:HGNC:11187] | 38132 | -0.341 | -0.0597 | Yes |
| 73 | AKT3 | AKT serine/threonine kinase 3 [Source:HGNC Symbol;Acc:HGNC:393] | 38196 | -0.362 | -0.0332 | Yes |
| 74 | PIK3AP1 | phosphoinositide-3-kinase adaptor protein 1 [Source:HGNC Symbol;Acc:HGNC:30034] | 38213 | -0.370 | -0.0048 | Yes |
| 75 | PIK3CG | "phosphatidylinositol-4,5-bisphosphate 3-kinase catalytic subunit gamma [Source:HGNC Symbol;Acc:HGNC:8978]" | 38302 | -0.426 | 0.0260 | Yes |
Table: GSEA details [plain text format]

  

Fig 2: KEGG\_B\_CELL\_RECEPTOR\_SIGNALING\_PATHWAY      
 Blue-Pink O' Gram in the Space of the Analyzed GeneSet

  

Fig 3: KEGG\_B\_CELL\_RECEPTOR\_SIGNALING\_PATHWAY: Random ES distribution      
 Gene set null distribution of ES for **KEGG\_B\_CELL\_RECEPTOR\_SIGNALING\_PATHWAY**

  
