# Supplementary material for: Simultaneous Presentation of Multiple Myeloma and Lung Cancer: Case Report and Gene Bioinformatics Analysis
Source: Front Oncol. 2022 Jun 13;12:859735. doi: 10.3389/fonc.2022.859735 (PMC9235397; doi:10.3389/fonc.2022.859735)
Supplement: Supplementary file 1 [file DataSheet_1.zip › The bioinformatic analysis of MM and lung cancer supplementary materials/Enrichment analysis/MECR/GSEA_4.1.0/LUAD TCGA/KEGG.Gsea.1639041756227/KEGG_CALCIUM_SIGNALING_PATHWAY.html]

Details for gene set KEGG\_CALCIUM\_SIGNALING\_PATHWAY[GSEA]

|  || Dataset | ExpData\_collapsed\_to\_symbols.ENSG00000116353\_profile\_in\_ExpData.cls #ENSG00000116353 |
| Phenotype | ENSG00000116353\_profile\_in\_ExpData.cls#ENSG00000116353 |
| Upregulated in class | ENSG00000116353\_neg |
| GeneSet | KEGG\_CALCIUM\_SIGNALING\_PATHWAY |
| Enrichment Score (ES) | -0.46590987 |
| Normalized Enrichment Score (NES) | -2.1116655 |
| Nominal p-value | 0.0 |
| FDR q-value | 2.3991261E-4 |
| FWER p-Value | 0.003 |
Table: GSEA Results Summary

  

Fig 1: Enrichment plot: KEGG\_CALCIUM\_SIGNALING\_PATHWAY      
 Profile of the Running ES Score & Positions of GeneSet Members on the Rank Ordered List

  

| SYMBOL | TITLE | RANK IN GENE LIST | RANK METRIC SCORE | RUNNING ES | CORE ENRICHMENT || 1 | PHKG2 | phosphorylase kinase catalytic subunit gamma 2 [Source:HGNC Symbol;Acc:HGNC:8931] | 345 | 0.342 | 0.0076 | No |
| 2 | PLCD1 | phospholipase C delta 1 [Source:HGNC Symbol;Acc:HGNC:9060] | 1410 | 0.241 | -0.0080 | No |
| 3 | GNAS | GNAS complex locus [Source:HGNC Symbol;Acc:HGNC:4392] | 2243 | 0.195 | -0.0199 | No |
| 4 | SLC25A4 | solute carrier family 25 member 4 [Source:HGNC Symbol;Acc:HGNC:10990] | 2531 | 0.183 | -0.0184 | No |
| 5 | SLC25A6 | solute carrier family 25 member 6 [Source:HGNC Symbol;Acc:HGNC:10992] | 3062 | 0.162 | -0.0241 | No |
| 6 | TNNC1 | "troponin C1, slow skeletal and cardiac type [Source:HGNC Symbol;Acc:HGNC:11943]" | 3100 | 0.161 | -0.0174 | No |
| 7 | CAMK2G | calcium/calmodulin dependent protein kinase II gamma [Source:HGNC Symbol;Acc:HGNC:1463] | 3282 | 0.155 | -0.0145 | No |
| 8 | ADORA2B | adenosine A2b receptor [Source:HGNC Symbol;Acc:HGNC:264] | 3536 | 0.147 | -0.0139 | No |
| 9 | CALML6 | calmodulin like 6 [Source:HGNC Symbol;Acc:HGNC:24193] | 3556 | 0.147 | -0.0074 | No |
| 10 | LHCGR | luteinizing hormone/choriogonadotropin receptor [Source:HGNC Symbol;Acc:HGNC:6585] | 3697 | 0.142 | -0.0041 | No |
| 11 | PHKG1 | phosphorylase kinase catalytic subunit gamma 1 [Source:HGNC Symbol;Acc:HGNC:8930] | 3912 | 0.136 | -0.0030 | No |
| 12 | ERBB2 | erb-b2 receptor tyrosine kinase 2 [Source:HGNC Symbol;Acc:HGNC:3430] | 4642 | 0.118 | -0.0159 | No |
| 13 | SLC25A5 | solute carrier family 25 member 5 [Source:HGNC Symbol;Acc:HGNC:10991] | 4760 | 0.116 | -0.0134 | No |
| 14 | PPP3CA | protein phosphatase 3 catalytic subunit alpha [Source:HGNC Symbol;Acc:HGNC:9314] | 5070 | 0.110 | -0.0160 | No |
| 15 | GNA15 | G protein subunit alpha 15 [Source:HGNC Symbol;Acc:HGNC:4383] | 5313 | 0.105 | -0.0171 | No |
| 16 | CALM2 | calmodulin 2 [Source:HGNC Symbol;Acc:HGNC:1445] | 5478 | 0.102 | -0.0164 | No |
| 17 | PLCB3 | phospholipase C beta 3 [Source:HGNC Symbol;Acc:HGNC:9056] | 6249 | 0.089 | -0.0317 | No |
| 18 | PRKACA | protein kinase cAMP-activated catalytic subunit alpha [Source:HGNC Symbol;Acc:HGNC:9380] | 6303 | 0.089 | -0.0288 | No |
| 19 | TNNC2 | "troponin C2, fast skeletal type [Source:HGNC Symbol;Acc:HGNC:11944]" | 6589 | 0.085 | -0.0320 | No |
| 20 | CAMK2D | calcium/calmodulin dependent protein kinase II delta [Source:HGNC Symbol;Acc:HGNC:1462] | 7201 | 0.077 | -0.0440 | No |
| 21 | CHP1 | calcineurin like EF-hand protein 1 [Source:HGNC Symbol;Acc:HGNC:17433] | 7211 | 0.077 | -0.0405 | No |
| 22 | ITPKA | inositol-trisphosphate 3-kinase A [Source:HGNC Symbol;Acc:HGNC:6178] | 7224 | 0.076 | -0.0372 | No |
| 23 | ADRA1B | adrenoceptor alpha 1B [Source:HGNC Symbol;Acc:HGNC:278] | 8272 | 0.065 | -0.0608 | No |
| 24 | SLC8A2 | solute carrier family 8 member A2 [Source:HGNC Symbol;Acc:HGNC:11069] | 8407 | 0.063 | -0.0612 | No |
| 25 | CACNA1F | calcium voltage-gated channel subunit alpha1 F [Source:HGNC Symbol;Acc:HGNC:1393] | 8784 | 0.059 | -0.0679 | No |
| 26 | PHKB | phosphorylase kinase regulatory subunit beta [Source:HGNC Symbol;Acc:HGNC:8927] | 9058 | 0.057 | -0.0722 | No |
| 27 | ADRB1 | adrenoceptor beta 1 [Source:HGNC Symbol;Acc:HGNC:285] | 9260 | 0.055 | -0.0747 | No |
| 28 | P2RX4 | purinergic receptor P2X 4 [Source:HGNC Symbol;Acc:HGNC:8535] | 9648 | 0.052 | -0.0821 | No |
| 29 | EGFR | epidermal growth factor receptor [Source:HGNC Symbol;Acc:HGNC:3236] | 9728 | 0.051 | -0.0816 | No |
| 30 | VDAC2 | voltage dependent anion channel 2 [Source:HGNC Symbol;Acc:HGNC:12672] | 10201 | 0.047 | -0.0914 | No |
| 31 | PPP3R1 | "protein phosphatase 3 regulatory subunit B, alpha [Source:HGNC Symbol;Acc:HGNC:9317]" | 10562 | 0.044 | -0.0985 | No |
| 32 | SLC25A31 | solute carrier family 25 member 31 [Source:HGNC Symbol;Acc:HGNC:25319] | 10585 | 0.044 | -0.0969 | No |
| 33 | RYR1 | ryanodine receptor 1 [Source:HGNC Symbol;Acc:HGNC:10483] | 11134 | 0.040 | -0.1090 | No |
| 34 | ADRA1D | adrenoceptor alpha 1D [Source:HGNC Symbol;Acc:HGNC:280] | 11153 | 0.040 | -0.1075 | No |
| 35 | TACR2 | tachykinin receptor 2 [Source:HGNC Symbol;Acc:HGNC:11527] | 11248 | 0.039 | -0.1081 | No |
| 36 | CCKBR | cholecystokinin B receptor [Source:HGNC Symbol;Acc:HGNC:1571] | 11320 | 0.038 | -0.1080 | No |
| 37 | VDAC2P5 | VDAC2 pseudogene 5 [Source:HGNC Symbol;Acc:HGNC:54753] | 11481 | 0.037 | -0.1104 | No |
| 38 | PTGER1 | prostaglandin E receptor 1 [Source:HGNC Symbol;Acc:HGNC:9593] | 11693 | 0.036 | -0.1140 | No |
| 39 | CHRM1 | cholinergic receptor muscarinic 1 [Source:HGNC Symbol;Acc:HGNC:1950] | 13298 | 0.024 | -0.1539 | No |
| 40 | ERBB3 | erb-b2 receptor tyrosine kinase 3 [Source:HGNC Symbol;Acc:HGNC:3431] | 13616 | 0.022 | -0.1609 | No |
| 41 | CHP2 | calcineurin like EF-hand protein 2 [Source:HGNC Symbol;Acc:HGNC:24927] | 13971 | 0.020 | -0.1690 | No |
| 42 | ADRB3 | adrenoceptor beta 3 [Source:HGNC Symbol;Acc:HGNC:288] | 14065 | 0.019 | -0.1705 | No |
| 43 | ATP2A1 | ATPase sarcoplasmic/endoplasmic reticulum Ca2+ transporting 1 [Source:HGNC Symbol;Acc:HGNC:811] | 14531 | 0.016 | -0.1816 | No |
| 44 | P2RX6 | purinergic receptor P2X 6 [Source:HGNC Symbol;Acc:HGNC:8538] | 14690 | 0.015 | -0.1849 | No |
| 45 | ADCY2 | adenylate cyclase 2 [Source:HGNC Symbol;Acc:HGNC:233] | 14873 | 0.014 | -0.1889 | No |
| 46 | TBXA2R | thromboxane A2 receptor [Source:HGNC Symbol;Acc:HGNC:11608] | 14991 | 0.013 | -0.1913 | No |
| 47 | SPHK2 | sphingosine kinase 2 [Source:HGNC Symbol;Acc:HGNC:18859] | 15118 | 0.012 | -0.1939 | No |
| 48 | GRIN2A | glutamate ionotropic receptor NMDA type subunit 2A [Source:HGNC Symbol;Acc:HGNC:4585] | 15647 | 0.009 | -0.2069 | No |
| 49 | GRIN1 | glutamate ionotropic receptor NMDA type subunit 1 [Source:HGNC Symbol;Acc:HGNC:4584] | 15818 | 0.008 | -0.2109 | No |
| 50 | GNA11 | G protein subunit alpha 11 [Source:HGNC Symbol;Acc:HGNC:4379] | 15941 | 0.007 | -0.2137 | No |
| 51 | CALML5 | calmodulin like 5 [Source:HGNC Symbol;Acc:HGNC:18180] | 16902 | 0.002 | -0.2381 | No |
| 52 | GRIN2C | glutamate ionotropic receptor NMDA type subunit 2C [Source:HGNC Symbol;Acc:HGNC:4587] | 17175 | 0.000 | -0.2450 | No |
| 53 | P2RX3 | purinergic receptor P2X 3 [Source:HGNC Symbol;Acc:HGNC:8534] | 18041 | -0.005 | -0.2669 | No |
| 54 | NOS3 | nitric oxide synthase 3 [Source:HGNC Symbol;Acc:HGNC:7876] | 18404 | -0.007 | -0.2758 | No |
| 55 | DRD5 | dopamine receptor D5 [Source:HGNC Symbol;Acc:HGNC:3026] | 18566 | -0.008 | -0.2795 | No |
| 56 | CAMK2B | calcium/calmodulin dependent protein kinase II beta [Source:HGNC Symbol;Acc:HGNC:1461] | 18698 | -0.009 | -0.2824 | No |
| 57 | PDE1A | phosphodiesterase 1A [Source:HGNC Symbol;Acc:HGNC:8774] | 18799 | -0.010 | -0.2845 | No |
| 58 | PHKA2 | phosphorylase kinase regulatory subunit alpha 2 [Source:HGNC Symbol;Acc:HGNC:8926] | 19085 | -0.011 | -0.2912 | No |
| 59 | GNA14 | G protein subunit alpha 14 [Source:HGNC Symbol;Acc:HGNC:4382] | 19185 | -0.012 | -0.2932 | No |
| 60 | VDAC3 | voltage dependent anion channel 3 [Source:HGNC Symbol;Acc:HGNC:12674] | 19385 | -0.013 | -0.2977 | No |
| 61 | HRH1 | histamine receptor H1 [Source:HGNC Symbol;Acc:HGNC:5182] | 20266 | -0.018 | -0.3193 | No |
| 62 | ADCY9 | adenylate cyclase 9 [Source:HGNC Symbol;Acc:HGNC:240] | 22247 | -0.030 | -0.3684 | No |
| 63 | GRM1 | glutamate metabotropic receptor 1 [Source:HGNC Symbol;Acc:HGNC:4593] | 22725 | -0.033 | -0.3790 | No |
| 64 | ATP2B3 | ATPase plasma membrane Ca2+ transporting 3 [Source:HGNC Symbol;Acc:HGNC:816] | 23963 | -0.042 | -0.4086 | No |
| 65 | P2RX2 | purinergic receptor P2X 2 [Source:HGNC Symbol;Acc:HGNC:15459] | 24014 | -0.042 | -0.4078 | No |
| 66 | PTGER3 | prostaglandin E receptor 3 [Source:HGNC Symbol;Acc:HGNC:9595] | 24213 | -0.043 | -0.4108 | No |
| 67 | ADCY1 | adenylate cyclase 1 [Source:HGNC Symbol;Acc:HGNC:232] | 24582 | -0.046 | -0.4180 | No |
| 68 | CACNA1B | calcium voltage-gated channel subunit alpha1 B [Source:HGNC Symbol;Acc:HGNC:1389] | 24691 | -0.046 | -0.4185 | No |
| 69 | GNAL | G protein subunit alpha L [Source:HGNC Symbol;Acc:HGNC:4388] | 24803 | -0.047 | -0.4191 | No |
| 70 | TRHR | thyrotropin releasing hormone receptor [Source:HGNC Symbol;Acc:HGNC:12299] | 25204 | -0.050 | -0.4269 | No |
| 71 | PHKA1 | phosphorylase kinase regulatory subunit alpha 1 [Source:HGNC Symbol;Acc:HGNC:8925] | 25333 | -0.051 | -0.4277 | No |
| 72 | SPHK1 | sphingosine kinase 1 [Source:HGNC Symbol;Acc:HGNC:11240] | 25700 | -0.054 | -0.4345 | No |
| 73 | CALM1 | calmodulin 1 [Source:HGNC Symbol;Acc:HGNC:1442] | 25939 | -0.055 | -0.4379 | No |
| 74 | HTR2C | 5-hydroxytryptamine receptor 2C [Source:HGNC Symbol;Acc:HGNC:5295] | 26208 | -0.057 | -0.4420 | No |
| 75 | GRIN2D | glutamate ionotropic receptor NMDA type subunit 2D [Source:HGNC Symbol;Acc:HGNC:4588] | 26595 | -0.060 | -0.4490 | No |
| 76 | ATP2B2 | ATPase plasma membrane Ca2+ transporting 2 [Source:HGNC Symbol;Acc:HGNC:815] | 26602 | -0.060 | -0.4463 | No |
| 77 | MYLK2 | myosin light chain kinase 2 [Source:HGNC Symbol;Acc:HGNC:16243] | 26644 | -0.061 | -0.4444 | No |
| 78 | TRPC1 | transient receptor potential cation channel subfamily C member 1 [Source:HGNC Symbol;Acc:HGNC:12333] | 27008 | -0.063 | -0.4506 | No |
| 79 | ADRA1A | adrenoceptor alpha 1A [Source:HGNC Symbol;Acc:HGNC:277] | 27013 | -0.063 | -0.4477 | No |
| 80 | CHRM2 | cholinergic receptor muscarinic 2 [Source:HGNC Symbol;Acc:HGNC:1951] | 27332 | -0.066 | -0.4526 | No |
| 81 | PLCE1 | phospholipase C epsilon 1 [Source:HGNC Symbol;Acc:HGNC:17175] | 27770 | -0.069 | -0.4605 | No |
| 82 | PLCB4 | phospholipase C beta 4 [Source:HGNC Symbol;Acc:HGNC:9059] | 27781 | -0.069 | -0.4574 | No |
| 83 | MYLK3 | myosin light chain kinase 3 [Source:HGNC Symbol;Acc:HGNC:29826] | 27794 | -0.069 | -0.4544 | No |
| 84 | ADCY8 | adenylate cyclase 8 [Source:HGNC Symbol;Acc:HGNC:239] | 27849 | -0.070 | -0.4524 | No |
| 85 | LTB4R2 | leukotriene B4 receptor 2 [Source:HGNC Symbol;Acc:HGNC:19260] | 28020 | -0.071 | -0.4533 | No |
| 86 | TACR3 | tachykinin receptor 3 [Source:HGNC Symbol;Acc:HGNC:11528] | 28021 | -0.071 | -0.4499 | No |
| 87 | BDKRB2 | bradykinin receptor B2 [Source:HGNC Symbol;Acc:HGNC:1030] | 28223 | -0.073 | -0.4515 | No |
| 88 | CHRM3 | cholinergic receptor muscarinic 3 [Source:HGNC Symbol;Acc:HGNC:1952] | 28286 | -0.074 | -0.4495 | No |
| 89 | ERBB4 | erb-b2 receptor tyrosine kinase 4 [Source:HGNC Symbol;Acc:HGNC:3432] | 28532 | -0.076 | -0.4522 | No |
| 90 | CALML3 | calmodulin like 3 [Source:HGNC Symbol;Acc:HGNC:1452] | 29009 | -0.080 | -0.4605 | No |
| 91 | PLCZ1 | phospholipase C zeta 1 [Source:HGNC Symbol;Acc:HGNC:19218] | 29059 | -0.080 | -0.4579 | No |
| 92 | ITPKB | inositol-trisphosphate 3-kinase B [Source:HGNC Symbol;Acc:HGNC:6179] | 29125 | -0.081 | -0.4556 | No |
| 93 | HTR2B | 5-hydroxytryptamine receptor 2B [Source:HGNC Symbol;Acc:HGNC:5294] | 29528 | -0.085 | -0.4618 | Yes |
| 94 | CCKAR | cholecystokinin A receptor [Source:HGNC Symbol;Acc:HGNC:1570] | 29625 | -0.086 | -0.4602 | Yes |
| 95 | VDAC1 | voltage dependent anion channel 1 [Source:HGNC Symbol;Acc:HGNC:12669] | 29848 | -0.088 | -0.4616 | Yes |
| 96 | EDNRB | endothelin receptor type B [Source:HGNC Symbol;Acc:HGNC:3180] | 29975 | -0.089 | -0.4605 | Yes |
| 97 | CACNA1S | calcium voltage-gated channel subunit alpha1 S [Source:HGNC Symbol;Acc:HGNC:1397] | 30104 | -0.091 | -0.4594 | Yes |
| 98 | EDNRA | endothelin receptor type A [Source:HGNC Symbol;Acc:HGNC:3179] | 30139 | -0.091 | -0.4559 | Yes |
| 99 | CACNA1H | calcium voltage-gated channel subunit alpha1 H [Source:HGNC Symbol;Acc:HGNC:1395] | 30151 | -0.091 | -0.4518 | Yes |
| 100 | CACNA1I | calcium voltage-gated channel subunit alpha1 I [Source:HGNC Symbol;Acc:HGNC:1396] | 30593 | -0.096 | -0.4585 | Yes |
| 101 | PLCD4 | phospholipase C delta 4 [Source:HGNC Symbol;Acc:HGNC:9062] | 30666 | -0.097 | -0.4557 | Yes |
| 102 | OXTR | oxytocin receptor [Source:HGNC Symbol;Acc:HGNC:8529] | 30681 | -0.097 | -0.4514 | Yes |
| 103 | HTR4 | 5-hydroxytryptamine receptor 4 [Source:HGNC Symbol;Acc:HGNC:5299] | 30735 | -0.098 | -0.4480 | Yes |
| 104 | ADCY4 | adenylate cyclase 4 [Source:HGNC Symbol;Acc:HGNC:235] | 30750 | -0.098 | -0.4437 | Yes |
| 105 | PPID | peptidylprolyl isomerase D [Source:HGNC Symbol;Acc:HGNC:9257] | 30753 | -0.098 | -0.4390 | Yes |
| 106 | PLCG1 | phospholipase C gamma 1 [Source:HGNC Symbol;Acc:HGNC:9065] | 30759 | -0.098 | -0.4345 | Yes |
| 107 | CACNA1G | calcium voltage-gated channel subunit alpha1 G [Source:HGNC Symbol;Acc:HGNC:1394] | 30789 | -0.098 | -0.4305 | Yes |
| 108 | HTR6 | 5-hydroxytryptamine receptor 6 [Source:HGNC Symbol;Acc:HGNC:5301] | 31282 | -0.104 | -0.4381 | Yes |
| 109 | PRKCG | protein kinase C gamma [Source:HGNC Symbol;Acc:HGNC:9402] | 31522 | -0.107 | -0.4391 | Yes |
| 110 | CD38 | CD38 molecule [Source:HGNC Symbol;Acc:HGNC:1667] | 31649 | -0.108 | -0.4371 | Yes |
| 111 | ADRB2 | adrenoceptor beta 2 [Source:HGNC Symbol;Acc:HGNC:286] | 31665 | -0.109 | -0.4322 | Yes |
| 112 | PDE1B | phosphodiesterase 1B [Source:HGNC Symbol;Acc:HGNC:8775] | 31673 | -0.109 | -0.4272 | Yes |
| 113 | PLCD3 | phospholipase C delta 3 [Source:HGNC Symbol;Acc:HGNC:9061] | 31690 | -0.109 | -0.4224 | Yes |
| 114 | CACNA1A | calcium voltage-gated channel subunit alpha1 A [Source:HGNC Symbol;Acc:HGNC:1388] | 31706 | -0.109 | -0.4175 | Yes |
| 115 | HTR5A | 5-hydroxytryptamine receptor 5A [Source:HGNC Symbol;Acc:HGNC:5300] | 31727 | -0.109 | -0.4127 | Yes |
| 116 | ITPR3 | "inositol 1,4,5-trisphosphate receptor type 3 [Source:HGNC Symbol;Acc:HGNC:6182]" | 31781 | -0.110 | -0.4088 | Yes |
| 117 | NTSR1 | neurotensin receptor 1 [Source:HGNC Symbol;Acc:HGNC:8039] | 31999 | -0.113 | -0.4089 | Yes |
| 118 | CAMK2A | calcium/calmodulin dependent protein kinase II alpha [Source:HGNC Symbol;Acc:HGNC:1460] | 32027 | -0.113 | -0.4041 | Yes |
| 119 | GRM5 | glutamate metabotropic receptor 5 [Source:HGNC Symbol;Acc:HGNC:4597] | 32047 | -0.114 | -0.3992 | Yes |
| 120 | PRKCA | protein kinase C alpha [Source:HGNC Symbol;Acc:HGNC:9393] | 32080 | -0.114 | -0.3945 | Yes |
| 121 | AVPR1B | arginine vasopressin receptor 1B [Source:HGNC Symbol;Acc:HGNC:896] | 32414 | -0.119 | -0.3973 | Yes |
| 122 | PPP3R2 | "protein phosphatase 3 regulatory subunit B, beta [Source:HGNC Symbol;Acc:HGNC:9318]" | 32695 | -0.123 | -0.3985 | Yes |
| 123 | CYSLTR1 | cysteinyl leukotriene receptor 1 [Source:HGNC Symbol;Acc:HGNC:17451] | 32748 | -0.124 | -0.3939 | Yes |
| 124 | DRD1 | dopamine receptor D1 [Source:HGNC Symbol;Acc:HGNC:3020] | 32942 | -0.127 | -0.3928 | Yes |
| 125 | PRKACG | protein kinase cAMP-activated catalytic subunit gamma [Source:HGNC Symbol;Acc:HGNC:9382] | 33040 | -0.129 | -0.3890 | Yes |
| 126 | PDE1C | phosphodiesterase 1C [Source:HGNC Symbol;Acc:HGNC:8776] | 33206 | -0.131 | -0.3870 | Yes |
| 127 | PPP3CB | protein phosphatase 3 catalytic subunit beta [Source:HGNC Symbol;Acc:HGNC:9315] | 33217 | -0.131 | -0.3809 | Yes |
| 128 | ATP2B4 | ATPase plasma membrane Ca2+ transporting 4 [Source:HGNC Symbol;Acc:HGNC:817] | 33242 | -0.132 | -0.3752 | Yes |
| 129 | PTGFR | prostaglandin F receptor [Source:HGNC Symbol;Acc:HGNC:9600] | 33350 | -0.134 | -0.3715 | Yes |
| 130 | NOS1 | nitric oxide synthase 1 [Source:HGNC Symbol;Acc:HGNC:7872] | 33519 | -0.137 | -0.3692 | Yes |
| 131 | CACNA1D | calcium voltage-gated channel subunit alpha1 D [Source:HGNC Symbol;Acc:HGNC:1391] | 33627 | -0.138 | -0.3653 | Yes |
| 132 | GNAQ | G protein subunit alpha q [Source:HGNC Symbol;Acc:HGNC:4390] | 33694 | -0.139 | -0.3603 | Yes |
| 133 | RYR3 | ryanodine receptor 3 [Source:HGNC Symbol;Acc:HGNC:10485] | 33737 | -0.140 | -0.3546 | Yes |
| 134 | BST1 | bone marrow stromal cell antigen 1 [Source:HGNC Symbol;Acc:HGNC:1118] | 33761 | -0.141 | -0.3484 | Yes |
| 135 | PLCB2 | phospholipase C beta 2 [Source:HGNC Symbol;Acc:HGNC:9055] | 33768 | -0.141 | -0.3418 | Yes |
| 136 | ITPR1 | "inositol 1,4,5-trisphosphate receptor type 1 [Source:HGNC Symbol;Acc:HGNC:6180]" | 33914 | -0.144 | -0.3386 | Yes |
| 137 | NOS2 | nitric oxide synthase 2 [Source:HGNC Symbol;Acc:HGNC:7873] | 33997 | -0.145 | -0.3337 | Yes |
| 138 | BDKRB1 | bradykinin receptor B1 [Source:HGNC Symbol;Acc:HGNC:1029] | 34460 | -0.154 | -0.3381 | Yes |
| 139 | TACR1 | tachykinin receptor 1 [Source:HGNC Symbol;Acc:HGNC:11526] | 34737 | -0.161 | -0.3374 | Yes |
| 140 | CALM3 | calmodulin 3 [Source:HGNC Symbol;Acc:HGNC:1449] | 34875 | -0.164 | -0.3331 | Yes |
| 141 | SLC8A3 | solute carrier family 8 member A3 [Source:HGNC Symbol;Acc:HGNC:11070] | 35067 | -0.168 | -0.3299 | Yes |
| 142 | CACNA1E | calcium voltage-gated channel subunit alpha1 E [Source:HGNC Symbol;Acc:HGNC:1392] | 35281 | -0.173 | -0.3270 | Yes |
| 143 | GRPR | gastrin releasing peptide receptor [Source:HGNC Symbol;Acc:HGNC:4609] | 35597 | -0.182 | -0.3263 | Yes |
| 144 | F2R | coagulation factor II thrombin receptor [Source:HGNC Symbol;Acc:HGNC:3537] | 35689 | -0.184 | -0.3198 | Yes |
| 145 | PPP3CC | protein phosphatase 3 catalytic subunit gamma [Source:HGNC Symbol;Acc:HGNC:9316] | 35754 | -0.186 | -0.3124 | Yes |
| 146 | CHRNA7 | cholinergic receptor nicotinic alpha 7 subunit [Source:HGNC Symbol;Acc:HGNC:1960] | 35812 | -0.188 | -0.3048 | Yes |
| 147 | CHRM5 | cholinergic receptor muscarinic 5 [Source:HGNC Symbol;Acc:HGNC:1954] | 35860 | -0.190 | -0.2969 | Yes |
| 148 | ADORA2A | adenosine A2a receptor [Source:HGNC Symbol;Acc:HGNC:263] | 35916 | -0.191 | -0.2892 | Yes |
| 149 | ADCY7 | adenylate cyclase 7 [Source:HGNC Symbol;Acc:HGNC:238] | 36103 | -0.197 | -0.2844 | Yes |
| 150 | HTR7 | 5-hydroxytryptamine receptor 7 [Source:HGNC Symbol;Acc:HGNC:5302] | 36186 | -0.200 | -0.2769 | Yes |
| 151 | PLN | phospholamban [Source:HGNC Symbol;Acc:HGNC:9080] | 36211 | -0.201 | -0.2679 | Yes |
| 152 | ADCY3 | adenylate cyclase 3 [Source:HGNC Symbol;Acc:HGNC:234] | 36213 | -0.201 | -0.2583 | Yes |
| 153 | PLCB1 | phospholipase C beta 1 [Source:HGNC Symbol;Acc:HGNC:15917] | 36397 | -0.208 | -0.2530 | Yes |
| 154 | PDGFRB | platelet derived growth factor receptor beta [Source:HGNC Symbol;Acc:HGNC:8804] | 36398 | -0.208 | -0.2430 | Yes |
| 155 | P2RX7 | purinergic receptor P2X 7 [Source:HGNC Symbol;Acc:HGNC:8537] | 36472 | -0.210 | -0.2347 | Yes |
| 156 | AGTR1 | angiotensin II receptor type 1 [Source:HGNC Symbol;Acc:HGNC:336] | 36675 | -0.219 | -0.2294 | Yes |
| 157 | P2RX5 | purinergic receptor P2X 5 [Source:HGNC Symbol;Acc:HGNC:8536] | 36776 | -0.224 | -0.2212 | Yes |
| 158 | AVPR1A | arginine vasopressin receptor 1A [Source:HGNC Symbol;Acc:HGNC:895] | 36808 | -0.225 | -0.2111 | Yes |
| 159 | PRKACB | protein kinase cAMP-activated catalytic subunit beta [Source:HGNC Symbol;Acc:HGNC:9381] | 36870 | -0.229 | -0.2017 | Yes |
| 160 | HTR2A | 5-hydroxytryptamine receptor 2A [Source:HGNC Symbol;Acc:HGNC:5293] | 36934 | -0.232 | -0.1922 | Yes |
| 161 | PTK2B | protein tyrosine kinase 2 beta [Source:HGNC Symbol;Acc:HGNC:9612] | 37013 | -0.237 | -0.1828 | Yes |
| 162 | ITPR2 | "inositol 1,4,5-trisphosphate receptor type 2 [Source:HGNC Symbol;Acc:HGNC:6181]" | 37066 | -0.239 | -0.1726 | Yes |
| 163 | P2RX1 | purinergic receptor P2X 1 [Source:HGNC Symbol;Acc:HGNC:8533] | 37178 | -0.247 | -0.1636 | Yes |
| 164 | MYLK | myosin light chain kinase [Source:HGNC Symbol;Acc:HGNC:7590] | 37197 | -0.248 | -0.1521 | Yes |
| 165 | CAMK4 | calcium/calmodulin dependent protein kinase IV [Source:HGNC Symbol;Acc:HGNC:1464] | 37227 | -0.249 | -0.1409 | Yes |
| 166 | ATP2A2 | ATPase sarcoplasmic/endoplasmic reticulum Ca2+ transporting 2 [Source:HGNC Symbol;Acc:HGNC:812] | 37541 | -0.269 | -0.1360 | Yes |
| 167 | PRKX | protein kinase X-linked [Source:HGNC Symbol;Acc:HGNC:9441] | 37543 | -0.269 | -0.1230 | Yes |
| 168 | PTAFR | platelet activating factor receptor [Source:HGNC Symbol;Acc:HGNC:9582] | 37638 | -0.277 | -0.1121 | Yes |
| 169 | PDGFRA | platelet derived growth factor receptor alpha [Source:HGNC Symbol;Acc:HGNC:8803] | 37662 | -0.280 | -0.0993 | Yes |
| 170 | CACNA1C | calcium voltage-gated channel subunit alpha1 C [Source:HGNC Symbol;Acc:HGNC:1390] | 37818 | -0.293 | -0.0892 | Yes |
| 171 | PRKCB | protein kinase C beta [Source:HGNC Symbol;Acc:HGNC:9395] | 37822 | -0.293 | -0.0752 | Yes |
| 172 | ATP2A3 | ATPase sarcoplasmic/endoplasmic reticulum Ca2+ transporting 3 [Source:HGNC Symbol;Acc:HGNC:813] | 37862 | -0.298 | -0.0618 | Yes |
| 173 | RYR2 | ryanodine receptor 2 [Source:HGNC Symbol;Acc:HGNC:10484] | 37888 | -0.301 | -0.0480 | Yes |
| 174 | HRH2 | histamine receptor H2 [Source:HGNC Symbol;Acc:HGNC:5183] | 38019 | -0.320 | -0.0360 | Yes |
| 175 | PLCG2 | phospholipase C gamma 2 [Source:HGNC Symbol;Acc:HGNC:9066] | 38119 | -0.338 | -0.0223 | Yes |
| 176 | ATP2B1 | ATPase plasma membrane Ca2+ transporting 1 [Source:HGNC Symbol;Acc:HGNC:814] | 38151 | -0.347 | -0.0064 | Yes |
| 177 | CYSLTR2 | cysteinyl leukotriene receptor 2 [Source:HGNC Symbol;Acc:HGNC:18274] | 38233 | -0.376 | 0.0096 | Yes |
| 178 | SLC8A1 | solute carrier family 8 member A1 [Source:HGNC Symbol;Acc:HGNC:11068] | 38241 | -0.379 | 0.0276 | Yes |
Table: GSEA details [plain text format]

  

Fig 2: KEGG\_CALCIUM\_SIGNALING\_PATHWAY      
 Blue-Pink O' Gram in the Space of the Analyzed GeneSet

  

Fig 3: KEGG\_CALCIUM\_SIGNALING\_PATHWAY: Random ES distribution      
 Gene set null distribution of ES for **KEGG\_CALCIUM\_SIGNALING\_PATHWAY**

  
