# Supplementary material for: Simultaneous Presentation of Multiple Myeloma and Lung Cancer: Case Report and Gene Bioinformatics Analysis
Source: Front Oncol. 2022 Jun 13;12:859735. doi: 10.3389/fonc.2022.859735 (PMC9235397; doi:10.3389/fonc.2022.859735)
Supplement: Supplementary file 1 [file DataSheet_1.zip › The bioinformatic analysis of MM and lung cancer supplementary materials/Enrichment analysis/MECR/GSEA_4.1.0/LUAD TCGA/KEGG.Gsea.1639041756227/KEGG_CELL_ADHESION_MOLECULES_CAMS.html]

Details for gene set KEGG\_CELL\_ADHESION\_MOLECULES\_CAMS[GSEA]

|  || Dataset | ExpData\_collapsed\_to\_symbols.ENSG00000116353\_profile\_in\_ExpData.cls #ENSG00000116353 |
| Phenotype | ENSG00000116353\_profile\_in\_ExpData.cls#ENSG00000116353 |
| Upregulated in class | ENSG00000116353\_neg |
| GeneSet | KEGG\_CELL\_ADHESION\_MOLECULES\_CAMS |
| Enrichment Score (ES) | -0.5267841 |
| Normalized Enrichment Score (NES) | -2.303676 |
| Nominal p-value | 0.0 |
| FDR q-value | 0.0 |
| FWER p-Value | 0.0 |
Table: GSEA Results Summary

  

Fig 1: Enrichment plot: KEGG\_CELL\_ADHESION\_MOLECULES\_CAMS      
 Profile of the Running ES Score & Positions of GeneSet Members on the Rank Ordered List

  

| SYMBOL | TITLE | RANK IN GENE LIST | RANK METRIC SCORE | RUNNING ES | CORE ENRICHMENT || 1 | CLDN7 | claudin 7 [Source:HGNC Symbol;Acc:HGNC:2049] | 1249 | 0.251 | -0.0174 | No |
| 2 | CLDN4 | claudin 4 [Source:HGNC Symbol;Acc:HGNC:2046] | 1316 | 0.246 | -0.0050 | No |
| 3 | CDH15 | cadherin 15 [Source:HGNC Symbol;Acc:HGNC:1754] | 1334 | 0.245 | 0.0087 | No |
| 4 | SDC4 | syndecan 4 [Source:HGNC Symbol;Acc:HGNC:10661] | 2069 | 0.203 | 0.0016 | No |
| 5 | CLDN3 | claudin 3 [Source:HGNC Symbol;Acc:HGNC:2045] | 2175 | 0.198 | 0.0103 | No |
| 6 | SDC1 | syndecan 1 [Source:HGNC Symbol;Acc:HGNC:10658] | 2445 | 0.186 | 0.0141 | No |
| 7 | CLDN6 | claudin 6 [Source:HGNC Symbol;Acc:HGNC:2048] | 3000 | 0.164 | 0.0094 | No |
| 8 | CLDN9 | claudin 9 [Source:HGNC Symbol;Acc:HGNC:2051] | 3014 | 0.163 | 0.0185 | No |
| 9 | F11R | F11 receptor [Source:HGNC Symbol;Acc:HGNC:14685] | 3831 | 0.139 | 0.0056 | No |
| 10 | HLA-DMA | "major histocompatibility complex, class II, DM alpha [Source:HGNC Symbol;Acc:HGNC:4934]" | 4341 | 0.126 | -0.0002 | No |
| 11 | HLA-A | "major histocompatibility complex, class I, A [Source:HGNC Symbol;Acc:HGNC:4931]" | 4980 | 0.111 | -0.0100 | No |
| 12 | CLDN8 | claudin 8 [Source:HGNC Symbol;Acc:HGNC:2050] | 5034 | 0.111 | -0.0050 | No |
| 13 | CLDN15 | claudin 15 [Source:HGNC Symbol;Acc:HGNC:2036] | 5297 | 0.106 | -0.0056 | No |
| 14 | ALCAM | activated leukocyte cell adhesion molecule [Source:HGNC Symbol;Acc:HGNC:400] | 5635 | 0.100 | -0.0085 | No |
| 15 | CD58 | CD58 molecule [Source:HGNC Symbol;Acc:HGNC:1688] | 5872 | 0.096 | -0.0090 | No |
| 16 | PTPRF | protein tyrosine phosphatase receptor type F [Source:HGNC Symbol;Acc:HGNC:9670] | 6015 | 0.093 | -0.0073 | No |
| 17 | CLDN23 | claudin 23 [Source:HGNC Symbol;Acc:HGNC:17591] | 6209 | 0.090 | -0.0070 | No |
| 18 | MADCAM1 | mucosal vascular addressin cell adhesion molecule 1 [Source:HGNC Symbol;Acc:HGNC:6765] | 6690 | 0.084 | -0.0145 | No |
| 19 | SDC3 | syndecan 3 [Source:HGNC Symbol;Acc:HGNC:10660] | 6930 | 0.080 | -0.0160 | No |
| 20 | HLA-F | "major histocompatibility complex, class I, F [Source:HGNC Symbol;Acc:HGNC:4963]" | 7370 | 0.075 | -0.0229 | No |
| 21 | ICAM1 | intercellular adhesion molecule 1 [Source:HGNC Symbol;Acc:HGNC:5344] | 8090 | 0.067 | -0.0374 | No |
| 22 | CDH3 | cadherin 3 [Source:HGNC Symbol;Acc:HGNC:1762] | 8184 | 0.066 | -0.0360 | No |
| 23 | MPZ | myelin protein zero [Source:HGNC Symbol;Acc:HGNC:7225] | 8465 | 0.063 | -0.0396 | No |
| 24 | MPZL1 | myelin protein zero like 1 [Source:HGNC Symbol;Acc:HGNC:7226] | 9073 | 0.057 | -0.0518 | No |
| 25 | HLA-C | "major histocompatibility complex, class I, C [Source:HGNC Symbol;Acc:HGNC:4933]" | 9612 | 0.052 | -0.0625 | No |
| 26 | CLDN16 | claudin 16 [Source:HGNC Symbol;Acc:HGNC:2037] | 9621 | 0.052 | -0.0598 | No |
| 27 | HLA-DRB1 | "major histocompatibility complex, class II, DR beta 1 [Source:HGNC Symbol;Acc:HGNC:4948]" | 10444 | 0.045 | -0.0781 | No |
| 28 | HLA-DRB5 | "major histocompatibility complex, class II, DR beta 5 [Source:HGNC Symbol;Acc:HGNC:4953]" | 10518 | 0.045 | -0.0774 | No |
| 29 | CD276 | CD276 molecule [Source:HGNC Symbol;Acc:HGNC:19137] | 10827 | 0.042 | -0.0829 | No |
| 30 | CNTN2 | contactin 2 [Source:HGNC Symbol;Acc:HGNC:2172] | 11907 | 0.034 | -0.1084 | No |
| 31 | CLDN17 | claudin 17 [Source:HGNC Symbol;Acc:HGNC:2038] | 12140 | 0.032 | -0.1125 | No |
| 32 | HLA-G | "major histocompatibility complex, class I, G [Source:HGNC Symbol;Acc:HGNC:4964]" | 12279 | 0.031 | -0.1143 | No |
| 33 | HLA-B | "major histocompatibility complex, class I, B [Source:HGNC Symbol;Acc:HGNC:4932]" | 13305 | 0.024 | -0.1390 | No |
| 34 | MAG | myelin associated glycoprotein [Source:HGNC Symbol;Acc:HGNC:6783] | 14072 | 0.019 | -0.1575 | No |
| 35 | CLDN5 | claudin 5 [Source:HGNC Symbol;Acc:HGNC:2047] | 15366 | 0.011 | -0.1899 | No |
| 36 | NRXN2 | neurexin 2 [Source:HGNC Symbol;Acc:HGNC:8009] | 15394 | 0.011 | -0.1900 | No |
| 37 | HLA-DPB1 | "major histocompatibility complex, class II, DP beta 1 [Source:HGNC Symbol;Acc:HGNC:4940]" | 16356 | 0.005 | -0.2142 | No |
| 38 | NECTIN3 | nectin cell adhesion molecule 3 [Source:HGNC Symbol;Acc:HGNC:17664] | 16706 | 0.003 | -0.2229 | No |
| 39 | CD40 | CD40 molecule [Source:HGNC Symbol;Acc:HGNC:11919] | 17012 | 0.001 | -0.2306 | No |
| 40 | HLA-DRA | "major histocompatibility complex, class II, DR alpha [Source:HGNC Symbol;Acc:HGNC:4947]" | 17663 | -0.003 | -0.2471 | No |
| 41 | HLA-DQB1 | "major histocompatibility complex, class II, DQ beta 1 [Source:HGNC Symbol;Acc:HGNC:4944]" | 17913 | -0.004 | -0.2532 | No |
| 42 | OCLN | occludin [Source:HGNC Symbol;Acc:HGNC:8104] | 18739 | -0.009 | -0.2737 | No |
| 43 | CNTNAP2 | contactin associated protein 2 [Source:HGNC Symbol;Acc:HGNC:13830] | 18816 | -0.010 | -0.2751 | No |
| 44 | HLA-DQA2 | "major histocompatibility complex, class II, DQ alpha 2 [Source:HGNC Symbol;Acc:HGNC:4943]" | 19230 | -0.012 | -0.2849 | No |
| 45 | ICOSLG | inducible T cell costimulator ligand [Source:HGNC Symbol;Acc:HGNC:17087] | 19606 | -0.014 | -0.2936 | No |
| 46 | NECTIN2 | nectin cell adhesion molecule 2 [Source:HGNC Symbol;Acc:HGNC:9707] | 20072 | -0.017 | -0.3045 | No |
| 47 | CDH4 | cadherin 4 [Source:HGNC Symbol;Acc:HGNC:1763] | 20349 | -0.019 | -0.3105 | No |
| 48 | HLA-DOB | "major histocompatibility complex, class II, DO beta [Source:HGNC Symbol;Acc:HGNC:4937]" | 20731 | -0.021 | -0.3190 | No |
| 49 | NECTIN1 | nectin cell adhesion molecule 1 [Source:HGNC Symbol;Acc:HGNC:9706] | 20870 | -0.022 | -0.3213 | No |
| 50 | CADM1 | cell adhesion molecule 1 [Source:HGNC Symbol;Acc:HGNC:5951] | 20934 | -0.022 | -0.3216 | No |
| 51 | CD99 | CD99 molecule (Xg blood group) [Source:HGNC Symbol;Acc:HGNC:7082] | 21637 | -0.027 | -0.3380 | No |
| 52 | HLA-E | "major histocompatibility complex, class I, E [Source:HGNC Symbol;Acc:HGNC:4962]" | 21659 | -0.027 | -0.3370 | No |
| 53 | NLGN2 | neuroligin 2 [Source:HGNC Symbol;Acc:HGNC:14290] | 21733 | -0.027 | -0.3372 | No |
| 54 | CLDN1 | claudin 1 [Source:HGNC Symbol;Acc:HGNC:2032] | 22989 | -0.035 | -0.3672 | No |
| 55 | CDH1 | cadherin 1 [Source:HGNC Symbol;Acc:HGNC:1748] | 23456 | -0.038 | -0.3769 | No |
| 56 | NEGR1 | neuronal growth regulator 1 [Source:HGNC Symbol;Acc:HGNC:17302] | 23572 | -0.039 | -0.3776 | No |
| 57 | NLGN1 | neuroligin 1 [Source:HGNC Symbol;Acc:HGNC:14291] | 24026 | -0.042 | -0.3868 | No |
| 58 | HLA-DPA1 | "major histocompatibility complex, class II, DP alpha 1 [Source:HGNC Symbol;Acc:HGNC:4938]" | 24356 | -0.044 | -0.3926 | No |
| 59 | HLA-DMB | "major histocompatibility complex, class II, DM beta [Source:HGNC Symbol;Acc:HGNC:4935]" | 24935 | -0.048 | -0.4046 | No |
| 60 | ITGA9 | integrin subunit alpha 9 [Source:HGNC Symbol;Acc:HGNC:6145] | 25285 | -0.051 | -0.4106 | No |
| 61 | ICAM2 | intercellular adhesion molecule 2 [Source:HGNC Symbol;Acc:HGNC:5345] | 25505 | -0.052 | -0.4132 | No |
| 62 | L1CAM | L1 cell adhesion molecule [Source:HGNC Symbol;Acc:HGNC:6470] | 25953 | -0.056 | -0.4214 | No |
| 63 | ESAM | endothelial cell adhesion molecule [Source:HGNC Symbol;Acc:HGNC:17474] | 26396 | -0.059 | -0.4293 | No |
| 64 | CNTN1 | contactin 1 [Source:HGNC Symbol;Acc:HGNC:2171] | 26402 | -0.059 | -0.4261 | No |
| 65 | NRXN1 | neurexin 1 [Source:HGNC Symbol;Acc:HGNC:8008] | 27760 | -0.069 | -0.4567 | No |
| 66 | CLDN14 | claudin 14 [Source:HGNC Symbol;Acc:HGNC:2035] | 28510 | -0.076 | -0.4715 | No |
| 67 | CLDN2 | claudin 2 [Source:HGNC Symbol;Acc:HGNC:2041] | 28925 | -0.079 | -0.4775 | No |
| 68 | SDC2 | syndecan 2 [Source:HGNC Symbol;Acc:HGNC:10659] | 30196 | -0.092 | -0.5047 | No |
| 69 | NLGN3 | neuroligin 3 [Source:HGNC Symbol;Acc:HGNC:14289] | 30677 | -0.097 | -0.5114 | No |
| 70 | NCAM1 | neural cell adhesion molecule 1 [Source:HGNC Symbol;Acc:HGNC:7656] | 30904 | -0.099 | -0.5114 | No |
| 71 | ITGB8 | integrin subunit beta 8 [Source:HGNC Symbol;Acc:HGNC:6163] | 31035 | -0.101 | -0.5089 | No |
| 72 | CADM3 | cell adhesion molecule 3 [Source:HGNC Symbol;Acc:HGNC:17601] | 31355 | -0.105 | -0.5111 | No |
| 73 | CD34 | CD34 molecule [Source:HGNC Symbol;Acc:HGNC:1662] | 31546 | -0.107 | -0.5098 | No |
| 74 | SELE | selectin E [Source:HGNC Symbol;Acc:HGNC:10718] | 32201 | -0.116 | -0.5198 | Yes |
| 75 | PTPRM | protein tyrosine phosphatase receptor type M [Source:HGNC Symbol;Acc:HGNC:9675] | 32375 | -0.118 | -0.5174 | Yes |
| 76 | ITGA8 | integrin subunit alpha 8 [Source:HGNC Symbol;Acc:HGNC:6144] | 32742 | -0.124 | -0.5197 | Yes |
| 77 | HLA-DQA1 | "major histocompatibility complex, class II, DQ alpha 1 [Source:HGNC Symbol;Acc:HGNC:4942]" | 32898 | -0.126 | -0.5164 | Yes |
| 78 | NCAM2 | neural cell adhesion molecule 2 [Source:HGNC Symbol;Acc:HGNC:7657] | 32916 | -0.127 | -0.5095 | Yes |
| 79 | HLA-DOA | "major histocompatibility complex, class II, DO alpha [Source:HGNC Symbol;Acc:HGNC:4936]" | 33498 | -0.136 | -0.5166 | Yes |
| 80 | ITGB2 | integrin subunit beta 2 [Source:HGNC Symbol;Acc:HGNC:6155] | 33691 | -0.139 | -0.5134 | Yes |
| 81 | CD22 | CD22 molecule [Source:HGNC Symbol;Acc:HGNC:1643] | 33891 | -0.143 | -0.5103 | Yes |
| 82 | CLDN19 | claudin 19 [Source:HGNC Symbol;Acc:HGNC:2040] | 33900 | -0.143 | -0.5023 | Yes |
| 83 | CLDN11 | claudin 11 [Source:HGNC Symbol;Acc:HGNC:8514] | 34044 | -0.146 | -0.4975 | Yes |
| 84 | CLDN18 | claudin 18 [Source:HGNC Symbol;Acc:HGNC:2039] | 34500 | -0.155 | -0.5002 | Yes |
| 85 | JAM2 | junctional adhesion molecule 2 [Source:HGNC Symbol;Acc:HGNC:14686] | 34555 | -0.156 | -0.4926 | Yes |
| 86 | NLGN4X | neuroligin 4 X-linked [Source:HGNC Symbol;Acc:HGNC:14287] | 34687 | -0.159 | -0.4868 | Yes |
| 87 | CLDN10 | claudin 10 [Source:HGNC Symbol;Acc:HGNC:2033] | 34859 | -0.163 | -0.4818 | Yes |
| 88 | PDCD1 | programmed cell death 1 [Source:HGNC Symbol;Acc:HGNC:8760] | 34965 | -0.165 | -0.4750 | Yes |
| 89 | PVR | PVR cell adhesion molecule [Source:HGNC Symbol;Acc:HGNC:9705] | 35125 | -0.169 | -0.4693 | Yes |
| 90 | CD40LG | CD40 ligand [Source:HGNC Symbol;Acc:HGNC:11935] | 35185 | -0.171 | -0.4610 | Yes |
| 91 | NEO1 | neogenin 1 [Source:HGNC Symbol;Acc:HGNC:7754] | 35223 | -0.172 | -0.4521 | Yes |
| 92 | GLG1 | golgi glycoprotein 1 [Source:HGNC Symbol;Acc:HGNC:4316] | 35552 | -0.181 | -0.4501 | Yes |
| 93 | CNTNAP1 | contactin associated protein 1 [Source:HGNC Symbol;Acc:HGNC:8011] | 35649 | -0.183 | -0.4420 | Yes |
| 94 | CD2 | CD2 molecule [Source:HGNC Symbol;Acc:HGNC:1639] | 35724 | -0.185 | -0.4332 | Yes |
| 95 | NFASC | neurofascin [Source:HGNC Symbol;Acc:HGNC:29866] | 35838 | -0.189 | -0.4253 | Yes |
| 96 | CLDN20 | claudin 20 [Source:HGNC Symbol;Acc:HGNC:2042] | 35870 | -0.190 | -0.4152 | Yes |
| 97 | CD274 | CD274 molecule [Source:HGNC Symbol;Acc:HGNC:17635] | 36147 | -0.199 | -0.4108 | Yes |
| 98 | CD8B | CD8b molecule [Source:HGNC Symbol;Acc:HGNC:1707] | 36153 | -0.199 | -0.3995 | Yes |
| 99 | SELPLG | selectin P ligand [Source:HGNC Symbol;Acc:HGNC:10722] | 36197 | -0.200 | -0.3891 | Yes |
| 100 | CDH5 | cadherin 5 [Source:HGNC Symbol;Acc:HGNC:1764] | 36210 | -0.201 | -0.3779 | Yes |
| 101 | SIGLEC1 | sialic acid binding Ig like lectin 1 [Source:HGNC Symbol;Acc:HGNC:11127] | 36233 | -0.202 | -0.3668 | Yes |
| 102 | CD8A | CD8a molecule [Source:HGNC Symbol;Acc:HGNC:1706] | 36547 | -0.213 | -0.3626 | Yes |
| 103 | CD6 | CD6 molecule [Source:HGNC Symbol;Acc:HGNC:1691] | 36601 | -0.215 | -0.3516 | Yes |
| 104 | CDH2 | cadherin 2 [Source:HGNC Symbol;Acc:HGNC:1759] | 36615 | -0.216 | -0.3395 | Yes |
| 105 | ITGAM | integrin subunit alpha M [Source:HGNC Symbol;Acc:HGNC:6149] | 36644 | -0.218 | -0.3278 | Yes |
| 106 | SELL | selectin L [Source:HGNC Symbol;Acc:HGNC:10720] | 36696 | -0.220 | -0.3164 | Yes |
| 107 | NRXN3 | neurexin 3 [Source:HGNC Symbol;Acc:HGNC:8010] | 36786 | -0.224 | -0.3058 | Yes |
| 108 | SELP | selectin P [Source:HGNC Symbol;Acc:HGNC:10721] | 36980 | -0.235 | -0.2972 | Yes |
| 109 | NRCAM | neuronal cell adhesion molecule [Source:HGNC Symbol;Acc:HGNC:7994] | 37016 | -0.237 | -0.2845 | Yes |
| 110 | SPN | sialophorin [Source:HGNC Symbol;Acc:HGNC:11249] | 37047 | -0.238 | -0.2716 | Yes |
| 111 | ITGA6 | integrin subunit alpha 6 [Source:HGNC Symbol;Acc:HGNC:6142] | 37064 | -0.239 | -0.2583 | Yes |
| 112 | ICOS | inducible T cell costimulator [Source:HGNC Symbol;Acc:HGNC:5351] | 37068 | -0.239 | -0.2446 | Yes |
| 113 | JAM3 | junctional adhesion molecule 3 [Source:HGNC Symbol;Acc:HGNC:15532] | 37103 | -0.242 | -0.2316 | Yes |
| 114 | CD4 | CD4 molecule [Source:HGNC Symbol;Acc:HGNC:1678] | 37205 | -0.248 | -0.2199 | Yes |
| 115 | VCAM1 | vascular cell adhesion molecule 1 [Source:HGNC Symbol;Acc:HGNC:12663] | 37250 | -0.250 | -0.2067 | Yes |
| 116 | PDCD1LG2 | programmed cell death 1 ligand 2 [Source:HGNC Symbol;Acc:HGNC:18731] | 37384 | -0.258 | -0.1952 | Yes |
| 117 | CTLA4 | cytotoxic T-lymphocyte associated protein 4 [Source:HGNC Symbol;Acc:HGNC:2505] | 37400 | -0.259 | -0.1807 | Yes |
| 118 | PECAM1 | platelet and endothelial cell adhesion molecule 1 [Source:HGNC Symbol;Acc:HGNC:8823] | 37427 | -0.261 | -0.1664 | Yes |
| 119 | ITGAV | integrin subunit alpha V [Source:HGNC Symbol;Acc:HGNC:6150] | 37606 | -0.275 | -0.1551 | Yes |
| 120 | ICAM3 | intercellular adhesion molecule 3 [Source:HGNC Symbol;Acc:HGNC:5346] | 37616 | -0.276 | -0.1395 | Yes |
| 121 | ITGAL | integrin subunit alpha L [Source:HGNC Symbol;Acc:HGNC:6148] | 37624 | -0.276 | -0.1238 | Yes |
| 122 | CD86 | CD86 molecule [Source:HGNC Symbol;Acc:HGNC:1705] | 37625 | -0.276 | -0.1080 | Yes |
| 123 | CD80 | CD80 molecule [Source:HGNC Symbol;Acc:HGNC:1700] | 37726 | -0.285 | -0.0941 | Yes |
| 124 | CD28 | CD28 molecule [Source:HGNC Symbol;Acc:HGNC:1653] | 37779 | -0.289 | -0.0789 | Yes |
| 125 | VCAN | versican [Source:HGNC Symbol;Acc:HGNC:2464] | 37798 | -0.290 | -0.0627 | Yes |
| 126 | ITGB1 | integrin subunit beta 1 [Source:HGNC Symbol;Acc:HGNC:6153] | 37850 | -0.297 | -0.0469 | Yes |
| 127 | ITGB7 | integrin subunit beta 7 [Source:HGNC Symbol;Acc:HGNC:6162] | 38154 | -0.347 | -0.0347 | Yes |
| 128 | CD226 | CD226 molecule [Source:HGNC Symbol;Acc:HGNC:16961] | 38155 | -0.347 | -0.0148 | Yes |
| 129 | PTPRC | protein tyrosine phosphatase receptor type C [Source:HGNC Symbol;Acc:HGNC:9666] | 38216 | -0.370 | 0.0050 | Yes |
| 130 | ITGA4 | integrin subunit alpha 4 [Source:HGNC Symbol;Acc:HGNC:6140] | 38275 | -0.394 | 0.0261 | Yes |
| 131 | CLDN22 | claudin 22 [Source:HGNC Symbol;Acc:HGNC:2044] | 39071 | NaN | 0.0064 | Yes |
Table: GSEA details [plain text format]

  

Fig 2: KEGG\_CELL\_ADHESION\_MOLECULES\_CAMS      
 Blue-Pink O' Gram in the Space of the Analyzed GeneSet

  

Fig 3: KEGG\_CELL\_ADHESION\_MOLECULES\_CAMS: Random ES distribution      
 Gene set null distribution of ES for **KEGG\_CELL\_ADHESION\_MOLECULES\_CAMS**

  
