# Supplementary material for: Simultaneous Presentation of Multiple Myeloma and Lung Cancer: Case Report and Gene Bioinformatics Analysis
Source: Front Oncol. 2022 Jun 13;12:859735. doi: 10.3389/fonc.2022.859735 (PMC9235397; doi:10.3389/fonc.2022.859735)
Supplement: Supplementary file 1 [file DataSheet_1.zip › The bioinformatic analysis of MM and lung cancer supplementary materials/Enrichment analysis/MECR/GSEA_4.1.0/LUAD TCGA/KEGG.Gsea.1639041756227/KEGG_CHEMOKINE_SIGNALING_PATHWAY.html]

Details for gene set KEGG\_CHEMOKINE\_SIGNALING\_PATHWAY[GSEA]

|  || Dataset | ExpData\_collapsed\_to\_symbols.ENSG00000116353\_profile\_in\_ExpData.cls #ENSG00000116353 |
| Phenotype | ENSG00000116353\_profile\_in\_ExpData.cls#ENSG00000116353 |
| Upregulated in class | ENSG00000116353\_neg |
| GeneSet | KEGG\_CHEMOKINE\_SIGNALING\_PATHWAY |
| Enrichment Score (ES) | -0.5365851 |
| Normalized Enrichment Score (NES) | -2.4694626 |
| Nominal p-value | 0.0 |
| FDR q-value | 0.0 |
| FWER p-Value | 0.0 |
Table: GSEA Results Summary

  

Fig 1: Enrichment plot: KEGG\_CHEMOKINE\_SIGNALING\_PATHWAY      
 Profile of the Running ES Score & Positions of GeneSet Members on the Rank Ordered List

  

| SYMBOL | TITLE | RANK IN GENE LIST | RANK METRIC SCORE | RUNNING ES | CORE ENRICHMENT || 1 | PIK3R2 | phosphoinositide-3-kinase regulatory subunit 2 [Source:HGNC Symbol;Acc:HGNC:8980] | 545 | 0.314 | -0.0019 | No |
| 2 | PRKCZ | protein kinase C zeta [Source:HGNC Symbol;Acc:HGNC:9412] | 720 | 0.295 | 0.0049 | No |
| 3 | GNB2 | G protein subunit beta 2 [Source:HGNC Symbol;Acc:HGNC:4398] | 805 | 0.287 | 0.0138 | No |
| 4 | GNG5 | G protein subunit gamma 5 [Source:HGNC Symbol;Acc:HGNC:4408] | 1505 | 0.234 | 0.0049 | No |
| 5 | HRAS | "HRas proto-oncogene, GTPase [Source:HGNC Symbol;Acc:HGNC:5173]" | 1513 | 0.234 | 0.0137 | No |
| 6 | MAPK3 | mitogen-activated protein kinase 3 [Source:HGNC Symbol;Acc:HGNC:6877] | 1645 | 0.226 | 0.0190 | No |
| 7 | CDC42 | cell division cycle 42 [Source:HGNC Symbol;Acc:HGNC:1736] | 2158 | 0.198 | 0.0135 | No |
| 8 | RAC1 | Rac family small GTPase 1 [Source:HGNC Symbol;Acc:HGNC:9801] | 2566 | 0.182 | 0.0101 | No |
| 9 | AKT1 | AKT serine/threonine kinase 1 [Source:HGNC Symbol;Acc:HGNC:391] | 3179 | 0.158 | 0.0005 | No |
| 10 | CCL17 | C-C motif chemokine ligand 17 [Source:HGNC Symbol;Acc:HGNC:10615] | 3522 | 0.148 | -0.0026 | No |
| 11 | GRK6 | G protein-coupled receptor kinase 6 [Source:HGNC Symbol;Acc:HGNC:4545] | 4123 | 0.130 | -0.0129 | No |
| 12 | CCR3 | C-C motif chemokine receptor 3 [Source:HGNC Symbol;Acc:HGNC:1604] | 4418 | 0.123 | -0.0157 | No |
| 13 | BCAR1 | "BCAR1 scaffold protein, Cas family member [Source:HGNC Symbol;Acc:HGNC:971]" | 4546 | 0.121 | -0.0143 | No |
| 14 | SHC1 | SHC adaptor protein 1 [Source:HGNC Symbol;Acc:HGNC:10840] | 5181 | 0.108 | -0.0264 | No |
| 15 | NFKBIB | NFKB inhibitor beta [Source:HGNC Symbol;Acc:HGNC:7798] | 5484 | 0.102 | -0.0302 | No |
| 16 | RELA | "RELA proto-oncogene, NF-kB subunit [Source:HGNC Symbol;Acc:HGNC:9955]" | 6022 | 0.093 | -0.0404 | No |
| 17 | GNG13 | G protein subunit gamma 13 [Source:HGNC Symbol;Acc:HGNC:14131] | 6126 | 0.091 | -0.0395 | No |
| 18 | PLCB3 | phospholipase C beta 3 [Source:HGNC Symbol;Acc:HGNC:9056] | 6249 | 0.089 | -0.0392 | No |
| 19 | SHC2 | SHC adaptor protein 2 [Source:HGNC Symbol;Acc:HGNC:29869] | 6275 | 0.089 | -0.0364 | No |
| 20 | PRKACA | protein kinase cAMP-activated catalytic subunit alpha [Source:HGNC Symbol;Acc:HGNC:9380] | 6303 | 0.089 | -0.0337 | No |
| 21 | CXCL16 | C-X-C motif chemokine ligand 16 [Source:HGNC Symbol;Acc:HGNC:16642] | 6347 | 0.088 | -0.0314 | No |
| 22 | PTK2 | protein tyrosine kinase 2 [Source:HGNC Symbol;Acc:HGNC:9611] | 6431 | 0.087 | -0.0302 | No |
| 23 | GNB1 | G protein subunit beta 1 [Source:HGNC Symbol;Acc:HGNC:4396] | 7596 | 0.072 | -0.0572 | No |
| 24 | NFKBIA | NFKB inhibitor alpha [Source:HGNC Symbol;Acc:HGNC:7797] | 7696 | 0.071 | -0.0571 | No |
| 25 | GRK1 | G protein-coupled receptor kinase 1 [Source:HGNC Symbol;Acc:HGNC:10013] | 7728 | 0.070 | -0.0552 | No |
| 26 | GNG7 | G protein subunit gamma 7 [Source:HGNC Symbol;Acc:HGNC:4410] | 7890 | 0.068 | -0.0566 | No |
| 27 | PXN | paxillin [Source:HGNC Symbol;Acc:HGNC:9718] | 8446 | 0.063 | -0.0684 | No |
| 28 | CCL28 | C-C motif chemokine ligand 28 [Source:HGNC Symbol;Acc:HGNC:17700] | 8536 | 0.062 | -0.0683 | No |
| 29 | RAF1 | "Raf-1 proto-oncogene, serine/threonine kinase [Source:HGNC Symbol;Acc:HGNC:9829]" | 8635 | 0.061 | -0.0685 | No |
| 30 | PIK3R3 | phosphoinositide-3-kinase regulatory subunit 3 [Source:HGNC Symbol;Acc:HGNC:8981] | 9028 | 0.057 | -0.0763 | No |
| 31 | GNG8 | G protein subunit gamma 8 [Source:HGNC Symbol;Acc:HGNC:19664] | 9194 | 0.056 | -0.0784 | No |
| 32 | IKBKG | inhibitor of nuclear factor kappa B kinase regulatory subunit gamma [Source:HGNC Symbol;Acc:HGNC:5961] | 9304 | 0.055 | -0.0791 | No |
| 33 | GNG3 | G protein subunit gamma 3 [Source:HGNC Symbol;Acc:HGNC:4405] | 9317 | 0.055 | -0.0773 | No |
| 34 | AKT2 | AKT serine/threonine kinase 2 [Source:HGNC Symbol;Acc:HGNC:392] | 10579 | 0.044 | -0.1078 | No |
| 35 | PPBP | pro-platelet basic protein [Source:HGNC Symbol;Acc:HGNC:9240] | 10823 | 0.042 | -0.1124 | No |
| 36 | GNG11 | G protein subunit gamma 11 [Source:HGNC Symbol;Acc:HGNC:4403] | 11239 | 0.039 | -0.1215 | No |
| 37 | GNAI1 | G protein subunit alpha i1 [Source:HGNC Symbol;Acc:HGNC:4384] | 11243 | 0.039 | -0.1201 | No |
| 38 | RHOA | ras homolog family member A [Source:HGNC Symbol;Acc:HGNC:667] | 11437 | 0.037 | -0.1236 | No |
| 39 | GRK7 | G protein-coupled receptor kinase 7 [Source:HGNC Symbol;Acc:HGNC:17031] | 11734 | 0.035 | -0.1298 | No |
| 40 | PARD3 | par-3 family cell polarity regulator [Source:HGNC Symbol;Acc:HGNC:16051] | 11922 | 0.034 | -0.1333 | No |
| 41 | GSK3A | glycogen synthase kinase 3 alpha [Source:HGNC Symbol;Acc:HGNC:4616] | 12040 | 0.033 | -0.1350 | No |
| 42 | CX3CL1 | C-X3-C motif chemokine ligand 1 [Source:HGNC Symbol;Acc:HGNC:10647] | 12417 | 0.030 | -0.1435 | No |
| 43 | PF4 | platelet factor 4 [Source:HGNC Symbol;Acc:HGNC:8861] | 13825 | 0.020 | -0.1787 | No |
| 44 | CXCL14 | C-X-C motif chemokine ligand 14 [Source:HGNC Symbol;Acc:HGNC:10640] | 14753 | 0.015 | -0.2018 | No |
| 45 | ADCY2 | adenylate cyclase 2 [Source:HGNC Symbol;Acc:HGNC:233] | 14873 | 0.014 | -0.2043 | No |
| 46 | SHC3 | SHC adaptor protein 3 [Source:HGNC Symbol;Acc:HGNC:18181] | 14963 | 0.013 | -0.2061 | No |
| 47 | GNAI2 | G protein subunit alpha i2 [Source:HGNC Symbol;Acc:HGNC:4385] | 15695 | 0.009 | -0.2244 | No |
| 48 | ARRB1 | arrestin beta 1 [Source:HGNC Symbol;Acc:HGNC:711] | 15942 | 0.007 | -0.2304 | No |
| 49 | GRK4 | G protein-coupled receptor kinase 4 [Source:HGNC Symbol;Acc:HGNC:4543] | 16680 | 0.003 | -0.2491 | No |
| 50 | ARRB2 | arrestin beta 2 [Source:HGNC Symbol;Acc:HGNC:712] | 16701 | 0.003 | -0.2495 | No |
| 51 | GNGT1 | G protein subunit gamma transducin 1 [Source:HGNC Symbol;Acc:HGNC:4411] | 17415 | -0.001 | -0.2677 | No |
| 52 | XCL1 | X-C motif chemokine ligand 1 [Source:HGNC Symbol;Acc:HGNC:10645] | 17667 | -0.003 | -0.2740 | No |
| 53 | GNG12 | G protein subunit gamma 12 [Source:HGNC Symbol;Acc:HGNC:19663] | 17711 | -0.003 | -0.2750 | No |
| 54 | CCL27 | C-C motif chemokine ligand 27 [Source:HGNC Symbol;Acc:HGNC:10626] | 18208 | -0.006 | -0.2874 | No |
| 55 | PRKCD | protein kinase C delta [Source:HGNC Symbol;Acc:HGNC:9399] | 18388 | -0.007 | -0.2917 | No |
| 56 | CRK | "CRK proto-oncogene, adaptor protein [Source:HGNC Symbol;Acc:HGNC:2362]" | 18444 | -0.007 | -0.2928 | No |
| 57 | CX3CR1 | C-X3-C motif chemokine receptor 1 [Source:HGNC Symbol;Acc:HGNC:2558] | 18890 | -0.010 | -0.3038 | No |
| 58 | CSK | C-terminal Src kinase [Source:HGNC Symbol;Acc:HGNC:2444] | 19694 | -0.015 | -0.3238 | No |
| 59 | GNB5 | G protein subunit beta 5 [Source:HGNC Symbol;Acc:HGNC:4401] | 19805 | -0.016 | -0.3260 | No |
| 60 | CCL22 | C-C motif chemokine ligand 22 [Source:HGNC Symbol;Acc:HGNC:10621] | 20341 | -0.019 | -0.3389 | No |
| 61 | RAP1A | "RAP1A, member of RAS oncogene family [Source:HGNC Symbol;Acc:HGNC:9855]" | 20560 | -0.020 | -0.3437 | No |
| 62 | IKBKB | inhibitor of nuclear factor kappa B kinase subunit beta [Source:HGNC Symbol;Acc:HGNC:5960] | 21393 | -0.025 | -0.3640 | No |
| 63 | WASL | WASP like actin nucleation promoting factor [Source:HGNC Symbol;Acc:HGNC:12735] | 22111 | -0.029 | -0.3812 | No |
| 64 | ADCY9 | adenylate cyclase 9 [Source:HGNC Symbol;Acc:HGNC:240] | 22247 | -0.030 | -0.3835 | No |
| 65 | CXCL2 | C-X-C motif chemokine ligand 2 [Source:HGNC Symbol;Acc:HGNC:4603] | 22636 | -0.033 | -0.3922 | No |
| 66 | CCL13 | C-C motif chemokine ligand 13 [Source:HGNC Symbol;Acc:HGNC:10611] | 22728 | -0.033 | -0.3932 | No |
| 67 | CCL7 | C-C motif chemokine ligand 7 [Source:HGNC Symbol;Acc:HGNC:10634] | 23180 | -0.037 | -0.4033 | No |
| 68 | GNGT2 | G protein subunit gamma transducin 2 [Source:HGNC Symbol;Acc:HGNC:4412] | 23834 | -0.041 | -0.4185 | No |
| 69 | RAC2 | Rac family small GTPase 2 [Source:HGNC Symbol;Acc:HGNC:9802] | 24228 | -0.043 | -0.4268 | No |
| 70 | SHC4 | SHC adaptor protein 4 [Source:HGNC Symbol;Acc:HGNC:16743] | 24342 | -0.044 | -0.4281 | No |
| 71 | CCL1 | C-C motif chemokine ligand 1 [Source:HGNC Symbol;Acc:HGNC:10609] | 24386 | -0.044 | -0.4275 | No |
| 72 | CCL11 | C-C motif chemokine ligand 11 [Source:HGNC Symbol;Acc:HGNC:10610] | 24418 | -0.045 | -0.4265 | No |
| 73 | VAV2 | vav guanine nucleotide exchange factor 2 [Source:HGNC Symbol;Acc:HGNC:12658] | 24578 | -0.046 | -0.4288 | No |
| 74 | ADCY1 | adenylate cyclase 1 [Source:HGNC Symbol;Acc:HGNC:232] | 24582 | -0.046 | -0.4272 | No |
| 75 | CCR10 | C-C motif chemokine receptor 10 [Source:HGNC Symbol;Acc:HGNC:4474] | 24820 | -0.047 | -0.4314 | No |
| 76 | GNG10 | G protein subunit gamma 10 [Source:HGNC Symbol;Acc:HGNC:4402] | 25664 | -0.053 | -0.4509 | No |
| 77 | PPBPP1 | pro-platelet basic protein pseudogene 1 [Source:HGNC Symbol;Acc:HGNC:9241] | 25765 | -0.054 | -0.4514 | No |
| 78 | CCL26 | C-C motif chemokine ligand 26 [Source:HGNC Symbol;Acc:HGNC:10625] | 26486 | -0.059 | -0.4675 | No |
| 79 | PF4V1 | platelet factor 4 variant 1 [Source:HGNC Symbol;Acc:HGNC:8862] | 26985 | -0.063 | -0.4778 | No |
| 80 | GRK2 | G protein-coupled receptor kinase 2 [Source:HGNC Symbol;Acc:HGNC:289] | 27682 | -0.069 | -0.4930 | No |
| 81 | PLCB4 | phospholipase C beta 4 [Source:HGNC Symbol;Acc:HGNC:9059] | 27781 | -0.069 | -0.4928 | No |
| 82 | XCL2 | X-C motif chemokine ligand 2 [Source:HGNC Symbol;Acc:HGNC:10646] | 27801 | -0.069 | -0.4907 | No |
| 83 | ADCY8 | adenylate cyclase 8 [Source:HGNC Symbol;Acc:HGNC:239] | 27849 | -0.070 | -0.4892 | No |
| 84 | LYN | "LYN proto-oncogene, Src family tyrosine kinase [Source:HGNC Symbol;Acc:HGNC:6735]" | 28456 | -0.075 | -0.5018 | No |
| 85 | CCL19 | C-C motif chemokine ligand 19 [Source:HGNC Symbol;Acc:HGNC:10617] | 28473 | -0.075 | -0.4993 | No |
| 86 | CXCL1 | C-X-C motif chemokine ligand 1 [Source:HGNC Symbol;Acc:HGNC:4602] | 28932 | -0.079 | -0.5080 | No |
| 87 | CCL15 | C-C motif chemokine ligand 15 [Source:HGNC Symbol;Acc:HGNC:10613] | 28986 | -0.080 | -0.5063 | No |
| 88 | VAV3 | vav guanine nucleotide exchange factor 3 [Source:HGNC Symbol;Acc:HGNC:12659] | 29000 | -0.080 | -0.5036 | No |
| 89 | PAK1 | p21 (RAC1) activated kinase 1 [Source:HGNC Symbol;Acc:HGNC:8590] | 29530 | -0.085 | -0.5138 | No |
| 90 | BRAF | "B-Raf proto-oncogene, serine/threonine kinase [Source:HGNC Symbol;Acc:HGNC:1097]" | 29630 | -0.086 | -0.5131 | No |
| 91 | ADCY6 | adenylate cyclase 6 [Source:HGNC Symbol;Acc:HGNC:237] | 29658 | -0.086 | -0.5105 | No |
| 92 | GNAI3 | G protein subunit alpha i3 [Source:HGNC Symbol;Acc:HGNC:4387] | 30483 | -0.095 | -0.5279 | No |
| 93 | ADCY4 | adenylate cyclase 4 [Source:HGNC Symbol;Acc:HGNC:235] | 30750 | -0.098 | -0.5310 | No |
| 94 | RASGRP2 | RAS guanyl releasing protein 2 [Source:HGNC Symbol;Acc:HGNC:9879] | 30829 | -0.098 | -0.5292 | No |
| 95 | CXCL3 | C-X-C motif chemokine ligand 3 [Source:HGNC Symbol;Acc:HGNC:4604] | 31096 | -0.102 | -0.5321 | No |
| 96 | CCL14 | C-C motif chemokine ligand 14 [Source:HGNC Symbol;Acc:HGNC:10612] | 31273 | -0.104 | -0.5326 | Yes |
| 97 | CXCL11 | C-X-C motif chemokine ligand 11 [Source:HGNC Symbol;Acc:HGNC:10638] | 31326 | -0.104 | -0.5299 | Yes |
| 98 | CCL23 | C-C motif chemokine ligand 23 [Source:HGNC Symbol;Acc:HGNC:10622] | 31536 | -0.107 | -0.5312 | Yes |
| 99 | CCL3L3 | C-C motif chemokine ligand 3 like 3 [Source:HGNC Symbol;Acc:HGNC:30554] | 31590 | -0.108 | -0.5284 | Yes |
| 100 | CCL16 | C-C motif chemokine ligand 16 [Source:HGNC Symbol;Acc:HGNC:10614] | 31612 | -0.108 | -0.5248 | Yes |
| 101 | ELMO1 | engulfment and cell motility 1 [Source:HGNC Symbol;Acc:HGNC:16286] | 31667 | -0.109 | -0.5220 | Yes |
| 102 | CXCR3 | C-X-C motif chemokine receptor 3 [Source:HGNC Symbol;Acc:HGNC:4540] | 31733 | -0.110 | -0.5195 | Yes |
| 103 | CXCL8 | C-X-C motif chemokine ligand 8 [Source:HGNC Symbol;Acc:HGNC:6025] | 31746 | -0.110 | -0.5156 | Yes |
| 104 | PIK3CD | "phosphatidylinositol-4,5-bisphosphate 3-kinase catalytic subunit delta [Source:HGNC Symbol;Acc:HGNC:8977]" | 31859 | -0.111 | -0.5142 | Yes |
| 105 | CCL2 | C-C motif chemokine ligand 2 [Source:HGNC Symbol;Acc:HGNC:10618] | 31950 | -0.112 | -0.5122 | Yes |
| 106 | CCL21 | C-C motif chemokine ligand 21 [Source:HGNC Symbol;Acc:HGNC:10620] | 32114 | -0.115 | -0.5120 | Yes |
| 107 | GNG4 | G protein subunit gamma 4 [Source:HGNC Symbol;Acc:HGNC:4407] | 32126 | -0.115 | -0.5079 | Yes |
| 108 | PRKACG | protein kinase cAMP-activated catalytic subunit gamma [Source:HGNC Symbol;Acc:HGNC:9382] | 33040 | -0.129 | -0.5263 | Yes |
| 109 | CCL4L2 | C-C motif chemokine ligand 4 like 2 [Source:HGNC Symbol;Acc:HGNC:24066] | 33115 | -0.130 | -0.5232 | Yes |
| 110 | CCL5 | C-C motif chemokine ligand 5 [Source:HGNC Symbol;Acc:HGNC:10632] | 33180 | -0.131 | -0.5198 | Yes |
| 111 | GNB3 | G protein subunit beta 3 [Source:HGNC Symbol;Acc:HGNC:4400] | 33262 | -0.132 | -0.5168 | Yes |
| 112 | CCL20 | C-C motif chemokine ligand 20 [Source:HGNC Symbol;Acc:HGNC:10619] | 33317 | -0.133 | -0.5131 | Yes |
| 113 | CXCL6 | C-X-C motif chemokine ligand 6 [Source:HGNC Symbol;Acc:HGNC:10643] | 33379 | -0.134 | -0.5095 | Yes |
| 114 | CCR7 | C-C motif chemokine receptor 7 [Source:HGNC Symbol;Acc:HGNC:1608] | 33392 | -0.135 | -0.5047 | Yes |
| 115 | CXCR5 | C-X-C motif chemokine receptor 5 [Source:HGNC Symbol;Acc:HGNC:1060] | 33556 | -0.137 | -0.5036 | Yes |
| 116 | MAPK1 | mitogen-activated protein kinase 1 [Source:HGNC Symbol;Acc:HGNC:6871] | 33636 | -0.139 | -0.5003 | Yes |
| 117 | PLCB2 | phospholipase C beta 2 [Source:HGNC Symbol;Acc:HGNC:9055] | 33768 | -0.141 | -0.4983 | Yes |
| 118 | CXCL13 | C-X-C motif chemokine ligand 13 [Source:HGNC Symbol;Acc:HGNC:10639] | 34100 | -0.147 | -0.5011 | Yes |
| 119 | CCL8 | C-C motif chemokine ligand 8 [Source:HGNC Symbol;Acc:HGNC:10635] | 34171 | -0.149 | -0.4972 | Yes |
| 120 | STAT3 | signal transducer and activator of transcription 3 [Source:HGNC Symbol;Acc:HGNC:11364] | 34312 | -0.151 | -0.4950 | Yes |
| 121 | CRKL | "CRK like proto-oncogene, adaptor protein [Source:HGNC Symbol;Acc:HGNC:2363]" | 34421 | -0.154 | -0.4918 | Yes |
| 122 | CCR9 | C-C motif chemokine receptor 9 [Source:HGNC Symbol;Acc:HGNC:1610] | 34438 | -0.154 | -0.4863 | Yes |
| 123 | JAK3 | Janus kinase 3 [Source:HGNC Symbol;Acc:HGNC:6193] | 34533 | -0.155 | -0.4828 | Yes |
| 124 | NRAS | "NRAS proto-oncogene, GTPase [Source:HGNC Symbol;Acc:HGNC:7989]" | 34553 | -0.156 | -0.4773 | Yes |
| 125 | RAP1B | "RAP1B, member of RAS oncogene family [Source:HGNC Symbol;Acc:HGNC:9857]" | 34571 | -0.156 | -0.4717 | Yes |
| 126 | SOS2 | SOS Ras/Rho guanine nucleotide exchange factor 2 [Source:HGNC Symbol;Acc:HGNC:11188] | 34598 | -0.157 | -0.4664 | Yes |
| 127 | CCL3 | C-C motif chemokine ligand 3 [Source:HGNC Symbol;Acc:HGNC:10627] | 34673 | -0.159 | -0.4622 | Yes |
| 128 | CXCL5 | C-X-C motif chemokine ligand 5 [Source:HGNC Symbol;Acc:HGNC:10642] | 34783 | -0.162 | -0.4588 | Yes |
| 129 | PIK3R1 | phosphoinositide-3-kinase regulatory subunit 1 [Source:HGNC Symbol;Acc:HGNC:8979] | 34788 | -0.162 | -0.4527 | Yes |
| 130 | TIAM2 | TIAM Rac1 associated GEF 2 [Source:HGNC Symbol;Acc:HGNC:11806] | 34809 | -0.162 | -0.4470 | Yes |
| 131 | VAV1 | vav guanine nucleotide exchange factor 1 [Source:HGNC Symbol;Acc:HGNC:12657] | 34853 | -0.163 | -0.4419 | Yes |
| 132 | PIK3CB | "phosphatidylinositol-4,5-bisphosphate 3-kinase catalytic subunit beta [Source:HGNC Symbol;Acc:HGNC:8976]" | 34871 | -0.164 | -0.4360 | Yes |
| 133 | CCL25 | C-C motif chemokine ligand 25 [Source:HGNC Symbol;Acc:HGNC:10624] | 35008 | -0.166 | -0.4331 | Yes |
| 134 | CXCL10 | C-X-C motif chemokine ligand 10 [Source:HGNC Symbol;Acc:HGNC:10637] | 35086 | -0.168 | -0.4286 | Yes |
| 135 | CXCR1 | C-X-C motif chemokine receptor 1 [Source:HGNC Symbol;Acc:HGNC:6026] | 35184 | -0.171 | -0.4246 | Yes |
| 136 | MAP2K1 | mitogen-activated protein kinase kinase 1 [Source:HGNC Symbol;Acc:HGNC:6840] | 35210 | -0.171 | -0.4187 | Yes |
| 137 | TIAM1 | TIAM Rac1 associated GEF 1 [Source:HGNC Symbol;Acc:HGNC:11805] | 35341 | -0.175 | -0.4153 | Yes |
| 138 | STAT2 | signal transducer and activator of transcription 2 [Source:HGNC Symbol;Acc:HGNC:11363] | 35536 | -0.180 | -0.4133 | Yes |
| 139 | ADCY5 | adenylate cyclase 5 [Source:HGNC Symbol;Acc:HGNC:236] | 35664 | -0.184 | -0.4095 | Yes |
| 140 | FOXO3 | forkhead box O3 [Source:HGNC Symbol;Acc:HGNC:3821] | 35897 | -0.191 | -0.4082 | Yes |
| 141 | CXCL12 | C-X-C motif chemokine ligand 12 [Source:HGNC Symbol;Acc:HGNC:10672] | 35899 | -0.191 | -0.4009 | Yes |
| 142 | WAS | WASP actin nucleation promoting factor [Source:HGNC Symbol;Acc:HGNC:12731] | 36061 | -0.196 | -0.3975 | Yes |
| 143 | ADCY7 | adenylate cyclase 7 [Source:HGNC Symbol;Acc:HGNC:238] | 36103 | -0.197 | -0.3910 | Yes |
| 144 | ADCY3 | adenylate cyclase 3 [Source:HGNC Symbol;Acc:HGNC:234] | 36213 | -0.201 | -0.3861 | Yes |
| 145 | NFKB1 | nuclear factor kappa B subunit 1 [Source:HGNC Symbol;Acc:HGNC:7794] | 36367 | -0.207 | -0.3821 | Yes |
| 146 | FGR | "FGR proto-oncogene, Src family tyrosine kinase [Source:HGNC Symbol;Acc:HGNC:3697]" | 36382 | -0.207 | -0.3745 | Yes |
| 147 | PLCB1 | phospholipase C beta 1 [Source:HGNC Symbol;Acc:HGNC:15917] | 36397 | -0.208 | -0.3669 | Yes |
| 148 | HCK | "HCK proto-oncogene, Src family tyrosine kinase [Source:HGNC Symbol;Acc:HGNC:4840]" | 36406 | -0.208 | -0.3591 | Yes |
| 149 | GRB2 | growth factor receptor bound protein 2 [Source:HGNC Symbol;Acc:HGNC:4566] | 36516 | -0.212 | -0.3538 | Yes |
| 150 | GRK3 | G protein-coupled receptor kinase 3 [Source:HGNC Symbol;Acc:HGNC:290] | 36549 | -0.213 | -0.3465 | Yes |
| 151 | CXCR4 | C-X-C motif chemokine receptor 4 [Source:HGNC Symbol;Acc:HGNC:2561] | 36579 | -0.215 | -0.3390 | Yes |
| 152 | CCR6 | C-C motif chemokine receptor 6 [Source:HGNC Symbol;Acc:HGNC:1607] | 36663 | -0.218 | -0.3327 | Yes |
| 153 | CCL18 | C-C motif chemokine ligand 18 [Source:HGNC Symbol;Acc:HGNC:10616] | 36793 | -0.225 | -0.3274 | Yes |
| 154 | CXCR2 | C-X-C motif chemokine receptor 2 [Source:HGNC Symbol;Acc:HGNC:6027] | 36832 | -0.227 | -0.3197 | Yes |
| 155 | PRKACB | protein kinase cAMP-activated catalytic subunit beta [Source:HGNC Symbol;Acc:HGNC:9381] | 36870 | -0.229 | -0.3119 | Yes |
| 156 | CXCL9 | C-X-C motif chemokine ligand 9 [Source:HGNC Symbol;Acc:HGNC:7098] | 36887 | -0.230 | -0.3035 | Yes |
| 157 | CCR8 | C-C motif chemokine receptor 8 [Source:HGNC Symbol;Acc:HGNC:1609] | 36930 | -0.232 | -0.2957 | Yes |
| 158 | NCF1 | neutrophil cytosolic factor 1 [Source:HGNC Symbol;Acc:HGNC:7660] | 36975 | -0.234 | -0.2879 | Yes |
| 159 | CCL4 | C-C motif chemokine ligand 4 [Source:HGNC Symbol;Acc:HGNC:10630] | 36977 | -0.234 | -0.2789 | Yes |
| 160 | PTK2B | protein tyrosine kinase 2 beta [Source:HGNC Symbol;Acc:HGNC:9612] | 37013 | -0.237 | -0.2708 | Yes |
| 161 | XCR1 | X-C motif chemokine receptor 1 [Source:HGNC Symbol;Acc:HGNC:1625] | 37135 | -0.244 | -0.2645 | Yes |
| 162 | STAT1 | signal transducer and activator of transcription 1 [Source:HGNC Symbol;Acc:HGNC:11362] | 37262 | -0.251 | -0.2581 | Yes |
| 163 | CCR2 | C-C motif chemokine receptor 2 [Source:HGNC Symbol;Acc:HGNC:1603] | 37267 | -0.252 | -0.2486 | Yes |
| 164 | CCR4 | C-C motif chemokine receptor 4 [Source:HGNC Symbol;Acc:HGNC:1605] | 37467 | -0.264 | -0.2436 | Yes |
| 165 | CXCR6 | C-X-C motif chemokine receptor 6 [Source:HGNC Symbol;Acc:HGNC:16647] | 37475 | -0.264 | -0.2336 | Yes |
| 166 | GNG2 | G protein subunit gamma 2 [Source:HGNC Symbol;Acc:HGNC:4404] | 37531 | -0.268 | -0.2248 | Yes |
| 167 | PRKX | protein kinase X-linked [Source:HGNC Symbol;Acc:HGNC:9441] | 37543 | -0.269 | -0.2147 | Yes |
| 168 | PIK3CA | "phosphatidylinositol-4,5-bisphosphate 3-kinase catalytic subunit alpha [Source:HGNC Symbol;Acc:HGNC:8975]" | 37556 | -0.270 | -0.2047 | Yes |
| 169 | GSK3B | glycogen synthase kinase 3 beta [Source:HGNC Symbol;Acc:HGNC:4617] | 37592 | -0.274 | -0.1951 | Yes |
| 170 | KRAS | "KRAS proto-oncogene, GTPase [Source:HGNC Symbol;Acc:HGNC:6407]" | 37629 | -0.277 | -0.1854 | Yes |
| 171 | CCL24 | C-C motif chemokine ligand 24 [Source:HGNC Symbol;Acc:HGNC:10623] | 37740 | -0.286 | -0.1773 | Yes |
| 172 | STAT5B | signal transducer and activator of transcription 5B [Source:HGNC Symbol;Acc:HGNC:11367] | 37756 | -0.287 | -0.1667 | Yes |
| 173 | CHUK | component of inhibitor of nuclear factor kappa B kinase complex [Source:HGNC Symbol;Acc:HGNC:1974] | 37762 | -0.287 | -0.1558 | Yes |
| 174 | PRKCB | protein kinase C beta [Source:HGNC Symbol;Acc:HGNC:9395] | 37822 | -0.293 | -0.1461 | Yes |
| 175 | CCR1 | C-C motif chemokine receptor 1 [Source:HGNC Symbol;Acc:HGNC:1602] | 37830 | -0.294 | -0.1350 | Yes |
| 176 | GRK5 | G protein-coupled receptor kinase 5 [Source:HGNC Symbol;Acc:HGNC:4544] | 37885 | -0.300 | -0.1249 | Yes |
| 177 | PREX1 | "phosphatidylinositol-3,4,5-trisphosphate dependent Rac exchange factor 1 [Source:HGNC Symbol;Acc:HGNC:32594]" | 37940 | -0.309 | -0.1144 | Yes |
| 178 | CCR5 | C-C motif chemokine receptor 5 [Source:HGNC Symbol;Acc:HGNC:1606] | 37948 | -0.309 | -0.1028 | Yes |
| 179 | PIK3R5 | phosphoinositide-3-kinase regulatory subunit 5 [Source:HGNC Symbol;Acc:HGNC:30035] | 37956 | -0.310 | -0.0911 | Yes |
| 180 | ITK | IL2 inducible T cell kinase [Source:HGNC Symbol;Acc:HGNC:6171] | 38024 | -0.320 | -0.0805 | Yes |
| 181 | SOS1 | SOS Ras/Rac guanine nucleotide exchange factor 1 [Source:HGNC Symbol;Acc:HGNC:11187] | 38132 | -0.341 | -0.0702 | Yes |
| 182 | DOCK2 | dedicator of cytokinesis 2 [Source:HGNC Symbol;Acc:HGNC:2988] | 38142 | -0.345 | -0.0572 | Yes |
| 183 | GNB4 | G protein subunit beta 4 [Source:HGNC Symbol;Acc:HGNC:20731] | 38158 | -0.348 | -0.0442 | Yes |
| 184 | AKT3 | AKT serine/threonine kinase 3 [Source:HGNC Symbol;Acc:HGNC:393] | 38196 | -0.362 | -0.0313 | Yes |
| 185 | ROCK2 | Rho associated coiled-coil containing protein kinase 2 [Source:HGNC Symbol;Acc:HGNC:10252] | 38215 | -0.370 | -0.0176 | Yes |
| 186 | JAK2 | Janus kinase 2 [Source:HGNC Symbol;Acc:HGNC:6192] | 38251 | -0.384 | -0.0038 | Yes |
| 187 | ROCK1 | Rho associated coiled-coil containing protein kinase 1 [Source:HGNC Symbol;Acc:HGNC:10251] | 38260 | -0.387 | 0.0108 | Yes |
| 188 | PIK3CG | "phosphatidylinositol-4,5-bisphosphate 3-kinase catalytic subunit gamma [Source:HGNC Symbol;Acc:HGNC:8978]" | 38302 | -0.426 | 0.0261 | Yes |
Table: GSEA details [plain text format]

  

Fig 2: KEGG\_CHEMOKINE\_SIGNALING\_PATHWAY      
 Blue-Pink O' Gram in the Space of the Analyzed GeneSet

  

Fig 3: KEGG\_CHEMOKINE\_SIGNALING\_PATHWAY: Random ES distribution      
 Gene set null distribution of ES for **KEGG\_CHEMOKINE\_SIGNALING\_PATHWAY**

  
