# Supplementary material for: Simultaneous Presentation of Multiple Myeloma and Lung Cancer: Case Report and Gene Bioinformatics Analysis
Source: Front Oncol. 2022 Jun 13;12:859735. doi: 10.3389/fonc.2022.859735 (PMC9235397; doi:10.3389/fonc.2022.859735)
Supplement: Supplementary file 1 [file DataSheet_1.zip › The bioinformatic analysis of MM and lung cancer supplementary materials/Enrichment analysis/MECR/GSEA_4.1.0/LUAD TCGA/KEGG.Gsea.1639041756227/KEGG_CITRATE_CYCLE_TCA_CYCLE.html]

Details for gene set KEGG\_CITRATE\_CYCLE\_TCA\_CYCLE[GSEA]

|  || Dataset | ExpData\_collapsed\_to\_symbols.ENSG00000116353\_profile\_in\_ExpData.cls #ENSG00000116353 |
| Phenotype | ENSG00000116353\_profile\_in\_ExpData.cls#ENSG00000116353 |
| Upregulated in class | ENSG00000116353\_pos |
| GeneSet | KEGG\_CITRATE\_CYCLE\_TCA\_CYCLE |
| Enrichment Score (ES) | 0.51688945 |
| Normalized Enrichment Score (NES) | 1.7221179 |
| Nominal p-value | 0.015151516 |
| FDR q-value | 0.015436861 |
| FWER p-Value | 0.303 |
Table: GSEA Results Summary

  

Fig 1: Enrichment plot: KEGG\_CITRATE\_CYCLE\_TCA\_CYCLE      
 Profile of the Running ES Score & Positions of GeneSet Members on the Rank Ordered List

  

| SYMBOL | TITLE | RANK IN GENE LIST | RANK METRIC SCORE | RUNNING ES | CORE ENRICHMENT || 1 | SDHB | succinate dehydrogenase complex iron sulfur subunit B [Source:HGNC Symbol;Acc:HGNC:10681] | 443 | 0.327 | 0.0683 | Yes |
| 2 | IDH3G | isocitrate dehydrogenase (NAD(+)) 3 non-catalytic subunit gamma [Source:HGNC Symbol;Acc:HGNC:5386] | 1193 | 0.255 | 0.1115 | Yes |
| 3 | MDH2 | malate dehydrogenase 2 [Source:HGNC Symbol;Acc:HGNC:6971] | 1614 | 0.227 | 0.1563 | Yes |
| 4 | PC | pyruvate carboxylase [Source:HGNC Symbol;Acc:HGNC:8636] | 1784 | 0.218 | 0.2051 | Yes |
| 5 | SDHC | succinate dehydrogenase complex subunit C [Source:HGNC Symbol;Acc:HGNC:10682] | 2041 | 0.204 | 0.2483 | Yes |
| 6 | FH | fumarate hydratase [Source:HGNC Symbol;Acc:HGNC:3700] | 2055 | 0.203 | 0.2976 | Yes |
| 7 | SUCLG1 | succinate-CoA ligase GDP/ADP-forming subunit alpha [Source:HGNC Symbol;Acc:HGNC:11449] | 2765 | 0.173 | 0.3217 | Yes |
| 8 | SUCLG2 | succinate-CoA ligase GDP-forming subunit beta [Source:HGNC Symbol;Acc:HGNC:11450] | 3409 | 0.151 | 0.3422 | Yes |
| 9 | PDHB | pyruvate dehydrogenase E1 subunit beta [Source:HGNC Symbol;Acc:HGNC:8808] | 3452 | 0.150 | 0.3776 | Yes |
| 10 | IDH3B | isocitrate dehydrogenase (NAD(+)) 3 non-catalytic subunit beta [Source:HGNC Symbol;Acc:HGNC:5385] | 3459 | 0.150 | 0.4140 | Yes |
| 11 | MDH1 | malate dehydrogenase 1 [Source:HGNC Symbol;Acc:HGNC:6970] | 3847 | 0.138 | 0.4378 | Yes |
| 12 | SDHA | succinate dehydrogenase complex flavoprotein subunit A [Source:HGNC Symbol;Acc:HGNC:10680] | 4074 | 0.132 | 0.4641 | Yes |
| 13 | PCK2 | "phosphoenolpyruvate carboxykinase 2, mitochondrial [Source:HGNC Symbol;Acc:HGNC:8725]" | 4563 | 0.120 | 0.4810 | Yes |
| 14 | PDHA1 | pyruvate dehydrogenase E1 subunit alpha 1 [Source:HGNC Symbol;Acc:HGNC:8806] | 5654 | 0.099 | 0.4775 | Yes |
| 15 | SDHD | succinate dehydrogenase complex subunit D [Source:HGNC Symbol;Acc:HGNC:10683] | 5864 | 0.096 | 0.4955 | Yes |
| 16 | IDH2 | isocitrate dehydrogenase (NADP(+)) 2 [Source:HGNC Symbol;Acc:HGNC:5383] | 5934 | 0.095 | 0.5169 | Yes |
| 17 | IDH1 | isocitrate dehydrogenase (NADP(+)) 1 [Source:HGNC Symbol;Acc:HGNC:5382] | 7478 | 0.073 | 0.4955 | No |
| 18 | OGDHL | oxoglutarate dehydrogenase L [Source:HGNC Symbol;Acc:HGNC:25590] | 7891 | 0.068 | 0.5017 | No |
| 19 | OGDH | oxoglutarate dehydrogenase [Source:HGNC Symbol;Acc:HGNC:8124] | 8661 | 0.061 | 0.4970 | No |
| 20 | ACO2 | aconitase 2 [Source:HGNC Symbol;Acc:HGNC:118] | 10882 | 0.042 | 0.4507 | No |
| 21 | SUCLG2P2 | SUCLG2 pseudogene 2 [Source:HGNC Symbol;Acc:HGNC:43997] | 17588 | -0.002 | 0.2806 | No |
| 22 | DLST | dihydrolipoamide S-succinyltransferase [Source:HGNC Symbol;Acc:HGNC:2911] | 19838 | -0.016 | 0.2272 | No |
| 23 | PDHA2 | pyruvate dehydrogenase E1 subunit alpha 2 [Source:HGNC Symbol;Acc:HGNC:8807] | 20512 | -0.020 | 0.2149 | No |
| 24 | DLD | dihydrolipoamide dehydrogenase [Source:HGNC Symbol;Acc:HGNC:2898] | 25309 | -0.051 | 0.1053 | No |
| 25 | DLAT | dihydrolipoamide S-acetyltransferase [Source:HGNC Symbol;Acc:HGNC:2896] | 25670 | -0.053 | 0.1091 | No |
| 26 | SUCLA2 | succinate-CoA ligase ADP-forming subunit beta [Source:HGNC Symbol;Acc:HGNC:11448] | 30414 | -0.094 | 0.0113 | No |
| 27 | PCK1 | phosphoenolpyruvate carboxykinase 1 [Source:HGNC Symbol;Acc:HGNC:8724] | 32207 | -0.116 | -0.0061 | No |
| 28 | ACO1 | aconitase 1 [Source:HGNC Symbol;Acc:HGNC:117] | 34008 | -0.145 | -0.0164 | No |
| 29 | CS | citrate synthase [Source:HGNC Symbol;Acc:HGNC:2422] | 34962 | -0.165 | -0.0004 | No |
| 30 | ACLY | ATP citrate lyase [Source:HGNC Symbol;Acc:HGNC:115] | 35619 | -0.183 | 0.0274 | No |
| 31 | IDH3A | isocitrate dehydrogenase (NAD(+)) 3 catalytic subunit alpha [Source:HGNC Symbol;Acc:HGNC:5384] | 37600 | -0.274 | 0.0438 | No |
Table: GSEA details [plain text format]

  

Fig 2: KEGG\_CITRATE\_CYCLE\_TCA\_CYCLE      
 Blue-Pink O' Gram in the Space of the Analyzed GeneSet

  

Fig 3: KEGG\_CITRATE\_CYCLE\_TCA\_CYCLE: Random ES distribution      
 Gene set null distribution of ES for **KEGG\_CITRATE\_CYCLE\_TCA\_CYCLE**

  
