# Supplementary material for: Simultaneous Presentation of Multiple Myeloma and Lung Cancer: Case Report and Gene Bioinformatics Analysis
Source: Front Oncol. 2022 Jun 13;12:859735. doi: 10.3389/fonc.2022.859735 (PMC9235397; doi:10.3389/fonc.2022.859735)
Supplement: Supplementary file 1 [file DataSheet_1.zip › The bioinformatic analysis of MM and lung cancer supplementary materials/Enrichment analysis/MECR/GSEA_4.1.0/LUAD TCGA/KEGG.Gsea.1639041756227/KEGG_CYTOKINE_CYTOKINE_RECEPTOR_INTERACTION.html]

Details for gene set KEGG\_CYTOKINE\_CYTOKINE\_RECEPTOR\_INTERACTION[GSEA]

|  || Dataset | ExpData\_collapsed\_to\_symbols.ENSG00000116353\_profile\_in\_ExpData.cls #ENSG00000116353 |
| Phenotype | ENSG00000116353\_profile\_in\_ExpData.cls#ENSG00000116353 |
| Upregulated in class | ENSG00000116353\_neg |
| GeneSet | KEGG\_CYTOKINE\_CYTOKINE\_RECEPTOR\_INTERACTION |
| Enrichment Score (ES) | -0.51484996 |
| Normalized Enrichment Score (NES) | -2.4488065 |
| Nominal p-value | 0.0 |
| FDR q-value | 0.0 |
| FWER p-Value | 0.0 |
Table: GSEA Results Summary

  

Fig 1: Enrichment plot: KEGG\_CYTOKINE\_CYTOKINE\_RECEPTOR\_INTERACTION      
 Profile of the Running ES Score & Positions of GeneSet Members on the Rank Ordered List

  

| SYMBOL | TITLE | RANK IN GENE LIST | RANK METRIC SCORE | RUNNING ES | CORE ENRICHMENT || 1 | VEGFB | vascular endothelial growth factor B [Source:HGNC Symbol;Acc:HGNC:12681] | 586 | 0.309 | -0.0054 | No |
| 2 | TNFRSF12A | TNF receptor superfamily member 12A [Source:HGNC Symbol;Acc:HGNC:18152] | 617 | 0.306 | 0.0034 | No |
| 3 | CTF1 | cardiotrophin 1 [Source:HGNC Symbol;Acc:HGNC:2499] | 1519 | 0.234 | -0.0124 | No |
| 4 | GDF5 | growth differentiation factor 5 [Source:HGNC Symbol;Acc:HGNC:4220] | 2574 | 0.181 | -0.0338 | No |
| 5 | IL11RA | interleukin 11 receptor subunit alpha [Source:HGNC Symbol;Acc:HGNC:5967] | 2627 | 0.179 | -0.0295 | No |
| 6 | IFNGR2 | interferon gamma receptor 2 [Source:HGNC Symbol;Acc:HGNC:5440] | 2702 | 0.176 | -0.0260 | No |
| 7 | PDGFA | platelet derived growth factor subunit A [Source:HGNC Symbol;Acc:HGNC:8799] | 2710 | 0.175 | -0.0207 | No |
| 8 | TNFSF9 | TNF superfamily member 9 [Source:HGNC Symbol;Acc:HGNC:11939] | 3134 | 0.160 | -0.0266 | No |
| 9 | CLCF1 | cardiotrophin like cytokine factor 1 [Source:HGNC Symbol;Acc:HGNC:17412] | 3362 | 0.152 | -0.0276 | No |
| 10 | CCL17 | C-C motif chemokine ligand 17 [Source:HGNC Symbol;Acc:HGNC:10615] | 3522 | 0.148 | -0.0271 | No |
| 11 | TNFSF12 | TNF superfamily member 12 [Source:HGNC Symbol;Acc:HGNC:11927] | 3649 | 0.144 | -0.0258 | No |
| 12 | EPOR | erythropoietin receptor [Source:HGNC Symbol;Acc:HGNC:3416] | 3751 | 0.141 | -0.0240 | No |
| 13 | EGF | epidermal growth factor [Source:HGNC Symbol;Acc:HGNC:3229] | 3795 | 0.140 | -0.0208 | No |
| 14 | TNFRSF14 | TNF receptor superfamily member 14 [Source:HGNC Symbol;Acc:HGNC:11912] | 4359 | 0.125 | -0.0313 | No |
| 15 | CCR3 | C-C motif chemokine receptor 3 [Source:HGNC Symbol;Acc:HGNC:1604] | 4418 | 0.123 | -0.0290 | No |
| 16 | IL10RB | interleukin 10 receptor subunit beta [Source:HGNC Symbol;Acc:HGNC:5965] | 4583 | 0.120 | -0.0294 | No |
| 17 | MET | "MET proto-oncogene, receptor tyrosine kinase [Source:HGNC Symbol;Acc:HGNC:7029]" | 4748 | 0.116 | -0.0300 | No |
| 18 | IL25 | interleukin 25 [Source:HGNC Symbol;Acc:HGNC:13765] | 5027 | 0.111 | -0.0337 | No |
| 19 | TNFRSF1A | TNF receptor superfamily member 1A [Source:HGNC Symbol;Acc:HGNC:11916] | 5380 | 0.104 | -0.0395 | No |
| 20 | PDGFC | platelet derived growth factor C [Source:HGNC Symbol;Acc:HGNC:8801] | 5463 | 0.103 | -0.0384 | No |
| 21 | TNFRSF18 | TNF receptor superfamily member 18 [Source:HGNC Symbol;Acc:HGNC:11914] | 5689 | 0.099 | -0.0411 | No |
| 22 | IFNA5 | interferon alpha 5 [Source:HGNC Symbol;Acc:HGNC:5426] | 6009 | 0.093 | -0.0463 | No |
| 23 | EPO | erythropoietin [Source:HGNC Symbol;Acc:HGNC:3415] | 6062 | 0.093 | -0.0448 | No |
| 24 | IFNL3 | interferon lambda 3 [Source:HGNC Symbol;Acc:HGNC:18365] | 6168 | 0.091 | -0.0446 | No |
| 25 | LTBR | lymphotoxin beta receptor [Source:HGNC Symbol;Acc:HGNC:6718] | 6244 | 0.090 | -0.0438 | No |
| 26 | CXCL16 | C-X-C motif chemokine ligand 16 [Source:HGNC Symbol;Acc:HGNC:16642] | 6347 | 0.088 | -0.0436 | No |
| 27 | IL20RB | interleukin 20 receptor subunit beta [Source:HGNC Symbol;Acc:HGNC:6004] | 6391 | 0.087 | -0.0420 | No |
| 28 | TPO | thyroid peroxidase [Source:HGNC Symbol;Acc:HGNC:12015] | 6966 | 0.080 | -0.0542 | No |
| 29 | IL4 | interleukin 4 [Source:HGNC Symbol;Acc:HGNC:6014] | 7378 | 0.075 | -0.0624 | No |
| 30 | AMHR2 | anti-Mullerian hormone receptor type 2 [Source:HGNC Symbol;Acc:HGNC:465] | 7688 | 0.071 | -0.0681 | No |
| 31 | IL3 | interleukin 3 [Source:HGNC Symbol;Acc:HGNC:6011] | 8074 | 0.067 | -0.0759 | No |
| 32 | VEGFA | vascular endothelial growth factor A [Source:HGNC Symbol;Acc:HGNC:12680] | 8089 | 0.067 | -0.0742 | No |
| 33 | IL6R | interleukin 6 receptor [Source:HGNC Symbol;Acc:HGNC:6019] | 8091 | 0.067 | -0.0722 | No |
| 34 | IFNL2 | interferon lambda 2 [Source:HGNC Symbol;Acc:HGNC:18364] | 8127 | 0.066 | -0.0710 | No |
| 35 | TNFSF13 | TNF superfamily member 13 [Source:HGNC Symbol;Acc:HGNC:11928] | 8318 | 0.064 | -0.0739 | No |
| 36 | CCL28 | C-C motif chemokine ligand 28 [Source:HGNC Symbol;Acc:HGNC:17700] | 8536 | 0.062 | -0.0775 | No |
| 37 | IFNA16 | interferon alpha 16 [Source:HGNC Symbol;Acc:HGNC:5421] | 8538 | 0.062 | -0.0756 | No |
| 38 | IFNA7 | interferon alpha 7 [Source:HGNC Symbol;Acc:HGNC:5428] | 9153 | 0.056 | -0.0896 | No |
| 39 | IFNB1 | interferon beta 1 [Source:HGNC Symbol;Acc:HGNC:5434] | 9334 | 0.054 | -0.0925 | No |
| 40 | TNFRSF4 | TNF receptor superfamily member 4 [Source:HGNC Symbol;Acc:HGNC:11918] | 9567 | 0.052 | -0.0968 | No |
| 41 | EGFR | epidermal growth factor receptor [Source:HGNC Symbol;Acc:HGNC:3236] | 9728 | 0.051 | -0.0993 | No |
| 42 | CSF2 | colony stimulating factor 2 [Source:HGNC Symbol;Acc:HGNC:2434] | 9888 | 0.050 | -0.1018 | No |
| 43 | AMH | anti-Mullerian hormone [Source:HGNC Symbol;Acc:HGNC:464] | 10516 | 0.045 | -0.1165 | No |
| 44 | IFNL1 | interferon lambda 1 [Source:HGNC Symbol;Acc:HGNC:18363] | 10754 | 0.043 | -0.1212 | No |
| 45 | MPL | "MPL proto-oncogene, thrombopoietin receptor [Source:HGNC Symbol;Acc:HGNC:7217]" | 10820 | 0.042 | -0.1216 | No |
| 46 | PPBP | pro-platelet basic protein [Source:HGNC Symbol;Acc:HGNC:9240] | 10823 | 0.042 | -0.1203 | No |
| 47 | IFNLR1 | interferon lambda receptor 1 [Source:HGNC Symbol;Acc:HGNC:18584] | 11373 | 0.038 | -0.1332 | No |
| 48 | INHBC | inhibin subunit beta C [Source:HGNC Symbol;Acc:HGNC:6068] | 11655 | 0.036 | -0.1392 | No |
| 49 | IFNA13 | interferon alpha 13 [Source:HGNC Symbol;Acc:HGNC:5419] | 11857 | 0.034 | -0.1433 | No |
| 50 | CRLF2 | cytokine receptor like factor 2 [Source:HGNC Symbol;Acc:HGNC:14281] | 12291 | 0.031 | -0.1534 | No |
| 51 | PRLR | prolactin receptor [Source:HGNC Symbol;Acc:HGNC:9446] | 12366 | 0.030 | -0.1544 | No |
| 52 | IL19 | interleukin 19 [Source:HGNC Symbol;Acc:HGNC:5990] | 12375 | 0.030 | -0.1536 | No |
| 53 | CX3CL1 | C-X3-C motif chemokine ligand 1 [Source:HGNC Symbol;Acc:HGNC:10647] | 12417 | 0.030 | -0.1538 | No |
| 54 | IL18 | interleukin 18 [Source:HGNC Symbol;Acc:HGNC:5986] | 12472 | 0.030 | -0.1542 | No |
| 55 | TNFSF15 | TNF superfamily member 15 [Source:HGNC Symbol;Acc:HGNC:11931] | 12776 | 0.027 | -0.1611 | No |
| 56 | TNFSF10 | TNF superfamily member 10 [Source:HGNC Symbol;Acc:HGNC:11925] | 13004 | 0.026 | -0.1661 | No |
| 57 | IFNA8 | interferon alpha 8 [Source:HGNC Symbol;Acc:HGNC:5429] | 13190 | 0.024 | -0.1701 | No |
| 58 | IL17B | interleukin 17B [Source:HGNC Symbol;Acc:HGNC:5982] | 13347 | 0.023 | -0.1734 | No |
| 59 | FLT3LG | fms related receptor tyrosine kinase 3 ligand [Source:HGNC Symbol;Acc:HGNC:3766] | 13668 | 0.021 | -0.1809 | No |
| 60 | PF4 | platelet factor 4 [Source:HGNC Symbol;Acc:HGNC:8861] | 13825 | 0.020 | -0.1843 | No |
| 61 | IL22RA1 | interleukin 22 receptor subunit alpha 1 [Source:HGNC Symbol;Acc:HGNC:13700] | 14064 | 0.019 | -0.1898 | No |
| 62 | INHBB | inhibin subunit beta B [Source:HGNC Symbol;Acc:HGNC:6067] | 14454 | 0.016 | -0.1992 | No |
| 63 | CXCL14 | C-X-C motif chemokine ligand 14 [Source:HGNC Symbol;Acc:HGNC:10640] | 14753 | 0.015 | -0.2064 | No |
| 64 | IL12B | interleukin 12B [Source:HGNC Symbol;Acc:HGNC:5970] | 15071 | 0.013 | -0.2141 | No |
| 65 | CNTFR | ciliary neurotrophic factor receptor [Source:HGNC Symbol;Acc:HGNC:2170] | 15115 | 0.012 | -0.2148 | No |
| 66 | BMPR1B | bone morphogenetic protein receptor type 1B [Source:HGNC Symbol;Acc:HGNC:1077] | 15125 | 0.012 | -0.2147 | No |
| 67 | IL9 | interleukin 9 [Source:HGNC Symbol;Acc:HGNC:6029] | 15668 | 0.009 | -0.2283 | No |
| 68 | IFNAR1 | interferon alpha and beta receptor subunit 1 [Source:HGNC Symbol;Acc:HGNC:5432] | 15859 | 0.008 | -0.2329 | No |
| 69 | IFNA4 | interferon alpha 4 [Source:HGNC Symbol;Acc:HGNC:5425] | 16049 | 0.007 | -0.2375 | No |
| 70 | IFNE | interferon epsilon [Source:HGNC Symbol;Acc:HGNC:18163] | 16104 | 0.006 | -0.2387 | No |
| 71 | OSMR | oncostatin M receptor [Source:HGNC Symbol;Acc:HGNC:8507] | 16481 | 0.004 | -0.2482 | No |
| 72 | TNFRSF10C | TNF receptor superfamily member 10c [Source:HGNC Symbol;Acc:HGNC:11906] | 16656 | 0.003 | -0.2526 | No |
| 73 | IFNA21 | interferon alpha 21 [Source:HGNC Symbol;Acc:HGNC:5424] | 16891 | 0.002 | -0.2585 | No |
| 74 | CD40 | CD40 molecule [Source:HGNC Symbol;Acc:HGNC:11919] | 17012 | 0.001 | -0.2615 | No |
| 75 | XCL1 | X-C motif chemokine ligand 1 [Source:HGNC Symbol;Acc:HGNC:10645] | 17667 | -0.003 | -0.2782 | No |
| 76 | CCL27 | C-C motif chemokine ligand 27 [Source:HGNC Symbol;Acc:HGNC:10626] | 18208 | -0.006 | -0.2918 | No |
| 77 | IFNA17 | interferon alpha 17 [Source:HGNC Symbol;Acc:HGNC:5422] | 18384 | -0.007 | -0.2961 | No |
| 78 | CX3CR1 | C-X3-C motif chemokine receptor 1 [Source:HGNC Symbol;Acc:HGNC:2558] | 18890 | -0.010 | -0.3087 | No |
| 79 | VEGFD | vascular endothelial growth factor D [Source:HGNC Symbol;Acc:HGNC:3708] | 19101 | -0.011 | -0.3137 | No |
| 80 | IL17RB | interleukin 17 receptor B [Source:HGNC Symbol;Acc:HGNC:18015] | 19183 | -0.012 | -0.3154 | No |
| 81 | ACVR1 | activin A receptor type 1 [Source:HGNC Symbol;Acc:HGNC:171] | 19508 | -0.014 | -0.3233 | No |
| 82 | IFNA14 | interferon alpha 14 [Source:HGNC Symbol;Acc:HGNC:5420] | 19637 | -0.014 | -0.3261 | No |
| 83 | IFNGR1 | interferon gamma receptor 1 [Source:HGNC Symbol;Acc:HGNC:5439] | 19762 | -0.015 | -0.3288 | No |
| 84 | IL13 | interleukin 13 [Source:HGNC Symbol;Acc:HGNC:5973] | 20238 | -0.018 | -0.3404 | No |
| 85 | CCL22 | C-C motif chemokine ligand 22 [Source:HGNC Symbol;Acc:HGNC:10621] | 20341 | -0.019 | -0.3424 | No |
| 86 | TNFRSF25 | TNF receptor superfamily member 25 [Source:HGNC Symbol;Acc:HGNC:11910] | 20439 | -0.019 | -0.3443 | No |
| 87 | IFNA1 | interferon alpha 1 [Source:HGNC Symbol;Acc:HGNC:5417] | 20479 | -0.020 | -0.3447 | No |
| 88 | EDA | ectodysplasin A [Source:HGNC Symbol;Acc:HGNC:3157] | 20772 | -0.021 | -0.3515 | No |
| 89 | IL2 | interleukin 2 [Source:HGNC Symbol;Acc:HGNC:6001] | 20777 | -0.021 | -0.3510 | No |
| 90 | IL4R | interleukin 4 receptor [Source:HGNC Symbol;Acc:HGNC:6015] | 21312 | -0.025 | -0.3639 | No |
| 91 | IFNA6 | interferon alpha 6 [Source:HGNC Symbol;Acc:HGNC:5427] | 21419 | -0.025 | -0.3658 | No |
| 92 | IL20 | interleukin 20 [Source:HGNC Symbol;Acc:HGNC:6002] | 22182 | -0.030 | -0.3844 | No |
| 93 | IL12A | interleukin 12A [Source:HGNC Symbol;Acc:HGNC:5969] | 22276 | -0.030 | -0.3858 | No |
| 94 | TGFB1 | transforming growth factor beta 1 [Source:HGNC Symbol;Acc:HGNC:11766] | 22340 | -0.031 | -0.3864 | No |
| 95 | CXCL2 | C-X-C motif chemokine ligand 2 [Source:HGNC Symbol;Acc:HGNC:4603] | 22636 | -0.033 | -0.3930 | No |
| 96 | CCL13 | C-C motif chemokine ligand 13 [Source:HGNC Symbol;Acc:HGNC:10611] | 22728 | -0.033 | -0.3943 | No |
| 97 | IL23A | interleukin 23 subunit alpha [Source:HGNC Symbol;Acc:HGNC:15488] | 22882 | -0.034 | -0.3971 | No |
| 98 | IFNW1 | interferon omega 1 [Source:HGNC Symbol;Acc:HGNC:5448] | 23048 | -0.035 | -0.4002 | No |
| 99 | BMP2 | bone morphogenetic protein 2 [Source:HGNC Symbol;Acc:HGNC:1069] | 23093 | -0.036 | -0.4002 | No |
| 100 | CCL7 | C-C motif chemokine ligand 7 [Source:HGNC Symbol;Acc:HGNC:10634] | 23180 | -0.037 | -0.4013 | No |
| 101 | IL11 | interleukin 11 [Source:HGNC Symbol;Acc:HGNC:5966] | 23228 | -0.037 | -0.4014 | No |
| 102 | TNFRSF11A | TNF receptor superfamily member 11a [Source:HGNC Symbol;Acc:HGNC:11908] | 23245 | -0.037 | -0.4006 | No |
| 103 | IFNA2 | interferon alpha 2 [Source:HGNC Symbol;Acc:HGNC:5423] | 23890 | -0.041 | -0.4158 | No |
| 104 | FAS | Fas cell surface death receptor [Source:HGNC Symbol;Acc:HGNC:11920] | 23994 | -0.042 | -0.4172 | No |
| 105 | CCL1 | C-C motif chemokine ligand 1 [Source:HGNC Symbol;Acc:HGNC:10609] | 24386 | -0.044 | -0.4258 | No |
| 106 | CCL11 | C-C motif chemokine ligand 11 [Source:HGNC Symbol;Acc:HGNC:10610] | 24418 | -0.045 | -0.4252 | No |
| 107 | CD70 | CD70 molecule [Source:HGNC Symbol;Acc:HGNC:11937] | 24706 | -0.047 | -0.4311 | No |
| 108 | CCR10 | C-C motif chemokine receptor 10 [Source:HGNC Symbol;Acc:HGNC:4474] | 24820 | -0.047 | -0.4325 | No |
| 109 | IL5 | interleukin 5 [Source:HGNC Symbol;Acc:HGNC:6016] | 25260 | -0.050 | -0.4422 | No |
| 110 | BMP7 | bone morphogenetic protein 7 [Source:HGNC Symbol;Acc:HGNC:1074] | 25274 | -0.051 | -0.4409 | No |
| 111 | EDA2R | ectodysplasin A2 receptor [Source:HGNC Symbol;Acc:HGNC:17756] | 25294 | -0.051 | -0.4398 | No |
| 112 | CSF2RA | colony stimulating factor 2 receptor subunit alpha [Source:HGNC Symbol;Acc:HGNC:2435] | 25630 | -0.053 | -0.4468 | No |
| 113 | PPBPP1 | pro-platelet basic protein pseudogene 1 [Source:HGNC Symbol;Acc:HGNC:9241] | 25765 | -0.054 | -0.4485 | No |
| 114 | VEGFC | vascular endothelial growth factor C [Source:HGNC Symbol;Acc:HGNC:12682] | 25822 | -0.055 | -0.4483 | No |
| 115 | TSLP | thymic stromal lymphopoietin [Source:HGNC Symbol;Acc:HGNC:30743] | 25864 | -0.055 | -0.4476 | No |
| 116 | TGFB2 | transforming growth factor beta 2 [Source:HGNC Symbol;Acc:HGNC:11768] | 26001 | -0.056 | -0.4493 | No |
| 117 | GH1 | growth hormone 1 [Source:HGNC Symbol;Acc:HGNC:4261] | 26168 | -0.057 | -0.4518 | No |
| 118 | IFNA10 | interferon alpha 10 [Source:HGNC Symbol;Acc:HGNC:5418] | 26311 | -0.058 | -0.4537 | No |
| 119 | CCL26 | C-C motif chemokine ligand 26 [Source:HGNC Symbol;Acc:HGNC:10625] | 26486 | -0.059 | -0.4563 | No |
| 120 | LTB | lymphotoxin beta [Source:HGNC Symbol;Acc:HGNC:6711] | 26670 | -0.061 | -0.4591 | No |
| 121 | PF4V1 | platelet factor 4 variant 1 [Source:HGNC Symbol;Acc:HGNC:8862] | 26985 | -0.063 | -0.4651 | No |
| 122 | IL15 | interleukin 15 [Source:HGNC Symbol;Acc:HGNC:5977] | 27093 | -0.064 | -0.4659 | No |
| 123 | IFNAR2 | interferon alpha and beta receptor subunit 2 [Source:HGNC Symbol;Acc:HGNC:5433] | 27247 | -0.065 | -0.4678 | No |
| 124 | IL26 | interleukin 26 [Source:HGNC Symbol;Acc:HGNC:17119] | 27301 | -0.066 | -0.4671 | No |
| 125 | LEP | leptin [Source:HGNC Symbol;Acc:HGNC:6553] | 27432 | -0.067 | -0.4683 | No |
| 126 | XCL2 | X-C motif chemokine ligand 2 [Source:HGNC Symbol;Acc:HGNC:10646] | 27801 | -0.069 | -0.4756 | No |
| 127 | KDR | kinase insert domain receptor [Source:HGNC Symbol;Acc:HGNC:6307] | 27955 | -0.071 | -0.4773 | No |
| 128 | TNFSF18 | TNF superfamily member 18 [Source:HGNC Symbol;Acc:HGNC:11932] | 28219 | -0.073 | -0.4818 | No |
| 129 | TNFSF14 | TNF superfamily member 14 [Source:HGNC Symbol;Acc:HGNC:11930] | 28284 | -0.074 | -0.4811 | No |
| 130 | CCL19 | C-C motif chemokine ligand 19 [Source:HGNC Symbol;Acc:HGNC:10617] | 28473 | -0.075 | -0.4836 | No |
| 131 | IL22RA2 | interleukin 22 receptor subunit alpha 2 [Source:HGNC Symbol;Acc:HGNC:14901] | 28551 | -0.076 | -0.4832 | No |
| 132 | CXCL1 | C-X-C motif chemokine ligand 1 [Source:HGNC Symbol;Acc:HGNC:4602] | 28932 | -0.079 | -0.4905 | No |
| 133 | CCL15 | C-C motif chemokine ligand 15 [Source:HGNC Symbol;Acc:HGNC:10613] | 28986 | -0.080 | -0.4893 | No |
| 134 | BMPR1A | bone morphogenetic protein receptor type 1A [Source:HGNC Symbol;Acc:HGNC:1076] | 29382 | -0.083 | -0.4969 | No |
| 135 | CSF1 | colony stimulating factor 1 [Source:HGNC Symbol;Acc:HGNC:2432] | 29384 | -0.083 | -0.4943 | No |
| 136 | PRL | prolactin [Source:HGNC Symbol;Acc:HGNC:9445] | 29427 | -0.084 | -0.4928 | No |
| 137 | IL22 | interleukin 22 [Source:HGNC Symbol;Acc:HGNC:14900] | 29805 | -0.088 | -0.4997 | No |
| 138 | EDAR | ectodysplasin A receptor [Source:HGNC Symbol;Acc:HGNC:2895] | 29965 | -0.089 | -0.5010 | No |
| 139 | TNFRSF10B | TNF receptor superfamily member 10b [Source:HGNC Symbol;Acc:HGNC:11905] | 30305 | -0.093 | -0.5068 | No |
| 140 | ACVR2A | activin A receptor type 2A [Source:HGNC Symbol;Acc:HGNC:173] | 30423 | -0.094 | -0.5068 | No |
| 141 | GH2 | growth hormone 2 [Source:HGNC Symbol;Acc:HGNC:4262] | 30618 | -0.096 | -0.5088 | No |
| 142 | ACVR2B | activin A receptor type 2B [Source:HGNC Symbol;Acc:HGNC:174] | 30713 | -0.097 | -0.5082 | No |
| 143 | TNFRSF10D | TNF receptor superfamily member 10d [Source:HGNC Symbol;Acc:HGNC:11907] | 30869 | -0.099 | -0.5091 | No |
| 144 | CXCL3 | C-X-C motif chemokine ligand 3 [Source:HGNC Symbol;Acc:HGNC:4604] | 31096 | -0.102 | -0.5117 | Yes |
| 145 | LIFR | LIF receptor subunit alpha [Source:HGNC Symbol;Acc:HGNC:6597] | 31193 | -0.103 | -0.5109 | Yes |
| 146 | CCL14 | C-C motif chemokine ligand 14 [Source:HGNC Symbol;Acc:HGNC:10612] | 31273 | -0.104 | -0.5097 | Yes |
| 147 | CXCL11 | C-X-C motif chemokine ligand 11 [Source:HGNC Symbol;Acc:HGNC:10638] | 31326 | -0.104 | -0.5078 | Yes |
| 148 | KIT | "KIT proto-oncogene, receptor tyrosine kinase [Source:HGNC Symbol;Acc:HGNC:6342]" | 31394 | -0.105 | -0.5063 | Yes |
| 149 | PDGFB | platelet derived growth factor subunit B [Source:HGNC Symbol;Acc:HGNC:8800] | 31529 | -0.107 | -0.5064 | Yes |
| 150 | CCL23 | C-C motif chemokine ligand 23 [Source:HGNC Symbol;Acc:HGNC:10622] | 31536 | -0.107 | -0.5032 | Yes |
| 151 | NGFR | nerve growth factor receptor [Source:HGNC Symbol;Acc:HGNC:7809] | 31563 | -0.107 | -0.5005 | Yes |
| 152 | CCL3L3 | C-C motif chemokine ligand 3 like 3 [Source:HGNC Symbol;Acc:HGNC:30554] | 31590 | -0.108 | -0.4978 | Yes |
| 153 | CCL16 | C-C motif chemokine ligand 16 [Source:HGNC Symbol;Acc:HGNC:10614] | 31612 | -0.108 | -0.4950 | Yes |
| 154 | IL20RA | interleukin 20 receptor subunit alpha [Source:HGNC Symbol;Acc:HGNC:6003] | 31685 | -0.109 | -0.4935 | Yes |
| 155 | CXCR3 | C-X-C motif chemokine receptor 3 [Source:HGNC Symbol;Acc:HGNC:4540] | 31733 | -0.110 | -0.4913 | Yes |
| 156 | CXCL8 | C-X-C motif chemokine ligand 8 [Source:HGNC Symbol;Acc:HGNC:6025] | 31746 | -0.110 | -0.4882 | Yes |
| 157 | CCL2 | C-C motif chemokine ligand 2 [Source:HGNC Symbol;Acc:HGNC:10618] | 31950 | -0.112 | -0.4899 | Yes |
| 158 | CCL21 | C-C motif chemokine ligand 21 [Source:HGNC Symbol;Acc:HGNC:10620] | 32114 | -0.115 | -0.4905 | Yes |
| 159 | IFNK | interferon kappa [Source:HGNC Symbol;Acc:HGNC:21714] | 32352 | -0.118 | -0.4929 | Yes |
| 160 | OSM | oncostatin M [Source:HGNC Symbol;Acc:HGNC:8506] | 32409 | -0.118 | -0.4906 | Yes |
| 161 | IL3RA | interleukin 3 receptor subunit alpha [Source:HGNC Symbol;Acc:HGNC:6012] | 32493 | -0.120 | -0.4890 | Yes |
| 162 | CCL4L2 | C-C motif chemokine ligand 4 like 2 [Source:HGNC Symbol;Acc:HGNC:24066] | 33115 | -0.130 | -0.5009 | Yes |
| 163 | CCL5 | C-C motif chemokine ligand 5 [Source:HGNC Symbol;Acc:HGNC:10632] | 33180 | -0.131 | -0.4984 | Yes |
| 164 | TNFRSF13B | TNF receptor superfamily member 13B [Source:HGNC Symbol;Acc:HGNC:18153] | 33212 | -0.131 | -0.4952 | Yes |
| 165 | TNFRSF19 | TNF receptor superfamily member 19 [Source:HGNC Symbol;Acc:HGNC:11915] | 33280 | -0.133 | -0.4927 | Yes |
| 166 | KITLG | KIT ligand [Source:HGNC Symbol;Acc:HGNC:6343] | 33298 | -0.133 | -0.4890 | Yes |
| 167 | TNFRSF21 | TNF receptor superfamily member 21 [Source:HGNC Symbol;Acc:HGNC:13469] | 33316 | -0.133 | -0.4853 | Yes |
| 168 | CCL20 | C-C motif chemokine ligand 20 [Source:HGNC Symbol;Acc:HGNC:10619] | 33317 | -0.133 | -0.4812 | Yes |
| 169 | CXCL6 | C-X-C motif chemokine ligand 6 [Source:HGNC Symbol;Acc:HGNC:10643] | 33379 | -0.134 | -0.4786 | Yes |
| 170 | CCR7 | C-C motif chemokine receptor 7 [Source:HGNC Symbol;Acc:HGNC:1608] | 33392 | -0.135 | -0.4747 | Yes |
| 171 | IL5RA | interleukin 5 receptor subunit alpha [Source:HGNC Symbol;Acc:HGNC:6017] | 33434 | -0.135 | -0.4715 | Yes |
| 172 | IL1RAP | interleukin 1 receptor accessory protein [Source:HGNC Symbol;Acc:HGNC:5995] | 33440 | -0.135 | -0.4675 | Yes |
| 173 | CSF3 | colony stimulating factor 3 [Source:HGNC Symbol;Acc:HGNC:2438] | 33514 | -0.137 | -0.4651 | Yes |
| 174 | CXCR5 | C-X-C motif chemokine receptor 5 [Source:HGNC Symbol;Acc:HGNC:1060] | 33556 | -0.137 | -0.4619 | Yes |
| 175 | IL13RA1 | interleukin 13 receptor subunit alpha 1 [Source:HGNC Symbol;Acc:HGNC:5974] | 33634 | -0.139 | -0.4595 | Yes |
| 176 | IL7 | interleukin 7 [Source:HGNC Symbol;Acc:HGNC:6023] | 33655 | -0.139 | -0.4557 | Yes |
| 177 | TNF | tumor necrosis factor [Source:HGNC Symbol;Acc:HGNC:11892] | 33732 | -0.140 | -0.4533 | Yes |
| 178 | LIF | LIF interleukin 6 family cytokine [Source:HGNC Symbol;Acc:HGNC:6596] | 33750 | -0.141 | -0.4493 | Yes |
| 179 | GHR | growth hormone receptor [Source:HGNC Symbol;Acc:HGNC:4263] | 34005 | -0.145 | -0.4513 | Yes |
| 180 | IL1A | interleukin 1 alpha [Source:HGNC Symbol;Acc:HGNC:5991] | 34080 | -0.147 | -0.4486 | Yes |
| 181 | CXCL13 | C-X-C motif chemokine ligand 13 [Source:HGNC Symbol;Acc:HGNC:10639] | 34100 | -0.147 | -0.4445 | Yes |
| 182 | IL6ST | interleukin 6 signal transducer [Source:HGNC Symbol;Acc:HGNC:6021] | 34111 | -0.148 | -0.4402 | Yes |
| 183 | IL17A | interleukin 17A [Source:HGNC Symbol;Acc:HGNC:5981] | 34115 | -0.148 | -0.4357 | Yes |
| 184 | FLT4 | fms related receptor tyrosine kinase 4 [Source:HGNC Symbol;Acc:HGNC:3767] | 34137 | -0.148 | -0.4316 | Yes |
| 185 | CCL8 | C-C motif chemokine ligand 8 [Source:HGNC Symbol;Acc:HGNC:10635] | 34171 | -0.149 | -0.4278 | Yes |
| 186 | ACVR1B | activin A receptor type 1B [Source:HGNC Symbol;Acc:HGNC:172] | 34193 | -0.149 | -0.4237 | Yes |
| 187 | IL24 | interleukin 24 [Source:HGNC Symbol;Acc:HGNC:11346] | 34254 | -0.150 | -0.4206 | Yes |
| 188 | IL9R | interleukin 9 receptor [Source:HGNC Symbol;Acc:HGNC:6030] | 34282 | -0.151 | -0.4166 | Yes |
| 189 | CCR9 | C-C motif chemokine receptor 9 [Source:HGNC Symbol;Acc:HGNC:1610] | 34438 | -0.154 | -0.4158 | Yes |
| 190 | LEPR | leptin receptor [Source:HGNC Symbol;Acc:HGNC:6554] | 34476 | -0.155 | -0.4119 | Yes |
| 191 | IL15RA | interleukin 15 receptor subunit alpha [Source:HGNC Symbol;Acc:HGNC:5978] | 34548 | -0.156 | -0.4089 | Yes |
| 192 | TNFRSF11B | TNF receptor superfamily member 11b [Source:HGNC Symbol;Acc:HGNC:11909] | 34572 | -0.156 | -0.4046 | Yes |
| 193 | CCL3 | C-C motif chemokine ligand 3 [Source:HGNC Symbol;Acc:HGNC:10627] | 34673 | -0.159 | -0.4022 | Yes |
| 194 | CXCL5 | C-X-C motif chemokine ligand 5 [Source:HGNC Symbol;Acc:HGNC:10642] | 34783 | -0.162 | -0.4000 | Yes |
| 195 | TNFRSF13C | TNF receptor superfamily member 13C [Source:HGNC Symbol;Acc:HGNC:17755] | 34842 | -0.163 | -0.3964 | Yes |
| 196 | CCL25 | C-C motif chemokine ligand 25 [Source:HGNC Symbol;Acc:HGNC:10624] | 35008 | -0.166 | -0.3954 | Yes |
| 197 | CXCL10 | C-X-C motif chemokine ligand 10 [Source:HGNC Symbol;Acc:HGNC:10637] | 35086 | -0.168 | -0.3922 | Yes |
| 198 | CXCR1 | C-X-C motif chemokine receptor 1 [Source:HGNC Symbol;Acc:HGNC:6026] | 35184 | -0.171 | -0.3893 | Yes |
| 199 | CD40LG | CD40 ligand [Source:HGNC Symbol;Acc:HGNC:11935] | 35185 | -0.171 | -0.3840 | Yes |
| 200 | ACVRL1 | activin A receptor like type 1 [Source:HGNC Symbol;Acc:HGNC:175] | 35429 | -0.177 | -0.3847 | Yes |
| 201 | TNFRSF9 | TNF receptor superfamily member 9 [Source:HGNC Symbol;Acc:HGNC:11924] | 35601 | -0.182 | -0.3834 | Yes |
| 202 | LTA | lymphotoxin alpha [Source:HGNC Symbol;Acc:HGNC:6709] | 35644 | -0.183 | -0.3788 | Yes |
| 203 | IFNG | interferon gamma [Source:HGNC Symbol;Acc:HGNC:5438] | 35652 | -0.183 | -0.3733 | Yes |
| 204 | TGFB3 | transforming growth factor beta 3 [Source:HGNC Symbol;Acc:HGNC:11769] | 35787 | -0.187 | -0.3709 | Yes |
| 205 | CXCL12 | C-X-C motif chemokine ligand 12 [Source:HGNC Symbol;Acc:HGNC:10672] | 35899 | -0.191 | -0.3678 | Yes |
| 206 | TNFRSF1B | TNF receptor superfamily member 1B [Source:HGNC Symbol;Acc:HGNC:11917] | 36134 | -0.198 | -0.3676 | Yes |
| 207 | CD27 | CD27 molecule [Source:HGNC Symbol;Acc:HGNC:11922] | 36138 | -0.198 | -0.3615 | Yes |
| 208 | TGFBR2 | transforming growth factor beta receptor 2 [Source:HGNC Symbol;Acc:HGNC:11773] | 36159 | -0.199 | -0.3558 | Yes |
| 209 | IL17RA | interleukin 17 receptor A [Source:HGNC Symbol;Acc:HGNC:5985] | 36313 | -0.205 | -0.3534 | Yes |
| 210 | CSF3R | colony stimulating factor 3 receptor [Source:HGNC Symbol;Acc:HGNC:2439] | 36344 | -0.206 | -0.3477 | Yes |
| 211 | FASLG | Fas ligand [Source:HGNC Symbol;Acc:HGNC:11936] | 36345 | -0.206 | -0.3413 | Yes |
| 212 | RELT | RELT TNF receptor [Source:HGNC Symbol;Acc:HGNC:13764] | 36379 | -0.207 | -0.3357 | Yes |
| 213 | IL6 | interleukin 6 [Source:HGNC Symbol;Acc:HGNC:6018] | 36396 | -0.208 | -0.3296 | Yes |
| 214 | PDGFRB | platelet derived growth factor receptor beta [Source:HGNC Symbol;Acc:HGNC:8804] | 36398 | -0.208 | -0.3232 | Yes |
| 215 | TNFSF4 | TNF superfamily member 4 [Source:HGNC Symbol;Acc:HGNC:11934] | 36521 | -0.212 | -0.3197 | Yes |
| 216 | CXCR4 | C-X-C motif chemokine receptor 4 [Source:HGNC Symbol;Acc:HGNC:2561] | 36579 | -0.215 | -0.3145 | Yes |
| 217 | TNFSF13B | TNF superfamily member 13b [Source:HGNC Symbol;Acc:HGNC:11929] | 36605 | -0.215 | -0.3084 | Yes |
| 218 | TNFRSF8 | TNF receptor superfamily member 8 [Source:HGNC Symbol;Acc:HGNC:11923] | 36616 | -0.216 | -0.3020 | Yes |
| 219 | PLEKHO2 | pleckstrin homology domain containing O2 [Source:HGNC Symbol;Acc:HGNC:30026] | 36622 | -0.216 | -0.2954 | Yes |
| 220 | INHBE | inhibin subunit beta E [Source:HGNC Symbol;Acc:HGNC:24029] | 36632 | -0.217 | -0.2888 | Yes |
| 221 | CCR6 | C-C motif chemokine receptor 6 [Source:HGNC Symbol;Acc:HGNC:1607] | 36663 | -0.218 | -0.2828 | Yes |
| 222 | TGFBR1 | transforming growth factor beta receptor 1 [Source:HGNC Symbol;Acc:HGNC:11772] | 36724 | -0.221 | -0.2775 | Yes |
| 223 | TNFRSF17 | TNF receptor superfamily member 17 [Source:HGNC Symbol;Acc:HGNC:11913] | 36751 | -0.222 | -0.2712 | Yes |
| 224 | CCL18 | C-C motif chemokine ligand 18 [Source:HGNC Symbol;Acc:HGNC:10616] | 36793 | -0.225 | -0.2653 | Yes |
| 225 | IL1R1 | interleukin 1 receptor type 1 [Source:HGNC Symbol;Acc:HGNC:5993] | 36824 | -0.226 | -0.2590 | Yes |
| 226 | CXCR2 | C-X-C motif chemokine receptor 2 [Source:HGNC Symbol;Acc:HGNC:6027] | 36832 | -0.227 | -0.2521 | Yes |
| 227 | TNFRSF10A | TNF receptor superfamily member 10a [Source:HGNC Symbol;Acc:HGNC:11904] | 36857 | -0.228 | -0.2457 | Yes |
| 228 | CXCL9 | C-X-C motif chemokine ligand 9 [Source:HGNC Symbol;Acc:HGNC:7098] | 36887 | -0.230 | -0.2393 | Yes |
| 229 | IL21 | interleukin 21 [Source:HGNC Symbol;Acc:HGNC:6005] | 36899 | -0.230 | -0.2324 | Yes |
| 230 | CCR8 | C-C motif chemokine receptor 8 [Source:HGNC Symbol;Acc:HGNC:1609] | 36930 | -0.232 | -0.2259 | Yes |
| 231 | CCL4 | C-C motif chemokine ligand 4 [Source:HGNC Symbol;Acc:HGNC:10630] | 36977 | -0.234 | -0.2198 | Yes |
| 232 | IL18RAP | interleukin 18 receptor accessory protein [Source:HGNC Symbol;Acc:HGNC:5989] | 36985 | -0.235 | -0.2127 | Yes |
| 233 | IL12RB2 | interleukin 12 receptor subunit beta 2 [Source:HGNC Symbol;Acc:HGNC:5972] | 37027 | -0.237 | -0.2063 | Yes |
| 234 | CSF1R | colony stimulating factor 1 receptor [Source:HGNC Symbol;Acc:HGNC:2433] | 37028 | -0.237 | -0.1989 | Yes |
| 235 | XCR1 | X-C motif chemokine receptor 1 [Source:HGNC Symbol;Acc:HGNC:1625] | 37135 | -0.244 | -0.1941 | Yes |
| 236 | IL2RG | interleukin 2 receptor subunit gamma [Source:HGNC Symbol;Acc:HGNC:6010] | 37182 | -0.247 | -0.1876 | Yes |
| 237 | CNTF | ciliary neurotrophic factor [Source:HGNC Symbol;Acc:HGNC:2169] | 37188 | -0.247 | -0.1800 | Yes |
| 238 | IL1B | interleukin 1 beta [Source:HGNC Symbol;Acc:HGNC:5992] | 37201 | -0.248 | -0.1726 | Yes |
| 239 | IL12RB1 | interleukin 12 receptor subunit beta 1 [Source:HGNC Symbol;Acc:HGNC:5971] | 37256 | -0.251 | -0.1662 | Yes |
| 240 | CCR2 | C-C motif chemokine receptor 2 [Source:HGNC Symbol;Acc:HGNC:1603] | 37267 | -0.252 | -0.1586 | Yes |
| 241 | IL10 | interleukin 10 [Source:HGNC Symbol;Acc:HGNC:5962] | 37315 | -0.254 | -0.1519 | Yes |
| 242 | INHBA | inhibin subunit beta A [Source:HGNC Symbol;Acc:HGNC:6066] | 37343 | -0.256 | -0.1446 | Yes |
| 243 | TNFSF11 | TNF superfamily member 11 [Source:HGNC Symbol;Acc:HGNC:11926] | 37359 | -0.257 | -0.1370 | Yes |
| 244 | IL21R | interleukin 21 receptor [Source:HGNC Symbol;Acc:HGNC:6006] | 37376 | -0.258 | -0.1294 | Yes |
| 245 | CCR4 | C-C motif chemokine receptor 4 [Source:HGNC Symbol;Acc:HGNC:1605] | 37467 | -0.264 | -0.1235 | Yes |
| 246 | CXCR6 | C-X-C motif chemokine receptor 6 [Source:HGNC Symbol;Acc:HGNC:16647] | 37475 | -0.264 | -0.1154 | Yes |
| 247 | IL23R | interleukin 23 receptor [Source:HGNC Symbol;Acc:HGNC:19100] | 37504 | -0.266 | -0.1079 | Yes |
| 248 | FLT3 | fms related receptor tyrosine kinase 3 [Source:HGNC Symbol;Acc:HGNC:3765] | 37510 | -0.267 | -0.0997 | Yes |
| 249 | PDGFRA | platelet derived growth factor receptor alpha [Source:HGNC Symbol;Acc:HGNC:8803] | 37662 | -0.280 | -0.0949 | Yes |
| 250 | IL2RB | interleukin 2 receptor subunit beta [Source:HGNC Symbol;Acc:HGNC:6009] | 37681 | -0.282 | -0.0866 | Yes |
| 251 | CCL24 | C-C motif chemokine ligand 24 [Source:HGNC Symbol;Acc:HGNC:10623] | 37740 | -0.286 | -0.0791 | Yes |
| 252 | TNFSF8 | TNF superfamily member 8 [Source:HGNC Symbol;Acc:HGNC:11938] | 37753 | -0.287 | -0.0705 | Yes |
| 253 | BMPR2 | bone morphogenetic protein receptor type 2 [Source:HGNC Symbol;Acc:HGNC:1078] | 37775 | -0.288 | -0.0621 | Yes |
| 254 | CCR1 | C-C motif chemokine receptor 1 [Source:HGNC Symbol;Acc:HGNC:1602] | 37830 | -0.294 | -0.0543 | Yes |
| 255 | HGF | hepatocyte growth factor [Source:HGNC Symbol;Acc:HGNC:4893] | 37908 | -0.304 | -0.0468 | Yes |
| 256 | IL7R | interleukin 7 receptor [Source:HGNC Symbol;Acc:HGNC:6024] | 37941 | -0.309 | -0.0380 | Yes |
| 257 | CCR5 | C-C motif chemokine receptor 5 [Source:HGNC Symbol;Acc:HGNC:1606] | 37948 | -0.309 | -0.0286 | Yes |
| 258 | IL1R2 | interleukin 1 receptor type 2 [Source:HGNC Symbol;Acc:HGNC:5994] | 37972 | -0.312 | -0.0194 | Yes |
| 259 | FLT1 | fms related receptor tyrosine kinase 1 [Source:HGNC Symbol;Acc:HGNC:3763] | 38056 | -0.325 | -0.0114 | Yes |
| 260 | IL10RA | interleukin 10 receptor subunit alpha [Source:HGNC Symbol;Acc:HGNC:5964] | 38059 | -0.325 | -0.0014 | Yes |
| 261 | IL2RA | interleukin 2 receptor subunit alpha [Source:HGNC Symbol;Acc:HGNC:6008] | 38105 | -0.335 | 0.0079 | Yes |
| 262 | CSF2RB | colony stimulating factor 2 receptor subunit beta [Source:HGNC Symbol;Acc:HGNC:2436] | 38193 | -0.360 | 0.0169 | Yes |
| 263 | IL18R1 | interleukin 18 receptor 1 [Source:HGNC Symbol;Acc:HGNC:5988] | 38231 | -0.376 | 0.0276 | Yes |
| 264 | TNFRSF6B | TNF receptor superfamily member 6b [Source:HGNC Symbol;Acc:HGNC:11921] | 39170 | NaN | 0.0039 | Yes |
Table: GSEA details [plain text format]

  

Fig 2: KEGG\_CYTOKINE\_CYTOKINE\_RECEPTOR\_INTERACTION      
 Blue-Pink O' Gram in the Space of the Analyzed GeneSet

  

Fig 3: KEGG\_CYTOKINE\_CYTOKINE\_RECEPTOR\_INTERACTION: Random ES distribution      
 Gene set null distribution of ES for **KEGG\_CYTOKINE\_CYTOKINE\_RECEPTOR\_INTERACTION**

  
