# Supplementary material for: Simultaneous Presentation of Multiple Myeloma and Lung Cancer: Case Report and Gene Bioinformatics Analysis
Source: Front Oncol. 2022 Jun 13;12:859735. doi: 10.3389/fonc.2022.859735 (PMC9235397; doi:10.3389/fonc.2022.859735)
Supplement: Supplementary file 1 [file DataSheet_1.zip › The bioinformatic analysis of MM and lung cancer supplementary materials/Enrichment analysis/MECR/GSEA_4.1.0/LUAD TCGA/KEGG.Gsea.1639041756227/KEGG_ECM_RECEPTOR_INTERACTION.html]

Details for gene set KEGG\_ECM\_RECEPTOR\_INTERACTION[GSEA]

|  || Dataset | ExpData\_collapsed\_to\_symbols.ENSG00000116353\_profile\_in\_ExpData.cls #ENSG00000116353 |
| Phenotype | ENSG00000116353\_profile\_in\_ExpData.cls#ENSG00000116353 |
| Upregulated in class | ENSG00000116353\_neg |
| GeneSet | KEGG\_ECM\_RECEPTOR\_INTERACTION |
| Enrichment Score (ES) | -0.566358 |
| Normalized Enrichment Score (NES) | -2.2878783 |
| Nominal p-value | 0.0 |
| FDR q-value | 0.0 |
| FWER p-Value | 0.0 |
Table: GSEA Results Summary

  

Fig 1: Enrichment plot: KEGG\_ECM\_RECEPTOR\_INTERACTION      
 Profile of the Running ES Score & Positions of GeneSet Members on the Rank Ordered List

  

| SYMBOL | TITLE | RANK IN GENE LIST | RANK METRIC SCORE | RUNNING ES | CORE ENRICHMENT || 1 | AGRN | agrin [Source:HGNC Symbol;Acc:HGNC:329] | 1170 | 0.257 | -0.0077 | No |
| 2 | CD47 | CD47 molecule [Source:HGNC Symbol;Acc:HGNC:1682] | 1318 | 0.246 | 0.0098 | No |
| 3 | SDC4 | syndecan 4 [Source:HGNC Symbol;Acc:HGNC:10661] | 2069 | 0.203 | 0.0081 | No |
| 4 | SDC1 | syndecan 1 [Source:HGNC Symbol;Acc:HGNC:10658] | 2445 | 0.186 | 0.0146 | No |
| 5 | ITGA3 | integrin subunit alpha 3 [Source:HGNC Symbol;Acc:HGNC:6139] | 3614 | 0.145 | -0.0028 | No |
| 6 | CHAD | chondroadherin [Source:HGNC Symbol;Acc:HGNC:1909] | 5631 | 0.100 | -0.0456 | No |
| 7 | THBS3 | thrombospondin 3 [Source:HGNC Symbol;Acc:HGNC:11787] | 5659 | 0.099 | -0.0377 | No |
| 8 | SDC3 | syndecan 3 [Source:HGNC Symbol;Acc:HGNC:10660] | 6930 | 0.080 | -0.0632 | No |
| 9 | LAMB2 | laminin subunit beta 2 [Source:HGNC Symbol;Acc:HGNC:6487] | 7162 | 0.077 | -0.0624 | No |
| 10 | ITGB4 | integrin subunit beta 4 [Source:HGNC Symbol;Acc:HGNC:6158] | 8518 | 0.062 | -0.0916 | No |
| 11 | ITGA2B | integrin subunit alpha 2b [Source:HGNC Symbol;Acc:HGNC:6138] | 9230 | 0.055 | -0.1050 | No |
| 12 | COMP | cartilage oligomeric matrix protein [Source:HGNC Symbol;Acc:HGNC:2227] | 9245 | 0.055 | -0.1006 | No |
| 13 | COL11A2 | collagen type XI alpha 2 chain [Source:HGNC Symbol;Acc:HGNC:2187] | 10230 | 0.047 | -0.1216 | No |
| 14 | GP6 | glycoprotein VI platelet [Source:HGNC Symbol;Acc:HGNC:14388] | 13009 | 0.026 | -0.1902 | No |
| 15 | LAMA5 | laminin subunit alpha 5 [Source:HGNC Symbol;Acc:HGNC:6485] | 13742 | 0.021 | -0.2071 | No |
| 16 | GP9 | glycoprotein IX platelet [Source:HGNC Symbol;Acc:HGNC:4444] | 13795 | 0.021 | -0.2066 | No |
| 17 | IBSP | integrin binding sialoprotein [Source:HGNC Symbol;Acc:HGNC:5341] | 14378 | 0.017 | -0.2200 | No |
| 18 | DAG1 | dystroglycan 1 [Source:HGNC Symbol;Acc:HGNC:2666] | 16322 | 0.005 | -0.2691 | No |
| 19 | LAMB3 | laminin subunit beta 3 [Source:HGNC Symbol;Acc:HGNC:6490] | 16710 | 0.003 | -0.2787 | No |
| 20 | COL2A1 | collagen type II alpha 1 chain [Source:HGNC Symbol;Acc:HGNC:2200] | 17172 | 0.000 | -0.2904 | No |
| 21 | ITGA7 | integrin subunit alpha 7 [Source:HGNC Symbol;Acc:HGNC:6143] | 18409 | -0.007 | -0.3213 | No |
| 22 | ITGB6 | integrin subunit beta 6 [Source:HGNC Symbol;Acc:HGNC:6161] | 19027 | -0.011 | -0.3361 | No |
| 23 | LAMC2 | laminin subunit gamma 2 [Source:HGNC Symbol;Acc:HGNC:6493] | 19324 | -0.013 | -0.3425 | No |
| 24 | COL4A4 | collagen type IV alpha 4 chain [Source:HGNC Symbol;Acc:HGNC:2206] | 20261 | -0.018 | -0.3648 | No |
| 25 | ITGA10 | integrin subunit alpha 10 [Source:HGNC Symbol;Acc:HGNC:6135] | 20447 | -0.019 | -0.3679 | No |
| 26 | THBS4 | thrombospondin 4 [Source:HGNC Symbol;Acc:HGNC:11788] | 20600 | -0.020 | -0.3700 | No |
| 27 | LAMC3 | laminin subunit gamma 3 [Source:HGNC Symbol;Acc:HGNC:6494] | 21084 | -0.023 | -0.3803 | No |
| 28 | ITGB5 | integrin subunit beta 5 [Source:HGNC Symbol;Acc:HGNC:6160] | 21391 | -0.025 | -0.3859 | No |
| 29 | TNN | tenascin N [Source:HGNC Symbol;Acc:HGNC:22942] | 21577 | -0.026 | -0.3884 | No |
| 30 | ITGA9 | integrin subunit alpha 9 [Source:HGNC Symbol;Acc:HGNC:6145] | 25285 | -0.051 | -0.4785 | No |
| 31 | COL6A1 | collagen type VI alpha 1 chain [Source:HGNC Symbol;Acc:HGNC:2211] | 26875 | -0.062 | -0.5137 | No |
| 32 | COL4A6 | collagen type IV alpha 6 chain [Source:HGNC Symbol;Acc:HGNC:2208] | 27266 | -0.065 | -0.5180 | No |
| 33 | HMMR | hyaluronan mediated motility receptor [Source:HGNC Symbol;Acc:HGNC:5012] | 27814 | -0.070 | -0.5259 | No |
| 34 | SV2C | synaptic vesicle glycoprotein 2C [Source:HGNC Symbol;Acc:HGNC:30670] | 27986 | -0.071 | -0.5242 | No |
| 35 | SPP1 | secreted phosphoprotein 1 [Source:HGNC Symbol;Acc:HGNC:11255] | 28195 | -0.073 | -0.5232 | No |
| 36 | CD44 | CD44 molecule (Indian blood group) [Source:HGNC Symbol;Acc:HGNC:1681] | 28518 | -0.076 | -0.5249 | No |
| 37 | COL6A2 | collagen type VI alpha 2 chain [Source:HGNC Symbol;Acc:HGNC:2212] | 28651 | -0.077 | -0.5217 | No |
| 38 | SDC2 | syndecan 2 [Source:HGNC Symbol;Acc:HGNC:10659] | 30196 | -0.092 | -0.5531 | No |
| 39 | TNC | tenascin C [Source:HGNC Symbol;Acc:HGNC:5318] | 30450 | -0.094 | -0.5515 | No |
| 40 | ITGB8 | integrin subunit beta 8 [Source:HGNC Symbol;Acc:HGNC:6163] | 31035 | -0.101 | -0.5577 | Yes |
| 41 | COL11A1 | collagen type XI alpha 1 chain [Source:HGNC Symbol;Acc:HGNC:2186] | 31149 | -0.102 | -0.5518 | Yes |
| 42 | LAMA3 | laminin subunit alpha 3 [Source:HGNC Symbol;Acc:HGNC:6483] | 31400 | -0.105 | -0.5491 | Yes |
| 43 | COL1A1 | collagen type I alpha 1 chain [Source:HGNC Symbol;Acc:HGNC:2197] | 31511 | -0.107 | -0.5427 | Yes |
| 44 | ITGA11 | integrin subunit alpha 11 [Source:HGNC Symbol;Acc:HGNC:6136] | 31569 | -0.107 | -0.5350 | Yes |
| 45 | ITGB3 | integrin subunit beta 3 [Source:HGNC Symbol;Acc:HGNC:6156] | 32139 | -0.115 | -0.5396 | Yes |
| 46 | ITGA8 | integrin subunit alpha 8 [Source:HGNC Symbol;Acc:HGNC:6144] | 32742 | -0.124 | -0.5443 | Yes |
| 47 | VTN | vitronectin [Source:HGNC Symbol;Acc:HGNC:12724] | 33455 | -0.135 | -0.5508 | Yes |
| 48 | LAMC1 | laminin subunit gamma 1 [Source:HGNC Symbol;Acc:HGNC:6492] | 33481 | -0.136 | -0.5397 | Yes |
| 49 | TNXB | tenascin XB [Source:HGNC Symbol;Acc:HGNC:11976] | 33559 | -0.137 | -0.5299 | Yes |
| 50 | GP1BA | glycoprotein Ib platelet subunit alpha [Source:HGNC Symbol;Acc:HGNC:4439] | 33922 | -0.144 | -0.5268 | Yes |
| 51 | HSPG2 | heparan sulfate proteoglycan 2 [Source:HGNC Symbol;Acc:HGNC:5273] | 34017 | -0.146 | -0.5166 | Yes |
| 52 | ITGA5 | integrin subunit alpha 5 [Source:HGNC Symbol;Acc:HGNC:6141] | 34763 | -0.161 | -0.5218 | Yes |
| 53 | COL5A3 | collagen type V alpha 3 chain [Source:HGNC Symbol;Acc:HGNC:14864] | 34873 | -0.164 | -0.5105 | Yes |
| 54 | LAMA1 | laminin subunit alpha 1 [Source:HGNC Symbol;Acc:HGNC:6481] | 34927 | -0.164 | -0.4977 | Yes |
| 55 | LAMB4 | laminin subunit beta 4 [Source:HGNC Symbol;Acc:HGNC:6491] | 35118 | -0.169 | -0.4880 | Yes |
| 56 | COL6A6 | collagen type VI alpha 6 chain [Source:HGNC Symbol;Acc:HGNC:27023] | 35177 | -0.171 | -0.4748 | Yes |
| 57 | COL4A2 | collagen type IV alpha 2 chain [Source:HGNC Symbol;Acc:HGNC:2203] | 35236 | -0.172 | -0.4615 | Yes |
| 58 | TNR | tenascin R [Source:HGNC Symbol;Acc:HGNC:11953] | 35437 | -0.178 | -0.4513 | Yes |
| 59 | CD36 | CD36 molecule [Source:HGNC Symbol;Acc:HGNC:1663] | 35748 | -0.186 | -0.4432 | Yes |
| 60 | ITGA2 | integrin subunit alpha 2 [Source:HGNC Symbol;Acc:HGNC:6137] | 35833 | -0.189 | -0.4291 | Yes |
| 61 | COL5A1 | collagen type V alpha 1 chain [Source:HGNC Symbol;Acc:HGNC:2209] | 35906 | -0.191 | -0.4145 | Yes |
| 62 | THBS2 | thrombospondin 2 [Source:HGNC Symbol;Acc:HGNC:11786] | 36117 | -0.198 | -0.4029 | Yes |
| 63 | COL1A2 | collagen type I alpha 2 chain [Source:HGNC Symbol;Acc:HGNC:2198] | 36385 | -0.207 | -0.3919 | Yes |
| 64 | SV2A | synaptic vesicle glycoprotein 2A [Source:HGNC Symbol;Acc:HGNC:20566] | 36628 | -0.217 | -0.3794 | Yes |
| 65 | FN1 | fibronectin 1 [Source:HGNC Symbol;Acc:HGNC:3778] | 36715 | -0.220 | -0.3626 | Yes |
| 66 | COL3A1 | collagen type III alpha 1 chain [Source:HGNC Symbol;Acc:HGNC:2201] | 36842 | -0.227 | -0.3463 | Yes |
| 67 | COL4A1 | collagen type IV alpha 1 chain [Source:HGNC Symbol;Acc:HGNC:2202] | 36855 | -0.228 | -0.3270 | Yes |
| 68 | ITGA6 | integrin subunit alpha 6 [Source:HGNC Symbol;Acc:HGNC:6142] | 37064 | -0.239 | -0.3118 | Yes |
| 69 | THBS1 | thrombospondin 1 [Source:HGNC Symbol;Acc:HGNC:11785] | 37079 | -0.240 | -0.2915 | Yes |
| 70 | COL5A2 | collagen type V alpha 2 chain [Source:HGNC Symbol;Acc:HGNC:2210] | 37272 | -0.252 | -0.2747 | Yes |
| 71 | ITGA1 | integrin subunit alpha 1 [Source:HGNC Symbol;Acc:HGNC:6134] | 37309 | -0.254 | -0.2538 | Yes |
| 72 | GP5 | glycoprotein V platelet [Source:HGNC Symbol;Acc:HGNC:4443] | 37320 | -0.254 | -0.2322 | Yes |
| 73 | RELN | reelin [Source:HGNC Symbol;Acc:HGNC:9957] | 37356 | -0.257 | -0.2111 | Yes |
| 74 | LAMA2 | laminin subunit alpha 2 [Source:HGNC Symbol;Acc:HGNC:6482] | 37457 | -0.263 | -0.1910 | Yes |
| 75 | VWF | von Willebrand factor [Source:HGNC Symbol;Acc:HGNC:12726] | 37581 | -0.272 | -0.1707 | Yes |
| 76 | ITGAV | integrin subunit alpha V [Source:HGNC Symbol;Acc:HGNC:6150] | 37606 | -0.275 | -0.1477 | Yes |
| 77 | COL6A3 | collagen type VI alpha 3 chain [Source:HGNC Symbol;Acc:HGNC:2213] | 37608 | -0.275 | -0.1240 | Yes |
| 78 | LAMB1 | laminin subunit beta 1 [Source:HGNC Symbol;Acc:HGNC:6486] | 37639 | -0.278 | -0.1009 | Yes |
| 79 | SV2B | synaptic vesicle glycoprotein 2B [Source:HGNC Symbol;Acc:HGNC:16874] | 37769 | -0.288 | -0.0795 | Yes |
| 80 | ITGB1 | integrin subunit beta 1 [Source:HGNC Symbol;Acc:HGNC:6153] | 37850 | -0.297 | -0.0560 | Yes |
| 81 | LAMA4 | laminin subunit alpha 4 [Source:HGNC Symbol;Acc:HGNC:6484] | 38106 | -0.335 | -0.0337 | Yes |
| 82 | ITGB7 | integrin subunit beta 7 [Source:HGNC Symbol;Acc:HGNC:6162] | 38154 | -0.347 | -0.0050 | Yes |
| 83 | ITGA4 | integrin subunit alpha 4 [Source:HGNC Symbol;Acc:HGNC:6140] | 38275 | -0.394 | 0.0258 | Yes |
| 84 | GP1BB | glycoprotein Ib platelet subunit beta [Source:HGNC Symbol;Acc:HGNC:4440] | 39021 | NaN | 0.0077 | Yes |
Table: GSEA details [plain text format]

  

Fig 2: KEGG\_ECM\_RECEPTOR\_INTERACTION      
 Blue-Pink O' Gram in the Space of the Analyzed GeneSet

  

Fig 3: KEGG\_ECM\_RECEPTOR\_INTERACTION: Random ES distribution      
 Gene set null distribution of ES for **KEGG\_ECM\_RECEPTOR\_INTERACTION**

  
