# Supplementary material for: Simultaneous Presentation of Multiple Myeloma and Lung Cancer: Case Report and Gene Bioinformatics Analysis
Source: Front Oncol. 2022 Jun 13;12:859735. doi: 10.3389/fonc.2022.859735 (PMC9235397; doi:10.3389/fonc.2022.859735)
Supplement: Supplementary file 1 [file DataSheet_1.zip › The bioinformatic analysis of MM and lung cancer supplementary materials/Enrichment analysis/MECR/GSEA_4.1.0/LUAD TCGA/KEGG.Gsea.1639041756227/KEGG_FATTY_ACID_METABOLISM.html]

Details for gene set KEGG\_FATTY\_ACID\_METABOLISM[GSEA]

|  || Dataset | ExpData\_collapsed\_to\_symbols.ENSG00000116353\_profile\_in\_ExpData.cls #ENSG00000116353 |
| Phenotype | ENSG00000116353\_profile\_in\_ExpData.cls#ENSG00000116353 |
| Upregulated in class | ENSG00000116353\_pos |
| GeneSet | KEGG\_FATTY\_ACID\_METABOLISM |
| Enrichment Score (ES) | 0.5199372 |
| Normalized Enrichment Score (NES) | 1.8335314 |
| Nominal p-value | 0.0019880715 |
| FDR q-value | 0.0061454833 |
| FWER p-Value | 0.109 |
Table: GSEA Results Summary

  

Fig 1: Enrichment plot: KEGG\_FATTY\_ACID\_METABOLISM      
 Profile of the Running ES Score & Positions of GeneSet Members on the Rank Ordered List

  

| SYMBOL | TITLE | RANK IN GENE LIST | RANK METRIC SCORE | RUNNING ES | CORE ENRICHMENT || 1 | ECI1 | enoyl-CoA delta isomerase 1 [Source:HGNC Symbol;Acc:HGNC:2703] | 50 | 0.433 | 0.0755 | Yes |
| 2 | ECHS1 | "enoyl-CoA hydratase, short chain 1 [Source:HGNC Symbol;Acc:HGNC:3151]" | 238 | 0.364 | 0.1354 | Yes |
| 3 | GCDH | glutaryl-CoA dehydrogenase [Source:HGNC Symbol;Acc:HGNC:4189] | 390 | 0.334 | 0.1908 | Yes |
| 4 | HADH | hydroxyacyl-CoA dehydrogenase [Source:HGNC Symbol;Acc:HGNC:4799] | 931 | 0.276 | 0.2260 | Yes |
| 5 | CPT2 | carnitine palmitoyltransferase 2 [Source:HGNC Symbol;Acc:HGNC:2330] | 970 | 0.272 | 0.2734 | Yes |
| 6 | ACAA1 | acetyl-CoA acyltransferase 1 [Source:HGNC Symbol;Acc:HGNC:82] | 1003 | 0.270 | 0.3204 | Yes |
| 7 | ACADS | acyl-CoA dehydrogenase short chain [Source:HGNC Symbol;Acc:HGNC:90] | 1276 | 0.250 | 0.3578 | Yes |
| 8 | ALDH7A1 | aldehyde dehydrogenase 7 family member A1 [Source:HGNC Symbol;Acc:HGNC:877] | 2018 | 0.205 | 0.3753 | Yes |
| 9 | ECI2 | enoyl-CoA delta isomerase 2 [Source:HGNC Symbol;Acc:HGNC:14601] | 2081 | 0.202 | 0.4095 | Yes |
| 10 | ACADVL | acyl-CoA dehydrogenase very long chain [Source:HGNC Symbol;Acc:HGNC:92] | 2359 | 0.190 | 0.4362 | Yes |
| 11 | ADH5 | "alcohol dehydrogenase 5 (class III), chi polypeptide [Source:HGNC Symbol;Acc:HGNC:253]" | 2442 | 0.187 | 0.4673 | Yes |
| 12 | ACOX3 | "acyl-CoA oxidase 3, pristanoyl [Source:HGNC Symbol;Acc:HGNC:121]" | 2763 | 0.173 | 0.4898 | Yes |
| 13 | CYP4A22 | cytochrome P450 family 4 subfamily A member 22 [Source:HGNC Symbol;Acc:HGNC:20575] | 3992 | 0.134 | 0.4823 | Yes |
| 14 | ALDH2 | aldehyde dehydrogenase 2 family member [Source:HGNC Symbol;Acc:HGNC:404] | 4910 | 0.113 | 0.4790 | Yes |
| 15 | CYP4A11 | cytochrome P450 family 4 subfamily A member 11 [Source:HGNC Symbol;Acc:HGNC:2642] | 4965 | 0.112 | 0.4974 | Yes |
| 16 | ALDH3A2 | aldehyde dehydrogenase 3 family member A2 [Source:HGNC Symbol;Acc:HGNC:403] | 5202 | 0.108 | 0.5105 | Yes |
| 17 | HADHB | hydroxyacyl-CoA dehydrogenase trifunctional multienzyme complex subunit beta [Source:HGNC Symbol;Acc:HGNC:4803] | 5537 | 0.101 | 0.5199 | Yes |
| 18 | ACAT1 | acetyl-CoA acetyltransferase 1 [Source:HGNC Symbol;Acc:HGNC:93] | 6430 | 0.087 | 0.5126 | No |
| 19 | ADH1C | "alcohol dehydrogenase 1C (class I), gamma polypeptide [Source:HGNC Symbol;Acc:HGNC:251]" | 7209 | 0.077 | 0.5064 | No |
| 20 | ALDH9A1 | aldehyde dehydrogenase 9 family member A1 [Source:HGNC Symbol;Acc:HGNC:412] | 7785 | 0.070 | 0.5041 | No |
| 21 | HADHA | hydroxyacyl-CoA dehydrogenase trifunctional multienzyme complex subunit alpha [Source:HGNC Symbol;Acc:HGNC:4801] | 9756 | 0.051 | 0.4630 | No |
| 22 | ACSL5 | acyl-CoA synthetase long chain family member 5 [Source:HGNC Symbol;Acc:HGNC:16526] | 10039 | 0.048 | 0.4644 | No |
| 23 | ADH6 | alcohol dehydrogenase 6 (class V) [Source:HGNC Symbol;Acc:HGNC:255] | 10170 | 0.047 | 0.4695 | No |
| 24 | ADH7 | "alcohol dehydrogenase 7 (class IV), mu or sigma polypeptide [Source:HGNC Symbol;Acc:HGNC:256]" | 11251 | 0.039 | 0.4489 | No |
| 25 | ACADM | acyl-CoA dehydrogenase medium chain [Source:HGNC Symbol;Acc:HGNC:89] | 11264 | 0.039 | 0.4555 | No |
| 26 | ACADSB | acyl-CoA dehydrogenase short/branched chain [Source:HGNC Symbol;Acc:HGNC:91] | 12023 | 0.033 | 0.4421 | No |
| 27 | ADH1A | "alcohol dehydrogenase 1A (class I), alpha polypeptide [Source:HGNC Symbol;Acc:HGNC:249]" | 12208 | 0.032 | 0.4430 | No |
| 28 | CPT1B | carnitine palmitoyltransferase 1B [Source:HGNC Symbol;Acc:HGNC:2329] | 16994 | 0.001 | 0.3214 | No |
| 29 | ACADL | acyl-CoA dehydrogenase long chain [Source:HGNC Symbol;Acc:HGNC:88] | 18552 | -0.008 | 0.2832 | No |
| 30 | ACAT2 | acetyl-CoA acetyltransferase 2 [Source:HGNC Symbol;Acc:HGNC:94] | 19909 | -0.016 | 0.2515 | No |
| 31 | ALDH1B1 | aldehyde dehydrogenase 1 family member B1 [Source:HGNC Symbol;Acc:HGNC:407] | 21379 | -0.025 | 0.2186 | No |
| 32 | ADH4 | "alcohol dehydrogenase 4 (class II), pi polypeptide [Source:HGNC Symbol;Acc:HGNC:252]" | 21527 | -0.026 | 0.2195 | No |
| 33 | EHHADH | enoyl-CoA hydratase and 3-hydroxyacyl CoA dehydrogenase [Source:HGNC Symbol;Acc:HGNC:3247] | 23047 | -0.035 | 0.1871 | No |
| 34 | ADH1B | "alcohol dehydrogenase 1B (class I), beta polypeptide [Source:HGNC Symbol;Acc:HGNC:250]" | 26940 | -0.063 | 0.0992 | No |
| 35 | ACSL1 | acyl-CoA synthetase long chain family member 1 [Source:HGNC Symbol;Acc:HGNC:3569] | 27078 | -0.064 | 0.1070 | No |
| 36 | ACOX1 | acyl-CoA oxidase 1 [Source:HGNC Symbol;Acc:HGNC:119] | 27845 | -0.070 | 0.0999 | No |
| 37 | ACSL6 | acyl-CoA synthetase long chain family member 6 [Source:HGNC Symbol;Acc:HGNC:16496] | 33792 | -0.142 | -0.0263 | No |
| 38 | CPT1C | carnitine palmitoyltransferase 1C [Source:HGNC Symbol;Acc:HGNC:18540] | 34855 | -0.163 | -0.0244 | No |
| 39 | ACAA2 | acetyl-CoA acyltransferase 2 [Source:HGNC Symbol;Acc:HGNC:83] | 34923 | -0.164 | 0.0030 | No |
| 40 | ACSL3 | acyl-CoA synthetase long chain family member 3 [Source:HGNC Symbol;Acc:HGNC:3570] | 35301 | -0.174 | 0.0243 | No |
| 41 | ACSL4 | acyl-CoA synthetase long chain family member 4 [Source:HGNC Symbol;Acc:HGNC:3571] | 35339 | -0.175 | 0.0544 | No |
| 42 | CPT1A | carnitine palmitoyltransferase 1A [Source:HGNC Symbol;Acc:HGNC:2328] | 37482 | -0.265 | 0.0469 | No |
Table: GSEA details [plain text format]

  

Fig 2: KEGG\_FATTY\_ACID\_METABOLISM      
 Blue-Pink O' Gram in the Space of the Analyzed GeneSet

  

Fig 3: KEGG\_FATTY\_ACID\_METABOLISM: Random ES distribution      
 Gene set null distribution of ES for **KEGG\_FATTY\_ACID\_METABOLISM**

  
