# Supplementary material for: Simultaneous Presentation of Multiple Myeloma and Lung Cancer: Case Report and Gene Bioinformatics Analysis
Source: Front Oncol. 2022 Jun 13;12:859735. doi: 10.3389/fonc.2022.859735 (PMC9235397; doi:10.3389/fonc.2022.859735)
Supplement: Supplementary file 1 [file DataSheet_1.zip › The bioinformatic analysis of MM and lung cancer supplementary materials/Enrichment analysis/MECR/GSEA_4.1.0/LUAD TCGA/KEGG.Gsea.1639041756227/KEGG_FOCAL_ADHESION.html]

Details for gene set KEGG\_FOCAL\_ADHESION[GSEA]

|  || Dataset | ExpData\_collapsed\_to\_symbols.ENSG00000116353\_profile\_in\_ExpData.cls #ENSG00000116353 |
| Phenotype | ENSG00000116353\_profile\_in\_ExpData.cls#ENSG00000116353 |
| Upregulated in class | ENSG00000116353\_neg |
| GeneSet | KEGG\_FOCAL\_ADHESION |
| Enrichment Score (ES) | -0.4810571 |
| Normalized Enrichment Score (NES) | -2.198601 |
| Nominal p-value | 0.0 |
| FDR q-value | 8.3540115E-5 |
| FWER p-Value | 0.001 |
Table: GSEA Results Summary

  

Fig 1: Enrichment plot: KEGG\_FOCAL\_ADHESION      
 Profile of the Running ES Score & Positions of GeneSet Members on the Rank Ordered List

  

| SYMBOL | TITLE | RANK IN GENE LIST | RANK METRIC SCORE | RUNNING ES | CORE ENRICHMENT || 1 | BAD | BCL2 associated agonist of cell death [Source:HGNC Symbol;Acc:HGNC:936] | 174 | 0.379 | 0.0089 | No |
| 2 | RAC3 | Rac family small GTPase 3 [Source:HGNC Symbol;Acc:HGNC:9803] | 528 | 0.316 | 0.0111 | No |
| 3 | PIK3R2 | phosphoinositide-3-kinase regulatory subunit 2 [Source:HGNC Symbol;Acc:HGNC:8980] | 545 | 0.314 | 0.0218 | No |
| 4 | VEGFB | vascular endothelial growth factor B [Source:HGNC Symbol;Acc:HGNC:12681] | 586 | 0.309 | 0.0317 | No |
| 5 | MYL5 | myosin light chain 5 [Source:HGNC Symbol;Acc:HGNC:7586] | 841 | 0.284 | 0.0352 | No |
| 6 | CCND3 | cyclin D3 [Source:HGNC Symbol;Acc:HGNC:1585] | 1042 | 0.267 | 0.0395 | No |
| 7 | HRAS | "HRas proto-oncogene, GTPase [Source:HGNC Symbol;Acc:HGNC:5173]" | 1513 | 0.234 | 0.0358 | No |
| 8 | MYLPF | "myosin light chain, phosphorylatable, fast skeletal muscle [Source:HGNC Symbol;Acc:HGNC:29824]" | 1542 | 0.232 | 0.0433 | No |
| 9 | MAPK3 | mitogen-activated protein kinase 3 [Source:HGNC Symbol;Acc:HGNC:6877] | 1645 | 0.226 | 0.0487 | No |
| 10 | PPP1CA | protein phosphatase 1 catalytic subunit alpha [Source:HGNC Symbol;Acc:HGNC:9281] | 1826 | 0.216 | 0.0517 | No |
| 11 | MYL12B | myosin light chain 12B [Source:HGNC Symbol;Acc:HGNC:29827] | 2099 | 0.201 | 0.0519 | No |
| 12 | CDC42 | cell division cycle 42 [Source:HGNC Symbol;Acc:HGNC:1736] | 2158 | 0.198 | 0.0574 | No |
| 13 | RAC1 | Rac family small GTPase 1 [Source:HGNC Symbol;Acc:HGNC:9801] | 2566 | 0.182 | 0.0534 | No |
| 14 | PDGFA | platelet derived growth factor subunit A [Source:HGNC Symbol;Acc:HGNC:8799] | 2710 | 0.175 | 0.0559 | No |
| 15 | AKT1 | AKT serine/threonine kinase 1 [Source:HGNC Symbol;Acc:HGNC:391] | 3179 | 0.158 | 0.0496 | No |
| 16 | ITGA3 | integrin subunit alpha 3 [Source:HGNC Symbol;Acc:HGNC:6139] | 3614 | 0.145 | 0.0436 | No |
| 17 | PDGFD | platelet derived growth factor D [Source:HGNC Symbol;Acc:HGNC:30620] | 3731 | 0.141 | 0.0456 | No |
| 18 | EGF | epidermal growth factor [Source:HGNC Symbol;Acc:HGNC:3229] | 3795 | 0.140 | 0.0489 | No |
| 19 | MYL12A | myosin light chain 12A [Source:HGNC Symbol;Acc:HGNC:16701] | 3874 | 0.138 | 0.0518 | No |
| 20 | ACTB | actin beta [Source:HGNC Symbol;Acc:HGNC:132] | 4499 | 0.122 | 0.0402 | No |
| 21 | BCAR1 | "BCAR1 scaffold protein, Cas family member [Source:HGNC Symbol;Acc:HGNC:971]" | 4546 | 0.121 | 0.0432 | No |
| 22 | ERBB2 | erb-b2 receptor tyrosine kinase 2 [Source:HGNC Symbol;Acc:HGNC:3430] | 4642 | 0.118 | 0.0450 | No |
| 23 | MET | "MET proto-oncogene, receptor tyrosine kinase [Source:HGNC Symbol;Acc:HGNC:7029]" | 4748 | 0.116 | 0.0464 | No |
| 24 | PAK4 | p21 (RAC1) activated kinase 4 [Source:HGNC Symbol;Acc:HGNC:16059] | 4914 | 0.113 | 0.0462 | No |
| 25 | JUN | "Jun proto-oncogene, AP-1 transcription factor subunit [Source:HGNC Symbol;Acc:HGNC:6204]" | 5045 | 0.110 | 0.0468 | No |
| 26 | SHC1 | SHC adaptor protein 1 [Source:HGNC Symbol;Acc:HGNC:10840] | 5181 | 0.108 | 0.0471 | No |
| 27 | PDGFC | platelet derived growth factor C [Source:HGNC Symbol;Acc:HGNC:8801] | 5463 | 0.103 | 0.0436 | No |
| 28 | ACTG1 | actin gamma 1 [Source:HGNC Symbol;Acc:HGNC:144] | 5516 | 0.102 | 0.0458 | No |
| 29 | CHAD | chondroadherin [Source:HGNC Symbol;Acc:HGNC:1909] | 5631 | 0.100 | 0.0464 | No |
| 30 | THBS3 | thrombospondin 3 [Source:HGNC Symbol;Acc:HGNC:11787] | 5659 | 0.099 | 0.0493 | No |
| 31 | CAV2 | caveolin 2 [Source:HGNC Symbol;Acc:HGNC:1528] | 5685 | 0.099 | 0.0521 | No |
| 32 | PAK6 | p21 (RAC1) activated kinase 6 [Source:HGNC Symbol;Acc:HGNC:16061] | 5767 | 0.098 | 0.0535 | No |
| 33 | SHC2 | SHC adaptor protein 2 [Source:HGNC Symbol;Acc:HGNC:29869] | 6275 | 0.089 | 0.0437 | No |
| 34 | PTK2 | protein tyrosine kinase 2 [Source:HGNC Symbol;Acc:HGNC:9611] | 6431 | 0.087 | 0.0428 | No |
| 35 | LAMB2 | laminin subunit beta 2 [Source:HGNC Symbol;Acc:HGNC:6487] | 7162 | 0.077 | 0.0269 | No |
| 36 | CAV3 | caveolin 3 [Source:HGNC Symbol;Acc:HGNC:1529] | 7783 | 0.070 | 0.0135 | No |
| 37 | VEGFA | vascular endothelial growth factor A [Source:HGNC Symbol;Acc:HGNC:12680] | 8089 | 0.067 | 0.0080 | No |
| 38 | PPP1CB | protein phosphatase 1 catalytic subunit beta [Source:HGNC Symbol;Acc:HGNC:9282] | 8186 | 0.066 | 0.0079 | No |
| 39 | MAPK9 | mitogen-activated protein kinase 9 [Source:HGNC Symbol;Acc:HGNC:6886] | 8347 | 0.064 | 0.0061 | No |
| 40 | CAPN2 | calpain 2 [Source:HGNC Symbol;Acc:HGNC:1479] | 8442 | 0.063 | 0.0059 | No |
| 41 | PXN | paxillin [Source:HGNC Symbol;Acc:HGNC:9718] | 8446 | 0.063 | 0.0080 | No |
| 42 | ITGB4 | integrin subunit beta 4 [Source:HGNC Symbol;Acc:HGNC:6158] | 8518 | 0.062 | 0.0084 | No |
| 43 | RAF1 | "Raf-1 proto-oncogene, serine/threonine kinase [Source:HGNC Symbol;Acc:HGNC:9829]" | 8635 | 0.061 | 0.0076 | No |
| 44 | PIK3R3 | phosphoinositide-3-kinase regulatory subunit 3 [Source:HGNC Symbol;Acc:HGNC:8981] | 9028 | 0.057 | -0.0004 | No |
| 45 | ITGA2B | integrin subunit alpha 2b [Source:HGNC Symbol;Acc:HGNC:6138] | 9230 | 0.055 | -0.0036 | No |
| 46 | COMP | cartilage oligomeric matrix protein [Source:HGNC Symbol;Acc:HGNC:2227] | 9245 | 0.055 | -0.0020 | No |
| 47 | EGFR | epidermal growth factor receptor [Source:HGNC Symbol;Acc:HGNC:3236] | 9728 | 0.051 | -0.0125 | No |
| 48 | COL11A2 | collagen type XI alpha 2 chain [Source:HGNC Symbol;Acc:HGNC:2187] | 10230 | 0.047 | -0.0237 | No |
| 49 | AKT2 | AKT serine/threonine kinase 2 [Source:HGNC Symbol;Acc:HGNC:392] | 10579 | 0.044 | -0.0310 | No |
| 50 | RHOA | ras homolog family member A [Source:HGNC Symbol;Acc:HGNC:667] | 11437 | 0.037 | -0.0516 | No |
| 51 | MYL9 | myosin light chain 9 [Source:HGNC Symbol;Acc:HGNC:15754] | 12602 | 0.029 | -0.0803 | No |
| 52 | MYL10 | myosin light chain 10 [Source:HGNC Symbol;Acc:HGNC:29825] | 13208 | 0.024 | -0.0949 | No |
| 53 | LAMA5 | laminin subunit alpha 5 [Source:HGNC Symbol;Acc:HGNC:6485] | 13742 | 0.021 | -0.1078 | No |
| 54 | IBSP | integrin binding sialoprotein [Source:HGNC Symbol;Acc:HGNC:5341] | 14378 | 0.017 | -0.1234 | No |
| 55 | SHC3 | SHC adaptor protein 3 [Source:HGNC Symbol;Acc:HGNC:18181] | 14963 | 0.013 | -0.1379 | No |
| 56 | MYL7 | myosin light chain 7 [Source:HGNC Symbol;Acc:HGNC:21719] | 15382 | 0.011 | -0.1482 | No |
| 57 | CAV1 | caveolin 1 [Source:HGNC Symbol;Acc:HGNC:1527] | 16424 | 0.005 | -0.1746 | No |
| 58 | LAMB3 | laminin subunit beta 3 [Source:HGNC Symbol;Acc:HGNC:6490] | 16710 | 0.003 | -0.1818 | No |
| 59 | COL2A1 | collagen type II alpha 1 chain [Source:HGNC Symbol;Acc:HGNC:2200] | 17172 | 0.000 | -0.1936 | No |
| 60 | MAPK10 | mitogen-activated protein kinase 10 [Source:HGNC Symbol;Acc:HGNC:6872] | 17304 | -0.001 | -0.1969 | No |
| 61 | PAK5 | p21 (RAC1) activated kinase 5 [Source:HGNC Symbol;Acc:HGNC:15916] | 17436 | -0.002 | -0.2002 | No |
| 62 | MYL2 | myosin light chain 2 [Source:HGNC Symbol;Acc:HGNC:7583] | 18201 | -0.006 | -0.2195 | No |
| 63 | ZYX | zyxin [Source:HGNC Symbol;Acc:HGNC:13200] | 18314 | -0.007 | -0.2222 | No |
| 64 | ITGA7 | integrin subunit alpha 7 [Source:HGNC Symbol;Acc:HGNC:6143] | 18409 | -0.007 | -0.2243 | No |
| 65 | CRK | "CRK proto-oncogene, adaptor protein [Source:HGNC Symbol;Acc:HGNC:2362]" | 18444 | -0.007 | -0.2249 | No |
| 66 | PGF | placental growth factor [Source:HGNC Symbol;Acc:HGNC:8893] | 18524 | -0.008 | -0.2267 | No |
| 67 | ITGB6 | integrin subunit beta 6 [Source:HGNC Symbol;Acc:HGNC:6161] | 19027 | -0.011 | -0.2391 | No |
| 68 | VEGFD | vascular endothelial growth factor D [Source:HGNC Symbol;Acc:HGNC:3708] | 19101 | -0.011 | -0.2406 | No |
| 69 | ACTN4 | actinin alpha 4 [Source:HGNC Symbol;Acc:HGNC:166] | 19317 | -0.013 | -0.2456 | No |
| 70 | LAMC2 | laminin subunit gamma 2 [Source:HGNC Symbol;Acc:HGNC:6493] | 19324 | -0.013 | -0.2453 | No |
| 71 | COL4A4 | collagen type IV alpha 4 chain [Source:HGNC Symbol;Acc:HGNC:2206] | 20261 | -0.018 | -0.2686 | No |
| 72 | PIP5K1C | phosphatidylinositol-4-phosphate 5-kinase type 1 gamma [Source:HGNC Symbol;Acc:HGNC:8996] | 20430 | -0.019 | -0.2722 | No |
| 73 | ITGA10 | integrin subunit alpha 10 [Source:HGNC Symbol;Acc:HGNC:6135] | 20447 | -0.019 | -0.2719 | No |
| 74 | RAP1A | "RAP1A, member of RAS oncogene family [Source:HGNC Symbol;Acc:HGNC:9855]" | 20560 | -0.020 | -0.2741 | No |
| 75 | THBS4 | thrombospondin 4 [Source:HGNC Symbol;Acc:HGNC:11788] | 20600 | -0.020 | -0.2744 | No |
| 76 | LAMC3 | laminin subunit gamma 3 [Source:HGNC Symbol;Acc:HGNC:6494] | 21084 | -0.023 | -0.2859 | No |
| 77 | ITGB5 | integrin subunit beta 5 [Source:HGNC Symbol;Acc:HGNC:6160] | 21391 | -0.025 | -0.2928 | No |
| 78 | RASGRF1 | Ras protein specific guanine nucleotide releasing factor 1 [Source:HGNC Symbol;Acc:HGNC:9875] | 21407 | -0.025 | -0.2923 | No |
| 79 | TNN | tenascin N [Source:HGNC Symbol;Acc:HGNC:22942] | 21577 | -0.026 | -0.2957 | No |
| 80 | VASP | vasodilator stimulated phosphoprotein [Source:HGNC Symbol;Acc:HGNC:12652] | 22726 | -0.033 | -0.3239 | No |
| 81 | SRC | "SRC proto-oncogene, non-receptor tyrosine kinase [Source:HGNC Symbol;Acc:HGNC:11283]" | 23652 | -0.039 | -0.3461 | No |
| 82 | RAC2 | Rac family small GTPase 2 [Source:HGNC Symbol;Acc:HGNC:9802] | 24228 | -0.043 | -0.3593 | No |
| 83 | FLNA | filamin A [Source:HGNC Symbol;Acc:HGNC:3754] | 24250 | -0.043 | -0.3583 | No |
| 84 | SHC4 | SHC adaptor protein 4 [Source:HGNC Symbol;Acc:HGNC:16743] | 24342 | -0.044 | -0.3591 | No |
| 85 | VAV2 | vav guanine nucleotide exchange factor 2 [Source:HGNC Symbol;Acc:HGNC:12658] | 24578 | -0.046 | -0.3634 | No |
| 86 | ITGA9 | integrin subunit alpha 9 [Source:HGNC Symbol;Acc:HGNC:6145] | 25285 | -0.051 | -0.3797 | No |
| 87 | CCND1 | cyclin D1 [Source:HGNC Symbol;Acc:HGNC:1582] | 25465 | -0.052 | -0.3824 | No |
| 88 | VEGFC | vascular endothelial growth factor C [Source:HGNC Symbol;Acc:HGNC:12682] | 25822 | -0.055 | -0.3896 | No |
| 89 | ELK1 | ETS transcription factor ELK1 [Source:HGNC Symbol;Acc:HGNC:3321] | 26567 | -0.060 | -0.4065 | No |
| 90 | MYLK2 | myosin light chain kinase 2 [Source:HGNC Symbol;Acc:HGNC:16243] | 26644 | -0.061 | -0.4063 | No |
| 91 | DIAPH1 | diaphanous related formin 1 [Source:HGNC Symbol;Acc:HGNC:2876] | 26753 | -0.061 | -0.4069 | No |
| 92 | COL6A1 | collagen type VI alpha 1 chain [Source:HGNC Symbol;Acc:HGNC:2211] | 26875 | -0.062 | -0.4078 | No |
| 93 | COL4A6 | collagen type IV alpha 6 chain [Source:HGNC Symbol;Acc:HGNC:2208] | 27266 | -0.065 | -0.4155 | No |
| 94 | PTEN | phosphatase and tensin homolog [Source:HGNC Symbol;Acc:HGNC:9588] | 27697 | -0.069 | -0.4240 | No |
| 95 | MYLK3 | myosin light chain kinase 3 [Source:HGNC Symbol;Acc:HGNC:29826] | 27794 | -0.069 | -0.4240 | No |
| 96 | KDR | kinase insert domain receptor [Source:HGNC Symbol;Acc:HGNC:6307] | 27955 | -0.071 | -0.4256 | No |
| 97 | SPP1 | secreted phosphoprotein 1 [Source:HGNC Symbol;Acc:HGNC:11255] | 28195 | -0.073 | -0.4291 | No |
| 98 | ILK | integrin linked kinase [Source:HGNC Symbol;Acc:HGNC:6040] | 28385 | -0.074 | -0.4313 | No |
| 99 | COL6A2 | collagen type VI alpha 2 chain [Source:HGNC Symbol;Acc:HGNC:2212] | 28651 | -0.077 | -0.4354 | No |
| 100 | VAV3 | vav guanine nucleotide exchange factor 3 [Source:HGNC Symbol;Acc:HGNC:12659] | 29000 | -0.080 | -0.4415 | No |
| 101 | CTNNB1 | catenin beta 1 [Source:HGNC Symbol;Acc:HGNC:2514] | 29356 | -0.083 | -0.4476 | No |
| 102 | PAK1 | p21 (RAC1) activated kinase 1 [Source:HGNC Symbol;Acc:HGNC:8590] | 29530 | -0.085 | -0.4490 | No |
| 103 | BRAF | "B-Raf proto-oncogene, serine/threonine kinase [Source:HGNC Symbol;Acc:HGNC:1097]" | 29630 | -0.086 | -0.4485 | No |
| 104 | TNC | tenascin C [Source:HGNC Symbol;Acc:HGNC:5318] | 30450 | -0.094 | -0.4661 | No |
| 105 | ITGB8 | integrin subunit beta 8 [Source:HGNC Symbol;Acc:HGNC:6163] | 31035 | -0.101 | -0.4775 | Yes |
| 106 | PAK3 | p21 (RAC1) activated kinase 3 [Source:HGNC Symbol;Acc:HGNC:8592] | 31145 | -0.102 | -0.4767 | Yes |
| 107 | COL11A1 | collagen type XI alpha 1 chain [Source:HGNC Symbol;Acc:HGNC:2186] | 31149 | -0.102 | -0.4731 | Yes |
| 108 | PARVB | parvin beta [Source:HGNC Symbol;Acc:HGNC:14653] | 31292 | -0.104 | -0.4731 | Yes |
| 109 | LAMA3 | laminin subunit alpha 3 [Source:HGNC Symbol;Acc:HGNC:6483] | 31400 | -0.105 | -0.4721 | Yes |
| 110 | COL1A1 | collagen type I alpha 1 chain [Source:HGNC Symbol;Acc:HGNC:2197] | 31511 | -0.107 | -0.4712 | Yes |
| 111 | PRKCG | protein kinase C gamma [Source:HGNC Symbol;Acc:HGNC:9402] | 31522 | -0.107 | -0.4676 | Yes |
| 112 | PDGFB | platelet derived growth factor subunit B [Source:HGNC Symbol;Acc:HGNC:8800] | 31529 | -0.107 | -0.4640 | Yes |
| 113 | ITGA11 | integrin subunit alpha 11 [Source:HGNC Symbol;Acc:HGNC:6136] | 31569 | -0.107 | -0.4612 | Yes |
| 114 | ACTN3 | actinin alpha 3 [Source:HGNC Symbol;Acc:HGNC:165] | 31627 | -0.108 | -0.4588 | Yes |
| 115 | DOCK1 | dedicator of cytokinesis 1 [Source:HGNC Symbol;Acc:HGNC:2987] | 31711 | -0.109 | -0.4571 | Yes |
| 116 | PIK3CD | "phosphatidylinositol-4,5-bisphosphate 3-kinase catalytic subunit delta [Source:HGNC Symbol;Acc:HGNC:8977]" | 31859 | -0.111 | -0.4569 | Yes |
| 117 | PRKCA | protein kinase C alpha [Source:HGNC Symbol;Acc:HGNC:9393] | 32080 | -0.114 | -0.4585 | Yes |
| 118 | ITGB3 | integrin subunit beta 3 [Source:HGNC Symbol;Acc:HGNC:6156] | 32139 | -0.115 | -0.4559 | Yes |
| 119 | ACTN2 | actinin alpha 2 [Source:HGNC Symbol;Acc:HGNC:164] | 32475 | -0.120 | -0.4603 | Yes |
| 120 | PARVG | parvin gamma [Source:HGNC Symbol;Acc:HGNC:14654] | 32507 | -0.120 | -0.4568 | Yes |
| 121 | PARVA | parvin alpha [Source:HGNC Symbol;Acc:HGNC:14652] | 32629 | -0.122 | -0.4556 | Yes |
| 122 | ITGA8 | integrin subunit alpha 8 [Source:HGNC Symbol;Acc:HGNC:6144] | 32742 | -0.124 | -0.4541 | Yes |
| 123 | XIAP | X-linked inhibitor of apoptosis [Source:HGNC Symbol;Acc:HGNC:592] | 32963 | -0.127 | -0.4552 | Yes |
| 124 | IGF1R | insulin like growth factor 1 receptor [Source:HGNC Symbol;Acc:HGNC:5465] | 33132 | -0.130 | -0.4549 | Yes |
| 125 | PDPK1 | 3-phosphoinositide dependent protein kinase 1 [Source:HGNC Symbol;Acc:HGNC:8816] | 33314 | -0.133 | -0.4549 | Yes |
| 126 | FLNB | filamin B [Source:HGNC Symbol;Acc:HGNC:3755] | 33363 | -0.134 | -0.4513 | Yes |
| 127 | VTN | vitronectin [Source:HGNC Symbol;Acc:HGNC:12724] | 33455 | -0.135 | -0.4489 | Yes |
| 128 | LAMC1 | laminin subunit gamma 1 [Source:HGNC Symbol;Acc:HGNC:6492] | 33481 | -0.136 | -0.4447 | Yes |
| 129 | TNXB | tenascin XB [Source:HGNC Symbol;Acc:HGNC:11976] | 33559 | -0.137 | -0.4418 | Yes |
| 130 | MAPK1 | mitogen-activated protein kinase 1 [Source:HGNC Symbol;Acc:HGNC:6871] | 33636 | -0.139 | -0.4389 | Yes |
| 131 | FLT4 | fms related receptor tyrosine kinase 4 [Source:HGNC Symbol;Acc:HGNC:3767] | 34137 | -0.148 | -0.4464 | Yes |
| 132 | BIRC2 | baculoviral IAP repeat containing 2 [Source:HGNC Symbol;Acc:HGNC:590] | 34221 | -0.149 | -0.4433 | Yes |
| 133 | CRKL | "CRK like proto-oncogene, adaptor protein [Source:HGNC Symbol;Acc:HGNC:2363]" | 34421 | -0.154 | -0.4429 | Yes |
| 134 | RAP1B | "RAP1B, member of RAS oncogene family [Source:HGNC Symbol;Acc:HGNC:9857]" | 34571 | -0.156 | -0.4412 | Yes |
| 135 | SOS2 | SOS Ras/Rho guanine nucleotide exchange factor 2 [Source:HGNC Symbol;Acc:HGNC:11188] | 34598 | -0.157 | -0.4363 | Yes |
| 136 | ITGA5 | integrin subunit alpha 5 [Source:HGNC Symbol;Acc:HGNC:6141] | 34763 | -0.161 | -0.4348 | Yes |
| 137 | PIK3R1 | phosphoinositide-3-kinase regulatory subunit 1 [Source:HGNC Symbol;Acc:HGNC:8979] | 34788 | -0.162 | -0.4297 | Yes |
| 138 | VAV1 | vav guanine nucleotide exchange factor 1 [Source:HGNC Symbol;Acc:HGNC:12657] | 34853 | -0.163 | -0.4256 | Yes |
| 139 | PIK3CB | "phosphatidylinositol-4,5-bisphosphate 3-kinase catalytic subunit beta [Source:HGNC Symbol;Acc:HGNC:8976]" | 34871 | -0.164 | -0.4202 | Yes |
| 140 | COL5A3 | collagen type V alpha 3 chain [Source:HGNC Symbol;Acc:HGNC:14864] | 34873 | -0.164 | -0.4145 | Yes |
| 141 | LAMA1 | laminin subunit alpha 1 [Source:HGNC Symbol;Acc:HGNC:6481] | 34927 | -0.164 | -0.4100 | Yes |
| 142 | FLNC | filamin C [Source:HGNC Symbol;Acc:HGNC:3756] | 34929 | -0.165 | -0.4042 | Yes |
| 143 | LAMB4 | laminin subunit beta 4 [Source:HGNC Symbol;Acc:HGNC:6491] | 35118 | -0.169 | -0.4031 | Yes |
| 144 | COL6A6 | collagen type VI alpha 6 chain [Source:HGNC Symbol;Acc:HGNC:27023] | 35177 | -0.171 | -0.3985 | Yes |
| 145 | TLN2 | talin 2 [Source:HGNC Symbol;Acc:HGNC:15447] | 35192 | -0.171 | -0.3928 | Yes |
| 146 | MAP2K1 | mitogen-activated protein kinase kinase 1 [Source:HGNC Symbol;Acc:HGNC:6840] | 35210 | -0.171 | -0.3872 | Yes |
| 147 | COL4A2 | collagen type IV alpha 2 chain [Source:HGNC Symbol;Acc:HGNC:2203] | 35236 | -0.172 | -0.3818 | Yes |
| 148 | PPP1CC | protein phosphatase 1 catalytic subunit gamma [Source:HGNC Symbol;Acc:HGNC:9283] | 35392 | -0.176 | -0.3795 | Yes |
| 149 | MAPK8 | mitogen-activated protein kinase 8 [Source:HGNC Symbol;Acc:HGNC:6881] | 35413 | -0.177 | -0.3738 | Yes |
| 150 | TNR | tenascin R [Source:HGNC Symbol;Acc:HGNC:11953] | 35437 | -0.178 | -0.3681 | Yes |
| 151 | ARHGAP35 | Rho GTPase activating protein 35 [Source:HGNC Symbol;Acc:HGNC:4591] | 35598 | -0.182 | -0.3657 | Yes |
| 152 | ARHGAP5 | Rho GTPase activating protein 5 [Source:HGNC Symbol;Acc:HGNC:675] | 35738 | -0.186 | -0.3627 | Yes |
| 153 | ITGA2 | integrin subunit alpha 2 [Source:HGNC Symbol;Acc:HGNC:6137] | 35833 | -0.189 | -0.3585 | Yes |
| 154 | BIRC3 | baculoviral IAP repeat containing 3 [Source:HGNC Symbol;Acc:HGNC:591] | 35840 | -0.189 | -0.3519 | Yes |
| 155 | COL5A1 | collagen type V alpha 1 chain [Source:HGNC Symbol;Acc:HGNC:2209] | 35906 | -0.191 | -0.3469 | Yes |
| 156 | VCL | vinculin [Source:HGNC Symbol;Acc:HGNC:12665] | 35933 | -0.192 | -0.3408 | Yes |
| 157 | ACTN1 | actinin alpha 1 [Source:HGNC Symbol;Acc:HGNC:163] | 36070 | -0.196 | -0.3373 | Yes |
| 158 | THBS2 | thrombospondin 2 [Source:HGNC Symbol;Acc:HGNC:11786] | 36117 | -0.198 | -0.3315 | Yes |
| 159 | CCND2 | cyclin D2 [Source:HGNC Symbol;Acc:HGNC:1583] | 36339 | -0.206 | -0.3299 | Yes |
| 160 | COL1A2 | collagen type I alpha 2 chain [Source:HGNC Symbol;Acc:HGNC:2198] | 36385 | -0.207 | -0.3237 | Yes |
| 161 | PDGFRB | platelet derived growth factor receptor beta [Source:HGNC Symbol;Acc:HGNC:8804] | 36398 | -0.208 | -0.3166 | Yes |
| 162 | IGF1 | insulin like growth factor 1 [Source:HGNC Symbol;Acc:HGNC:5464] | 36418 | -0.208 | -0.3098 | Yes |
| 163 | GRB2 | growth factor receptor bound protein 2 [Source:HGNC Symbol;Acc:HGNC:4566] | 36516 | -0.212 | -0.3048 | Yes |
| 164 | FN1 | fibronectin 1 [Source:HGNC Symbol;Acc:HGNC:3778] | 36715 | -0.220 | -0.3020 | Yes |
| 165 | COL3A1 | collagen type III alpha 1 chain [Source:HGNC Symbol;Acc:HGNC:2201] | 36842 | -0.227 | -0.2972 | Yes |
| 166 | COL4A1 | collagen type IV alpha 1 chain [Source:HGNC Symbol;Acc:HGNC:2202] | 36855 | -0.228 | -0.2895 | Yes |
| 167 | ITGA6 | integrin subunit alpha 6 [Source:HGNC Symbol;Acc:HGNC:6142] | 37064 | -0.239 | -0.2864 | Yes |
| 168 | THBS1 | thrombospondin 1 [Source:HGNC Symbol;Acc:HGNC:11785] | 37079 | -0.240 | -0.2782 | Yes |
| 169 | FYN | "FYN proto-oncogene, Src family tyrosine kinase [Source:HGNC Symbol;Acc:HGNC:4037]" | 37102 | -0.242 | -0.2703 | Yes |
| 170 | MYLK | myosin light chain kinase [Source:HGNC Symbol;Acc:HGNC:7590] | 37197 | -0.248 | -0.2639 | Yes |
| 171 | COL5A2 | collagen type V alpha 2 chain [Source:HGNC Symbol;Acc:HGNC:2210] | 37272 | -0.252 | -0.2569 | Yes |
| 172 | ITGA1 | integrin subunit alpha 1 [Source:HGNC Symbol;Acc:HGNC:6134] | 37309 | -0.254 | -0.2488 | Yes |
| 173 | RELN | reelin [Source:HGNC Symbol;Acc:HGNC:9957] | 37356 | -0.257 | -0.2409 | Yes |
| 174 | LAMA2 | laminin subunit alpha 2 [Source:HGNC Symbol;Acc:HGNC:6482] | 37457 | -0.263 | -0.2342 | Yes |
| 175 | PIK3CA | "phosphatidylinositol-4,5-bisphosphate 3-kinase catalytic subunit alpha [Source:HGNC Symbol;Acc:HGNC:8975]" | 37556 | -0.270 | -0.2271 | Yes |
| 176 | VWF | von Willebrand factor [Source:HGNC Symbol;Acc:HGNC:12726] | 37581 | -0.272 | -0.2181 | Yes |
| 177 | GSK3B | glycogen synthase kinase 3 beta [Source:HGNC Symbol;Acc:HGNC:4617] | 37592 | -0.274 | -0.2087 | Yes |
| 178 | ITGAV | integrin subunit alpha V [Source:HGNC Symbol;Acc:HGNC:6150] | 37606 | -0.275 | -0.1993 | Yes |
| 179 | COL6A3 | collagen type VI alpha 3 chain [Source:HGNC Symbol;Acc:HGNC:2213] | 37608 | -0.275 | -0.1896 | Yes |
| 180 | PAK2 | p21 (RAC1) activated kinase 2 [Source:HGNC Symbol;Acc:HGNC:8591] | 37627 | -0.276 | -0.1803 | Yes |
| 181 | LAMB1 | laminin subunit beta 1 [Source:HGNC Symbol;Acc:HGNC:6486] | 37639 | -0.278 | -0.1708 | Yes |
| 182 | TLN1 | talin 1 [Source:HGNC Symbol;Acc:HGNC:11845] | 37645 | -0.278 | -0.1611 | Yes |
| 183 | PDGFRA | platelet derived growth factor receptor alpha [Source:HGNC Symbol;Acc:HGNC:8803] | 37662 | -0.280 | -0.1516 | Yes |
| 184 | BCL2 | BCL2 apoptosis regulator [Source:HGNC Symbol;Acc:HGNC:990] | 37702 | -0.283 | -0.1426 | Yes |
| 185 | PRKCB | protein kinase C beta [Source:HGNC Symbol;Acc:HGNC:9395] | 37822 | -0.293 | -0.1353 | Yes |
| 186 | ITGB1 | integrin subunit beta 1 [Source:HGNC Symbol;Acc:HGNC:6153] | 37850 | -0.297 | -0.1255 | Yes |
| 187 | HGF | hepatocyte growth factor [Source:HGNC Symbol;Acc:HGNC:4893] | 37908 | -0.304 | -0.1162 | Yes |
| 188 | PIK3R5 | phosphoinositide-3-kinase regulatory subunit 5 [Source:HGNC Symbol;Acc:HGNC:30035] | 37956 | -0.310 | -0.1064 | Yes |
| 189 | RAPGEF1 | Rap guanine nucleotide exchange factor 1 [Source:HGNC Symbol;Acc:HGNC:4568] | 38009 | -0.318 | -0.0965 | Yes |
| 190 | FLT1 | fms related receptor tyrosine kinase 1 [Source:HGNC Symbol;Acc:HGNC:3763] | 38056 | -0.325 | -0.0862 | Yes |
| 191 | LAMA4 | laminin subunit alpha 4 [Source:HGNC Symbol;Acc:HGNC:6484] | 38106 | -0.335 | -0.0756 | Yes |
| 192 | SOS1 | SOS Ras/Rac guanine nucleotide exchange factor 1 [Source:HGNC Symbol;Acc:HGNC:11187] | 38132 | -0.341 | -0.0642 | Yes |
| 193 | ITGB7 | integrin subunit beta 7 [Source:HGNC Symbol;Acc:HGNC:6162] | 38154 | -0.347 | -0.0525 | Yes |
| 194 | AKT3 | AKT serine/threonine kinase 3 [Source:HGNC Symbol;Acc:HGNC:393] | 38196 | -0.362 | -0.0407 | Yes |
| 195 | ROCK2 | Rho associated coiled-coil containing protein kinase 2 [Source:HGNC Symbol;Acc:HGNC:10252] | 38215 | -0.370 | -0.0281 | Yes |
| 196 | PPP1R12A | protein phosphatase 1 regulatory subunit 12A [Source:HGNC Symbol;Acc:HGNC:7618] | 38258 | -0.387 | -0.0155 | Yes |
| 197 | ROCK1 | Rho associated coiled-coil containing protein kinase 1 [Source:HGNC Symbol;Acc:HGNC:10251] | 38260 | -0.387 | -0.0019 | Yes |
| 198 | ITGA4 | integrin subunit alpha 4 [Source:HGNC Symbol;Acc:HGNC:6140] | 38275 | -0.394 | 0.0117 | Yes |
| 199 | PIK3CG | "phosphatidylinositol-4,5-bisphosphate 3-kinase catalytic subunit gamma [Source:HGNC Symbol;Acc:HGNC:8978]" | 38302 | -0.426 | 0.0261 | Yes |
Table: GSEA details [plain text format]

  

Fig 2: KEGG\_FOCAL\_ADHESION      
 Blue-Pink O' Gram in the Space of the Analyzed GeneSet

  

Fig 3: KEGG\_FOCAL\_ADHESION: Random ES distribution      
 Gene set null distribution of ES for **KEGG\_FOCAL\_ADHESION**

  
