# Supplementary material for: Simultaneous Presentation of Multiple Myeloma and Lung Cancer: Case Report and Gene Bioinformatics Analysis
Source: Front Oncol. 2022 Jun 13;12:859735. doi: 10.3389/fonc.2022.859735 (PMC9235397; doi:10.3389/fonc.2022.859735)
Supplement: Supplementary file 1 [file DataSheet_1.zip › The bioinformatic analysis of MM and lung cancer supplementary materials/Enrichment analysis/MECR/GSEA_4.1.0/LUAD TCGA/KEGG.Gsea.1639041756227/KEGG_GLUTATHIONE_METABOLISM.html]

Details for gene set KEGG\_GLUTATHIONE\_METABOLISM[GSEA]

|  || Dataset | ExpData\_collapsed\_to\_symbols.ENSG00000116353\_profile\_in\_ExpData.cls #ENSG00000116353 |
| Phenotype | ENSG00000116353\_profile\_in\_ExpData.cls#ENSG00000116353 |
| Upregulated in class | ENSG00000116353\_pos |
| GeneSet | KEGG\_GLUTATHIONE\_METABOLISM |
| Enrichment Score (ES) | 0.6331704 |
| Normalized Enrichment Score (NES) | 2.2893744 |
| Nominal p-value | 0.0 |
| FDR q-value | 0.0 |
| FWER p-Value | 0.0 |
Table: GSEA Results Summary

  

Fig 1: Enrichment plot: KEGG\_GLUTATHIONE\_METABOLISM      
 Profile of the Running ES Score & Positions of GeneSet Members on the Rank Ordered List

  

| SYMBOL | TITLE | RANK IN GENE LIST | RANK METRIC SCORE | RUNNING ES | CORE ENRICHMENT || 1 | GPX4 | glutathione peroxidase 4 [Source:HGNC Symbol;Acc:HGNC:4556] | 37 | 0.442 | 0.0726 | Yes |
| 2 | SRM | spermidine synthase [Source:HGNC Symbol;Acc:HGNC:11296] | 367 | 0.338 | 0.1204 | Yes |
| 3 | GPX1 | glutathione peroxidase 1 [Source:HGNC Symbol;Acc:HGNC:4553] | 572 | 0.311 | 0.1669 | Yes |
| 4 | GSTK1 | glutathione S-transferase kappa 1 [Source:HGNC Symbol;Acc:HGNC:16906] | 629 | 0.305 | 0.2162 | Yes |
| 5 | MGST1 | microsomal glutathione S-transferase 1 [Source:HGNC Symbol;Acc:HGNC:7061] | 796 | 0.288 | 0.2599 | Yes |
| 6 | GSTA5 | glutathione S-transferase alpha 5 [Source:HGNC Symbol;Acc:HGNC:19662] | 1236 | 0.252 | 0.2907 | Yes |
| 7 | GSS | glutathione synthetase [Source:HGNC Symbol;Acc:HGNC:4624] | 1273 | 0.250 | 0.3314 | Yes |
| 8 | GSTP1 | glutathione S-transferase pi 1 [Source:HGNC Symbol;Acc:HGNC:4638] | 1346 | 0.245 | 0.3703 | Yes |
| 9 | GSTZ1 | glutathione S-transferase zeta 1 [Source:HGNC Symbol;Acc:HGNC:4643] | 1423 | 0.240 | 0.4082 | Yes |
| 10 | GGCT | gamma-glutamylcyclotransferase [Source:HGNC Symbol;Acc:HGNC:21705] | 1669 | 0.225 | 0.4394 | Yes |
| 11 | MGST2 | microsomal glutathione S-transferase 2 [Source:HGNC Symbol;Acc:HGNC:7063] | 2011 | 0.205 | 0.4648 | Yes |
| 12 | TXNDC12 | thioredoxin domain containing 12 [Source:HGNC Symbol;Acc:HGNC:24626] | 2022 | 0.205 | 0.4987 | Yes |
| 13 | OPLAH | "5-oxoprolinase, ATP-hydrolysing [Source:HGNC Symbol;Acc:HGNC:8149]" | 2628 | 0.179 | 0.5130 | Yes |
| 14 | PGD | phosphogluconate dehydrogenase [Source:HGNC Symbol;Acc:HGNC:8891] | 2772 | 0.173 | 0.5381 | Yes |
| 15 | GPX3 | glutathione peroxidase 3 [Source:HGNC Symbol;Acc:HGNC:4555] | 2870 | 0.169 | 0.5638 | Yes |
| 16 | MGST3 | microsomal glutathione S-transferase 3 [Source:HGNC Symbol;Acc:HGNC:7064] | 3158 | 0.159 | 0.5829 | Yes |
| 17 | GGT6 | gamma-glutamyltransferase 6 [Source:HGNC Symbol;Acc:HGNC:26891] | 3980 | 0.134 | 0.5843 | Yes |
| 18 | GSTO1 | glutathione S-transferase omega 1 [Source:HGNC Symbol;Acc:HGNC:13312] | 4874 | 0.113 | 0.5804 | Yes |
| 19 | GSTM2 | glutathione S-transferase mu 2 [Source:HGNC Symbol;Acc:HGNC:4634] | 4963 | 0.112 | 0.5968 | Yes |
| 20 | GSTO2 | glutathione S-transferase omega 2 [Source:HGNC Symbol;Acc:HGNC:23064] | 5147 | 0.109 | 0.6102 | Yes |
| 21 | IDH2 | isocitrate dehydrogenase (NADP(+)) 2 [Source:HGNC Symbol;Acc:HGNC:5383] | 5934 | 0.095 | 0.6059 | Yes |
| 22 | GSTA4 | glutathione S-transferase alpha 4 [Source:HGNC Symbol;Acc:HGNC:4629] | 6058 | 0.093 | 0.6182 | Yes |
| 23 | G6PD | glucose-6-phosphate dehydrogenase [Source:HGNC Symbol;Acc:HGNC:4057] | 6075 | 0.092 | 0.6332 | Yes |
| 24 | IDH1 | isocitrate dehydrogenase (NADP(+)) 1 [Source:HGNC Symbol;Acc:HGNC:5382] | 7478 | 0.073 | 0.6097 | No |
| 25 | GGT1 | gamma-glutamyltransferase 1 [Source:HGNC Symbol;Acc:HGNC:4250] | 8690 | 0.060 | 0.5889 | No |
| 26 | GPX5 | glutathione peroxidase 5 [Source:HGNC Symbol;Acc:HGNC:4557] | 9231 | 0.055 | 0.5843 | No |
| 27 | GSTT2 | glutathione S-transferase theta 2 (gene/pseudogene) [Source:HGNC Symbol;Acc:HGNC:4642] | 9678 | 0.051 | 0.5815 | No |
| 28 | ANPEP | "alanyl aminopeptidase, membrane [Source:HGNC Symbol;Acc:HGNC:500]" | 9759 | 0.051 | 0.5879 | No |
| 29 | GSR | glutathione-disulfide reductase [Source:HGNC Symbol;Acc:HGNC:4623] | 10763 | 0.043 | 0.5695 | No |
| 30 | ODC1 | ornithine decarboxylase 1 [Source:HGNC Symbol;Acc:HGNC:8109] | 10765 | 0.043 | 0.5766 | No |
| 31 | GSTA3 | glutathione S-transferase alpha 3 [Source:HGNC Symbol;Acc:HGNC:4628] | 11293 | 0.039 | 0.5696 | No |
| 32 | GGT7 | gamma-glutamyltransferase 7 [Source:HGNC Symbol;Acc:HGNC:4259] | 12487 | 0.030 | 0.5441 | No |
| 33 | GSTM3 | glutathione S-transferase mu 3 [Source:HGNC Symbol;Acc:HGNC:4635] | 16443 | 0.004 | 0.4442 | No |
| 34 | GSTM4 | glutathione S-transferase mu 4 [Source:HGNC Symbol;Acc:HGNC:4636] | 17953 | -0.005 | 0.4065 | No |
| 35 | GPX7 | glutathione peroxidase 7 [Source:HGNC Symbol;Acc:HGNC:4559] | 18280 | -0.006 | 0.3993 | No |
| 36 | GCLM | glutamate-cysteine ligase modifier subunit [Source:HGNC Symbol;Acc:HGNC:4312] | 18457 | -0.007 | 0.3961 | No |
| 37 | GGT5 | gamma-glutamyltransferase 5 [Source:HGNC Symbol;Acc:HGNC:4260] | 18515 | -0.008 | 0.3959 | No |
| 38 | RRM2B | ribonucleotide reductase regulatory TP53 inducible subunit M2B [Source:HGNC Symbol;Acc:HGNC:17296] | 19336 | -0.013 | 0.3772 | No |
| 39 | GCLC | glutamate-cysteine ligase catalytic subunit [Source:HGNC Symbol;Acc:HGNC:4311] | 19721 | -0.015 | 0.3699 | No |
| 40 | GSTM1 | glutathione S-transferase mu 1 [Source:HGNC Symbol;Acc:HGNC:4632] | 20208 | -0.018 | 0.3605 | No |
| 41 | SMS | spermine synthase [Source:HGNC Symbol;Acc:HGNC:11123] | 20548 | -0.020 | 0.3552 | No |
| 42 | GPX6 | glutathione peroxidase 6 [Source:HGNC Symbol;Acc:HGNC:4558] | 22293 | -0.031 | 0.3159 | No |
| 43 | GSTA1 | glutathione S-transferase alpha 1 [Source:HGNC Symbol;Acc:HGNC:4626] | 22460 | -0.032 | 0.3169 | No |
| 44 | GPX2 | glutathione peroxidase 2 [Source:HGNC Symbol;Acc:HGNC:4554] | 25030 | -0.049 | 0.2596 | No |
| 45 | GSTA2 | glutathione S-transferase alpha 2 [Source:HGNC Symbol;Acc:HGNC:4627] | 25276 | -0.051 | 0.2618 | No |
| 46 | GSTM5 | glutathione S-transferase mu 5 [Source:HGNC Symbol;Acc:HGNC:4637] | 28592 | -0.076 | 0.1901 | No |
| 47 | RRM2 | ribonucleotide reductase regulatory subunit M2 [Source:HGNC Symbol;Acc:HGNC:10452] | 33676 | -0.139 | 0.0838 | No |
| 48 | RRM1 | ribonucleotide reductase catalytic subunit M1 [Source:HGNC Symbol;Acc:HGNC:10451] | 33985 | -0.145 | 0.1001 | No |
| 49 | LAP3 | leucine aminopeptidase 3 [Source:HGNC Symbol;Acc:HGNC:18449] | 36597 | -0.215 | 0.0694 | No |
Table: GSEA details [plain text format]

  

Fig 2: KEGG\_GLUTATHIONE\_METABOLISM      
 Blue-Pink O' Gram in the Space of the Analyzed GeneSet

  

Fig 3: KEGG\_GLUTATHIONE\_METABOLISM: Random ES distribution      
 Gene set null distribution of ES for **KEGG\_GLUTATHIONE\_METABOLISM**

  
