# Supplementary material for: Simultaneous Presentation of Multiple Myeloma and Lung Cancer: Case Report and Gene Bioinformatics Analysis
Source: Front Oncol. 2022 Jun 13;12:859735. doi: 10.3389/fonc.2022.859735 (PMC9235397; doi:10.3389/fonc.2022.859735)
Supplement: Supplementary file 1 [file DataSheet_1.zip › The bioinformatic analysis of MM and lung cancer supplementary materials/Enrichment analysis/MECR/GSEA_4.1.0/LUAD TCGA/KEGG.Gsea.1639041756227/KEGG_GLYCOSYLPHOSPHATIDYLINOSITOL_GPI_ANCHOR_BIOSYNTHESIS.html]

Details for gene set KEGG\_GLYCOSYLPHOSPHATIDYLINOSITOL\_GPI\_ANCHOR\_BIOSYNTHESIS[GSEA]

|  || Dataset | ExpData\_collapsed\_to\_symbols.ENSG00000116353\_profile\_in\_ExpData.cls #ENSG00000116353 |
| Phenotype | ENSG00000116353\_profile\_in\_ExpData.cls#ENSG00000116353 |
| Upregulated in class | ENSG00000116353\_pos |
| GeneSet | KEGG\_GLYCOSYLPHOSPHATIDYLINOSITOL\_GPI\_ANCHOR\_BIOSYNTHESIS |
| Enrichment Score (ES) | 0.57164437 |
| Normalized Enrichment Score (NES) | 1.7633643 |
| Nominal p-value | 0.0061099795 |
| FDR q-value | 0.010492252 |
| FWER p-Value | 0.202 |
Table: GSEA Results Summary

  

Fig 1: Enrichment plot: KEGG\_GLYCOSYLPHOSPHATIDYLINOSITOL\_GPI\_ANCHOR\_BIOSYNTHESIS      
 Profile of the Running ES Score & Positions of GeneSet Members on the Rank Ordered List

  

| SYMBOL | TITLE | RANK IN GENE LIST | RANK METRIC SCORE | RUNNING ES | CORE ENRICHMENT || 1 | PIGV | phosphatidylinositol glycan anchor biosynthesis class V [Source:HGNC Symbol;Acc:HGNC:26031] | 49 | 0.434 | 0.1171 | Yes |
| 2 | DPM2 | "dolichyl-phosphate mannosyltransferase subunit 2, regulatory [Source:HGNC Symbol;Acc:HGNC:3006]" | 134 | 0.388 | 0.2208 | Yes |
| 3 | PIGQ | phosphatidylinositol glycan anchor biosynthesis class Q [Source:HGNC Symbol;Acc:HGNC:14135] | 247 | 0.361 | 0.3165 | Yes |
| 4 | GPAA1 | glycosylphosphatidylinositol anchor attachment 1 [Source:HGNC Symbol;Acc:HGNC:4446] | 261 | 0.358 | 0.4139 | Yes |
| 5 | PIGP | phosphatidylinositol glycan anchor biosynthesis class P [Source:HGNC Symbol;Acc:HGNC:3046] | 1000 | 0.270 | 0.4687 | Yes |
| 6 | PIGU | phosphatidylinositol glycan anchor biosynthesis class U [Source:HGNC Symbol;Acc:HGNC:15791] | 1417 | 0.240 | 0.5237 | Yes |
| 7 | PIGT | phosphatidylinositol glycan anchor biosynthesis class T [Source:HGNC Symbol;Acc:HGNC:14938] | 2346 | 0.191 | 0.5522 | Yes |
| 8 | PIGC | phosphatidylinositol glycan anchor biosynthesis class C [Source:HGNC Symbol;Acc:HGNC:8960] | 3253 | 0.156 | 0.5716 | Yes |
| 9 | PIGL | phosphatidylinositol glycan anchor biosynthesis class L [Source:HGNC Symbol;Acc:HGNC:8966] | 4649 | 0.118 | 0.5684 | No |
| 10 | PIGF | phosphatidylinositol glycan anchor biosynthesis class F [Source:HGNC Symbol;Acc:HGNC:8962] | 5646 | 0.100 | 0.5702 | No |
| 11 | PIGZ | phosphatidylinositol glycan anchor biosynthesis class Z [Source:HGNC Symbol;Acc:HGNC:30596] | 7218 | 0.076 | 0.5511 | No |
| 12 | PIGM | phosphatidylinositol glycan anchor biosynthesis class M [Source:HGNC Symbol;Acc:HGNC:18858] | 8501 | 0.062 | 0.5354 | No |
| 13 | PIGG | phosphatidylinositol glycan anchor biosynthesis class G [Source:HGNC Symbol;Acc:HGNC:25985] | 10934 | 0.041 | 0.4849 | No |
| 14 | PIGW | phosphatidylinositol glycan anchor biosynthesis class W [Source:HGNC Symbol;Acc:HGNC:23213] | 13899 | 0.020 | 0.4149 | No |
| 15 | PIGA | phosphatidylinositol glycan anchor biosynthesis class A [Source:HGNC Symbol;Acc:HGNC:8957] | 14013 | 0.019 | 0.4173 | No |
| 16 | PIGK | phosphatidylinositol glycan anchor biosynthesis class K [Source:HGNC Symbol;Acc:HGNC:8965] | 14940 | 0.013 | 0.3974 | No |
| 17 | PIGH | phosphatidylinositol glycan anchor biosynthesis class H [Source:HGNC Symbol;Acc:HGNC:8964] | 18571 | -0.008 | 0.3073 | No |
| 18 | PIGB | phosphatidylinositol glycan anchor biosynthesis class B [Source:HGNC Symbol;Acc:HGNC:8959] | 20737 | -0.021 | 0.2579 | No |
| 19 | PIGX | phosphatidylinositol glycan anchor biosynthesis class X [Source:HGNC Symbol;Acc:HGNC:26046] | 25598 | -0.053 | 0.1487 | No |
| 20 | GPLD1 | glycosylphosphatidylinositol specific phospholipase D1 [Source:HGNC Symbol;Acc:HGNC:4459] | 26597 | -0.060 | 0.1397 | No |
| 21 | PIGN | phosphatidylinositol glycan anchor biosynthesis class N [Source:HGNC Symbol;Acc:HGNC:8967] | 28826 | -0.078 | 0.1044 | No |
| 22 | PIGS | phosphatidylinositol glycan anchor biosynthesis class S [Source:HGNC Symbol;Acc:HGNC:14937] | 32914 | -0.127 | 0.0349 | No |
| 23 | PIGO | phosphatidylinositol glycan anchor biosynthesis class O [Source:HGNC Symbol;Acc:HGNC:23215] | 36630 | -0.217 | -0.0004 | No |
| 24 | PGAP1 | post-GPI attachment to proteins inositol deacylase 1 [Source:HGNC Symbol;Acc:HGNC:25712] | 37114 | -0.242 | 0.0535 | No |
| 25 | PIGY | phosphatidylinositol glycan anchor biosynthesis class Y [Source:HGNC Symbol;Acc:HGNC:28213] | 38850 | NaN | 0.0120 | No |
Table: GSEA details [plain text format]

  

Fig 2: KEGG\_GLYCOSYLPHOSPHATIDYLINOSITOL\_GPI\_ANCHOR\_BIOSYNTHESIS      
 Blue-Pink O' Gram in the Space of the Analyzed GeneSet

  

Fig 3: KEGG\_GLYCOSYLPHOSPHATIDYLINOSITOL\_GPI\_ANCHOR\_BIOSYNTHESIS: Random ES distribution      
 Gene set null distribution of ES for **KEGG\_GLYCOSYLPHOSPHATIDYLINOSITOL\_GPI\_ANCHOR\_BIOSYNTHESIS**

  
