# Supplementary material for: Simultaneous Presentation of Multiple Myeloma and Lung Cancer: Case Report and Gene Bioinformatics Analysis
Source: Front Oncol. 2022 Jun 13;12:859735. doi: 10.3389/fonc.2022.859735 (PMC9235397; doi:10.3389/fonc.2022.859735)
Supplement: Supplementary file 1 [file DataSheet_1.zip › The bioinformatic analysis of MM and lung cancer supplementary materials/Enrichment analysis/MECR/GSEA_4.1.0/LUAD TCGA/KEGG.Gsea.1639041756227/KEGG_GRAFT_VERSUS_HOST_DISEASE.html]

Details for gene set KEGG\_GRAFT\_VERSUS\_HOST\_DISEASE[GSEA]

|  || Dataset | ExpData\_collapsed\_to\_symbols.ENSG00000116353\_profile\_in\_ExpData.cls #ENSG00000116353 |
| Phenotype | ENSG00000116353\_profile\_in\_ExpData.cls#ENSG00000116353 |
| Upregulated in class | ENSG00000116353\_neg |
| GeneSet | KEGG\_GRAFT\_VERSUS\_HOST\_DISEASE |
| Enrichment Score (ES) | -0.6518434 |
| Normalized Enrichment Score (NES) | -2.2767832 |
| Nominal p-value | 0.0 |
| FDR q-value | 0.0 |
| FWER p-Value | 0.0 |
Table: GSEA Results Summary

  

Fig 1: Enrichment plot: KEGG\_GRAFT\_VERSUS\_HOST\_DISEASE      
 Profile of the Running ES Score & Positions of GeneSet Members on the Rank Ordered List

  

| SYMBOL | TITLE | RANK IN GENE LIST | RANK METRIC SCORE | RUNNING ES | CORE ENRICHMENT || 1 | HLA-DMA | "major histocompatibility complex, class II, DM alpha [Source:HGNC Symbol;Acc:HGNC:4934]" | 4341 | 0.126 | -0.0818 | No |
| 2 | HLA-A | "major histocompatibility complex, class I, A [Source:HGNC Symbol;Acc:HGNC:4931]" | 4980 | 0.111 | -0.0726 | No |
| 3 | HLA-F | "major histocompatibility complex, class I, F [Source:HGNC Symbol;Acc:HGNC:4963]" | 7370 | 0.075 | -0.1163 | No |
| 4 | HLA-C | "major histocompatibility complex, class I, C [Source:HGNC Symbol;Acc:HGNC:4933]" | 9612 | 0.052 | -0.1615 | No |
| 5 | HLA-DRB1 | "major histocompatibility complex, class II, DR beta 1 [Source:HGNC Symbol;Acc:HGNC:4948]" | 10444 | 0.045 | -0.1723 | No |
| 6 | HLA-DRB5 | "major histocompatibility complex, class II, DR beta 5 [Source:HGNC Symbol;Acc:HGNC:4953]" | 10518 | 0.045 | -0.1639 | No |
| 7 | HLA-G | "major histocompatibility complex, class I, G [Source:HGNC Symbol;Acc:HGNC:4964]" | 12279 | 0.031 | -0.2016 | No |
| 8 | HLA-B | "major histocompatibility complex, class I, B [Source:HGNC Symbol;Acc:HGNC:4932]" | 13305 | 0.024 | -0.2223 | No |
| 9 | HLA-DPB1 | "major histocompatibility complex, class II, DP beta 1 [Source:HGNC Symbol;Acc:HGNC:4940]" | 16356 | 0.005 | -0.2988 | No |
| 10 | HLA-DRA | "major histocompatibility complex, class II, DR alpha [Source:HGNC Symbol;Acc:HGNC:4947]" | 17663 | -0.003 | -0.3314 | No |
| 11 | HLA-DQB1 | "major histocompatibility complex, class II, DQ beta 1 [Source:HGNC Symbol;Acc:HGNC:4944]" | 17913 | -0.004 | -0.3367 | No |
| 12 | HLA-DQA2 | "major histocompatibility complex, class II, DQ alpha 2 [Source:HGNC Symbol;Acc:HGNC:4943]" | 19230 | -0.012 | -0.3674 | No |
| 13 | HLA-DOB | "major histocompatibility complex, class II, DO beta [Source:HGNC Symbol;Acc:HGNC:4937]" | 20731 | -0.021 | -0.4008 | No |
| 14 | IL2 | interleukin 2 [Source:HGNC Symbol;Acc:HGNC:6001] | 20777 | -0.021 | -0.3970 | No |
| 15 | HLA-E | "major histocompatibility complex, class I, E [Source:HGNC Symbol;Acc:HGNC:4962]" | 21659 | -0.027 | -0.4133 | No |
| 16 | FAS | Fas cell surface death receptor [Source:HGNC Symbol;Acc:HGNC:11920] | 23994 | -0.042 | -0.4632 | No |
| 17 | HLA-DPA1 | "major histocompatibility complex, class II, DP alpha 1 [Source:HGNC Symbol;Acc:HGNC:4938]" | 24356 | -0.044 | -0.4623 | No |
| 18 | HLA-DMB | "major histocompatibility complex, class II, DM beta [Source:HGNC Symbol;Acc:HGNC:4935]" | 24935 | -0.048 | -0.4660 | No |
| 19 | KLRC1 | killer cell lectin like receptor C1 [Source:HGNC Symbol;Acc:HGNC:6374] | 28252 | -0.073 | -0.5336 | No |
| 20 | HLA-DQA1 | "major histocompatibility complex, class II, DQ alpha 1 [Source:HGNC Symbol;Acc:HGNC:4942]" | 32898 | -0.126 | -0.6230 | Yes |
| 21 | KIR2DL1 | "killer cell immunoglobulin like receptor, two Ig domains and long cytoplasmic tail 1 [Source:HGNC Symbol;Acc:HGNC:6329]" | 32995 | -0.128 | -0.5962 | Yes |
| 22 | HLA-DOA | "major histocompatibility complex, class II, DO alpha [Source:HGNC Symbol;Acc:HGNC:4936]" | 33498 | -0.136 | -0.5778 | Yes |
| 23 | KIR2DL3 | "killer cell immunoglobulin like receptor, two Ig domains and long cytoplasmic tail 3 [Source:HGNC Symbol;Acc:HGNC:6331]" | 33593 | -0.138 | -0.5487 | Yes |
| 24 | TNF | tumor necrosis factor [Source:HGNC Symbol;Acc:HGNC:11892] | 33732 | -0.140 | -0.5202 | Yes |
| 25 | IL1A | interleukin 1 alpha [Source:HGNC Symbol;Acc:HGNC:5991] | 34080 | -0.147 | -0.4954 | Yes |
| 26 | KIR3DL1 | "killer cell immunoglobulin like receptor, three Ig domains and long cytoplasmic tail 1 [Source:HGNC Symbol;Acc:HGNC:6338]" | 34105 | -0.147 | -0.4623 | Yes |
| 27 | PRF1 | perforin 1 [Source:HGNC Symbol;Acc:HGNC:9360] | 35249 | -0.172 | -0.4520 | Yes |
| 28 | IFNG | interferon gamma [Source:HGNC Symbol;Acc:HGNC:5438] | 35652 | -0.183 | -0.4203 | Yes |
| 29 | FASLG | Fas ligand [Source:HGNC Symbol;Acc:HGNC:11936] | 36345 | -0.206 | -0.3908 | Yes |
| 30 | IL6 | interleukin 6 [Source:HGNC Symbol;Acc:HGNC:6018] | 36396 | -0.208 | -0.3446 | Yes |
| 31 | KIR3DL2 | "killer cell immunoglobulin like receptor, three Ig domains and long cytoplasmic tail 2 [Source:HGNC Symbol;Acc:HGNC:6339]" | 36649 | -0.218 | -0.3012 | Yes |
| 32 | GZMB | granzyme B [Source:HGNC Symbol;Acc:HGNC:4709] | 37092 | -0.241 | -0.2574 | Yes |
| 33 | IL1B | interleukin 1 beta [Source:HGNC Symbol;Acc:HGNC:5992] | 37201 | -0.248 | -0.2034 | Yes |
| 34 | KLRD1 | killer cell lectin like receptor D1 [Source:HGNC Symbol;Acc:HGNC:6378] | 37615 | -0.276 | -0.1509 | Yes |
| 35 | CD86 | CD86 molecule [Source:HGNC Symbol;Acc:HGNC:1705] | 37625 | -0.276 | -0.0880 | Yes |
| 36 | CD80 | CD80 molecule [Source:HGNC Symbol;Acc:HGNC:1700] | 37726 | -0.285 | -0.0253 | Yes |
| 37 | CD28 | CD28 molecule [Source:HGNC Symbol;Acc:HGNC:1653] | 37779 | -0.289 | 0.0393 | Yes |
Table: GSEA details [plain text format]

  

Fig 2: KEGG\_GRAFT\_VERSUS\_HOST\_DISEASE      
 Blue-Pink O' Gram in the Space of the Analyzed GeneSet

  

Fig 3: KEGG\_GRAFT\_VERSUS\_HOST\_DISEASE: Random ES distribution      
 Gene set null distribution of ES for **KEGG\_GRAFT\_VERSUS\_HOST\_DISEASE**

  
