# Supplementary material for: Simultaneous Presentation of Multiple Myeloma and Lung Cancer: Case Report and Gene Bioinformatics Analysis
Source: Front Oncol. 2022 Jun 13;12:859735. doi: 10.3389/fonc.2022.859735 (PMC9235397; doi:10.3389/fonc.2022.859735)
Supplement: Supplementary file 1 [file DataSheet_1.zip › The bioinformatic analysis of MM and lung cancer supplementary materials/Enrichment analysis/MECR/GSEA_4.1.0/LUAD TCGA/KEGG.Gsea.1639041756227/KEGG_HEMATOPOIETIC_CELL_LINEAGE.html]

Details for gene set KEGG\_HEMATOPOIETIC\_CELL\_LINEAGE[GSEA]

|  || Dataset | ExpData\_collapsed\_to\_symbols.ENSG00000116353\_profile\_in\_ExpData.cls #ENSG00000116353 |
| Phenotype | ENSG00000116353\_profile\_in\_ExpData.cls#ENSG00000116353 |
| Upregulated in class | ENSG00000116353\_neg |
| GeneSet | KEGG\_HEMATOPOIETIC\_CELL\_LINEAGE |
| Enrichment Score (ES) | -0.6063363 |
| Normalized Enrichment Score (NES) | -2.443159 |
| Nominal p-value | 0.0 |
| FDR q-value | 0.0 |
| FWER p-Value | 0.0 |
Table: GSEA Results Summary

  

Fig 1: Enrichment plot: KEGG\_HEMATOPOIETIC\_CELL\_LINEAGE      
 Profile of the Running ES Score & Positions of GeneSet Members on the Rank Ordered List

  

| SYMBOL | TITLE | RANK IN GENE LIST | RANK METRIC SCORE | RUNNING ES | CORE ENRICHMENT || 1 | IL11RA | interleukin 11 receptor subunit alpha [Source:HGNC Symbol;Acc:HGNC:5967] | 2627 | 0.179 | -0.0523 | No |
| 2 | ITGA3 | integrin subunit alpha 3 [Source:HGNC Symbol;Acc:HGNC:6139] | 3614 | 0.145 | -0.0656 | No |
| 3 | EPOR | erythropoietin receptor [Source:HGNC Symbol;Acc:HGNC:3416] | 3751 | 0.141 | -0.0576 | No |
| 4 | CD9 | CD9 molecule [Source:HGNC Symbol;Acc:HGNC:1709] | 4045 | 0.132 | -0.0542 | No |
| 5 | EPO | erythropoietin [Source:HGNC Symbol;Acc:HGNC:3415] | 6062 | 0.093 | -0.0980 | No |
| 6 | CD59 | CD59 molecule (CD59 blood group) [Source:HGNC Symbol;Acc:HGNC:1689] | 6549 | 0.085 | -0.1035 | No |
| 7 | TPO | thyroid peroxidase [Source:HGNC Symbol;Acc:HGNC:12015] | 6966 | 0.080 | -0.1075 | No |
| 8 | CD1A | CD1a molecule [Source:HGNC Symbol;Acc:HGNC:1634] | 7129 | 0.078 | -0.1053 | No |
| 9 | IL4 | interleukin 4 [Source:HGNC Symbol;Acc:HGNC:6014] | 7378 | 0.075 | -0.1055 | No |
| 10 | CD55 | CD55 molecule (Cromer blood group) [Source:HGNC Symbol;Acc:HGNC:2665] | 7403 | 0.074 | -0.1001 | No |
| 11 | IL3 | interleukin 3 [Source:HGNC Symbol;Acc:HGNC:6011] | 8074 | 0.067 | -0.1117 | No |
| 12 | IL6R | interleukin 6 receptor [Source:HGNC Symbol;Acc:HGNC:6019] | 8091 | 0.067 | -0.1067 | No |
| 13 | ITGA2B | integrin subunit alpha 2b [Source:HGNC Symbol;Acc:HGNC:6138] | 9230 | 0.055 | -0.1311 | No |
| 14 | CD1C | CD1c molecule [Source:HGNC Symbol;Acc:HGNC:1636] | 9521 | 0.053 | -0.1342 | No |
| 15 | ANPEP | "alanyl aminopeptidase, membrane [Source:HGNC Symbol;Acc:HGNC:500]" | 9759 | 0.051 | -0.1361 | No |
| 16 | CSF2 | colony stimulating factor 2 [Source:HGNC Symbol;Acc:HGNC:2434] | 9888 | 0.050 | -0.1353 | No |
| 17 | CD1E | CD1e molecule [Source:HGNC Symbol;Acc:HGNC:1638] | 9987 | 0.049 | -0.1338 | No |
| 18 | HLA-DRB1 | "major histocompatibility complex, class II, DR beta 1 [Source:HGNC Symbol;Acc:HGNC:4948]" | 10444 | 0.045 | -0.1417 | No |
| 19 | HLA-DRB5 | "major histocompatibility complex, class II, DR beta 5 [Source:HGNC Symbol;Acc:HGNC:4953]" | 10518 | 0.045 | -0.1400 | No |
| 20 | FLT3LG | fms related receptor tyrosine kinase 3 ligand [Source:HGNC Symbol;Acc:HGNC:3766] | 13668 | 0.021 | -0.2185 | No |
| 21 | GP9 | glycoprotein IX platelet [Source:HGNC Symbol;Acc:HGNC:4444] | 13795 | 0.021 | -0.2200 | No |
| 22 | CD1B | CD1b molecule [Source:HGNC Symbol;Acc:HGNC:1635] | 15033 | 0.013 | -0.2505 | No |
| 23 | GYPA | glycophorin A (MNS blood group) [Source:HGNC Symbol;Acc:HGNC:4702] | 16677 | 0.003 | -0.2921 | No |
| 24 | HLA-DRA | "major histocompatibility complex, class II, DR alpha [Source:HGNC Symbol;Acc:HGNC:4947]" | 17663 | -0.003 | -0.3170 | No |
| 25 | IL4R | interleukin 4 receptor [Source:HGNC Symbol;Acc:HGNC:6015] | 21312 | -0.025 | -0.4079 | No |
| 26 | IL11 | interleukin 11 [Source:HGNC Symbol;Acc:HGNC:5966] | 23228 | -0.037 | -0.4537 | No |
| 27 | IL5 | interleukin 5 [Source:HGNC Symbol;Acc:HGNC:6016] | 25260 | -0.050 | -0.5013 | No |
| 28 | CSF2RA | colony stimulating factor 2 receptor subunit alpha [Source:HGNC Symbol;Acc:HGNC:2435] | 25630 | -0.053 | -0.5064 | No |
| 29 | CD44 | CD44 molecule (Indian blood group) [Source:HGNC Symbol;Acc:HGNC:1681] | 28518 | -0.076 | -0.5738 | No |
| 30 | THPO | thrombopoietin [Source:HGNC Symbol;Acc:HGNC:11795] | 28687 | -0.077 | -0.5718 | No |
| 31 | CSF1 | colony stimulating factor 1 [Source:HGNC Symbol;Acc:HGNC:2432] | 29384 | -0.083 | -0.5827 | No |
| 32 | MME | membrane metalloendopeptidase [Source:HGNC Symbol;Acc:HGNC:7154] | 29524 | -0.085 | -0.5793 | No |
| 33 | CD7 | CD7 molecule [Source:HGNC Symbol;Acc:HGNC:1695] | 30419 | -0.094 | -0.5944 | No |
| 34 | FCER2 | Fc fragment of IgE receptor II [Source:HGNC Symbol;Acc:HGNC:3612] | 30496 | -0.095 | -0.5886 | No |
| 35 | CR2 | complement C3d receptor 2 [Source:HGNC Symbol;Acc:HGNC:2336] | 30834 | -0.099 | -0.5891 | No |
| 36 | KIT | "KIT proto-oncogene, receptor tyrosine kinase [Source:HGNC Symbol;Acc:HGNC:6342]" | 31394 | -0.105 | -0.5948 | No |
| 37 | CD34 | CD34 molecule [Source:HGNC Symbol;Acc:HGNC:1662] | 31546 | -0.107 | -0.5898 | No |
| 38 | CD38 | CD38 molecule [Source:HGNC Symbol;Acc:HGNC:1667] | 31649 | -0.108 | -0.5836 | No |
| 39 | ITGB3 | integrin subunit beta 3 [Source:HGNC Symbol;Acc:HGNC:6156] | 32139 | -0.115 | -0.5867 | No |
| 40 | IL3RA | interleukin 3 receptor subunit alpha [Source:HGNC Symbol;Acc:HGNC:6012] | 32493 | -0.120 | -0.5858 | No |
| 41 | KITLG | KIT ligand [Source:HGNC Symbol;Acc:HGNC:6343] | 33298 | -0.133 | -0.5955 | Yes |
| 42 | IL5RA | interleukin 5 receptor subunit alpha [Source:HGNC Symbol;Acc:HGNC:6017] | 33434 | -0.135 | -0.5879 | Yes |
| 43 | CSF3 | colony stimulating factor 3 [Source:HGNC Symbol;Acc:HGNC:2438] | 33514 | -0.137 | -0.5787 | Yes |
| 44 | CD14 | CD14 molecule [Source:HGNC Symbol;Acc:HGNC:1628] | 33530 | -0.137 | -0.5679 | Yes |
| 45 | IL7 | interleukin 7 [Source:HGNC Symbol;Acc:HGNC:6023] | 33655 | -0.139 | -0.5597 | Yes |
| 46 | TNF | tumor necrosis factor [Source:HGNC Symbol;Acc:HGNC:11892] | 33732 | -0.140 | -0.5502 | Yes |
| 47 | CD22 | CD22 molecule [Source:HGNC Symbol;Acc:HGNC:1643] | 33891 | -0.143 | -0.5425 | Yes |
| 48 | GP1BA | glycoprotein Ib platelet subunit alpha [Source:HGNC Symbol;Acc:HGNC:4439] | 33922 | -0.144 | -0.5315 | Yes |
| 49 | IL1A | interleukin 1 alpha [Source:HGNC Symbol;Acc:HGNC:5991] | 34080 | -0.147 | -0.5235 | Yes |
| 50 | IL9R | interleukin 9 receptor [Source:HGNC Symbol;Acc:HGNC:6030] | 34282 | -0.151 | -0.5163 | Yes |
| 51 | CD37 | CD37 molecule [Source:HGNC Symbol;Acc:HGNC:1666] | 34576 | -0.157 | -0.5110 | Yes |
| 52 | FCGR1A | Fc fragment of IgG receptor Ia [Source:HGNC Symbol;Acc:HGNC:3613] | 34659 | -0.159 | -0.5001 | Yes |
| 53 | DNTT | DNA nucleotidylexotransferase [Source:HGNC Symbol;Acc:HGNC:2983] | 34665 | -0.159 | -0.4872 | Yes |
| 54 | CD33 | CD33 molecule [Source:HGNC Symbol;Acc:HGNC:1659] | 34729 | -0.160 | -0.4757 | Yes |
| 55 | ITGA5 | integrin subunit alpha 5 [Source:HGNC Symbol;Acc:HGNC:6141] | 34763 | -0.161 | -0.4634 | Yes |
| 56 | CD3D | CD3d molecule [Source:HGNC Symbol;Acc:HGNC:1673] | 35025 | -0.167 | -0.4564 | Yes |
| 57 | MS4A1 | membrane spanning 4-domains A1 [Source:HGNC Symbol;Acc:HGNC:7315] | 35653 | -0.183 | -0.4574 | Yes |
| 58 | CD2 | CD2 molecule [Source:HGNC Symbol;Acc:HGNC:1639] | 35724 | -0.185 | -0.4441 | Yes |
| 59 | CD36 | CD36 molecule [Source:HGNC Symbol;Acc:HGNC:1663] | 35748 | -0.186 | -0.4294 | Yes |
| 60 | CD3E | CD3e molecule [Source:HGNC Symbol;Acc:HGNC:1674] | 35751 | -0.186 | -0.4143 | Yes |
| 61 | ITGA2 | integrin subunit alpha 2 [Source:HGNC Symbol;Acc:HGNC:6137] | 35833 | -0.189 | -0.4009 | Yes |
| 62 | CD19 | CD19 molecule [Source:HGNC Symbol;Acc:HGNC:1633] | 35881 | -0.190 | -0.3866 | Yes |
| 63 | CD1D | CD1d molecule [Source:HGNC Symbol;Acc:HGNC:1637] | 36101 | -0.197 | -0.3760 | Yes |
| 64 | CD8B | CD8b molecule [Source:HGNC Symbol;Acc:HGNC:1707] | 36153 | -0.199 | -0.3611 | Yes |
| 65 | CSF3R | colony stimulating factor 3 receptor [Source:HGNC Symbol;Acc:HGNC:2439] | 36344 | -0.206 | -0.3491 | Yes |
| 66 | IL6 | interleukin 6 [Source:HGNC Symbol;Acc:HGNC:6018] | 36396 | -0.208 | -0.3334 | Yes |
| 67 | CD8A | CD8a molecule [Source:HGNC Symbol;Acc:HGNC:1706] | 36547 | -0.213 | -0.3198 | Yes |
| 68 | ITGAM | integrin subunit alpha M [Source:HGNC Symbol;Acc:HGNC:6149] | 36644 | -0.218 | -0.3045 | Yes |
| 69 | IL1R1 | interleukin 1 receptor type 1 [Source:HGNC Symbol;Acc:HGNC:5993] | 36824 | -0.226 | -0.2905 | Yes |
| 70 | CSF1R | colony stimulating factor 1 receptor [Source:HGNC Symbol;Acc:HGNC:2433] | 37028 | -0.237 | -0.2763 | Yes |
| 71 | ITGA6 | integrin subunit alpha 6 [Source:HGNC Symbol;Acc:HGNC:6142] | 37064 | -0.239 | -0.2576 | Yes |
| 72 | IL1B | interleukin 1 beta [Source:HGNC Symbol;Acc:HGNC:5992] | 37201 | -0.248 | -0.2408 | Yes |
| 73 | CD4 | CD4 molecule [Source:HGNC Symbol;Acc:HGNC:1678] | 37205 | -0.248 | -0.2206 | Yes |
| 74 | ITGA1 | integrin subunit alpha 1 [Source:HGNC Symbol;Acc:HGNC:6134] | 37309 | -0.254 | -0.2025 | Yes |
| 75 | GP5 | glycoprotein V platelet [Source:HGNC Symbol;Acc:HGNC:4443] | 37320 | -0.254 | -0.1820 | Yes |
| 76 | FLT3 | fms related receptor tyrosine kinase 3 [Source:HGNC Symbol;Acc:HGNC:3765] | 37510 | -0.267 | -0.1650 | Yes |
| 77 | CD5 | CD5 molecule [Source:HGNC Symbol;Acc:HGNC:1685] | 37540 | -0.269 | -0.1437 | Yes |
| 78 | TFRC | transferrin receptor [Source:HGNC Symbol;Acc:HGNC:11763] | 37597 | -0.274 | -0.1228 | Yes |
| 79 | CD3G | CD3g molecule [Source:HGNC Symbol;Acc:HGNC:1675] | 37917 | -0.306 | -0.1059 | Yes |
| 80 | IL7R | interleukin 7 receptor [Source:HGNC Symbol;Acc:HGNC:6024] | 37941 | -0.309 | -0.0812 | Yes |
| 81 | IL1R2 | interleukin 1 receptor type 2 [Source:HGNC Symbol;Acc:HGNC:5994] | 37972 | -0.312 | -0.0564 | Yes |
| 82 | IL2RA | interleukin 2 receptor subunit alpha [Source:HGNC Symbol;Acc:HGNC:6008] | 38105 | -0.335 | -0.0324 | Yes |
| 83 | CR1 | complement C3b/C4b receptor 1 (Knops blood group) [Source:HGNC Symbol;Acc:HGNC:2334] | 38221 | -0.371 | -0.0050 | Yes |
| 84 | ITGA4 | integrin subunit alpha 4 [Source:HGNC Symbol;Acc:HGNC:6140] | 38275 | -0.394 | 0.0259 | Yes |
| 85 | GP1BB | glycoprotein Ib platelet subunit beta [Source:HGNC Symbol;Acc:HGNC:4440] | 39021 | NaN | 0.0077 | Yes |
Table: GSEA details [plain text format]

  

Fig 2: KEGG\_HEMATOPOIETIC\_CELL\_LINEAGE      
 Blue-Pink O' Gram in the Space of the Analyzed GeneSet

  

Fig 3: KEGG\_HEMATOPOIETIC\_CELL\_LINEAGE: Random ES distribution      
 Gene set null distribution of ES for **KEGG\_HEMATOPOIETIC\_CELL\_LINEAGE**

  
