# Supplementary material for: Simultaneous Presentation of Multiple Myeloma and Lung Cancer: Case Report and Gene Bioinformatics Analysis
Source: Front Oncol. 2022 Jun 13;12:859735. doi: 10.3389/fonc.2022.859735 (PMC9235397; doi:10.3389/fonc.2022.859735)
Supplement: Supplementary file 1 [file DataSheet_1.zip › The bioinformatic analysis of MM and lung cancer supplementary materials/Enrichment analysis/MECR/GSEA_4.1.0/LUAD TCGA/KEGG.Gsea.1639041756227/KEGG_HUNTINGTONS_DISEASE.html]

Details for gene set KEGG\_HUNTINGTONS\_DISEASE[GSEA]

|  || Dataset | ExpData\_collapsed\_to\_symbols.ENSG00000116353\_profile\_in\_ExpData.cls #ENSG00000116353 |
| Phenotype | ENSG00000116353\_profile\_in\_ExpData.cls#ENSG00000116353 |
| Upregulated in class | ENSG00000116353\_pos |
| GeneSet | KEGG\_HUNTINGTONS\_DISEASE |
| Enrichment Score (ES) | 0.5976797 |
| Normalized Enrichment Score (NES) | 2.685366 |
| Nominal p-value | 0.0 |
| FDR q-value | 0.0 |
| FWER p-Value | 0.0 |
Table: GSEA Results Summary

  

Fig 1: Enrichment plot: KEGG\_HUNTINGTONS\_DISEASE      
 Profile of the Running ES Score & Positions of GeneSet Members on the Rank Ordered List

  

| SYMBOL | TITLE | RANK IN GENE LIST | RANK METRIC SCORE | RUNNING ES | CORE ENRICHMENT || 1 | NDUFS8 | NADH:ubiquinone oxidoreductase core subunit S8 [Source:HGNC Symbol;Acc:HGNC:7715] | 34 | 0.446 | 0.0137 | Yes |
| 2 | NDUFS5 | NADH:ubiquinone oxidoreductase subunit S5 [Source:HGNC Symbol;Acc:HGNC:7712] | 78 | 0.411 | 0.0259 | Yes |
| 3 | NDUFA2 | NADH:ubiquinone oxidoreductase subunit A2 [Source:HGNC Symbol;Acc:HGNC:7685] | 110 | 0.398 | 0.0381 | Yes |
| 4 | POLR2L | "RNA polymerase II, I and III subunit L [Source:HGNC Symbol;Acc:HGNC:9199]" | 117 | 0.396 | 0.0509 | Yes |
| 5 | NDUFB10 | NADH:ubiquinone oxidoreductase subunit B10 [Source:HGNC Symbol;Acc:HGNC:7696] | 142 | 0.385 | 0.0628 | Yes |
| 6 | ATP5PO | ATP synthase peripheral stalk subunit OSCP [Source:HGNC Symbol;Acc:HGNC:850] | 231 | 0.366 | 0.0725 | Yes |
| 7 | NDUFB7 | NADH:ubiquinone oxidoreductase subunit B7 [Source:HGNC Symbol;Acc:HGNC:7702] | 278 | 0.355 | 0.0829 | Yes |
| 8 | NDUFV1 | NADH:ubiquinone oxidoreductase core subunit V1 [Source:HGNC Symbol;Acc:HGNC:7716] | 308 | 0.349 | 0.0935 | Yes |
| 9 | NDUFV3 | NADH:ubiquinone oxidoreductase subunit V3 [Source:HGNC Symbol;Acc:HGNC:7719] | 321 | 0.346 | 0.1045 | Yes |
| 10 | ATP5PD | ATP synthase peripheral stalk subunit d [Source:HGNC Symbol;Acc:HGNC:845] | 341 | 0.343 | 0.1152 | Yes |
| 11 | DNAL4 | dynein axonemal light chain 4 [Source:HGNC Symbol;Acc:HGNC:2955] | 363 | 0.338 | 0.1257 | Yes |
| 12 | NDUFB2 | NADH:ubiquinone oxidoreductase subunit B2 [Source:HGNC Symbol;Acc:HGNC:7697] | 440 | 0.327 | 0.1344 | Yes |
| 13 | SDHB | succinate dehydrogenase complex iron sulfur subunit B [Source:HGNC Symbol;Acc:HGNC:10681] | 443 | 0.327 | 0.1450 | Yes |
| 14 | NDUFA6 | NADH:ubiquinone oxidoreductase subunit A6 [Source:HGNC Symbol;Acc:HGNC:7690] | 471 | 0.324 | 0.1548 | Yes |
| 15 | ATP5F1D | ATP synthase F1 subunit delta [Source:HGNC Symbol;Acc:HGNC:837] | 480 | 0.321 | 0.1651 | Yes |
| 16 | NDUFS7 | NADH:ubiquinone oxidoreductase core subunit S7 [Source:HGNC Symbol;Acc:HGNC:7714] | 511 | 0.317 | 0.1747 | Yes |
| 17 | GPX1 | glutathione peroxidase 1 [Source:HGNC Symbol;Acc:HGNC:4553] | 572 | 0.311 | 0.1833 | Yes |
| 18 | POLR2E | "RNA polymerase II, I and III subunit E [Source:HGNC Symbol;Acc:HGNC:9192]" | 607 | 0.307 | 0.1924 | Yes |
| 19 | UQCR10 | "ubiquinol-cytochrome c reductase, complex III subunit X [Source:HGNC Symbol;Acc:HGNC:30863]" | 623 | 0.305 | 0.2019 | Yes |
| 20 | SOD1 | superoxide dismutase 1 [Source:HGNC Symbol;Acc:HGNC:11179] | 641 | 0.304 | 0.2114 | Yes |
| 21 | NDUFC1 | NADH:ubiquinone oxidoreductase subunit C1 [Source:HGNC Symbol;Acc:HGNC:7705] | 648 | 0.303 | 0.2211 | Yes |
| 22 | UQCRC1 | ubiquinol-cytochrome c reductase core protein 1 [Source:HGNC Symbol;Acc:HGNC:12585] | 653 | 0.302 | 0.2309 | Yes |
| 23 | NDUFS6 | NADH:ubiquinone oxidoreductase subunit S6 [Source:HGNC Symbol;Acc:HGNC:7713] | 687 | 0.298 | 0.2398 | Yes |
| 24 | ATP5PF | ATP synthase peripheral stalk subunit F6 [Source:HGNC Symbol;Acc:HGNC:847] | 702 | 0.297 | 0.2491 | Yes |
| 25 | CYC1 | cytochrome c1 [Source:HGNC Symbol;Acc:HGNC:2579] | 712 | 0.296 | 0.2585 | Yes |
| 26 | POLR2J | RNA polymerase II subunit J [Source:HGNC Symbol;Acc:HGNC:9197] | 736 | 0.294 | 0.2675 | Yes |
| 27 | NDUFA4 | NDUFA4 mitochondrial complex associated [Source:HGNC Symbol;Acc:HGNC:7687] | 738 | 0.293 | 0.2770 | Yes |
| 28 | NDUFB8 | NADH:ubiquinone oxidoreductase subunit B8 [Source:HGNC Symbol;Acc:HGNC:7703] | 748 | 0.293 | 0.2863 | Yes |
| 29 | ATP5MC1 | ATP synthase membrane subunit c locus 1 [Source:HGNC Symbol;Acc:HGNC:841] | 755 | 0.293 | 0.2957 | Yes |
| 30 | UQCR11 | "ubiquinol-cytochrome c reductase, complex III subunit XI [Source:HGNC Symbol;Acc:HGNC:30862]" | 764 | 0.292 | 0.3050 | Yes |
| 31 | POLR2I | RNA polymerase II subunit I [Source:HGNC Symbol;Acc:HGNC:9196] | 813 | 0.287 | 0.3131 | Yes |
| 32 | UQCRQ | ubiquinol-cytochrome c reductase complex III subunit VII [Source:HGNC Symbol;Acc:HGNC:29594] | 818 | 0.286 | 0.3223 | Yes |
| 33 | NDUFS3 | NADH:ubiquinone oxidoreductase core subunit S3 [Source:HGNC Symbol;Acc:HGNC:7710] | 842 | 0.284 | 0.3310 | Yes |
| 34 | NDUFA3 | NADH:ubiquinone oxidoreductase subunit A3 [Source:HGNC Symbol;Acc:HGNC:7686] | 864 | 0.282 | 0.3397 | Yes |
| 35 | NDUFA7 | NADH:ubiquinone oxidoreductase subunit A7 [Source:HGNC Symbol;Acc:HGNC:7691] | 878 | 0.281 | 0.3485 | Yes |
| 36 | NDUFB9 | NADH:ubiquinone oxidoreductase subunit B9 [Source:HGNC Symbol;Acc:HGNC:7704] | 915 | 0.278 | 0.3566 | Yes |
| 37 | UQCRH | ubiquinol-cytochrome c reductase hinge protein [Source:HGNC Symbol;Acc:HGNC:12590] | 937 | 0.275 | 0.3650 | Yes |
| 38 | POLR2G | RNA polymerase II subunit G [Source:HGNC Symbol;Acc:HGNC:9194] | 999 | 0.270 | 0.3723 | Yes |
| 39 | COX5B | cytochrome c oxidase subunit 5B [Source:HGNC Symbol;Acc:HGNC:2269] | 1019 | 0.268 | 0.3805 | Yes |
| 40 | COX4I1 | cytochrome c oxidase subunit 4I1 [Source:HGNC Symbol;Acc:HGNC:2265] | 1051 | 0.266 | 0.3884 | Yes |
| 41 | POLR2H | "RNA polymerase II, I and III subunit H [Source:HGNC Symbol;Acc:HGNC:9195]" | 1077 | 0.264 | 0.3964 | Yes |
| 42 | NDUFB1 | NADH:ubiquinone oxidoreductase subunit B1 [Source:HGNC Symbol;Acc:HGNC:7695] | 1087 | 0.263 | 0.4047 | Yes |
| 43 | ATP5MC2 | ATP synthase membrane subunit c locus 2 [Source:HGNC Symbol;Acc:HGNC:842] | 1094 | 0.263 | 0.4131 | Yes |
| 44 | COX8A | cytochrome c oxidase subunit 8A [Source:HGNC Symbol;Acc:HGNC:2294] | 1130 | 0.261 | 0.4207 | Yes |
| 45 | NDUFA8 | NADH:ubiquinone oxidoreductase subunit A8 [Source:HGNC Symbol;Acc:HGNC:7692] | 1138 | 0.261 | 0.4291 | Yes |
| 46 | NDUFB4 | NADH:ubiquinone oxidoreductase subunit B4 [Source:HGNC Symbol;Acc:HGNC:7699] | 1212 | 0.254 | 0.4355 | Yes |
| 47 | CREB3L4 | cAMP responsive element binding protein 3 like 4 [Source:HGNC Symbol;Acc:HGNC:18854] | 1223 | 0.253 | 0.4435 | Yes |
| 48 | COX7C | cytochrome c oxidase subunit 7C [Source:HGNC Symbol;Acc:HGNC:2292] | 1244 | 0.252 | 0.4512 | Yes |
| 49 | UQCRHL | ubiquinol-cytochrome c reductase hinge protein like [Source:HGNC Symbol;Acc:HGNC:51714] | 1439 | 0.239 | 0.4540 | Yes |
| 50 | ATP5F1E | ATP synthase F1 subunit epsilon [Source:HGNC Symbol;Acc:HGNC:838] | 1468 | 0.237 | 0.4610 | Yes |
| 51 | NDUFAB1 | NADH:ubiquinone oxidoreductase subunit AB1 [Source:HGNC Symbol;Acc:HGNC:7694] | 1579 | 0.230 | 0.4657 | Yes |
| 52 | COX6B1 | cytochrome c oxidase subunit 6B1 [Source:HGNC Symbol;Acc:HGNC:2280] | 1782 | 0.218 | 0.4676 | Yes |
| 53 | COX6A1 | cytochrome c oxidase subunit 6A1 [Source:HGNC Symbol;Acc:HGNC:2277] | 1827 | 0.216 | 0.4735 | Yes |
| 54 | BBC3 | BCL2 binding component 3 [Source:HGNC Symbol;Acc:HGNC:17868] | 1985 | 0.206 | 0.4762 | Yes |
| 55 | CLTB | clathrin light chain B [Source:HGNC Symbol;Acc:HGNC:2091] | 1990 | 0.206 | 0.4829 | Yes |
| 56 | SDHC | succinate dehydrogenase complex subunit C [Source:HGNC Symbol;Acc:HGNC:10682] | 2041 | 0.204 | 0.4882 | Yes |
| 57 | NDUFC2 | NADH:ubiquinone oxidoreductase subunit C2 [Source:HGNC Symbol;Acc:HGNC:7706] | 2151 | 0.199 | 0.4919 | Yes |
| 58 | NDUFA1 | NADH:ubiquinone oxidoreductase subunit A1 [Source:HGNC Symbol;Acc:HGNC:7683] | 2163 | 0.198 | 0.4981 | Yes |
| 59 | ATP5F1C | ATP synthase F1 subunit gamma [Source:HGNC Symbol;Acc:HGNC:833] | 2375 | 0.190 | 0.4989 | Yes |
| 60 | NDUFV2 | NADH:ubiquinone oxidoreductase core subunit V2 [Source:HGNC Symbol;Acc:HGNC:7717] | 2413 | 0.188 | 0.5041 | Yes |
| 61 | IFT57 | intraflagellar transport 57 [Source:HGNC Symbol;Acc:HGNC:17367] | 2438 | 0.187 | 0.5095 | Yes |
| 62 | NDUFS2 | NADH:ubiquinone oxidoreductase core subunit S2 [Source:HGNC Symbol;Acc:HGNC:7708] | 2516 | 0.184 | 0.5136 | Yes |
| 63 | SLC25A4 | solute carrier family 25 member 4 [Source:HGNC Symbol;Acc:HGNC:10990] | 2531 | 0.183 | 0.5192 | Yes |
| 64 | COX7B | cytochrome c oxidase subunit 7B [Source:HGNC Symbol;Acc:HGNC:2291] | 2582 | 0.181 | 0.5238 | Yes |
| 65 | MT-CO2 | mitochondrially encoded cytochrome c oxidase II [Source:HGNC Symbol;Acc:HGNC:7421] | 2703 | 0.176 | 0.5265 | Yes |
| 66 | COX7A2L | cytochrome c oxidase subunit 7A2 like [Source:HGNC Symbol;Acc:HGNC:2289] | 2825 | 0.171 | 0.5289 | Yes |
| 67 | UQCRB | ubiquinol-cytochrome c reductase binding protein [Source:HGNC Symbol;Acc:HGNC:12582] | 2840 | 0.170 | 0.5341 | Yes |
| 68 | COX6C | cytochrome c oxidase subunit 6C [Source:HGNC Symbol;Acc:HGNC:2285] | 2863 | 0.169 | 0.5391 | Yes |
| 69 | MT-CO3 | mitochondrially encoded cytochrome c oxidase III [Source:HGNC Symbol;Acc:HGNC:7422] | 2913 | 0.167 | 0.5433 | Yes |
| 70 | NDUFA5 | NADH:ubiquinone oxidoreductase subunit A5 [Source:HGNC Symbol;Acc:HGNC:7688] | 3044 | 0.163 | 0.5452 | Yes |
| 71 | SLC25A6 | solute carrier family 25 member 6 [Source:HGNC Symbol;Acc:HGNC:10992] | 3062 | 0.162 | 0.5501 | Yes |
| 72 | NDUFS4 | NADH:ubiquinone oxidoreductase subunit S4 [Source:HGNC Symbol;Acc:HGNC:7711] | 3070 | 0.162 | 0.5552 | Yes |
| 73 | NDUFB5 | NADH:ubiquinone oxidoreductase subunit B5 [Source:HGNC Symbol;Acc:HGNC:7700] | 3101 | 0.161 | 0.5596 | Yes |
| 74 | CASP9 | caspase 9 [Source:HGNC Symbol;Acc:HGNC:1511] | 3419 | 0.151 | 0.5564 | Yes |
| 75 | NDUFB3 | NADH:ubiquinone oxidoreductase subunit B3 [Source:HGNC Symbol;Acc:HGNC:7698] | 3463 | 0.150 | 0.5602 | Yes |
| 76 | COX5A | cytochrome c oxidase subunit 5A [Source:HGNC Symbol;Acc:HGNC:2267] | 3688 | 0.142 | 0.5591 | Yes |
| 77 | COX7A2 | cytochrome c oxidase subunit 7A2 [Source:HGNC Symbol;Acc:HGNC:2288] | 3752 | 0.141 | 0.5621 | Yes |
| 78 | MT-CO1 | mitochondrially encoded cytochrome c oxidase I [Source:HGNC Symbol;Acc:HGNC:7419] | 3805 | 0.139 | 0.5653 | Yes |
| 79 | NDUFB6 | NADH:ubiquinone oxidoreductase subunit B6 [Source:HGNC Symbol;Acc:HGNC:7701] | 3968 | 0.134 | 0.5656 | Yes |
| 80 | CREB3 | cAMP responsive element binding protein 3 [Source:HGNC Symbol;Acc:HGNC:2347] | 4068 | 0.132 | 0.5673 | Yes |
| 81 | SDHA | succinate dehydrogenase complex flavoprotein subunit A [Source:HGNC Symbol;Acc:HGNC:10680] | 4074 | 0.132 | 0.5715 | Yes |
| 82 | AP2M1 | adaptor related protein complex 2 subunit mu 1 [Source:HGNC Symbol;Acc:HGNC:564] | 4126 | 0.130 | 0.5744 | Yes |
| 83 | ATP5PB | ATP synthase peripheral stalk-membrane subunit b [Source:HGNC Symbol;Acc:HGNC:840] | 4227 | 0.128 | 0.5761 | Yes |
| 84 | NDUFA10 | NADH:ubiquinone oxidoreductase subunit A10 [Source:HGNC Symbol;Acc:HGNC:7684] | 4232 | 0.128 | 0.5801 | Yes |
| 85 | POLR2K | "RNA polymerase II, I and III subunit K [Source:HGNC Symbol;Acc:HGNC:9198]" | 4390 | 0.124 | 0.5802 | Yes |
| 86 | AP2S1 | adaptor related protein complex 2 subunit sigma 1 [Source:HGNC Symbol;Acc:HGNC:565] | 4439 | 0.123 | 0.5830 | Yes |
| 87 | MT-CYB | mitochondrially encoded cytochrome b [Source:HGNC Symbol;Acc:HGNC:7427] | 4594 | 0.119 | 0.5829 | Yes |
| 88 | ATP5MC3 | ATP synthase membrane subunit c locus 3 [Source:HGNC Symbol;Acc:HGNC:843] | 4619 | 0.119 | 0.5862 | Yes |
| 89 | MT-ATP6 | mitochondrially encoded ATP synthase membrane subunit 6 [Source:HGNC Symbol;Acc:HGNC:7414] | 4718 | 0.116 | 0.5875 | Yes |
| 90 | SLC25A5 | solute carrier family 25 member 5 [Source:HGNC Symbol;Acc:HGNC:10991] | 4760 | 0.116 | 0.5902 | Yes |
| 91 | COX7A1 | cytochrome c oxidase subunit 7A1 [Source:HGNC Symbol;Acc:HGNC:2287] | 4947 | 0.112 | 0.5891 | Yes |
| 92 | DCTN2 | dynactin subunit 2 [Source:HGNC Symbol;Acc:HGNC:2712] | 4993 | 0.111 | 0.5916 | Yes |
| 93 | COX4I2 | cytochrome c oxidase subunit 4I2 [Source:HGNC Symbol;Acc:HGNC:16232] | 5116 | 0.109 | 0.5920 | Yes |
| 94 | UQCR10P1 | UQCR10 pseudogene 1 [Source:HGNC Symbol;Acc:HGNC:54960] | 5130 | 0.109 | 0.5952 | Yes |
| 95 | POLR2C | RNA polymerase II subunit C [Source:HGNC Symbol;Acc:HGNC:9189] | 5263 | 0.106 | 0.5953 | Yes |
| 96 | CLTA | clathrin light chain A [Source:HGNC Symbol;Acc:HGNC:2090] | 5346 | 0.105 | 0.5966 | Yes |
| 97 | POLR2F | "RNA polymerase II, I and III subunit F [Source:HGNC Symbol;Acc:HGNC:9193]" | 5558 | 0.101 | 0.5945 | Yes |
| 98 | COX6A2 | cytochrome c oxidase subunit 6A2 [Source:HGNC Symbol;Acc:HGNC:2279] | 5599 | 0.100 | 0.5968 | Yes |
| 99 | ATP5F1A | ATP synthase F1 subunit alpha [Source:HGNC Symbol;Acc:HGNC:823] | 5690 | 0.099 | 0.5977 | Yes |
| 100 | SDHD | succinate dehydrogenase complex subunit D [Source:HGNC Symbol;Acc:HGNC:10683] | 5864 | 0.096 | 0.5964 | No |
| 101 | HDAC1 | histone deacetylase 1 [Source:HGNC Symbol;Acc:HGNC:4852] | 6184 | 0.090 | 0.5912 | No |
| 102 | PLCB3 | phospholipase C beta 3 [Source:HGNC Symbol;Acc:HGNC:9056] | 6249 | 0.089 | 0.5925 | No |
| 103 | POLR2J3 | RNA polymerase II subunit J3 [Source:HGNC Symbol;Acc:HGNC:33853] | 6597 | 0.085 | 0.5864 | No |
| 104 | DNALI1 | dynein axonemal light intermediate chain 1 [Source:HGNC Symbol;Acc:HGNC:14353] | 6742 | 0.083 | 0.5854 | No |
| 105 | BAX | "BCL2 associated X, apoptosis regulator [Source:HGNC Symbol;Acc:HGNC:959]" | 7334 | 0.075 | 0.5727 | No |
| 106 | UQCRC2 | ubiquinol-cytochrome c reductase core protein 2 [Source:HGNC Symbol;Acc:HGNC:12586] | 7737 | 0.070 | 0.5648 | No |
| 107 | NDUFA9 | NADH:ubiquinone oxidoreductase subunit A9 [Source:HGNC Symbol;Acc:HGNC:7693] | 7750 | 0.070 | 0.5667 | No |
| 108 | CYCS | "cytochrome c, somatic [Source:HGNC Symbol;Acc:HGNC:19986]" | 8363 | 0.064 | 0.5532 | No |
| 109 | TP53 | tumor protein p53 [Source:HGNC Symbol;Acc:HGNC:11998] | 8681 | 0.061 | 0.5470 | No |
| 110 | CLTCL1 | clathrin heavy chain like 1 [Source:HGNC Symbol;Acc:HGNC:2093] | 9093 | 0.057 | 0.5384 | No |
| 111 | UQCRFS1 | "ubiquinol-cytochrome c reductase, Rieske iron-sulfur polypeptide 1 [Source:HGNC Symbol;Acc:HGNC:12587]" | 9310 | 0.055 | 0.5346 | No |
| 112 | MT-ATP8 | mitochondrially encoded ATP synthase membrane subunit 8 [Source:HGNC Symbol;Acc:HGNC:7415] | 9337 | 0.054 | 0.5358 | No |
| 113 | VDAC2 | voltage dependent anion channel 2 [Source:HGNC Symbol;Acc:HGNC:12672] | 10201 | 0.047 | 0.5152 | No |
| 114 | SLC25A31 | solute carrier family 25 member 31 [Source:HGNC Symbol;Acc:HGNC:25319] | 10585 | 0.044 | 0.5069 | No |
| 115 | POLR2J2 | RNA polymerase II subunit J2 [Source:HGNC Symbol;Acc:HGNC:23208] | 10680 | 0.044 | 0.5059 | No |
| 116 | DCTN4 | dynactin subunit 4 [Source:HGNC Symbol;Acc:HGNC:15518] | 10730 | 0.043 | 0.5061 | No |
| 117 | VDAC2P5 | VDAC2 pseudogene 5 [Source:HGNC Symbol;Acc:HGNC:54753] | 11481 | 0.037 | 0.4881 | No |
| 118 | DNAI1 | dynein axonemal intermediate chain 1 [Source:HGNC Symbol;Acc:HGNC:2954] | 11663 | 0.036 | 0.4847 | No |
| 119 | DCTN1 | dynactin subunit 1 [Source:HGNC Symbol;Acc:HGNC:2711] | 13723 | 0.021 | 0.4327 | No |
| 120 | POLR2D | RNA polymerase II subunit D [Source:HGNC Symbol;Acc:HGNC:9191] | 14063 | 0.019 | 0.4247 | No |
| 121 | NDUFA4L2 | NDUFA4 mitochondrial complex associated like 2 [Source:HGNC Symbol;Acc:HGNC:29836] | 15776 | 0.008 | 0.3812 | No |
| 122 | GRIN1 | glutamate ionotropic receptor NMDA type subunit 1 [Source:HGNC Symbol;Acc:HGNC:4584] | 15818 | 0.008 | 0.3805 | No |
| 123 | DNAH2 | dynein axonemal heavy chain 2 [Source:HGNC Symbol;Acc:HGNC:2948] | 16420 | 0.005 | 0.3652 | No |
| 124 | DNAI2 | dynein axonemal intermediate chain 2 [Source:HGNC Symbol;Acc:HGNC:18744] | 19213 | -0.012 | 0.2943 | No |
| 125 | UCP1 | uncoupling protein 1 [Source:HGNC Symbol;Acc:HGNC:12517] | 19214 | -0.012 | 0.2947 | No |
| 126 | VDAC3 | voltage dependent anion channel 3 [Source:HGNC Symbol;Acc:HGNC:12674] | 19385 | -0.013 | 0.2908 | No |
| 127 | HIP1 | huntingtin interacting protein 1 [Source:HGNC Symbol;Acc:HGNC:4913] | 20132 | -0.017 | 0.2723 | No |
| 128 | COX8C | cytochrome c oxidase subunit 8C [Source:HGNC Symbol;Acc:HGNC:24382] | 20162 | -0.018 | 0.2721 | No |
| 129 | AP2B1 | adaptor related protein complex 2 subunit beta 1 [Source:HGNC Symbol;Acc:HGNC:563] | 20728 | -0.021 | 0.2584 | No |
| 130 | ATP5MC1P5 | ATP synthase membrane subunit c locus 1 pseudogene 5 [Source:HGNC Symbol;Acc:HGNC:39508] | 20955 | -0.022 | 0.2533 | No |
| 131 | AP2A2 | adaptor related protein complex 2 subunit alpha 2 [Source:HGNC Symbol;Acc:HGNC:562] | 21173 | -0.024 | 0.2486 | No |
| 132 | COX6B2 | cytochrome c oxidase subunit 6B2 [Source:HGNC Symbol;Acc:HGNC:24380] | 21295 | -0.025 | 0.2463 | No |
| 133 | COX6CP3 | cytochrome c oxidase subunit 6C pseudogene 3 [Source:HGNC Symbol;Acc:HGNC:31721] | 21611 | -0.027 | 0.2391 | No |
| 134 | DNAH3 | dynein axonemal heavy chain 3 [Source:HGNC Symbol;Acc:HGNC:2949] | 21847 | -0.028 | 0.2340 | No |
| 135 | PPARG | peroxisome proliferator activated receptor gamma [Source:HGNC Symbol;Acc:HGNC:9236] | 21861 | -0.028 | 0.2346 | No |
| 136 | TBPL2 | TATA-box binding protein like 2 [Source:HGNC Symbol;Acc:HGNC:19841] | 21961 | -0.029 | 0.2330 | No |
| 137 | COX7B2 | cytochrome c oxidase subunit 7B2 [Source:HGNC Symbol;Acc:HGNC:24381] | 22662 | -0.033 | 0.2162 | No |
| 138 | NRF1 | nuclear respiratory factor 1 [Source:HGNC Symbol;Acc:HGNC:7996] | 23357 | -0.038 | 0.1997 | No |
| 139 | BDNF | brain derived neurotrophic factor [Source:HGNC Symbol;Acc:HGNC:1033] | 25218 | -0.050 | 0.1538 | No |
| 140 | TFAM | "transcription factor A, mitochondrial [Source:HGNC Symbol;Acc:HGNC:11741]" | 25296 | -0.051 | 0.1535 | No |
| 141 | PPARGC1A | PPARG coactivator 1 alpha [Source:HGNC Symbol;Acc:HGNC:9237] | 25931 | -0.055 | 0.1391 | No |
| 142 | TAF4B | TATA-box binding protein associated factor 4b [Source:HGNC Symbol;Acc:HGNC:11538] | 26646 | -0.061 | 0.1228 | No |
| 143 | CREB5 | cAMP responsive element binding protein 5 [Source:HGNC Symbol;Acc:HGNC:16844] | 26714 | -0.061 | 0.1231 | No |
| 144 | CASP8 | caspase 8 [Source:HGNC Symbol;Acc:HGNC:1509] | 27243 | -0.065 | 0.1117 | No |
| 145 | PLCB4 | phospholipase C beta 4 [Source:HGNC Symbol;Acc:HGNC:9059] | 27781 | -0.069 | 0.1003 | No |
| 146 | AP2A1 | adaptor related protein complex 2 subunit alpha 1 [Source:HGNC Symbol;Acc:HGNC:561] | 28078 | -0.072 | 0.0950 | No |
| 147 | ATP5F1B | ATP synthase F1 subunit beta [Source:HGNC Symbol;Acc:HGNC:830] | 28627 | -0.077 | 0.0835 | No |
| 148 | TBP | TATA-box binding protein [Source:HGNC Symbol;Acc:HGNC:11588] | 29083 | -0.081 | 0.0745 | No |
| 149 | GRIN2B | glutamate ionotropic receptor NMDA type subunit 2B [Source:HGNC Symbol;Acc:HGNC:4586] | 29542 | -0.085 | 0.0656 | No |
| 150 | VDAC1 | voltage dependent anion channel 1 [Source:HGNC Symbol;Acc:HGNC:12669] | 29848 | -0.088 | 0.0607 | No |
| 151 | TAF4 | TATA-box binding protein associated factor 4 [Source:HGNC Symbol;Acc:HGNC:11537] | 30131 | -0.091 | 0.0564 | No |
| 152 | CREB3L1 | cAMP responsive element binding protein 3 like 1 [Source:HGNC Symbol;Acc:HGNC:18856] | 30283 | -0.093 | 0.0556 | No |
| 153 | TBPL1 | TATA-box binding protein like 1 [Source:HGNC Symbol;Acc:HGNC:11589] | 30445 | -0.094 | 0.0546 | No |
| 154 | NDUFS1 | NADH:ubiquinone oxidoreductase core subunit S1 [Source:HGNC Symbol;Acc:HGNC:7707] | 30490 | -0.095 | 0.0565 | No |
| 155 | PPID | peptidylprolyl isomerase D [Source:HGNC Symbol;Acc:HGNC:9257] | 30753 | -0.098 | 0.0530 | No |
| 156 | HTT | huntingtin [Source:HGNC Symbol;Acc:HGNC:4851] | 30977 | -0.100 | 0.0506 | No |
| 157 | CASP3 | caspase 3 [Source:HGNC Symbol;Acc:HGNC:1504] | 31110 | -0.102 | 0.0505 | No |
| 158 | RCOR1 | REST corepressor 1 [Source:HGNC Symbol;Acc:HGNC:17441] | 32012 | -0.113 | 0.0312 | No |
| 159 | GRM5 | glutamate metabotropic receptor 5 [Source:HGNC Symbol;Acc:HGNC:4597] | 32047 | -0.114 | 0.0340 | No |
| 160 | POLR2B | RNA polymerase II subunit B [Source:HGNC Symbol;Acc:HGNC:9188] | 32968 | -0.127 | 0.0147 | No |
| 161 | HDAC2 | histone deacetylase 2 [Source:HGNC Symbol;Acc:HGNC:4853] | 33490 | -0.136 | 0.0058 | No |
| 162 | GNAQ | G protein subunit alpha q [Source:HGNC Symbol;Acc:HGNC:4390] | 33694 | -0.139 | 0.0052 | No |
| 163 | PLCB2 | phospholipase C beta 2 [Source:HGNC Symbol;Acc:HGNC:9055] | 33768 | -0.141 | 0.0079 | No |
| 164 | DLG4 | discs large MAGUK scaffold protein 4 [Source:HGNC Symbol;Acc:HGNC:2903] | 33780 | -0.141 | 0.0122 | No |
| 165 | CLTC | clathrin heavy chain [Source:HGNC Symbol;Acc:HGNC:2092] | 33799 | -0.142 | 0.0164 | No |
| 166 | ITPR1 | "inositol 1,4,5-trisphosphate receptor type 1 [Source:HGNC Symbol;Acc:HGNC:6180]" | 33914 | -0.144 | 0.0181 | No |
| 167 | POLR2A | RNA polymerase II subunit A [Source:HGNC Symbol;Acc:HGNC:9187] | 34410 | -0.153 | 0.0105 | No |
| 168 | CREBBP | CREB binding protein [Source:HGNC Symbol;Acc:HGNC:2348] | 34790 | -0.162 | 0.0061 | No |
| 169 | SIN3A | SIN3 transcription regulator family member A [Source:HGNC Symbol;Acc:HGNC:19353] | 34829 | -0.163 | 0.0104 | No |
| 170 | DNAL1 | dynein axonemal light chain 1 [Source:HGNC Symbol;Acc:HGNC:23247] | 34961 | -0.165 | 0.0125 | No |
| 171 | DNAH1 | dynein axonemal heavy chain 1 [Source:HGNC Symbol;Acc:HGNC:2940] | 35707 | -0.185 | -0.0006 | No |
| 172 | CREB3L3 | cAMP responsive element binding protein 3 like 3 [Source:HGNC Symbol;Acc:HGNC:18855] | 35894 | -0.190 | 0.0009 | No |
| 173 | HAP1 | huntingtin associated protein 1 [Source:HGNC Symbol;Acc:HGNC:4812] | 36161 | -0.199 | 0.0006 | No |
| 174 | PLCB1 | phospholipase C beta 1 [Source:HGNC Symbol;Acc:HGNC:15917] | 36397 | -0.208 | 0.0014 | No |
| 175 | CREB3L2 | cAMP responsive element binding protein 3 like 2 [Source:HGNC Symbol;Acc:HGNC:23720] | 36510 | -0.212 | 0.0054 | No |
| 176 | TGM2 | transglutaminase 2 [Source:HGNC Symbol;Acc:HGNC:11778] | 37352 | -0.256 | -0.0077 | No |
| 177 | CREB1 | cAMP responsive element binding protein 1 [Source:HGNC Symbol;Acc:HGNC:2345] | 37371 | -0.257 | 0.0002 | No |
| 178 | REST | RE1 silencing transcription factor [Source:HGNC Symbol;Acc:HGNC:9966] | 37379 | -0.258 | 0.0084 | No |
| 179 | EP300 | E1A binding protein p300 [Source:HGNC Symbol;Acc:HGNC:3373] | 37388 | -0.259 | 0.0166 | No |
| 180 | SOD2 | superoxide dismutase 2 [Source:HGNC Symbol;Acc:HGNC:11180] | 37689 | -0.283 | 0.0182 | No |
| 181 | SP1 | Sp1 transcription factor [Source:HGNC Symbol;Acc:HGNC:11205] | 38109 | -0.336 | 0.0184 | No |
| 182 | APAF1 | apoptotic peptidase activating factor 1 [Source:HGNC Symbol;Acc:HGNC:576] | 38254 | -0.386 | 0.0273 | No |
Table: GSEA details [plain text format]

  

Fig 2: KEGG\_HUNTINGTONS\_DISEASE      
 Blue-Pink O' Gram in the Space of the Analyzed GeneSet

  

Fig 3: KEGG\_HUNTINGTONS\_DISEASE: Random ES distribution      
 Gene set null distribution of ES for **KEGG\_HUNTINGTONS\_DISEASE**

  
