# Supplementary material for: Simultaneous Presentation of Multiple Myeloma and Lung Cancer: Case Report and Gene Bioinformatics Analysis
Source: Front Oncol. 2022 Jun 13;12:859735. doi: 10.3389/fonc.2022.859735 (PMC9235397; doi:10.3389/fonc.2022.859735)
Supplement: Supplementary file 1 [file DataSheet_1.zip › The bioinformatic analysis of MM and lung cancer supplementary materials/Enrichment analysis/MECR/GSEA_4.1.0/LUAD TCGA/KEGG.Gsea.1639041756227/KEGG_INTESTINAL_IMMUNE_NETWORK_FOR_IGA_PRODUCTION.html]

Details for gene set KEGG\_INTESTINAL\_IMMUNE\_NETWORK\_FOR\_IGA\_PRODUCTION[GSEA]

|  || Dataset | ExpData\_collapsed\_to\_symbols.ENSG00000116353\_profile\_in\_ExpData.cls #ENSG00000116353 |
| Phenotype | ENSG00000116353\_profile\_in\_ExpData.cls#ENSG00000116353 |
| Upregulated in class | ENSG00000116353\_neg |
| GeneSet | KEGG\_INTESTINAL\_IMMUNE\_NETWORK\_FOR\_IGA\_PRODUCTION |
| Enrichment Score (ES) | -0.64146054 |
| Normalized Enrichment Score (NES) | -2.2902768 |
| Nominal p-value | 0.0 |
| FDR q-value | 0.0 |
| FWER p-Value | 0.0 |
Table: GSEA Results Summary

  

Fig 1: Enrichment plot: KEGG\_INTESTINAL\_IMMUNE\_NETWORK\_FOR\_IGA\_PRODUCTION      
 Profile of the Running ES Score & Positions of GeneSet Members on the Rank Ordered List

  

| SYMBOL | TITLE | RANK IN GENE LIST | RANK METRIC SCORE | RUNNING ES | CORE ENRICHMENT || 1 | HLA-DMA | "major histocompatibility complex, class II, DM alpha [Source:HGNC Symbol;Acc:HGNC:4934]" | 4341 | 0.126 | -0.0882 | No |
| 2 | LTBR | lymphotoxin beta receptor [Source:HGNC Symbol;Acc:HGNC:6718] | 6244 | 0.090 | -0.1207 | No |
| 3 | MADCAM1 | mucosal vascular addressin cell adhesion molecule 1 [Source:HGNC Symbol;Acc:HGNC:6765] | 6690 | 0.084 | -0.1171 | No |
| 4 | IL4 | interleukin 4 [Source:HGNC Symbol;Acc:HGNC:6014] | 7378 | 0.075 | -0.1213 | No |
| 5 | TNFSF13 | TNF superfamily member 13 [Source:HGNC Symbol;Acc:HGNC:11928] | 8318 | 0.064 | -0.1338 | No |
| 6 | CCL28 | C-C motif chemokine ligand 28 [Source:HGNC Symbol;Acc:HGNC:17700] | 8536 | 0.062 | -0.1282 | No |
| 7 | HLA-DRB1 | "major histocompatibility complex, class II, DR beta 1 [Source:HGNC Symbol;Acc:HGNC:4948]" | 10444 | 0.045 | -0.1687 | No |
| 8 | HLA-DRB5 | "major histocompatibility complex, class II, DR beta 5 [Source:HGNC Symbol;Acc:HGNC:4953]" | 10518 | 0.045 | -0.1626 | No |
| 9 | HLA-DPB1 | "major histocompatibility complex, class II, DP beta 1 [Source:HGNC Symbol;Acc:HGNC:4940]" | 16356 | 0.005 | -0.3104 | No |
| 10 | CD40 | CD40 molecule [Source:HGNC Symbol;Acc:HGNC:11919] | 17012 | 0.001 | -0.3268 | No |
| 11 | HLA-DRA | "major histocompatibility complex, class II, DR alpha [Source:HGNC Symbol;Acc:HGNC:4947]" | 17663 | -0.003 | -0.3429 | No |
| 12 | HLA-DQB1 | "major histocompatibility complex, class II, DQ beta 1 [Source:HGNC Symbol;Acc:HGNC:4944]" | 17913 | -0.004 | -0.3484 | No |
| 13 | CCL27 | C-C motif chemokine ligand 27 [Source:HGNC Symbol;Acc:HGNC:10626] | 18208 | -0.006 | -0.3548 | No |
| 14 | HLA-DQA2 | "major histocompatibility complex, class II, DQ alpha 2 [Source:HGNC Symbol;Acc:HGNC:4943]" | 19230 | -0.012 | -0.3786 | No |
| 15 | ICOSLG | inducible T cell costimulator ligand [Source:HGNC Symbol;Acc:HGNC:17087] | 19606 | -0.014 | -0.3857 | No |
| 16 | HLA-DOB | "major histocompatibility complex, class II, DO beta [Source:HGNC Symbol;Acc:HGNC:4937]" | 20731 | -0.021 | -0.4105 | No |
| 17 | IL2 | interleukin 2 [Source:HGNC Symbol;Acc:HGNC:6001] | 20777 | -0.021 | -0.4079 | No |
| 18 | PIGR | polymeric immunoglobulin receptor [Source:HGNC Symbol;Acc:HGNC:8968] | 21074 | -0.023 | -0.4113 | No |
| 19 | TGFB1 | transforming growth factor beta 1 [Source:HGNC Symbol;Acc:HGNC:11766] | 22340 | -0.031 | -0.4380 | No |
| 20 | HLA-DPA1 | "major histocompatibility complex, class II, DP alpha 1 [Source:HGNC Symbol;Acc:HGNC:4938]" | 24356 | -0.044 | -0.4814 | No |
| 21 | CCR10 | C-C motif chemokine receptor 10 [Source:HGNC Symbol;Acc:HGNC:4474] | 24820 | -0.047 | -0.4848 | No |
| 22 | HLA-DMB | "major histocompatibility complex, class II, DM beta [Source:HGNC Symbol;Acc:HGNC:4935]" | 24935 | -0.048 | -0.4791 | No |
| 23 | IL5 | interleukin 5 [Source:HGNC Symbol;Acc:HGNC:6016] | 25260 | -0.050 | -0.4784 | No |
| 24 | IL15 | interleukin 15 [Source:HGNC Symbol;Acc:HGNC:5977] | 27093 | -0.064 | -0.5136 | No |
| 25 | MAP3K14 | mitogen-activated protein kinase kinase kinase 14 [Source:HGNC Symbol;Acc:HGNC:6853] | 31902 | -0.112 | -0.6161 | No |
| 26 | HLA-DQA1 | "major histocompatibility complex, class II, DQ alpha 1 [Source:HGNC Symbol;Acc:HGNC:4942]" | 32898 | -0.126 | -0.6190 | Yes |
| 27 | TNFRSF13B | TNF receptor superfamily member 13B [Source:HGNC Symbol;Acc:HGNC:18153] | 33212 | -0.131 | -0.6036 | Yes |
| 28 | HLA-DOA | "major histocompatibility complex, class II, DO alpha [Source:HGNC Symbol;Acc:HGNC:4936]" | 33498 | -0.136 | -0.5866 | Yes |
| 29 | CCR9 | C-C motif chemokine receptor 9 [Source:HGNC Symbol;Acc:HGNC:1610] | 34438 | -0.154 | -0.5831 | Yes |
| 30 | IL15RA | interleukin 15 receptor subunit alpha [Source:HGNC Symbol;Acc:HGNC:5978] | 34548 | -0.156 | -0.5582 | Yes |
| 31 | TNFRSF13C | TNF receptor superfamily member 13C [Source:HGNC Symbol;Acc:HGNC:17755] | 34842 | -0.163 | -0.5366 | Yes |
| 32 | CCL25 | C-C motif chemokine ligand 25 [Source:HGNC Symbol;Acc:HGNC:10624] | 35008 | -0.166 | -0.5112 | Yes |
| 33 | CD40LG | CD40 ligand [Source:HGNC Symbol;Acc:HGNC:11935] | 35185 | -0.171 | -0.4853 | Yes |
| 34 | AICDA | activation induced cytidine deaminase [Source:HGNC Symbol;Acc:HGNC:13203] | 35567 | -0.181 | -0.4627 | Yes |
| 35 | CXCL12 | C-X-C motif chemokine ligand 12 [Source:HGNC Symbol;Acc:HGNC:10672] | 35899 | -0.191 | -0.4372 | Yes |
| 36 | IL6 | interleukin 6 [Source:HGNC Symbol;Acc:HGNC:6018] | 36396 | -0.208 | -0.4129 | Yes |
| 37 | CXCR4 | C-X-C motif chemokine receptor 4 [Source:HGNC Symbol;Acc:HGNC:2561] | 36579 | -0.215 | -0.3793 | Yes |
| 38 | TNFSF13B | TNF superfamily member 13b [Source:HGNC Symbol;Acc:HGNC:11929] | 36605 | -0.215 | -0.3416 | Yes |
| 39 | TNFRSF17 | TNF receptor superfamily member 17 [Source:HGNC Symbol;Acc:HGNC:11913] | 36751 | -0.222 | -0.3058 | Yes |
| 40 | ICOS | inducible T cell costimulator [Source:HGNC Symbol;Acc:HGNC:5351] | 37068 | -0.239 | -0.2712 | Yes |
| 41 | IL10 | interleukin 10 [Source:HGNC Symbol;Acc:HGNC:5962] | 37315 | -0.254 | -0.2323 | Yes |
| 42 | CD86 | CD86 molecule [Source:HGNC Symbol;Acc:HGNC:1705] | 37625 | -0.276 | -0.1910 | Yes |
| 43 | CD80 | CD80 molecule [Source:HGNC Symbol;Acc:HGNC:1700] | 37726 | -0.285 | -0.1428 | Yes |
| 44 | CD28 | CD28 molecule [Source:HGNC Symbol;Acc:HGNC:1653] | 37779 | -0.289 | -0.0927 | Yes |
| 45 | ITGB7 | integrin subunit beta 7 [Source:HGNC Symbol;Acc:HGNC:6162] | 38154 | -0.347 | -0.0405 | Yes |
| 46 | ITGA4 | integrin subunit alpha 4 [Source:HGNC Symbol;Acc:HGNC:6140] | 38275 | -0.394 | 0.0267 | Yes |
Table: GSEA details [plain text format]

  

Fig 2: KEGG\_INTESTINAL\_IMMUNE\_NETWORK\_FOR\_IGA\_PRODUCTION      
 Blue-Pink O' Gram in the Space of the Analyzed GeneSet

  

Fig 3: KEGG\_INTESTINAL\_IMMUNE\_NETWORK\_FOR\_IGA\_PRODUCTION: Random ES distribution      
 Gene set null distribution of ES for **KEGG\_INTESTINAL\_IMMUNE\_NETWORK\_FOR\_IGA\_PRODUCTION**

  
