# Supplementary material for: Simultaneous Presentation of Multiple Myeloma and Lung Cancer: Case Report and Gene Bioinformatics Analysis
Source: Front Oncol. 2022 Jun 13;12:859735. doi: 10.3389/fonc.2022.859735 (PMC9235397; doi:10.3389/fonc.2022.859735)
Supplement: Supplementary file 1 [file DataSheet_1.zip › The bioinformatic analysis of MM and lung cancer supplementary materials/Enrichment analysis/MECR/GSEA_4.1.0/LUAD TCGA/KEGG.Gsea.1639041756227/KEGG_JAK_STAT_SIGNALING_PATHWAY.html]

Details for gene set KEGG\_JAK\_STAT\_SIGNALING\_PATHWAY[GSEA]

|  || Dataset | ExpData\_collapsed\_to\_symbols.ENSG00000116353\_profile\_in\_ExpData.cls #ENSG00000116353 |
| Phenotype | ENSG00000116353\_profile\_in\_ExpData.cls#ENSG00000116353 |
| Upregulated in class | ENSG00000116353\_neg |
| GeneSet | KEGG\_JAK\_STAT\_SIGNALING\_PATHWAY |
| Enrichment Score (ES) | -0.5006221 |
| Normalized Enrichment Score (NES) | -2.2154014 |
| Nominal p-value | 0.0 |
| FDR q-value | 9.872923E-5 |
| FWER p-Value | 0.001 |
Table: GSEA Results Summary

  

Fig 1: Enrichment plot: KEGG\_JAK\_STAT\_SIGNALING\_PATHWAY      
 Profile of the Running ES Score & Positions of GeneSet Members on the Rank Ordered List

  

| SYMBOL | TITLE | RANK IN GENE LIST | RANK METRIC SCORE | RUNNING ES | CORE ENRICHMENT || 1 | PIK3R2 | phosphoinositide-3-kinase regulatory subunit 2 [Source:HGNC Symbol;Acc:HGNC:8980] | 545 | 0.314 | 0.0024 | No |
| 2 | CCND3 | cyclin D3 [Source:HGNC Symbol;Acc:HGNC:1585] | 1042 | 0.267 | 0.0036 | No |
| 3 | CTF1 | cardiotrophin 1 [Source:HGNC Symbol;Acc:HGNC:2499] | 1519 | 0.234 | 0.0036 | No |
| 4 | CISH | cytokine inducible SH2 containing protein [Source:HGNC Symbol;Acc:HGNC:1984] | 2302 | 0.193 | -0.0064 | No |
| 5 | IL11RA | interleukin 11 receptor subunit alpha [Source:HGNC Symbol;Acc:HGNC:5967] | 2627 | 0.179 | -0.0054 | No |
| 6 | IFNGR2 | interferon gamma receptor 2 [Source:HGNC Symbol;Acc:HGNC:5440] | 2702 | 0.176 | 0.0019 | No |
| 7 | AKT1 | AKT serine/threonine kinase 1 [Source:HGNC Symbol;Acc:HGNC:391] | 3179 | 0.158 | -0.0021 | No |
| 8 | CLCF1 | cardiotrophin like cytokine factor 1 [Source:HGNC Symbol;Acc:HGNC:17412] | 3362 | 0.152 | 0.0012 | No |
| 9 | EPOR | erythropoietin receptor [Source:HGNC Symbol;Acc:HGNC:3416] | 3751 | 0.141 | -0.0014 | No |
| 10 | IL10RB | interleukin 10 receptor subunit beta [Source:HGNC Symbol;Acc:HGNC:5965] | 4583 | 0.120 | -0.0164 | No |
| 11 | SPRY2 | sprouty RTK signaling antagonist 2 [Source:HGNC Symbol;Acc:HGNC:11270] | 4873 | 0.114 | -0.0179 | No |
| 12 | PIAS3 | protein inhibitor of activated STAT 3 [Source:HGNC Symbol;Acc:HGNC:16861] | 5647 | 0.100 | -0.0325 | No |
| 13 | IFNA5 | interferon alpha 5 [Source:HGNC Symbol;Acc:HGNC:5426] | 6009 | 0.093 | -0.0368 | No |
| 14 | EPO | erythropoietin [Source:HGNC Symbol;Acc:HGNC:3415] | 6062 | 0.093 | -0.0333 | No |
| 15 | IFNL3 | interferon lambda 3 [Source:HGNC Symbol;Acc:HGNC:18365] | 6168 | 0.091 | -0.0313 | No |
| 16 | IL20RB | interleukin 20 receptor subunit beta [Source:HGNC Symbol;Acc:HGNC:6004] | 6391 | 0.087 | -0.0324 | No |
| 17 | BCL2L1 | BCL2 like 1 [Source:HGNC Symbol;Acc:HGNC:992] | 6882 | 0.081 | -0.0407 | No |
| 18 | TPO | thyroid peroxidase [Source:HGNC Symbol;Acc:HGNC:12015] | 6966 | 0.080 | -0.0387 | No |
| 19 | IL4 | interleukin 4 [Source:HGNC Symbol;Acc:HGNC:6014] | 7378 | 0.075 | -0.0453 | No |
| 20 | IL3 | interleukin 3 [Source:HGNC Symbol;Acc:HGNC:6011] | 8074 | 0.067 | -0.0596 | No |
| 21 | IL6R | interleukin 6 receptor [Source:HGNC Symbol;Acc:HGNC:6019] | 8091 | 0.067 | -0.0566 | No |
| 22 | IFNL2 | interferon lambda 2 [Source:HGNC Symbol;Acc:HGNC:18364] | 8127 | 0.066 | -0.0540 | No |
| 23 | IFNA16 | interferon alpha 16 [Source:HGNC Symbol;Acc:HGNC:5421] | 8538 | 0.062 | -0.0613 | No |
| 24 | STAT6 | signal transducer and activator of transcription 6 [Source:HGNC Symbol;Acc:HGNC:11368] | 8586 | 0.061 | -0.0593 | No |
| 25 | PIK3R3 | phosphoinositide-3-kinase regulatory subunit 3 [Source:HGNC Symbol;Acc:HGNC:8981] | 9028 | 0.057 | -0.0676 | No |
| 26 | IFNA7 | interferon alpha 7 [Source:HGNC Symbol;Acc:HGNC:5428] | 9153 | 0.056 | -0.0678 | No |
| 27 | IFNB1 | interferon beta 1 [Source:HGNC Symbol;Acc:HGNC:5434] | 9334 | 0.054 | -0.0696 | No |
| 28 | CSF2 | colony stimulating factor 2 [Source:HGNC Symbol;Acc:HGNC:2434] | 9888 | 0.050 | -0.0811 | No |
| 29 | AKT2 | AKT serine/threonine kinase 2 [Source:HGNC Symbol;Acc:HGNC:392] | 10579 | 0.044 | -0.0964 | No |
| 30 | IFNL1 | interferon lambda 1 [Source:HGNC Symbol;Acc:HGNC:18363] | 10754 | 0.043 | -0.0986 | No |
| 31 | MPL | "MPL proto-oncogene, thrombopoietin receptor [Source:HGNC Symbol;Acc:HGNC:7217]" | 10820 | 0.042 | -0.0981 | No |
| 32 | PIAS4 | protein inhibitor of activated STAT 4 [Source:HGNC Symbol;Acc:HGNC:17002] | 11249 | 0.039 | -0.1070 | No |
| 33 | IFNLR1 | interferon lambda receptor 1 [Source:HGNC Symbol;Acc:HGNC:18584] | 11373 | 0.038 | -0.1082 | No |
| 34 | IFNA13 | interferon alpha 13 [Source:HGNC Symbol;Acc:HGNC:5419] | 11857 | 0.034 | -0.1187 | No |
| 35 | TYK2 | tyrosine kinase 2 [Source:HGNC Symbol;Acc:HGNC:12440] | 11977 | 0.033 | -0.1200 | No |
| 36 | CRLF2 | cytokine receptor like factor 2 [Source:HGNC Symbol;Acc:HGNC:14281] | 12291 | 0.031 | -0.1264 | No |
| 37 | PRLR | prolactin receptor [Source:HGNC Symbol;Acc:HGNC:9446] | 12366 | 0.030 | -0.1267 | No |
| 38 | IL19 | interleukin 19 [Source:HGNC Symbol;Acc:HGNC:5990] | 12375 | 0.030 | -0.1254 | No |
| 39 | SOCS1 | suppressor of cytokine signaling 1 [Source:HGNC Symbol;Acc:HGNC:19383] | 12731 | 0.028 | -0.1330 | No |
| 40 | CSH1 | chorionic somatomammotropin hormone 1 [Source:HGNC Symbol;Acc:HGNC:2440] | 13107 | 0.025 | -0.1413 | No |
| 41 | IFNA8 | interferon alpha 8 [Source:HGNC Symbol;Acc:HGNC:5429] | 13190 | 0.024 | -0.1421 | No |
| 42 | SPRED2 | sprouty related EVH1 domain containing 2 [Source:HGNC Symbol;Acc:HGNC:17722] | 13454 | 0.023 | -0.1476 | No |
| 43 | IL22RA1 | interleukin 22 receptor subunit alpha 1 [Source:HGNC Symbol;Acc:HGNC:13700] | 14064 | 0.019 | -0.1622 | No |
| 44 | IL12B | interleukin 12B [Source:HGNC Symbol;Acc:HGNC:5970] | 15071 | 0.013 | -0.1872 | No |
| 45 | CNTFR | ciliary neurotrophic factor receptor [Source:HGNC Symbol;Acc:HGNC:2170] | 15115 | 0.012 | -0.1877 | No |
| 46 | IL13RA2 | interleukin 13 receptor subunit alpha 2 [Source:HGNC Symbol;Acc:HGNC:5975] | 15267 | 0.011 | -0.1909 | No |
| 47 | IL9 | interleukin 9 [Source:HGNC Symbol;Acc:HGNC:6029] | 15668 | 0.009 | -0.2007 | No |
| 48 | IFNAR1 | interferon alpha and beta receptor subunit 1 [Source:HGNC Symbol;Acc:HGNC:5432] | 15859 | 0.008 | -0.2051 | No |
| 49 | IFNA4 | interferon alpha 4 [Source:HGNC Symbol;Acc:HGNC:5425] | 16049 | 0.007 | -0.2096 | No |
| 50 | IFNE | interferon epsilon [Source:HGNC Symbol;Acc:HGNC:18163] | 16104 | 0.006 | -0.2106 | No |
| 51 | OSMR | oncostatin M receptor [Source:HGNC Symbol;Acc:HGNC:8507] | 16481 | 0.004 | -0.2200 | No |
| 52 | CBLC | Cbl proto-oncogene C [Source:HGNC Symbol;Acc:HGNC:15961] | 16554 | 0.004 | -0.2217 | No |
| 53 | SPRY1 | sprouty RTK signaling antagonist 1 [Source:HGNC Symbol;Acc:HGNC:11269] | 16625 | 0.003 | -0.2233 | No |
| 54 | IFNA21 | interferon alpha 21 [Source:HGNC Symbol;Acc:HGNC:5424] | 16891 | 0.002 | -0.2299 | No |
| 55 | MYC | "MYC proto-oncogene, bHLH transcription factor [Source:HGNC Symbol;Acc:HGNC:7553]" | 16923 | 0.002 | -0.2306 | No |
| 56 | IFNA17 | interferon alpha 17 [Source:HGNC Symbol;Acc:HGNC:5422] | 18384 | -0.007 | -0.2675 | No |
| 57 | SPRY4 | sprouty RTK signaling antagonist 4 [Source:HGNC Symbol;Acc:HGNC:15533] | 19028 | -0.011 | -0.2834 | No |
| 58 | IFNA14 | interferon alpha 14 [Source:HGNC Symbol;Acc:HGNC:5420] | 19637 | -0.014 | -0.2982 | No |
| 59 | IFNGR1 | interferon gamma receptor 1 [Source:HGNC Symbol;Acc:HGNC:5439] | 19762 | -0.015 | -0.3005 | No |
| 60 | IL13 | interleukin 13 [Source:HGNC Symbol;Acc:HGNC:5973] | 20238 | -0.018 | -0.3117 | No |
| 61 | IFNA1 | interferon alpha 1 [Source:HGNC Symbol;Acc:HGNC:5417] | 20479 | -0.020 | -0.3168 | No |
| 62 | IL2 | interleukin 2 [Source:HGNC Symbol;Acc:HGNC:6001] | 20777 | -0.021 | -0.3233 | No |
| 63 | IL4R | interleukin 4 receptor [Source:HGNC Symbol;Acc:HGNC:6015] | 21312 | -0.025 | -0.3357 | No |
| 64 | IFNA6 | interferon alpha 6 [Source:HGNC Symbol;Acc:HGNC:5427] | 21419 | -0.025 | -0.3370 | No |
| 65 | IL20 | interleukin 20 [Source:HGNC Symbol;Acc:HGNC:6002] | 22182 | -0.030 | -0.3549 | No |
| 66 | IL12A | interleukin 12A [Source:HGNC Symbol;Acc:HGNC:5969] | 22276 | -0.030 | -0.3557 | No |
| 67 | IL23A | interleukin 23 subunit alpha [Source:HGNC Symbol;Acc:HGNC:15488] | 22882 | -0.034 | -0.3694 | No |
| 68 | IFNW1 | interferon omega 1 [Source:HGNC Symbol;Acc:HGNC:5448] | 23048 | -0.035 | -0.3718 | No |
| 69 | IL11 | interleukin 11 [Source:HGNC Symbol;Acc:HGNC:5966] | 23228 | -0.037 | -0.3744 | No |
| 70 | PTPN6 | protein tyrosine phosphatase non-receptor type 6 [Source:HGNC Symbol;Acc:HGNC:9658] | 23581 | -0.039 | -0.3814 | No |
| 71 | IFNA2 | interferon alpha 2 [Source:HGNC Symbol;Acc:HGNC:5423] | 23890 | -0.041 | -0.3871 | No |
| 72 | STAM2 | signal transducing adaptor molecule 2 [Source:HGNC Symbol;Acc:HGNC:11358] | 24080 | -0.042 | -0.3897 | No |
| 73 | IL5 | interleukin 5 [Source:HGNC Symbol;Acc:HGNC:6016] | 25260 | -0.050 | -0.4172 | No |
| 74 | CCND1 | cyclin D1 [Source:HGNC Symbol;Acc:HGNC:1582] | 25465 | -0.052 | -0.4197 | No |
| 75 | CSF2RA | colony stimulating factor 2 receptor subunit alpha [Source:HGNC Symbol;Acc:HGNC:2435] | 25630 | -0.053 | -0.4211 | No |
| 76 | TSLP | thymic stromal lymphopoietin [Source:HGNC Symbol;Acc:HGNC:30743] | 25864 | -0.055 | -0.4242 | No |
| 77 | GH1 | growth hormone 1 [Source:HGNC Symbol;Acc:HGNC:4261] | 26168 | -0.057 | -0.4290 | No |
| 78 | IFNA10 | interferon alpha 10 [Source:HGNC Symbol;Acc:HGNC:5418] | 26311 | -0.058 | -0.4296 | No |
| 79 | IL15 | interleukin 15 [Source:HGNC Symbol;Acc:HGNC:5977] | 27093 | -0.064 | -0.4463 | No |
| 80 | SPRY3 | sprouty RTK signaling antagonist 3 [Source:HGNC Symbol;Acc:HGNC:11271] | 27104 | -0.064 | -0.4432 | No |
| 81 | IFNAR2 | interferon alpha and beta receptor subunit 2 [Source:HGNC Symbol;Acc:HGNC:5433] | 27247 | -0.065 | -0.4434 | No |
| 82 | IL26 | interleukin 26 [Source:HGNC Symbol;Acc:HGNC:17119] | 27301 | -0.066 | -0.4414 | No |
| 83 | LEP | leptin [Source:HGNC Symbol;Acc:HGNC:6553] | 27432 | -0.067 | -0.4412 | No |
| 84 | IRF9 | interferon regulatory factor 9 [Source:HGNC Symbol;Acc:HGNC:6131] | 28411 | -0.075 | -0.4623 | No |
| 85 | IL22RA2 | interleukin 22 receptor subunit alpha 2 [Source:HGNC Symbol;Acc:HGNC:14901] | 28551 | -0.076 | -0.4619 | No |
| 86 | SOCS5 | suppressor of cytokine signaling 5 [Source:HGNC Symbol;Acc:HGNC:16852] | 29157 | -0.081 | -0.4732 | No |
| 87 | PIAS2 | protein inhibitor of activated STAT 2 [Source:HGNC Symbol;Acc:HGNC:17311] | 29251 | -0.082 | -0.4713 | No |
| 88 | PRL | prolactin [Source:HGNC Symbol;Acc:HGNC:9445] | 29427 | -0.084 | -0.4714 | No |
| 89 | IL22 | interleukin 22 [Source:HGNC Symbol;Acc:HGNC:14900] | 29805 | -0.088 | -0.4765 | No |
| 90 | GH2 | growth hormone 2 [Source:HGNC Symbol;Acc:HGNC:4262] | 30618 | -0.096 | -0.4922 | No |
| 91 | STAM | signal transducing adaptor molecule [Source:HGNC Symbol;Acc:HGNC:11357] | 30768 | -0.098 | -0.4909 | No |
| 92 | SPRED1 | sprouty related EVH1 domain containing 1 [Source:HGNC Symbol;Acc:HGNC:20249] | 30841 | -0.099 | -0.4876 | No |
| 93 | LIFR | LIF receptor subunit alpha [Source:HGNC Symbol;Acc:HGNC:6597] | 31193 | -0.103 | -0.4913 | No |
| 94 | STAT4 | signal transducer and activator of transcription 4 [Source:HGNC Symbol;Acc:HGNC:11365] | 31561 | -0.107 | -0.4950 | Yes |
| 95 | IL20RA | interleukin 20 receptor subunit alpha [Source:HGNC Symbol;Acc:HGNC:6003] | 31685 | -0.109 | -0.4925 | Yes |
| 96 | PIK3CD | "phosphatidylinositol-4,5-bisphosphate 3-kinase catalytic subunit delta [Source:HGNC Symbol;Acc:HGNC:8977]" | 31859 | -0.111 | -0.4912 | Yes |
| 97 | PIM1 | "Pim-1 proto-oncogene, serine/threonine kinase [Source:HGNC Symbol;Acc:HGNC:8986]" | 32147 | -0.115 | -0.4925 | Yes |
| 98 | IFNK | interferon kappa [Source:HGNC Symbol;Acc:HGNC:21714] | 32352 | -0.118 | -0.4916 | Yes |
| 99 | SOCS2 | suppressor of cytokine signaling 2 [Source:HGNC Symbol;Acc:HGNC:19382] | 32391 | -0.118 | -0.4865 | Yes |
| 100 | OSM | oncostatin M [Source:HGNC Symbol;Acc:HGNC:8506] | 32409 | -0.118 | -0.4807 | Yes |
| 101 | IL3RA | interleukin 3 receptor subunit alpha [Source:HGNC Symbol;Acc:HGNC:6012] | 32493 | -0.120 | -0.4766 | Yes |
| 102 | SOCS7 | suppressor of cytokine signaling 7 [Source:HGNC Symbol;Acc:HGNC:29846] | 32842 | -0.125 | -0.4790 | Yes |
| 103 | IL5RA | interleukin 5 receptor subunit alpha [Source:HGNC Symbol;Acc:HGNC:6017] | 33434 | -0.135 | -0.4871 | Yes |
| 104 | CSF3 | colony stimulating factor 3 [Source:HGNC Symbol;Acc:HGNC:2438] | 33514 | -0.137 | -0.4820 | Yes |
| 105 | IL13RA1 | interleukin 13 receptor subunit alpha 1 [Source:HGNC Symbol;Acc:HGNC:5974] | 33634 | -0.139 | -0.4778 | Yes |
| 106 | IL7 | interleukin 7 [Source:HGNC Symbol;Acc:HGNC:6023] | 33655 | -0.139 | -0.4711 | Yes |
| 107 | LIF | LIF interleukin 6 family cytokine [Source:HGNC Symbol;Acc:HGNC:6596] | 33750 | -0.141 | -0.4662 | Yes |
| 108 | GHR | growth hormone receptor [Source:HGNC Symbol;Acc:HGNC:4263] | 34005 | -0.145 | -0.4652 | Yes |
| 109 | IL6ST | interleukin 6 signal transducer [Source:HGNC Symbol;Acc:HGNC:6021] | 34111 | -0.148 | -0.4602 | Yes |
| 110 | IL24 | interleukin 24 [Source:HGNC Symbol;Acc:HGNC:11346] | 34254 | -0.150 | -0.4560 | Yes |
| 111 | IL9R | interleukin 9 receptor [Source:HGNC Symbol;Acc:HGNC:6030] | 34282 | -0.151 | -0.4489 | Yes |
| 112 | STAT3 | signal transducer and activator of transcription 3 [Source:HGNC Symbol;Acc:HGNC:11364] | 34312 | -0.151 | -0.4418 | Yes |
| 113 | LEPR | leptin receptor [Source:HGNC Symbol;Acc:HGNC:6554] | 34476 | -0.155 | -0.4379 | Yes |
| 114 | JAK3 | Janus kinase 3 [Source:HGNC Symbol;Acc:HGNC:6193] | 34533 | -0.155 | -0.4312 | Yes |
| 115 | IL15RA | interleukin 15 receptor subunit alpha [Source:HGNC Symbol;Acc:HGNC:5978] | 34548 | -0.156 | -0.4235 | Yes |
| 116 | SOS2 | SOS Ras/Rho guanine nucleotide exchange factor 2 [Source:HGNC Symbol;Acc:HGNC:11188] | 34598 | -0.157 | -0.4166 | Yes |
| 117 | PIK3R1 | phosphoinositide-3-kinase regulatory subunit 1 [Source:HGNC Symbol;Acc:HGNC:8979] | 34788 | -0.162 | -0.4130 | Yes |
| 118 | CREBBP | CREB binding protein [Source:HGNC Symbol;Acc:HGNC:2348] | 34790 | -0.162 | -0.4047 | Yes |
| 119 | PIK3CB | "phosphatidylinositol-4,5-bisphosphate 3-kinase catalytic subunit beta [Source:HGNC Symbol;Acc:HGNC:8976]" | 34871 | -0.164 | -0.3982 | Yes |
| 120 | SOCS3 | suppressor of cytokine signaling 3 [Source:HGNC Symbol;Acc:HGNC:19391] | 35446 | -0.178 | -0.4036 | Yes |
| 121 | STAT2 | signal transducer and activator of transcription 2 [Source:HGNC Symbol;Acc:HGNC:11363] | 35536 | -0.180 | -0.3965 | Yes |
| 122 | IFNG | interferon gamma [Source:HGNC Symbol;Acc:HGNC:5438] | 35652 | -0.183 | -0.3900 | Yes |
| 123 | CBLB | Cbl proto-oncogene B [Source:HGNC Symbol;Acc:HGNC:1542] | 35958 | -0.192 | -0.3878 | Yes |
| 124 | STAT5A | signal transducer and activator of transcription 5A [Source:HGNC Symbol;Acc:HGNC:11366] | 36022 | -0.195 | -0.3792 | Yes |
| 125 | CCND2 | cyclin D2 [Source:HGNC Symbol;Acc:HGNC:1583] | 36339 | -0.206 | -0.3766 | Yes |
| 126 | CSF3R | colony stimulating factor 3 receptor [Source:HGNC Symbol;Acc:HGNC:2439] | 36344 | -0.206 | -0.3660 | Yes |
| 127 | IL6 | interleukin 6 [Source:HGNC Symbol;Acc:HGNC:6018] | 36396 | -0.208 | -0.3565 | Yes |
| 128 | GRB2 | growth factor receptor bound protein 2 [Source:HGNC Symbol;Acc:HGNC:4566] | 36516 | -0.212 | -0.3486 | Yes |
| 129 | IL21 | interleukin 21 [Source:HGNC Symbol;Acc:HGNC:6005] | 36899 | -0.230 | -0.3464 | Yes |
| 130 | IL12RB2 | interleukin 12 receptor subunit beta 2 [Source:HGNC Symbol;Acc:HGNC:5972] | 37027 | -0.237 | -0.3373 | Yes |
| 131 | IL2RG | interleukin 2 receptor subunit gamma [Source:HGNC Symbol;Acc:HGNC:6010] | 37182 | -0.247 | -0.3284 | Yes |
| 132 | CNTF | ciliary neurotrophic factor [Source:HGNC Symbol;Acc:HGNC:2169] | 37188 | -0.247 | -0.3157 | Yes |
| 133 | IL12RB1 | interleukin 12 receptor subunit beta 1 [Source:HGNC Symbol;Acc:HGNC:5971] | 37256 | -0.251 | -0.3043 | Yes |
| 134 | STAT1 | signal transducer and activator of transcription 1 [Source:HGNC Symbol;Acc:HGNC:11362] | 37262 | -0.251 | -0.2914 | Yes |
| 135 | IL10 | interleukin 10 [Source:HGNC Symbol;Acc:HGNC:5962] | 37315 | -0.254 | -0.2796 | Yes |
| 136 | IL21R | interleukin 21 receptor [Source:HGNC Symbol;Acc:HGNC:6006] | 37376 | -0.258 | -0.2677 | Yes |
| 137 | EP300 | E1A binding protein p300 [Source:HGNC Symbol;Acc:HGNC:3373] | 37388 | -0.259 | -0.2546 | Yes |
| 138 | SOCS4 | suppressor of cytokine signaling 4 [Source:HGNC Symbol;Acc:HGNC:19392] | 37477 | -0.264 | -0.2431 | Yes |
| 139 | IL23R | interleukin 23 receptor [Source:HGNC Symbol;Acc:HGNC:19100] | 37504 | -0.266 | -0.2299 | Yes |
| 140 | PIK3CA | "phosphatidylinositol-4,5-bisphosphate 3-kinase catalytic subunit alpha [Source:HGNC Symbol;Acc:HGNC:8975]" | 37556 | -0.270 | -0.2172 | Yes |
| 141 | IL2RB | interleukin 2 receptor subunit beta [Source:HGNC Symbol;Acc:HGNC:6009] | 37681 | -0.282 | -0.2057 | Yes |
| 142 | STAT5B | signal transducer and activator of transcription 5B [Source:HGNC Symbol;Acc:HGNC:11367] | 37756 | -0.287 | -0.1927 | Yes |
| 143 | JAK1 | Janus kinase 1 [Source:HGNC Symbol;Acc:HGNC:6190] | 37793 | -0.290 | -0.1786 | Yes |
| 144 | IL7R | interleukin 7 receptor [Source:HGNC Symbol;Acc:HGNC:6024] | 37941 | -0.309 | -0.1663 | Yes |
| 145 | PIK3R5 | phosphoinositide-3-kinase regulatory subunit 5 [Source:HGNC Symbol;Acc:HGNC:30035] | 37956 | -0.310 | -0.1505 | Yes |
| 146 | PIAS1 | protein inhibitor of activated STAT 1 [Source:HGNC Symbol;Acc:HGNC:2752] | 37975 | -0.313 | -0.1347 | Yes |
| 147 | IL10RA | interleukin 10 receptor subunit alpha [Source:HGNC Symbol;Acc:HGNC:5964] | 38059 | -0.325 | -0.1199 | Yes |
| 148 | CBL | Cbl proto-oncogene [Source:HGNC Symbol;Acc:HGNC:1541] | 38098 | -0.332 | -0.1037 | Yes |
| 149 | IL2RA | interleukin 2 receptor subunit alpha [Source:HGNC Symbol;Acc:HGNC:6008] | 38105 | -0.335 | -0.0865 | Yes |
| 150 | SOS1 | SOS Ras/Rac guanine nucleotide exchange factor 1 [Source:HGNC Symbol;Acc:HGNC:11187] | 38132 | -0.341 | -0.0694 | Yes |
| 151 | CSF2RB | colony stimulating factor 2 receptor subunit beta [Source:HGNC Symbol;Acc:HGNC:2436] | 38193 | -0.360 | -0.0523 | Yes |
| 152 | AKT3 | AKT serine/threonine kinase 3 [Source:HGNC Symbol;Acc:HGNC:393] | 38196 | -0.362 | -0.0335 | Yes |
| 153 | JAK2 | Janus kinase 2 [Source:HGNC Symbol;Acc:HGNC:6192] | 38251 | -0.384 | -0.0149 | Yes |
| 154 | PTPN11 | protein tyrosine phosphatase non-receptor type 11 [Source:HGNC Symbol;Acc:HGNC:9644] | 38264 | -0.388 | 0.0049 | Yes |
| 155 | PIK3CG | "phosphatidylinositol-4,5-bisphosphate 3-kinase catalytic subunit gamma [Source:HGNC Symbol;Acc:HGNC:8978]" | 38302 | -0.426 | 0.0261 | Yes |
Table: GSEA details [plain text format]

  

Fig 2: KEGG\_JAK\_STAT\_SIGNALING\_PATHWAY      
 Blue-Pink O' Gram in the Space of the Analyzed GeneSet

  

Fig 3: KEGG\_JAK\_STAT\_SIGNALING\_PATHWAY: Random ES distribution      
 Gene set null distribution of ES for **KEGG\_JAK\_STAT\_SIGNALING\_PATHWAY**

  
