# Supplementary material for: Simultaneous Presentation of Multiple Myeloma and Lung Cancer: Case Report and Gene Bioinformatics Analysis
Source: Front Oncol. 2022 Jun 13;12:859735. doi: 10.3389/fonc.2022.859735 (PMC9235397; doi:10.3389/fonc.2022.859735)
Supplement: Supplementary file 1 [file DataSheet_1.zip › The bioinformatic analysis of MM and lung cancer supplementary materials/Enrichment analysis/MECR/GSEA_4.1.0/LUAD TCGA/KEGG.Gsea.1639041756227/KEGG_LEISHMANIA_INFECTION.html]

Details for gene set KEGG\_LEISHMANIA\_INFECTION[GSEA]

|  || Dataset | ExpData\_collapsed\_to\_symbols.ENSG00000116353\_profile\_in\_ExpData.cls #ENSG00000116353 |
| Phenotype | ENSG00000116353\_profile\_in\_ExpData.cls#ENSG00000116353 |
| Upregulated in class | ENSG00000116353\_neg |
| GeneSet | KEGG\_LEISHMANIA\_INFECTION |
| Enrichment Score (ES) | -0.59231794 |
| Normalized Enrichment Score (NES) | -2.3470647 |
| Nominal p-value | 0.0 |
| FDR q-value | 0.0 |
| FWER p-Value | 0.0 |
Table: GSEA Results Summary

  

Fig 1: Enrichment plot: KEGG\_LEISHMANIA\_INFECTION      
 Profile of the Running ES Score & Positions of GeneSet Members on the Rank Ordered List

  

| SYMBOL | TITLE | RANK IN GENE LIST | RANK METRIC SCORE | RUNNING ES | CORE ENRICHMENT || 1 | TAB1 | TGF-beta activated kinase 1 (MAP3K7) binding protein 1 [Source:HGNC Symbol;Acc:HGNC:18157] | 1258 | 0.251 | -0.0061 | No |
| 2 | MAPK3 | mitogen-activated protein kinase 3 [Source:HGNC Symbol;Acc:HGNC:6877] | 1645 | 0.226 | 0.0075 | No |
| 3 | IFNGR2 | interferon gamma receptor 2 [Source:HGNC Symbol;Acc:HGNC:5440] | 2702 | 0.176 | -0.0012 | No |
| 4 | HLA-DMA | "major histocompatibility complex, class II, DM alpha [Source:HGNC Symbol;Acc:HGNC:4934]" | 4341 | 0.126 | -0.0299 | No |
| 5 | JUN | "Jun proto-oncogene, AP-1 transcription factor subunit [Source:HGNC Symbol;Acc:HGNC:6204]" | 5045 | 0.110 | -0.0364 | No |
| 6 | NFKBIB | NFKB inhibitor beta [Source:HGNC Symbol;Acc:HGNC:7798] | 5484 | 0.102 | -0.0369 | No |
| 7 | RELA | "RELA proto-oncogene, NF-kB subunit [Source:HGNC Symbol;Acc:HGNC:9955]" | 6022 | 0.093 | -0.0409 | No |
| 8 | MAPK13 | mitogen-activated protein kinase 13 [Source:HGNC Symbol;Acc:HGNC:6875] | 6377 | 0.088 | -0.0409 | No |
| 9 | MARCKSL1 | MARCKS like 1 [Source:HGNC Symbol;Acc:HGNC:7142] | 7019 | 0.079 | -0.0490 | No |
| 10 | IL4 | interleukin 4 [Source:HGNC Symbol;Acc:HGNC:6014] | 7378 | 0.075 | -0.0504 | No |
| 11 | NFKBIA | NFKB inhibitor alpha [Source:HGNC Symbol;Acc:HGNC:7797] | 7696 | 0.071 | -0.0512 | No |
| 12 | CYBA | cytochrome b-245 alpha chain [Source:HGNC Symbol;Acc:HGNC:2577] | 8411 | 0.063 | -0.0628 | No |
| 13 | MAPK12 | mitogen-activated protein kinase 12 [Source:HGNC Symbol;Acc:HGNC:6874] | 9265 | 0.055 | -0.0788 | No |
| 14 | MAPK11 | mitogen-activated protein kinase 11 [Source:HGNC Symbol;Acc:HGNC:6873] | 9370 | 0.054 | -0.0759 | No |
| 15 | HLA-DRB1 | "major histocompatibility complex, class II, DR beta 1 [Source:HGNC Symbol;Acc:HGNC:4948]" | 10444 | 0.045 | -0.0985 | No |
| 16 | HLA-DRB5 | "major histocompatibility complex, class II, DR beta 5 [Source:HGNC Symbol;Acc:HGNC:4953]" | 10518 | 0.045 | -0.0957 | No |
| 17 | IRAK1 | interleukin 1 receptor associated kinase 1 [Source:HGNC Symbol;Acc:HGNC:6112] | 10971 | 0.041 | -0.1030 | No |
| 18 | IL12B | interleukin 12B [Source:HGNC Symbol;Acc:HGNC:5970] | 15071 | 0.013 | -0.2061 | No |
| 19 | HLA-DPB1 | "major histocompatibility complex, class II, DP beta 1 [Source:HGNC Symbol;Acc:HGNC:4940]" | 16356 | 0.005 | -0.2383 | No |
| 20 | HLA-DRA | "major histocompatibility complex, class II, DR alpha [Source:HGNC Symbol;Acc:HGNC:4947]" | 17663 | -0.003 | -0.2713 | No |
| 21 | HLA-DQB1 | "major histocompatibility complex, class II, DQ beta 1 [Source:HGNC Symbol;Acc:HGNC:4944]" | 17913 | -0.004 | -0.2771 | No |
| 22 | HLA-DQA2 | "major histocompatibility complex, class II, DQ alpha 2 [Source:HGNC Symbol;Acc:HGNC:4943]" | 19230 | -0.012 | -0.3094 | No |
| 23 | IFNGR1 | interferon gamma receptor 1 [Source:HGNC Symbol;Acc:HGNC:5439] | 19762 | -0.015 | -0.3213 | No |
| 24 | MYD88 | MYD88 innate immune signal transduction adaptor [Source:HGNC Symbol;Acc:HGNC:7562] | 19901 | -0.016 | -0.3232 | No |
| 25 | NCF4 | neutrophil cytosolic factor 4 [Source:HGNC Symbol;Acc:HGNC:7662] | 20418 | -0.019 | -0.3343 | No |
| 26 | HLA-DOB | "major histocompatibility complex, class II, DO beta [Source:HGNC Symbol;Acc:HGNC:4937]" | 20731 | -0.021 | -0.3401 | No |
| 27 | TLR2 | toll like receptor 2 [Source:HGNC Symbol;Acc:HGNC:11848] | 21828 | -0.028 | -0.3651 | No |
| 28 | IL12A | interleukin 12A [Source:HGNC Symbol;Acc:HGNC:5969] | 22276 | -0.030 | -0.3734 | No |
| 29 | TGFB1 | transforming growth factor beta 1 [Source:HGNC Symbol;Acc:HGNC:11766] | 22340 | -0.031 | -0.3718 | No |
| 30 | FOS | "Fos proto-oncogene, AP-1 transcription factor subunit [Source:HGNC Symbol;Acc:HGNC:3796]" | 23563 | -0.039 | -0.3989 | No |
| 31 | PTPN6 | protein tyrosine phosphatase non-receptor type 6 [Source:HGNC Symbol;Acc:HGNC:9658] | 23581 | -0.039 | -0.3952 | No |
| 32 | HLA-DPA1 | "major histocompatibility complex, class II, DP alpha 1 [Source:HGNC Symbol;Acc:HGNC:4938]" | 24356 | -0.044 | -0.4104 | No |
| 33 | HLA-DMB | "major histocompatibility complex, class II, DM beta [Source:HGNC Symbol;Acc:HGNC:4935]" | 24935 | -0.048 | -0.4201 | No |
| 34 | MAPK14 | mitogen-activated protein kinase 14 [Source:HGNC Symbol;Acc:HGNC:6876] | 25018 | -0.049 | -0.4171 | No |
| 35 | C3 | complement C3 [Source:HGNC Symbol;Acc:HGNC:1318] | 25059 | -0.049 | -0.4131 | No |
| 36 | TGFB2 | transforming growth factor beta 2 [Source:HGNC Symbol;Acc:HGNC:11768] | 26001 | -0.056 | -0.4312 | No |
| 37 | ELK1 | ETS transcription factor ELK1 [Source:HGNC Symbol;Acc:HGNC:3321] | 26567 | -0.060 | -0.4394 | No |
| 38 | PTGS2 | prostaglandin-endoperoxide synthase 2 [Source:HGNC Symbol;Acc:HGNC:9605] | 32570 | -0.121 | -0.5798 | Yes |
| 39 | HLA-DQA1 | "major histocompatibility complex, class II, DQ alpha 1 [Source:HGNC Symbol;Acc:HGNC:4942]" | 32898 | -0.126 | -0.5750 | Yes |
| 40 | HLA-DOA | "major histocompatibility complex, class II, DO alpha [Source:HGNC Symbol;Acc:HGNC:4936]" | 33498 | -0.136 | -0.5762 | Yes |
| 41 | MAPK1 | mitogen-activated protein kinase 1 [Source:HGNC Symbol;Acc:HGNC:6871] | 33636 | -0.139 | -0.5653 | Yes |
| 42 | ITGB2 | integrin subunit beta 2 [Source:HGNC Symbol;Acc:HGNC:6155] | 33691 | -0.139 | -0.5522 | Yes |
| 43 | NCF2 | neutrophil cytosolic factor 2 [Source:HGNC Symbol;Acc:HGNC:7661] | 33711 | -0.140 | -0.5382 | Yes |
| 44 | TNF | tumor necrosis factor [Source:HGNC Symbol;Acc:HGNC:11892] | 33732 | -0.140 | -0.5242 | Yes |
| 45 | NOS2 | nitric oxide synthase 2 [Source:HGNC Symbol;Acc:HGNC:7873] | 33997 | -0.145 | -0.5158 | Yes |
| 46 | IL1A | interleukin 1 alpha [Source:HGNC Symbol;Acc:HGNC:5991] | 34080 | -0.147 | -0.5027 | Yes |
| 47 | FCGR1A | Fc fragment of IgG receptor Ia [Source:HGNC Symbol;Acc:HGNC:3613] | 34659 | -0.159 | -0.5010 | Yes |
| 48 | MAP3K7 | mitogen-activated protein kinase kinase kinase 7 [Source:HGNC Symbol;Acc:HGNC:6859] | 34786 | -0.162 | -0.4874 | Yes |
| 49 | FCGR2A | Fc fragment of IgG receptor IIa [Source:HGNC Symbol;Acc:HGNC:3616] | 34941 | -0.165 | -0.4742 | Yes |
| 50 | TAB2 | TGF-beta activated kinase 1 (MAP3K7) binding protein 2 [Source:HGNC Symbol;Acc:HGNC:17075] | 35022 | -0.167 | -0.4590 | Yes |
| 51 | IFNG | interferon gamma [Source:HGNC Symbol;Acc:HGNC:5438] | 35652 | -0.183 | -0.4560 | Yes |
| 52 | TGFB3 | transforming growth factor beta 3 [Source:HGNC Symbol;Acc:HGNC:11769] | 35787 | -0.187 | -0.4400 | Yes |
| 53 | NFKB1 | nuclear factor kappa B subunit 1 [Source:HGNC Symbol;Acc:HGNC:7794] | 36367 | -0.207 | -0.4333 | Yes |
| 54 | FCGR2C | Fc fragment of IgG receptor IIc (gene/pseudogene) [Source:HGNC Symbol;Acc:HGNC:15626] | 36485 | -0.211 | -0.4145 | Yes |
| 55 | ITGAM | integrin subunit alpha M [Source:HGNC Symbol;Acc:HGNC:6149] | 36644 | -0.218 | -0.3960 | Yes |
| 56 | NCF1 | neutrophil cytosolic factor 1 [Source:HGNC Symbol;Acc:HGNC:7660] | 36975 | -0.234 | -0.3801 | Yes |
| 57 | IL1B | interleukin 1 beta [Source:HGNC Symbol;Acc:HGNC:5992] | 37201 | -0.248 | -0.3601 | Yes |
| 58 | STAT1 | signal transducer and activator of transcription 1 [Source:HGNC Symbol;Acc:HGNC:11362] | 37262 | -0.251 | -0.3356 | Yes |
| 59 | IL10 | interleukin 10 [Source:HGNC Symbol;Acc:HGNC:5962] | 37315 | -0.254 | -0.3106 | Yes |
| 60 | FCGR3A | Fc fragment of IgG receptor IIIa [Source:HGNC Symbol;Acc:HGNC:3619] | 37450 | -0.263 | -0.2868 | Yes |
| 61 | IRAK4 | interleukin 1 receptor associated kinase 4 [Source:HGNC Symbol;Acc:HGNC:17967] | 37489 | -0.265 | -0.2603 | Yes |
| 62 | TRAF6 | TNF receptor associated factor 6 [Source:HGNC Symbol;Acc:HGNC:12036] | 37748 | -0.286 | -0.2371 | Yes |
| 63 | JAK1 | Janus kinase 1 [Source:HGNC Symbol;Acc:HGNC:6190] | 37793 | -0.290 | -0.2082 | Yes |
| 64 | PRKCB | protein kinase C beta [Source:HGNC Symbol;Acc:HGNC:9395] | 37822 | -0.293 | -0.1785 | Yes |
| 65 | ITGB1 | integrin subunit beta 1 [Source:HGNC Symbol;Acc:HGNC:6153] | 37850 | -0.297 | -0.1485 | Yes |
| 66 | FCGR3B | Fc fragment of IgG receptor IIIb [Source:HGNC Symbol;Acc:HGNC:3620] | 37877 | -0.299 | -0.1181 | Yes |
| 67 | TLR4 | toll like receptor 4 [Source:HGNC Symbol;Acc:HGNC:11850] | 38140 | -0.345 | -0.0891 | Yes |
| 68 | CR1 | complement C3b/C4b receptor 1 (Knops blood group) [Source:HGNC Symbol;Acc:HGNC:2334] | 38221 | -0.371 | -0.0527 | Yes |
| 69 | JAK2 | Janus kinase 2 [Source:HGNC Symbol;Acc:HGNC:6192] | 38251 | -0.384 | -0.0136 | Yes |
| 70 | ITGA4 | integrin subunit alpha 4 [Source:HGNC Symbol;Acc:HGNC:6140] | 38275 | -0.394 | 0.0267 | Yes |
Table: GSEA details [plain text format]

  

Fig 2: KEGG\_LEISHMANIA\_INFECTION      
 Blue-Pink O' Gram in the Space of the Analyzed GeneSet

  

Fig 3: KEGG\_LEISHMANIA\_INFECTION: Random ES distribution      
 Gene set null distribution of ES for **KEGG\_LEISHMANIA\_INFECTION**

  
