# Supplementary material for: Simultaneous Presentation of Multiple Myeloma and Lung Cancer: Case Report and Gene Bioinformatics Analysis
Source: Front Oncol. 2022 Jun 13;12:859735. doi: 10.3389/fonc.2022.859735 (PMC9235397; doi:10.3389/fonc.2022.859735)
Supplement: Supplementary file 1 [file DataSheet_1.zip › The bioinformatic analysis of MM and lung cancer supplementary materials/Enrichment analysis/MECR/GSEA_4.1.0/LUAD TCGA/KEGG.Gsea.1639041756227/KEGG_LEUKOCYTE_TRANSENDOTHELIAL_MIGRATION.html]

Details for gene set KEGG\_LEUKOCYTE\_TRANSENDOTHELIAL\_MIGRATION[GSEA]

|  || Dataset | ExpData\_collapsed\_to\_symbols.ENSG00000116353\_profile\_in\_ExpData.cls #ENSG00000116353 |
| Phenotype | ENSG00000116353\_profile\_in\_ExpData.cls#ENSG00000116353 |
| Upregulated in class | ENSG00000116353\_neg |
| GeneSet | KEGG\_LEUKOCYTE\_TRANSENDOTHELIAL\_MIGRATION |
| Enrichment Score (ES) | -0.479639 |
| Normalized Enrichment Score (NES) | -2.0367258 |
| Nominal p-value | 0.0 |
| FDR q-value | 3.741956E-4 |
| FWER p-Value | 0.006 |
Table: GSEA Results Summary

  

Fig 1: Enrichment plot: KEGG\_LEUKOCYTE\_TRANSENDOTHELIAL\_MIGRATION      
 Profile of the Running ES Score & Positions of GeneSet Members on the Rank Ordered List

  

| SYMBOL | TITLE | RANK IN GENE LIST | RANK METRIC SCORE | RUNNING ES | CORE ENRICHMENT || 1 | PIK3R2 | phosphoinositide-3-kinase regulatory subunit 2 [Source:HGNC Symbol;Acc:HGNC:8980] | 545 | 0.314 | 0.0046 | No |
| 2 | MYL5 | myosin light chain 5 [Source:HGNC Symbol;Acc:HGNC:7586] | 841 | 0.284 | 0.0138 | No |
| 3 | CLDN7 | claudin 7 [Source:HGNC Symbol;Acc:HGNC:2049] | 1249 | 0.251 | 0.0183 | No |
| 4 | CLDN4 | claudin 4 [Source:HGNC Symbol;Acc:HGNC:2046] | 1316 | 0.246 | 0.0311 | No |
| 5 | MYLPF | "myosin light chain, phosphorylatable, fast skeletal muscle [Source:HGNC Symbol;Acc:HGNC:29824]" | 1542 | 0.232 | 0.0391 | No |
| 6 | SIPA1 | signal-induced proliferation-associated 1 [Source:HGNC Symbol;Acc:HGNC:10885] | 1996 | 0.206 | 0.0397 | No |
| 7 | MYL12B | myosin light chain 12B [Source:HGNC Symbol;Acc:HGNC:29827] | 2099 | 0.201 | 0.0489 | No |
| 8 | CDC42 | cell division cycle 42 [Source:HGNC Symbol;Acc:HGNC:1736] | 2158 | 0.198 | 0.0592 | No |
| 9 | CLDN3 | claudin 3 [Source:HGNC Symbol;Acc:HGNC:2045] | 2175 | 0.198 | 0.0704 | No |
| 10 | RAC1 | Rac family small GTPase 1 [Source:HGNC Symbol;Acc:HGNC:9801] | 2566 | 0.182 | 0.0712 | No |
| 11 | CLDN6 | claudin 6 [Source:HGNC Symbol;Acc:HGNC:2048] | 3000 | 0.164 | 0.0698 | No |
| 12 | CLDN9 | claudin 9 [Source:HGNC Symbol;Acc:HGNC:2051] | 3014 | 0.163 | 0.0791 | No |
| 13 | F11R | F11 receptor [Source:HGNC Symbol;Acc:HGNC:14685] | 3831 | 0.139 | 0.0665 | No |
| 14 | MYL12A | myosin light chain 12A [Source:HGNC Symbol;Acc:HGNC:16701] | 3874 | 0.138 | 0.0735 | No |
| 15 | ACTB | actin beta [Source:HGNC Symbol;Acc:HGNC:132] | 4499 | 0.122 | 0.0647 | No |
| 16 | BCAR1 | "BCAR1 scaffold protein, Cas family member [Source:HGNC Symbol;Acc:HGNC:971]" | 4546 | 0.121 | 0.0707 | No |
| 17 | CLDN8 | claudin 8 [Source:HGNC Symbol;Acc:HGNC:2050] | 5034 | 0.111 | 0.0648 | No |
| 18 | NOX1 | NADPH oxidase 1 [Source:HGNC Symbol;Acc:HGNC:7889] | 5067 | 0.110 | 0.0705 | No |
| 19 | CLDN15 | claudin 15 [Source:HGNC Symbol;Acc:HGNC:2036] | 5297 | 0.106 | 0.0708 | No |
| 20 | ACTG1 | actin gamma 1 [Source:HGNC Symbol;Acc:HGNC:144] | 5516 | 0.102 | 0.0713 | No |
| 21 | EZR | ezrin [Source:HGNC Symbol;Acc:HGNC:12691] | 6125 | 0.091 | 0.0612 | No |
| 22 | CLDN23 | claudin 23 [Source:HGNC Symbol;Acc:HGNC:17591] | 6209 | 0.090 | 0.0644 | No |
| 23 | MAPK13 | mitogen-activated protein kinase 13 [Source:HGNC Symbol;Acc:HGNC:6875] | 6377 | 0.088 | 0.0653 | No |
| 24 | PTK2 | protein tyrosine kinase 2 [Source:HGNC Symbol;Acc:HGNC:9611] | 6431 | 0.087 | 0.0691 | No |
| 25 | ICAM1 | intercellular adhesion molecule 1 [Source:HGNC Symbol;Acc:HGNC:5344] | 8090 | 0.067 | 0.0307 | No |
| 26 | CYBA | cytochrome b-245 alpha chain [Source:HGNC Symbol;Acc:HGNC:2577] | 8411 | 0.063 | 0.0263 | No |
| 27 | PXN | paxillin [Source:HGNC Symbol;Acc:HGNC:9718] | 8446 | 0.063 | 0.0291 | No |
| 28 | CTNNA1 | catenin alpha 1 [Source:HGNC Symbol;Acc:HGNC:2509] | 8967 | 0.058 | 0.0192 | No |
| 29 | PIK3R3 | phosphoinositide-3-kinase regulatory subunit 3 [Source:HGNC Symbol;Acc:HGNC:8981] | 9028 | 0.057 | 0.0211 | No |
| 30 | MAPK12 | mitogen-activated protein kinase 12 [Source:HGNC Symbol;Acc:HGNC:6874] | 9265 | 0.055 | 0.0183 | No |
| 31 | MAPK11 | mitogen-activated protein kinase 11 [Source:HGNC Symbol;Acc:HGNC:6873] | 9370 | 0.054 | 0.0188 | No |
| 32 | CLDN16 | claudin 16 [Source:HGNC Symbol;Acc:HGNC:2037] | 9621 | 0.052 | 0.0155 | No |
| 33 | MSN | moesin [Source:HGNC Symbol;Acc:HGNC:7373] | 10857 | 0.042 | -0.0135 | No |
| 34 | GNAI1 | G protein subunit alpha i1 [Source:HGNC Symbol;Acc:HGNC:4384] | 11243 | 0.039 | -0.0210 | No |
| 35 | RHOA | ras homolog family member A [Source:HGNC Symbol;Acc:HGNC:667] | 11437 | 0.037 | -0.0238 | No |
| 36 | CLDN17 | claudin 17 [Source:HGNC Symbol;Acc:HGNC:2038] | 12140 | 0.032 | -0.0398 | No |
| 37 | MYL9 | myosin light chain 9 [Source:HGNC Symbol;Acc:HGNC:15754] | 12602 | 0.029 | -0.0498 | No |
| 38 | MYL10 | myosin light chain 10 [Source:HGNC Symbol;Acc:HGNC:29825] | 13208 | 0.024 | -0.0638 | No |
| 39 | RAPGEF3 | Rap guanine nucleotide exchange factor 3 [Source:HGNC Symbol;Acc:HGNC:16629] | 13416 | 0.023 | -0.0678 | No |
| 40 | CLDN5 | claudin 5 [Source:HGNC Symbol;Acc:HGNC:2047] | 15366 | 0.011 | -0.1168 | No |
| 41 | MYL7 | myosin light chain 7 [Source:HGNC Symbol;Acc:HGNC:21719] | 15382 | 0.011 | -0.1166 | No |
| 42 | GNAI2 | G protein subunit alpha i2 [Source:HGNC Symbol;Acc:HGNC:4385] | 15695 | 0.009 | -0.1240 | No |
| 43 | MYL2 | myosin light chain 2 [Source:HGNC Symbol;Acc:HGNC:7583] | 18201 | -0.006 | -0.1876 | No |
| 44 | CTNNA2 | catenin alpha 2 [Source:HGNC Symbol;Acc:HGNC:2510] | 18724 | -0.009 | -0.2003 | No |
| 45 | OCLN | occludin [Source:HGNC Symbol;Acc:HGNC:8104] | 18739 | -0.009 | -0.2001 | No |
| 46 | ACTN4 | actinin alpha 4 [Source:HGNC Symbol;Acc:HGNC:166] | 19317 | -0.013 | -0.2141 | No |
| 47 | NCF4 | neutrophil cytosolic factor 4 [Source:HGNC Symbol;Acc:HGNC:7662] | 20418 | -0.019 | -0.2410 | No |
| 48 | CTNNA3 | catenin alpha 3 [Source:HGNC Symbol;Acc:HGNC:2511] | 20543 | -0.020 | -0.2430 | No |
| 49 | RAP1A | "RAP1A, member of RAS oncogene family [Source:HGNC Symbol;Acc:HGNC:9855]" | 20560 | -0.020 | -0.2422 | No |
| 50 | CD99 | CD99 molecule (Xg blood group) [Source:HGNC Symbol;Acc:HGNC:7082] | 21637 | -0.027 | -0.2681 | No |
| 51 | VASP | vasodilator stimulated phosphoprotein [Source:HGNC Symbol;Acc:HGNC:12652] | 22726 | -0.033 | -0.2939 | No |
| 52 | CLDN1 | claudin 1 [Source:HGNC Symbol;Acc:HGNC:2032] | 22989 | -0.035 | -0.2985 | No |
| 53 | RAC2 | Rac family small GTPase 2 [Source:HGNC Symbol;Acc:HGNC:9802] | 24228 | -0.043 | -0.3275 | No |
| 54 | VAV2 | vav guanine nucleotide exchange factor 2 [Source:HGNC Symbol;Acc:HGNC:12658] | 24578 | -0.046 | -0.3337 | No |
| 55 | MAPK14 | mitogen-activated protein kinase 14 [Source:HGNC Symbol;Acc:HGNC:6876] | 25018 | -0.049 | -0.3421 | No |
| 56 | ESAM | endothelial cell adhesion molecule [Source:HGNC Symbol;Acc:HGNC:17474] | 26396 | -0.059 | -0.3737 | No |
| 57 | THY1 | Thy-1 cell surface antigen [Source:HGNC Symbol;Acc:HGNC:11801] | 28091 | -0.072 | -0.4127 | No |
| 58 | CLDN14 | claudin 14 [Source:HGNC Symbol;Acc:HGNC:2035] | 28510 | -0.076 | -0.4189 | No |
| 59 | CLDN2 | claudin 2 [Source:HGNC Symbol;Acc:HGNC:2041] | 28925 | -0.079 | -0.4248 | No |
| 60 | VAV3 | vav guanine nucleotide exchange factor 3 [Source:HGNC Symbol;Acc:HGNC:12659] | 29000 | -0.080 | -0.4220 | No |
| 61 | CTNNB1 | catenin beta 1 [Source:HGNC Symbol;Acc:HGNC:2514] | 29356 | -0.083 | -0.4261 | No |
| 62 | GNAI3 | G protein subunit alpha i3 [Source:HGNC Symbol;Acc:HGNC:4387] | 30483 | -0.095 | -0.4493 | No |
| 63 | NOX3 | NADPH oxidase 3 [Source:HGNC Symbol;Acc:HGNC:7890] | 30690 | -0.097 | -0.4488 | No |
| 64 | PLCG1 | phospholipase C gamma 1 [Source:HGNC Symbol;Acc:HGNC:9065] | 30759 | -0.098 | -0.4448 | No |
| 65 | PRKCG | protein kinase C gamma [Source:HGNC Symbol;Acc:HGNC:9402] | 31522 | -0.107 | -0.4579 | No |
| 66 | ACTN3 | actinin alpha 3 [Source:HGNC Symbol;Acc:HGNC:165] | 31627 | -0.108 | -0.4542 | No |
| 67 | PIK3CD | "phosphatidylinositol-4,5-bisphosphate 3-kinase catalytic subunit delta [Source:HGNC Symbol;Acc:HGNC:8977]" | 31859 | -0.111 | -0.4535 | No |
| 68 | MMP9 | matrix metallopeptidase 9 [Source:HGNC Symbol;Acc:HGNC:7176] | 32051 | -0.114 | -0.4517 | No |
| 69 | PRKCA | protein kinase C alpha [Source:HGNC Symbol;Acc:HGNC:9393] | 32080 | -0.114 | -0.4457 | No |
| 70 | ACTN2 | actinin alpha 2 [Source:HGNC Symbol;Acc:HGNC:164] | 32475 | -0.120 | -0.4487 | No |
| 71 | ITGB2 | integrin subunit beta 2 [Source:HGNC Symbol;Acc:HGNC:6155] | 33691 | -0.139 | -0.4714 | Yes |
| 72 | NCF2 | neutrophil cytosolic factor 2 [Source:HGNC Symbol;Acc:HGNC:7661] | 33711 | -0.140 | -0.4637 | Yes |
| 73 | AFDN | "afadin, adherens junction formation factor [Source:HGNC Symbol;Acc:HGNC:7137]" | 33852 | -0.142 | -0.4588 | Yes |
| 74 | CLDN19 | claudin 19 [Source:HGNC Symbol;Acc:HGNC:2040] | 33900 | -0.143 | -0.4516 | Yes |
| 75 | CLDN11 | claudin 11 [Source:HGNC Symbol;Acc:HGNC:8514] | 34044 | -0.146 | -0.4466 | Yes |
| 76 | RASSF5 | Ras association domain family member 5 [Source:HGNC Symbol;Acc:HGNC:17609] | 34138 | -0.148 | -0.4402 | Yes |
| 77 | CLDN18 | claudin 18 [Source:HGNC Symbol;Acc:HGNC:2039] | 34500 | -0.155 | -0.4403 | Yes |
| 78 | JAM2 | junctional adhesion molecule 2 [Source:HGNC Symbol;Acc:HGNC:14686] | 34555 | -0.156 | -0.4325 | Yes |
| 79 | RAP1B | "RAP1B, member of RAS oncogene family [Source:HGNC Symbol;Acc:HGNC:9857]" | 34571 | -0.156 | -0.4236 | Yes |
| 80 | PIK3R1 | phosphoinositide-3-kinase regulatory subunit 1 [Source:HGNC Symbol;Acc:HGNC:8979] | 34788 | -0.162 | -0.4196 | Yes |
| 81 | VAV1 | vav guanine nucleotide exchange factor 1 [Source:HGNC Symbol;Acc:HGNC:12657] | 34853 | -0.163 | -0.4116 | Yes |
| 82 | CLDN10 | claudin 10 [Source:HGNC Symbol;Acc:HGNC:2033] | 34859 | -0.163 | -0.4021 | Yes |
| 83 | PIK3CB | "phosphatidylinositol-4,5-bisphosphate 3-kinase catalytic subunit beta [Source:HGNC Symbol;Acc:HGNC:8976]" | 34871 | -0.164 | -0.3928 | Yes |
| 84 | CTNND1 | catenin delta 1 [Source:HGNC Symbol;Acc:HGNC:2515] | 35375 | -0.176 | -0.3952 | Yes |
| 85 | MMP2 | matrix metallopeptidase 2 [Source:HGNC Symbol;Acc:HGNC:7166] | 35423 | -0.177 | -0.3860 | Yes |
| 86 | ARHGAP35 | Rho GTPase activating protein 35 [Source:HGNC Symbol;Acc:HGNC:4591] | 35598 | -0.182 | -0.3797 | Yes |
| 87 | ARHGAP5 | Rho GTPase activating protein 5 [Source:HGNC Symbol;Acc:HGNC:675] | 35738 | -0.186 | -0.3723 | Yes |
| 88 | CLDN20 | claudin 20 [Source:HGNC Symbol;Acc:HGNC:2042] | 35870 | -0.190 | -0.3644 | Yes |
| 89 | CXCL12 | C-X-C motif chemokine ligand 12 [Source:HGNC Symbol;Acc:HGNC:10672] | 35899 | -0.191 | -0.3539 | Yes |
| 90 | VCL | vinculin [Source:HGNC Symbol;Acc:HGNC:12665] | 35933 | -0.192 | -0.3435 | Yes |
| 91 | ACTN1 | actinin alpha 1 [Source:HGNC Symbol;Acc:HGNC:163] | 36070 | -0.196 | -0.3354 | Yes |
| 92 | CDH5 | cadherin 5 [Source:HGNC Symbol;Acc:HGNC:1764] | 36210 | -0.201 | -0.3271 | Yes |
| 93 | CXCR4 | C-X-C motif chemokine receptor 4 [Source:HGNC Symbol;Acc:HGNC:2561] | 36579 | -0.215 | -0.3238 | Yes |
| 94 | ITGAM | integrin subunit alpha M [Source:HGNC Symbol;Acc:HGNC:6149] | 36644 | -0.218 | -0.3126 | Yes |
| 95 | NCF1 | neutrophil cytosolic factor 1 [Source:HGNC Symbol;Acc:HGNC:7660] | 36975 | -0.234 | -0.3072 | Yes |
| 96 | PTK2B | protein tyrosine kinase 2 beta [Source:HGNC Symbol;Acc:HGNC:9612] | 37013 | -0.237 | -0.2942 | Yes |
| 97 | JAM3 | junctional adhesion molecule 3 [Source:HGNC Symbol;Acc:HGNC:15532] | 37103 | -0.242 | -0.2822 | Yes |
| 98 | RAPGEF4 | Rap guanine nucleotide exchange factor 4 [Source:HGNC Symbol;Acc:HGNC:16626] | 37241 | -0.250 | -0.2710 | Yes |
| 99 | VCAM1 | vascular cell adhesion molecule 1 [Source:HGNC Symbol;Acc:HGNC:12663] | 37250 | -0.250 | -0.2564 | Yes |
| 100 | PECAM1 | platelet and endothelial cell adhesion molecule 1 [Source:HGNC Symbol;Acc:HGNC:8823] | 37427 | -0.261 | -0.2455 | Yes |
| 101 | PIK3CA | "phosphatidylinositol-4,5-bisphosphate 3-kinase catalytic subunit alpha [Source:HGNC Symbol;Acc:HGNC:8975]" | 37556 | -0.270 | -0.2328 | Yes |
| 102 | TXK | TXK tyrosine kinase [Source:HGNC Symbol;Acc:HGNC:12434] | 37579 | -0.272 | -0.2173 | Yes |
| 103 | ITGAL | integrin subunit alpha L [Source:HGNC Symbol;Acc:HGNC:6148] | 37624 | -0.276 | -0.2022 | Yes |
| 104 | PRKCB | protein kinase C beta [Source:HGNC Symbol;Acc:HGNC:9395] | 37822 | -0.293 | -0.1899 | Yes |
| 105 | ITGB1 | integrin subunit beta 1 [Source:HGNC Symbol;Acc:HGNC:6153] | 37850 | -0.297 | -0.1731 | Yes |
| 106 | RHOH | ras homolog family member H [Source:HGNC Symbol;Acc:HGNC:686] | 37911 | -0.304 | -0.1567 | Yes |
| 107 | PIK3R5 | phosphoinositide-3-kinase regulatory subunit 5 [Source:HGNC Symbol;Acc:HGNC:30035] | 37956 | -0.310 | -0.1395 | Yes |
| 108 | CYBB | cytochrome b-245 beta chain [Source:HGNC Symbol;Acc:HGNC:2578] | 38023 | -0.320 | -0.1223 | Yes |
| 109 | ITK | IL2 inducible T cell kinase [Source:HGNC Symbol;Acc:HGNC:6171] | 38024 | -0.320 | -0.1034 | Yes |
| 110 | PLCG2 | phospholipase C gamma 2 [Source:HGNC Symbol;Acc:HGNC:9066] | 38119 | -0.338 | -0.0859 | Yes |
| 111 | ROCK2 | Rho associated coiled-coil containing protein kinase 2 [Source:HGNC Symbol;Acc:HGNC:10252] | 38215 | -0.370 | -0.0665 | Yes |
| 112 | ROCK1 | Rho associated coiled-coil containing protein kinase 1 [Source:HGNC Symbol;Acc:HGNC:10251] | 38260 | -0.387 | -0.0448 | Yes |
| 113 | PTPN11 | protein tyrosine phosphatase non-receptor type 11 [Source:HGNC Symbol;Acc:HGNC:9644] | 38264 | -0.388 | -0.0220 | Yes |
| 114 | ITGA4 | integrin subunit alpha 4 [Source:HGNC Symbol;Acc:HGNC:6140] | 38275 | -0.394 | 0.0010 | Yes |
| 115 | PIK3CG | "phosphatidylinositol-4,5-bisphosphate 3-kinase catalytic subunit gamma [Source:HGNC Symbol;Acc:HGNC:8978]" | 38302 | -0.426 | 0.0254 | Yes |
| 116 | CLDN22 | claudin 22 [Source:HGNC Symbol;Acc:HGNC:2044] | 39071 | NaN | 0.0064 | Yes |
Table: GSEA details [plain text format]

  

Fig 2: KEGG\_LEUKOCYTE\_TRANSENDOTHELIAL\_MIGRATION      
 Blue-Pink O' Gram in the Space of the Analyzed GeneSet

  

Fig 3: KEGG\_LEUKOCYTE\_TRANSENDOTHELIAL\_MIGRATION: Random ES distribution      
 Gene set null distribution of ES for **KEGG\_LEUKOCYTE\_TRANSENDOTHELIAL\_MIGRATION**

  
