# Supplementary material for: Simultaneous Presentation of Multiple Myeloma and Lung Cancer: Case Report and Gene Bioinformatics Analysis
Source: Front Oncol. 2022 Jun 13;12:859735. doi: 10.3389/fonc.2022.859735 (PMC9235397; doi:10.3389/fonc.2022.859735)
Supplement: Supplementary file 1 [file DataSheet_1.zip › The bioinformatic analysis of MM and lung cancer supplementary materials/Enrichment analysis/MECR/GSEA_4.1.0/LUAD TCGA/KEGG.Gsea.1639041756227/KEGG_NATURAL_KILLER_CELL_MEDIATED_CYTOTOXICITY.html]

Details for gene set KEGG\_NATURAL\_KILLER\_CELL\_MEDIATED\_CYTOTOXICITY[GSEA]

|  || Dataset | ExpData\_collapsed\_to\_symbols.ENSG00000116353\_profile\_in\_ExpData.cls #ENSG00000116353 |
| Phenotype | ENSG00000116353\_profile\_in\_ExpData.cls#ENSG00000116353 |
| Upregulated in class | ENSG00000116353\_neg |
| GeneSet | KEGG\_NATURAL\_KILLER\_CELL\_MEDIATED\_CYTOTOXICITY |
| Enrichment Score (ES) | -0.5144807 |
| Normalized Enrichment Score (NES) | -2.2407413 |
| Nominal p-value | 0.0 |
| FDR q-value | 0.0 |
| FWER p-Value | 0.0 |
Table: GSEA Results Summary

  

Fig 1: Enrichment plot: KEGG\_NATURAL\_KILLER\_CELL\_MEDIATED\_CYTOTOXICITY      
 Profile of the Running ES Score & Positions of GeneSet Members on the Rank Ordered List

  

| SYMBOL | TITLE | RANK IN GENE LIST | RANK METRIC SCORE | RUNNING ES | CORE ENRICHMENT || 1 | RAC3 | Rac family small GTPase 3 [Source:HGNC Symbol;Acc:HGNC:9803] | 528 | 0.316 | 0.0046 | No |
| 2 | PIK3R2 | phosphoinositide-3-kinase regulatory subunit 2 [Source:HGNC Symbol;Acc:HGNC:8980] | 545 | 0.314 | 0.0222 | No |
| 3 | HRAS | "HRas proto-oncogene, GTPase [Source:HGNC Symbol;Acc:HGNC:5173]" | 1513 | 0.234 | 0.0109 | No |
| 4 | MAPK3 | mitogen-activated protein kinase 3 [Source:HGNC Symbol;Acc:HGNC:6877] | 1645 | 0.226 | 0.0206 | No |
| 5 | MAP2K2 | mitogen-activated protein kinase kinase 2 [Source:HGNC Symbol;Acc:HGNC:6842] | 1791 | 0.218 | 0.0293 | No |
| 6 | RAC1 | Rac family small GTPase 1 [Source:HGNC Symbol;Acc:HGNC:9801] | 2566 | 0.182 | 0.0200 | No |
| 7 | IFNGR2 | interferon gamma receptor 2 [Source:HGNC Symbol;Acc:HGNC:5440] | 2702 | 0.176 | 0.0266 | No |
| 8 | NFATC4 | nuclear factor of activated T cells 4 [Source:HGNC Symbol;Acc:HGNC:7778] | 2871 | 0.169 | 0.0320 | No |
| 9 | MICA | MHC class I polypeptide-related sequence A [Source:HGNC Symbol;Acc:HGNC:7090] | 4344 | 0.125 | 0.0017 | No |
| 10 | HLA-A | "major histocompatibility complex, class I, A [Source:HGNC Symbol;Acc:HGNC:4931]" | 4980 | 0.111 | -0.0082 | No |
| 11 | PPP3CA | protein phosphatase 3 catalytic subunit alpha [Source:HGNC Symbol;Acc:HGNC:9314] | 5070 | 0.110 | -0.0041 | No |
| 12 | SHC1 | SHC adaptor protein 1 [Source:HGNC Symbol;Acc:HGNC:10840] | 5181 | 0.108 | -0.0008 | No |
| 13 | IFNA5 | interferon alpha 5 [Source:HGNC Symbol;Acc:HGNC:5426] | 6009 | 0.093 | -0.0165 | No |
| 14 | SHC2 | SHC adaptor protein 2 [Source:HGNC Symbol;Acc:HGNC:29869] | 6275 | 0.089 | -0.0182 | No |
| 15 | BID | BH3 interacting domain death agonist [Source:HGNC Symbol;Acc:HGNC:1050] | 6499 | 0.086 | -0.0189 | No |
| 16 | CHP1 | calcineurin like EF-hand protein 1 [Source:HGNC Symbol;Acc:HGNC:17433] | 7211 | 0.077 | -0.0327 | No |
| 17 | ICAM1 | intercellular adhesion molecule 1 [Source:HGNC Symbol;Acc:HGNC:5344] | 8090 | 0.067 | -0.0513 | No |
| 18 | RAET1L | retinoic acid early transcript 1L [Source:HGNC Symbol;Acc:HGNC:16798] | 8304 | 0.064 | -0.0530 | No |
| 19 | IFNA16 | interferon alpha 16 [Source:HGNC Symbol;Acc:HGNC:5421] | 8538 | 0.062 | -0.0554 | No |
| 20 | RAF1 | "Raf-1 proto-oncogene, serine/threonine kinase [Source:HGNC Symbol;Acc:HGNC:9829]" | 8635 | 0.061 | -0.0544 | No |
| 21 | PIK3R3 | phosphoinositide-3-kinase regulatory subunit 3 [Source:HGNC Symbol;Acc:HGNC:8981] | 9028 | 0.057 | -0.0611 | No |
| 22 | ULBP1 | UL16 binding protein 1 [Source:HGNC Symbol;Acc:HGNC:14893] | 9035 | 0.057 | -0.0580 | No |
| 23 | IFNA7 | interferon alpha 7 [Source:HGNC Symbol;Acc:HGNC:5428] | 9153 | 0.056 | -0.0577 | No |
| 24 | NFATC1 | nuclear factor of activated T cells 1 [Source:HGNC Symbol;Acc:HGNC:7775] | 9184 | 0.056 | -0.0553 | No |
| 25 | RAET1G | retinoic acid early transcript 1G [Source:HGNC Symbol;Acc:HGNC:16795] | 9259 | 0.055 | -0.0540 | No |
| 26 | IFNB1 | interferon beta 1 [Source:HGNC Symbol;Acc:HGNC:5434] | 9334 | 0.054 | -0.0528 | No |
| 27 | HLA-C | "major histocompatibility complex, class I, C [Source:HGNC Symbol;Acc:HGNC:4933]" | 9612 | 0.052 | -0.0569 | No |
| 28 | CSF2 | colony stimulating factor 2 [Source:HGNC Symbol;Acc:HGNC:2434] | 9888 | 0.050 | -0.0611 | No |
| 29 | PPP3R1 | "protein phosphatase 3 regulatory subunit B, alpha [Source:HGNC Symbol;Acc:HGNC:9317]" | 10562 | 0.044 | -0.0757 | No |
| 30 | HCST | hematopoietic cell signal transducer [Source:HGNC Symbol;Acc:HGNC:16977] | 11016 | 0.041 | -0.0849 | No |
| 31 | IFNA13 | interferon alpha 13 [Source:HGNC Symbol;Acc:HGNC:5419] | 11857 | 0.034 | -0.1044 | No |
| 32 | HLA-G | "major histocompatibility complex, class I, G [Source:HGNC Symbol;Acc:HGNC:4964]" | 12279 | 0.031 | -0.1133 | No |
| 33 | ULBP2 | UL16 binding protein 2 [Source:HGNC Symbol;Acc:HGNC:14894] | 12356 | 0.030 | -0.1135 | No |
| 34 | TNFSF10 | TNF superfamily member 10 [Source:HGNC Symbol;Acc:HGNC:11925] | 13004 | 0.026 | -0.1286 | No |
| 35 | IFNA8 | interferon alpha 8 [Source:HGNC Symbol;Acc:HGNC:5429] | 13190 | 0.024 | -0.1319 | No |
| 36 | HLA-B | "major histocompatibility complex, class I, B [Source:HGNC Symbol;Acc:HGNC:4932]" | 13305 | 0.024 | -0.1335 | No |
| 37 | ARAF | "A-Raf proto-oncogene, serine/threonine kinase [Source:HGNC Symbol;Acc:HGNC:646]" | 13933 | 0.020 | -0.1483 | No |
| 38 | CHP2 | calcineurin like EF-hand protein 2 [Source:HGNC Symbol;Acc:HGNC:24927] | 13971 | 0.020 | -0.1481 | No |
| 39 | SHC3 | SHC adaptor protein 3 [Source:HGNC Symbol;Acc:HGNC:18181] | 14963 | 0.013 | -0.1727 | No |
| 40 | IFNAR1 | interferon alpha and beta receptor subunit 1 [Source:HGNC Symbol;Acc:HGNC:5432] | 15859 | 0.008 | -0.1951 | No |
| 41 | IFNA4 | interferon alpha 4 [Source:HGNC Symbol;Acc:HGNC:5425] | 16049 | 0.007 | -0.1995 | No |
| 42 | ULBP3 | UL16 binding protein 3 [Source:HGNC Symbol;Acc:HGNC:14895] | 16266 | 0.005 | -0.2047 | No |
| 43 | TNFRSF10C | TNF receptor superfamily member 10c [Source:HGNC Symbol;Acc:HGNC:11906] | 16656 | 0.003 | -0.2144 | No |
| 44 | IFNA21 | interferon alpha 21 [Source:HGNC Symbol;Acc:HGNC:5424] | 16891 | 0.002 | -0.2203 | No |
| 45 | IFNA17 | interferon alpha 17 [Source:HGNC Symbol;Acc:HGNC:5422] | 18384 | -0.007 | -0.2580 | No |
| 46 | IFNA14 | interferon alpha 14 [Source:HGNC Symbol;Acc:HGNC:5420] | 19637 | -0.014 | -0.2891 | No |
| 47 | IFNGR1 | interferon gamma receptor 1 [Source:HGNC Symbol;Acc:HGNC:5439] | 19762 | -0.015 | -0.2914 | No |
| 48 | LAT | linker for activation of T cells [Source:HGNC Symbol;Acc:HGNC:18874] | 20384 | -0.019 | -0.3061 | No |
| 49 | IFNA1 | interferon alpha 1 [Source:HGNC Symbol;Acc:HGNC:5417] | 20479 | -0.020 | -0.3074 | No |
| 50 | NCR2 | natural cytotoxicity triggering receptor 2 [Source:HGNC Symbol;Acc:HGNC:6732] | 20891 | -0.022 | -0.3166 | No |
| 51 | IFNA6 | interferon alpha 6 [Source:HGNC Symbol;Acc:HGNC:5427] | 21419 | -0.025 | -0.3286 | No |
| 52 | HLA-E | "major histocompatibility complex, class I, E [Source:HGNC Symbol;Acc:HGNC:4962]" | 21659 | -0.027 | -0.3332 | No |
| 53 | TYROBP | transmembrane immune signaling adaptor TYROBP [Source:HGNC Symbol;Acc:HGNC:12449] | 22350 | -0.031 | -0.3490 | No |
| 54 | PTPN6 | protein tyrosine phosphatase non-receptor type 6 [Source:HGNC Symbol;Acc:HGNC:9658] | 23581 | -0.039 | -0.3782 | No |
| 55 | IFNA2 | interferon alpha 2 [Source:HGNC Symbol;Acc:HGNC:5423] | 23890 | -0.041 | -0.3837 | No |
| 56 | FAS | Fas cell surface death receptor [Source:HGNC Symbol;Acc:HGNC:11920] | 23994 | -0.042 | -0.3839 | No |
| 57 | RAC2 | Rac family small GTPase 2 [Source:HGNC Symbol;Acc:HGNC:9802] | 24228 | -0.043 | -0.3874 | No |
| 58 | SHC4 | SHC adaptor protein 4 [Source:HGNC Symbol;Acc:HGNC:16743] | 24342 | -0.044 | -0.3877 | No |
| 59 | VAV2 | vav guanine nucleotide exchange factor 2 [Source:HGNC Symbol;Acc:HGNC:12658] | 24578 | -0.046 | -0.3911 | No |
| 60 | RAET1E | retinoic acid early transcript 1E [Source:HGNC Symbol;Acc:HGNC:16793] | 25080 | -0.049 | -0.4011 | No |
| 61 | ICAM2 | intercellular adhesion molecule 2 [Source:HGNC Symbol;Acc:HGNC:5345] | 25505 | -0.052 | -0.4089 | No |
| 62 | KIR2DS4 | "killer cell immunoglobulin like receptor, two Ig domains and short cytoplasmic tail 4 [Source:HGNC Symbol;Acc:HGNC:6336]" | 25804 | -0.054 | -0.4134 | No |
| 63 | IFNA10 | interferon alpha 10 [Source:HGNC Symbol;Acc:HGNC:5418] | 26311 | -0.058 | -0.4230 | No |
| 64 | KLRC2 | killer cell lectin like receptor C2 [Source:HGNC Symbol;Acc:HGNC:6375] | 26343 | -0.058 | -0.4204 | No |
| 65 | IFNAR2 | interferon alpha and beta receptor subunit 2 [Source:HGNC Symbol;Acc:HGNC:5433] | 27247 | -0.065 | -0.4397 | No |
| 66 | KLRC1 | killer cell lectin like receptor C1 [Source:HGNC Symbol;Acc:HGNC:6374] | 28252 | -0.073 | -0.4611 | No |
| 67 | VAV3 | vav guanine nucleotide exchange factor 3 [Source:HGNC Symbol;Acc:HGNC:12659] | 29000 | -0.080 | -0.4756 | No |
| 68 | SH3BP2 | SH3 domain binding protein 2 [Source:HGNC Symbol;Acc:HGNC:10825] | 29467 | -0.084 | -0.4827 | No |
| 69 | PAK1 | p21 (RAC1) activated kinase 1 [Source:HGNC Symbol;Acc:HGNC:8590] | 29530 | -0.085 | -0.4794 | No |
| 70 | BRAF | "B-Raf proto-oncogene, serine/threonine kinase [Source:HGNC Symbol;Acc:HGNC:1097]" | 29630 | -0.086 | -0.4770 | No |
| 71 | TNFRSF10B | TNF receptor superfamily member 10b [Source:HGNC Symbol;Acc:HGNC:11905] | 30305 | -0.093 | -0.4889 | No |
| 72 | PLCG1 | phospholipase C gamma 1 [Source:HGNC Symbol;Acc:HGNC:9065] | 30759 | -0.098 | -0.4948 | No |
| 73 | TNFRSF10D | TNF receptor superfamily member 10d [Source:HGNC Symbol;Acc:HGNC:11907] | 30869 | -0.099 | -0.4920 | No |
| 74 | CASP3 | caspase 3 [Source:HGNC Symbol;Acc:HGNC:1504] | 31110 | -0.102 | -0.4922 | No |
| 75 | PRKCG | protein kinase C gamma [Source:HGNC Symbol;Acc:HGNC:9402] | 31522 | -0.107 | -0.4966 | No |
| 76 | PIK3CD | "phosphatidylinositol-4,5-bisphosphate 3-kinase catalytic subunit delta [Source:HGNC Symbol;Acc:HGNC:8977]" | 31859 | -0.111 | -0.4988 | No |
| 77 | PRKCA | protein kinase C alpha [Source:HGNC Symbol;Acc:HGNC:9393] | 32080 | -0.114 | -0.4979 | No |
| 78 | PPP3R2 | "protein phosphatase 3 regulatory subunit B, beta [Source:HGNC Symbol;Acc:HGNC:9318]" | 32695 | -0.123 | -0.5065 | No |
| 79 | KIR2DL1 | "killer cell immunoglobulin like receptor, two Ig domains and long cytoplasmic tail 1 [Source:HGNC Symbol;Acc:HGNC:6329]" | 32995 | -0.128 | -0.5068 | Yes |
| 80 | PPP3CB | protein phosphatase 3 catalytic subunit beta [Source:HGNC Symbol;Acc:HGNC:9315] | 33217 | -0.131 | -0.5049 | Yes |
| 81 | KIR2DL3 | "killer cell immunoglobulin like receptor, two Ig domains and long cytoplasmic tail 3 [Source:HGNC Symbol;Acc:HGNC:6331]" | 33593 | -0.138 | -0.5066 | Yes |
| 82 | MAPK1 | mitogen-activated protein kinase 1 [Source:HGNC Symbol;Acc:HGNC:6871] | 33636 | -0.139 | -0.4997 | Yes |
| 83 | ITGB2 | integrin subunit beta 2 [Source:HGNC Symbol;Acc:HGNC:6155] | 33691 | -0.139 | -0.4931 | Yes |
| 84 | NCR3 | natural cytotoxicity triggering receptor 3 [Source:HGNC Symbol;Acc:HGNC:19077] | 33714 | -0.140 | -0.4856 | Yes |
| 85 | TNF | tumor necrosis factor [Source:HGNC Symbol;Acc:HGNC:11892] | 33732 | -0.140 | -0.4780 | Yes |
| 86 | MICB | MHC class I polypeptide-related sequence B [Source:HGNC Symbol;Acc:HGNC:7091] | 33888 | -0.143 | -0.4738 | Yes |
| 87 | KIR2DL4 | "killer cell immunoglobulin like receptor, two Ig domains and long cytoplasmic tail 4 [Source:HGNC Symbol;Acc:HGNC:6332]" | 33950 | -0.144 | -0.4670 | Yes |
| 88 | KIR3DL1 | "killer cell immunoglobulin like receptor, three Ig domains and long cytoplasmic tail 1 [Source:HGNC Symbol;Acc:HGNC:6338]" | 34105 | -0.147 | -0.4625 | Yes |
| 89 | NRAS | "NRAS proto-oncogene, GTPase [Source:HGNC Symbol;Acc:HGNC:7989]" | 34553 | -0.156 | -0.4650 | Yes |
| 90 | SOS2 | SOS Ras/Rho guanine nucleotide exchange factor 2 [Source:HGNC Symbol;Acc:HGNC:11188] | 34598 | -0.157 | -0.4571 | Yes |
| 91 | CD48 | CD48 molecule [Source:HGNC Symbol;Acc:HGNC:1683] | 34692 | -0.159 | -0.4504 | Yes |
| 92 | PIK3R1 | phosphoinositide-3-kinase regulatory subunit 1 [Source:HGNC Symbol;Acc:HGNC:8979] | 34788 | -0.162 | -0.4435 | Yes |
| 93 | VAV1 | vav guanine nucleotide exchange factor 1 [Source:HGNC Symbol;Acc:HGNC:12657] | 34853 | -0.163 | -0.4358 | Yes |
| 94 | PIK3CB | "phosphatidylinositol-4,5-bisphosphate 3-kinase catalytic subunit beta [Source:HGNC Symbol;Acc:HGNC:8976]" | 34871 | -0.164 | -0.4269 | Yes |
| 95 | ZAP70 | zeta chain of T cell receptor associated protein kinase 70 [Source:HGNC Symbol;Acc:HGNC:12858] | 34981 | -0.166 | -0.4201 | Yes |
| 96 | MAP2K1 | mitogen-activated protein kinase kinase 1 [Source:HGNC Symbol;Acc:HGNC:6840] | 35210 | -0.171 | -0.4161 | Yes |
| 97 | PRF1 | perforin 1 [Source:HGNC Symbol;Acc:HGNC:9360] | 35249 | -0.172 | -0.4072 | Yes |
| 98 | KLRK1 | killer cell lectin like receptor K1 [Source:HGNC Symbol;Acc:HGNC:18788] | 35321 | -0.175 | -0.3990 | Yes |
| 99 | FCER1G | Fc fragment of IgE receptor Ig [Source:HGNC Symbol;Acc:HGNC:3611] | 35448 | -0.178 | -0.3921 | Yes |
| 100 | IFNG | interferon gamma [Source:HGNC Symbol;Acc:HGNC:5438] | 35652 | -0.183 | -0.3867 | Yes |
| 101 | NCR1 | natural cytotoxicity triggering receptor 1 [Source:HGNC Symbol;Acc:HGNC:6731] | 35714 | -0.185 | -0.3777 | Yes |
| 102 | PPP3CC | protein phosphatase 3 catalytic subunit gamma [Source:HGNC Symbol;Acc:HGNC:9316] | 35754 | -0.186 | -0.3680 | Yes |
| 103 | SH2D1B | SH2 domain containing 1B [Source:HGNC Symbol;Acc:HGNC:30416] | 35989 | -0.194 | -0.3629 | Yes |
| 104 | KLRC3 | killer cell lectin like receptor C3 [Source:HGNC Symbol;Acc:HGNC:6376] | 36043 | -0.195 | -0.3530 | Yes |
| 105 | FASLG | Fas ligand [Source:HGNC Symbol;Acc:HGNC:11936] | 36345 | -0.206 | -0.3489 | Yes |
| 106 | CD247 | CD247 molecule [Source:HGNC Symbol;Acc:HGNC:1677] | 36507 | -0.211 | -0.3409 | Yes |
| 107 | GRB2 | growth factor receptor bound protein 2 [Source:HGNC Symbol;Acc:HGNC:4566] | 36516 | -0.212 | -0.3289 | Yes |
| 108 | KIR3DL2 | "killer cell immunoglobulin like receptor, three Ig domains and long cytoplasmic tail 2 [Source:HGNC Symbol;Acc:HGNC:6339]" | 36649 | -0.218 | -0.3198 | Yes |
| 109 | NFATC2 | nuclear factor of activated T cells 2 [Source:HGNC Symbol;Acc:HGNC:7776] | 36732 | -0.221 | -0.3093 | Yes |
| 110 | CD244 | CD244 molecule [Source:HGNC Symbol;Acc:HGNC:18171] | 36770 | -0.223 | -0.2974 | Yes |
| 111 | SH2D1A | SH2 domain containing 1A [Source:HGNC Symbol;Acc:HGNC:10820] | 36784 | -0.224 | -0.2849 | Yes |
| 112 | TNFRSF10A | TNF receptor superfamily member 10a [Source:HGNC Symbol;Acc:HGNC:11904] | 36857 | -0.228 | -0.2737 | Yes |
| 113 | PTK2B | protein tyrosine kinase 2 beta [Source:HGNC Symbol;Acc:HGNC:9612] | 37013 | -0.237 | -0.2640 | Yes |
| 114 | LCK | "LCK proto-oncogene, Src family tyrosine kinase [Source:HGNC Symbol;Acc:HGNC:6524]" | 37085 | -0.240 | -0.2521 | Yes |
| 115 | GZMB | granzyme B [Source:HGNC Symbol;Acc:HGNC:4709] | 37092 | -0.241 | -0.2384 | Yes |
| 116 | FYN | "FYN proto-oncogene, Src family tyrosine kinase [Source:HGNC Symbol;Acc:HGNC:4037]" | 37102 | -0.242 | -0.2248 | Yes |
| 117 | FCGR3A | Fc fragment of IgG receptor IIIa [Source:HGNC Symbol;Acc:HGNC:3619] | 37450 | -0.263 | -0.2186 | Yes |
| 118 | PIK3CA | "phosphatidylinositol-4,5-bisphosphate 3-kinase catalytic subunit alpha [Source:HGNC Symbol;Acc:HGNC:8975]" | 37556 | -0.270 | -0.2058 | Yes |
| 119 | KLRD1 | killer cell lectin like receptor D1 [Source:HGNC Symbol;Acc:HGNC:6378] | 37615 | -0.276 | -0.1915 | Yes |
| 120 | ITGAL | integrin subunit alpha L [Source:HGNC Symbol;Acc:HGNC:6148] | 37624 | -0.276 | -0.1758 | Yes |
| 121 | KRAS | "KRAS proto-oncogene, GTPase [Source:HGNC Symbol;Acc:HGNC:6407]" | 37629 | -0.277 | -0.1601 | Yes |
| 122 | NFAT5 | nuclear factor of activated T cells 5 [Source:HGNC Symbol;Acc:HGNC:7774] | 37631 | -0.277 | -0.1442 | Yes |
| 123 | NFATC3 | nuclear factor of activated T cells 3 [Source:HGNC Symbol;Acc:HGNC:7777] | 37672 | -0.281 | -0.1292 | Yes |
| 124 | SYK | spleen associated tyrosine kinase [Source:HGNC Symbol;Acc:HGNC:11491] | 37729 | -0.285 | -0.1142 | Yes |
| 125 | PRKCB | protein kinase C beta [Source:HGNC Symbol;Acc:HGNC:9395] | 37822 | -0.293 | -0.0998 | Yes |
| 126 | FCGR3B | Fc fragment of IgG receptor IIIb [Source:HGNC Symbol;Acc:HGNC:3620] | 37877 | -0.299 | -0.0840 | Yes |
| 127 | LCP2 | lymphocyte cytosolic protein 2 [Source:HGNC Symbol;Acc:HGNC:6529] | 37904 | -0.304 | -0.0673 | Yes |
| 128 | PIK3R5 | phosphoinositide-3-kinase regulatory subunit 5 [Source:HGNC Symbol;Acc:HGNC:30035] | 37956 | -0.310 | -0.0508 | Yes |
| 129 | PLCG2 | phospholipase C gamma 2 [Source:HGNC Symbol;Acc:HGNC:9066] | 38119 | -0.338 | -0.0355 | Yes |
| 130 | SOS1 | SOS Ras/Rac guanine nucleotide exchange factor 1 [Source:HGNC Symbol;Acc:HGNC:11187] | 38132 | -0.341 | -0.0163 | Yes |
| 131 | PTPN11 | protein tyrosine phosphatase non-receptor type 11 [Source:HGNC Symbol;Acc:HGNC:9644] | 38264 | -0.388 | 0.0026 | Yes |
| 132 | PIK3CG | "phosphatidylinositol-4,5-bisphosphate 3-kinase catalytic subunit gamma [Source:HGNC Symbol;Acc:HGNC:8978]" | 38302 | -0.426 | 0.0261 | Yes |
Table: GSEA details [plain text format]

  

Fig 2: KEGG\_NATURAL\_KILLER\_CELL\_MEDIATED\_CYTOTOXICITY      
 Blue-Pink O' Gram in the Space of the Analyzed GeneSet

  

Fig 3: KEGG\_NATURAL\_KILLER\_CELL\_MEDIATED\_CYTOTOXICITY: Random ES distribution      
 Gene set null distribution of ES for **KEGG\_NATURAL\_KILLER\_CELL\_MEDIATED\_CYTOTOXICITY**

  
