# Supplementary material for: Simultaneous Presentation of Multiple Myeloma and Lung Cancer: Case Report and Gene Bioinformatics Analysis
Source: Front Oncol. 2022 Jun 13;12:859735. doi: 10.3389/fonc.2022.859735 (PMC9235397; doi:10.3389/fonc.2022.859735)
Supplement: Supplementary file 1 [file DataSheet_1.zip › The bioinformatic analysis of MM and lung cancer supplementary materials/Enrichment analysis/MECR/GSEA_4.1.0/LUAD TCGA/KEGG.Gsea.1639041756227/KEGG_NEUROACTIVE_LIGAND_RECEPTOR_INTERACTION.html]

Details for gene set KEGG\_NEUROACTIVE\_LIGAND\_RECEPTOR\_INTERACTION[GSEA]

|  || Dataset | ExpData\_collapsed\_to\_symbols.ENSG00000116353\_profile\_in\_ExpData.cls #ENSG00000116353 |
| Phenotype | ENSG00000116353\_profile\_in\_ExpData.cls#ENSG00000116353 |
| Upregulated in class | ENSG00000116353\_neg |
| GeneSet | KEGG\_NEUROACTIVE\_LIGAND\_RECEPTOR\_INTERACTION |
| Enrichment Score (ES) | -0.45954767 |
| Normalized Enrichment Score (NES) | -2.1847515 |
| Nominal p-value | 0.0 |
| FDR q-value | 7.7572964E-5 |
| FWER p-Value | 0.001 |
Table: GSEA Results Summary

  

Fig 1: Enrichment plot: KEGG\_NEUROACTIVE\_LIGAND\_RECEPTOR\_INTERACTION      
 Profile of the Running ES Score & Positions of GeneSet Members on the Rank Ordered List

  

| SYMBOL | TITLE | RANK IN GENE LIST | RANK METRIC SCORE | RUNNING ES | CORE ENRICHMENT || 1 | TSPO | translocator protein [Source:HGNC Symbol;Acc:HGNC:1158] | 644 | 0.304 | -0.0045 | No |
| 2 | CHRNB1 | cholinergic receptor nicotinic beta 1 subunit [Source:HGNC Symbol;Acc:HGNC:1961] | 1280 | 0.250 | -0.0110 | No |
| 3 | GABRD | gamma-aminobutyric acid type A receptor subunit delta [Source:HGNC Symbol;Acc:HGNC:4084] | 2380 | 0.190 | -0.0317 | No |
| 4 | LPAR2 | lysophosphatidic acid receptor 2 [Source:HGNC Symbol;Acc:HGNC:3168] | 2760 | 0.173 | -0.0345 | No |
| 5 | ADORA2B | adenosine A2b receptor [Source:HGNC Symbol;Acc:HGNC:264] | 3536 | 0.147 | -0.0486 | No |
| 6 | GABRE | gamma-aminobutyric acid type A receptor subunit epsilon [Source:HGNC Symbol;Acc:HGNC:4085] | 3563 | 0.146 | -0.0435 | No |
| 7 | LHCGR | luteinizing hormone/choriogonadotropin receptor [Source:HGNC Symbol;Acc:HGNC:6585] | 3697 | 0.142 | -0.0413 | No |
| 8 | MC1R | melanocortin 1 receptor [Source:HGNC Symbol;Acc:HGNC:6929] | 4169 | 0.129 | -0.0483 | No |
| 9 | CHRNE | cholinergic receptor nicotinic epsilon subunit [Source:HGNC Symbol;Acc:HGNC:1966] | 4228 | 0.128 | -0.0447 | No |
| 10 | OPRL1 | opioid related nociceptin receptor 1 [Source:HGNC Symbol;Acc:HGNC:8155] | 4599 | 0.119 | -0.0495 | No |
| 11 | P2RY2 | purinergic receptor P2Y2 [Source:HGNC Symbol;Acc:HGNC:8541] | 5047 | 0.110 | -0.0566 | No |
| 12 | GRIK2 | glutamate ionotropic receptor kainate type subunit 2 [Source:HGNC Symbol;Acc:HGNC:4580] | 5406 | 0.104 | -0.0617 | No |
| 13 | ADRA2C | adrenoceptor alpha 2C [Source:HGNC Symbol;Acc:HGNC:283] | 5590 | 0.100 | -0.0624 | No |
| 14 | LHB | luteinizing hormone subunit beta [Source:HGNC Symbol;Acc:HGNC:6584] | 6321 | 0.088 | -0.0776 | No |
| 15 | THRA | thyroid hormone receptor alpha [Source:HGNC Symbol;Acc:HGNC:11796] | 6325 | 0.088 | -0.0742 | No |
| 16 | GABRA6 | gamma-aminobutyric acid type A receptor subunit alpha6 [Source:HGNC Symbol;Acc:HGNC:4080] | 6470 | 0.086 | -0.0745 | No |
| 17 | GLP2R | glucagon like peptide 2 receptor [Source:HGNC Symbol;Acc:HGNC:4325] | 6652 | 0.084 | -0.0758 | No |
| 18 | P2RY11 | purinergic receptor P2Y11 [Source:HGNC Symbol;Acc:HGNC:8540] | 6914 | 0.080 | -0.0794 | No |
| 19 | OPRK1 | opioid receptor kappa 1 [Source:HGNC Symbol;Acc:HGNC:8154] | 8046 | 0.067 | -0.1057 | No |
| 20 | NMBR | neuromedin B receptor [Source:HGNC Symbol;Acc:HGNC:7843] | 8115 | 0.066 | -0.1048 | No |
| 21 | ADRA1B | adrenoceptor alpha 1B [Source:HGNC Symbol;Acc:HGNC:278] | 8272 | 0.065 | -0.1063 | No |
| 22 | OPRM1 | opioid receptor mu 1 [Source:HGNC Symbol;Acc:HGNC:8156] | 9147 | 0.056 | -0.1264 | No |
| 23 | CRHR2 | corticotropin releasing hormone receptor 2 [Source:HGNC Symbol;Acc:HGNC:2358] | 9165 | 0.056 | -0.1247 | No |
| 24 | ADRA2B | adrenoceptor alpha 2B [Source:HGNC Symbol;Acc:HGNC:282] | 9172 | 0.056 | -0.1226 | No |
| 25 | ADRB1 | adrenoceptor beta 1 [Source:HGNC Symbol;Acc:HGNC:285] | 9260 | 0.055 | -0.1227 | No |
| 26 | GLRB | glycine receptor beta [Source:HGNC Symbol;Acc:HGNC:4329] | 9514 | 0.053 | -0.1271 | No |
| 27 | GALR2 | galanin receptor 2 [Source:HGNC Symbol;Acc:HGNC:4133] | 9532 | 0.053 | -0.1255 | No |
| 28 | GPR156 | G protein-coupled receptor 156 [Source:HGNC Symbol;Acc:HGNC:20844] | 9606 | 0.052 | -0.1253 | No |
| 29 | P2RX4 | purinergic receptor P2X 4 [Source:HGNC Symbol;Acc:HGNC:8535] | 9648 | 0.052 | -0.1243 | No |
| 30 | THRB | thyroid hormone receptor beta [Source:HGNC Symbol;Acc:HGNC:11799] | 9739 | 0.051 | -0.1246 | No |
| 31 | CRHR1 | corticotropin releasing hormone receptor 1 [Source:HGNC Symbol;Acc:HGNC:2357] | 9829 | 0.050 | -0.1249 | No |
| 32 | VIPR1 | vasoactive intestinal peptide receptor 1 [Source:HGNC Symbol;Acc:HGNC:12694] | 9944 | 0.049 | -0.1259 | No |
| 33 | GRID1 | glutamate ionotropic receptor delta type subunit 1 [Source:HGNC Symbol;Acc:HGNC:4575] | 10269 | 0.047 | -0.1324 | No |
| 34 | OPRD1 | opioid receptor delta 1 [Source:HGNC Symbol;Acc:HGNC:8153] | 10278 | 0.047 | -0.1307 | No |
| 35 | F2RL1 | F2R like trypsin receptor 1 [Source:HGNC Symbol;Acc:HGNC:3538] | 10675 | 0.044 | -0.1392 | No |
| 36 | ADRA1D | adrenoceptor alpha 1D [Source:HGNC Symbol;Acc:HGNC:280] | 11153 | 0.040 | -0.1498 | No |
| 37 | VIPR2 | vasoactive intestinal peptide receptor 2 [Source:HGNC Symbol;Acc:HGNC:12695] | 11179 | 0.040 | -0.1489 | No |
| 38 | GLRA3 | glycine receptor alpha 3 [Source:HGNC Symbol;Acc:HGNC:4328] | 11242 | 0.039 | -0.1490 | No |
| 39 | TACR2 | tachykinin receptor 2 [Source:HGNC Symbol;Acc:HGNC:11527] | 11248 | 0.039 | -0.1476 | No |
| 40 | CCKBR | cholecystokinin B receptor [Source:HGNC Symbol;Acc:HGNC:1571] | 11320 | 0.038 | -0.1479 | No |
| 41 | NTSR2 | neurotensin receptor 2 [Source:HGNC Symbol;Acc:HGNC:8040] | 11459 | 0.037 | -0.1499 | No |
| 42 | GABBR2 | gamma-aminobutyric acid type B receptor subunit 2 [Source:HGNC Symbol;Acc:HGNC:4507] | 11486 | 0.037 | -0.1491 | No |
| 43 | PTGER1 | prostaglandin E receptor 1 [Source:HGNC Symbol;Acc:HGNC:9593] | 11693 | 0.036 | -0.1530 | No |
| 44 | PARD3 | par-3 family cell polarity regulator [Source:HGNC Symbol;Acc:HGNC:16051] | 11922 | 0.034 | -0.1575 | No |
| 45 | DRD4 | dopamine receptor D4 [Source:HGNC Symbol;Acc:HGNC:3025] | 12142 | 0.032 | -0.1619 | No |
| 46 | GLRA1 | glycine receptor alpha 1 [Source:HGNC Symbol;Acc:HGNC:4326] | 12256 | 0.031 | -0.1635 | No |
| 47 | SCTR | secretin receptor [Source:HGNC Symbol;Acc:HGNC:10608] | 12358 | 0.030 | -0.1649 | No |
| 48 | PRLR | prolactin receptor [Source:HGNC Symbol;Acc:HGNC:9446] | 12366 | 0.030 | -0.1639 | No |
| 49 | BRS3 | bombesin receptor subtype 3 [Source:HGNC Symbol;Acc:HGNC:1113] | 12444 | 0.030 | -0.1647 | No |
| 50 | ADORA1 | adenosine A1 receptor [Source:HGNC Symbol;Acc:HGNC:262] | 12676 | 0.028 | -0.1695 | No |
| 51 | CSH1 | chorionic somatomammotropin hormone 1 [Source:HGNC Symbol;Acc:HGNC:2440] | 13107 | 0.025 | -0.1795 | No |
| 52 | DRD2 | dopamine receptor D2 [Source:HGNC Symbol;Acc:HGNC:3023] | 13125 | 0.025 | -0.1790 | No |
| 53 | TAAR2 | trace amine associated receptor 2 [Source:HGNC Symbol;Acc:HGNC:4514] | 13150 | 0.025 | -0.1786 | No |
| 54 | LPAR1 | lysophosphatidic acid receptor 1 [Source:HGNC Symbol;Acc:HGNC:3166] | 13175 | 0.024 | -0.1783 | No |
| 55 | GABRG2 | gamma-aminobutyric acid type A receptor subunit gamma2 [Source:HGNC Symbol;Acc:HGNC:4087] | 13202 | 0.024 | -0.1780 | No |
| 56 | CHRM1 | cholinergic receptor muscarinic 1 [Source:HGNC Symbol;Acc:HGNC:1950] | 13298 | 0.024 | -0.1795 | No |
| 57 | GABRR1 | gamma-aminobutyric acid type A receptor subunit rho1 [Source:HGNC Symbol;Acc:HGNC:4090] | 13535 | 0.022 | -0.1847 | No |
| 58 | HCRTR1 | hypocretin receptor 1 [Source:HGNC Symbol;Acc:HGNC:4848] | 13577 | 0.022 | -0.1849 | No |
| 59 | GABRG1 | gamma-aminobutyric acid type A receptor subunit gamma1 [Source:HGNC Symbol;Acc:HGNC:4086] | 13633 | 0.022 | -0.1854 | No |
| 60 | GRID2 | glutamate ionotropic receptor delta type subunit 2 [Source:HGNC Symbol;Acc:HGNC:4576] | 13719 | 0.021 | -0.1868 | No |
| 61 | GRIN3B | glutamate ionotropic receptor NMDA type subunit 3B [Source:HGNC Symbol;Acc:HGNC:16768] | 13844 | 0.020 | -0.1892 | No |
| 62 | LTB4R | leukotriene B4 receptor [Source:HGNC Symbol;Acc:HGNC:6713] | 13896 | 0.020 | -0.1897 | No |
| 63 | HTR1E | 5-hydroxytryptamine receptor 1E [Source:HGNC Symbol;Acc:HGNC:5291] | 14008 | 0.019 | -0.1918 | No |
| 64 | ADRB3 | adrenoceptor beta 3 [Source:HGNC Symbol;Acc:HGNC:288] | 14065 | 0.019 | -0.1924 | No |
| 65 | GALR3 | galanin receptor 3 [Source:HGNC Symbol;Acc:HGNC:4134] | 14286 | 0.017 | -0.1974 | No |
| 66 | RXFP2 | relaxin family peptide receptor 2 [Source:HGNC Symbol;Acc:HGNC:17318] | 14311 | 0.017 | -0.1973 | No |
| 67 | NPBWR2 | neuropeptides B and W receptor 2 [Source:HGNC Symbol;Acc:HGNC:4530] | 14384 | 0.017 | -0.1985 | No |
| 68 | S1PR4 | sphingosine-1-phosphate receptor 4 [Source:HGNC Symbol;Acc:HGNC:3170] | 14506 | 0.016 | -0.2010 | No |
| 69 | GRIA2 | glutamate ionotropic receptor AMPA type subunit 2 [Source:HGNC Symbol;Acc:HGNC:4572] | 14571 | 0.016 | -0.2020 | No |
| 70 | P2RX6 | purinergic receptor P2X 6 [Source:HGNC Symbol;Acc:HGNC:8538] | 14690 | 0.015 | -0.2044 | No |
| 71 | TBXA2R | thromboxane A2 receptor [Source:HGNC Symbol;Acc:HGNC:11608] | 14991 | 0.013 | -0.2116 | No |
| 72 | GABRA5 | gamma-aminobutyric acid type A receptor subunit alpha5 [Source:HGNC Symbol;Acc:HGNC:4079] | 15112 | 0.012 | -0.2142 | No |
| 73 | HRH4 | histamine receptor H4 [Source:HGNC Symbol;Acc:HGNC:17383] | 15282 | 0.011 | -0.2180 | No |
| 74 | GABRA1 | gamma-aminobutyric acid type A receptor subunit alpha1 [Source:HGNC Symbol;Acc:HGNC:4075] | 15603 | 0.009 | -0.2259 | No |
| 75 | GRIN2A | glutamate ionotropic receptor NMDA type subunit 2A [Source:HGNC Symbol;Acc:HGNC:4585] | 15647 | 0.009 | -0.2266 | No |
| 76 | GABRA3 | gamma-aminobutyric acid type A receptor subunit alpha3 [Source:HGNC Symbol;Acc:HGNC:4077] | 15720 | 0.009 | -0.2281 | No |
| 77 | CHRNA4 | cholinergic receptor nicotinic alpha 4 subunit [Source:HGNC Symbol;Acc:HGNC:1958] | 15795 | 0.008 | -0.2297 | No |
| 78 | GRIN1 | glutamate ionotropic receptor NMDA type subunit 1 [Source:HGNC Symbol;Acc:HGNC:4584] | 15818 | 0.008 | -0.2299 | No |
| 79 | CHRNB4 | cholinergic receptor nicotinic beta 4 subunit [Source:HGNC Symbol;Acc:HGNC:1964] | 15855 | 0.008 | -0.2305 | No |
| 80 | DRD3 | dopamine receptor D3 [Source:HGNC Symbol;Acc:HGNC:3024] | 15863 | 0.008 | -0.2304 | No |
| 81 | GPR83 | G protein-coupled receptor 83 [Source:HGNC Symbol;Acc:HGNC:4523] | 16446 | 0.004 | -0.2451 | No |
| 82 | GRM6 | glutamate metabotropic receptor 6 [Source:HGNC Symbol;Acc:HGNC:4598] | 16475 | 0.004 | -0.2457 | No |
| 83 | PTH1R | parathyroid hormone 1 receptor [Source:HGNC Symbol;Acc:HGNC:9608] | 17069 | 0.001 | -0.2608 | No |
| 84 | GRIN2C | glutamate ionotropic receptor NMDA type subunit 2C [Source:HGNC Symbol;Acc:HGNC:4587] | 17175 | 0.000 | -0.2635 | No |
| 85 | GABRA4 | gamma-aminobutyric acid type A receptor subunit alpha4 [Source:HGNC Symbol;Acc:HGNC:4078] | 17261 | -0.000 | -0.2657 | No |
| 86 | MC5R | melanocortin 5 receptor [Source:HGNC Symbol;Acc:HGNC:6933] | 17406 | -0.001 | -0.2693 | No |
| 87 | NPY2R | neuropeptide Y receptor Y2 [Source:HGNC Symbol;Acc:HGNC:7957] | 17459 | -0.002 | -0.2706 | No |
| 88 | GABBR1 | gamma-aminobutyric acid type B receptor subunit 1 [Source:HGNC Symbol;Acc:HGNC:4070] | 17584 | -0.002 | -0.2737 | No |
| 89 | GABRG3 | gamma-aminobutyric acid type A receptor subunit gamma3 [Source:HGNC Symbol;Acc:HGNC:4088] | 17702 | -0.003 | -0.2765 | No |
| 90 | AVPR2 | arginine vasopressin receptor 2 [Source:HGNC Symbol;Acc:HGNC:897] | 17899 | -0.004 | -0.2814 | No |
| 91 | GABRQ | gamma-aminobutyric acid type A receptor subunit theta [Source:HGNC Symbol;Acc:HGNC:14454] | 17977 | -0.005 | -0.2832 | No |
| 92 | MC2R | melanocortin 2 receptor [Source:HGNC Symbol;Acc:HGNC:6930] | 18033 | -0.005 | -0.2844 | No |
| 93 | P2RX3 | purinergic receptor P2X 3 [Source:HGNC Symbol;Acc:HGNC:8534] | 18041 | -0.005 | -0.2844 | No |
| 94 | DRD5 | dopamine receptor D5 [Source:HGNC Symbol;Acc:HGNC:3026] | 18566 | -0.008 | -0.2975 | No |
| 95 | CNR1 | cannabinoid receptor 1 [Source:HGNC Symbol;Acc:HGNC:2159] | 18661 | -0.009 | -0.2995 | No |
| 96 | HTR1D | 5-hydroxytryptamine receptor 1D [Source:HGNC Symbol;Acc:HGNC:5289] | 18972 | -0.011 | -0.3070 | No |
| 97 | NMUR2 | neuromedin U receptor 2 [Source:HGNC Symbol;Acc:HGNC:16454] | 19334 | -0.013 | -0.3158 | No |
| 98 | LPAR3 | lysophosphatidic acid receptor 3 [Source:HGNC Symbol;Acc:HGNC:14298] | 19407 | -0.013 | -0.3171 | No |
| 99 | GRM3 | glutamate metabotropic receptor 3 [Source:HGNC Symbol;Acc:HGNC:4595] | 19704 | -0.015 | -0.3241 | No |
| 100 | S1PR2 | sphingosine-1-phosphate receptor 2 [Source:HGNC Symbol;Acc:HGNC:3169] | 19798 | -0.016 | -0.3259 | No |
| 101 | GABRB1 | gamma-aminobutyric acid type A receptor subunit beta1 [Source:HGNC Symbol;Acc:HGNC:4081] | 19868 | -0.016 | -0.3270 | No |
| 102 | CTSG | cathepsin G [Source:HGNC Symbol;Acc:HGNC:2532] | 19906 | -0.016 | -0.3273 | No |
| 103 | CHRNA10 | cholinergic receptor nicotinic alpha 10 subunit [Source:HGNC Symbol;Acc:HGNC:13800] | 20219 | -0.018 | -0.3346 | No |
| 104 | HRH1 | histamine receptor H1 [Source:HGNC Symbol;Acc:HGNC:5182] | 20266 | -0.018 | -0.3351 | No |
| 105 | GHRHR | growth hormone releasing hormone receptor [Source:HGNC Symbol;Acc:HGNC:4266] | 20469 | -0.020 | -0.3395 | No |
| 106 | F2RL3 | F2R like thrombin or trypsin receptor 3 [Source:HGNC Symbol;Acc:HGNC:3540] | 20927 | -0.022 | -0.3503 | No |
| 107 | GRM7 | glutamate metabotropic receptor 7 [Source:HGNC Symbol;Acc:HGNC:4599] | 21142 | -0.024 | -0.3548 | No |
| 108 | GLP1R | glucagon like peptide 1 receptor [Source:HGNC Symbol;Acc:HGNC:4324] | 21151 | -0.024 | -0.3541 | No |
| 109 | SSTR4 | somatostatin receptor 4 [Source:HGNC Symbol;Acc:HGNC:11333] | 21281 | -0.024 | -0.3565 | No |
| 110 | FSHB | follicle stimulating hormone subunit beta [Source:HGNC Symbol;Acc:HGNC:3964] | 21302 | -0.025 | -0.3560 | No |
| 111 | GABRB3 | gamma-aminobutyric acid type A receptor subunit beta3 [Source:HGNC Symbol;Acc:HGNC:4083] | 21309 | -0.025 | -0.3552 | No |
| 112 | GRIA4 | glutamate ionotropic receptor AMPA type subunit 4 [Source:HGNC Symbol;Acc:HGNC:4574] | 21649 | -0.027 | -0.3628 | No |
| 113 | GIPR | gastric inhibitory polypeptide receptor [Source:HGNC Symbol;Acc:HGNC:4271] | 21736 | -0.027 | -0.3639 | No |
| 114 | MC4R | melanocortin 4 receptor [Source:HGNC Symbol;Acc:HGNC:6932] | 22041 | -0.029 | -0.3706 | No |
| 115 | CHRND | cholinergic receptor nicotinic delta subunit [Source:HGNC Symbol;Acc:HGNC:1965] | 22306 | -0.031 | -0.3761 | No |
| 116 | CHRNA5 | cholinergic receptor nicotinic alpha 5 subunit [Source:HGNC Symbol;Acc:HGNC:1959] | 22612 | -0.033 | -0.3826 | No |
| 117 | TRPV1 | transient receptor potential cation channel subfamily V member 1 [Source:HGNC Symbol;Acc:HGNC:12716] | 22668 | -0.033 | -0.3828 | No |
| 118 | GRM1 | glutamate metabotropic receptor 1 [Source:HGNC Symbol;Acc:HGNC:4593] | 22725 | -0.033 | -0.3829 | No |
| 119 | KISS1R | KISS1 receptor [Source:HGNC Symbol;Acc:HGNC:4510] | 22816 | -0.034 | -0.3838 | No |
| 120 | GLRA2 | glycine receptor alpha 2 [Source:HGNC Symbol;Acc:HGNC:4327] | 23024 | -0.035 | -0.3878 | No |
| 121 | GRIA3 | glutamate ionotropic receptor AMPA type subunit 3 [Source:HGNC Symbol;Acc:HGNC:4573] | 23399 | -0.038 | -0.3958 | No |
| 122 | NPFFR2 | neuropeptide FF receptor 2 [Source:HGNC Symbol;Acc:HGNC:4525] | 23481 | -0.038 | -0.3964 | No |
| 123 | GHSR | growth hormone secretagogue receptor [Source:HGNC Symbol;Acc:HGNC:4267] | 23612 | -0.039 | -0.3982 | No |
| 124 | S1PR5 | sphingosine-1-phosphate receptor 5 [Source:HGNC Symbol;Acc:HGNC:14299] | 23881 | -0.041 | -0.4034 | No |
| 125 | P2RX2 | purinergic receptor P2X 2 [Source:HGNC Symbol;Acc:HGNC:15459] | 24014 | -0.042 | -0.4052 | No |
| 126 | GNRHR | gonadotropin releasing hormone receptor [Source:HGNC Symbol;Acc:HGNC:4421] | 24065 | -0.042 | -0.4048 | No |
| 127 | PTGER3 | prostaglandin E receptor 3 [Source:HGNC Symbol;Acc:HGNC:9595] | 24213 | -0.043 | -0.4069 | No |
| 128 | MLNR | motilin receptor [Source:HGNC Symbol;Acc:HGNC:4495] | 24449 | -0.045 | -0.4111 | No |
| 129 | SSTR5 | somatostatin receptor 5 [Source:HGNC Symbol;Acc:HGNC:11334] | 24937 | -0.048 | -0.4217 | No |
| 130 | MC3R | melanocortin 3 receptor [Source:HGNC Symbol;Acc:HGNC:6931] | 25175 | -0.050 | -0.4258 | No |
| 131 | CHRNB3 | cholinergic receptor nicotinic beta 3 subunit [Source:HGNC Symbol;Acc:HGNC:1963] | 25182 | -0.050 | -0.4240 | No |
| 132 | TRHR | thyrotropin releasing hormone receptor [Source:HGNC Symbol;Acc:HGNC:12299] | 25204 | -0.050 | -0.4225 | No |
| 133 | GRIK5 | glutamate ionotropic receptor kainate type subunit 5 [Source:HGNC Symbol;Acc:HGNC:4583] | 25417 | -0.052 | -0.4259 | No |
| 134 | MTNR1B | melatonin receptor 1B [Source:HGNC Symbol;Acc:HGNC:7464] | 25551 | -0.052 | -0.4273 | No |
| 135 | PRLHR | prolactin releasing hormone receptor [Source:HGNC Symbol;Acc:HGNC:4464] | 25714 | -0.054 | -0.4293 | No |
| 136 | GH1 | growth hormone 1 [Source:HGNC Symbol;Acc:HGNC:4261] | 26168 | -0.057 | -0.4387 | No |
| 137 | HTR2C | 5-hydroxytryptamine receptor 2C [Source:HGNC Symbol;Acc:HGNC:5295] | 26208 | -0.057 | -0.4374 | No |
| 138 | GRIN2D | glutamate ionotropic receptor NMDA type subunit 2D [Source:HGNC Symbol;Acc:HGNC:4588] | 26595 | -0.060 | -0.4449 | No |
| 139 | HTR1A | 5-hydroxytryptamine receptor 1A [Source:HGNC Symbol;Acc:HGNC:5286] | 26705 | -0.061 | -0.4453 | No |
| 140 | ADRA1A | adrenoceptor alpha 1A [Source:HGNC Symbol;Acc:HGNC:277] | 27013 | -0.063 | -0.4507 | No |
| 141 | TAAR8 | trace amine associated receptor 8 [Source:HGNC Symbol;Acc:HGNC:14964] | 27294 | -0.065 | -0.4553 | No |
| 142 | GRIK4 | glutamate ionotropic receptor kainate type subunit 4 [Source:HGNC Symbol;Acc:HGNC:4582] | 27307 | -0.066 | -0.4530 | No |
| 143 | MCHR2 | melanin concentrating hormone receptor 2 [Source:HGNC Symbol;Acc:HGNC:20867] | 27325 | -0.066 | -0.4509 | No |
| 144 | CHRM2 | cholinergic receptor muscarinic 2 [Source:HGNC Symbol;Acc:HGNC:1951] | 27332 | -0.066 | -0.4484 | No |
| 145 | LEP | leptin [Source:HGNC Symbol;Acc:HGNC:6553] | 27432 | -0.067 | -0.4483 | No |
| 146 | NPY4R | neuropeptide Y receptor Y4 [Source:HGNC Symbol;Acc:HGNC:9329] | 27597 | -0.068 | -0.4499 | No |
| 147 | HRH3 | histamine receptor H3 [Source:HGNC Symbol;Acc:HGNC:5184] | 27724 | -0.069 | -0.4504 | No |
| 148 | TAAR5 | trace amine associated receptor 5 [Source:HGNC Symbol;Acc:HGNC:30236] | 27810 | -0.070 | -0.4498 | No |
| 149 | NPY1R | neuropeptide Y receptor Y1 [Source:HGNC Symbol;Acc:HGNC:7956] | 27895 | -0.070 | -0.4492 | No |
| 150 | LTB4R2 | leukotriene B4 receptor 2 [Source:HGNC Symbol;Acc:HGNC:19260] | 28020 | -0.071 | -0.4496 | No |
| 151 | TACR3 | tachykinin receptor 3 [Source:HGNC Symbol;Acc:HGNC:11528] | 28021 | -0.071 | -0.4468 | No |
| 152 | NR3C1 | nuclear receptor subfamily 3 group C member 1 [Source:HGNC Symbol;Acc:HGNC:7978] | 28209 | -0.073 | -0.4487 | No |
| 153 | BDKRB2 | bradykinin receptor B2 [Source:HGNC Symbol;Acc:HGNC:1030] | 28223 | -0.073 | -0.4462 | No |
| 154 | CHRNG | cholinergic receptor nicotinic gamma subunit [Source:HGNC Symbol;Acc:HGNC:1967] | 28230 | -0.073 | -0.4434 | No |
| 155 | CHRM3 | cholinergic receptor muscarinic 3 [Source:HGNC Symbol;Acc:HGNC:1952] | 28286 | -0.074 | -0.4420 | No |
| 156 | TAAR6 | trace amine associated receptor 6 [Source:HGNC Symbol;Acc:HGNC:20978] | 28974 | -0.080 | -0.4564 | Yes |
| 157 | GCGR | glucagon receptor [Source:HGNC Symbol;Acc:HGNC:4192] | 29050 | -0.080 | -0.4552 | Yes |
| 158 | FSHR | follicle stimulating hormone receptor [Source:HGNC Symbol;Acc:HGNC:3969] | 29086 | -0.081 | -0.4529 | Yes |
| 159 | GRIA1 | glutamate ionotropic receptor AMPA type subunit 1 [Source:HGNC Symbol;Acc:HGNC:4571] | 29188 | -0.082 | -0.4523 | Yes |
| 160 | CHRNA2 | cholinergic receptor nicotinic alpha 2 subunit [Source:HGNC Symbol;Acc:HGNC:1956] | 29236 | -0.082 | -0.4503 | Yes |
| 161 | PRL | prolactin [Source:HGNC Symbol;Acc:HGNC:9445] | 29427 | -0.084 | -0.4518 | Yes |
| 162 | HTR2B | 5-hydroxytryptamine receptor 2B [Source:HGNC Symbol;Acc:HGNC:5294] | 29528 | -0.085 | -0.4511 | Yes |
| 163 | GRIN2B | glutamate ionotropic receptor NMDA type subunit 2B [Source:HGNC Symbol;Acc:HGNC:4586] | 29542 | -0.085 | -0.4481 | Yes |
| 164 | GRM4 | glutamate metabotropic receptor 4 [Source:HGNC Symbol;Acc:HGNC:4596] | 29568 | -0.085 | -0.4453 | Yes |
| 165 | CCKAR | cholecystokinin A receptor [Source:HGNC Symbol;Acc:HGNC:1570] | 29625 | -0.086 | -0.4434 | Yes |
| 166 | GRM2 | glutamate metabotropic receptor 2 [Source:HGNC Symbol;Acc:HGNC:4594] | 29626 | -0.086 | -0.4400 | Yes |
| 167 | EDNRB | endothelin receptor type B [Source:HGNC Symbol;Acc:HGNC:3180] | 29975 | -0.089 | -0.4454 | Yes |
| 168 | AGTR2 | angiotensin II receptor type 2 [Source:HGNC Symbol;Acc:HGNC:338] | 30049 | -0.090 | -0.4437 | Yes |
| 169 | F2RL2 | coagulation factor II thrombin receptor like 2 [Source:HGNC Symbol;Acc:HGNC:3539] | 30124 | -0.091 | -0.4420 | Yes |
| 170 | EDNRA | endothelin receptor type A [Source:HGNC Symbol;Acc:HGNC:3179] | 30139 | -0.091 | -0.4388 | Yes |
| 171 | GABRB2 | gamma-aminobutyric acid type A receptor subunit beta2 [Source:HGNC Symbol;Acc:HGNC:4082] | 30418 | -0.094 | -0.4422 | Yes |
| 172 | S1PR3 | sphingosine-1-phosphate receptor 3 [Source:HGNC Symbol;Acc:HGNC:3167] | 30452 | -0.094 | -0.4394 | Yes |
| 173 | F2 | "coagulation factor II, thrombin [Source:HGNC Symbol;Acc:HGNC:3535]" | 30493 | -0.095 | -0.4366 | Yes |
| 174 | GALR1 | galanin receptor 1 [Source:HGNC Symbol;Acc:HGNC:4132] | 30585 | -0.096 | -0.4352 | Yes |
| 175 | GABRP | gamma-aminobutyric acid type A receptor subunit pi [Source:HGNC Symbol;Acc:HGNC:4089] | 30606 | -0.096 | -0.4319 | Yes |
| 176 | GH2 | growth hormone 2 [Source:HGNC Symbol;Acc:HGNC:4262] | 30618 | -0.096 | -0.4284 | Yes |
| 177 | OXTR | oxytocin receptor [Source:HGNC Symbol;Acc:HGNC:8529] | 30681 | -0.097 | -0.4262 | Yes |
| 178 | HTR4 | 5-hydroxytryptamine receptor 4 [Source:HGNC Symbol;Acc:HGNC:5299] | 30735 | -0.098 | -0.4237 | Yes |
| 179 | GABRA2 | gamma-aminobutyric acid type A receptor subunit alpha2 [Source:HGNC Symbol;Acc:HGNC:4076] | 30806 | -0.098 | -0.4217 | Yes |
| 180 | PRSS3 | serine protease 3 [Source:HGNC Symbol;Acc:HGNC:9486] | 31002 | -0.100 | -0.4227 | Yes |
| 181 | APLNR | apelin receptor [Source:HGNC Symbol;Acc:HGNC:339] | 31007 | -0.100 | -0.4188 | Yes |
| 182 | HCRTR2 | hypocretin receptor 2 [Source:HGNC Symbol;Acc:HGNC:4849] | 31012 | -0.101 | -0.4150 | Yes |
| 183 | GRIK3 | glutamate ionotropic receptor kainate type subunit 3 [Source:HGNC Symbol;Acc:HGNC:4581] | 31257 | -0.104 | -0.4172 | Yes |
| 184 | HTR6 | 5-hydroxytryptamine receptor 6 [Source:HGNC Symbol;Acc:HGNC:5301] | 31282 | -0.104 | -0.4137 | Yes |
| 185 | CGA | "glycoprotein hormones, alpha polypeptide [Source:HGNC Symbol;Acc:HGNC:1885]" | 31409 | -0.105 | -0.4128 | Yes |
| 186 | P2RY6 | pyrimidinergic receptor P2Y6 [Source:HGNC Symbol;Acc:HGNC:8543] | 31486 | -0.106 | -0.4105 | Yes |
| 187 | NPY5R | neuropeptide Y receptor Y5 [Source:HGNC Symbol;Acc:HGNC:7958] | 31638 | -0.108 | -0.4102 | Yes |
| 188 | PLG | plasminogen [Source:HGNC Symbol;Acc:HGNC:9071] | 31650 | -0.108 | -0.4062 | Yes |
| 189 | ADRB2 | adrenoceptor beta 2 [Source:HGNC Symbol;Acc:HGNC:286] | 31665 | -0.109 | -0.4023 | Yes |
| 190 | HTR5A | 5-hydroxytryptamine receptor 5A [Source:HGNC Symbol;Acc:HGNC:5300] | 31727 | -0.109 | -0.3995 | Yes |
| 191 | CHRNB2 | cholinergic receptor nicotinic beta 2 subunit [Source:HGNC Symbol;Acc:HGNC:1962] | 31735 | -0.110 | -0.3954 | Yes |
| 192 | RXFP1 | relaxin family peptide receptor 1 [Source:HGNC Symbol;Acc:HGNC:19718] | 31775 | -0.110 | -0.3920 | Yes |
| 193 | CHRNA1 | cholinergic receptor nicotinic alpha 1 subunit [Source:HGNC Symbol;Acc:HGNC:1955] | 31796 | -0.110 | -0.3882 | Yes |
| 194 | GRM8 | glutamate metabotropic receptor 8 [Source:HGNC Symbol;Acc:HGNC:4600] | 31826 | -0.111 | -0.3846 | Yes |
| 195 | NTSR1 | neurotensin receptor 1 [Source:HGNC Symbol;Acc:HGNC:8039] | 31999 | -0.113 | -0.3846 | Yes |
| 196 | TAAR9 | trace amine associated receptor 9 [Source:HGNC Symbol;Acc:HGNC:20977] | 32015 | -0.113 | -0.3805 | Yes |
| 197 | GRM5 | glutamate metabotropic receptor 5 [Source:HGNC Symbol;Acc:HGNC:4597] | 32047 | -0.114 | -0.3768 | Yes |
| 198 | P2RY4 | pyrimidinergic receptor P2Y4 [Source:HGNC Symbol;Acc:HGNC:8542] | 32110 | -0.114 | -0.3739 | Yes |
| 199 | SSTR1 | somatostatin receptor 1 [Source:HGNC Symbol;Acc:HGNC:11330] | 32282 | -0.117 | -0.3737 | Yes |
| 200 | AVPR1B | arginine vasopressin receptor 1B [Source:HGNC Symbol;Acc:HGNC:896] | 32414 | -0.119 | -0.3724 | Yes |
| 201 | PTGIR | prostaglandin I2 receptor [Source:HGNC Symbol;Acc:HGNC:9602] | 32575 | -0.121 | -0.3717 | Yes |
| 202 | TSHB | thyroid stimulating hormone subunit beta [Source:HGNC Symbol;Acc:HGNC:12372] | 32688 | -0.123 | -0.3697 | Yes |
| 203 | NPFFR1 | neuropeptide FF receptor 1 [Source:HGNC Symbol;Acc:HGNC:17425] | 32744 | -0.124 | -0.3663 | Yes |
| 204 | CYSLTR1 | cysteinyl leukotriene receptor 1 [Source:HGNC Symbol;Acc:HGNC:17451] | 32748 | -0.124 | -0.3615 | Yes |
| 205 | DRD1 | dopamine receptor D1 [Source:HGNC Symbol;Acc:HGNC:3020] | 32942 | -0.127 | -0.3614 | Yes |
| 206 | MTNR1A | melatonin receptor 1A [Source:HGNC Symbol;Acc:HGNC:7463] | 33114 | -0.130 | -0.3607 | Yes |
| 207 | PTGFR | prostaglandin F receptor [Source:HGNC Symbol;Acc:HGNC:9600] | 33350 | -0.134 | -0.3615 | Yes |
| 208 | GPR50 | G protein-coupled receptor 50 [Source:HGNC Symbol;Acc:HGNC:4506] | 33435 | -0.135 | -0.3583 | Yes |
| 209 | CHRNA3 | cholinergic receptor nicotinic alpha 3 subunit [Source:HGNC Symbol;Acc:HGNC:1957] | 33459 | -0.136 | -0.3536 | Yes |
| 210 | TAAR1 | trace amine associated receptor 1 [Source:HGNC Symbol;Acc:HGNC:17734] | 33512 | -0.137 | -0.3495 | Yes |
| 211 | CALCR | calcitonin receptor [Source:HGNC Symbol;Acc:HGNC:1440] | 33574 | -0.138 | -0.3457 | Yes |
| 212 | CNR2 | cannabinoid receptor 2 [Source:HGNC Symbol;Acc:HGNC:2160] | 33695 | -0.140 | -0.3432 | Yes |
| 213 | TSHR | thyroid stimulating hormone receptor [Source:HGNC Symbol;Acc:HGNC:12373] | 33713 | -0.140 | -0.3382 | Yes |
| 214 | LPAR4 | lysophosphatidic acid receptor 4 [Source:HGNC Symbol;Acc:HGNC:4478] | 33816 | -0.142 | -0.3352 | Yes |
| 215 | MAS1 | "MAS1 proto-oncogene, G protein-coupled receptor [Source:HGNC Symbol;Acc:HGNC:6899]" | 33967 | -0.145 | -0.3333 | Yes |
| 216 | GPR35 | G protein-coupled receptor 35 [Source:HGNC Symbol;Acc:HGNC:4492] | 33999 | -0.145 | -0.3284 | Yes |
| 217 | GHR | growth hormone receptor [Source:HGNC Symbol;Acc:HGNC:4263] | 34005 | -0.145 | -0.3228 | Yes |
| 218 | HTR1B | 5-hydroxytryptamine receptor 1B [Source:HGNC Symbol;Acc:HGNC:5287] | 34167 | -0.149 | -0.3211 | Yes |
| 219 | SSTR3 | somatostatin receptor 3 [Source:HGNC Symbol;Acc:HGNC:11332] | 34207 | -0.149 | -0.3162 | Yes |
| 220 | BDKRB1 | bradykinin receptor B1 [Source:HGNC Symbol;Acc:HGNC:1029] | 34460 | -0.154 | -0.3166 | Yes |
| 221 | LEPR | leptin receptor [Source:HGNC Symbol;Acc:HGNC:6554] | 34476 | -0.155 | -0.3109 | Yes |
| 222 | GRIK1 | glutamate ionotropic receptor kainate type subunit 1 [Source:HGNC Symbol;Acc:HGNC:4579] | 34703 | -0.160 | -0.3104 | Yes |
| 223 | TACR1 | tachykinin receptor 1 [Source:HGNC Symbol;Acc:HGNC:11526] | 34737 | -0.161 | -0.3049 | Yes |
| 224 | PRSS2 | serine protease 2 [Source:HGNC Symbol;Acc:HGNC:9483] | 34755 | -0.161 | -0.2990 | Yes |
| 225 | CHRNA9 | cholinergic receptor nicotinic alpha 9 subunit [Source:HGNC Symbol;Acc:HGNC:14079] | 34761 | -0.161 | -0.2928 | Yes |
| 226 | NMUR1 | neuromedin U receptor 1 [Source:HGNC Symbol;Acc:HGNC:4518] | 34940 | -0.165 | -0.2909 | Yes |
| 227 | CHRNA6 | cholinergic receptor nicotinic alpha 6 subunit [Source:HGNC Symbol;Acc:HGNC:15963] | 35112 | -0.169 | -0.2886 | Yes |
| 228 | P2RY14 | purinergic receptor P2Y14 [Source:HGNC Symbol;Acc:HGNC:16442] | 35366 | -0.176 | -0.2882 | Yes |
| 229 | PTGER2 | prostaglandin E receptor 2 [Source:HGNC Symbol;Acc:HGNC:9594] | 35529 | -0.180 | -0.2853 | Yes |
| 230 | CHRM4 | cholinergic receptor muscarinic 4 [Source:HGNC Symbol;Acc:HGNC:1953] | 35560 | -0.181 | -0.2789 | Yes |
| 231 | GRPR | gastrin releasing peptide receptor [Source:HGNC Symbol;Acc:HGNC:4609] | 35597 | -0.182 | -0.2727 | Yes |
| 232 | F2R | coagulation factor II thrombin receptor [Source:HGNC Symbol;Acc:HGNC:3537] | 35689 | -0.184 | -0.2678 | Yes |
| 233 | CHRNA7 | cholinergic receptor nicotinic alpha 7 subunit [Source:HGNC Symbol;Acc:HGNC:1960] | 35812 | -0.188 | -0.2635 | Yes |
| 234 | SSTR2 | somatostatin receptor 2 [Source:HGNC Symbol;Acc:HGNC:11331] | 35828 | -0.189 | -0.2564 | Yes |
| 235 | ADRA2A | adrenoceptor alpha 2A [Source:HGNC Symbol;Acc:HGNC:281] | 35832 | -0.189 | -0.2491 | Yes |
| 236 | CHRM5 | cholinergic receptor muscarinic 5 [Source:HGNC Symbol;Acc:HGNC:1954] | 35860 | -0.190 | -0.2423 | Yes |
| 237 | PTH2R | parathyroid hormone 2 receptor [Source:HGNC Symbol;Acc:HGNC:9609] | 35874 | -0.190 | -0.2352 | Yes |
| 238 | ADORA2A | adenosine A2a receptor [Source:HGNC Symbol;Acc:HGNC:263] | 35916 | -0.191 | -0.2287 | Yes |
| 239 | UTS2R | urotensin 2 receptor [Source:HGNC Symbol;Acc:HGNC:4468] | 35941 | -0.192 | -0.2218 | Yes |
| 240 | CALCRL | calcitonin receptor like receptor [Source:HGNC Symbol;Acc:HGNC:16709] | 35962 | -0.193 | -0.2147 | Yes |
| 241 | PRSS1 | serine protease 1 [Source:HGNC Symbol;Acc:HGNC:9475] | 36163 | -0.199 | -0.2120 | Yes |
| 242 | HTR7 | 5-hydroxytryptamine receptor 7 [Source:HGNC Symbol;Acc:HGNC:5302] | 36186 | -0.200 | -0.2047 | Yes |
| 243 | HTR1F | 5-hydroxytryptamine receptor 1F [Source:HGNC Symbol;Acc:HGNC:5292] | 36201 | -0.200 | -0.1972 | Yes |
| 244 | GZMA | granzyme A [Source:HGNC Symbol;Acc:HGNC:4708] | 36316 | -0.205 | -0.1921 | Yes |
| 245 | P2RX7 | purinergic receptor P2X 7 [Source:HGNC Symbol;Acc:HGNC:8537] | 36472 | -0.210 | -0.1878 | Yes |
| 246 | LPAR6 | lysophosphatidic acid receptor 6 [Source:HGNC Symbol;Acc:HGNC:15520] | 36586 | -0.215 | -0.1822 | Yes |
| 247 | S1PR1 | sphingosine-1-phosphate receptor 1 [Source:HGNC Symbol;Acc:HGNC:3165] | 36641 | -0.217 | -0.1750 | Yes |
| 248 | AGTR1 | angiotensin II receptor type 1 [Source:HGNC Symbol;Acc:HGNC:336] | 36675 | -0.219 | -0.1673 | Yes |
| 249 | GABRR2 | gamma-aminobutyric acid type A receptor subunit rho2 [Source:HGNC Symbol;Acc:HGNC:4091] | 36691 | -0.220 | -0.1590 | Yes |
| 250 | PTGDR | prostaglandin D2 receptor [Source:HGNC Symbol;Acc:HGNC:9591] | 36694 | -0.220 | -0.1504 | Yes |
| 251 | ADCYAP1R1 | ADCYAP receptor type I [Source:HGNC Symbol;Acc:HGNC:242] | 36735 | -0.221 | -0.1427 | Yes |
| 252 | P2RX5 | purinergic receptor P2X 5 [Source:HGNC Symbol;Acc:HGNC:8536] | 36776 | -0.224 | -0.1350 | Yes |
| 253 | P2RY13 | purinergic receptor P2Y13 [Source:HGNC Symbol;Acc:HGNC:4537] | 36787 | -0.224 | -0.1264 | Yes |
| 254 | AVPR1A | arginine vasopressin receptor 1A [Source:HGNC Symbol;Acc:HGNC:895] | 36808 | -0.225 | -0.1180 | Yes |
| 255 | HTR2A | 5-hydroxytryptamine receptor 2A [Source:HGNC Symbol;Acc:HGNC:5293] | 36934 | -0.232 | -0.1121 | Yes |
| 256 | PTGER4 | prostaglandin E receptor 4 [Source:HGNC Symbol;Acc:HGNC:9596] | 36996 | -0.236 | -0.1044 | Yes |
| 257 | P2RX1 | purinergic receptor P2X 1 [Source:HGNC Symbol;Acc:HGNC:8533] | 37178 | -0.247 | -0.0993 | Yes |
| 258 | FPR1 | formyl peptide receptor 1 [Source:HGNC Symbol;Acc:HGNC:3826] | 37446 | -0.262 | -0.0958 | Yes |
| 259 | MCHR1 | melanin concentrating hormone receptor 1 [Source:HGNC Symbol;Acc:HGNC:4479] | 37524 | -0.268 | -0.0872 | Yes |
| 260 | P2RY8 | P2Y receptor family member 8 [Source:HGNC Symbol;Acc:HGNC:15524] | 37533 | -0.269 | -0.0769 | Yes |
| 261 | C5AR1 | complement C5a receptor 1 [Source:HGNC Symbol;Acc:HGNC:1338] | 37574 | -0.272 | -0.0672 | Yes |
| 262 | PTAFR | platelet activating factor receptor [Source:HGNC Symbol;Acc:HGNC:9582] | 37638 | -0.277 | -0.0579 | Yes |
| 263 | C3AR1 | complement C3a receptor 1 [Source:HGNC Symbol;Acc:HGNC:1319] | 37679 | -0.281 | -0.0479 | Yes |
| 264 | P2RY1 | purinergic receptor P2Y1 [Source:HGNC Symbol;Acc:HGNC:8539] | 37922 | -0.307 | -0.0420 | Yes |
| 265 | FPR2 | formyl peptide receptor 2 [Source:HGNC Symbol;Acc:HGNC:3827] | 37958 | -0.311 | -0.0307 | Yes |
| 266 | GRIN3A | glutamate ionotropic receptor NMDA type subunit 3A [Source:HGNC Symbol;Acc:HGNC:16767] | 37998 | -0.317 | -0.0192 | Yes |
| 267 | HRH2 | histamine receptor H2 [Source:HGNC Symbol;Acc:HGNC:5183] | 38019 | -0.320 | -0.0072 | Yes |
| 268 | P2RY10 | P2Y receptor family member 10 [Source:HGNC Symbol;Acc:HGNC:19906] | 38040 | -0.323 | 0.0050 | Yes |
| 269 | FPR3 | formyl peptide receptor 3 [Source:HGNC Symbol;Acc:HGNC:3828] | 38086 | -0.330 | 0.0169 | Yes |
| 270 | CYSLTR2 | cysteinyl leukotriene receptor 2 [Source:HGNC Symbol;Acc:HGNC:18274] | 38233 | -0.376 | 0.0279 | Yes |
Table: GSEA details [plain text format]

  

Fig 2: KEGG\_NEUROACTIVE\_LIGAND\_RECEPTOR\_INTERACTION      
 Blue-Pink O' Gram in the Space of the Analyzed GeneSet

  

Fig 3: KEGG\_NEUROACTIVE\_LIGAND\_RECEPTOR\_INTERACTION: Random ES distribution      
 Gene set null distribution of ES for **KEGG\_NEUROACTIVE\_LIGAND\_RECEPTOR\_INTERACTION**

  
