# Supplementary material for: Simultaneous Presentation of Multiple Myeloma and Lung Cancer: Case Report and Gene Bioinformatics Analysis
Source: Front Oncol. 2022 Jun 13;12:859735. doi: 10.3389/fonc.2022.859735 (PMC9235397; doi:10.3389/fonc.2022.859735)
Supplement: Supplementary file 1 [file DataSheet_1.zip › The bioinformatic analysis of MM and lung cancer supplementary materials/Enrichment analysis/MECR/GSEA_4.1.0/LUAD TCGA/KEGG.Gsea.1639041756227/KEGG_OXIDATIVE_PHOSPHORYLATION.html]

Details for gene set KEGG\_OXIDATIVE\_PHOSPHORYLATION[GSEA]

|  || Dataset | ExpData\_collapsed\_to\_symbols.ENSG00000116353\_profile\_in\_ExpData.cls #ENSG00000116353 |
| Phenotype | ENSG00000116353\_profile\_in\_ExpData.cls#ENSG00000116353 |
| Upregulated in class | ENSG00000116353\_pos |
| GeneSet | KEGG\_OXIDATIVE\_PHOSPHORYLATION |
| Enrichment Score (ES) | 0.7611882 |
| Normalized Enrichment Score (NES) | 3.2493382 |
| Nominal p-value | 0.0 |
| FDR q-value | 0.0 |
| FWER p-Value | 0.0 |
Table: GSEA Results Summary

  

Fig 1: Enrichment plot: KEGG\_OXIDATIVE\_PHOSPHORYLATION      
 Profile of the Running ES Score & Positions of GeneSet Members on the Rank Ordered List

  

| SYMBOL | TITLE | RANK IN GENE LIST | RANK METRIC SCORE | RUNNING ES | CORE ENRICHMENT || 1 | NDUFS8 | NADH:ubiquinone oxidoreductase core subunit S8 [Source:HGNC Symbol;Acc:HGNC:7715] | 34 | 0.446 | 0.0172 | Yes |
| 2 | NDUFS5 | NADH:ubiquinone oxidoreductase subunit S5 [Source:HGNC Symbol;Acc:HGNC:7712] | 78 | 0.411 | 0.0327 | Yes |
| 3 | NDUFA2 | NADH:ubiquinone oxidoreductase subunit A2 [Source:HGNC Symbol;Acc:HGNC:7685] | 110 | 0.398 | 0.0480 | Yes |
| 4 | NDUFB10 | NADH:ubiquinone oxidoreductase subunit B10 [Source:HGNC Symbol;Acc:HGNC:7696] | 142 | 0.385 | 0.0628 | Yes |
| 5 | ATP5PO | ATP synthase peripheral stalk subunit OSCP [Source:HGNC Symbol;Acc:HGNC:850] | 231 | 0.366 | 0.0754 | Yes |
| 6 | NDUFA11 | NADH:ubiquinone oxidoreductase subunit A11 [Source:HGNC Symbol;Acc:HGNC:20371] | 257 | 0.359 | 0.0893 | Yes |
| 7 | NDUFB7 | NADH:ubiquinone oxidoreductase subunit B7 [Source:HGNC Symbol;Acc:HGNC:7702] | 278 | 0.355 | 0.1031 | Yes |
| 8 | NDUFV1 | NADH:ubiquinone oxidoreductase core subunit V1 [Source:HGNC Symbol;Acc:HGNC:7716] | 308 | 0.349 | 0.1165 | Yes |
| 9 | NDUFV3 | NADH:ubiquinone oxidoreductase subunit V3 [Source:HGNC Symbol;Acc:HGNC:7719] | 321 | 0.346 | 0.1302 | Yes |
| 10 | ATP5PD | ATP synthase peripheral stalk subunit d [Source:HGNC Symbol;Acc:HGNC:845] | 341 | 0.343 | 0.1436 | Yes |
| 11 | NDUFB2 | NADH:ubiquinone oxidoreductase subunit B2 [Source:HGNC Symbol;Acc:HGNC:7697] | 440 | 0.327 | 0.1544 | Yes |
| 12 | SDHB | succinate dehydrogenase complex iron sulfur subunit B [Source:HGNC Symbol;Acc:HGNC:10681] | 443 | 0.327 | 0.1676 | Yes |
| 13 | NDUFA6 | NADH:ubiquinone oxidoreductase subunit A6 [Source:HGNC Symbol;Acc:HGNC:7690] | 471 | 0.324 | 0.1800 | Yes |
| 14 | ATP5F1D | ATP synthase F1 subunit delta [Source:HGNC Symbol;Acc:HGNC:837] | 480 | 0.321 | 0.1928 | Yes |
| 15 | NDUFS7 | NADH:ubiquinone oxidoreductase core subunit S7 [Source:HGNC Symbol;Acc:HGNC:7714] | 511 | 0.317 | 0.2049 | Yes |
| 16 | LHPP | phospholysine phosphohistidine inorganic pyrophosphate phosphatase [Source:HGNC Symbol;Acc:HGNC:30042] | 554 | 0.312 | 0.2164 | Yes |
| 17 | ATP6V0B | ATPase H+ transporting V0 subunit b [Source:HGNC Symbol;Acc:HGNC:861] | 605 | 0.307 | 0.2276 | Yes |
| 18 | UQCR10 | "ubiquinol-cytochrome c reductase, complex III subunit X [Source:HGNC Symbol;Acc:HGNC:30863]" | 623 | 0.305 | 0.2395 | Yes |
| 19 | NDUFC1 | NADH:ubiquinone oxidoreductase subunit C1 [Source:HGNC Symbol;Acc:HGNC:7705] | 648 | 0.303 | 0.2512 | Yes |
| 20 | UQCRC1 | ubiquinol-cytochrome c reductase core protein 1 [Source:HGNC Symbol;Acc:HGNC:12585] | 653 | 0.302 | 0.2633 | Yes |
| 21 | NDUFS6 | NADH:ubiquinone oxidoreductase subunit S6 [Source:HGNC Symbol;Acc:HGNC:7713] | 687 | 0.298 | 0.2745 | Yes |
| 22 | ATP5PF | ATP synthase peripheral stalk subunit F6 [Source:HGNC Symbol;Acc:HGNC:847] | 702 | 0.297 | 0.2862 | Yes |
| 23 | CYC1 | cytochrome c1 [Source:HGNC Symbol;Acc:HGNC:2579] | 712 | 0.296 | 0.2979 | Yes |
| 24 | NDUFA4 | NDUFA4 mitochondrial complex associated [Source:HGNC Symbol;Acc:HGNC:7687] | 738 | 0.293 | 0.3092 | Yes |
| 25 | NDUFB8 | NADH:ubiquinone oxidoreductase subunit B8 [Source:HGNC Symbol;Acc:HGNC:7703] | 748 | 0.293 | 0.3208 | Yes |
| 26 | ATP5MC1 | ATP synthase membrane subunit c locus 1 [Source:HGNC Symbol;Acc:HGNC:841] | 755 | 0.293 | 0.3325 | Yes |
| 27 | UQCR11 | "ubiquinol-cytochrome c reductase, complex III subunit XI [Source:HGNC Symbol;Acc:HGNC:30862]" | 764 | 0.292 | 0.3441 | Yes |
| 28 | ATP5ME | ATP synthase membrane subunit e [Source:HGNC Symbol;Acc:HGNC:846] | 767 | 0.291 | 0.3558 | Yes |
| 29 | ATP6V1F | ATPase H+ transporting V1 subunit F [Source:HGNC Symbol;Acc:HGNC:16832] | 808 | 0.287 | 0.3664 | Yes |
| 30 | UQCRQ | ubiquinol-cytochrome c reductase complex III subunit VII [Source:HGNC Symbol;Acc:HGNC:29594] | 818 | 0.286 | 0.3778 | Yes |
| 31 | PPA2 | inorganic pyrophosphatase 2 [Source:HGNC Symbol;Acc:HGNC:28883] | 836 | 0.285 | 0.3889 | Yes |
| 32 | NDUFS3 | NADH:ubiquinone oxidoreductase core subunit S3 [Source:HGNC Symbol;Acc:HGNC:7710] | 842 | 0.284 | 0.4003 | Yes |
| 33 | NDUFA3 | NADH:ubiquinone oxidoreductase subunit A3 [Source:HGNC Symbol;Acc:HGNC:7686] | 864 | 0.282 | 0.4112 | Yes |
| 34 | NDUFA7 | NADH:ubiquinone oxidoreductase subunit A7 [Source:HGNC Symbol;Acc:HGNC:7691] | 878 | 0.281 | 0.4222 | Yes |
| 35 | NDUFB9 | NADH:ubiquinone oxidoreductase subunit B9 [Source:HGNC Symbol;Acc:HGNC:7704] | 915 | 0.278 | 0.4325 | Yes |
| 36 | UQCRH | ubiquinol-cytochrome c reductase hinge protein [Source:HGNC Symbol;Acc:HGNC:12590] | 937 | 0.275 | 0.4431 | Yes |
| 37 | ATP5MF | ATP synthase membrane subunit f [Source:HGNC Symbol;Acc:HGNC:848] | 975 | 0.271 | 0.4532 | Yes |
| 38 | COX5B | cytochrome c oxidase subunit 5B [Source:HGNC Symbol;Acc:HGNC:2269] | 1019 | 0.268 | 0.4629 | Yes |
| 39 | COX4I1 | cytochrome c oxidase subunit 4I1 [Source:HGNC Symbol;Acc:HGNC:2265] | 1051 | 0.266 | 0.4729 | Yes |
| 40 | NDUFB1 | NADH:ubiquinone oxidoreductase subunit B1 [Source:HGNC Symbol;Acc:HGNC:7695] | 1087 | 0.263 | 0.4827 | Yes |
| 41 | ATP5MC2 | ATP synthase membrane subunit c locus 2 [Source:HGNC Symbol;Acc:HGNC:842] | 1094 | 0.263 | 0.4932 | Yes |
| 42 | ATP6V0E1 | ATPase H+ transporting V0 subunit e1 [Source:HGNC Symbol;Acc:HGNC:863] | 1105 | 0.263 | 0.5035 | Yes |
| 43 | COX8A | cytochrome c oxidase subunit 8A [Source:HGNC Symbol;Acc:HGNC:2294] | 1130 | 0.261 | 0.5135 | Yes |
| 44 | NDUFA8 | NADH:ubiquinone oxidoreductase subunit A8 [Source:HGNC Symbol;Acc:HGNC:7692] | 1138 | 0.261 | 0.5239 | Yes |
| 45 | NDUFB4 | NADH:ubiquinone oxidoreductase subunit B4 [Source:HGNC Symbol;Acc:HGNC:7699] | 1212 | 0.254 | 0.5323 | Yes |
| 46 | COX7C | cytochrome c oxidase subunit 7C [Source:HGNC Symbol;Acc:HGNC:2292] | 1244 | 0.252 | 0.5417 | Yes |
| 47 | UQCRHL | ubiquinol-cytochrome c reductase hinge protein like [Source:HGNC Symbol;Acc:HGNC:51714] | 1439 | 0.239 | 0.5464 | Yes |
| 48 | ATP5MG | ATP synthase membrane subunit g [Source:HGNC Symbol;Acc:HGNC:14247] | 1453 | 0.238 | 0.5557 | Yes |
| 49 | ATP5F1E | ATP synthase F1 subunit epsilon [Source:HGNC Symbol;Acc:HGNC:838] | 1468 | 0.237 | 0.5650 | Yes |
| 50 | NDUFAB1 | NADH:ubiquinone oxidoreductase subunit AB1 [Source:HGNC Symbol;Acc:HGNC:7694] | 1579 | 0.230 | 0.5715 | Yes |
| 51 | COX6B1 | cytochrome c oxidase subunit 6B1 [Source:HGNC Symbol;Acc:HGNC:2280] | 1782 | 0.218 | 0.5752 | Yes |
| 52 | COX6A1 | cytochrome c oxidase subunit 6A1 [Source:HGNC Symbol;Acc:HGNC:2277] | 1827 | 0.216 | 0.5828 | Yes |
| 53 | SDHC | succinate dehydrogenase complex subunit C [Source:HGNC Symbol;Acc:HGNC:10682] | 2041 | 0.204 | 0.5856 | Yes |
| 54 | NDUFC2 | NADH:ubiquinone oxidoreductase subunit C2 [Source:HGNC Symbol;Acc:HGNC:7706] | 2151 | 0.199 | 0.5909 | Yes |
| 55 | NDUFA1 | NADH:ubiquinone oxidoreductase subunit A1 [Source:HGNC Symbol;Acc:HGNC:7683] | 2163 | 0.198 | 0.5986 | Yes |
| 56 | ATP6V0E2 | ATPase H+ transporting V0 subunit e2 [Source:HGNC Symbol;Acc:HGNC:21723] | 2345 | 0.191 | 0.6017 | Yes |
| 57 | ATP5F1C | ATP synthase F1 subunit gamma [Source:HGNC Symbol;Acc:HGNC:833] | 2375 | 0.190 | 0.6087 | Yes |
| 58 | NDUFV2 | NADH:ubiquinone oxidoreductase core subunit V2 [Source:HGNC Symbol;Acc:HGNC:7717] | 2413 | 0.188 | 0.6153 | Yes |
| 59 | ATP6V0C | ATPase H+ transporting V0 subunit c [Source:HGNC Symbol;Acc:HGNC:855] | 2497 | 0.184 | 0.6207 | Yes |
| 60 | NDUFS2 | NADH:ubiquinone oxidoreductase core subunit S2 [Source:HGNC Symbol;Acc:HGNC:7708] | 2516 | 0.184 | 0.6277 | Yes |
| 61 | COX7B | cytochrome c oxidase subunit 7B [Source:HGNC Symbol;Acc:HGNC:2291] | 2582 | 0.181 | 0.6333 | Yes |
| 62 | MT-CO2 | mitochondrially encoded cytochrome c oxidase II [Source:HGNC Symbol;Acc:HGNC:7421] | 2703 | 0.176 | 0.6374 | Yes |
| 63 | COX7A2L | cytochrome c oxidase subunit 7A2 like [Source:HGNC Symbol;Acc:HGNC:2289] | 2825 | 0.171 | 0.6412 | Yes |
| 64 | UQCRB | ubiquinol-cytochrome c reductase binding protein [Source:HGNC Symbol;Acc:HGNC:12582] | 2840 | 0.170 | 0.6477 | Yes |
| 65 | COX6C | cytochrome c oxidase subunit 6C [Source:HGNC Symbol;Acc:HGNC:2285] | 2863 | 0.169 | 0.6540 | Yes |
| 66 | MT-ND1 | mitochondrially encoded NADH:ubiquinone oxidoreductase core subunit 1 [Source:HGNC Symbol;Acc:HGNC:7455] | 2906 | 0.168 | 0.6597 | Yes |
| 67 | MT-CO3 | mitochondrially encoded cytochrome c oxidase III [Source:HGNC Symbol;Acc:HGNC:7422] | 2913 | 0.167 | 0.6663 | Yes |
| 68 | MT-ND3 | mitochondrially encoded NADH:ubiquinone oxidoreductase core subunit 3 [Source:HGNC Symbol;Acc:HGNC:7458] | 2921 | 0.167 | 0.6729 | Yes |
| 69 | NDUFA5 | NADH:ubiquinone oxidoreductase subunit A5 [Source:HGNC Symbol;Acc:HGNC:7688] | 3044 | 0.163 | 0.6764 | Yes |
| 70 | NDUFS4 | NADH:ubiquinone oxidoreductase subunit S4 [Source:HGNC Symbol;Acc:HGNC:7711] | 3070 | 0.162 | 0.6823 | Yes |
| 71 | NDUFB5 | NADH:ubiquinone oxidoreductase subunit B5 [Source:HGNC Symbol;Acc:HGNC:7700] | 3101 | 0.161 | 0.6880 | Yes |
| 72 | TCIRG1 | "T cell immune regulator 1, ATPase H+ transporting V0 subunit a3 [Source:HGNC Symbol;Acc:HGNC:11647]" | 3304 | 0.154 | 0.6891 | Yes |
| 73 | ATP6V1E1 | ATPase H+ transporting V1 subunit E1 [Source:HGNC Symbol;Acc:HGNC:857] | 3314 | 0.154 | 0.6951 | Yes |
| 74 | ATP6V1G1 | ATPase H+ transporting V1 subunit G1 [Source:HGNC Symbol;Acc:HGNC:864] | 3414 | 0.151 | 0.6987 | Yes |
| 75 | NDUFB3 | NADH:ubiquinone oxidoreductase subunit B3 [Source:HGNC Symbol;Acc:HGNC:7698] | 3463 | 0.150 | 0.7035 | Yes |
| 76 | MT-ND2 | mitochondrially encoded NADH:ubiquinone oxidoreductase core subunit 2 [Source:HGNC Symbol;Acc:HGNC:7456] | 3586 | 0.146 | 0.7063 | Yes |
| 77 | COX5A | cytochrome c oxidase subunit 5A [Source:HGNC Symbol;Acc:HGNC:2267] | 3688 | 0.142 | 0.7095 | Yes |
| 78 | COX7A2 | cytochrome c oxidase subunit 7A2 [Source:HGNC Symbol;Acc:HGNC:2288] | 3752 | 0.141 | 0.7136 | Yes |
| 79 | MT-CO1 | mitochondrially encoded cytochrome c oxidase I [Source:HGNC Symbol;Acc:HGNC:7419] | 3805 | 0.139 | 0.7179 | Yes |
| 80 | MT-ND6 | mitochondrially encoded NADH:ubiquinone oxidoreductase core subunit 6 [Source:HGNC Symbol;Acc:HGNC:7462] | 3841 | 0.139 | 0.7226 | Yes |
| 81 | NDUFB6 | NADH:ubiquinone oxidoreductase subunit B6 [Source:HGNC Symbol;Acc:HGNC:7701] | 3968 | 0.134 | 0.7249 | Yes |
| 82 | SDHA | succinate dehydrogenase complex flavoprotein subunit A [Source:HGNC Symbol;Acc:HGNC:10680] | 4074 | 0.132 | 0.7275 | Yes |
| 83 | ATP5PB | ATP synthase peripheral stalk-membrane subunit b [Source:HGNC Symbol;Acc:HGNC:840] | 4227 | 0.128 | 0.7288 | Yes |
| 84 | NDUFA10 | NADH:ubiquinone oxidoreductase subunit A10 [Source:HGNC Symbol;Acc:HGNC:7684] | 4232 | 0.128 | 0.7339 | Yes |
| 85 | MT-ND4 | mitochondrially encoded NADH:ubiquinone oxidoreductase core subunit 4 [Source:HGNC Symbol;Acc:HGNC:7459] | 4312 | 0.126 | 0.7370 | Yes |
| 86 | ATP4B | ATPase H+/K+ transporting subunit beta [Source:HGNC Symbol;Acc:HGNC:820] | 4313 | 0.126 | 0.7421 | Yes |
| 87 | ATP6V1C2 | ATPase H+ transporting V1 subunit C2 [Source:HGNC Symbol;Acc:HGNC:18264] | 4345 | 0.125 | 0.7464 | Yes |
| 88 | MT-CYB | mitochondrially encoded cytochrome b [Source:HGNC Symbol;Acc:HGNC:7427] | 4594 | 0.119 | 0.7449 | Yes |
| 89 | ATP5MC3 | ATP synthase membrane subunit c locus 3 [Source:HGNC Symbol;Acc:HGNC:843] | 4619 | 0.119 | 0.7491 | Yes |
| 90 | MT-ATP6 | mitochondrially encoded ATP synthase membrane subunit 6 [Source:HGNC Symbol;Acc:HGNC:7414] | 4718 | 0.116 | 0.7514 | Yes |
| 91 | COX17 | cytochrome c oxidase copper chaperone COX17 [Source:HGNC Symbol;Acc:HGNC:2264] | 4804 | 0.115 | 0.7538 | Yes |
| 92 | COX7A1 | cytochrome c oxidase subunit 7A1 [Source:HGNC Symbol;Acc:HGNC:2287] | 4947 | 0.112 | 0.7547 | Yes |
| 93 | COX4I2 | cytochrome c oxidase subunit 4I2 [Source:HGNC Symbol;Acc:HGNC:16232] | 5116 | 0.109 | 0.7549 | Yes |
| 94 | UQCR10P1 | UQCR10 pseudogene 1 [Source:HGNC Symbol;Acc:HGNC:54960] | 5130 | 0.109 | 0.7589 | Yes |
| 95 | PPA1 | inorganic pyrophosphatase 1 [Source:HGNC Symbol;Acc:HGNC:9226] | 5397 | 0.104 | 0.7564 | Yes |
| 96 | ATP6V1E2 | ATPase H+ transporting V1 subunit E2 [Source:HGNC Symbol;Acc:HGNC:18125] | 5437 | 0.103 | 0.7595 | Yes |
| 97 | COX6A2 | cytochrome c oxidase subunit 6A2 [Source:HGNC Symbol;Acc:HGNC:2279] | 5599 | 0.100 | 0.7595 | Yes |
| 98 | ATP5F1A | ATP synthase F1 subunit alpha [Source:HGNC Symbol;Acc:HGNC:823] | 5690 | 0.099 | 0.7612 | Yes |
| 99 | SDHD | succinate dehydrogenase complex subunit D [Source:HGNC Symbol;Acc:HGNC:10683] | 5864 | 0.096 | 0.7607 | No |
| 100 | MT-ND5 | mitochondrially encoded NADH:ubiquinone oxidoreductase core subunit 5 [Source:HGNC Symbol;Acc:HGNC:7461] | 6253 | 0.089 | 0.7544 | No |
| 101 | UQCRC2 | ubiquinol-cytochrome c reductase core protein 2 [Source:HGNC Symbol;Acc:HGNC:12586] | 7737 | 0.070 | 0.7194 | No |
| 102 | NDUFA9 | NADH:ubiquinone oxidoreductase subunit A9 [Source:HGNC Symbol;Acc:HGNC:7693] | 7750 | 0.070 | 0.7219 | No |
| 103 | ATP6V0D1 | ATPase H+ transporting V0 subunit d1 [Source:HGNC Symbol;Acc:HGNC:13724] | 8381 | 0.063 | 0.7084 | No |
| 104 | ATP6AP1 | ATPase H+ transporting accessory protein 1 [Source:HGNC Symbol;Acc:HGNC:868] | 8560 | 0.062 | 0.7064 | No |
| 105 | UQCRFS1 | "ubiquinol-cytochrome c reductase, Rieske iron-sulfur polypeptide 1 [Source:HGNC Symbol;Acc:HGNC:12587]" | 9310 | 0.055 | 0.6895 | No |
| 106 | MT-ATP8 | mitochondrially encoded ATP synthase membrane subunit 8 [Source:HGNC Symbol;Acc:HGNC:7415] | 9337 | 0.054 | 0.6910 | No |
| 107 | COX15 | cytochrome c oxidase assembly homolog COX15 [Source:HGNC Symbol;Acc:HGNC:2263] | 9577 | 0.052 | 0.6870 | No |
| 108 | ATP6V1G3 | ATPase H+ transporting V1 subunit G3 [Source:HGNC Symbol;Acc:HGNC:18265] | 9628 | 0.052 | 0.6878 | No |
| 109 | COX11 | cytochrome c oxidase copper chaperone COX11 [Source:HGNC Symbol;Acc:HGNC:2261] | 10280 | 0.047 | 0.6731 | No |
| 110 | MT-ND4L | mitochondrially encoded NADH:ubiquinone oxidoreductase core subunit 4L [Source:HGNC Symbol;Acc:HGNC:7460] | 11209 | 0.039 | 0.6510 | No |
| 111 | ATP6V1H | ATPase H+ transporting V1 subunit H [Source:HGNC Symbol;Acc:HGNC:18303] | 12118 | 0.032 | 0.6292 | No |
| 112 | ATP6V1B1 | ATPase H+ transporting V1 subunit B1 [Source:HGNC Symbol;Acc:HGNC:853] | 12217 | 0.032 | 0.6279 | No |
| 113 | NDUFA4L2 | NDUFA4 mitochondrial complex associated like 2 [Source:HGNC Symbol;Acc:HGNC:29836] | 15776 | 0.008 | 0.5375 | No |
| 114 | ATP6V0A1 | ATPase H+ transporting V0 subunit a1 [Source:HGNC Symbol;Acc:HGNC:865] | 16603 | 0.003 | 0.5166 | No |
| 115 | ATP6V1D | ATPase H+ transporting V1 subunit D [Source:HGNC Symbol;Acc:HGNC:13527] | 18057 | -0.005 | 0.4797 | No |
| 116 | COX8C | cytochrome c oxidase subunit 8C [Source:HGNC Symbol;Acc:HGNC:24382] | 20162 | -0.018 | 0.4267 | No |
| 117 | COX10 | cytochrome c oxidase assembly factor heme A:farnesyltransferase COX10 [Source:HGNC Symbol;Acc:HGNC:2260] | 20308 | -0.018 | 0.4238 | No |
| 118 | ATP5MC1P5 | ATP synthase membrane subunit c locus 1 pseudogene 5 [Source:HGNC Symbol;Acc:HGNC:39508] | 20955 | -0.022 | 0.4082 | No |
| 119 | COX6B2 | cytochrome c oxidase subunit 6B2 [Source:HGNC Symbol;Acc:HGNC:24380] | 21295 | -0.025 | 0.4005 | No |
| 120 | COX6CP3 | cytochrome c oxidase subunit 6C pseudogene 3 [Source:HGNC Symbol;Acc:HGNC:31721] | 21611 | -0.027 | 0.3936 | No |
| 121 | ATP12A | ATPase H+/K+ transporting non-gastric alpha2 subunit [Source:HGNC Symbol;Acc:HGNC:13816] | 21838 | -0.028 | 0.3889 | No |
| 122 | COX7B2 | cytochrome c oxidase subunit 7B2 [Source:HGNC Symbol;Acc:HGNC:24381] | 22662 | -0.033 | 0.3693 | No |
| 123 | ATP4A | ATPase H+/K+ transporting subunit alpha [Source:HGNC Symbol;Acc:HGNC:819] | 25319 | -0.051 | 0.3036 | No |
| 124 | ATP6V1G2 | ATPase H+ transporting V1 subunit G2 [Source:HGNC Symbol;Acc:HGNC:862] | 27144 | -0.064 | 0.2596 | No |
| 125 | ATP6V0A4 | ATPase H+ transporting V0 subunit a4 [Source:HGNC Symbol;Acc:HGNC:866] | 27407 | -0.066 | 0.2556 | No |
| 126 | ATP5F1B | ATP synthase F1 subunit beta [Source:HGNC Symbol;Acc:HGNC:830] | 28627 | -0.077 | 0.2276 | No |
| 127 | NDUFS1 | NADH:ubiquinone oxidoreductase core subunit S1 [Source:HGNC Symbol;Acc:HGNC:7707] | 30490 | -0.095 | 0.1840 | No |
| 128 | ATP6V1A | ATPase H+ transporting V1 subunit A [Source:HGNC Symbol;Acc:HGNC:851] | 33767 | -0.141 | 0.1061 | No |
| 129 | ATP6V1C1 | ATPase H+ transporting V1 subunit C1 [Source:HGNC Symbol;Acc:HGNC:856] | 34036 | -0.146 | 0.1052 | No |
| 130 | ATP6V0D2 | ATPase H+ transporting V0 subunit d2 [Source:HGNC Symbol;Acc:HGNC:18266] | 34205 | -0.149 | 0.1069 | No |
| 131 | ATP6V0A2 | ATPase H+ transporting V0 subunit a2 [Source:HGNC Symbol;Acc:HGNC:18481] | 37716 | -0.284 | 0.0289 | No |
| 132 | ATP6V1B2 | ATPase H+ transporting V1 subunit B2 [Source:HGNC Symbol;Acc:HGNC:854] | 37881 | -0.299 | 0.0368 | No |
Table: GSEA details [plain text format]

  

Fig 2: KEGG\_OXIDATIVE\_PHOSPHORYLATION      
 Blue-Pink O' Gram in the Space of the Analyzed GeneSet

  

Fig 3: KEGG\_OXIDATIVE\_PHOSPHORYLATION: Random ES distribution      
 Gene set null distribution of ES for **KEGG\_OXIDATIVE\_PHOSPHORYLATION**

  
