# Supplementary material for: Simultaneous Presentation of Multiple Myeloma and Lung Cancer: Case Report and Gene Bioinformatics Analysis
Source: Front Oncol. 2022 Jun 13;12:859735. doi: 10.3389/fonc.2022.859735 (PMC9235397; doi:10.3389/fonc.2022.859735)
Supplement: Supplementary file 1 [file DataSheet_1.zip › The bioinformatic analysis of MM and lung cancer supplementary materials/Enrichment analysis/MECR/GSEA_4.1.0/LUAD TCGA/KEGG.Gsea.1639041756227/KEGG_PARKINSONS_DISEASE.html]

Details for gene set KEGG\_PARKINSONS\_DISEASE[GSEA]

|  || Dataset | ExpData\_collapsed\_to\_symbols.ENSG00000116353\_profile\_in\_ExpData.cls #ENSG00000116353 |
| Phenotype | ENSG00000116353\_profile\_in\_ExpData.cls#ENSG00000116353 |
| Upregulated in class | ENSG00000116353\_pos |
| GeneSet | KEGG\_PARKINSONS\_DISEASE |
| Enrichment Score (ES) | 0.7183023 |
| Normalized Enrichment Score (NES) | 3.0875423 |
| Nominal p-value | 0.0 |
| FDR q-value | 0.0 |
| FWER p-Value | 0.0 |
Table: GSEA Results Summary

  

Fig 1: Enrichment plot: KEGG\_PARKINSONS\_DISEASE      
 Profile of the Running ES Score & Positions of GeneSet Members on the Rank Ordered List

  

| SYMBOL | TITLE | RANK IN GENE LIST | RANK METRIC SCORE | RUNNING ES | CORE ENRICHMENT || 1 | NDUFS8 | NADH:ubiquinone oxidoreductase core subunit S8 [Source:HGNC Symbol;Acc:HGNC:7715] | 34 | 0.446 | 0.0176 | Yes |
| 2 | PARK7 | Parkinsonism associated deglycase [Source:HGNC Symbol;Acc:HGNC:16369] | 41 | 0.438 | 0.0357 | Yes |
| 3 | NDUFS5 | NADH:ubiquinone oxidoreductase subunit S5 [Source:HGNC Symbol;Acc:HGNC:7712] | 78 | 0.411 | 0.0518 | Yes |
| 4 | UBE2J2 | ubiquitin conjugating enzyme E2 J2 [Source:HGNC Symbol;Acc:HGNC:19268] | 101 | 0.403 | 0.0680 | Yes |
| 5 | NDUFA2 | NADH:ubiquinone oxidoreductase subunit A2 [Source:HGNC Symbol;Acc:HGNC:7685] | 110 | 0.398 | 0.0843 | Yes |
| 6 | NDUFB10 | NADH:ubiquinone oxidoreductase subunit B10 [Source:HGNC Symbol;Acc:HGNC:7696] | 142 | 0.385 | 0.0996 | Yes |
| 7 | ATP5PO | ATP synthase peripheral stalk subunit OSCP [Source:HGNC Symbol;Acc:HGNC:850] | 231 | 0.366 | 0.1125 | Yes |
| 8 | NDUFB7 | NADH:ubiquinone oxidoreductase subunit B7 [Source:HGNC Symbol;Acc:HGNC:7702] | 278 | 0.355 | 0.1261 | Yes |
| 9 | NDUFV1 | NADH:ubiquinone oxidoreductase core subunit V1 [Source:HGNC Symbol;Acc:HGNC:7716] | 308 | 0.349 | 0.1398 | Yes |
| 10 | NDUFV3 | NADH:ubiquinone oxidoreductase subunit V3 [Source:HGNC Symbol;Acc:HGNC:7719] | 321 | 0.346 | 0.1539 | Yes |
| 11 | ATP5PD | ATP synthase peripheral stalk subunit d [Source:HGNC Symbol;Acc:HGNC:845] | 341 | 0.343 | 0.1677 | Yes |
| 12 | NDUFB2 | NADH:ubiquinone oxidoreductase subunit B2 [Source:HGNC Symbol;Acc:HGNC:7697] | 440 | 0.327 | 0.1788 | Yes |
| 13 | SDHB | succinate dehydrogenase complex iron sulfur subunit B [Source:HGNC Symbol;Acc:HGNC:10681] | 443 | 0.327 | 0.1923 | Yes |
| 14 | NDUFA6 | NADH:ubiquinone oxidoreductase subunit A6 [Source:HGNC Symbol;Acc:HGNC:7690] | 471 | 0.324 | 0.2050 | Yes |
| 15 | ATP5F1D | ATP synthase F1 subunit delta [Source:HGNC Symbol;Acc:HGNC:837] | 480 | 0.321 | 0.2182 | Yes |
| 16 | NDUFS7 | NADH:ubiquinone oxidoreductase core subunit S7 [Source:HGNC Symbol;Acc:HGNC:7714] | 511 | 0.317 | 0.2306 | Yes |
| 17 | UQCR10 | "ubiquinol-cytochrome c reductase, complex III subunit X [Source:HGNC Symbol;Acc:HGNC:30863]" | 623 | 0.305 | 0.2405 | Yes |
| 18 | HTRA2 | HtrA serine peptidase 2 [Source:HGNC Symbol;Acc:HGNC:14348] | 645 | 0.304 | 0.2526 | Yes |
| 19 | NDUFC1 | NADH:ubiquinone oxidoreductase subunit C1 [Source:HGNC Symbol;Acc:HGNC:7705] | 648 | 0.303 | 0.2651 | Yes |
| 20 | UQCRC1 | ubiquinol-cytochrome c reductase core protein 1 [Source:HGNC Symbol;Acc:HGNC:12585] | 653 | 0.302 | 0.2776 | Yes |
| 21 | NDUFS6 | NADH:ubiquinone oxidoreductase subunit S6 [Source:HGNC Symbol;Acc:HGNC:7713] | 687 | 0.298 | 0.2891 | Yes |
| 22 | ATP5PF | ATP synthase peripheral stalk subunit F6 [Source:HGNC Symbol;Acc:HGNC:847] | 702 | 0.297 | 0.3011 | Yes |
| 23 | CYC1 | cytochrome c1 [Source:HGNC Symbol;Acc:HGNC:2579] | 712 | 0.296 | 0.3131 | Yes |
| 24 | NDUFA4 | NDUFA4 mitochondrial complex associated [Source:HGNC Symbol;Acc:HGNC:7687] | 738 | 0.293 | 0.3247 | Yes |
| 25 | NDUFB8 | NADH:ubiquinone oxidoreductase subunit B8 [Source:HGNC Symbol;Acc:HGNC:7703] | 748 | 0.293 | 0.3366 | Yes |
| 26 | ATP5MC1 | ATP synthase membrane subunit c locus 1 [Source:HGNC Symbol;Acc:HGNC:841] | 755 | 0.293 | 0.3486 | Yes |
| 27 | UQCR11 | "ubiquinol-cytochrome c reductase, complex III subunit XI [Source:HGNC Symbol;Acc:HGNC:30862]" | 764 | 0.292 | 0.3605 | Yes |
| 28 | UQCRQ | ubiquinol-cytochrome c reductase complex III subunit VII [Source:HGNC Symbol;Acc:HGNC:29594] | 818 | 0.286 | 0.3711 | Yes |
| 29 | NDUFS3 | NADH:ubiquinone oxidoreductase core subunit S3 [Source:HGNC Symbol;Acc:HGNC:7710] | 842 | 0.284 | 0.3823 | Yes |
| 30 | NDUFA3 | NADH:ubiquinone oxidoreductase subunit A3 [Source:HGNC Symbol;Acc:HGNC:7686] | 864 | 0.282 | 0.3935 | Yes |
| 31 | NDUFA7 | NADH:ubiquinone oxidoreductase subunit A7 [Source:HGNC Symbol;Acc:HGNC:7691] | 878 | 0.281 | 0.4048 | Yes |
| 32 | NDUFB9 | NADH:ubiquinone oxidoreductase subunit B9 [Source:HGNC Symbol;Acc:HGNC:7704] | 915 | 0.278 | 0.4154 | Yes |
| 33 | UQCRH | ubiquinol-cytochrome c reductase hinge protein [Source:HGNC Symbol;Acc:HGNC:12590] | 937 | 0.275 | 0.4263 | Yes |
| 34 | COX5B | cytochrome c oxidase subunit 5B [Source:HGNC Symbol;Acc:HGNC:2269] | 1019 | 0.268 | 0.4354 | Yes |
| 35 | COX4I1 | cytochrome c oxidase subunit 4I1 [Source:HGNC Symbol;Acc:HGNC:2265] | 1051 | 0.266 | 0.4457 | Yes |
| 36 | NDUFB1 | NADH:ubiquinone oxidoreductase subunit B1 [Source:HGNC Symbol;Acc:HGNC:7695] | 1087 | 0.263 | 0.4557 | Yes |
| 37 | ATP5MC2 | ATP synthase membrane subunit c locus 2 [Source:HGNC Symbol;Acc:HGNC:842] | 1094 | 0.263 | 0.4665 | Yes |
| 38 | COX8A | cytochrome c oxidase subunit 8A [Source:HGNC Symbol;Acc:HGNC:2294] | 1130 | 0.261 | 0.4764 | Yes |
| 39 | NDUFA8 | NADH:ubiquinone oxidoreductase subunit A8 [Source:HGNC Symbol;Acc:HGNC:7692] | 1138 | 0.261 | 0.4871 | Yes |
| 40 | NDUFB4 | NADH:ubiquinone oxidoreductase subunit B4 [Source:HGNC Symbol;Acc:HGNC:7699] | 1212 | 0.254 | 0.4958 | Yes |
| 41 | COX7C | cytochrome c oxidase subunit 7C [Source:HGNC Symbol;Acc:HGNC:2292] | 1244 | 0.252 | 0.5055 | Yes |
| 42 | UQCRHL | ubiquinol-cytochrome c reductase hinge protein like [Source:HGNC Symbol;Acc:HGNC:51714] | 1439 | 0.239 | 0.5105 | Yes |
| 43 | ATP5F1E | ATP synthase F1 subunit epsilon [Source:HGNC Symbol;Acc:HGNC:838] | 1468 | 0.237 | 0.5196 | Yes |
| 44 | PINK1 | PTEN induced kinase 1 [Source:HGNC Symbol;Acc:HGNC:14581] | 1576 | 0.230 | 0.5264 | Yes |
| 45 | NDUFAB1 | NADH:ubiquinone oxidoreductase subunit AB1 [Source:HGNC Symbol;Acc:HGNC:7694] | 1579 | 0.230 | 0.5359 | Yes |
| 46 | COX6B1 | cytochrome c oxidase subunit 6B1 [Source:HGNC Symbol;Acc:HGNC:2280] | 1782 | 0.218 | 0.5398 | Yes |
| 47 | COX6A1 | cytochrome c oxidase subunit 6A1 [Source:HGNC Symbol;Acc:HGNC:2277] | 1827 | 0.216 | 0.5477 | Yes |
| 48 | SDHC | succinate dehydrogenase complex subunit C [Source:HGNC Symbol;Acc:HGNC:10682] | 2041 | 0.204 | 0.5507 | Yes |
| 49 | NDUFC2 | NADH:ubiquinone oxidoreductase subunit C2 [Source:HGNC Symbol;Acc:HGNC:7706] | 2151 | 0.199 | 0.5562 | Yes |
| 50 | NDUFA1 | NADH:ubiquinone oxidoreductase subunit A1 [Source:HGNC Symbol;Acc:HGNC:7683] | 2163 | 0.198 | 0.5641 | Yes |
| 51 | ATP5F1C | ATP synthase F1 subunit gamma [Source:HGNC Symbol;Acc:HGNC:833] | 2375 | 0.190 | 0.5667 | Yes |
| 52 | NDUFV2 | NADH:ubiquinone oxidoreductase core subunit V2 [Source:HGNC Symbol;Acc:HGNC:7717] | 2413 | 0.188 | 0.5735 | Yes |
| 53 | NDUFS2 | NADH:ubiquinone oxidoreductase core subunit S2 [Source:HGNC Symbol;Acc:HGNC:7708] | 2516 | 0.184 | 0.5786 | Yes |
| 54 | SLC25A4 | solute carrier family 25 member 4 [Source:HGNC Symbol;Acc:HGNC:10990] | 2531 | 0.183 | 0.5858 | Yes |
| 55 | COX7B | cytochrome c oxidase subunit 7B [Source:HGNC Symbol;Acc:HGNC:2291] | 2582 | 0.181 | 0.5921 | Yes |
| 56 | MT-CO2 | mitochondrially encoded cytochrome c oxidase II [Source:HGNC Symbol;Acc:HGNC:7421] | 2703 | 0.176 | 0.5963 | Yes |
| 57 | COX7A2L | cytochrome c oxidase subunit 7A2 like [Source:HGNC Symbol;Acc:HGNC:2289] | 2825 | 0.171 | 0.6003 | Yes |
| 58 | UQCRB | ubiquinol-cytochrome c reductase binding protein [Source:HGNC Symbol;Acc:HGNC:12582] | 2840 | 0.170 | 0.6070 | Yes |
| 59 | COX6C | cytochrome c oxidase subunit 6C [Source:HGNC Symbol;Acc:HGNC:2285] | 2863 | 0.169 | 0.6135 | Yes |
| 60 | MT-ND1 | mitochondrially encoded NADH:ubiquinone oxidoreductase core subunit 1 [Source:HGNC Symbol;Acc:HGNC:7455] | 2906 | 0.168 | 0.6193 | Yes |
| 61 | MT-CO3 | mitochondrially encoded cytochrome c oxidase III [Source:HGNC Symbol;Acc:HGNC:7422] | 2913 | 0.167 | 0.6261 | Yes |
| 62 | MT-ND3 | mitochondrially encoded NADH:ubiquinone oxidoreductase core subunit 3 [Source:HGNC Symbol;Acc:HGNC:7458] | 2921 | 0.167 | 0.6329 | Yes |
| 63 | NDUFA5 | NADH:ubiquinone oxidoreductase subunit A5 [Source:HGNC Symbol;Acc:HGNC:7688] | 3044 | 0.163 | 0.6366 | Yes |
| 64 | SLC25A6 | solute carrier family 25 member 6 [Source:HGNC Symbol;Acc:HGNC:10992] | 3062 | 0.162 | 0.6429 | Yes |
| 65 | NDUFS4 | NADH:ubiquinone oxidoreductase subunit S4 [Source:HGNC Symbol;Acc:HGNC:7711] | 3070 | 0.162 | 0.6494 | Yes |
| 66 | NDUFB5 | NADH:ubiquinone oxidoreductase subunit B5 [Source:HGNC Symbol;Acc:HGNC:7700] | 3101 | 0.161 | 0.6553 | Yes |
| 67 | UBE2G2 | ubiquitin conjugating enzyme E2 G2 [Source:HGNC Symbol;Acc:HGNC:12483] | 3402 | 0.151 | 0.6539 | Yes |
| 68 | CASP9 | caspase 9 [Source:HGNC Symbol;Acc:HGNC:1511] | 3419 | 0.151 | 0.6598 | Yes |
| 69 | NDUFB3 | NADH:ubiquinone oxidoreductase subunit B3 [Source:HGNC Symbol;Acc:HGNC:7698] | 3463 | 0.150 | 0.6649 | Yes |
| 70 | MT-ND2 | mitochondrially encoded NADH:ubiquinone oxidoreductase core subunit 2 [Source:HGNC Symbol;Acc:HGNC:7456] | 3586 | 0.146 | 0.6678 | Yes |
| 71 | COX5A | cytochrome c oxidase subunit 5A [Source:HGNC Symbol;Acc:HGNC:2267] | 3688 | 0.142 | 0.6712 | Yes |
| 72 | UBE2L3 | ubiquitin conjugating enzyme E2 L3 [Source:HGNC Symbol;Acc:HGNC:12488] | 3710 | 0.142 | 0.6765 | Yes |
| 73 | COX7A2 | cytochrome c oxidase subunit 7A2 [Source:HGNC Symbol;Acc:HGNC:2288] | 3752 | 0.141 | 0.6813 | Yes |
| 74 | MT-CO1 | mitochondrially encoded cytochrome c oxidase I [Source:HGNC Symbol;Acc:HGNC:7419] | 3805 | 0.139 | 0.6858 | Yes |
| 75 | MT-ND6 | mitochondrially encoded NADH:ubiquinone oxidoreductase core subunit 6 [Source:HGNC Symbol;Acc:HGNC:7462] | 3841 | 0.139 | 0.6907 | Yes |
| 76 | NDUFB6 | NADH:ubiquinone oxidoreductase subunit B6 [Source:HGNC Symbol;Acc:HGNC:7701] | 3968 | 0.134 | 0.6930 | Yes |
| 77 | SDHA | succinate dehydrogenase complex flavoprotein subunit A [Source:HGNC Symbol;Acc:HGNC:10680] | 4074 | 0.132 | 0.6958 | Yes |
| 78 | ATP5PB | ATP synthase peripheral stalk-membrane subunit b [Source:HGNC Symbol;Acc:HGNC:840] | 4227 | 0.128 | 0.6973 | Yes |
| 79 | NDUFA10 | NADH:ubiquinone oxidoreductase subunit A10 [Source:HGNC Symbol;Acc:HGNC:7684] | 4232 | 0.128 | 0.7025 | Yes |
| 80 | MT-ND4 | mitochondrially encoded NADH:ubiquinone oxidoreductase core subunit 4 [Source:HGNC Symbol;Acc:HGNC:7459] | 4312 | 0.126 | 0.7057 | Yes |
| 81 | MT-CYB | mitochondrially encoded cytochrome b [Source:HGNC Symbol;Acc:HGNC:7427] | 4594 | 0.119 | 0.7035 | Yes |
| 82 | ATP5MC3 | ATP synthase membrane subunit c locus 3 [Source:HGNC Symbol;Acc:HGNC:843] | 4619 | 0.119 | 0.7079 | Yes |
| 83 | MT-ATP6 | mitochondrially encoded ATP synthase membrane subunit 6 [Source:HGNC Symbol;Acc:HGNC:7414] | 4718 | 0.116 | 0.7102 | Yes |
| 84 | SLC25A5 | solute carrier family 25 member 5 [Source:HGNC Symbol;Acc:HGNC:10991] | 4760 | 0.116 | 0.7140 | Yes |
| 85 | COX7A1 | cytochrome c oxidase subunit 7A1 [Source:HGNC Symbol;Acc:HGNC:2287] | 4947 | 0.112 | 0.7139 | Yes |
| 86 | COX4I2 | cytochrome c oxidase subunit 4I2 [Source:HGNC Symbol;Acc:HGNC:16232] | 5116 | 0.109 | 0.7141 | Yes |
| 87 | UQCR10P1 | UQCR10 pseudogene 1 [Source:HGNC Symbol;Acc:HGNC:54960] | 5130 | 0.109 | 0.7183 | Yes |
| 88 | COX6A2 | cytochrome c oxidase subunit 6A2 [Source:HGNC Symbol;Acc:HGNC:2279] | 5599 | 0.100 | 0.7105 | No |
| 89 | ATP5F1A | ATP synthase F1 subunit alpha [Source:HGNC Symbol;Acc:HGNC:823] | 5690 | 0.099 | 0.7123 | No |
| 90 | SDHD | succinate dehydrogenase complex subunit D [Source:HGNC Symbol;Acc:HGNC:10683] | 5864 | 0.096 | 0.7119 | No |
| 91 | MT-ND5 | mitochondrially encoded NADH:ubiquinone oxidoreductase core subunit 5 [Source:HGNC Symbol;Acc:HGNC:7461] | 6253 | 0.089 | 0.7057 | No |
| 92 | UBB | ubiquitin B [Source:HGNC Symbol;Acc:HGNC:12463] | 6965 | 0.080 | 0.6909 | No |
| 93 | UQCRC2 | ubiquinol-cytochrome c reductase core protein 2 [Source:HGNC Symbol;Acc:HGNC:12586] | 7737 | 0.070 | 0.6741 | No |
| 94 | NDUFA9 | NADH:ubiquinone oxidoreductase subunit A9 [Source:HGNC Symbol;Acc:HGNC:7693] | 7750 | 0.070 | 0.6767 | No |
| 95 | CYCS | "cytochrome c, somatic [Source:HGNC Symbol;Acc:HGNC:19986]" | 8363 | 0.064 | 0.6638 | No |
| 96 | UQCRFS1 | "ubiquinol-cytochrome c reductase, Rieske iron-sulfur polypeptide 1 [Source:HGNC Symbol;Acc:HGNC:12587]" | 9310 | 0.055 | 0.6419 | No |
| 97 | MT-ATP8 | mitochondrially encoded ATP synthase membrane subunit 8 [Source:HGNC Symbol;Acc:HGNC:7415] | 9337 | 0.054 | 0.6435 | No |
| 98 | TH | tyrosine hydroxylase [Source:HGNC Symbol;Acc:HGNC:11782] | 9464 | 0.053 | 0.6425 | No |
| 99 | VDAC2 | voltage dependent anion channel 2 [Source:HGNC Symbol;Acc:HGNC:12672] | 10201 | 0.047 | 0.6257 | No |
| 100 | SLC25A31 | solute carrier family 25 member 31 [Source:HGNC Symbol;Acc:HGNC:25319] | 10585 | 0.044 | 0.6177 | No |
| 101 | MT-ND4L | mitochondrially encoded NADH:ubiquinone oxidoreductase core subunit 4L [Source:HGNC Symbol;Acc:HGNC:7460] | 11209 | 0.039 | 0.6035 | No |
| 102 | UCHL1 | ubiquitin C-terminal hydrolase L1 [Source:HGNC Symbol;Acc:HGNC:12513] | 11262 | 0.039 | 0.6038 | No |
| 103 | VDAC2P5 | VDAC2 pseudogene 5 [Source:HGNC Symbol;Acc:HGNC:54753] | 11481 | 0.037 | 0.5997 | No |
| 104 | UBA7 | ubiquitin like modifier activating enzyme 7 [Source:HGNC Symbol;Acc:HGNC:12471] | 12039 | 0.033 | 0.5869 | No |
| 105 | SEPTIN5 | septin 5 [Source:HGNC Symbol;Acc:HGNC:9164] | 15158 | 0.012 | 0.5078 | No |
| 106 | NDUFA4L2 | NDUFA4 mitochondrial complex associated like 2 [Source:HGNC Symbol;Acc:HGNC:29836] | 15776 | 0.008 | 0.4925 | No |
| 107 | GPR37 | G protein-coupled receptor 37 [Source:HGNC Symbol;Acc:HGNC:4494] | 17220 | -0.000 | 0.4556 | No |
| 108 | UBA1 | ubiquitin like modifier activating enzyme 1 [Source:HGNC Symbol;Acc:HGNC:12469] | 19005 | -0.011 | 0.4106 | No |
| 109 | VDAC3 | voltage dependent anion channel 3 [Source:HGNC Symbol;Acc:HGNC:12674] | 19385 | -0.013 | 0.4014 | No |
| 110 | COX8C | cytochrome c oxidase subunit 8C [Source:HGNC Symbol;Acc:HGNC:24382] | 20162 | -0.018 | 0.3824 | No |
| 111 | ATP5MC1P5 | ATP synthase membrane subunit c locus 1 pseudogene 5 [Source:HGNC Symbol;Acc:HGNC:39508] | 20955 | -0.022 | 0.3631 | No |
| 112 | COX6B2 | cytochrome c oxidase subunit 6B2 [Source:HGNC Symbol;Acc:HGNC:24380] | 21295 | -0.025 | 0.3555 | No |
| 113 | COX6CP3 | cytochrome c oxidase subunit 6C pseudogene 3 [Source:HGNC Symbol;Acc:HGNC:31721] | 21611 | -0.027 | 0.3485 | No |
| 114 | COX7B2 | cytochrome c oxidase subunit 7B2 [Source:HGNC Symbol;Acc:HGNC:24381] | 22662 | -0.033 | 0.3231 | No |
| 115 | UBE2L6 | ubiquitin conjugating enzyme E2 L6 [Source:HGNC Symbol;Acc:HGNC:12490] | 25893 | -0.055 | 0.2430 | No |
| 116 | ATP5F1B | ATP synthase F1 subunit beta [Source:HGNC Symbol;Acc:HGNC:830] | 28627 | -0.077 | 0.1765 | No |
| 117 | VDAC1 | voltage dependent anion channel 1 [Source:HGNC Symbol;Acc:HGNC:12669] | 29848 | -0.088 | 0.1490 | No |
| 118 | SLC6A3 | solute carrier family 6 member 3 [Source:HGNC Symbol;Acc:HGNC:11049] | 30045 | -0.090 | 0.1477 | No |
| 119 | NDUFS1 | NADH:ubiquinone oxidoreductase core subunit S1 [Source:HGNC Symbol;Acc:HGNC:7707] | 30490 | -0.095 | 0.1403 | No |
| 120 | LRRK2 | leucine rich repeat kinase 2 [Source:HGNC Symbol;Acc:HGNC:18618] | 30704 | -0.097 | 0.1390 | No |
| 121 | PPID | peptidylprolyl isomerase D [Source:HGNC Symbol;Acc:HGNC:9257] | 30753 | -0.098 | 0.1418 | No |
| 122 | CASP3 | caspase 3 [Source:HGNC Symbol;Acc:HGNC:1504] | 31110 | -0.102 | 0.1369 | No |
| 123 | PRKN | parkin RBR E3 ubiquitin protein ligase [Source:HGNC Symbol;Acc:HGNC:8607] | 31449 | -0.106 | 0.1327 | No |
| 124 | SLC18A1 | solute carrier family 18 member A1 [Source:HGNC Symbol;Acc:HGNC:10934] | 33366 | -0.134 | 0.0894 | No |
| 125 | SNCAIP | synuclein alpha interacting protein [Source:HGNC Symbol;Acc:HGNC:11139] | 34051 | -0.146 | 0.0780 | No |
| 126 | SNCA | synuclein alpha [Source:HGNC Symbol;Acc:HGNC:11138] | 36532 | -0.212 | 0.0236 | No |
| 127 | UBE2G1 | ubiquitin conjugating enzyme E2 G1 [Source:HGNC Symbol;Acc:HGNC:12482] | 36594 | -0.215 | 0.0309 | No |
| 128 | SLC18A2 | solute carrier family 18 member A2 [Source:HGNC Symbol;Acc:HGNC:10935] | 36647 | -0.218 | 0.0387 | No |
| 129 | UBE2J1 | ubiquitin conjugating enzyme E2 J1 [Source:HGNC Symbol;Acc:HGNC:17598] | 38064 | -0.326 | 0.0161 | No |
| 130 | APAF1 | apoptotic peptidase activating factor 1 [Source:HGNC Symbol;Acc:HGNC:576] | 38254 | -0.386 | 0.0273 | No |
Table: GSEA details [plain text format]

  

Fig 2: KEGG\_PARKINSONS\_DISEASE      
 Blue-Pink O' Gram in the Space of the Analyzed GeneSet

  

Fig 3: KEGG\_PARKINSONS\_DISEASE: Random ES distribution      
 Gene set null distribution of ES for **KEGG\_PARKINSONS\_DISEASE**

  
