# Supplementary material for: Simultaneous Presentation of Multiple Myeloma and Lung Cancer: Case Report and Gene Bioinformatics Analysis
Source: Front Oncol. 2022 Jun 13;12:859735. doi: 10.3389/fonc.2022.859735 (PMC9235397; doi:10.3389/fonc.2022.859735)
Supplement: Supplementary file 1 [file DataSheet_1.zip › The bioinformatic analysis of MM and lung cancer supplementary materials/Enrichment analysis/MECR/GSEA_4.1.0/LUAD TCGA/KEGG.Gsea.1639041756227/KEGG_PEROXISOME.html]

Details for gene set KEGG\_PEROXISOME[GSEA]

|  || Dataset | ExpData\_collapsed\_to\_symbols.ENSG00000116353\_profile\_in\_ExpData.cls #ENSG00000116353 |
| Phenotype | ENSG00000116353\_profile\_in\_ExpData.cls#ENSG00000116353 |
| Upregulated in class | ENSG00000116353\_pos |
| GeneSet | KEGG\_PEROXISOME |
| Enrichment Score (ES) | 0.55537504 |
| Normalized Enrichment Score (NES) | 2.2024796 |
| Nominal p-value | 0.0 |
| FDR q-value | 0.0 |
| FWER p-Value | 0.0 |
Table: GSEA Results Summary

  

Fig 1: Enrichment plot: KEGG\_PEROXISOME      
 Profile of the Running ES Score & Positions of GeneSet Members on the Rank Ordered List

  

| SYMBOL | TITLE | RANK IN GENE LIST | RANK METRIC SCORE | RUNNING ES | CORE ENRICHMENT || 1 | HMGCL | 3-hydroxy-3-methylglutaryl-CoA lyase [Source:HGNC Symbol;Acc:HGNC:5005] | 8 | 0.505 | 0.0381 | Yes |
| 2 | PEX14 | peroxisomal biogenesis factor 14 [Source:HGNC Symbol;Acc:HGNC:8856] | 11 | 0.494 | 0.0754 | Yes |
| 3 | PMVK | phosphomevalonate kinase [Source:HGNC Symbol;Acc:HGNC:9141] | 20 | 0.466 | 0.1106 | Yes |
| 4 | PRDX5 | peroxiredoxin 5 [Source:HGNC Symbol;Acc:HGNC:9355] | 63 | 0.423 | 0.1416 | Yes |
| 5 | PEX10 | peroxisomal biogenesis factor 10 [Source:HGNC Symbol;Acc:HGNC:8851] | 75 | 0.412 | 0.1725 | Yes |
| 6 | PEX11G | peroxisomal biogenesis factor 11 gamma [Source:HGNC Symbol;Acc:HGNC:20208] | 128 | 0.390 | 0.2007 | Yes |
| 7 | DECR2 | "2,4-dienoyl-CoA reductase 2 [Source:HGNC Symbol;Acc:HGNC:2754]" | 168 | 0.381 | 0.2286 | Yes |
| 8 | MPV17 | mitochondrial inner membrane protein MPV17 [Source:HGNC Symbol;Acc:HGNC:7224] | 180 | 0.378 | 0.2570 | Yes |
| 9 | PEX16 | peroxisomal biogenesis factor 16 [Source:HGNC Symbol;Acc:HGNC:8857] | 514 | 0.317 | 0.2726 | Yes |
| 10 | GSTK1 | glutathione S-transferase kappa 1 [Source:HGNC Symbol;Acc:HGNC:16906] | 629 | 0.305 | 0.2928 | Yes |
| 11 | SOD1 | superoxide dismutase 1 [Source:HGNC Symbol;Acc:HGNC:11179] | 641 | 0.304 | 0.3155 | Yes |
| 12 | ACOT8 | acyl-CoA thioesterase 8 [Source:HGNC Symbol;Acc:HGNC:15919] | 722 | 0.295 | 0.3359 | Yes |
| 13 | ECH1 | enoyl-CoA hydratase 1 [Source:HGNC Symbol;Acc:HGNC:3149] | 867 | 0.282 | 0.3536 | Yes |
| 14 | SLC25A17 | solute carrier family 25 member 17 [Source:HGNC Symbol;Acc:HGNC:10987] | 921 | 0.277 | 0.3732 | Yes |
| 15 | PEX11B | peroxisomal biogenesis factor 11 beta [Source:HGNC Symbol;Acc:HGNC:8853] | 974 | 0.271 | 0.3925 | Yes |
| 16 | ACAA1 | acetyl-CoA acyltransferase 1 [Source:HGNC Symbol;Acc:HGNC:82] | 1003 | 0.270 | 0.4122 | Yes |
| 17 | MVK | mevalonate kinase [Source:HGNC Symbol;Acc:HGNC:7530] | 1155 | 0.259 | 0.4280 | Yes |
| 18 | PEX11A | peroxisomal biogenesis factor 11 alpha [Source:HGNC Symbol;Acc:HGNC:8852] | 1217 | 0.254 | 0.4457 | Yes |
| 19 | PEX19 | peroxisomal biogenesis factor 19 [Source:HGNC Symbol;Acc:HGNC:9713] | 1250 | 0.251 | 0.4639 | Yes |
| 20 | CRAT | carnitine O-acetyltransferase [Source:HGNC Symbol;Acc:HGNC:2342] | 1322 | 0.246 | 0.4808 | Yes |
| 21 | PRDX1 | peroxiredoxin 1 [Source:HGNC Symbol;Acc:HGNC:9352] | 1404 | 0.241 | 0.4970 | Yes |
| 22 | DHRS4 | dehydrogenase/reductase 4 [Source:HGNC Symbol;Acc:HGNC:16985] | 1515 | 0.234 | 0.5119 | Yes |
| 23 | PEX6 | peroxisomal biogenesis factor 6 [Source:HGNC Symbol;Acc:HGNC:8859] | 1672 | 0.225 | 0.5250 | Yes |
| 24 | ECI2 | enoyl-CoA delta isomerase 2 [Source:HGNC Symbol;Acc:HGNC:14601] | 2081 | 0.202 | 0.5299 | Yes |
| 25 | SCP2 | sterol carrier protein 2 [Source:HGNC Symbol;Acc:HGNC:10606] | 2368 | 0.190 | 0.5370 | Yes |
| 26 | PHYH | phytanoyl-CoA 2-hydroxylase [Source:HGNC Symbol;Acc:HGNC:8940] | 2630 | 0.179 | 0.5439 | Yes |
| 27 | ACOX3 | "acyl-CoA oxidase 3, pristanoyl [Source:HGNC Symbol;Acc:HGNC:121]" | 2763 | 0.173 | 0.5537 | Yes |
| 28 | PEX7 | peroxisomal biogenesis factor 7 [Source:HGNC Symbol;Acc:HGNC:8860] | 3169 | 0.159 | 0.5554 | Yes |
| 29 | PEX13 | peroxisomal biogenesis factor 13 [Source:HGNC Symbol;Acc:HGNC:8855] | 4998 | 0.111 | 0.5172 | No |
| 30 | HAO1 | hydroxyacid oxidase 1 [Source:HGNC Symbol;Acc:HGNC:4809] | 5062 | 0.110 | 0.5240 | No |
| 31 | NUDT12 | nudix hydrolase 12 [Source:HGNC Symbol;Acc:HGNC:18826] | 5606 | 0.100 | 0.5177 | No |
| 32 | IDH2 | isocitrate dehydrogenase (NADP(+)) 2 [Source:HGNC Symbol;Acc:HGNC:5383] | 5934 | 0.095 | 0.5166 | No |
| 33 | EPHX2 | epoxide hydrolase 2 [Source:HGNC Symbol;Acc:HGNC:3402] | 6219 | 0.090 | 0.5162 | No |
| 34 | GNPAT | glyceronephosphate O-acyltransferase [Source:HGNC Symbol;Acc:HGNC:4416] | 6435 | 0.087 | 0.5173 | No |
| 35 | PXMP2 | peroxisomal membrane protein 2 [Source:HGNC Symbol;Acc:HGNC:9716] | 6485 | 0.086 | 0.5225 | No |
| 36 | AMACR | alpha-methylacyl-CoA racemase [Source:HGNC Symbol;Acc:HGNC:451] | 6873 | 0.081 | 0.5188 | No |
| 37 | NUDT19 | nudix hydrolase 19 [Source:HGNC Symbol;Acc:HGNC:32036] | 7025 | 0.079 | 0.5210 | No |
| 38 | ABCD1 | ATP binding cassette subfamily D member 1 [Source:HGNC Symbol;Acc:HGNC:61] | 7082 | 0.078 | 0.5255 | No |
| 39 | ABCD4 | ATP binding cassette subfamily D member 4 [Source:HGNC Symbol;Acc:HGNC:68] | 7235 | 0.076 | 0.5274 | No |
| 40 | IDH1 | isocitrate dehydrogenase (NADP(+)) 1 [Source:HGNC Symbol;Acc:HGNC:5382] | 7478 | 0.073 | 0.5268 | No |
| 41 | ACOX2 | acyl-CoA oxidase 2 [Source:HGNC Symbol;Acc:HGNC:120] | 7993 | 0.067 | 0.5188 | No |
| 42 | BAAT | bile acid-CoA:amino acid N-acyltransferase [Source:HGNC Symbol;Acc:HGNC:932] | 8903 | 0.058 | 0.5001 | No |
| 43 | PEX2 | peroxisomal biogenesis factor 2 [Source:HGNC Symbol;Acc:HGNC:9717] | 9239 | 0.055 | 0.4957 | No |
| 44 | PAOX | polyamine oxidase [Source:HGNC Symbol;Acc:HGNC:20837] | 9246 | 0.055 | 0.4997 | No |
| 45 | PXMP4 | peroxisomal membrane protein 4 [Source:HGNC Symbol;Acc:HGNC:15920] | 9738 | 0.051 | 0.4911 | No |
| 46 | ACSL5 | acyl-CoA synthetase long chain family member 5 [Source:HGNC Symbol;Acc:HGNC:16526] | 10039 | 0.048 | 0.4871 | No |
| 47 | PEX12 | peroxisomal biogenesis factor 12 [Source:HGNC Symbol;Acc:HGNC:8854] | 10111 | 0.048 | 0.4889 | No |
| 48 | CROT | carnitine O-octanoyltransferase [Source:HGNC Symbol;Acc:HGNC:2366] | 10799 | 0.043 | 0.4746 | No |
| 49 | MPV17L | MPV17 mitochondrial inner membrane protein like [Source:HGNC Symbol;Acc:HGNC:26827] | 11573 | 0.036 | 0.4577 | No |
| 50 | HAO2 | hydroxyacid oxidase 2 [Source:HGNC Symbol;Acc:HGNC:4810] | 13721 | 0.021 | 0.4046 | No |
| 51 | PEX3 | peroxisomal biogenesis factor 3 [Source:HGNC Symbol;Acc:HGNC:8858] | 15119 | 0.012 | 0.3699 | No |
| 52 | AGXT | alanine--glyoxylate and serine--pyruvate aminotransferase [Source:HGNC Symbol;Acc:HGNC:341] | 17143 | 0.000 | 0.3184 | No |
| 53 | ABCD3 | ATP binding cassette subfamily D member 3 [Source:HGNC Symbol;Acc:HGNC:67] | 17644 | -0.003 | 0.3059 | No |
| 54 | XDH | xanthine dehydrogenase [Source:HGNC Symbol;Acc:HGNC:12805] | 19063 | -0.011 | 0.2706 | No |
| 55 | EHHADH | enoyl-CoA hydratase and 3-hydroxyacyl CoA dehydrogenase [Source:HGNC Symbol;Acc:HGNC:3247] | 23047 | -0.035 | 0.1718 | No |
| 56 | MLYCD | malonyl-CoA decarboxylase [Source:HGNC Symbol;Acc:HGNC:7150] | 24396 | -0.044 | 0.1408 | No |
| 57 | HACL1 | 2-hydroxyacyl-CoA lyase 1 [Source:HGNC Symbol;Acc:HGNC:17856] | 24447 | -0.045 | 0.1430 | No |
| 58 | HSD17B4 | hydroxysteroid 17-beta dehydrogenase 4 [Source:HGNC Symbol;Acc:HGNC:5213] | 25563 | -0.053 | 0.1185 | No |
| 59 | CAT | catalase [Source:HGNC Symbol;Acc:HGNC:1516] | 26690 | -0.061 | 0.0945 | No |
| 60 | ACSL1 | acyl-CoA synthetase long chain family member 1 [Source:HGNC Symbol;Acc:HGNC:3569] | 27078 | -0.064 | 0.0894 | No |
| 61 | PECR | peroxisomal trans-2-enoyl-CoA reductase [Source:HGNC Symbol;Acc:HGNC:18281] | 27272 | -0.065 | 0.0895 | No |
| 62 | ACOX1 | acyl-CoA oxidase 1 [Source:HGNC Symbol;Acc:HGNC:119] | 27845 | -0.070 | 0.0802 | No |
| 63 | DDO | D-aspartate oxidase [Source:HGNC Symbol;Acc:HGNC:2727] | 29249 | -0.082 | 0.0507 | No |
| 64 | DAO | D-amino acid oxidase [Source:HGNC Symbol;Acc:HGNC:2671] | 29678 | -0.086 | 0.0463 | No |
| 65 | PIPOX | pipecolic acid and sarcosine oxidase [Source:HGNC Symbol;Acc:HGNC:17804] | 29738 | -0.087 | 0.0514 | No |
| 66 | PEX26 | peroxisomal biogenesis factor 26 [Source:HGNC Symbol;Acc:HGNC:22965] | 30876 | -0.099 | 0.0299 | No |
| 67 | SLC27A2 | solute carrier family 27 member 2 [Source:HGNC Symbol;Acc:HGNC:10996] | 32500 | -0.120 | -0.0023 | No |
| 68 | PEX5 | peroxisomal biogenesis factor 5 [Source:HGNC Symbol;Acc:HGNC:9719] | 33632 | -0.139 | -0.0206 | No |
| 69 | ACSL6 | acyl-CoA synthetase long chain family member 6 [Source:HGNC Symbol;Acc:HGNC:16496] | 33792 | -0.142 | -0.0140 | No |
| 70 | NOS2 | nitric oxide synthase 2 [Source:HGNC Symbol;Acc:HGNC:7873] | 33997 | -0.145 | -0.0081 | No |
| 71 | PEX1 | peroxisomal biogenesis factor 1 [Source:HGNC Symbol;Acc:HGNC:8850] | 34014 | -0.146 | 0.0025 | No |
| 72 | ACSL3 | acyl-CoA synthetase long chain family member 3 [Source:HGNC Symbol;Acc:HGNC:3570] | 35301 | -0.174 | -0.0171 | No |
| 73 | ACSL4 | acyl-CoA synthetase long chain family member 4 [Source:HGNC Symbol;Acc:HGNC:3571] | 35339 | -0.175 | -0.0048 | No |
| 74 | FAR2 | fatty acyl-CoA reductase 2 [Source:HGNC Symbol;Acc:HGNC:25531] | 36963 | -0.234 | -0.0284 | No |
| 75 | AGPS | alkylglycerone phosphate synthase [Source:HGNC Symbol;Acc:HGNC:327] | 37056 | -0.239 | -0.0127 | No |
| 76 | SOD2 | superoxide dismutase 2 [Source:HGNC Symbol;Acc:HGNC:11180] | 37689 | -0.283 | -0.0074 | No |
| 77 | FAR1 | fatty acyl-CoA reductase 1 [Source:HGNC Symbol;Acc:HGNC:26222] | 37907 | -0.304 | 0.0102 | No |
| 78 | ABCD2 | ATP binding cassette subfamily D member 2 [Source:HGNC Symbol;Acc:HGNC:66] | 38133 | -0.341 | 0.0303 | No |
Table: GSEA details [plain text format]

  

Fig 2: KEGG\_PEROXISOME      
 Blue-Pink O' Gram in the Space of the Analyzed GeneSet

  

Fig 3: KEGG\_PEROXISOME: Random ES distribution      
 Gene set null distribution of ES for **KEGG\_PEROXISOME**

  
