# Supplementary material for: Simultaneous Presentation of Multiple Myeloma and Lung Cancer: Case Report and Gene Bioinformatics Analysis
Source: Front Oncol. 2022 Jun 13;12:859735. doi: 10.3389/fonc.2022.859735 (PMC9235397; doi:10.3389/fonc.2022.859735)
Supplement: Supplementary file 1 [file DataSheet_1.zip › The bioinformatic analysis of MM and lung cancer supplementary materials/Enrichment analysis/MECR/GSEA_4.1.0/LUAD TCGA/KEGG.Gsea.1639041756227/KEGG_PORPHYRIN_AND_CHLOROPHYLL_METABOLISM.html]

Details for gene set KEGG\_PORPHYRIN\_AND\_CHLOROPHYLL\_METABOLISM[GSEA]

|  || Dataset | ExpData\_collapsed\_to\_symbols.ENSG00000116353\_profile\_in\_ExpData.cls #ENSG00000116353 |
| Phenotype | ENSG00000116353\_profile\_in\_ExpData.cls#ENSG00000116353 |
| Upregulated in class | ENSG00000116353\_pos |
| GeneSet | KEGG\_PORPHYRIN\_AND\_CHLOROPHYLL\_METABOLISM |
| Enrichment Score (ES) | 0.566545 |
| Normalized Enrichment Score (NES) | 1.9514928 |
| Nominal p-value | 0.0 |
| FDR q-value | 0.0018870025 |
| FWER p-Value | 0.029 |
Table: GSEA Results Summary

  

Fig 1: Enrichment plot: KEGG\_PORPHYRIN\_AND\_CHLOROPHYLL\_METABOLISM      
 Profile of the Running ES Score & Positions of GeneSet Members on the Rank Ordered List

  

| SYMBOL | TITLE | RANK IN GENE LIST | RANK METRIC SCORE | RUNNING ES | CORE ENRICHMENT || 1 | UROD | uroporphyrinogen decarboxylase [Source:HGNC Symbol;Acc:HGNC:12591] | 14 | 0.488 | 0.1030 | Yes |
| 2 | PPOX | protoporphyrinogen oxidase [Source:HGNC Symbol;Acc:HGNC:9280] | 245 | 0.362 | 0.1736 | Yes |
| 3 | UROS | uroporphyrinogen III synthase [Source:HGNC Symbol;Acc:HGNC:12592] | 248 | 0.361 | 0.2500 | Yes |
| 4 | HMBS | hydroxymethylbilane synthase [Source:HGNC Symbol;Acc:HGNC:4982] | 614 | 0.306 | 0.3055 | Yes |
| 5 | BLVRA | biliverdin reductase A [Source:HGNC Symbol;Acc:HGNC:1062] | 746 | 0.293 | 0.3641 | Yes |
| 6 | BLVRB | biliverdin reductase B [Source:HGNC Symbol;Acc:HGNC:1063] | 762 | 0.292 | 0.4255 | Yes |
| 7 | GUSB | glucuronidase beta [Source:HGNC Symbol;Acc:HGNC:4696] | 929 | 0.276 | 0.4798 | Yes |
| 8 | HMOX2 | heme oxygenase 2 [Source:HGNC Symbol;Acc:HGNC:5014] | 1422 | 0.240 | 0.5181 | Yes |
| 9 | MMAB | metabolism of cobalamin associated B [Source:HGNC Symbol;Acc:HGNC:19331] | 2105 | 0.201 | 0.5432 | Yes |
| 10 | EARS2 | "glutamyl-tRNA synthetase 2, mitochondrial [Source:HGNC Symbol;Acc:HGNC:29419]" | 3261 | 0.156 | 0.5468 | Yes |
| 11 | FTH1 | ferritin heavy chain 1 [Source:HGNC Symbol;Acc:HGNC:3976] | 3673 | 0.143 | 0.5665 | Yes |
| 12 | ALAD | aminolevulinate dehydratase [Source:HGNC Symbol;Acc:HGNC:395] | 6931 | 0.080 | 0.5006 | No |
| 13 | FTMT | ferritin mitochondrial [Source:HGNC Symbol;Acc:HGNC:17345] | 7921 | 0.068 | 0.4899 | No |
| 14 | UGT1A8 | UDP glucuronosyltransferase family 1 member A8 [Source:HGNC Symbol;Acc:HGNC:12540] | 8116 | 0.066 | 0.4990 | No |
| 15 | COX15 | cytochrome c oxidase assembly homolog COX15 [Source:HGNC Symbol;Acc:HGNC:2263] | 9577 | 0.052 | 0.4729 | No |
| 16 | CPOX | coproporphyrinogen oxidase [Source:HGNC Symbol;Acc:HGNC:2321] | 11067 | 0.041 | 0.4435 | No |
| 17 | UGT2B15 | UDP glucuronosyltransferase family 2 member B15 [Source:HGNC Symbol;Acc:HGNC:12546] | 15020 | 0.013 | 0.3457 | No |
| 18 | UGT2B11 | UDP glucuronosyltransferase family 2 member B11 [Source:HGNC Symbol;Acc:HGNC:12545] | 15519 | 0.010 | 0.3351 | No |
| 19 | UGT1A6 | UDP glucuronosyltransferase family 1 member A6 [Source:HGNC Symbol;Acc:HGNC:12538] | 15522 | 0.010 | 0.3371 | No |
| 20 | UGT2B10 | UDP glucuronosyltransferase family 2 member B10 [Source:HGNC Symbol;Acc:HGNC:12544] | 16213 | 0.006 | 0.3208 | No |
| 21 | UGT1A4 | UDP glucuronosyltransferase family 1 member A4 [Source:HGNC Symbol;Acc:HGNC:12536] | 16628 | 0.003 | 0.3109 | No |
| 22 | UGT1A5 | UDP glucuronosyltransferase family 1 member A5 [Source:HGNC Symbol;Acc:HGNC:12537] | 19034 | -0.011 | 0.2521 | No |
| 23 | UGT1A7 | UDP glucuronosyltransferase family 1 member A7 [Source:HGNC Symbol;Acc:HGNC:12539] | 19950 | -0.016 | 0.2322 | No |
| 24 | COX10 | cytochrome c oxidase assembly factor heme A:farnesyltransferase COX10 [Source:HGNC Symbol;Acc:HGNC:2260] | 20308 | -0.018 | 0.2271 | No |
| 25 | UGT2B17 | UDP glucuronosyltransferase family 2 member B17 [Source:HGNC Symbol;Acc:HGNC:12547] | 22054 | -0.029 | 0.1888 | No |
| 26 | UGT1A9 | UDP glucuronosyltransferase family 1 member A9 [Source:HGNC Symbol;Acc:HGNC:12541] | 22409 | -0.031 | 0.1864 | No |
| 27 | UGT2B7 | UDP glucuronosyltransferase family 2 member B7 [Source:HGNC Symbol;Acc:HGNC:12554] | 22724 | -0.033 | 0.1855 | No |
| 28 | FECH | ferrochelatase [Source:HGNC Symbol;Acc:HGNC:3647] | 23037 | -0.035 | 0.1851 | No |
| 29 | UGT1A10 | UDP glucuronosyltransferase family 1 member A10 [Source:HGNC Symbol;Acc:HGNC:12531] | 24365 | -0.044 | 0.1607 | No |
| 30 | HCCS | holocytochrome c synthase [Source:HGNC Symbol;Acc:HGNC:4837] | 25110 | -0.050 | 0.1522 | No |
| 31 | ALAS2 | 5'-aminolevulinate synthase 2 [Source:HGNC Symbol;Acc:HGNC:397] | 25866 | -0.055 | 0.1446 | No |
| 32 | UGT2A3 | UDP glucuronosyltransferase family 2 member A3 [Source:HGNC Symbol;Acc:HGNC:28528] | 26505 | -0.059 | 0.1409 | No |
| 33 | HMOX1 | heme oxygenase 1 [Source:HGNC Symbol;Acc:HGNC:5013] | 28775 | -0.078 | 0.0997 | No |
| 34 | UGT1A3 | UDP glucuronosyltransferase family 1 member A3 [Source:HGNC Symbol;Acc:HGNC:12535] | 29128 | -0.081 | 0.1078 | No |
| 35 | ALAS1 | 5'-aminolevulinate synthase 1 [Source:HGNC Symbol;Acc:HGNC:396] | 29495 | -0.085 | 0.1164 | No |
| 36 | CP | ceruloplasmin [Source:HGNC Symbol;Acc:HGNC:2295] | 30144 | -0.091 | 0.1192 | No |
| 37 | UGT1A1 | UDP glucuronosyltransferase family 1 member A1 [Source:HGNC Symbol;Acc:HGNC:12530] | 30535 | -0.095 | 0.1294 | No |
| 38 | EPRS1 | glutamyl-prolyl-tRNA synthetase 1 [Source:HGNC Symbol;Acc:HGNC:3418] | 31328 | -0.104 | 0.1314 | No |
| 39 | UGT2B28 | UDP glucuronosyltransferase family 2 member B28 [Source:HGNC Symbol;Acc:HGNC:13479] | 31788 | -0.110 | 0.1430 | No |
| 40 | UGT2B4 | UDP glucuronosyltransferase family 2 member B4 [Source:HGNC Symbol;Acc:HGNC:12553] | 31920 | -0.112 | 0.1634 | No |
| 41 | UGT2A1 | UDP glucuronosyltransferase family 2 member A1 complex locus [Source:HGNC Symbol;Acc:HGNC:12542] | 32392 | -0.118 | 0.1764 | No |
Table: GSEA details [plain text format]

  

Fig 2: KEGG\_PORPHYRIN\_AND\_CHLOROPHYLL\_METABOLISM      
 Blue-Pink O' Gram in the Space of the Analyzed GeneSet

  

Fig 3: KEGG\_PORPHYRIN\_AND\_CHLOROPHYLL\_METABOLISM: Random ES distribution      
 Gene set null distribution of ES for **KEGG\_PORPHYRIN\_AND\_CHLOROPHYLL\_METABOLISM**

  
