# Supplementary material for: Simultaneous Presentation of Multiple Myeloma and Lung Cancer: Case Report and Gene Bioinformatics Analysis
Source: Front Oncol. 2022 Jun 13;12:859735. doi: 10.3389/fonc.2022.859735 (PMC9235397; doi:10.3389/fonc.2022.859735)
Supplement: Supplementary file 1 [file DataSheet_1.zip › The bioinformatic analysis of MM and lung cancer supplementary materials/Enrichment analysis/MECR/GSEA_4.1.0/LUAD TCGA/KEGG.Gsea.1639041756227/KEGG_PRIMARY_IMMUNODEFICIENCY.html]

Details for gene set KEGG\_PRIMARY\_IMMUNODEFICIENCY[GSEA]

|  || Dataset | ExpData\_collapsed\_to\_symbols.ENSG00000116353\_profile\_in\_ExpData.cls #ENSG00000116353 |
| Phenotype | ENSG00000116353\_profile\_in\_ExpData.cls#ENSG00000116353 |
| Upregulated in class | ENSG00000116353\_neg |
| GeneSet | KEGG\_PRIMARY\_IMMUNODEFICIENCY |
| Enrichment Score (ES) | -0.654759 |
| Normalized Enrichment Score (NES) | -2.20849 |
| Nominal p-value | 0.0 |
| FDR q-value | 9.0501795E-5 |
| FWER p-Value | 0.001 |
Table: GSEA Results Summary

  

Fig 1: Enrichment plot: KEGG\_PRIMARY\_IMMUNODEFICIENCY      
 Profile of the Running ES Score & Positions of GeneSet Members on the Rank Ordered List

  

| SYMBOL | TITLE | RANK IN GENE LIST | RANK METRIC SCORE | RUNNING ES | CORE ENRICHMENT || 1 | RFXANK | regulatory factor X associated ankyrin containing protein [Source:HGNC Symbol;Acc:HGNC:9987] | 62 | 0.423 | 0.0726 | No |
| 2 | IKBKG | inhibitor of nuclear factor kappa B kinase regulatory subunit gamma [Source:HGNC Symbol;Acc:HGNC:5961] | 9304 | 0.055 | -0.1530 | No |
| 3 | RFX5 | regulatory factor X5 [Source:HGNC Symbol;Acc:HGNC:9986] | 10954 | 0.041 | -0.1878 | No |
| 4 | CD40 | CD40 molecule [Source:HGNC Symbol;Acc:HGNC:11919] | 17012 | 0.001 | -0.3417 | No |
| 5 | RFXAP | regulatory factor X associated protein [Source:HGNC Symbol;Acc:HGNC:9988] | 22759 | -0.034 | -0.4821 | No |
| 6 | RAG2 | recombination activating 2 [Source:HGNC Symbol;Acc:HGNC:9832] | 23204 | -0.037 | -0.4870 | No |
| 7 | UNG | uracil DNA glycosylase [Source:HGNC Symbol;Acc:HGNC:12572] | 23496 | -0.039 | -0.4876 | No |
| 8 | DCLRE1C | DNA cross-link repair 1C [Source:HGNC Symbol;Acc:HGNC:17642] | 29760 | -0.087 | -0.6318 | No |
| 9 | IGLL1 | immunoglobulin lambda like polypeptide 1 [Source:HGNC Symbol;Acc:HGNC:5870] | 30458 | -0.095 | -0.6329 | No |
| 10 | TAP2 | "transporter 2, ATP binding cassette subfamily B member [Source:HGNC Symbol;Acc:HGNC:44]" | 30866 | -0.099 | -0.6260 | No |
| 11 | BLNK | B cell linker [Source:HGNC Symbol;Acc:HGNC:14211] | 31998 | -0.113 | -0.6350 | Yes |
| 12 | TAP1 | "transporter 1, ATP binding cassette subfamily B member [Source:HGNC Symbol;Acc:HGNC:43]" | 32668 | -0.123 | -0.6305 | Yes |
| 13 | CIITA | class II major histocompatibility complex transactivator [Source:HGNC Symbol;Acc:HGNC:7067] | 32961 | -0.127 | -0.6157 | Yes |
| 14 | RAG1 | recombination activating 1 [Source:HGNC Symbol;Acc:HGNC:9831] | 32971 | -0.127 | -0.5936 | Yes |
| 15 | TNFRSF13B | TNF receptor superfamily member 13B [Source:HGNC Symbol;Acc:HGNC:18153] | 33212 | -0.131 | -0.5767 | Yes |
| 16 | AIRE | autoimmune regulator [Source:HGNC Symbol;Acc:HGNC:360] | 34018 | -0.146 | -0.5717 | Yes |
| 17 | ADA | adenosine deaminase [Source:HGNC Symbol;Acc:HGNC:186] | 34279 | -0.151 | -0.5519 | Yes |
| 18 | JAK3 | Janus kinase 3 [Source:HGNC Symbol;Acc:HGNC:6193] | 34533 | -0.155 | -0.5312 | Yes |
| 19 | TNFRSF13C | TNF receptor superfamily member 13C [Source:HGNC Symbol;Acc:HGNC:17755] | 34842 | -0.163 | -0.5105 | Yes |
| 20 | ZAP70 | zeta chain of T cell receptor associated protein kinase 70 [Source:HGNC Symbol;Acc:HGNC:12858] | 34981 | -0.166 | -0.4850 | Yes |
| 21 | CD3D | CD3d molecule [Source:HGNC Symbol;Acc:HGNC:1673] | 35025 | -0.167 | -0.4568 | Yes |
| 22 | CD40LG | CD40 ligand [Source:HGNC Symbol;Acc:HGNC:11935] | 35185 | -0.171 | -0.4310 | Yes |
| 23 | AICDA | activation induced cytidine deaminase [Source:HGNC Symbol;Acc:HGNC:13203] | 35567 | -0.181 | -0.4089 | Yes |
| 24 | CD3E | CD3e molecule [Source:HGNC Symbol;Acc:HGNC:1674] | 35751 | -0.186 | -0.3810 | Yes |
| 25 | CD19 | CD19 molecule [Source:HGNC Symbol;Acc:HGNC:1633] | 35881 | -0.190 | -0.3510 | Yes |
| 26 | CD79A | CD79a molecule [Source:HGNC Symbol;Acc:HGNC:1698] | 35992 | -0.194 | -0.3199 | Yes |
| 27 | CD8B | CD8b molecule [Source:HGNC Symbol;Acc:HGNC:1707] | 36153 | -0.199 | -0.2891 | Yes |
| 28 | CD8A | CD8a molecule [Source:HGNC Symbol;Acc:HGNC:1706] | 36547 | -0.213 | -0.2618 | Yes |
| 29 | ICOS | inducible T cell costimulator [Source:HGNC Symbol;Acc:HGNC:5351] | 37068 | -0.239 | -0.2332 | Yes |
| 30 | LCK | "LCK proto-oncogene, Src family tyrosine kinase [Source:HGNC Symbol;Acc:HGNC:6524]" | 37085 | -0.240 | -0.1914 | Yes |
| 31 | BTK | Bruton tyrosine kinase [Source:HGNC Symbol;Acc:HGNC:1133] | 37120 | -0.243 | -0.1498 | Yes |
| 32 | IL2RG | interleukin 2 receptor subunit gamma [Source:HGNC Symbol;Acc:HGNC:6010] | 37182 | -0.247 | -0.1081 | Yes |
| 33 | CD4 | CD4 molecule [Source:HGNC Symbol;Acc:HGNC:1678] | 37205 | -0.248 | -0.0651 | Yes |
| 34 | IL7R | interleukin 7 receptor [Source:HGNC Symbol;Acc:HGNC:6024] | 37941 | -0.309 | -0.0297 | Yes |
| 35 | PTPRC | protein tyrosine phosphatase receptor type C [Source:HGNC Symbol;Acc:HGNC:9666] | 38216 | -0.370 | 0.0282 | Yes |
Table: GSEA details [plain text format]

  

Fig 2: KEGG\_PRIMARY\_IMMUNODEFICIENCY      
 Blue-Pink O' Gram in the Space of the Analyzed GeneSet

  

Fig 3: KEGG\_PRIMARY\_IMMUNODEFICIENCY: Random ES distribution      
 Gene set null distribution of ES for **KEGG\_PRIMARY\_IMMUNODEFICIENCY**

  
