# Supplementary material for: Simultaneous Presentation of Multiple Myeloma and Lung Cancer: Case Report and Gene Bioinformatics Analysis
Source: Front Oncol. 2022 Jun 13;12:859735. doi: 10.3389/fonc.2022.859735 (PMC9235397; doi:10.3389/fonc.2022.859735)
Supplement: Supplementary file 1 [file DataSheet_1.zip › The bioinformatic analysis of MM and lung cancer supplementary materials/Enrichment analysis/MECR/GSEA_4.1.0/LUAD TCGA/KEGG.Gsea.1639041756227/KEGG_PROTEASOME.html]

Details for gene set KEGG\_PROTEASOME[GSEA]

|  || Dataset | ExpData\_collapsed\_to\_symbols.ENSG00000116353\_profile\_in\_ExpData.cls #ENSG00000116353 |
| Phenotype | ENSG00000116353\_profile\_in\_ExpData.cls#ENSG00000116353 |
| Upregulated in class | ENSG00000116353\_pos |
| GeneSet | KEGG\_PROTEASOME |
| Enrichment Score (ES) | 0.6407937 |
| Normalized Enrichment Score (NES) | 2.2702491 |
| Nominal p-value | 0.0 |
| FDR q-value | 0.0 |
| FWER p-Value | 0.0 |
Table: GSEA Results Summary

  

Fig 1: Enrichment plot: KEGG\_PROTEASOME      
 Profile of the Running ES Score & Positions of GeneSet Members on the Rank Ordered List

  

| SYMBOL | TITLE | RANK IN GENE LIST | RANK METRIC SCORE | RUNNING ES | CORE ENRICHMENT || 1 | PSMB2 | proteasome 20S subunit beta 2 [Source:HGNC Symbol;Acc:HGNC:9539] | 169 | 0.381 | 0.0555 | Yes |
| 2 | PSMD4 | "proteasome 26S subunit, non-ATPase 4 [Source:HGNC Symbol;Acc:HGNC:9561]" | 331 | 0.346 | 0.1057 | Yes |
| 3 | PSMB4 | proteasome 20S subunit beta 4 [Source:HGNC Symbol;Acc:HGNC:9541] | 381 | 0.336 | 0.1571 | Yes |
| 4 | PSMB3 | proteasome 20S subunit beta 3 [Source:HGNC Symbol;Acc:HGNC:9540] | 848 | 0.284 | 0.1898 | Yes |
| 5 | PSMC3 | "proteasome 26S subunit, ATPase 3 [Source:HGNC Symbol;Acc:HGNC:9549]" | 971 | 0.272 | 0.2294 | Yes |
| 6 | PSMB6 | proteasome 20S subunit beta 6 [Source:HGNC Symbol;Acc:HGNC:9543] | 1292 | 0.248 | 0.2602 | Yes |
| 7 | PSMA2 | proteasome 20S subunit alpha 2 [Source:HGNC Symbol;Acc:HGNC:9531] | 1335 | 0.245 | 0.2976 | Yes |
| 8 | PSMD13 | "proteasome 26S subunit, non-ATPase 13 [Source:HGNC Symbol;Acc:HGNC:9558]" | 1401 | 0.241 | 0.3339 | Yes |
| 9 | PSMB5 | proteasome 20S subunit beta 5 [Source:HGNC Symbol;Acc:HGNC:9542] | 1436 | 0.239 | 0.3705 | Yes |
| 10 | PSMC5 | "proteasome 26S subunit, ATPase 5 [Source:HGNC Symbol;Acc:HGNC:9552]" | 1458 | 0.238 | 0.4073 | Yes |
| 11 | SEM1 | SEM1 26S proteasome complex subunit [Source:HGNC Symbol;Acc:HGNC:10845] | 1871 | 0.214 | 0.4304 | Yes |
| 12 | PSMB7 | proteasome 20S subunit beta 7 [Source:HGNC Symbol;Acc:HGNC:9544] | 2308 | 0.192 | 0.4495 | Yes |
| 13 | PSMD3 | "proteasome 26S subunit, non-ATPase 3 [Source:HGNC Symbol;Acc:HGNC:9560]" | 2960 | 0.166 | 0.4589 | Yes |
| 14 | PSMB1 | proteasome 20S subunit beta 1 [Source:HGNC Symbol;Acc:HGNC:9537] | 3075 | 0.161 | 0.4813 | Yes |
| 15 | PSME1 | proteasome activator subunit 1 [Source:HGNC Symbol;Acc:HGNC:9568] | 3098 | 0.161 | 0.5060 | Yes |
| 16 | PSMA7 | proteasome 20S subunit alpha 7 [Source:HGNC Symbol;Acc:HGNC:9536] | 3538 | 0.147 | 0.5179 | Yes |
| 17 | PSMC2 | "proteasome 26S subunit, ATPase 2 [Source:HGNC Symbol;Acc:HGNC:9548]" | 4065 | 0.132 | 0.5252 | Yes |
| 18 | PSME2 | proteasome activator subunit 2 [Source:HGNC Symbol;Acc:HGNC:9569] | 4405 | 0.124 | 0.5360 | Yes |
| 19 | PSMD8 | "proteasome 26S subunit, non-ATPase 8 [Source:HGNC Symbol;Acc:HGNC:9566]" | 4437 | 0.123 | 0.5545 | Yes |
| 20 | PSMC4 | "proteasome 26S subunit, ATPase 4 [Source:HGNC Symbol;Acc:HGNC:9551]" | 4897 | 0.113 | 0.5605 | Yes |
| 21 | PSMA1 | proteasome 20S subunit alpha 1 [Source:HGNC Symbol;Acc:HGNC:9530] | 5035 | 0.111 | 0.5744 | Yes |
| 22 | PSMA5 | proteasome 20S subunit alpha 5 [Source:HGNC Symbol;Acc:HGNC:9534] | 5335 | 0.105 | 0.5833 | Yes |
| 23 | PSMB10 | proteasome 20S subunit beta 10 [Source:HGNC Symbol;Acc:HGNC:9538] | 5813 | 0.097 | 0.5863 | Yes |
| 24 | PSMD6 | "proteasome 26S subunit, non-ATPase 6 [Source:HGNC Symbol;Acc:HGNC:9564]" | 6100 | 0.092 | 0.5935 | Yes |
| 25 | PSMB8 | proteasome 20S subunit beta 8 [Source:HGNC Symbol;Acc:HGNC:9545] | 6106 | 0.092 | 0.6078 | Yes |
| 26 | PSMA4 | proteasome 20S subunit alpha 4 [Source:HGNC Symbol;Acc:HGNC:9533] | 6119 | 0.092 | 0.6218 | Yes |
| 27 | PSMF1 | proteasome inhibitor subunit 1 [Source:HGNC Symbol;Acc:HGNC:9571] | 6352 | 0.088 | 0.6297 | Yes |
| 28 | PSMA3 | proteasome 20S subunit alpha 3 [Source:HGNC Symbol;Acc:HGNC:9532] | 6453 | 0.087 | 0.6408 | Yes |
| 29 | PSMD14 | "proteasome 26S subunit, non-ATPase 14 [Source:HGNC Symbol;Acc:HGNC:16889]" | 7047 | 0.079 | 0.6381 | No |
| 30 | PSMD12 | "proteasome 26S subunit, non-ATPase 12 [Source:HGNC Symbol;Acc:HGNC:9557]" | 7400 | 0.074 | 0.6408 | No |
| 31 | PSMA6P4 | proteasome subunit alpha 6 pseudogene 4 [Source:HGNC Symbol;Acc:HGNC:39609] | 8568 | 0.062 | 0.6207 | No |
| 32 | POMP | proteasome maturation protein [Source:HGNC Symbol;Acc:HGNC:20330] | 8691 | 0.060 | 0.6271 | No |
| 33 | PSMC6 | "proteasome 26S subunit, ATPase 6 [Source:HGNC Symbol;Acc:HGNC:9553]" | 10808 | 0.042 | 0.5799 | No |
| 34 | PSMC1P4 | "proteasome 26S subunit, ATPase 1 pseudogene 4 [Source:HGNC Symbol;Acc:HGNC:39779]" | 11113 | 0.040 | 0.5784 | No |
| 35 | PSMA6 | proteasome 20S subunit alpha 6 [Source:HGNC Symbol;Acc:HGNC:9535] | 13682 | 0.021 | 0.5164 | No |
| 36 | PSMB11 | proteasome subunit beta 11 [Source:HGNC Symbol;Acc:HGNC:31963] | 14428 | 0.017 | 0.5000 | No |
| 37 | PSMD2 | "proteasome 26S subunit, non-ATPase 2 [Source:HGNC Symbol;Acc:HGNC:9559]" | 15749 | 0.008 | 0.4677 | No |
| 38 | PSMD11 | "proteasome 26S subunit, non-ATPase 11 [Source:HGNC Symbol;Acc:HGNC:9556]" | 16838 | 0.002 | 0.4404 | No |
| 39 | PSMD7 | "proteasome 26S subunit, non-ATPase 7 [Source:HGNC Symbol;Acc:HGNC:9565]" | 18206 | -0.006 | 0.4065 | No |
| 40 | PSMB9 | proteasome 20S subunit beta 9 [Source:HGNC Symbol;Acc:HGNC:9546] | 25243 | -0.050 | 0.2353 | No |
| 41 | PSME3 | proteasome activator subunit 3 [Source:HGNC Symbol;Acc:HGNC:9570] | 25891 | -0.055 | 0.2275 | No |
| 42 | PSMC1 | "proteasome 26S subunit, ATPase 1 [Source:HGNC Symbol;Acc:HGNC:9547]" | 29874 | -0.088 | 0.1400 | No |
| 43 | PSMD1 | "proteasome 26S subunit, non-ATPase 1 [Source:HGNC Symbol;Acc:HGNC:9554]" | 33963 | -0.145 | 0.0586 | No |
| 44 | PSME4 | proteasome activator subunit 4 [Source:HGNC Symbol;Acc:HGNC:20635] | 34408 | -0.153 | 0.0713 | No |
| 45 | PSMA8 | proteasome 20S subunit alpha 8 [Source:HGNC Symbol;Acc:HGNC:22985] | 34681 | -0.159 | 0.0894 | No |
| 46 | IFNG | interferon gamma [Source:HGNC Symbol;Acc:HGNC:5438] | 35652 | -0.183 | 0.0935 | No |
Table: GSEA details [plain text format]

  

Fig 2: KEGG\_PROTEASOME      
 Blue-Pink O' Gram in the Space of the Analyzed GeneSet

  

Fig 3: KEGG\_PROTEASOME: Random ES distribution      
 Gene set null distribution of ES for **KEGG\_PROTEASOME**

  
