# Supplementary material for: Simultaneous Presentation of Multiple Myeloma and Lung Cancer: Case Report and Gene Bioinformatics Analysis
Source: Front Oncol. 2022 Jun 13;12:859735. doi: 10.3389/fonc.2022.859735 (PMC9235397; doi:10.3389/fonc.2022.859735)
Supplement: Supplementary file 1 [file DataSheet_1.zip › The bioinformatic analysis of MM and lung cancer supplementary materials/Enrichment analysis/MECR/GSEA_4.1.0/LUAD TCGA/KEGG.Gsea.1639041756227/KEGG_PURINE_METABOLISM.html]

Details for gene set KEGG\_PURINE\_METABOLISM[GSEA]

|  || Dataset | ExpData\_collapsed\_to\_symbols.ENSG00000116353\_profile\_in\_ExpData.cls #ENSG00000116353 |
| Phenotype | ENSG00000116353\_profile\_in\_ExpData.cls#ENSG00000116353 |
| Upregulated in class | ENSG00000116353\_pos |
| GeneSet | KEGG\_PURINE\_METABOLISM |
| Enrichment Score (ES) | 0.40961534 |
| Normalized Enrichment Score (NES) | 1.8366693 |
| Nominal p-value | 0.0 |
| FDR q-value | 0.0064954925 |
| FWER p-Value | 0.108 |
Table: GSEA Results Summary

  

Fig 1: Enrichment plot: KEGG\_PURINE\_METABOLISM      
 Profile of the Running ES Score & Positions of GeneSet Members on the Rank Ordered List

  

| SYMBOL | TITLE | RANK IN GENE LIST | RANK METRIC SCORE | RUNNING ES | CORE ENRICHMENT || 1 | NME3 | NME/NM23 nucleoside diphosphate kinase 3 [Source:HGNC Symbol;Acc:HGNC:7851] | 35 | 0.444 | 0.0184 | Yes |
| 2 | AK2 | adenylate kinase 2 [Source:HGNC Symbol;Acc:HGNC:362] | 99 | 0.403 | 0.0343 | Yes |
| 3 | POLR2L | "RNA polymerase II, I and III subunit L [Source:HGNC Symbol;Acc:HGNC:9199]" | 117 | 0.396 | 0.0510 | Yes |
| 4 | APRT | adenine phosphoribosyltransferase [Source:HGNC Symbol;Acc:HGNC:626] | 160 | 0.383 | 0.0666 | Yes |
| 5 | GUK1 | guanylate kinase 1 [Source:HGNC Symbol;Acc:HGNC:4693] | 167 | 0.382 | 0.0830 | Yes |
| 6 | NME4 | NME/NM23 nucleoside diphosphate kinase 4 [Source:HGNC Symbol;Acc:HGNC:7852] | 216 | 0.370 | 0.0978 | Yes |
| 7 | POLD2 | "DNA polymerase delta 2, accessory subunit [Source:HGNC Symbol;Acc:HGNC:9176]" | 355 | 0.340 | 0.1090 | Yes |
| 8 | NT5C | "5', 3'-nucleotidase, cytosolic [Source:HGNC Symbol;Acc:HGNC:17144]" | 408 | 0.332 | 0.1221 | Yes |
| 9 | POLR1H | RNA polymerase I subunit H [Source:HGNC Symbol;Acc:HGNC:13182] | 575 | 0.310 | 0.1313 | Yes |
| 10 | FHIT | fragile histidine triad diadenosine triphosphatase [Source:HGNC Symbol;Acc:HGNC:3701] | 606 | 0.307 | 0.1439 | Yes |
| 11 | POLR2E | "RNA polymerase II, I and III subunit E [Source:HGNC Symbol;Acc:HGNC:9192]" | 607 | 0.307 | 0.1572 | Yes |
| 12 | ITPA | inosine triphosphatase [Source:HGNC Symbol;Acc:HGNC:6176] | 640 | 0.304 | 0.1696 | Yes |
| 13 | POLR2J | RNA polymerase II subunit J [Source:HGNC Symbol;Acc:HGNC:9197] | 736 | 0.294 | 0.1799 | Yes |
| 14 | POLR1C | RNA polymerase I and III subunit C [Source:HGNC Symbol;Acc:HGNC:20194] | 749 | 0.293 | 0.1923 | Yes |
| 15 | POLR3H | RNA polymerase III subunit H [Source:HGNC Symbol;Acc:HGNC:30349] | 797 | 0.288 | 0.2036 | Yes |
| 16 | POLR2I | RNA polymerase II subunit I [Source:HGNC Symbol;Acc:HGNC:9196] | 813 | 0.287 | 0.2157 | Yes |
| 17 | NT5M | "5',3'-nucleotidase, mitochondrial [Source:HGNC Symbol;Acc:HGNC:15769]" | 821 | 0.286 | 0.2279 | Yes |
| 18 | POLR2G | RNA polymerase II subunit G [Source:HGNC Symbol;Acc:HGNC:9194] | 999 | 0.270 | 0.2351 | Yes |
| 19 | ADK | adenosine kinase [Source:HGNC Symbol;Acc:HGNC:257] | 1002 | 0.270 | 0.2467 | Yes |
| 20 | POLR2H | "RNA polymerase II, I and III subunit H [Source:HGNC Symbol;Acc:HGNC:9195]" | 1077 | 0.264 | 0.2563 | Yes |
| 21 | POLE4 | "DNA polymerase epsilon 4, accessory subunit [Source:HGNC Symbol;Acc:HGNC:18755]" | 1121 | 0.262 | 0.2666 | Yes |
| 22 | AK1 | adenylate kinase 1 [Source:HGNC Symbol;Acc:HGNC:361] | 1225 | 0.253 | 0.2749 | Yes |
| 23 | NME2 | NME/NM23 nucleoside diphosphate kinase 2 [Source:HGNC Symbol;Acc:HGNC:7850] | 1309 | 0.247 | 0.2835 | Yes |
| 24 | POLR3C | RNA polymerase III subunit C [Source:HGNC Symbol;Acc:HGNC:30076] | 1448 | 0.238 | 0.2903 | Yes |
| 25 | GMPR | guanosine monophosphate reductase [Source:HGNC Symbol;Acc:HGNC:4376] | 1470 | 0.237 | 0.3001 | Yes |
| 26 | GUCY2D | "guanylate cyclase 2D, retinal [Source:HGNC Symbol;Acc:HGNC:4689]" | 1487 | 0.235 | 0.3099 | Yes |
| 27 | DGUOK | deoxyguanosine kinase [Source:HGNC Symbol;Acc:HGNC:2858] | 1561 | 0.231 | 0.3180 | Yes |
| 28 | NME1 | NME/NM23 nucleoside diphosphate kinase 1 [Source:HGNC Symbol;Acc:HGNC:7849] | 1565 | 0.231 | 0.3280 | Yes |
| 29 | IMPDH2 | inosine monophosphate dehydrogenase 2 [Source:HGNC Symbol;Acc:HGNC:6053] | 1693 | 0.224 | 0.3344 | Yes |
| 30 | NUDT5 | nudix hydrolase 5 [Source:HGNC Symbol;Acc:HGNC:8052] | 1712 | 0.222 | 0.3436 | Yes |
| 31 | GMPR2 | guanosine monophosphate reductase 2 [Source:HGNC Symbol;Acc:HGNC:4377] | 1800 | 0.217 | 0.3508 | Yes |
| 32 | POLR3GL | RNA polymerase III subunit GL [Source:HGNC Symbol;Acc:HGNC:28466] | 2080 | 0.202 | 0.3525 | Yes |
| 33 | POLR3K | RNA polymerase III subunit K [Source:HGNC Symbol;Acc:HGNC:14121] | 2176 | 0.198 | 0.3586 | Yes |
| 34 | NME1-NME2 | NME1-NME2 readthrough [Source:HGNC Symbol;Acc:HGNC:33531] | 2299 | 0.193 | 0.3639 | Yes |
| 35 | NME6 | NME/NM23 nucleoside diphosphate kinase 6 [Source:HGNC Symbol;Acc:HGNC:20567] | 2407 | 0.188 | 0.3693 | Yes |
| 36 | IMPDH1 | inosine monophosphate dehydrogenase 1 [Source:HGNC Symbol;Acc:HGNC:6052] | 2479 | 0.185 | 0.3756 | Yes |
| 37 | ADSS1 | adenylosuccinate synthase 1 [Source:HGNC Symbol;Acc:HGNC:20093] | 2619 | 0.179 | 0.3798 | Yes |
| 38 | POLR1D | RNA polymerase I and III subunit D [Source:HGNC Symbol;Acc:HGNC:20422] | 2640 | 0.179 | 0.3870 | Yes |
| 39 | PDE6D | phosphodiesterase 6D [Source:HGNC Symbol;Acc:HGNC:8788] | 2881 | 0.169 | 0.3882 | Yes |
| 40 | PDE9A | phosphodiesterase 9A [Source:HGNC Symbol;Acc:HGNC:8795] | 2976 | 0.165 | 0.3930 | Yes |
| 41 | ATIC | 5-aminoimidazole-4-carboxamide ribonucleotide formyltransferase/IMP cyclohydrolase [Source:HGNC Symbol;Acc:HGNC:794] | 3002 | 0.164 | 0.3995 | Yes |
| 42 | PRUNE1 | prune exopolyphosphatase 1 [Source:HGNC Symbol;Acc:HGNC:13420] | 3218 | 0.157 | 0.4008 | Yes |
| 43 | POLD4 | "DNA polymerase delta 4, accessory subunit [Source:HGNC Symbol;Acc:HGNC:14106]" | 3360 | 0.153 | 0.4038 | Yes |
| 44 | ADSL | adenylosuccinate lyase [Source:HGNC Symbol;Acc:HGNC:291] | 3391 | 0.152 | 0.4096 | Yes |
| 45 | PKM | pyruvate kinase M1/2 [Source:HGNC Symbol;Acc:HGNC:9021] | 4157 | 0.130 | 0.3957 | No |
| 46 | POLR2K | "RNA polymerase II, I and III subunit K [Source:HGNC Symbol;Acc:HGNC:9198]" | 4390 | 0.124 | 0.3952 | No |
| 47 | PDE4C | phosphodiesterase 4C [Source:HGNC Symbol;Acc:HGNC:8782] | 4578 | 0.120 | 0.3956 | No |
| 48 | POLR2C | RNA polymerase II subunit C [Source:HGNC Symbol;Acc:HGNC:9189] | 5263 | 0.106 | 0.3827 | No |
| 49 | NME7 | NME/NM23 family member 7 [Source:HGNC Symbol;Acc:HGNC:20461] | 5422 | 0.103 | 0.3832 | No |
| 50 | PAICS | phosphoribosylaminoimidazole carboxylase and phosphoribosylaminoimidazolesuccinocarboxamide synthase [Source:HGNC Symbol;Acc:HGNC:8587] | 5546 | 0.101 | 0.3844 | No |
| 51 | POLR2F | "RNA polymerase II, I and III subunit F [Source:HGNC Symbol;Acc:HGNC:9193]" | 5558 | 0.101 | 0.3886 | No |
| 52 | PDE6B | phosphodiesterase 6B [Source:HGNC Symbol;Acc:HGNC:8786] | 5679 | 0.099 | 0.3898 | No |
| 53 | POLR1E | RNA polymerase I subunit E [Source:HGNC Symbol;Acc:HGNC:17631] | 6313 | 0.089 | 0.3775 | No |
| 54 | POLD1 | "DNA polymerase delta 1, catalytic subunit [Source:HGNC Symbol;Acc:HGNC:9175]" | 6464 | 0.087 | 0.3774 | No |
| 55 | ENTPD3 | ectonucleoside triphosphate diphosphohydrolase 3 [Source:HGNC Symbol;Acc:HGNC:3365] | 6543 | 0.085 | 0.3791 | No |
| 56 | POLR2J3 | RNA polymerase II subunit J3 [Source:HGNC Symbol;Acc:HGNC:33853] | 6597 | 0.085 | 0.3814 | No |
| 57 | ENTPD6 | ectonucleoside triphosphate diphosphohydrolase 6 [Source:HGNC Symbol;Acc:HGNC:3368] | 6762 | 0.083 | 0.3808 | No |
| 58 | POLE3 | "DNA polymerase epsilon 3, accessory subunit [Source:HGNC Symbol;Acc:HGNC:13546]" | 6932 | 0.080 | 0.3800 | No |
| 59 | GART | "phosphoribosylglycinamide formyltransferase, phosphoribosylglycinamide synthetase, phosphoribosylaminoimidazole synthetase [Source:HGNC Symbol;Acc:HGNC:4163]" | 7316 | 0.075 | 0.3735 | No |
| 60 | PRIM2 | DNA primase subunit 2 [Source:HGNC Symbol;Acc:HGNC:9370] | 7571 | 0.072 | 0.3701 | No |
| 61 | ADSS2 | adenylosuccinate synthase 2 [Source:HGNC Symbol;Acc:HGNC:292] | 8385 | 0.063 | 0.3521 | No |
| 62 | ADPRM | "ADP-ribose/CDP-alcohol diphosphatase, manganese dependent [Source:HGNC Symbol;Acc:HGNC:30925]" | 8544 | 0.062 | 0.3508 | No |
| 63 | NME5 | NME/NM23 family member 5 [Source:HGNC Symbol;Acc:HGNC:7853] | 8714 | 0.060 | 0.3491 | No |
| 64 | NT5C1A | "5'-nucleotidase, cytosolic IA [Source:HGNC Symbol;Acc:HGNC:17819]" | 8730 | 0.060 | 0.3513 | No |
| 65 | ENTPD8 | ectonucleoside triphosphate diphosphohydrolase 8 [Source:HGNC Symbol;Acc:HGNC:24860] | 9095 | 0.057 | 0.3445 | No |
| 66 | PDE4A | phosphodiesterase 4A [Source:HGNC Symbol;Acc:HGNC:8780] | 9857 | 0.050 | 0.3272 | No |
| 67 | NT5E | 5'-nucleotidase ecto [Source:HGNC Symbol;Acc:HGNC:8021] | 10027 | 0.049 | 0.3250 | No |
| 68 | PRPS1L1 | phosphoribosyl pyrophosphate synthetase 1 like 1 [Source:HGNC Symbol;Acc:HGNC:9463] | 10228 | 0.047 | 0.3219 | No |
| 69 | POLR2J2 | RNA polymerase II subunit J2 [Source:HGNC Symbol;Acc:HGNC:23208] | 10680 | 0.044 | 0.3123 | No |
| 70 | NUDT9 | nudix hydrolase 9 [Source:HGNC Symbol;Acc:HGNC:8056] | 10776 | 0.043 | 0.3117 | No |
| 71 | HPRT1 | hypoxanthine phosphoribosyltransferase 1 [Source:HGNC Symbol;Acc:HGNC:5157] | 12865 | 0.027 | 0.2596 | No |
| 72 | AMPD2 | adenosine monophosphate deaminase 2 [Source:HGNC Symbol;Acc:HGNC:469] | 13669 | 0.021 | 0.2400 | No |
| 73 | POLR2D | RNA polymerase II subunit D [Source:HGNC Symbol;Acc:HGNC:9191] | 14063 | 0.019 | 0.2308 | No |
| 74 | NUDT2 | nudix hydrolase 2 [Source:HGNC Symbol;Acc:HGNC:8049] | 14585 | 0.016 | 0.2182 | No |
| 75 | PKLR | pyruvate kinase L/R [Source:HGNC Symbol;Acc:HGNC:9020] | 14801 | 0.014 | 0.2133 | No |
| 76 | ADCY2 | adenylate cyclase 2 [Source:HGNC Symbol;Acc:HGNC:233] | 14873 | 0.014 | 0.2121 | No |
| 77 | ENPP3 | ectonucleotide pyrophosphatase/phosphodiesterase 3 [Source:HGNC Symbol;Acc:HGNC:3358] | 16011 | 0.007 | 0.1834 | No |
| 78 | ADCY10 | adenylate cyclase 10 [Source:HGNC Symbol;Acc:HGNC:21285] | 17366 | -0.001 | 0.1488 | No |
| 79 | ENTPD2 | ectonucleoside triphosphate diphosphohydrolase 2 [Source:HGNC Symbol;Acc:HGNC:3364] | 17634 | -0.003 | 0.1421 | No |
| 80 | NPR1 | natriuretic peptide receptor 1 [Source:HGNC Symbol;Acc:HGNC:7943] | 18279 | -0.006 | 0.1260 | No |
| 81 | PDE1A | phosphodiesterase 1A [Source:HGNC Symbol;Acc:HGNC:8774] | 18799 | -0.010 | 0.1131 | No |
| 82 | XDH | xanthine dehydrogenase [Source:HGNC Symbol;Acc:HGNC:12805] | 19063 | -0.011 | 0.1069 | No |
| 83 | RRM2B | ribonucleotide reductase regulatory TP53 inducible subunit M2B [Source:HGNC Symbol;Acc:HGNC:17296] | 19336 | -0.013 | 0.1005 | No |
| 84 | PFAS | phosphoribosylformylglycinamidine synthase [Source:HGNC Symbol;Acc:HGNC:8863] | 21253 | -0.024 | 0.0527 | No |
| 85 | POLA2 | "DNA polymerase alpha 2, accessory subunit [Source:HGNC Symbol;Acc:HGNC:30073]" | 21468 | -0.026 | 0.0483 | No |
| 86 | PDE6C | phosphodiesterase 6C [Source:HGNC Symbol;Acc:HGNC:8787] | 21656 | -0.027 | 0.0447 | No |
| 87 | PPAT | phosphoribosyl pyrophosphate amidotransferase [Source:HGNC Symbol;Acc:HGNC:9238] | 21985 | -0.029 | 0.0376 | No |
| 88 | POLR3G | RNA polymerase III subunit G [Source:HGNC Symbol;Acc:HGNC:30075] | 21988 | -0.029 | 0.0388 | No |
| 89 | PNPT1 | polyribonucleotide nucleotidyltransferase 1 [Source:HGNC Symbol;Acc:HGNC:23166] | 22014 | -0.029 | 0.0394 | No |
| 90 | PRPS1 | phosphoribosyl pyrophosphate synthetase 1 [Source:HGNC Symbol;Acc:HGNC:9462] | 22068 | -0.029 | 0.0393 | No |
| 91 | ADCY9 | adenylate cyclase 9 [Source:HGNC Symbol;Acc:HGNC:240] | 22247 | -0.030 | 0.0361 | No |
| 92 | PRPS2 | phosphoribosyl pyrophosphate synthetase 2 [Source:HGNC Symbol;Acc:HGNC:9465] | 22942 | -0.035 | 0.0199 | No |
| 93 | URAD | ureidoimidazoline (2-oxo-4-hydroxy-4-carboxy-5-) decarboxylase [Source:HGNC Symbol;Acc:HGNC:17785] | 23923 | -0.041 | -0.0034 | No |
| 94 | NT5C3A | "5'-nucleotidase, cytosolic IIIA [Source:HGNC Symbol;Acc:HGNC:17820]" | 23996 | -0.042 | -0.0034 | No |
| 95 | ADCY1 | adenylate cyclase 1 [Source:HGNC Symbol;Acc:HGNC:232] | 24582 | -0.046 | -0.0163 | No |
| 96 | PDE8B | phosphodiesterase 8B [Source:HGNC Symbol;Acc:HGNC:8794] | 25581 | -0.053 | -0.0395 | No |
| 97 | AK7 | adenylate kinase 7 [Source:HGNC Symbol;Acc:HGNC:20091] | 25699 | -0.054 | -0.0402 | No |
| 98 | AK5 | adenylate kinase 5 [Source:HGNC Symbol;Acc:HGNC:365] | 25899 | -0.055 | -0.0429 | No |
| 99 | PDE6H | phosphodiesterase 6H [Source:HGNC Symbol;Acc:HGNC:8790] | 26353 | -0.058 | -0.0519 | No |
| 100 | POLE2 | "DNA polymerase epsilon 2, accessory subunit [Source:HGNC Symbol;Acc:HGNC:9178]" | 27164 | -0.065 | -0.0698 | No |
| 101 | GUCY1B1 | guanylate cyclase 1 soluble subunit beta 1 [Source:HGNC Symbol;Acc:HGNC:4687] | 27386 | -0.066 | -0.0726 | No |
| 102 | PDE7B | phosphodiesterase 7B [Source:HGNC Symbol;Acc:HGNC:8792] | 27399 | -0.066 | -0.0700 | No |
| 103 | PNP | purine nucleoside phosphorylase [Source:HGNC Symbol;Acc:HGNC:7892] | 27422 | -0.067 | -0.0677 | No |
| 104 | POLR1B | RNA polymerase I subunit B [Source:HGNC Symbol;Acc:HGNC:20454] | 27448 | -0.067 | -0.0654 | No |
| 105 | POLR3F | RNA polymerase III subunit F [Source:HGNC Symbol;Acc:HGNC:15763] | 27481 | -0.067 | -0.0633 | No |
| 106 | ADCY8 | adenylate cyclase 8 [Source:HGNC Symbol;Acc:HGNC:239] | 27849 | -0.070 | -0.0697 | No |
| 107 | CANT1 | calcium activated nucleotidase 1 [Source:HGNC Symbol;Acc:HGNC:19721] | 27949 | -0.071 | -0.0691 | No |
| 108 | GUCY2F | "guanylate cyclase 2F, retinal [Source:HGNC Symbol;Acc:HGNC:4691]" | 27983 | -0.071 | -0.0669 | No |
| 109 | PAPSS1 | 3'-phosphoadenosine 5'-phosphosulfate synthase 1 [Source:HGNC Symbol;Acc:HGNC:8603] | 28290 | -0.074 | -0.0715 | No |
| 110 | PDE7A | phosphodiesterase 7A [Source:HGNC Symbol;Acc:HGNC:8791] | 28819 | -0.078 | -0.0816 | No |
| 111 | PDE4D | phosphodiesterase 4D [Source:HGNC Symbol;Acc:HGNC:8783] | 29039 | -0.080 | -0.0837 | No |
| 112 | ADCY6 | adenylate cyclase 6 [Source:HGNC Symbol;Acc:HGNC:237] | 29658 | -0.086 | -0.0957 | No |
| 113 | PDE8A | phosphodiesterase 8A [Source:HGNC Symbol;Acc:HGNC:8793] | 30204 | -0.092 | -0.1057 | No |
| 114 | GUCY1A1 | guanylate cyclase 1 soluble subunit alpha 1 [Source:HGNC Symbol;Acc:HGNC:4685] | 30364 | -0.094 | -0.1057 | No |
| 115 | ADCY4 | adenylate cyclase 4 [Source:HGNC Symbol;Acc:HGNC:235] | 30750 | -0.098 | -0.1113 | No |
| 116 | PDE6G | phosphodiesterase 6G [Source:HGNC Symbol;Acc:HGNC:8789] | 31516 | -0.107 | -0.1262 | No |
| 117 | PDE1B | phosphodiesterase 1B [Source:HGNC Symbol;Acc:HGNC:8775] | 31673 | -0.109 | -0.1254 | No |
| 118 | ENTPD5 | ectonucleoside triphosphate diphosphohydrolase 5 (inactive) [Source:HGNC Symbol;Acc:HGNC:3367] | 31824 | -0.111 | -0.1245 | No |
| 119 | NT5C1B | "5'-nucleotidase, cytosolic IB [Source:HGNC Symbol;Acc:HGNC:17818]" | 31838 | -0.111 | -0.1200 | No |
| 120 | ALLC | allantoicase [Source:HGNC Symbol;Acc:HGNC:17377] | 32141 | -0.115 | -0.1227 | No |
| 121 | POLR1A | RNA polymerase I subunit A [Source:HGNC Symbol;Acc:HGNC:17264] | 32355 | -0.118 | -0.1230 | No |
| 122 | GDA | guanine deaminase [Source:HGNC Symbol;Acc:HGNC:4212] | 32379 | -0.118 | -0.1185 | No |
| 123 | GUCY2C | guanylate cyclase 2C [Source:HGNC Symbol;Acc:HGNC:4688] | 32909 | -0.126 | -0.1265 | No |
| 124 | POLR2B | RNA polymerase II subunit B [Source:HGNC Symbol;Acc:HGNC:9188] | 32968 | -0.127 | -0.1225 | No |
| 125 | PDE1C | phosphodiesterase 1C [Source:HGNC Symbol;Acc:HGNC:8776] | 33206 | -0.131 | -0.1228 | No |
| 126 | PRIM1 | DNA primase subunit 1 [Source:HGNC Symbol;Acc:HGNC:9369] | 33352 | -0.134 | -0.1207 | No |
| 127 | RRM2 | ribonucleotide reductase regulatory subunit M2 [Source:HGNC Symbol;Acc:HGNC:10452] | 33676 | -0.139 | -0.1229 | No |
| 128 | PDE2A | phosphodiesterase 2A [Source:HGNC Symbol;Acc:HGNC:8777] | 33701 | -0.140 | -0.1175 | No |
| 129 | POLD3 | "DNA polymerase delta 3, accessory subunit [Source:HGNC Symbol;Acc:HGNC:20932]" | 33712 | -0.140 | -0.1117 | No |
| 130 | GMPS | guanine monophosphate synthase [Source:HGNC Symbol;Acc:HGNC:4378] | 33774 | -0.141 | -0.1071 | No |
| 131 | PAPSS2 | 3'-phosphoadenosine 5'-phosphosulfate synthase 2 [Source:HGNC Symbol;Acc:HGNC:8604] | 33817 | -0.142 | -0.1020 | No |
| 132 | PDE11A | phosphodiesterase 11A [Source:HGNC Symbol;Acc:HGNC:8773] | 33863 | -0.143 | -0.0970 | No |
| 133 | NT5C2 | "5'-nucleotidase, cytosolic II [Source:HGNC Symbol;Acc:HGNC:8022]" | 33868 | -0.143 | -0.0909 | No |
| 134 | PDE6A | phosphodiesterase 6A [Source:HGNC Symbol;Acc:HGNC:8785] | 33931 | -0.144 | -0.0862 | No |
| 135 | RRM1 | ribonucleotide reductase catalytic subunit M1 [Source:HGNC Symbol;Acc:HGNC:10451] | 33985 | -0.145 | -0.0813 | No |
| 136 | PDE3A | phosphodiesterase 3A [Source:HGNC Symbol;Acc:HGNC:8778] | 34023 | -0.146 | -0.0759 | No |
| 137 | POLR3B | RNA polymerase III subunit B [Source:HGNC Symbol;Acc:HGNC:30348] | 34273 | -0.150 | -0.0757 | No |
| 138 | ADA | adenosine deaminase [Source:HGNC Symbol;Acc:HGNC:186] | 34279 | -0.151 | -0.0693 | No |
| 139 | POLR2A | RNA polymerase II subunit A [Source:HGNC Symbol;Acc:HGNC:9187] | 34410 | -0.153 | -0.0660 | No |
| 140 | POLR3D | RNA polymerase III subunit D [Source:HGNC Symbol;Acc:HGNC:1080] | 34776 | -0.162 | -0.0683 | No |
| 141 | ENPP1 | ectonucleotide pyrophosphatase/phosphodiesterase 1 [Source:HGNC Symbol;Acc:HGNC:3356] | 35202 | -0.171 | -0.0717 | No |
| 142 | POLR3A | RNA polymerase III subunit A [Source:HGNC Symbol;Acc:HGNC:30074] | 35364 | -0.176 | -0.0682 | No |
| 143 | POLE | "DNA polymerase epsilon, catalytic subunit [Source:HGNC Symbol;Acc:HGNC:9177]" | 35511 | -0.179 | -0.0641 | No |
| 144 | ADCY5 | adenylate cyclase 5 [Source:HGNC Symbol;Acc:HGNC:236] | 35664 | -0.184 | -0.0600 | No |
| 145 | DCK | deoxycytidine kinase [Source:HGNC Symbol;Acc:HGNC:2704] | 35927 | -0.191 | -0.0584 | No |
| 146 | NPR2 | natriuretic peptide receptor 2 [Source:HGNC Symbol;Acc:HGNC:7944] | 35942 | -0.192 | -0.0505 | No |
| 147 | AK4 | adenylate kinase 4 [Source:HGNC Symbol;Acc:HGNC:363] | 35985 | -0.193 | -0.0431 | No |
| 148 | ADCY7 | adenylate cyclase 7 [Source:HGNC Symbol;Acc:HGNC:238] | 36103 | -0.197 | -0.0376 | No |
| 149 | ENTPD1 | ectonucleoside triphosphate diphosphohydrolase 1 [Source:HGNC Symbol;Acc:HGNC:3363] | 36135 | -0.198 | -0.0298 | No |
| 150 | ADCY3 | adenylate cyclase 3 [Source:HGNC Symbol;Acc:HGNC:234] | 36213 | -0.201 | -0.0230 | No |
| 151 | AMPD1 | adenosine monophosphate deaminase 1 [Source:HGNC Symbol;Acc:HGNC:468] | 36476 | -0.210 | -0.0206 | No |
| 152 | GUCY1A2 | guanylate cyclase 1 soluble subunit alpha 2 [Source:HGNC Symbol;Acc:HGNC:4684] | 36747 | -0.222 | -0.0179 | No |
| 153 | PDE10A | phosphodiesterase 10A [Source:HGNC Symbol;Acc:HGNC:8772] | 37017 | -0.237 | -0.0144 | No |
| 154 | POLA1 | "DNA polymerase alpha 1, catalytic subunit [Source:HGNC Symbol;Acc:HGNC:9173]" | 37136 | -0.244 | -0.0069 | No |
| 155 | PDE4B | phosphodiesterase 4B [Source:HGNC Symbol;Acc:HGNC:8781] | 37438 | -0.262 | -0.0032 | No |
| 156 | AMPD3 | adenosine monophosphate deaminase 3 [Source:HGNC Symbol;Acc:HGNC:470] | 37572 | -0.272 | 0.0052 | No |
| 157 | PDE5A | phosphodiesterase 5A [Source:HGNC Symbol;Acc:HGNC:8784] | 37690 | -0.283 | 0.0145 | No |
| 158 | PDE3B | phosphodiesterase 3B [Source:HGNC Symbol;Acc:HGNC:8779] | 37970 | -0.312 | 0.0209 | No |
| 159 | ENTPD4 | ectonucleoside triphosphate diphosphohydrolase 4 [Source:HGNC Symbol;Acc:HGNC:14573] | 37980 | -0.314 | 0.0343 | No |
Table: GSEA details [plain text format]

  

Fig 2: KEGG\_PURINE\_METABOLISM      
 Blue-Pink O' Gram in the Space of the Analyzed GeneSet

  

Fig 3: KEGG\_PURINE\_METABOLISM: Random ES distribution      
 Gene set null distribution of ES for **KEGG\_PURINE\_METABOLISM**

  
