# Supplementary material for: Simultaneous Presentation of Multiple Myeloma and Lung Cancer: Case Report and Gene Bioinformatics Analysis
Source: Front Oncol. 2022 Jun 13;12:859735. doi: 10.3389/fonc.2022.859735 (PMC9235397; doi:10.3389/fonc.2022.859735)
Supplement: Supplementary file 1 [file DataSheet_1.zip › The bioinformatic analysis of MM and lung cancer supplementary materials/Enrichment analysis/MECR/GSEA_4.1.0/LUAD TCGA/KEGG.Gsea.1639041756227/KEGG_PYRIMIDINE_METABOLISM.html]

Details for gene set KEGG\_PYRIMIDINE\_METABOLISM[GSEA]

|  || Dataset | ExpData\_collapsed\_to\_symbols.ENSG00000116353\_profile\_in\_ExpData.cls #ENSG00000116353 |
| Phenotype | ENSG00000116353\_profile\_in\_ExpData.cls#ENSG00000116353 |
| Upregulated in class | ENSG00000116353\_pos |
| GeneSet | KEGG\_PYRIMIDINE\_METABOLISM |
| Enrichment Score (ES) | 0.49116325 |
| Normalized Enrichment Score (NES) | 2.0165808 |
| Nominal p-value | 0.0 |
| FDR q-value | 0.0011663132 |
| FWER p-Value | 0.014 |
Table: GSEA Results Summary

  

Fig 1: Enrichment plot: KEGG\_PYRIMIDINE\_METABOLISM      
 Profile of the Running ES Score & Positions of GeneSet Members on the Rank Ordered List

  

| SYMBOL | TITLE | RANK IN GENE LIST | RANK METRIC SCORE | RUNNING ES | CORE ENRICHMENT || 1 | NME3 | NME/NM23 nucleoside diphosphate kinase 3 [Source:HGNC Symbol;Acc:HGNC:7851] | 35 | 0.444 | 0.0294 | Yes |
| 2 | POLR2L | "RNA polymerase II, I and III subunit L [Source:HGNC Symbol;Acc:HGNC:9199]" | 117 | 0.396 | 0.0544 | Yes |
| 3 | NME4 | NME/NM23 nucleoside diphosphate kinase 4 [Source:HGNC Symbol;Acc:HGNC:7852] | 216 | 0.370 | 0.0771 | Yes |
| 4 | POLD2 | "DNA polymerase delta 2, accessory subunit [Source:HGNC Symbol;Acc:HGNC:9176]" | 355 | 0.340 | 0.0968 | Yes |
| 5 | NT5C | "5', 3'-nucleotidase, cytosolic [Source:HGNC Symbol;Acc:HGNC:17144]" | 408 | 0.332 | 0.1182 | Yes |
| 6 | POLR1H | RNA polymerase I subunit H [Source:HGNC Symbol;Acc:HGNC:13182] | 575 | 0.310 | 0.1351 | Yes |
| 7 | POLR2E | "RNA polymerase II, I and III subunit E [Source:HGNC Symbol;Acc:HGNC:9192]" | 607 | 0.307 | 0.1552 | Yes |
| 8 | TXNRD2 | thioredoxin reductase 2 [Source:HGNC Symbol;Acc:HGNC:18155] | 630 | 0.305 | 0.1755 | Yes |
| 9 | ITPA | inosine triphosphatase [Source:HGNC Symbol;Acc:HGNC:6176] | 640 | 0.304 | 0.1960 | Yes |
| 10 | POLR2J | RNA polymerase II subunit J [Source:HGNC Symbol;Acc:HGNC:9197] | 736 | 0.294 | 0.2136 | Yes |
| 11 | POLR1C | RNA polymerase I and III subunit C [Source:HGNC Symbol;Acc:HGNC:20194] | 749 | 0.293 | 0.2333 | Yes |
| 12 | POLR3H | RNA polymerase III subunit H [Source:HGNC Symbol;Acc:HGNC:30349] | 797 | 0.288 | 0.2518 | Yes |
| 13 | POLR2I | RNA polymerase II subunit I [Source:HGNC Symbol;Acc:HGNC:9196] | 813 | 0.287 | 0.2710 | Yes |
| 14 | NT5M | "5',3'-nucleotidase, mitochondrial [Source:HGNC Symbol;Acc:HGNC:15769]" | 821 | 0.286 | 0.2903 | Yes |
| 15 | UCK1 | uridine-cytidine kinase 1 [Source:HGNC Symbol;Acc:HGNC:14859] | 930 | 0.276 | 0.3064 | Yes |
| 16 | POLR2G | RNA polymerase II subunit G [Source:HGNC Symbol;Acc:HGNC:9194] | 999 | 0.270 | 0.3231 | Yes |
| 17 | DHODH | dihydroorotate dehydrogenase (quinone) [Source:HGNC Symbol;Acc:HGNC:2867] | 1050 | 0.266 | 0.3400 | Yes |
| 18 | POLR2H | "RNA polymerase II, I and III subunit H [Source:HGNC Symbol;Acc:HGNC:9195]" | 1077 | 0.264 | 0.3573 | Yes |
| 19 | POLE4 | "DNA polymerase epsilon 4, accessory subunit [Source:HGNC Symbol;Acc:HGNC:18755]" | 1121 | 0.262 | 0.3741 | Yes |
| 20 | NME2 | NME/NM23 nucleoside diphosphate kinase 2 [Source:HGNC Symbol;Acc:HGNC:7850] | 1309 | 0.247 | 0.3862 | Yes |
| 21 | POLR3C | RNA polymerase III subunit C [Source:HGNC Symbol;Acc:HGNC:30076] | 1448 | 0.238 | 0.3989 | Yes |
| 22 | NME1 | NME/NM23 nucleoside diphosphate kinase 1 [Source:HGNC Symbol;Acc:HGNC:7849] | 1565 | 0.231 | 0.4117 | Yes |
| 23 | POLR3GL | RNA polymerase III subunit GL [Source:HGNC Symbol;Acc:HGNC:28466] | 2080 | 0.202 | 0.4124 | Yes |
| 24 | POLR3K | RNA polymerase III subunit K [Source:HGNC Symbol;Acc:HGNC:14121] | 2176 | 0.198 | 0.4235 | Yes |
| 25 | NME1-NME2 | NME1-NME2 readthrough [Source:HGNC Symbol;Acc:HGNC:33531] | 2299 | 0.193 | 0.4335 | Yes |
| 26 | DTYMK | deoxythymidylate kinase [Source:HGNC Symbol;Acc:HGNC:3061] | 2337 | 0.191 | 0.4456 | Yes |
| 27 | NME6 | NME/NM23 nucleoside diphosphate kinase 6 [Source:HGNC Symbol;Acc:HGNC:20567] | 2407 | 0.188 | 0.4567 | Yes |
| 28 | POLR1D | RNA polymerase I and III subunit D [Source:HGNC Symbol;Acc:HGNC:20422] | 2640 | 0.179 | 0.4630 | Yes |
| 29 | POLD4 | "DNA polymerase delta 4, accessory subunit [Source:HGNC Symbol;Acc:HGNC:14106]" | 3360 | 0.153 | 0.4551 | Yes |
| 30 | UCKL1 | uridine-cytidine kinase 1 like 1 [Source:HGNC Symbol;Acc:HGNC:15938] | 3594 | 0.146 | 0.4591 | Yes |
| 31 | UPP2 | uridine phosphorylase 2 [Source:HGNC Symbol;Acc:HGNC:23061] | 4139 | 0.130 | 0.4541 | Yes |
| 32 | DUT | deoxyuridine triphosphatase [Source:HGNC Symbol;Acc:HGNC:3078] | 4149 | 0.130 | 0.4627 | Yes |
| 33 | DCTD | dCMP deaminase [Source:HGNC Symbol;Acc:HGNC:2710] | 4319 | 0.126 | 0.4670 | Yes |
| 34 | POLR2K | "RNA polymerase II, I and III subunit K [Source:HGNC Symbol;Acc:HGNC:9198]" | 4390 | 0.124 | 0.4737 | Yes |
| 35 | UPP1 | uridine phosphorylase 1 [Source:HGNC Symbol;Acc:HGNC:12576] | 5140 | 0.109 | 0.4620 | Yes |
| 36 | POLR2C | RNA polymerase II subunit C [Source:HGNC Symbol;Acc:HGNC:9189] | 5263 | 0.106 | 0.4662 | Yes |
| 37 | NME7 | NME/NM23 family member 7 [Source:HGNC Symbol;Acc:HGNC:20461] | 5422 | 0.103 | 0.4692 | Yes |
| 38 | POLR2F | "RNA polymerase II, I and III subunit F [Source:HGNC Symbol;Acc:HGNC:9193]" | 5558 | 0.101 | 0.4726 | Yes |
| 39 | TK1 | thymidine kinase 1 [Source:HGNC Symbol;Acc:HGNC:11830] | 5851 | 0.096 | 0.4718 | Yes |
| 40 | CDA | cytidine deaminase [Source:HGNC Symbol;Acc:HGNC:1712] | 6151 | 0.091 | 0.4704 | Yes |
| 41 | POLR1E | RNA polymerase I subunit E [Source:HGNC Symbol;Acc:HGNC:17631] | 6313 | 0.089 | 0.4723 | Yes |
| 42 | POLD1 | "DNA polymerase delta 1, catalytic subunit [Source:HGNC Symbol;Acc:HGNC:9175]" | 6464 | 0.087 | 0.4744 | Yes |
| 43 | CMPK1 | cytidine/uridine monophosphate kinase 1 [Source:HGNC Symbol;Acc:HGNC:18170] | 6511 | 0.086 | 0.4791 | Yes |
| 44 | ENTPD3 | ectonucleoside triphosphate diphosphohydrolase 3 [Source:HGNC Symbol;Acc:HGNC:3365] | 6543 | 0.085 | 0.4841 | Yes |
| 45 | POLR2J3 | RNA polymerase II subunit J3 [Source:HGNC Symbol;Acc:HGNC:33853] | 6597 | 0.085 | 0.4885 | Yes |
| 46 | ENTPD6 | ectonucleoside triphosphate diphosphohydrolase 6 [Source:HGNC Symbol;Acc:HGNC:3368] | 6762 | 0.083 | 0.4900 | Yes |
| 47 | POLE3 | "DNA polymerase epsilon 3, accessory subunit [Source:HGNC Symbol;Acc:HGNC:13546]" | 6932 | 0.080 | 0.4912 | Yes |
| 48 | UMPS | uridine monophosphate synthetase [Source:HGNC Symbol;Acc:HGNC:12563] | 7321 | 0.075 | 0.4864 | No |
| 49 | PRIM2 | DNA primase subunit 2 [Source:HGNC Symbol;Acc:HGNC:9370] | 7571 | 0.072 | 0.4850 | No |
| 50 | CTPS1 | CTP synthase 1 [Source:HGNC Symbol;Acc:HGNC:2519] | 7636 | 0.071 | 0.4882 | No |
| 51 | UPRT | uracil phosphoribosyltransferase homolog [Source:HGNC Symbol;Acc:HGNC:28334] | 8601 | 0.061 | 0.4678 | No |
| 52 | NME5 | NME/NM23 family member 5 [Source:HGNC Symbol;Acc:HGNC:7853] | 8714 | 0.060 | 0.4691 | No |
| 53 | NT5C1A | "5'-nucleotidase, cytosolic IA [Source:HGNC Symbol;Acc:HGNC:17819]" | 8730 | 0.060 | 0.4728 | No |
| 54 | ENTPD8 | ectonucleoside triphosphate diphosphohydrolase 8 [Source:HGNC Symbol;Acc:HGNC:24860] | 9095 | 0.057 | 0.4674 | No |
| 55 | NT5E | 5'-nucleotidase ecto [Source:HGNC Symbol;Acc:HGNC:8021] | 10027 | 0.049 | 0.4470 | No |
| 56 | POLR2J2 | RNA polymerase II subunit J2 [Source:HGNC Symbol;Acc:HGNC:23208] | 10680 | 0.044 | 0.4333 | No |
| 57 | POLR2D | RNA polymerase II subunit D [Source:HGNC Symbol;Acc:HGNC:9191] | 14063 | 0.019 | 0.3484 | No |
| 58 | NUDT2 | nudix hydrolase 2 [Source:HGNC Symbol;Acc:HGNC:8049] | 14585 | 0.016 | 0.3362 | No |
| 59 | TK2 | thymidine kinase 2 [Source:HGNC Symbol;Acc:HGNC:11831] | 16987 | 0.001 | 0.2751 | No |
| 60 | RRM2B | ribonucleotide reductase regulatory TP53 inducible subunit M2B [Source:HGNC Symbol;Acc:HGNC:17296] | 19336 | -0.013 | 0.2161 | No |
| 61 | CMPK2 | cytidine/uridine monophosphate kinase 2 [Source:HGNC Symbol;Acc:HGNC:27015] | 19643 | -0.014 | 0.2093 | No |
| 62 | TYMP | thymidine phosphorylase [Source:HGNC Symbol;Acc:HGNC:3148] | 20378 | -0.019 | 0.1918 | No |
| 63 | POLA2 | "DNA polymerase alpha 2, accessory subunit [Source:HGNC Symbol;Acc:HGNC:30073]" | 21468 | -0.026 | 0.1658 | No |
| 64 | POLR3G | RNA polymerase III subunit G [Source:HGNC Symbol;Acc:HGNC:30075] | 21988 | -0.029 | 0.1546 | No |
| 65 | PNPT1 | polyribonucleotide nucleotidyltransferase 1 [Source:HGNC Symbol;Acc:HGNC:23166] | 22014 | -0.029 | 0.1559 | No |
| 66 | NT5C3A | "5'-nucleotidase, cytosolic IIIA [Source:HGNC Symbol;Acc:HGNC:17820]" | 23996 | -0.042 | 0.1083 | No |
| 67 | CAD | "carbamoyl-phosphate synthetase 2, aspartate transcarbamylase, and dihydroorotase [Source:HGNC Symbol;Acc:HGNC:1424]" | 26439 | -0.059 | 0.0500 | No |
| 68 | CTPS2 | CTP synthase 2 [Source:HGNC Symbol;Acc:HGNC:2520] | 26825 | -0.062 | 0.0444 | No |
| 69 | TYMS | thymidylate synthetase [Source:HGNC Symbol;Acc:HGNC:12441] | 26993 | -0.063 | 0.0445 | No |
| 70 | POLE2 | "DNA polymerase epsilon 2, accessory subunit [Source:HGNC Symbol;Acc:HGNC:9178]" | 27164 | -0.065 | 0.0446 | No |
| 71 | PNP | purine nucleoside phosphorylase [Source:HGNC Symbol;Acc:HGNC:7892] | 27422 | -0.067 | 0.0426 | No |
| 72 | POLR1B | RNA polymerase I subunit B [Source:HGNC Symbol;Acc:HGNC:20454] | 27448 | -0.067 | 0.0465 | No |
| 73 | POLR3F | RNA polymerase III subunit F [Source:HGNC Symbol;Acc:HGNC:15763] | 27481 | -0.067 | 0.0502 | No |
| 74 | CANT1 | calcium activated nucleotidase 1 [Source:HGNC Symbol;Acc:HGNC:19721] | 27949 | -0.071 | 0.0431 | No |
| 75 | AK3 | adenylate kinase 3 [Source:HGNC Symbol;Acc:HGNC:17376] | 29564 | -0.085 | 0.0078 | No |
| 76 | DPYS | dihydropyrimidinase [Source:HGNC Symbol;Acc:HGNC:3013] | 29749 | -0.087 | 0.0091 | No |
| 77 | UPB1 | beta-ureidopropionase 1 [Source:HGNC Symbol;Acc:HGNC:16297] | 29902 | -0.089 | 0.0113 | No |
| 78 | TXNRD1 | thioredoxin reductase 1 [Source:HGNC Symbol;Acc:HGNC:12437] | 31523 | -0.107 | -0.0228 | No |
| 79 | ENTPD5 | ectonucleoside triphosphate diphosphohydrolase 5 (inactive) [Source:HGNC Symbol;Acc:HGNC:3367] | 31824 | -0.111 | -0.0229 | No |
| 80 | NT5C1B | "5'-nucleotidase, cytosolic IB [Source:HGNC Symbol;Acc:HGNC:17818]" | 31838 | -0.111 | -0.0156 | No |
| 81 | POLR1A | RNA polymerase I subunit A [Source:HGNC Symbol;Acc:HGNC:17264] | 32355 | -0.118 | -0.0207 | No |
| 82 | DPYD | dihydropyrimidine dehydrogenase [Source:HGNC Symbol;Acc:HGNC:3012] | 32915 | -0.127 | -0.0264 | No |
| 83 | POLR2B | RNA polymerase II subunit B [Source:HGNC Symbol;Acc:HGNC:9188] | 32968 | -0.127 | -0.0190 | No |
| 84 | PRIM1 | DNA primase subunit 1 [Source:HGNC Symbol;Acc:HGNC:9369] | 33352 | -0.134 | -0.0196 | No |
| 85 | RRM2 | ribonucleotide reductase regulatory subunit M2 [Source:HGNC Symbol;Acc:HGNC:10452] | 33676 | -0.139 | -0.0184 | No |
| 86 | POLD3 | "DNA polymerase delta 3, accessory subunit [Source:HGNC Symbol;Acc:HGNC:20932]" | 33712 | -0.140 | -0.0097 | No |
| 87 | NT5C2 | "5'-nucleotidase, cytosolic II [Source:HGNC Symbol;Acc:HGNC:8022]" | 33868 | -0.143 | -0.0039 | No |
| 88 | RRM1 | ribonucleotide reductase catalytic subunit M1 [Source:HGNC Symbol;Acc:HGNC:10451] | 33985 | -0.145 | 0.0030 | No |
| 89 | POLR3B | RNA polymerase III subunit B [Source:HGNC Symbol;Acc:HGNC:30348] | 34273 | -0.150 | 0.0060 | No |
| 90 | POLR2A | RNA polymerase II subunit A [Source:HGNC Symbol;Acc:HGNC:9187] | 34410 | -0.153 | 0.0130 | No |
| 91 | POLR3D | RNA polymerase III subunit D [Source:HGNC Symbol;Acc:HGNC:1080] | 34776 | -0.162 | 0.0147 | No |
| 92 | POLR3A | RNA polymerase III subunit A [Source:HGNC Symbol;Acc:HGNC:30074] | 35364 | -0.176 | 0.0117 | No |
| 93 | UCK2 | uridine-cytidine kinase 2 [Source:HGNC Symbol;Acc:HGNC:12562] | 35445 | -0.178 | 0.0218 | No |
| 94 | POLE | "DNA polymerase epsilon, catalytic subunit [Source:HGNC Symbol;Acc:HGNC:9177]" | 35511 | -0.179 | 0.0324 | No |
| 95 | DCK | deoxycytidine kinase [Source:HGNC Symbol;Acc:HGNC:2704] | 35927 | -0.191 | 0.0349 | No |
| 96 | ENTPD1 | ectonucleoside triphosphate diphosphohydrolase 1 [Source:HGNC Symbol;Acc:HGNC:3363] | 36135 | -0.198 | 0.0431 | No |
| 97 | POLA1 | "DNA polymerase alpha 1, catalytic subunit [Source:HGNC Symbol;Acc:HGNC:9173]" | 37136 | -0.244 | 0.0343 | No |
| 98 | ENTPD4 | ectonucleoside triphosphate diphosphohydrolase 4 [Source:HGNC Symbol;Acc:HGNC:14573] | 37980 | -0.314 | 0.0342 | No |
Table: GSEA details [plain text format]

  

Fig 2: KEGG\_PYRIMIDINE\_METABOLISM      
 Blue-Pink O' Gram in the Space of the Analyzed GeneSet

  

Fig 3: KEGG\_PYRIMIDINE\_METABOLISM: Random ES distribution      
 Gene set null distribution of ES for **KEGG\_PYRIMIDINE\_METABOLISM**

  
