# Supplementary material for: Simultaneous Presentation of Multiple Myeloma and Lung Cancer: Case Report and Gene Bioinformatics Analysis
Source: Front Oncol. 2022 Jun 13;12:859735. doi: 10.3389/fonc.2022.859735 (PMC9235397; doi:10.3389/fonc.2022.859735)
Supplement: Supplementary file 1 [file DataSheet_1.zip › The bioinformatic analysis of MM and lung cancer supplementary materials/Enrichment analysis/MECR/GSEA_4.1.0/LUAD TCGA/KEGG.Gsea.1639041756227/KEGG_PYRUVATE_METABOLISM.html]

Details for gene set KEGG\_PYRUVATE\_METABOLISM[GSEA]

|  || Dataset | ExpData\_collapsed\_to\_symbols.ENSG00000116353\_profile\_in\_ExpData.cls #ENSG00000116353 |
| Phenotype | ENSG00000116353\_profile\_in\_ExpData.cls#ENSG00000116353 |
| Upregulated in class | ENSG00000116353\_pos |
| GeneSet | KEGG\_PYRUVATE\_METABOLISM |
| Enrichment Score (ES) | 0.5403186 |
| Normalized Enrichment Score (NES) | 1.8733666 |
| Nominal p-value | 0.0 |
| FDR q-value | 0.004225574 |
| FWER p-Value | 0.066 |
Table: GSEA Results Summary

  

Fig 1: Enrichment plot: KEGG\_PYRUVATE\_METABOLISM      
 Profile of the Running ES Score & Positions of GeneSet Members on the Rank Ordered List

  

| SYMBOL | TITLE | RANK IN GENE LIST | RANK METRIC SCORE | RUNNING ES | CORE ENRICHMENT || 1 | HAGH | hydroxyacylglutathione hydrolase [Source:HGNC Symbol;Acc:HGNC:4805] | 464 | 0.324 | 0.0589 | Yes |
| 2 | ACYP2 | acylphosphatase 2 [Source:HGNC Symbol;Acc:HGNC:180] | 855 | 0.283 | 0.1106 | Yes |
| 3 | HAGHL | hydroxyacylglutathione hydrolase like [Source:HGNC Symbol;Acc:HGNC:14177] | 887 | 0.280 | 0.1709 | Yes |
| 4 | MDH2 | malate dehydrogenase 2 [Source:HGNC Symbol;Acc:HGNC:6971] | 1614 | 0.227 | 0.2020 | Yes |
| 5 | PC | pyruvate carboxylase [Source:HGNC Symbol;Acc:HGNC:8636] | 1784 | 0.218 | 0.2452 | Yes |
| 6 | ALDH7A1 | aldehyde dehydrogenase 7 family member A1 [Source:HGNC Symbol;Acc:HGNC:877] | 2018 | 0.205 | 0.2840 | Yes |
| 7 | LDHD | lactate dehydrogenase D [Source:HGNC Symbol;Acc:HGNC:19708] | 2647 | 0.178 | 0.3069 | Yes |
| 8 | GRHPR | glyoxylate and hydroxypyruvate reductase [Source:HGNC Symbol;Acc:HGNC:4570] | 2891 | 0.168 | 0.3374 | Yes |
| 9 | GLO1 | glyoxalase I [Source:HGNC Symbol;Acc:HGNC:4323] | 3008 | 0.164 | 0.3701 | Yes |
| 10 | PDHB | pyruvate dehydrogenase E1 subunit beta [Source:HGNC Symbol;Acc:HGNC:8808] | 3452 | 0.150 | 0.3915 | Yes |
| 11 | MDH1 | malate dehydrogenase 1 [Source:HGNC Symbol;Acc:HGNC:6970] | 3847 | 0.138 | 0.4116 | Yes |
| 12 | PKM | pyruvate kinase M1/2 [Source:HGNC Symbol;Acc:HGNC:9021] | 4157 | 0.130 | 0.4320 | Yes |
| 13 | ME3 | malic enzyme 3 [Source:HGNC Symbol;Acc:HGNC:6985] | 4539 | 0.121 | 0.4486 | Yes |
| 14 | PCK2 | "phosphoenolpyruvate carboxykinase 2, mitochondrial [Source:HGNC Symbol;Acc:HGNC:8725]" | 4563 | 0.120 | 0.4742 | Yes |
| 15 | ALDH2 | aldehyde dehydrogenase 2 family member [Source:HGNC Symbol;Acc:HGNC:404] | 4910 | 0.113 | 0.4900 | Yes |
| 16 | ALDH3A2 | aldehyde dehydrogenase 3 family member A2 [Source:HGNC Symbol;Acc:HGNC:403] | 5202 | 0.108 | 0.5060 | Yes |
| 17 | PDHA1 | pyruvate dehydrogenase E1 subunit alpha 1 [Source:HGNC Symbol;Acc:HGNC:8806] | 5654 | 0.099 | 0.5162 | Yes |
| 18 | ACSS2 | acyl-CoA synthetase short chain family member 2 [Source:HGNC Symbol;Acc:HGNC:15814] | 6408 | 0.087 | 0.5160 | Yes |
| 19 | ACAT1 | acetyl-CoA acetyltransferase 1 [Source:HGNC Symbol;Acc:HGNC:93] | 6430 | 0.087 | 0.5344 | Yes |
| 20 | ACSS1 | acyl-CoA synthetase short chain family member 1 [Source:HGNC Symbol;Acc:HGNC:16091] | 7679 | 0.071 | 0.5181 | Yes |
| 21 | ALDH9A1 | aldehyde dehydrogenase 9 family member A1 [Source:HGNC Symbol;Acc:HGNC:412] | 7785 | 0.070 | 0.5306 | Yes |
| 22 | LDHC | lactate dehydrogenase C [Source:HGNC Symbol;Acc:HGNC:6544] | 7982 | 0.068 | 0.5403 | Yes |
| 23 | PKLR | pyruvate kinase L/R [Source:HGNC Symbol;Acc:HGNC:9020] | 14801 | 0.014 | 0.3699 | No |
| 24 | LDHA | lactate dehydrogenase A [Source:HGNC Symbol;Acc:HGNC:6535] | 15849 | 0.008 | 0.3449 | No |
| 25 | ACYP1 | acylphosphatase 1 [Source:HGNC Symbol;Acc:HGNC:179] | 16831 | 0.002 | 0.3205 | No |
| 26 | ACAT2 | acetyl-CoA acetyltransferase 2 [Source:HGNC Symbol;Acc:HGNC:94] | 19909 | -0.016 | 0.2457 | No |
| 27 | PDHA2 | pyruvate dehydrogenase E1 subunit alpha 2 [Source:HGNC Symbol;Acc:HGNC:8807] | 20512 | -0.020 | 0.2347 | No |
| 28 | ALDH1B1 | aldehyde dehydrogenase 1 family member B1 [Source:HGNC Symbol;Acc:HGNC:407] | 21379 | -0.025 | 0.2181 | No |
| 29 | DLD | dihydrolipoamide dehydrogenase [Source:HGNC Symbol;Acc:HGNC:2898] | 25309 | -0.051 | 0.1292 | No |
| 30 | ACACA | acetyl-CoA carboxylase alpha [Source:HGNC Symbol;Acc:HGNC:84] | 25618 | -0.053 | 0.1329 | No |
| 31 | DLAT | dihydrolipoamide S-acetyltransferase [Source:HGNC Symbol;Acc:HGNC:2896] | 25670 | -0.053 | 0.1432 | No |
| 32 | ME1 | malic enzyme 1 [Source:HGNC Symbol;Acc:HGNC:6983] | 26052 | -0.056 | 0.1458 | No |
| 33 | AKR1B1 | aldo-keto reductase family 1 member B [Source:HGNC Symbol;Acc:HGNC:381] | 26764 | -0.061 | 0.1411 | No |
| 34 | ME2 | malic enzyme 2 [Source:HGNC Symbol;Acc:HGNC:6984] | 28103 | -0.072 | 0.1227 | No |
| 35 | LDHB | lactate dehydrogenase B [Source:HGNC Symbol;Acc:HGNC:6541] | 29281 | -0.082 | 0.1107 | No |
| 36 | ACOT12 | acyl-CoA thioesterase 12 [Source:HGNC Symbol;Acc:HGNC:24436] | 29892 | -0.088 | 0.1145 | No |
| 37 | LDHAL6B | lactate dehydrogenase A like 6B [Source:HGNC Symbol;Acc:HGNC:21481] | 31434 | -0.106 | 0.0983 | No |
| 38 | PCK1 | phosphoenolpyruvate carboxykinase 1 [Source:HGNC Symbol;Acc:HGNC:8724] | 32207 | -0.116 | 0.1039 | No |
| 39 | ACACB | acetyl-CoA carboxylase beta [Source:HGNC Symbol;Acc:HGNC:85] | 34330 | -0.151 | 0.0829 | No |
| 40 | LDHAL6A | lactate dehydrogenase A like 6A [Source:HGNC Symbol;Acc:HGNC:28335] | 36267 | -0.203 | 0.0778 | No |
Table: GSEA details [plain text format]

  

Fig 2: KEGG\_PYRUVATE\_METABOLISM      
 Blue-Pink O' Gram in the Space of the Analyzed GeneSet

  

Fig 3: KEGG\_PYRUVATE\_METABOLISM: Random ES distribution      
 Gene set null distribution of ES for **KEGG\_PYRUVATE\_METABOLISM**

  
