# Supplementary material for: Simultaneous Presentation of Multiple Myeloma and Lung Cancer: Case Report and Gene Bioinformatics Analysis
Source: Front Oncol. 2022 Jun 13;12:859735. doi: 10.3389/fonc.2022.859735 (PMC9235397; doi:10.3389/fonc.2022.859735)
Supplement: Supplementary file 1 [file DataSheet_1.zip › The bioinformatic analysis of MM and lung cancer supplementary materials/Enrichment analysis/MECR/GSEA_4.1.0/LUAD TCGA/KEGG.Gsea.1639041756227/KEGG_RIBOSOME.html]

Details for gene set KEGG\_RIBOSOME[GSEA]

|  || Dataset | ExpData\_collapsed\_to\_symbols.ENSG00000116353\_profile\_in\_ExpData.cls #ENSG00000116353 |
| Phenotype | ENSG00000116353\_profile\_in\_ExpData.cls#ENSG00000116353 |
| Upregulated in class | ENSG00000116353\_pos |
| GeneSet | KEGG\_RIBOSOME |
| Enrichment Score (ES) | 0.8526685 |
| Normalized Enrichment Score (NES) | 3.4376059 |
| Nominal p-value | 0.0 |
| FDR q-value | 0.0 |
| FWER p-Value | 0.0 |
Table: GSEA Results Summary

  

Fig 1: Enrichment plot: KEGG\_RIBOSOME      
 Profile of the Running ES Score & Positions of GeneSet Members on the Rank Ordered List

  

| SYMBOL | TITLE | RANK IN GENE LIST | RANK METRIC SCORE | RUNNING ES | CORE ENRICHMENT || 1 | RPL11 | ribosomal protein L11 [Source:HGNC Symbol;Acc:HGNC:10301] | 89 | 0.407 | 0.0175 | Yes |
| 2 | RPS8 | ribosomal protein S8 [Source:HGNC Symbol;Acc:HGNC:10441] | 175 | 0.379 | 0.0337 | Yes |
| 3 | FAU | FAU ubiquitin like and ribosomal protein S30 fusion [Source:HGNC Symbol;Acc:HGNC:3597] | 272 | 0.355 | 0.0485 | Yes |
| 4 | RPL35 | ribosomal protein L35 [Source:HGNC Symbol;Acc:HGNC:10344] | 330 | 0.346 | 0.0639 | Yes |
| 5 | RPLP2 | ribosomal protein lateral stalk subunit P2 [Source:HGNC Symbol;Acc:HGNC:10377] | 369 | 0.337 | 0.0793 | Yes |
| 6 | RPS2 | ribosomal protein S2 [Source:HGNC Symbol;Acc:HGNC:10404] | 384 | 0.335 | 0.0952 | Yes |
| 7 | RPL8 | ribosomal protein L8 [Source:HGNC Symbol;Acc:HGNC:10368] | 410 | 0.332 | 0.1107 | Yes |
| 8 | RPL29 | ribosomal protein L29 [Source:HGNC Symbol;Acc:HGNC:10331] | 423 | 0.330 | 0.1264 | Yes |
| 9 | RPL19 | ribosomal protein L19 [Source:HGNC Symbol;Acc:HGNC:10312] | 431 | 0.328 | 0.1422 | Yes |
| 10 | RPL13 | ribosomal protein L13 [Source:HGNC Symbol;Acc:HGNC:10303] | 446 | 0.326 | 0.1577 | Yes |
| 11 | RPL36 | ribosomal protein L36 [Source:HGNC Symbol;Acc:HGNC:13631] | 452 | 0.326 | 0.1734 | Yes |
| 12 | RPL24 | ribosomal protein L24 [Source:HGNC Symbol;Acc:HGNC:10325] | 466 | 0.324 | 0.1887 | Yes |
| 13 | RPL34 | ribosomal protein L34 [Source:HGNC Symbol;Acc:HGNC:10340] | 537 | 0.315 | 0.2023 | Yes |
| 14 | RPL18A | ribosomal protein L18a [Source:HGNC Symbol;Acc:HGNC:10311] | 560 | 0.312 | 0.2168 | Yes |
| 15 | RPS15 | ribosomal protein S15 [Source:HGNC Symbol;Acc:HGNC:10388] | 591 | 0.309 | 0.2311 | Yes |
| 16 | RPL12 | ribosomal protein L12 [Source:HGNC Symbol;Acc:HGNC:10302] | 618 | 0.306 | 0.2452 | Yes |
| 17 | RPL37 | ribosomal protein L37 [Source:HGNC Symbol;Acc:HGNC:10347] | 672 | 0.300 | 0.2585 | Yes |
| 18 | RPS9 | ribosomal protein S9 [Source:HGNC Symbol;Acc:HGNC:10442] | 676 | 0.300 | 0.2729 | Yes |
| 19 | RPL10A | ribosomal protein L10a [Source:HGNC Symbol;Acc:HGNC:10299] | 725 | 0.295 | 0.2860 | Yes |
| 20 | RPL26L1 | ribosomal protein L26 like 1 [Source:HGNC Symbol;Acc:HGNC:17050] | 730 | 0.294 | 0.3002 | Yes |
| 21 | RPL37A | ribosomal protein L37a [Source:HGNC Symbol;Acc:HGNC:10348] | 731 | 0.294 | 0.3145 | Yes |
| 22 | RPS18 | ribosomal protein S18 [Source:HGNC Symbol;Acc:HGNC:10401] | 743 | 0.293 | 0.3284 | Yes |
| 23 | UBA52 | ubiquitin A-52 residue ribosomal protein fusion product 1 [Source:HGNC Symbol;Acc:HGNC:12458] | 769 | 0.291 | 0.3419 | Yes |
| 24 | RPS21 | ribosomal protein S21 [Source:HGNC Symbol;Acc:HGNC:10409] | 776 | 0.291 | 0.3559 | Yes |
| 25 | RPL27 | ribosomal protein L27 [Source:HGNC Symbol;Acc:HGNC:10328] | 795 | 0.289 | 0.3694 | Yes |
| 26 | RPS10 | ribosomal protein S10 [Source:HGNC Symbol;Acc:HGNC:10383] | 798 | 0.288 | 0.3834 | Yes |
| 27 | RPS20 | ribosomal protein S20 [Source:HGNC Symbol;Acc:HGNC:10405] | 803 | 0.288 | 0.3972 | Yes |
| 28 | RPL14 | ribosomal protein L14 [Source:HGNC Symbol;Acc:HGNC:10305] | 812 | 0.287 | 0.4110 | Yes |
| 29 | RPL3 | ribosomal protein L3 [Source:HGNC Symbol;Acc:HGNC:10332] | 820 | 0.286 | 0.4247 | Yes |
| 30 | RPL28 | ribosomal protein L28 [Source:HGNC Symbol;Acc:HGNC:10330] | 829 | 0.285 | 0.4383 | Yes |
| 31 | RPLP1 | ribosomal protein lateral stalk subunit P1 [Source:HGNC Symbol;Acc:HGNC:10372] | 831 | 0.285 | 0.4521 | Yes |
| 32 | RPL38 | ribosomal protein L38 [Source:HGNC Symbol;Acc:HGNC:10349] | 899 | 0.279 | 0.4640 | Yes |
| 33 | RPL27A | ribosomal protein L27a [Source:HGNC Symbol;Acc:HGNC:10329] | 945 | 0.274 | 0.4761 | Yes |
| 34 | RPL31 | ribosomal protein L31 [Source:HGNC Symbol;Acc:HGNC:10334] | 989 | 0.271 | 0.4882 | Yes |
| 35 | RPL32 | ribosomal protein L32 [Source:HGNC Symbol;Acc:HGNC:10336] | 1001 | 0.270 | 0.5010 | Yes |
| 36 | RPS28 | ribosomal protein S28 [Source:HGNC Symbol;Acc:HGNC:10418] | 1020 | 0.268 | 0.5136 | Yes |
| 37 | RPL22 | ribosomal protein L22 [Source:HGNC Symbol;Acc:HGNC:10315] | 1036 | 0.267 | 0.5261 | Yes |
| 38 | RPL23 | ribosomal protein L23 [Source:HGNC Symbol;Acc:HGNC:10316] | 1073 | 0.264 | 0.5381 | Yes |
| 39 | RPL9 | ribosomal protein L9 [Source:HGNC Symbol;Acc:HGNC:10369] | 1099 | 0.263 | 0.5502 | Yes |
| 40 | RPL39 | ribosomal protein L39 [Source:HGNC Symbol;Acc:HGNC:10350] | 1141 | 0.260 | 0.5618 | Yes |
| 41 | RPS15A | ribosomal protein S15a [Source:HGNC Symbol;Acc:HGNC:10389] | 1151 | 0.259 | 0.5741 | Yes |
| 42 | RPS13 | ribosomal protein S13 [Source:HGNC Symbol;Acc:HGNC:10386] | 1226 | 0.253 | 0.5845 | Yes |
| 43 | RPSA | ribosomal protein SA [Source:HGNC Symbol;Acc:HGNC:6502] | 1231 | 0.253 | 0.5967 | Yes |
| 44 | RPL15 | ribosomal protein L15 [Source:HGNC Symbol;Acc:HGNC:10306] | 1290 | 0.248 | 0.6073 | Yes |
| 45 | RPL18 | ribosomal protein L18 [Source:HGNC Symbol;Acc:HGNC:10310] | 1323 | 0.246 | 0.6184 | Yes |
| 46 | RPL35A | ribosomal protein L35a [Source:HGNC Symbol;Acc:HGNC:10345] | 1386 | 0.242 | 0.6286 | Yes |
| 47 | RPS7 | ribosomal protein S7 [Source:HGNC Symbol;Acc:HGNC:10440] | 1444 | 0.239 | 0.6387 | Yes |
| 48 | RPS25 | ribosomal protein S25 [Source:HGNC Symbol;Acc:HGNC:10413] | 1503 | 0.234 | 0.6486 | Yes |
| 49 | RPL7A | ribosomal protein L7a [Source:HGNC Symbol;Acc:HGNC:10364] | 1524 | 0.233 | 0.6595 | Yes |
| 50 | RPL5 | ribosomal protein L5 [Source:HGNC Symbol;Acc:HGNC:10360] | 1529 | 0.233 | 0.6707 | Yes |
| 51 | RPL36A | ribosomal protein L36a [Source:HGNC Symbol;Acc:HGNC:10359] | 1604 | 0.228 | 0.6799 | Yes |
| 52 | RPL3L | ribosomal protein L3 like [Source:HGNC Symbol;Acc:HGNC:10351] | 1627 | 0.227 | 0.6903 | Yes |
| 53 | RPS16 | ribosomal protein S16 [Source:HGNC Symbol;Acc:HGNC:10396] | 1631 | 0.227 | 0.7013 | Yes |
| 54 | RPL30 | ribosomal protein L30 [Source:HGNC Symbol;Acc:HGNC:10333] | 1635 | 0.226 | 0.7122 | Yes |
| 55 | RPL10 | ribosomal protein L10 [Source:HGNC Symbol;Acc:HGNC:10298] | 1660 | 0.225 | 0.7225 | Yes |
| 56 | RPS27 | ribosomal protein S27 [Source:HGNC Symbol;Acc:HGNC:10416] | 1675 | 0.224 | 0.7331 | Yes |
| 57 | RPS24 | ribosomal protein S24 [Source:HGNC Symbol;Acc:HGNC:10411] | 1697 | 0.223 | 0.7433 | Yes |
| 58 | RPS3A | ribosomal protein S3A [Source:HGNC Symbol;Acc:HGNC:10421] | 1702 | 0.223 | 0.7541 | Yes |
| 59 | RPS3 | ribosomal protein S3 [Source:HGNC Symbol;Acc:HGNC:10420] | 1803 | 0.217 | 0.7621 | Yes |
| 60 | RPS17 | ribosomal protein S17 [Source:HGNC Symbol;Acc:HGNC:10397] | 1809 | 0.217 | 0.7725 | Yes |
| 61 | RPS19 | ribosomal protein S19 [Source:HGNC Symbol;Acc:HGNC:10402] | 1867 | 0.214 | 0.7814 | Yes |
| 62 | RPS11 | ribosomal protein S11 [Source:HGNC Symbol;Acc:HGNC:10384] | 1924 | 0.210 | 0.7902 | Yes |
| 63 | RPS23 | ribosomal protein S23 [Source:HGNC Symbol;Acc:HGNC:10410] | 2033 | 0.204 | 0.7974 | Yes |
| 64 | RPS4X | ribosomal protein S4 X-linked [Source:HGNC Symbol;Acc:HGNC:10424] | 2135 | 0.199 | 0.8045 | Yes |
| 65 | RPL13A | ribosomal protein L13a [Source:HGNC Symbol;Acc:HGNC:10304] | 2165 | 0.198 | 0.8133 | Yes |
| 66 | RPS5 | ribosomal protein S5 [Source:HGNC Symbol;Acc:HGNC:10426] | 2169 | 0.198 | 0.8229 | Yes |
| 67 | RPL26 | ribosomal protein L26 [Source:HGNC Symbol;Acc:HGNC:10327] | 2245 | 0.195 | 0.8304 | Yes |
| 68 | RPS27A | ribosomal protein S27a [Source:HGNC Symbol;Acc:HGNC:10417] | 2311 | 0.192 | 0.8381 | Yes |
| 69 | RPS6 | ribosomal protein S6 [Source:HGNC Symbol;Acc:HGNC:10429] | 2370 | 0.190 | 0.8459 | Yes |
| 70 | RPS29 | ribosomal protein S29 [Source:HGNC Symbol;Acc:HGNC:10419] | 2491 | 0.185 | 0.8518 | Yes |
| 71 | RPL4 | ribosomal protein L4 [Source:HGNC Symbol;Acc:HGNC:10353] | 2982 | 0.165 | 0.8473 | Yes |
| 72 | RPLP0 | ribosomal protein lateral stalk subunit P0 [Source:HGNC Symbol;Acc:HGNC:10371] | 3306 | 0.154 | 0.8466 | Yes |
| 73 | RPL23A | ribosomal protein L23a [Source:HGNC Symbol;Acc:HGNC:10317] | 3836 | 0.139 | 0.8398 | Yes |
| 74 | MRPL13 | mitochondrial ribosomal protein L13 [Source:HGNC Symbol;Acc:HGNC:14278] | 3864 | 0.138 | 0.8458 | Yes |
| 75 | RPL6 | ribosomal protein L6 [Source:HGNC Symbol;Acc:HGNC:10362] | 4205 | 0.129 | 0.8434 | Yes |
| 76 | RPL41 | ribosomal protein L41 [Source:HGNC Symbol;Acc:HGNC:10354] | 4315 | 0.126 | 0.8468 | Yes |
| 77 | RPS12 | ribosomal protein S12 [Source:HGNC Symbol;Acc:HGNC:10385] | 4442 | 0.123 | 0.8495 | Yes |
| 78 | RPL17 | ribosomal protein L17 [Source:HGNC Symbol;Acc:HGNC:10307] | 4681 | 0.117 | 0.8491 | Yes |
| 79 | RPL7 | ribosomal protein L7 [Source:HGNC Symbol;Acc:HGNC:10363] | 4764 | 0.116 | 0.8527 | Yes |
| 80 | RPL21 | ribosomal protein L21 [Source:HGNC Symbol;Acc:HGNC:10313] | 5146 | 0.109 | 0.8482 | No |
| 81 | RPL36AL | ribosomal protein L36a like [Source:HGNC Symbol;Acc:HGNC:10346] | 6162 | 0.091 | 0.8268 | No |
| 82 | RPS26 | ribosomal protein S26 [Source:HGNC Symbol;Acc:HGNC:10414] | 6518 | 0.086 | 0.8219 | No |
| 83 | RPL22L1 | ribosomal protein L22 like 1 [Source:HGNC Symbol;Acc:HGNC:27610] | 7494 | 0.073 | 0.8006 | No |
| 84 | RPL10L | ribosomal protein L10 like [Source:HGNC Symbol;Acc:HGNC:17976] | 8178 | 0.066 | 0.7864 | No |
| 85 | RSL24D1 | ribosomal L24 domain containing 1 [Source:HGNC Symbol;Acc:HGNC:18479] | 8633 | 0.061 | 0.7778 | No |
| 86 | RPS27L | ribosomal protein S27 like [Source:HGNC Symbol;Acc:HGNC:18476] | 13158 | 0.025 | 0.6637 | No |
| 87 | RPS4Y1 | ribosomal protein S4 Y-linked 1 [Source:HGNC Symbol;Acc:HGNC:10425] | 16444 | 0.004 | 0.5802 | No |
| 88 | RSL24D1P11 | ribosomal L24 domain containing 1 pseudogene 11 [Source:HGNC Symbol;Acc:HGNC:37881] | 26628 | -0.060 | 0.3236 | No |
Table: GSEA details [plain text format]

  

Fig 2: KEGG\_RIBOSOME      
 Blue-Pink O' Gram in the Space of the Analyzed GeneSet

  

Fig 3: KEGG\_RIBOSOME: Random ES distribution      
 Gene set null distribution of ES for **KEGG\_RIBOSOME**

  
