# Supplementary material for: Simultaneous Presentation of Multiple Myeloma and Lung Cancer: Case Report and Gene Bioinformatics Analysis
Source: Front Oncol. 2022 Jun 13;12:859735. doi: 10.3389/fonc.2022.859735 (PMC9235397; doi:10.3389/fonc.2022.859735)
Supplement: Supplementary file 1 [file DataSheet_1.zip › The bioinformatic analysis of MM and lung cancer supplementary materials/Enrichment analysis/MECR/GSEA_4.1.0/LUAD TCGA/KEGG.Gsea.1639041756227/KEGG_RNA_POLYMERASE.html]

Details for gene set KEGG\_RNA\_POLYMERASE[GSEA]

|  || Dataset | ExpData\_collapsed\_to\_symbols.ENSG00000116353\_profile\_in\_ExpData.cls #ENSG00000116353 |
| Phenotype | ENSG00000116353\_profile\_in\_ExpData.cls#ENSG00000116353 |
| Upregulated in class | ENSG00000116353\_pos |
| GeneSet | KEGG\_RNA\_POLYMERASE |
| Enrichment Score (ES) | 0.6187338 |
| Normalized Enrichment Score (NES) | 1.9739654 |
| Nominal p-value | 0.0020876827 |
| FDR q-value | 0.0016032106 |
| FWER p-Value | 0.023 |
Table: GSEA Results Summary

  

Fig 1: Enrichment plot: KEGG\_RNA\_POLYMERASE      
 Profile of the Running ES Score & Positions of GeneSet Members on the Rank Ordered List

  

| SYMBOL | TITLE | RANK IN GENE LIST | RANK METRIC SCORE | RUNNING ES | CORE ENRICHMENT || 1 | POLR2L | "RNA polymerase II, I and III subunit L [Source:HGNC Symbol;Acc:HGNC:9199]" | 117 | 0.396 | 0.0741 | Yes |
| 2 | POLR1H | RNA polymerase I subunit H [Source:HGNC Symbol;Acc:HGNC:13182] | 575 | 0.310 | 0.1228 | Yes |
| 3 | POLR2E | "RNA polymerase II, I and III subunit E [Source:HGNC Symbol;Acc:HGNC:9192]" | 607 | 0.307 | 0.1817 | Yes |
| 4 | POLR2J | RNA polymerase II subunit J [Source:HGNC Symbol;Acc:HGNC:9197] | 736 | 0.294 | 0.2355 | Yes |
| 5 | POLR1C | RNA polymerase I and III subunit C [Source:HGNC Symbol;Acc:HGNC:20194] | 749 | 0.293 | 0.2922 | Yes |
| 6 | POLR3H | RNA polymerase III subunit H [Source:HGNC Symbol;Acc:HGNC:30349] | 797 | 0.288 | 0.3471 | Yes |
| 7 | POLR2I | RNA polymerase II subunit I [Source:HGNC Symbol;Acc:HGNC:9196] | 813 | 0.287 | 0.4024 | Yes |
| 8 | POLR2G | RNA polymerase II subunit G [Source:HGNC Symbol;Acc:HGNC:9194] | 999 | 0.270 | 0.4502 | Yes |
| 9 | POLR2H | "RNA polymerase II, I and III subunit H [Source:HGNC Symbol;Acc:HGNC:9195]" | 1077 | 0.264 | 0.4996 | Yes |
| 10 | POLR3C | RNA polymerase III subunit C [Source:HGNC Symbol;Acc:HGNC:30076] | 1448 | 0.238 | 0.5365 | Yes |
| 11 | POLR3GL | RNA polymerase III subunit GL [Source:HGNC Symbol;Acc:HGNC:28466] | 2080 | 0.202 | 0.5597 | Yes |
| 12 | POLR3K | RNA polymerase III subunit K [Source:HGNC Symbol;Acc:HGNC:14121] | 2176 | 0.198 | 0.5958 | Yes |
| 13 | POLR1D | RNA polymerase I and III subunit D [Source:HGNC Symbol;Acc:HGNC:20422] | 2640 | 0.179 | 0.6187 | Yes |
| 14 | POLR2K | "RNA polymerase II, I and III subunit K [Source:HGNC Symbol;Acc:HGNC:9198]" | 4390 | 0.124 | 0.5984 | No |
| 15 | POLR2C | RNA polymerase II subunit C [Source:HGNC Symbol;Acc:HGNC:9189] | 5263 | 0.106 | 0.5969 | No |
| 16 | POLR2F | "RNA polymerase II, I and III subunit F [Source:HGNC Symbol;Acc:HGNC:9193]" | 5558 | 0.101 | 0.6090 | No |
| 17 | POLR1E | RNA polymerase I subunit E [Source:HGNC Symbol;Acc:HGNC:17631] | 6313 | 0.089 | 0.6071 | No |
| 18 | POLR2J3 | RNA polymerase II subunit J3 [Source:HGNC Symbol;Acc:HGNC:33853] | 6597 | 0.085 | 0.6163 | No |
| 19 | POLR2J2 | RNA polymerase II subunit J2 [Source:HGNC Symbol;Acc:HGNC:23208] | 10680 | 0.044 | 0.5209 | No |
| 20 | POLR2D | RNA polymerase II subunit D [Source:HGNC Symbol;Acc:HGNC:9191] | 14063 | 0.019 | 0.4386 | No |
| 21 | POLR3G | RNA polymerase III subunit G [Source:HGNC Symbol;Acc:HGNC:30075] | 21988 | -0.029 | 0.2425 | No |
| 22 | POLR1B | RNA polymerase I subunit B [Source:HGNC Symbol;Acc:HGNC:20454] | 27448 | -0.067 | 0.1166 | No |
| 23 | POLR3F | RNA polymerase III subunit F [Source:HGNC Symbol;Acc:HGNC:15763] | 27481 | -0.067 | 0.1288 | No |
| 24 | POLR1A | RNA polymerase I subunit A [Source:HGNC Symbol;Acc:HGNC:17264] | 32355 | -0.118 | 0.0277 | No |
| 25 | POLR2B | RNA polymerase II subunit B [Source:HGNC Symbol;Acc:HGNC:9188] | 32968 | -0.127 | 0.0369 | No |
| 26 | POLR3B | RNA polymerase III subunit B [Source:HGNC Symbol;Acc:HGNC:30348] | 34273 | -0.150 | 0.0330 | No |
| 27 | POLR2A | RNA polymerase II subunit A [Source:HGNC Symbol;Acc:HGNC:9187] | 34410 | -0.153 | 0.0594 | No |
| 28 | POLR3D | RNA polymerase III subunit D [Source:HGNC Symbol;Acc:HGNC:1080] | 34776 | -0.162 | 0.0815 | No |
| 29 | POLR3A | RNA polymerase III subunit A [Source:HGNC Symbol;Acc:HGNC:30074] | 35364 | -0.176 | 0.1008 | No |
Table: GSEA details [plain text format]

  

Fig 2: KEGG\_RNA\_POLYMERASE      
 Blue-Pink O' Gram in the Space of the Analyzed GeneSet

  

Fig 3: KEGG\_RNA\_POLYMERASE: Random ES distribution      
 Gene set null distribution of ES for **KEGG\_RNA\_POLYMERASE**

  
