# Supplementary material for: Simultaneous Presentation of Multiple Myeloma and Lung Cancer: Case Report and Gene Bioinformatics Analysis
Source: Front Oncol. 2022 Jun 13;12:859735. doi: 10.3389/fonc.2022.859735 (PMC9235397; doi:10.3389/fonc.2022.859735)
Supplement: Supplementary file 1 [file DataSheet_1.zip › The bioinformatic analysis of MM and lung cancer supplementary materials/Enrichment analysis/MECR/GSEA_4.1.0/LUAD TCGA/KEGG.Gsea.1639041756227/KEGG_SMALL_CELL_LUNG_CANCER.html]

Details for gene set KEGG\_SMALL\_CELL\_LUNG\_CANCER[GSEA]

|  || Dataset | ExpData\_collapsed\_to\_symbols.ENSG00000116353\_profile\_in\_ExpData.cls #ENSG00000116353 |
| Phenotype | ENSG00000116353\_profile\_in\_ExpData.cls#ENSG00000116353 |
| Upregulated in class | ENSG00000116353\_neg |
| GeneSet | KEGG\_SMALL\_CELL\_LUNG\_CANCER |
| Enrichment Score (ES) | -0.5204514 |
| Normalized Enrichment Score (NES) | -2.101663 |
| Nominal p-value | 0.0 |
| FDR q-value | 2.2491808E-4 |
| FWER p-Value | 0.003 |
Table: GSEA Results Summary

  

Fig 1: Enrichment plot: KEGG\_SMALL\_CELL\_LUNG\_CANCER      
 Profile of the Running ES Score & Positions of GeneSet Members on the Rank Ordered List

  

| SYMBOL | TITLE | RANK IN GENE LIST | RANK METRIC SCORE | RUNNING ES | CORE ENRICHMENT || 1 | PIK3R2 | phosphoinositide-3-kinase regulatory subunit 2 [Source:HGNC Symbol;Acc:HGNC:8980] | 545 | 0.314 | 0.0111 | No |
| 2 | FHIT | fragile histidine triad diadenosine triphosphatase [Source:HGNC Symbol;Acc:HGNC:3701] | 606 | 0.307 | 0.0340 | No |
| 3 | RXRB | retinoid X receptor beta [Source:HGNC Symbol;Acc:HGNC:10478] | 1265 | 0.250 | 0.0372 | No |
| 4 | TRAF4 | TNF receptor associated factor 4 [Source:HGNC Symbol;Acc:HGNC:12034] | 1540 | 0.232 | 0.0487 | No |
| 5 | RXRA | retinoid X receptor alpha [Source:HGNC Symbol;Acc:HGNC:10477] | 2344 | 0.191 | 0.0435 | No |
| 6 | AKT1 | AKT serine/threonine kinase 1 [Source:HGNC Symbol;Acc:HGNC:391] | 3179 | 0.158 | 0.0348 | No |
| 7 | CASP9 | caspase 9 [Source:HGNC Symbol;Acc:HGNC:1511] | 3419 | 0.151 | 0.0407 | No |
| 8 | ITGA3 | integrin subunit alpha 3 [Source:HGNC Symbol;Acc:HGNC:6139] | 3614 | 0.145 | 0.0473 | No |
| 9 | TRAF2 | TNF receptor associated factor 2 [Source:HGNC Symbol;Acc:HGNC:12032] | 4769 | 0.116 | 0.0271 | No |
| 10 | CKS1B | CDC28 protein kinase regulatory subunit 1B [Source:HGNC Symbol;Acc:HGNC:19083] | 5563 | 0.101 | 0.0149 | No |
| 11 | PIAS3 | protein inhibitor of activated STAT 3 [Source:HGNC Symbol;Acc:HGNC:16861] | 5647 | 0.100 | 0.0207 | No |
| 12 | RELA | "RELA proto-oncogene, NF-kB subunit [Source:HGNC Symbol;Acc:HGNC:9955]" | 6022 | 0.093 | 0.0186 | No |
| 13 | PTK2 | protein tyrosine kinase 2 [Source:HGNC Symbol;Acc:HGNC:9611] | 6431 | 0.087 | 0.0151 | No |
| 14 | E2F1 | E2F transcription factor 1 [Source:HGNC Symbol;Acc:HGNC:3113] | 6628 | 0.084 | 0.0169 | No |
| 15 | BCL2L1 | BCL2 like 1 [Source:HGNC Symbol;Acc:HGNC:992] | 6882 | 0.081 | 0.0169 | No |
| 16 | LAMB2 | laminin subunit beta 2 [Source:HGNC Symbol;Acc:HGNC:6487] | 7162 | 0.077 | 0.0159 | No |
| 17 | NFKBIA | NFKB inhibitor alpha [Source:HGNC Symbol;Acc:HGNC:7797] | 7696 | 0.071 | 0.0080 | No |
| 18 | CYCS | "cytochrome c, somatic [Source:HGNC Symbol;Acc:HGNC:19986]" | 8363 | 0.064 | -0.0040 | No |
| 19 | TP53 | tumor protein p53 [Source:HGNC Symbol;Acc:HGNC:11998] | 8681 | 0.061 | -0.0072 | No |
| 20 | PIK3R3 | phosphoinositide-3-kinase regulatory subunit 3 [Source:HGNC Symbol;Acc:HGNC:8981] | 9028 | 0.057 | -0.0115 | No |
| 21 | ITGA2B | integrin subunit alpha 2b [Source:HGNC Symbol;Acc:HGNC:6138] | 9230 | 0.055 | -0.0122 | No |
| 22 | IKBKG | inhibitor of nuclear factor kappa B kinase regulatory subunit gamma [Source:HGNC Symbol;Acc:HGNC:5961] | 9304 | 0.055 | -0.0097 | No |
| 23 | AKT2 | AKT serine/threonine kinase 2 [Source:HGNC Symbol;Acc:HGNC:392] | 10579 | 0.044 | -0.0386 | No |
| 24 | PIAS4 | protein inhibitor of activated STAT 4 [Source:HGNC Symbol;Acc:HGNC:17002] | 11249 | 0.039 | -0.0526 | No |
| 25 | LAMA5 | laminin subunit alpha 5 [Source:HGNC Symbol;Acc:HGNC:6485] | 13742 | 0.021 | -0.1144 | No |
| 26 | CDK4 | cyclin dependent kinase 4 [Source:HGNC Symbol;Acc:HGNC:1773] | 15379 | 0.011 | -0.1553 | No |
| 27 | LAMB3 | laminin subunit beta 3 [Source:HGNC Symbol;Acc:HGNC:6490] | 16710 | 0.003 | -0.1889 | No |
| 28 | MYC | "MYC proto-oncogene, bHLH transcription factor [Source:HGNC Symbol;Acc:HGNC:7553]" | 16923 | 0.002 | -0.1942 | No |
| 29 | CCNE1 | cyclin E1 [Source:HGNC Symbol;Acc:HGNC:1589] | 17516 | -0.002 | -0.2091 | No |
| 30 | MAX | MYC associated factor X [Source:HGNC Symbol;Acc:HGNC:6913] | 18706 | -0.009 | -0.2387 | No |
| 31 | LAMC2 | laminin subunit gamma 2 [Source:HGNC Symbol;Acc:HGNC:6493] | 19324 | -0.013 | -0.2534 | No |
| 32 | RXRG | retinoid X receptor gamma [Source:HGNC Symbol;Acc:HGNC:10479] | 19690 | -0.015 | -0.2615 | No |
| 33 | COL4A4 | collagen type IV alpha 4 chain [Source:HGNC Symbol;Acc:HGNC:2206] | 20261 | -0.018 | -0.2746 | No |
| 34 | LAMC3 | laminin subunit gamma 3 [Source:HGNC Symbol;Acc:HGNC:6494] | 21084 | -0.023 | -0.2937 | No |
| 35 | IKBKB | inhibitor of nuclear factor kappa B kinase subunit beta [Source:HGNC Symbol;Acc:HGNC:5960] | 21393 | -0.025 | -0.2996 | No |
| 36 | CDKN2B | cyclin dependent kinase inhibitor 2B [Source:HGNC Symbol;Acc:HGNC:1788] | 21665 | -0.027 | -0.3043 | No |
| 37 | E2F3 | E2F transcription factor 3 [Source:HGNC Symbol;Acc:HGNC:3115] | 23219 | -0.037 | -0.3410 | No |
| 38 | CCND1 | cyclin D1 [Source:HGNC Symbol;Acc:HGNC:1582] | 25465 | -0.052 | -0.3940 | No |
| 39 | COL4A6 | collagen type IV alpha 6 chain [Source:HGNC Symbol;Acc:HGNC:2208] | 27266 | -0.065 | -0.4347 | No |
| 40 | PTEN | phosphatase and tensin homolog [Source:HGNC Symbol;Acc:HGNC:9588] | 27697 | -0.069 | -0.4402 | No |
| 41 | SKP2 | S-phase kinase associated protein 2 [Source:HGNC Symbol;Acc:HGNC:10901] | 27776 | -0.069 | -0.4367 | No |
| 42 | PIAS2 | protein inhibitor of activated STAT 2 [Source:HGNC Symbol;Acc:HGNC:17311] | 29251 | -0.082 | -0.4677 | No |
| 43 | TRAF1 | TNF receptor associated factor 1 [Source:HGNC Symbol;Acc:HGNC:12031] | 29596 | -0.085 | -0.4697 | No |
| 44 | E2F2 | E2F transcription factor 2 [Source:HGNC Symbol;Acc:HGNC:3114] | 31025 | -0.101 | -0.4980 | No |
| 45 | LAMA3 | laminin subunit alpha 3 [Source:HGNC Symbol;Acc:HGNC:6483] | 31400 | -0.105 | -0.4992 | No |
| 46 | PIK3CD | "phosphatidylinositol-4,5-bisphosphate 3-kinase catalytic subunit delta [Source:HGNC Symbol;Acc:HGNC:8977]" | 31859 | -0.111 | -0.5020 | No |
| 47 | PTGS2 | prostaglandin-endoperoxide synthase 2 [Source:HGNC Symbol;Acc:HGNC:9605] | 32570 | -0.121 | -0.5105 | Yes |
| 48 | XIAP | X-linked inhibitor of apoptosis [Source:HGNC Symbol;Acc:HGNC:592] | 32963 | -0.127 | -0.5103 | Yes |
| 49 | CDKN1B | cyclin dependent kinase inhibitor 1B [Source:HGNC Symbol;Acc:HGNC:1785] | 33152 | -0.130 | -0.5047 | Yes |
| 50 | LAMC1 | laminin subunit gamma 1 [Source:HGNC Symbol;Acc:HGNC:6492] | 33481 | -0.136 | -0.5023 | Yes |
| 51 | CDK2 | cyclin dependent kinase 2 [Source:HGNC Symbol;Acc:HGNC:1771] | 33735 | -0.140 | -0.4975 | Yes |
| 52 | NOS2 | nitric oxide synthase 2 [Source:HGNC Symbol;Acc:HGNC:7873] | 33997 | -0.145 | -0.4926 | Yes |
| 53 | CCNE2 | cyclin E2 [Source:HGNC Symbol;Acc:HGNC:1590] | 34088 | -0.147 | -0.4832 | Yes |
| 54 | BIRC2 | baculoviral IAP repeat containing 2 [Source:HGNC Symbol;Acc:HGNC:590] | 34221 | -0.149 | -0.4747 | Yes |
| 55 | PIK3R1 | phosphoinositide-3-kinase regulatory subunit 1 [Source:HGNC Symbol;Acc:HGNC:8979] | 34788 | -0.162 | -0.4762 | Yes |
| 56 | PIK3CB | "phosphatidylinositol-4,5-bisphosphate 3-kinase catalytic subunit beta [Source:HGNC Symbol;Acc:HGNC:8976]" | 34871 | -0.164 | -0.4653 | Yes |
| 57 | LAMA1 | laminin subunit alpha 1 [Source:HGNC Symbol;Acc:HGNC:6481] | 34927 | -0.164 | -0.4536 | Yes |
| 58 | LAMB4 | laminin subunit beta 4 [Source:HGNC Symbol;Acc:HGNC:6491] | 35118 | -0.169 | -0.4449 | Yes |
| 59 | COL4A2 | collagen type IV alpha 2 chain [Source:HGNC Symbol;Acc:HGNC:2203] | 35236 | -0.172 | -0.4342 | Yes |
| 60 | TRAF5 | TNF receptor associated factor 5 [Source:HGNC Symbol;Acc:HGNC:12035] | 35693 | -0.184 | -0.4311 | Yes |
| 61 | TRAF3 | TNF receptor associated factor 3 [Source:HGNC Symbol;Acc:HGNC:12033] | 35778 | -0.187 | -0.4184 | Yes |
| 62 | ITGA2 | integrin subunit alpha 2 [Source:HGNC Symbol;Acc:HGNC:6137] | 35833 | -0.189 | -0.4047 | Yes |
| 63 | BIRC3 | baculoviral IAP repeat containing 3 [Source:HGNC Symbol;Acc:HGNC:591] | 35840 | -0.189 | -0.3898 | Yes |
| 64 | NFKB1 | nuclear factor kappa B subunit 1 [Source:HGNC Symbol;Acc:HGNC:7794] | 36367 | -0.207 | -0.3868 | Yes |
| 65 | FN1 | fibronectin 1 [Source:HGNC Symbol;Acc:HGNC:3778] | 36715 | -0.220 | -0.3781 | Yes |
| 66 | RB1 | RB transcriptional corepressor 1 [Source:HGNC Symbol;Acc:HGNC:9884] | 36794 | -0.225 | -0.3621 | Yes |
| 67 | COL4A1 | collagen type IV alpha 1 chain [Source:HGNC Symbol;Acc:HGNC:2202] | 36855 | -0.228 | -0.3455 | Yes |
| 68 | CDK6 | cyclin dependent kinase 6 [Source:HGNC Symbol;Acc:HGNC:1777] | 36874 | -0.229 | -0.3278 | Yes |
| 69 | ITGA6 | integrin subunit alpha 6 [Source:HGNC Symbol;Acc:HGNC:6142] | 37064 | -0.239 | -0.3135 | Yes |
| 70 | LAMA2 | laminin subunit alpha 2 [Source:HGNC Symbol;Acc:HGNC:6482] | 37457 | -0.263 | -0.3026 | Yes |
| 71 | PIK3CA | "phosphatidylinositol-4,5-bisphosphate 3-kinase catalytic subunit alpha [Source:HGNC Symbol;Acc:HGNC:8975]" | 37556 | -0.270 | -0.2835 | Yes |
| 72 | ITGAV | integrin subunit alpha V [Source:HGNC Symbol;Acc:HGNC:6150] | 37606 | -0.275 | -0.2629 | Yes |
| 73 | LAMB1 | laminin subunit beta 1 [Source:HGNC Symbol;Acc:HGNC:6486] | 37639 | -0.278 | -0.2416 | Yes |
| 74 | RARB | retinoic acid receptor beta [Source:HGNC Symbol;Acc:HGNC:9865] | 37692 | -0.283 | -0.2204 | Yes |
| 75 | BCL2 | BCL2 apoptosis regulator [Source:HGNC Symbol;Acc:HGNC:990] | 37702 | -0.283 | -0.1980 | Yes |
| 76 | TRAF6 | TNF receptor associated factor 6 [Source:HGNC Symbol;Acc:HGNC:12036] | 37748 | -0.286 | -0.1764 | Yes |
| 77 | CHUK | component of inhibitor of nuclear factor kappa B kinase complex [Source:HGNC Symbol;Acc:HGNC:1974] | 37762 | -0.287 | -0.1538 | Yes |
| 78 | ITGB1 | integrin subunit beta 1 [Source:HGNC Symbol;Acc:HGNC:6153] | 37850 | -0.297 | -0.1324 | Yes |
| 79 | PIK3R5 | phosphoinositide-3-kinase regulatory subunit 5 [Source:HGNC Symbol;Acc:HGNC:30035] | 37956 | -0.310 | -0.1104 | Yes |
| 80 | PIAS1 | protein inhibitor of activated STAT 1 [Source:HGNC Symbol;Acc:HGNC:2752] | 37975 | -0.313 | -0.0859 | Yes |
| 81 | LAMA4 | laminin subunit alpha 4 [Source:HGNC Symbol;Acc:HGNC:6484] | 38106 | -0.335 | -0.0626 | Yes |
| 82 | AKT3 | AKT serine/threonine kinase 3 [Source:HGNC Symbol;Acc:HGNC:393] | 38196 | -0.362 | -0.0360 | Yes |
| 83 | APAF1 | apoptotic peptidase activating factor 1 [Source:HGNC Symbol;Acc:HGNC:576] | 38254 | -0.386 | -0.0067 | Yes |
| 84 | PIK3CG | "phosphatidylinositol-4,5-bisphosphate 3-kinase catalytic subunit gamma [Source:HGNC Symbol;Acc:HGNC:8978]" | 38302 | -0.426 | 0.0260 | Yes |
Table: GSEA details [plain text format]

  

Fig 2: KEGG\_SMALL\_CELL\_LUNG\_CANCER      
 Blue-Pink O' Gram in the Space of the Analyzed GeneSet

  

Fig 3: KEGG\_SMALL\_CELL\_LUNG\_CANCER: Random ES distribution      
 Gene set null distribution of ES for **KEGG\_SMALL\_CELL\_LUNG\_CANCER**

  
