# Supplementary material for: Simultaneous Presentation of Multiple Myeloma and Lung Cancer: Case Report and Gene Bioinformatics Analysis
Source: Front Oncol. 2022 Jun 13;12:859735. doi: 10.3389/fonc.2022.859735 (PMC9235397; doi:10.3389/fonc.2022.859735)
Supplement: Supplementary file 1 [file DataSheet_1.zip › The bioinformatic analysis of MM and lung cancer supplementary materials/Enrichment analysis/MECR/GSEA_4.1.0/LUAD TCGA/KEGG.Gsea.1639041756227/KEGG_SPLICEOSOME.html]

Details for gene set KEGG\_SPLICEOSOME[GSEA]

|  || Dataset | ExpData\_collapsed\_to\_symbols.ENSG00000116353\_profile\_in\_ExpData.cls #ENSG00000116353 |
| Phenotype | ENSG00000116353\_profile\_in\_ExpData.cls#ENSG00000116353 |
| Upregulated in class | ENSG00000116353\_pos |
| GeneSet | KEGG\_SPLICEOSOME |
| Enrichment Score (ES) | 0.47124943 |
| Normalized Enrichment Score (NES) | 2.0015059 |
| Nominal p-value | 0.0 |
| FDR q-value | 0.0013631961 |
| FWER p-Value | 0.018 |
Table: GSEA Results Summary

  

Fig 1: Enrichment plot: KEGG\_SPLICEOSOME      
 Profile of the Running ES Score & Positions of GeneSet Members on the Rank Ordered List

  

| SYMBOL | TITLE | RANK IN GENE LIST | RANK METRIC SCORE | RUNNING ES | CORE ENRICHMENT || 1 | PPIE | peptidylprolyl isomerase E [Source:HGNC Symbol;Acc:HGNC:9258] | 107 | 0.401 | 0.0201 | Yes |
| 2 | CCDC12 | coiled-coil domain containing 12 [Source:HGNC Symbol;Acc:HGNC:28332] | 136 | 0.387 | 0.0415 | Yes |
| 3 | LSM4 | "LSM4 homolog, U6 small nuclear RNA and mRNA degradation associated [Source:HGNC Symbol;Acc:HGNC:17259]" | 171 | 0.381 | 0.0623 | Yes |
| 4 | SNU13 | small nuclear ribonucleoprotein 13 [Source:HGNC Symbol;Acc:HGNC:7819] | 277 | 0.355 | 0.0798 | Yes |
| 5 | SYF2 | SYF2 pre-mRNA splicing factor [Source:HGNC Symbol;Acc:HGNC:19824] | 312 | 0.348 | 0.0988 | Yes |
| 6 | SRSF4 | serine and arginine rich splicing factor 4 [Source:HGNC Symbol;Acc:HGNC:10786] | 486 | 0.320 | 0.1126 | Yes |
| 7 | PQBP1 | polyglutamine binding protein 1 [Source:HGNC Symbol;Acc:HGNC:9330] | 565 | 0.311 | 0.1284 | Yes |
| 8 | PPIH | peptidylprolyl isomerase H [Source:HGNC Symbol;Acc:HGNC:14651] | 620 | 0.305 | 0.1444 | Yes |
| 9 | SNRPC | small nuclear ribonucleoprotein polypeptide C [Source:HGNC Symbol;Acc:HGNC:11157] | 733 | 0.294 | 0.1583 | Yes |
| 10 | LSM2 | "LSM2 homolog, U6 small nuclear RNA and mRNA degradation associated [Source:HGNC Symbol;Acc:HGNC:13940]" | 773 | 0.291 | 0.1739 | Yes |
| 11 | PUF60 | poly(U) binding splicing factor 60 [Source:HGNC Symbol;Acc:HGNC:17042] | 904 | 0.279 | 0.1864 | Yes |
| 12 | SNRNP40 | small nuclear ribonucleoprotein U5 subunit 40 [Source:HGNC Symbol;Acc:HGNC:30857] | 977 | 0.271 | 0.2001 | Yes |
| 13 | SNRPA | small nuclear ribonucleoprotein polypeptide A [Source:HGNC Symbol;Acc:HGNC:11151] | 1081 | 0.264 | 0.2125 | Yes |
| 14 | SF3B5 | splicing factor 3b subunit 5 [Source:HGNC Symbol;Acc:HGNC:21083] | 1146 | 0.260 | 0.2257 | Yes |
| 15 | XAB2 | XPA binding protein 2 [Source:HGNC Symbol;Acc:HGNC:14089] | 1156 | 0.259 | 0.2402 | Yes |
| 16 | LSM7 | "LSM7 homolog, U6 small nuclear RNA and mRNA degradation associated [Source:HGNC Symbol;Acc:HGNC:20470]" | 1180 | 0.256 | 0.2542 | Yes |
| 17 | SF3B4 | splicing factor 3b subunit 4 [Source:HGNC Symbol;Acc:HGNC:10771] | 1358 | 0.244 | 0.2636 | Yes |
| 18 | CWC15 | CWC15 spliceosome associated protein homolog [Source:HGNC Symbol;Acc:HGNC:26939] | 1534 | 0.233 | 0.2724 | Yes |
| 19 | SNRPE | small nuclear ribonucleoprotein polypeptide E [Source:HGNC Symbol;Acc:HGNC:11161] | 1563 | 0.231 | 0.2849 | Yes |
| 20 | SF3B6 | splicing factor 3b subunit 6 [Source:HGNC Symbol;Acc:HGNC:30096] | 1674 | 0.224 | 0.2949 | Yes |
| 21 | BUD31 | BUD31 homolog [Source:HGNC Symbol;Acc:HGNC:29629] | 1709 | 0.223 | 0.3067 | Yes |
| 22 | SF3A3 | splicing factor 3a subunit 3 [Source:HGNC Symbol;Acc:HGNC:10767] | 1801 | 0.217 | 0.3168 | Yes |
| 23 | USP39 | ubiquitin specific peptidase 39 [Source:HGNC Symbol;Acc:HGNC:20071] | 1882 | 0.213 | 0.3268 | Yes |
| 24 | LSM5 | "LSM5 homolog, U6 small nuclear RNA and mRNA degradation associated [Source:HGNC Symbol;Acc:HGNC:17162]" | 2079 | 0.202 | 0.3334 | Yes |
| 25 | ISY1 | ISY1 splicing factor homolog [Source:HGNC Symbol;Acc:HGNC:29201] | 2212 | 0.196 | 0.3412 | Yes |
| 26 | MAGOH | "mago homolog, exon junction complex subunit [Source:HGNC Symbol;Acc:HGNC:6815]" | 2343 | 0.191 | 0.3488 | Yes |
| 27 | PHF5A | PHD finger protein 5A [Source:HGNC Symbol;Acc:HGNC:18000] | 2388 | 0.189 | 0.3584 | Yes |
| 28 | TRA2A | transformer 2 alpha homolog [Source:HGNC Symbol;Acc:HGNC:16645] | 2410 | 0.188 | 0.3686 | Yes |
| 29 | PRPF31 | pre-mRNA processing factor 31 [Source:HGNC Symbol;Acc:HGNC:15446] | 2437 | 0.187 | 0.3786 | Yes |
| 30 | SRSF3 | serine and arginine rich splicing factor 3 [Source:HGNC Symbol;Acc:HGNC:10785] | 2501 | 0.184 | 0.3875 | Yes |
| 31 | SF3A2 | splicing factor 3a subunit 2 [Source:HGNC Symbol;Acc:HGNC:10766] | 2680 | 0.177 | 0.3930 | Yes |
| 32 | LSM3 | "LSM3 homolog, U6 small nuclear RNA and mRNA degradation associated [Source:HGNC Symbol;Acc:HGNC:17874]" | 2740 | 0.174 | 0.4014 | Yes |
| 33 | PRPF6 | pre-mRNA processing factor 6 [Source:HGNC Symbol;Acc:HGNC:15860] | 2766 | 0.173 | 0.4107 | Yes |
| 34 | THOC3 | THO complex 3 [Source:HGNC Symbol;Acc:HGNC:19072] | 2919 | 0.167 | 0.4163 | Yes |
| 35 | TXNL4A | thioredoxin like 4A [Source:HGNC Symbol;Acc:HGNC:30551] | 2963 | 0.166 | 0.4247 | Yes |
| 36 | SNRPD2 | small nuclear ribonucleoprotein D2 polypeptide [Source:HGNC Symbol;Acc:HGNC:11159] | 3012 | 0.163 | 0.4327 | Yes |
| 37 | SNRPB | small nuclear ribonucleoprotein polypeptides B and B1 [Source:HGNC Symbol;Acc:HGNC:11153] | 3349 | 0.153 | 0.4329 | Yes |
| 38 | SNRPD3 | small nuclear ribonucleoprotein D3 polypeptide [Source:HGNC Symbol;Acc:HGNC:11160] | 3567 | 0.146 | 0.4357 | Yes |
| 39 | SNRPG | small nuclear ribonucleoprotein polypeptide G [Source:HGNC Symbol;Acc:HGNC:11163] | 3604 | 0.145 | 0.4430 | Yes |
| 40 | HNRNPC | heterogeneous nuclear ribonucleoprotein C [Source:HGNC Symbol;Acc:HGNC:5035] | 3684 | 0.142 | 0.4491 | Yes |
| 41 | SRSF8 | serine and arginine rich splicing factor 8 [Source:HGNC Symbol;Acc:HGNC:16988] | 3859 | 0.138 | 0.4526 | Yes |
| 42 | SRSF2 | serine and arginine rich splicing factor 2 [Source:HGNC Symbol;Acc:HGNC:10783] | 4193 | 0.129 | 0.4514 | Yes |
| 43 | SART1 | "spliceosome associated factor 1, recruiter of U4/U6.U5 tri-snRNP [Source:HGNC Symbol;Acc:HGNC:10538]" | 4486 | 0.122 | 0.4509 | Yes |
| 44 | ZMAT2 | zinc finger matrin-type 2 [Source:HGNC Symbol;Acc:HGNC:26433] | 4589 | 0.120 | 0.4551 | Yes |
| 45 | PPIL1 | peptidylprolyl isomerase like 1 [Source:HGNC Symbol;Acc:HGNC:9260] | 5210 | 0.107 | 0.4454 | Yes |
| 46 | BCAS2 | BCAS2 pre-mRNA processing factor [Source:HGNC Symbol;Acc:HGNC:975] | 5399 | 0.104 | 0.4466 | Yes |
| 47 | SNRNP70 | small nuclear ribonucleoprotein U1 subunit 70 [Source:HGNC Symbol;Acc:HGNC:11150] | 5479 | 0.102 | 0.4504 | Yes |
| 48 | SRSF10 | serine and arginine rich splicing factor 10 [Source:HGNC Symbol;Acc:HGNC:16713] | 5562 | 0.101 | 0.4540 | Yes |
| 49 | LSM6 | "LSM6 homolog, U6 small nuclear RNA and mRNA degradation associated [Source:HGNC Symbol;Acc:HGNC:17017]" | 5938 | 0.095 | 0.4499 | Yes |
| 50 | SRSF7 | serine and arginine rich splicing factor 7 [Source:HGNC Symbol;Acc:HGNC:10789] | 6007 | 0.094 | 0.4535 | Yes |
| 51 | RBM8A | RNA binding motif protein 8A [Source:HGNC Symbol;Acc:HGNC:9905] | 6115 | 0.092 | 0.4559 | Yes |
| 52 | PRPF38A | pre-mRNA processing factor 38A [Source:HGNC Symbol;Acc:HGNC:25930] | 6165 | 0.091 | 0.4599 | Yes |
| 53 | HSPA1A | heat shock protein family A (Hsp70) member 1A [Source:HGNC Symbol;Acc:HGNC:5232] | 6240 | 0.090 | 0.4631 | Yes |
| 54 | EIF4A3 | eukaryotic translation initiation factor 4A3 [Source:HGNC Symbol;Acc:HGNC:18683] | 6370 | 0.088 | 0.4648 | Yes |
| 55 | PLRG1 | pleiotropic regulator 1 [Source:HGNC Symbol;Acc:HGNC:9089] | 6588 | 0.085 | 0.4641 | Yes |
| 56 | SNRPB2 | small nuclear ribonucleoprotein polypeptide B2 [Source:HGNC Symbol;Acc:HGNC:11155] | 6627 | 0.084 | 0.4679 | Yes |
| 57 | HSPA1L | heat shock protein family A (Hsp70) member 1 like [Source:HGNC Symbol;Acc:HGNC:5234] | 6686 | 0.084 | 0.4712 | Yes |
| 58 | ALYREF | Aly/REF export factor [Source:HGNC Symbol;Acc:HGNC:19071] | 7031 | 0.079 | 0.4670 | No |
| 59 | PRPF3 | pre-mRNA processing factor 3 [Source:HGNC Symbol;Acc:HGNC:17348] | 7473 | 0.073 | 0.4599 | No |
| 60 | PRPF19 | pre-mRNA processing factor 19 [Source:HGNC Symbol;Acc:HGNC:17896] | 7798 | 0.069 | 0.4556 | No |
| 61 | RBMX | RNA binding motif protein X-linked [Source:HGNC Symbol;Acc:HGNC:9910] | 8191 | 0.065 | 0.4493 | No |
| 62 | SNRPF | small nuclear ribonucleoprotein polypeptide F [Source:HGNC Symbol;Acc:HGNC:11162] | 9588 | 0.052 | 0.4167 | No |
| 63 | MAGOHB | "mago homolog B, exon junction complex subunit [Source:HGNC Symbol;Acc:HGNC:25504]" | 9870 | 0.050 | 0.4124 | No |
| 64 | SRSF6 | serine and arginine rich splicing factor 6 [Source:HGNC Symbol;Acc:HGNC:10788] | 9894 | 0.050 | 0.4146 | No |
| 65 | HNRNPA1 | heterogeneous nuclear ribonucleoprotein A1 [Source:HGNC Symbol;Acc:HGNC:5031] | 10145 | 0.048 | 0.4109 | No |
| 66 | CHERP | calcium homeostasis endoplasmic reticulum protein [Source:HGNC Symbol;Acc:HGNC:16930] | 10209 | 0.047 | 0.4120 | No |
| 67 | PCBP1 | poly(rC) binding protein 1 [Source:HGNC Symbol;Acc:HGNC:8647] | 11198 | 0.039 | 0.3891 | No |
| 68 | NCBP2 | nuclear cap binding protein subunit 2 [Source:HGNC Symbol;Acc:HGNC:7659] | 11457 | 0.037 | 0.3846 | No |
| 69 | HSPA1B | heat shock protein family A (Hsp70) member 1B [Source:HGNC Symbol;Acc:HGNC:5233] | 11746 | 0.035 | 0.3793 | No |
| 70 | SRSF5 | serine and arginine rich splicing factor 5 [Source:HGNC Symbol;Acc:HGNC:10787] | 11874 | 0.034 | 0.3780 | No |
| 71 | RBM22 | RNA binding motif protein 22 [Source:HGNC Symbol;Acc:HGNC:25503] | 12254 | 0.031 | 0.3701 | No |
| 72 | HSPA6 | heat shock protein family A (Hsp70) member 6 [Source:HGNC Symbol;Acc:HGNC:5239] | 12654 | 0.028 | 0.3615 | No |
| 73 | CTNNBL1 | catenin beta like 1 [Source:HGNC Symbol;Acc:HGNC:15879] | 13240 | 0.024 | 0.3480 | No |
| 74 | U2AF2 | U2 small nuclear RNA auxiliary factor 2 [Source:HGNC Symbol;Acc:HGNC:23156] | 13662 | 0.021 | 0.3384 | No |
| 75 | DDX39B | DExD-box helicase 39B [Source:HGNC Symbol;Acc:HGNC:13917] | 13755 | 0.021 | 0.3373 | No |
| 76 | SF3B2 | splicing factor 3b subunit 2 [Source:HGNC Symbol;Acc:HGNC:10769] | 13920 | 0.020 | 0.3342 | No |
| 77 | U2AF1 | U2 small nuclear RNA auxiliary factor 1 [Source:HGNC Symbol;Acc:HGNC:12453] | 15573 | 0.010 | 0.2926 | No |
| 78 | SNRPD1 | small nuclear ribonucleoprotein D1 polypeptide [Source:HGNC Symbol;Acc:HGNC:11158] | 16397 | 0.005 | 0.2719 | No |
| 79 | SNRPA1 | small nuclear ribonucleoprotein polypeptide A' [Source:HGNC Symbol;Acc:HGNC:11152] | 16828 | 0.002 | 0.2610 | No |
| 80 | ACIN1 | apoptotic chromatin condensation inducer 1 [Source:HGNC Symbol;Acc:HGNC:17066] | 16848 | 0.002 | 0.2607 | No |
| 81 | SNRNP27 | small nuclear ribonucleoprotein U4/U6.U5 subunit 27 [Source:HGNC Symbol;Acc:HGNC:30240] | 17166 | 0.000 | 0.2526 | No |
| 82 | PRPF40B | pre-mRNA processing factor 40 homolog B [Source:HGNC Symbol;Acc:HGNC:25031] | 17302 | -0.001 | 0.2492 | No |
| 83 | SMNDC1 | survival motor neuron domain containing 1 [Source:HGNC Symbol;Acc:HGNC:16900] | 19051 | -0.011 | 0.2052 | No |
| 84 | LSM8 | "LSM8 homolog, U6 small nuclear RNA associated [Source:HGNC Symbol;Acc:HGNC:20471]" | 19453 | -0.013 | 0.1958 | No |
| 85 | HNRNPA1L2 | heterogeneous nuclear ribonucleoprotein A1 like 2 [Source:HGNC Symbol;Acc:HGNC:27067] | 19816 | -0.016 | 0.1874 | No |
| 86 | DHX16 | DEAH-box helicase 16 [Source:HGNC Symbol;Acc:HGNC:2739] | 20048 | -0.017 | 0.1825 | No |
| 87 | HNRNPA1P60 | heterogeneous nuclear ribonucleoprotein A1 pseudogene 60 [Source:HGNC Symbol;Acc:HGNC:48790] | 20765 | -0.021 | 0.1654 | No |
| 88 | PRPF18 | pre-mRNA processing factor 18 [Source:HGNC Symbol;Acc:HGNC:17351] | 21237 | -0.024 | 0.1548 | No |
| 89 | SLU7 | "SLU7 homolog, splicing factor [Source:HGNC Symbol;Acc:HGNC:16939]" | 22552 | -0.032 | 0.1231 | No |
| 90 | DDX42 | DEAD-box helicase 42 [Source:HGNC Symbol;Acc:HGNC:18676] | 22832 | -0.034 | 0.1180 | No |
| 91 | THOC1 | THO complex 1 [Source:HGNC Symbol;Acc:HGNC:19070] | 23200 | -0.037 | 0.1107 | No |
| 92 | HNRNPK | heterogeneous nuclear ribonucleoprotein K [Source:HGNC Symbol;Acc:HGNC:5044] | 24051 | -0.042 | 0.0914 | No |
| 93 | TRA2B | transformer 2 beta homolog [Source:HGNC Symbol;Acc:HGNC:10781] | 24379 | -0.044 | 0.0856 | No |
| 94 | EFTUD2 | elongation factor Tu GTP binding domain containing 2 [Source:HGNC Symbol;Acc:HGNC:30858] | 25951 | -0.056 | 0.0487 | No |
| 95 | TCERG1 | transcription elongation regulator 1 [Source:HGNC Symbol;Acc:HGNC:15630] | 26101 | -0.057 | 0.0481 | No |
| 96 | SRSF1 | serine and arginine rich splicing factor 1 [Source:HGNC Symbol;Acc:HGNC:10780] | 27641 | -0.068 | 0.0127 | No |
| 97 | CDC5L | cell division cycle 5 like [Source:HGNC Symbol;Acc:HGNC:1743] | 27889 | -0.070 | 0.0104 | No |
| 98 | SNW1 | SNW domain containing 1 [Source:HGNC Symbol;Acc:HGNC:16696] | 27999 | -0.071 | 0.0117 | No |
| 99 | HNRNPU | heterogeneous nuclear ribonucleoprotein U [Source:HGNC Symbol;Acc:HGNC:5048] | 28332 | -0.074 | 0.0074 | No |
| 100 | HSPA2 | heat shock protein family A (Hsp70) member 2 [Source:HGNC Symbol;Acc:HGNC:5235] | 28545 | -0.076 | 0.0063 | No |
| 101 | SRSF9 | serine and arginine rich splicing factor 9 [Source:HGNC Symbol;Acc:HGNC:10791] | 28629 | -0.077 | 0.0086 | No |
| 102 | PRPF4 | pre-mRNA processing factor 4 [Source:HGNC Symbol;Acc:HGNC:17349] | 28788 | -0.078 | 0.0090 | No |
| 103 | DDX5 | DEAD-box helicase 5 [Source:HGNC Symbol;Acc:HGNC:2746] | 29280 | -0.082 | 0.0012 | No |
| 104 | RBM17 | RNA binding motif protein 17 [Source:HGNC Symbol;Acc:HGNC:16944] | 29914 | -0.089 | -0.0099 | No |
| 105 | SF3A1 | splicing factor 3a subunit 1 [Source:HGNC Symbol;Acc:HGNC:10765] | 31768 | -0.110 | -0.0509 | No |
| 106 | HNRNPA3 | heterogeneous nuclear ribonucleoprotein A3 [Source:HGNC Symbol;Acc:HGNC:24941] | 31943 | -0.112 | -0.0490 | No |
| 107 | DHX38 | DEAH-box helicase 38 [Source:HGNC Symbol;Acc:HGNC:17211] | 32010 | -0.113 | -0.0442 | No |
| 108 | SF3B3 | splicing factor 3b subunit 3 [Source:HGNC Symbol;Acc:HGNC:10770] | 32319 | -0.117 | -0.0454 | No |
| 109 | DHX15 | DEAH-box helicase 15 [Source:HGNC Symbol;Acc:HGNC:2738] | 33118 | -0.130 | -0.0583 | No |
| 110 | HSPA8 | heat shock protein family A (Hsp70) member 8 [Source:HGNC Symbol;Acc:HGNC:5241] | 33901 | -0.143 | -0.0701 | No |
| 111 | RBM25 | RNA binding motif protein 25 [Source:HGNC Symbol;Acc:HGNC:23244] | 33952 | -0.145 | -0.0632 | No |
| 112 | CRNKL1 | crooked neck pre-mRNA splicing factor 1 [Source:HGNC Symbol;Acc:HGNC:15762] | 34024 | -0.146 | -0.0567 | No |
| 113 | THOC2 | THO complex 2 [Source:HGNC Symbol;Acc:HGNC:19073] | 34241 | -0.150 | -0.0536 | No |
| 114 | U2SURP | U2 snRNP associated SURP domain containing [Source:HGNC Symbol;Acc:HGNC:30855] | 34412 | -0.153 | -0.0492 | No |
| 115 | HNRNPM | heterogeneous nuclear ribonucleoprotein M [Source:HGNC Symbol;Acc:HGNC:5046] | 34658 | -0.159 | -0.0464 | No |
| 116 | PRPF8 | pre-mRNA processing factor 8 [Source:HGNC Symbol;Acc:HGNC:17340] | 35220 | -0.172 | -0.0510 | No |
| 117 | DDX23 | DEAD-box helicase 23 [Source:HGNC Symbol;Acc:HGNC:17347] | 35258 | -0.173 | -0.0421 | No |
| 118 | DHX8 | DEAH-box helicase 8 [Source:HGNC Symbol;Acc:HGNC:2749] | 35966 | -0.193 | -0.0491 | No |
| 119 | PRPF38B | pre-mRNA processing factor 38B [Source:HGNC Symbol;Acc:HGNC:25512] | 36269 | -0.203 | -0.0453 | No |
| 120 | WBP11 | WW domain binding protein 11 [Source:HGNC Symbol;Acc:HGNC:16461] | 36280 | -0.203 | -0.0339 | No |
| 121 | SNRNP200 | small nuclear ribonucleoprotein U5 subunit 200 [Source:HGNC Symbol;Acc:HGNC:30859] | 36286 | -0.204 | -0.0224 | No |
| 122 | PRPF40A | pre-mRNA processing factor 40 homolog A [Source:HGNC Symbol;Acc:HGNC:16463] | 36815 | -0.226 | -0.0230 | No |
| 123 | SF3B1 | splicing factor 3b subunit 1 [Source:HGNC Symbol;Acc:HGNC:10768] | 37462 | -0.263 | -0.0245 | No |
| 124 | AQR | aquarius intron-binding spliceosomal factor [Source:HGNC Symbol;Acc:HGNC:29513] | 37783 | -0.289 | -0.0162 | No |
| 125 | NCBP1 | nuclear cap binding protein subunit 1 [Source:HGNC Symbol;Acc:HGNC:7658] | 37973 | -0.313 | -0.0032 | No |
| 126 | DDX46 | DEAD-box helicase 46 [Source:HGNC Symbol;Acc:HGNC:18681] | 37978 | -0.314 | 0.0146 | No |
| 127 | CDC40 | cell division cycle 40 [Source:HGNC Symbol;Acc:HGNC:17350] | 38147 | -0.346 | 0.0300 | No |
Table: GSEA details [plain text format]

  

Fig 2: KEGG\_SPLICEOSOME      
 Blue-Pink O' Gram in the Space of the Analyzed GeneSet

  

Fig 3: KEGG\_SPLICEOSOME: Random ES distribution      
 Gene set null distribution of ES for **KEGG\_SPLICEOSOME**

  
