# Supplementary material for: Simultaneous Presentation of Multiple Myeloma and Lung Cancer: Case Report and Gene Bioinformatics Analysis
Source: Front Oncol. 2022 Jun 13;12:859735. doi: 10.3389/fonc.2022.859735 (PMC9235397; doi:10.3389/fonc.2022.859735)
Supplement: Supplementary file 1 [file DataSheet_1.zip › The bioinformatic analysis of MM and lung cancer supplementary materials/Enrichment analysis/MECR/GSEA_4.1.0/LUAD TCGA/KEGG.Gsea.1639041756227/KEGG_TGF_BETA_SIGNALING_PATHWAY.html]

Details for gene set KEGG\_TGF\_BETA\_SIGNALING\_PATHWAY[GSEA]

|  || Dataset | ExpData\_collapsed\_to\_symbols.ENSG00000116353\_profile\_in\_ExpData.cls #ENSG00000116353 |
| Phenotype | ENSG00000116353\_profile\_in\_ExpData.cls#ENSG00000116353 |
| Upregulated in class | ENSG00000116353\_neg |
| GeneSet | KEGG\_TGF\_BETA\_SIGNALING\_PATHWAY |
| Enrichment Score (ES) | -0.50342935 |
| Normalized Enrichment Score (NES) | -2.0361388 |
| Nominal p-value | 0.0 |
| FDR q-value | 3.5548583E-4 |
| FWER p-Value | 0.006 |
Table: GSEA Results Summary

  

Fig 1: Enrichment plot: KEGG\_TGF\_BETA\_SIGNALING\_PATHWAY      
 Profile of the Running ES Score & Positions of GeneSet Members on the Rank Ordered List

  

| SYMBOL | TITLE | RANK IN GENE LIST | RANK METRIC SCORE | RUNNING ES | CORE ENRICHMENT || 1 | RBX1 | ring-box 1 [Source:HGNC Symbol;Acc:HGNC:9928] | 236 | 0.365 | 0.0295 | No |
| 2 | RPS6KB2 | ribosomal protein S6 kinase B2 [Source:HGNC Symbol;Acc:HGNC:10437] | 1294 | 0.248 | 0.0266 | No |
| 3 | MAPK3 | mitogen-activated protein kinase 3 [Source:HGNC Symbol;Acc:HGNC:6877] | 1645 | 0.226 | 0.0397 | No |
| 4 | GDF5 | growth differentiation factor 5 [Source:HGNC Symbol;Acc:HGNC:4220] | 2574 | 0.181 | 0.0336 | No |
| 5 | PPP2R1A | protein phosphatase 2 scaffold subunit Aalpha [Source:HGNC Symbol;Acc:HGNC:9302] | 4377 | 0.125 | -0.0002 | No |
| 6 | SKP1 | S-phase kinase associated protein 1 [Source:HGNC Symbol;Acc:HGNC:10899] | 5588 | 0.101 | -0.0213 | No |
| 7 | THBS3 | thrombospondin 3 [Source:HGNC Symbol;Acc:HGNC:11787] | 5659 | 0.099 | -0.0134 | No |
| 8 | BMP4 | bone morphogenetic protein 4 [Source:HGNC Symbol;Acc:HGNC:1071] | 6943 | 0.080 | -0.0383 | No |
| 9 | CUL1 | cullin 1 [Source:HGNC Symbol;Acc:HGNC:2551] | 7229 | 0.076 | -0.0381 | No |
| 10 | AMHR2 | anti-Mullerian hormone receptor type 2 [Source:HGNC Symbol;Acc:HGNC:465] | 7688 | 0.071 | -0.0429 | No |
| 11 | COMP | cartilage oligomeric matrix protein [Source:HGNC Symbol;Acc:HGNC:2227] | 9245 | 0.055 | -0.0772 | No |
| 12 | PPP2CA | protein phosphatase 2 catalytic subunit alpha [Source:HGNC Symbol;Acc:HGNC:9299] | 9403 | 0.054 | -0.0760 | No |
| 13 | E2F4 | E2F transcription factor 4 [Source:HGNC Symbol;Acc:HGNC:3118] | 9838 | 0.050 | -0.0822 | No |
| 14 | AMH | anti-Mullerian hormone [Source:HGNC Symbol;Acc:HGNC:464] | 10516 | 0.045 | -0.0951 | No |
| 15 | GDF7 | growth differentiation factor 7 [Source:HGNC Symbol;Acc:HGNC:4222] | 11253 | 0.039 | -0.1101 | No |
| 16 | RHOA | ras homolog family member A [Source:HGNC Symbol;Acc:HGNC:667] | 11437 | 0.037 | -0.1111 | No |
| 17 | INHBC | inhibin subunit beta C [Source:HGNC Symbol;Acc:HGNC:6068] | 11655 | 0.036 | -0.1132 | No |
| 18 | E2F5 | E2F transcription factor 5 [Source:HGNC Symbol;Acc:HGNC:3119] | 11958 | 0.033 | -0.1176 | No |
| 19 | SMURF1 | SMAD specific E3 ubiquitin protein ligase 1 [Source:HGNC Symbol;Acc:HGNC:16807] | 12786 | 0.027 | -0.1360 | No |
| 20 | LEFTY2 | left-right determination factor 2 [Source:HGNC Symbol;Acc:HGNC:3122] | 13286 | 0.024 | -0.1464 | No |
| 21 | ID3 | "inhibitor of DNA binding 3, HLH protein [Source:HGNC Symbol;Acc:HGNC:5362]" | 13729 | 0.021 | -0.1557 | No |
| 22 | PITX2 | paired like homeodomain 2 [Source:HGNC Symbol;Acc:HGNC:9005] | 14328 | 0.017 | -0.1692 | No |
| 23 | INHBB | inhibin subunit beta B [Source:HGNC Symbol;Acc:HGNC:6067] | 14454 | 0.016 | -0.1708 | No |
| 24 | NOG | noggin [Source:HGNC Symbol;Acc:HGNC:7866] | 14996 | 0.013 | -0.1833 | No |
| 25 | BMPR1B | bone morphogenetic protein receptor type 1B [Source:HGNC Symbol;Acc:HGNC:1077] | 15125 | 0.012 | -0.1854 | No |
| 26 | ID1 | "inhibitor of DNA binding 1, HLH protein [Source:HGNC Symbol;Acc:HGNC:5360]" | 16585 | 0.004 | -0.2222 | No |
| 27 | MYC | "MYC proto-oncogene, bHLH transcription factor [Source:HGNC Symbol;Acc:HGNC:7553]" | 16923 | 0.002 | -0.2307 | No |
| 28 | ID4 | "inhibitor of DNA binding 4, HLH protein [Source:HGNC Symbol;Acc:HGNC:5363]" | 18008 | -0.005 | -0.2578 | No |
| 29 | SMAD7 | SMAD family member 7 [Source:HGNC Symbol;Acc:HGNC:6773] | 18155 | -0.006 | -0.2610 | No |
| 30 | SKP1P2 | S-phase kinase associated protein 1 pseudogene 2 [Source:HGNC Symbol;Acc:HGNC:10900] | 19458 | -0.013 | -0.2928 | No |
| 31 | ACVR1 | activin A receptor type 1 [Source:HGNC Symbol;Acc:HGNC:171] | 19508 | -0.014 | -0.2928 | No |
| 32 | THBS4 | thrombospondin 4 [Source:HGNC Symbol;Acc:HGNC:11788] | 20600 | -0.020 | -0.3186 | No |
| 33 | SMAD6 | SMAD family member 6 [Source:HGNC Symbol;Acc:HGNC:6772] | 21342 | -0.025 | -0.3350 | No |
| 34 | CDKN2B | cyclin dependent kinase inhibitor 2B [Source:HGNC Symbol;Acc:HGNC:1788] | 21665 | -0.027 | -0.3406 | No |
| 35 | SMAD2 | SMAD family member 2 [Source:HGNC Symbol;Acc:HGNC:6768] | 22253 | -0.030 | -0.3526 | No |
| 36 | TGFB1 | transforming growth factor beta 1 [Source:HGNC Symbol;Acc:HGNC:11766] | 22340 | -0.031 | -0.3518 | No |
| 37 | BMP2 | bone morphogenetic protein 2 [Source:HGNC Symbol;Acc:HGNC:1069] | 23093 | -0.036 | -0.3675 | No |
| 38 | TFDP1 | transcription factor Dp-1 [Source:HGNC Symbol;Acc:HGNC:11749] | 24863 | -0.048 | -0.4080 | No |
| 39 | BMP7 | bone morphogenetic protein 7 [Source:HGNC Symbol;Acc:HGNC:1074] | 25274 | -0.051 | -0.4135 | No |
| 40 | TGFB2 | transforming growth factor beta 2 [Source:HGNC Symbol;Acc:HGNC:11768] | 26001 | -0.056 | -0.4266 | No |
| 41 | SMURF2 | SMAD specific E3 ubiquitin protein ligase 2 [Source:HGNC Symbol;Acc:HGNC:16809] | 27082 | -0.064 | -0.4479 | No |
| 42 | PPP2CB | protein phosphatase 2 catalytic subunit beta [Source:HGNC Symbol;Acc:HGNC:9300] | 27728 | -0.069 | -0.4576 | No |
| 43 | BMP8B | bone morphogenetic protein 8b [Source:HGNC Symbol;Acc:HGNC:1075] | 28206 | -0.073 | -0.4627 | No |
| 44 | GDF6 | growth differentiation factor 6 [Source:HGNC Symbol;Acc:HGNC:4221] | 28703 | -0.077 | -0.4678 | No |
| 45 | LEFTY1 | left-right determination factor 1 [Source:HGNC Symbol;Acc:HGNC:6552] | 28806 | -0.078 | -0.4628 | No |
| 46 | NODAL | nodal growth differentiation factor [Source:HGNC Symbol;Acc:HGNC:7865] | 28980 | -0.080 | -0.4595 | No |
| 47 | SMAD4 | SMAD family member 4 [Source:HGNC Symbol;Acc:HGNC:6770] | 29082 | -0.081 | -0.4542 | No |
| 48 | BMPR1A | bone morphogenetic protein receptor type 1A [Source:HGNC Symbol;Acc:HGNC:1076] | 29382 | -0.083 | -0.4538 | No |
| 49 | FST | follistatin [Source:HGNC Symbol;Acc:HGNC:3971] | 29400 | -0.083 | -0.4461 | No |
| 50 | SMAD9 | SMAD family member 9 [Source:HGNC Symbol;Acc:HGNC:6774] | 30256 | -0.092 | -0.4589 | No |
| 51 | ACVR2A | activin A receptor type 2A [Source:HGNC Symbol;Acc:HGNC:173] | 30423 | -0.094 | -0.4540 | No |
| 52 | ACVR2B | activin A receptor type 2B [Source:HGNC Symbol;Acc:HGNC:174] | 30713 | -0.097 | -0.4519 | No |
| 53 | CHRD | chordin [Source:HGNC Symbol;Acc:HGNC:1949] | 31640 | -0.108 | -0.4649 | No |
| 54 | BMP8A | bone morphogenetic protein 8a [Source:HGNC Symbol;Acc:HGNC:21650] | 32933 | -0.127 | -0.4855 | No |
| 55 | MAPK1 | mitogen-activated protein kinase 1 [Source:HGNC Symbol;Acc:HGNC:6871] | 33636 | -0.139 | -0.4900 | Yes |
| 56 | TNF | tumor necrosis factor [Source:HGNC Symbol;Acc:HGNC:11892] | 33732 | -0.140 | -0.4787 | Yes |
| 57 | ACVR1C | activin A receptor type 1C [Source:HGNC Symbol;Acc:HGNC:18123] | 33803 | -0.142 | -0.4667 | Yes |
| 58 | DCN | decorin [Source:HGNC Symbol;Acc:HGNC:2705] | 33827 | -0.142 | -0.4535 | Yes |
| 59 | BMP5 | bone morphogenetic protein 5 [Source:HGNC Symbol;Acc:HGNC:1072] | 33836 | -0.142 | -0.4399 | Yes |
| 60 | RPS6KB1 | ribosomal protein S6 kinase B1 [Source:HGNC Symbol;Acc:HGNC:10436] | 34494 | -0.155 | -0.4416 | Yes |
| 61 | PPP2R1B | protein phosphatase 2 scaffold subunit Abeta [Source:HGNC Symbol;Acc:HGNC:9303] | 34595 | -0.157 | -0.4289 | Yes |
| 62 | ID2 | inhibitor of DNA binding 2 [Source:HGNC Symbol;Acc:HGNC:5361] | 34691 | -0.159 | -0.4158 | Yes |
| 63 | CREBBP | CREB binding protein [Source:HGNC Symbol;Acc:HGNC:2348] | 34790 | -0.162 | -0.4026 | Yes |
| 64 | LTBP1 | latent transforming growth factor beta binding protein 1 [Source:HGNC Symbol;Acc:HGNC:6714] | 34948 | -0.165 | -0.3905 | Yes |
| 65 | SMAD1 | SMAD family member 1 [Source:HGNC Symbol;Acc:HGNC:6767] | 35267 | -0.173 | -0.3818 | Yes |
| 66 | SMAD3 | SMAD family member 3 [Source:HGNC Symbol;Acc:HGNC:6769] | 35395 | -0.176 | -0.3679 | Yes |
| 67 | ACVRL1 | activin A receptor like type 1 [Source:HGNC Symbol;Acc:HGNC:175] | 35429 | -0.177 | -0.3515 | Yes |
| 68 | RBL2 | RB transcriptional corepressor like 2 [Source:HGNC Symbol;Acc:HGNC:9894] | 35626 | -0.183 | -0.3388 | Yes |
| 69 | IFNG | interferon gamma [Source:HGNC Symbol;Acc:HGNC:5438] | 35652 | -0.183 | -0.3216 | Yes |
| 70 | SMAD5 | SMAD family member 5 [Source:HGNC Symbol;Acc:HGNC:6771] | 35735 | -0.186 | -0.3056 | Yes |
| 71 | TGFB3 | transforming growth factor beta 3 [Source:HGNC Symbol;Acc:HGNC:11769] | 35787 | -0.187 | -0.2887 | Yes |
| 72 | THBS2 | thrombospondin 2 [Source:HGNC Symbol;Acc:HGNC:11786] | 36117 | -0.198 | -0.2779 | Yes |
| 73 | TGFBR2 | transforming growth factor beta receptor 2 [Source:HGNC Symbol;Acc:HGNC:11773] | 36159 | -0.199 | -0.2596 | Yes |
| 74 | ZFYVE9 | zinc finger FYVE-type containing 9 [Source:HGNC Symbol;Acc:HGNC:6775] | 36215 | -0.201 | -0.2414 | Yes |
| 75 | INHBE | inhibin subunit beta E [Source:HGNC Symbol;Acc:HGNC:24029] | 36632 | -0.217 | -0.2309 | Yes |
| 76 | RBL1 | RB transcriptional corepressor like 1 [Source:HGNC Symbol;Acc:HGNC:9893] | 36716 | -0.220 | -0.2116 | Yes |
| 77 | TGFBR1 | transforming growth factor beta receptor 1 [Source:HGNC Symbol;Acc:HGNC:11772] | 36724 | -0.221 | -0.1903 | Yes |
| 78 | BMP6 | bone morphogenetic protein 6 [Source:HGNC Symbol;Acc:HGNC:1073] | 36882 | -0.229 | -0.1720 | Yes |
| 79 | THBS1 | thrombospondin 1 [Source:HGNC Symbol;Acc:HGNC:11785] | 37079 | -0.240 | -0.1537 | Yes |
| 80 | INHBA | inhibin subunit beta A [Source:HGNC Symbol;Acc:HGNC:6066] | 37343 | -0.256 | -0.1356 | Yes |
| 81 | EP300 | E1A binding protein p300 [Source:HGNC Symbol;Acc:HGNC:3373] | 37388 | -0.259 | -0.1115 | Yes |
| 82 | ZFYVE16 | zinc finger FYVE-type containing 16 [Source:HGNC Symbol;Acc:HGNC:20756] | 37578 | -0.272 | -0.0899 | Yes |
| 83 | BMPR2 | bone morphogenetic protein receptor type 2 [Source:HGNC Symbol;Acc:HGNC:1078] | 37775 | -0.288 | -0.0669 | Yes |
| 84 | SP1 | Sp1 transcription factor [Source:HGNC Symbol;Acc:HGNC:11205] | 38109 | -0.336 | -0.0427 | Yes |
| 85 | ROCK2 | Rho associated coiled-coil containing protein kinase 2 [Source:HGNC Symbol;Acc:HGNC:10252] | 38215 | -0.370 | -0.0094 | Yes |
| 86 | ROCK1 | Rho associated coiled-coil containing protein kinase 1 [Source:HGNC Symbol;Acc:HGNC:10251] | 38260 | -0.387 | 0.0271 | Yes |
Table: GSEA details [plain text format]

  

Fig 2: KEGG\_TGF\_BETA\_SIGNALING\_PATHWAY      
 Blue-Pink O' Gram in the Space of the Analyzed GeneSet

  

Fig 3: KEGG\_TGF\_BETA\_SIGNALING\_PATHWAY: Random ES distribution      
 Gene set null distribution of ES for **KEGG\_TGF\_BETA\_SIGNALING\_PATHWAY**

  
