# Supplementary material for: Simultaneous Presentation of Multiple Myeloma and Lung Cancer: Case Report and Gene Bioinformatics Analysis
Source: Front Oncol. 2022 Jun 13;12:859735. doi: 10.3389/fonc.2022.859735 (PMC9235397; doi:10.3389/fonc.2022.859735)
Supplement: Supplementary file 1 [file DataSheet_1.zip › The bioinformatic analysis of MM and lung cancer supplementary materials/Enrichment analysis/MECR/GSEA_4.1.0/LUAD TCGA/KEGG.Gsea.1639041756227/KEGG_T_CELL_RECEPTOR_SIGNALING_PATHWAY.html]

Details for gene set KEGG\_T\_CELL\_RECEPTOR\_SIGNALING\_PATHWAY[GSEA]

|  || Dataset | ExpData\_collapsed\_to\_symbols.ENSG00000116353\_profile\_in\_ExpData.cls #ENSG00000116353 |
| Phenotype | ENSG00000116353\_profile\_in\_ExpData.cls#ENSG00000116353 |
| Upregulated in class | ENSG00000116353\_neg |
| GeneSet | KEGG\_T\_CELL\_RECEPTOR\_SIGNALING\_PATHWAY |
| Enrichment Score (ES) | -0.5768203 |
| Normalized Enrichment Score (NES) | -2.4143016 |
| Nominal p-value | 0.0 |
| FDR q-value | 0.0 |
| FWER p-Value | 0.0 |
Table: GSEA Results Summary

  

Fig 1: Enrichment plot: KEGG\_T\_CELL\_RECEPTOR\_SIGNALING\_PATHWAY      
 Profile of the Running ES Score & Positions of GeneSet Members on the Rank Ordered List

  

| SYMBOL | TITLE | RANK IN GENE LIST | RANK METRIC SCORE | RUNNING ES | CORE ENRICHMENT || 1 | PIK3R2 | phosphoinositide-3-kinase regulatory subunit 2 [Source:HGNC Symbol;Acc:HGNC:8980] | 545 | 0.314 | 0.0047 | No |
| 2 | HRAS | "HRas proto-oncogene, GTPase [Source:HGNC Symbol;Acc:HGNC:5173]" | 1513 | 0.234 | -0.0061 | No |
| 3 | MAPK3 | mitogen-activated protein kinase 3 [Source:HGNC Symbol;Acc:HGNC:6877] | 1645 | 0.226 | 0.0040 | No |
| 4 | MAP2K2 | mitogen-activated protein kinase kinase 2 [Source:HGNC Symbol;Acc:HGNC:6842] | 1791 | 0.218 | 0.0132 | No |
| 5 | CDC42 | cell division cycle 42 [Source:HGNC Symbol;Acc:HGNC:1736] | 2158 | 0.198 | 0.0157 | No |
| 6 | NFATC4 | nuclear factor of activated T cells 4 [Source:HGNC Symbol;Acc:HGNC:7778] | 2871 | 0.169 | 0.0076 | No |
| 7 | AKT1 | AKT serine/threonine kinase 1 [Source:HGNC Symbol;Acc:HGNC:391] | 3179 | 0.158 | 0.0091 | No |
| 8 | MAP2K7 | mitogen-activated protein kinase kinase 7 [Source:HGNC Symbol;Acc:HGNC:6847] | 3849 | 0.138 | 0.0003 | No |
| 9 | PAK4 | p21 (RAC1) activated kinase 4 [Source:HGNC Symbol;Acc:HGNC:16059] | 4914 | 0.113 | -0.0202 | No |
| 10 | JUN | "Jun proto-oncogene, AP-1 transcription factor subunit [Source:HGNC Symbol;Acc:HGNC:6204]" | 5045 | 0.110 | -0.0169 | No |
| 11 | PPP3CA | protein phosphatase 3 catalytic subunit alpha [Source:HGNC Symbol;Acc:HGNC:9314] | 5070 | 0.110 | -0.0110 | No |
| 12 | NFKBIE | NFKB inhibitor epsilon [Source:HGNC Symbol;Acc:HGNC:7799] | 5113 | 0.109 | -0.0056 | No |
| 13 | NFKBIB | NFKB inhibitor beta [Source:HGNC Symbol;Acc:HGNC:7798] | 5484 | 0.102 | -0.0090 | No |
| 14 | PAK6 | p21 (RAC1) activated kinase 6 [Source:HGNC Symbol;Acc:HGNC:16061] | 5767 | 0.098 | -0.0104 | No |
| 15 | RELA | "RELA proto-oncogene, NF-kB subunit [Source:HGNC Symbol;Acc:HGNC:9955]" | 6022 | 0.093 | -0.0113 | No |
| 16 | MAPK13 | mitogen-activated protein kinase 13 [Source:HGNC Symbol;Acc:HGNC:6875] | 6377 | 0.088 | -0.0152 | No |
| 17 | CHP1 | calcineurin like EF-hand protein 1 [Source:HGNC Symbol;Acc:HGNC:17433] | 7211 | 0.077 | -0.0319 | No |
| 18 | IL4 | interleukin 4 [Source:HGNC Symbol;Acc:HGNC:6014] | 7378 | 0.075 | -0.0317 | No |
| 19 | NFKBIA | NFKB inhibitor alpha [Source:HGNC Symbol;Acc:HGNC:7797] | 7696 | 0.071 | -0.0356 | No |
| 20 | MAPK9 | mitogen-activated protein kinase 9 [Source:HGNC Symbol;Acc:HGNC:6886] | 8347 | 0.064 | -0.0484 | No |
| 21 | NCK2 | NCK adaptor protein 2 [Source:HGNC Symbol;Acc:HGNC:7665] | 8361 | 0.064 | -0.0449 | No |
| 22 | RAF1 | "Raf-1 proto-oncogene, serine/threonine kinase [Source:HGNC Symbol;Acc:HGNC:9829]" | 8635 | 0.061 | -0.0483 | No |
| 23 | PIK3R3 | phosphoinositide-3-kinase regulatory subunit 3 [Source:HGNC Symbol;Acc:HGNC:8981] | 9028 | 0.057 | -0.0549 | No |
| 24 | NFATC1 | nuclear factor of activated T cells 1 [Source:HGNC Symbol;Acc:HGNC:7775] | 9184 | 0.056 | -0.0555 | No |
| 25 | MAPK12 | mitogen-activated protein kinase 12 [Source:HGNC Symbol;Acc:HGNC:6874] | 9265 | 0.055 | -0.0543 | No |
| 26 | IKBKG | inhibitor of nuclear factor kappa B kinase regulatory subunit gamma [Source:HGNC Symbol;Acc:HGNC:5961] | 9304 | 0.055 | -0.0520 | No |
| 27 | MAPK11 | mitogen-activated protein kinase 11 [Source:HGNC Symbol;Acc:HGNC:6873] | 9370 | 0.054 | -0.0505 | No |
| 28 | CSF2 | colony stimulating factor 2 [Source:HGNC Symbol;Acc:HGNC:2434] | 9888 | 0.050 | -0.0607 | No |
| 29 | PPP3R1 | "protein phosphatase 3 regulatory subunit B, alpha [Source:HGNC Symbol;Acc:HGNC:9317]" | 10562 | 0.044 | -0.0752 | No |
| 30 | AKT2 | AKT serine/threonine kinase 2 [Source:HGNC Symbol;Acc:HGNC:392] | 10579 | 0.044 | -0.0730 | No |
| 31 | RHOA | ras homolog family member A [Source:HGNC Symbol;Acc:HGNC:667] | 11437 | 0.037 | -0.0926 | No |
| 32 | CHP2 | calcineurin like EF-hand protein 2 [Source:HGNC Symbol;Acc:HGNC:24927] | 13971 | 0.020 | -0.1561 | No |
| 33 | CDK4 | cyclin dependent kinase 4 [Source:HGNC Symbol;Acc:HGNC:1773] | 15379 | 0.011 | -0.1913 | No |
| 34 | CBLC | Cbl proto-oncogene C [Source:HGNC Symbol;Acc:HGNC:15961] | 16554 | 0.004 | -0.2210 | No |
| 35 | PAK5 | p21 (RAC1) activated kinase 5 [Source:HGNC Symbol;Acc:HGNC:15916] | 17436 | -0.002 | -0.2434 | No |
| 36 | BCL10 | BCL10 immune signaling adaptor [Source:HGNC Symbol;Acc:HGNC:989] | 18926 | -0.010 | -0.2807 | No |
| 37 | LAT | linker for activation of T cells [Source:HGNC Symbol;Acc:HGNC:18874] | 20384 | -0.019 | -0.3168 | No |
| 38 | IL2 | interleukin 2 [Source:HGNC Symbol;Acc:HGNC:6001] | 20777 | -0.021 | -0.3255 | No |
| 39 | IKBKB | inhibitor of nuclear factor kappa B kinase subunit beta [Source:HGNC Symbol;Acc:HGNC:5960] | 21393 | -0.025 | -0.3397 | No |
| 40 | TEC | tec protein tyrosine kinase [Source:HGNC Symbol;Acc:HGNC:11719] | 22189 | -0.030 | -0.3582 | No |
| 41 | FOS | "Fos proto-oncogene, AP-1 transcription factor subunit [Source:HGNC Symbol;Acc:HGNC:3796]" | 23563 | -0.039 | -0.3909 | No |
| 42 | PTPN6 | protein tyrosine phosphatase non-receptor type 6 [Source:HGNC Symbol;Acc:HGNC:9658] | 23581 | -0.039 | -0.3890 | No |
| 43 | VAV2 | vav guanine nucleotide exchange factor 2 [Source:HGNC Symbol;Acc:HGNC:12658] | 24578 | -0.046 | -0.4117 | No |
| 44 | MAPK14 | mitogen-activated protein kinase 14 [Source:HGNC Symbol;Acc:HGNC:6876] | 25018 | -0.049 | -0.4200 | No |
| 45 | IL5 | interleukin 5 [Source:HGNC Symbol;Acc:HGNC:6016] | 25260 | -0.050 | -0.4231 | No |
| 46 | CARD11 | caspase recruitment domain family member 11 [Source:HGNC Symbol;Acc:HGNC:16393] | 27575 | -0.068 | -0.4781 | No |
| 47 | NCK1 | NCK adaptor protein 1 [Source:HGNC Symbol;Acc:HGNC:7664] | 28533 | -0.076 | -0.4980 | No |
| 48 | VAV3 | vav guanine nucleotide exchange factor 3 [Source:HGNC Symbol;Acc:HGNC:12659] | 29000 | -0.080 | -0.5052 | No |
| 49 | PAK1 | p21 (RAC1) activated kinase 1 [Source:HGNC Symbol;Acc:HGNC:8590] | 29530 | -0.085 | -0.5136 | No |
| 50 | PLCG1 | phospholipase C gamma 1 [Source:HGNC Symbol;Acc:HGNC:9065] | 30759 | -0.098 | -0.5391 | No |
| 51 | PAK3 | p21 (RAC1) activated kinase 3 [Source:HGNC Symbol;Acc:HGNC:8592] | 31145 | -0.102 | -0.5429 | No |
| 52 | PIK3CD | "phosphatidylinositol-4,5-bisphosphate 3-kinase catalytic subunit delta [Source:HGNC Symbol;Acc:HGNC:8977]" | 31859 | -0.111 | -0.5545 | No |
| 53 | MAP3K14 | mitogen-activated protein kinase kinase kinase 14 [Source:HGNC Symbol;Acc:HGNC:6853] | 31902 | -0.112 | -0.5489 | No |
| 54 | PPP3R2 | "protein phosphatase 3 regulatory subunit B, beta [Source:HGNC Symbol;Acc:HGNC:9318]" | 32695 | -0.123 | -0.5618 | No |
| 55 | PPP3CB | protein phosphatase 3 catalytic subunit beta [Source:HGNC Symbol;Acc:HGNC:9315] | 33217 | -0.131 | -0.5673 | No |
| 56 | PDPK1 | 3-phosphoinositide dependent protein kinase 1 [Source:HGNC Symbol;Acc:HGNC:8816] | 33314 | -0.133 | -0.5618 | No |
| 57 | MAPK1 | mitogen-activated protein kinase 1 [Source:HGNC Symbol;Acc:HGNC:6871] | 33636 | -0.139 | -0.5618 | No |
| 58 | TNF | tumor necrosis factor [Source:HGNC Symbol;Acc:HGNC:11892] | 33732 | -0.140 | -0.5559 | No |
| 59 | NRAS | "NRAS proto-oncogene, GTPase [Source:HGNC Symbol;Acc:HGNC:7989]" | 34553 | -0.156 | -0.5676 | Yes |
| 60 | SOS2 | SOS Ras/Rho guanine nucleotide exchange factor 2 [Source:HGNC Symbol;Acc:HGNC:11188] | 34598 | -0.157 | -0.5594 | Yes |
| 61 | MAP3K7 | mitogen-activated protein kinase kinase kinase 7 [Source:HGNC Symbol;Acc:HGNC:6859] | 34786 | -0.162 | -0.5545 | Yes |
| 62 | PIK3R1 | phosphoinositide-3-kinase regulatory subunit 1 [Source:HGNC Symbol;Acc:HGNC:8979] | 34788 | -0.162 | -0.5450 | Yes |
| 63 | VAV1 | vav guanine nucleotide exchange factor 1 [Source:HGNC Symbol;Acc:HGNC:12657] | 34853 | -0.163 | -0.5369 | Yes |
| 64 | PIK3CB | "phosphatidylinositol-4,5-bisphosphate 3-kinase catalytic subunit beta [Source:HGNC Symbol;Acc:HGNC:8976]" | 34871 | -0.164 | -0.5277 | Yes |
| 65 | RASGRP1 | RAS guanyl releasing protein 1 [Source:HGNC Symbol;Acc:HGNC:9878] | 34945 | -0.165 | -0.5197 | Yes |
| 66 | PDCD1 | programmed cell death 1 [Source:HGNC Symbol;Acc:HGNC:8760] | 34965 | -0.165 | -0.5104 | Yes |
| 67 | DLG1 | discs large MAGUK scaffold protein 1 [Source:HGNC Symbol;Acc:HGNC:2900] | 34972 | -0.166 | -0.5007 | Yes |
| 68 | ZAP70 | zeta chain of T cell receptor associated protein kinase 70 [Source:HGNC Symbol;Acc:HGNC:12858] | 34981 | -0.166 | -0.4911 | Yes |
| 69 | CD3D | CD3d molecule [Source:HGNC Symbol;Acc:HGNC:1673] | 35025 | -0.167 | -0.4823 | Yes |
| 70 | CD40LG | CD40 ligand [Source:HGNC Symbol;Acc:HGNC:11935] | 35185 | -0.171 | -0.4762 | Yes |
| 71 | MAP2K1 | mitogen-activated protein kinase kinase 1 [Source:HGNC Symbol;Acc:HGNC:6840] | 35210 | -0.171 | -0.4667 | Yes |
| 72 | MAP3K8 | mitogen-activated protein kinase kinase kinase 8 [Source:HGNC Symbol;Acc:HGNC:6860] | 35251 | -0.172 | -0.4574 | Yes |
| 73 | PRKCQ | protein kinase C theta [Source:HGNC Symbol;Acc:HGNC:9410] | 35304 | -0.174 | -0.4484 | Yes |
| 74 | IFNG | interferon gamma [Source:HGNC Symbol;Acc:HGNC:5438] | 35652 | -0.183 | -0.4464 | Yes |
| 75 | CD3E | CD3e molecule [Source:HGNC Symbol;Acc:HGNC:1674] | 35751 | -0.186 | -0.4379 | Yes |
| 76 | PPP3CC | protein phosphatase 3 catalytic subunit gamma [Source:HGNC Symbol;Acc:HGNC:9316] | 35754 | -0.186 | -0.4269 | Yes |
| 77 | MALT1 | MALT1 paracaspase [Source:HGNC Symbol;Acc:HGNC:6819] | 35793 | -0.188 | -0.4167 | Yes |
| 78 | CBLB | Cbl proto-oncogene B [Source:HGNC Symbol;Acc:HGNC:1542] | 35958 | -0.192 | -0.4095 | Yes |
| 79 | CD8B | CD8b molecule [Source:HGNC Symbol;Acc:HGNC:1707] | 36153 | -0.199 | -0.4026 | Yes |
| 80 | NFKB1 | nuclear factor kappa B subunit 1 [Source:HGNC Symbol;Acc:HGNC:7794] | 36367 | -0.207 | -0.3958 | Yes |
| 81 | CD247 | CD247 molecule [Source:HGNC Symbol;Acc:HGNC:1677] | 36507 | -0.211 | -0.3868 | Yes |
| 82 | GRB2 | growth factor receptor bound protein 2 [Source:HGNC Symbol;Acc:HGNC:4566] | 36516 | -0.212 | -0.3744 | Yes |
| 83 | CD8A | CD8a molecule [Source:HGNC Symbol;Acc:HGNC:1706] | 36547 | -0.213 | -0.3625 | Yes |
| 84 | NFATC2 | nuclear factor of activated T cells 2 [Source:HGNC Symbol;Acc:HGNC:7776] | 36732 | -0.221 | -0.3541 | Yes |
| 85 | ICOS | inducible T cell costimulator [Source:HGNC Symbol;Acc:HGNC:5351] | 37068 | -0.239 | -0.3484 | Yes |
| 86 | LCK | "LCK proto-oncogene, Src family tyrosine kinase [Source:HGNC Symbol;Acc:HGNC:6524]" | 37085 | -0.240 | -0.3346 | Yes |
| 87 | FYN | "FYN proto-oncogene, Src family tyrosine kinase [Source:HGNC Symbol;Acc:HGNC:4037]" | 37102 | -0.242 | -0.3207 | Yes |
| 88 | CD4 | CD4 molecule [Source:HGNC Symbol;Acc:HGNC:1678] | 37205 | -0.248 | -0.3085 | Yes |
| 89 | IL10 | interleukin 10 [Source:HGNC Symbol;Acc:HGNC:5962] | 37315 | -0.254 | -0.2962 | Yes |
| 90 | CTLA4 | cytotoxic T-lymphocyte associated protein 4 [Source:HGNC Symbol;Acc:HGNC:2505] | 37400 | -0.259 | -0.2830 | Yes |
| 91 | PIK3CA | "phosphatidylinositol-4,5-bisphosphate 3-kinase catalytic subunit alpha [Source:HGNC Symbol;Acc:HGNC:8975]" | 37556 | -0.270 | -0.2709 | Yes |
| 92 | GSK3B | glycogen synthase kinase 3 beta [Source:HGNC Symbol;Acc:HGNC:4617] | 37592 | -0.274 | -0.2556 | Yes |
| 93 | PAK2 | p21 (RAC1) activated kinase 2 [Source:HGNC Symbol;Acc:HGNC:8591] | 37627 | -0.276 | -0.2400 | Yes |
| 94 | KRAS | "KRAS proto-oncogene, GTPase [Source:HGNC Symbol;Acc:HGNC:6407]" | 37629 | -0.277 | -0.2236 | Yes |
| 95 | NFAT5 | nuclear factor of activated T cells 5 [Source:HGNC Symbol;Acc:HGNC:7774] | 37631 | -0.277 | -0.2072 | Yes |
| 96 | NFATC3 | nuclear factor of activated T cells 3 [Source:HGNC Symbol;Acc:HGNC:7777] | 37672 | -0.281 | -0.1916 | Yes |
| 97 | GRAP2 | GRB2 related adaptor protein 2 [Source:HGNC Symbol;Acc:HGNC:4563] | 37728 | -0.285 | -0.1760 | Yes |
| 98 | CHUK | component of inhibitor of nuclear factor kappa B kinase complex [Source:HGNC Symbol;Acc:HGNC:1974] | 37762 | -0.287 | -0.1598 | Yes |
| 99 | CD28 | CD28 molecule [Source:HGNC Symbol;Acc:HGNC:1653] | 37779 | -0.289 | -0.1431 | Yes |
| 100 | LCP2 | lymphocyte cytosolic protein 2 [Source:HGNC Symbol;Acc:HGNC:6529] | 37904 | -0.304 | -0.1283 | Yes |
| 101 | CD3G | CD3g molecule [Source:HGNC Symbol;Acc:HGNC:1675] | 37917 | -0.306 | -0.1104 | Yes |
| 102 | PIK3R5 | phosphoinositide-3-kinase regulatory subunit 5 [Source:HGNC Symbol;Acc:HGNC:30035] | 37956 | -0.310 | -0.0930 | Yes |
| 103 | ITK | IL2 inducible T cell kinase [Source:HGNC Symbol;Acc:HGNC:6171] | 38024 | -0.320 | -0.0757 | Yes |
| 104 | CBL | Cbl proto-oncogene [Source:HGNC Symbol;Acc:HGNC:1541] | 38098 | -0.332 | -0.0578 | Yes |
| 105 | SOS1 | SOS Ras/Rac guanine nucleotide exchange factor 1 [Source:HGNC Symbol;Acc:HGNC:11187] | 38132 | -0.341 | -0.0384 | Yes |
| 106 | AKT3 | AKT serine/threonine kinase 3 [Source:HGNC Symbol;Acc:HGNC:393] | 38196 | -0.362 | -0.0186 | Yes |
| 107 | PTPRC | protein tyrosine phosphatase receptor type C [Source:HGNC Symbol;Acc:HGNC:9666] | 38216 | -0.370 | 0.0029 | Yes |
| 108 | PIK3CG | "phosphatidylinositol-4,5-bisphosphate 3-kinase catalytic subunit gamma [Source:HGNC Symbol;Acc:HGNC:8978]" | 38302 | -0.426 | 0.0260 | Yes |
Table: GSEA details [plain text format]

  

Fig 2: KEGG\_T\_CELL\_RECEPTOR\_SIGNALING\_PATHWAY      
 Blue-Pink O' Gram in the Space of the Analyzed GeneSet

  

Fig 3: KEGG\_T\_CELL\_RECEPTOR\_SIGNALING\_PATHWAY: Random ES distribution      
 Gene set null distribution of ES for **KEGG\_T\_CELL\_RECEPTOR\_SIGNALING\_PATHWAY**

  
