# Supplementary material for: Simultaneous Presentation of Multiple Myeloma and Lung Cancer: Case Report and Gene Bioinformatics Analysis
Source: Front Oncol. 2022 Jun 13;12:859735. doi: 10.3389/fonc.2022.859735 (PMC9235397; doi:10.3389/fonc.2022.859735)
Supplement: Supplementary file 1 [file DataSheet_1.zip › The bioinformatic analysis of MM and lung cancer supplementary materials/Enrichment analysis/MECR/GSEA_4.1.0/LUAD TCGA/KEGG.Gsea.1639041756227/KEGG_VALINE_LEUCINE_AND_ISOLEUCINE_DEGRADATION.html]

Details for gene set KEGG\_VALINE\_LEUCINE\_AND\_ISOLEUCINE\_DEGRADATION[GSEA]

|  || Dataset | ExpData\_collapsed\_to\_symbols.ENSG00000116353\_profile\_in\_ExpData.cls #ENSG00000116353 |
| Phenotype | ENSG00000116353\_profile\_in\_ExpData.cls#ENSG00000116353 |
| Upregulated in class | ENSG00000116353\_pos |
| GeneSet | KEGG\_VALINE\_LEUCINE\_AND\_ISOLEUCINE\_DEGRADATION |
| Enrichment Score (ES) | 0.5858354 |
| Normalized Enrichment Score (NES) | 2.097366 |
| Nominal p-value | 0.0 |
| FDR q-value | 1.7201006E-4 |
| FWER p-Value | 0.002 |
Table: GSEA Results Summary

  

Fig 1: Enrichment plot: KEGG\_VALINE\_LEUCINE\_AND\_ISOLEUCINE\_DEGRADATION      
 Profile of the Running ES Score & Positions of GeneSet Members on the Rank Ordered List

  

| SYMBOL | TITLE | RANK IN GENE LIST | RANK METRIC SCORE | RUNNING ES | CORE ENRICHMENT || 1 | HMGCL | 3-hydroxy-3-methylglutaryl-CoA lyase [Source:HGNC Symbol;Acc:HGNC:5005] | 8 | 0.505 | 0.0869 | Yes |
| 2 | ECHS1 | "enoyl-CoA hydratase, short chain 1 [Source:HGNC Symbol;Acc:HGNC:3151]" | 238 | 0.364 | 0.1439 | Yes |
| 3 | HADH | hydroxyacyl-CoA dehydrogenase [Source:HGNC Symbol;Acc:HGNC:4799] | 931 | 0.276 | 0.1738 | Yes |
| 4 | ACAA1 | acetyl-CoA acyltransferase 1 [Source:HGNC Symbol;Acc:HGNC:82] | 1003 | 0.270 | 0.2185 | Yes |
| 5 | MCEE | methylmalonyl-CoA epimerase [Source:HGNC Symbol;Acc:HGNC:16732] | 1055 | 0.266 | 0.2630 | Yes |
| 6 | HIBADH | 3-hydroxyisobutyrate dehydrogenase [Source:HGNC Symbol;Acc:HGNC:4907] | 1066 | 0.265 | 0.3085 | Yes |
| 7 | ACADS | acyl-CoA dehydrogenase short chain [Source:HGNC Symbol;Acc:HGNC:90] | 1276 | 0.250 | 0.3462 | Yes |
| 8 | BCAT2 | branched chain amino acid transaminase 2 [Source:HGNC Symbol;Acc:HGNC:977] | 1762 | 0.220 | 0.3718 | Yes |
| 9 | ALDH7A1 | aldehyde dehydrogenase 7 family member A1 [Source:HGNC Symbol;Acc:HGNC:877] | 2018 | 0.205 | 0.4007 | Yes |
| 10 | HSD17B10 | hydroxysteroid 17-beta dehydrogenase 10 [Source:HGNC Symbol;Acc:HGNC:4800] | 2183 | 0.197 | 0.4305 | Yes |
| 11 | BCKDHA | branched chain keto acid dehydrogenase E1 subunit alpha [Source:HGNC Symbol;Acc:HGNC:986] | 2239 | 0.195 | 0.4628 | Yes |
| 12 | PCCB | propionyl-CoA carboxylase subunit beta [Source:HGNC Symbol;Acc:HGNC:8654] | 2848 | 0.170 | 0.4766 | Yes |
| 13 | MCCC1 | methylcrotonoyl-CoA carboxylase 1 [Source:HGNC Symbol;Acc:HGNC:6936] | 4349 | 0.125 | 0.4600 | Yes |
| 14 | IVD | isovaleryl-CoA dehydrogenase [Source:HGNC Symbol;Acc:HGNC:6186] | 4585 | 0.120 | 0.4747 | Yes |
| 15 | ALDH2 | aldehyde dehydrogenase 2 family member [Source:HGNC Symbol;Acc:HGNC:404] | 4910 | 0.113 | 0.4859 | Yes |
| 16 | ALDH3A2 | aldehyde dehydrogenase 3 family member A2 [Source:HGNC Symbol;Acc:HGNC:403] | 5202 | 0.108 | 0.4970 | Yes |
| 17 | HIBCH | 3-hydroxyisobutyryl-CoA hydrolase [Source:HGNC Symbol;Acc:HGNC:4908] | 5246 | 0.107 | 0.5143 | Yes |
| 18 | HADHB | hydroxyacyl-CoA dehydrogenase trifunctional multienzyme complex subunit beta [Source:HGNC Symbol;Acc:HGNC:4803] | 5537 | 0.101 | 0.5244 | Yes |
| 19 | ACAD8 | acyl-CoA dehydrogenase family member 8 [Source:HGNC Symbol;Acc:HGNC:87] | 5869 | 0.096 | 0.5325 | Yes |
| 20 | ALDH6A1 | aldehyde dehydrogenase 6 family member A1 [Source:HGNC Symbol;Acc:HGNC:7179] | 5939 | 0.095 | 0.5471 | Yes |
| 21 | BCKDHB | branched chain keto acid dehydrogenase E1 subunit beta [Source:HGNC Symbol;Acc:HGNC:987] | 6067 | 0.092 | 0.5598 | Yes |
| 22 | ACAT1 | acetyl-CoA acetyltransferase 1 [Source:HGNC Symbol;Acc:HGNC:93] | 6430 | 0.087 | 0.5656 | Yes |
| 23 | PCCA | propionyl-CoA carboxylase subunit alpha [Source:HGNC Symbol;Acc:HGNC:8653] | 6502 | 0.086 | 0.5786 | Yes |
| 24 | MMUT | methylmalonyl-CoA mutase [Source:HGNC Symbol;Acc:HGNC:7526] | 7532 | 0.073 | 0.5649 | Yes |
| 25 | OXCT2 | 3-oxoacid CoA-transferase 2 [Source:HGNC Symbol;Acc:HGNC:18606] | 7577 | 0.072 | 0.5762 | Yes |
| 26 | ALDH9A1 | aldehyde dehydrogenase 9 family member A1 [Source:HGNC Symbol;Acc:HGNC:412] | 7785 | 0.070 | 0.5830 | Yes |
| 27 | AUH | AU RNA binding methylglutaconyl-CoA hydratase [Source:HGNC Symbol;Acc:HGNC:890] | 8122 | 0.066 | 0.5858 | Yes |
| 28 | HADHA | hydroxyacyl-CoA dehydrogenase trifunctional multienzyme complex subunit alpha [Source:HGNC Symbol;Acc:HGNC:4801] | 9756 | 0.051 | 0.5530 | No |
| 29 | ACADM | acyl-CoA dehydrogenase medium chain [Source:HGNC Symbol;Acc:HGNC:89] | 11264 | 0.039 | 0.5213 | No |
| 30 | DBT | dihydrolipoamide branched chain transacylase E2 [Source:HGNC Symbol;Acc:HGNC:2698] | 11887 | 0.034 | 0.5114 | No |
| 31 | ACADSB | acyl-CoA dehydrogenase short/branched chain [Source:HGNC Symbol;Acc:HGNC:91] | 12023 | 0.033 | 0.5136 | No |
| 32 | MCCC2 | methylcrotonoyl-CoA carboxylase 2 [Source:HGNC Symbol;Acc:HGNC:6937] | 18156 | -0.006 | 0.3585 | No |
| 33 | ABAT | 4-aminobutyrate aminotransferase [Source:HGNC Symbol;Acc:HGNC:23] | 19169 | -0.012 | 0.3348 | No |
| 34 | ACAT2 | acetyl-CoA acetyltransferase 2 [Source:HGNC Symbol;Acc:HGNC:94] | 19909 | -0.016 | 0.3188 | No |
| 35 | ALDH1B1 | aldehyde dehydrogenase 1 family member B1 [Source:HGNC Symbol;Acc:HGNC:407] | 21379 | -0.025 | 0.2857 | No |
| 36 | EHHADH | enoyl-CoA hydratase and 3-hydroxyacyl CoA dehydrogenase [Source:HGNC Symbol;Acc:HGNC:3247] | 23047 | -0.035 | 0.2494 | No |
| 37 | BCAT1 | branched chain amino acid transaminase 1 [Source:HGNC Symbol;Acc:HGNC:976] | 24071 | -0.042 | 0.2307 | No |
| 38 | HMGCS2 | 3-hydroxy-3-methylglutaryl-CoA synthase 2 [Source:HGNC Symbol;Acc:HGNC:5008] | 24319 | -0.044 | 0.2319 | No |
| 39 | DLD | dihydrolipoamide dehydrogenase [Source:HGNC Symbol;Acc:HGNC:2898] | 25309 | -0.051 | 0.2155 | No |
| 40 | OXCT1 | 3-oxoacid CoA-transferase 1 [Source:HGNC Symbol;Acc:HGNC:8527] | 29565 | -0.085 | 0.1219 | No |
| 41 | ACAA2 | acetyl-CoA acyltransferase 2 [Source:HGNC Symbol;Acc:HGNC:83] | 34923 | -0.164 | 0.0139 | No |
| 42 | HMGCS1 | 3-hydroxy-3-methylglutaryl-CoA synthase 1 [Source:HGNC Symbol;Acc:HGNC:5007] | 35221 | -0.172 | 0.0359 | No |
| 43 | IL4I1 | interleukin 4 induced 1 [Source:HGNC Symbol;Acc:HGNC:19094] | 36098 | -0.197 | 0.0476 | No |
| 44 | AOX1 | aldehyde oxidase 1 [Source:HGNC Symbol;Acc:HGNC:553] | 36190 | -0.200 | 0.0798 | No |
Table: GSEA details [plain text format]

  

Fig 2: KEGG\_VALINE\_LEUCINE\_AND\_ISOLEUCINE\_DEGRADATION      
 Blue-Pink O' Gram in the Space of the Analyzed GeneSet

  

Fig 3: KEGG\_VALINE\_LEUCINE\_AND\_ISOLEUCINE\_DEGRADATION: Random ES distribution      
 Gene set null distribution of ES for **KEGG\_VALINE\_LEUCINE\_AND\_ISOLEUCINE\_DEGRADATION**

  
