# Supplementary figures and images for: Simultaneous Presentation of Multiple Myeloma and Lung Cancer: Case Report and Gene Bioinformatics Analysis
Source: Front Oncol. 2022 Jun 13;12:859735. doi: 10.3389/fonc.2022.859735 (PMC9235397; doi:10.3389/fonc.2022.859735)

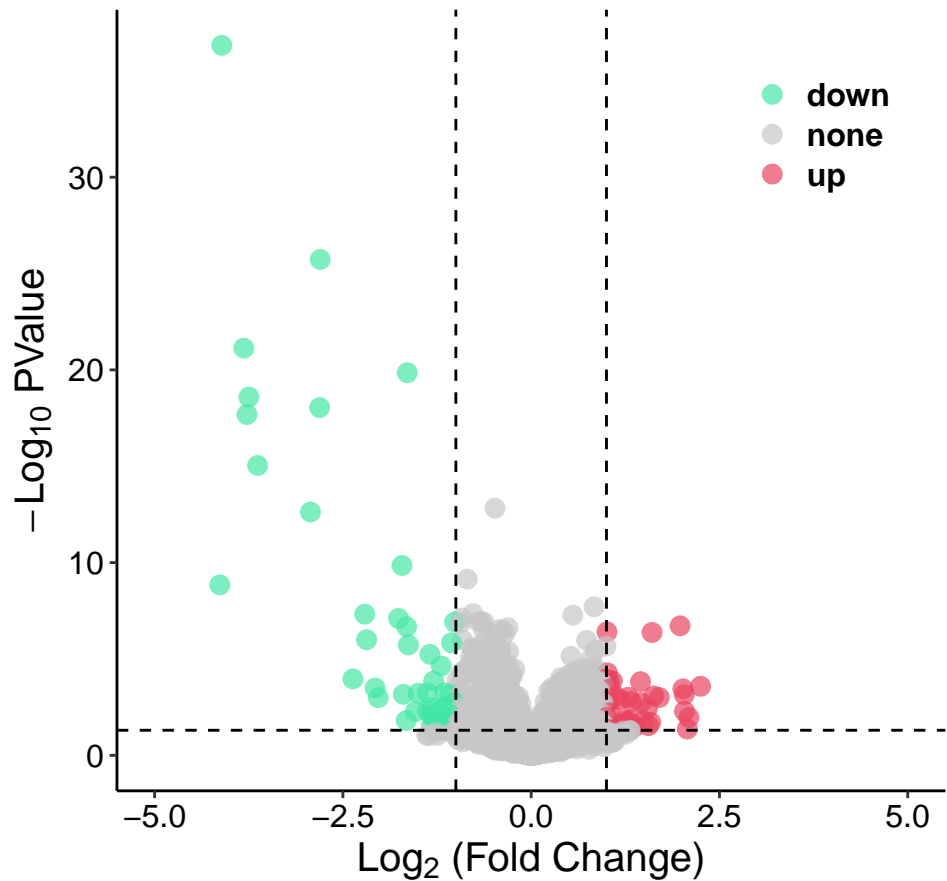

Supplement: Supplementary file 1 [file DataSheet_1.zip › The bioinformatic analysis of MM and lung cancer supplementary materials/Different expression genes analysis/GEO 7╕÷MM/GSE13591.pdf]

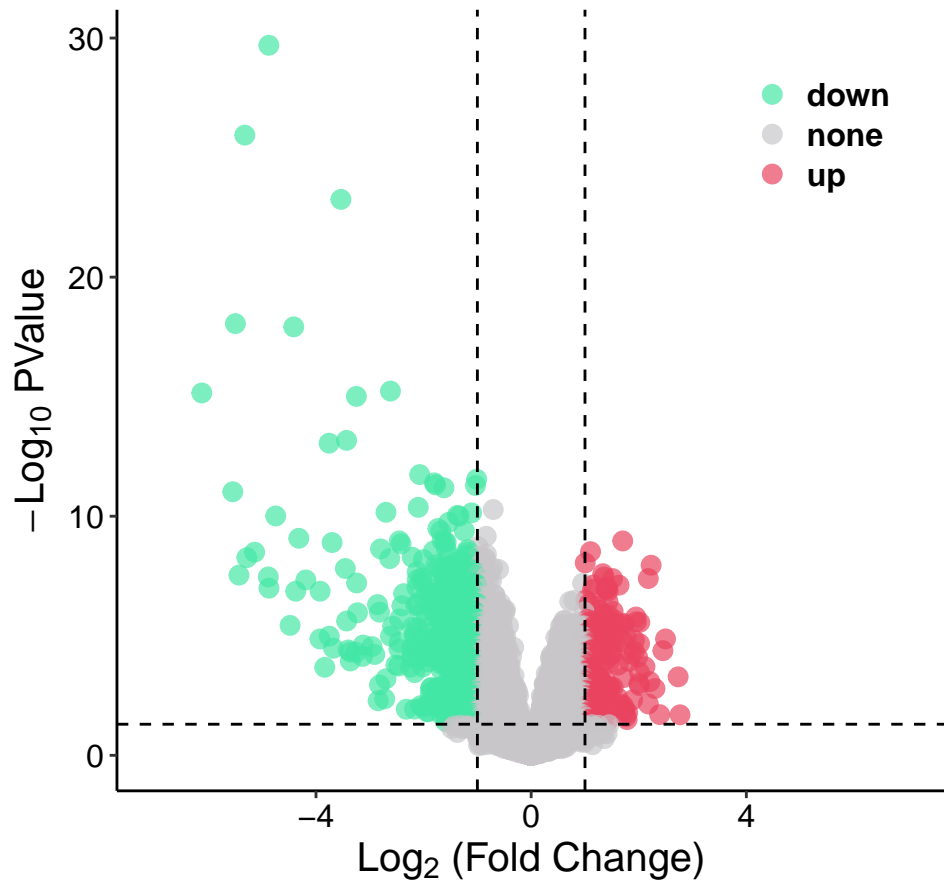

Supplement: Supplementary file 1 [file DataSheet_1.zip › The bioinformatic analysis of MM and lung cancer supplementary materials/Different expression genes analysis/GEO 7╕÷MM/GSE16558.pdf]

# GSE2658&GSE5900

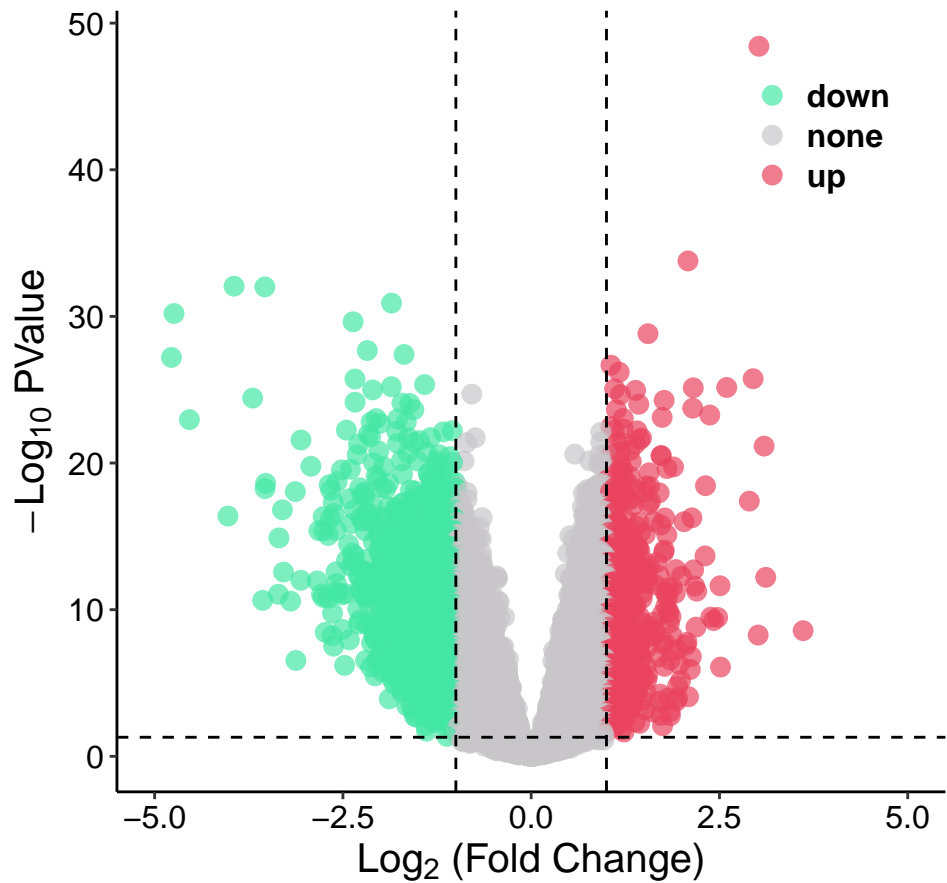

Supplement: Supplementary file 1 [file DataSheet_1.zip › The bioinformatic analysis of MM and lung cancer supplementary materials/Different expression genes analysis/GEO 7╕÷MM/GSE2658&GSE5900.pdf]

GSE39754

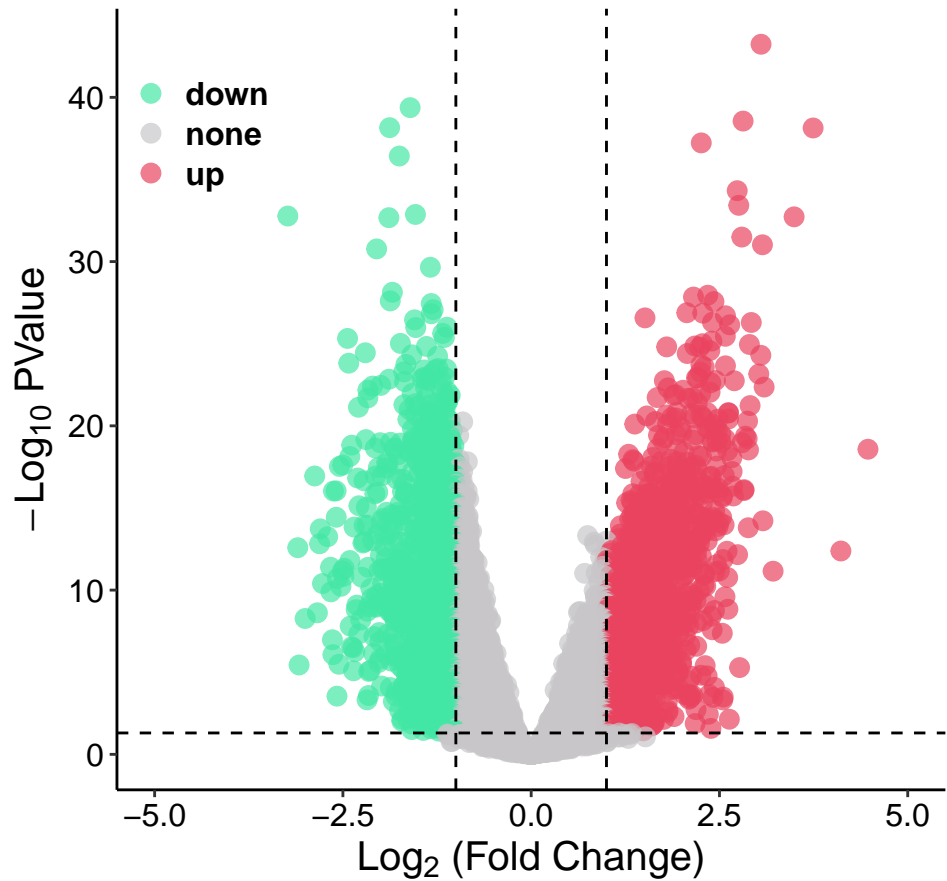

Supplement: Supplementary file 1 [file DataSheet_1.zip › The bioinformatic analysis of MM and lung cancer supplementary materials/Different expression genes analysis/GEO 7╕÷MM/GSE39754.pdf]

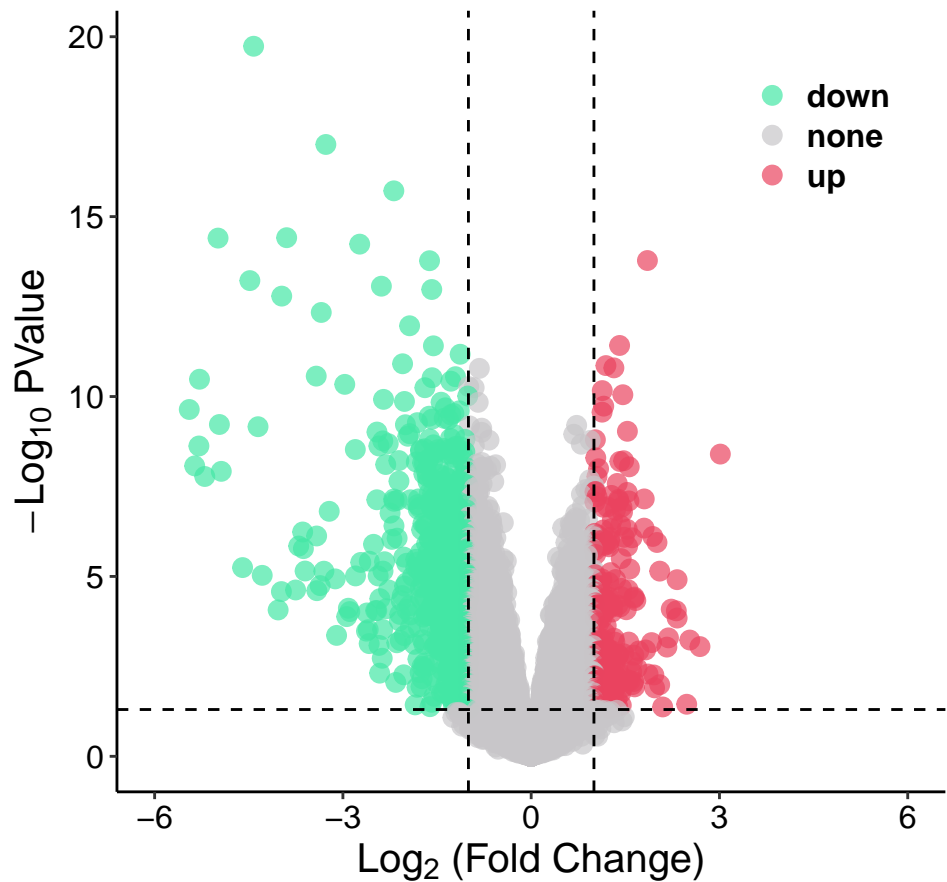

Supplement: Supplementary file 1 [file DataSheet_1.zip › The bioinformatic analysis of MM and lung cancer supplementary materials/Different expression genes analysis/GEO 7╕÷MM/GSE47552.pdf]

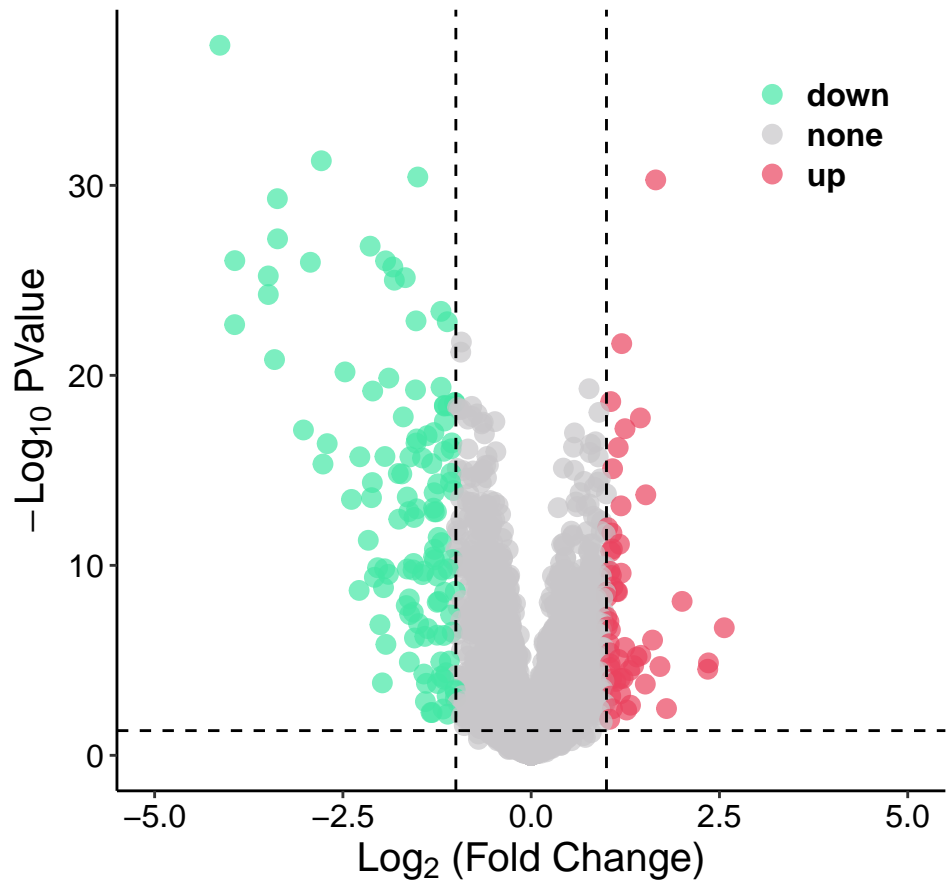

Supplement: Supplementary file 1 [file DataSheet_1.zip › The bioinformatic analysis of MM and lung cancer supplementary materials/Different expression genes analysis/GEO 7╕÷MM/GSE6477.pdf]

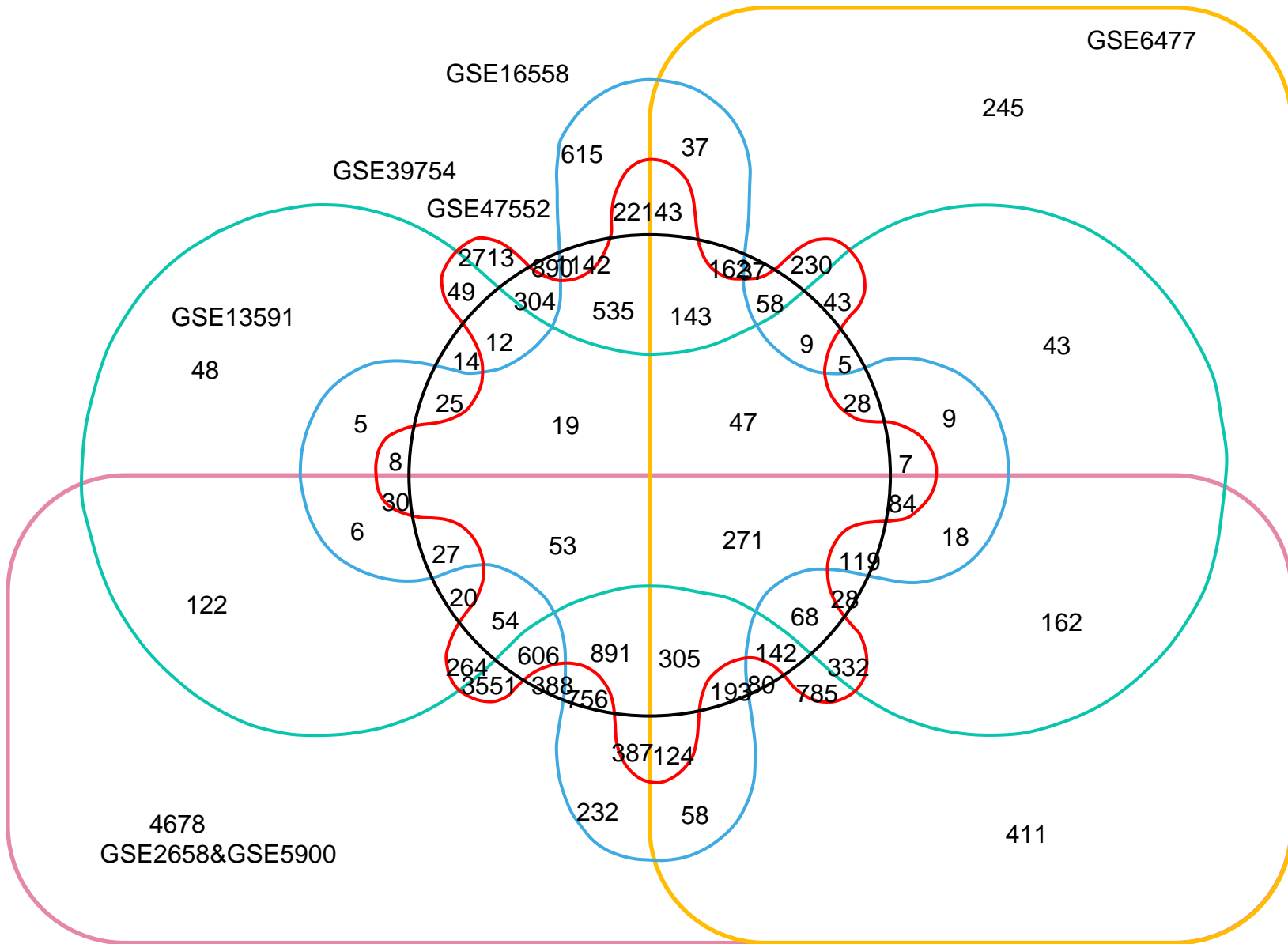

Supplement: Supplementary file 1 [file DataSheet_1.zip › The bioinformatic analysis of MM and lung cancer supplementary materials/Different expression genes analysis/GEO 7╕÷MM/Venn/Venn 6 MM GEO.pdf]

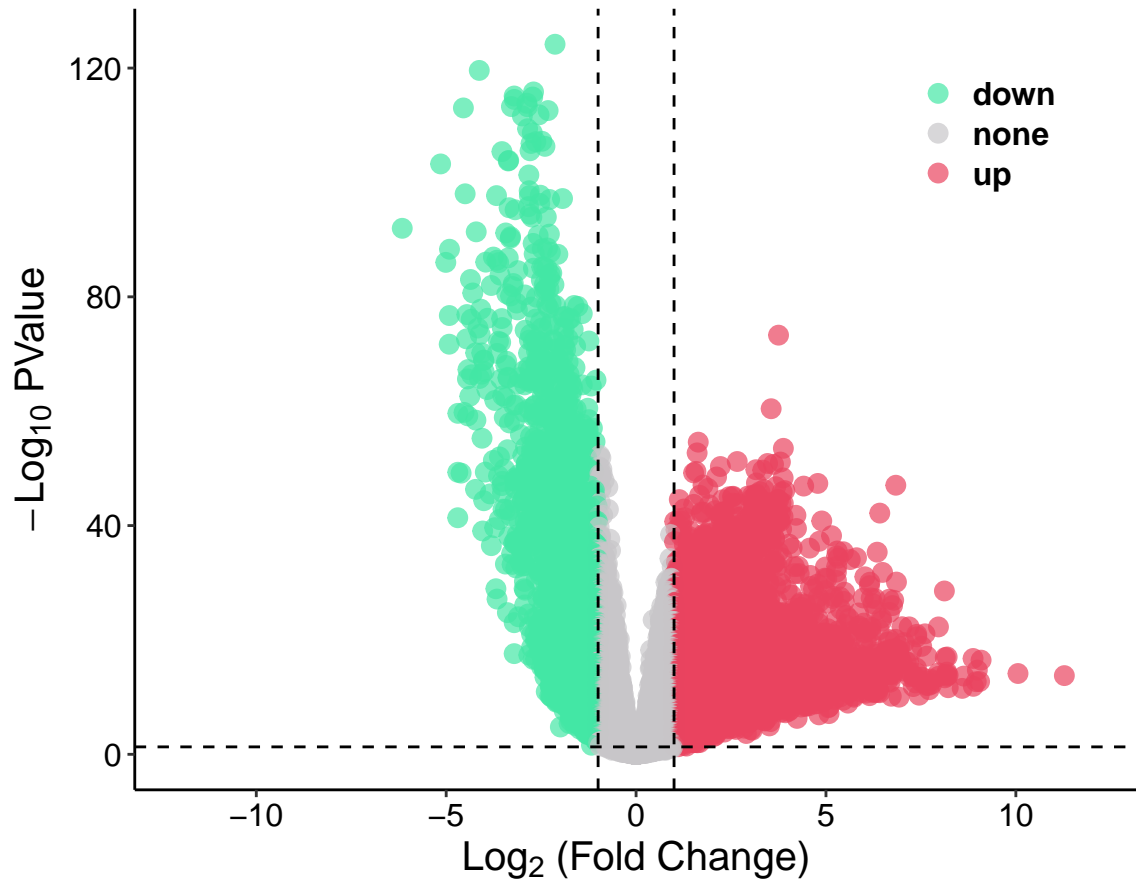

Supplement: Supplementary file 1 [file DataSheet_1.zip › The bioinformatic analysis of MM and lung cancer supplementary materials/Different expression genes analysis/TCGA (LUAD LUSC)/LUAD TCGA.pdf]

# LUSC TCGA

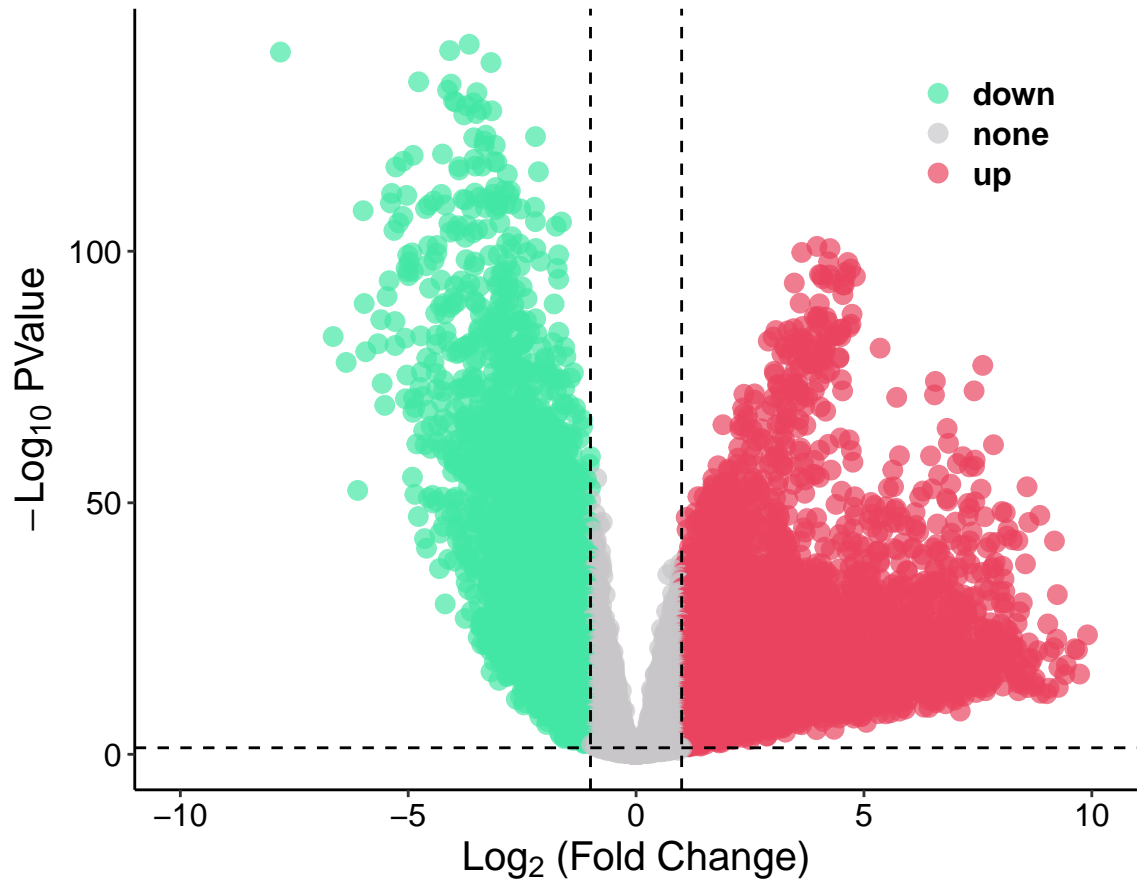

Supplement: Supplementary file 1 [file DataSheet_1.zip › The bioinformatic analysis of MM and lung cancer supplementary materials/Different expression genes analysis/TCGA (LUAD LUSC)/LUSC TCGA.pdf]

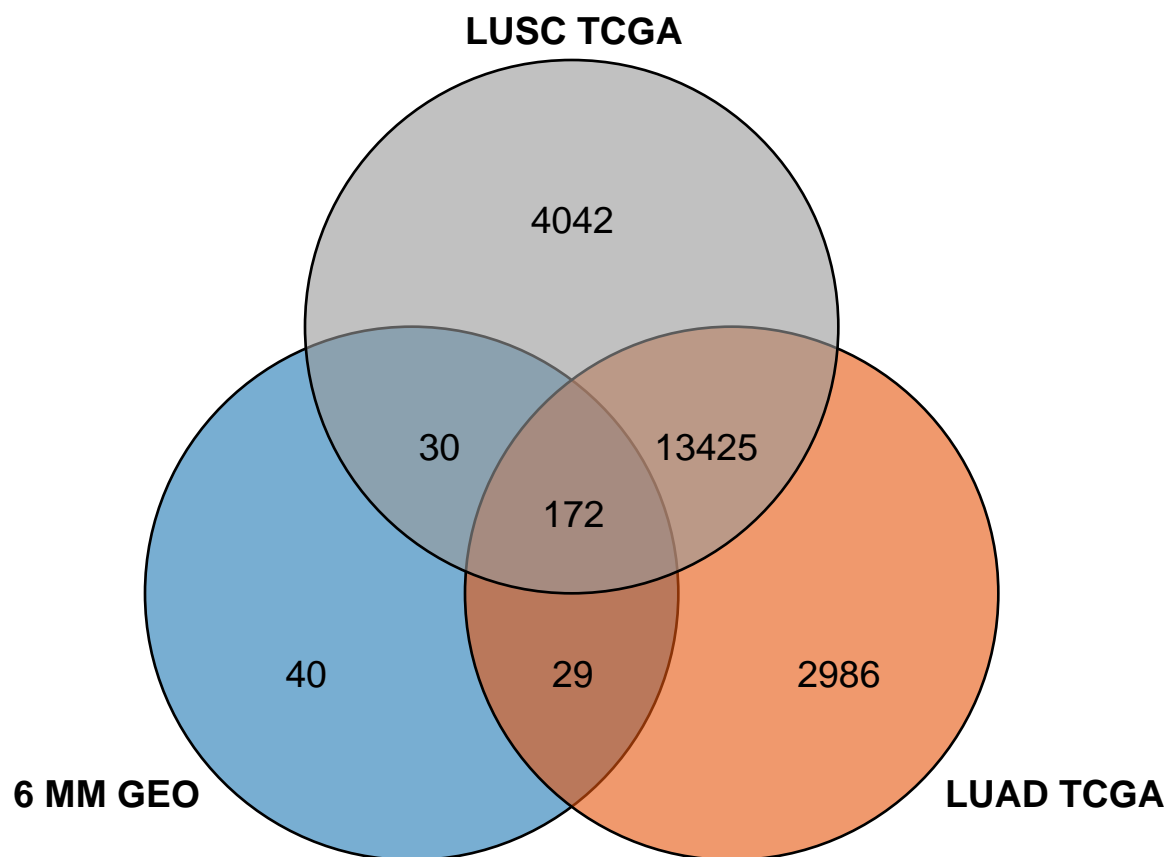

Supplement: Supplementary file 1 [file DataSheet_1.zip › The bioinformatic analysis of MM and lung cancer supplementary materials/Different expression genes analysis/TCGA (LUAD LUSC)/Venn/Venn.pdf]

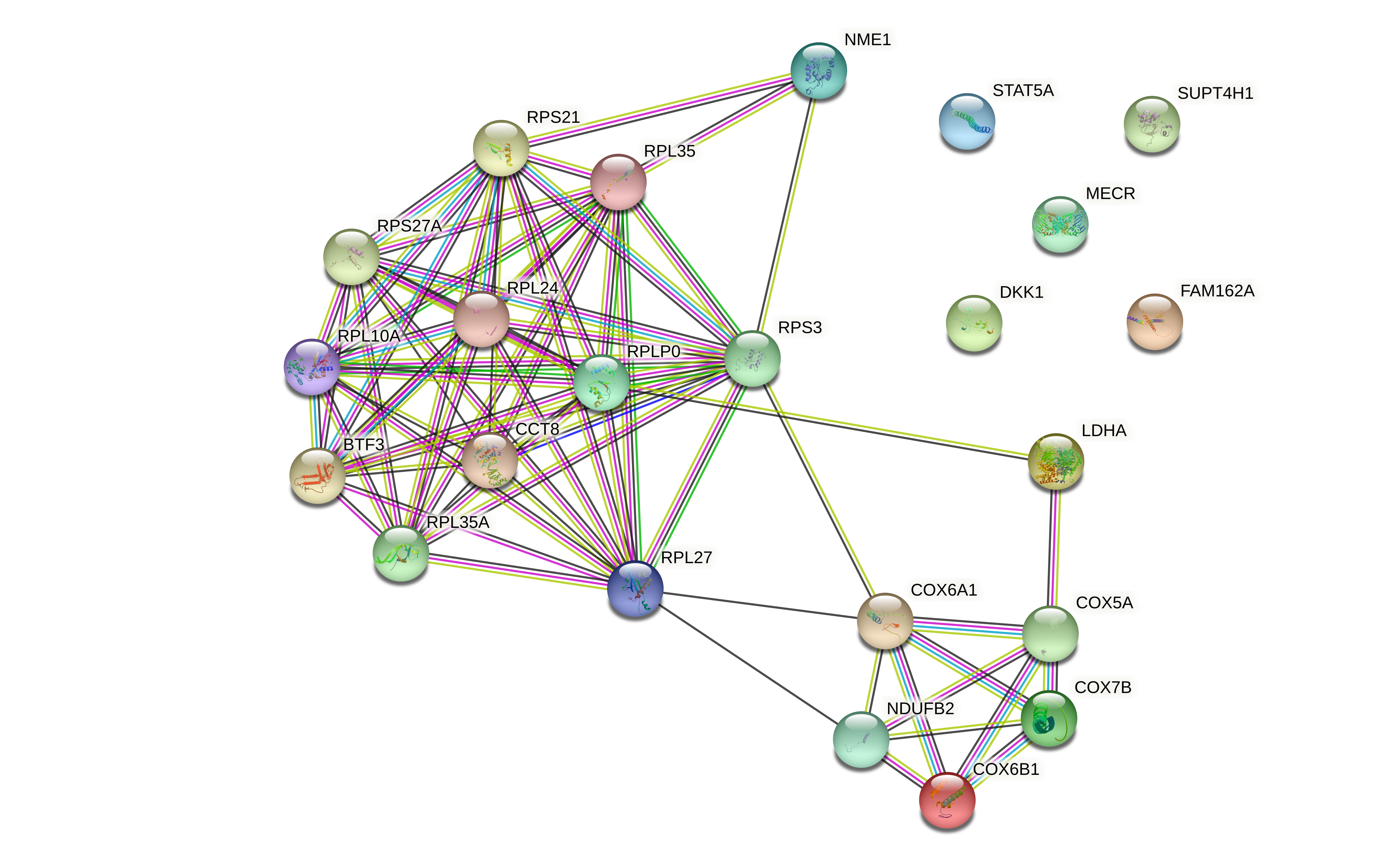

Supplement: Supplementary file 1 [file DataSheet_1.zip › The bioinformatic analysis of MM and lung cancer supplementary materials/Enrichment analysis/23╕÷DEG/STRING/string_hires_image.png]

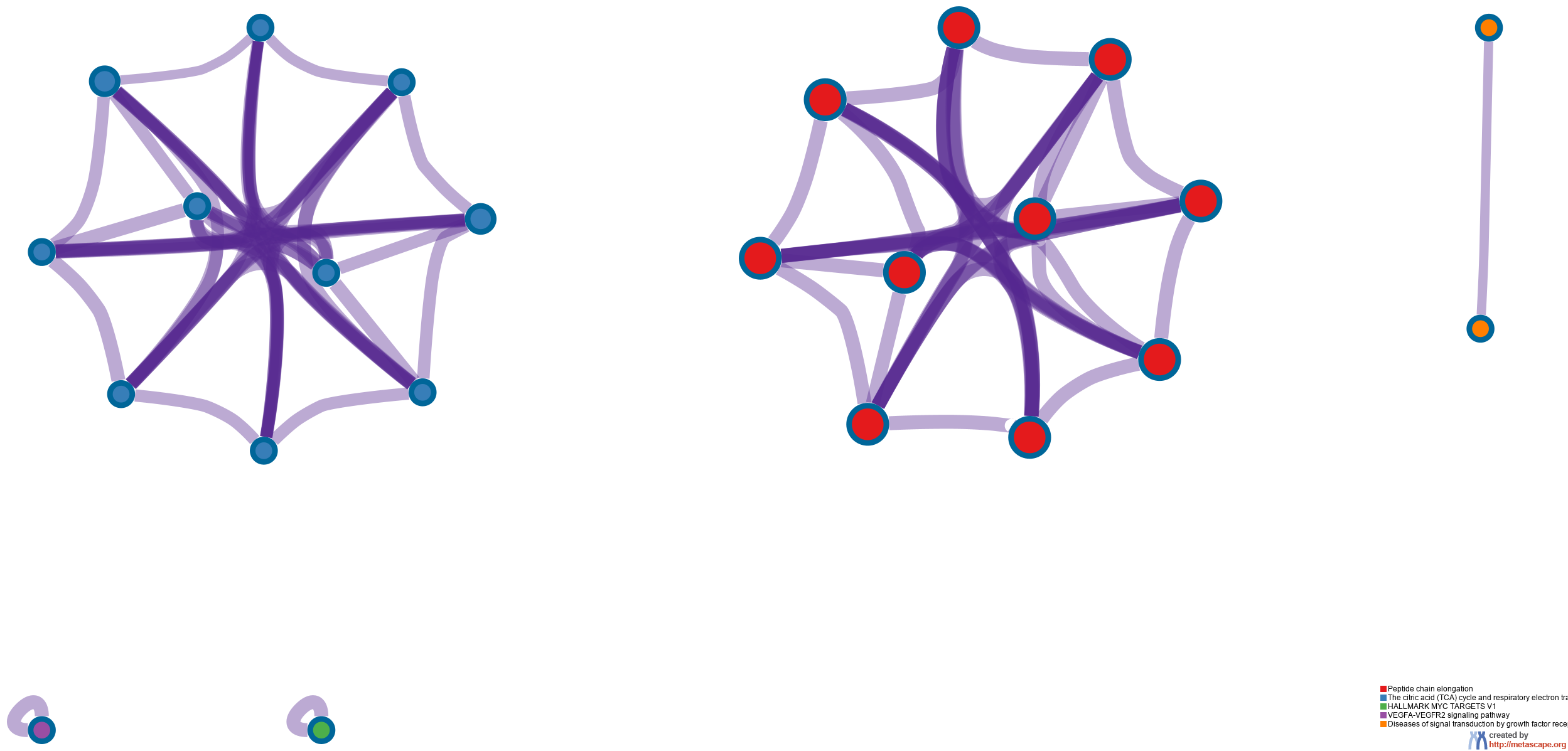

Supplement: Supplementary file 1 [file DataSheet_1.zip › The bioinformatic analysis of MM and lung cancer supplementary materials/Enrichment analysis/23╕÷DEG/metascape/Enrichment_GO/ColorByCluster.png]

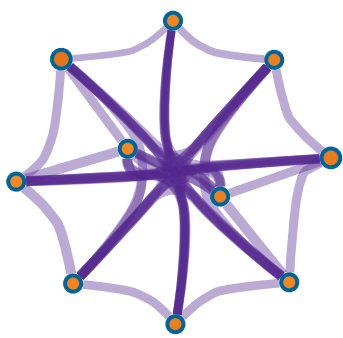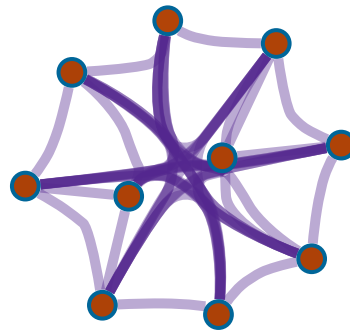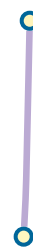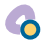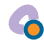

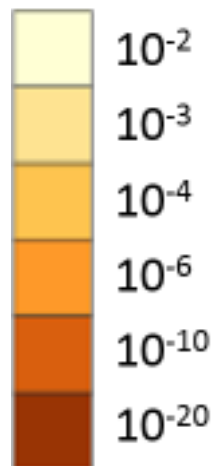

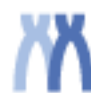 created by  
<http://metascape.org>

Supplement: Supplementary file 1 [file DataSheet_1.zip › The bioinformatic analysis of MM and lung cancer supplementary materials/Enrichment analysis/23╕÷DEG/metascape/Enrichment_GO/ColorByPValue.pdf]

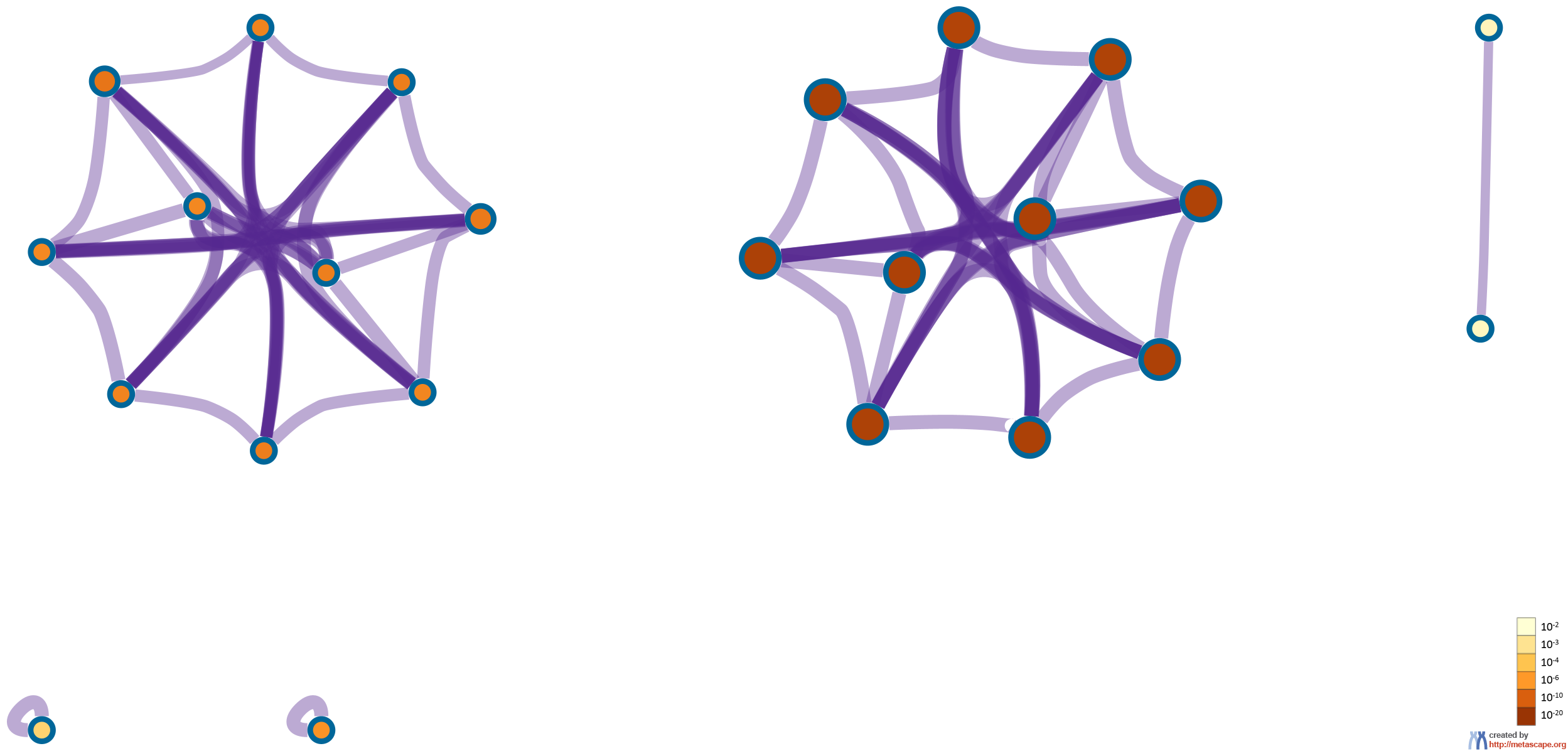

Supplement: Supplementary file 1 [file DataSheet_1.zip › The bioinformatic analysis of MM and lung cancer supplementary materials/Enrichment analysis/23╕÷DEG/metascape/Enrichment_GO/ColorByPValue.png]

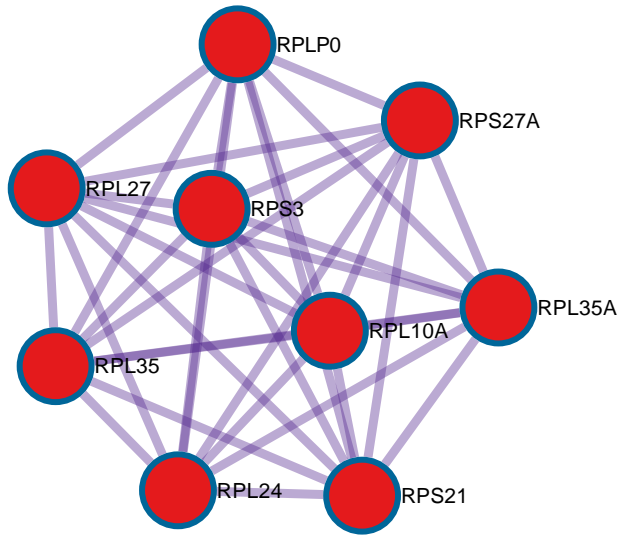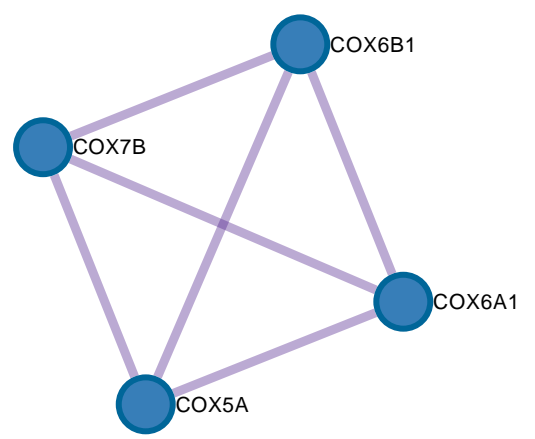

■ MCODE1  
■ MCODE2

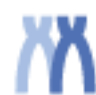 created by  
<http://metascape.org>

Supplement: Supplementary file 1 [file DataSheet_1.zip › The bioinformatic analysis of MM and lung cancer supplementary materials/Enrichment analysis/23╕÷DEG/metascape/Enrichment_PPI/MyList_MCODE_ALL_PPIColorByCluster.pdf]

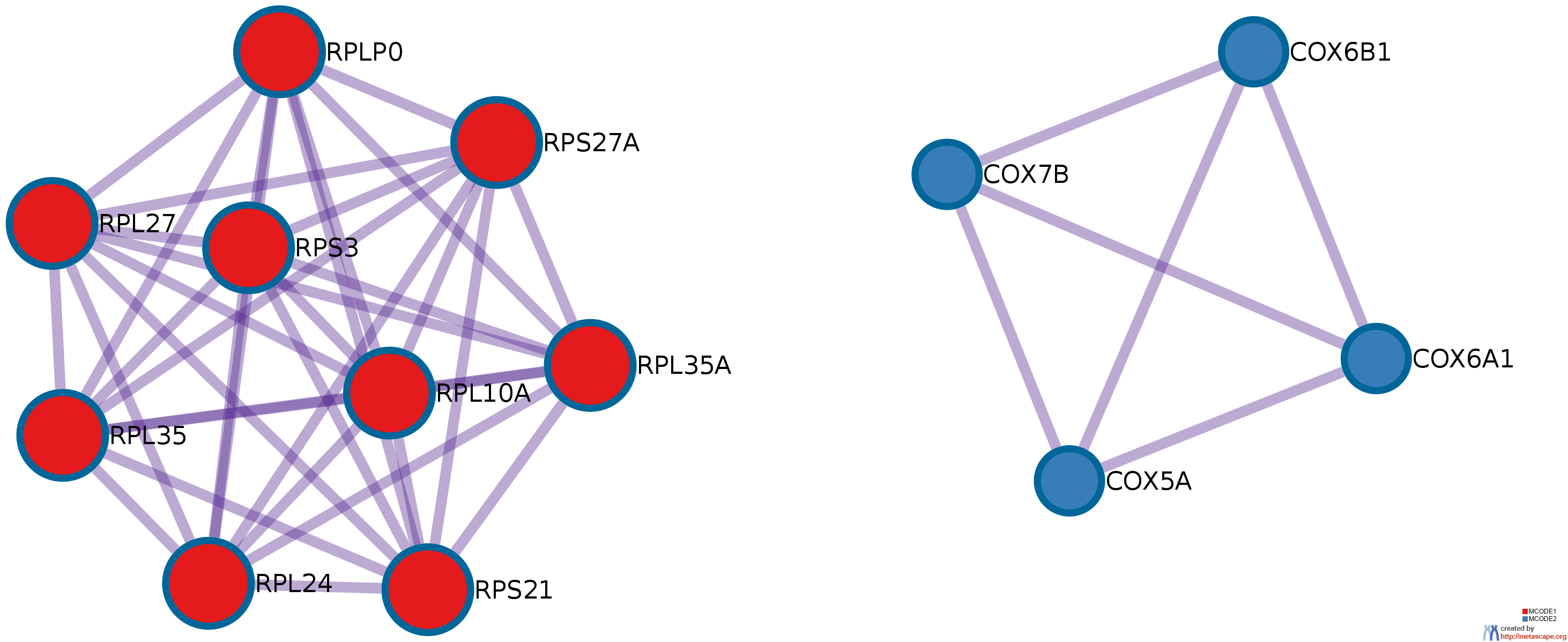

Supplement: Supplementary file 1 [file DataSheet_1.zip › The bioinformatic analysis of MM and lung cancer supplementary materials/Enrichment analysis/23╕÷DEG/metascape/Enrichment_PPI/MyList_MCODE_ALL_PPIColorByCluster.png]

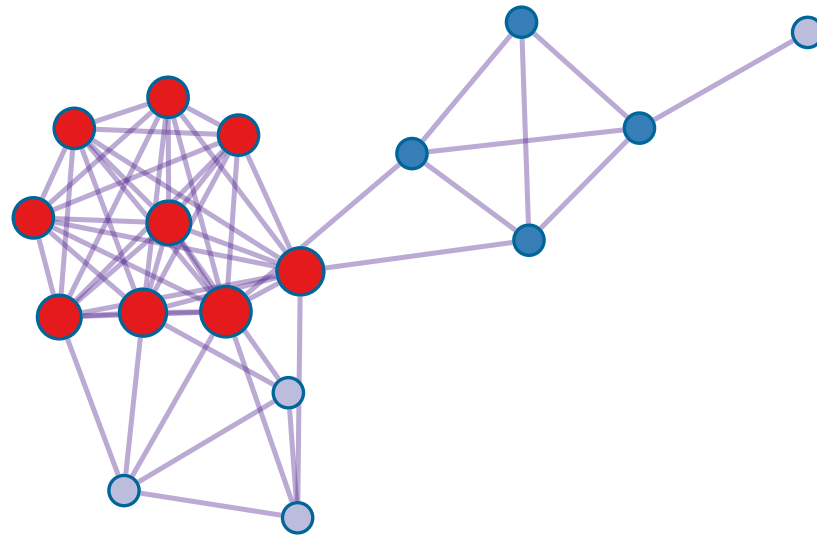

■ MCODE1  
■ MCODE2

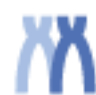 created by  
<http://metascape.org>

Supplement: Supplementary file 1 [file DataSheet_1.zip › The bioinformatic analysis of MM and lung cancer supplementary materials/Enrichment analysis/23╕÷DEG/metascape/Enrichment_PPI/MyList_PPIColorByCluster.pdf]

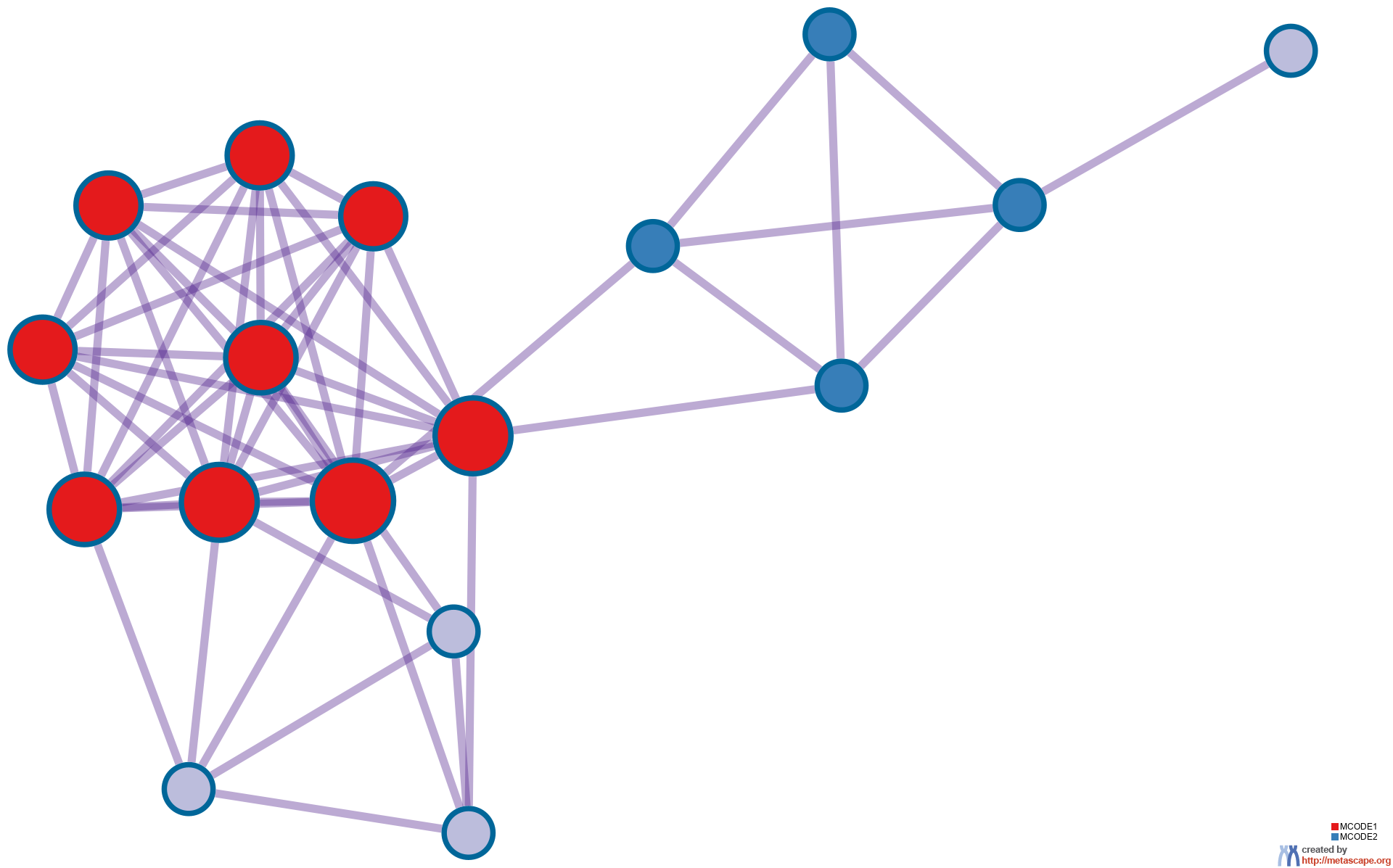

Supplement: Supplementary file 1 [file DataSheet_1.zip › The bioinformatic analysis of MM and lung cancer supplementary materials/Enrichment analysis/23╕÷DEG/metascape/Enrichment_PPI/MyList_PPIColorByCluster.png]

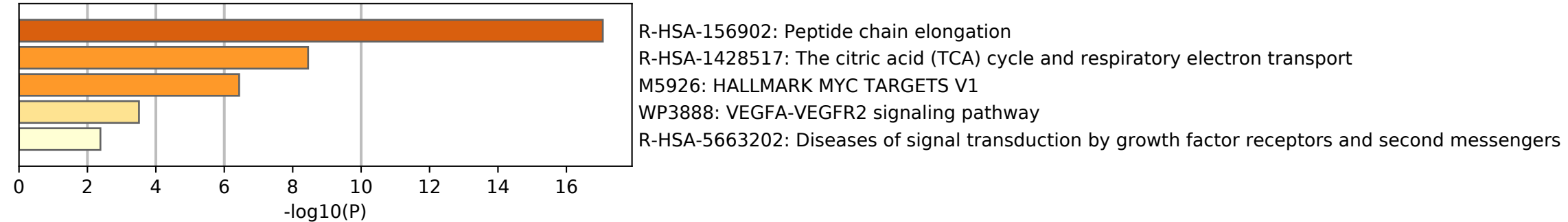

Supplement: Supplementary file 1 [file DataSheet_1.zip › The bioinformatic analysis of MM and lung cancer supplementary materials/Enrichment analysis/23╕÷DEG/metascape/Enrichment_heatmap/HeatmapSelectedGO.pdf]

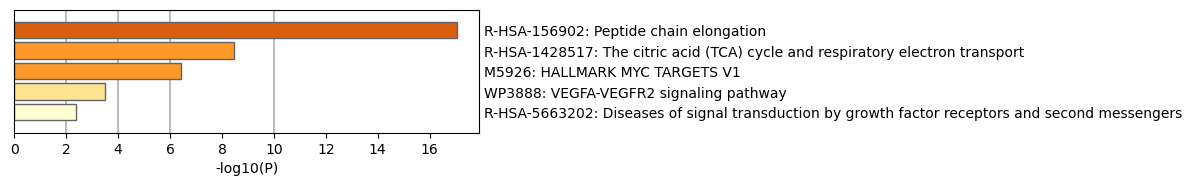

Supplement: Supplementary file 1 [file DataSheet_1.zip › The bioinformatic analysis of MM and lung cancer supplementary materials/Enrichment analysis/23╕÷DEG/metascape/Enrichment_heatmap/HeatmapSelectedGO.png]

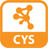

Supplement: Supplementary file 1 [file DataSheet_1.zip › The bioinformatic analysis of MM and lung cancer supplementary materials/Enrichment analysis/23╕÷DEG/metascape/icon/CYS48.png]

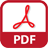

Supplement: Supplementary file 1 [file DataSheet_1.zip › The bioinformatic analysis of MM and lung cancer supplementary materials/Enrichment analysis/23╕÷DEG/metascape/icon/PDF48.png]

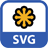

Supplement: Supplementary file 1 [file DataSheet_1.zip › The bioinformatic analysis of MM and lung cancer supplementary materials/Enrichment analysis/23╕÷DEG/metascape/icon/SVG48.png]

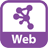

Supplement: Supplementary file 1 [file DataSheet_1.zip › The bioinformatic analysis of MM and lung cancer supplementary materials/Enrichment analysis/23╕÷DEG/metascape/icon/WEB_CYS48.png]

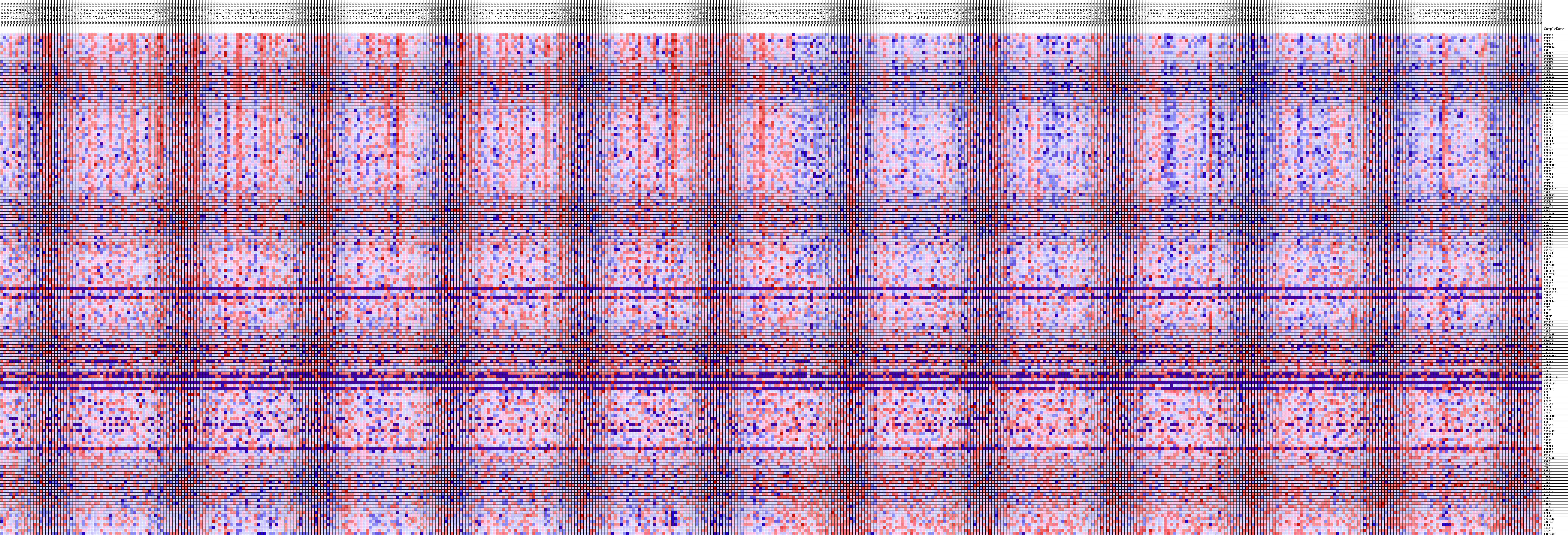

Supplement: Supplementary file 1 [file DataSheet_1.zip › The bioinformatic analysis of MM and lung cancer supplementary materials/Enrichment analysis/MECR/GSEA_4.1.0/LUAD TCGA/KEGG.Gsea.1639041756227/KEGG_ALZHEIMERS_DISEASE_16.png]

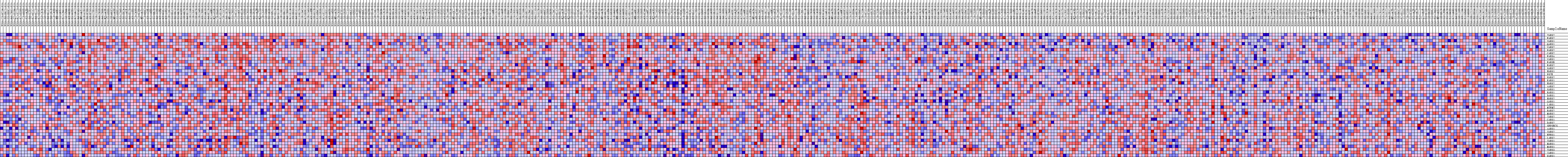

Supplement: Supplementary file 1 [file DataSheet_1.zip › The bioinformatic analysis of MM and lung cancer supplementary materials/Enrichment analysis/MECR/GSEA_4.1.0/LUAD TCGA/KEGG.Gsea.1639041756227/KEGG_AMINOACYL_TRNA_BIOSYNTHESIS_52.png]

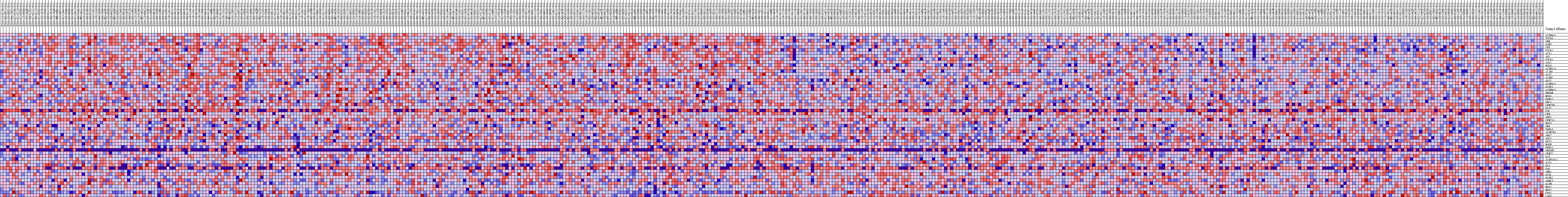

Supplement: Supplementary file 1 [file DataSheet_1.zip › The bioinformatic analysis of MM and lung cancer supplementary materials/Enrichment analysis/MECR/GSEA_4.1.0/LUAD TCGA/KEGG.Gsea.1639041756227/KEGG_ARGININE_AND_PROLINE_METABOLISM_55.png]

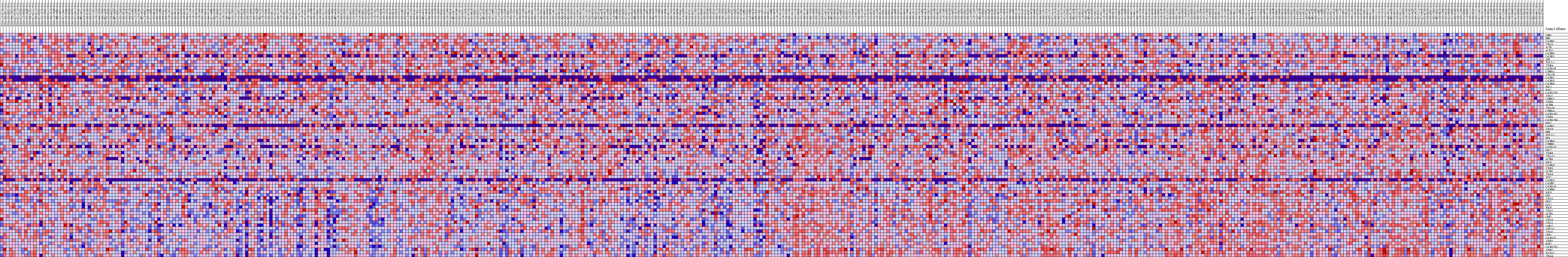

Supplement: Supplementary file 1 [file DataSheet_1.zip › The bioinformatic analysis of MM and lung cancer supplementary materials/Enrichment analysis/MECR/GSEA_4.1.0/LUAD TCGA/KEGG.Gsea.1639041756227/KEGG_ARRHYTHMOGENIC_RIGHT_VENTRICULAR_CARDIOMYOPATHY_ARVC_115.png]

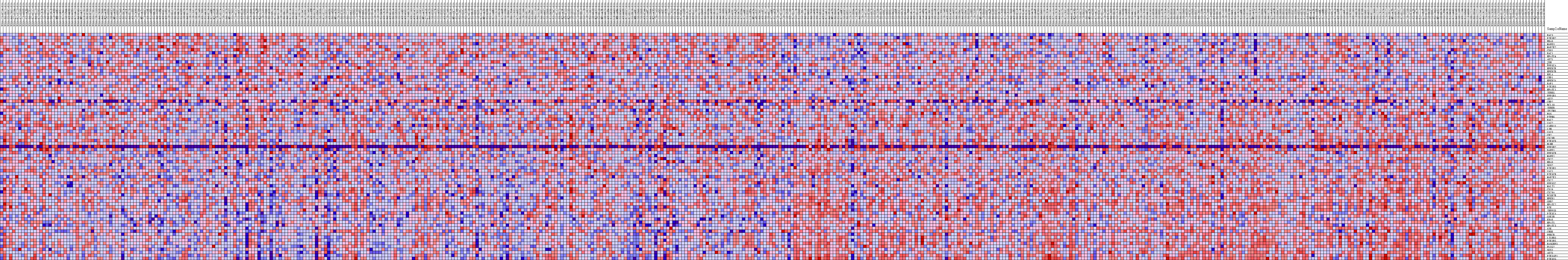

Supplement: Supplementary file 1 [file DataSheet_1.zip › The bioinformatic analysis of MM and lung cancer supplementary materials/Enrichment analysis/MECR/GSEA_4.1.0/LUAD TCGA/KEGG.Gsea.1639041756227/KEGG_B_CELL_RECEPTOR_SIGNALING_PATHWAY_112.png]

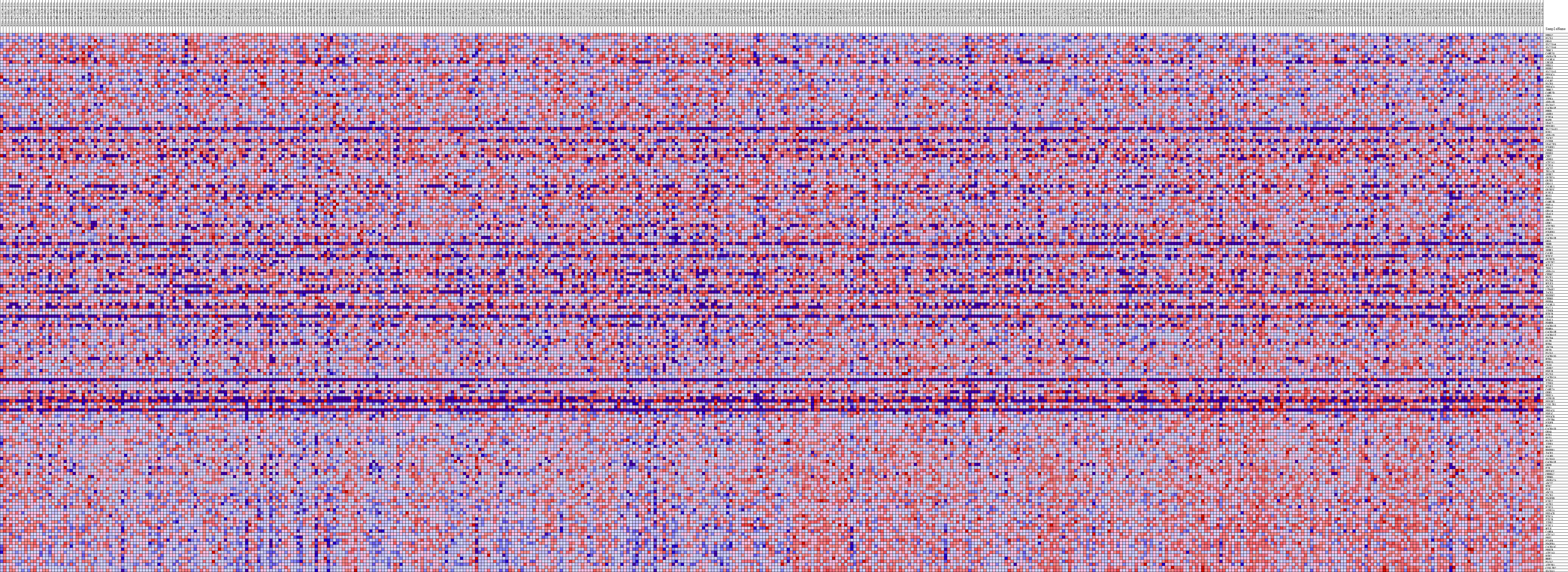

Supplement: Supplementary file 1 [file DataSheet_1.zip › The bioinformatic analysis of MM and lung cancer supplementary materials/Enrichment analysis/MECR/GSEA_4.1.0/LUAD TCGA/KEGG.Gsea.1639041756227/KEGG_CALCIUM_SIGNALING_PATHWAY_106.png]

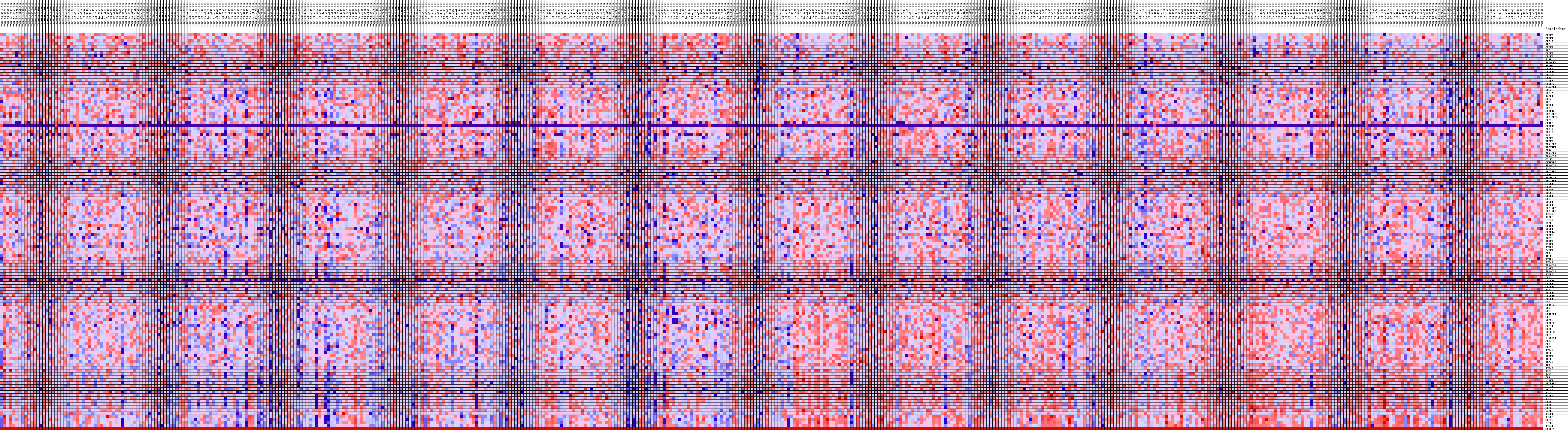

Supplement: Supplementary file 1 [file DataSheet_1.zip › The bioinformatic analysis of MM and lung cancer supplementary materials/Enrichment analysis/MECR/GSEA_4.1.0/LUAD TCGA/KEGG.Gsea.1639041756227/KEGG_CELL_ADHESION_MOLECULES_CAMS_79.png]

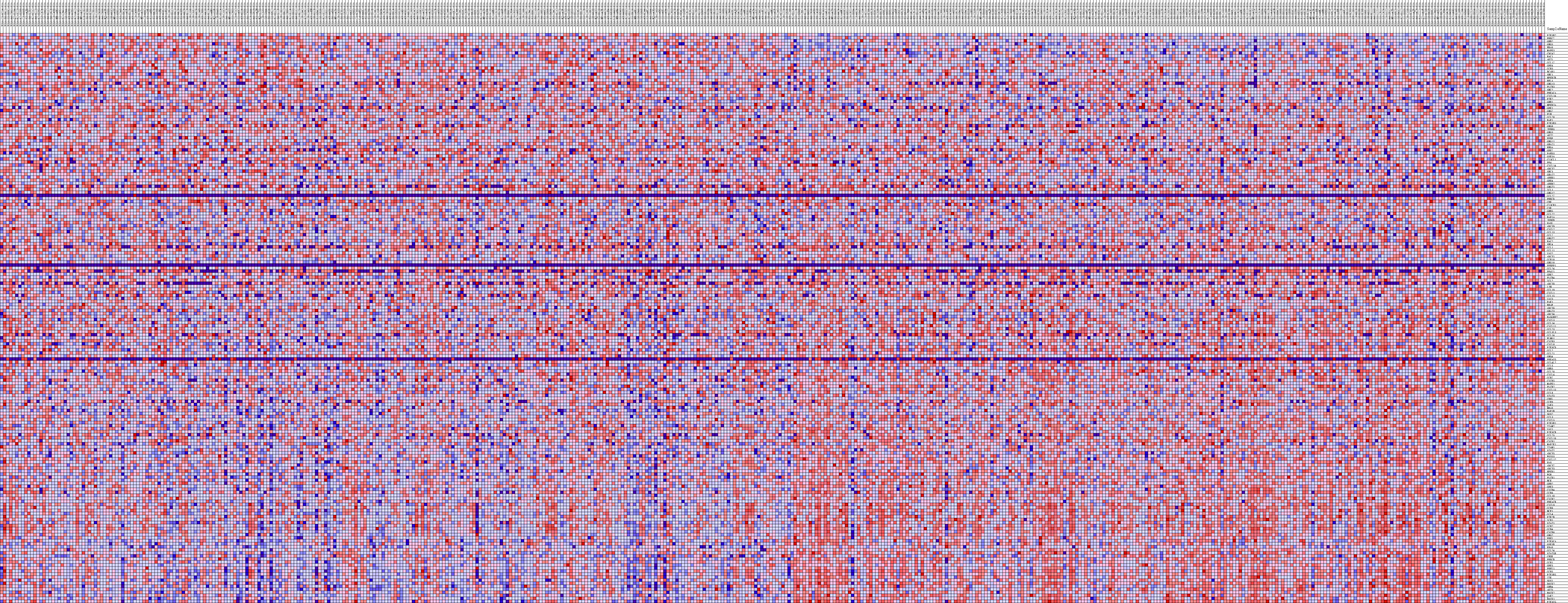

Supplement: Supplementary file 1 [file DataSheet_1.zip › The bioinformatic analysis of MM and lung cancer supplementary materials/Enrichment analysis/MECR/GSEA_4.1.0/LUAD TCGA/KEGG.Gsea.1639041756227/KEGG_CHEMOKINE_SIGNALING_PATHWAY_64.png]

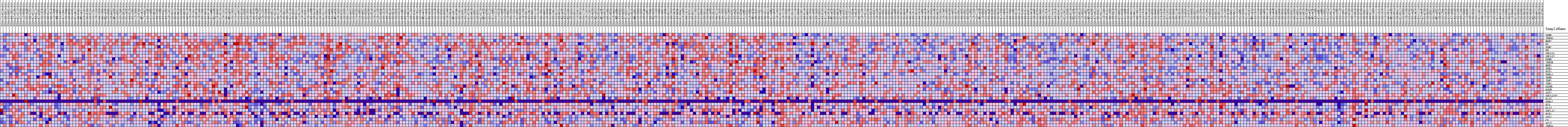

Supplement: Supplementary file 1 [file DataSheet_1.zip › The bioinformatic analysis of MM and lung cancer supplementary materials/Enrichment analysis/MECR/GSEA_4.1.0/LUAD TCGA/KEGG.Gsea.1639041756227/KEGG_CITRATE_CYCLE_TCA_CYCLE_61.png]

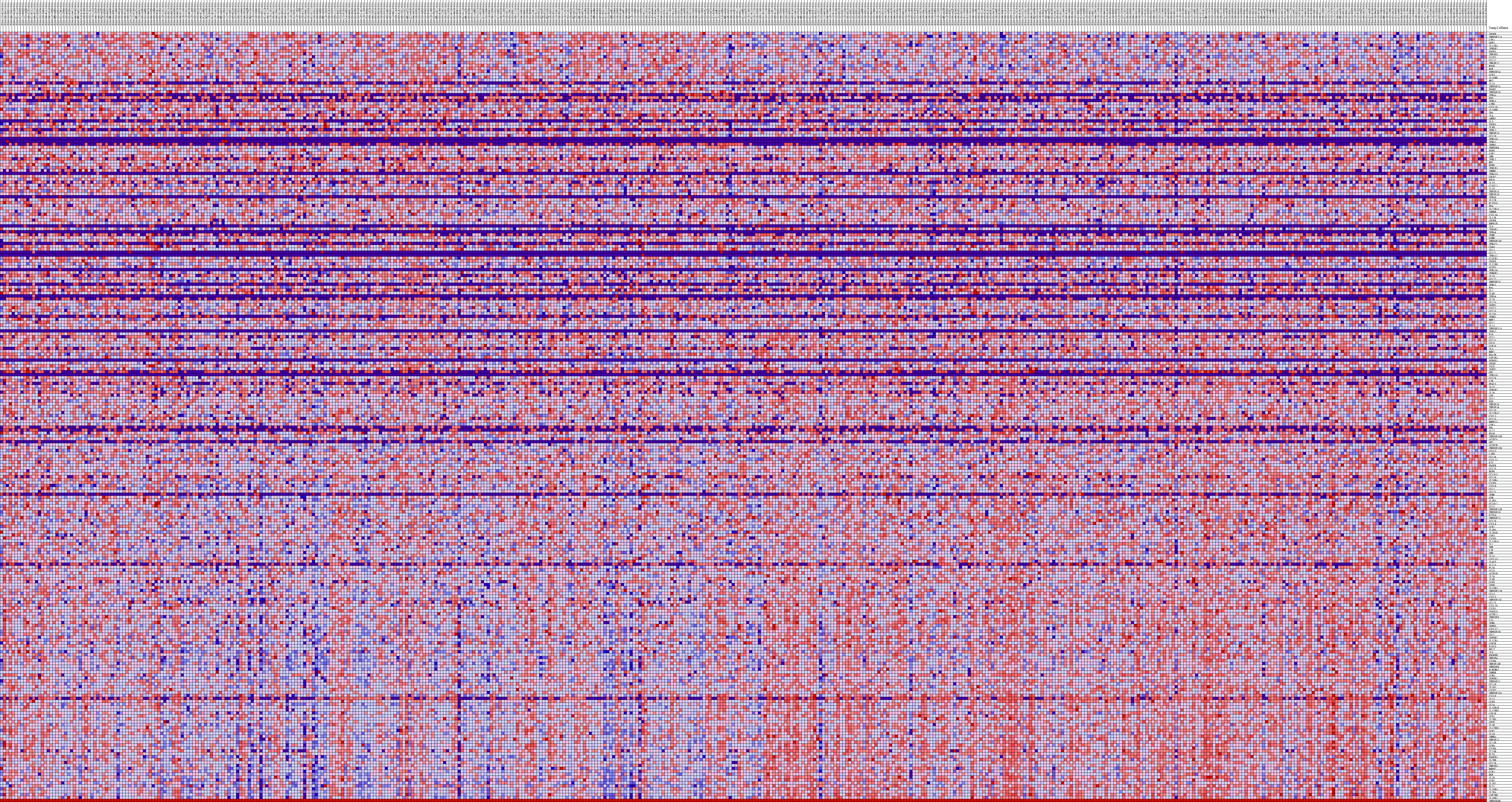

Supplement: Supplementary file 1 [file DataSheet_1.zip › The bioinformatic analysis of MM and lung cancer supplementary materials/Enrichment analysis/MECR/GSEA_4.1.0/LUAD TCGA/KEGG.Gsea.1639041756227/KEGG_CYTOKINE_CYTOKINE_RECEPTOR_INTERACTION_67.png]

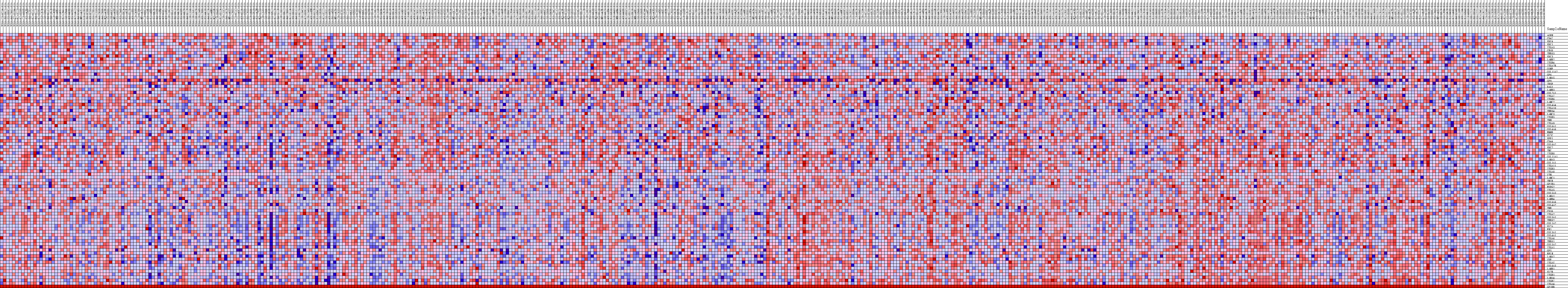

Supplement: Supplementary file 1 [file DataSheet_1.zip › The bioinformatic analysis of MM and lung cancer supplementary materials/Enrichment analysis/MECR/GSEA_4.1.0/LUAD TCGA/KEGG.Gsea.1639041756227/KEGG_ECM_RECEPTOR_INTERACTION_85.png]

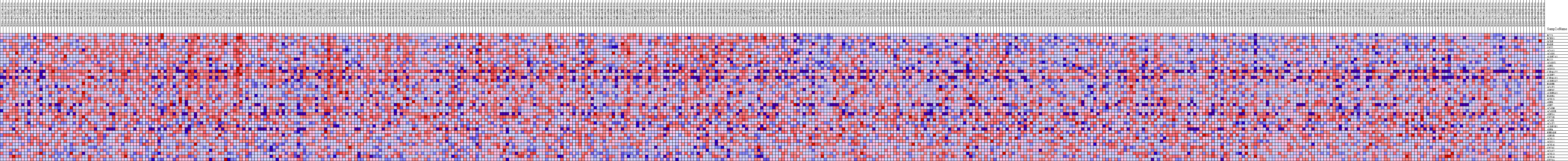

Supplement: Supplementary file 1 [file DataSheet_1.zip › The bioinformatic analysis of MM and lung cancer supplementary materials/Enrichment analysis/MECR/GSEA_4.1.0/LUAD TCGA/KEGG.Gsea.1639041756227/KEGG_FATTY_ACID_METABOLISM_49.png]

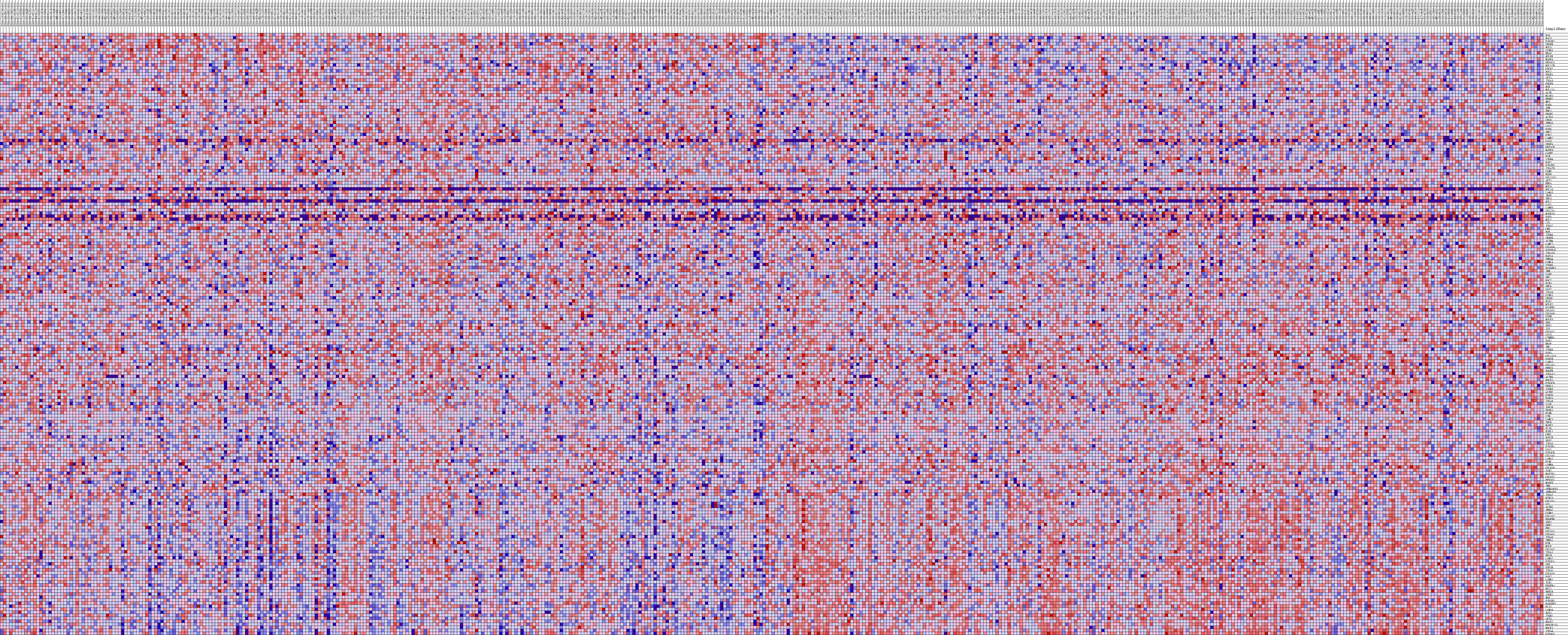

Supplement: Supplementary file 1 [file DataSheet_1.zip › The bioinformatic analysis of MM and lung cancer supplementary materials/Enrichment analysis/MECR/GSEA_4.1.0/LUAD TCGA/KEGG.Gsea.1639041756227/KEGG_FOCAL_ADHESION_100.png]

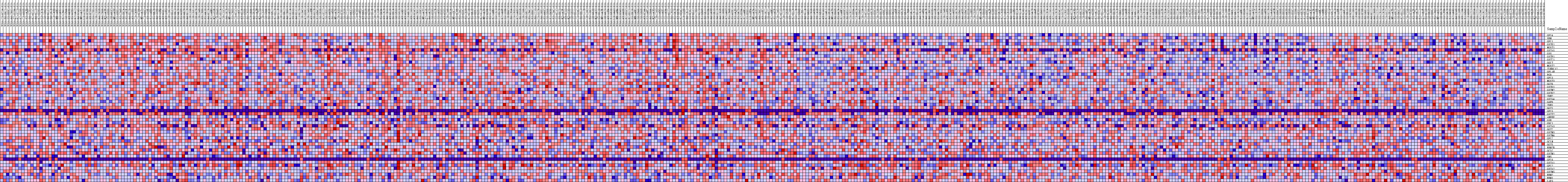

Supplement: Supplementary file 1 [file DataSheet_1.zip › The bioinformatic analysis of MM and lung cancer supplementary materials/Enrichment analysis/MECR/GSEA_4.1.0/LUAD TCGA/KEGG.Gsea.1639041756227/KEGG_GLUTATHIONE_METABOLISM_19.png]

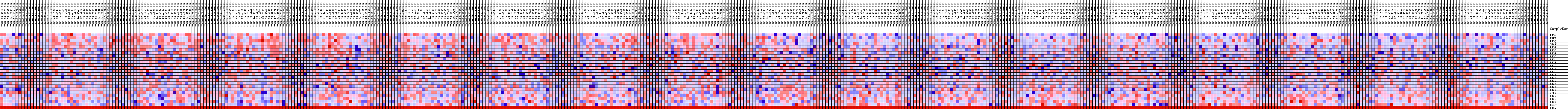

Supplement: Supplementary file 1 [file DataSheet_1.zip › The bioinformatic analysis of MM and lung cancer supplementary materials/Enrichment analysis/MECR/GSEA_4.1.0/LUAD TCGA/KEGG.Gsea.1639041756227/KEGG_GLYCOSYLPHOSPHATIDYLINOSITOL_GPI_ANCHOR_BIOSYNTHESIS_58.png]

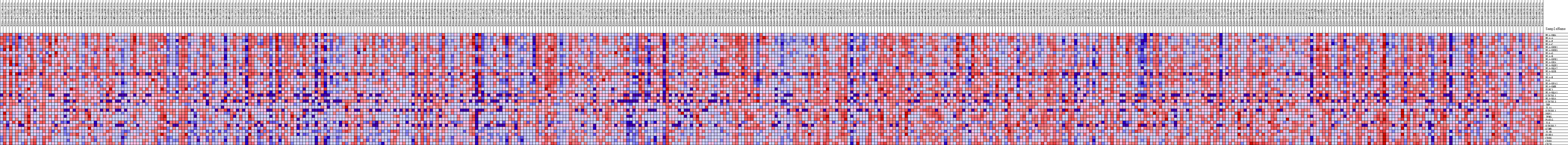

Supplement: Supplementary file 1 [file DataSheet_1.zip › The bioinformatic analysis of MM and lung cancer supplementary materials/Enrichment analysis/MECR/GSEA_4.1.0/LUAD TCGA/KEGG.Gsea.1639041756227/KEGG_GRAFT_VERSUS_HOST_DISEASE_88.png]

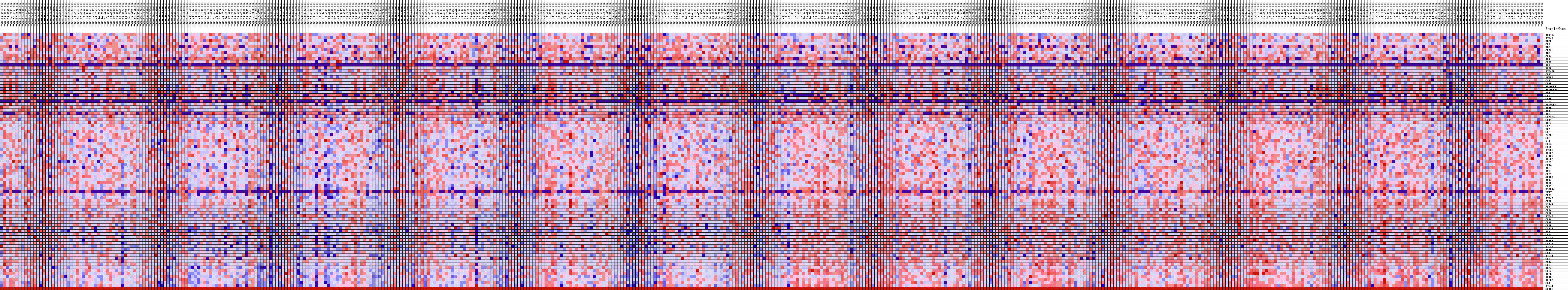

Supplement: Supplementary file 1 [file DataSheet_1.zip › The bioinformatic analysis of MM and lung cancer supplementary materials/Enrichment analysis/MECR/GSEA_4.1.0/LUAD TCGA/KEGG.Gsea.1639041756227/KEGG_HEMATOPOIETIC_CELL_LINEAGE_70.png]

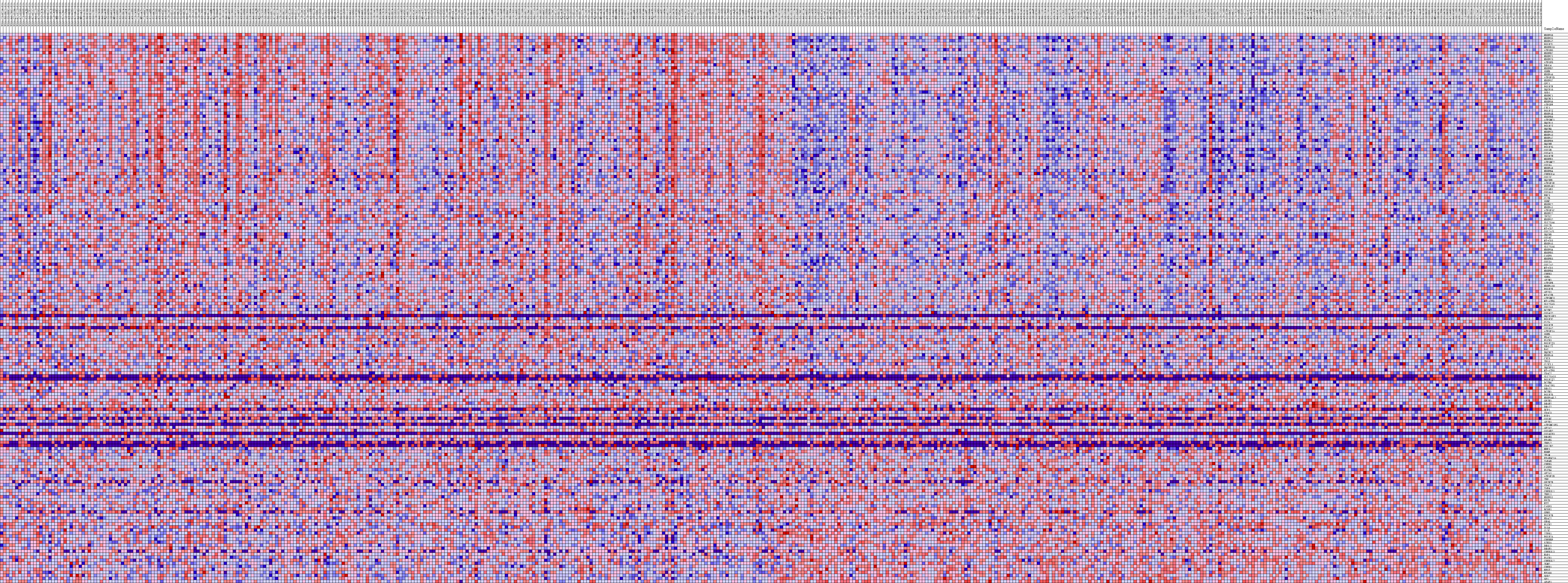

Supplement: Supplementary file 1 [file DataSheet_1.zip › The bioinformatic analysis of MM and lung cancer supplementary materials/Enrichment analysis/MECR/GSEA_4.1.0/LUAD TCGA/KEGG.Gsea.1639041756227/KEGG_HUNTINGTONS_DISEASE_13.png]

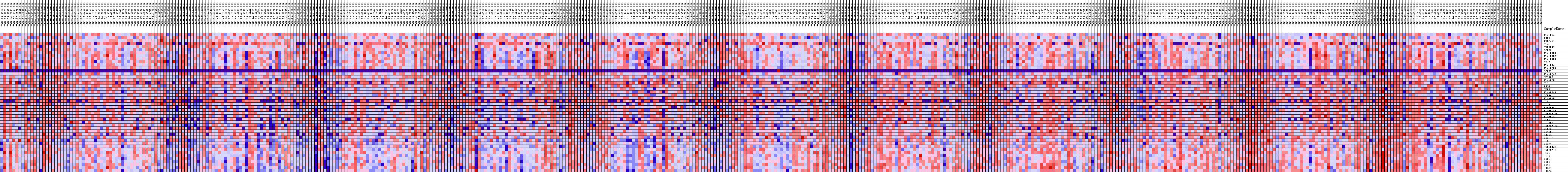

Supplement: Supplementary file 1 [file DataSheet_1.zip › The bioinformatic analysis of MM and lung cancer supplementary materials/Enrichment analysis/MECR/GSEA_4.1.0/LUAD TCGA/KEGG.Gsea.1639041756227/KEGG_INTESTINAL_IMMUNE_NETWORK_FOR_IGA_PRODUCTION_82.png]

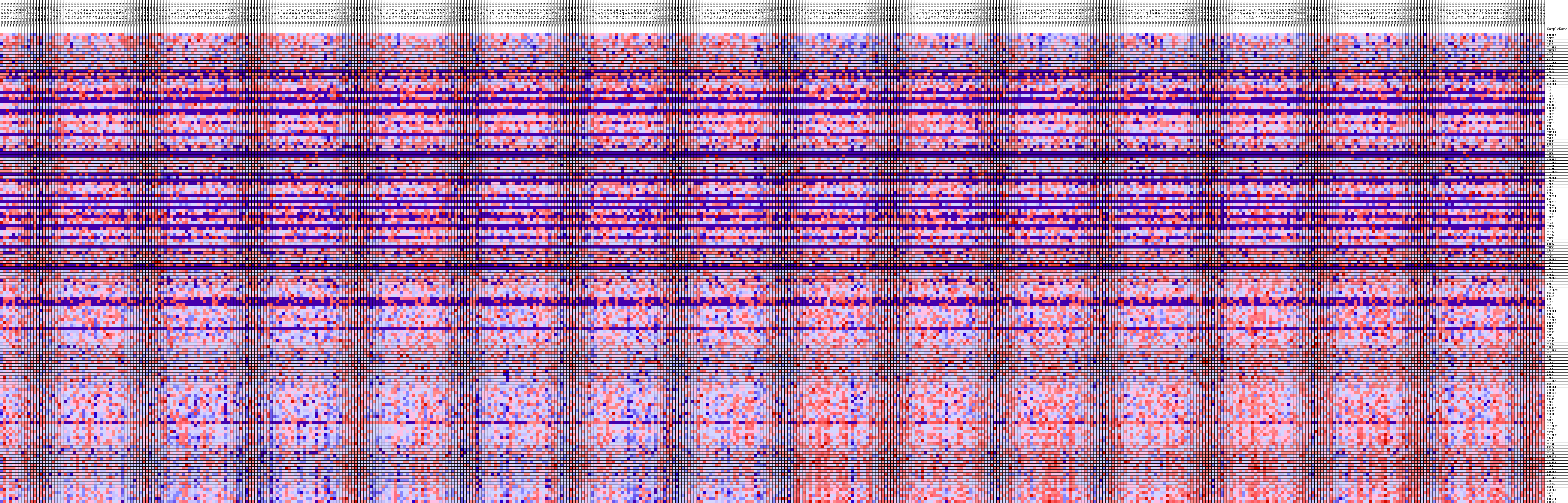

Supplement: Supplementary file 1 [file DataSheet_1.zip › The bioinformatic analysis of MM and lung cancer supplementary materials/Enrichment analysis/MECR/GSEA_4.1.0/LUAD TCGA/KEGG.Gsea.1639041756227/KEGG_JAK_STAT_SIGNALING_PATHWAY_94.png]

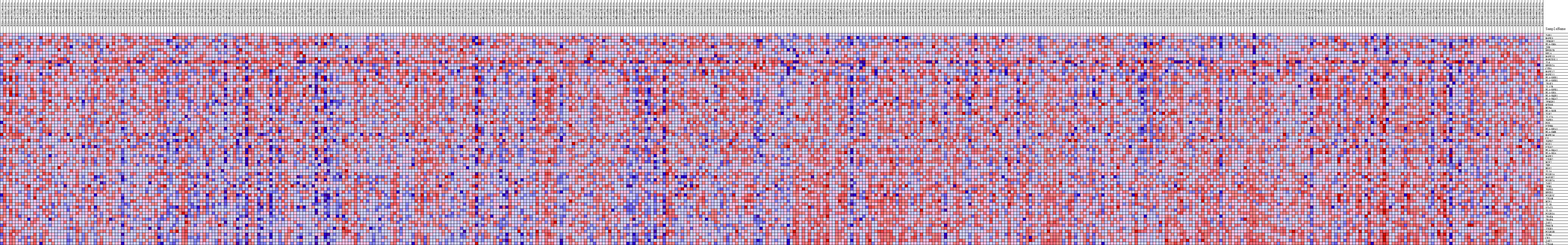

Supplement: Supplementary file 1 [file DataSheet_1.zip › The bioinformatic analysis of MM and lung cancer supplementary materials/Enrichment analysis/MECR/GSEA_4.1.0/LUAD TCGA/KEGG.Gsea.1639041756227/KEGG_LEISHMANIA_INFECTION_76.png]

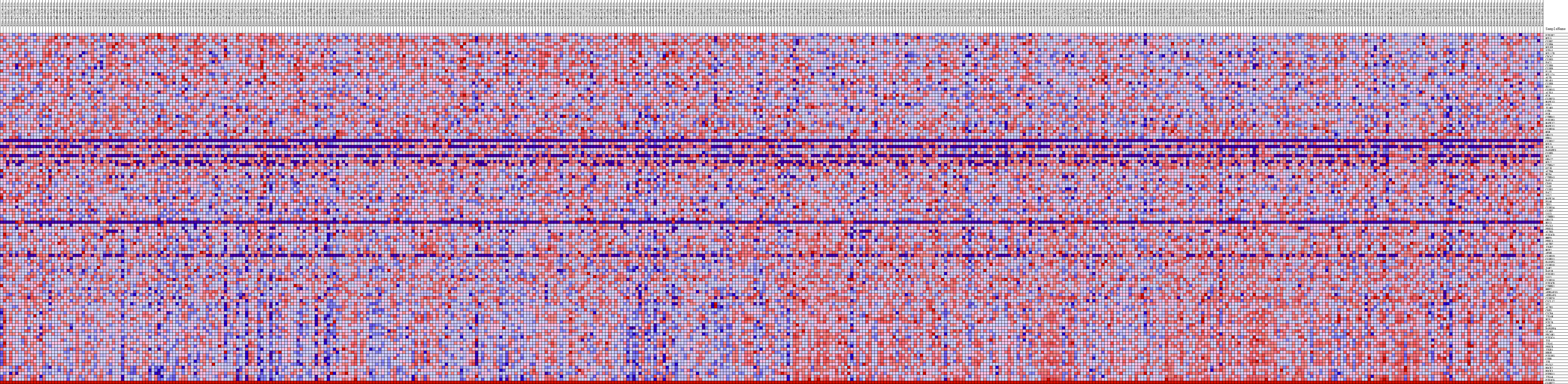

Supplement: Supplementary file 1 [file DataSheet_1.zip › The bioinformatic analysis of MM and lung cancer supplementary materials/Enrichment analysis/MECR/GSEA_4.1.0/LUAD TCGA/KEGG.Gsea.1639041756227/KEGG_LEUKOCYTE_TRANSENDOTHELIAL_MIGRATION_118.png]

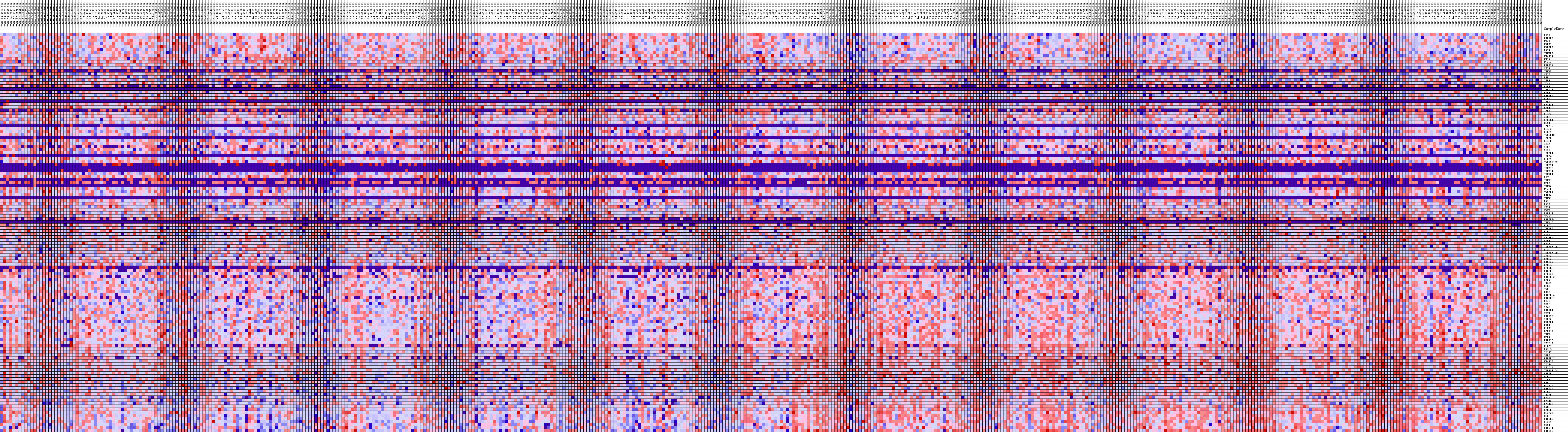

Supplement: Supplementary file 1 [file DataSheet_1.zip › The bioinformatic analysis of MM and lung cancer supplementary materials/Enrichment analysis/MECR/GSEA_4.1.0/LUAD TCGA/KEGG.Gsea.1639041756227/KEGG_NATURAL_KILLER_CELL_MEDIATED_CYTOTOXICITY_91.png]

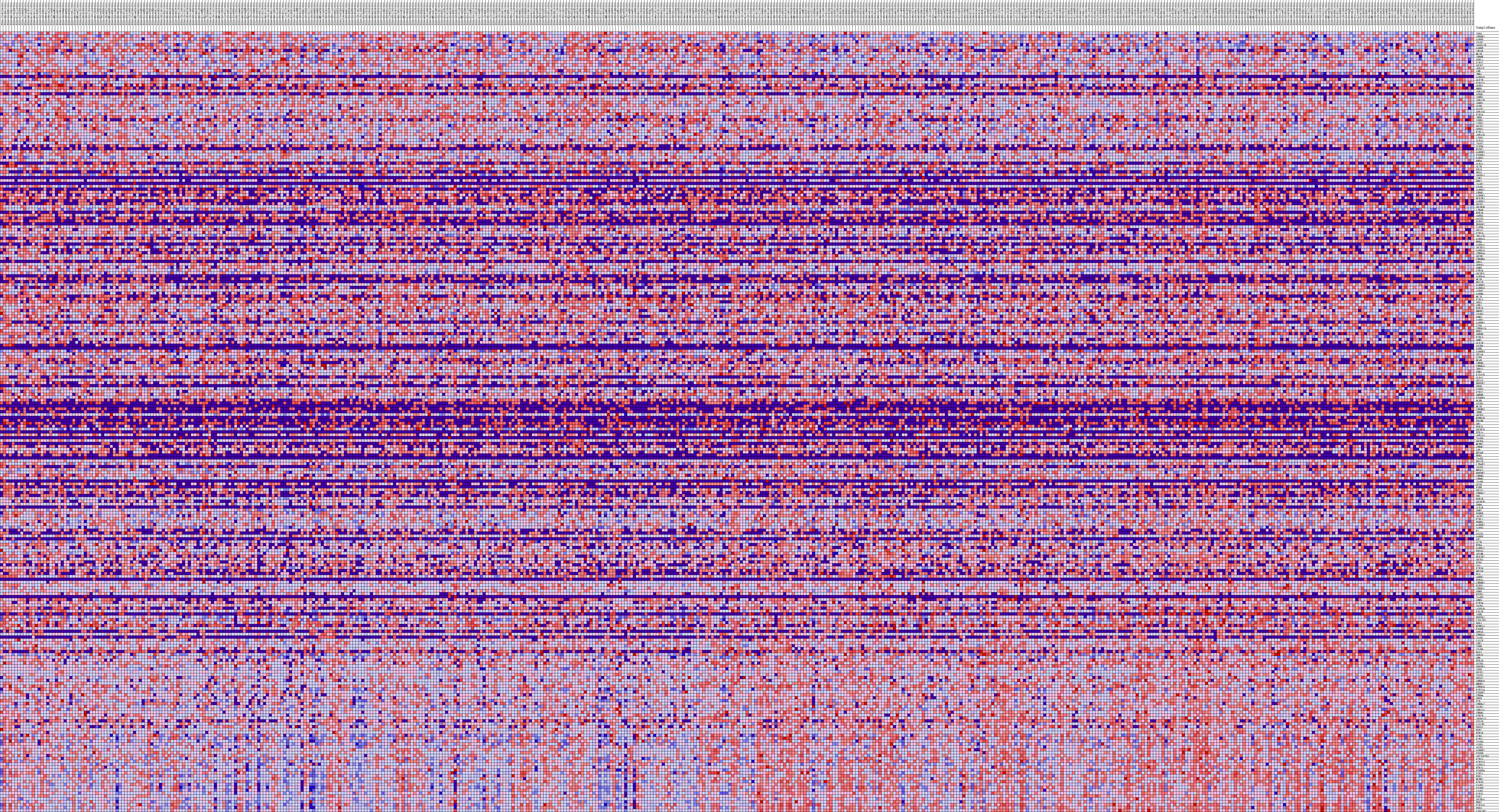

Supplement: Supplementary file 1 [file DataSheet_1.zip › The bioinformatic analysis of MM and lung cancer supplementary materials/Enrichment analysis/MECR/GSEA_4.1.0/LUAD TCGA/KEGG.Gsea.1639041756227/KEGG_NEUROACTIVE_LIGAND_RECEPTOR_INTERACTION_103.png]

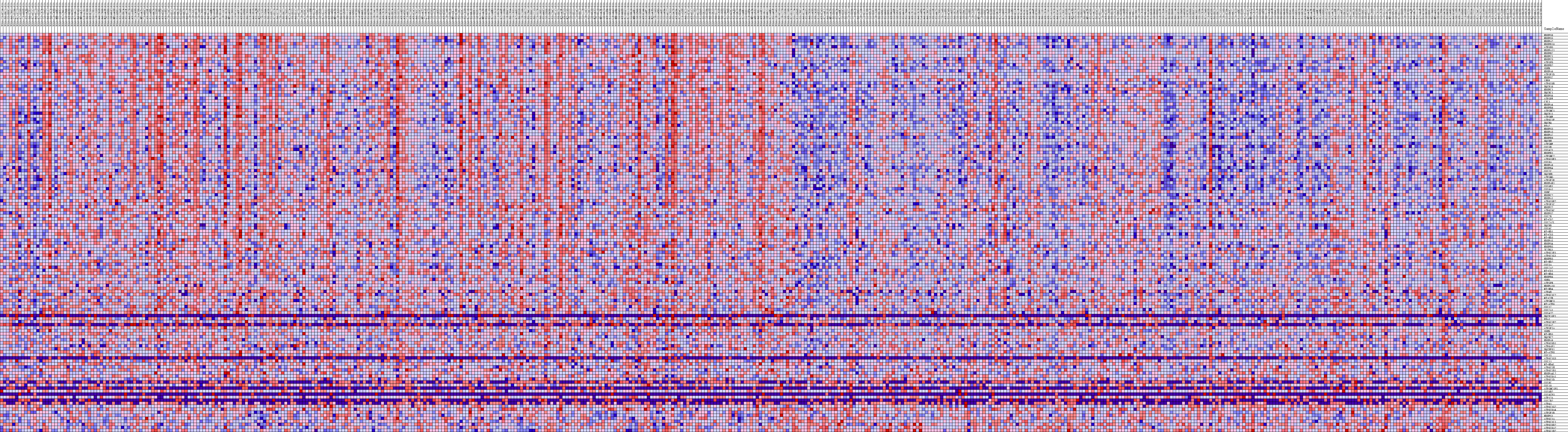

Supplement: Supplementary file 1 [file DataSheet_1.zip › The bioinformatic analysis of MM and lung cancer supplementary materials/Enrichment analysis/MECR/GSEA_4.1.0/LUAD TCGA/KEGG.Gsea.1639041756227/KEGG_OXIDATIVE_PHOSPHORYLATION_7.png]

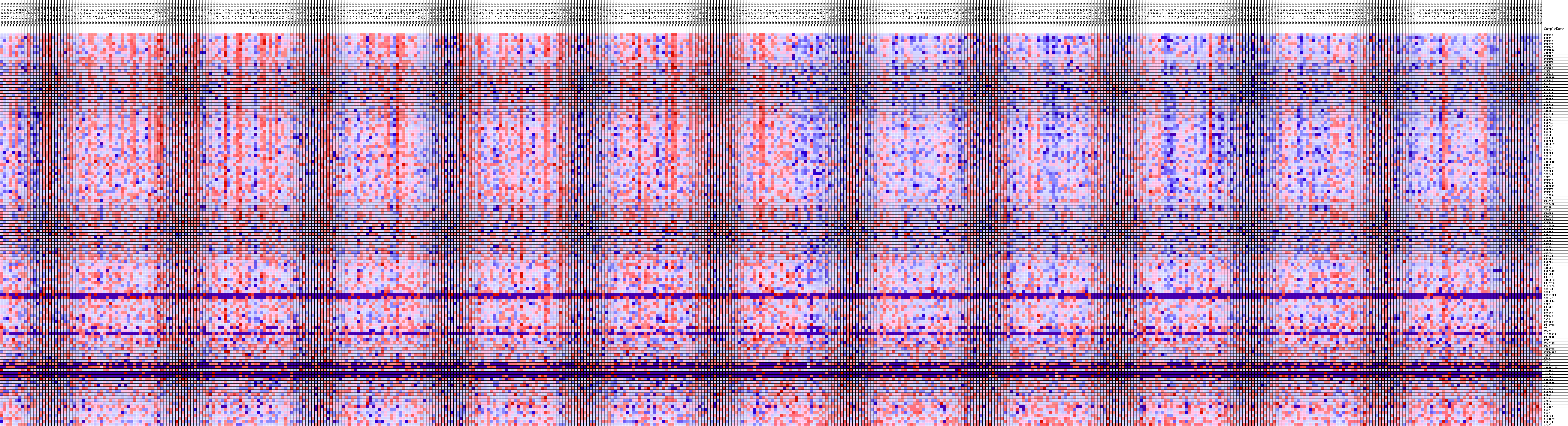

Supplement: Supplementary file 1 [file DataSheet_1.zip › The bioinformatic analysis of MM and lung cancer supplementary materials/Enrichment analysis/MECR/GSEA_4.1.0/LUAD TCGA/KEGG.Gsea.1639041756227/KEGG_PARKINSONS_DISEASE_10.png]

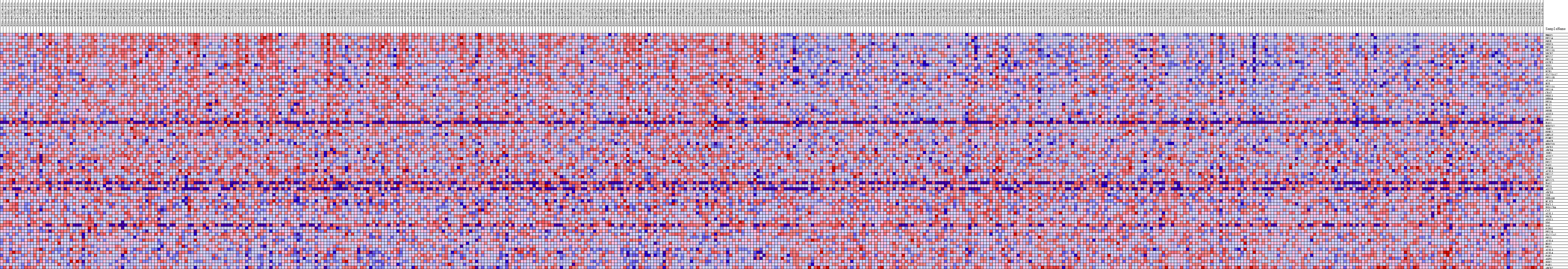

Supplement: Supplementary file 1 [file DataSheet_1.zip › The bioinformatic analysis of MM and lung cancer supplementary materials/Enrichment analysis/MECR/GSEA_4.1.0/LUAD TCGA/KEGG.Gsea.1639041756227/KEGG_PEROXISOME_25.png]

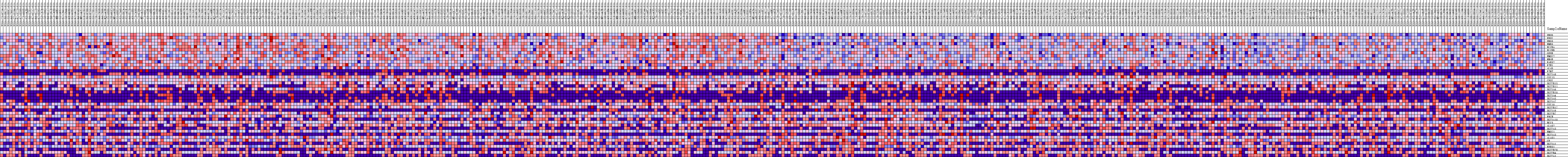

Supplement: Supplementary file 1 [file DataSheet_1.zip › The bioinformatic analysis of MM and lung cancer supplementary materials/Enrichment analysis/MECR/GSEA_4.1.0/LUAD TCGA/KEGG.Gsea.1639041756227/KEGG_PORPHYRIN_AND_CHLOROPHYLL_METABOLISM_40.png]

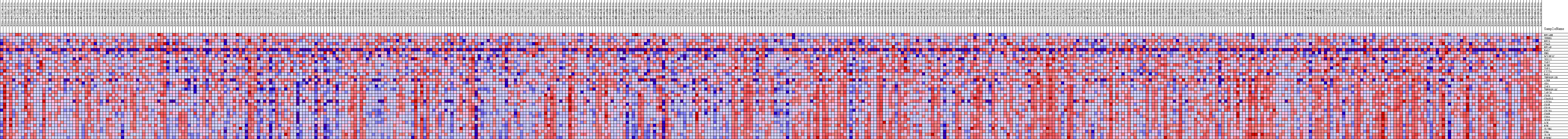

Supplement: Supplementary file 1 [file DataSheet_1.zip › The bioinformatic analysis of MM and lung cancer supplementary materials/Enrichment analysis/MECR/GSEA_4.1.0/LUAD TCGA/KEGG.Gsea.1639041756227/KEGG_PRIMARY_IMMUNODEFICIENCY_97.png]

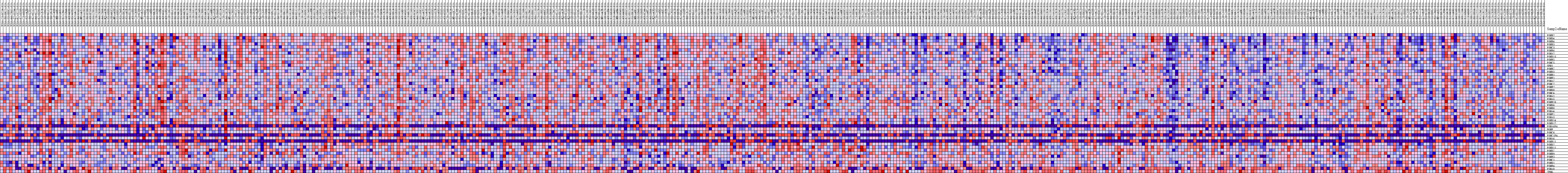

Supplement: Supplementary file 1 [file DataSheet_1.zip › The bioinformatic analysis of MM and lung cancer supplementary materials/Enrichment analysis/MECR/GSEA_4.1.0/LUAD TCGA/KEGG.Gsea.1639041756227/KEGG_PROTEASOME_22.png]

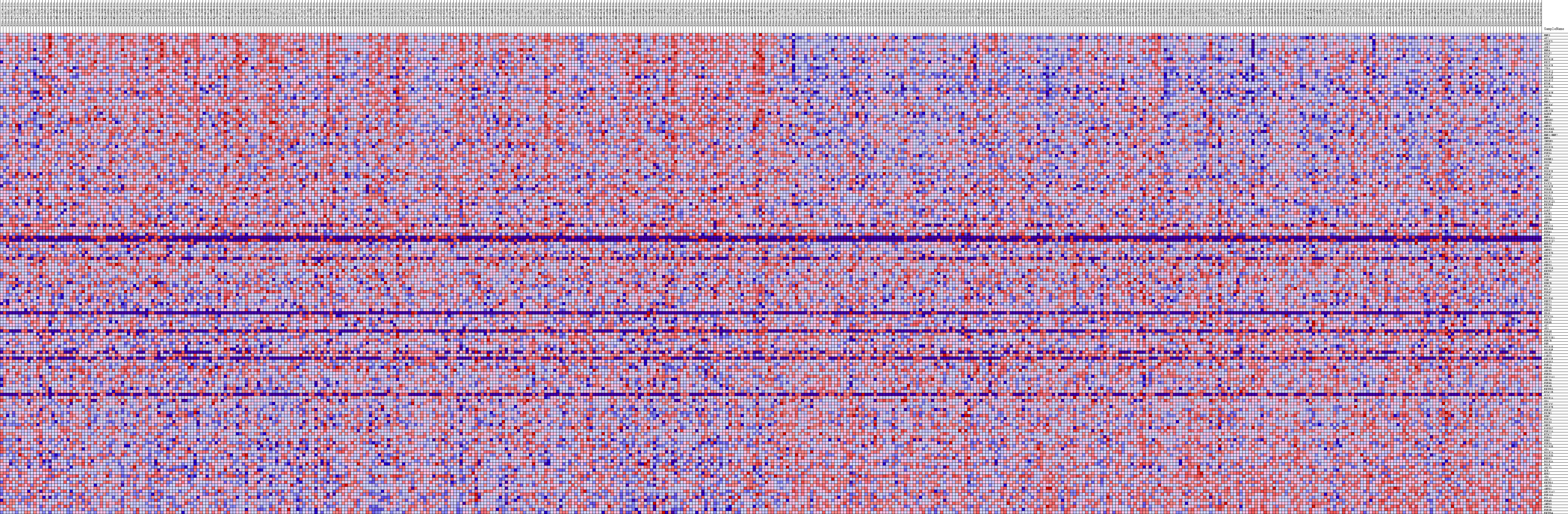

Supplement: Supplementary file 1 [file DataSheet_1.zip › The bioinformatic analysis of MM and lung cancer supplementary materials/Enrichment analysis/MECR/GSEA_4.1.0/LUAD TCGA/KEGG.Gsea.1639041756227/KEGG_PURINE_METABOLISM_46.png]

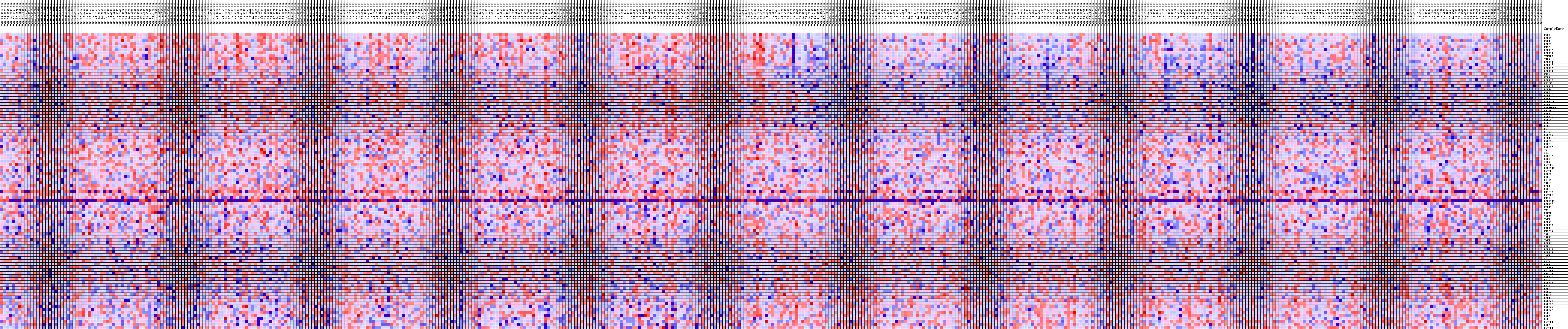

Supplement: Supplementary file 1 [file DataSheet_1.zip › The bioinformatic analysis of MM and lung cancer supplementary materials/Enrichment analysis/MECR/GSEA_4.1.0/LUAD TCGA/KEGG.Gsea.1639041756227/KEGG_PYRIMIDINE_METABOLISM_31.png]

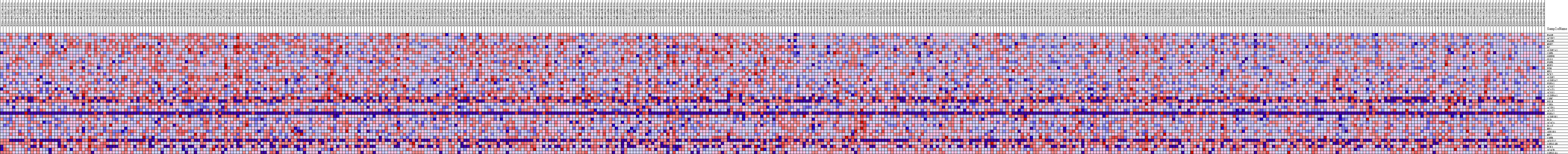

Supplement: Supplementary file 1 [file DataSheet_1.zip › The bioinformatic analysis of MM and lung cancer supplementary materials/Enrichment analysis/MECR/GSEA_4.1.0/LUAD TCGA/KEGG.Gsea.1639041756227/KEGG_PYRUVATE_METABOLISM_43.png]

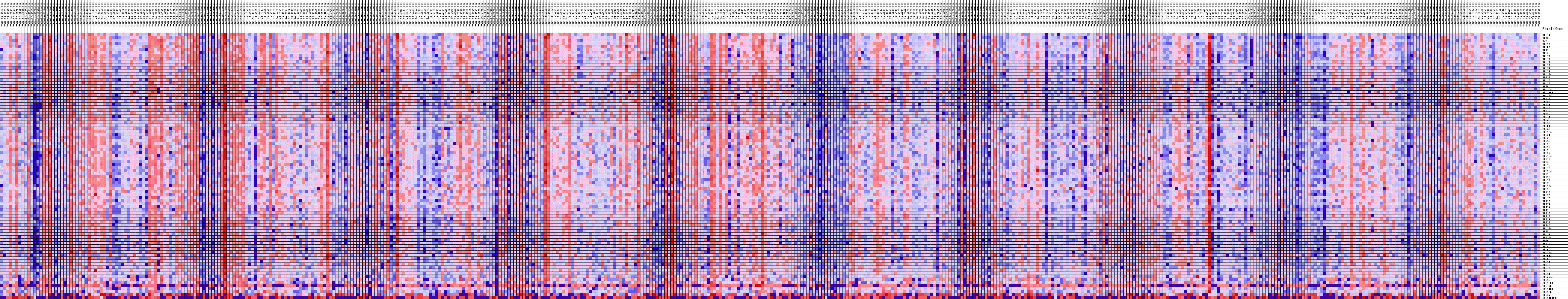

Supplement: Supplementary file 1 [file DataSheet_1.zip › The bioinformatic analysis of MM and lung cancer supplementary materials/Enrichment analysis/MECR/GSEA_4.1.0/LUAD TCGA/KEGG.Gsea.1639041756227/KEGG_RIBOSOME_4.png]

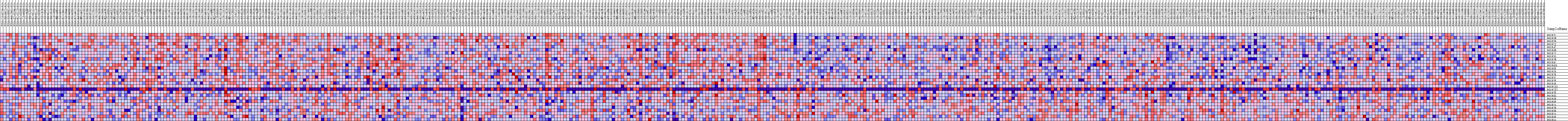

Supplement: Supplementary file 1 [file DataSheet_1.zip › The bioinformatic analysis of MM and lung cancer supplementary materials/Enrichment analysis/MECR/GSEA_4.1.0/LUAD TCGA/KEGG.Gsea.1639041756227/KEGG_RNA_POLYMERASE_37.png]

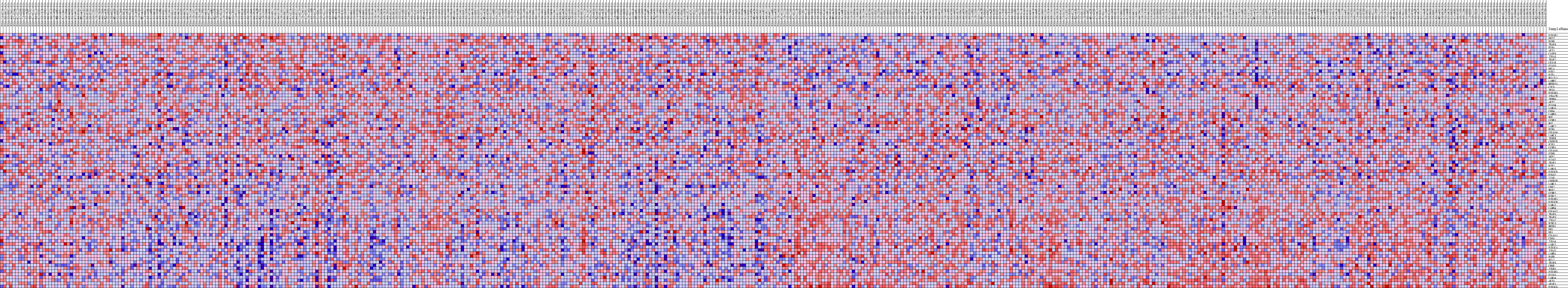

Supplement: Supplementary file 1 [file DataSheet_1.zip › The bioinformatic analysis of MM and lung cancer supplementary materials/Enrichment analysis/MECR/GSEA_4.1.0/LUAD TCGA/KEGG.Gsea.1639041756227/KEGG_SMALL_CELL_LUNG_CANCER_109.png]

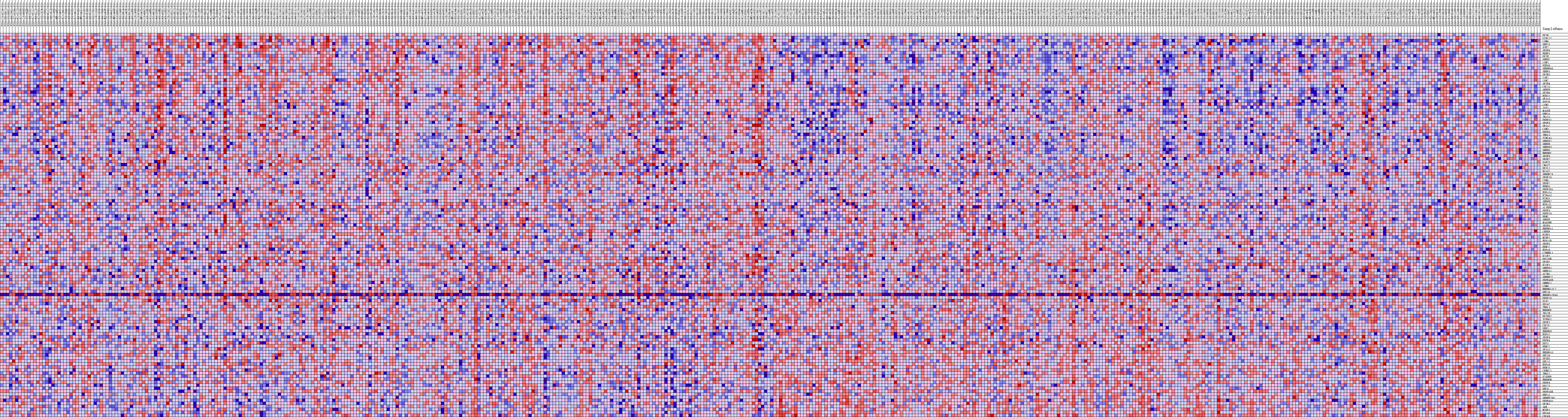

Supplement: Supplementary file 1 [file DataSheet_1.zip › The bioinformatic analysis of MM and lung cancer supplementary materials/Enrichment analysis/MECR/GSEA_4.1.0/LUAD TCGA/KEGG.Gsea.1639041756227/KEGG_SPLICEOSOME_34.png]

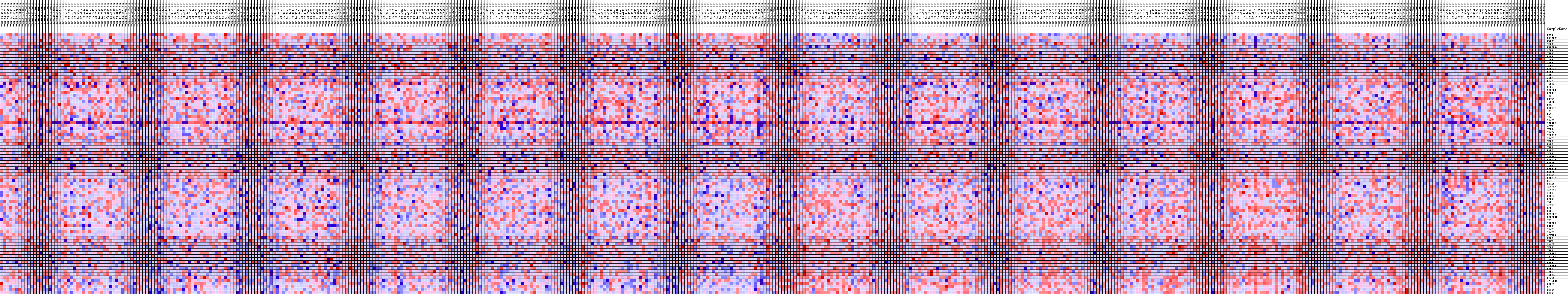

Supplement: Supplementary file 1 [file DataSheet_1.zip › The bioinformatic analysis of MM and lung cancer supplementary materials/Enrichment analysis/MECR/GSEA_4.1.0/LUAD TCGA/KEGG.Gsea.1639041756227/KEGG_TGF_BETA_SIGNALING_PATHWAY_121.png]

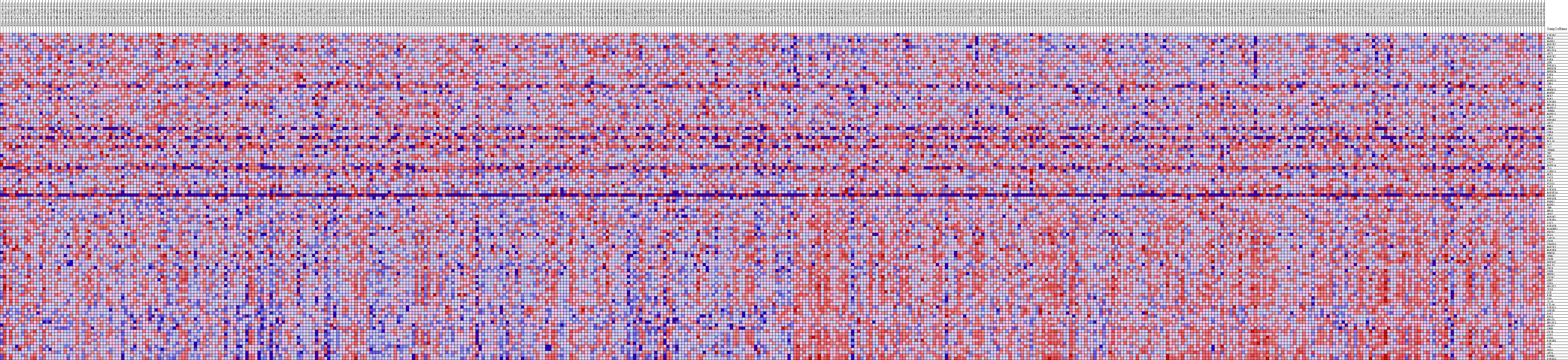

Supplement: Supplementary file 1 [file DataSheet_1.zip › The bioinformatic analysis of MM and lung cancer supplementary materials/Enrichment analysis/MECR/GSEA_4.1.0/LUAD TCGA/KEGG.Gsea.1639041756227/KEGG_T_CELL_RECEPTOR_SIGNALING_PATHWAY_73.png]

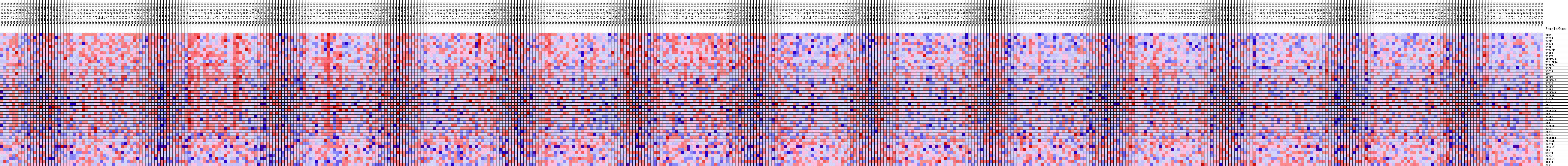

Supplement: Supplementary file 1 [file DataSheet_1.zip › The bioinformatic analysis of MM and lung cancer supplementary materials/Enrichment analysis/MECR/GSEA_4.1.0/LUAD TCGA/KEGG.Gsea.1639041756227/KEGG_VALINE_LEUCINE_AND_ISOLEUCINE_DEGRADATION_28.png]

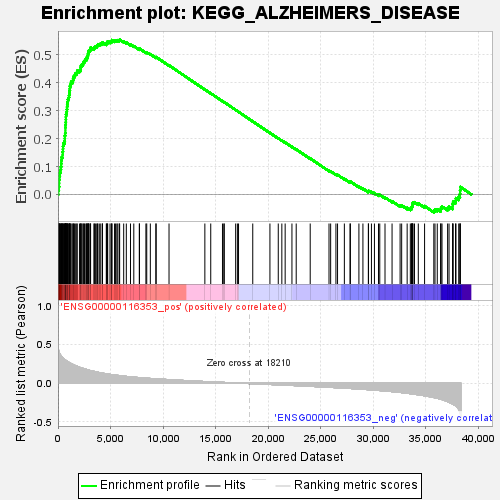

Supplement: Supplementary file 1 [file DataSheet_1.zip › The bioinformatic analysis of MM and lung cancer supplementary materials/Enrichment analysis/MECR/GSEA_4.1.0/LUAD TCGA/KEGG.Gsea.1639041756227/enplot_KEGG_ALZHEIMERS_DISEASE_15.png]

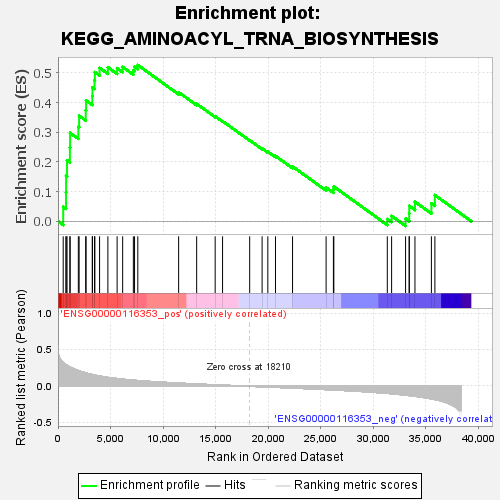

Supplement: Supplementary file 1 [file DataSheet_1.zip › The bioinformatic analysis of MM and lung cancer supplementary materials/Enrichment analysis/MECR/GSEA_4.1.0/LUAD TCGA/KEGG.Gsea.1639041756227/enplot_KEGG_AMINOACYL_TRNA_BIOSYNTHESIS_51.png]
