# Supplementary material for: Simultaneous Presentation of Multiple Myeloma and Lung Cancer: Case Report and Gene Bioinformatics Analysis
Source: Front Oncol. 2022 Jun 13;12:859735. doi: 10.3389/fonc.2022.859735 (PMC9235397; doi:10.3389/fonc.2022.859735)
Supplement: Supplementary file 1 [file DataSheet_1.zip › The bioinformatic analysis of MM and lung cancer supplementary materials/Enrichment analysis/MECR/GSEA_4.1.0/LUAD TCGA/KEGG.Gsea.1639041756227/gsea_report_for_ENSG00000116353_neg_1639041756227.html]

Report for ENSG00000116353\_neg 1639041756227 [GSEA]

| GS  follow link to MSigDB | GS DETAILS | SIZE | ES | NES | NOM p-val | FDR q-val | FWER p-val | RANK AT MAX | LEADING EDGE || 1 | KEGG\_CHEMOKINE\_SIGNALING\_PATHWAY | Details ... | 188 | -0.54 | -2.47 | 0.000 | 0.000 | 0.000 | 8052 | tags=49%, list=20%, signal=62% |
| 2 | KEGG\_CYTOKINE\_CYTOKINE\_RECEPTOR\_INTERACTION | Details ... | 264 | -0.51 | -2.45 | 0.000 | 0.000 | 0.000 | 8229 | tags=46%, list=21%, signal=58% |
| 3 | KEGG\_HEMATOPOIETIC\_CELL\_LINEAGE | Details ... | 85 | -0.61 | -2.44 | 0.000 | 0.000 | 0.000 | 6027 | tags=53%, list=15%, signal=62% |
| 4 | KEGG\_T\_CELL\_RECEPTOR\_SIGNALING\_PATHWAY | Details ... | 108 | -0.58 | -2.41 | 0.000 | 0.000 | 0.000 | 4772 | tags=46%, list=12%, signal=53% |
| 5 | KEGG\_LEISHMANIA\_INFECTION | Details ... | 70 | -0.59 | -2.35 | 0.000 | 0.000 | 0.000 | 6755 | tags=47%, list=17%, signal=57% |
| 6 | KEGG\_CELL\_ADHESION\_MOLECULES\_CAMS | Details ... | 131 | -0.53 | -2.30 | 0.000 | 0.000 | 0.000 | 6583 | tags=43%, list=17%, signal=51% |
| 7 | KEGG\_INTESTINAL\_IMMUNE\_NETWORK\_FOR\_IGA\_PRODUCTION | Details ... | 46 | -0.64 | -2.29 | 0.000 | 0.000 | 0.000 | 6427 | tags=46%, list=16%, signal=55% |
| 8 | KEGG\_ECM\_RECEPTOR\_INTERACTION | Details ... | 84 | -0.57 | -2.29 | 0.000 | 0.000 | 0.000 | 8290 | tags=54%, list=21%, signal=68% |
| 9 | KEGG\_GRAFT\_VERSUS\_HOST\_DISEASE | Details ... | 37 | -0.65 | -2.28 | 0.000 | 0.000 | 0.000 | 6427 | tags=49%, list=16%, signal=58% |
| 10 | KEGG\_NATURAL\_KILLER\_CELL\_MEDIATED\_CYTOTOXICITY | Details ... | 132 | -0.51 | -2.24 | 0.000 | 0.000 | 0.000 | 5732 | tags=39%, list=15%, signal=46% |
| 11 | KEGG\_JAK\_STAT\_SIGNALING\_PATHWAY | Details ... | 155 | -0.50 | -2.22 | 0.000 | 0.000 | 0.001 | 7764 | tags=40%, list=20%, signal=50% |
| 12 | KEGG\_PRIMARY\_IMMUNODEFICIENCY | Details ... | 35 | -0.65 | -2.21 | 0.000 | 0.000 | 0.001 | 7327 | tags=71%, list=19%, signal=88% |
| 13 | KEGG\_FOCAL\_ADHESION | Details ... | 199 | -0.48 | -2.20 | 0.000 | 0.000 | 0.001 | 8290 | tags=48%, list=21%, signal=60% |
| 14 | KEGG\_NEUROACTIVE\_LIGAND\_RECEPTOR\_INTERACTION | Details ... | 270 | -0.46 | -2.18 | 0.000 | 0.000 | 0.001 | 10351 | tags=43%, list=26%, signal=57% |
| 15 | KEGG\_CALCIUM\_SIGNALING\_PATHWAY | Details ... | 178 | -0.47 | -2.11 | 0.000 | 0.000 | 0.003 | 9797 | tags=48%, list=25%, signal=64% |
| 16 | KEGG\_SMALL\_CELL\_LUNG\_CANCER | Details ... | 84 | -0.52 | -2.10 | 0.000 | 0.000 | 0.003 | 6362 | tags=44%, list=16%, signal=52% |
| 17 | KEGG\_B\_CELL\_RECEPTOR\_SIGNALING\_PATHWAY | Details ... | 75 | -0.53 | -2.10 | 0.000 | 0.000 | 0.003 | 7471 | tags=55%, list=19%, signal=67% |
| 18 | KEGG\_ARRHYTHMOGENIC\_RIGHT\_VENTRICULAR\_CARDIOMYOPATHY\_ARVC | Details ... | 74 | -0.51 | -2.04 | 0.000 | 0.000 | 0.006 | 7756 | tags=46%, list=20%, signal=57% |
| 19 | KEGG\_LEUKOCYTE\_TRANSENDOTHELIAL\_MIGRATION | Details ... | 116 | -0.48 | -2.04 | 0.000 | 0.000 | 0.006 | 5634 | tags=40%, list=14%, signal=46% |
| 20 | KEGG\_TGF\_BETA\_SIGNALING\_PATHWAY | Details ... | 86 | -0.50 | -2.04 | 0.000 | 0.000 | 0.006 | 5689 | tags=37%, list=14%, signal=43% |
| 21 | KEGG\_PATHWAYS\_IN\_CANCER |  | 325 | -0.42 | -2.02 | 0.000 | 0.001 | 0.011 | 7975 | tags=40%, list=20%, signal=50% |
| 22 | KEGG\_FC\_GAMMA\_R\_MEDIATED\_PHAGOCYTOSIS |  | 96 | -0.48 | -2.02 | 0.000 | 0.001 | 0.011 | 4904 | tags=36%, list=12%, signal=42% |
| 23 | KEGG\_TOLL\_LIKE\_RECEPTOR\_SIGNALING\_PATHWAY |  | 102 | -0.48 | -2.01 | 0.000 | 0.001 | 0.012 | 6145 | tags=36%, list=16%, signal=43% |
| 24 | KEGG\_DILATED\_CARDIOMYOPATHY |  | 90 | -0.49 | -2.00 | 0.000 | 0.001 | 0.012 | 8575 | tags=48%, list=22%, signal=61% |
| 25 | KEGG\_COMPLEMENT\_AND\_COAGULATION\_CASCADES |  | 69 | -0.50 | -1.99 | 0.000 | 0.001 | 0.013 | 10139 | tags=48%, list=26%, signal=64% |
| 26 | KEGG\_NOD\_LIKE\_RECEPTOR\_SIGNALING\_PATHWAY |  | 62 | -0.52 | -1.98 | 0.000 | 0.001 | 0.013 | 6362 | tags=45%, list=16%, signal=54% |
| 27 | KEGG\_HYPERTROPHIC\_CARDIOMYOPATHY\_HCM |  | 83 | -0.49 | -1.97 | 0.000 | 0.001 | 0.014 | 8290 | tags=48%, list=21%, signal=61% |
| 28 | KEGG\_ALLOGRAFT\_REJECTION |  | 35 | -0.57 | -1.96 | 0.000 | 0.001 | 0.014 | 6427 | tags=34%, list=16%, signal=41% |
| 29 | KEGG\_TYPE\_I\_DIABETES\_MELLITUS |  | 41 | -0.57 | -1.96 | 0.000 | 0.001 | 0.015 | 7238 | tags=41%, list=18%, signal=51% |
| 30 | KEGG\_VASCULAR\_SMOOTH\_MUSCLE\_CONTRACTION |  | 115 | -0.46 | -1.95 | 0.000 | 0.001 | 0.017 | 7820 | tags=44%, list=20%, signal=55% |
| 31 | KEGG\_REGULATION\_OF\_ACTIN\_CYTOSKELETON |  | 213 | -0.42 | -1.94 | 0.000 | 0.001 | 0.021 | 5795 | tags=33%, list=15%, signal=39% |
| 32 | KEGG\_DORSO\_VENTRAL\_AXIS\_FORMATION |  | 24 | -0.63 | -1.93 | 0.000 | 0.001 | 0.021 | 5689 | tags=54%, list=14%, signal=63% |
| 33 | KEGG\_PHOSPHATIDYLINOSITOL\_SIGNALING\_SYSTEM |  | 76 | -0.49 | -1.91 | 0.000 | 0.001 | 0.030 | 8659 | tags=53%, list=22%, signal=67% |
| 34 | KEGG\_CHRONIC\_MYELOID\_LEUKEMIA |  | 73 | -0.48 | -1.89 | 0.000 | 0.001 | 0.035 | 6173 | tags=41%, list=16%, signal=49% |
| 35 | KEGG\_ADHERENS\_JUNCTION |  | 73 | -0.48 | -1.88 | 0.000 | 0.001 | 0.038 | 7698 | tags=49%, list=20%, signal=61% |
| 36 | KEGG\_TYPE\_II\_DIABETES\_MELLITUS |  | 47 | -0.51 | -1.83 | 0.002 | 0.002 | 0.065 | 8837 | tags=55%, list=22%, signal=71% |
| 37 | KEGG\_FC\_EPSILON\_RI\_SIGNALING\_PATHWAY |  | 79 | -0.45 | -1.82 | 0.000 | 0.002 | 0.071 | 7820 | tags=44%, list=20%, signal=55% |
| 38 | KEGG\_PROSTATE\_CANCER |  | 89 | -0.44 | -1.81 | 0.000 | 0.002 | 0.079 | 6335 | tags=42%, list=16%, signal=49% |
| 39 | KEGG\_GAP\_JUNCTION |  | 90 | -0.43 | -1.75 | 0.000 | 0.005 | 0.164 | 9817 | tags=50%, list=25%, signal=66% |
| 40 | KEGG\_MATURITY\_ONSET\_DIABETES\_OF\_THE\_YOUNG |  | 25 | -0.56 | -1.75 | 0.002 | 0.005 | 0.172 | 8837 | tags=52%, list=22%, signal=67% |
| 41 | KEGG\_MAPK\_SIGNALING\_PATHWAY |  | 267 | -0.37 | -1.75 | 0.000 | 0.005 | 0.176 | 8760 | tags=39%, list=22%, signal=49% |
| 42 | KEGG\_SPHINGOLIPID\_METABOLISM |  | 39 | -0.50 | -1.71 | 0.004 | 0.008 | 0.256 | 5615 | tags=41%, list=14%, signal=48% |
| 43 | KEGG\_AUTOIMMUNE\_THYROID\_DISEASE |  | 50 | -0.48 | -1.71 | 0.000 | 0.008 | 0.265 | 7916 | tags=30%, list=20%, signal=38% |
| 44 | KEGG\_GNRH\_SIGNALING\_PATHWAY |  | 101 | -0.41 | -1.71 | 0.000 | 0.008 | 0.270 | 7916 | tags=41%, list=20%, signal=51% |
| 45 | KEGG\_INOSITOL\_PHOSPHATE\_METABOLISM |  | 54 | -0.46 | -1.70 | 0.002 | 0.008 | 0.273 | 5557 | tags=39%, list=14%, signal=45% |
| 46 | KEGG\_ACUTE\_MYELOID\_LEUKEMIA |  | 57 | -0.46 | -1.70 | 0.002 | 0.008 | 0.288 | 7931 | tags=49%, list=20%, signal=61% |
| 47 | KEGG\_LONG\_TERM\_POTENTIATION |  | 70 | -0.43 | -1.70 | 0.002 | 0.008 | 0.288 | 7803 | tags=43%, list=20%, signal=53% |
| 48 | KEGG\_PRION\_DISEASES |  | 35 | -0.50 | -1.69 | 0.004 | 0.008 | 0.293 | 6761 | tags=54%, list=17%, signal=65% |
| 49 | KEGG\_RENAL\_CELL\_CARCINOMA |  | 70 | -0.43 | -1.69 | 0.002 | 0.008 | 0.303 | 5027 | tags=39%, list=13%, signal=44% |
| 50 | KEGG\_ENDOCYTOSIS |  | 181 | -0.37 | -1.67 | 0.000 | 0.009 | 0.342 | 5685 | tags=31%, list=14%, signal=37% |
| 51 | KEGG\_GLIOMA |  | 65 | -0.43 | -1.67 | 0.004 | 0.010 | 0.354 | 8566 | tags=45%, list=22%, signal=57% |
| 52 | KEGG\_ADIPOCYTOKINE\_SIGNALING\_PATHWAY |  | 67 | -0.43 | -1.66 | 0.002 | 0.010 | 0.375 | 7118 | tags=40%, list=18%, signal=49% |
| 53 | KEGG\_PROGESTERONE\_MEDIATED\_OOCYTE\_MATURATION |  | 85 | -0.41 | -1.66 | 0.000 | 0.010 | 0.376 | 8842 | tags=45%, list=22%, signal=58% |
| 54 | KEGG\_COLORECTAL\_CANCER |  | 62 | -0.43 | -1.65 | 0.004 | 0.011 | 0.418 | 5689 | tags=34%, list=14%, signal=40% |
| 55 | KEGG\_PANCREATIC\_CANCER |  | 70 | -0.42 | -1.64 | 0.006 | 0.012 | 0.431 | 5013 | tags=33%, list=13%, signal=38% |
| 56 | KEGG\_HEDGEHOG\_SIGNALING\_PATHWAY |  | 56 | -0.44 | -1.62 | 0.006 | 0.013 | 0.491 | 8773 | tags=45%, list=22%, signal=57% |
| 57 | KEGG\_ERBB\_SIGNALING\_PATHWAY |  | 87 | -0.40 | -1.62 | 0.006 | 0.013 | 0.497 | 8641 | tags=41%, list=22%, signal=53% |
| 58 | KEGG\_O\_GLYCAN\_BIOSYNTHESIS |  | 30 | -0.48 | -1.62 | 0.006 | 0.014 | 0.506 | 9013 | tags=47%, list=23%, signal=60% |
| 59 | KEGG\_AXON\_GUIDANCE |  | 129 | -0.38 | -1.60 | 0.002 | 0.016 | 0.557 | 6730 | tags=33%, list=17%, signal=40% |
| 60 | KEGG\_APOPTOSIS |  | 87 | -0.39 | -1.60 | 0.004 | 0.016 | 0.569 | 7466 | tags=39%, list=19%, signal=48% |
| 61 | KEGG\_NEUROTROPHIN\_SIGNALING\_PATHWAY |  | 126 | -0.37 | -1.60 | 0.002 | 0.016 | 0.572 | 6011 | tags=31%, list=15%, signal=36% |
| 62 | KEGG\_LONG\_TERM\_DEPRESSION |  | 70 | -0.41 | -1.59 | 0.006 | 0.016 | 0.586 | 8961 | tags=44%, list=23%, signal=57% |
| 63 | KEGG\_NON\_SMALL\_CELL\_LUNG\_CANCER |  | 54 | -0.42 | -1.58 | 0.012 | 0.018 | 0.637 | 6011 | tags=41%, list=15%, signal=48% |
| 64 | KEGG\_ABC\_TRANSPORTERS |  | 44 | -0.44 | -1.56 | 0.020 | 0.021 | 0.695 | 10505 | tags=59%, list=27%, signal=81% |
| 65 | KEGG\_ANTIGEN\_PROCESSING\_AND\_PRESENTATION |  | 81 | -0.39 | -1.55 | 0.004 | 0.022 | 0.715 | 7042 | tags=32%, list=18%, signal=39% |
| 66 | KEGG\_ENDOMETRIAL\_CANCER |  | 52 | -0.42 | -1.52 | 0.022 | 0.029 | 0.801 | 6335 | tags=37%, list=16%, signal=43% |
| 67 | KEGG\_ALDOSTERONE\_REGULATED\_SODIUM\_REABSORPTION |  | 42 | -0.42 | -1.51 | 0.021 | 0.031 | 0.834 | 8837 | tags=48%, list=22%, signal=61% |
| 68 | KEGG\_CELL\_CYCLE |  | 125 | -0.35 | -1.51 | 0.000 | 0.030 | 0.835 | 9814 | tags=40%, list=25%, signal=53% |
| 69 | KEGG\_MELANOMA |  | 71 | -0.39 | -1.51 | 0.010 | 0.030 | 0.837 | 4772 | tags=28%, list=12%, signal=32% |
| 70 | KEGG\_TASTE\_TRANSDUCTION |  | 51 | -0.42 | -1.50 | 0.026 | 0.031 | 0.845 | 11476 | tags=39%, list=29%, signal=55% |
| 71 | KEGG\_MELANOGENESIS |  | 101 | -0.36 | -1.50 | 0.008 | 0.032 | 0.852 | 9049 | tags=43%, list=23%, signal=55% |
| 72 | KEGG\_INSULIN\_SIGNALING\_PATHWAY |  | 137 | -0.34 | -1.49 | 0.002 | 0.033 | 0.873 | 7118 | tags=34%, list=18%, signal=42% |
| 73 | KEGG\_VIRAL\_MYOCARDITIS |  | 68 | -0.38 | -1.48 | 0.020 | 0.034 | 0.885 | 8215 | tags=32%, list=21%, signal=41% |
| 74 | KEGG\_OOCYTE\_MEIOSIS |  | 113 | -0.35 | -1.46 | 0.015 | 0.040 | 0.932 | 8881 | tags=41%, list=23%, signal=52% |
| 75 | KEGG\_WNT\_SIGNALING\_PATHWAY |  | 151 | -0.32 | -1.40 | 0.002 | 0.061 | 0.982 | 8341 | tags=36%, list=21%, signal=45% |
| 76 | KEGG\_ETHER\_LIPID\_METABOLISM |  | 33 | -0.41 | -1.38 | 0.063 | 0.074 | 0.991 | 7820 | tags=36%, list=20%, signal=45% |
| 77 | KEGG\_ASTHMA |  | 28 | -0.42 | -1.34 | 0.095 | 0.095 | 0.997 | 6427 | tags=25%, list=16%, signal=30% |
| 78 | KEGG\_UBIQUITIN\_MEDIATED\_PROTEOLYSIS |  | 135 | -0.31 | -1.34 | 0.031 | 0.095 | 0.997 | 5307 | tags=25%, list=13%, signal=29% |
| 79 | KEGG\_SYSTEMIC\_LUPUS\_ERYTHEMATOSUS |  | 135 | -0.31 | -1.33 | 0.032 | 0.098 | 0.998 | 11161 | tags=34%, list=28%, signal=47% |
| 80 | KEGG\_RENIN\_ANGIOTENSIN\_SYSTEM |  | 17 | -0.46 | -1.31 | 0.138 | 0.111 | 1.000 | 5585 | tags=35%, list=14%, signal=41% |
| 81 | KEGG\_CYTOSOLIC\_DNA\_SENSING\_PATHWAY |  | 55 | -0.33 | -1.24 | 0.126 | 0.171 | 1.000 | 7049 | tags=35%, list=18%, signal=42% |
| 82 | KEGG\_TIGHT\_JUNCTION |  | 132 | -0.29 | -1.23 | 0.094 | 0.173 | 1.000 | 5473 | tags=26%, list=14%, signal=30% |
| 83 | KEGG\_VEGF\_SIGNALING\_PATHWAY |  | 76 | -0.31 | -1.23 | 0.112 | 0.172 | 1.000 | 7820 | tags=37%, list=20%, signal=46% |
| 84 | KEGG\_EPITHELIAL\_CELL\_SIGNALING\_IN\_HELICOBACTER\_PYLORI\_INFECTION |  | 68 | -0.31 | -1.22 | 0.149 | 0.187 | 1.000 | 8641 | tags=35%, list=22%, signal=45% |
| 85 | KEGG\_BASAL\_CELL\_CARCINOMA |  | 55 | -0.31 | -1.16 | 0.200 | 0.253 | 1.000 | 7562 | tags=29%, list=19%, signal=36% |
| 86 | KEGG\_GLYCOSAMINOGLYCAN\_BIOSYNTHESIS\_HEPARAN\_SULFATE |  | 26 | -0.37 | -1.16 | 0.247 | 0.254 | 1.000 | 6726 | tags=42%, list=17%, signal=51% |
| 87 | KEGG\_RIG\_I\_LIKE\_RECEPTOR\_SIGNALING\_PATHWAY |  | 71 | -0.29 | -1.14 | 0.242 | 0.278 | 1.000 | 6973 | tags=27%, list=18%, signal=32% |
| 88 | KEGG\_GLYCOSAMINOGLYCAN\_BIOSYNTHESIS\_CHONDROITIN\_SULFATE |  | 22 | -0.37 | -1.11 | 0.309 | 0.317 | 1.000 | 3001 | tags=32%, list=8%, signal=34% |
| 89 | KEGG\_VIBRIO\_CHOLERAE\_INFECTION |  | 54 | -0.29 | -1.08 | 0.317 | 0.357 | 1.000 | 6469 | tags=31%, list=16%, signal=38% |
| 90 | KEGG\_MTOR\_SIGNALING\_PATHWAY |  | 52 | -0.29 | -1.08 | 0.339 | 0.359 | 1.000 | 6116 | tags=33%, list=16%, signal=39% |
| 91 | KEGG\_P53\_SIGNALING\_PATHWAY |  | 68 | -0.27 | -1.05 | 0.348 | 0.398 | 1.000 | 9824 | tags=34%, list=25%, signal=45% |
| 92 | KEGG\_PATHOGENIC\_ESCHERICHIA\_COLI\_INFECTION |  | 56 | -0.27 | -1.02 | 0.402 | 0.449 | 1.000 | 3390 | tags=14%, list=9%, signal=16% |
| 93 | KEGG\_NOTCH\_SIGNALING\_PATHWAY |  | 47 | -0.28 | -1.01 | 0.438 | 0.465 | 1.000 | 4696 | tags=30%, list=12%, signal=34% |
| 94 | KEGG\_VASOPRESSIN\_REGULATED\_WATER\_REABSORPTION |  | 44 | -0.28 | -1.00 | 0.452 | 0.486 | 1.000 | 6285 | tags=25%, list=16%, signal=30% |
| 95 | KEGG\_LYSINE\_DEGRADATION |  | 44 | -0.27 | -0.98 | 0.502 | 0.512 | 1.000 | 6488 | tags=30%, list=16%, signal=35% |
| 96 | KEGG\_THYROID\_CANCER |  | 29 | -0.31 | -0.98 | 0.479 | 0.516 | 1.000 | 6335 | tags=31%, list=16%, signal=37% |
| 97 | KEGG\_REGULATION\_OF\_AUTOPHAGY |  | 35 | -0.28 | -0.94 | 0.538 | 0.602 | 1.000 | 9401 | tags=31%, list=24%, signal=41% |
| 98 | KEGG\_STARCH\_AND\_SUCROSE\_METABOLISM |  | 52 | -0.21 | -0.79 | 0.854 | 0.880 | 1.000 | 10467 | tags=29%, list=27%, signal=39% |
| 99 | KEGG\_LINOLEIC\_ACID\_METABOLISM |  | 29 | -0.23 | -0.76 | 0.816 | 0.907 | 1.000 | 7820 | tags=24%, list=20%, signal=30% |
| 100 | KEGG\_ALPHA\_LINOLENIC\_ACID\_METABOLISM |  | 19 | -0.25 | -0.73 | 0.832 | 0.923 | 1.000 | 7820 | tags=32%, list=20%, signal=39% |
| 101 | KEGG\_OLFACTORY\_TRANSDUCTION |  | 386 | -0.11 | -0.53 | 1.000 | 0.996 | 1.000 | 16709 | tags=36%, list=42%, signal=62% |
Table: Gene sets enriched in phenotype **negative correlation with profile **[plain text format]****
