# Supplementary material for: Simultaneous Presentation of Multiple Myeloma and Lung Cancer: Case Report and Gene Bioinformatics Analysis
Source: Front Oncol. 2022 Jun 13;12:859735. doi: 10.3389/fonc.2022.859735 (PMC9235397; doi:10.3389/fonc.2022.859735)
Supplement: Supplementary file 1 [file DataSheet_1.zip › The bioinformatic analysis of MM and lung cancer supplementary materials/Enrichment analysis/MECR/GSEA_4.1.0/LUAD TCGA/KEGG.Gsea.1639041756227/gsea_report_for_ENSG00000116353_pos_1639041756227.html]

Report for ENSG00000116353\_pos 1639041756227 [GSEA]

| GS  follow link to MSigDB | GS DETAILS | SIZE | ES | NES | NOM p-val | FDR q-val | FWER p-val | RANK AT MAX | LEADING EDGE || 1 | KEGG\_RIBOSOME | Details ... | 88 | 0.85 | 3.44 | 0.000 | 0.000 | 0.000 | 4764 | tags=90%, list=12%, signal=102% |
| 2 | KEGG\_OXIDATIVE\_PHOSPHORYLATION | Details ... | 132 | 0.76 | 3.25 | 0.000 | 0.000 | 0.000 | 5690 | tags=74%, list=14%, signal=87% |
| 3 | KEGG\_PARKINSONS\_DISEASE | Details ... | 130 | 0.72 | 3.09 | 0.000 | 0.000 | 0.000 | 5130 | tags=67%, list=13%, signal=77% |
| 4 | KEGG\_HUNTINGTONS\_DISEASE | Details ... | 182 | 0.60 | 2.69 | 0.000 | 0.000 | 0.000 | 5690 | tags=54%, list=14%, signal=63% |
| 5 | KEGG\_ALZHEIMERS\_DISEASE | Details ... | 166 | 0.55 | 2.46 | 0.000 | 0.000 | 0.000 | 5864 | tags=55%, list=15%, signal=64% |
| 6 | KEGG\_GLUTATHIONE\_METABOLISM | Details ... | 49 | 0.63 | 2.29 | 0.000 | 0.000 | 0.000 | 6075 | tags=47%, list=15%, signal=55% |
| 7 | KEGG\_PROTEASOME | Details ... | 46 | 0.64 | 2.27 | 0.000 | 0.000 | 0.000 | 6453 | tags=61%, list=16%, signal=73% |
| 8 | KEGG\_PEROXISOME | Details ... | 78 | 0.56 | 2.20 | 0.000 | 0.000 | 0.000 | 3169 | tags=36%, list=8%, signal=39% |
| 9 | KEGG\_VALINE\_LEUCINE\_AND\_ISOLEUCINE\_DEGRADATION | Details ... | 44 | 0.59 | 2.10 | 0.000 | 0.000 | 0.002 | 8122 | tags=61%, list=21%, signal=77% |
| 10 | KEGG\_PYRIMIDINE\_METABOLISM | Details ... | 98 | 0.49 | 2.02 | 0.000 | 0.001 | 0.014 | 6932 | tags=48%, list=18%, signal=58% |
| 11 | KEGG\_SPLICEOSOME | Details ... | 127 | 0.47 | 2.00 | 0.000 | 0.001 | 0.018 | 6686 | tags=45%, list=17%, signal=54% |
| 12 | KEGG\_RNA\_POLYMERASE | Details ... | 29 | 0.62 | 1.97 | 0.002 | 0.002 | 0.023 | 2640 | tags=45%, list=7%, signal=48% |
| 13 | KEGG\_PORPHYRIN\_AND\_CHLOROPHYLL\_METABOLISM | Details ... | 41 | 0.57 | 1.95 | 0.000 | 0.002 | 0.029 | 3673 | tags=27%, list=9%, signal=30% |
| 14 | KEGG\_PYRUVATE\_METABOLISM | Details ... | 40 | 0.54 | 1.87 | 0.000 | 0.004 | 0.066 | 7982 | tags=55%, list=20%, signal=69% |
| 15 | KEGG\_PURINE\_METABOLISM | Details ... | 159 | 0.41 | 1.84 | 0.000 | 0.006 | 0.108 | 3391 | tags=28%, list=9%, signal=30% |
| 16 | KEGG\_FATTY\_ACID\_METABOLISM | Details ... | 42 | 0.52 | 1.83 | 0.002 | 0.006 | 0.109 | 5537 | tags=40%, list=14%, signal=47% |
| 17 | KEGG\_AMINOACYL\_TRNA\_BIOSYNTHESIS | Details ... | 41 | 0.53 | 1.83 | 0.002 | 0.006 | 0.113 | 7593 | tags=51%, list=19%, signal=63% |
| 18 | KEGG\_ARGININE\_AND\_PROLINE\_METABOLISM | Details ... | 54 | 0.50 | 1.81 | 0.000 | 0.007 | 0.137 | 5202 | tags=31%, list=13%, signal=36% |
| 19 | KEGG\_GLYCOSYLPHOSPHATIDYLINOSITOL\_GPI\_ANCHOR\_BIOSYNTHESIS | Details ... | 25 | 0.57 | 1.76 | 0.006 | 0.010 | 0.202 | 3253 | tags=32%, list=8%, signal=35% |
| 20 | KEGG\_CITRATE\_CYCLE\_TCA\_CYCLE | Details ... | 31 | 0.52 | 1.72 | 0.015 | 0.015 | 0.303 | 5934 | tags=52%, list=15%, signal=61% |
| 21 | KEGG\_BASE\_EXCISION\_REPAIR |  | 35 | 0.51 | 1.71 | 0.008 | 0.015 | 0.312 | 6932 | tags=49%, list=18%, signal=59% |
| 22 | KEGG\_CARDIAC\_MUSCLE\_CONTRACTION |  | 79 | 0.43 | 1.71 | 0.002 | 0.015 | 0.323 | 5130 | tags=37%, list=13%, signal=42% |
| 23 | KEGG\_TYROSINE\_METABOLISM |  | 42 | 0.49 | 1.70 | 0.008 | 0.016 | 0.343 | 3057 | tags=29%, list=8%, signal=31% |
| 24 | KEGG\_SNARE\_INTERACTIONS\_IN\_VESICULAR\_TRANSPORT |  | 38 | 0.47 | 1.65 | 0.011 | 0.023 | 0.483 | 5030 | tags=45%, list=13%, signal=51% |
| 25 | KEGG\_NUCLEOTIDE\_EXCISION\_REPAIR |  | 44 | 0.47 | 1.65 | 0.006 | 0.022 | 0.489 | 3360 | tags=30%, list=9%, signal=32% |
| 26 | KEGG\_BUTANOATE\_METABOLISM |  | 34 | 0.50 | 1.65 | 0.012 | 0.021 | 0.490 | 6430 | tags=41%, list=16%, signal=49% |
| 27 | KEGG\_TERPENOID\_BACKBONE\_BIOSYNTHESIS |  | 15 | 0.61 | 1.64 | 0.030 | 0.024 | 0.544 | 2665 | tags=40%, list=7%, signal=43% |
| 28 | KEGG\_PENTOSE\_PHOSPHATE\_PATHWAY |  | 27 | 0.53 | 1.64 | 0.010 | 0.024 | 0.550 | 2772 | tags=26%, list=7%, signal=28% |
| 29 | KEGG\_AMINO\_SUGAR\_AND\_NUCLEOTIDE\_SUGAR\_METABOLISM |  | 44 | 0.45 | 1.58 | 0.018 | 0.036 | 0.716 | 3575 | tags=30%, list=9%, signal=32% |
| 30 | KEGG\_PROPANOATE\_METABOLISM |  | 33 | 0.48 | 1.58 | 0.012 | 0.035 | 0.722 | 7982 | tags=52%, list=20%, signal=65% |
| 31 | KEGG\_SELENOAMINO\_ACID\_METABOLISM |  | 26 | 0.49 | 1.53 | 0.028 | 0.049 | 0.819 | 3980 | tags=27%, list=10%, signal=30% |
| 32 | KEGG\_GLYOXYLATE\_AND\_DICARBOXYLATE\_METABOLISM |  | 16 | 0.55 | 1.53 | 0.049 | 0.049 | 0.833 | 5062 | tags=50%, list=13%, signal=57% |
| 33 | KEGG\_GLYCOLYSIS\_GLUCONEOGENESIS |  | 62 | 0.40 | 1.51 | 0.012 | 0.053 | 0.861 | 7982 | tags=40%, list=20%, signal=51% |
| 34 | KEGG\_HISTIDINE\_METABOLISM |  | 29 | 0.46 | 1.51 | 0.027 | 0.053 | 0.866 | 6855 | tags=45%, list=17%, signal=54% |
| 35 | KEGG\_METABOLISM\_OF\_XENOBIOTICS\_BY\_CYTOCHROME\_P450 |  | 69 | 0.38 | 1.49 | 0.022 | 0.060 | 0.912 | 6655 | tags=26%, list=17%, signal=31% |
| 36 | KEGG\_GLYCOSAMINOGLYCAN\_DEGRADATION |  | 21 | 0.48 | 1.44 | 0.085 | 0.084 | 0.971 | 3182 | tags=33%, list=8%, signal=36% |
| 37 | KEGG\_FRUCTOSE\_AND\_MANNOSE\_METABOLISM |  | 34 | 0.42 | 1.40 | 0.062 | 0.103 | 0.989 | 2747 | tags=24%, list=7%, signal=25% |
| 38 | KEGG\_PRIMARY\_BILE\_ACID\_BIOSYNTHESIS |  | 16 | 0.50 | 1.36 | 0.126 | 0.127 | 0.997 | 9764 | tags=63%, list=25%, signal=83% |
| 39 | KEGG\_OTHER\_GLYCAN\_DEGRADATION |  | 16 | 0.48 | 1.36 | 0.122 | 0.124 | 0.997 | 4190 | tags=38%, list=11%, signal=42% |
| 40 | KEGG\_LYSOSOME |  | 121 | 0.32 | 1.36 | 0.037 | 0.125 | 0.999 | 4380 | tags=28%, list=11%, signal=32% |
| 41 | KEGG\_ARACHIDONIC\_ACID\_METABOLISM |  | 58 | 0.35 | 1.34 | 0.069 | 0.134 | 0.999 | 4965 | tags=28%, list=13%, signal=32% |
| 42 | KEGG\_ALANINE\_ASPARTATE\_AND\_GLUTAMATE\_METABOLISM |  | 32 | 0.40 | 1.33 | 0.082 | 0.143 | 1.000 | 3391 | tags=25%, list=9%, signal=27% |
| 43 | KEGG\_N\_GLYCAN\_BIOSYNTHESIS |  | 46 | 0.37 | 1.33 | 0.085 | 0.143 | 1.000 | 3007 | tags=30%, list=8%, signal=33% |
| 44 | KEGG\_BIOSYNTHESIS\_OF\_UNSATURATED\_FATTY\_ACIDS |  | 22 | 0.43 | 1.30 | 0.152 | 0.163 | 1.000 | 3394 | tags=27%, list=9%, signal=30% |
| 45 | KEGG\_DRUG\_METABOLISM\_CYTOCHROME\_P450 |  | 71 | 0.33 | 1.30 | 0.076 | 0.167 | 1.000 | 5147 | tags=20%, list=13%, signal=23% |
| 46 | KEGG\_GALACTOSE\_METABOLISM |  | 26 | 0.41 | 1.28 | 0.157 | 0.177 | 1.000 | 5508 | tags=31%, list=14%, signal=36% |
| 47 | KEGG\_DRUG\_METABOLISM\_OTHER\_ENZYMES |  | 51 | 0.35 | 1.28 | 0.120 | 0.176 | 1.000 | 6151 | tags=22%, list=16%, signal=26% |
| 48 | KEGG\_PROTEIN\_EXPORT |  | 24 | 0.41 | 1.27 | 0.171 | 0.182 | 1.000 | 4707 | tags=33%, list=12%, signal=38% |
| 49 | KEGG\_BETA\_ALANINE\_METABOLISM |  | 22 | 0.42 | 1.27 | 0.164 | 0.180 | 1.000 | 6492 | tags=32%, list=17%, signal=38% |
| 50 | KEGG\_GLYCINE\_SERINE\_AND\_THREONINE\_METABOLISM |  | 31 | 0.38 | 1.25 | 0.146 | 0.193 | 1.000 | 7061 | tags=39%, list=18%, signal=47% |
| 51 | KEGG\_DNA\_REPLICATION |  | 36 | 0.37 | 1.22 | 0.168 | 0.226 | 1.000 | 3360 | tags=22%, list=9%, signal=24% |
| 52 | KEGG\_PHENYLALANINE\_METABOLISM |  | 18 | 0.43 | 1.22 | 0.233 | 0.228 | 1.000 | 4966 | tags=33%, list=13%, signal=38% |
| 53 | KEGG\_MISMATCH\_REPAIR |  | 23 | 0.40 | 1.22 | 0.201 | 0.224 | 1.000 | 3360 | tags=26%, list=9%, signal=29% |
| 54 | KEGG\_RNA\_DEGRADATION |  | 59 | 0.32 | 1.21 | 0.174 | 0.236 | 1.000 | 3310 | tags=24%, list=8%, signal=26% |
| 55 | KEGG\_STEROID\_BIOSYNTHESIS |  | 17 | 0.44 | 1.20 | 0.236 | 0.236 | 1.000 | 5770 | tags=47%, list=15%, signal=55% |
| 56 | KEGG\_PPAR\_SIGNALING\_PATHWAY |  | 69 | 0.31 | 1.19 | 0.159 | 0.248 | 1.000 | 5168 | tags=25%, list=13%, signal=28% |
| 57 | KEGG\_HOMOLOGOUS\_RECOMBINATION |  | 28 | 0.36 | 1.16 | 0.235 | 0.292 | 1.000 | 4629 | tags=32%, list=12%, signal=36% |
| 58 | KEGG\_GLYCOSAMINOGLYCAN\_BIOSYNTHESIS\_KERATAN\_SULFATE |  | 15 | 0.42 | 1.15 | 0.266 | 0.304 | 1.000 | 2027 | tags=27%, list=5%, signal=28% |
| 59 | KEGG\_NICOTINATE\_AND\_NICOTINAMIDE\_METABOLISM |  | 24 | 0.37 | 1.14 | 0.257 | 0.311 | 1.000 | 2861 | tags=25%, list=7%, signal=27% |
| 60 | KEGG\_PANTOTHENATE\_AND\_COA\_BIOSYNTHESIS |  | 16 | 0.40 | 1.10 | 0.338 | 0.375 | 1.000 | 2220 | tags=31%, list=6%, signal=33% |
| 61 | KEGG\_GLYCEROLIPID\_METABOLISM |  | 49 | 0.30 | 1.08 | 0.315 | 0.405 | 1.000 | 6726 | tags=33%, list=17%, signal=39% |
| 62 | KEGG\_PENTOSE\_AND\_GLUCURONATE\_INTERCONVERSIONS |  | 28 | 0.34 | 1.08 | 0.362 | 0.399 | 1.000 | 2492 | tags=11%, list=6%, signal=11% |
| 63 | KEGG\_GLYCEROPHOSPHOLIPID\_METABOLISM |  | 77 | 0.27 | 1.08 | 0.309 | 0.395 | 1.000 | 5503 | tags=26%, list=14%, signal=30% |
| 64 | KEGG\_ONE\_CARBON\_POOL\_BY\_FOLATE |  | 17 | 0.39 | 1.07 | 0.391 | 0.397 | 1.000 | 5423 | tags=35%, list=14%, signal=41% |
| 65 | KEGG\_RIBOFLAVIN\_METABOLISM |  | 16 | 0.38 | 1.07 | 0.379 | 0.394 | 1.000 | 1737 | tags=19%, list=4%, signal=20% |
| 66 | KEGG\_BLADDER\_CANCER |  | 42 | 0.28 | 0.97 | 0.500 | 0.589 | 1.000 | 4642 | tags=24%, list=12%, signal=27% |
| 67 | KEGG\_GLYCOSPHINGOLIPID\_BIOSYNTHESIS\_LACTO\_AND\_NEOLACTO\_SERIES |  | 26 | 0.29 | 0.91 | 0.587 | 0.711 | 1.000 | 2027 | tags=15%, list=5%, signal=16% |
| 68 | KEGG\_TRYPTOPHAN\_METABOLISM |  | 40 | 0.26 | 0.91 | 0.592 | 0.716 | 1.000 | 8946 | tags=35%, list=23%, signal=45% |
| 69 | KEGG\_BASAL\_TRANSCRIPTION\_FACTORS |  | 35 | 0.27 | 0.90 | 0.596 | 0.715 | 1.000 | 7619 | tags=37%, list=19%, signal=46% |
| 70 | KEGG\_GLYCOSPHINGOLIPID\_BIOSYNTHESIS\_GANGLIO\_SERIES |  | 15 | 0.32 | 0.89 | 0.606 | 0.744 | 1.000 | 4612 | tags=33%, list=12%, signal=38% |
| 71 | KEGG\_AMYOTROPHIC\_LATERAL\_SCLEROSIS\_ALS |  | 53 | 0.23 | 0.85 | 0.724 | 0.805 | 1.000 | 7334 | tags=30%, list=19%, signal=37% |
| 72 | KEGG\_RETINOL\_METABOLISM |  | 64 | 0.21 | 0.80 | 0.821 | 0.883 | 1.000 | 6236 | tags=17%, list=16%, signal=20% |
| 73 | KEGG\_CYSTEINE\_AND\_METHIONINE\_METABOLISM |  | 34 | 0.24 | 0.79 | 0.828 | 0.897 | 1.000 | 5555 | tags=21%, list=14%, signal=24% |
| 74 | KEGG\_NITROGEN\_METABOLISM |  | 23 | 0.26 | 0.79 | 0.764 | 0.885 | 1.000 | 5423 | tags=26%, list=14%, signal=30% |
| 75 | KEGG\_PROXIMAL\_TUBULE\_BICARBONATE\_RECLAMATION |  | 23 | 0.22 | 0.67 | 0.921 | 0.988 | 1.000 | 7671 | tags=22%, list=20%, signal=27% |
| 76 | KEGG\_STEROID\_HORMONE\_BIOSYNTHESIS |  | 55 | 0.18 | 0.65 | 0.975 | 0.984 | 1.000 | 2573 | tags=7%, list=7%, signal=8% |
| 77 | KEGG\_ASCORBATE\_AND\_ALDARATE\_METABOLISM |  | 25 | 0.18 | 0.57 | 0.983 | 0.992 | 1.000 | 8116 | tags=20%, list=21%, signal=25% |
Table: Gene sets enriched in phenotype **positive correlation with profile **[plain text format]****
