# Supplementary material for: Simultaneous Presentation of Multiple Myeloma and Lung Cancer: Case Report and Gene Bioinformatics Analysis
Source: Front Oncol. 2022 Jun 13;12:859735. doi: 10.3389/fonc.2022.859735 (PMC9235397; doi:10.3389/fonc.2022.859735)
Supplement: Supplementary file 1 [file DataSheet_1.zip › The bioinformatic analysis of MM and lung cancer supplementary materials/Enrichment analysis/MECR/GSEA_4.1.0/LUAD TCGA/KEGG.Gsea.1639041756227/heat_map_corr_plot.html]

Heat map and correlation plot for ExpData\_collapsed\_to\_symbols  

Fig 1: heat\_map      
 Heat Map of the top 50 features for each phenotype in ExpData\_collapsed\_to\_symbols

  
  

Fig 2: Ranked Gene List Correlation Profile      
 Ranked list correlations for ExpData\_collapsed\_to\_symbols

  
  
    
