# Supplementary material for: Simultaneous Presentation of Multiple Myeloma and Lung Cancer: Case Report and Gene Bioinformatics Analysis
Source: Front Oncol. 2022 Jun 13;12:859735. doi: 10.3389/fonc.2022.859735 (PMC9235397; doi:10.3389/fonc.2022.859735)
Supplement: Supplementary file 1 [file DataSheet_1.zip › The bioinformatic analysis of MM and lung cancer supplementary materials/Enrichment analysis/MECR/GSEA_4.1.0/LUAD TCGA/KEGG.Gsea.1639041756227/index.html]

Index for xtools.gsea.Gsea KEGG.Gsea.1639041756227

### GSEA Report for Dataset ExpData

#### Enrichment in phenotype: **positive correlation with profile**

- 77 / 178 gene sets are upregulated in phenotype **ENSG00000116353\_pos**- 56 gene sets are significant at FDR < 25%- 24 gene sets are significantly enriched at nominal pvalue < 1%- 36 gene sets are significantly enriched at nominal pvalue < 5%- Snapshot of enrichment results- Detailed enrichment results in html format- Detailed enrichment results in TSV format (tab delimited text)- Guide to interpret results

#### Enrichment in phenotype: **negative correlation with profile**

- 101 / 178 gene sets are upregulated in phenotype **ENSG00000116353\_neg**- 84 gene sets are significantly enriched at FDR < 25%- 68 gene sets are significantly enriched at nominal pvalue < 1%- 77 gene sets are significantly enriched at nominal pvalue < 5%- Snapshot of enrichment results- Detailed enrichment results in html format- Detailed enrichment results in TSV format (tab delimited text)- Guide to interpret results

#### Dataset details

- The dataset has 60483 native features- After collapsing features into gene symbols, there are: 39324 genes

#### Gene set details

- Gene set size filters (min=15, max=500) resulted in filtering out 8 / 186 gene sets- The remaining 178 gene sets were used in the analysis- List of gene sets used and their sizes (restricted to features in the specified dataset)

#### Gene markers for the neighbors of ENSG00000116353\_pos

- The dataset has 39324 features (genes)- Detailed rank ordered gene list for all features in the dataset- Heat map and gene list correlation  profile for all features in the dataset

#### Global statistics and plots

- Plot of p-values *vs.* NES- Global ES histogram

#### Other

- Parameters used for this analysis

#### Comments

- Timestamp used as random seed: 1639041873938

---

Report: KEGG.Gsea.1639041756227.rpt   by user: abcd

xtools.gsea.Gsea [Thu, Dec 9, '21 5 PM 22]

Website: www.gsea-msigdb.org/gsea
Questions & Suggestions: Contact page
