# Supplementary material for: Simultaneous Presentation of Multiple Myeloma and Lung Cancer: Case Report and Gene Bioinformatics Analysis
Source: Front Oncol. 2022 Jun 13;12:859735. doi: 10.3389/fonc.2022.859735 (PMC9235397; doi:10.3389/fonc.2022.859735)
Supplement: Supplementary file 1 [file DataSheet_1.zip › The bioinformatic analysis of MM and lung cancer supplementary materials/Enrichment analysis/MECR/GSEA_4.1.0/LUAD TCGA/KEGG_pos_bubble.pdf]

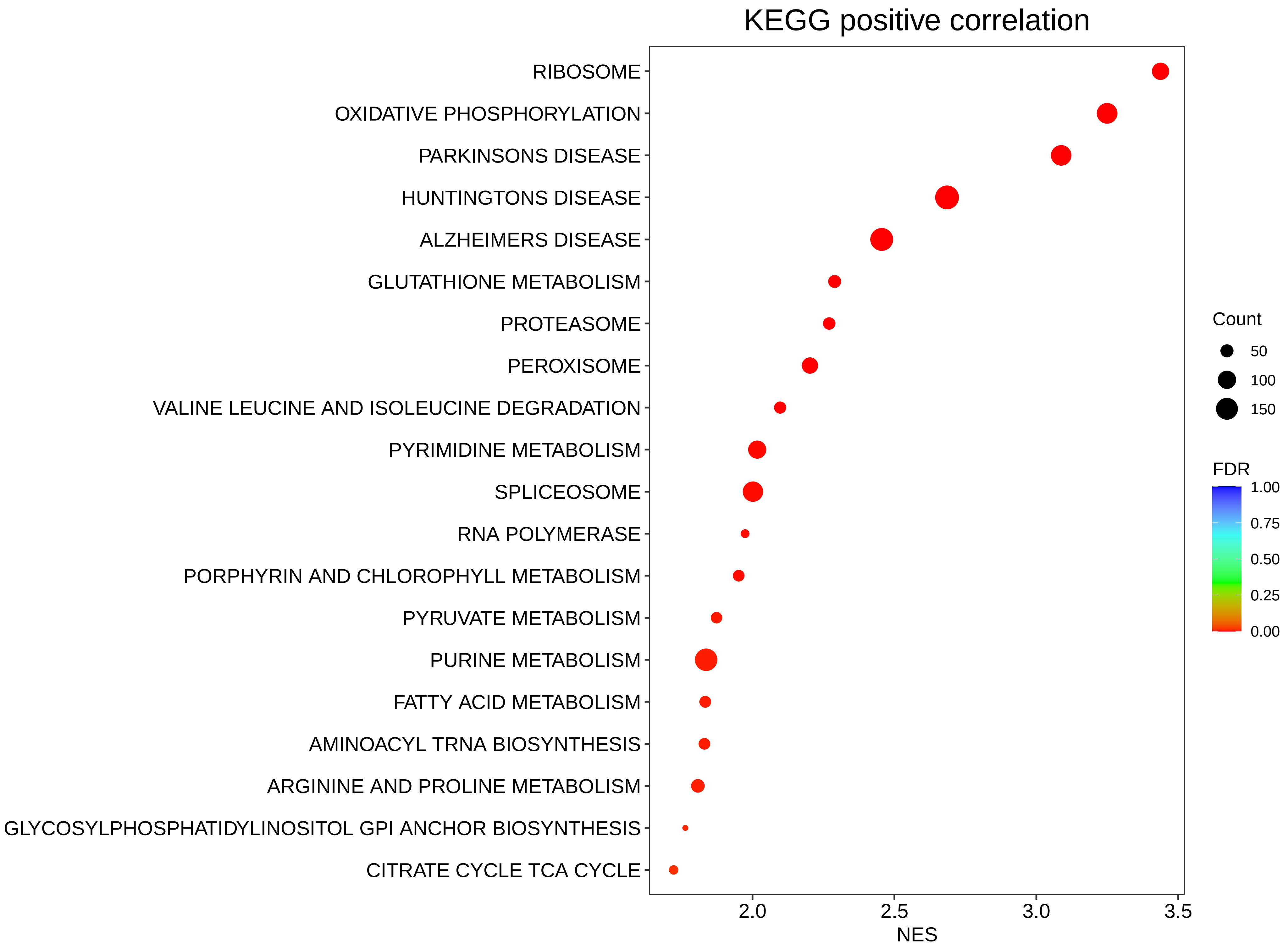

KEGG positive correlation
RIBOSOME
OXIDATIVE PHOSPHORYLATION
PARKINSONS DISEASE
HUNTINGTONS DISEASE
ALZHEIMERS DISEASE
GLUTATHIONE METABOLISM
PROTEASOME
PEROXISOME
VALINE LEUCINE AND ISOLEUCINE DEGRADATION
PYRIMIDINE METABOLISM
SPLICEOSOME
RNA POLYMERASE
PORPHYRIN AND CHLOROPHYLL METABOLISM
PYRUVATE METABOLISM
PURINE METABOLISM
FATTY ACID METABOLISM
AMINOACYL TRNA BIOSYNTHESIS
ARGININE AND PROLINE METABOLISM
GLYCOSYLPHOSPHATIDYLINOSITOL GPI ANCHOR BIOSYNTHESIS
CITRATE CYCLE TCA CYCLE
2.0
2.5
3.0
3.5
NES
Count
50
100
150
FDR
1.00
0.75
0.50
0.25
0.00
