# Supplementary material for: Simultaneous Presentation of Multiple Myeloma and Lung Cancer: Case Report and Gene Bioinformatics Analysis
Source: Front Oncol. 2022 Jun 13;12:859735. doi: 10.3389/fonc.2022.859735 (PMC9235397; doi:10.3389/fonc.2022.859735)
Supplement: Supplementary file 1 [file DataSheet_1.zip › The bioinformatic analysis of MM and lung cancer supplementary materials/Enrichment analysis/MECR/GSEA_4.1.0/LUSC TCGA/KEGG.Gsea.1639042374131/KEGG_ABC_TRANSPORTERS.html]

Details for gene set KEGG\_ABC\_TRANSPORTERS[GSEA]

|  || Dataset | ExpData\_collapsed\_to\_symbols.ENSG00000116353\_profile\_in\_ExpData.cls #ENSG00000116353 |
| Phenotype | ENSG00000116353\_profile\_in\_ExpData.cls#ENSG00000116353 |
| Upregulated in class | ENSG00000116353\_neg |
| GeneSet | KEGG\_ABC\_TRANSPORTERS |
| Enrichment Score (ES) | -0.54309195 |
| Normalized Enrichment Score (NES) | -1.9773829 |
| Nominal p-value | 0.0 |
| FDR q-value | 0.0029291245 |
| FWER p-Value | 0.014 |
Table: GSEA Results Summary

  

Fig 1: Enrichment plot: KEGG\_ABC\_TRANSPORTERS      
 Profile of the Running ES Score & Positions of GeneSet Members on the Rank Ordered List

  

| SYMBOL | TITLE | RANK IN GENE LIST | RANK METRIC SCORE | RUNNING ES | CORE ENRICHMENT || 1 | ABCD3 | ATP binding cassette subfamily D member 3 [Source:HGNC Symbol;Acc:HGNC:67] | 636 | 0.253 | 0.0294 | No |
| 2 | ABCB8 | ATP binding cassette subfamily B member 8 [Source:HGNC Symbol;Acc:HGNC:49] | 693 | 0.245 | 0.0723 | No |
| 3 | ABCD4 | ATP binding cassette subfamily D member 4 [Source:HGNC Symbol;Acc:HGNC:68] | 1665 | 0.180 | 0.0800 | No |
| 4 | ABCB9 | ATP binding cassette subfamily B member 9 [Source:HGNC Symbol;Acc:HGNC:50] | 6681 | 0.087 | -0.0321 | No |
| 5 | ABCG5 | ATP binding cassette subfamily G member 5 [Source:HGNC Symbol;Acc:HGNC:13886] | 7993 | 0.075 | -0.0519 | No |
| 6 | ABCC11 | ATP binding cassette subfamily C member 11 [Source:HGNC Symbol;Acc:HGNC:14639] | 9755 | 0.062 | -0.0856 | No |
| 7 | ABCB11 | ATP binding cassette subfamily B member 11 [Source:HGNC Symbol;Acc:HGNC:42] | 15059 | 0.029 | -0.2154 | No |
| 8 | ABCC10 | ATP binding cassette subfamily C member 10 [Source:HGNC Symbol;Acc:HGNC:52] | 15467 | 0.026 | -0.2209 | No |
| 9 | CFTR | CF transmembrane conductance regulator [Source:HGNC Symbol;Acc:HGNC:1884] | 15906 | 0.024 | -0.2278 | No |
| 10 | ABCA3 | ATP binding cassette subfamily A member 3 [Source:HGNC Symbol;Acc:HGNC:33] | 19338 | 0.004 | -0.3143 | No |
| 11 | ABCB7 | ATP binding cassette subfamily B member 7 [Source:HGNC Symbol;Acc:HGNC:48] | 19420 | 0.004 | -0.3157 | No |
| 12 | ABCC9 | ATP binding cassette subfamily C member 9 [Source:HGNC Symbol;Acc:HGNC:60] | 20732 | -0.004 | -0.3484 | No |
| 13 | ABCG8 | ATP binding cassette subfamily G member 8 [Source:HGNC Symbol;Acc:HGNC:13887] | 21179 | -0.006 | -0.3586 | No |
| 14 | TAP1 | "transporter 1, ATP binding cassette subfamily B member [Source:HGNC Symbol;Acc:HGNC:43]" | 21598 | -0.009 | -0.3677 | No |
| 15 | TAP2 | "transporter 2, ATP binding cassette subfamily B member [Source:HGNC Symbol;Acc:HGNC:44]" | 25244 | -0.033 | -0.4545 | No |
| 16 | ABCD1 | ATP binding cassette subfamily D member 1 [Source:HGNC Symbol;Acc:HGNC:61] | 25280 | -0.033 | -0.4494 | No |
| 17 | ABCA4 | ATP binding cassette subfamily A member 4 [Source:HGNC Symbol;Acc:HGNC:34] | 25992 | -0.038 | -0.4606 | No |
| 18 | ABCA7 | ATP binding cassette subfamily A member 7 [Source:HGNC Symbol;Acc:HGNC:37] | 26367 | -0.041 | -0.4628 | No |
| 19 | ABCC12 | ATP binding cassette subfamily C member 12 [Source:HGNC Symbol;Acc:HGNC:14640] | 26436 | -0.041 | -0.4571 | No |
| 20 | ABCC2 | ATP binding cassette subfamily C member 2 [Source:HGNC Symbol;Acc:HGNC:53] | 27195 | -0.047 | -0.4679 | No |
| 21 | ABCA8 | ATP binding cassette subfamily A member 8 [Source:HGNC Symbol;Acc:HGNC:38] | 30057 | -0.071 | -0.5279 | Yes |
| 22 | ABCA12 | ATP binding cassette subfamily A member 12 [Source:HGNC Symbol;Acc:HGNC:14637] | 30423 | -0.075 | -0.5237 | Yes |
| 23 | ABCA2 | ATP binding cassette subfamily A member 2 [Source:HGNC Symbol;Acc:HGNC:32] | 31170 | -0.082 | -0.5279 | Yes |
| 24 | ABCB4 | ATP binding cassette subfamily B member 4 [Source:HGNC Symbol;Acc:HGNC:45] | 31492 | -0.085 | -0.5207 | Yes |
| 25 | ABCA10 | ATP binding cassette subfamily A member 10 [Source:HGNC Symbol;Acc:HGNC:30] | 31610 | -0.086 | -0.5081 | Yes |
| 26 | ABCG1 | ATP binding cassette subfamily G member 1 [Source:HGNC Symbol;Acc:HGNC:73] | 32143 | -0.092 | -0.5049 | Yes |
| 27 | ABCB5 | ATP binding cassette subfamily B member 5 [Source:HGNC Symbol;Acc:HGNC:46] | 32822 | -0.101 | -0.5040 | Yes |
| 28 | ABCC8 | ATP binding cassette subfamily C member 8 [Source:HGNC Symbol;Acc:HGNC:59] | 33989 | -0.118 | -0.5124 | Yes |
| 29 | ABCA6 | ATP binding cassette subfamily A member 6 [Source:HGNC Symbol;Acc:HGNC:36] | 35194 | -0.139 | -0.5179 | Yes |
| 30 | ABCC4 | ATP binding cassette subfamily C member 4 [Source:HGNC Symbol;Acc:HGNC:55] | 35811 | -0.155 | -0.5057 | Yes |
| 31 | ABCD2 | ATP binding cassette subfamily D member 2 [Source:HGNC Symbol;Acc:HGNC:66] | 36224 | -0.167 | -0.4859 | Yes |
| 32 | ABCB6 | ATP binding cassette subfamily B member 6 (Langereis blood group) [Source:HGNC Symbol;Acc:HGNC:47] | 36392 | -0.173 | -0.4590 | Yes |
| 33 | ABCC6 | ATP binding cassette subfamily C member 6 [Source:HGNC Symbol;Acc:HGNC:57] | 36480 | -0.176 | -0.4295 | Yes |
| 34 | ABCB1 | ATP binding cassette subfamily B member 1 [Source:HGNC Symbol;Acc:HGNC:40] | 37017 | -0.198 | -0.4075 | Yes |
| 35 | ABCG4 | ATP binding cassette subfamily G member 4 [Source:HGNC Symbol;Acc:HGNC:13884] | 37086 | -0.202 | -0.3727 | Yes |
| 36 | ABCG2 | ATP binding cassette subfamily G member 2 (Junior blood group) [Source:HGNC Symbol;Acc:HGNC:74] | 37440 | -0.220 | -0.3421 | Yes |
| 37 | ABCC3 | ATP binding cassette subfamily C member 3 [Source:HGNC Symbol;Acc:HGNC:54] | 37458 | -0.221 | -0.3027 | Yes |
| 38 | ABCB10 | ATP binding cassette subfamily B member 10 [Source:HGNC Symbol;Acc:HGNC:41] | 37477 | -0.222 | -0.2631 | Yes |
| 39 | ABCA9 | ATP binding cassette subfamily A member 9 [Source:HGNC Symbol;Acc:HGNC:39] | 37526 | -0.226 | -0.2236 | Yes |
| 40 | ABCA5 | ATP binding cassette subfamily A member 5 [Source:HGNC Symbol;Acc:HGNC:35] | 37971 | -0.262 | -0.1877 | Yes |
| 41 | ABCA1 | ATP binding cassette subfamily A member 1 [Source:HGNC Symbol;Acc:HGNC:29] | 38082 | -0.274 | -0.1411 | Yes |
| 42 | ABCA13 | ATP binding cassette subfamily A member 13 [Source:HGNC Symbol;Acc:HGNC:14638] | 38148 | -0.285 | -0.0913 | Yes |
| 43 | ABCC5 | ATP binding cassette subfamily C member 5 [Source:HGNC Symbol;Acc:HGNC:56] | 38326 | -0.312 | -0.0395 | Yes |
| 44 | ABCC1 | ATP binding cassette subfamily C member 1 [Source:HGNC Symbol;Acc:HGNC:51] | 38477 | -0.360 | 0.0215 | Yes |
Table: GSEA details [plain text format]

  

Fig 2: KEGG\_ABC\_TRANSPORTERS      
 Blue-Pink O' Gram in the Space of the Analyzed GeneSet

  

Fig 3: KEGG\_ABC\_TRANSPORTERS: Random ES distribution      
 Gene set null distribution of ES for **KEGG\_ABC\_TRANSPORTERS**
