# Supplementary material for: Simultaneous Presentation of Multiple Myeloma and Lung Cancer: Case Report and Gene Bioinformatics Analysis
Source: Front Oncol. 2022 Jun 13;12:859735. doi: 10.3389/fonc.2022.859735 (PMC9235397; doi:10.3389/fonc.2022.859735)
Supplement: Supplementary file 1 [file DataSheet_1.zip › The bioinformatic analysis of MM and lung cancer supplementary materials/Enrichment analysis/MECR/GSEA_4.1.0/LUSC TCGA/KEGG.Gsea.1639042374131/KEGG_ALZHEIMERS_DISEASE.html]

Details for gene set KEGG\_ALZHEIMERS\_DISEASE[GSEA]

|  || Dataset | ExpData\_collapsed\_to\_symbols.ENSG00000116353\_profile\_in\_ExpData.cls #ENSG00000116353 |
| Phenotype | ENSG00000116353\_profile\_in\_ExpData.cls#ENSG00000116353 |
| Upregulated in class | ENSG00000116353\_pos |
| GeneSet | KEGG\_ALZHEIMERS\_DISEASE |
| Enrichment Score (ES) | 0.5261765 |
| Normalized Enrichment Score (NES) | 2.4289427 |
| Nominal p-value | 0.0 |
| FDR q-value | 0.0 |
| FWER p-Value | 0.0 |
Table: GSEA Results Summary

  

Fig 1: Enrichment plot: KEGG\_ALZHEIMERS\_DISEASE      
 Profile of the Running ES Score & Positions of GeneSet Members on the Rank Ordered List

  

| SYMBOL | TITLE | RANK IN GENE LIST | RANK METRIC SCORE | RUNNING ES | CORE ENRICHMENT || 1 | SDHB | succinate dehydrogenase complex iron sulfur subunit B [Source:HGNC Symbol;Acc:HGNC:10681] | 66 | 0.423 | 0.0190 | Yes |
| 2 | NDUFS5 | NADH:ubiquinone oxidoreductase subunit S5 [Source:HGNC Symbol;Acc:HGNC:7712] | 85 | 0.408 | 0.0385 | Yes |
| 3 | NDUFA3 | NADH:ubiquinone oxidoreductase subunit A3 [Source:HGNC Symbol;Acc:HGNC:7686] | 141 | 0.368 | 0.0550 | Yes |
| 4 | UQCRH | ubiquinol-cytochrome c reductase hinge protein [Source:HGNC Symbol;Acc:HGNC:12590] | 167 | 0.353 | 0.0716 | Yes |
| 5 | NDUFS6 | NADH:ubiquinone oxidoreductase subunit S6 [Source:HGNC Symbol;Acc:HGNC:7713] | 194 | 0.343 | 0.0877 | Yes |
| 6 | ATP5PB | ATP synthase peripheral stalk-membrane subunit b [Source:HGNC Symbol;Acc:HGNC:840] | 213 | 0.335 | 0.1036 | Yes |
| 7 | CASP9 | caspase 9 [Source:HGNC Symbol;Acc:HGNC:1511] | 300 | 0.306 | 0.1164 | Yes |
| 8 | UQCRHL | ubiquinol-cytochrome c reductase hinge protein like [Source:HGNC Symbol;Acc:HGNC:51714] | 377 | 0.290 | 0.1286 | Yes |
| 9 | PSENEN | "presenilin enhancer, gamma-secretase subunit [Source:HGNC Symbol;Acc:HGNC:30100]" | 416 | 0.283 | 0.1414 | Yes |
| 10 | SDHA | succinate dehydrogenase complex flavoprotein subunit A [Source:HGNC Symbol;Acc:HGNC:10680] | 452 | 0.277 | 0.1541 | Yes |
| 11 | MAPK3 | mitogen-activated protein kinase 3 [Source:HGNC Symbol;Acc:HGNC:6877] | 473 | 0.274 | 0.1669 | Yes |
| 12 | NDUFS8 | NADH:ubiquinone oxidoreductase core subunit S8 [Source:HGNC Symbol;Acc:HGNC:7715] | 512 | 0.268 | 0.1791 | Yes |
| 13 | ATP5PO | ATP synthase peripheral stalk subunit OSCP [Source:HGNC Symbol;Acc:HGNC:850] | 517 | 0.268 | 0.1921 | Yes |
| 14 | NDUFV1 | NADH:ubiquinone oxidoreductase core subunit V1 [Source:HGNC Symbol;Acc:HGNC:7716] | 525 | 0.266 | 0.2049 | Yes |
| 15 | CYC1 | cytochrome c1 [Source:HGNC Symbol;Acc:HGNC:2579] | 644 | 0.252 | 0.2142 | Yes |
| 16 | ATP5F1E | ATP synthase F1 subunit epsilon [Source:HGNC Symbol;Acc:HGNC:838] | 731 | 0.241 | 0.2237 | Yes |
| 17 | CALM3 | calmodulin 3 [Source:HGNC Symbol;Acc:HGNC:1449] | 850 | 0.227 | 0.2318 | Yes |
| 18 | BAD | BCL2 associated agonist of cell death [Source:HGNC Symbol;Acc:HGNC:936] | 945 | 0.219 | 0.2401 | Yes |
| 19 | UQCR10 | "ubiquinol-cytochrome c reductase, complex III subunit X [Source:HGNC Symbol;Acc:HGNC:30863]" | 976 | 0.217 | 0.2499 | Yes |
| 20 | NDUFA2 | NADH:ubiquinone oxidoreductase subunit A2 [Source:HGNC Symbol;Acc:HGNC:7685] | 1078 | 0.210 | 0.2576 | Yes |
| 21 | ATP5F1C | ATP synthase F1 subunit gamma [Source:HGNC Symbol;Acc:HGNC:833] | 1084 | 0.210 | 0.2677 | Yes |
| 22 | UQCRQ | ubiquinol-cytochrome c reductase complex III subunit VII [Source:HGNC Symbol;Acc:HGNC:29594] | 1244 | 0.200 | 0.2734 | Yes |
| 23 | BACE2 | beta-secretase 2 [Source:HGNC Symbol;Acc:HGNC:934] | 1291 | 0.198 | 0.2819 | Yes |
| 24 | NDUFC1 | NADH:ubiquinone oxidoreductase subunit C1 [Source:HGNC Symbol;Acc:HGNC:7705] | 1333 | 0.195 | 0.2904 | Yes |
| 25 | NDUFA10 | NADH:ubiquinone oxidoreductase subunit A10 [Source:HGNC Symbol;Acc:HGNC:7684] | 1484 | 0.187 | 0.2957 | Yes |
| 26 | COX6B2 | cytochrome c oxidase subunit 6B2 [Source:HGNC Symbol;Acc:HGNC:24380] | 1508 | 0.186 | 0.3042 | Yes |
| 27 | NDUFB8 | NADH:ubiquinone oxidoreductase subunit B8 [Source:HGNC Symbol;Acc:HGNC:7703] | 1568 | 0.183 | 0.3117 | Yes |
| 28 | CAPN2 | calpain 2 [Source:HGNC Symbol;Acc:HGNC:1479] | 1575 | 0.183 | 0.3205 | Yes |
| 29 | ATP5MC2 | ATP synthase membrane subunit c locus 2 [Source:HGNC Symbol;Acc:HGNC:842] | 1577 | 0.183 | 0.3294 | Yes |
| 30 | HSD17B10 | hydroxysteroid 17-beta dehydrogenase 10 [Source:HGNC Symbol;Acc:HGNC:4800] | 1578 | 0.183 | 0.3383 | Yes |
| 31 | UQCRB | ubiquinol-cytochrome c reductase binding protein [Source:HGNC Symbol;Acc:HGNC:12582] | 1639 | 0.181 | 0.3456 | Yes |
| 32 | COX8A | cytochrome c oxidase subunit 8A [Source:HGNC Symbol;Acc:HGNC:2294] | 1644 | 0.181 | 0.3543 | Yes |
| 33 | NDUFB3 | NADH:ubiquinone oxidoreductase subunit B3 [Source:HGNC Symbol;Acc:HGNC:7698] | 1692 | 0.178 | 0.3619 | Yes |
| 34 | CAPN1 | calpain 1 [Source:HGNC Symbol;Acc:HGNC:1476] | 1693 | 0.178 | 0.3705 | Yes |
| 35 | UQCRC1 | ubiquinol-cytochrome c reductase core protein 1 [Source:HGNC Symbol;Acc:HGNC:12585] | 1764 | 0.175 | 0.3773 | Yes |
| 36 | NDUFB4 | NADH:ubiquinone oxidoreductase subunit B4 [Source:HGNC Symbol;Acc:HGNC:7699] | 1870 | 0.171 | 0.3830 | Yes |
| 37 | NDUFB9 | NADH:ubiquinone oxidoreductase subunit B9 [Source:HGNC Symbol;Acc:HGNC:7704] | 1879 | 0.171 | 0.3911 | Yes |
| 38 | NDUFB1 | NADH:ubiquinone oxidoreductase subunit B1 [Source:HGNC Symbol;Acc:HGNC:7695] | 1941 | 0.168 | 0.3977 | Yes |
| 39 | PLCB3 | phospholipase C beta 3 [Source:HGNC Symbol;Acc:HGNC:9056] | 1971 | 0.167 | 0.4051 | Yes |
| 40 | NDUFAB1 | NADH:ubiquinone oxidoreductase subunit AB1 [Source:HGNC Symbol;Acc:HGNC:7694] | 2058 | 0.164 | 0.4109 | Yes |
| 41 | APP | amyloid beta precursor protein [Source:HGNC Symbol;Acc:HGNC:620] | 2385 | 0.153 | 0.4100 | Yes |
| 42 | PPP3CA | protein phosphatase 3 catalytic subunit alpha [Source:HGNC Symbol;Acc:HGNC:9314] | 2508 | 0.149 | 0.4142 | Yes |
| 43 | NDUFB10 | NADH:ubiquinone oxidoreductase subunit B10 [Source:HGNC Symbol;Acc:HGNC:7696] | 2658 | 0.145 | 0.4175 | Yes |
| 44 | MT-CO1 | mitochondrially encoded cytochrome c oxidase I [Source:HGNC Symbol;Acc:HGNC:7419] | 2684 | 0.144 | 0.4239 | Yes |
| 45 | NDUFA9 | NADH:ubiquinone oxidoreductase subunit A9 [Source:HGNC Symbol;Acc:HGNC:7693] | 2762 | 0.142 | 0.4289 | Yes |
| 46 | NDUFS3 | NADH:ubiquinone oxidoreductase core subunit S3 [Source:HGNC Symbol;Acc:HGNC:7710] | 2883 | 0.140 | 0.4327 | Yes |
| 47 | ATP5PF | ATP synthase peripheral stalk subunit F6 [Source:HGNC Symbol;Acc:HGNC:847] | 2949 | 0.138 | 0.4377 | Yes |
| 48 | COX4I1 | cytochrome c oxidase subunit 4I1 [Source:HGNC Symbol;Acc:HGNC:2265] | 3073 | 0.135 | 0.4412 | Yes |
| 49 | NDUFS7 | NADH:ubiquinone oxidoreductase core subunit S7 [Source:HGNC Symbol;Acc:HGNC:7714] | 3210 | 0.132 | 0.4442 | Yes |
| 50 | CACNA1D | calcium voltage-gated channel subunit alpha1 D [Source:HGNC Symbol;Acc:HGNC:1391] | 3402 | 0.128 | 0.4456 | Yes |
| 51 | COX7C | cytochrome c oxidase subunit 7C [Source:HGNC Symbol;Acc:HGNC:2292] | 3427 | 0.128 | 0.4512 | Yes |
| 52 | COX7A2 | cytochrome c oxidase subunit 7A2 [Source:HGNC Symbol;Acc:HGNC:2288] | 3468 | 0.127 | 0.4564 | Yes |
| 53 | NDUFB2 | NADH:ubiquinone oxidoreductase subunit B2 [Source:HGNC Symbol;Acc:HGNC:7697] | 3507 | 0.126 | 0.4616 | Yes |
| 54 | NDUFV2 | NADH:ubiquinone oxidoreductase core subunit V2 [Source:HGNC Symbol;Acc:HGNC:7717] | 3550 | 0.125 | 0.4666 | Yes |
| 55 | COX6B1 | cytochrome c oxidase subunit 6B1 [Source:HGNC Symbol;Acc:HGNC:2280] | 3572 | 0.125 | 0.4722 | Yes |
| 56 | NDUFB7 | NADH:ubiquinone oxidoreductase subunit B7 [Source:HGNC Symbol;Acc:HGNC:7702] | 3667 | 0.123 | 0.4758 | Yes |
| 57 | NDUFC2 | NADH:ubiquinone oxidoreductase subunit C2 [Source:HGNC Symbol;Acc:HGNC:7706] | 3963 | 0.118 | 0.4741 | Yes |
| 58 | NDUFS4 | NADH:ubiquinone oxidoreductase subunit S4 [Source:HGNC Symbol;Acc:HGNC:7711] | 4123 | 0.116 | 0.4756 | Yes |
| 59 | COX4I2 | cytochrome c oxidase subunit 4I2 [Source:HGNC Symbol;Acc:HGNC:16232] | 4182 | 0.115 | 0.4798 | Yes |
| 60 | ATP5PD | ATP synthase peripheral stalk subunit d [Source:HGNC Symbol;Acc:HGNC:845] | 4285 | 0.113 | 0.4827 | Yes |
| 61 | SDHC | succinate dehydrogenase complex subunit C [Source:HGNC Symbol;Acc:HGNC:10682] | 4323 | 0.112 | 0.4872 | Yes |
| 62 | COX6C | cytochrome c oxidase subunit 6C [Source:HGNC Symbol;Acc:HGNC:2285] | 4326 | 0.112 | 0.4927 | Yes |
| 63 | MT-CO3 | mitochondrially encoded cytochrome c oxidase III [Source:HGNC Symbol;Acc:HGNC:7422] | 4561 | 0.109 | 0.4920 | Yes |
| 64 | PSEN2 | presenilin 2 [Source:HGNC Symbol;Acc:HGNC:9509] | 4651 | 0.108 | 0.4950 | Yes |
| 65 | COX7A2L | cytochrome c oxidase subunit 7A2 like [Source:HGNC Symbol;Acc:HGNC:2289] | 4754 | 0.106 | 0.4976 | Yes |
| 66 | COX7B | cytochrome c oxidase subunit 7B [Source:HGNC Symbol;Acc:HGNC:2291] | 4828 | 0.105 | 0.5009 | Yes |
| 67 | COX6CP3 | cytochrome c oxidase subunit 6C pseudogene 3 [Source:HGNC Symbol;Acc:HGNC:31721] | 4943 | 0.104 | 0.5030 | Yes |
| 68 | COX6A1 | cytochrome c oxidase subunit 6A1 [Source:HGNC Symbol;Acc:HGNC:2277] | 5113 | 0.101 | 0.5036 | Yes |
| 69 | NDUFA4 | NDUFA4 mitochondrial complex associated [Source:HGNC Symbol;Acc:HGNC:7687] | 5480 | 0.097 | 0.4990 | Yes |
| 70 | UQCRC2 | ubiquinol-cytochrome c reductase core protein 2 [Source:HGNC Symbol;Acc:HGNC:12586] | 5923 | 0.095 | 0.4924 | Yes |
| 71 | ATP5MC1 | ATP synthase membrane subunit c locus 1 [Source:HGNC Symbol;Acc:HGNC:841] | 5965 | 0.094 | 0.4959 | Yes |
| 72 | NDUFV3 | NADH:ubiquinone oxidoreductase subunit V3 [Source:HGNC Symbol;Acc:HGNC:7719] | 6013 | 0.094 | 0.4993 | Yes |
| 73 | GRIN2C | glutamate ionotropic receptor NMDA type subunit 2C [Source:HGNC Symbol;Acc:HGNC:4587] | 6018 | 0.094 | 0.5038 | Yes |
| 74 | NDUFA1 | NADH:ubiquinone oxidoreductase subunit A1 [Source:HGNC Symbol;Acc:HGNC:7683] | 6036 | 0.094 | 0.5079 | Yes |
| 75 | CALM2 | calmodulin 2 [Source:HGNC Symbol;Acc:HGNC:1445] | 6292 | 0.091 | 0.5059 | Yes |
| 76 | COX6A2 | cytochrome c oxidase subunit 6A2 [Source:HGNC Symbol;Acc:HGNC:2279] | 6345 | 0.090 | 0.5089 | Yes |
| 77 | BID | BH3 interacting domain death agonist [Source:HGNC Symbol;Acc:HGNC:1050] | 6572 | 0.088 | 0.5075 | Yes |
| 78 | CALML5 | calmodulin like 5 [Source:HGNC Symbol;Acc:HGNC:18180] | 6619 | 0.087 | 0.5106 | Yes |
| 79 | NDUFA6 | NADH:ubiquinone oxidoreductase subunit A6 [Source:HGNC Symbol;Acc:HGNC:7690] | 6634 | 0.087 | 0.5145 | Yes |
| 80 | GRIN2D | glutamate ionotropic receptor NMDA type subunit 2D [Source:HGNC Symbol;Acc:HGNC:4588] | 6650 | 0.087 | 0.5183 | Yes |
| 81 | COX7B2 | cytochrome c oxidase subunit 7B2 [Source:HGNC Symbol;Acc:HGNC:24381] | 6753 | 0.086 | 0.5199 | Yes |
| 82 | CDK5 | cyclin dependent kinase 5 [Source:HGNC Symbol;Acc:HGNC:1774] | 7034 | 0.083 | 0.5169 | Yes |
| 83 | MT-CO2 | mitochondrially encoded cytochrome c oxidase II [Source:HGNC Symbol;Acc:HGNC:7421] | 7144 | 0.082 | 0.5181 | Yes |
| 84 | TNF | tumor necrosis factor [Source:HGNC Symbol;Acc:HGNC:11892] | 7309 | 0.081 | 0.5179 | Yes |
| 85 | MME | membrane metalloendopeptidase [Source:HGNC Symbol;Acc:HGNC:7154] | 7533 | 0.079 | 0.5160 | Yes |
| 86 | NDUFA5 | NADH:ubiquinone oxidoreductase subunit A5 [Source:HGNC Symbol;Acc:HGNC:7688] | 7600 | 0.078 | 0.5182 | Yes |
| 87 | ATP5F1D | ATP synthase F1 subunit delta [Source:HGNC Symbol;Acc:HGNC:837] | 7651 | 0.078 | 0.5207 | Yes |
| 88 | CASP7 | caspase 7 [Source:HGNC Symbol;Acc:HGNC:1508] | 7698 | 0.078 | 0.5233 | Yes |
| 89 | ATP5F1A | ATP synthase F1 subunit alpha [Source:HGNC Symbol;Acc:HGNC:823] | 7734 | 0.077 | 0.5262 | Yes |
| 90 | PPP3CB | protein phosphatase 3 catalytic subunit beta [Source:HGNC Symbol;Acc:HGNC:9315] | 8034 | 0.075 | 0.5222 | No |
| 91 | COX5B | cytochrome c oxidase subunit 5B [Source:HGNC Symbol;Acc:HGNC:2269] | 8271 | 0.072 | 0.5197 | No |
| 92 | APH1A | "aph-1 homolog A, gamma-secretase subunit [Source:HGNC Symbol;Acc:HGNC:29509]" | 8531 | 0.070 | 0.5165 | No |
| 93 | MT-CYB | mitochondrially encoded cytochrome b [Source:HGNC Symbol;Acc:HGNC:7427] | 9843 | 0.061 | 0.4860 | No |
| 94 | UQCR11 | "ubiquinol-cytochrome c reductase, complex III subunit XI [Source:HGNC Symbol;Acc:HGNC:30862]" | 9868 | 0.061 | 0.4884 | No |
| 95 | UQCRFS1 | "ubiquinol-cytochrome c reductase, Rieske iron-sulfur polypeptide 1 [Source:HGNC Symbol;Acc:HGNC:12587]" | 10480 | 0.057 | 0.4756 | No |
| 96 | FADD | Fas associated via death domain [Source:HGNC Symbol;Acc:HGNC:3573] | 10793 | 0.055 | 0.4703 | No |
| 97 | TNFRSF1A | TNF receptor superfamily member 1A [Source:HGNC Symbol;Acc:HGNC:11916] | 10931 | 0.054 | 0.4694 | No |
| 98 | COX8C | cytochrome c oxidase subunit 8C [Source:HGNC Symbol;Acc:HGNC:24382] | 11354 | 0.051 | 0.4612 | No |
| 99 | MT-ATP6 | mitochondrially encoded ATP synthase membrane subunit 6 [Source:HGNC Symbol;Acc:HGNC:7414] | 11361 | 0.051 | 0.4635 | No |
| 100 | NDUFA7 | NADH:ubiquinone oxidoreductase subunit A7 [Source:HGNC Symbol;Acc:HGNC:7691] | 11506 | 0.050 | 0.4623 | No |
| 101 | NAE1 | NEDD8 activating enzyme E1 subunit 1 [Source:HGNC Symbol;Acc:HGNC:621] | 11985 | 0.047 | 0.4524 | No |
| 102 | CHP1 | calcineurin like EF-hand protein 1 [Source:HGNC Symbol;Acc:HGNC:17433] | 12465 | 0.044 | 0.4423 | No |
| 103 | NDUFS2 | NADH:ubiquinone oxidoreductase core subunit S2 [Source:HGNC Symbol;Acc:HGNC:7708] | 13107 | 0.040 | 0.4279 | No |
| 104 | GRIN1 | glutamate ionotropic receptor NMDA type subunit 1 [Source:HGNC Symbol;Acc:HGNC:4584] | 13266 | 0.039 | 0.4258 | No |
| 105 | ATP5F1B | ATP synthase F1 subunit beta [Source:HGNC Symbol;Acc:HGNC:830] | 14082 | 0.035 | 0.4067 | No |
| 106 | GSK3B | glycogen synthase kinase 3 beta [Source:HGNC Symbol;Acc:HGNC:4617] | 15772 | 0.025 | 0.3648 | No |
| 107 | NDUFB6 | NADH:ubiquinone oxidoreductase subunit B6 [Source:HGNC Symbol;Acc:HGNC:7701] | 15775 | 0.025 | 0.3659 | No |
| 108 | PPP3R2 | "protein phosphatase 3 regulatory subunit B, beta [Source:HGNC Symbol;Acc:HGNC:9318]" | 15798 | 0.024 | 0.3666 | No |
| 109 | MT-ATP8 | mitochondrially encoded ATP synthase membrane subunit 8 [Source:HGNC Symbol;Acc:HGNC:7415] | 16163 | 0.022 | 0.3583 | No |
| 110 | GAPDH | glyceraldehyde-3-phosphate dehydrogenase [Source:HGNC Symbol;Acc:HGNC:4141] | 16461 | 0.021 | 0.3518 | No |
| 111 | COX7A1 | cytochrome c oxidase subunit 7A1 [Source:HGNC Symbol;Acc:HGNC:2287] | 16527 | 0.020 | 0.3511 | No |
| 112 | NDUFA4L2 | NDUFA4 mitochondrial complex associated like 2 [Source:HGNC Symbol;Acc:HGNC:29836] | 16755 | 0.019 | 0.3462 | No |
| 113 | ATP5MC3 | ATP synthase membrane subunit c locus 3 [Source:HGNC Symbol;Acc:HGNC:843] | 16867 | 0.018 | 0.3443 | No |
| 114 | NDUFS1 | NADH:ubiquinone oxidoreductase core subunit S1 [Source:HGNC Symbol;Acc:HGNC:7707] | 17437 | 0.015 | 0.3305 | No |
| 115 | ATP2A1 | ATPase sarcoplasmic/endoplasmic reticulum Ca2+ transporting 1 [Source:HGNC Symbol;Acc:HGNC:811] | 18670 | 0.008 | 0.2995 | No |
| 116 | NCSTN | nicastrin [Source:HGNC Symbol;Acc:HGNC:17091] | 19179 | 0.005 | 0.2868 | No |
| 117 | ATP5MC1P5 | ATP synthase membrane subunit c locus 1 pseudogene 5 [Source:HGNC Symbol;Acc:HGNC:39508] | 19858 | 0.002 | 0.2695 | No |
| 118 | CALML6 | calmodulin like 6 [Source:HGNC Symbol;Acc:HGNC:24193] | 20499 | -0.002 | 0.2533 | No |
| 119 | APAF1 | apoptotic peptidase activating factor 1 [Source:HGNC Symbol;Acc:HGNC:576] | 20948 | -0.005 | 0.2421 | No |
| 120 | CACNA1C | calcium voltage-gated channel subunit alpha1 C [Source:HGNC Symbol;Acc:HGNC:1390] | 21681 | -0.009 | 0.2239 | No |
| 121 | CALM1 | calmodulin 1 [Source:HGNC Symbol;Acc:HGNC:1442] | 21884 | -0.010 | 0.2192 | No |
| 122 | GRIN2A | glutamate ionotropic receptor NMDA type subunit 2A [Source:HGNC Symbol;Acc:HGNC:4585] | 21887 | -0.010 | 0.2197 | No |
| 123 | COX5A | cytochrome c oxidase subunit 5A [Source:HGNC Symbol;Acc:HGNC:2267] | 22458 | -0.014 | 0.2058 | No |
| 124 | PPP3R1 | "protein phosphatase 3 regulatory subunit B, alpha [Source:HGNC Symbol;Acc:HGNC:9317]" | 22525 | -0.015 | 0.2048 | No |
| 125 | LPL | lipoprotein lipase [Source:HGNC Symbol;Acc:HGNC:6677] | 22732 | -0.016 | 0.2003 | No |
| 126 | SDHD | succinate dehydrogenase complex subunit D [Source:HGNC Symbol;Acc:HGNC:10683] | 22998 | -0.017 | 0.1944 | No |
| 127 | PSEN1 | presenilin 1 [Source:HGNC Symbol;Acc:HGNC:9508] | 23228 | -0.019 | 0.1895 | No |
| 128 | FAS | Fas cell surface death receptor [Source:HGNC Symbol;Acc:HGNC:11920] | 23910 | -0.023 | 0.1732 | No |
| 129 | CYCS | "cytochrome c, somatic [Source:HGNC Symbol;Acc:HGNC:19986]" | 24187 | -0.025 | 0.1674 | No |
| 130 | ATP2A3 | ATPase sarcoplasmic/endoplasmic reticulum Ca2+ transporting 3 [Source:HGNC Symbol;Acc:HGNC:813] | 24272 | -0.026 | 0.1665 | No |
| 131 | APOE | apolipoprotein E [Source:HGNC Symbol;Acc:HGNC:613] | 24989 | -0.031 | 0.1497 | No |
| 132 | CACNA1S | calcium voltage-gated channel subunit alpha1 S [Source:HGNC Symbol;Acc:HGNC:1397] | 25589 | -0.035 | 0.1362 | No |
| 133 | UQCR10P1 | UQCR10 pseudogene 1 [Source:HGNC Symbol;Acc:HGNC:54960] | 25693 | -0.036 | 0.1353 | No |
| 134 | SNCA | synuclein alpha [Source:HGNC Symbol;Acc:HGNC:11138] | 25948 | -0.038 | 0.1307 | No |
| 135 | PLCB1 | phospholipase C beta 1 [Source:HGNC Symbol;Acc:HGNC:15917] | 27781 | -0.052 | 0.0864 | No |
| 136 | CASP8 | caspase 8 [Source:HGNC Symbol;Acc:HGNC:1509] | 27944 | -0.053 | 0.0848 | No |
| 137 | MAPT | microtubule associated protein tau [Source:HGNC Symbol;Acc:HGNC:6893] | 28320 | -0.056 | 0.0780 | No |
| 138 | ITPR3 | "inositol 1,4,5-trisphosphate receptor type 3 [Source:HGNC Symbol;Acc:HGNC:6182]" | 29471 | -0.066 | 0.0518 | No |
| 139 | IL1B | interleukin 1 beta [Source:HGNC Symbol;Acc:HGNC:5992] | 29697 | -0.068 | 0.0494 | No |
| 140 | NDUFB5 | NADH:ubiquinone oxidoreductase subunit B5 [Source:HGNC Symbol;Acc:HGNC:7700] | 29913 | -0.070 | 0.0473 | No |
| 141 | PPP3CC | protein phosphatase 3 catalytic subunit gamma [Source:HGNC Symbol;Acc:HGNC:9316] | 30963 | -0.080 | 0.0244 | No |
| 142 | GRIN2B | glutamate ionotropic receptor NMDA type subunit 2B [Source:HGNC Symbol;Acc:HGNC:4586] | 31248 | -0.083 | 0.0212 | No |
| 143 | NDUFA8 | NADH:ubiquinone oxidoreductase subunit A8 [Source:HGNC Symbol;Acc:HGNC:7692] | 31722 | -0.088 | 0.0134 | No |
| 144 | PLCB2 | phospholipase C beta 2 [Source:HGNC Symbol;Acc:HGNC:9055] | 32369 | -0.095 | 0.0016 | No |
| 145 | ADAM10 | ADAM metallopeptidase domain 10 [Source:HGNC Symbol;Acc:HGNC:188] | 32901 | -0.102 | -0.0070 | No |
| 146 | CASP3 | caspase 3 [Source:HGNC Symbol;Acc:HGNC:1504] | 32939 | -0.103 | -0.0029 | No |
| 147 | MAPK1 | mitogen-activated protein kinase 1 [Source:HGNC Symbol;Acc:HGNC:6871] | 33350 | -0.108 | -0.0081 | No |
| 148 | CALML3 | calmodulin like 3 [Source:HGNC Symbol;Acc:HGNC:1452] | 33436 | -0.110 | -0.0049 | No |
| 149 | IDE | insulin degrading enzyme [Source:HGNC Symbol;Acc:HGNC:5381] | 33615 | -0.112 | -0.0040 | No |
| 150 | ATF6 | activating transcription factor 6 [Source:HGNC Symbol;Acc:HGNC:791] | 33814 | -0.115 | -0.0034 | No |
| 151 | ITPR2 | "inositol 1,4,5-trisphosphate receptor type 2 [Source:HGNC Symbol;Acc:HGNC:6181]" | 34058 | -0.119 | -0.0038 | No |
| 152 | CACNA1F | calcium voltage-gated channel subunit alpha1 F [Source:HGNC Symbol;Acc:HGNC:1393] | 34492 | -0.126 | -0.0088 | No |
| 153 | CHP2 | calcineurin like EF-hand protein 2 [Source:HGNC Symbol;Acc:HGNC:24927] | 34531 | -0.126 | -0.0036 | No |
| 154 | RYR3 | ryanodine receptor 3 [Source:HGNC Symbol;Acc:HGNC:10485] | 34793 | -0.131 | -0.0038 | No |
| 155 | LRP1 | LDL receptor related protein 1 [Source:HGNC Symbol;Acc:HGNC:6692] | 34823 | -0.132 | 0.0019 | No |
| 156 | APBB1 | amyloid beta precursor protein binding family B member 1 [Source:HGNC Symbol;Acc:HGNC:581] | 35277 | -0.141 | -0.0028 | No |
| 157 | ERN1 | endoplasmic reticulum to nucleus signaling 1 [Source:HGNC Symbol;Acc:HGNC:3449] | 35748 | -0.153 | -0.0073 | No |
| 158 | ATP2A2 | ATPase sarcoplasmic/endoplasmic reticulum Ca2+ transporting 2 [Source:HGNC Symbol;Acc:HGNC:812] | 35828 | -0.156 | -0.0017 | No |
| 159 | ITPR1 | "inositol 1,4,5-trisphosphate receptor type 1 [Source:HGNC Symbol;Acc:HGNC:6180]" | 36283 | -0.169 | -0.0050 | No |
| 160 | GNAQ | G protein subunit alpha q [Source:HGNC Symbol;Acc:HGNC:4390] | 36400 | -0.173 | 0.0005 | No |
| 161 | BACE1 | beta-secretase 1 [Source:HGNC Symbol;Acc:HGNC:933] | 36963 | -0.196 | -0.0043 | No |
| 162 | EIF2AK3 | eukaryotic translation initiation factor 2 alpha kinase 3 [Source:HGNC Symbol;Acc:HGNC:3255] | 37011 | -0.198 | 0.0041 | No |
| 163 | ADAM17 | ADAM metallopeptidase domain 17 [Source:HGNC Symbol;Acc:HGNC:195] | 37966 | -0.261 | -0.0075 | No |
| 164 | NOS1 | nitric oxide synthase 1 [Source:HGNC Symbol;Acc:HGNC:7872] | 38042 | -0.270 | 0.0038 | No |
| 165 | CDK5R1 | cyclin dependent kinase 5 regulatory subunit 1 [Source:HGNC Symbol;Acc:HGNC:1775] | 38130 | -0.282 | 0.0153 | No |
| 166 | PLCB4 | phospholipase C beta 4 [Source:HGNC Symbol;Acc:HGNC:9059] | 38308 | -0.309 | 0.0259 | No |
Table: GSEA details [plain text format]

  

Fig 2: KEGG\_ALZHEIMERS\_DISEASE      
 Blue-Pink O' Gram in the Space of the Analyzed GeneSet

  

Fig 3: KEGG\_ALZHEIMERS\_DISEASE: Random ES distribution      
 Gene set null distribution of ES for **KEGG\_ALZHEIMERS\_DISEASE**
