# Supplementary material for: Simultaneous Presentation of Multiple Myeloma and Lung Cancer: Case Report and Gene Bioinformatics Analysis
Source: Front Oncol. 2022 Jun 13;12:859735. doi: 10.3389/fonc.2022.859735 (PMC9235397; doi:10.3389/fonc.2022.859735)
Supplement: Supplementary file 1 [file DataSheet_1.zip › The bioinformatic analysis of MM and lung cancer supplementary materials/Enrichment analysis/MECR/GSEA_4.1.0/LUSC TCGA/KEGG.Gsea.1639042374131/KEGG_AMINOACYL_TRNA_BIOSYNTHESIS.html]

Details for gene set KEGG\_AMINOACYL\_TRNA\_BIOSYNTHESIS[GSEA]

|  || Dataset | ExpData\_collapsed\_to\_symbols.ENSG00000116353\_profile\_in\_ExpData.cls #ENSG00000116353 |
| Phenotype | ENSG00000116353\_profile\_in\_ExpData.cls#ENSG00000116353 |
| Upregulated in class | ENSG00000116353\_pos |
| GeneSet | KEGG\_AMINOACYL\_TRNA\_BIOSYNTHESIS |
| Enrichment Score (ES) | 0.5663876 |
| Normalized Enrichment Score (NES) | 1.9860454 |
| Nominal p-value | 0.0 |
| FDR q-value | 0.0014752655 |
| FWER p-Value | 0.019 |
Table: GSEA Results Summary

  

Fig 1: Enrichment plot: KEGG\_AMINOACYL\_TRNA\_BIOSYNTHESIS      
 Profile of the Running ES Score & Positions of GeneSet Members on the Rank Ordered List

  

| SYMBOL | TITLE | RANK IN GENE LIST | RANK METRIC SCORE | RUNNING ES | CORE ENRICHMENT || 1 | YARS1 | tyrosyl-tRNA synthetase 1 [Source:HGNC Symbol;Acc:HGNC:12840] | 112 | 0.385 | 0.0755 | Yes |
| 2 | SARS1 | seryl-tRNA synthetase 1 [Source:HGNC Symbol;Acc:HGNC:10537] | 212 | 0.335 | 0.1411 | Yes |
| 3 | PSTK | phosphoseryl-tRNA kinase [Source:HGNC Symbol;Acc:HGNC:28578] | 523 | 0.267 | 0.1876 | Yes |
| 4 | FARS2 | "phenylalanyl-tRNA synthetase 2, mitochondrial [Source:HGNC Symbol;Acc:HGNC:21062]" | 664 | 0.249 | 0.2347 | Yes |
| 5 | QARS1 | glutaminyl-tRNA synthetase 1 [Source:HGNC Symbol;Acc:HGNC:9751] | 826 | 0.229 | 0.2771 | Yes |
| 6 | WARS2 | "tryptophanyl tRNA synthetase 2, mitochondrial [Source:HGNC Symbol;Acc:HGNC:12730]" | 903 | 0.222 | 0.3203 | Yes |
| 7 | KARS1 | lysyl-tRNA synthetase 1 [Source:HGNC Symbol;Acc:HGNC:6215] | 1030 | 0.213 | 0.3604 | Yes |
| 8 | PARS2 | "prolyl-tRNA synthetase 2, mitochondrial [Source:HGNC Symbol;Acc:HGNC:30563]" | 1161 | 0.205 | 0.3988 | Yes |
| 9 | FARSB | phenylalanyl-tRNA synthetase subunit beta [Source:HGNC Symbol;Acc:HGNC:17800] | 1346 | 0.194 | 0.4336 | Yes |
| 10 | VARS1 | valyl-tRNA synthetase 1 [Source:HGNC Symbol;Acc:HGNC:12651] | 1528 | 0.185 | 0.4666 | Yes |
| 11 | NARS2 | "asparaginyl-tRNA synthetase 2, mitochondrial [Source:HGNC Symbol;Acc:HGNC:26274]" | 2520 | 0.149 | 0.4717 | Yes |
| 12 | CARS2 | "cysteinyl-tRNA synthetase 2, mitochondrial [Source:HGNC Symbol;Acc:HGNC:25695]" | 3083 | 0.135 | 0.4848 | Yes |
| 13 | SARS2 | "seryl-tRNA synthetase 2, mitochondrial [Source:HGNC Symbol;Acc:HGNC:17697]" | 3262 | 0.131 | 0.5069 | Yes |
| 14 | RARS1 | arginyl-tRNA synthetase 1 [Source:HGNC Symbol;Acc:HGNC:9870] | 3394 | 0.128 | 0.5297 | Yes |
| 15 | TARS2 | "threonyl-tRNA synthetase 2, mitochondrial [Source:HGNC Symbol;Acc:HGNC:30740]" | 4030 | 0.117 | 0.5374 | Yes |
| 16 | HARS1 | histidyl-tRNA synthetase 1 [Source:HGNC Symbol;Acc:HGNC:4816] | 4033 | 0.117 | 0.5612 | Yes |
| 17 | HARS2 | "histidyl-tRNA synthetase 2, mitochondrial [Source:HGNC Symbol;Acc:HGNC:4817]" | 4685 | 0.107 | 0.5664 | Yes |
| 18 | TARS1 | threonyl-tRNA synthetase 1 [Source:HGNC Symbol;Acc:HGNC:11572] | 7573 | 0.079 | 0.5089 | No |
| 19 | VARS2 | "valyl-tRNA synthetase 2, mitochondrial [Source:HGNC Symbol;Acc:HGNC:21642]" | 8649 | 0.070 | 0.4957 | No |
| 20 | AARS2 | "alanyl-tRNA synthetase 2, mitochondrial [Source:HGNC Symbol;Acc:HGNC:21022]" | 11801 | 0.048 | 0.4253 | No |
| 21 | FARSA | phenylalanyl-tRNA synthetase subunit alpha [Source:HGNC Symbol;Acc:HGNC:3592] | 13035 | 0.041 | 0.4022 | No |
| 22 | EARS2 | "glutamyl-tRNA synthetase 2, mitochondrial [Source:HGNC Symbol;Acc:HGNC:29419]" | 13513 | 0.038 | 0.3977 | No |
| 23 | YARS2 | tyrosyl-tRNA synthetase 2 [Source:HGNC Symbol;Acc:HGNC:24249] | 17962 | 0.012 | 0.2870 | No |
| 24 | WARS1 | tryptophanyl-tRNA synthetase 1 [Source:HGNC Symbol;Acc:HGNC:12729] | 19561 | 0.003 | 0.2470 | No |
| 25 | LARS1 | leucyl-tRNA synthetase 1 [Source:HGNC Symbol;Acc:HGNC:6512] | 21826 | -0.010 | 0.1914 | No |
| 26 | MARS2 | "methionyl-tRNA synthetase 2, mitochondrial [Source:HGNC Symbol;Acc:HGNC:25133]" | 22652 | -0.015 | 0.1735 | No |
| 27 | MARS1 | methionyl-tRNA synthetase 1 [Source:HGNC Symbol;Acc:HGNC:6898] | 23032 | -0.018 | 0.1674 | No |
| 28 | RARS2 | "arginyl-tRNA synthetase 2, mitochondrial [Source:HGNC Symbol;Acc:HGNC:21406]" | 25213 | -0.033 | 0.1186 | No |
| 29 | AARS1 | alanyl-tRNA synthetase 1 [Source:HGNC Symbol;Acc:HGNC:20] | 25225 | -0.033 | 0.1250 | No |
| 30 | CARS1 | cysteinyl-tRNA synthetase 1 [Source:HGNC Symbol;Acc:HGNC:1493] | 25823 | -0.037 | 0.1173 | No |
| 31 | SEPSECS | Sep (O-phosphoserine) tRNA:Sec (selenocysteine) tRNA synthase [Source:HGNC Symbol;Acc:HGNC:30605] | 27411 | -0.049 | 0.0868 | No |
| 32 | EPRS1 | glutamyl-prolyl-tRNA synthetase 1 [Source:HGNC Symbol;Acc:HGNC:3418] | 28287 | -0.056 | 0.0759 | No |
| 33 | NARS1 | asparaginyl-tRNA synthetase 1 [Source:HGNC Symbol;Acc:HGNC:7643] | 29757 | -0.068 | 0.0524 | No |
| 34 | TARS3 | threonyl-tRNA synthetase 3 [Source:HGNC Symbol;Acc:HGNC:24728] | 29806 | -0.069 | 0.0652 | No |
| 35 | LARS2 | "leucyl-tRNA synthetase 2, mitochondrial [Source:HGNC Symbol;Acc:HGNC:17095]" | 30688 | -0.077 | 0.0585 | No |
| 36 | DARS2 | "aspartyl-tRNA synthetase 2, mitochondrial [Source:HGNC Symbol;Acc:HGNC:25538]" | 31153 | -0.082 | 0.0634 | No |
| 37 | DARS1 | aspartyl-tRNA synthetase 1 [Source:HGNC Symbol;Acc:HGNC:2678] | 32782 | -0.100 | 0.0424 | No |
| 38 | MTFMT | mitochondrial methionyl-tRNA formyltransferase [Source:HGNC Symbol;Acc:HGNC:29666] | 33338 | -0.108 | 0.0502 | No |
| 39 | IARS2 | "isoleucyl-tRNA synthetase 2, mitochondrial [Source:HGNC Symbol;Acc:HGNC:29685]" | 36088 | -0.163 | 0.0133 | No |
| 40 | GARS1 | glycyl-tRNA synthetase 1 [Source:HGNC Symbol;Acc:HGNC:4162] | 36169 | -0.166 | 0.0450 | No |
| 41 | IARS1 | isoleucyl-tRNA synthetase 1 [Source:HGNC Symbol;Acc:HGNC:5330] | 36412 | -0.173 | 0.0741 | No |
Table: GSEA details [plain text format]

  

Fig 2: KEGG\_AMINOACYL\_TRNA\_BIOSYNTHESIS      
 Blue-Pink O' Gram in the Space of the Analyzed GeneSet

  

Fig 3: KEGG\_AMINOACYL\_TRNA\_BIOSYNTHESIS: Random ES distribution      
 Gene set null distribution of ES for **KEGG\_AMINOACYL\_TRNA\_BIOSYNTHESIS**
