# Supplementary material for: Simultaneous Presentation of Multiple Myeloma and Lung Cancer: Case Report and Gene Bioinformatics Analysis
Source: Front Oncol. 2022 Jun 13;12:859735. doi: 10.3389/fonc.2022.859735 (PMC9235397; doi:10.3389/fonc.2022.859735)
Supplement: Supplementary file 1 [file DataSheet_1.zip › The bioinformatic analysis of MM and lung cancer supplementary materials/Enrichment analysis/MECR/GSEA_4.1.0/LUSC TCGA/KEGG.Gsea.1639042374131/KEGG_AMINO_SUGAR_AND_NUCLEOTIDE_SUGAR_METABOLISM.html]

Details for gene set KEGG\_AMINO\_SUGAR\_AND\_NUCLEOTIDE\_SUGAR\_METABOLISM[GSEA]

|  || Dataset | ExpData\_collapsed\_to\_symbols.ENSG00000116353\_profile\_in\_ExpData.cls #ENSG00000116353 |
| Phenotype | ENSG00000116353\_profile\_in\_ExpData.cls#ENSG00000116353 |
| Upregulated in class | ENSG00000116353\_pos |
| GeneSet | KEGG\_AMINO\_SUGAR\_AND\_NUCLEOTIDE\_SUGAR\_METABOLISM |
| Enrichment Score (ES) | 0.4888111 |
| Normalized Enrichment Score (NES) | 1.7657372 |
| Nominal p-value | 0.001992032 |
| FDR q-value | 0.01145264 |
| FWER p-Value | 0.209 |
Table: GSEA Results Summary

  

Fig 1: Enrichment plot: KEGG\_AMINO\_SUGAR\_AND\_NUCLEOTIDE\_SUGAR\_METABOLISM      
 Profile of the Running ES Score & Positions of GeneSet Members on the Rank Ordered List

  

| SYMBOL | TITLE | RANK IN GENE LIST | RANK METRIC SCORE | RUNNING ES | CORE ENRICHMENT || 1 | GALE | UDP-galactose-4-epimerase [Source:HGNC Symbol;Acc:HGNC:4116] | 16 | 0.513 | 0.0792 | Yes |
| 2 | GFUS | GDP-L-fucose synthase [Source:HGNC Symbol;Acc:HGNC:12390] | 47 | 0.455 | 0.1490 | Yes |
| 3 | GMDS | "GDP-mannose 4,6-dehydratase [Source:HGNC Symbol;Acc:HGNC:4369]" | 92 | 0.404 | 0.2105 | Yes |
| 4 | CYB5R1 | cytochrome b5 reductase 1 [Source:HGNC Symbol;Acc:HGNC:13397] | 332 | 0.300 | 0.2509 | Yes |
| 5 | GMPPA | GDP-mannose pyrophosphorylase A [Source:HGNC Symbol;Acc:HGNC:22923] | 406 | 0.285 | 0.2932 | Yes |
| 6 | PGM1 | phosphoglucomutase 1 [Source:HGNC Symbol;Acc:HGNC:8905] | 514 | 0.268 | 0.3321 | Yes |
| 7 | GMPPB | GDP-mannose pyrophosphorylase B [Source:HGNC Symbol;Acc:HGNC:22932] | 1035 | 0.212 | 0.3518 | Yes |
| 8 | FCSK | fucose kinase [Source:HGNC Symbol;Acc:HGNC:29500] | 1069 | 0.211 | 0.3836 | Yes |
| 9 | AMDHD2 | amidohydrolase domain containing 2 [Source:HGNC Symbol;Acc:HGNC:24262] | 1491 | 0.187 | 0.4018 | Yes |
| 10 | GALK1 | galactokinase 1 [Source:HGNC Symbol;Acc:HGNC:4118] | 1823 | 0.172 | 0.4201 | Yes |
| 11 | FPGT | fucose-1-phosphate guanylyltransferase [Source:HGNC Symbol;Acc:HGNC:3825] | 2275 | 0.156 | 0.4329 | Yes |
| 12 | CYB5R3 | cytochrome b5 reductase 3 [Source:HGNC Symbol;Acc:HGNC:2873] | 2332 | 0.154 | 0.4554 | Yes |
| 13 | HEXA | hexosaminidase subunit alpha [Source:HGNC Symbol;Acc:HGNC:4878] | 2835 | 0.141 | 0.4645 | Yes |
| 14 | PMM1 | phosphomannomutase 1 [Source:HGNC Symbol;Acc:HGNC:9114] | 3355 | 0.129 | 0.4713 | Yes |
| 15 | UAP1 | UDP-N-acetylglucosamine pyrophosphorylase 1 [Source:HGNC Symbol;Acc:HGNC:12457] | 3443 | 0.127 | 0.4888 | Yes |
| 16 | GNPDA1 | glucosamine-6-phosphate deaminase 1 [Source:HGNC Symbol;Acc:HGNC:4417] | 6268 | 0.091 | 0.4310 | No |
| 17 | MPI | mannose phosphate isomerase [Source:HGNC Symbol;Acc:HGNC:7216] | 6332 | 0.090 | 0.4434 | No |
| 18 | NAGK | N-acetylglucosamine kinase [Source:HGNC Symbol;Acc:HGNC:17174] | 6503 | 0.088 | 0.4528 | No |
| 19 | GNPNAT1 | glucosamine-phosphate N-acetyltransferase 1 [Source:HGNC Symbol;Acc:HGNC:19980] | 7249 | 0.081 | 0.4465 | No |
| 20 | PMM2 | phosphomannomutase 2 [Source:HGNC Symbol;Acc:HGNC:9115] | 7611 | 0.078 | 0.4494 | No |
| 21 | CHIA | chitinase acidic [Source:HGNC Symbol;Acc:HGNC:17432] | 12832 | 0.042 | 0.3230 | No |
| 22 | GALT | galactose-1-phosphate uridylyltransferase [Source:HGNC Symbol;Acc:HGNC:4135] | 14188 | 0.034 | 0.2938 | No |
| 23 | GALK2 | galactokinase 2 [Source:HGNC Symbol;Acc:HGNC:4119] | 16523 | 0.020 | 0.2375 | No |
| 24 | NANP | N-acetylneuraminic acid phosphatase [Source:HGNC Symbol;Acc:HGNC:16140] | 16945 | 0.018 | 0.2296 | No |
| 25 | PGM2 | phosphoglucomutase 2 [Source:HGNC Symbol;Acc:HGNC:8906] | 17180 | 0.017 | 0.2262 | No |
| 26 | GFPT1 | glutamine--fructose-6-phosphate transaminase 1 [Source:HGNC Symbol;Acc:HGNC:4241] | 20424 | -0.002 | 0.1439 | No |
| 27 | CMAS | cytidine monophosphate N-acetylneuraminic acid synthetase [Source:HGNC Symbol;Acc:HGNC:18290] | 21437 | -0.008 | 0.1194 | No |
| 28 | UGP2 | UDP-glucose pyrophosphorylase 2 [Source:HGNC Symbol;Acc:HGNC:12527] | 22481 | -0.014 | 0.0950 | No |
| 29 | NANS | N-acetylneuraminate synthase [Source:HGNC Symbol;Acc:HGNC:19237] | 22568 | -0.015 | 0.0951 | No |
| 30 | GFPT2 | glutamine-fructose-6-phosphate transaminase 2 [Source:HGNC Symbol;Acc:HGNC:4242] | 23775 | -0.022 | 0.0679 | No |
| 31 | GCK | glucokinase [Source:HGNC Symbol;Acc:HGNC:4195] | 26227 | -0.040 | 0.0117 | No |
| 32 | GNPDA2 | glucosamine-6-phosphate deaminase 2 [Source:HGNC Symbol;Acc:HGNC:21526] | 27119 | -0.046 | -0.0038 | No |
| 33 | RENBP | renin binding protein [Source:HGNC Symbol;Acc:HGNC:9959] | 29136 | -0.063 | -0.0454 | No |
| 34 | GNE | glucosamine (UDP-N-acetyl)-2-epimerase/N-acetylmannosamine kinase [Source:HGNC Symbol;Acc:HGNC:23657] | 32131 | -0.092 | -0.1073 | No |
| 35 | HEXB | hexosaminidase subunit beta [Source:HGNC Symbol;Acc:HGNC:4879] | 32486 | -0.097 | -0.1014 | No |
| 36 | HK3 | hexokinase 3 [Source:HGNC Symbol;Acc:HGNC:4925] | 34398 | -0.124 | -0.1307 | No |
| 37 | PGM3 | phosphoglucomutase 3 [Source:HGNC Symbol;Acc:HGNC:8907] | 35437 | -0.145 | -0.1347 | No |
| 38 | GPI | glucose-6-phosphate isomerase [Source:HGNC Symbol;Acc:HGNC:4458] | 35660 | -0.151 | -0.1169 | No |
| 39 | UXS1 | UDP-glucuronate decarboxylase 1 [Source:HGNC Symbol;Acc:HGNC:17729] | 36145 | -0.165 | -0.1037 | No |
| 40 | HK2 | hexokinase 2 [Source:HGNC Symbol;Acc:HGNC:4923] | 36351 | -0.171 | -0.0823 | No |
| 41 | CHIT1 | chitinase 1 [Source:HGNC Symbol;Acc:HGNC:1936] | 36702 | -0.184 | -0.0626 | No |
| 42 | HK1 | hexokinase 1 [Source:HGNC Symbol;Acc:HGNC:4922] | 37648 | -0.234 | -0.0504 | No |
| 43 | UGDH | UDP-glucose 6-dehydrogenase [Source:HGNC Symbol;Acc:HGNC:12525] | 38224 | -0.296 | -0.0191 | No |
| 44 | NPL | N-acetylneuraminate pyruvate lyase [Source:HGNC Symbol;Acc:HGNC:16781] | 38277 | -0.304 | 0.0266 | No |
Table: GSEA details [plain text format]

  

Fig 2: KEGG\_AMINO\_SUGAR\_AND\_NUCLEOTIDE\_SUGAR\_METABOLISM      
 Blue-Pink O' Gram in the Space of the Analyzed GeneSet

  

Fig 3: KEGG\_AMINO\_SUGAR\_AND\_NUCLEOTIDE\_SUGAR\_METABOLISM: Random ES distribution      
 Gene set null distribution of ES for **KEGG\_AMINO\_SUGAR\_AND\_NUCLEOTIDE\_SUGAR\_METABOLISM**
