# Supplementary material for: Simultaneous Presentation of Multiple Myeloma and Lung Cancer: Case Report and Gene Bioinformatics Analysis
Source: Front Oncol. 2022 Jun 13;12:859735. doi: 10.3389/fonc.2022.859735 (PMC9235397; doi:10.3389/fonc.2022.859735)
Supplement: Supplementary file 1 [file DataSheet_1.zip › The bioinformatic analysis of MM and lung cancer supplementary materials/Enrichment analysis/MECR/GSEA_4.1.0/LUSC TCGA/KEGG.Gsea.1639042374131/KEGG_BASAL_CELL_CARCINOMA.html]

Details for gene set KEGG\_BASAL\_CELL\_CARCINOMA[GSEA]

|  || Dataset | ExpData\_collapsed\_to\_symbols.ENSG00000116353\_profile\_in\_ExpData.cls #ENSG00000116353 |
| Phenotype | ENSG00000116353\_profile\_in\_ExpData.cls#ENSG00000116353 |
| Upregulated in class | ENSG00000116353\_neg |
| GeneSet | KEGG\_BASAL\_CELL\_CARCINOMA |
| Enrichment Score (ES) | -0.519311 |
| Normalized Enrichment Score (NES) | -1.9838917 |
| Nominal p-value | 0.0 |
| FDR q-value | 0.003015587 |
| FWER p-Value | 0.012 |
Table: GSEA Results Summary

  

Fig 1: Enrichment plot: KEGG\_BASAL\_CELL\_CARCINOMA      
 Profile of the Running ES Score & Positions of GeneSet Members on the Rank Ordered List

  

| SYMBOL | TITLE | RANK IN GENE LIST | RANK METRIC SCORE | RUNNING ES | CORE ENRICHMENT || 1 | DVL1 | dishevelled segment polarity protein 1 [Source:HGNC Symbol;Acc:HGNC:3084] | 49 | 0.451 | 0.0556 | No |
| 2 | WNT10A | Wnt family member 10A [Source:HGNC Symbol;Acc:HGNC:13829] | 839 | 0.228 | 0.0642 | No |
| 3 | WNT4 | Wnt family member 4 [Source:HGNC Symbol;Acc:HGNC:12783] | 1086 | 0.210 | 0.0844 | No |
| 4 | TCF7L2 | transcription factor 7 like 2 [Source:HGNC Symbol;Acc:HGNC:11641] | 1930 | 0.168 | 0.0841 | No |
| 5 | GLI3 | GLI family zinc finger 3 [Source:HGNC Symbol;Acc:HGNC:4319] | 3544 | 0.126 | 0.0588 | No |
| 6 | SUFU | SUFU negative regulator of hedgehog signaling [Source:HGNC Symbol;Acc:HGNC:16466] | 3947 | 0.119 | 0.0635 | No |
| 7 | WNT6 | Wnt family member 6 [Source:HGNC Symbol;Acc:HGNC:12785] | 4672 | 0.107 | 0.0586 | No |
| 8 | WNT16 | Wnt family member 16 [Source:HGNC Symbol;Acc:HGNC:16267] | 6037 | 0.094 | 0.0357 | No |
| 9 | WNT8A | Wnt family member 8A [Source:HGNC Symbol;Acc:HGNC:12788] | 8416 | 0.071 | -0.0159 | No |
| 10 | WNT7A | Wnt family member 7A [Source:HGNC Symbol;Acc:HGNC:12786] | 10774 | 0.055 | -0.0690 | No |
| 11 | FZD5 | frizzled class receptor 5 [Source:HGNC Symbol;Acc:HGNC:4043] | 11161 | 0.053 | -0.0722 | No |
| 12 | STK36 | serine/threonine kinase 36 [Source:HGNC Symbol;Acc:HGNC:17209] | 12694 | 0.043 | -0.1058 | No |
| 13 | TCF7 | transcription factor 7 [Source:HGNC Symbol;Acc:HGNC:11639] | 13002 | 0.041 | -0.1085 | No |
| 14 | AXIN1 | axin 1 [Source:HGNC Symbol;Acc:HGNC:903] | 15360 | 0.027 | -0.1651 | No |
| 15 | GSK3B | glycogen synthase kinase 3 beta [Source:HGNC Symbol;Acc:HGNC:4617] | 15772 | 0.025 | -0.1725 | No |
| 16 | APC2 | APC regulator of WNT signaling pathway 2 [Source:HGNC Symbol;Acc:HGNC:24036] | 18523 | 0.009 | -0.2413 | No |
| 17 | WNT1 | Wnt family member 1 [Source:HGNC Symbol;Acc:HGNC:12774] | 19377 | 0.004 | -0.2625 | No |
| 18 | FZD8 | frizzled class receptor 8 [Source:HGNC Symbol;Acc:HGNC:4046] | 20256 | -0.001 | -0.2848 | No |
| 19 | TCF7L1 | transcription factor 7 like 1 [Source:HGNC Symbol;Acc:HGNC:11640] | 20720 | -0.004 | -0.2961 | No |
| 20 | FZD4 | frizzled class receptor 4 [Source:HGNC Symbol;Acc:HGNC:4042] | 20770 | -0.004 | -0.2969 | No |
| 21 | BMP2 | bone morphogenetic protein 2 [Source:HGNC Symbol;Acc:HGNC:1069] | 20791 | -0.004 | -0.2969 | No |
| 22 | WNT7B | Wnt family member 7B [Source:HGNC Symbol;Acc:HGNC:12787] | 22905 | -0.017 | -0.3486 | No |
| 23 | WNT9A | Wnt family member 9A [Source:HGNC Symbol;Acc:HGNC:12778] | 25791 | -0.037 | -0.4174 | No |
| 24 | FZD1 | frizzled class receptor 1 [Source:HGNC Symbol;Acc:HGNC:4038] | 26540 | -0.042 | -0.4312 | No |
| 25 | WNT10B | Wnt family member 10B [Source:HGNC Symbol;Acc:HGNC:12775] | 26603 | -0.042 | -0.4274 | No |
| 26 | HHIP | hedgehog interacting protein [Source:HGNC Symbol;Acc:HGNC:14866] | 28238 | -0.055 | -0.4620 | No |
| 27 | FZD9 | frizzled class receptor 9 [Source:HGNC Symbol;Acc:HGNC:4047] | 28404 | -0.057 | -0.4591 | No |
| 28 | WNT3A | Wnt family member 3A [Source:HGNC Symbol;Acc:HGNC:15983] | 29041 | -0.062 | -0.4675 | No |
| 29 | BMP4 | bone morphogenetic protein 4 [Source:HGNC Symbol;Acc:HGNC:1071] | 29894 | -0.070 | -0.4804 | No |
| 30 | TP53 | tumor protein p53 [Source:HGNC Symbol;Acc:HGNC:11998] | 30908 | -0.079 | -0.4962 | No |
| 31 | FZD3 | frizzled class receptor 3 [Source:HGNC Symbol;Acc:HGNC:4041] | 31252 | -0.083 | -0.4945 | No |
| 32 | DVL2 | dishevelled segment polarity protein 2 [Source:HGNC Symbol;Acc:HGNC:3086] | 31825 | -0.089 | -0.4979 | No |
| 33 | WNT5B | Wnt family member 5B [Source:HGNC Symbol;Acc:HGNC:16265] | 32250 | -0.094 | -0.4969 | No |
| 34 | WNT2 | Wnt family member 2 [Source:HGNC Symbol;Acc:HGNC:12780] | 32471 | -0.096 | -0.4904 | No |
| 35 | AXIN2 | axin 2 [Source:HGNC Symbol;Acc:HGNC:904] | 32772 | -0.100 | -0.4853 | No |
| 36 | WNT8B | Wnt family member 8B [Source:HGNC Symbol;Acc:HGNC:12789] | 33128 | -0.105 | -0.4811 | No |
| 37 | FZD2 | frizzled class receptor 2 [Source:HGNC Symbol;Acc:HGNC:4040] | 34628 | -0.128 | -0.5032 | Yes |
| 38 | FZD6 | frizzled class receptor 6 [Source:HGNC Symbol;Acc:HGNC:4044] | 35028 | -0.136 | -0.4962 | Yes |
| 39 | PTCH2 | patched 2 [Source:HGNC Symbol;Acc:HGNC:9586] | 35779 | -0.154 | -0.4959 | Yes |
| 40 | CTNNB1 | catenin beta 1 [Source:HGNC Symbol;Acc:HGNC:2514] | 35880 | -0.157 | -0.4786 | Yes |
| 41 | WNT9B | Wnt family member 9B [Source:HGNC Symbol;Acc:HGNC:12779] | 35896 | -0.157 | -0.4592 | Yes |
| 42 | WNT2B | Wnt family member 2B [Source:HGNC Symbol;Acc:HGNC:12781] | 36087 | -0.163 | -0.4435 | Yes |
| 43 | SHH | sonic hedgehog signaling molecule [Source:HGNC Symbol;Acc:HGNC:10848] | 36592 | -0.179 | -0.4337 | Yes |
| 44 | WNT11 | Wnt family member 11 [Source:HGNC Symbol;Acc:HGNC:12776] | 37666 | -0.236 | -0.4314 | Yes |
| 45 | DVL3 | dishevelled segment polarity protein 3 [Source:HGNC Symbol;Acc:HGNC:3087] | 37983 | -0.264 | -0.4062 | Yes |
| 46 | WNT3 | Wnt family member 3 [Source:HGNC Symbol;Acc:HGNC:12782] | 38054 | -0.271 | -0.3738 | Yes |
| 47 | SMO | "smoothened, frizzled class receptor [Source:HGNC Symbol;Acc:HGNC:11119]" | 38185 | -0.291 | -0.3404 | Yes |
| 48 | LEF1 | lymphoid enhancer binding factor 1 [Source:HGNC Symbol;Acc:HGNC:6551] | 38341 | -0.316 | -0.3046 | Yes |
| 49 | FZD10 | frizzled class receptor 10 [Source:HGNC Symbol;Acc:HGNC:4039] | 38368 | -0.322 | -0.2647 | Yes |
| 50 | APC | APC regulator of WNT signaling pathway [Source:HGNC Symbol;Acc:HGNC:583] | 38413 | -0.336 | -0.2235 | Yes |
| 51 | FZD7 | frizzled class receptor 7 [Source:HGNC Symbol;Acc:HGNC:4045] | 38485 | -0.367 | -0.1791 | Yes |
| 52 | WNT5A | Wnt family member 5A [Source:HGNC Symbol;Acc:HGNC:12784] | 38488 | -0.368 | -0.1328 | Yes |
| 53 | GLI1 | GLI family zinc finger 1 [Source:HGNC Symbol;Acc:HGNC:4317] | 38507 | -0.378 | -0.0856 | Yes |
| 54 | PTCH1 | patched 1 [Source:HGNC Symbol;Acc:HGNC:9585] | 38519 | -0.389 | -0.0369 | Yes |
| 55 | GLI2 | GLI family zinc finger 2 [Source:HGNC Symbol;Acc:HGNC:4318] | 38549 | -0.455 | 0.0197 | Yes |
Table: GSEA details [plain text format]

  

Fig 2: KEGG\_BASAL\_CELL\_CARCINOMA      
 Blue-Pink O' Gram in the Space of the Analyzed GeneSet

  

Fig 3: KEGG\_BASAL\_CELL\_CARCINOMA: Random ES distribution      
 Gene set null distribution of ES for **KEGG\_BASAL\_CELL\_CARCINOMA**
