# Supplementary material for: Simultaneous Presentation of Multiple Myeloma and Lung Cancer: Case Report and Gene Bioinformatics Analysis
Source: Front Oncol. 2022 Jun 13;12:859735. doi: 10.3389/fonc.2022.859735 (PMC9235397; doi:10.3389/fonc.2022.859735)
Supplement: Supplementary file 1 [file DataSheet_1.zip › The bioinformatic analysis of MM and lung cancer supplementary materials/Enrichment analysis/MECR/GSEA_4.1.0/LUSC TCGA/KEGG.Gsea.1639042374131/KEGG_BASE_EXCISION_REPAIR.html]

Details for gene set KEGG\_BASE\_EXCISION\_REPAIR[GSEA]

|  || Dataset | ExpData\_collapsed\_to\_symbols.ENSG00000116353\_profile\_in\_ExpData.cls #ENSG00000116353 |
| Phenotype | ENSG00000116353\_profile\_in\_ExpData.cls#ENSG00000116353 |
| Upregulated in class | ENSG00000116353\_pos |
| GeneSet | KEGG\_BASE\_EXCISION\_REPAIR |
| Enrichment Score (ES) | 0.55701613 |
| Normalized Enrichment Score (NES) | 1.9334145 |
| Nominal p-value | 0.0 |
| FDR q-value | 0.0020984395 |
| FWER p-Value | 0.035 |
Table: GSEA Results Summary

  

Fig 1: Enrichment plot: KEGG\_BASE\_EXCISION\_REPAIR      
 Profile of the Running ES Score & Positions of GeneSet Members on the Rank Ordered List

  

| SYMBOL | TITLE | RANK IN GENE LIST | RANK METRIC SCORE | RUNNING ES | CORE ENRICHMENT || 1 | MUTYH | mutY DNA glycosylase [Source:HGNC Symbol;Acc:HGNC:7527] | 333 | 0.300 | 0.0646 | Yes |
| 2 | POLE4 | "DNA polymerase epsilon 4, accessory subunit [Source:HGNC Symbol;Acc:HGNC:18755]" | 759 | 0.237 | 0.1116 | Yes |
| 3 | OGG1 | 8-oxoguanine DNA glycosylase [Source:HGNC Symbol;Acc:HGNC:8125] | 1002 | 0.214 | 0.1577 | Yes |
| 4 | APEX1 | apurinic/apyrimidinic endodeoxyribonuclease 1 [Source:HGNC Symbol;Acc:HGNC:587] | 1125 | 0.207 | 0.2051 | Yes |
| 5 | POLL | DNA polymerase lambda [Source:HGNC Symbol;Acc:HGNC:9184] | 1217 | 0.202 | 0.2520 | Yes |
| 6 | NTHL1 | nth like DNA glycosylase 1 [Source:HGNC Symbol;Acc:HGNC:8028] | 1264 | 0.199 | 0.2994 | Yes |
| 7 | POLD1 | "DNA polymerase delta 1, catalytic subunit [Source:HGNC Symbol;Acc:HGNC:9175]" | 1356 | 0.194 | 0.3443 | Yes |
| 8 | POLB | DNA polymerase beta [Source:HGNC Symbol;Acc:HGNC:9174] | 1483 | 0.187 | 0.3867 | Yes |
| 9 | POLE2 | "DNA polymerase epsilon 2, accessory subunit [Source:HGNC Symbol;Acc:HGNC:9178]" | 1540 | 0.184 | 0.4303 | Yes |
| 10 | XRCC1 | X-ray repair cross complementing 1 [Source:HGNC Symbol;Acc:HGNC:12828] | 1623 | 0.181 | 0.4724 | Yes |
| 11 | MPG | N-methylpurine DNA glycosylase [Source:HGNC Symbol;Acc:HGNC:7211] | 2192 | 0.159 | 0.4967 | Yes |
| 12 | LIG1 | DNA ligase 1 [Source:HGNC Symbol;Acc:HGNC:6598] | 2424 | 0.152 | 0.5278 | Yes |
| 13 | POLD4 | "DNA polymerase delta 4, accessory subunit [Source:HGNC Symbol;Acc:HGNC:14106]" | 3573 | 0.125 | 0.5290 | Yes |
| 14 | HMGB1P1 | high mobility group box 1 pseudogene 1 [Source:HGNC Symbol;Acc:HGNC:4993] | 3657 | 0.124 | 0.5570 | Yes |
| 15 | PCNA | proliferating cell nuclear antigen [Source:HGNC Symbol;Acc:HGNC:8729] | 6790 | 0.086 | 0.4982 | No |
| 16 | POLD2 | "DNA polymerase delta 2, accessory subunit [Source:HGNC Symbol;Acc:HGNC:9176]" | 7505 | 0.079 | 0.4993 | No |
| 17 | SMUG1 | single-strand-selective monofunctional uracil-DNA glycosylase 1 [Source:HGNC Symbol;Acc:HGNC:17148] | 8926 | 0.068 | 0.4797 | No |
| 18 | PARP2 | poly(ADP-ribose) polymerase 2 [Source:HGNC Symbol;Acc:HGNC:272] | 9819 | 0.061 | 0.4720 | No |
| 19 | APEX2 | apurinic/apyrimidinic endodeoxyribonuclease 2 [Source:HGNC Symbol;Acc:HGNC:17889] | 11687 | 0.049 | 0.4364 | No |
| 20 | MBD4 | "methyl-CpG binding domain 4, DNA glycosylase [Source:HGNC Symbol;Acc:HGNC:6919]" | 16464 | 0.021 | 0.3199 | No |
| 21 | POLD3 | "DNA polymerase delta 3, accessory subunit [Source:HGNC Symbol;Acc:HGNC:20932]" | 16528 | 0.020 | 0.3233 | No |
| 22 | TDG | thymine DNA glycosylase [Source:HGNC Symbol;Acc:HGNC:11700] | 18894 | 0.007 | 0.2648 | No |
| 23 | UNG | uracil DNA glycosylase [Source:HGNC Symbol;Acc:HGNC:12572] | 19034 | 0.006 | 0.2629 | No |
| 24 | POLE | "DNA polymerase epsilon, catalytic subunit [Source:HGNC Symbol;Acc:HGNC:9177]" | 19623 | 0.003 | 0.2486 | No |
| 25 | NEIL2 | nei like DNA glycosylase 2 [Source:HGNC Symbol;Acc:HGNC:18956] | 21399 | -0.008 | 0.2053 | No |
| 26 | HMGB1 | high mobility group box 1 [Source:HGNC Symbol;Acc:HGNC:4983] | 21518 | -0.008 | 0.2043 | No |
| 27 | PARP3 | poly(ADP-ribose) polymerase family member 3 [Source:HGNC Symbol;Acc:HGNC:273] | 24726 | -0.029 | 0.1298 | No |
| 28 | HMGB1P40 | high mobility group box 1 pseudogene 40 [Source:HGNC Symbol;Acc:HGNC:39187] | 28477 | -0.057 | 0.0483 | No |
| 29 | PARP1 | poly(ADP-ribose) polymerase 1 [Source:HGNC Symbol;Acc:HGNC:270] | 28579 | -0.058 | 0.0598 | No |
| 30 | NEIL3 | nei like DNA glycosylase 3 [Source:HGNC Symbol;Acc:HGNC:24573] | 29852 | -0.069 | 0.0443 | No |
| 31 | FEN1 | flap structure-specific endonuclease 1 [Source:HGNC Symbol;Acc:HGNC:3650] | 34204 | -0.121 | -0.0369 | No |
| 32 | POLE3 | "DNA polymerase epsilon 3, accessory subunit [Source:HGNC Symbol;Acc:HGNC:13546]" | 34528 | -0.126 | -0.0144 | No |
| 33 | NEIL1 | nei like DNA glycosylase 1 [Source:HGNC Symbol;Acc:HGNC:18448] | 36098 | -0.163 | -0.0146 | No |
| 34 | LIG3 | DNA ligase 3 [Source:HGNC Symbol;Acc:HGNC:6600] | 36738 | -0.186 | 0.0144 | No |
| 35 | PARP4 | poly(ADP-ribose) polymerase family member 4 [Source:HGNC Symbol;Acc:HGNC:271] | 37265 | -0.211 | 0.0524 | No |
Table: GSEA details [plain text format]

  

Fig 2: KEGG\_BASE\_EXCISION\_REPAIR      
 Blue-Pink O' Gram in the Space of the Analyzed GeneSet

  

Fig 3: KEGG\_BASE\_EXCISION\_REPAIR: Random ES distribution      
 Gene set null distribution of ES for **KEGG\_BASE\_EXCISION\_REPAIR**
