# Supplementary material for: Simultaneous Presentation of Multiple Myeloma and Lung Cancer: Case Report and Gene Bioinformatics Analysis
Source: Front Oncol. 2022 Jun 13;12:859735. doi: 10.3389/fonc.2022.859735 (PMC9235397; doi:10.3389/fonc.2022.859735)
Supplement: Supplementary file 1 [file DataSheet_1.zip › The bioinformatic analysis of MM and lung cancer supplementary materials/Enrichment analysis/MECR/GSEA_4.1.0/LUSC TCGA/KEGG.Gsea.1639042374131/KEGG_CALCIUM_SIGNALING_PATHWAY.html]

Details for gene set KEGG\_CALCIUM\_SIGNALING\_PATHWAY[GSEA]

|  || Dataset | ExpData\_collapsed\_to\_symbols.ENSG00000116353\_profile\_in\_ExpData.cls #ENSG00000116353 |
| Phenotype | ENSG00000116353\_profile\_in\_ExpData.cls#ENSG00000116353 |
| Upregulated in class | ENSG00000116353\_neg |
| GeneSet | KEGG\_CALCIUM\_SIGNALING\_PATHWAY |
| Enrichment Score (ES) | -0.43919662 |
| Normalized Enrichment Score (NES) | -2.0581248 |
| Nominal p-value | 0.0 |
| FDR q-value | 0.0011524332 |
| FWER p-Value | 0.003 |
Table: GSEA Results Summary

  

Fig 1: Enrichment plot: KEGG\_CALCIUM\_SIGNALING\_PATHWAY      
 Profile of the Running ES Score & Positions of GeneSet Members on the Rank Ordered List

  

| SYMBOL | TITLE | RANK IN GENE LIST | RANK METRIC SCORE | RUNNING ES | CORE ENRICHMENT || 1 | CALM3 | calmodulin 3 [Source:HGNC Symbol;Acc:HGNC:1449] | 850 | 0.227 | -0.0083 | No |
| 2 | CAMK2G | calcium/calmodulin dependent protein kinase II gamma [Source:HGNC Symbol;Acc:HGNC:1463] | 1746 | 0.175 | -0.0207 | No |
| 3 | PHKG2 | phosphorylase kinase catalytic subunit gamma 2 [Source:HGNC Symbol;Acc:HGNC:8931] | 1948 | 0.167 | -0.0159 | No |
| 4 | PLCB3 | phospholipase C beta 3 [Source:HGNC Symbol;Acc:HGNC:9056] | 1971 | 0.167 | -0.0066 | No |
| 5 | PPP3CA | protein phosphatase 3 catalytic subunit alpha [Source:HGNC Symbol;Acc:HGNC:9314] | 2508 | 0.149 | -0.0115 | No |
| 6 | ADRB1 | adrenoceptor beta 1 [Source:HGNC Symbol;Acc:HGNC:285] | 2785 | 0.142 | -0.0101 | No |
| 7 | SLC25A6 | solute carrier family 25 member 6 [Source:HGNC Symbol;Acc:HGNC:10992] | 2903 | 0.139 | -0.0049 | No |
| 8 | BDKRB1 | bradykinin receptor B1 [Source:HGNC Symbol;Acc:HGNC:1029] | 3078 | 0.135 | -0.0013 | No |
| 9 | CACNA1D | calcium voltage-gated channel subunit alpha1 D [Source:HGNC Symbol;Acc:HGNC:1391] | 3402 | 0.128 | -0.0020 | No |
| 10 | PLCD3 | phospholipase C delta 3 [Source:HGNC Symbol;Acc:HGNC:9061] | 3547 | 0.126 | 0.0018 | No |
| 11 | TNNC2 | "troponin C2, fast skeletal type [Source:HGNC Symbol;Acc:HGNC:11944]" | 3651 | 0.124 | 0.0065 | No |
| 12 | MYLK2 | myosin light chain kinase 2 [Source:HGNC Symbol;Acc:HGNC:16243] | 3703 | 0.123 | 0.0124 | No |
| 13 | SPHK2 | sphingosine kinase 2 [Source:HGNC Symbol;Acc:HGNC:18859] | 3888 | 0.120 | 0.0148 | No |
| 14 | GNAS | GNAS complex locus [Source:HGNC Symbol;Acc:HGNC:4392] | 4003 | 0.118 | 0.0189 | No |
| 15 | PHKG1 | phosphorylase kinase catalytic subunit gamma 1 [Source:HGNC Symbol;Acc:HGNC:8930] | 4730 | 0.107 | 0.0067 | No |
| 16 | GRIN2C | glutamate ionotropic receptor NMDA type subunit 2C [Source:HGNC Symbol;Acc:HGNC:4587] | 6018 | 0.094 | -0.0207 | No |
| 17 | CALM2 | calmodulin 2 [Source:HGNC Symbol;Acc:HGNC:1445] | 6292 | 0.091 | -0.0223 | No |
| 18 | AGTR1 | angiotensin II receptor type 1 [Source:HGNC Symbol;Acc:HGNC:336] | 6417 | 0.089 | -0.0201 | No |
| 19 | CALML5 | calmodulin like 5 [Source:HGNC Symbol;Acc:HGNC:18180] | 6619 | 0.087 | -0.0201 | No |
| 20 | GRIN2D | glutamate ionotropic receptor NMDA type subunit 2D [Source:HGNC Symbol;Acc:HGNC:4588] | 6650 | 0.087 | -0.0157 | No |
| 21 | PTGER1 | prostaglandin E receptor 1 [Source:HGNC Symbol;Acc:HGNC:9593] | 6996 | 0.084 | -0.0195 | No |
| 22 | SLC25A5 | solute carrier family 25 member 5 [Source:HGNC Symbol;Acc:HGNC:10991] | 7217 | 0.082 | -0.0203 | No |
| 23 | TNNC1 | "troponin C1, slow skeletal and cardiac type [Source:HGNC Symbol;Acc:HGNC:11943]" | 8001 | 0.075 | -0.0359 | No |
| 24 | PPP3CB | protein phosphatase 3 catalytic subunit beta [Source:HGNC Symbol;Acc:HGNC:9315] | 8034 | 0.075 | -0.0323 | No |
| 25 | ERBB3 | erb-b2 receptor tyrosine kinase 3 [Source:HGNC Symbol;Acc:HGNC:3431] | 8229 | 0.073 | -0.0329 | No |
| 26 | CAMK2D | calcium/calmodulin dependent protein kinase II delta [Source:HGNC Symbol;Acc:HGNC:1462] | 8249 | 0.073 | -0.0291 | No |
| 27 | MYLK3 | myosin light chain kinase 3 [Source:HGNC Symbol;Acc:HGNC:29826] | 8377 | 0.072 | -0.0281 | No |
| 28 | DRD5 | dopamine receptor D5 [Source:HGNC Symbol;Acc:HGNC:3026] | 8680 | 0.069 | -0.0317 | No |
| 29 | CACNA1A | calcium voltage-gated channel subunit alpha1 A [Source:HGNC Symbol;Acc:HGNC:1388] | 8843 | 0.068 | -0.0318 | No |
| 30 | ADRB2 | adrenoceptor beta 2 [Source:HGNC Symbol;Acc:HGNC:286] | 8846 | 0.068 | -0.0278 | No |
| 31 | BDKRB2 | bradykinin receptor B2 [Source:HGNC Symbol;Acc:HGNC:1030] | 8996 | 0.067 | -0.0276 | No |
| 32 | ERBB2 | erb-b2 receptor tyrosine kinase 2 [Source:HGNC Symbol;Acc:HGNC:3430] | 9497 | 0.064 | -0.0366 | No |
| 33 | SLC25A4 | solute carrier family 25 member 4 [Source:HGNC Symbol;Acc:HGNC:10990] | 9848 | 0.061 | -0.0419 | No |
| 34 | TACR3 | tachykinin receptor 3 [Source:HGNC Symbol;Acc:HGNC:11528] | 10021 | 0.060 | -0.0427 | No |
| 35 | CAMK2B | calcium/calmodulin dependent protein kinase II beta [Source:HGNC Symbol;Acc:HGNC:1461] | 10058 | 0.060 | -0.0401 | No |
| 36 | ADRA1B | adrenoceptor alpha 1B [Source:HGNC Symbol;Acc:HGNC:278] | 10678 | 0.056 | -0.0526 | No |
| 37 | PTAFR | platelet activating factor receptor [Source:HGNC Symbol;Acc:HGNC:9582] | 11052 | 0.053 | -0.0590 | No |
| 38 | HRH1 | histamine receptor H1 [Source:HGNC Symbol;Acc:HGNC:5182] | 11260 | 0.052 | -0.0612 | No |
| 39 | CHP1 | calcineurin like EF-hand protein 1 [Source:HGNC Symbol;Acc:HGNC:17433] | 12465 | 0.044 | -0.0893 | No |
| 40 | CAMK2A | calcium/calmodulin dependent protein kinase II alpha [Source:HGNC Symbol;Acc:HGNC:1460] | 13120 | 0.040 | -0.1037 | No |
| 41 | GRIN1 | glutamate ionotropic receptor NMDA type subunit 1 [Source:HGNC Symbol;Acc:HGNC:4584] | 13266 | 0.039 | -0.1050 | No |
| 42 | VDAC1 | voltage dependent anion channel 1 [Source:HGNC Symbol;Acc:HGNC:12669] | 13291 | 0.039 | -0.1033 | No |
| 43 | PLCG1 | phospholipase C gamma 1 [Source:HGNC Symbol;Acc:HGNC:9065] | 13655 | 0.037 | -0.1104 | No |
| 44 | ERBB4 | erb-b2 receptor tyrosine kinase 4 [Source:HGNC Symbol;Acc:HGNC:3432] | 14022 | 0.035 | -0.1177 | No |
| 45 | ADORA2B | adenosine A2b receptor [Source:HGNC Symbol;Acc:HGNC:264] | 14239 | 0.033 | -0.1212 | No |
| 46 | SLC25A31 | solute carrier family 25 member 31 [Source:HGNC Symbol;Acc:HGNC:25319] | 14788 | 0.030 | -0.1334 | No |
| 47 | PRKCG | protein kinase C gamma [Source:HGNC Symbol;Acc:HGNC:9402] | 15351 | 0.027 | -0.1462 | No |
| 48 | PPP3R2 | "protein phosphatase 3 regulatory subunit B, beta [Source:HGNC Symbol;Acc:HGNC:9318]" | 15798 | 0.024 | -0.1561 | No |
| 49 | ADRB3 | adrenoceptor beta 3 [Source:HGNC Symbol;Acc:HGNC:288] | 15881 | 0.024 | -0.1568 | No |
| 50 | ADRA1D | adrenoceptor alpha 1D [Source:HGNC Symbol;Acc:HGNC:280] | 16099 | 0.023 | -0.1610 | No |
| 51 | PDE1A | phosphodiesterase 1A [Source:HGNC Symbol;Acc:HGNC:8774] | 16561 | 0.020 | -0.1715 | No |
| 52 | TBXA2R | thromboxane A2 receptor [Source:HGNC Symbol;Acc:HGNC:11608] | 16620 | 0.020 | -0.1719 | No |
| 53 | EDNRA | endothelin receptor type A [Source:HGNC Symbol;Acc:HGNC:3179] | 16969 | 0.018 | -0.1797 | No |
| 54 | SLC8A2 | solute carrier family 8 member A2 [Source:HGNC Symbol;Acc:HGNC:11069] | 17898 | 0.013 | -0.2026 | No |
| 55 | P2RX3 | purinergic receptor P2X 3 [Source:HGNC Symbol;Acc:HGNC:8534] | 18177 | 0.011 | -0.2091 | No |
| 56 | ATP2A1 | ATPase sarcoplasmic/endoplasmic reticulum Ca2+ transporting 1 [Source:HGNC Symbol;Acc:HGNC:811] | 18670 | 0.008 | -0.2211 | No |
| 57 | NTSR1 | neurotensin receptor 1 [Source:HGNC Symbol;Acc:HGNC:8039] | 18697 | 0.008 | -0.2213 | No |
| 58 | PLCE1 | phospholipase C epsilon 1 [Source:HGNC Symbol;Acc:HGNC:17175] | 19244 | 0.005 | -0.2350 | No |
| 59 | HTR5A | 5-hydroxytryptamine receptor 5A [Source:HGNC Symbol;Acc:HGNC:5300] | 19365 | 0.004 | -0.2378 | No |
| 60 | ADORA2A | adenosine A2a receptor [Source:HGNC Symbol;Acc:HGNC:263] | 19525 | 0.003 | -0.2416 | No |
| 61 | HTR7 | 5-hydroxytryptamine receptor 7 [Source:HGNC Symbol;Acc:HGNC:5302] | 19671 | 0.003 | -0.2452 | No |
| 62 | P2RX6 | purinergic receptor P2X 6 [Source:HGNC Symbol;Acc:HGNC:8538] | 19684 | 0.003 | -0.2453 | No |
| 63 | ATP2B3 | ATPase plasma membrane Ca2+ transporting 3 [Source:HGNC Symbol;Acc:HGNC:816] | 19755 | 0.002 | -0.2470 | No |
| 64 | TRHR | thyrotropin releasing hormone receptor [Source:HGNC Symbol;Acc:HGNC:12299] | 20367 | -0.002 | -0.2625 | No |
| 65 | CALML6 | calmodulin like 6 [Source:HGNC Symbol;Acc:HGNC:24193] | 20499 | -0.002 | -0.2657 | No |
| 66 | PPID | peptidylprolyl isomerase D [Source:HGNC Symbol;Acc:HGNC:9257] | 20517 | -0.002 | -0.2660 | No |
| 67 | GNA14 | G protein subunit alpha 14 [Source:HGNC Symbol;Acc:HGNC:4382] | 20806 | -0.004 | -0.2731 | No |
| 68 | HTR2B | 5-hydroxytryptamine receptor 2B [Source:HGNC Symbol;Acc:HGNC:5294] | 20981 | -0.005 | -0.2773 | No |
| 69 | PLN | phospholamban [Source:HGNC Symbol;Acc:HGNC:9080] | 21301 | -0.007 | -0.2850 | No |
| 70 | PRKACA | protein kinase cAMP-activated catalytic subunit alpha [Source:HGNC Symbol;Acc:HGNC:9380] | 21332 | -0.007 | -0.2853 | No |
| 71 | GNA15 | G protein subunit alpha 15 [Source:HGNC Symbol;Acc:HGNC:4383] | 21430 | -0.008 | -0.2874 | No |
| 72 | VDAC2 | voltage dependent anion channel 2 [Source:HGNC Symbol;Acc:HGNC:12672] | 21450 | -0.008 | -0.2874 | No |
| 73 | CACNA1C | calcium voltage-gated channel subunit alpha1 C [Source:HGNC Symbol;Acc:HGNC:1390] | 21681 | -0.009 | -0.2927 | No |
| 74 | CALM1 | calmodulin 1 [Source:HGNC Symbol;Acc:HGNC:1442] | 21884 | -0.010 | -0.2972 | No |
| 75 | GRIN2A | glutamate ionotropic receptor NMDA type subunit 2A [Source:HGNC Symbol;Acc:HGNC:4585] | 21887 | -0.010 | -0.2967 | No |
| 76 | AVPR1B | arginine vasopressin receptor 1B [Source:HGNC Symbol;Acc:HGNC:896] | 21931 | -0.011 | -0.2971 | No |
| 77 | HTR2C | 5-hydroxytryptamine receptor 2C [Source:HGNC Symbol;Acc:HGNC:5295] | 22469 | -0.014 | -0.3100 | No |
| 78 | PPP3R1 | "protein phosphatase 3 regulatory subunit B, alpha [Source:HGNC Symbol;Acc:HGNC:9317]" | 22525 | -0.015 | -0.3106 | No |
| 79 | NOS2 | nitric oxide synthase 2 [Source:HGNC Symbol;Acc:HGNC:7873] | 22937 | -0.017 | -0.3200 | No |
| 80 | HTR6 | 5-hydroxytryptamine receptor 6 [Source:HGNC Symbol;Acc:HGNC:5301] | 23751 | -0.022 | -0.3395 | No |
| 81 | MYLK | myosin light chain kinase [Source:HGNC Symbol;Acc:HGNC:7590] | 23932 | -0.023 | -0.3427 | No |
| 82 | ATP2A3 | ATPase sarcoplasmic/endoplasmic reticulum Ca2+ transporting 3 [Source:HGNC Symbol;Acc:HGNC:813] | 24272 | -0.026 | -0.3499 | No |
| 83 | HRH2 | histamine receptor H2 [Source:HGNC Symbol;Acc:HGNC:5183] | 24462 | -0.027 | -0.3531 | No |
| 84 | CYSLTR1 | cysteinyl leukotriene receptor 1 [Source:HGNC Symbol;Acc:HGNC:17451] | 24477 | -0.027 | -0.3518 | No |
| 85 | LHCGR | luteinizing hormone/choriogonadotropin receptor [Source:HGNC Symbol;Acc:HGNC:6585] | 24877 | -0.030 | -0.3602 | No |
| 86 | EDNRB | endothelin receptor type B [Source:HGNC Symbol;Acc:HGNC:3180] | 25161 | -0.032 | -0.3655 | No |
| 87 | P2RX4 | purinergic receptor P2X 4 [Source:HGNC Symbol;Acc:HGNC:8535] | 25205 | -0.033 | -0.3647 | No |
| 88 | HTR4 | 5-hydroxytryptamine receptor 4 [Source:HGNC Symbol;Acc:HGNC:5299] | 25429 | -0.034 | -0.3684 | No |
| 89 | CACNA1S | calcium voltage-gated channel subunit alpha1 S [Source:HGNC Symbol;Acc:HGNC:1397] | 25589 | -0.035 | -0.3703 | No |
| 90 | OXTR | oxytocin receptor [Source:HGNC Symbol;Acc:HGNC:8529] | 25650 | -0.036 | -0.3698 | No |
| 91 | ADCY1 | adenylate cyclase 1 [Source:HGNC Symbol;Acc:HGNC:232] | 25830 | -0.037 | -0.3721 | No |
| 92 | VDAC3 | voltage dependent anion channel 3 [Source:HGNC Symbol;Acc:HGNC:12674] | 25866 | -0.037 | -0.3708 | No |
| 93 | PRKACB | protein kinase cAMP-activated catalytic subunit beta [Source:HGNC Symbol;Acc:HGNC:9381] | 26088 | -0.039 | -0.3742 | No |
| 94 | CACNA1H | calcium voltage-gated channel subunit alpha1 H [Source:HGNC Symbol;Acc:HGNC:1395] | 26141 | -0.039 | -0.3732 | No |
| 95 | VDAC2P5 | VDAC2 pseudogene 5 [Source:HGNC Symbol;Acc:HGNC:54753] | 26724 | -0.043 | -0.3855 | No |
| 96 | GRM5 | glutamate metabotropic receptor 5 [Source:HGNC Symbol;Acc:HGNC:4597] | 27139 | -0.047 | -0.3933 | No |
| 97 | P2RX1 | purinergic receptor P2X 1 [Source:HGNC Symbol;Acc:HGNC:8533] | 27587 | -0.050 | -0.4018 | No |
| 98 | PLCB1 | phospholipase C beta 1 [Source:HGNC Symbol;Acc:HGNC:15917] | 27781 | -0.052 | -0.4036 | No |
| 99 | SLC8A3 | solute carrier family 8 member A3 [Source:HGNC Symbol;Acc:HGNC:11070] | 27882 | -0.052 | -0.4031 | No |
| 100 | TACR1 | tachykinin receptor 1 [Source:HGNC Symbol;Acc:HGNC:11526] | 27911 | -0.053 | -0.4007 | No |
| 101 | PDE1B | phosphodiesterase 1B [Source:HGNC Symbol;Acc:HGNC:8775] | 27913 | -0.053 | -0.3976 | No |
| 102 | ADCY2 | adenylate cyclase 2 [Source:HGNC Symbol;Acc:HGNC:233] | 27981 | -0.053 | -0.3961 | No |
| 103 | GNA11 | G protein subunit alpha 11 [Source:HGNC Symbol;Acc:HGNC:4379] | 28013 | -0.054 | -0.3938 | No |
| 104 | CHRM2 | cholinergic receptor muscarinic 2 [Source:HGNC Symbol;Acc:HGNC:1951] | 28670 | -0.059 | -0.4070 | No |
| 105 | SPHK1 | sphingosine kinase 1 [Source:HGNC Symbol;Acc:HGNC:11240] | 28774 | -0.059 | -0.4061 | No |
| 106 | PRKACG | protein kinase cAMP-activated catalytic subunit gamma [Source:HGNC Symbol;Acc:HGNC:9382] | 28777 | -0.059 | -0.4027 | No |
| 107 | ATP2B2 | ATPase plasma membrane Ca2+ transporting 2 [Source:HGNC Symbol;Acc:HGNC:815] | 29306 | -0.064 | -0.4124 | No |
| 108 | P2RX2 | purinergic receptor P2X 2 [Source:HGNC Symbol;Acc:HGNC:15459] | 29406 | -0.065 | -0.4110 | No |
| 109 | ITPR3 | "inositol 1,4,5-trisphosphate receptor type 3 [Source:HGNC Symbol;Acc:HGNC:6182]" | 29471 | -0.066 | -0.4088 | No |
| 110 | DRD1 | dopamine receptor D1 [Source:HGNC Symbol;Acc:HGNC:3020] | 29908 | -0.070 | -0.4158 | No |
| 111 | PDE1C | phosphodiesterase 1C [Source:HGNC Symbol;Acc:HGNC:8776] | 30211 | -0.073 | -0.4192 | No |
| 112 | CACNA1G | calcium voltage-gated channel subunit alpha1 G [Source:HGNC Symbol;Acc:HGNC:1394] | 30317 | -0.074 | -0.4175 | No |
| 113 | PDGFRA | platelet derived growth factor receptor alpha [Source:HGNC Symbol;Acc:HGNC:8803] | 30377 | -0.074 | -0.4146 | No |
| 114 | CCKBR | cholecystokinin B receptor [Source:HGNC Symbol;Acc:HGNC:1571] | 30725 | -0.077 | -0.4188 | No |
| 115 | PPP3CC | protein phosphatase 3 catalytic subunit gamma [Source:HGNC Symbol;Acc:HGNC:9316] | 30963 | -0.080 | -0.4202 | No |
| 116 | TRPC1 | transient receptor potential cation channel subfamily C member 1 [Source:HGNC Symbol;Acc:HGNC:12333] | 31523 | -0.086 | -0.4294 | No |
| 117 | LTB4R2 | leukotriene B4 receptor 2 [Source:HGNC Symbol;Acc:HGNC:19260] | 31909 | -0.090 | -0.4339 | Yes |
| 118 | PHKB | phosphorylase kinase regulatory subunit beta [Source:HGNC Symbol;Acc:HGNC:8927] | 31973 | -0.091 | -0.4301 | Yes |
| 119 | PLCD4 | phospholipase C delta 4 [Source:HGNC Symbol;Acc:HGNC:9062] | 31989 | -0.091 | -0.4251 | Yes |
| 120 | BST1 | bone marrow stromal cell antigen 1 [Source:HGNC Symbol;Acc:HGNC:1118] | 32035 | -0.091 | -0.4209 | Yes |
| 121 | EGFR | epidermal growth factor receptor [Source:HGNC Symbol;Acc:HGNC:3236] | 32133 | -0.092 | -0.4179 | Yes |
| 122 | PDGFRB | platelet derived growth factor receptor beta [Source:HGNC Symbol;Acc:HGNC:8804] | 32165 | -0.093 | -0.4132 | Yes |
| 123 | CAMK4 | calcium/calmodulin dependent protein kinase IV [Source:HGNC Symbol;Acc:HGNC:1464] | 32292 | -0.094 | -0.4108 | Yes |
| 124 | PLCB2 | phospholipase C beta 2 [Source:HGNC Symbol;Acc:HGNC:9055] | 32369 | -0.095 | -0.4071 | Yes |
| 125 | RYR1 | ryanodine receptor 1 [Source:HGNC Symbol;Acc:HGNC:10483] | 32462 | -0.096 | -0.4038 | Yes |
| 126 | CCKAR | cholecystokinin A receptor [Source:HGNC Symbol;Acc:HGNC:1570] | 32762 | -0.100 | -0.4055 | Yes |
| 127 | CHRM1 | cholinergic receptor muscarinic 1 [Source:HGNC Symbol;Acc:HGNC:1950] | 32818 | -0.101 | -0.4009 | Yes |
| 128 | PTGFR | prostaglandin F receptor [Source:HGNC Symbol;Acc:HGNC:9600] | 32894 | -0.102 | -0.3968 | Yes |
| 129 | TACR2 | tachykinin receptor 2 [Source:HGNC Symbol;Acc:HGNC:11527] | 32918 | -0.102 | -0.3913 | Yes |
| 130 | PLCZ1 | phospholipase C zeta 1 [Source:HGNC Symbol;Acc:HGNC:19218] | 33095 | -0.105 | -0.3896 | Yes |
| 131 | PHKA2 | phosphorylase kinase regulatory subunit alpha 2 [Source:HGNC Symbol;Acc:HGNC:8926] | 33175 | -0.106 | -0.3853 | Yes |
| 132 | CALML3 | calmodulin like 3 [Source:HGNC Symbol;Acc:HGNC:1452] | 33436 | -0.110 | -0.3855 | Yes |
| 133 | GNAL | G protein subunit alpha L [Source:HGNC Symbol;Acc:HGNC:4388] | 33855 | -0.116 | -0.3893 | Yes |
| 134 | ITPR2 | "inositol 1,4,5-trisphosphate receptor type 2 [Source:HGNC Symbol;Acc:HGNC:6181]" | 34058 | -0.119 | -0.3874 | Yes |
| 135 | HTR2A | 5-hydroxytryptamine receptor 2A [Source:HGNC Symbol;Acc:HGNC:5293] | 34078 | -0.119 | -0.3808 | Yes |
| 136 | ADRA1A | adrenoceptor alpha 1A [Source:HGNC Symbol;Acc:HGNC:277] | 34203 | -0.121 | -0.3768 | Yes |
| 137 | PTK2B | protein tyrosine kinase 2 beta [Source:HGNC Symbol;Acc:HGNC:9612] | 34307 | -0.122 | -0.3722 | Yes |
| 138 | CACNA1F | calcium voltage-gated channel subunit alpha1 F [Source:HGNC Symbol;Acc:HGNC:1393] | 34492 | -0.126 | -0.3695 | Yes |
| 139 | CHP2 | calcineurin like EF-hand protein 2 [Source:HGNC Symbol;Acc:HGNC:24927] | 34531 | -0.126 | -0.3630 | Yes |
| 140 | PRKCB | protein kinase C beta [Source:HGNC Symbol;Acc:HGNC:9395] | 34603 | -0.128 | -0.3572 | Yes |
| 141 | CHRM5 | cholinergic receptor muscarinic 5 [Source:HGNC Symbol;Acc:HGNC:1954] | 34629 | -0.128 | -0.3503 | Yes |
| 142 | ADCY7 | adenylate cyclase 7 [Source:HGNC Symbol;Acc:HGNC:238] | 34661 | -0.129 | -0.3434 | Yes |
| 143 | RYR3 | ryanodine receptor 3 [Source:HGNC Symbol;Acc:HGNC:10485] | 34793 | -0.131 | -0.3390 | Yes |
| 144 | ADCY4 | adenylate cyclase 4 [Source:HGNC Symbol;Acc:HGNC:235] | 34840 | -0.132 | -0.3324 | Yes |
| 145 | PTGER3 | prostaglandin E receptor 3 [Source:HGNC Symbol;Acc:HGNC:9595] | 34952 | -0.134 | -0.3272 | Yes |
| 146 | SLC8A1 | solute carrier family 8 member A1 [Source:HGNC Symbol;Acc:HGNC:11068] | 34954 | -0.135 | -0.3193 | Yes |
| 147 | F2R | coagulation factor II thrombin receptor [Source:HGNC Symbol;Acc:HGNC:3537] | 35000 | -0.135 | -0.3124 | Yes |
| 148 | PRKCA | protein kinase C alpha [Source:HGNC Symbol;Acc:HGNC:9393] | 35223 | -0.140 | -0.3098 | Yes |
| 149 | CD38 | CD38 molecule [Source:HGNC Symbol;Acc:HGNC:1667] | 35362 | -0.143 | -0.3048 | Yes |
| 150 | ADCY9 | adenylate cyclase 9 [Source:HGNC Symbol;Acc:HGNC:240] | 35385 | -0.144 | -0.2968 | Yes |
| 151 | NOS3 | nitric oxide synthase 3 [Source:HGNC Symbol;Acc:HGNC:7876] | 35403 | -0.144 | -0.2887 | Yes |
| 152 | CHRNA7 | cholinergic receptor nicotinic alpha 7 subunit [Source:HGNC Symbol;Acc:HGNC:1960] | 35561 | -0.148 | -0.2839 | Yes |
| 153 | PLCG2 | phospholipase C gamma 2 [Source:HGNC Symbol;Acc:HGNC:9066] | 35650 | -0.151 | -0.2772 | Yes |
| 154 | CHRM3 | cholinergic receptor muscarinic 3 [Source:HGNC Symbol;Acc:HGNC:1952] | 35766 | -0.154 | -0.2710 | Yes |
| 155 | ATP2A2 | ATPase sarcoplasmic/endoplasmic reticulum Ca2+ transporting 2 [Source:HGNC Symbol;Acc:HGNC:812] | 35828 | -0.156 | -0.2634 | Yes |
| 156 | P2RX7 | purinergic receptor P2X 7 [Source:HGNC Symbol;Acc:HGNC:8537] | 35972 | -0.159 | -0.2576 | Yes |
| 157 | P2RX5 | purinergic receptor P2X 5 [Source:HGNC Symbol;Acc:HGNC:8536] | 36186 | -0.166 | -0.2532 | Yes |
| 158 | ITPR1 | "inositol 1,4,5-trisphosphate receptor type 1 [Source:HGNC Symbol;Acc:HGNC:6180]" | 36283 | -0.169 | -0.2456 | Yes |
| 159 | GNAQ | G protein subunit alpha q [Source:HGNC Symbol;Acc:HGNC:4390] | 36400 | -0.173 | -0.2383 | Yes |
| 160 | PHKA1 | phosphorylase kinase regulatory subunit alpha 1 [Source:HGNC Symbol;Acc:HGNC:8925] | 36534 | -0.178 | -0.2312 | Yes |
| 161 | ATP2B4 | ATPase plasma membrane Ca2+ transporting 4 [Source:HGNC Symbol;Acc:HGNC:817] | 36609 | -0.180 | -0.2224 | Yes |
| 162 | GRPR | gastrin releasing peptide receptor [Source:HGNC Symbol;Acc:HGNC:4609] | 36990 | -0.197 | -0.2204 | Yes |
| 163 | CACNA1E | calcium voltage-gated channel subunit alpha1 E [Source:HGNC Symbol;Acc:HGNC:1392] | 37145 | -0.205 | -0.2122 | Yes |
| 164 | RYR2 | ryanodine receptor 2 [Source:HGNC Symbol;Acc:HGNC:10484] | 37202 | -0.207 | -0.2013 | Yes |
| 165 | CACNA1I | calcium voltage-gated channel subunit alpha1 I [Source:HGNC Symbol;Acc:HGNC:1396] | 37273 | -0.211 | -0.1906 | Yes |
| 166 | AVPR1A | arginine vasopressin receptor 1A [Source:HGNC Symbol;Acc:HGNC:895] | 37496 | -0.223 | -0.1831 | Yes |
| 167 | ITPKA | inositol-trisphosphate 3-kinase A [Source:HGNC Symbol;Acc:HGNC:6178] | 37678 | -0.236 | -0.1737 | Yes |
| 168 | CYSLTR2 | cysteinyl leukotriene receptor 2 [Source:HGNC Symbol;Acc:HGNC:18274] | 37909 | -0.255 | -0.1644 | Yes |
| 169 | PLCD1 | phospholipase C delta 1 [Source:HGNC Symbol;Acc:HGNC:9060] | 38013 | -0.267 | -0.1513 | Yes |
| 170 | NOS1 | nitric oxide synthase 1 [Source:HGNC Symbol;Acc:HGNC:7872] | 38042 | -0.270 | -0.1360 | Yes |
| 171 | CACNA1B | calcium voltage-gated channel subunit alpha1 B [Source:HGNC Symbol;Acc:HGNC:1389] | 38121 | -0.281 | -0.1214 | Yes |
| 172 | ADCY8 | adenylate cyclase 8 [Source:HGNC Symbol;Acc:HGNC:239] | 38268 | -0.302 | -0.1072 | Yes |
| 173 | PLCB4 | phospholipase C beta 4 [Source:HGNC Symbol;Acc:HGNC:9059] | 38308 | -0.309 | -0.0898 | Yes |
| 174 | ADCY3 | adenylate cyclase 3 [Source:HGNC Symbol;Acc:HGNC:234] | 38423 | -0.337 | -0.0728 | Yes |
| 175 | GRM1 | glutamate metabotropic receptor 1 [Source:HGNC Symbol;Acc:HGNC:4593] | 38453 | -0.351 | -0.0527 | Yes |
| 176 | PRKX | protein kinase X-linked [Source:HGNC Symbol;Acc:HGNC:9441] | 38524 | -0.395 | -0.0310 | Yes |
| 177 | ITPKB | inositol-trisphosphate 3-kinase B [Source:HGNC Symbol;Acc:HGNC:6179] | 38542 | -0.426 | -0.0062 | Yes |
| 178 | ATP2B1 | ATPase plasma membrane Ca2+ transporting 1 [Source:HGNC Symbol;Acc:HGNC:814] | 38547 | -0.441 | 0.0198 | Yes |
Table: GSEA details [plain text format]

  

Fig 2: KEGG\_CALCIUM\_SIGNALING\_PATHWAY      
 Blue-Pink O' Gram in the Space of the Analyzed GeneSet

  

Fig 3: KEGG\_CALCIUM\_SIGNALING\_PATHWAY: Random ES distribution      
 Gene set null distribution of ES for **KEGG\_CALCIUM\_SIGNALING\_PATHWAY**
