# Supplementary material for: Simultaneous Presentation of Multiple Myeloma and Lung Cancer: Case Report and Gene Bioinformatics Analysis
Source: Front Oncol. 2022 Jun 13;12:859735. doi: 10.3389/fonc.2022.859735 (PMC9235397; doi:10.3389/fonc.2022.859735)
Supplement: Supplementary file 1 [file DataSheet_1.zip › The bioinformatic analysis of MM and lung cancer supplementary materials/Enrichment analysis/MECR/GSEA_4.1.0/LUSC TCGA/KEGG.Gsea.1639042374131/KEGG_CELL_ADHESION_MOLECULES_CAMS.html]

Details for gene set KEGG\_CELL\_ADHESION\_MOLECULES\_CAMS[GSEA]

|  || Dataset | ExpData\_collapsed\_to\_symbols.ENSG00000116353\_profile\_in\_ExpData.cls #ENSG00000116353 |
| Phenotype | ENSG00000116353\_profile\_in\_ExpData.cls#ENSG00000116353 |
| Upregulated in class | ENSG00000116353\_neg |
| GeneSet | KEGG\_CELL\_ADHESION\_MOLECULES\_CAMS |
| Enrichment Score (ES) | -0.4495109 |
| Normalized Enrichment Score (NES) | -2.0142512 |
| Nominal p-value | 0.0 |
| FDR q-value | 0.0027784372 |
| FWER p-Value | 0.009 |
Table: GSEA Results Summary

  

Fig 1: Enrichment plot: KEGG\_CELL\_ADHESION\_MOLECULES\_CAMS      
 Profile of the Running ES Score & Positions of GeneSet Members on the Rank Ordered List

  

| SYMBOL | TITLE | RANK IN GENE LIST | RANK METRIC SCORE | RUNNING ES | CORE ENRICHMENT || 1 | CLDN9 | claudin 9 [Source:HGNC Symbol;Acc:HGNC:2051] | 347 | 0.296 | 0.0120 | No |
| 2 | SDC4 | syndecan 4 [Source:HGNC Symbol;Acc:HGNC:10661] | 360 | 0.293 | 0.0322 | No |
| 3 | CDH15 | cadherin 15 [Source:HGNC Symbol;Acc:HGNC:1754] | 1082 | 0.210 | 0.0286 | No |
| 4 | CLDN10 | claudin 10 [Source:HGNC Symbol;Acc:HGNC:2033] | 1329 | 0.195 | 0.0361 | No |
| 5 | PTPRF | protein tyrosine phosphatase receptor type F [Source:HGNC Symbol;Acc:HGNC:9670] | 1835 | 0.172 | 0.0353 | No |
| 6 | CLDN7 | claudin 7 [Source:HGNC Symbol;Acc:HGNC:2049] | 1907 | 0.169 | 0.0453 | No |
| 7 | PVR | PVR cell adhesion molecule [Source:HGNC Symbol;Acc:HGNC:9705] | 2632 | 0.146 | 0.0371 | No |
| 8 | CLDN19 | claudin 19 [Source:HGNC Symbol;Acc:HGNC:2040] | 2806 | 0.141 | 0.0426 | No |
| 9 | CDH3 | cadherin 3 [Source:HGNC Symbol;Acc:HGNC:1762] | 2926 | 0.139 | 0.0494 | No |
| 10 | SDC3 | syndecan 3 [Source:HGNC Symbol;Acc:HGNC:10660] | 3243 | 0.132 | 0.0505 | No |
| 11 | L1CAM | L1 cell adhesion molecule [Source:HGNC Symbol;Acc:HGNC:6470] | 3329 | 0.130 | 0.0575 | No |
| 12 | HLA-G | "major histocompatibility complex, class I, G [Source:HGNC Symbol;Acc:HGNC:4964]" | 3433 | 0.127 | 0.0638 | No |
| 13 | CLDN3 | claudin 3 [Source:HGNC Symbol;Acc:HGNC:2045] | 4157 | 0.115 | 0.0534 | No |
| 14 | ITGB1 | integrin subunit beta 1 [Source:HGNC Symbol;Acc:HGNC:6153] | 4161 | 0.115 | 0.0614 | No |
| 15 | CLDN6 | claudin 6 [Source:HGNC Symbol;Acc:HGNC:2048] | 4315 | 0.113 | 0.0655 | No |
| 16 | NECTIN2 | nectin cell adhesion molecule 2 [Source:HGNC Symbol;Acc:HGNC:9707] | 5526 | 0.096 | 0.0414 | No |
| 17 | CLDN16 | claudin 16 [Source:HGNC Symbol;Acc:HGNC:2037] | 5838 | 0.096 | 0.0402 | No |
| 18 | CLDN1 | claudin 1 [Source:HGNC Symbol;Acc:HGNC:2032] | 5878 | 0.095 | 0.0459 | No |
| 19 | CLDN14 | claudin 14 [Source:HGNC Symbol;Acc:HGNC:2035] | 5936 | 0.095 | 0.0511 | No |
| 20 | MAG | myelin associated glycoprotein [Source:HGNC Symbol;Acc:HGNC:6783] | 8896 | 0.068 | -0.0196 | No |
| 21 | CD40 | CD40 molecule [Source:HGNC Symbol;Acc:HGNC:11919] | 10160 | 0.059 | -0.0477 | No |
| 22 | ICAM1 | intercellular adhesion molecule 1 [Source:HGNC Symbol;Acc:HGNC:5344] | 10261 | 0.058 | -0.0462 | No |
| 23 | HLA-A | "major histocompatibility complex, class I, A [Source:HGNC Symbol;Acc:HGNC:4931]" | 10414 | 0.057 | -0.0460 | No |
| 24 | CLDN23 | claudin 23 [Source:HGNC Symbol;Acc:HGNC:17591] | 10786 | 0.055 | -0.0516 | No |
| 25 | CD99 | CD99 molecule (Xg blood group) [Source:HGNC Symbol;Acc:HGNC:7082] | 11478 | 0.051 | -0.0657 | No |
| 26 | CD58 | CD58 molecule [Source:HGNC Symbol;Acc:HGNC:1688] | 11882 | 0.048 | -0.0726 | No |
| 27 | HLA-C | "major histocompatibility complex, class I, C [Source:HGNC Symbol;Acc:HGNC:4933]" | 12052 | 0.047 | -0.0736 | No |
| 28 | HLA-E | "major histocompatibility complex, class I, E [Source:HGNC Symbol;Acc:HGNC:4962]" | 13160 | 0.040 | -0.0991 | No |
| 29 | NECTIN3 | nectin cell adhesion molecule 3 [Source:HGNC Symbol;Acc:HGNC:17664] | 14244 | 0.033 | -0.1244 | No |
| 30 | HLA-F | "major histocompatibility complex, class I, F [Source:HGNC Symbol;Acc:HGNC:4963]" | 15549 | 0.026 | -0.1558 | No |
| 31 | CLDN4 | claudin 4 [Source:HGNC Symbol;Acc:HGNC:2046] | 16544 | 0.020 | -0.1798 | No |
| 32 | CDH4 | cadherin 4 [Source:HGNC Symbol;Acc:HGNC:1763] | 17956 | 0.012 | -0.2149 | No |
| 33 | CADM3 | cell adhesion molecule 3 [Source:HGNC Symbol;Acc:HGNC:17601] | 18888 | 0.007 | -0.2381 | No |
| 34 | HLA-B | "major histocompatibility complex, class I, B [Source:HGNC Symbol;Acc:HGNC:4932]" | 18889 | 0.007 | -0.2376 | No |
| 35 | CDH2 | cadherin 2 [Source:HGNC Symbol;Acc:HGNC:1759] | 19131 | 0.006 | -0.2434 | No |
| 36 | CLDN11 | claudin 11 [Source:HGNC Symbol;Acc:HGNC:8514] | 20125 | -0.000 | -0.2687 | No |
| 37 | OCLN | occludin [Source:HGNC Symbol;Acc:HGNC:8104] | 20779 | -0.004 | -0.2851 | No |
| 38 | CD8B | CD8b molecule [Source:HGNC Symbol;Acc:HGNC:1707] | 22034 | -0.011 | -0.3163 | No |
| 39 | CLDN2 | claudin 2 [Source:HGNC Symbol;Acc:HGNC:2041] | 22628 | -0.015 | -0.3303 | No |
| 40 | HLA-DMA | "major histocompatibility complex, class II, DM alpha [Source:HGNC Symbol;Acc:HGNC:4934]" | 22645 | -0.015 | -0.3297 | No |
| 41 | MPZ | myelin protein zero [Source:HGNC Symbol;Acc:HGNC:7225] | 22684 | -0.015 | -0.3296 | No |
| 42 | MPZL1 | myelin protein zero like 1 [Source:HGNC Symbol;Acc:HGNC:7226] | 23849 | -0.023 | -0.3577 | No |
| 43 | ICAM2 | intercellular adhesion molecule 2 [Source:HGNC Symbol;Acc:HGNC:5345] | 24161 | -0.025 | -0.3639 | No |
| 44 | CADM1 | cell adhesion molecule 1 [Source:HGNC Symbol;Acc:HGNC:5951] | 25245 | -0.033 | -0.3892 | No |
| 45 | NCAM2 | neural cell adhesion molecule 2 [Source:HGNC Symbol;Acc:HGNC:7657] | 25454 | -0.034 | -0.3921 | No |
| 46 | HLA-DRA | "major histocompatibility complex, class II, DR alpha [Source:HGNC Symbol;Acc:HGNC:4947]" | 26550 | -0.042 | -0.4171 | No |
| 47 | HLA-DRB1 | "major histocompatibility complex, class II, DR beta 1 [Source:HGNC Symbol;Acc:HGNC:4948]" | 26773 | -0.044 | -0.4196 | No |
| 48 | ESAM | endothelial cell adhesion molecule [Source:HGNC Symbol;Acc:HGNC:17474] | 27473 | -0.049 | -0.4340 | No |
| 49 | PTPRM | protein tyrosine phosphatase receptor type M [Source:HGNC Symbol;Acc:HGNC:9675] | 27539 | -0.050 | -0.4322 | No |
| 50 | JAM3 | junctional adhesion molecule 3 [Source:HGNC Symbol;Acc:HGNC:15532] | 27652 | -0.050 | -0.4315 | No |
| 51 | HLA-DQA2 | "major histocompatibility complex, class II, DQ alpha 2 [Source:HGNC Symbol;Acc:HGNC:4943]" | 27660 | -0.051 | -0.4281 | No |
| 52 | HLA-DQB1 | "major histocompatibility complex, class II, DQ beta 1 [Source:HGNC Symbol;Acc:HGNC:4944]" | 27794 | -0.052 | -0.4279 | No |
| 53 | HLA-DPB1 | "major histocompatibility complex, class II, DP beta 1 [Source:HGNC Symbol;Acc:HGNC:4940]" | 28094 | -0.054 | -0.4317 | No |
| 54 | CD86 | CD86 molecule [Source:HGNC Symbol;Acc:HGNC:1705] | 28574 | -0.058 | -0.4399 | No |
| 55 | CLDN18 | claudin 18 [Source:HGNC Symbol;Acc:HGNC:2039] | 28696 | -0.059 | -0.4388 | No |
| 56 | HLA-DPA1 | "major histocompatibility complex, class II, DP alpha 1 [Source:HGNC Symbol;Acc:HGNC:4938]" | 29080 | -0.062 | -0.4442 | No |
| 57 | NEGR1 | neuronal growth regulator 1 [Source:HGNC Symbol;Acc:HGNC:17302] | 29189 | -0.063 | -0.4425 | No |
| 58 | ICAM3 | intercellular adhesion molecule 3 [Source:HGNC Symbol;Acc:HGNC:5346] | 29248 | -0.064 | -0.4395 | No |
| 59 | HLA-DMB | "major histocompatibility complex, class II, DM beta [Source:HGNC Symbol;Acc:HGNC:4935]" | 29388 | -0.065 | -0.4385 | No |
| 60 | JAM2 | junctional adhesion molecule 2 [Source:HGNC Symbol;Acc:HGNC:14686] | 29521 | -0.066 | -0.4372 | No |
| 61 | HLA-DRB5 | "major histocompatibility complex, class II, DR beta 5 [Source:HGNC Symbol;Acc:HGNC:4953]" | 29919 | -0.070 | -0.4424 | No |
| 62 | CLDN5 | claudin 5 [Source:HGNC Symbol;Acc:HGNC:2047] | 30197 | -0.073 | -0.4444 | Yes |
| 63 | ICOSLG | inducible T cell costimulator ligand [Source:HGNC Symbol;Acc:HGNC:17087] | 30237 | -0.073 | -0.4403 | Yes |
| 64 | HLA-DOA | "major histocompatibility complex, class II, DO alpha [Source:HGNC Symbol;Acc:HGNC:4936]" | 30255 | -0.073 | -0.4356 | Yes |
| 65 | CLDN17 | claudin 17 [Source:HGNC Symbol;Acc:HGNC:2038] | 30477 | -0.075 | -0.4359 | Yes |
| 66 | ITGA8 | integrin subunit alpha 8 [Source:HGNC Symbol;Acc:HGNC:6144] | 30487 | -0.075 | -0.4309 | Yes |
| 67 | CD4 | CD4 molecule [Source:HGNC Symbol;Acc:HGNC:1678] | 30573 | -0.076 | -0.4277 | Yes |
| 68 | ITGB7 | integrin subunit beta 7 [Source:HGNC Symbol;Acc:HGNC:6162] | 31210 | -0.082 | -0.4381 | Yes |
| 69 | PDCD1 | programmed cell death 1 [Source:HGNC Symbol;Acc:HGNC:8760] | 31593 | -0.086 | -0.4418 | Yes |
| 70 | CD276 | CD276 molecule [Source:HGNC Symbol;Acc:HGNC:19137] | 31594 | -0.086 | -0.4357 | Yes |
| 71 | CD6 | CD6 molecule [Source:HGNC Symbol;Acc:HGNC:1691] | 31658 | -0.087 | -0.4312 | Yes |
| 72 | CDH5 | cadherin 5 [Source:HGNC Symbol;Acc:HGNC:1764] | 31980 | -0.091 | -0.4331 | Yes |
| 73 | SDC1 | syndecan 1 [Source:HGNC Symbol;Acc:HGNC:10658] | 32197 | -0.093 | -0.4320 | Yes |
| 74 | NLGN2 | neuroligin 2 [Source:HGNC Symbol;Acc:HGNC:14290] | 32404 | -0.096 | -0.4306 | Yes |
| 75 | NECTIN1 | nectin cell adhesion molecule 1 [Source:HGNC Symbol;Acc:HGNC:9706] | 32484 | -0.097 | -0.4258 | Yes |
| 76 | ITGA4 | integrin subunit alpha 4 [Source:HGNC Symbol;Acc:HGNC:6140] | 32537 | -0.097 | -0.4203 | Yes |
| 77 | HLA-DQA1 | "major histocompatibility complex, class II, DQ alpha 1 [Source:HGNC Symbol;Acc:HGNC:4942]" | 32609 | -0.098 | -0.4152 | Yes |
| 78 | SIGLEC1 | sialic acid binding Ig like lectin 1 [Source:HGNC Symbol;Acc:HGNC:11127] | 32848 | -0.101 | -0.4142 | Yes |
| 79 | VCAM1 | vascular cell adhesion molecule 1 [Source:HGNC Symbol;Acc:HGNC:12663] | 33006 | -0.104 | -0.4109 | Yes |
| 80 | SELPLG | selectin P ligand [Source:HGNC Symbol;Acc:HGNC:10722] | 33051 | -0.104 | -0.4047 | Yes |
| 81 | ITGAV | integrin subunit alpha V [Source:HGNC Symbol;Acc:HGNC:6150] | 33146 | -0.105 | -0.3997 | Yes |
| 82 | CD2 | CD2 molecule [Source:HGNC Symbol;Acc:HGNC:1639] | 33236 | -0.107 | -0.3945 | Yes |
| 83 | CLDN15 | claudin 15 [Source:HGNC Symbol;Acc:HGNC:2036] | 33333 | -0.108 | -0.3893 | Yes |
| 84 | CD8A | CD8a molecule [Source:HGNC Symbol;Acc:HGNC:1706] | 33518 | -0.111 | -0.3863 | Yes |
| 85 | ITGA9 | integrin subunit alpha 9 [Source:HGNC Symbol;Acc:HGNC:6145] | 33520 | -0.111 | -0.3785 | Yes |
| 86 | CD80 | CD80 molecule [Source:HGNC Symbol;Acc:HGNC:1700] | 33643 | -0.113 | -0.3737 | Yes |
| 87 | NRXN1 | neurexin 1 [Source:HGNC Symbol;Acc:HGNC:8008] | 33650 | -0.113 | -0.3660 | Yes |
| 88 | ITGB2 | integrin subunit beta 2 [Source:HGNC Symbol;Acc:HGNC:6155] | 33663 | -0.113 | -0.3583 | Yes |
| 89 | ITGAM | integrin subunit alpha M [Source:HGNC Symbol;Acc:HGNC:6149] | 33733 | -0.114 | -0.3521 | Yes |
| 90 | VCAN | versican [Source:HGNC Symbol;Acc:HGNC:2464] | 33834 | -0.116 | -0.3465 | Yes |
| 91 | PECAM1 | platelet and endothelial cell adhesion molecule 1 [Source:HGNC Symbol;Acc:HGNC:8823] | 33863 | -0.116 | -0.3391 | Yes |
| 92 | CD226 | CD226 molecule [Source:HGNC Symbol;Acc:HGNC:16961] | 34015 | -0.118 | -0.3347 | Yes |
| 93 | CDH1 | cadherin 1 [Source:HGNC Symbol;Acc:HGNC:1748] | 34025 | -0.118 | -0.3266 | Yes |
| 94 | CLDN8 | claudin 8 [Source:HGNC Symbol;Acc:HGNC:2050] | 34192 | -0.121 | -0.3224 | Yes |
| 95 | NFASC | neurofascin [Source:HGNC Symbol;Acc:HGNC:29866] | 34272 | -0.122 | -0.3158 | Yes |
| 96 | ITGA6 | integrin subunit alpha 6 [Source:HGNC Symbol;Acc:HGNC:6142] | 34278 | -0.122 | -0.3074 | Yes |
| 97 | CD34 | CD34 molecule [Source:HGNC Symbol;Acc:HGNC:1662] | 34380 | -0.124 | -0.3013 | Yes |
| 98 | CD40LG | CD40 ligand [Source:HGNC Symbol;Acc:HGNC:11935] | 34481 | -0.125 | -0.2950 | Yes |
| 99 | SELL | selectin L [Source:HGNC Symbol;Acc:HGNC:10720] | 34516 | -0.126 | -0.2870 | Yes |
| 100 | HLA-DOB | "major histocompatibility complex, class II, DO beta [Source:HGNC Symbol;Acc:HGNC:4937]" | 34609 | -0.128 | -0.2804 | Yes |
| 101 | GLG1 | golgi glycoprotein 1 [Source:HGNC Symbol;Acc:HGNC:4316] | 34625 | -0.128 | -0.2718 | Yes |
| 102 | NCAM1 | neural cell adhesion molecule 1 [Source:HGNC Symbol;Acc:HGNC:7656] | 34762 | -0.131 | -0.2661 | Yes |
| 103 | CNTN2 | contactin 2 [Source:HGNC Symbol;Acc:HGNC:2172] | 34794 | -0.131 | -0.2576 | Yes |
| 104 | CNTN1 | contactin 1 [Source:HGNC Symbol;Acc:HGNC:2171] | 34815 | -0.132 | -0.2489 | Yes |
| 105 | ICOS | inducible T cell costimulator [Source:HGNC Symbol;Acc:HGNC:5351] | 34857 | -0.132 | -0.2407 | Yes |
| 106 | CD22 | CD22 molecule [Source:HGNC Symbol;Acc:HGNC:1643] | 34875 | -0.133 | -0.2318 | Yes |
| 107 | SPN | sialophorin [Source:HGNC Symbol;Acc:HGNC:11249] | 35152 | -0.139 | -0.2291 | Yes |
| 108 | F11R | F11 receptor [Source:HGNC Symbol;Acc:HGNC:14685] | 35158 | -0.139 | -0.2195 | Yes |
| 109 | ITGB8 | integrin subunit beta 8 [Source:HGNC Symbol;Acc:HGNC:6163] | 35215 | -0.140 | -0.2111 | Yes |
| 110 | CTLA4 | cytotoxic T-lymphocyte associated protein 4 [Source:HGNC Symbol;Acc:HGNC:2505] | 35267 | -0.141 | -0.2025 | Yes |
| 111 | PTPRC | protein tyrosine phosphatase receptor type C [Source:HGNC Symbol;Acc:HGNC:9666] | 35701 | -0.152 | -0.2028 | Yes |
| 112 | MADCAM1 | mucosal vascular addressin cell adhesion molecule 1 [Source:HGNC Symbol;Acc:HGNC:6765] | 35787 | -0.154 | -0.1941 | Yes |
| 113 | SDC2 | syndecan 2 [Source:HGNC Symbol;Acc:HGNC:10659] | 35826 | -0.156 | -0.1842 | Yes |
| 114 | SELP | selectin P [Source:HGNC Symbol;Acc:HGNC:10721] | 36256 | -0.168 | -0.1833 | Yes |
| 115 | ITGAL | integrin subunit alpha L [Source:HGNC Symbol;Acc:HGNC:6148] | 36366 | -0.172 | -0.1740 | Yes |
| 116 | CNTNAP1 | contactin associated protein 1 [Source:HGNC Symbol;Acc:HGNC:8011] | 36478 | -0.176 | -0.1645 | Yes |
| 117 | CD28 | CD28 molecule [Source:HGNC Symbol;Acc:HGNC:1653] | 36550 | -0.178 | -0.1538 | Yes |
| 118 | SELE | selectin E [Source:HGNC Symbol;Acc:HGNC:10718] | 36893 | -0.193 | -0.1489 | Yes |
| 119 | NLGN4X | neuroligin 4 X-linked [Source:HGNC Symbol;Acc:HGNC:14287] | 36905 | -0.193 | -0.1356 | Yes |
| 120 | NRXN3 | neurexin 3 [Source:HGNC Symbol;Acc:HGNC:8010] | 37075 | -0.201 | -0.1258 | Yes |
| 121 | ALCAM | activated leukocyte cell adhesion molecule [Source:HGNC Symbol;Acc:HGNC:400] | 37194 | -0.207 | -0.1143 | Yes |
| 122 | NRXN2 | neurexin 2 [Source:HGNC Symbol;Acc:HGNC:8009] | 37267 | -0.211 | -0.1013 | Yes |
| 123 | NEO1 | neogenin 1 [Source:HGNC Symbol;Acc:HGNC:7754] | 37504 | -0.224 | -0.0916 | Yes |
| 124 | PDCD1LG2 | programmed cell death 1 ligand 2 [Source:HGNC Symbol;Acc:HGNC:18731] | 37588 | -0.231 | -0.0775 | Yes |
| 125 | NLGN1 | neuroligin 1 [Source:HGNC Symbol;Acc:HGNC:14291] | 37607 | -0.232 | -0.0617 | Yes |
| 126 | NLGN3 | neuroligin 3 [Source:HGNC Symbol;Acc:HGNC:14289] | 38079 | -0.273 | -0.0545 | Yes |
| 127 | NRCAM | neuronal cell adhesion molecule [Source:HGNC Symbol;Acc:HGNC:7994] | 38116 | -0.280 | -0.0358 | Yes |
| 128 | CLDN20 | claudin 20 [Source:HGNC Symbol;Acc:HGNC:2042] | 38252 | -0.300 | -0.0182 | Yes |
| 129 | CNTNAP2 | contactin associated protein 2 [Source:HGNC Symbol;Acc:HGNC:13830] | 38328 | -0.313 | 0.0019 | Yes |
| 130 | CD274 | CD274 molecule [Source:HGNC Symbol;Acc:HGNC:17635] | 38379 | -0.324 | 0.0234 | Yes |
| 131 | CLDN22 | claudin 22 [Source:HGNC Symbol;Acc:HGNC:2044] | 39134 | NaN | 0.0048 | Yes |
Table: GSEA details [plain text format]

  

Fig 2: KEGG\_CELL\_ADHESION\_MOLECULES\_CAMS      
 Blue-Pink O' Gram in the Space of the Analyzed GeneSet

  

Fig 3: KEGG\_CELL\_ADHESION\_MOLECULES\_CAMS: Random ES distribution      
 Gene set null distribution of ES for **KEGG\_CELL\_ADHESION\_MOLECULES\_CAMS**
