# Supplementary material for: Simultaneous Presentation of Multiple Myeloma and Lung Cancer: Case Report and Gene Bioinformatics Analysis
Source: Front Oncol. 2022 Jun 13;12:859735. doi: 10.3389/fonc.2022.859735 (PMC9235397; doi:10.3389/fonc.2022.859735)
Supplement: Supplementary file 1 [file DataSheet_1.zip › The bioinformatic analysis of MM and lung cancer supplementary materials/Enrichment analysis/MECR/GSEA_4.1.0/LUSC TCGA/KEGG.Gsea.1639042374131/KEGG_DILATED_CARDIOMYOPATHY.html]

Details for gene set KEGG\_DILATED\_CARDIOMYOPATHY[GSEA]

|  || Dataset | ExpData\_collapsed\_to\_symbols.ENSG00000116353\_profile\_in\_ExpData.cls #ENSG00000116353 |
| Phenotype | ENSG00000116353\_profile\_in\_ExpData.cls#ENSG00000116353 |
| Upregulated in class | ENSG00000116353\_neg |
| GeneSet | KEGG\_DILATED\_CARDIOMYOPATHY |
| Enrichment Score (ES) | -0.41424894 |
| Normalized Enrichment Score (NES) | -1.7245159 |
| Nominal p-value | 0.0 |
| FDR q-value | 0.018923098 |
| FWER p-Value | 0.259 |
Table: GSEA Results Summary

  

Fig 1: Enrichment plot: KEGG\_DILATED\_CARDIOMYOPATHY      
 Profile of the Running ES Score & Positions of GeneSet Members on the Rank Ordered List

  

| SYMBOL | TITLE | RANK IN GENE LIST | RANK METRIC SCORE | RUNNING ES | CORE ENRICHMENT || 1 | ACTB | actin beta [Source:HGNC Symbol;Acc:HGNC:132] | 1566 | 0.183 | -0.0200 | No |
| 2 | TPM2 | tropomyosin 2 [Source:HGNC Symbol;Acc:HGNC:12011] | 2006 | 0.165 | -0.0133 | No |
| 3 | ITGA3 | integrin subunit alpha 3 [Source:HGNC Symbol;Acc:HGNC:6139] | 2053 | 0.164 | 0.0033 | No |
| 4 | ADRB1 | adrenoceptor beta 1 [Source:HGNC Symbol;Acc:HGNC:285] | 2785 | 0.142 | 0.0000 | No |
| 5 | ITGB6 | integrin subunit beta 6 [Source:HGNC Symbol;Acc:HGNC:6161] | 3012 | 0.136 | 0.0090 | No |
| 6 | TPM3 | tropomyosin 3 [Source:HGNC Symbol;Acc:HGNC:12012] | 3045 | 0.135 | 0.0229 | No |
| 7 | CACNA1D | calcium voltage-gated channel subunit alpha1 D [Source:HGNC Symbol;Acc:HGNC:1391] | 3402 | 0.128 | 0.0277 | No |
| 8 | GNAS | GNAS complex locus [Source:HGNC Symbol;Acc:HGNC:4392] | 4003 | 0.118 | 0.0252 | No |
| 9 | ITGB1 | integrin subunit beta 1 [Source:HGNC Symbol;Acc:HGNC:6153] | 4161 | 0.115 | 0.0337 | No |
| 10 | TPM1 | tropomyosin 1 [Source:HGNC Symbol;Acc:HGNC:12010] | 4820 | 0.105 | 0.0283 | No |
| 11 | EMD | emerin [Source:HGNC Symbol;Acc:HGNC:3331] | 5060 | 0.102 | 0.0333 | No |
| 12 | CACNB3 | calcium voltage-gated channel auxiliary subunit beta 3 [Source:HGNC Symbol;Acc:HGNC:1403] | 5478 | 0.097 | 0.0331 | No |
| 13 | CACNB2 | calcium voltage-gated channel auxiliary subunit beta 2 [Source:HGNC Symbol;Acc:HGNC:1402] | 6229 | 0.091 | 0.0239 | No |
| 14 | DMD | dystrophin [Source:HGNC Symbol;Acc:HGNC:2928] | 6422 | 0.089 | 0.0287 | No |
| 15 | TNF | tumor necrosis factor [Source:HGNC Symbol;Acc:HGNC:11892] | 7309 | 0.081 | 0.0149 | No |
| 16 | CACNG1 | calcium voltage-gated channel auxiliary subunit gamma 1 [Source:HGNC Symbol;Acc:HGNC:1405] | 7895 | 0.076 | 0.0082 | No |
| 17 | TNNC1 | "troponin C1, slow skeletal and cardiac type [Source:HGNC Symbol;Acc:HGNC:11943]" | 8001 | 0.075 | 0.0136 | No |
| 18 | TNNI3 | "troponin I3, cardiac type [Source:HGNC Symbol;Acc:HGNC:11947]" | 8522 | 0.070 | 0.0080 | No |
| 19 | TGFB2 | transforming growth factor beta 2 [Source:HGNC Symbol;Acc:HGNC:11768] | 8941 | 0.068 | 0.0047 | No |
| 20 | MYL3 | myosin light chain 3 [Source:HGNC Symbol;Acc:HGNC:7584] | 9287 | 0.065 | 0.0030 | No |
| 21 | ITGB4 | integrin subunit beta 4 [Source:HGNC Symbol;Acc:HGNC:6158] | 11679 | 0.049 | -0.0526 | No |
| 22 | CACNG6 | calcium voltage-gated channel auxiliary subunit gamma 6 [Source:HGNC Symbol;Acc:HGNC:13625] | 13079 | 0.040 | -0.0839 | No |
| 23 | ITGB3 | integrin subunit beta 3 [Source:HGNC Symbol;Acc:HGNC:6156] | 15690 | 0.025 | -0.1477 | No |
| 24 | ACTG1 | actin gamma 1 [Source:HGNC Symbol;Acc:HGNC:144] | 15809 | 0.024 | -0.1481 | No |
| 25 | TGFB1 | transforming growth factor beta 1 [Source:HGNC Symbol;Acc:HGNC:11766] | 17301 | 0.016 | -0.1843 | No |
| 26 | SGCA | sarcoglycan alpha [Source:HGNC Symbol;Acc:HGNC:10805] | 18569 | 0.009 | -0.2156 | No |
| 27 | LMNA | lamin A/C [Source:HGNC Symbol;Acc:HGNC:6636] | 19070 | 0.006 | -0.2277 | No |
| 28 | DES | desmin [Source:HGNC Symbol;Acc:HGNC:2770] | 19713 | 0.002 | -0.2438 | No |
| 29 | ADCY6 | adenylate cyclase 6 [Source:HGNC Symbol;Acc:HGNC:237] | 20109 | 0.000 | -0.2539 | No |
| 30 | PLN | phospholamban [Source:HGNC Symbol;Acc:HGNC:9080] | 21301 | -0.007 | -0.2835 | No |
| 31 | PRKACA | protein kinase cAMP-activated catalytic subunit alpha [Source:HGNC Symbol;Acc:HGNC:9380] | 21332 | -0.007 | -0.2835 | No |
| 32 | CACNA1C | calcium voltage-gated channel subunit alpha1 C [Source:HGNC Symbol;Acc:HGNC:1390] | 21681 | -0.009 | -0.2913 | No |
| 33 | MYBPC3 | myosin binding protein C3 [Source:HGNC Symbol;Acc:HGNC:7551] | 22092 | -0.012 | -0.3005 | No |
| 34 | CACNG4 | calcium voltage-gated channel auxiliary subunit gamma 4 [Source:HGNC Symbol;Acc:HGNC:1408] | 22838 | -0.016 | -0.3177 | No |
| 35 | ITGB5 | integrin subunit beta 5 [Source:HGNC Symbol;Acc:HGNC:6160] | 23125 | -0.018 | -0.3230 | No |
| 36 | TGFB3 | transforming growth factor beta 3 [Source:HGNC Symbol;Acc:HGNC:11769] | 23618 | -0.021 | -0.3332 | No |
| 37 | CACNG8 | calcium voltage-gated channel auxiliary subunit gamma 8 [Source:HGNC Symbol;Acc:HGNC:13628] | 25438 | -0.034 | -0.3759 | No |
| 38 | CACNA1S | calcium voltage-gated channel subunit alpha1 S [Source:HGNC Symbol;Acc:HGNC:1397] | 25589 | -0.035 | -0.3759 | No |
| 39 | MYL2 | myosin light chain 2 [Source:HGNC Symbol;Acc:HGNC:7583] | 25828 | -0.037 | -0.3779 | No |
| 40 | ADCY1 | adenylate cyclase 1 [Source:HGNC Symbol;Acc:HGNC:232] | 25830 | -0.037 | -0.3740 | No |
| 41 | PRKACB | protein kinase cAMP-activated catalytic subunit beta [Source:HGNC Symbol;Acc:HGNC:9381] | 26088 | -0.039 | -0.3763 | No |
| 42 | ITGA7 | integrin subunit alpha 7 [Source:HGNC Symbol;Acc:HGNC:6143] | 26481 | -0.042 | -0.3818 | No |
| 43 | ITGA5 | integrin subunit alpha 5 [Source:HGNC Symbol;Acc:HGNC:6141] | 27755 | -0.051 | -0.4087 | Yes |
| 44 | ITGA2B | integrin subunit alpha 2b [Source:HGNC Symbol;Acc:HGNC:6138] | 27955 | -0.053 | -0.4080 | Yes |
| 45 | ADCY2 | adenylate cyclase 2 [Source:HGNC Symbol;Acc:HGNC:233] | 27981 | -0.053 | -0.4029 | Yes |
| 46 | CACNG3 | calcium voltage-gated channel auxiliary subunit gamma 3 [Source:HGNC Symbol;Acc:HGNC:1407] | 28055 | -0.054 | -0.3989 | Yes |
| 47 | CACNG7 | calcium voltage-gated channel auxiliary subunit gamma 7 [Source:HGNC Symbol;Acc:HGNC:13626] | 28606 | -0.058 | -0.4066 | Yes |
| 48 | MYH7 | myosin heavy chain 7 [Source:HGNC Symbol;Acc:HGNC:7577] | 28671 | -0.059 | -0.4019 | Yes |
| 49 | PRKACG | protein kinase cAMP-activated catalytic subunit gamma [Source:HGNC Symbol;Acc:HGNC:9382] | 28777 | -0.059 | -0.3981 | Yes |
| 50 | ITGA11 | integrin subunit alpha 11 [Source:HGNC Symbol;Acc:HGNC:6136] | 29036 | -0.062 | -0.3980 | Yes |
| 51 | LAMA2 | laminin subunit alpha 2 [Source:HGNC Symbol;Acc:HGNC:6482] | 29277 | -0.064 | -0.3972 | Yes |
| 52 | ITGA10 | integrin subunit alpha 10 [Source:HGNC Symbol;Acc:HGNC:6135] | 29376 | -0.065 | -0.3926 | Yes |
| 53 | CACNA2D2 | calcium voltage-gated channel auxiliary subunit alpha2delta 2 [Source:HGNC Symbol;Acc:HGNC:1400] | 29928 | -0.070 | -0.3991 | Yes |
| 54 | SGCB | sarcoglycan beta [Source:HGNC Symbol;Acc:HGNC:10806] | 30091 | -0.072 | -0.3954 | Yes |
| 55 | ITGA8 | integrin subunit alpha 8 [Source:HGNC Symbol;Acc:HGNC:6144] | 30487 | -0.075 | -0.3973 | Yes |
| 56 | ACTC1 | actin alpha cardiac muscle 1 [Source:HGNC Symbol;Acc:HGNC:143] | 30632 | -0.077 | -0.3927 | Yes |
| 57 | MYH6 | myosin heavy chain 6 [Source:HGNC Symbol;Acc:HGNC:7576] | 31177 | -0.082 | -0.3977 | Yes |
| 58 | ITGB7 | integrin subunit beta 7 [Source:HGNC Symbol;Acc:HGNC:6162] | 31210 | -0.082 | -0.3895 | Yes |
| 59 | TPM4 | tropomyosin 4 [Source:HGNC Symbol;Acc:HGNC:12013] | 31590 | -0.086 | -0.3898 | Yes |
| 60 | SGCG | sarcoglycan gamma [Source:HGNC Symbol;Acc:HGNC:10809] | 31734 | -0.088 | -0.3840 | Yes |
| 61 | ITGA4 | integrin subunit alpha 4 [Source:HGNC Symbol;Acc:HGNC:6140] | 32537 | -0.097 | -0.3939 | Yes |
| 62 | ITGAV | integrin subunit alpha V [Source:HGNC Symbol;Acc:HGNC:6150] | 33146 | -0.105 | -0.3979 | Yes |
| 63 | DAG1 | dystroglycan 1 [Source:HGNC Symbol;Acc:HGNC:2666] | 33311 | -0.108 | -0.3904 | Yes |
| 64 | IGF1 | insulin like growth factor 1 [Source:HGNC Symbol;Acc:HGNC:5464] | 33450 | -0.110 | -0.3821 | Yes |
| 65 | ITGA9 | integrin subunit alpha 9 [Source:HGNC Symbol;Acc:HGNC:6145] | 33520 | -0.111 | -0.3718 | Yes |
| 66 | CACNB1 | calcium voltage-gated channel auxiliary subunit beta 1 [Source:HGNC Symbol;Acc:HGNC:1401] | 34159 | -0.120 | -0.3751 | Yes |
| 67 | ITGA6 | integrin subunit alpha 6 [Source:HGNC Symbol;Acc:HGNC:6142] | 34278 | -0.122 | -0.3648 | Yes |
| 68 | CACNA1F | calcium voltage-gated channel subunit alpha1 F [Source:HGNC Symbol;Acc:HGNC:1393] | 34492 | -0.126 | -0.3567 | Yes |
| 69 | ADCY7 | adenylate cyclase 7 [Source:HGNC Symbol;Acc:HGNC:238] | 34661 | -0.129 | -0.3470 | Yes |
| 70 | SGCD | sarcoglycan delta [Source:HGNC Symbol;Acc:HGNC:10807] | 34699 | -0.130 | -0.3339 | Yes |
| 71 | ADCY4 | adenylate cyclase 4 [Source:HGNC Symbol;Acc:HGNC:235] | 34840 | -0.132 | -0.3231 | Yes |
| 72 | SLC8A1 | solute carrier family 8 member A1 [Source:HGNC Symbol;Acc:HGNC:11068] | 34954 | -0.135 | -0.3114 | Yes |
| 73 | ITGA2 | integrin subunit alpha 2 [Source:HGNC Symbol;Acc:HGNC:6137] | 35118 | -0.138 | -0.3006 | Yes |
| 74 | ITGB8 | integrin subunit beta 8 [Source:HGNC Symbol;Acc:HGNC:6163] | 35215 | -0.140 | -0.2879 | Yes |
| 75 | ADCY9 | adenylate cyclase 9 [Source:HGNC Symbol;Acc:HGNC:240] | 35385 | -0.144 | -0.2766 | Yes |
| 76 | CACNG5 | calcium voltage-gated channel auxiliary subunit gamma 5 [Source:HGNC Symbol;Acc:HGNC:1409] | 35813 | -0.155 | -0.2706 | Yes |
| 77 | ATP2A2 | ATPase sarcoplasmic/endoplasmic reticulum Ca2+ transporting 2 [Source:HGNC Symbol;Acc:HGNC:812] | 35828 | -0.156 | -0.2541 | Yes |
| 78 | ITGA1 | integrin subunit alpha 1 [Source:HGNC Symbol;Acc:HGNC:6134] | 35922 | -0.158 | -0.2393 | Yes |
| 79 | CACNA2D4 | calcium voltage-gated channel auxiliary subunit alpha2delta 4 [Source:HGNC Symbol;Acc:HGNC:20202] | 35958 | -0.159 | -0.2230 | Yes |
| 80 | CACNG2 | calcium voltage-gated channel auxiliary subunit gamma 2 [Source:HGNC Symbol;Acc:HGNC:1406] | 36107 | -0.163 | -0.2090 | Yes |
| 81 | TNNT2 | "troponin T2, cardiac type [Source:HGNC Symbol;Acc:HGNC:11949]" | 36689 | -0.184 | -0.2039 | Yes |
| 82 | TTN | titin [Source:HGNC Symbol;Acc:HGNC:12403] | 36764 | -0.187 | -0.1855 | Yes |
| 83 | CACNB4 | calcium voltage-gated channel auxiliary subunit beta 4 [Source:HGNC Symbol;Acc:HGNC:1404] | 37195 | -0.207 | -0.1741 | Yes |
| 84 | RYR2 | ryanodine receptor 2 [Source:HGNC Symbol;Acc:HGNC:10484] | 37202 | -0.207 | -0.1518 | Yes |
| 85 | ADCY5 | adenylate cyclase 5 [Source:HGNC Symbol;Acc:HGNC:236] | 37762 | -0.242 | -0.1398 | Yes |
| 86 | CACNA2D3 | calcium voltage-gated channel auxiliary subunit alpha2delta 3 [Source:HGNC Symbol;Acc:HGNC:15460] | 37984 | -0.264 | -0.1168 | Yes |
| 87 | ADCY8 | adenylate cyclase 8 [Source:HGNC Symbol;Acc:HGNC:239] | 38268 | -0.302 | -0.0912 | Yes |
| 88 | ADCY3 | adenylate cyclase 3 [Source:HGNC Symbol;Acc:HGNC:234] | 38423 | -0.337 | -0.0586 | Yes |
| 89 | CACNA2D1 | calcium voltage-gated channel auxiliary subunit alpha2delta 1 [Source:HGNC Symbol;Acc:HGNC:1399] | 38467 | -0.356 | -0.0211 | Yes |
| 90 | PRKX | protein kinase X-linked [Source:HGNC Symbol;Acc:HGNC:9441] | 38524 | -0.395 | 0.0204 | Yes |
Table: GSEA details [plain text format]

  

Fig 2: KEGG\_DILATED\_CARDIOMYOPATHY      
 Blue-Pink O' Gram in the Space of the Analyzed GeneSet

  

Fig 3: KEGG\_DILATED\_CARDIOMYOPATHY: Random ES distribution      
 Gene set null distribution of ES for **KEGG\_DILATED\_CARDIOMYOPATHY**
