# Supplementary material for: Simultaneous Presentation of Multiple Myeloma and Lung Cancer: Case Report and Gene Bioinformatics Analysis
Source: Front Oncol. 2022 Jun 13;12:859735. doi: 10.3389/fonc.2022.859735 (PMC9235397; doi:10.3389/fonc.2022.859735)
Supplement: Supplementary file 1 [file DataSheet_1.zip › The bioinformatic analysis of MM and lung cancer supplementary materials/Enrichment analysis/MECR/GSEA_4.1.0/LUSC TCGA/KEGG.Gsea.1639042374131/KEGG_FATTY_ACID_METABOLISM.html]

Details for gene set KEGG\_FATTY\_ACID\_METABOLISM[GSEA]

|  || Dataset | ExpData\_collapsed\_to\_symbols.ENSG00000116353\_profile\_in\_ExpData.cls #ENSG00000116353 |
| Phenotype | ENSG00000116353\_profile\_in\_ExpData.cls#ENSG00000116353 |
| Upregulated in class | ENSG00000116353\_pos |
| GeneSet | KEGG\_FATTY\_ACID\_METABOLISM |
| Enrichment Score (ES) | 0.45977604 |
| Normalized Enrichment Score (NES) | 1.6560559 |
| Nominal p-value | 0.015936255 |
| FDR q-value | 0.02657032 |
| FWER p-Value | 0.486 |
Table: GSEA Results Summary

  

Fig 1: Enrichment plot: KEGG\_FATTY\_ACID\_METABOLISM      
 Profile of the Running ES Score & Positions of GeneSet Members on the Rank Ordered List

  

| SYMBOL | TITLE | RANK IN GENE LIST | RANK METRIC SCORE | RUNNING ES | CORE ENRICHMENT || 1 | ECHS1 | "enoyl-CoA hydratase, short chain 1 [Source:HGNC Symbol;Acc:HGNC:3151]" | 132 | 0.372 | 0.0695 | Yes |
| 2 | CPT2 | carnitine palmitoyltransferase 2 [Source:HGNC Symbol;Acc:HGNC:2330] | 158 | 0.357 | 0.1387 | Yes |
| 3 | ACOX3 | "acyl-CoA oxidase 3, pristanoyl [Source:HGNC Symbol;Acc:HGNC:121]" | 331 | 0.300 | 0.1930 | Yes |
| 4 | HADH | hydroxyacyl-CoA dehydrogenase [Source:HGNC Symbol;Acc:HGNC:4799] | 366 | 0.293 | 0.2494 | Yes |
| 5 | ACADM | acyl-CoA dehydrogenase medium chain [Source:HGNC Symbol;Acc:HGNC:89] | 665 | 0.249 | 0.2905 | Yes |
| 6 | ALDH7A1 | aldehyde dehydrogenase 7 family member A1 [Source:HGNC Symbol;Acc:HGNC:877] | 1270 | 0.199 | 0.3140 | Yes |
| 7 | ECI1 | enoyl-CoA delta isomerase 1 [Source:HGNC Symbol;Acc:HGNC:2703] | 1311 | 0.197 | 0.3515 | Yes |
| 8 | ACADS | acyl-CoA dehydrogenase short chain [Source:HGNC Symbol;Acc:HGNC:90] | 1766 | 0.175 | 0.3741 | Yes |
| 9 | ADH5 | "alcohol dehydrogenase 5 (class III), chi polypeptide [Source:HGNC Symbol;Acc:HGNC:253]" | 1983 | 0.166 | 0.4012 | Yes |
| 10 | ACAA2 | acetyl-CoA acyltransferase 2 [Source:HGNC Symbol;Acc:HGNC:83] | 2060 | 0.163 | 0.4312 | Yes |
| 11 | HADHB | hydroxyacyl-CoA dehydrogenase trifunctional multienzyme complex subunit beta [Source:HGNC Symbol;Acc:HGNC:4803] | 3434 | 0.127 | 0.4212 | Yes |
| 12 | CYP4A11 | cytochrome P450 family 4 subfamily A member 11 [Source:HGNC Symbol;Acc:HGNC:2642] | 3622 | 0.124 | 0.4407 | Yes |
| 13 | HADHA | hydroxyacyl-CoA dehydrogenase trifunctional multienzyme complex subunit alpha [Source:HGNC Symbol;Acc:HGNC:4801] | 3803 | 0.121 | 0.4598 | Yes |
| 14 | ADH1C | "alcohol dehydrogenase 1C (class I), gamma polypeptide [Source:HGNC Symbol;Acc:HGNC:251]" | 5472 | 0.097 | 0.4363 | No |
| 15 | GCDH | glutaryl-CoA dehydrogenase [Source:HGNC Symbol;Acc:HGNC:4189] | 5976 | 0.094 | 0.4420 | No |
| 16 | ACSL5 | acyl-CoA synthetase long chain family member 5 [Source:HGNC Symbol;Acc:HGNC:16526] | 6915 | 0.084 | 0.4346 | No |
| 17 | CYP4A22 | cytochrome P450 family 4 subfamily A member 22 [Source:HGNC Symbol;Acc:HGNC:20575] | 7705 | 0.077 | 0.4297 | No |
| 18 | ACAA1 | acetyl-CoA acyltransferase 1 [Source:HGNC Symbol;Acc:HGNC:82] | 8047 | 0.075 | 0.4356 | No |
| 19 | ACADVL | acyl-CoA dehydrogenase very long chain [Source:HGNC Symbol;Acc:HGNC:92] | 8242 | 0.073 | 0.4449 | No |
| 20 | ACOX1 | acyl-CoA oxidase 1 [Source:HGNC Symbol;Acc:HGNC:119] | 11899 | 0.048 | 0.3612 | No |
| 21 | CPT1C | carnitine palmitoyltransferase 1C [Source:HGNC Symbol;Acc:HGNC:18540] | 15489 | 0.026 | 0.2749 | No |
| 22 | ACSL3 | acyl-CoA synthetase long chain family member 3 [Source:HGNC Symbol;Acc:HGNC:3570] | 16191 | 0.022 | 0.2614 | No |
| 23 | ALDH2 | aldehyde dehydrogenase 2 family member [Source:HGNC Symbol;Acc:HGNC:404] | 17769 | 0.013 | 0.2239 | No |
| 24 | ADH6 | alcohol dehydrogenase 6 (class V) [Source:HGNC Symbol;Acc:HGNC:255] | 19630 | 0.003 | 0.1771 | No |
| 25 | ADH1A | "alcohol dehydrogenase 1A (class I), alpha polypeptide [Source:HGNC Symbol;Acc:HGNC:249]" | 22804 | -0.016 | 0.0995 | No |
| 26 | ACSL4 | acyl-CoA synthetase long chain family member 4 [Source:HGNC Symbol;Acc:HGNC:3571] | 24666 | -0.029 | 0.0577 | No |
| 27 | ACADSB | acyl-CoA dehydrogenase short/branched chain [Source:HGNC Symbol;Acc:HGNC:91] | 24794 | -0.030 | 0.0603 | No |
| 28 | ADH1B | "alcohol dehydrogenase 1B (class I), beta polypeptide [Source:HGNC Symbol;Acc:HGNC:250]" | 24848 | -0.030 | 0.0648 | No |
| 29 | ECI2 | enoyl-CoA delta isomerase 2 [Source:HGNC Symbol;Acc:HGNC:14601] | 25126 | -0.032 | 0.0641 | No |
| 30 | ADH4 | "alcohol dehydrogenase 4 (class II), pi polypeptide [Source:HGNC Symbol;Acc:HGNC:252]" | 27558 | -0.050 | 0.0119 | No |
| 31 | ACAT2 | acetyl-CoA acetyltransferase 2 [Source:HGNC Symbol;Acc:HGNC:94] | 28042 | -0.054 | 0.0101 | No |
| 32 | ACAT1 | acetyl-CoA acetyltransferase 1 [Source:HGNC Symbol;Acc:HGNC:93] | 28412 | -0.057 | 0.0118 | No |
| 33 | EHHADH | enoyl-CoA hydratase and 3-hydroxyacyl CoA dehydrogenase [Source:HGNC Symbol;Acc:HGNC:3247] | 29653 | -0.067 | -0.0066 | No |
| 34 | ALDH9A1 | aldehyde dehydrogenase 9 family member A1 [Source:HGNC Symbol;Acc:HGNC:412] | 30215 | -0.073 | -0.0066 | No |
| 35 | ACADL | acyl-CoA dehydrogenase long chain [Source:HGNC Symbol;Acc:HGNC:88] | 30298 | -0.074 | 0.0057 | No |
| 36 | ALDH1B1 | aldehyde dehydrogenase 1 family member B1 [Source:HGNC Symbol;Acc:HGNC:407] | 32656 | -0.099 | -0.0350 | No |
| 37 | CPT1B | carnitine palmitoyltransferase 1B [Source:HGNC Symbol;Acc:HGNC:2329] | 34244 | -0.121 | -0.0516 | No |
| 38 | CPT1A | carnitine palmitoyltransferase 1A [Source:HGNC Symbol;Acc:HGNC:2328] | 35185 | -0.139 | -0.0483 | No |
| 39 | ACSL1 | acyl-CoA synthetase long chain family member 1 [Source:HGNC Symbol;Acc:HGNC:3569] | 36148 | -0.165 | -0.0406 | No |
| 40 | ADH7 | "alcohol dehydrogenase 7 (class IV), mu or sigma polypeptide [Source:HGNC Symbol;Acc:HGNC:256]" | 37003 | -0.197 | -0.0237 | No |
| 41 | ACSL6 | acyl-CoA synthetase long chain family member 6 [Source:HGNC Symbol;Acc:HGNC:16496] | 37189 | -0.207 | 0.0120 | No |
| 42 | ALDH3A2 | aldehyde dehydrogenase 3 family member A2 [Source:HGNC Symbol;Acc:HGNC:403] | 37378 | -0.216 | 0.0495 | No |
Table: GSEA details [plain text format]

  

Fig 2: KEGG\_FATTY\_ACID\_METABOLISM      
 Blue-Pink O' Gram in the Space of the Analyzed GeneSet

  

Fig 3: KEGG\_FATTY\_ACID\_METABOLISM: Random ES distribution      
 Gene set null distribution of ES for **KEGG\_FATTY\_ACID\_METABOLISM**
