# Supplementary material for: Simultaneous Presentation of Multiple Myeloma and Lung Cancer: Case Report and Gene Bioinformatics Analysis
Source: Front Oncol. 2022 Jun 13;12:859735. doi: 10.3389/fonc.2022.859735 (PMC9235397; doi:10.3389/fonc.2022.859735)
Supplement: Supplementary file 1 [file DataSheet_1.zip › The bioinformatic analysis of MM and lung cancer supplementary materials/Enrichment analysis/MECR/GSEA_4.1.0/LUSC TCGA/KEGG.Gsea.1639042374131/KEGG_GAP_JUNCTION.html]

Details for gene set KEGG\_GAP\_JUNCTION[GSEA]

|  || Dataset | ExpData\_collapsed\_to\_symbols.ENSG00000116353\_profile\_in\_ExpData.cls #ENSG00000116353 |
| Phenotype | ENSG00000116353\_profile\_in\_ExpData.cls#ENSG00000116353 |
| Upregulated in class | ENSG00000116353\_neg |
| GeneSet | KEGG\_GAP\_JUNCTION |
| Enrichment Score (ES) | -0.43256167 |
| Normalized Enrichment Score (NES) | -1.8292546 |
| Nominal p-value | 0.0 |
| FDR q-value | 0.008038147 |
| FWER p-Value | 0.089 |
Table: GSEA Results Summary

  

Fig 1: Enrichment plot: KEGG\_GAP\_JUNCTION      
 Profile of the Running ES Score & Positions of GeneSet Members on the Rank Ordered List

  

| SYMBOL | TITLE | RANK IN GENE LIST | RANK METRIC SCORE | RUNNING ES | CORE ENRICHMENT || 1 | MAPK3 | mitogen-activated protein kinase 3 [Source:HGNC Symbol;Acc:HGNC:6877] | 473 | 0.274 | 0.0151 | No |
| 2 | TUBB | tubulin beta class I [Source:HGNC Symbol;Acc:HGNC:20778] | 527 | 0.266 | 0.0400 | No |
| 3 | NRAS | "NRAS proto-oncogene, GTPase [Source:HGNC Symbol;Acc:HGNC:7989]" | 774 | 0.235 | 0.0570 | No |
| 4 | GNAI3 | G protein subunit alpha i3 [Source:HGNC Symbol;Acc:HGNC:4387] | 869 | 0.226 | 0.0769 | No |
| 5 | PLCB3 | phospholipase C beta 3 [Source:HGNC Symbol;Acc:HGNC:9056] | 1971 | 0.167 | 0.0654 | No |
| 6 | TUBB3 | tubulin beta 3 class III [Source:HGNC Symbol;Acc:HGNC:20772] | 1974 | 0.167 | 0.0818 | No |
| 7 | TUBA1B | tubulin alpha 1b [Source:HGNC Symbol;Acc:HGNC:18809] | 2173 | 0.160 | 0.0925 | No |
| 8 | ADRB1 | adrenoceptor beta 1 [Source:HGNC Symbol;Acc:HGNC:285] | 2785 | 0.142 | 0.0910 | No |
| 9 | SOS2 | SOS Ras/Rho guanine nucleotide exchange factor 2 [Source:HGNC Symbol;Acc:HGNC:11188] | 3713 | 0.123 | 0.0795 | No |
| 10 | GNAS | GNAS complex locus [Source:HGNC Symbol;Acc:HGNC:4392] | 4003 | 0.118 | 0.0837 | No |
| 11 | SRC | "SRC proto-oncogene, non-receptor tyrosine kinase [Source:HGNC Symbol;Acc:HGNC:11283]" | 4857 | 0.105 | 0.0724 | No |
| 12 | TUBB8 | tubulin beta 8 class VIII [Source:HGNC Symbol;Acc:HGNC:20773] | 4983 | 0.103 | 0.0794 | No |
| 13 | GJD2 | gap junction protein delta 2 [Source:HGNC Symbol;Acc:HGNC:19154] | 5949 | 0.095 | 0.0641 | No |
| 14 | TUBA1A | tubulin alpha 1a [Source:HGNC Symbol;Acc:HGNC:20766] | 6944 | 0.084 | 0.0471 | No |
| 15 | PDGFA | platelet derived growth factor subunit A [Source:HGNC Symbol;Acc:HGNC:8799] | 10013 | 0.060 | -0.0252 | No |
| 16 | TUBA4A | tubulin alpha 4a [Source:HGNC Symbol;Acc:HGNC:12407] | 11015 | 0.053 | -0.0454 | No |
| 17 | GNAI1 | G protein subunit alpha i1 [Source:HGNC Symbol;Acc:HGNC:4384] | 11240 | 0.052 | -0.0459 | No |
| 18 | CSNK1D | casein kinase 1 delta [Source:HGNC Symbol;Acc:HGNC:2452] | 12383 | 0.045 | -0.0706 | No |
| 19 | CDK1 | cyclin dependent kinase 1 [Source:HGNC Symbol;Acc:HGNC:1722] | 12387 | 0.045 | -0.0663 | No |
| 20 | TUBA1C | tubulin alpha 1c [Source:HGNC Symbol;Acc:HGNC:20768] | 13029 | 0.041 | -0.0786 | No |
| 21 | PDGFC | platelet derived growth factor C [Source:HGNC Symbol;Acc:HGNC:8801] | 14044 | 0.035 | -0.1010 | No |
| 22 | TUBAL3 | tubulin alpha like 3 [Source:HGNC Symbol;Acc:HGNC:23534] | 14974 | 0.029 | -0.1218 | No |
| 23 | GNAI2 | G protein subunit alpha i2 [Source:HGNC Symbol;Acc:HGNC:4385] | 15092 | 0.029 | -0.1219 | No |
| 24 | PRKCG | protein kinase C gamma [Source:HGNC Symbol;Acc:HGNC:9402] | 15351 | 0.027 | -0.1258 | No |
| 25 | MAP2K2 | mitogen-activated protein kinase kinase 2 [Source:HGNC Symbol;Acc:HGNC:6842] | 15510 | 0.026 | -0.1272 | No |
| 26 | TUBA3C | tubulin alpha 3c [Source:HGNC Symbol;Acc:HGNC:12408] | 15604 | 0.026 | -0.1271 | No |
| 27 | RAF1 | "Raf-1 proto-oncogene, serine/threonine kinase [Source:HGNC Symbol;Acc:HGNC:9829]" | 16140 | 0.022 | -0.1385 | No |
| 28 | TUBA3E | tubulin alpha 3e [Source:HGNC Symbol;Acc:HGNC:20765] | 16937 | 0.018 | -0.1570 | No |
| 29 | TUBB2A | tubulin beta 2A class IIa [Source:HGNC Symbol;Acc:HGNC:12412] | 19013 | 0.007 | -0.2092 | No |
| 30 | TUBB7P | tubulin beta 7 pseudogene [Source:HGNC Symbol;Acc:HGNC:12413] | 19664 | 0.003 | -0.2255 | No |
| 31 | ADCY6 | adenylate cyclase 6 [Source:HGNC Symbol;Acc:HGNC:237] | 20109 | 0.000 | -0.2368 | No |
| 32 | HRAS | "HRas proto-oncogene, GTPase [Source:HGNC Symbol;Acc:HGNC:5173]" | 20556 | -0.003 | -0.2479 | No |
| 33 | PDGFD | platelet derived growth factor D [Source:HGNC Symbol;Acc:HGNC:30620] | 20737 | -0.004 | -0.2522 | No |
| 34 | HTR2B | 5-hydroxytryptamine receptor 2B [Source:HGNC Symbol;Acc:HGNC:5294] | 20981 | -0.005 | -0.2578 | No |
| 35 | PRKACA | protein kinase cAMP-activated catalytic subunit alpha [Source:HGNC Symbol;Acc:HGNC:9380] | 21332 | -0.007 | -0.2660 | No |
| 36 | MAPK7 | mitogen-activated protein kinase 7 [Source:HGNC Symbol;Acc:HGNC:6880] | 21460 | -0.008 | -0.2685 | No |
| 37 | HTR2C | 5-hydroxytryptamine receptor 2C [Source:HGNC Symbol;Acc:HGNC:5295] | 22469 | -0.014 | -0.2928 | No |
| 38 | PDGFB | platelet derived growth factor subunit B [Source:HGNC Symbol;Acc:HGNC:8800] | 23487 | -0.021 | -0.3167 | No |
| 39 | ADCY1 | adenylate cyclase 1 [Source:HGNC Symbol;Acc:HGNC:232] | 25830 | -0.037 | -0.3727 | No |
| 40 | PRKACB | protein kinase cAMP-activated catalytic subunit beta [Source:HGNC Symbol;Acc:HGNC:9381] | 26088 | -0.039 | -0.3754 | No |
| 41 | GRM5 | glutamate metabotropic receptor 5 [Source:HGNC Symbol;Acc:HGNC:4597] | 27139 | -0.047 | -0.3976 | No |
| 42 | TUBA3D | tubulin alpha 3d [Source:HGNC Symbol;Acc:HGNC:24071] | 27665 | -0.051 | -0.4060 | No |
| 43 | PLCB1 | phospholipase C beta 1 [Source:HGNC Symbol;Acc:HGNC:15917] | 27781 | -0.052 | -0.4038 | No |
| 44 | ADCY2 | adenylate cyclase 2 [Source:HGNC Symbol;Acc:HGNC:233] | 27981 | -0.053 | -0.4036 | No |
| 45 | GNA11 | G protein subunit alpha 11 [Source:HGNC Symbol;Acc:HGNC:4379] | 28013 | -0.054 | -0.3991 | No |
| 46 | TUBB1 | tubulin beta 1 class VI [Source:HGNC Symbol;Acc:HGNC:16257] | 28471 | -0.057 | -0.4051 | No |
| 47 | PRKACG | protein kinase cAMP-activated catalytic subunit gamma [Source:HGNC Symbol;Acc:HGNC:9382] | 28777 | -0.059 | -0.4070 | No |
| 48 | DRD2 | dopamine receptor D2 [Source:HGNC Symbol;Acc:HGNC:3023] | 28890 | -0.060 | -0.4039 | No |
| 49 | ITPR3 | "inositol 1,4,5-trisphosphate receptor type 3 [Source:HGNC Symbol;Acc:HGNC:6182]" | 29471 | -0.066 | -0.4121 | No |
| 50 | TUBB6 | tubulin beta 6 class V [Source:HGNC Symbol;Acc:HGNC:20776] | 29744 | -0.068 | -0.4123 | No |
| 51 | DRD1 | dopamine receptor D1 [Source:HGNC Symbol;Acc:HGNC:3020] | 29908 | -0.070 | -0.4096 | No |
| 52 | PDGFRA | platelet derived growth factor receptor alpha [Source:HGNC Symbol;Acc:HGNC:8803] | 30377 | -0.074 | -0.4141 | No |
| 53 | PRKG2 | protein kinase cGMP-dependent 2 [Source:HGNC Symbol;Acc:HGNC:9416] | 30839 | -0.079 | -0.4181 | No |
| 54 | TUBA8 | tubulin alpha 8 [Source:HGNC Symbol;Acc:HGNC:12410] | 30983 | -0.080 | -0.4138 | No |
| 55 | MAP2K5 | mitogen-activated protein kinase kinase 5 [Source:HGNC Symbol;Acc:HGNC:6845] | 31433 | -0.085 | -0.4169 | No |
| 56 | MAP2K1 | mitogen-activated protein kinase kinase 1 [Source:HGNC Symbol;Acc:HGNC:6840] | 32048 | -0.091 | -0.4235 | Yes |
| 57 | EGFR | epidermal growth factor receptor [Source:HGNC Symbol;Acc:HGNC:3236] | 32133 | -0.092 | -0.4165 | Yes |
| 58 | PDGFRB | platelet derived growth factor receptor beta [Source:HGNC Symbol;Acc:HGNC:8804] | 32165 | -0.093 | -0.4082 | Yes |
| 59 | PLCB2 | phospholipase C beta 2 [Source:HGNC Symbol;Acc:HGNC:9055] | 32369 | -0.095 | -0.4039 | Yes |
| 60 | GUCY1A2 | guanylate cyclase 1 soluble subunit alpha 2 [Source:HGNC Symbol;Acc:HGNC:4684] | 32385 | -0.095 | -0.3949 | Yes |
| 61 | GRB2 | growth factor receptor bound protein 2 [Source:HGNC Symbol;Acc:HGNC:4566] | 32433 | -0.096 | -0.3866 | Yes |
| 62 | MAPK1 | mitogen-activated protein kinase 1 [Source:HGNC Symbol;Acc:HGNC:6871] | 33350 | -0.108 | -0.3992 | Yes |
| 63 | ITPR2 | "inositol 1,4,5-trisphosphate receptor type 2 [Source:HGNC Symbol;Acc:HGNC:6181]" | 34058 | -0.119 | -0.4055 | Yes |
| 64 | HTR2A | 5-hydroxytryptamine receptor 2A [Source:HGNC Symbol;Acc:HGNC:5293] | 34078 | -0.119 | -0.3942 | Yes |
| 65 | GJA1 | gap junction protein alpha 1 [Source:HGNC Symbol;Acc:HGNC:4274] | 34093 | -0.119 | -0.3828 | Yes |
| 66 | PRKCB | protein kinase C beta [Source:HGNC Symbol;Acc:HGNC:9395] | 34603 | -0.128 | -0.3832 | Yes |
| 67 | TUBB4B | tubulin beta 4B class IVb [Source:HGNC Symbol;Acc:HGNC:20771] | 34640 | -0.128 | -0.3714 | Yes |
| 68 | ADCY7 | adenylate cyclase 7 [Source:HGNC Symbol;Acc:HGNC:238] | 34661 | -0.129 | -0.3592 | Yes |
| 69 | ADCY4 | adenylate cyclase 4 [Source:HGNC Symbol;Acc:HGNC:235] | 34840 | -0.132 | -0.3507 | Yes |
| 70 | PRKCA | protein kinase C alpha [Source:HGNC Symbol;Acc:HGNC:9393] | 35223 | -0.140 | -0.3465 | Yes |
| 71 | GUCY1A1 | guanylate cyclase 1 soluble subunit alpha 1 [Source:HGNC Symbol;Acc:HGNC:4685] | 35261 | -0.141 | -0.3335 | Yes |
| 72 | ADCY9 | adenylate cyclase 9 [Source:HGNC Symbol;Acc:HGNC:240] | 35385 | -0.144 | -0.3224 | Yes |
| 73 | TUBB2B | tubulin beta 2B class IIb [Source:HGNC Symbol;Acc:HGNC:30829] | 35749 | -0.153 | -0.3165 | Yes |
| 74 | TUBB4A | tubulin beta 4A class IVa [Source:HGNC Symbol;Acc:HGNC:20774] | 35751 | -0.154 | -0.3013 | Yes |
| 75 | GUCY1B1 | guanylate cyclase 1 soluble subunit beta 1 [Source:HGNC Symbol;Acc:HGNC:4687] | 36017 | -0.161 | -0.2922 | Yes |
| 76 | ITPR1 | "inositol 1,4,5-trisphosphate receptor type 1 [Source:HGNC Symbol;Acc:HGNC:6180]" | 36283 | -0.169 | -0.2822 | Yes |
| 77 | TJP1 | tight junction protein 1 [Source:HGNC Symbol;Acc:HGNC:11827] | 36324 | -0.171 | -0.2663 | Yes |
| 78 | GNAQ | G protein subunit alpha q [Source:HGNC Symbol;Acc:HGNC:4390] | 36400 | -0.173 | -0.2511 | Yes |
| 79 | PRKG1 | protein kinase cGMP-dependent 1 [Source:HGNC Symbol;Acc:HGNC:9414] | 36496 | -0.176 | -0.2361 | Yes |
| 80 | MAP3K2 | mitogen-activated protein kinase kinase kinase 2 [Source:HGNC Symbol;Acc:HGNC:6854] | 36502 | -0.177 | -0.2188 | Yes |
| 81 | SOS1 | SOS Ras/Rac guanine nucleotide exchange factor 1 [Source:HGNC Symbol;Acc:HGNC:11187] | 36506 | -0.177 | -0.2014 | Yes |
| 82 | KRAS | "KRAS proto-oncogene, GTPase [Source:HGNC Symbol;Acc:HGNC:6407]" | 36928 | -0.194 | -0.1929 | Yes |
| 83 | EGF | epidermal growth factor [Source:HGNC Symbol;Acc:HGNC:3229] | 37597 | -0.231 | -0.1871 | Yes |
| 84 | ADCY5 | adenylate cyclase 5 [Source:HGNC Symbol;Acc:HGNC:236] | 37762 | -0.242 | -0.1673 | Yes |
| 85 | ADCY8 | adenylate cyclase 8 [Source:HGNC Symbol;Acc:HGNC:239] | 38268 | -0.302 | -0.1503 | Yes |
| 86 | PLCB4 | phospholipase C beta 4 [Source:HGNC Symbol;Acc:HGNC:9059] | 38308 | -0.309 | -0.1207 | Yes |
| 87 | ADCY3 | adenylate cyclase 3 [Source:HGNC Symbol;Acc:HGNC:234] | 38423 | -0.337 | -0.0902 | Yes |
| 88 | GRM1 | glutamate metabotropic receptor 1 [Source:HGNC Symbol;Acc:HGNC:4593] | 38453 | -0.351 | -0.0562 | Yes |
| 89 | PRKX | protein kinase X-linked [Source:HGNC Symbol;Acc:HGNC:9441] | 38524 | -0.395 | -0.0189 | Yes |
| 90 | LPAR1 | lysophosphatidic acid receptor 1 [Source:HGNC Symbol;Acc:HGNC:3166] | 38527 | -0.397 | 0.0203 | Yes |
Table: GSEA details [plain text format]

  

Fig 2: KEGG\_GAP\_JUNCTION      
 Blue-Pink O' Gram in the Space of the Analyzed GeneSet

  

Fig 3: KEGG\_GAP\_JUNCTION: Random ES distribution      
 Gene set null distribution of ES for **KEGG\_GAP\_JUNCTION**
