# Supplementary material for: Simultaneous Presentation of Multiple Myeloma and Lung Cancer: Case Report and Gene Bioinformatics Analysis
Source: Front Oncol. 2022 Jun 13;12:859735. doi: 10.3389/fonc.2022.859735 (PMC9235397; doi:10.3389/fonc.2022.859735)
Supplement: Supplementary file 1 [file DataSheet_1.zip › The bioinformatic analysis of MM and lung cancer supplementary materials/Enrichment analysis/MECR/GSEA_4.1.0/LUSC TCGA/KEGG.Gsea.1639042374131/KEGG_GLYOXYLATE_AND_DICARBOXYLATE_METABOLISM.html]

Details for gene set KEGG\_GLYOXYLATE\_AND\_DICARBOXYLATE\_METABOLISM[GSEA]

|  || Dataset | ExpData\_collapsed\_to\_symbols.ENSG00000116353\_profile\_in\_ExpData.cls #ENSG00000116353 |
| Phenotype | ENSG00000116353\_profile\_in\_ExpData.cls#ENSG00000116353 |
| Upregulated in class | ENSG00000116353\_pos |
| GeneSet | KEGG\_GLYOXYLATE\_AND\_DICARBOXYLATE\_METABOLISM |
| Enrichment Score (ES) | 0.6782122 |
| Normalized Enrichment Score (NES) | 1.9395978 |
| Nominal p-value | 0.0 |
| FDR q-value | 0.0021974545 |
| FWER p-Value | 0.034 |
Table: GSEA Results Summary

  

Fig 1: Enrichment plot: KEGG\_GLYOXYLATE\_AND\_DICARBOXYLATE\_METABOLISM      
 Profile of the Running ES Score & Positions of GeneSet Members on the Rank Ordered List

  

| SYMBOL | TITLE | RANK IN GENE LIST | RANK METRIC SCORE | RUNNING ES | CORE ENRICHMENT || 1 | HYI | hydroxypyruvate isomerase (putative) [Source:HGNC Symbol;Acc:HGNC:26948] | 720 | 0.242 | 0.1348 | Yes |
| 2 | MTHFD1 | "methylenetetrahydrofolate dehydrogenase, cyclohydrolase and formyltetrahydrofolate synthetase 1 [Source:HGNC Symbol;Acc:HGNC:7432]" | 1248 | 0.200 | 0.2480 | Yes |
| 3 | MDH2 | malate dehydrogenase 2 [Source:HGNC Symbol;Acc:HGNC:6971] | 1509 | 0.186 | 0.3590 | Yes |
| 4 | MTHFD2L | methylenetetrahydrofolate dehydrogenase (NADP+ dependent) 2 like [Source:HGNC Symbol;Acc:HGNC:31865] | 1813 | 0.173 | 0.4607 | Yes |
| 5 | HAO2 | hydroxyacid oxidase 2 [Source:HGNC Symbol;Acc:HGNC:4810] | 3063 | 0.135 | 0.5143 | Yes |
| 6 | AFMID | arylformamidase [Source:HGNC Symbol;Acc:HGNC:20910] | 3753 | 0.122 | 0.5738 | Yes |
| 7 | MDH1 | malate dehydrogenase 1 [Source:HGNC Symbol;Acc:HGNC:6970] | 5087 | 0.102 | 0.6041 | Yes |
| 8 | PGP | phosphoglycolate phosphatase [Source:HGNC Symbol;Acc:HGNC:8909] | 6093 | 0.093 | 0.6373 | Yes |
| 9 | MTHFD1L | methylenetetrahydrofolate dehydrogenase (NADP+ dependent) 1 like [Source:HGNC Symbol;Acc:HGNC:21055] | 6651 | 0.087 | 0.6782 | Yes |
| 10 | HAO1 | hydroxyacid oxidase 1 [Source:HGNC Symbol;Acc:HGNC:4809] | 15295 | 0.028 | 0.4758 | No |
| 11 | ACO2 | aconitase 2 [Source:HGNC Symbol;Acc:HGNC:118] | 16445 | 0.021 | 0.4597 | No |
| 12 | GRHPR | glyoxylate and hydroxypyruvate reductase [Source:HGNC Symbol;Acc:HGNC:4570] | 17476 | 0.015 | 0.4429 | No |
| 13 | GLYCTK | glycerate kinase [Source:HGNC Symbol;Acc:HGNC:24247] | 22115 | -0.012 | 0.3326 | No |
| 14 | CS | citrate synthase [Source:HGNC Symbol;Acc:HGNC:2422] | 23879 | -0.023 | 0.3022 | No |
| 15 | MTHFD2 | "methylenetetrahydrofolate dehydrogenase (NADP+ dependent) 2, methenyltetrahydrofolate cyclohydrolase [Source:HGNC Symbol;Acc:HGNC:7434]" | 25356 | -0.034 | 0.2860 | No |
| 16 | ACO1 | aconitase 1 [Source:HGNC Symbol;Acc:HGNC:117] | 33442 | -0.110 | 0.1496 | No |
Table: GSEA details [plain text format]

  

Fig 2: KEGG\_GLYOXYLATE\_AND\_DICARBOXYLATE\_METABOLISM      
 Blue-Pink O' Gram in the Space of the Analyzed GeneSet

  

Fig 3: KEGG\_GLYOXYLATE\_AND\_DICARBOXYLATE\_METABOLISM: Random ES distribution      
 Gene set null distribution of ES for **KEGG\_GLYOXYLATE\_AND\_DICARBOXYLATE\_METABOLISM**
