# Supplementary material for: Simultaneous Presentation of Multiple Myeloma and Lung Cancer: Case Report and Gene Bioinformatics Analysis
Source: Front Oncol. 2022 Jun 13;12:859735. doi: 10.3389/fonc.2022.859735 (PMC9235397; doi:10.3389/fonc.2022.859735)
Supplement: Supplementary file 1 [file DataSheet_1.zip › The bioinformatic analysis of MM and lung cancer supplementary materials/Enrichment analysis/MECR/GSEA_4.1.0/LUSC TCGA/KEGG.Gsea.1639042374131/KEGG_HEDGEHOG_SIGNALING_PATHWAY.html]

Details for gene set KEGG\_HEDGEHOG\_SIGNALING\_PATHWAY[GSEA]

|  || Dataset | ExpData\_collapsed\_to\_symbols.ENSG00000116353\_profile\_in\_ExpData.cls #ENSG00000116353 |
| Phenotype | ENSG00000116353\_profile\_in\_ExpData.cls#ENSG00000116353 |
| Upregulated in class | ENSG00000116353\_neg |
| GeneSet | KEGG\_HEDGEHOG\_SIGNALING\_PATHWAY |
| Enrichment Score (ES) | -0.5016084 |
| Normalized Enrichment Score (NES) | -1.9447964 |
| Nominal p-value | 0.0 |
| FDR q-value | 0.003933334 |
| FWER p-Value | 0.022 |
Table: GSEA Results Summary

  

Fig 1: Enrichment plot: KEGG\_HEDGEHOG\_SIGNALING\_PATHWAY      
 Profile of the Running ES Score & Positions of GeneSet Members on the Rank Ordered List

  

| SYMBOL | TITLE | RANK IN GENE LIST | RANK METRIC SCORE | RUNNING ES | CORE ENRICHMENT || 1 | WNT10A | Wnt family member 10A [Source:HGNC Symbol;Acc:HGNC:13829] | 839 | 0.228 | 0.0120 | No |
| 2 | WNT4 | Wnt family member 4 [Source:HGNC Symbol;Acc:HGNC:12783] | 1086 | 0.210 | 0.0364 | No |
| 3 | CSNK1E | casein kinase 1 epsilon [Source:HGNC Symbol;Acc:HGNC:2453] | 2470 | 0.150 | 0.0231 | No |
| 4 | GLI3 | GLI family zinc finger 3 [Source:HGNC Symbol;Acc:HGNC:4319] | 3544 | 0.126 | 0.0142 | No |
| 5 | SUFU | SUFU negative regulator of hedgehog signaling [Source:HGNC Symbol;Acc:HGNC:16466] | 3947 | 0.119 | 0.0213 | No |
| 6 | WNT6 | Wnt family member 6 [Source:HGNC Symbol;Acc:HGNC:12785] | 4672 | 0.107 | 0.0186 | No |
| 7 | WNT16 | Wnt family member 16 [Source:HGNC Symbol;Acc:HGNC:16267] | 6037 | 0.094 | -0.0024 | No |
| 8 | WNT8A | Wnt family member 8A [Source:HGNC Symbol;Acc:HGNC:12788] | 8416 | 0.071 | -0.0526 | No |
| 9 | WNT7A | Wnt family member 7A [Source:HGNC Symbol;Acc:HGNC:12786] | 10774 | 0.055 | -0.1045 | No |
| 10 | CSNK1A1L | casein kinase 1 alpha 1 like [Source:HGNC Symbol;Acc:HGNC:20289] | 11977 | 0.047 | -0.1282 | No |
| 11 | IHH | Indian hedgehog signaling molecule [Source:HGNC Symbol;Acc:HGNC:5956] | 11991 | 0.047 | -0.1216 | No |
| 12 | BMP5 | bone morphogenetic protein 5 [Source:HGNC Symbol;Acc:HGNC:1072] | 12002 | 0.047 | -0.1150 | No |
| 13 | CSNK1D | casein kinase 1 delta [Source:HGNC Symbol;Acc:HGNC:2452] | 12383 | 0.045 | -0.1181 | No |
| 14 | STK36 | serine/threonine kinase 36 [Source:HGNC Symbol;Acc:HGNC:17209] | 12694 | 0.043 | -0.1198 | No |
| 15 | GSK3B | glycogen synthase kinase 3 beta [Source:HGNC Symbol;Acc:HGNC:4617] | 15772 | 0.025 | -0.1945 | No |
| 16 | LRP2 | LDL receptor related protein 2 [Source:HGNC Symbol;Acc:HGNC:6694] | 17470 | 0.015 | -0.2355 | No |
| 17 | BTRC | beta-transducin repeat containing E3 ubiquitin protein ligase [Source:HGNC Symbol;Acc:HGNC:1144] | 18315 | 0.011 | -0.2555 | No |
| 18 | WNT1 | Wnt family member 1 [Source:HGNC Symbol;Acc:HGNC:12774] | 19377 | 0.004 | -0.2819 | No |
| 19 | BMP2 | bone morphogenetic protein 2 [Source:HGNC Symbol;Acc:HGNC:1069] | 20791 | -0.004 | -0.3173 | No |
| 20 | PRKACA | protein kinase cAMP-activated catalytic subunit alpha [Source:HGNC Symbol;Acc:HGNC:9380] | 21332 | -0.007 | -0.3300 | No |
| 21 | CSNK1G2 | casein kinase 1 gamma 2 [Source:HGNC Symbol;Acc:HGNC:2455] | 22616 | -0.015 | -0.3604 | No |
| 22 | WNT7B | Wnt family member 7B [Source:HGNC Symbol;Acc:HGNC:12787] | 22905 | -0.017 | -0.3653 | No |
| 23 | BMP8B | bone morphogenetic protein 8b [Source:HGNC Symbol;Acc:HGNC:1075] | 23652 | -0.022 | -0.3812 | No |
| 24 | RAB23 | "RAB23, member RAS oncogene family [Source:HGNC Symbol;Acc:HGNC:14263]" | 24052 | -0.024 | -0.3878 | No |
| 25 | WNT9A | Wnt family member 9A [Source:HGNC Symbol;Acc:HGNC:12778] | 25791 | -0.037 | -0.4267 | No |
| 26 | PRKACB | protein kinase cAMP-activated catalytic subunit beta [Source:HGNC Symbol;Acc:HGNC:9381] | 26088 | -0.039 | -0.4286 | No |
| 27 | WNT10B | Wnt family member 10B [Source:HGNC Symbol;Acc:HGNC:12775] | 26603 | -0.042 | -0.4354 | No |
| 28 | CSNK1G3 | casein kinase 1 gamma 3 [Source:HGNC Symbol;Acc:HGNC:2456] | 27790 | -0.052 | -0.4581 | No |
| 29 | HHIP | hedgehog interacting protein [Source:HGNC Symbol;Acc:HGNC:14866] | 28238 | -0.055 | -0.4614 | No |
| 30 | PRKACG | protein kinase cAMP-activated catalytic subunit gamma [Source:HGNC Symbol;Acc:HGNC:9382] | 28777 | -0.059 | -0.4664 | No |
| 31 | WNT3A | Wnt family member 3A [Source:HGNC Symbol;Acc:HGNC:15983] | 29041 | -0.062 | -0.4640 | No |
| 32 | BMP4 | bone morphogenetic protein 4 [Source:HGNC Symbol;Acc:HGNC:1071] | 29894 | -0.070 | -0.4755 | No |
| 33 | BMP8A | bone morphogenetic protein 8a [Source:HGNC Symbol;Acc:HGNC:21650] | 30182 | -0.072 | -0.4722 | No |
| 34 | GAS1 | growth arrest specific 1 [Source:HGNC Symbol;Acc:HGNC:4165] | 30802 | -0.078 | -0.4765 | No |
| 35 | DHH | desert hedgehog signaling molecule [Source:HGNC Symbol;Acc:HGNC:2865] | 30991 | -0.080 | -0.4696 | No |
| 36 | WNT5B | Wnt family member 5B [Source:HGNC Symbol;Acc:HGNC:16265] | 32250 | -0.094 | -0.4879 | Yes |
| 37 | WNT2 | Wnt family member 2 [Source:HGNC Symbol;Acc:HGNC:12780] | 32471 | -0.096 | -0.4794 | Yes |
| 38 | CSNK1A1 | casein kinase 1 alpha 1 [Source:HGNC Symbol;Acc:HGNC:2451] | 33103 | -0.105 | -0.4801 | Yes |
| 39 | WNT8B | Wnt family member 8B [Source:HGNC Symbol;Acc:HGNC:12789] | 33128 | -0.105 | -0.4653 | Yes |
| 40 | BMP7 | bone morphogenetic protein 7 [Source:HGNC Symbol;Acc:HGNC:1074] | 33291 | -0.107 | -0.4537 | Yes |
| 41 | ZIC2 | Zic family member 2 [Source:HGNC Symbol;Acc:HGNC:12873] | 34785 | -0.131 | -0.4726 | Yes |
| 42 | FBXW11 | F-box and WD repeat domain containing 11 [Source:HGNC Symbol;Acc:HGNC:13607] | 35478 | -0.146 | -0.4688 | Yes |
| 43 | PTCH2 | patched 2 [Source:HGNC Symbol;Acc:HGNC:9586] | 35779 | -0.154 | -0.4539 | Yes |
| 44 | WNT9B | Wnt family member 9B [Source:HGNC Symbol;Acc:HGNC:12779] | 35896 | -0.157 | -0.4338 | Yes |
| 45 | WNT2B | Wnt family member 2B [Source:HGNC Symbol;Acc:HGNC:12781] | 36087 | -0.163 | -0.4148 | Yes |
| 46 | SHH | sonic hedgehog signaling molecule [Source:HGNC Symbol;Acc:HGNC:10848] | 36592 | -0.179 | -0.4014 | Yes |
| 47 | BMP6 | bone morphogenetic protein 6 [Source:HGNC Symbol;Acc:HGNC:1073] | 36657 | -0.182 | -0.3763 | Yes |
| 48 | WNT11 | Wnt family member 11 [Source:HGNC Symbol;Acc:HGNC:12776] | 37666 | -0.236 | -0.3675 | Yes |
| 49 | CSNK1G1 | casein kinase 1 gamma 1 [Source:HGNC Symbol;Acc:HGNC:2454] | 37862 | -0.250 | -0.3359 | Yes |
| 50 | WNT3 | Wnt family member 3 [Source:HGNC Symbol;Acc:HGNC:12782] | 38054 | -0.271 | -0.3011 | Yes |
| 51 | SMO | "smoothened, frizzled class receptor [Source:HGNC Symbol;Acc:HGNC:11119]" | 38185 | -0.291 | -0.2618 | Yes |
| 52 | WNT5A | Wnt family member 5A [Source:HGNC Symbol;Acc:HGNC:12784] | 38488 | -0.368 | -0.2156 | Yes |
| 53 | GLI1 | GLI family zinc finger 1 [Source:HGNC Symbol;Acc:HGNC:4317] | 38507 | -0.378 | -0.1607 | Yes |
| 54 | PTCH1 | patched 1 [Source:HGNC Symbol;Acc:HGNC:9585] | 38519 | -0.389 | -0.1041 | Yes |
| 55 | PRKX | protein kinase X-linked [Source:HGNC Symbol;Acc:HGNC:9441] | 38524 | -0.395 | -0.0463 | Yes |
| 56 | GLI2 | GLI family zinc finger 2 [Source:HGNC Symbol;Acc:HGNC:4318] | 38549 | -0.455 | 0.0197 | Yes |
Table: GSEA details [plain text format]

  

Fig 2: KEGG\_HEDGEHOG\_SIGNALING\_PATHWAY      
 Blue-Pink O' Gram in the Space of the Analyzed GeneSet

  

Fig 3: KEGG\_HEDGEHOG\_SIGNALING\_PATHWAY: Random ES distribution      
 Gene set null distribution of ES for **KEGG\_HEDGEHOG\_SIGNALING\_PATHWAY**
