# Supplementary material for: Simultaneous Presentation of Multiple Myeloma and Lung Cancer: Case Report and Gene Bioinformatics Analysis
Source: Front Oncol. 2022 Jun 13;12:859735. doi: 10.3389/fonc.2022.859735 (PMC9235397; doi:10.3389/fonc.2022.859735)
Supplement: Supplementary file 1 [file DataSheet_1.zip › The bioinformatic analysis of MM and lung cancer supplementary materials/Enrichment analysis/MECR/GSEA_4.1.0/LUSC TCGA/KEGG.Gsea.1639042374131/KEGG_HEMATOPOIETIC_CELL_LINEAGE.html]

Details for gene set KEGG\_HEMATOPOIETIC\_CELL\_LINEAGE[GSEA]

|  || Dataset | ExpData\_collapsed\_to\_symbols.ENSG00000116353\_profile\_in\_ExpData.cls #ENSG00000116353 |
| Phenotype | ENSG00000116353\_profile\_in\_ExpData.cls#ENSG00000116353 |
| Upregulated in class | ENSG00000116353\_neg |
| GeneSet | KEGG\_HEMATOPOIETIC\_CELL\_LINEAGE |
| Enrichment Score (ES) | -0.50509167 |
| Normalized Enrichment Score (NES) | -2.0978222 |
| Nominal p-value | 0.0 |
| FDR q-value | 0.002319002 |
| FWER p-Value | 0.002 |
Table: GSEA Results Summary

  

Fig 1: Enrichment plot: KEGG\_HEMATOPOIETIC\_CELL\_LINEAGE      
 Profile of the Running ES Score & Positions of GeneSet Members on the Rank Ordered List

  

| SYMBOL | TITLE | RANK IN GENE LIST | RANK METRIC SCORE | RUNNING ES | CORE ENRICHMENT || 1 | ITGA3 | integrin subunit alpha 3 [Source:HGNC Symbol;Acc:HGNC:6139] | 2053 | 0.164 | -0.0310 | No |
| 2 | KIT | "KIT proto-oncogene, receptor tyrosine kinase [Source:HGNC Symbol;Acc:HGNC:6342]" | 4449 | 0.110 | -0.0777 | No |
| 3 | CSF3R | colony stimulating factor 3 receptor [Source:HGNC Symbol;Acc:HGNC:2439] | 5008 | 0.103 | -0.0785 | No |
| 4 | IL4R | interleukin 4 receptor [Source:HGNC Symbol;Acc:HGNC:6015] | 6304 | 0.091 | -0.0997 | No |
| 5 | EPOR | erythropoietin receptor [Source:HGNC Symbol;Acc:HGNC:3416] | 6351 | 0.090 | -0.0892 | No |
| 6 | IL4 | interleukin 4 [Source:HGNC Symbol;Acc:HGNC:6014] | 6756 | 0.086 | -0.0883 | No |
| 7 | TNF | tumor necrosis factor [Source:HGNC Symbol;Acc:HGNC:11892] | 7309 | 0.081 | -0.0918 | No |
| 8 | MME | membrane metalloendopeptidase [Source:HGNC Symbol;Acc:HGNC:7154] | 7533 | 0.079 | -0.0872 | No |
| 9 | IL3 | interleukin 3 [Source:HGNC Symbol;Acc:HGNC:6011] | 7558 | 0.079 | -0.0776 | No |
| 10 | CD59 | CD59 molecule (CD59 blood group) [Source:HGNC Symbol;Acc:HGNC:1689] | 7764 | 0.077 | -0.0728 | No |
| 11 | GYPA | glycophorin A (MNS blood group) [Source:HGNC Symbol;Acc:HGNC:4702] | 8585 | 0.070 | -0.0846 | No |
| 12 | IL11 | interleukin 11 [Source:HGNC Symbol;Acc:HGNC:5966] | 10308 | 0.058 | -0.1209 | No |
| 13 | GP9 | glycoprotein IX platelet [Source:HGNC Symbol;Acc:HGNC:4444] | 10868 | 0.054 | -0.1280 | No |
| 14 | FLT3LG | fms related receptor tyrosine kinase 3 ligand [Source:HGNC Symbol;Acc:HGNC:3766] | 11406 | 0.051 | -0.1351 | No |
| 15 | ITGB3 | integrin subunit beta 3 [Source:HGNC Symbol;Acc:HGNC:6156] | 15690 | 0.025 | -0.2410 | No |
| 16 | IL1A | interleukin 1 alpha [Source:HGNC Symbol;Acc:HGNC:5991] | 15828 | 0.024 | -0.2413 | No |
| 17 | CD55 | CD55 molecule (Cromer blood group) [Source:HGNC Symbol;Acc:HGNC:2665] | 15855 | 0.024 | -0.2388 | No |
| 18 | EPO | erythropoietin [Source:HGNC Symbol;Acc:HGNC:3415] | 17085 | 0.017 | -0.2679 | No |
| 19 | CSF3 | colony stimulating factor 3 [Source:HGNC Symbol;Acc:HGNC:2438] | 17260 | 0.016 | -0.2702 | No |
| 20 | CD36 | CD36 molecule [Source:HGNC Symbol;Acc:HGNC:1663] | 18170 | 0.011 | -0.2919 | No |
| 21 | CD8B | CD8b molecule [Source:HGNC Symbol;Acc:HGNC:1707] | 22034 | -0.011 | -0.3889 | No |
| 22 | IL7 | interleukin 7 [Source:HGNC Symbol;Acc:HGNC:6023] | 23197 | -0.019 | -0.4161 | No |
| 23 | CD1C | CD1c molecule [Source:HGNC Symbol;Acc:HGNC:1636] | 23222 | -0.019 | -0.4142 | No |
| 24 | CSF2 | colony stimulating factor 2 [Source:HGNC Symbol;Acc:HGNC:2434] | 23839 | -0.023 | -0.4270 | No |
| 25 | CSF2RA | colony stimulating factor 2 receptor subunit alpha [Source:HGNC Symbol;Acc:HGNC:2435] | 24630 | -0.028 | -0.4434 | No |
| 26 | CD1A | CD1a molecule [Source:HGNC Symbol;Acc:HGNC:1634] | 25310 | -0.033 | -0.4564 | No |
| 27 | HLA-DRA | "major histocompatibility complex, class II, DR alpha [Source:HGNC Symbol;Acc:HGNC:4947]" | 26550 | -0.042 | -0.4824 | No |
| 28 | CSF1 | colony stimulating factor 1 [Source:HGNC Symbol;Acc:HGNC:2432] | 26607 | -0.043 | -0.4783 | No |
| 29 | IL5RA | interleukin 5 receptor subunit alpha [Source:HGNC Symbol;Acc:HGNC:6017] | 26723 | -0.043 | -0.4756 | No |
| 30 | HLA-DRB1 | "major histocompatibility complex, class II, DR beta 1 [Source:HGNC Symbol;Acc:HGNC:4948]" | 26773 | -0.044 | -0.4711 | No |
| 31 | CD14 | CD14 molecule [Source:HGNC Symbol;Acc:HGNC:1628] | 27300 | -0.048 | -0.4783 | No |
| 32 | ITGA5 | integrin subunit alpha 5 [Source:HGNC Symbol;Acc:HGNC:6141] | 27755 | -0.051 | -0.4832 | No |
| 33 | ITGA2B | integrin subunit alpha 2b [Source:HGNC Symbol;Acc:HGNC:6138] | 27955 | -0.053 | -0.4814 | No |
| 34 | FCER2 | Fc fragment of IgE receptor II [Source:HGNC Symbol;Acc:HGNC:3612] | 28848 | -0.060 | -0.4963 | No |
| 35 | IL11RA | interleukin 11 receptor subunit alpha [Source:HGNC Symbol;Acc:HGNC:5967] | 28902 | -0.061 | -0.4897 | No |
| 36 | CD1D | CD1d molecule [Source:HGNC Symbol;Acc:HGNC:1637] | 29506 | -0.066 | -0.4965 | Yes |
| 37 | IL5 | interleukin 5 [Source:HGNC Symbol;Acc:HGNC:6016] | 29693 | -0.068 | -0.4924 | Yes |
| 38 | IL1B | interleukin 1 beta [Source:HGNC Symbol;Acc:HGNC:5992] | 29697 | -0.068 | -0.4836 | Yes |
| 39 | CD1E | CD1e molecule [Source:HGNC Symbol;Acc:HGNC:1638] | 29860 | -0.069 | -0.4787 | Yes |
| 40 | HLA-DRB5 | "major histocompatibility complex, class II, DR beta 5 [Source:HGNC Symbol;Acc:HGNC:4953]" | 29919 | -0.070 | -0.4711 | Yes |
| 41 | DNTT | DNA nucleotidylexotransferase [Source:HGNC Symbol;Acc:HGNC:2983] | 29939 | -0.070 | -0.4625 | Yes |
| 42 | IL6R | interleukin 6 receptor [Source:HGNC Symbol;Acc:HGNC:6019] | 30245 | -0.073 | -0.4607 | Yes |
| 43 | CSF1R | colony stimulating factor 1 receptor [Source:HGNC Symbol;Acc:HGNC:2433] | 30287 | -0.073 | -0.4522 | Yes |
| 44 | FCGR1A | Fc fragment of IgG receptor Ia [Source:HGNC Symbol;Acc:HGNC:3613] | 30441 | -0.075 | -0.4463 | Yes |
| 45 | CD4 | CD4 molecule [Source:HGNC Symbol;Acc:HGNC:1678] | 30573 | -0.076 | -0.4397 | Yes |
| 46 | CD33 | CD33 molecule [Source:HGNC Symbol;Acc:HGNC:1659] | 30708 | -0.077 | -0.4331 | Yes |
| 47 | FLT3 | fms related receptor tyrosine kinase 3 [Source:HGNC Symbol;Acc:HGNC:3765] | 30863 | -0.079 | -0.4267 | Yes |
| 48 | CD3D | CD3d molecule [Source:HGNC Symbol;Acc:HGNC:1673] | 31455 | -0.085 | -0.4307 | Yes |
| 49 | CD7 | CD7 molecule [Source:HGNC Symbol;Acc:HGNC:1695] | 31522 | -0.086 | -0.4213 | Yes |
| 50 | CR2 | complement C3d receptor 2 [Source:HGNC Symbol;Acc:HGNC:2336] | 31896 | -0.090 | -0.4191 | Yes |
| 51 | ANPEP | "alanyl aminopeptidase, membrane [Source:HGNC Symbol;Acc:HGNC:500]" | 32112 | -0.092 | -0.4126 | Yes |
| 52 | IL1R1 | interleukin 1 receptor type 1 [Source:HGNC Symbol;Acc:HGNC:5993] | 32518 | -0.097 | -0.4103 | Yes |
| 53 | ITGA4 | integrin subunit alpha 4 [Source:HGNC Symbol;Acc:HGNC:6140] | 32537 | -0.097 | -0.3981 | Yes |
| 54 | MS4A1 | membrane spanning 4-domains A1 [Source:HGNC Symbol;Acc:HGNC:7315] | 32688 | -0.099 | -0.3890 | Yes |
| 55 | CD2 | CD2 molecule [Source:HGNC Symbol;Acc:HGNC:1639] | 33236 | -0.107 | -0.3890 | Yes |
| 56 | TPO | thyroid peroxidase [Source:HGNC Symbol;Acc:HGNC:12015] | 33383 | -0.109 | -0.3786 | Yes |
| 57 | IL9R | interleukin 9 receptor [Source:HGNC Symbol;Acc:HGNC:6030] | 33398 | -0.109 | -0.3648 | Yes |
| 58 | CD19 | CD19 molecule [Source:HGNC Symbol;Acc:HGNC:1633] | 33399 | -0.109 | -0.3506 | Yes |
| 59 | CD8A | CD8a molecule [Source:HGNC Symbol;Acc:HGNC:1706] | 33518 | -0.111 | -0.3392 | Yes |
| 60 | GP5 | glycoprotein V platelet [Source:HGNC Symbol;Acc:HGNC:4443] | 33536 | -0.111 | -0.3251 | Yes |
| 61 | CD1B | CD1b molecule [Source:HGNC Symbol;Acc:HGNC:1635] | 33696 | -0.113 | -0.3145 | Yes |
| 62 | CD37 | CD37 molecule [Source:HGNC Symbol;Acc:HGNC:1666] | 33721 | -0.114 | -0.3003 | Yes |
| 63 | ITGAM | integrin subunit alpha M [Source:HGNC Symbol;Acc:HGNC:6149] | 33733 | -0.114 | -0.2857 | Yes |
| 64 | CD3E | CD3e molecule [Source:HGNC Symbol;Acc:HGNC:1674] | 33847 | -0.116 | -0.2735 | Yes |
| 65 | ITGA6 | integrin subunit alpha 6 [Source:HGNC Symbol;Acc:HGNC:6142] | 34278 | -0.122 | -0.2686 | Yes |
| 66 | CD34 | CD34 molecule [Source:HGNC Symbol;Acc:HGNC:1662] | 34380 | -0.124 | -0.2551 | Yes |
| 67 | CD3G | CD3g molecule [Source:HGNC Symbol;Acc:HGNC:1675] | 34390 | -0.124 | -0.2392 | Yes |
| 68 | IL1R2 | interleukin 1 receptor type 2 [Source:HGNC Symbol;Acc:HGNC:5994] | 34576 | -0.127 | -0.2273 | Yes |
| 69 | KITLG | KIT ligand [Source:HGNC Symbol;Acc:HGNC:6343] | 34776 | -0.131 | -0.2153 | Yes |
| 70 | CD22 | CD22 molecule [Source:HGNC Symbol;Acc:HGNC:1643] | 34875 | -0.133 | -0.2006 | Yes |
| 71 | IL2RA | interleukin 2 receptor subunit alpha [Source:HGNC Symbol;Acc:HGNC:6008] | 35087 | -0.137 | -0.1880 | Yes |
| 72 | ITGA2 | integrin subunit alpha 2 [Source:HGNC Symbol;Acc:HGNC:6137] | 35118 | -0.138 | -0.1708 | Yes |
| 73 | CR1 | complement C3b/C4b receptor 1 (Knops blood group) [Source:HGNC Symbol;Acc:HGNC:2334] | 35201 | -0.140 | -0.1547 | Yes |
| 74 | THPO | thrombopoietin [Source:HGNC Symbol;Acc:HGNC:11795] | 35346 | -0.143 | -0.1398 | Yes |
| 75 | CD38 | CD38 molecule [Source:HGNC Symbol;Acc:HGNC:1667] | 35362 | -0.143 | -0.1215 | Yes |
| 76 | CD5 | CD5 molecule [Source:HGNC Symbol;Acc:HGNC:1685] | 35625 | -0.150 | -0.1086 | Yes |
| 77 | IL7R | interleukin 7 receptor [Source:HGNC Symbol;Acc:HGNC:6024] | 35643 | -0.151 | -0.0894 | Yes |
| 78 | CD9 | CD9 molecule [Source:HGNC Symbol;Acc:HGNC:1709] | 35785 | -0.154 | -0.0729 | Yes |
| 79 | ITGA1 | integrin subunit alpha 1 [Source:HGNC Symbol;Acc:HGNC:6134] | 35922 | -0.158 | -0.0558 | Yes |
| 80 | IL3RA | interleukin 3 receptor subunit alpha [Source:HGNC Symbol;Acc:HGNC:6012] | 36082 | -0.162 | -0.0387 | Yes |
| 81 | IL6 | interleukin 6 [Source:HGNC Symbol;Acc:HGNC:6018] | 37409 | -0.218 | -0.0441 | Yes |
| 82 | CD44 | CD44 molecule (Indian blood group) [Source:HGNC Symbol;Acc:HGNC:1681] | 37470 | -0.221 | -0.0168 | Yes |
| 83 | GP1BA | glycoprotein Ib platelet subunit alpha [Source:HGNC Symbol;Acc:HGNC:4439] | 37665 | -0.235 | 0.0089 | Yes |
| 84 | TFRC | transferrin receptor [Source:HGNC Symbol;Acc:HGNC:11763] | 37809 | -0.246 | 0.0373 | Yes |
| 85 | GP1BB | glycoprotein Ib platelet subunit beta [Source:HGNC Symbol;Acc:HGNC:4440] | 39100 | NaN | 0.0057 | Yes |
Table: GSEA details [plain text format]

  

Fig 2: KEGG\_HEMATOPOIETIC\_CELL\_LINEAGE      
 Blue-Pink O' Gram in the Space of the Analyzed GeneSet

  

Fig 3: KEGG\_HEMATOPOIETIC\_CELL\_LINEAGE: Random ES distribution      
 Gene set null distribution of ES for **KEGG\_HEMATOPOIETIC\_CELL\_LINEAGE**
