# Supplementary material for: Simultaneous Presentation of Multiple Myeloma and Lung Cancer: Case Report and Gene Bioinformatics Analysis
Source: Front Oncol. 2022 Jun 13;12:859735. doi: 10.3389/fonc.2022.859735 (PMC9235397; doi:10.3389/fonc.2022.859735)
Supplement: Supplementary file 1 [file DataSheet_1.zip › The bioinformatic analysis of MM and lung cancer supplementary materials/Enrichment analysis/MECR/GSEA_4.1.0/LUSC TCGA/KEGG.Gsea.1639042374131/KEGG_HUNTINGTONS_DISEASE.html]

Details for gene set KEGG\_HUNTINGTONS\_DISEASE[GSEA]

|  || Dataset | ExpData\_collapsed\_to\_symbols.ENSG00000116353\_profile\_in\_ExpData.cls #ENSG00000116353 |
| Phenotype | ENSG00000116353\_profile\_in\_ExpData.cls#ENSG00000116353 |
| Upregulated in class | ENSG00000116353\_pos |
| GeneSet | KEGG\_HUNTINGTONS\_DISEASE |
| Enrichment Score (ES) | 0.508511 |
| Normalized Enrichment Score (NES) | 2.38021 |
| Nominal p-value | 0.0 |
| FDR q-value | 0.0 |
| FWER p-Value | 0.0 |
Table: GSEA Results Summary

  

Fig 1: Enrichment plot: KEGG\_HUNTINGTONS\_DISEASE      
 Profile of the Running ES Score & Positions of GeneSet Members on the Rank Ordered List

  

| SYMBOL | TITLE | RANK IN GENE LIST | RANK METRIC SCORE | RUNNING ES | CORE ENRICHMENT || 1 | SDHB | succinate dehydrogenase complex iron sulfur subunit B [Source:HGNC Symbol;Acc:HGNC:10681] | 66 | 0.423 | 0.0165 | Yes |
| 2 | NDUFS5 | NADH:ubiquinone oxidoreductase subunit S5 [Source:HGNC Symbol;Acc:HGNC:7712] | 85 | 0.408 | 0.0336 | Yes |
| 3 | NDUFA3 | NADH:ubiquinone oxidoreductase subunit A3 [Source:HGNC Symbol;Acc:HGNC:7686] | 141 | 0.368 | 0.0480 | Yes |
| 4 | UQCRH | ubiquinol-cytochrome c reductase hinge protein [Source:HGNC Symbol;Acc:HGNC:12590] | 167 | 0.353 | 0.0626 | Yes |
| 5 | NDUFS6 | NADH:ubiquinone oxidoreductase subunit S6 [Source:HGNC Symbol;Acc:HGNC:7713] | 194 | 0.343 | 0.0767 | Yes |
| 6 | ATP5PB | ATP synthase peripheral stalk-membrane subunit b [Source:HGNC Symbol;Acc:HGNC:840] | 213 | 0.335 | 0.0906 | Yes |
| 7 | POLR2K | "RNA polymerase II, I and III subunit K [Source:HGNC Symbol;Acc:HGNC:9198]" | 217 | 0.333 | 0.1048 | Yes |
| 8 | POLR2L | "RNA polymerase II, I and III subunit L [Source:HGNC Symbol;Acc:HGNC:9199]" | 221 | 0.331 | 0.1190 | Yes |
| 9 | HDAC1 | histone deacetylase 1 [Source:HGNC Symbol;Acc:HGNC:4852] | 246 | 0.325 | 0.1324 | Yes |
| 10 | CASP9 | caspase 9 [Source:HGNC Symbol;Acc:HGNC:1511] | 300 | 0.306 | 0.1442 | Yes |
| 11 | DNAL4 | dynein axonemal light chain 4 [Source:HGNC Symbol;Acc:HGNC:2955] | 359 | 0.293 | 0.1554 | Yes |
| 12 | UQCRHL | ubiquinol-cytochrome c reductase hinge protein like [Source:HGNC Symbol;Acc:HGNC:51714] | 377 | 0.290 | 0.1674 | Yes |
| 13 | AP2S1 | adaptor related protein complex 2 subunit sigma 1 [Source:HGNC Symbol;Acc:HGNC:565] | 427 | 0.281 | 0.1782 | Yes |
| 14 | SDHA | succinate dehydrogenase complex flavoprotein subunit A [Source:HGNC Symbol;Acc:HGNC:10680] | 452 | 0.277 | 0.1895 | Yes |
| 15 | POLR2G | RNA polymerase II subunit G [Source:HGNC Symbol;Acc:HGNC:9194] | 461 | 0.275 | 0.2012 | Yes |
| 16 | NDUFS8 | NADH:ubiquinone oxidoreductase core subunit S8 [Source:HGNC Symbol;Acc:HGNC:7715] | 512 | 0.268 | 0.2114 | Yes |
| 17 | ATP5PO | ATP synthase peripheral stalk subunit OSCP [Source:HGNC Symbol;Acc:HGNC:850] | 517 | 0.268 | 0.2229 | Yes |
| 18 | NDUFV1 | NADH:ubiquinone oxidoreductase core subunit V1 [Source:HGNC Symbol;Acc:HGNC:7716] | 525 | 0.266 | 0.2341 | Yes |
| 19 | BAX | "BCL2 associated X, apoptosis regulator [Source:HGNC Symbol;Acc:HGNC:959]" | 585 | 0.258 | 0.2437 | Yes |
| 20 | AP2A1 | adaptor related protein complex 2 subunit alpha 1 [Source:HGNC Symbol;Acc:HGNC:561] | 592 | 0.257 | 0.2546 | Yes |
| 21 | CYC1 | cytochrome c1 [Source:HGNC Symbol;Acc:HGNC:2579] | 644 | 0.252 | 0.2642 | Yes |
| 22 | ATP5F1E | ATP synthase F1 subunit epsilon [Source:HGNC Symbol;Acc:HGNC:838] | 731 | 0.241 | 0.2723 | Yes |
| 23 | UQCR10 | "ubiquinol-cytochrome c reductase, complex III subunit X [Source:HGNC Symbol;Acc:HGNC:30863]" | 976 | 0.217 | 0.2754 | Yes |
| 24 | POLR2I | RNA polymerase II subunit I [Source:HGNC Symbol;Acc:HGNC:9196] | 1025 | 0.213 | 0.2833 | Yes |
| 25 | NDUFA2 | NADH:ubiquinone oxidoreductase subunit A2 [Source:HGNC Symbol;Acc:HGNC:7685] | 1078 | 0.210 | 0.2910 | Yes |
| 26 | ATP5F1C | ATP synthase F1 subunit gamma [Source:HGNC Symbol;Acc:HGNC:833] | 1084 | 0.210 | 0.2999 | Yes |
| 27 | POLR2J | RNA polymerase II subunit J [Source:HGNC Symbol;Acc:HGNC:9197] | 1233 | 0.201 | 0.3048 | Yes |
| 28 | UQCRQ | ubiquinol-cytochrome c reductase complex III subunit VII [Source:HGNC Symbol;Acc:HGNC:29594] | 1244 | 0.200 | 0.3132 | Yes |
| 29 | NDUFC1 | NADH:ubiquinone oxidoreductase subunit C1 [Source:HGNC Symbol;Acc:HGNC:7705] | 1333 | 0.195 | 0.3193 | Yes |
| 30 | GPX1 | glutathione peroxidase 1 [Source:HGNC Symbol;Acc:HGNC:4553] | 1470 | 0.188 | 0.3239 | Yes |
| 31 | NDUFA10 | NADH:ubiquinone oxidoreductase subunit A10 [Source:HGNC Symbol;Acc:HGNC:7684] | 1484 | 0.187 | 0.3317 | Yes |
| 32 | COX6B2 | cytochrome c oxidase subunit 6B2 [Source:HGNC Symbol;Acc:HGNC:24380] | 1508 | 0.186 | 0.3391 | Yes |
| 33 | NDUFB8 | NADH:ubiquinone oxidoreductase subunit B8 [Source:HGNC Symbol;Acc:HGNC:7703] | 1568 | 0.183 | 0.3454 | Yes |
| 34 | ATP5MC2 | ATP synthase membrane subunit c locus 2 [Source:HGNC Symbol;Acc:HGNC:842] | 1577 | 0.183 | 0.3531 | Yes |
| 35 | UQCRB | ubiquinol-cytochrome c reductase binding protein [Source:HGNC Symbol;Acc:HGNC:12582] | 1639 | 0.181 | 0.3593 | Yes |
| 36 | COX8A | cytochrome c oxidase subunit 8A [Source:HGNC Symbol;Acc:HGNC:2294] | 1644 | 0.181 | 0.3670 | Yes |
| 37 | NDUFB3 | NADH:ubiquinone oxidoreductase subunit B3 [Source:HGNC Symbol;Acc:HGNC:7698] | 1692 | 0.178 | 0.3735 | Yes |
| 38 | UQCRC1 | ubiquinol-cytochrome c reductase core protein 1 [Source:HGNC Symbol;Acc:HGNC:12585] | 1764 | 0.175 | 0.3792 | Yes |
| 39 | NDUFB4 | NADH:ubiquinone oxidoreductase subunit B4 [Source:HGNC Symbol;Acc:HGNC:7699] | 1870 | 0.171 | 0.3838 | Yes |
| 40 | NDUFB9 | NADH:ubiquinone oxidoreductase subunit B9 [Source:HGNC Symbol;Acc:HGNC:7704] | 1879 | 0.171 | 0.3910 | Yes |
| 41 | NDUFB1 | NADH:ubiquinone oxidoreductase subunit B1 [Source:HGNC Symbol;Acc:HGNC:7695] | 1941 | 0.168 | 0.3966 | Yes |
| 42 | PLCB3 | phospholipase C beta 3 [Source:HGNC Symbol;Acc:HGNC:9056] | 1971 | 0.167 | 0.4030 | Yes |
| 43 | NDUFAB1 | NADH:ubiquinone oxidoreductase subunit AB1 [Source:HGNC Symbol;Acc:HGNC:7694] | 2058 | 0.164 | 0.4079 | Yes |
| 44 | NDUFB10 | NADH:ubiquinone oxidoreductase subunit B10 [Source:HGNC Symbol;Acc:HGNC:7696] | 2658 | 0.145 | 0.3988 | Yes |
| 45 | MT-CO1 | mitochondrially encoded cytochrome c oxidase I [Source:HGNC Symbol;Acc:HGNC:7419] | 2684 | 0.144 | 0.4044 | Yes |
| 46 | NDUFA9 | NADH:ubiquinone oxidoreductase subunit A9 [Source:HGNC Symbol;Acc:HGNC:7693] | 2762 | 0.142 | 0.4085 | Yes |
| 47 | POLR2C | RNA polymerase II subunit C [Source:HGNC Symbol;Acc:HGNC:9189] | 2813 | 0.141 | 0.4133 | Yes |
| 48 | NDUFS3 | NADH:ubiquinone oxidoreductase core subunit S3 [Source:HGNC Symbol;Acc:HGNC:7710] | 2883 | 0.140 | 0.4176 | Yes |
| 49 | SLC25A6 | solute carrier family 25 member 6 [Source:HGNC Symbol;Acc:HGNC:10992] | 2903 | 0.139 | 0.4231 | Yes |
| 50 | ATP5PF | ATP synthase peripheral stalk subunit F6 [Source:HGNC Symbol;Acc:HGNC:847] | 2949 | 0.138 | 0.4279 | Yes |
| 51 | COX4I1 | cytochrome c oxidase subunit 4I1 [Source:HGNC Symbol;Acc:HGNC:2265] | 3073 | 0.135 | 0.4305 | Yes |
| 52 | NDUFS7 | NADH:ubiquinone oxidoreductase core subunit S7 [Source:HGNC Symbol;Acc:HGNC:7714] | 3210 | 0.132 | 0.4327 | Yes |
| 53 | DCTN2 | dynactin subunit 2 [Source:HGNC Symbol;Acc:HGNC:2712] | 3348 | 0.129 | 0.4348 | Yes |
| 54 | COX7C | cytochrome c oxidase subunit 7C [Source:HGNC Symbol;Acc:HGNC:2292] | 3427 | 0.128 | 0.4383 | Yes |
| 55 | DNAL1 | dynein axonemal light chain 1 [Source:HGNC Symbol;Acc:HGNC:23247] | 3454 | 0.127 | 0.4431 | Yes |
| 56 | COX7A2 | cytochrome c oxidase subunit 7A2 [Source:HGNC Symbol;Acc:HGNC:2288] | 3468 | 0.127 | 0.4482 | Yes |
| 57 | NDUFB2 | NADH:ubiquinone oxidoreductase subunit B2 [Source:HGNC Symbol;Acc:HGNC:7697] | 3507 | 0.126 | 0.4527 | Yes |
| 58 | TFAM | "transcription factor A, mitochondrial [Source:HGNC Symbol;Acc:HGNC:11741]" | 3538 | 0.126 | 0.4573 | Yes |
| 59 | NDUFV2 | NADH:ubiquinone oxidoreductase core subunit V2 [Source:HGNC Symbol;Acc:HGNC:7717] | 3550 | 0.125 | 0.4624 | Yes |
| 60 | COX6B1 | cytochrome c oxidase subunit 6B1 [Source:HGNC Symbol;Acc:HGNC:2280] | 3572 | 0.125 | 0.4673 | Yes |
| 61 | NDUFB7 | NADH:ubiquinone oxidoreductase subunit B7 [Source:HGNC Symbol;Acc:HGNC:7702] | 3667 | 0.123 | 0.4702 | Yes |
| 62 | POLR2F | "RNA polymerase II, I and III subunit F [Source:HGNC Symbol;Acc:HGNC:9193]" | 3719 | 0.122 | 0.4741 | Yes |
| 63 | BBC3 | BCL2 binding component 3 [Source:HGNC Symbol;Acc:HGNC:17868] | 3940 | 0.119 | 0.4736 | Yes |
| 64 | NDUFC2 | NADH:ubiquinone oxidoreductase subunit C2 [Source:HGNC Symbol;Acc:HGNC:7706] | 3963 | 0.118 | 0.4782 | Yes |
| 65 | NDUFS4 | NADH:ubiquinone oxidoreductase subunit S4 [Source:HGNC Symbol;Acc:HGNC:7711] | 4123 | 0.116 | 0.4791 | Yes |
| 66 | COX4I2 | cytochrome c oxidase subunit 4I2 [Source:HGNC Symbol;Acc:HGNC:16232] | 4182 | 0.115 | 0.4825 | Yes |
| 67 | ATP5PD | ATP synthase peripheral stalk subunit d [Source:HGNC Symbol;Acc:HGNC:845] | 4285 | 0.113 | 0.4848 | Yes |
| 68 | SDHC | succinate dehydrogenase complex subunit C [Source:HGNC Symbol;Acc:HGNC:10682] | 4323 | 0.112 | 0.4887 | Yes |
| 69 | COX6C | cytochrome c oxidase subunit 6C [Source:HGNC Symbol;Acc:HGNC:2285] | 4326 | 0.112 | 0.4935 | Yes |
| 70 | MT-CO3 | mitochondrially encoded cytochrome c oxidase III [Source:HGNC Symbol;Acc:HGNC:7422] | 4561 | 0.109 | 0.4922 | Yes |
| 71 | CLTB | clathrin light chain B [Source:HGNC Symbol;Acc:HGNC:2091] | 4712 | 0.107 | 0.4929 | Yes |
| 72 | COX7A2L | cytochrome c oxidase subunit 7A2 like [Source:HGNC Symbol;Acc:HGNC:2289] | 4754 | 0.106 | 0.4965 | Yes |
| 73 | DNALI1 | dynein axonemal light intermediate chain 1 [Source:HGNC Symbol;Acc:HGNC:14353] | 4758 | 0.106 | 0.5009 | Yes |
| 74 | COX7B | cytochrome c oxidase subunit 7B [Source:HGNC Symbol;Acc:HGNC:2291] | 4828 | 0.105 | 0.5037 | Yes |
| 75 | COX6CP3 | cytochrome c oxidase subunit 6C pseudogene 3 [Source:HGNC Symbol;Acc:HGNC:31721] | 4943 | 0.104 | 0.5052 | Yes |
| 76 | COX6A1 | cytochrome c oxidase subunit 6A1 [Source:HGNC Symbol;Acc:HGNC:2277] | 5113 | 0.101 | 0.5053 | Yes |
| 77 | NDUFA4 | NDUFA4 mitochondrial complex associated [Source:HGNC Symbol;Acc:HGNC:7687] | 5480 | 0.097 | 0.5001 | Yes |
| 78 | UQCRC2 | ubiquinol-cytochrome c reductase core protein 2 [Source:HGNC Symbol;Acc:HGNC:12586] | 5923 | 0.095 | 0.4929 | Yes |
| 79 | ATP5MC1 | ATP synthase membrane subunit c locus 1 [Source:HGNC Symbol;Acc:HGNC:841] | 5965 | 0.094 | 0.4959 | Yes |
| 80 | CREB3L4 | cAMP responsive element binding protein 3 like 4 [Source:HGNC Symbol;Acc:HGNC:18854] | 5984 | 0.094 | 0.4995 | Yes |
| 81 | NDUFV3 | NADH:ubiquinone oxidoreductase subunit V3 [Source:HGNC Symbol;Acc:HGNC:7719] | 6013 | 0.094 | 0.5028 | Yes |
| 82 | NDUFA1 | NADH:ubiquinone oxidoreductase subunit A1 [Source:HGNC Symbol;Acc:HGNC:7683] | 6036 | 0.094 | 0.5063 | Yes |
| 83 | DNAI1 | dynein axonemal intermediate chain 1 [Source:HGNC Symbol;Acc:HGNC:2954] | 6106 | 0.093 | 0.5085 | Yes |
| 84 | COX6A2 | cytochrome c oxidase subunit 6A2 [Source:HGNC Symbol;Acc:HGNC:2279] | 6345 | 0.090 | 0.5063 | No |
| 85 | NDUFA6 | NADH:ubiquinone oxidoreductase subunit A6 [Source:HGNC Symbol;Acc:HGNC:7690] | 6634 | 0.087 | 0.5027 | No |
| 86 | COX7B2 | cytochrome c oxidase subunit 7B2 [Source:HGNC Symbol;Acc:HGNC:24381] | 6753 | 0.086 | 0.5034 | No |
| 87 | MT-CO2 | mitochondrially encoded cytochrome c oxidase II [Source:HGNC Symbol;Acc:HGNC:7421] | 7144 | 0.082 | 0.4970 | No |
| 88 | SLC25A5 | solute carrier family 25 member 5 [Source:HGNC Symbol;Acc:HGNC:10991] | 7217 | 0.082 | 0.4987 | No |
| 89 | NDUFA5 | NADH:ubiquinone oxidoreductase subunit A5 [Source:HGNC Symbol;Acc:HGNC:7688] | 7600 | 0.078 | 0.4923 | No |
| 90 | ATP5F1D | ATP synthase F1 subunit delta [Source:HGNC Symbol;Acc:HGNC:837] | 7651 | 0.078 | 0.4943 | No |
| 91 | ATP5F1A | ATP synthase F1 subunit alpha [Source:HGNC Symbol;Acc:HGNC:823] | 7734 | 0.077 | 0.4956 | No |
| 92 | POLR2E | "RNA polymerase II, I and III subunit E [Source:HGNC Symbol;Acc:HGNC:9192]" | 7792 | 0.077 | 0.4974 | No |
| 93 | COX5B | cytochrome c oxidase subunit 5B [Source:HGNC Symbol;Acc:HGNC:2269] | 8271 | 0.072 | 0.4883 | No |
| 94 | DNAH2 | dynein axonemal heavy chain 2 [Source:HGNC Symbol;Acc:HGNC:2948] | 8386 | 0.072 | 0.4885 | No |
| 95 | TBPL2 | TATA-box binding protein like 2 [Source:HGNC Symbol;Acc:HGNC:19841] | 8814 | 0.069 | 0.4805 | No |
| 96 | TGM2 | transglutaminase 2 [Source:HGNC Symbol;Acc:HGNC:11778] | 9074 | 0.067 | 0.4768 | No |
| 97 | IFT57 | intraflagellar transport 57 [Source:HGNC Symbol;Acc:HGNC:17367] | 9203 | 0.066 | 0.4763 | No |
| 98 | PPARGC1A | PPARG coactivator 1 alpha [Source:HGNC Symbol;Acc:HGNC:9237] | 9400 | 0.065 | 0.4741 | No |
| 99 | MT-CYB | mitochondrially encoded cytochrome b [Source:HGNC Symbol;Acc:HGNC:7427] | 9843 | 0.061 | 0.4654 | No |
| 100 | SLC25A4 | solute carrier family 25 member 4 [Source:HGNC Symbol;Acc:HGNC:10990] | 9848 | 0.061 | 0.4680 | No |
| 101 | UQCR11 | "ubiquinol-cytochrome c reductase, complex III subunit XI [Source:HGNC Symbol;Acc:HGNC:30862]" | 9868 | 0.061 | 0.4701 | No |
| 102 | DCTN1 | dynactin subunit 1 [Source:HGNC Symbol;Acc:HGNC:2711] | 10095 | 0.059 | 0.4669 | No |
| 103 | UQCRFS1 | "ubiquinol-cytochrome c reductase, Rieske iron-sulfur polypeptide 1 [Source:HGNC Symbol;Acc:HGNC:12587]" | 10480 | 0.057 | 0.4595 | No |
| 104 | HTT | huntingtin [Source:HGNC Symbol;Acc:HGNC:4851] | 10597 | 0.056 | 0.4590 | No |
| 105 | COX8C | cytochrome c oxidase subunit 8C [Source:HGNC Symbol;Acc:HGNC:24382] | 11354 | 0.051 | 0.4419 | No |
| 106 | MT-ATP6 | mitochondrially encoded ATP synthase membrane subunit 6 [Source:HGNC Symbol;Acc:HGNC:7414] | 11361 | 0.051 | 0.4439 | No |
| 107 | NDUFA7 | NADH:ubiquinone oxidoreductase subunit A7 [Source:HGNC Symbol;Acc:HGNC:7691] | 11506 | 0.050 | 0.4424 | No |
| 108 | TBP | TATA-box binding protein [Source:HGNC Symbol;Acc:HGNC:11588] | 11646 | 0.049 | 0.4410 | No |
| 109 | AP2M1 | adaptor related protein complex 2 subunit mu 1 [Source:HGNC Symbol;Acc:HGNC:564] | 11821 | 0.048 | 0.4386 | No |
| 110 | HDAC2 | histone deacetylase 2 [Source:HGNC Symbol;Acc:HGNC:4853] | 11971 | 0.047 | 0.4369 | No |
| 111 | NDUFS2 | NADH:ubiquinone oxidoreductase core subunit S2 [Source:HGNC Symbol;Acc:HGNC:7708] | 13107 | 0.040 | 0.4096 | No |
| 112 | GRIN1 | glutamate ionotropic receptor NMDA type subunit 1 [Source:HGNC Symbol;Acc:HGNC:4584] | 13266 | 0.039 | 0.4072 | No |
| 113 | VDAC1 | voltage dependent anion channel 1 [Source:HGNC Symbol;Acc:HGNC:12669] | 13291 | 0.039 | 0.4083 | No |
| 114 | POLR2B | RNA polymerase II subunit B [Source:HGNC Symbol;Acc:HGNC:9188] | 13423 | 0.038 | 0.4066 | No |
| 115 | ATP5F1B | ATP synthase F1 subunit beta [Source:HGNC Symbol;Acc:HGNC:830] | 14082 | 0.035 | 0.3913 | No |
| 116 | SLC25A31 | solute carrier family 25 member 31 [Source:HGNC Symbol;Acc:HGNC:25319] | 14788 | 0.030 | 0.3746 | No |
| 117 | TAF4 | TATA-box binding protein associated factor 4 [Source:HGNC Symbol;Acc:HGNC:11537] | 15013 | 0.029 | 0.3701 | No |
| 118 | NDUFB6 | NADH:ubiquinone oxidoreductase subunit B6 [Source:HGNC Symbol;Acc:HGNC:7701] | 15775 | 0.025 | 0.3517 | No |
| 119 | MT-ATP8 | mitochondrially encoded ATP synthase membrane subunit 8 [Source:HGNC Symbol;Acc:HGNC:7415] | 16163 | 0.022 | 0.3428 | No |
| 120 | CREB3L1 | cAMP responsive element binding protein 3 like 1 [Source:HGNC Symbol;Acc:HGNC:18856] | 16289 | 0.022 | 0.3406 | No |
| 121 | COX7A1 | cytochrome c oxidase subunit 7A1 [Source:HGNC Symbol;Acc:HGNC:2287] | 16527 | 0.020 | 0.3354 | No |
| 122 | NDUFA4L2 | NDUFA4 mitochondrial complex associated like 2 [Source:HGNC Symbol;Acc:HGNC:29836] | 16755 | 0.019 | 0.3304 | No |
| 123 | ATP5MC3 | ATP synthase membrane subunit c locus 3 [Source:HGNC Symbol;Acc:HGNC:843] | 16867 | 0.018 | 0.3283 | No |
| 124 | NDUFS1 | NADH:ubiquinone oxidoreductase core subunit S1 [Source:HGNC Symbol;Acc:HGNC:7707] | 17437 | 0.015 | 0.3145 | No |
| 125 | ATP5MC1P5 | ATP synthase membrane subunit c locus 1 pseudogene 5 [Source:HGNC Symbol;Acc:HGNC:39508] | 19858 | 0.002 | 0.2527 | No |
| 126 | PPID | peptidylprolyl isomerase D [Source:HGNC Symbol;Acc:HGNC:9257] | 20517 | -0.002 | 0.2360 | No |
| 127 | POLR2J2 | RNA polymerase II subunit J2 [Source:HGNC Symbol;Acc:HGNC:23208] | 20932 | -0.005 | 0.2256 | No |
| 128 | APAF1 | apoptotic peptidase activating factor 1 [Source:HGNC Symbol;Acc:HGNC:576] | 20948 | -0.005 | 0.2255 | No |
| 129 | VDAC2 | voltage dependent anion channel 2 [Source:HGNC Symbol;Acc:HGNC:12672] | 21450 | -0.008 | 0.2130 | No |
| 130 | AP2A2 | adaptor related protein complex 2 subunit alpha 2 [Source:HGNC Symbol;Acc:HGNC:562] | 21599 | -0.009 | 0.2096 | No |
| 131 | SOD1 | superoxide dismutase 1 [Source:HGNC Symbol;Acc:HGNC:11179] | 22374 | -0.014 | 0.1904 | No |
| 132 | COX5A | cytochrome c oxidase subunit 5A [Source:HGNC Symbol;Acc:HGNC:2267] | 22458 | -0.014 | 0.1889 | No |
| 133 | SDHD | succinate dehydrogenase complex subunit D [Source:HGNC Symbol;Acc:HGNC:10683] | 22998 | -0.017 | 0.1759 | No |
| 134 | RCOR1 | REST corepressor 1 [Source:HGNC Symbol;Acc:HGNC:17441] | 23566 | -0.021 | 0.1623 | No |
| 135 | POLR2A | RNA polymerase II subunit A [Source:HGNC Symbol;Acc:HGNC:9187] | 24164 | -0.025 | 0.1481 | No |
| 136 | CYCS | "cytochrome c, somatic [Source:HGNC Symbol;Acc:HGNC:19986]" | 24187 | -0.025 | 0.1486 | No |
| 137 | UQCR10P1 | UQCR10 pseudogene 1 [Source:HGNC Symbol;Acc:HGNC:54960] | 25693 | -0.036 | 0.1117 | No |
| 138 | VDAC3 | voltage dependent anion channel 3 [Source:HGNC Symbol;Acc:HGNC:12674] | 25866 | -0.037 | 0.1089 | No |
| 139 | POLR2J3 | RNA polymerase II subunit J3 [Source:HGNC Symbol;Acc:HGNC:33853] | 25994 | -0.038 | 0.1073 | No |
| 140 | VDAC2P5 | VDAC2 pseudogene 5 [Source:HGNC Symbol;Acc:HGNC:54753] | 26724 | -0.043 | 0.0906 | No |
| 141 | POLR2H | "RNA polymerase II, I and III subunit H [Source:HGNC Symbol;Acc:HGNC:9195]" | 26753 | -0.044 | 0.0917 | No |
| 142 | GRM5 | glutamate metabotropic receptor 5 [Source:HGNC Symbol;Acc:HGNC:4597] | 27139 | -0.047 | 0.0839 | No |
| 143 | AP2B1 | adaptor related protein complex 2 subunit beta 1 [Source:HGNC Symbol;Acc:HGNC:563] | 27392 | -0.049 | 0.0795 | No |
| 144 | PLCB1 | phospholipase C beta 1 [Source:HGNC Symbol;Acc:HGNC:15917] | 27781 | -0.052 | 0.0718 | No |
| 145 | DCTN4 | dynactin subunit 4 [Source:HGNC Symbol;Acc:HGNC:15518] | 27798 | -0.052 | 0.0737 | No |
| 146 | CASP8 | caspase 8 [Source:HGNC Symbol;Acc:HGNC:1509] | 27944 | -0.053 | 0.0722 | No |
| 147 | CREB3 | cAMP responsive element binding protein 3 [Source:HGNC Symbol;Acc:HGNC:2347] | 28414 | -0.057 | 0.0627 | No |
| 148 | CREB1 | cAMP responsive element binding protein 1 [Source:HGNC Symbol;Acc:HGNC:2345] | 28807 | -0.060 | 0.0552 | No |
| 149 | DNAI2 | dynein axonemal intermediate chain 2 [Source:HGNC Symbol;Acc:HGNC:18744] | 29474 | -0.066 | 0.0411 | No |
| 150 | NRF1 | nuclear respiratory factor 1 [Source:HGNC Symbol;Acc:HGNC:7996] | 29883 | -0.070 | 0.0336 | No |
| 151 | NDUFB5 | NADH:ubiquinone oxidoreductase subunit B5 [Source:HGNC Symbol;Acc:HGNC:7700] | 29913 | -0.070 | 0.0359 | No |
| 152 | CREB3L3 | cAMP responsive element binding protein 3 like 3 [Source:HGNC Symbol;Acc:HGNC:18855] | 30002 | -0.071 | 0.0367 | No |
| 153 | DNAH3 | dynein axonemal heavy chain 3 [Source:HGNC Symbol;Acc:HGNC:2949] | 30085 | -0.072 | 0.0377 | No |
| 154 | TP53 | tumor protein p53 [Source:HGNC Symbol;Acc:HGNC:11998] | 30908 | -0.079 | 0.0201 | No |
| 155 | GRIN2B | glutamate ionotropic receptor NMDA type subunit 2B [Source:HGNC Symbol;Acc:HGNC:4586] | 31248 | -0.083 | 0.0150 | No |
| 156 | POLR2D | RNA polymerase II subunit D [Source:HGNC Symbol;Acc:HGNC:9191] | 31342 | -0.084 | 0.0162 | No |
| 157 | PPARG | peroxisome proliferator activated receptor gamma [Source:HGNC Symbol;Acc:HGNC:9236] | 31491 | -0.085 | 0.0161 | No |
| 158 | CREB3L2 | cAMP responsive element binding protein 3 like 2 [Source:HGNC Symbol;Acc:HGNC:23720] | 31647 | -0.087 | 0.0159 | No |
| 159 | NDUFA8 | NADH:ubiquinone oxidoreductase subunit A8 [Source:HGNC Symbol;Acc:HGNC:7692] | 31722 | -0.088 | 0.0178 | No |
| 160 | DNAH1 | dynein axonemal heavy chain 1 [Source:HGNC Symbol;Acc:HGNC:2940] | 31908 | -0.090 | 0.0169 | No |
| 161 | PLCB2 | phospholipase C beta 2 [Source:HGNC Symbol;Acc:HGNC:9055] | 32369 | -0.095 | 0.0092 | No |
| 162 | TBPL1 | TATA-box binding protein like 1 [Source:HGNC Symbol;Acc:HGNC:11589] | 32392 | -0.095 | 0.0128 | No |
| 163 | CASP3 | caspase 3 [Source:HGNC Symbol;Acc:HGNC:1504] | 32939 | -0.103 | 0.0032 | No |
| 164 | DLG4 | discs large MAGUK scaffold protein 4 [Source:HGNC Symbol;Acc:HGNC:2903] | 33186 | -0.106 | 0.0015 | No |
| 165 | CLTA | clathrin light chain A [Source:HGNC Symbol;Acc:HGNC:2090] | 33839 | -0.116 | -0.0102 | No |
| 166 | TAF4B | TATA-box binding protein associated factor 4b [Source:HGNC Symbol;Acc:HGNC:11538] | 34768 | -0.131 | -0.0282 | No |
| 167 | CLTCL1 | clathrin heavy chain like 1 [Source:HGNC Symbol;Acc:HGNC:2093] | 34769 | -0.131 | -0.0226 | No |
| 168 | REST | RE1 silencing transcription factor [Source:HGNC Symbol;Acc:HGNC:9966] | 35336 | -0.143 | -0.0309 | No |
| 169 | CLTC | clathrin heavy chain [Source:HGNC Symbol;Acc:HGNC:2092] | 35421 | -0.145 | -0.0269 | No |
| 170 | SOD2 | superoxide dismutase 2 [Source:HGNC Symbol;Acc:HGNC:11180] | 35681 | -0.152 | -0.0270 | No |
| 171 | UCP1 | uncoupling protein 1 [Source:HGNC Symbol;Acc:HGNC:12517] | 35901 | -0.158 | -0.0258 | No |
| 172 | ITPR1 | "inositol 1,4,5-trisphosphate receptor type 1 [Source:HGNC Symbol;Acc:HGNC:6180]" | 36283 | -0.169 | -0.0282 | No |
| 173 | GNAQ | G protein subunit alpha q [Source:HGNC Symbol;Acc:HGNC:4390] | 36400 | -0.173 | -0.0237 | No |
| 174 | EP300 | E1A binding protein p300 [Source:HGNC Symbol;Acc:HGNC:3373] | 36409 | -0.173 | -0.0165 | No |
| 175 | SP1 | Sp1 transcription factor [Source:HGNC Symbol;Acc:HGNC:11205] | 37072 | -0.201 | -0.0248 | No |
| 176 | HIP1 | huntingtin interacting protein 1 [Source:HGNC Symbol;Acc:HGNC:4913] | 37404 | -0.218 | -0.0238 | No |
| 177 | CREBBP | CREB binding protein [Source:HGNC Symbol;Acc:HGNC:2348] | 37476 | -0.222 | -0.0161 | No |
| 178 | HAP1 | huntingtin associated protein 1 [Source:HGNC Symbol;Acc:HGNC:4812] | 37853 | -0.249 | -0.0150 | No |
| 179 | SIN3A | SIN3 transcription regulator family member A [Source:HGNC Symbol;Acc:HGNC:19353] | 37892 | -0.254 | -0.0051 | No |
| 180 | CREB5 | cAMP responsive element binding protein 5 [Source:HGNC Symbol;Acc:HGNC:16844] | 38284 | -0.305 | -0.0019 | No |
| 181 | PLCB4 | phospholipase C beta 4 [Source:HGNC Symbol;Acc:HGNC:9059] | 38308 | -0.309 | 0.0108 | No |
| 182 | BDNF | brain derived neurotrophic factor [Source:HGNC Symbol;Acc:HGNC:1033] | 38454 | -0.352 | 0.0222 | No |
Table: GSEA details [plain text format]

  

Fig 2: KEGG\_HUNTINGTONS\_DISEASE      
 Blue-Pink O' Gram in the Space of the Analyzed GeneSet

  

Fig 3: KEGG\_HUNTINGTONS\_DISEASE: Random ES distribution      
 Gene set null distribution of ES for **KEGG\_HUNTINGTONS\_DISEASE**
