# Supplementary material for: Simultaneous Presentation of Multiple Myeloma and Lung Cancer: Case Report and Gene Bioinformatics Analysis
Source: Front Oncol. 2022 Jun 13;12:859735. doi: 10.3389/fonc.2022.859735 (PMC9235397; doi:10.3389/fonc.2022.859735)
Supplement: Supplementary file 1 [file DataSheet_1.zip › The bioinformatic analysis of MM and lung cancer supplementary materials/Enrichment analysis/MECR/GSEA_4.1.0/LUSC TCGA/KEGG.Gsea.1639042374131/KEGG_INOSITOL_PHOSPHATE_METABOLISM.html]

Details for gene set KEGG\_INOSITOL\_PHOSPHATE\_METABOLISM[GSEA]

|  || Dataset | ExpData\_collapsed\_to\_symbols.ENSG00000116353\_profile\_in\_ExpData.cls #ENSG00000116353 |
| Phenotype | ENSG00000116353\_profile\_in\_ExpData.cls#ENSG00000116353 |
| Upregulated in class | ENSG00000116353\_neg |
| GeneSet | KEGG\_INOSITOL\_PHOSPHATE\_METABOLISM |
| Enrichment Score (ES) | -0.4760986 |
| Normalized Enrichment Score (NES) | -1.8296769 |
| Nominal p-value | 0.0020449897 |
| FDR q-value | 0.008656466 |
| FWER p-Value | 0.089 |
Table: GSEA Results Summary

  

Fig 1: Enrichment plot: KEGG\_INOSITOL\_PHOSPHATE\_METABOLISM      
 Profile of the Running ES Score & Positions of GeneSet Members on the Rank Ordered List

  

| SYMBOL | TITLE | RANK IN GENE LIST | RANK METRIC SCORE | RUNNING ES | CORE ENRICHMENT || 1 | ISYNA1 | inositol-3-phosphate synthase 1 [Source:HGNC Symbol;Acc:HGNC:29821] | 1227 | 0.202 | -0.0005 | No |
| 2 | INPP5B | inositol polyphosphate-5-phosphatase B [Source:HGNC Symbol;Acc:HGNC:6077] | 1305 | 0.197 | 0.0275 | No |
| 3 | PLCB3 | phospholipase C beta 3 [Source:HGNC Symbol;Acc:HGNC:9056] | 1971 | 0.167 | 0.0360 | No |
| 4 | INPP4B | inositol polyphosphate-4-phosphatase type II B [Source:HGNC Symbol;Acc:HGNC:6075] | 3228 | 0.132 | 0.0241 | No |
| 5 | PLCD3 | phospholipase C delta 3 [Source:HGNC Symbol;Acc:HGNC:9061] | 3547 | 0.126 | 0.0351 | No |
| 6 | IMPA1 | inositol monophosphatase 1 [Source:HGNC Symbol;Acc:HGNC:6050] | 4067 | 0.116 | 0.0396 | No |
| 7 | INPP5A | inositol polyphosphate-5-phosphatase A [Source:HGNC Symbol;Acc:HGNC:6076] | 4089 | 0.116 | 0.0568 | No |
| 8 | INPP1 | inositol polyphosphate-1-phosphatase [Source:HGNC Symbol;Acc:HGNC:6071] | 4119 | 0.116 | 0.0736 | No |
| 9 | CDIPT | CDP-diacylglycerol--inositol 3-phosphatidyltransferase [Source:HGNC Symbol;Acc:HGNC:1769] | 7957 | 0.075 | -0.0126 | No |
| 10 | SYNJ2 | synaptojanin 2 [Source:HGNC Symbol;Acc:HGNC:11504] | 8064 | 0.074 | -0.0040 | No |
| 11 | ALDH6A1 | aldehyde dehydrogenase 6 family member A1 [Source:HGNC Symbol;Acc:HGNC:7179] | 9302 | 0.065 | -0.0256 | No |
| 12 | PIP4K2C | phosphatidylinositol-5-phosphate 4-kinase type 2 gamma [Source:HGNC Symbol;Acc:HGNC:23786] | 9671 | 0.063 | -0.0254 | No |
| 13 | PTEN | phosphatase and tensin homolog [Source:HGNC Symbol;Acc:HGNC:9588] | 9897 | 0.061 | -0.0219 | No |
| 14 | PLCG1 | phospholipase C gamma 1 [Source:HGNC Symbol;Acc:HGNC:9065] | 13655 | 0.037 | -0.1119 | No |
| 15 | TPI1 | triosephosphate isomerase 1 [Source:HGNC Symbol;Acc:HGNC:12009] | 14369 | 0.033 | -0.1251 | No |
| 16 | MIOX | myo-inositol oxygenase [Source:HGNC Symbol;Acc:HGNC:14522] | 17788 | 0.013 | -0.2101 | No |
| 17 | PLCE1 | phospholipase C epsilon 1 [Source:HGNC Symbol;Acc:HGNC:17175] | 19244 | 0.005 | -0.2464 | No |
| 18 | PIK3CB | "phosphatidylinositol-4,5-bisphosphate 3-kinase catalytic subunit beta [Source:HGNC Symbol;Acc:HGNC:8976]" | 20368 | -0.002 | -0.2747 | No |
| 19 | MINPP1 | multiple inositol-polyphosphate phosphatase 1 [Source:HGNC Symbol;Acc:HGNC:7102] | 21579 | -0.009 | -0.3042 | No |
| 20 | ITPK1 | inositol-tetrakisphosphate 1-kinase [Source:HGNC Symbol;Acc:HGNC:6177] | 23438 | -0.020 | -0.3484 | No |
| 21 | IPMK | inositol polyphosphate multikinase [Source:HGNC Symbol;Acc:HGNC:20739] | 23554 | -0.021 | -0.3482 | No |
| 22 | INPPL1 | inositol polyphosphate phosphatase like 1 [Source:HGNC Symbol;Acc:HGNC:6080] | 23995 | -0.024 | -0.3558 | No |
| 23 | PIK3CD | "phosphatidylinositol-4,5-bisphosphate 3-kinase catalytic subunit delta [Source:HGNC Symbol;Acc:HGNC:8977]" | 24002 | -0.024 | -0.3523 | No |
| 24 | SYNJ1 | synaptojanin 1 [Source:HGNC Symbol;Acc:HGNC:11503] | 25444 | -0.034 | -0.3838 | No |
| 25 | INPP5K | inositol polyphosphate-5-phosphatase K [Source:HGNC Symbol;Acc:HGNC:33882] | 25816 | -0.037 | -0.3876 | No |
| 26 | PLCB1 | phospholipase C beta 1 [Source:HGNC Symbol;Acc:HGNC:15917] | 27781 | -0.052 | -0.4298 | No |
| 27 | INPP5J | inositol polyphosphate-5-phosphatase J [Source:HGNC Symbol;Acc:HGNC:8956] | 28392 | -0.056 | -0.4367 | No |
| 28 | IMPA2 | inositol monophosphatase 2 [Source:HGNC Symbol;Acc:HGNC:6051] | 28426 | -0.057 | -0.4289 | No |
| 29 | PI4KB | phosphatidylinositol 4-kinase beta [Source:HGNC Symbol;Acc:HGNC:8984] | 29012 | -0.062 | -0.4345 | No |
| 30 | PIK3C2G | phosphatidylinositol-4-phosphate 3-kinase catalytic subunit type 2 gamma [Source:HGNC Symbol;Acc:HGNC:8973] | 29981 | -0.071 | -0.4484 | No |
| 31 | PIP5K1C | phosphatidylinositol-4-phosphate 5-kinase type 1 gamma [Source:HGNC Symbol;Acc:HGNC:8996] | 30186 | -0.072 | -0.4425 | No |
| 32 | PIK3C2B | phosphatidylinositol-4-phosphate 3-kinase catalytic subunit type 2 beta [Source:HGNC Symbol;Acc:HGNC:8972] | 31042 | -0.081 | -0.4520 | No |
| 33 | PLCD4 | phospholipase C delta 4 [Source:HGNC Symbol;Acc:HGNC:9062] | 31989 | -0.091 | -0.4623 | Yes |
| 34 | PLCB2 | phospholipase C beta 2 [Source:HGNC Symbol;Acc:HGNC:9055] | 32369 | -0.095 | -0.4575 | Yes |
| 35 | PLCZ1 | phospholipase C zeta 1 [Source:HGNC Symbol;Acc:HGNC:19218] | 33095 | -0.105 | -0.4600 | Yes |
| 36 | PIK3CG | "phosphatidylinositol-4,5-bisphosphate 3-kinase catalytic subunit gamma [Source:HGNC Symbol;Acc:HGNC:8978]" | 33133 | -0.105 | -0.4449 | Yes |
| 37 | PI4KA | phosphatidylinositol 4-kinase alpha [Source:HGNC Symbol;Acc:HGNC:8983] | 33228 | -0.107 | -0.4311 | Yes |
| 38 | OCRL | OCRL inositol polyphosphate-5-phosphatase [Source:HGNC Symbol;Acc:HGNC:8108] | 34364 | -0.123 | -0.4412 | Yes |
| 39 | PLCG2 | phospholipase C gamma 2 [Source:HGNC Symbol;Acc:HGNC:9066] | 35650 | -0.151 | -0.4509 | Yes |
| 40 | PIP5K1A | phosphatidylinositol-4-phosphate 5-kinase type 1 alpha [Source:HGNC Symbol;Acc:HGNC:8994] | 35739 | -0.153 | -0.4299 | Yes |
| 41 | PIP4K2A | phosphatidylinositol-5-phosphate 4-kinase type 2 alpha [Source:HGNC Symbol;Acc:HGNC:8997] | 35842 | -0.156 | -0.4087 | Yes |
| 42 | PIK3C3 | phosphatidylinositol 3-kinase catalytic subunit type 3 [Source:HGNC Symbol;Acc:HGNC:8974] | 36105 | -0.163 | -0.3905 | Yes |
| 43 | INPP4A | inositol polyphosphate-4-phosphatase type I A [Source:HGNC Symbol;Acc:HGNC:6074] | 36783 | -0.188 | -0.3792 | Yes |
| 44 | INPP5E | inositol polyphosphate-5-phosphatase E [Source:HGNC Symbol;Acc:HGNC:21474] | 36989 | -0.197 | -0.3544 | Yes |
| 45 | PIP4K2B | phosphatidylinositol-5-phosphate 4-kinase type 2 beta [Source:HGNC Symbol;Acc:HGNC:8998] | 37226 | -0.209 | -0.3287 | Yes |
| 46 | PIKFYVE | "phosphoinositide kinase, FYVE-type zinc finger containing [Source:HGNC Symbol;Acc:HGNC:23785]" | 37420 | -0.218 | -0.3004 | Yes |
| 47 | PIP5K1B | phosphatidylinositol-4-phosphate 5-kinase type 1 beta [Source:HGNC Symbol;Acc:HGNC:8995] | 37463 | -0.221 | -0.2678 | Yes |
| 48 | ITPKA | inositol-trisphosphate 3-kinase A [Source:HGNC Symbol;Acc:HGNC:6178] | 37678 | -0.236 | -0.2373 | Yes |
| 49 | IPPK | inositol-pentakisphosphate 2-kinase [Source:HGNC Symbol;Acc:HGNC:14645] | 37705 | -0.238 | -0.2018 | Yes |
| 50 | PLCD1 | phospholipase C delta 1 [Source:HGNC Symbol;Acc:HGNC:9060] | 38013 | -0.267 | -0.1690 | Yes |
| 51 | PIK3CA | "phosphatidylinositol-4,5-bisphosphate 3-kinase catalytic subunit alpha [Source:HGNC Symbol;Acc:HGNC:8975]" | 38226 | -0.296 | -0.1293 | Yes |
| 52 | PIK3C2A | phosphatidylinositol-4-phosphate 3-kinase catalytic subunit type 2 alpha [Source:HGNC Symbol;Acc:HGNC:8971] | 38233 | -0.297 | -0.0842 | Yes |
| 53 | PLCB4 | phospholipase C beta 4 [Source:HGNC Symbol;Acc:HGNC:9059] | 38308 | -0.309 | -0.0390 | Yes |
| 54 | ITPKB | inositol-trisphosphate 3-kinase B [Source:HGNC Symbol;Acc:HGNC:6179] | 38542 | -0.426 | 0.0199 | Yes |
Table: GSEA details [plain text format]

  

Fig 2: KEGG\_INOSITOL\_PHOSPHATE\_METABOLISM      
 Blue-Pink O' Gram in the Space of the Analyzed GeneSet

  

Fig 3: KEGG\_INOSITOL\_PHOSPHATE\_METABOLISM: Random ES distribution      
 Gene set null distribution of ES for **KEGG\_INOSITOL\_PHOSPHATE\_METABOLISM**
