# Supplementary material for: Simultaneous Presentation of Multiple Myeloma and Lung Cancer: Case Report and Gene Bioinformatics Analysis
Source: Front Oncol. 2022 Jun 13;12:859735. doi: 10.3389/fonc.2022.859735 (PMC9235397; doi:10.3389/fonc.2022.859735)
Supplement: Supplementary file 1 [file DataSheet_1.zip › The bioinformatic analysis of MM and lung cancer supplementary materials/Enrichment analysis/MECR/GSEA_4.1.0/LUSC TCGA/KEGG.Gsea.1639042374131/KEGG_LONG_TERM_DEPRESSION.html]

Details for gene set KEGG\_LONG\_TERM\_DEPRESSION[GSEA]

|  || Dataset | ExpData\_collapsed\_to\_symbols.ENSG00000116353\_profile\_in\_ExpData.cls #ENSG00000116353 |
| Phenotype | ENSG00000116353\_profile\_in\_ExpData.cls#ENSG00000116353 |
| Upregulated in class | ENSG00000116353\_neg |
| GeneSet | KEGG\_LONG\_TERM\_DEPRESSION |
| Enrichment Score (ES) | -0.43450207 |
| Normalized Enrichment Score (NES) | -1.7333926 |
| Nominal p-value | 0.0 |
| FDR q-value | 0.019222088 |
| FWER p-Value | 0.236 |
Table: GSEA Results Summary

  

Fig 1: Enrichment plot: KEGG\_LONG\_TERM\_DEPRESSION      
 Profile of the Running ES Score & Positions of GeneSet Members on the Rank Ordered List

  

| SYMBOL | TITLE | RANK IN GENE LIST | RANK METRIC SCORE | RUNNING ES | CORE ENRICHMENT || 1 | MAPK3 | mitogen-activated protein kinase 3 [Source:HGNC Symbol;Acc:HGNC:6877] | 473 | 0.274 | 0.0211 | No |
| 2 | PPP2R1A | protein phosphatase 2 scaffold subunit Aalpha [Source:HGNC Symbol;Acc:HGNC:9302] | 486 | 0.272 | 0.0538 | No |
| 3 | PLA2G12A | phospholipase A2 group XIIA [Source:HGNC Symbol;Acc:HGNC:18554] | 582 | 0.258 | 0.0826 | No |
| 4 | NRAS | "NRAS proto-oncogene, GTPase [Source:HGNC Symbol;Acc:HGNC:7989]" | 774 | 0.235 | 0.1062 | No |
| 5 | GNAI3 | G protein subunit alpha i3 [Source:HGNC Symbol;Acc:HGNC:4387] | 869 | 0.226 | 0.1311 | No |
| 6 | PLCB3 | phospholipase C beta 3 [Source:HGNC Symbol;Acc:HGNC:9056] | 1971 | 0.167 | 0.1232 | No |
| 7 | GNAS | GNAS complex locus [Source:HGNC Symbol;Acc:HGNC:4392] | 4003 | 0.118 | 0.0857 | No |
| 8 | PLA2G2C | phospholipase A2 group IIC [Source:HGNC Symbol;Acc:HGNC:9032] | 4582 | 0.109 | 0.0841 | No |
| 9 | PLA2G1B | phospholipase A2 group IB [Source:HGNC Symbol;Acc:HGNC:9030] | 4747 | 0.106 | 0.0928 | No |
| 10 | ARAF | "A-Raf proto-oncogene, serine/threonine kinase [Source:HGNC Symbol;Acc:HGNC:646]" | 5337 | 0.099 | 0.0897 | No |
| 11 | CACNA1A | calcium voltage-gated channel subunit alpha1 A [Source:HGNC Symbol;Acc:HGNC:1388] | 8843 | 0.068 | 0.0087 | No |
| 12 | CRH | corticotropin releasing hormone [Source:HGNC Symbol;Acc:HGNC:2355] | 8973 | 0.067 | 0.0136 | No |
| 13 | PLA2G12B | phospholipase A2 group XIIB [Source:HGNC Symbol;Acc:HGNC:18555] | 9402 | 0.065 | 0.0105 | No |
| 14 | PLA2G2E | phospholipase A2 group IIE [Source:HGNC Symbol;Acc:HGNC:13414] | 9748 | 0.062 | 0.0092 | No |
| 15 | PLA2G2F | phospholipase A2 group IIF [Source:HGNC Symbol;Acc:HGNC:30040] | 10874 | 0.054 | -0.0129 | No |
| 16 | GNAI1 | G protein subunit alpha i1 [Source:HGNC Symbol;Acc:HGNC:4384] | 11240 | 0.052 | -0.0159 | No |
| 17 | GRIA2 | glutamate ionotropic receptor AMPA type subunit 2 [Source:HGNC Symbol;Acc:HGNC:4572] | 11976 | 0.047 | -0.0289 | No |
| 18 | GRID2 | glutamate ionotropic receptor delta type subunit 2 [Source:HGNC Symbol;Acc:HGNC:4576] | 12687 | 0.043 | -0.0418 | No |
| 19 | PPP2CA | protein phosphatase 2 catalytic subunit alpha [Source:HGNC Symbol;Acc:HGNC:9299] | 13727 | 0.037 | -0.0638 | No |
| 20 | GNAI2 | G protein subunit alpha i2 [Source:HGNC Symbol;Acc:HGNC:4385] | 15092 | 0.029 | -0.0951 | No |
| 21 | PRKCG | protein kinase C gamma [Source:HGNC Symbol;Acc:HGNC:9402] | 15351 | 0.027 | -0.0984 | No |
| 22 | MAP2K2 | mitogen-activated protein kinase kinase 2 [Source:HGNC Symbol;Acc:HGNC:6842] | 15510 | 0.026 | -0.0993 | No |
| 23 | PLA2G5 | phospholipase A2 group V [Source:HGNC Symbol;Acc:HGNC:9038] | 15541 | 0.026 | -0.0969 | No |
| 24 | RAF1 | "Raf-1 proto-oncogene, serine/threonine kinase [Source:HGNC Symbol;Acc:HGNC:9829]" | 16140 | 0.022 | -0.1094 | No |
| 25 | PPP1R17 | protein phosphatase 1 regulatory subunit 17 [Source:HGNC Symbol;Acc:HGNC:16973] | 19274 | 0.005 | -0.1886 | No |
| 26 | HRAS | "HRas proto-oncogene, GTPase [Source:HGNC Symbol;Acc:HGNC:5173]" | 20556 | -0.003 | -0.2209 | No |
| 27 | GNA12 | G protein subunit alpha 12 [Source:HGNC Symbol;Acc:HGNC:4380] | 24506 | -0.027 | -0.3182 | No |
| 28 | PPP2CB | protein phosphatase 2 catalytic subunit beta [Source:HGNC Symbol;Acc:HGNC:9300] | 25064 | -0.032 | -0.3286 | No |
| 29 | PLA2G6 | phospholipase A2 group VI [Source:HGNC Symbol;Acc:HGNC:9039] | 25215 | -0.033 | -0.3284 | No |
| 30 | PLA2G4A | phospholipase A2 group IVA [Source:HGNC Symbol;Acc:HGNC:9035] | 25224 | -0.033 | -0.3247 | No |
| 31 | GRIA1 | glutamate ionotropic receptor AMPA type subunit 1 [Source:HGNC Symbol;Acc:HGNC:4571] | 25381 | -0.034 | -0.3245 | No |
| 32 | GRM5 | glutamate metabotropic receptor 5 [Source:HGNC Symbol;Acc:HGNC:4597] | 27139 | -0.047 | -0.3637 | No |
| 33 | PLCB1 | phospholipase C beta 1 [Source:HGNC Symbol;Acc:HGNC:15917] | 27781 | -0.052 | -0.3738 | No |
| 34 | GNA11 | G protein subunit alpha 11 [Source:HGNC Symbol;Acc:HGNC:4379] | 28013 | -0.054 | -0.3732 | No |
| 35 | GNAO1 | G protein subunit alpha o1 [Source:HGNC Symbol;Acc:HGNC:4389] | 28043 | -0.054 | -0.3674 | No |
| 36 | PLA2G2A | phospholipase A2 group IIA [Source:HGNC Symbol;Acc:HGNC:9031] | 29183 | -0.063 | -0.3888 | No |
| 37 | ITPR3 | "inositol 1,4,5-trisphosphate receptor type 3 [Source:HGNC Symbol;Acc:HGNC:6182]" | 29471 | -0.066 | -0.3881 | No |
| 38 | PRKG2 | protein kinase cGMP-dependent 2 [Source:HGNC Symbol;Acc:HGNC:9416] | 30839 | -0.079 | -0.4135 | No |
| 39 | GRIA3 | glutamate ionotropic receptor AMPA type subunit 3 [Source:HGNC Symbol;Acc:HGNC:4573] | 30996 | -0.080 | -0.4077 | No |
| 40 | MAP2K1 | mitogen-activated protein kinase kinase 1 [Source:HGNC Symbol;Acc:HGNC:6840] | 32048 | -0.091 | -0.4234 | Yes |
| 41 | PLCB2 | phospholipase C beta 2 [Source:HGNC Symbol;Acc:HGNC:9055] | 32369 | -0.095 | -0.4201 | Yes |
| 42 | GUCY1A2 | guanylate cyclase 1 soluble subunit alpha 2 [Source:HGNC Symbol;Acc:HGNC:4684] | 32385 | -0.095 | -0.4089 | Yes |
| 43 | RYR1 | ryanodine receptor 1 [Source:HGNC Symbol;Acc:HGNC:10483] | 32462 | -0.096 | -0.3992 | Yes |
| 44 | PLA2G10 | phospholipase A2 group X [Source:HGNC Symbol;Acc:HGNC:9029] | 32963 | -0.103 | -0.3995 | Yes |
| 45 | MAPK1 | mitogen-activated protein kinase 1 [Source:HGNC Symbol;Acc:HGNC:6871] | 33350 | -0.108 | -0.3962 | Yes |
| 46 | BRAF | "B-Raf proto-oncogene, serine/threonine kinase [Source:HGNC Symbol;Acc:HGNC:1097]" | 33362 | -0.108 | -0.3834 | Yes |
| 47 | IGF1 | insulin like growth factor 1 [Source:HGNC Symbol;Acc:HGNC:5464] | 33450 | -0.110 | -0.3723 | Yes |
| 48 | GNAZ | G protein subunit alpha z [Source:HGNC Symbol;Acc:HGNC:4395] | 33788 | -0.115 | -0.3670 | Yes |
| 49 | ITPR2 | "inositol 1,4,5-trisphosphate receptor type 2 [Source:HGNC Symbol;Acc:HGNC:6181]" | 34058 | -0.119 | -0.3595 | Yes |
| 50 | PRKCB | protein kinase C beta [Source:HGNC Symbol;Acc:HGNC:9395] | 34603 | -0.128 | -0.3579 | Yes |
| 51 | PLA2G4B | phospholipase A2 group IVB [Source:HGNC Symbol;Acc:HGNC:9036] | 34964 | -0.135 | -0.3508 | Yes |
| 52 | PPP2R1B | protein phosphatase 2 scaffold subunit Abeta [Source:HGNC Symbol;Acc:HGNC:9303] | 35047 | -0.137 | -0.3364 | Yes |
| 53 | PRKCA | protein kinase C alpha [Source:HGNC Symbol;Acc:HGNC:9393] | 35223 | -0.140 | -0.3239 | Yes |
| 54 | GUCY1A1 | guanylate cyclase 1 soluble subunit alpha 1 [Source:HGNC Symbol;Acc:HGNC:4685] | 35261 | -0.141 | -0.3078 | Yes |
| 55 | LYN | "LYN proto-oncogene, Src family tyrosine kinase [Source:HGNC Symbol;Acc:HGNC:6735]" | 35435 | -0.145 | -0.2946 | Yes |
| 56 | JMJD7-PLA2G4B | JMJD7-PLA2G4B readthrough [Source:HGNC Symbol;Acc:HGNC:34449] | 36012 | -0.161 | -0.2899 | Yes |
| 57 | GUCY1B1 | guanylate cyclase 1 soluble subunit beta 1 [Source:HGNC Symbol;Acc:HGNC:4687] | 36017 | -0.161 | -0.2705 | Yes |
| 58 | IGF1R | insulin like growth factor 1 receptor [Source:HGNC Symbol;Acc:HGNC:5465] | 36113 | -0.163 | -0.2532 | Yes |
| 59 | PLA2G3 | phospholipase A2 group III [Source:HGNC Symbol;Acc:HGNC:17934] | 36216 | -0.167 | -0.2356 | Yes |
| 60 | ITPR1 | "inositol 1,4,5-trisphosphate receptor type 1 [Source:HGNC Symbol;Acc:HGNC:6180]" | 36283 | -0.169 | -0.2168 | Yes |
| 61 | PLA2G4E | phospholipase A2 group IVE [Source:HGNC Symbol;Acc:HGNC:24791] | 36372 | -0.172 | -0.1982 | Yes |
| 62 | GNAQ | G protein subunit alpha q [Source:HGNC Symbol;Acc:HGNC:4390] | 36400 | -0.173 | -0.1780 | Yes |
| 63 | PRKG1 | protein kinase cGMP-dependent 1 [Source:HGNC Symbol;Acc:HGNC:9414] | 36496 | -0.176 | -0.1590 | Yes |
| 64 | KRAS | "KRAS proto-oncogene, GTPase [Source:HGNC Symbol;Acc:HGNC:6407]" | 36928 | -0.194 | -0.1465 | Yes |
| 65 | PLA2G2D | phospholipase A2 group IID [Source:HGNC Symbol;Acc:HGNC:9033] | 36979 | -0.196 | -0.1240 | Yes |
| 66 | CRHR1 | corticotropin releasing hormone receptor 1 [Source:HGNC Symbol;Acc:HGNC:2357] | 37136 | -0.204 | -0.1033 | Yes |
| 67 | NOS1 | nitric oxide synthase 1 [Source:HGNC Symbol;Acc:HGNC:7872] | 38042 | -0.270 | -0.0937 | Yes |
| 68 | PLCB4 | phospholipase C beta 4 [Source:HGNC Symbol;Acc:HGNC:9059] | 38308 | -0.309 | -0.0631 | Yes |
| 69 | GRM1 | glutamate metabotropic receptor 1 [Source:HGNC Symbol;Acc:HGNC:4593] | 38453 | -0.351 | -0.0242 | Yes |
| 70 | GNA13 | G protein subunit alpha 13 [Source:HGNC Symbol;Acc:HGNC:4381] | 38514 | -0.383 | 0.0206 | Yes |
Table: GSEA details [plain text format]

  

Fig 2: KEGG\_LONG\_TERM\_DEPRESSION      
 Blue-Pink O' Gram in the Space of the Analyzed GeneSet

  

Fig 3: KEGG\_LONG\_TERM\_DEPRESSION: Random ES distribution      
 Gene set null distribution of ES for **KEGG\_LONG\_TERM\_DEPRESSION**
