# Supplementary material for: Simultaneous Presentation of Multiple Myeloma and Lung Cancer: Case Report and Gene Bioinformatics Analysis
Source: Front Oncol. 2022 Jun 13;12:859735. doi: 10.3389/fonc.2022.859735 (PMC9235397; doi:10.3389/fonc.2022.859735)
Supplement: Supplementary file 1 [file DataSheet_1.zip › The bioinformatic analysis of MM and lung cancer supplementary materials/Enrichment analysis/MECR/GSEA_4.1.0/LUSC TCGA/KEGG.Gsea.1639042374131/KEGG_MAPK_SIGNALING_PATHWAY.html]

Details for gene set KEGG\_MAPK\_SIGNALING\_PATHWAY[GSEA]

|  || Dataset | ExpData\_collapsed\_to\_symbols.ENSG00000116353\_profile\_in\_ExpData.cls #ENSG00000116353 |
| Phenotype | ENSG00000116353\_profile\_in\_ExpData.cls#ENSG00000116353 |
| Upregulated in class | ENSG00000116353\_neg |
| GeneSet | KEGG\_MAPK\_SIGNALING\_PATHWAY |
| Enrichment Score (ES) | -0.37608972 |
| Normalized Enrichment Score (NES) | -1.8601123 |
| Nominal p-value | 0.0 |
| FDR q-value | 0.00733915 |
| FWER p-Value | 0.064 |
Table: GSEA Results Summary

  

Fig 1: Enrichment plot: KEGG\_MAPK\_SIGNALING\_PATHWAY      
 Profile of the Running ES Score & Positions of GeneSet Members on the Rank Ordered List

  

| SYMBOL | TITLE | RANK IN GENE LIST | RANK METRIC SCORE | RUNNING ES | CORE ENRICHMENT || 1 | CDC42 | cell division cycle 42 [Source:HGNC Symbol;Acc:HGNC:1736] | 81 | 0.411 | 0.0118 | No |
| 2 | RPS6KA1 | ribosomal protein S6 kinase A1 [Source:HGNC Symbol;Acc:HGNC:10430] | 185 | 0.348 | 0.0209 | No |
| 3 | STMN1 | stathmin 1 [Source:HGNC Symbol;Acc:HGNC:6510] | 251 | 0.324 | 0.0302 | No |
| 4 | RRAS | RAS related [Source:HGNC Symbol;Acc:HGNC:10447] | 436 | 0.280 | 0.0350 | No |
| 5 | MAPK3 | mitogen-activated protein kinase 3 [Source:HGNC Symbol;Acc:HGNC:6877] | 473 | 0.274 | 0.0433 | No |
| 6 | MAP3K6 | mitogen-activated protein kinase kinase kinase 6 [Source:HGNC Symbol;Acc:HGNC:6858] | 559 | 0.261 | 0.0500 | No |
| 7 | PLA2G12A | phospholipase A2 group XIIA [Source:HGNC Symbol;Acc:HGNC:18554] | 582 | 0.258 | 0.0581 | No |
| 8 | NRAS | "NRAS proto-oncogene, GTPase [Source:HGNC Symbol;Acc:HGNC:7989]" | 774 | 0.235 | 0.0612 | No |
| 9 | PPP5C | protein phosphatase 5 catalytic subunit [Source:HGNC Symbol;Acc:HGNC:9322] | 778 | 0.234 | 0.0690 | No |
| 10 | HSPA1A | heat shock protein family A (Hsp70) member 1A [Source:HGNC Symbol;Acc:HGNC:5232] | 918 | 0.221 | 0.0729 | No |
| 11 | MAPK8 | mitogen-activated protein kinase 8 [Source:HGNC Symbol;Acc:HGNC:6881] | 948 | 0.218 | 0.0796 | No |
| 12 | PTPRR | protein tyrosine phosphatase receptor type R [Source:HGNC Symbol;Acc:HGNC:9680] | 1117 | 0.207 | 0.0823 | No |
| 13 | HSPA2 | heat shock protein family A (Hsp70) member 2 [Source:HGNC Symbol;Acc:HGNC:5235] | 1167 | 0.205 | 0.0879 | No |
| 14 | RPS6KA4 | ribosomal protein S6 kinase A4 [Source:HGNC Symbol;Acc:HGNC:10433] | 1636 | 0.181 | 0.0821 | No |
| 15 | LAMTOR3 | "late endosomal/lysosomal adaptor, MAPK and MTOR activator 3 [Source:HGNC Symbol;Acc:HGNC:15606]" | 1774 | 0.174 | 0.0844 | No |
| 16 | RAC3 | Rac family small GTPase 3 [Source:HGNC Symbol;Acc:HGNC:9803] | 1777 | 0.174 | 0.0903 | No |
| 17 | GNG12 | G protein subunit gamma 12 [Source:HGNC Symbol;Acc:HGNC:19663] | 1849 | 0.172 | 0.0943 | No |
| 18 | RAP1A | "RAP1A, member of RAS oncogene family [Source:HGNC Symbol;Acc:HGNC:9855]" | 1949 | 0.167 | 0.0974 | No |
| 19 | GADD45A | growth arrest and DNA damage inducible alpha [Source:HGNC Symbol;Acc:HGNC:4095] | 1977 | 0.166 | 0.1023 | No |
| 20 | TAB1 | TGF-beta activated kinase 1 (MAP3K7) binding protein 1 [Source:HGNC Symbol;Acc:HGNC:18157] | 2416 | 0.152 | 0.0962 | No |
| 21 | PPP3CA | protein phosphatase 3 catalytic subunit alpha [Source:HGNC Symbol;Acc:HGNC:9314] | 2508 | 0.149 | 0.0989 | No |
| 22 | MAP3K11 | mitogen-activated protein kinase kinase kinase 11 [Source:HGNC Symbol;Acc:HGNC:6850] | 2654 | 0.145 | 0.1001 | No |
| 23 | FGF11 | fibroblast growth factor 11 [Source:HGNC Symbol;Acc:HGNC:3667] | 2838 | 0.141 | 0.1002 | No |
| 24 | AKT2 | AKT serine/threonine kinase 2 [Source:HGNC Symbol;Acc:HGNC:392] | 2866 | 0.140 | 0.1042 | No |
| 25 | DAXX | death domain associated protein [Source:HGNC Symbol;Acc:HGNC:2681] | 2928 | 0.138 | 0.1074 | No |
| 26 | DUSP4 | dual specificity phosphatase 4 [Source:HGNC Symbol;Acc:HGNC:3070] | 3200 | 0.132 | 0.1049 | No |
| 27 | RASGRF1 | Ras protein specific guanine nucleotide releasing factor 1 [Source:HGNC Symbol;Acc:HGNC:9875] | 3321 | 0.130 | 0.1062 | No |
| 28 | CACNA1D | calcium voltage-gated channel subunit alpha1 D [Source:HGNC Symbol;Acc:HGNC:1391] | 3402 | 0.128 | 0.1085 | No |
| 29 | SOS2 | SOS Ras/Rho guanine nucleotide exchange factor 2 [Source:HGNC Symbol;Acc:HGNC:11188] | 3713 | 0.123 | 0.1047 | No |
| 30 | AKT1 | AKT serine/threonine kinase 1 [Source:HGNC Symbol;Acc:HGNC:391] | 3889 | 0.120 | 0.1042 | No |
| 31 | ARRB1 | arrestin beta 1 [Source:HGNC Symbol;Acc:HGNC:711] | 3990 | 0.118 | 0.1057 | No |
| 32 | RAC1 | Rac family small GTPase 1 [Source:HGNC Symbol;Acc:HGNC:9801] | 4156 | 0.115 | 0.1053 | No |
| 33 | MAPKAPK3 | MAPK activated protein kinase 3 [Source:HGNC Symbol;Acc:HGNC:6888] | 4405 | 0.111 | 0.1027 | No |
| 34 | PLA2G2C | phospholipase A2 group IIC [Source:HGNC Symbol;Acc:HGNC:9032] | 4582 | 0.109 | 0.1019 | No |
| 35 | PLA2G1B | phospholipase A2 group IB [Source:HGNC Symbol;Acc:HGNC:9030] | 4747 | 0.106 | 0.1013 | No |
| 36 | MRAS | muscle RAS oncogene homolog [Source:HGNC Symbol;Acc:HGNC:7227] | 4760 | 0.106 | 0.1046 | No |
| 37 | MAP2K3 | mitogen-activated protein kinase kinase 3 [Source:HGNC Symbol;Acc:HGNC:6843] | 5059 | 0.102 | 0.1004 | No |
| 38 | MAP4K2 | mitogen-activated protein kinase kinase kinase kinase 2 [Source:HGNC Symbol;Acc:HGNC:6864] | 5359 | 0.098 | 0.0960 | No |
| 39 | CACNB3 | calcium voltage-gated channel auxiliary subunit beta 3 [Source:HGNC Symbol;Acc:HGNC:1403] | 5478 | 0.097 | 0.0963 | No |
| 40 | RELA | "RELA proto-oncogene, NF-kB subunit [Source:HGNC Symbol;Acc:HGNC:9955]" | 5531 | 0.096 | 0.0982 | No |
| 41 | CACNB2 | calcium voltage-gated channel auxiliary subunit beta 2 [Source:HGNC Symbol;Acc:HGNC:1402] | 6229 | 0.091 | 0.0835 | No |
| 42 | ARRB2 | arrestin beta 2 [Source:HGNC Symbol;Acc:HGNC:712] | 6252 | 0.091 | 0.0860 | No |
| 43 | FLNA | filamin A [Source:HGNC Symbol;Acc:HGNC:3754] | 6884 | 0.085 | 0.0727 | No |
| 44 | MAPKAPK5 | MAPK activated protein kinase 5 [Source:HGNC Symbol;Acc:HGNC:6889] | 7001 | 0.084 | 0.0725 | No |
| 45 | TNF | tumor necrosis factor [Source:HGNC Symbol;Acc:HGNC:11892] | 7309 | 0.081 | 0.0674 | No |
| 46 | TAOK2 | TAO kinase 2 [Source:HGNC Symbol;Acc:HGNC:16835] | 7670 | 0.078 | 0.0608 | No |
| 47 | NFKB2 | nuclear factor kappa B subunit 2 [Source:HGNC Symbol;Acc:HGNC:7795] | 7816 | 0.076 | 0.0597 | No |
| 48 | CACNG1 | calcium voltage-gated channel auxiliary subunit gamma 1 [Source:HGNC Symbol;Acc:HGNC:1405] | 7895 | 0.076 | 0.0602 | No |
| 49 | DUSP5 | dual specificity phosphatase 5 [Source:HGNC Symbol;Acc:HGNC:3071] | 8016 | 0.075 | 0.0597 | No |
| 50 | PPP3CB | protein phosphatase 3 catalytic subunit beta [Source:HGNC Symbol;Acc:HGNC:9315] | 8034 | 0.075 | 0.0618 | No |
| 51 | FGF20 | fibroblast growth factor 20 [Source:HGNC Symbol;Acc:HGNC:3677] | 8718 | 0.069 | 0.0466 | No |
| 52 | CACNA1A | calcium voltage-gated channel subunit alpha1 A [Source:HGNC Symbol;Acc:HGNC:1388] | 8843 | 0.068 | 0.0458 | No |
| 53 | TGFB2 | transforming growth factor beta 2 [Source:HGNC Symbol;Acc:HGNC:11768] | 8941 | 0.068 | 0.0456 | No |
| 54 | FLNB | filamin B [Source:HGNC Symbol;Acc:HGNC:3755] | 9034 | 0.067 | 0.0455 | No |
| 55 | PLA2G12B | phospholipase A2 group XIIB [Source:HGNC Symbol;Acc:HGNC:18555] | 9402 | 0.065 | 0.0383 | No |
| 56 | MAP3K4 | mitogen-activated protein kinase kinase kinase 4 [Source:HGNC Symbol;Acc:HGNC:6856] | 9638 | 0.063 | 0.0344 | No |
| 57 | PLA2G2E | phospholipase A2 group IIE [Source:HGNC Symbol;Acc:HGNC:13414] | 9748 | 0.062 | 0.0337 | No |
| 58 | PDGFA | platelet derived growth factor subunit A [Source:HGNC Symbol;Acc:HGNC:8799] | 10013 | 0.060 | 0.0289 | No |
| 59 | TAB2 | TGF-beta activated kinase 1 (MAP3K7) binding protein 2 [Source:HGNC Symbol;Acc:HGNC:17075] | 10465 | 0.057 | 0.0193 | No |
| 60 | PLA2G2F | phospholipase A2 group IIF [Source:HGNC Symbol;Acc:HGNC:30040] | 10874 | 0.054 | 0.0107 | No |
| 61 | STK3 | serine/threonine kinase 3 [Source:HGNC Symbol;Acc:HGNC:11406] | 10900 | 0.054 | 0.0119 | No |
| 62 | TNFRSF1A | TNF receptor superfamily member 1A [Source:HGNC Symbol;Acc:HGNC:11916] | 10931 | 0.054 | 0.0130 | No |
| 63 | MAPK14 | mitogen-activated protein kinase 14 [Source:HGNC Symbol;Acc:HGNC:6876] | 11437 | 0.051 | 0.0017 | No |
| 64 | MECOM | MDS1 and EVI1 complex locus [Source:HGNC Symbol;Acc:HGNC:3498] | 11729 | 0.049 | -0.0041 | No |
| 65 | MAPK8IP3 | mitogen-activated protein kinase 8 interacting protein 3 [Source:HGNC Symbol;Acc:HGNC:6884] | 11806 | 0.048 | -0.0044 | No |
| 66 | FGFR3 | fibroblast growth factor receptor 3 [Source:HGNC Symbol;Acc:HGNC:3690] | 12058 | 0.047 | -0.0092 | No |
| 67 | CHP1 | calcineurin like EF-hand protein 1 [Source:HGNC Symbol;Acc:HGNC:17433] | 12465 | 0.044 | -0.0181 | No |
| 68 | CDC25B | cell division cycle 25B [Source:HGNC Symbol;Acc:HGNC:1726] | 12580 | 0.044 | -0.0196 | No |
| 69 | CACNG6 | calcium voltage-gated channel auxiliary subunit gamma 6 [Source:HGNC Symbol;Acc:HGNC:13625] | 13079 | 0.040 | -0.0309 | No |
| 70 | FGF5 | fibroblast growth factor 5 [Source:HGNC Symbol;Acc:HGNC:3683] | 13398 | 0.039 | -0.0378 | No |
| 71 | STK4 | serine/threonine kinase 4 [Source:HGNC Symbol;Acc:HGNC:11408] | 13459 | 0.038 | -0.0380 | No |
| 72 | TAOK3 | TAO kinase 3 [Source:HGNC Symbol;Acc:HGNC:18133] | 13491 | 0.038 | -0.0375 | No |
| 73 | MKNK2 | MAPK interacting serine/threonine kinase 2 [Source:HGNC Symbol;Acc:HGNC:7111] | 13666 | 0.037 | -0.0407 | No |
| 74 | ATF4 | activating transcription factor 4 [Source:HGNC Symbol;Acc:HGNC:786] | 13757 | 0.036 | -0.0418 | No |
| 75 | HSPB1 | heat shock protein family B (small) member 1 [Source:HGNC Symbol;Acc:HGNC:5246] | 14517 | 0.032 | -0.0602 | No |
| 76 | MAP3K5 | mitogen-activated protein kinase kinase kinase 5 [Source:HGNC Symbol;Acc:HGNC:6857] | 14862 | 0.030 | -0.0680 | No |
| 77 | NFATC4 | nuclear factor of activated T cells 4 [Source:HGNC Symbol;Acc:HGNC:7778] | 14991 | 0.029 | -0.0703 | No |
| 78 | CHUK | component of inhibitor of nuclear factor kappa B kinase complex [Source:HGNC Symbol;Acc:HGNC:1974] | 15052 | 0.029 | -0.0708 | No |
| 79 | GADD45B | growth arrest and DNA damage inducible beta [Source:HGNC Symbol;Acc:HGNC:4096] | 15300 | 0.028 | -0.0762 | No |
| 80 | PRKCG | protein kinase C gamma [Source:HGNC Symbol;Acc:HGNC:9402] | 15351 | 0.027 | -0.0766 | No |
| 81 | MAP2K2 | mitogen-activated protein kinase kinase 2 [Source:HGNC Symbol;Acc:HGNC:6842] | 15510 | 0.026 | -0.0797 | No |
| 82 | PLA2G5 | phospholipase A2 group V [Source:HGNC Symbol;Acc:HGNC:9038] | 15541 | 0.026 | -0.0796 | No |
| 83 | NTF4 | neurotrophin 4 [Source:HGNC Symbol;Acc:HGNC:8024] | 15760 | 0.025 | -0.0844 | No |
| 84 | PPP3R2 | "protein phosphatase 3 regulatory subunit B, beta [Source:HGNC Symbol;Acc:HGNC:9318]" | 15798 | 0.024 | -0.0845 | No |
| 85 | IL1A | interleukin 1 alpha [Source:HGNC Symbol;Acc:HGNC:5991] | 15828 | 0.024 | -0.0844 | No |
| 86 | TGFBR2 | transforming growth factor beta receptor 2 [Source:HGNC Symbol;Acc:HGNC:11773] | 15982 | 0.023 | -0.0875 | No |
| 87 | RAF1 | "Raf-1 proto-oncogene, serine/threonine kinase [Source:HGNC Symbol;Acc:HGNC:9829]" | 16140 | 0.022 | -0.0908 | No |
| 88 | MOS | "MOS proto-oncogene, serine/threonine kinase [Source:HGNC Symbol;Acc:HGNC:7199]" | 16328 | 0.021 | -0.0949 | No |
| 89 | FGF4 | fibroblast growth factor 4 [Source:HGNC Symbol;Acc:HGNC:3682] | 16737 | 0.019 | -0.1047 | No |
| 90 | MAPK12 | mitogen-activated protein kinase 12 [Source:HGNC Symbol;Acc:HGNC:6874] | 17175 | 0.017 | -0.1153 | No |
| 91 | TGFB1 | transforming growth factor beta 1 [Source:HGNC Symbol;Acc:HGNC:11766] | 17301 | 0.016 | -0.1179 | No |
| 92 | ELK1 | ETS transcription factor ELK1 [Source:HGNC Symbol;Acc:HGNC:3321] | 18252 | 0.011 | -0.1419 | No |
| 93 | HSPA8 | heat shock protein family A (Hsp70) member 8 [Source:HGNC Symbol;Acc:HGNC:5241] | 18296 | 0.011 | -0.1426 | No |
| 94 | FGF6 | fibroblast growth factor 6 [Source:HGNC Symbol;Acc:HGNC:3684] | 18414 | 0.010 | -0.1453 | No |
| 95 | MAPK13 | mitogen-activated protein kinase 13 [Source:HGNC Symbol;Acc:HGNC:6875] | 18818 | 0.008 | -0.1554 | No |
| 96 | DUSP9 | dual specificity phosphatase 9 [Source:HGNC Symbol;Acc:HGNC:3076] | 18882 | 0.007 | -0.1567 | No |
| 97 | JUN | "Jun proto-oncogene, AP-1 transcription factor subunit [Source:HGNC Symbol;Acc:HGNC:6204]" | 18921 | 0.007 | -0.1575 | No |
| 98 | MAPKAPK2 | MAPK activated protein kinase 2 [Source:HGNC Symbol;Acc:HGNC:6887] | 19019 | 0.007 | -0.1597 | No |
| 99 | MAPK8IP1 | mitogen-activated protein kinase 8 interacting protein 1 [Source:HGNC Symbol;Acc:HGNC:6882] | 19293 | 0.005 | -0.1666 | No |
| 100 | MYC | "MYC proto-oncogene, bHLH transcription factor [Source:HGNC Symbol;Acc:HGNC:7553]" | 19309 | 0.005 | -0.1668 | No |
| 101 | MAPK11 | mitogen-activated protein kinase 11 [Source:HGNC Symbol;Acc:HGNC:6873] | 19398 | 0.004 | -0.1689 | No |
| 102 | PPM1A | "protein phosphatase, Mg2+/Mn2+ dependent 1A [Source:HGNC Symbol;Acc:HGNC:9275]" | 19642 | 0.003 | -0.1750 | No |
| 103 | ECSIT | ECSIT signaling integrator [Source:HGNC Symbol;Acc:HGNC:29548] | 19998 | 0.001 | -0.1841 | No |
| 104 | NTRK1 | neurotrophic receptor tyrosine kinase 1 [Source:HGNC Symbol;Acc:HGNC:8031] | 20383 | -0.002 | -0.1939 | No |
| 105 | NFKB1 | nuclear factor kappa B subunit 1 [Source:HGNC Symbol;Acc:HGNC:7794] | 20504 | -0.002 | -0.1969 | No |
| 106 | HRAS | "HRas proto-oncogene, GTPase [Source:HGNC Symbol;Acc:HGNC:5173]" | 20556 | -0.003 | -0.1981 | No |
| 107 | PRKACA | protein kinase cAMP-activated catalytic subunit alpha [Source:HGNC Symbol;Acc:HGNC:9380] | 21332 | -0.007 | -0.2177 | No |
| 108 | MAPK7 | mitogen-activated protein kinase 7 [Source:HGNC Symbol;Acc:HGNC:6880] | 21460 | -0.008 | -0.2207 | No |
| 109 | DUSP2 | dual specificity phosphatase 2 [Source:HGNC Symbol;Acc:HGNC:3068] | 21664 | -0.009 | -0.2255 | No |
| 110 | CACNA1C | calcium voltage-gated channel subunit alpha1 C [Source:HGNC Symbol;Acc:HGNC:1390] | 21681 | -0.009 | -0.2256 | No |
| 111 | PPP3R1 | "protein phosphatase 3 regulatory subunit B, alpha [Source:HGNC Symbol;Acc:HGNC:9317]" | 22525 | -0.015 | -0.2467 | No |
| 112 | RASGRF2 | Ras protein specific guanine nucleotide releasing factor 2 [Source:HGNC Symbol;Acc:HGNC:9876] | 22695 | -0.015 | -0.2505 | No |
| 113 | RRAS2 | RAS related 2 [Source:HGNC Symbol;Acc:HGNC:17271] | 22773 | -0.016 | -0.2520 | No |
| 114 | CACNG4 | calcium voltage-gated channel auxiliary subunit gamma 4 [Source:HGNC Symbol;Acc:HGNC:1408] | 22838 | -0.016 | -0.2531 | No |
| 115 | IKBKB | inhibitor of nuclear factor kappa B kinase subunit beta [Source:HGNC Symbol;Acc:HGNC:5960] | 22918 | -0.017 | -0.2545 | No |
| 116 | MAP2K7 | mitogen-activated protein kinase kinase 7 [Source:HGNC Symbol;Acc:HGNC:6847] | 22959 | -0.017 | -0.2550 | No |
| 117 | FGF7 | fibroblast growth factor 7 [Source:HGNC Symbol;Acc:HGNC:3685] | 23088 | -0.018 | -0.2576 | No |
| 118 | PDGFB | platelet derived growth factor subunit B [Source:HGNC Symbol;Acc:HGNC:8800] | 23487 | -0.021 | -0.2671 | No |
| 119 | TGFB3 | transforming growth factor beta 3 [Source:HGNC Symbol;Acc:HGNC:11769] | 23618 | -0.021 | -0.2697 | No |
| 120 | FAS | Fas cell surface death receptor [Source:HGNC Symbol;Acc:HGNC:11920] | 23910 | -0.023 | -0.2764 | No |
| 121 | DUSP6 | dual specificity phosphatase 6 [Source:HGNC Symbol;Acc:HGNC:3072] | 24279 | -0.026 | -0.2850 | No |
| 122 | DUSP10 | dual specificity phosphatase 10 [Source:HGNC Symbol;Acc:HGNC:3065] | 24388 | -0.026 | -0.2868 | No |
| 123 | GNA12 | G protein subunit alpha 12 [Source:HGNC Symbol;Acc:HGNC:4380] | 24506 | -0.027 | -0.2889 | No |
| 124 | FGF9 | fibroblast growth factor 9 [Source:HGNC Symbol;Acc:HGNC:3687] | 25006 | -0.031 | -0.3006 | No |
| 125 | PLA2G6 | phospholipase A2 group VI [Source:HGNC Symbol;Acc:HGNC:9039] | 25215 | -0.033 | -0.3048 | No |
| 126 | PLA2G4A | phospholipase A2 group IVA [Source:HGNC Symbol;Acc:HGNC:9035] | 25224 | -0.033 | -0.3039 | No |
| 127 | FGF10 | fibroblast growth factor 10 [Source:HGNC Symbol;Acc:HGNC:3666] | 25431 | -0.034 | -0.3080 | No |
| 128 | CACNG8 | calcium voltage-gated channel auxiliary subunit gamma 8 [Source:HGNC Symbol;Acc:HGNC:13628] | 25438 | -0.034 | -0.3070 | No |
| 129 | FGFR4 | fibroblast growth factor receptor 4 [Source:HGNC Symbol;Acc:HGNC:3691] | 25446 | -0.034 | -0.3061 | No |
| 130 | RASGRP4 | RAS guanyl releasing protein 4 [Source:HGNC Symbol;Acc:HGNC:18958] | 25482 | -0.034 | -0.3058 | No |
| 131 | CACNA1S | calcium voltage-gated channel subunit alpha1 S [Source:HGNC Symbol;Acc:HGNC:1397] | 25589 | -0.035 | -0.3073 | No |
| 132 | RELB | "RELB proto-oncogene, NF-kB subunit [Source:HGNC Symbol;Acc:HGNC:9956]" | 25674 | -0.036 | -0.3083 | No |
| 133 | RPS6KA5 | ribosomal protein S6 kinase A5 [Source:HGNC Symbol;Acc:HGNC:10434] | 25924 | -0.038 | -0.3134 | No |
| 134 | MKNK1 | MAPK interacting serine/threonine kinase 1 [Source:HGNC Symbol;Acc:HGNC:7110] | 25944 | -0.038 | -0.3126 | No |
| 135 | PRKACB | protein kinase cAMP-activated catalytic subunit beta [Source:HGNC Symbol;Acc:HGNC:9381] | 26088 | -0.039 | -0.3149 | No |
| 136 | CACNA1H | calcium voltage-gated channel subunit alpha1 H [Source:HGNC Symbol;Acc:HGNC:1395] | 26141 | -0.039 | -0.3149 | No |
| 137 | MAP3K8 | mitogen-activated protein kinase kinase kinase 8 [Source:HGNC Symbol;Acc:HGNC:6860] | 26194 | -0.040 | -0.3149 | No |
| 138 | MAP3K14 | mitogen-activated protein kinase kinase kinase 14 [Source:HGNC Symbol;Acc:HGNC:6853] | 26525 | -0.042 | -0.3220 | No |
| 139 | FGF1 | fibroblast growth factor 1 [Source:HGNC Symbol;Acc:HGNC:3665] | 26735 | -0.044 | -0.3259 | No |
| 140 | CD14 | CD14 molecule [Source:HGNC Symbol;Acc:HGNC:1628] | 27300 | -0.048 | -0.3387 | No |
| 141 | HSPA6 | heat shock protein family A (Hsp70) member 6 [Source:HGNC Symbol;Acc:HGNC:5239] | 27959 | -0.053 | -0.3537 | No |
| 142 | CACNG3 | calcium voltage-gated channel auxiliary subunit gamma 3 [Source:HGNC Symbol;Acc:HGNC:1407] | 28055 | -0.054 | -0.3543 | No |
| 143 | TRAF2 | TNF receptor associated factor 2 [Source:HGNC Symbol;Acc:HGNC:12032] | 28161 | -0.055 | -0.3552 | No |
| 144 | MAPT | microtubule associated protein tau [Source:HGNC Symbol;Acc:HGNC:6893] | 28320 | -0.056 | -0.3573 | No |
| 145 | MAPK9 | mitogen-activated protein kinase 9 [Source:HGNC Symbol;Acc:HGNC:6886] | 28470 | -0.057 | -0.3592 | No |
| 146 | CACNG7 | calcium voltage-gated channel auxiliary subunit gamma 7 [Source:HGNC Symbol;Acc:HGNC:13626] | 28606 | -0.058 | -0.3607 | No |
| 147 | SRF | serum response factor [Source:HGNC Symbol;Acc:HGNC:11291] | 28655 | -0.059 | -0.3600 | No |
| 148 | FGF18 | fibroblast growth factor 18 [Source:HGNC Symbol;Acc:HGNC:3674] | 28718 | -0.059 | -0.3596 | No |
| 149 | MAP3K7 | mitogen-activated protein kinase kinase kinase 7 [Source:HGNC Symbol;Acc:HGNC:6859] | 28753 | -0.059 | -0.3584 | No |
| 150 | PRKACG | protein kinase cAMP-activated catalytic subunit gamma [Source:HGNC Symbol;Acc:HGNC:9382] | 28777 | -0.059 | -0.3570 | No |
| 151 | DDIT3 | DNA damage inducible transcript 3 [Source:HGNC Symbol;Acc:HGNC:2726] | 28838 | -0.060 | -0.3565 | No |
| 152 | FGFR1 | fibroblast growth factor receptor 1 [Source:HGNC Symbol;Acc:HGNC:3688] | 28851 | -0.060 | -0.3548 | No |
| 153 | FGF21 | fibroblast growth factor 21 [Source:HGNC Symbol;Acc:HGNC:3678] | 28858 | -0.060 | -0.3529 | No |
| 154 | ATF2 | activating transcription factor 2 [Source:HGNC Symbol;Acc:HGNC:784] | 29013 | -0.062 | -0.3548 | No |
| 155 | HSPA1L | heat shock protein family A (Hsp70) member 1 like [Source:HGNC Symbol;Acc:HGNC:5234] | 29116 | -0.063 | -0.3553 | No |
| 156 | PLA2G2A | phospholipase A2 group IIA [Source:HGNC Symbol;Acc:HGNC:9031] | 29183 | -0.063 | -0.3548 | No |
| 157 | FGF23 | fibroblast growth factor 23 [Source:HGNC Symbol;Acc:HGNC:3680] | 29547 | -0.066 | -0.3619 | No |
| 158 | IL1B | interleukin 1 beta [Source:HGNC Symbol;Acc:HGNC:5992] | 29697 | -0.068 | -0.3634 | No |
| 159 | RAC2 | Rac family small GTPase 2 [Source:HGNC Symbol;Acc:HGNC:9802] | 29782 | -0.069 | -0.3632 | No |
| 160 | FASLG | Fas ligand [Source:HGNC Symbol;Acc:HGNC:11936] | 29910 | -0.070 | -0.3641 | No |
| 161 | CACNA2D2 | calcium voltage-gated channel auxiliary subunit alpha2delta 2 [Source:HGNC Symbol;Acc:HGNC:1400] | 29928 | -0.070 | -0.3622 | No |
| 162 | PTPN7 | protein tyrosine phosphatase non-receptor type 7 [Source:HGNC Symbol;Acc:HGNC:9659] | 29965 | -0.070 | -0.3607 | No |
| 163 | MAP2K6 | mitogen-activated protein kinase kinase 6 [Source:HGNC Symbol;Acc:HGNC:6846] | 30038 | -0.071 | -0.3602 | No |
| 164 | CACNA1G | calcium voltage-gated channel subunit alpha1 G [Source:HGNC Symbol;Acc:HGNC:1394] | 30317 | -0.074 | -0.3648 | No |
| 165 | PDGFRA | platelet derived growth factor receptor alpha [Source:HGNC Symbol;Acc:HGNC:8803] | 30377 | -0.074 | -0.3638 | No |
| 166 | FLNC | filamin C [Source:HGNC Symbol;Acc:HGNC:3756] | 30439 | -0.075 | -0.3628 | No |
| 167 | RPS6KA2 | ribosomal protein S6 kinase A2 [Source:HGNC Symbol;Acc:HGNC:10431] | 30862 | -0.079 | -0.3710 | No |
| 168 | TP53 | tumor protein p53 [Source:HGNC Symbol;Acc:HGNC:11998] | 30908 | -0.079 | -0.3695 | No |
| 169 | PTPN5 | protein tyrosine phosphatase non-receptor type 5 [Source:HGNC Symbol;Acc:HGNC:9657] | 30962 | -0.080 | -0.3681 | No |
| 170 | PPP3CC | protein phosphatase 3 catalytic subunit gamma [Source:HGNC Symbol;Acc:HGNC:9316] | 30963 | -0.080 | -0.3654 | No |
| 171 | FGF22 | fibroblast growth factor 22 [Source:HGNC Symbol;Acc:HGNC:3679] | 31031 | -0.081 | -0.3644 | No |
| 172 | MAP2K5 | mitogen-activated protein kinase kinase 5 [Source:HGNC Symbol;Acc:HGNC:6845] | 31433 | -0.085 | -0.3718 | No |
| 173 | RASGRP3 | RAS guanyl releasing protein 3 [Source:HGNC Symbol;Acc:HGNC:14545] | 31447 | -0.085 | -0.3693 | No |
| 174 | JUND | "JunD proto-oncogene, AP-1 transcription factor subunit [Source:HGNC Symbol;Acc:HGNC:6206]" | 31507 | -0.085 | -0.3679 | No |
| 175 | GADD45G | growth arrest and DNA damage inducible gamma [Source:HGNC Symbol;Acc:HGNC:4097] | 31691 | -0.087 | -0.3696 | No |
| 176 | DUSP7 | dual specificity phosphatase 7 [Source:HGNC Symbol;Acc:HGNC:3073] | 31944 | -0.090 | -0.3730 | Yes |
| 177 | MAP2K1 | mitogen-activated protein kinase kinase 1 [Source:HGNC Symbol;Acc:HGNC:6840] | 32048 | -0.091 | -0.3726 | Yes |
| 178 | EGFR | epidermal growth factor receptor [Source:HGNC Symbol;Acc:HGNC:3236] | 32133 | -0.092 | -0.3716 | Yes |
| 179 | PDGFRB | platelet derived growth factor receptor beta [Source:HGNC Symbol;Acc:HGNC:8804] | 32165 | -0.093 | -0.3693 | Yes |
| 180 | FGF3 | fibroblast growth factor 3 [Source:HGNC Symbol;Acc:HGNC:3681] | 32274 | -0.094 | -0.3689 | Yes |
| 181 | MEF2C | myocyte enhancer factor 2C [Source:HGNC Symbol;Acc:HGNC:6996] | 32279 | -0.094 | -0.3658 | Yes |
| 182 | GRB2 | growth factor receptor bound protein 2 [Source:HGNC Symbol;Acc:HGNC:4566] | 32433 | -0.096 | -0.3665 | Yes |
| 183 | IL1R1 | interleukin 1 receptor type 1 [Source:HGNC Symbol;Acc:HGNC:5993] | 32518 | -0.097 | -0.3654 | Yes |
| 184 | RASA1 | RAS p21 protein activator 1 [Source:HGNC Symbol;Acc:HGNC:9871] | 32558 | -0.097 | -0.3631 | Yes |
| 185 | NFATC2 | nuclear factor of activated T cells 2 [Source:HGNC Symbol;Acc:HGNC:7776] | 32713 | -0.100 | -0.3637 | Yes |
| 186 | MAP4K3 | mitogen-activated protein kinase kinase kinase kinase 3 [Source:HGNC Symbol;Acc:HGNC:6865] | 32740 | -0.100 | -0.3609 | Yes |
| 187 | MAP3K13 | mitogen-activated protein kinase kinase kinase 13 [Source:HGNC Symbol;Acc:HGNC:6852] | 32829 | -0.101 | -0.3598 | Yes |
| 188 | CASP3 | caspase 3 [Source:HGNC Symbol;Acc:HGNC:1504] | 32939 | -0.103 | -0.3591 | Yes |
| 189 | PLA2G10 | phospholipase A2 group X [Source:HGNC Symbol;Acc:HGNC:9029] | 32963 | -0.103 | -0.3562 | Yes |
| 190 | NTF3 | neurotrophin 3 [Source:HGNC Symbol;Acc:HGNC:8023] | 33265 | -0.107 | -0.3603 | Yes |
| 191 | MAPK8IP2 | mitogen-activated protein kinase 8 interacting protein 2 [Source:HGNC Symbol;Acc:HGNC:6883] | 33335 | -0.108 | -0.3584 | Yes |
| 192 | MAPK1 | mitogen-activated protein kinase 1 [Source:HGNC Symbol;Acc:HGNC:6871] | 33350 | -0.108 | -0.3551 | Yes |
| 193 | BRAF | "B-Raf proto-oncogene, serine/threonine kinase [Source:HGNC Symbol;Acc:HGNC:1097]" | 33362 | -0.108 | -0.3517 | Yes |
| 194 | MAX | MYC associated factor X [Source:HGNC Symbol;Acc:HGNC:6913] | 33370 | -0.109 | -0.3483 | Yes |
| 195 | CRK | "CRK proto-oncogene, adaptor protein [Source:HGNC Symbol;Acc:HGNC:2362]" | 33404 | -0.109 | -0.3454 | Yes |
| 196 | MAP3K1 | mitogen-activated protein kinase kinase kinase 1 [Source:HGNC Symbol;Acc:HGNC:6848] | 33451 | -0.110 | -0.3429 | Yes |
| 197 | RAPGEF2 | Rap guanine nucleotide exchange factor 2 [Source:HGNC Symbol;Acc:HGNC:16854] | 33668 | -0.113 | -0.3446 | Yes |
| 198 | MAP3K3 | mitogen-activated protein kinase kinase kinase 3 [Source:HGNC Symbol;Acc:HGNC:6855] | 33687 | -0.113 | -0.3412 | Yes |
| 199 | RASGRP2 | RAS guanyl releasing protein 2 [Source:HGNC Symbol;Acc:HGNC:9879] | 33892 | -0.116 | -0.3425 | Yes |
| 200 | RASA2 | RAS p21 protein activator 2 [Source:HGNC Symbol;Acc:HGNC:9872] | 34117 | -0.120 | -0.3442 | Yes |
| 201 | CACNB1 | calcium voltage-gated channel auxiliary subunit beta 1 [Source:HGNC Symbol;Acc:HGNC:1401] | 34159 | -0.120 | -0.3412 | Yes |
| 202 | CRKL | "CRK like proto-oncogene, adaptor protein [Source:HGNC Symbol;Acc:HGNC:2363]" | 34209 | -0.121 | -0.3384 | Yes |
| 203 | CACNA1F | calcium voltage-gated channel subunit alpha1 F [Source:HGNC Symbol;Acc:HGNC:1393] | 34492 | -0.126 | -0.3414 | Yes |
| 204 | CHP2 | calcineurin like EF-hand protein 2 [Source:HGNC Symbol;Acc:HGNC:24927] | 34531 | -0.126 | -0.3381 | Yes |
| 205 | IL1R2 | interleukin 1 receptor type 2 [Source:HGNC Symbol;Acc:HGNC:5994] | 34576 | -0.127 | -0.3349 | Yes |
| 206 | NGF | nerve growth factor [Source:HGNC Symbol;Acc:HGNC:7808] | 34581 | -0.127 | -0.3307 | Yes |
| 207 | PRKCB | protein kinase C beta [Source:HGNC Symbol;Acc:HGNC:9395] | 34603 | -0.128 | -0.3270 | Yes |
| 208 | RAP1B | "RAP1B, member of RAS oncogene family [Source:HGNC Symbol;Acc:HGNC:9857]" | 34656 | -0.129 | -0.3239 | Yes |
| 209 | DUSP1 | dual specificity phosphatase 1 [Source:HGNC Symbol;Acc:HGNC:3064] | 34684 | -0.129 | -0.3203 | Yes |
| 210 | MAP3K20 | mitogen-activated protein kinase kinase kinase 20 [Source:HGNC Symbol;Acc:HGNC:17797] | 34938 | -0.134 | -0.3222 | Yes |
| 211 | PLA2G4B | phospholipase A2 group IVB [Source:HGNC Symbol;Acc:HGNC:9036] | 34964 | -0.135 | -0.3183 | Yes |
| 212 | MAP3K12 | mitogen-activated protein kinase kinase kinase 12 [Source:HGNC Symbol;Acc:HGNC:6851] | 35096 | -0.138 | -0.3170 | Yes |
| 213 | PRKCA | protein kinase C alpha [Source:HGNC Symbol;Acc:HGNC:9393] | 35223 | -0.140 | -0.3155 | Yes |
| 214 | FGF16 | fibroblast growth factor 16 [Source:HGNC Symbol;Acc:HGNC:3672] | 35237 | -0.140 | -0.3111 | Yes |
| 215 | MAP4K4 | mitogen-activated protein kinase kinase kinase kinase 4 [Source:HGNC Symbol;Acc:HGNC:6866] | 35278 | -0.141 | -0.3073 | Yes |
| 216 | FGF17 | fibroblast growth factor 17 [Source:HGNC Symbol;Acc:HGNC:3673] | 35557 | -0.148 | -0.3095 | Yes |
| 217 | NR4A1 | nuclear receptor subfamily 4 group A member 1 [Source:HGNC Symbol;Acc:HGNC:7980] | 35649 | -0.151 | -0.3067 | Yes |
| 218 | DUSP16 | dual specificity phosphatase 16 [Source:HGNC Symbol;Acc:HGNC:17909] | 35741 | -0.153 | -0.3038 | Yes |
| 219 | CACNG5 | calcium voltage-gated channel auxiliary subunit gamma 5 [Source:HGNC Symbol;Acc:HGNC:1409] | 35813 | -0.155 | -0.3004 | Yes |
| 220 | CACNA2D4 | calcium voltage-gated channel auxiliary subunit alpha2delta 4 [Source:HGNC Symbol;Acc:HGNC:20202] | 35958 | -0.159 | -0.2987 | Yes |
| 221 | JMJD7-PLA2G4B | JMJD7-PLA2G4B readthrough [Source:HGNC Symbol;Acc:HGNC:34449] | 36012 | -0.161 | -0.2947 | Yes |
| 222 | FGF12 | fibroblast growth factor 12 [Source:HGNC Symbol;Acc:HGNC:3668] | 36028 | -0.161 | -0.2896 | Yes |
| 223 | PAK1 | p21 (RAC1) activated kinase 1 [Source:HGNC Symbol;Acc:HGNC:8590] | 36099 | -0.163 | -0.2859 | Yes |
| 224 | CACNG2 | calcium voltage-gated channel auxiliary subunit gamma 2 [Source:HGNC Symbol;Acc:HGNC:1406] | 36107 | -0.163 | -0.2806 | Yes |
| 225 | FOS | "Fos proto-oncogene, AP-1 transcription factor subunit [Source:HGNC Symbol;Acc:HGNC:3796]" | 36141 | -0.164 | -0.2758 | Yes |
| 226 | MAPK10 | mitogen-activated protein kinase 10 [Source:HGNC Symbol;Acc:HGNC:6872] | 36201 | -0.166 | -0.2717 | Yes |
| 227 | PLA2G3 | phospholipase A2 group III [Source:HGNC Symbol;Acc:HGNC:17934] | 36216 | -0.167 | -0.2664 | Yes |
| 228 | PLA2G4E | phospholipase A2 group IVE [Source:HGNC Symbol;Acc:HGNC:24791] | 36372 | -0.172 | -0.2646 | Yes |
| 229 | MAP4K1 | mitogen-activated protein kinase kinase kinase kinase 1 [Source:HGNC Symbol;Acc:HGNC:6863] | 36401 | -0.173 | -0.2595 | Yes |
| 230 | MAP3K2 | mitogen-activated protein kinase kinase kinase 2 [Source:HGNC Symbol;Acc:HGNC:6854] | 36502 | -0.177 | -0.2561 | Yes |
| 231 | SOS1 | SOS Ras/Rac guanine nucleotide exchange factor 1 [Source:HGNC Symbol;Acc:HGNC:11187] | 36506 | -0.177 | -0.2502 | Yes |
| 232 | FGF14 | fibroblast growth factor 14 [Source:HGNC Symbol;Acc:HGNC:3671] | 36577 | -0.179 | -0.2459 | Yes |
| 233 | HSPA1B | heat shock protein family A (Hsp70) member 1B [Source:HGNC Symbol;Acc:HGNC:5233] | 36628 | -0.181 | -0.2411 | Yes |
| 234 | MAP2K4 | mitogen-activated protein kinase kinase 4 [Source:HGNC Symbol;Acc:HGNC:6844] | 36685 | -0.184 | -0.2363 | Yes |
| 235 | PAK2 | p21 (RAC1) activated kinase 2 [Source:HGNC Symbol;Acc:HGNC:8591] | 36837 | -0.190 | -0.2338 | Yes |
| 236 | DUSP3 | dual specificity phosphatase 3 [Source:HGNC Symbol;Acc:HGNC:3069] | 36881 | -0.192 | -0.2284 | Yes |
| 237 | KRAS | "KRAS proto-oncogene, GTPase [Source:HGNC Symbol;Acc:HGNC:6407]" | 36928 | -0.194 | -0.2230 | Yes |
| 238 | PLA2G2D | phospholipase A2 group IID [Source:HGNC Symbol;Acc:HGNC:9033] | 36979 | -0.196 | -0.2176 | Yes |
| 239 | FGF8 | fibroblast growth factor 8 [Source:HGNC Symbol;Acc:HGNC:3686] | 37023 | -0.198 | -0.2120 | Yes |
| 240 | CACNA1E | calcium voltage-gated channel subunit alpha1 E [Source:HGNC Symbol;Acc:HGNC:1392] | 37145 | -0.205 | -0.2082 | Yes |
| 241 | DUSP8 | dual specificity phosphatase 8 [Source:HGNC Symbol;Acc:HGNC:3074] | 37157 | -0.205 | -0.2016 | Yes |
| 242 | CACNB4 | calcium voltage-gated channel auxiliary subunit beta 4 [Source:HGNC Symbol;Acc:HGNC:1404] | 37195 | -0.207 | -0.1955 | Yes |
| 243 | CACNA1I | calcium voltage-gated channel subunit alpha1 I [Source:HGNC Symbol;Acc:HGNC:1396] | 37273 | -0.211 | -0.1904 | Yes |
| 244 | IKBKG | inhibitor of nuclear factor kappa B kinase regulatory subunit gamma [Source:HGNC Symbol;Acc:HGNC:5961] | 37365 | -0.216 | -0.1854 | Yes |
| 245 | FGFR2 | fibroblast growth factor receptor 2 [Source:HGNC Symbol;Acc:HGNC:3689] | 37585 | -0.230 | -0.1832 | Yes |
| 246 | EGF | epidermal growth factor [Source:HGNC Symbol;Acc:HGNC:3229] | 37597 | -0.231 | -0.1757 | Yes |
| 247 | FGF13 | fibroblast growth factor 13 [Source:HGNC Symbol;Acc:HGNC:3670] | 37611 | -0.232 | -0.1682 | Yes |
| 248 | FGF19 | fibroblast growth factor 19 [Source:HGNC Symbol;Acc:HGNC:3675] | 37781 | -0.243 | -0.1643 | Yes |
| 249 | DUSP14 | dual specificity phosphatase 14 [Source:HGNC Symbol;Acc:HGNC:17007] | 37798 | -0.245 | -0.1565 | Yes |
| 250 | TGFBR1 | transforming growth factor beta receptor 1 [Source:HGNC Symbol;Acc:HGNC:11772] | 37905 | -0.255 | -0.1506 | Yes |
| 251 | ELK4 | ETS transcription factor ELK4 [Source:HGNC Symbol;Acc:HGNC:3326] | 37925 | -0.257 | -0.1424 | Yes |
| 252 | CACNA2D3 | calcium voltage-gated channel auxiliary subunit alpha2delta 3 [Source:HGNC Symbol;Acc:HGNC:15460] | 37984 | -0.264 | -0.1349 | Yes |
| 253 | PPM1B | "protein phosphatase, Mg2+/Mn2+ dependent 1B [Source:HGNC Symbol;Acc:HGNC:9276]" | 38066 | -0.272 | -0.1278 | Yes |
| 254 | TRAF6 | TNF receptor associated factor 6 [Source:HGNC Symbol;Acc:HGNC:12036] | 38104 | -0.278 | -0.1194 | Yes |
| 255 | CACNA1B | calcium voltage-gated channel subunit alpha1 B [Source:HGNC Symbol;Acc:HGNC:1389] | 38121 | -0.281 | -0.1103 | Yes |
| 256 | RPS6KA6 | ribosomal protein S6 kinase A6 [Source:HGNC Symbol;Acc:HGNC:10435] | 38151 | -0.285 | -0.1014 | Yes |
| 257 | FGF2 | fibroblast growth factor 2 [Source:HGNC Symbol;Acc:HGNC:3676] | 38191 | -0.292 | -0.0925 | Yes |
| 258 | NLK | nemo like kinase [Source:HGNC Symbol;Acc:HGNC:29858] | 38317 | -0.310 | -0.0852 | Yes |
| 259 | AKT3 | AKT serine/threonine kinase 3 [Source:HGNC Symbol;Acc:HGNC:393] | 38358 | -0.320 | -0.0755 | Yes |
| 260 | NF1 | neurofibromin 1 [Source:HGNC Symbol;Acc:HGNC:7765] | 38412 | -0.335 | -0.0655 | Yes |
| 261 | RASGRP1 | RAS guanyl releasing protein 1 [Source:HGNC Symbol;Acc:HGNC:9878] | 38416 | -0.336 | -0.0542 | Yes |
| 262 | BDNF | brain derived neurotrophic factor [Source:HGNC Symbol;Acc:HGNC:1033] | 38454 | -0.352 | -0.0433 | Yes |
| 263 | CACNA2D1 | calcium voltage-gated channel auxiliary subunit alpha2delta 1 [Source:HGNC Symbol;Acc:HGNC:1399] | 38467 | -0.356 | -0.0316 | Yes |
| 264 | RPS6KA3 | ribosomal protein S6 kinase A3 [Source:HGNC Symbol;Acc:HGNC:10432] | 38483 | -0.366 | -0.0196 | Yes |
| 265 | PRKX | protein kinase X-linked [Source:HGNC Symbol;Acc:HGNC:9441] | 38524 | -0.395 | -0.0073 | Yes |
| 266 | TAOK1 | TAO kinase 1 [Source:HGNC Symbol;Acc:HGNC:29259] | 38531 | -0.404 | 0.0062 | Yes |
| 267 | NTRK2 | neurotrophic receptor tyrosine kinase 2 [Source:HGNC Symbol;Acc:HGNC:8032] | 38537 | -0.416 | 0.0201 | Yes |
Table: GSEA details [plain text format]

  

Fig 2: KEGG\_MAPK\_SIGNALING\_PATHWAY      
 Blue-Pink O' Gram in the Space of the Analyzed GeneSet

  

Fig 3: KEGG\_MAPK\_SIGNALING\_PATHWAY: Random ES distribution      
 Gene set null distribution of ES for **KEGG\_MAPK\_SIGNALING\_PATHWAY**
