# Supplementary material for: Simultaneous Presentation of Multiple Myeloma and Lung Cancer: Case Report and Gene Bioinformatics Analysis
Source: Front Oncol. 2022 Jun 13;12:859735. doi: 10.3389/fonc.2022.859735 (PMC9235397; doi:10.3389/fonc.2022.859735)
Supplement: Supplementary file 1 [file DataSheet_1.zip › The bioinformatic analysis of MM and lung cancer supplementary materials/Enrichment analysis/MECR/GSEA_4.1.0/LUSC TCGA/KEGG.Gsea.1639042374131/KEGG_MELANOGENESIS.html]

Details for gene set KEGG\_MELANOGENESIS[GSEA]

|  || Dataset | ExpData\_collapsed\_to\_symbols.ENSG00000116353\_profile\_in\_ExpData.cls #ENSG00000116353 |
| Phenotype | ENSG00000116353\_profile\_in\_ExpData.cls#ENSG00000116353 |
| Upregulated in class | ENSG00000116353\_neg |
| GeneSet | KEGG\_MELANOGENESIS |
| Enrichment Score (ES) | -0.42658758 |
| Normalized Enrichment Score (NES) | -1.815271 |
| Nominal p-value | 0.0020242915 |
| FDR q-value | 0.008665631 |
| FWER p-Value | 0.1 |
Table: GSEA Results Summary

  

Fig 1: Enrichment plot: KEGG\_MELANOGENESIS      
 Profile of the Running ES Score & Positions of GeneSet Members on the Rank Ordered List

  

| SYMBOL | TITLE | RANK IN GENE LIST | RANK METRIC SCORE | RUNNING ES | CORE ENRICHMENT || 1 | DVL1 | dishevelled segment polarity protein 1 [Source:HGNC Symbol;Acc:HGNC:3084] | 49 | 0.451 | 0.0347 | No |
| 2 | MAPK3 | mitogen-activated protein kinase 3 [Source:HGNC Symbol;Acc:HGNC:6877] | 473 | 0.274 | 0.0457 | No |
| 3 | NRAS | "NRAS proto-oncogene, GTPase [Source:HGNC Symbol;Acc:HGNC:7989]" | 774 | 0.235 | 0.0568 | No |
| 4 | WNT10A | Wnt family member 10A [Source:HGNC Symbol;Acc:HGNC:13829] | 839 | 0.228 | 0.0733 | No |
| 5 | CALM3 | calmodulin 3 [Source:HGNC Symbol;Acc:HGNC:1449] | 850 | 0.227 | 0.0911 | No |
| 6 | GNAI3 | G protein subunit alpha i3 [Source:HGNC Symbol;Acc:HGNC:4387] | 869 | 0.226 | 0.1086 | No |
| 7 | WNT4 | Wnt family member 4 [Source:HGNC Symbol;Acc:HGNC:12783] | 1086 | 0.210 | 0.1198 | No |
| 8 | CAMK2G | calcium/calmodulin dependent protein kinase II gamma [Source:HGNC Symbol;Acc:HGNC:1463] | 1746 | 0.175 | 0.1169 | No |
| 9 | TCF7L2 | transcription factor 7 like 2 [Source:HGNC Symbol;Acc:HGNC:11641] | 1930 | 0.168 | 0.1256 | No |
| 10 | PLCB3 | phospholipase C beta 3 [Source:HGNC Symbol;Acc:HGNC:9056] | 1971 | 0.167 | 0.1379 | No |
| 11 | ASIP | agouti signaling protein [Source:HGNC Symbol;Acc:HGNC:745] | 3840 | 0.120 | 0.0998 | No |
| 12 | GNAS | GNAS complex locus [Source:HGNC Symbol;Acc:HGNC:4392] | 4003 | 0.118 | 0.1051 | No |
| 13 | KIT | "KIT proto-oncogene, receptor tyrosine kinase [Source:HGNC Symbol;Acc:HGNC:6342]" | 4449 | 0.110 | 0.1025 | No |
| 14 | WNT6 | Wnt family member 6 [Source:HGNC Symbol;Acc:HGNC:12785] | 4672 | 0.107 | 0.1054 | No |
| 15 | CREB3L4 | cAMP responsive element binding protein 3 like 4 [Source:HGNC Symbol;Acc:HGNC:18854] | 5984 | 0.094 | 0.0795 | No |
| 16 | WNT16 | Wnt family member 16 [Source:HGNC Symbol;Acc:HGNC:16267] | 6037 | 0.094 | 0.0856 | No |
| 17 | CALM2 | calmodulin 2 [Source:HGNC Symbol;Acc:HGNC:1445] | 6292 | 0.091 | 0.0863 | No |
| 18 | CALML5 | calmodulin like 5 [Source:HGNC Symbol;Acc:HGNC:18180] | 6619 | 0.087 | 0.0850 | No |
| 19 | CAMK2D | calcium/calmodulin dependent protein kinase II delta [Source:HGNC Symbol;Acc:HGNC:1462] | 8249 | 0.073 | 0.0492 | No |
| 20 | WNT8A | Wnt family member 8A [Source:HGNC Symbol;Acc:HGNC:12788] | 8416 | 0.071 | 0.0507 | No |
| 21 | CAMK2B | calcium/calmodulin dependent protein kinase II beta [Source:HGNC Symbol;Acc:HGNC:1461] | 10058 | 0.060 | 0.0136 | No |
| 22 | WNT7A | Wnt family member 7A [Source:HGNC Symbol;Acc:HGNC:12786] | 10774 | 0.055 | -0.0003 | No |
| 23 | FZD5 | frizzled class receptor 5 [Source:HGNC Symbol;Acc:HGNC:4043] | 11161 | 0.053 | -0.0059 | No |
| 24 | GNAI1 | G protein subunit alpha i1 [Source:HGNC Symbol;Acc:HGNC:4384] | 11240 | 0.052 | -0.0038 | No |
| 25 | TCF7 | transcription factor 7 [Source:HGNC Symbol;Acc:HGNC:11639] | 13002 | 0.041 | -0.0454 | No |
| 26 | CAMK2A | calcium/calmodulin dependent protein kinase II alpha [Source:HGNC Symbol;Acc:HGNC:1460] | 13120 | 0.040 | -0.0452 | No |
| 27 | GNAI2 | G protein subunit alpha i2 [Source:HGNC Symbol;Acc:HGNC:4385] | 15092 | 0.029 | -0.0932 | No |
| 28 | PRKCG | protein kinase C gamma [Source:HGNC Symbol;Acc:HGNC:9402] | 15351 | 0.027 | -0.0976 | No |
| 29 | MAP2K2 | mitogen-activated protein kinase kinase 2 [Source:HGNC Symbol;Acc:HGNC:6842] | 15510 | 0.026 | -0.0995 | No |
| 30 | GSK3B | glycogen synthase kinase 3 beta [Source:HGNC Symbol;Acc:HGNC:4617] | 15772 | 0.025 | -0.1042 | No |
| 31 | RAF1 | "Raf-1 proto-oncogene, serine/threonine kinase [Source:HGNC Symbol;Acc:HGNC:9829]" | 16140 | 0.022 | -0.1118 | No |
| 32 | CREB3L1 | cAMP responsive element binding protein 3 like 1 [Source:HGNC Symbol;Acc:HGNC:18856] | 16289 | 0.022 | -0.1138 | No |
| 33 | WNT1 | Wnt family member 1 [Source:HGNC Symbol;Acc:HGNC:12774] | 19377 | 0.004 | -0.1922 | No |
| 34 | ADCY6 | adenylate cyclase 6 [Source:HGNC Symbol;Acc:HGNC:237] | 20109 | 0.000 | -0.2108 | No |
| 35 | FZD8 | frizzled class receptor 8 [Source:HGNC Symbol;Acc:HGNC:4046] | 20256 | -0.001 | -0.2145 | No |
| 36 | CALML6 | calmodulin like 6 [Source:HGNC Symbol;Acc:HGNC:24193] | 20499 | -0.002 | -0.2205 | No |
| 37 | HRAS | "HRas proto-oncogene, GTPase [Source:HGNC Symbol;Acc:HGNC:5173]" | 20556 | -0.003 | -0.2217 | No |
| 38 | TCF7L1 | transcription factor 7 like 1 [Source:HGNC Symbol;Acc:HGNC:11640] | 20720 | -0.004 | -0.2256 | No |
| 39 | FZD4 | frizzled class receptor 4 [Source:HGNC Symbol;Acc:HGNC:4042] | 20770 | -0.004 | -0.2265 | No |
| 40 | PRKACA | protein kinase cAMP-activated catalytic subunit alpha [Source:HGNC Symbol;Acc:HGNC:9380] | 21332 | -0.007 | -0.2402 | No |
| 41 | CALM1 | calmodulin 1 [Source:HGNC Symbol;Acc:HGNC:1442] | 21884 | -0.010 | -0.2534 | No |
| 42 | WNT7B | Wnt family member 7B [Source:HGNC Symbol;Acc:HGNC:12787] | 22905 | -0.017 | -0.2781 | No |
| 43 | EDNRB | endothelin receptor type B [Source:HGNC Symbol;Acc:HGNC:3180] | 25161 | -0.032 | -0.3330 | No |
| 44 | WNT9A | Wnt family member 9A [Source:HGNC Symbol;Acc:HGNC:12778] | 25791 | -0.037 | -0.3462 | No |
| 45 | ADCY1 | adenylate cyclase 1 [Source:HGNC Symbol;Acc:HGNC:232] | 25830 | -0.037 | -0.3442 | No |
| 46 | PRKACB | protein kinase cAMP-activated catalytic subunit beta [Source:HGNC Symbol;Acc:HGNC:9381] | 26088 | -0.039 | -0.3477 | No |
| 47 | TYRP1 | tyrosinase related protein 1 [Source:HGNC Symbol;Acc:HGNC:12450] | 26457 | -0.041 | -0.3537 | No |
| 48 | FZD1 | frizzled class receptor 1 [Source:HGNC Symbol;Acc:HGNC:4038] | 26540 | -0.042 | -0.3525 | No |
| 49 | WNT10B | Wnt family member 10B [Source:HGNC Symbol;Acc:HGNC:12775] | 26603 | -0.042 | -0.3507 | No |
| 50 | PLCB1 | phospholipase C beta 1 [Source:HGNC Symbol;Acc:HGNC:15917] | 27781 | -0.052 | -0.3766 | No |
| 51 | ADCY2 | adenylate cyclase 2 [Source:HGNC Symbol;Acc:HGNC:233] | 27981 | -0.053 | -0.3774 | No |
| 52 | GNAO1 | G protein subunit alpha o1 [Source:HGNC Symbol;Acc:HGNC:4389] | 28043 | -0.054 | -0.3747 | No |
| 53 | FZD9 | frizzled class receptor 9 [Source:HGNC Symbol;Acc:HGNC:4047] | 28404 | -0.057 | -0.3794 | No |
| 54 | CREB3 | cAMP responsive element binding protein 3 [Source:HGNC Symbol;Acc:HGNC:2347] | 28414 | -0.057 | -0.3751 | No |
| 55 | MITF | melanocyte inducing transcription factor [Source:HGNC Symbol;Acc:HGNC:7105] | 28642 | -0.058 | -0.3762 | No |
| 56 | PRKACG | protein kinase cAMP-activated catalytic subunit gamma [Source:HGNC Symbol;Acc:HGNC:9382] | 28777 | -0.059 | -0.3749 | No |
| 57 | CREB1 | cAMP responsive element binding protein 1 [Source:HGNC Symbol;Acc:HGNC:2345] | 28807 | -0.060 | -0.3709 | No |
| 58 | TYR | tyrosinase [Source:HGNC Symbol;Acc:HGNC:12442] | 29017 | -0.062 | -0.3713 | No |
| 59 | WNT3A | Wnt family member 3A [Source:HGNC Symbol;Acc:HGNC:15983] | 29041 | -0.062 | -0.3670 | No |
| 60 | CREB3L3 | cAMP responsive element binding protein 3 like 3 [Source:HGNC Symbol;Acc:HGNC:18855] | 30002 | -0.071 | -0.3858 | No |
| 61 | FZD3 | frizzled class receptor 3 [Source:HGNC Symbol;Acc:HGNC:4041] | 31252 | -0.083 | -0.4111 | No |
| 62 | CREB3L2 | cAMP responsive element binding protein 3 like 2 [Source:HGNC Symbol;Acc:HGNC:23720] | 31647 | -0.087 | -0.4142 | No |
| 63 | DVL2 | dishevelled segment polarity protein 2 [Source:HGNC Symbol;Acc:HGNC:3086] | 31825 | -0.089 | -0.4116 | No |
| 64 | MAP2K1 | mitogen-activated protein kinase kinase 1 [Source:HGNC Symbol;Acc:HGNC:6840] | 32048 | -0.091 | -0.4100 | No |
| 65 | WNT5B | Wnt family member 5B [Source:HGNC Symbol;Acc:HGNC:16265] | 32250 | -0.094 | -0.4077 | No |
| 66 | PLCB2 | phospholipase C beta 2 [Source:HGNC Symbol;Acc:HGNC:9055] | 32369 | -0.095 | -0.4031 | No |
| 67 | WNT2 | Wnt family member 2 [Source:HGNC Symbol;Acc:HGNC:12780] | 32471 | -0.096 | -0.3980 | No |
| 68 | WNT8B | Wnt family member 8B [Source:HGNC Symbol;Acc:HGNC:12789] | 33128 | -0.105 | -0.4064 | No |
| 69 | MAPK1 | mitogen-activated protein kinase 1 [Source:HGNC Symbol;Acc:HGNC:6871] | 33350 | -0.108 | -0.4034 | No |
| 70 | CALML3 | calmodulin like 3 [Source:HGNC Symbol;Acc:HGNC:1452] | 33436 | -0.110 | -0.3969 | No |
| 71 | PRKCB | protein kinase C beta [Source:HGNC Symbol;Acc:HGNC:9395] | 34603 | -0.128 | -0.4164 | Yes |
| 72 | FZD2 | frizzled class receptor 2 [Source:HGNC Symbol;Acc:HGNC:4040] | 34628 | -0.128 | -0.4068 | Yes |
| 73 | ADCY7 | adenylate cyclase 7 [Source:HGNC Symbol;Acc:HGNC:238] | 34661 | -0.129 | -0.3974 | Yes |
| 74 | KITLG | KIT ligand [Source:HGNC Symbol;Acc:HGNC:6343] | 34776 | -0.131 | -0.3899 | Yes |
| 75 | ADCY4 | adenylate cyclase 4 [Source:HGNC Symbol;Acc:HGNC:235] | 34840 | -0.132 | -0.3810 | Yes |
| 76 | FZD6 | frizzled class receptor 6 [Source:HGNC Symbol;Acc:HGNC:4044] | 35028 | -0.136 | -0.3749 | Yes |
| 77 | PRKCA | protein kinase C alpha [Source:HGNC Symbol;Acc:HGNC:9393] | 35223 | -0.140 | -0.3687 | Yes |
| 78 | ADCY9 | adenylate cyclase 9 [Source:HGNC Symbol;Acc:HGNC:240] | 35385 | -0.144 | -0.3614 | Yes |
| 79 | CTNNB1 | catenin beta 1 [Source:HGNC Symbol;Acc:HGNC:2514] | 35880 | -0.157 | -0.3615 | Yes |
| 80 | WNT9B | Wnt family member 9B [Source:HGNC Symbol;Acc:HGNC:12779] | 35896 | -0.157 | -0.3493 | Yes |
| 81 | WNT2B | Wnt family member 2B [Source:HGNC Symbol;Acc:HGNC:12781] | 36087 | -0.163 | -0.3412 | Yes |
| 82 | MC1R | melanocortin 1 receptor [Source:HGNC Symbol;Acc:HGNC:6929] | 36187 | -0.166 | -0.3305 | Yes |
| 83 | GNAQ | G protein subunit alpha q [Source:HGNC Symbol;Acc:HGNC:4390] | 36400 | -0.173 | -0.3222 | Yes |
| 84 | EP300 | E1A binding protein p300 [Source:HGNC Symbol;Acc:HGNC:3373] | 36409 | -0.173 | -0.3086 | Yes |
| 85 | EDN1 | endothelin 1 [Source:HGNC Symbol;Acc:HGNC:3176] | 36917 | -0.194 | -0.3061 | Yes |
| 86 | KRAS | "KRAS proto-oncogene, GTPase [Source:HGNC Symbol;Acc:HGNC:6407]" | 36928 | -0.194 | -0.2909 | Yes |
| 87 | DCT | dopachrome tautomerase [Source:HGNC Symbol;Acc:HGNC:2709] | 37432 | -0.219 | -0.2862 | Yes |
| 88 | CREBBP | CREB binding protein [Source:HGNC Symbol;Acc:HGNC:2348] | 37476 | -0.222 | -0.2697 | Yes |
| 89 | WNT11 | Wnt family member 11 [Source:HGNC Symbol;Acc:HGNC:12776] | 37666 | -0.236 | -0.2558 | Yes |
| 90 | ADCY5 | adenylate cyclase 5 [Source:HGNC Symbol;Acc:HGNC:236] | 37762 | -0.242 | -0.2389 | Yes |
| 91 | POMC | proopiomelanocortin [Source:HGNC Symbol;Acc:HGNC:9201] | 37823 | -0.246 | -0.2209 | Yes |
| 92 | DVL3 | dishevelled segment polarity protein 3 [Source:HGNC Symbol;Acc:HGNC:3087] | 37983 | -0.264 | -0.2039 | Yes |
| 93 | WNT3 | Wnt family member 3 [Source:HGNC Symbol;Acc:HGNC:12782] | 38054 | -0.271 | -0.1841 | Yes |
| 94 | ADCY8 | adenylate cyclase 8 [Source:HGNC Symbol;Acc:HGNC:239] | 38268 | -0.302 | -0.1655 | Yes |
| 95 | PLCB4 | phospholipase C beta 4 [Source:HGNC Symbol;Acc:HGNC:9059] | 38308 | -0.309 | -0.1419 | Yes |
| 96 | LEF1 | lymphoid enhancer binding factor 1 [Source:HGNC Symbol;Acc:HGNC:6551] | 38341 | -0.316 | -0.1176 | Yes |
| 97 | FZD10 | frizzled class receptor 10 [Source:HGNC Symbol;Acc:HGNC:4039] | 38368 | -0.322 | -0.0926 | Yes |
| 98 | ADCY3 | adenylate cyclase 3 [Source:HGNC Symbol;Acc:HGNC:234] | 38423 | -0.337 | -0.0671 | Yes |
| 99 | FZD7 | frizzled class receptor 7 [Source:HGNC Symbol;Acc:HGNC:4045] | 38485 | -0.367 | -0.0394 | Yes |
| 100 | WNT5A | Wnt family member 5A [Source:HGNC Symbol;Acc:HGNC:12784] | 38488 | -0.368 | -0.0102 | Yes |
| 101 | PRKX | protein kinase X-linked [Source:HGNC Symbol;Acc:HGNC:9441] | 38524 | -0.395 | 0.0204 | Yes |
Table: GSEA details [plain text format]

  

Fig 2: KEGG\_MELANOGENESIS      
 Blue-Pink O' Gram in the Space of the Analyzed GeneSet

  

Fig 3: KEGG\_MELANOGENESIS: Random ES distribution      
 Gene set null distribution of ES for **KEGG\_MELANOGENESIS**
