# Supplementary material for: Simultaneous Presentation of Multiple Myeloma and Lung Cancer: Case Report and Gene Bioinformatics Analysis
Source: Front Oncol. 2022 Jun 13;12:859735. doi: 10.3389/fonc.2022.859735 (PMC9235397; doi:10.3389/fonc.2022.859735)
Supplement: Supplementary file 1 [file DataSheet_1.zip › The bioinformatic analysis of MM and lung cancer supplementary materials/Enrichment analysis/MECR/GSEA_4.1.0/LUSC TCGA/KEGG.Gsea.1639042374131/KEGG_METABOLISM_OF_XENOBIOTICS_BY_CYTOCHROME_P450.html]

Details for gene set KEGG\_METABOLISM\_OF\_XENOBIOTICS\_BY\_CYTOCHROME\_P450[GSEA]

|  || Dataset | ExpData\_collapsed\_to\_symbols.ENSG00000116353\_profile\_in\_ExpData.cls #ENSG00000116353 |
| Phenotype | ENSG00000116353\_profile\_in\_ExpData.cls#ENSG00000116353 |
| Upregulated in class | ENSG00000116353\_neg |
| GeneSet | KEGG\_METABOLISM\_OF\_XENOBIOTICS\_BY\_CYTOCHROME\_P450 |
| Enrichment Score (ES) | -0.4513911 |
| Normalized Enrichment Score (NES) | -1.8139774 |
| Nominal p-value | 0.0 |
| FDR q-value | 0.008270946 |
| FWER p-Value | 0.101 |
Table: GSEA Results Summary

  

Fig 1: Enrichment plot: KEGG\_METABOLISM\_OF\_XENOBIOTICS\_BY\_CYTOCHROME\_P450      
 Profile of the Running ES Score & Positions of GeneSet Members on the Rank Ordered List

  

| SYMBOL | TITLE | RANK IN GENE LIST | RANK METRIC SCORE | RUNNING ES | CORE ENRICHMENT || 1 | GSTK1 | glutathione S-transferase kappa 1 [Source:HGNC Symbol;Acc:HGNC:16906] | 626 | 0.254 | 0.0133 | No |
| 2 | UGT2B7 | UDP glucuronosyltransferase family 2 member B7 [Source:HGNC Symbol;Acc:HGNC:12554] | 1240 | 0.201 | 0.0207 | No |
| 3 | MGST3 | microsomal glutathione S-transferase 3 [Source:HGNC Symbol;Acc:HGNC:7064] | 1757 | 0.175 | 0.0277 | No |
| 4 | ADH5 | "alcohol dehydrogenase 5 (class III), chi polypeptide [Source:HGNC Symbol;Acc:HGNC:253]" | 1983 | 0.166 | 0.0411 | No |
| 5 | GSTO2 | glutathione S-transferase omega 2 [Source:HGNC Symbol;Acc:HGNC:23064] | 2291 | 0.156 | 0.0512 | No |
| 6 | UGT2B11 | UDP glucuronosyltransferase family 2 member B11 [Source:HGNC Symbol;Acc:HGNC:12545] | 2400 | 0.152 | 0.0659 | No |
| 7 | DHDH | dihydrodiol dehydrogenase [Source:HGNC Symbol;Acc:HGNC:17887] | 2561 | 0.148 | 0.0789 | No |
| 8 | UGT2B10 | UDP glucuronosyltransferase family 2 member B10 [Source:HGNC Symbol;Acc:HGNC:12544] | 3275 | 0.131 | 0.0758 | No |
| 9 | ALDH1A3 | aldehyde dehydrogenase 1 family member A3 [Source:HGNC Symbol;Acc:HGNC:409] | 3482 | 0.126 | 0.0851 | No |
| 10 | GSTZ1 | glutathione S-transferase zeta 1 [Source:HGNC Symbol;Acc:HGNC:4643] | 3655 | 0.124 | 0.0949 | No |
| 11 | UGT2B4 | UDP glucuronosyltransferase family 2 member B4 [Source:HGNC Symbol;Acc:HGNC:12553] | 4348 | 0.112 | 0.0902 | No |
| 12 | ALDH3B1 | aldehyde dehydrogenase 3 family member B1 [Source:HGNC Symbol;Acc:HGNC:410] | 4477 | 0.110 | 0.0996 | No |
| 13 | CYP3A4 | cytochrome P450 family 3 subfamily A member 4 [Source:HGNC Symbol;Acc:HGNC:2637] | 5264 | 0.099 | 0.0910 | No |
| 14 | UGT2B15 | UDP glucuronosyltransferase family 2 member B15 [Source:HGNC Symbol;Acc:HGNC:12546] | 5453 | 0.097 | 0.0974 | No |
| 15 | ADH1C | "alcohol dehydrogenase 1C (class I), gamma polypeptide [Source:HGNC Symbol;Acc:HGNC:251]" | 5472 | 0.097 | 0.1081 | No |
| 16 | CYP2B6 | cytochrome P450 family 2 subfamily B member 6 [Source:HGNC Symbol;Acc:HGNC:2615] | 5481 | 0.097 | 0.1191 | No |
| 17 | MGST1 | microsomal glutathione S-transferase 1 [Source:HGNC Symbol;Acc:HGNC:7061] | 6530 | 0.088 | 0.1025 | No |
| 18 | CYP2E1 | cytochrome P450 family 2 subfamily E member 1 [Source:HGNC Symbol;Acc:HGNC:2631] | 6760 | 0.086 | 0.1066 | No |
| 19 | CYP3A43 | cytochrome P450 family 3 subfamily A member 43 [Source:HGNC Symbol;Acc:HGNC:17450] | 10149 | 0.059 | 0.0270 | No |
| 20 | GSTP1 | glutathione S-transferase pi 1 [Source:HGNC Symbol;Acc:HGNC:4638] | 10456 | 0.057 | 0.0258 | No |
| 21 | UGT2A3 | UDP glucuronosyltransferase family 2 member A3 [Source:HGNC Symbol;Acc:HGNC:28528] | 11980 | 0.047 | -0.0075 | No |
| 22 | CYP3A7 | cytochrome P450 family 3 subfamily A member 7 [Source:HGNC Symbol;Acc:HGNC:2640] | 12000 | 0.047 | -0.0026 | No |
| 23 | CYP3A5 | cytochrome P450 family 3 subfamily A member 5 [Source:HGNC Symbol;Acc:HGNC:2638] | 12007 | 0.047 | 0.0027 | No |
| 24 | CYP2F1 | cytochrome P450 family 2 subfamily F member 1 [Source:HGNC Symbol;Acc:HGNC:2632] | 14139 | 0.034 | -0.0477 | No |
| 25 | UGT2B28 | UDP glucuronosyltransferase family 2 member B28 [Source:HGNC Symbol;Acc:HGNC:13479] | 14971 | 0.030 | -0.0655 | No |
| 26 | MGST2 | microsomal glutathione S-transferase 2 [Source:HGNC Symbol;Acc:HGNC:7063] | 15416 | 0.027 | -0.0737 | No |
| 27 | CYP2C8 | cytochrome P450 family 2 subfamily C member 8 [Source:HGNC Symbol;Acc:HGNC:2622] | 16429 | 0.021 | -0.0971 | No |
| 28 | CYP1A2 | cytochrome P450 family 1 subfamily A member 2 [Source:HGNC Symbol;Acc:HGNC:2596] | 17828 | 0.013 | -0.1312 | No |
| 29 | UGT2A1 | UDP glucuronosyltransferase family 2 member A1 complex locus [Source:HGNC Symbol;Acc:HGNC:12542] | 18439 | 0.010 | -0.1456 | No |
| 30 | ADH6 | alcohol dehydrogenase 6 (class V) [Source:HGNC Symbol;Acc:HGNC:255] | 19630 | 0.003 | -0.1756 | No |
| 31 | GSTA5 | glutathione S-transferase alpha 5 [Source:HGNC Symbol;Acc:HGNC:19662] | 19725 | 0.002 | -0.1777 | No |
| 32 | ADH1A | "alcohol dehydrogenase 1A (class I), alpha polypeptide [Source:HGNC Symbol;Acc:HGNC:249]" | 22804 | -0.016 | -0.2543 | No |
| 33 | GSTA2 | glutathione S-transferase alpha 2 [Source:HGNC Symbol;Acc:HGNC:4627] | 24463 | -0.027 | -0.2934 | No |
| 34 | ALDH3B2 | aldehyde dehydrogenase 3 family member B2 [Source:HGNC Symbol;Acc:HGNC:411] | 24548 | -0.028 | -0.2923 | No |
| 35 | ADH1B | "alcohol dehydrogenase 1B (class I), beta polypeptide [Source:HGNC Symbol;Acc:HGNC:250]" | 24848 | -0.030 | -0.2965 | No |
| 36 | ADH4 | "alcohol dehydrogenase 4 (class II), pi polypeptide [Source:HGNC Symbol;Acc:HGNC:252]" | 27558 | -0.050 | -0.3598 | No |
| 37 | GSTM5 | glutathione S-transferase mu 5 [Source:HGNC Symbol;Acc:HGNC:4637] | 27920 | -0.053 | -0.3629 | No |
| 38 | UGT1A10 | UDP glucuronosyltransferase family 1 member A10 [Source:HGNC Symbol;Acc:HGNC:12531] | 28144 | -0.054 | -0.3623 | No |
| 39 | UGT2B17 | UDP glucuronosyltransferase family 2 member B17 [Source:HGNC Symbol;Acc:HGNC:12547] | 29065 | -0.062 | -0.3786 | No |
| 40 | CYP1B1 | cytochrome P450 family 1 subfamily B member 1 [Source:HGNC Symbol;Acc:HGNC:2597] | 29846 | -0.069 | -0.3905 | No |
| 41 | EPHX1 | epoxide hydrolase 1 [Source:HGNC Symbol;Acc:HGNC:3401] | 29987 | -0.071 | -0.3860 | No |
| 42 | GSTT2 | glutathione S-transferase theta 2 (gene/pseudogene) [Source:HGNC Symbol;Acc:HGNC:4642] | 31663 | -0.087 | -0.4186 | No |
| 43 | UGT1A8 | UDP glucuronosyltransferase family 1 member A8 [Source:HGNC Symbol;Acc:HGNC:12540] | 32067 | -0.092 | -0.4183 | No |
| 44 | GSTO1 | glutathione S-transferase omega 1 [Source:HGNC Symbol;Acc:HGNC:13312] | 32498 | -0.097 | -0.4182 | No |
| 45 | GSTA3 | glutathione S-transferase alpha 3 [Source:HGNC Symbol;Acc:HGNC:4628] | 32853 | -0.101 | -0.4155 | No |
| 46 | GSTM1 | glutathione S-transferase mu 1 [Source:HGNC Symbol;Acc:HGNC:4632] | 33080 | -0.105 | -0.4092 | No |
| 47 | CYP1A1 | cytochrome P450 family 1 subfamily A member 1 [Source:HGNC Symbol;Acc:HGNC:2595] | 33824 | -0.115 | -0.4149 | No |
| 48 | UGT1A4 | UDP glucuronosyltransferase family 1 member A4 [Source:HGNC Symbol;Acc:HGNC:12536] | 35257 | -0.141 | -0.4352 | Yes |
| 49 | CYP2C9 | cytochrome P450 family 2 subfamily C member 9 [Source:HGNC Symbol;Acc:HGNC:2623] | 35384 | -0.144 | -0.4218 | Yes |
| 50 | UGT1A1 | UDP glucuronosyltransferase family 1 member A1 [Source:HGNC Symbol;Acc:HGNC:12530] | 35998 | -0.160 | -0.4190 | Yes |
| 51 | GSTM3 | glutathione S-transferase mu 3 [Source:HGNC Symbol;Acc:HGNC:4635] | 36236 | -0.168 | -0.4057 | Yes |
| 52 | UGT1A9 | UDP glucuronosyltransferase family 1 member A9 [Source:HGNC Symbol;Acc:HGNC:12541] | 36291 | -0.170 | -0.3876 | Yes |
| 53 | UGT1A3 | UDP glucuronosyltransferase family 1 member A3 [Source:HGNC Symbol;Acc:HGNC:12535] | 36403 | -0.173 | -0.3705 | Yes |
| 54 | UGT1A6 | UDP glucuronosyltransferase family 1 member A6 [Source:HGNC Symbol;Acc:HGNC:12538] | 36644 | -0.182 | -0.3557 | Yes |
| 55 | CYP2C19 | cytochrome P450 family 2 subfamily C member 19 [Source:HGNC Symbol;Acc:HGNC:2621] | 36692 | -0.184 | -0.3357 | Yes |
| 56 | ADH7 | "alcohol dehydrogenase 7 (class IV), mu or sigma polypeptide [Source:HGNC Symbol;Acc:HGNC:256]" | 37003 | -0.197 | -0.3209 | Yes |
| 57 | GSTA1 | glutathione S-transferase alpha 1 [Source:HGNC Symbol;Acc:HGNC:4626] | 37045 | -0.199 | -0.2990 | Yes |
| 58 | UGT1A5 | UDP glucuronosyltransferase family 1 member A5 [Source:HGNC Symbol;Acc:HGNC:12537] | 37114 | -0.203 | -0.2774 | Yes |
| 59 | CYP2S1 | cytochrome P450 family 2 subfamily S member 1 [Source:HGNC Symbol;Acc:HGNC:15654] | 37397 | -0.217 | -0.2596 | Yes |
| 60 | CYP2C18 | cytochrome P450 family 2 subfamily C member 18 [Source:HGNC Symbol;Acc:HGNC:2620] | 37643 | -0.234 | -0.2389 | Yes |
| 61 | AKR1C2 | aldo-keto reductase family 1 member C2 [Source:HGNC Symbol;Acc:HGNC:385] | 37784 | -0.243 | -0.2145 | Yes |
| 62 | GSTM4 | glutathione S-transferase mu 4 [Source:HGNC Symbol;Acc:HGNC:4636] | 37817 | -0.246 | -0.1870 | Yes |
| 63 | GSTM2 | glutathione S-transferase mu 2 [Source:HGNC Symbol;Acc:HGNC:4634] | 37913 | -0.256 | -0.1600 | Yes |
| 64 | ALDH3A1 | aldehyde dehydrogenase 3 family member A1 [Source:HGNC Symbol;Acc:HGNC:405] | 37960 | -0.261 | -0.1312 | Yes |
| 65 | AKR1C3 | aldo-keto reductase family 1 member C3 [Source:HGNC Symbol;Acc:HGNC:386] | 38007 | -0.266 | -0.1017 | Yes |
| 66 | AKR1C4 | aldo-keto reductase family 1 member C4 [Source:HGNC Symbol;Acc:HGNC:387] | 38037 | -0.269 | -0.0715 | Yes |
| 67 | GSTA4 | glutathione S-transferase alpha 4 [Source:HGNC Symbol;Acc:HGNC:4629] | 38228 | -0.297 | -0.0422 | Yes |
| 68 | AKR1C1 | aldo-keto reductase family 1 member C1 [Source:HGNC Symbol;Acc:HGNC:384] | 38240 | -0.299 | -0.0082 | Yes |
| 69 | UGT1A7 | UDP glucuronosyltransferase family 1 member A7 [Source:HGNC Symbol;Acc:HGNC:12539] | 38318 | -0.310 | 0.0256 | Yes |
Table: GSEA details [plain text format]

  

Fig 2: KEGG\_METABOLISM\_OF\_XENOBIOTICS\_BY\_CYTOCHROME\_P450      
 Blue-Pink O' Gram in the Space of the Analyzed GeneSet

  

Fig 3: KEGG\_METABOLISM\_OF\_XENOBIOTICS\_BY\_CYTOCHROME\_P450: Random ES distribution      
 Gene set null distribution of ES for **KEGG\_METABOLISM\_OF\_XENOBIOTICS\_BY\_CYTOCHROME\_P450**
