# Supplementary material for: Simultaneous Presentation of Multiple Myeloma and Lung Cancer: Case Report and Gene Bioinformatics Analysis
Source: Front Oncol. 2022 Jun 13;12:859735. doi: 10.3389/fonc.2022.859735 (PMC9235397; doi:10.3389/fonc.2022.859735)
Supplement: Supplementary file 1 [file DataSheet_1.zip › The bioinformatic analysis of MM and lung cancer supplementary materials/Enrichment analysis/MECR/GSEA_4.1.0/LUSC TCGA/KEGG.Gsea.1639042374131/KEGG_NEUROACTIVE_LIGAND_RECEPTOR_INTERACTION.html]

Details for gene set KEGG\_NEUROACTIVE\_LIGAND\_RECEPTOR\_INTERACTION[GSEA]

|  || Dataset | ExpData\_collapsed\_to\_symbols.ENSG00000116353\_profile\_in\_ExpData.cls #ENSG00000116353 |
| Phenotype | ENSG00000116353\_profile\_in\_ExpData.cls#ENSG00000116353 |
| Upregulated in class | ENSG00000116353\_neg |
| GeneSet | KEGG\_NEUROACTIVE\_LIGAND\_RECEPTOR\_INTERACTION |
| Enrichment Score (ES) | -0.38025674 |
| Normalized Enrichment Score (NES) | -1.8787692 |
| Nominal p-value | 0.0 |
| FDR q-value | 0.0060126595 |
| FWER p-Value | 0.048 |
Table: GSEA Results Summary

  

Fig 1: Enrichment plot: KEGG\_NEUROACTIVE\_LIGAND\_RECEPTOR\_INTERACTION      
 Profile of the Running ES Score & Positions of GeneSet Members on the Rank Ordered List

  

| SYMBOL | TITLE | RANK IN GENE LIST | RANK METRIC SCORE | RUNNING ES | CORE ENRICHMENT || 1 | GRM4 | glutamate metabotropic receptor 4 [Source:HGNC Symbol;Acc:HGNC:4596] | 566 | 0.261 | -0.0026 | No |
| 2 | CHRNB1 | cholinergic receptor nicotinic beta 1 subunit [Source:HGNC Symbol;Acc:HGNC:1961] | 662 | 0.250 | 0.0064 | No |
| 3 | TSPO | translocator protein [Source:HGNC Symbol;Acc:HGNC:1158] | 1499 | 0.186 | -0.0064 | No |
| 4 | GPR156 | G protein-coupled receptor 156 [Source:HGNC Symbol;Acc:HGNC:20844] | 2781 | 0.142 | -0.0327 | No |
| 5 | ADRB1 | adrenoceptor beta 1 [Source:HGNC Symbol;Acc:HGNC:285] | 2785 | 0.142 | -0.0263 | No |
| 6 | BDKRB1 | bradykinin receptor B1 [Source:HGNC Symbol;Acc:HGNC:1029] | 3078 | 0.135 | -0.0276 | No |
| 7 | GRIN3B | glutamate ionotropic receptor NMDA type subunit 3B [Source:HGNC Symbol;Acc:HGNC:16768] | 3531 | 0.126 | -0.0334 | No |
| 8 | CSH1 | chorionic somatomammotropin hormone 1 [Source:HGNC Symbol;Acc:HGNC:2440] | 3610 | 0.124 | -0.0297 | No |
| 9 | GABRA6 | gamma-aminobutyric acid type A receptor subunit alpha6 [Source:HGNC Symbol;Acc:HGNC:4080] | 3730 | 0.122 | -0.0272 | No |
| 10 | CHRNA9 | cholinergic receptor nicotinic alpha 9 subunit [Source:HGNC Symbol;Acc:HGNC:14079] | 4099 | 0.116 | -0.0313 | No |
| 11 | GRIK1 | glutamate ionotropic receptor kainate type subunit 1 [Source:HGNC Symbol;Acc:HGNC:4579] | 4238 | 0.114 | -0.0296 | No |
| 12 | OPRD1 | opioid receptor delta 1 [Source:HGNC Symbol;Acc:HGNC:8153] | 4303 | 0.113 | -0.0261 | No |
| 13 | TAAR5 | trace amine associated receptor 5 [Source:HGNC Symbol;Acc:HGNC:30236] | 4629 | 0.108 | -0.0295 | No |
| 14 | HTR1D | 5-hydroxytryptamine receptor 1D [Source:HGNC Symbol;Acc:HGNC:5289] | 4872 | 0.104 | -0.0309 | No |
| 15 | NPY4R | neuropeptide Y receptor Y4 [Source:HGNC Symbol;Acc:HGNC:9329] | 5110 | 0.101 | -0.0323 | No |
| 16 | NMBR | neuromedin B receptor [Source:HGNC Symbol;Acc:HGNC:7843] | 5369 | 0.098 | -0.0344 | No |
| 17 | KISS1R | KISS1 receptor [Source:HGNC Symbol;Acc:HGNC:4510] | 5978 | 0.094 | -0.0457 | No |
| 18 | GRIN2C | glutamate ionotropic receptor NMDA type subunit 2C [Source:HGNC Symbol;Acc:HGNC:4587] | 6018 | 0.094 | -0.0424 | No |
| 19 | GALR3 | galanin receptor 3 [Source:HGNC Symbol;Acc:HGNC:4134] | 6143 | 0.092 | -0.0413 | No |
| 20 | LHB | luteinizing hormone subunit beta [Source:HGNC Symbol;Acc:HGNC:6584] | 6189 | 0.092 | -0.0383 | No |
| 21 | AGTR1 | angiotensin II receptor type 1 [Source:HGNC Symbol;Acc:HGNC:336] | 6417 | 0.089 | -0.0400 | No |
| 22 | PRSS1 | serine protease 1 [Source:HGNC Symbol;Acc:HGNC:9475] | 6502 | 0.088 | -0.0381 | No |
| 23 | GRIN2D | glutamate ionotropic receptor NMDA type subunit 2D [Source:HGNC Symbol;Acc:HGNC:4588] | 6650 | 0.087 | -0.0379 | No |
| 24 | PTGER1 | prostaglandin E receptor 1 [Source:HGNC Symbol;Acc:HGNC:9593] | 6996 | 0.084 | -0.0429 | No |
| 25 | CGA | "glycoprotein hormones, alpha polypeptide [Source:HGNC Symbol;Acc:HGNC:1885]" | 7429 | 0.080 | -0.0503 | No |
| 26 | MTNR1B | melatonin receptor 1B [Source:HGNC Symbol;Acc:HGNC:7464] | 8026 | 0.075 | -0.0621 | No |
| 27 | GRM3 | glutamate metabotropic receptor 3 [Source:HGNC Symbol;Acc:HGNC:4595] | 8031 | 0.075 | -0.0588 | No |
| 28 | GABRA4 | gamma-aminobutyric acid type A receptor subunit alpha4 [Source:HGNC Symbol;Acc:HGNC:4078] | 8246 | 0.073 | -0.0610 | No |
| 29 | GH2 | growth hormone 2 [Source:HGNC Symbol;Acc:HGNC:4262] | 8363 | 0.072 | -0.0606 | No |
| 30 | F2 | "coagulation factor II, thrombin [Source:HGNC Symbol;Acc:HGNC:3535]" | 8593 | 0.070 | -0.0633 | No |
| 31 | DRD5 | dopamine receptor D5 [Source:HGNC Symbol;Acc:HGNC:3026] | 8680 | 0.069 | -0.0623 | No |
| 32 | ADRB2 | adrenoceptor beta 2 [Source:HGNC Symbol;Acc:HGNC:286] | 8846 | 0.068 | -0.0634 | No |
| 33 | PRLR | prolactin receptor [Source:HGNC Symbol;Acc:HGNC:9446] | 8928 | 0.068 | -0.0624 | No |
| 34 | BDKRB2 | bradykinin receptor B2 [Source:HGNC Symbol;Acc:HGNC:1030] | 8996 | 0.067 | -0.0610 | No |
| 35 | PRSS2 | serine protease 2 [Source:HGNC Symbol;Acc:HGNC:9483] | 9033 | 0.067 | -0.0589 | No |
| 36 | GLP2R | glucagon like peptide 2 receptor [Source:HGNC Symbol;Acc:HGNC:4325] | 9044 | 0.067 | -0.0561 | No |
| 37 | TAAR9 | trace amine associated receptor 9 [Source:HGNC Symbol;Acc:HGNC:20977] | 9166 | 0.066 | -0.0561 | No |
| 38 | PLG | plasminogen [Source:HGNC Symbol;Acc:HGNC:9071] | 9319 | 0.065 | -0.0571 | No |
| 39 | ADORA1 | adenosine A1 receptor [Source:HGNC Symbol;Acc:HGNC:262] | 9545 | 0.064 | -0.0599 | No |
| 40 | THRA | thyroid hormone receptor alpha [Source:HGNC Symbol;Acc:HGNC:11796] | 9967 | 0.060 | -0.0679 | No |
| 41 | TACR3 | tachykinin receptor 3 [Source:HGNC Symbol;Acc:HGNC:11528] | 10021 | 0.060 | -0.0665 | No |
| 42 | PRLHR | prolactin releasing hormone receptor [Source:HGNC Symbol;Acc:HGNC:4464] | 10121 | 0.059 | -0.0664 | No |
| 43 | TAAR1 | trace amine associated receptor 1 [Source:HGNC Symbol;Acc:HGNC:17734] | 10511 | 0.057 | -0.0737 | No |
| 44 | GABRG2 | gamma-aminobutyric acid type A receptor subunit gamma2 [Source:HGNC Symbol;Acc:HGNC:4087] | 10548 | 0.056 | -0.0721 | No |
| 45 | TAAR2 | trace amine associated receptor 2 [Source:HGNC Symbol;Acc:HGNC:4514] | 10583 | 0.056 | -0.0704 | No |
| 46 | GABRA1 | gamma-aminobutyric acid type A receptor subunit alpha1 [Source:HGNC Symbol;Acc:HGNC:4075] | 10590 | 0.056 | -0.0679 | No |
| 47 | MTNR1A | melatonin receptor 1A [Source:HGNC Symbol;Acc:HGNC:7463] | 10645 | 0.056 | -0.0668 | No |
| 48 | ADRA1B | adrenoceptor alpha 1B [Source:HGNC Symbol;Acc:HGNC:278] | 10678 | 0.056 | -0.0650 | No |
| 49 | GABRD | gamma-aminobutyric acid type A receptor subunit delta [Source:HGNC Symbol;Acc:HGNC:4084] | 10889 | 0.054 | -0.0679 | No |
| 50 | PRL | prolactin [Source:HGNC Symbol;Acc:HGNC:9445] | 10903 | 0.054 | -0.0658 | No |
| 51 | PTAFR | platelet activating factor receptor [Source:HGNC Symbol;Acc:HGNC:9582] | 11052 | 0.053 | -0.0671 | No |
| 52 | HTR1E | 5-hydroxytryptamine receptor 1E [Source:HGNC Symbol;Acc:HGNC:5291] | 11115 | 0.053 | -0.0663 | No |
| 53 | HRH1 | histamine receptor H1 [Source:HGNC Symbol;Acc:HGNC:5182] | 11260 | 0.052 | -0.0676 | No |
| 54 | HRH3 | histamine receptor H3 [Source:HGNC Symbol;Acc:HGNC:5184] | 11572 | 0.050 | -0.0733 | No |
| 55 | LEP | leptin [Source:HGNC Symbol;Acc:HGNC:6553] | 11598 | 0.050 | -0.0716 | No |
| 56 | GABRB2 | gamma-aminobutyric acid type A receptor subunit beta2 [Source:HGNC Symbol;Acc:HGNC:4082] | 11662 | 0.049 | -0.0710 | No |
| 57 | SSTR1 | somatostatin receptor 1 [Source:HGNC Symbol;Acc:HGNC:11330] | 11722 | 0.049 | -0.0703 | No |
| 58 | PTH1R | parathyroid hormone 1 receptor [Source:HGNC Symbol;Acc:HGNC:9608] | 11746 | 0.049 | -0.0686 | No |
| 59 | FPR2 | formyl peptide receptor 2 [Source:HGNC Symbol;Acc:HGNC:3827] | 11750 | 0.049 | -0.0665 | No |
| 60 | S1PR2 | sphingosine-1-phosphate receptor 2 [Source:HGNC Symbol;Acc:HGNC:3169] | 11947 | 0.047 | -0.0693 | No |
| 61 | GRIA2 | glutamate ionotropic receptor AMPA type subunit 2 [Source:HGNC Symbol;Acc:HGNC:4572] | 11976 | 0.047 | -0.0679 | No |
| 62 | P2RY2 | purinergic receptor P2Y2 [Source:HGNC Symbol;Acc:HGNC:8541] | 12355 | 0.045 | -0.0755 | No |
| 63 | NPFFR2 | neuropeptide FF receptor 2 [Source:HGNC Symbol;Acc:HGNC:4525] | 12447 | 0.044 | -0.0758 | No |
| 64 | GRID2 | glutamate ionotropic receptor delta type subunit 2 [Source:HGNC Symbol;Acc:HGNC:4576] | 12687 | 0.043 | -0.0799 | No |
| 65 | TSHB | thyroid stimulating hormone subunit beta [Source:HGNC Symbol;Acc:HGNC:12372] | 12729 | 0.042 | -0.0791 | No |
| 66 | P2RY6 | pyrimidinergic receptor P2Y6 [Source:HGNC Symbol;Acc:HGNC:8543] | 12763 | 0.042 | -0.0780 | No |
| 67 | GLRA1 | glycine receptor alpha 1 [Source:HGNC Symbol;Acc:HGNC:4326] | 13213 | 0.040 | -0.0876 | No |
| 68 | GRIN1 | glutamate ionotropic receptor NMDA type subunit 1 [Source:HGNC Symbol;Acc:HGNC:4584] | 13266 | 0.039 | -0.0872 | No |
| 69 | ADORA2B | adenosine A2b receptor [Source:HGNC Symbol;Acc:HGNC:264] | 14239 | 0.033 | -0.1105 | No |
| 70 | AVPR2 | arginine vasopressin receptor 2 [Source:HGNC Symbol;Acc:HGNC:897] | 14243 | 0.033 | -0.1091 | No |
| 71 | SSTR5 | somatostatin receptor 5 [Source:HGNC Symbol;Acc:HGNC:11334] | 14745 | 0.031 | -0.1205 | No |
| 72 | TAAR8 | trace amine associated receptor 8 [Source:HGNC Symbol;Acc:HGNC:14964] | 15125 | 0.029 | -0.1289 | No |
| 73 | OPRL1 | opioid related nociceptin receptor 1 [Source:HGNC Symbol;Acc:HGNC:8155] | 15632 | 0.025 | -0.1407 | No |
| 74 | CHRND | cholinergic receptor nicotinic delta subunit [Source:HGNC Symbol;Acc:HGNC:1965] | 15684 | 0.025 | -0.1408 | No |
| 75 | ADRB3 | adrenoceptor beta 3 [Source:HGNC Symbol;Acc:HGNC:288] | 15881 | 0.024 | -0.1448 | No |
| 76 | ADRA1D | adrenoceptor alpha 1D [Source:HGNC Symbol;Acc:HGNC:280] | 16099 | 0.023 | -0.1493 | No |
| 77 | MLNR | motilin receptor [Source:HGNC Symbol;Acc:HGNC:4495] | 16503 | 0.020 | -0.1587 | No |
| 78 | FPR1 | formyl peptide receptor 1 [Source:HGNC Symbol;Acc:HGNC:3826] | 16615 | 0.020 | -0.1606 | No |
| 79 | TBXA2R | thromboxane A2 receptor [Source:HGNC Symbol;Acc:HGNC:11608] | 16620 | 0.020 | -0.1598 | No |
| 80 | GPR35 | G protein-coupled receptor 35 [Source:HGNC Symbol;Acc:HGNC:4492] | 16673 | 0.020 | -0.1602 | No |
| 81 | SSTR4 | somatostatin receptor 4 [Source:HGNC Symbol;Acc:HGNC:11333] | 16714 | 0.019 | -0.1604 | No |
| 82 | EDNRA | endothelin receptor type A [Source:HGNC Symbol;Acc:HGNC:3179] | 16969 | 0.018 | -0.1661 | No |
| 83 | SCTR | secretin receptor [Source:HGNC Symbol;Acc:HGNC:10608] | 17350 | 0.016 | -0.1751 | No |
| 84 | MC2R | melanocortin 2 receptor [Source:HGNC Symbol;Acc:HGNC:6930] | 17625 | 0.014 | -0.1814 | No |
| 85 | GABRB1 | gamma-aminobutyric acid type A receptor subunit beta1 [Source:HGNC Symbol;Acc:HGNC:4081] | 17629 | 0.014 | -0.1809 | No |
| 86 | FSHB | follicle stimulating hormone subunit beta [Source:HGNC Symbol;Acc:HGNC:3964] | 17867 | 0.013 | -0.1863 | No |
| 87 | P2RX3 | purinergic receptor P2X 3 [Source:HGNC Symbol;Acc:HGNC:8534] | 18177 | 0.011 | -0.1937 | No |
| 88 | F2RL3 | F2R like thrombin or trypsin receptor 3 [Source:HGNC Symbol;Acc:HGNC:3540] | 18442 | 0.010 | -0.2001 | No |
| 89 | NTSR1 | neurotensin receptor 1 [Source:HGNC Symbol;Acc:HGNC:8039] | 18697 | 0.008 | -0.2062 | No |
| 90 | GLRA3 | glycine receptor alpha 3 [Source:HGNC Symbol;Acc:HGNC:4328] | 18966 | 0.007 | -0.2127 | No |
| 91 | GRM6 | glutamate metabotropic receptor 6 [Source:HGNC Symbol;Acc:HGNC:4598] | 19172 | 0.006 | -0.2177 | No |
| 92 | HTR5A | 5-hydroxytryptamine receptor 5A [Source:HGNC Symbol;Acc:HGNC:5300] | 19365 | 0.004 | -0.2224 | No |
| 93 | HCRTR1 | hypocretin receptor 1 [Source:HGNC Symbol;Acc:HGNC:4848] | 19423 | 0.004 | -0.2237 | No |
| 94 | NPBWR2 | neuropeptides B and W receptor 2 [Source:HGNC Symbol;Acc:HGNC:4530] | 19479 | 0.004 | -0.2250 | No |
| 95 | ADORA2A | adenosine A2a receptor [Source:HGNC Symbol;Acc:HGNC:263] | 19525 | 0.003 | -0.2260 | No |
| 96 | LEPR | leptin receptor [Source:HGNC Symbol;Acc:HGNC:6554] | 19563 | 0.003 | -0.2268 | No |
| 97 | HTR7 | 5-hydroxytryptamine receptor 7 [Source:HGNC Symbol;Acc:HGNC:5302] | 19671 | 0.003 | -0.2294 | No |
| 98 | P2RX6 | purinergic receptor P2X 6 [Source:HGNC Symbol;Acc:HGNC:8538] | 19684 | 0.003 | -0.2296 | No |
| 99 | AGTR2 | angiotensin II receptor type 2 [Source:HGNC Symbol;Acc:HGNC:338] | 19894 | 0.001 | -0.2349 | No |
| 100 | MC3R | melanocortin 3 receptor [Source:HGNC Symbol;Acc:HGNC:6931] | 20005 | 0.001 | -0.2376 | No |
| 101 | FSHR | follicle stimulating hormone receptor [Source:HGNC Symbol;Acc:HGNC:3969] | 20329 | -0.001 | -0.2459 | No |
| 102 | TRHR | thyrotropin releasing hormone receptor [Source:HGNC Symbol;Acc:HGNC:12299] | 20367 | -0.002 | -0.2467 | No |
| 103 | NPFFR1 | neuropeptide FF receptor 1 [Source:HGNC Symbol;Acc:HGNC:17425] | 20922 | -0.005 | -0.2607 | No |
| 104 | LPAR3 | lysophosphatidic acid receptor 3 [Source:HGNC Symbol;Acc:HGNC:14298] | 20967 | -0.005 | -0.2616 | No |
| 105 | HTR2B | 5-hydroxytryptamine receptor 2B [Source:HGNC Symbol;Acc:HGNC:5294] | 20981 | -0.005 | -0.2617 | No |
| 106 | LPAR2 | lysophosphatidic acid receptor 2 [Source:HGNC Symbol;Acc:HGNC:3168] | 21083 | -0.006 | -0.2640 | No |
| 107 | RXFP2 | relaxin family peptide receptor 2 [Source:HGNC Symbol;Acc:HGNC:17318] | 21261 | -0.007 | -0.2682 | No |
| 108 | LPAR6 | lysophosphatidic acid receptor 6 [Source:HGNC Symbol;Acc:HGNC:15520] | 21700 | -0.009 | -0.2790 | No |
| 109 | TAAR6 | trace amine associated receptor 6 [Source:HGNC Symbol;Acc:HGNC:20978] | 21760 | -0.010 | -0.2801 | No |
| 110 | GRIN2A | glutamate ionotropic receptor NMDA type subunit 2A [Source:HGNC Symbol;Acc:HGNC:4585] | 21887 | -0.010 | -0.2828 | No |
| 111 | AVPR1B | arginine vasopressin receptor 1B [Source:HGNC Symbol;Acc:HGNC:896] | 21931 | -0.011 | -0.2834 | No |
| 112 | CHRNA5 | cholinergic receptor nicotinic alpha 5 subunit [Source:HGNC Symbol;Acc:HGNC:1959] | 22032 | -0.011 | -0.2855 | No |
| 113 | PTGER2 | prostaglandin E receptor 2 [Source:HGNC Symbol;Acc:HGNC:9594] | 22067 | -0.012 | -0.2858 | No |
| 114 | CHRNG | cholinergic receptor nicotinic gamma subunit [Source:HGNC Symbol;Acc:HGNC:1967] | 22078 | -0.012 | -0.2855 | No |
| 115 | THRB | thyroid hormone receptor beta [Source:HGNC Symbol;Acc:HGNC:11799] | 22129 | -0.012 | -0.2862 | No |
| 116 | HTR2C | 5-hydroxytryptamine receptor 2C [Source:HGNC Symbol;Acc:HGNC:5295] | 22469 | -0.014 | -0.2943 | No |
| 117 | MC5R | melanocortin 5 receptor [Source:HGNC Symbol;Acc:HGNC:6933] | 22512 | -0.014 | -0.2947 | No |
| 118 | CHRNE | cholinergic receptor nicotinic epsilon subunit [Source:HGNC Symbol;Acc:HGNC:1966] | 22817 | -0.016 | -0.3017 | No |
| 119 | HTR1F | 5-hydroxytryptamine receptor 1F [Source:HGNC Symbol;Acc:HGNC:5292] | 23321 | -0.019 | -0.3137 | No |
| 120 | HRH4 | histamine receptor H4 [Source:HGNC Symbol;Acc:HGNC:17383] | 23482 | -0.021 | -0.3169 | No |
| 121 | MC4R | melanocortin 4 receptor [Source:HGNC Symbol;Acc:HGNC:6932] | 23510 | -0.021 | -0.3166 | No |
| 122 | HTR6 | 5-hydroxytryptamine receptor 6 [Source:HGNC Symbol;Acc:HGNC:5301] | 23751 | -0.022 | -0.3218 | No |
| 123 | CHRM4 | cholinergic receptor muscarinic 4 [Source:HGNC Symbol;Acc:HGNC:1953] | 23958 | -0.023 | -0.3260 | No |
| 124 | NPY1R | neuropeptide Y receptor Y1 [Source:HGNC Symbol;Acc:HGNC:7956] | 23985 | -0.024 | -0.3255 | No |
| 125 | GRIK5 | glutamate ionotropic receptor kainate type subunit 5 [Source:HGNC Symbol;Acc:HGNC:4583] | 24254 | -0.026 | -0.3312 | No |
| 126 | GIPR | gastric inhibitory polypeptide receptor [Source:HGNC Symbol;Acc:HGNC:4271] | 24284 | -0.026 | -0.3308 | No |
| 127 | HRH2 | histamine receptor H2 [Source:HGNC Symbol;Acc:HGNC:5183] | 24462 | -0.027 | -0.3341 | No |
| 128 | CYSLTR1 | cysteinyl leukotriene receptor 1 [Source:HGNC Symbol;Acc:HGNC:17451] | 24477 | -0.027 | -0.3332 | No |
| 129 | LHCGR | luteinizing hormone/choriogonadotropin receptor [Source:HGNC Symbol;Acc:HGNC:6585] | 24877 | -0.030 | -0.3420 | No |
| 130 | OPRM1 | opioid receptor mu 1 [Source:HGNC Symbol;Acc:HGNC:8156] | 24947 | -0.031 | -0.3424 | No |
| 131 | MCHR1 | melanin concentrating hormone receptor 1 [Source:HGNC Symbol;Acc:HGNC:4479] | 25069 | -0.032 | -0.3440 | No |
| 132 | CHRNA1 | cholinergic receptor nicotinic alpha 1 subunit [Source:HGNC Symbol;Acc:HGNC:1955] | 25119 | -0.032 | -0.3438 | No |
| 133 | GRIA4 | glutamate ionotropic receptor AMPA type subunit 4 [Source:HGNC Symbol;Acc:HGNC:4574] | 25155 | -0.032 | -0.3432 | No |
| 134 | EDNRB | endothelin receptor type B [Source:HGNC Symbol;Acc:HGNC:3180] | 25161 | -0.032 | -0.3419 | No |
| 135 | PTGIR | prostaglandin I2 receptor [Source:HGNC Symbol;Acc:HGNC:9602] | 25187 | -0.033 | -0.3410 | No |
| 136 | P2RX4 | purinergic receptor P2X 4 [Source:HGNC Symbol;Acc:HGNC:8535] | 25205 | -0.033 | -0.3400 | No |
| 137 | GH1 | growth hormone 1 [Source:HGNC Symbol;Acc:HGNC:4261] | 25279 | -0.033 | -0.3403 | No |
| 138 | GRIA1 | glutamate ionotropic receptor AMPA type subunit 1 [Source:HGNC Symbol;Acc:HGNC:4571] | 25381 | -0.034 | -0.3413 | No |
| 139 | HTR4 | 5-hydroxytryptamine receptor 4 [Source:HGNC Symbol;Acc:HGNC:5299] | 25429 | -0.034 | -0.3410 | No |
| 140 | GABRA3 | gamma-aminobutyric acid type A receptor subunit alpha3 [Source:HGNC Symbol;Acc:HGNC:4077] | 25621 | -0.036 | -0.3442 | No |
| 141 | OXTR | oxytocin receptor [Source:HGNC Symbol;Acc:HGNC:8529] | 25650 | -0.036 | -0.3433 | No |
| 142 | GLRB | glycine receptor beta [Source:HGNC Symbol;Acc:HGNC:4329] | 25719 | -0.036 | -0.3434 | No |
| 143 | CHRNB3 | cholinergic receptor nicotinic beta 3 subunit [Source:HGNC Symbol;Acc:HGNC:1963] | 25965 | -0.038 | -0.3480 | No |
| 144 | CHRNA4 | cholinergic receptor nicotinic alpha 4 subunit [Source:HGNC Symbol;Acc:HGNC:1958] | 25968 | -0.038 | -0.3463 | No |
| 145 | GNRHR | gonadotropin releasing hormone receptor [Source:HGNC Symbol;Acc:HGNC:4421] | 26237 | -0.040 | -0.3513 | No |
| 146 | S1PR4 | sphingosine-1-phosphate receptor 4 [Source:HGNC Symbol;Acc:HGNC:3170] | 26322 | -0.040 | -0.3516 | No |
| 147 | GALR2 | galanin receptor 2 [Source:HGNC Symbol;Acc:HGNC:4133] | 26467 | -0.041 | -0.3534 | No |
| 148 | DRD4 | dopamine receptor D4 [Source:HGNC Symbol;Acc:HGNC:3025] | 26798 | -0.044 | -0.3598 | No |
| 149 | GRM5 | glutamate metabotropic receptor 5 [Source:HGNC Symbol;Acc:HGNC:4597] | 27139 | -0.047 | -0.3664 | No |
| 150 | GABBR2 | gamma-aminobutyric acid type B receptor subunit 2 [Source:HGNC Symbol;Acc:HGNC:4507] | 27194 | -0.047 | -0.3656 | No |
| 151 | P2RY4 | pyrimidinergic receptor P2Y4 [Source:HGNC Symbol;Acc:HGNC:8542] | 27198 | -0.047 | -0.3635 | No |
| 152 | CALCR | calcitonin receptor [Source:HGNC Symbol;Acc:HGNC:1440] | 27274 | -0.048 | -0.3633 | No |
| 153 | UTS2R | urotensin 2 receptor [Source:HGNC Symbol;Acc:HGNC:4468] | 27320 | -0.048 | -0.3622 | No |
| 154 | MCHR2 | melanin concentrating hormone receptor 2 [Source:HGNC Symbol;Acc:HGNC:20867] | 27372 | -0.048 | -0.3613 | No |
| 155 | HTR1A | 5-hydroxytryptamine receptor 1A [Source:HGNC Symbol;Acc:HGNC:5286] | 27410 | -0.049 | -0.3600 | No |
| 156 | GRIK2 | glutamate ionotropic receptor kainate type subunit 2 [Source:HGNC Symbol;Acc:HGNC:4580] | 27444 | -0.049 | -0.3586 | No |
| 157 | P2RX1 | purinergic receptor P2X 1 [Source:HGNC Symbol;Acc:HGNC:8533] | 27587 | -0.050 | -0.3600 | No |
| 158 | PTGER4 | prostaglandin E receptor 4 [Source:HGNC Symbol;Acc:HGNC:9596] | 27772 | -0.052 | -0.3623 | No |
| 159 | TACR1 | tachykinin receptor 1 [Source:HGNC Symbol;Acc:HGNC:11526] | 27911 | -0.053 | -0.3635 | No |
| 160 | GABRQ | gamma-aminobutyric acid type A receptor subunit theta [Source:HGNC Symbol;Acc:HGNC:14454] | 27930 | -0.053 | -0.3615 | No |
| 161 | GRIK4 | glutamate ionotropic receptor kainate type subunit 4 [Source:HGNC Symbol;Acc:HGNC:4582] | 28329 | -0.056 | -0.3691 | No |
| 162 | GZMA | granzyme A [Source:HGNC Symbol;Acc:HGNC:4708] | 28420 | -0.057 | -0.3688 | No |
| 163 | GABRP | gamma-aminobutyric acid type A receptor subunit pi [Source:HGNC Symbol;Acc:HGNC:4089] | 28504 | -0.057 | -0.3683 | No |
| 164 | NTSR2 | neurotensin receptor 2 [Source:HGNC Symbol;Acc:HGNC:8040] | 28540 | -0.058 | -0.3666 | No |
| 165 | CHRM2 | cholinergic receptor muscarinic 2 [Source:HGNC Symbol;Acc:HGNC:1951] | 28670 | -0.059 | -0.3672 | No |
| 166 | DRD2 | dopamine receptor D2 [Source:HGNC Symbol;Acc:HGNC:3023] | 28890 | -0.060 | -0.3701 | No |
| 167 | OPRK1 | opioid receptor kappa 1 [Source:HGNC Symbol;Acc:HGNC:8154] | 29226 | -0.064 | -0.3757 | No |
| 168 | CHRNA2 | cholinergic receptor nicotinic alpha 2 subunit [Source:HGNC Symbol;Acc:HGNC:1956] | 29404 | -0.065 | -0.3773 | Yes |
| 169 | P2RX2 | purinergic receptor P2X 2 [Source:HGNC Symbol;Acc:HGNC:15459] | 29406 | -0.065 | -0.3743 | Yes |
| 170 | CTSG | cathepsin G [Source:HGNC Symbol;Acc:HGNC:2532] | 29467 | -0.066 | -0.3728 | Yes |
| 171 | NMUR1 | neuromedin U receptor 1 [Source:HGNC Symbol;Acc:HGNC:4518] | 29540 | -0.066 | -0.3716 | Yes |
| 172 | SSTR2 | somatostatin receptor 2 [Source:HGNC Symbol;Acc:HGNC:11331] | 29636 | -0.067 | -0.3710 | Yes |
| 173 | P2RY14 | purinergic receptor P2Y14 [Source:HGNC Symbol;Acc:HGNC:16442] | 29658 | -0.067 | -0.3685 | Yes |
| 174 | TSHR | thyroid stimulating hormone receptor [Source:HGNC Symbol;Acc:HGNC:12373] | 29716 | -0.068 | -0.3668 | Yes |
| 175 | HCRTR2 | hypocretin receptor 2 [Source:HGNC Symbol;Acc:HGNC:4849] | 29906 | -0.070 | -0.3684 | Yes |
| 176 | DRD1 | dopamine receptor D1 [Source:HGNC Symbol;Acc:HGNC:3020] | 29908 | -0.070 | -0.3653 | Yes |
| 177 | DRD3 | dopamine receptor D3 [Source:HGNC Symbol;Acc:HGNC:3024] | 30078 | -0.071 | -0.3663 | Yes |
| 178 | P2RY13 | purinergic receptor P2Y13 [Source:HGNC Symbol;Acc:HGNC:4537] | 30173 | -0.072 | -0.3654 | Yes |
| 179 | TRPV1 | transient receptor potential cation channel subfamily V member 1 [Source:HGNC Symbol;Acc:HGNC:12716] | 30323 | -0.074 | -0.3659 | Yes |
| 180 | GALR1 | galanin receptor 1 [Source:HGNC Symbol;Acc:HGNC:4132] | 30340 | -0.074 | -0.3629 | Yes |
| 181 | VIPR1 | vasoactive intestinal peptide receptor 1 [Source:HGNC Symbol;Acc:HGNC:12694] | 30638 | -0.077 | -0.3670 | Yes |
| 182 | CCKBR | cholecystokinin B receptor [Source:HGNC Symbol;Acc:HGNC:1571] | 30725 | -0.077 | -0.3656 | Yes |
| 183 | CNR1 | cannabinoid receptor 1 [Source:HGNC Symbol;Acc:HGNC:2159] | 30736 | -0.078 | -0.3623 | Yes |
| 184 | GRIA3 | glutamate ionotropic receptor AMPA type subunit 3 [Source:HGNC Symbol;Acc:HGNC:4573] | 30996 | -0.080 | -0.3653 | Yes |
| 185 | GABRB3 | gamma-aminobutyric acid type A receptor subunit beta3 [Source:HGNC Symbol;Acc:HGNC:4083] | 31016 | -0.080 | -0.3621 | Yes |
| 186 | VIPR2 | vasoactive intestinal peptide receptor 2 [Source:HGNC Symbol;Acc:HGNC:12695] | 31061 | -0.081 | -0.3595 | Yes |
| 187 | S1PR1 | sphingosine-1-phosphate receptor 1 [Source:HGNC Symbol;Acc:HGNC:3165] | 31090 | -0.081 | -0.3565 | Yes |
| 188 | GRIN2B | glutamate ionotropic receptor NMDA type subunit 2B [Source:HGNC Symbol;Acc:HGNC:4586] | 31248 | -0.083 | -0.3567 | Yes |
| 189 | C3AR1 | complement C3a receptor 1 [Source:HGNC Symbol;Acc:HGNC:1319] | 31452 | -0.085 | -0.3581 | Yes |
| 190 | GCGR | glucagon receptor [Source:HGNC Symbol;Acc:HGNC:4192] | 31501 | -0.085 | -0.3554 | Yes |
| 191 | ADRA2C | adrenoceptor alpha 2C [Source:HGNC Symbol;Acc:HGNC:283] | 31670 | -0.087 | -0.3557 | Yes |
| 192 | CHRNA6 | cholinergic receptor nicotinic alpha 6 subunit [Source:HGNC Symbol;Acc:HGNC:15963] | 31785 | -0.088 | -0.3546 | Yes |
| 193 | BRS3 | bombesin receptor subtype 3 [Source:HGNC Symbol;Acc:HGNC:1113] | 31857 | -0.089 | -0.3523 | Yes |
| 194 | CHRNA3 | cholinergic receptor nicotinic alpha 3 subunit [Source:HGNC Symbol;Acc:HGNC:1957] | 31866 | -0.089 | -0.3484 | Yes |
| 195 | GABRG1 | gamma-aminobutyric acid type A receptor subunit gamma1 [Source:HGNC Symbol;Acc:HGNC:4086] | 31887 | -0.089 | -0.3448 | Yes |
| 196 | LTB4R2 | leukotriene B4 receptor 2 [Source:HGNC Symbol;Acc:HGNC:19260] | 31909 | -0.090 | -0.3413 | Yes |
| 197 | RXFP1 | relaxin family peptide receptor 1 [Source:HGNC Symbol;Acc:HGNC:19718] | 32273 | -0.094 | -0.3463 | Yes |
| 198 | SSTR3 | somatostatin receptor 3 [Source:HGNC Symbol;Acc:HGNC:11332] | 32366 | -0.095 | -0.3443 | Yes |
| 199 | GRM7 | glutamate metabotropic receptor 7 [Source:HGNC Symbol;Acc:HGNC:4599] | 32635 | -0.098 | -0.3466 | Yes |
| 200 | GABRR2 | gamma-aminobutyric acid type A receptor subunit rho2 [Source:HGNC Symbol;Acc:HGNC:4091] | 32665 | -0.099 | -0.3428 | Yes |
| 201 | CCKAR | cholecystokinin A receptor [Source:HGNC Symbol;Acc:HGNC:1570] | 32762 | -0.100 | -0.3407 | Yes |
| 202 | CHRM1 | cholinergic receptor muscarinic 1 [Source:HGNC Symbol;Acc:HGNC:1950] | 32818 | -0.101 | -0.3375 | Yes |
| 203 | GLRA2 | glycine receptor alpha 2 [Source:HGNC Symbol;Acc:HGNC:4327] | 32865 | -0.102 | -0.3340 | Yes |
| 204 | PTGFR | prostaglandin F receptor [Source:HGNC Symbol;Acc:HGNC:9600] | 32894 | -0.102 | -0.3301 | Yes |
| 205 | TACR2 | tachykinin receptor 2 [Source:HGNC Symbol;Acc:HGNC:11527] | 32918 | -0.102 | -0.3260 | Yes |
| 206 | PRSS3 | serine protease 3 [Source:HGNC Symbol;Acc:HGNC:9486] | 33123 | -0.105 | -0.3264 | Yes |
| 207 | CHRNB4 | cholinergic receptor nicotinic beta 4 subunit [Source:HGNC Symbol;Acc:HGNC:1964] | 33257 | -0.107 | -0.3249 | Yes |
| 208 | GHR | growth hormone receptor [Source:HGNC Symbol;Acc:HGNC:4263] | 33791 | -0.115 | -0.3333 | Yes |
| 209 | GPR83 | G protein-coupled receptor 83 [Source:HGNC Symbol;Acc:HGNC:4523] | 33860 | -0.116 | -0.3297 | Yes |
| 210 | ADCYAP1R1 | ADCYAP receptor type I [Source:HGNC Symbol;Acc:HGNC:242] | 33894 | -0.116 | -0.3252 | Yes |
| 211 | C5AR1 | complement C5a receptor 1 [Source:HGNC Symbol;Acc:HGNC:1338] | 34034 | -0.118 | -0.3234 | Yes |
| 212 | GABRR1 | gamma-aminobutyric acid type A receptor subunit rho1 [Source:HGNC Symbol;Acc:HGNC:4090] | 34069 | -0.119 | -0.3188 | Yes |
| 213 | HTR2A | 5-hydroxytryptamine receptor 2A [Source:HGNC Symbol;Acc:HGNC:5293] | 34078 | -0.119 | -0.3136 | Yes |
| 214 | ADRA1A | adrenoceptor alpha 1A [Source:HGNC Symbol;Acc:HGNC:277] | 34203 | -0.121 | -0.3112 | Yes |
| 215 | P2RY8 | P2Y receptor family member 8 [Source:HGNC Symbol;Acc:HGNC:15524] | 34268 | -0.122 | -0.3073 | Yes |
| 216 | GHSR | growth hormone secretagogue receptor [Source:HGNC Symbol;Acc:HGNC:4267] | 34326 | -0.123 | -0.3031 | Yes |
| 217 | PTGDR | prostaglandin D2 receptor [Source:HGNC Symbol;Acc:HGNC:9591] | 34389 | -0.124 | -0.2990 | Yes |
| 218 | CNR2 | cannabinoid receptor 2 [Source:HGNC Symbol;Acc:HGNC:2160] | 34392 | -0.124 | -0.2934 | Yes |
| 219 | GHRHR | growth hormone releasing hormone receptor [Source:HGNC Symbol;Acc:HGNC:4266] | 34444 | -0.125 | -0.2890 | Yes |
| 220 | FPR3 | formyl peptide receptor 3 [Source:HGNC Symbol;Acc:HGNC:3828] | 34491 | -0.126 | -0.2844 | Yes |
| 221 | PARD3 | par-3 family cell polarity regulator [Source:HGNC Symbol;Acc:HGNC:16051] | 34530 | -0.126 | -0.2796 | Yes |
| 222 | NPY2R | neuropeptide Y receptor Y2 [Source:HGNC Symbol;Acc:HGNC:7957] | 34605 | -0.128 | -0.2756 | Yes |
| 223 | CHRM5 | cholinergic receptor muscarinic 5 [Source:HGNC Symbol;Acc:HGNC:1954] | 34629 | -0.128 | -0.2704 | Yes |
| 224 | GRIK3 | glutamate ionotropic receptor kainate type subunit 3 [Source:HGNC Symbol;Acc:HGNC:4581] | 34690 | -0.129 | -0.2660 | Yes |
| 225 | S1PR5 | sphingosine-1-phosphate receptor 5 [Source:HGNC Symbol;Acc:HGNC:14299] | 34788 | -0.131 | -0.2625 | Yes |
| 226 | P2RY1 | purinergic receptor P2Y1 [Source:HGNC Symbol;Acc:HGNC:8539] | 34837 | -0.132 | -0.2576 | Yes |
| 227 | P2RY10 | P2Y receptor family member 10 [Source:HGNC Symbol;Acc:HGNC:19906] | 34907 | -0.133 | -0.2533 | Yes |
| 228 | NPY5R | neuropeptide Y receptor Y5 [Source:HGNC Symbol;Acc:HGNC:7958] | 34908 | -0.133 | -0.2472 | Yes |
| 229 | PTGER3 | prostaglandin E receptor 3 [Source:HGNC Symbol;Acc:HGNC:9595] | 34952 | -0.134 | -0.2421 | Yes |
| 230 | F2R | coagulation factor II thrombin receptor [Source:HGNC Symbol;Acc:HGNC:3537] | 35000 | -0.135 | -0.2371 | Yes |
| 231 | ADRA2A | adrenoceptor alpha 2A [Source:HGNC Symbol;Acc:HGNC:281] | 35010 | -0.136 | -0.2312 | Yes |
| 232 | CHRNA10 | cholinergic receptor nicotinic alpha 10 subunit [Source:HGNC Symbol;Acc:HGNC:13800] | 35022 | -0.136 | -0.2252 | Yes |
| 233 | PTH2R | parathyroid hormone 2 receptor [Source:HGNC Symbol;Acc:HGNC:9609] | 35042 | -0.136 | -0.2194 | Yes |
| 234 | HTR1B | 5-hydroxytryptamine receptor 1B [Source:HGNC Symbol;Acc:HGNC:5287] | 35113 | -0.138 | -0.2149 | Yes |
| 235 | GABRA2 | gamma-aminobutyric acid type A receptor subunit alpha2 [Source:HGNC Symbol;Acc:HGNC:4076] | 35116 | -0.138 | -0.2086 | Yes |
| 236 | GRIN3A | glutamate ionotropic receptor NMDA type subunit 3A [Source:HGNC Symbol;Acc:HGNC:16767] | 35122 | -0.138 | -0.2025 | Yes |
| 237 | CHRNB2 | cholinergic receptor nicotinic beta 2 subunit [Source:HGNC Symbol;Acc:HGNC:1962] | 35263 | -0.141 | -0.1996 | Yes |
| 238 | LTB4R | leukotriene B4 receptor [Source:HGNC Symbol;Acc:HGNC:6713] | 35304 | -0.142 | -0.1941 | Yes |
| 239 | CRHR2 | corticotropin releasing hormone receptor 2 [Source:HGNC Symbol;Acc:HGNC:2358] | 35515 | -0.147 | -0.1927 | Yes |
| 240 | APLNR | apelin receptor [Source:HGNC Symbol;Acc:HGNC:339] | 35558 | -0.148 | -0.1870 | Yes |
| 241 | CHRNA7 | cholinergic receptor nicotinic alpha 7 subunit [Source:HGNC Symbol;Acc:HGNC:1960] | 35561 | -0.148 | -0.1803 | Yes |
| 242 | GRM2 | glutamate metabotropic receptor 2 [Source:HGNC Symbol;Acc:HGNC:4594] | 35605 | -0.149 | -0.1745 | Yes |
| 243 | GABBR1 | gamma-aminobutyric acid type B receptor subunit 1 [Source:HGNC Symbol;Acc:HGNC:4070] | 35743 | -0.153 | -0.1710 | Yes |
| 244 | CHRM3 | cholinergic receptor muscarinic 3 [Source:HGNC Symbol;Acc:HGNC:1952] | 35766 | -0.154 | -0.1645 | Yes |
| 245 | MAS1 | "MAS1 proto-oncogene, G protein-coupled receptor [Source:HGNC Symbol;Acc:HGNC:6899]" | 35847 | -0.156 | -0.1594 | Yes |
| 246 | S1PR3 | sphingosine-1-phosphate receptor 3 [Source:HGNC Symbol;Acc:HGNC:3167] | 35885 | -0.157 | -0.1532 | Yes |
| 247 | LPAR4 | lysophosphatidic acid receptor 4 [Source:HGNC Symbol;Acc:HGNC:4478] | 35911 | -0.158 | -0.1466 | Yes |
| 248 | GRM8 | glutamate metabotropic receptor 8 [Source:HGNC Symbol;Acc:HGNC:4600] | 35943 | -0.159 | -0.1401 | Yes |
| 249 | P2RX7 | purinergic receptor P2X 7 [Source:HGNC Symbol;Acc:HGNC:8537] | 35972 | -0.159 | -0.1335 | Yes |
| 250 | GABRE | gamma-aminobutyric acid type A receptor subunit epsilon [Source:HGNC Symbol;Acc:HGNC:4085] | 36009 | -0.161 | -0.1271 | Yes |
| 251 | GLP1R | glucagon like peptide 1 receptor [Source:HGNC Symbol;Acc:HGNC:4324] | 36156 | -0.165 | -0.1233 | Yes |
| 252 | P2RX5 | purinergic receptor P2X 5 [Source:HGNC Symbol;Acc:HGNC:8536] | 36186 | -0.166 | -0.1164 | Yes |
| 253 | MC1R | melanocortin 1 receptor [Source:HGNC Symbol;Acc:HGNC:6929] | 36187 | -0.166 | -0.1088 | Yes |
| 254 | P2RY11 | purinergic receptor P2Y11 [Source:HGNC Symbol;Acc:HGNC:8540] | 36355 | -0.172 | -0.1052 | Yes |
| 255 | GRID1 | glutamate ionotropic receptor delta type subunit 1 [Source:HGNC Symbol;Acc:HGNC:4575] | 36359 | -0.172 | -0.0975 | Yes |
| 256 | NR3C1 | nuclear receptor subfamily 3 group C member 1 [Source:HGNC Symbol;Acc:HGNC:7978] | 36484 | -0.176 | -0.0926 | Yes |
| 257 | GRPR | gastrin releasing peptide receptor [Source:HGNC Symbol;Acc:HGNC:4609] | 36990 | -0.197 | -0.0965 | Yes |
| 258 | CALCRL | calcitonin receptor like receptor [Source:HGNC Symbol;Acc:HGNC:16709] | 37035 | -0.199 | -0.0885 | Yes |
| 259 | CRHR1 | corticotropin releasing hormone receptor 1 [Source:HGNC Symbol;Acc:HGNC:2357] | 37136 | -0.204 | -0.0817 | Yes |
| 260 | GABRA5 | gamma-aminobutyric acid type A receptor subunit alpha5 [Source:HGNC Symbol;Acc:HGNC:4079] | 37480 | -0.222 | -0.0803 | Yes |
| 261 | ADRA2B | adrenoceptor alpha 2B [Source:HGNC Symbol;Acc:HGNC:282] | 37489 | -0.223 | -0.0703 | Yes |
| 262 | AVPR1A | arginine vasopressin receptor 1A [Source:HGNC Symbol;Acc:HGNC:895] | 37496 | -0.223 | -0.0602 | Yes |
| 263 | F2RL1 | F2R like trypsin receptor 1 [Source:HGNC Symbol;Acc:HGNC:3538] | 37560 | -0.228 | -0.0514 | Yes |
| 264 | NMUR2 | neuromedin U receptor 2 [Source:HGNC Symbol;Acc:HGNC:16454] | 37835 | -0.247 | -0.0471 | Yes |
| 265 | GABRG3 | gamma-aminobutyric acid type A receptor subunit gamma3 [Source:HGNC Symbol;Acc:HGNC:4088] | 37878 | -0.252 | -0.0366 | Yes |
| 266 | CYSLTR2 | cysteinyl leukotriene receptor 2 [Source:HGNC Symbol;Acc:HGNC:18274] | 37909 | -0.255 | -0.0257 | Yes |
| 267 | GPR50 | G protein-coupled receptor 50 [Source:HGNC Symbol;Acc:HGNC:4506] | 38089 | -0.275 | -0.0177 | Yes |
| 268 | F2RL2 | coagulation factor II thrombin receptor like 2 [Source:HGNC Symbol;Acc:HGNC:3539] | 38386 | -0.327 | -0.0103 | Yes |
| 269 | GRM1 | glutamate metabotropic receptor 1 [Source:HGNC Symbol;Acc:HGNC:4593] | 38453 | -0.351 | 0.0041 | Yes |
| 270 | LPAR1 | lysophosphatidic acid receptor 1 [Source:HGNC Symbol;Acc:HGNC:3166] | 38527 | -0.397 | 0.0204 | Yes |
Table: GSEA details [plain text format]

  

Fig 2: KEGG\_NEUROACTIVE\_LIGAND\_RECEPTOR\_INTERACTION      
 Blue-Pink O' Gram in the Space of the Analyzed GeneSet

  

Fig 3: KEGG\_NEUROACTIVE\_LIGAND\_RECEPTOR\_INTERACTION: Random ES distribution      
 Gene set null distribution of ES for **KEGG\_NEUROACTIVE\_LIGAND\_RECEPTOR\_INTERACTION**
