# Supplementary material for: Simultaneous Presentation of Multiple Myeloma and Lung Cancer: Case Report and Gene Bioinformatics Analysis
Source: Front Oncol. 2022 Jun 13;12:859735. doi: 10.3389/fonc.2022.859735 (PMC9235397; doi:10.3389/fonc.2022.859735)
Supplement: Supplementary file 1 [file DataSheet_1.zip › The bioinformatic analysis of MM and lung cancer supplementary materials/Enrichment analysis/MECR/GSEA_4.1.0/LUSC TCGA/KEGG.Gsea.1639042374131/KEGG_OXIDATIVE_PHOSPHORYLATION.html]

Details for gene set KEGG\_OXIDATIVE\_PHOSPHORYLATION[GSEA]

|  || Dataset | ExpData\_collapsed\_to\_symbols.ENSG00000116353\_profile\_in\_ExpData.cls #ENSG00000116353 |
| Phenotype | ENSG00000116353\_profile\_in\_ExpData.cls#ENSG00000116353 |
| Upregulated in class | ENSG00000116353\_pos |
| GeneSet | KEGG\_OXIDATIVE\_PHOSPHORYLATION |
| Enrichment Score (ES) | 0.6491364 |
| Normalized Enrichment Score (NES) | 2.89299 |
| Nominal p-value | 0.0 |
| FDR q-value | 0.0 |
| FWER p-Value | 0.0 |
Table: GSEA Results Summary

  

Fig 1: Enrichment plot: KEGG\_OXIDATIVE\_PHOSPHORYLATION      
 Profile of the Running ES Score & Positions of GeneSet Members on the Rank Ordered List

  

| SYMBOL | TITLE | RANK IN GENE LIST | RANK METRIC SCORE | RUNNING ES | CORE ENRICHMENT || 1 | SDHB | succinate dehydrogenase complex iron sulfur subunit B [Source:HGNC Symbol;Acc:HGNC:10681] | 66 | 0.423 | 0.0225 | Yes |
| 2 | LHPP | phospholysine phosphohistidine inorganic pyrophosphate phosphatase [Source:HGNC Symbol;Acc:HGNC:30042] | 84 | 0.409 | 0.0454 | Yes |
| 3 | NDUFS5 | NADH:ubiquinone oxidoreductase subunit S5 [Source:HGNC Symbol;Acc:HGNC:7712] | 85 | 0.408 | 0.0686 | Yes |
| 4 | ATP6V0B | ATPase H+ transporting V0 subunit b [Source:HGNC Symbol;Acc:HGNC:861] | 140 | 0.368 | 0.0883 | Yes |
| 5 | NDUFA3 | NADH:ubiquinone oxidoreductase subunit A3 [Source:HGNC Symbol;Acc:HGNC:7686] | 141 | 0.368 | 0.1092 | Yes |
| 6 | UQCRH | ubiquinol-cytochrome c reductase hinge protein [Source:HGNC Symbol;Acc:HGNC:12590] | 167 | 0.353 | 0.1287 | Yes |
| 7 | NDUFS6 | NADH:ubiquinone oxidoreductase subunit S6 [Source:HGNC Symbol;Acc:HGNC:7713] | 194 | 0.343 | 0.1476 | Yes |
| 8 | ATP5PB | ATP synthase peripheral stalk-membrane subunit b [Source:HGNC Symbol;Acc:HGNC:840] | 213 | 0.335 | 0.1663 | Yes |
| 9 | UQCRHL | ubiquinol-cytochrome c reductase hinge protein like [Source:HGNC Symbol;Acc:HGNC:51714] | 377 | 0.290 | 0.1786 | Yes |
| 10 | SDHA | succinate dehydrogenase complex flavoprotein subunit A [Source:HGNC Symbol;Acc:HGNC:10680] | 452 | 0.277 | 0.1925 | Yes |
| 11 | NDUFS8 | NADH:ubiquinone oxidoreductase core subunit S8 [Source:HGNC Symbol;Acc:HGNC:7715] | 512 | 0.268 | 0.2064 | Yes |
| 12 | ATP5PO | ATP synthase peripheral stalk subunit OSCP [Source:HGNC Symbol;Acc:HGNC:850] | 517 | 0.268 | 0.2215 | Yes |
| 13 | NDUFV1 | NADH:ubiquinone oxidoreductase core subunit V1 [Source:HGNC Symbol;Acc:HGNC:7716] | 525 | 0.266 | 0.2366 | Yes |
| 14 | PPA2 | inorganic pyrophosphatase 2 [Source:HGNC Symbol;Acc:HGNC:28883] | 540 | 0.264 | 0.2512 | Yes |
| 15 | CYC1 | cytochrome c1 [Source:HGNC Symbol;Acc:HGNC:2579] | 644 | 0.252 | 0.2630 | Yes |
| 16 | ATP5F1E | ATP synthase F1 subunit epsilon [Source:HGNC Symbol;Acc:HGNC:838] | 731 | 0.241 | 0.2745 | Yes |
| 17 | ATP5ME | ATP synthase membrane subunit e [Source:HGNC Symbol;Acc:HGNC:846] | 844 | 0.227 | 0.2846 | Yes |
| 18 | ATP6V0D1 | ATPase H+ transporting V0 subunit d1 [Source:HGNC Symbol;Acc:HGNC:13724] | 858 | 0.227 | 0.2972 | Yes |
| 19 | ATP5MF | ATP synthase membrane subunit f [Source:HGNC Symbol;Acc:HGNC:848] | 890 | 0.223 | 0.3091 | Yes |
| 20 | UQCR10 | "ubiquinol-cytochrome c reductase, complex III subunit X [Source:HGNC Symbol;Acc:HGNC:30863]" | 976 | 0.217 | 0.3193 | Yes |
| 21 | NDUFA2 | NADH:ubiquinone oxidoreductase subunit A2 [Source:HGNC Symbol;Acc:HGNC:7685] | 1078 | 0.210 | 0.3287 | Yes |
| 22 | ATP5F1C | ATP synthase F1 subunit gamma [Source:HGNC Symbol;Acc:HGNC:833] | 1084 | 0.210 | 0.3406 | Yes |
| 23 | UQCRQ | ubiquinol-cytochrome c reductase complex III subunit VII [Source:HGNC Symbol;Acc:HGNC:29594] | 1244 | 0.200 | 0.3480 | Yes |
| 24 | NDUFC1 | NADH:ubiquinone oxidoreductase subunit C1 [Source:HGNC Symbol;Acc:HGNC:7705] | 1333 | 0.195 | 0.3569 | Yes |
| 25 | NDUFA10 | NADH:ubiquinone oxidoreductase subunit A10 [Source:HGNC Symbol;Acc:HGNC:7684] | 1484 | 0.187 | 0.3637 | Yes |
| 26 | COX6B2 | cytochrome c oxidase subunit 6B2 [Source:HGNC Symbol;Acc:HGNC:24380] | 1508 | 0.186 | 0.3737 | Yes |
| 27 | NDUFB8 | NADH:ubiquinone oxidoreductase subunit B8 [Source:HGNC Symbol;Acc:HGNC:7703] | 1568 | 0.183 | 0.3827 | Yes |
| 28 | ATP5MC2 | ATP synthase membrane subunit c locus 2 [Source:HGNC Symbol;Acc:HGNC:842] | 1577 | 0.183 | 0.3929 | Yes |
| 29 | UQCRB | ubiquinol-cytochrome c reductase binding protein [Source:HGNC Symbol;Acc:HGNC:12582] | 1639 | 0.181 | 0.4017 | Yes |
| 30 | COX8A | cytochrome c oxidase subunit 8A [Source:HGNC Symbol;Acc:HGNC:2294] | 1644 | 0.181 | 0.4119 | Yes |
| 31 | NDUFB3 | NADH:ubiquinone oxidoreductase subunit B3 [Source:HGNC Symbol;Acc:HGNC:7698] | 1692 | 0.178 | 0.4208 | Yes |
| 32 | UQCRC1 | ubiquinol-cytochrome c reductase core protein 1 [Source:HGNC Symbol;Acc:HGNC:12585] | 1764 | 0.175 | 0.4290 | Yes |
| 33 | ATP6V1D | ATPase H+ transporting V1 subunit D [Source:HGNC Symbol;Acc:HGNC:13527] | 1768 | 0.174 | 0.4389 | Yes |
| 34 | ATP5MG | ATP synthase membrane subunit g [Source:HGNC Symbol;Acc:HGNC:14247] | 1859 | 0.171 | 0.4463 | Yes |
| 35 | NDUFB4 | NADH:ubiquinone oxidoreductase subunit B4 [Source:HGNC Symbol;Acc:HGNC:7699] | 1870 | 0.171 | 0.4558 | Yes |
| 36 | NDUFB9 | NADH:ubiquinone oxidoreductase subunit B9 [Source:HGNC Symbol;Acc:HGNC:7704] | 1879 | 0.171 | 0.4654 | Yes |
| 37 | NDUFB1 | NADH:ubiquinone oxidoreductase subunit B1 [Source:HGNC Symbol;Acc:HGNC:7695] | 1941 | 0.168 | 0.4734 | Yes |
| 38 | NDUFAB1 | NADH:ubiquinone oxidoreductase subunit AB1 [Source:HGNC Symbol;Acc:HGNC:7694] | 2058 | 0.164 | 0.4798 | Yes |
| 39 | NDUFA11 | NADH:ubiquinone oxidoreductase subunit A11 [Source:HGNC Symbol;Acc:HGNC:20371] | 2565 | 0.148 | 0.4753 | Yes |
| 40 | NDUFB10 | NADH:ubiquinone oxidoreductase subunit B10 [Source:HGNC Symbol;Acc:HGNC:7696] | 2658 | 0.145 | 0.4812 | Yes |
| 41 | MT-CO1 | mitochondrially encoded cytochrome c oxidase I [Source:HGNC Symbol;Acc:HGNC:7419] | 2684 | 0.144 | 0.4888 | Yes |
| 42 | NDUFA9 | NADH:ubiquinone oxidoreductase subunit A9 [Source:HGNC Symbol;Acc:HGNC:7693] | 2762 | 0.142 | 0.4950 | Yes |
| 43 | MT-ND6 | mitochondrially encoded NADH:ubiquinone oxidoreductase core subunit 6 [Source:HGNC Symbol;Acc:HGNC:7462] | 2862 | 0.140 | 0.5004 | Yes |
| 44 | NDUFS3 | NADH:ubiquinone oxidoreductase core subunit S3 [Source:HGNC Symbol;Acc:HGNC:7710] | 2883 | 0.140 | 0.5079 | Yes |
| 45 | ATP5PF | ATP synthase peripheral stalk subunit F6 [Source:HGNC Symbol;Acc:HGNC:847] | 2949 | 0.138 | 0.5141 | Yes |
| 46 | COX17 | cytochrome c oxidase copper chaperone COX17 [Source:HGNC Symbol;Acc:HGNC:2264] | 3006 | 0.136 | 0.5205 | Yes |
| 47 | COX4I1 | cytochrome c oxidase subunit 4I1 [Source:HGNC Symbol;Acc:HGNC:2265] | 3073 | 0.135 | 0.5265 | Yes |
| 48 | NDUFS7 | NADH:ubiquinone oxidoreductase core subunit S7 [Source:HGNC Symbol;Acc:HGNC:7714] | 3210 | 0.132 | 0.5306 | Yes |
| 49 | COX7C | cytochrome c oxidase subunit 7C [Source:HGNC Symbol;Acc:HGNC:2292] | 3427 | 0.128 | 0.5323 | Yes |
| 50 | COX7A2 | cytochrome c oxidase subunit 7A2 [Source:HGNC Symbol;Acc:HGNC:2288] | 3468 | 0.127 | 0.5385 | Yes |
| 51 | NDUFB2 | NADH:ubiquinone oxidoreductase subunit B2 [Source:HGNC Symbol;Acc:HGNC:7697] | 3507 | 0.126 | 0.5448 | Yes |
| 52 | NDUFV2 | NADH:ubiquinone oxidoreductase core subunit V2 [Source:HGNC Symbol;Acc:HGNC:7717] | 3550 | 0.125 | 0.5509 | Yes |
| 53 | COX6B1 | cytochrome c oxidase subunit 6B1 [Source:HGNC Symbol;Acc:HGNC:2280] | 3572 | 0.125 | 0.5575 | Yes |
| 54 | MT-ND1 | mitochondrially encoded NADH:ubiquinone oxidoreductase core subunit 1 [Source:HGNC Symbol;Acc:HGNC:7455] | 3588 | 0.125 | 0.5642 | Yes |
| 55 | NDUFB7 | NADH:ubiquinone oxidoreductase subunit B7 [Source:HGNC Symbol;Acc:HGNC:7702] | 3667 | 0.123 | 0.5692 | Yes |
| 56 | PPA1 | inorganic pyrophosphatase 1 [Source:HGNC Symbol;Acc:HGNC:9226] | 3716 | 0.123 | 0.5750 | Yes |
| 57 | NDUFC2 | NADH:ubiquinone oxidoreductase subunit C2 [Source:HGNC Symbol;Acc:HGNC:7706] | 3963 | 0.118 | 0.5755 | Yes |
| 58 | NDUFS4 | NADH:ubiquinone oxidoreductase subunit S4 [Source:HGNC Symbol;Acc:HGNC:7711] | 4123 | 0.116 | 0.5780 | Yes |
| 59 | COX4I2 | cytochrome c oxidase subunit 4I2 [Source:HGNC Symbol;Acc:HGNC:16232] | 4182 | 0.115 | 0.5831 | Yes |
| 60 | ATP5PD | ATP synthase peripheral stalk subunit d [Source:HGNC Symbol;Acc:HGNC:845] | 4285 | 0.113 | 0.5869 | Yes |
| 61 | SDHC | succinate dehydrogenase complex subunit C [Source:HGNC Symbol;Acc:HGNC:10682] | 4323 | 0.112 | 0.5924 | Yes |
| 62 | COX6C | cytochrome c oxidase subunit 6C [Source:HGNC Symbol;Acc:HGNC:2285] | 4326 | 0.112 | 0.5988 | Yes |
| 63 | ATP6V1C1 | ATPase H+ transporting V1 subunit C1 [Source:HGNC Symbol;Acc:HGNC:856] | 4461 | 0.110 | 0.6017 | Yes |
| 64 | COX15 | cytochrome c oxidase assembly homolog COX15 [Source:HGNC Symbol;Acc:HGNC:2263] | 4553 | 0.109 | 0.6055 | Yes |
| 65 | MT-CO3 | mitochondrially encoded cytochrome c oxidase III [Source:HGNC Symbol;Acc:HGNC:7422] | 4561 | 0.109 | 0.6116 | Yes |
| 66 | ATP6V0C | ATPase H+ transporting V0 subunit c [Source:HGNC Symbol;Acc:HGNC:855] | 4694 | 0.107 | 0.6143 | Yes |
| 67 | COX7A2L | cytochrome c oxidase subunit 7A2 like [Source:HGNC Symbol;Acc:HGNC:2289] | 4754 | 0.106 | 0.6189 | Yes |
| 68 | COX7B | cytochrome c oxidase subunit 7B [Source:HGNC Symbol;Acc:HGNC:2291] | 4828 | 0.105 | 0.6230 | Yes |
| 69 | COX6CP3 | cytochrome c oxidase subunit 6C pseudogene 3 [Source:HGNC Symbol;Acc:HGNC:31721] | 4943 | 0.104 | 0.6260 | Yes |
| 70 | MT-ND5 | mitochondrially encoded NADH:ubiquinone oxidoreductase core subunit 5 [Source:HGNC Symbol;Acc:HGNC:7461] | 5023 | 0.102 | 0.6298 | Yes |
| 71 | COX6A1 | cytochrome c oxidase subunit 6A1 [Source:HGNC Symbol;Acc:HGNC:2277] | 5113 | 0.101 | 0.6333 | Yes |
| 72 | NDUFA4 | NDUFA4 mitochondrial complex associated [Source:HGNC Symbol;Acc:HGNC:7687] | 5480 | 0.097 | 0.6295 | Yes |
| 73 | UQCRC2 | ubiquinol-cytochrome c reductase core protein 2 [Source:HGNC Symbol;Acc:HGNC:12586] | 5923 | 0.095 | 0.6237 | Yes |
| 74 | ATP5MC1 | ATP synthase membrane subunit c locus 1 [Source:HGNC Symbol;Acc:HGNC:841] | 5965 | 0.094 | 0.6280 | Yes |
| 75 | ATP6V1H | ATPase H+ transporting V1 subunit H [Source:HGNC Symbol;Acc:HGNC:18303] | 5968 | 0.094 | 0.6333 | Yes |
| 76 | NDUFV3 | NADH:ubiquinone oxidoreductase subunit V3 [Source:HGNC Symbol;Acc:HGNC:7719] | 6013 | 0.094 | 0.6376 | Yes |
| 77 | NDUFA1 | NADH:ubiquinone oxidoreductase subunit A1 [Source:HGNC Symbol;Acc:HGNC:7683] | 6036 | 0.094 | 0.6423 | Yes |
| 78 | COX6A2 | cytochrome c oxidase subunit 6A2 [Source:HGNC Symbol;Acc:HGNC:2279] | 6345 | 0.090 | 0.6396 | Yes |
| 79 | ATP6AP1 | ATPase H+ transporting accessory protein 1 [Source:HGNC Symbol;Acc:HGNC:868] | 6555 | 0.088 | 0.6393 | Yes |
| 80 | NDUFA6 | NADH:ubiquinone oxidoreductase subunit A6 [Source:HGNC Symbol;Acc:HGNC:7690] | 6634 | 0.087 | 0.6423 | Yes |
| 81 | ATP6V1F | ATPase H+ transporting V1 subunit F [Source:HGNC Symbol;Acc:HGNC:16832] | 6743 | 0.086 | 0.6445 | Yes |
| 82 | COX7B2 | cytochrome c oxidase subunit 7B2 [Source:HGNC Symbol;Acc:HGNC:24381] | 6753 | 0.086 | 0.6491 | Yes |
| 83 | MT-CO2 | mitochondrially encoded cytochrome c oxidase II [Source:HGNC Symbol;Acc:HGNC:7421] | 7144 | 0.082 | 0.6439 | No |
| 84 | NDUFA5 | NADH:ubiquinone oxidoreductase subunit A5 [Source:HGNC Symbol;Acc:HGNC:7688] | 7600 | 0.078 | 0.6367 | No |
| 85 | ATP5F1D | ATP synthase F1 subunit delta [Source:HGNC Symbol;Acc:HGNC:837] | 7651 | 0.078 | 0.6399 | No |
| 86 | ATP5F1A | ATP synthase F1 subunit alpha [Source:HGNC Symbol;Acc:HGNC:823] | 7734 | 0.077 | 0.6422 | No |
| 87 | ATP6V1E1 | ATPase H+ transporting V1 subunit E1 [Source:HGNC Symbol;Acc:HGNC:857] | 8003 | 0.075 | 0.6397 | No |
| 88 | MT-ND3 | mitochondrially encoded NADH:ubiquinone oxidoreductase core subunit 3 [Source:HGNC Symbol;Acc:HGNC:7458] | 8252 | 0.073 | 0.6375 | No |
| 89 | COX5B | cytochrome c oxidase subunit 5B [Source:HGNC Symbol;Acc:HGNC:2269] | 8271 | 0.072 | 0.6411 | No |
| 90 | TCIRG1 | "T cell immune regulator 1, ATPase H+ transporting V0 subunit a3 [Source:HGNC Symbol;Acc:HGNC:11647]" | 9630 | 0.063 | 0.6101 | No |
| 91 | MT-ND4 | mitochondrially encoded NADH:ubiquinone oxidoreductase core subunit 4 [Source:HGNC Symbol;Acc:HGNC:7459] | 9732 | 0.062 | 0.6111 | No |
| 92 | MT-CYB | mitochondrially encoded cytochrome b [Source:HGNC Symbol;Acc:HGNC:7427] | 9843 | 0.061 | 0.6118 | No |
| 93 | UQCR11 | "ubiquinol-cytochrome c reductase, complex III subunit XI [Source:HGNC Symbol;Acc:HGNC:30862]" | 9868 | 0.061 | 0.6146 | No |
| 94 | UQCRFS1 | "ubiquinol-cytochrome c reductase, Rieske iron-sulfur polypeptide 1 [Source:HGNC Symbol;Acc:HGNC:12587]" | 10480 | 0.057 | 0.6023 | No |
| 95 | ATP6V0E2 | ATPase H+ transporting V0 subunit e2 [Source:HGNC Symbol;Acc:HGNC:21723] | 11007 | 0.053 | 0.5919 | No |
| 96 | ATP6V1G2 | ATPase H+ transporting V1 subunit G2 [Source:HGNC Symbol;Acc:HGNC:862] | 11269 | 0.052 | 0.5882 | No |
| 97 | COX8C | cytochrome c oxidase subunit 8C [Source:HGNC Symbol;Acc:HGNC:24382] | 11354 | 0.051 | 0.5890 | No |
| 98 | MT-ATP6 | mitochondrially encoded ATP synthase membrane subunit 6 [Source:HGNC Symbol;Acc:HGNC:7414] | 11361 | 0.051 | 0.5918 | No |
| 99 | NDUFA7 | NADH:ubiquinone oxidoreductase subunit A7 [Source:HGNC Symbol;Acc:HGNC:7691] | 11506 | 0.050 | 0.5910 | No |
| 100 | ATP4B | ATPase H+/K+ transporting subunit beta [Source:HGNC Symbol;Acc:HGNC:820] | 11516 | 0.050 | 0.5936 | No |
| 101 | MT-ND2 | mitochondrially encoded NADH:ubiquinone oxidoreductase core subunit 2 [Source:HGNC Symbol;Acc:HGNC:7456] | 11807 | 0.048 | 0.5890 | No |
| 102 | NDUFS2 | NADH:ubiquinone oxidoreductase core subunit S2 [Source:HGNC Symbol;Acc:HGNC:7708] | 13107 | 0.040 | 0.5581 | No |
| 103 | ATP6V1C2 | ATPase H+ transporting V1 subunit C2 [Source:HGNC Symbol;Acc:HGNC:18264] | 13152 | 0.040 | 0.5593 | No |
| 104 | ATP6V1B1 | ATPase H+ transporting V1 subunit B1 [Source:HGNC Symbol;Acc:HGNC:853] | 13460 | 0.038 | 0.5536 | No |
| 105 | MT-ND4L | mitochondrially encoded NADH:ubiquinone oxidoreductase core subunit 4L [Source:HGNC Symbol;Acc:HGNC:7460] | 13712 | 0.037 | 0.5493 | No |
| 106 | ATP5F1B | ATP synthase F1 subunit beta [Source:HGNC Symbol;Acc:HGNC:830] | 14082 | 0.035 | 0.5419 | No |
| 107 | ATP6V1A | ATPase H+ transporting V1 subunit A [Source:HGNC Symbol;Acc:HGNC:851] | 15747 | 0.025 | 0.5008 | No |
| 108 | NDUFB6 | NADH:ubiquinone oxidoreductase subunit B6 [Source:HGNC Symbol;Acc:HGNC:7701] | 15775 | 0.025 | 0.5015 | No |
| 109 | MT-ATP8 | mitochondrially encoded ATP synthase membrane subunit 8 [Source:HGNC Symbol;Acc:HGNC:7415] | 16163 | 0.022 | 0.4929 | No |
| 110 | COX7A1 | cytochrome c oxidase subunit 7A1 [Source:HGNC Symbol;Acc:HGNC:2287] | 16527 | 0.020 | 0.4848 | No |
| 111 | NDUFA4L2 | NDUFA4 mitochondrial complex associated like 2 [Source:HGNC Symbol;Acc:HGNC:29836] | 16755 | 0.019 | 0.4801 | No |
| 112 | ATP5MC3 | ATP synthase membrane subunit c locus 3 [Source:HGNC Symbol;Acc:HGNC:843] | 16867 | 0.018 | 0.4783 | No |
| 113 | NDUFS1 | NADH:ubiquinone oxidoreductase core subunit S1 [Source:HGNC Symbol;Acc:HGNC:7707] | 17437 | 0.015 | 0.4647 | No |
| 114 | ATP6V0A4 | ATPase H+ transporting V0 subunit a4 [Source:HGNC Symbol;Acc:HGNC:866] | 18458 | 0.010 | 0.4392 | No |
| 115 | ATP6V0E1 | ATPase H+ transporting V0 subunit e1 [Source:HGNC Symbol;Acc:HGNC:863] | 18763 | 0.008 | 0.4319 | No |
| 116 | ATP5MC1P5 | ATP synthase membrane subunit c locus 1 pseudogene 5 [Source:HGNC Symbol;Acc:HGNC:39508] | 19858 | 0.002 | 0.4041 | No |
| 117 | ATP6V1B2 | ATPase H+ transporting V1 subunit B2 [Source:HGNC Symbol;Acc:HGNC:854] | 20048 | 0.000 | 0.3993 | No |
| 118 | COX5A | cytochrome c oxidase subunit 5A [Source:HGNC Symbol;Acc:HGNC:2267] | 22458 | -0.014 | 0.3386 | No |
| 119 | COX10 | cytochrome c oxidase assembly factor heme A:farnesyltransferase COX10 [Source:HGNC Symbol;Acc:HGNC:2260] | 22985 | -0.017 | 0.3262 | No |
| 120 | SDHD | succinate dehydrogenase complex subunit D [Source:HGNC Symbol;Acc:HGNC:10683] | 22998 | -0.017 | 0.3269 | No |
| 121 | UQCR10P1 | UQCR10 pseudogene 1 [Source:HGNC Symbol;Acc:HGNC:54960] | 25693 | -0.036 | 0.2602 | No |
| 122 | ATP6V1G1 | ATPase H+ transporting V1 subunit G1 [Source:HGNC Symbol;Acc:HGNC:864] | 27797 | -0.052 | 0.2095 | No |
| 123 | ATP6V1G3 | ATPase H+ transporting V1 subunit G3 [Source:HGNC Symbol;Acc:HGNC:18265] | 28852 | -0.060 | 0.1860 | No |
| 124 | ATP6V0D2 | ATPase H+ transporting V0 subunit d2 [Source:HGNC Symbol;Acc:HGNC:18266] | 29413 | -0.065 | 0.1755 | No |
| 125 | NDUFB5 | NADH:ubiquinone oxidoreductase subunit B5 [Source:HGNC Symbol;Acc:HGNC:7700] | 29913 | -0.070 | 0.1667 | No |
| 126 | NDUFA8 | NADH:ubiquinone oxidoreductase subunit A8 [Source:HGNC Symbol;Acc:HGNC:7692] | 31722 | -0.088 | 0.1256 | No |
| 127 | ATP6V1E2 | ATPase H+ transporting V1 subunit E2 [Source:HGNC Symbol;Acc:HGNC:18125] | 33852 | -0.116 | 0.0779 | No |
| 128 | COX11 | cytochrome c oxidase copper chaperone COX11 [Source:HGNC Symbol;Acc:HGNC:2261] | 35029 | -0.136 | 0.0556 | No |
| 129 | ATP6V0A2 | ATPase H+ transporting V0 subunit a2 [Source:HGNC Symbol;Acc:HGNC:18481] | 37060 | -0.200 | 0.0152 | No |
| 130 | ATP6V0A1 | ATPase H+ transporting V0 subunit a1 [Source:HGNC Symbol;Acc:HGNC:865] | 37577 | -0.230 | 0.0152 | No |
| 131 | ATP12A | ATPase H+/K+ transporting non-gastric alpha2 subunit [Source:HGNC Symbol;Acc:HGNC:13816] | 37845 | -0.248 | 0.0225 | No |
| 132 | ATP4A | ATPase H+/K+ transporting subunit alpha [Source:HGNC Symbol;Acc:HGNC:819] | 37998 | -0.265 | 0.0338 | No |
Table: GSEA details [plain text format]

  

Fig 2: KEGG\_OXIDATIVE\_PHOSPHORYLATION      
 Blue-Pink O' Gram in the Space of the Analyzed GeneSet

  

Fig 3: KEGG\_OXIDATIVE\_PHOSPHORYLATION: Random ES distribution      
 Gene set null distribution of ES for **KEGG\_OXIDATIVE\_PHOSPHORYLATION**
