# Supplementary material for: Simultaneous Presentation of Multiple Myeloma and Lung Cancer: Case Report and Gene Bioinformatics Analysis
Source: Front Oncol. 2022 Jun 13;12:859735. doi: 10.3389/fonc.2022.859735 (PMC9235397; doi:10.3389/fonc.2022.859735)
Supplement: Supplementary file 1 [file DataSheet_1.zip › The bioinformatic analysis of MM and lung cancer supplementary materials/Enrichment analysis/MECR/GSEA_4.1.0/LUSC TCGA/KEGG.Gsea.1639042374131/KEGG_PARKINSONS_DISEASE.html]

Details for gene set KEGG\_PARKINSONS\_DISEASE[GSEA]

|  || Dataset | ExpData\_collapsed\_to\_symbols.ENSG00000116353\_profile\_in\_ExpData.cls #ENSG00000116353 |
| Phenotype | ENSG00000116353\_profile\_in\_ExpData.cls#ENSG00000116353 |
| Upregulated in class | ENSG00000116353\_pos |
| GeneSet | KEGG\_PARKINSONS\_DISEASE |
| Enrichment Score (ES) | 0.6456266 |
| Normalized Enrichment Score (NES) | 2.8768263 |
| Nominal p-value | 0.0 |
| FDR q-value | 0.0 |
| FWER p-Value | 0.0 |
Table: GSEA Results Summary

  

Fig 1: Enrichment plot: KEGG\_PARKINSONS\_DISEASE      
 Profile of the Running ES Score & Positions of GeneSet Members on the Rank Ordered List

  

| SYMBOL | TITLE | RANK IN GENE LIST | RANK METRIC SCORE | RUNNING ES | CORE ENRICHMENT || 1 | UBE2J2 | ubiquitin conjugating enzyme E2 J2 [Source:HGNC Symbol;Acc:HGNC:19268] | 31 | 0.477 | 0.0279 | Yes |
| 2 | PARK7 | Parkinsonism associated deglycase [Source:HGNC Symbol;Acc:HGNC:16369] | 59 | 0.438 | 0.0536 | Yes |
| 3 | SDHB | succinate dehydrogenase complex iron sulfur subunit B [Source:HGNC Symbol;Acc:HGNC:10681] | 66 | 0.423 | 0.0789 | Yes |
| 4 | NDUFS5 | NADH:ubiquinone oxidoreductase subunit S5 [Source:HGNC Symbol;Acc:HGNC:7712] | 85 | 0.408 | 0.1030 | Yes |
| 5 | NDUFA3 | NADH:ubiquinone oxidoreductase subunit A3 [Source:HGNC Symbol;Acc:HGNC:7686] | 141 | 0.368 | 0.1237 | Yes |
| 6 | UQCRH | ubiquinol-cytochrome c reductase hinge protein [Source:HGNC Symbol;Acc:HGNC:12590] | 167 | 0.353 | 0.1444 | Yes |
| 7 | NDUFS6 | NADH:ubiquinone oxidoreductase subunit S6 [Source:HGNC Symbol;Acc:HGNC:7713] | 194 | 0.343 | 0.1643 | Yes |
| 8 | ATP5PB | ATP synthase peripheral stalk-membrane subunit b [Source:HGNC Symbol;Acc:HGNC:840] | 213 | 0.335 | 0.1840 | Yes |
| 9 | CASP9 | caspase 9 [Source:HGNC Symbol;Acc:HGNC:1511] | 300 | 0.306 | 0.2002 | Yes |
| 10 | UQCRHL | ubiquinol-cytochrome c reductase hinge protein like [Source:HGNC Symbol;Acc:HGNC:51714] | 377 | 0.290 | 0.2158 | Yes |
| 11 | SDHA | succinate dehydrogenase complex flavoprotein subunit A [Source:HGNC Symbol;Acc:HGNC:10680] | 452 | 0.277 | 0.2305 | Yes |
| 12 | NDUFS8 | NADH:ubiquinone oxidoreductase core subunit S8 [Source:HGNC Symbol;Acc:HGNC:7715] | 512 | 0.268 | 0.2452 | Yes |
| 13 | ATP5PO | ATP synthase peripheral stalk subunit OSCP [Source:HGNC Symbol;Acc:HGNC:850] | 517 | 0.268 | 0.2612 | Yes |
| 14 | NDUFV1 | NADH:ubiquinone oxidoreductase core subunit V1 [Source:HGNC Symbol;Acc:HGNC:7716] | 525 | 0.266 | 0.2771 | Yes |
| 15 | CYC1 | cytochrome c1 [Source:HGNC Symbol;Acc:HGNC:2579] | 644 | 0.252 | 0.2892 | Yes |
| 16 | ATP5F1E | ATP synthase F1 subunit epsilon [Source:HGNC Symbol;Acc:HGNC:838] | 731 | 0.241 | 0.3015 | Yes |
| 17 | UQCR10 | "ubiquinol-cytochrome c reductase, complex III subunit X [Source:HGNC Symbol;Acc:HGNC:30863]" | 976 | 0.217 | 0.3083 | Yes |
| 18 | NDUFA2 | NADH:ubiquinone oxidoreductase subunit A2 [Source:HGNC Symbol;Acc:HGNC:7685] | 1078 | 0.210 | 0.3183 | Yes |
| 19 | ATP5F1C | ATP synthase F1 subunit gamma [Source:HGNC Symbol;Acc:HGNC:833] | 1084 | 0.210 | 0.3308 | Yes |
| 20 | PINK1 | PTEN induced kinase 1 [Source:HGNC Symbol;Acc:HGNC:14581] | 1185 | 0.204 | 0.3406 | Yes |
| 21 | UQCRQ | ubiquinol-cytochrome c reductase complex III subunit VII [Source:HGNC Symbol;Acc:HGNC:29594] | 1244 | 0.200 | 0.3511 | Yes |
| 22 | GPR37 | G protein-coupled receptor 37 [Source:HGNC Symbol;Acc:HGNC:4494] | 1282 | 0.198 | 0.3621 | Yes |
| 23 | NDUFC1 | NADH:ubiquinone oxidoreductase subunit C1 [Source:HGNC Symbol;Acc:HGNC:7705] | 1333 | 0.195 | 0.3726 | Yes |
| 24 | NDUFA10 | NADH:ubiquinone oxidoreductase subunit A10 [Source:HGNC Symbol;Acc:HGNC:7684] | 1484 | 0.187 | 0.3800 | Yes |
| 25 | COX6B2 | cytochrome c oxidase subunit 6B2 [Source:HGNC Symbol;Acc:HGNC:24380] | 1508 | 0.186 | 0.3907 | Yes |
| 26 | NDUFB8 | NADH:ubiquinone oxidoreductase subunit B8 [Source:HGNC Symbol;Acc:HGNC:7703] | 1568 | 0.183 | 0.4002 | Yes |
| 27 | ATP5MC2 | ATP synthase membrane subunit c locus 2 [Source:HGNC Symbol;Acc:HGNC:842] | 1577 | 0.183 | 0.4110 | Yes |
| 28 | UQCRB | ubiquinol-cytochrome c reductase binding protein [Source:HGNC Symbol;Acc:HGNC:12582] | 1639 | 0.181 | 0.4203 | Yes |
| 29 | COX8A | cytochrome c oxidase subunit 8A [Source:HGNC Symbol;Acc:HGNC:2294] | 1644 | 0.181 | 0.4311 | Yes |
| 30 | NDUFB3 | NADH:ubiquinone oxidoreductase subunit B3 [Source:HGNC Symbol;Acc:HGNC:7698] | 1692 | 0.178 | 0.4406 | Yes |
| 31 | UQCRC1 | ubiquinol-cytochrome c reductase core protein 1 [Source:HGNC Symbol;Acc:HGNC:12585] | 1764 | 0.175 | 0.4493 | Yes |
| 32 | NDUFB4 | NADH:ubiquinone oxidoreductase subunit B4 [Source:HGNC Symbol;Acc:HGNC:7699] | 1870 | 0.171 | 0.4569 | Yes |
| 33 | NDUFB9 | NADH:ubiquinone oxidoreductase subunit B9 [Source:HGNC Symbol;Acc:HGNC:7704] | 1879 | 0.171 | 0.4670 | Yes |
| 34 | NDUFB1 | NADH:ubiquinone oxidoreductase subunit B1 [Source:HGNC Symbol;Acc:HGNC:7695] | 1941 | 0.168 | 0.4755 | Yes |
| 35 | NDUFAB1 | NADH:ubiquinone oxidoreductase subunit AB1 [Source:HGNC Symbol;Acc:HGNC:7694] | 2058 | 0.164 | 0.4824 | Yes |
| 36 | HTRA2 | HtrA serine peptidase 2 [Source:HGNC Symbol;Acc:HGNC:14348] | 2250 | 0.157 | 0.4870 | Yes |
| 37 | UBB | ubiquitin B [Source:HGNC Symbol;Acc:HGNC:12463] | 2566 | 0.148 | 0.4879 | Yes |
| 38 | NDUFB10 | NADH:ubiquinone oxidoreductase subunit B10 [Source:HGNC Symbol;Acc:HGNC:7696] | 2658 | 0.145 | 0.4943 | Yes |
| 39 | MT-CO1 | mitochondrially encoded cytochrome c oxidase I [Source:HGNC Symbol;Acc:HGNC:7419] | 2684 | 0.144 | 0.5023 | Yes |
| 40 | NDUFA9 | NADH:ubiquinone oxidoreductase subunit A9 [Source:HGNC Symbol;Acc:HGNC:7693] | 2762 | 0.142 | 0.5089 | Yes |
| 41 | MT-ND6 | mitochondrially encoded NADH:ubiquinone oxidoreductase core subunit 6 [Source:HGNC Symbol;Acc:HGNC:7462] | 2862 | 0.140 | 0.5148 | Yes |
| 42 | NDUFS3 | NADH:ubiquinone oxidoreductase core subunit S3 [Source:HGNC Symbol;Acc:HGNC:7710] | 2883 | 0.140 | 0.5227 | Yes |
| 43 | SLC25A6 | solute carrier family 25 member 6 [Source:HGNC Symbol;Acc:HGNC:10992] | 2903 | 0.139 | 0.5306 | Yes |
| 44 | ATP5PF | ATP synthase peripheral stalk subunit F6 [Source:HGNC Symbol;Acc:HGNC:847] | 2949 | 0.138 | 0.5378 | Yes |
| 45 | COX4I1 | cytochrome c oxidase subunit 4I1 [Source:HGNC Symbol;Acc:HGNC:2265] | 3073 | 0.135 | 0.5428 | Yes |
| 46 | NDUFS7 | NADH:ubiquinone oxidoreductase core subunit S7 [Source:HGNC Symbol;Acc:HGNC:7714] | 3210 | 0.132 | 0.5473 | Yes |
| 47 | TH | tyrosine hydroxylase [Source:HGNC Symbol;Acc:HGNC:11782] | 3306 | 0.130 | 0.5527 | Yes |
| 48 | COX7C | cytochrome c oxidase subunit 7C [Source:HGNC Symbol;Acc:HGNC:2292] | 3427 | 0.128 | 0.5573 | Yes |
| 49 | COX7A2 | cytochrome c oxidase subunit 7A2 [Source:HGNC Symbol;Acc:HGNC:2288] | 3468 | 0.127 | 0.5639 | Yes |
| 50 | NDUFB2 | NADH:ubiquinone oxidoreductase subunit B2 [Source:HGNC Symbol;Acc:HGNC:7697] | 3507 | 0.126 | 0.5705 | Yes |
| 51 | NDUFV2 | NADH:ubiquinone oxidoreductase core subunit V2 [Source:HGNC Symbol;Acc:HGNC:7717] | 3550 | 0.125 | 0.5770 | Yes |
| 52 | COX6B1 | cytochrome c oxidase subunit 6B1 [Source:HGNC Symbol;Acc:HGNC:2280] | 3572 | 0.125 | 0.5840 | Yes |
| 53 | MT-ND1 | mitochondrially encoded NADH:ubiquinone oxidoreductase core subunit 1 [Source:HGNC Symbol;Acc:HGNC:7455] | 3588 | 0.125 | 0.5911 | Yes |
| 54 | NDUFB7 | NADH:ubiquinone oxidoreductase subunit B7 [Source:HGNC Symbol;Acc:HGNC:7702] | 3667 | 0.123 | 0.5966 | Yes |
| 55 | NDUFC2 | NADH:ubiquinone oxidoreductase subunit C2 [Source:HGNC Symbol;Acc:HGNC:7706] | 3963 | 0.118 | 0.5962 | Yes |
| 56 | NDUFS4 | NADH:ubiquinone oxidoreductase subunit S4 [Source:HGNC Symbol;Acc:HGNC:7711] | 4123 | 0.116 | 0.5991 | Yes |
| 57 | COX4I2 | cytochrome c oxidase subunit 4I2 [Source:HGNC Symbol;Acc:HGNC:16232] | 4182 | 0.115 | 0.6045 | Yes |
| 58 | ATP5PD | ATP synthase peripheral stalk subunit d [Source:HGNC Symbol;Acc:HGNC:845] | 4285 | 0.113 | 0.6087 | Yes |
| 59 | SDHC | succinate dehydrogenase complex subunit C [Source:HGNC Symbol;Acc:HGNC:10682] | 4323 | 0.112 | 0.6145 | Yes |
| 60 | COX6C | cytochrome c oxidase subunit 6C [Source:HGNC Symbol;Acc:HGNC:2285] | 4326 | 0.112 | 0.6212 | Yes |
| 61 | MT-CO3 | mitochondrially encoded cytochrome c oxidase III [Source:HGNC Symbol;Acc:HGNC:7422] | 4561 | 0.109 | 0.6218 | Yes |
| 62 | COX7A2L | cytochrome c oxidase subunit 7A2 like [Source:HGNC Symbol;Acc:HGNC:2289] | 4754 | 0.106 | 0.6233 | Yes |
| 63 | COX7B | cytochrome c oxidase subunit 7B [Source:HGNC Symbol;Acc:HGNC:2291] | 4828 | 0.105 | 0.6278 | Yes |
| 64 | COX6CP3 | cytochrome c oxidase subunit 6C pseudogene 3 [Source:HGNC Symbol;Acc:HGNC:31721] | 4943 | 0.104 | 0.6311 | Yes |
| 65 | MT-ND5 | mitochondrially encoded NADH:ubiquinone oxidoreductase core subunit 5 [Source:HGNC Symbol;Acc:HGNC:7461] | 5023 | 0.102 | 0.6352 | Yes |
| 66 | COX6A1 | cytochrome c oxidase subunit 6A1 [Source:HGNC Symbol;Acc:HGNC:2277] | 5113 | 0.101 | 0.6391 | Yes |
| 67 | NDUFA4 | NDUFA4 mitochondrial complex associated [Source:HGNC Symbol;Acc:HGNC:7687] | 5480 | 0.097 | 0.6356 | Yes |
| 68 | UQCRC2 | ubiquinol-cytochrome c reductase core protein 2 [Source:HGNC Symbol;Acc:HGNC:12586] | 5923 | 0.095 | 0.6300 | Yes |
| 69 | ATP5MC1 | ATP synthase membrane subunit c locus 1 [Source:HGNC Symbol;Acc:HGNC:841] | 5965 | 0.094 | 0.6346 | Yes |
| 70 | NDUFV3 | NADH:ubiquinone oxidoreductase subunit V3 [Source:HGNC Symbol;Acc:HGNC:7719] | 6013 | 0.094 | 0.6391 | Yes |
| 71 | NDUFA1 | NADH:ubiquinone oxidoreductase subunit A1 [Source:HGNC Symbol;Acc:HGNC:7683] | 6036 | 0.094 | 0.6442 | Yes |
| 72 | COX6A2 | cytochrome c oxidase subunit 6A2 [Source:HGNC Symbol;Acc:HGNC:2279] | 6345 | 0.090 | 0.6417 | Yes |
| 73 | NDUFA6 | NADH:ubiquinone oxidoreductase subunit A6 [Source:HGNC Symbol;Acc:HGNC:7690] | 6634 | 0.087 | 0.6396 | Yes |
| 74 | COX7B2 | cytochrome c oxidase subunit 7B2 [Source:HGNC Symbol;Acc:HGNC:24381] | 6753 | 0.086 | 0.6418 | Yes |
| 75 | MT-CO2 | mitochondrially encoded cytochrome c oxidase II [Source:HGNC Symbol;Acc:HGNC:7421] | 7144 | 0.082 | 0.6368 | Yes |
| 76 | SLC25A5 | solute carrier family 25 member 5 [Source:HGNC Symbol;Acc:HGNC:10991] | 7217 | 0.082 | 0.6399 | Yes |
| 77 | UBA1 | ubiquitin like modifier activating enzyme 1 [Source:HGNC Symbol;Acc:HGNC:12469] | 7495 | 0.079 | 0.6376 | Yes |
| 78 | NDUFA5 | NADH:ubiquinone oxidoreductase subunit A5 [Source:HGNC Symbol;Acc:HGNC:7688] | 7600 | 0.078 | 0.6397 | Yes |
| 79 | ATP5F1D | ATP synthase F1 subunit delta [Source:HGNC Symbol;Acc:HGNC:837] | 7651 | 0.078 | 0.6431 | Yes |
| 80 | ATP5F1A | ATP synthase F1 subunit alpha [Source:HGNC Symbol;Acc:HGNC:823] | 7734 | 0.077 | 0.6456 | Yes |
| 81 | MT-ND3 | mitochondrially encoded NADH:ubiquinone oxidoreductase core subunit 3 [Source:HGNC Symbol;Acc:HGNC:7458] | 8252 | 0.073 | 0.6368 | No |
| 82 | COX5B | cytochrome c oxidase subunit 5B [Source:HGNC Symbol;Acc:HGNC:2269] | 8271 | 0.072 | 0.6407 | No |
| 83 | UBE2G2 | ubiquitin conjugating enzyme E2 G2 [Source:HGNC Symbol;Acc:HGNC:12483] | 9220 | 0.066 | 0.6205 | No |
| 84 | MT-ND4 | mitochondrially encoded NADH:ubiquinone oxidoreductase core subunit 4 [Source:HGNC Symbol;Acc:HGNC:7459] | 9732 | 0.062 | 0.6112 | No |
| 85 | MT-CYB | mitochondrially encoded cytochrome b [Source:HGNC Symbol;Acc:HGNC:7427] | 9843 | 0.061 | 0.6121 | No |
| 86 | SLC25A4 | solute carrier family 25 member 4 [Source:HGNC Symbol;Acc:HGNC:10990] | 9848 | 0.061 | 0.6157 | No |
| 87 | UQCR11 | "ubiquinol-cytochrome c reductase, complex III subunit XI [Source:HGNC Symbol;Acc:HGNC:30862]" | 9868 | 0.061 | 0.6188 | No |
| 88 | UBE2L3 | ubiquitin conjugating enzyme E2 L3 [Source:HGNC Symbol;Acc:HGNC:12488] | 10263 | 0.058 | 0.6123 | No |
| 89 | UQCRFS1 | "ubiquinol-cytochrome c reductase, Rieske iron-sulfur polypeptide 1 [Source:HGNC Symbol;Acc:HGNC:12587]" | 10480 | 0.057 | 0.6102 | No |
| 90 | COX8C | cytochrome c oxidase subunit 8C [Source:HGNC Symbol;Acc:HGNC:24382] | 11354 | 0.051 | 0.5910 | No |
| 91 | MT-ATP6 | mitochondrially encoded ATP synthase membrane subunit 6 [Source:HGNC Symbol;Acc:HGNC:7414] | 11361 | 0.051 | 0.5940 | No |
| 92 | NDUFA7 | NADH:ubiquinone oxidoreductase subunit A7 [Source:HGNC Symbol;Acc:HGNC:7691] | 11506 | 0.050 | 0.5933 | No |
| 93 | MT-ND2 | mitochondrially encoded NADH:ubiquinone oxidoreductase core subunit 2 [Source:HGNC Symbol;Acc:HGNC:7456] | 11807 | 0.048 | 0.5886 | No |
| 94 | NDUFS2 | NADH:ubiquinone oxidoreductase core subunit S2 [Source:HGNC Symbol;Acc:HGNC:7708] | 13107 | 0.040 | 0.5579 | No |
| 95 | VDAC1 | voltage dependent anion channel 1 [Source:HGNC Symbol;Acc:HGNC:12669] | 13291 | 0.039 | 0.5555 | No |
| 96 | MT-ND4L | mitochondrially encoded NADH:ubiquinone oxidoreductase core subunit 4L [Source:HGNC Symbol;Acc:HGNC:7460] | 13712 | 0.037 | 0.5470 | No |
| 97 | ATP5F1B | ATP synthase F1 subunit beta [Source:HGNC Symbol;Acc:HGNC:830] | 14082 | 0.035 | 0.5397 | No |
| 98 | SLC18A1 | solute carrier family 18 member A1 [Source:HGNC Symbol;Acc:HGNC:10934] | 14297 | 0.033 | 0.5362 | No |
| 99 | SLC25A31 | solute carrier family 25 member 31 [Source:HGNC Symbol;Acc:HGNC:25319] | 14788 | 0.030 | 0.5256 | No |
| 100 | NDUFB6 | NADH:ubiquinone oxidoreductase subunit B6 [Source:HGNC Symbol;Acc:HGNC:7701] | 15775 | 0.025 | 0.5019 | No |
| 101 | MT-ATP8 | mitochondrially encoded ATP synthase membrane subunit 8 [Source:HGNC Symbol;Acc:HGNC:7415] | 16163 | 0.022 | 0.4934 | No |
| 102 | COX7A1 | cytochrome c oxidase subunit 7A1 [Source:HGNC Symbol;Acc:HGNC:2287] | 16527 | 0.020 | 0.4853 | No |
| 103 | NDUFA4L2 | NDUFA4 mitochondrial complex associated like 2 [Source:HGNC Symbol;Acc:HGNC:29836] | 16755 | 0.019 | 0.4807 | No |
| 104 | ATP5MC3 | ATP synthase membrane subunit c locus 3 [Source:HGNC Symbol;Acc:HGNC:843] | 16867 | 0.018 | 0.4790 | No |
| 105 | NDUFS1 | NADH:ubiquinone oxidoreductase core subunit S1 [Source:HGNC Symbol;Acc:HGNC:7707] | 17437 | 0.015 | 0.4654 | No |
| 106 | ATP5MC1P5 | ATP synthase membrane subunit c locus 1 pseudogene 5 [Source:HGNC Symbol;Acc:HGNC:39508] | 19858 | 0.002 | 0.4037 | No |
| 107 | UBE2L6 | ubiquitin conjugating enzyme E2 L6 [Source:HGNC Symbol;Acc:HGNC:12490] | 20023 | 0.001 | 0.3996 | No |
| 108 | PPID | peptidylprolyl isomerase D [Source:HGNC Symbol;Acc:HGNC:9257] | 20517 | -0.002 | 0.3871 | No |
| 109 | APAF1 | apoptotic peptidase activating factor 1 [Source:HGNC Symbol;Acc:HGNC:576] | 20948 | -0.005 | 0.3765 | No |
| 110 | VDAC2 | voltage dependent anion channel 2 [Source:HGNC Symbol;Acc:HGNC:12672] | 21450 | -0.008 | 0.3641 | No |
| 111 | UBA7 | ubiquitin like modifier activating enzyme 7 [Source:HGNC Symbol;Acc:HGNC:12471] | 22255 | -0.013 | 0.3444 | No |
| 112 | COX5A | cytochrome c oxidase subunit 5A [Source:HGNC Symbol;Acc:HGNC:2267] | 22458 | -0.014 | 0.3401 | No |
| 113 | SDHD | succinate dehydrogenase complex subunit D [Source:HGNC Symbol;Acc:HGNC:10683] | 22998 | -0.017 | 0.3274 | No |
| 114 | CYCS | "cytochrome c, somatic [Source:HGNC Symbol;Acc:HGNC:19986]" | 24187 | -0.025 | 0.2986 | No |
| 115 | SNCAIP | synuclein alpha interacting protein [Source:HGNC Symbol;Acc:HGNC:11139] | 24309 | -0.026 | 0.2971 | No |
| 116 | UQCR10P1 | UQCR10 pseudogene 1 [Source:HGNC Symbol;Acc:HGNC:54960] | 25693 | -0.036 | 0.2639 | No |
| 117 | VDAC3 | voltage dependent anion channel 3 [Source:HGNC Symbol;Acc:HGNC:12674] | 25866 | -0.037 | 0.2618 | No |
| 118 | SNCA | synuclein alpha [Source:HGNC Symbol;Acc:HGNC:11138] | 25948 | -0.038 | 0.2620 | No |
| 119 | VDAC2P5 | VDAC2 pseudogene 5 [Source:HGNC Symbol;Acc:HGNC:54753] | 26724 | -0.043 | 0.2448 | No |
| 120 | NDUFB5 | NADH:ubiquinone oxidoreductase subunit B5 [Source:HGNC Symbol;Acc:HGNC:7700] | 29913 | -0.070 | 0.1677 | No |
| 121 | NDUFA8 | NADH:ubiquinone oxidoreductase subunit A8 [Source:HGNC Symbol;Acc:HGNC:7692] | 31722 | -0.088 | 0.1269 | No |
| 122 | UBE2J1 | ubiquitin conjugating enzyme E2 J1 [Source:HGNC Symbol;Acc:HGNC:17598] | 32696 | -0.099 | 0.1080 | No |
| 123 | CASP3 | caspase 3 [Source:HGNC Symbol;Acc:HGNC:1504] | 32939 | -0.103 | 0.1080 | No |
| 124 | SEPTIN5 | septin 5 [Source:HGNC Symbol;Acc:HGNC:9164] | 33017 | -0.104 | 0.1123 | No |
| 125 | LRRK2 | leucine rich repeat kinase 2 [Source:HGNC Symbol;Acc:HGNC:18618] | 33578 | -0.111 | 0.1047 | No |
| 126 | SLC18A2 | solute carrier family 18 member A2 [Source:HGNC Symbol;Acc:HGNC:10935] | 33958 | -0.117 | 0.1021 | No |
| 127 | UBE2G1 | ubiquitin conjugating enzyme E2 G1 [Source:HGNC Symbol;Acc:HGNC:12482] | 34277 | -0.122 | 0.1013 | No |
| 128 | SLC6A3 | solute carrier family 6 member 3 [Source:HGNC Symbol;Acc:HGNC:11049] | 35322 | -0.142 | 0.0832 | No |
| 129 | PRKN | parkin RBR E3 ubiquitin protein ligase [Source:HGNC Symbol;Acc:HGNC:8607] | 35712 | -0.153 | 0.0825 | No |
| 130 | UCHL1 | ubiquitin C-terminal hydrolase L1 [Source:HGNC Symbol;Acc:HGNC:12513] | 35974 | -0.160 | 0.0854 | No |
Table: GSEA details [plain text format]

  

Fig 2: KEGG\_PARKINSONS\_DISEASE      
 Blue-Pink O' Gram in the Space of the Analyzed GeneSet

  

Fig 3: KEGG\_PARKINSONS\_DISEASE: Random ES distribution      
 Gene set null distribution of ES for **KEGG\_PARKINSONS\_DISEASE**
