# Supplementary material for: Simultaneous Presentation of Multiple Myeloma and Lung Cancer: Case Report and Gene Bioinformatics Analysis
Source: Front Oncol. 2022 Jun 13;12:859735. doi: 10.3389/fonc.2022.859735 (PMC9235397; doi:10.3389/fonc.2022.859735)
Supplement: Supplementary file 1 [file DataSheet_1.zip › The bioinformatic analysis of MM and lung cancer supplementary materials/Enrichment analysis/MECR/GSEA_4.1.0/LUSC TCGA/KEGG.Gsea.1639042374131/KEGG_PATHWAYS_IN_CANCER.html]

Details for gene set KEGG\_PATHWAYS\_IN\_CANCER[GSEA]

|  || Dataset | ExpData\_collapsed\_to\_symbols.ENSG00000116353\_profile\_in\_ExpData.cls #ENSG00000116353 |
| Phenotype | ENSG00000116353\_profile\_in\_ExpData.cls#ENSG00000116353 |
| Upregulated in class | ENSG00000116353\_neg |
| GeneSet | KEGG\_PATHWAYS\_IN\_CANCER |
| Enrichment Score (ES) | -0.36368406 |
| Normalized Enrichment Score (NES) | -1.8389059 |
| Nominal p-value | 0.0 |
| FDR q-value | 0.008995739 |
| FWER p-Value | 0.085 |
Table: GSEA Results Summary

  

Fig 1: Enrichment plot: KEGG\_PATHWAYS\_IN\_CANCER      
 Profile of the Running ES Score & Positions of GeneSet Members on the Rank Ordered List

  

| SYMBOL | TITLE | RANK IN GENE LIST | RANK METRIC SCORE | RUNNING ES | CORE ENRICHMENT || 1 | DVL1 | dishevelled segment polarity protein 1 [Source:HGNC Symbol;Acc:HGNC:3084] | 49 | 0.451 | 0.0113 | No |
| 2 | CDC42 | cell division cycle 42 [Source:HGNC Symbol;Acc:HGNC:1736] | 81 | 0.411 | 0.0219 | No |
| 3 | HDAC1 | histone deacetylase 1 [Source:HGNC Symbol;Acc:HGNC:4852] | 246 | 0.325 | 0.0267 | No |
| 4 | CASP9 | caspase 9 [Source:HGNC Symbol;Acc:HGNC:1511] | 300 | 0.306 | 0.0339 | No |
| 5 | MTOR | mechanistic target of rapamycin kinase [Source:HGNC Symbol;Acc:HGNC:3942] | 414 | 0.283 | 0.0388 | No |
| 6 | MAPK3 | mitogen-activated protein kinase 3 [Source:HGNC Symbol;Acc:HGNC:6877] | 473 | 0.274 | 0.0449 | No |
| 7 | ELOB | elongin B [Source:HGNC Symbol;Acc:HGNC:11619] | 509 | 0.269 | 0.0515 | No |
| 8 | ELOC | elongin C [Source:HGNC Symbol;Acc:HGNC:11617] | 576 | 0.259 | 0.0570 | No |
| 9 | BAX | "BCL2 associated X, apoptosis regulator [Source:HGNC Symbol;Acc:HGNC:959]" | 585 | 0.258 | 0.0640 | No |
| 10 | RBX1 | ring-box 1 [Source:HGNC Symbol;Acc:HGNC:9928] | 689 | 0.246 | 0.0682 | No |
| 11 | NRAS | "NRAS proto-oncogene, GTPase [Source:HGNC Symbol;Acc:HGNC:7989]" | 774 | 0.235 | 0.0725 | No |
| 12 | WNT10A | Wnt family member 10A [Source:HGNC Symbol;Acc:HGNC:13829] | 839 | 0.228 | 0.0772 | No |
| 13 | BCR | BCR activator of RhoGEF and GTPase [Source:HGNC Symbol;Acc:HGNC:1014] | 928 | 0.220 | 0.0811 | No |
| 14 | BAD | BCL2 associated agonist of cell death [Source:HGNC Symbol;Acc:HGNC:936] | 945 | 0.219 | 0.0867 | No |
| 15 | MAPK8 | mitogen-activated protein kinase 8 [Source:HGNC Symbol;Acc:HGNC:6881] | 948 | 0.218 | 0.0927 | No |
| 16 | BCL2L1 | BCL2 like 1 [Source:HGNC Symbol;Acc:HGNC:992] | 967 | 0.217 | 0.0983 | No |
| 17 | WNT4 | Wnt family member 4 [Source:HGNC Symbol;Acc:HGNC:12783] | 1086 | 0.210 | 0.1011 | No |
| 18 | VEGFB | vascular endothelial growth factor B [Source:HGNC Symbol;Acc:HGNC:12681] | 1423 | 0.190 | 0.0978 | No |
| 19 | CTBP1 | C-terminal binding protein 1 [Source:HGNC Symbol;Acc:HGNC:2494] | 1610 | 0.182 | 0.0981 | No |
| 20 | CTBP2 | C-terminal binding protein 2 [Source:HGNC Symbol;Acc:HGNC:2495] | 1773 | 0.174 | 0.0987 | No |
| 21 | RAC3 | Rac family small GTPase 3 [Source:HGNC Symbol;Acc:HGNC:9803] | 1777 | 0.174 | 0.1035 | No |
| 22 | TCF7L2 | transcription factor 7 like 2 [Source:HGNC Symbol;Acc:HGNC:11641] | 1930 | 0.168 | 0.1043 | No |
| 23 | ITGA3 | integrin subunit alpha 3 [Source:HGNC Symbol;Acc:HGNC:6139] | 2053 | 0.164 | 0.1057 | No |
| 24 | CUL2 | cullin 2 [Source:HGNC Symbol;Acc:HGNC:2552] | 2274 | 0.156 | 0.1044 | No |
| 25 | PIK3R3 | phosphoinositide-3-kinase regulatory subunit 3 [Source:HGNC Symbol;Acc:HGNC:8981] | 2364 | 0.153 | 0.1064 | No |
| 26 | RXRB | retinoid X receptor beta [Source:HGNC Symbol;Acc:HGNC:10478] | 2453 | 0.151 | 0.1083 | No |
| 27 | CXCL8 | C-X-C motif chemokine ligand 8 [Source:HGNC Symbol;Acc:HGNC:6025] | 2635 | 0.146 | 0.1077 | No |
| 28 | FGF11 | fibroblast growth factor 11 [Source:HGNC Symbol;Acc:HGNC:3667] | 2838 | 0.141 | 0.1064 | No |
| 29 | AKT2 | AKT serine/threonine kinase 2 [Source:HGNC Symbol;Acc:HGNC:392] | 2866 | 0.140 | 0.1096 | No |
| 30 | CCDC6 | coiled-coil domain containing 6 [Source:HGNC Symbol;Acc:HGNC:18782] | 2925 | 0.139 | 0.1120 | No |
| 31 | TPM3 | tropomyosin 3 [Source:HGNC Symbol;Acc:HGNC:12012] | 3045 | 0.135 | 0.1127 | No |
| 32 | TRAF4 | TNF receptor associated factor 4 [Source:HGNC Symbol;Acc:HGNC:12034] | 3282 | 0.131 | 0.1103 | No |
| 33 | E2F2 | E2F transcription factor 2 [Source:HGNC Symbol;Acc:HGNC:3114] | 3336 | 0.129 | 0.1125 | No |
| 34 | GLI3 | GLI family zinc finger 3 [Source:HGNC Symbol;Acc:HGNC:4319] | 3544 | 0.126 | 0.1107 | No |
| 35 | RASSF1 | Ras association domain family member 1 [Source:HGNC Symbol;Acc:HGNC:9882] | 3571 | 0.125 | 0.1135 | No |
| 36 | LAMB4 | laminin subunit beta 4 [Source:HGNC Symbol;Acc:HGNC:6491] | 3596 | 0.125 | 0.1163 | No |
| 37 | SOS2 | SOS Ras/Rho guanine nucleotide exchange factor 2 [Source:HGNC Symbol;Acc:HGNC:11188] | 3713 | 0.123 | 0.1168 | No |
| 38 | AKT1 | AKT serine/threonine kinase 1 [Source:HGNC Symbol;Acc:HGNC:391] | 3889 | 0.120 | 0.1156 | No |
| 39 | SUFU | SUFU negative regulator of hedgehog signaling [Source:HGNC Symbol;Acc:HGNC:16466] | 3947 | 0.119 | 0.1174 | No |
| 40 | RAC1 | Rac family small GTPase 1 [Source:HGNC Symbol;Acc:HGNC:9801] | 4156 | 0.115 | 0.1153 | No |
| 41 | ITGB1 | integrin subunit beta 1 [Source:HGNC Symbol;Acc:HGNC:6153] | 4161 | 0.115 | 0.1184 | No |
| 42 | KIT | "KIT proto-oncogene, receptor tyrosine kinase [Source:HGNC Symbol;Acc:HGNC:6342]" | 4449 | 0.110 | 0.1141 | No |
| 43 | PTGS2 | prostaglandin-endoperoxide synthase 2 [Source:HGNC Symbol;Acc:HGNC:9605] | 4545 | 0.109 | 0.1147 | No |
| 44 | WNT6 | Wnt family member 6 [Source:HGNC Symbol;Acc:HGNC:12785] | 4672 | 0.107 | 0.1144 | No |
| 45 | RHOA | ras homolog family member A [Source:HGNC Symbol;Acc:HGNC:667] | 4969 | 0.103 | 0.1097 | No |
| 46 | CSF3R | colony stimulating factor 3 receptor [Source:HGNC Symbol;Acc:HGNC:2439] | 5008 | 0.103 | 0.1116 | No |
| 47 | PTK2 | protein tyrosine kinase 2 [Source:HGNC Symbol;Acc:HGNC:9611] | 5065 | 0.102 | 0.1130 | No |
| 48 | EGLN3 | egl-9 family hypoxia inducible factor 3 [Source:HGNC Symbol;Acc:HGNC:14661] | 5299 | 0.099 | 0.1097 | No |
| 49 | ARAF | "A-Raf proto-oncogene, serine/threonine kinase [Source:HGNC Symbol;Acc:HGNC:646]" | 5337 | 0.099 | 0.1115 | No |
| 50 | RELA | "RELA proto-oncogene, NF-kB subunit [Source:HGNC Symbol;Acc:HGNC:9955]" | 5531 | 0.096 | 0.1092 | No |
| 51 | MMP1 | matrix metallopeptidase 1 [Source:HGNC Symbol;Acc:HGNC:7155] | 5893 | 0.095 | 0.1026 | No |
| 52 | WNT16 | Wnt family member 16 [Source:HGNC Symbol;Acc:HGNC:16267] | 6037 | 0.094 | 0.1016 | No |
| 53 | E2F1 | E2F transcription factor 1 [Source:HGNC Symbol;Acc:HGNC:3113] | 6233 | 0.091 | 0.0991 | No |
| 54 | JAK1 | Janus kinase 1 [Source:HGNC Symbol;Acc:HGNC:6190] | 6266 | 0.091 | 0.1008 | No |
| 55 | CBLC | Cbl proto-oncogene C [Source:HGNC Symbol;Acc:HGNC:15961] | 6343 | 0.090 | 0.1014 | No |
| 56 | EGLN2 | egl-9 family hypoxia inducible factor 2 [Source:HGNC Symbol;Acc:HGNC:14660] | 6429 | 0.089 | 0.1016 | No |
| 57 | CCND1 | cyclin D1 [Source:HGNC Symbol;Acc:HGNC:1582] | 6470 | 0.089 | 0.1031 | No |
| 58 | KLK3 | kallikrein related peptidase 3 [Source:HGNC Symbol;Acc:HGNC:6364] | 6472 | 0.089 | 0.1055 | No |
| 59 | BID | BH3 interacting domain death agonist [Source:HGNC Symbol;Acc:HGNC:1050] | 6572 | 0.088 | 0.1054 | No |
| 60 | DAPK3 | death associated protein kinase 3 [Source:HGNC Symbol;Acc:HGNC:2676] | 7303 | 0.081 | 0.0890 | No |
| 61 | EPAS1 | endothelial PAS domain protein 1 [Source:HGNC Symbol;Acc:HGNC:3374] | 7346 | 0.081 | 0.0901 | No |
| 62 | CCNE1 | cyclin E1 [Source:HGNC Symbol;Acc:HGNC:1589] | 7411 | 0.080 | 0.0907 | No |
| 63 | NFKB2 | nuclear factor kappa B subunit 2 [Source:HGNC Symbol;Acc:HGNC:7795] | 7816 | 0.076 | 0.0825 | No |
| 64 | CDK6 | cyclin dependent kinase 6 [Source:HGNC Symbol;Acc:HGNC:1777] | 7904 | 0.076 | 0.0823 | No |
| 65 | CKS1B | CDC28 protein kinase regulatory subunit 1B [Source:HGNC Symbol;Acc:HGNC:19083] | 8004 | 0.075 | 0.0819 | No |
| 66 | RARA | retinoic acid receptor alpha [Source:HGNC Symbol;Acc:HGNC:9864] | 8357 | 0.072 | 0.0748 | No |
| 67 | WNT8A | Wnt family member 8A [Source:HGNC Symbol;Acc:HGNC:12788] | 8416 | 0.071 | 0.0753 | No |
| 68 | FGF20 | fibroblast growth factor 20 [Source:HGNC Symbol;Acc:HGNC:3677] | 8718 | 0.069 | 0.0695 | No |
| 69 | TGFB2 | transforming growth factor beta 2 [Source:HGNC Symbol;Acc:HGNC:11768] | 8941 | 0.068 | 0.0657 | No |
| 70 | NCOA4 | nuclear receptor coactivator 4 [Source:HGNC Symbol;Acc:HGNC:7671] | 9204 | 0.066 | 0.0608 | No |
| 71 | ERBB2 | erb-b2 receptor tyrosine kinase 2 [Source:HGNC Symbol;Acc:HGNC:3430] | 9497 | 0.064 | 0.0551 | No |
| 72 | LAMA5 | laminin subunit alpha 5 [Source:HGNC Symbol;Acc:HGNC:6485] | 9649 | 0.063 | 0.0530 | No |
| 73 | CDKN2A | cyclin dependent kinase inhibitor 2A [Source:HGNC Symbol;Acc:HGNC:1787] | 9790 | 0.062 | 0.0511 | No |
| 74 | PTEN | phosphatase and tensin homolog [Source:HGNC Symbol;Acc:HGNC:9588] | 9897 | 0.061 | 0.0501 | No |
| 75 | PDGFA | platelet derived growth factor subunit A [Source:HGNC Symbol;Acc:HGNC:8799] | 10013 | 0.060 | 0.0488 | No |
| 76 | GSTP1 | glutathione S-transferase pi 1 [Source:HGNC Symbol;Acc:HGNC:4638] | 10456 | 0.057 | 0.0390 | No |
| 77 | LAMC2 | laminin subunit gamma 2 [Source:HGNC Symbol;Acc:HGNC:6493] | 10475 | 0.057 | 0.0402 | No |
| 78 | FH | fumarate hydratase [Source:HGNC Symbol;Acc:HGNC:3700] | 10622 | 0.056 | 0.0380 | No |
| 79 | WNT7A | Wnt family member 7A [Source:HGNC Symbol;Acc:HGNC:12786] | 10774 | 0.055 | 0.0356 | No |
| 80 | FADD | Fas associated via death domain [Source:HGNC Symbol;Acc:HGNC:3573] | 10793 | 0.055 | 0.0367 | No |
| 81 | FZD5 | frizzled class receptor 5 [Source:HGNC Symbol;Acc:HGNC:4043] | 11161 | 0.053 | 0.0287 | No |
| 82 | FLT3LG | fms related receptor tyrosine kinase 3 ligand [Source:HGNC Symbol;Acc:HGNC:3766] | 11406 | 0.051 | 0.0239 | No |
| 83 | BIRC5 | baculoviral IAP repeat containing 5 [Source:HGNC Symbol;Acc:HGNC:593] | 11614 | 0.050 | 0.0200 | No |
| 84 | MECOM | MDS1 and EVI1 complex locus [Source:HGNC Symbol;Acc:HGNC:3498] | 11729 | 0.049 | 0.0184 | No |
| 85 | HDAC2 | histone deacetylase 2 [Source:HGNC Symbol;Acc:HGNC:4853] | 11971 | 0.047 | 0.0135 | No |
| 86 | FGFR3 | fibroblast growth factor receptor 3 [Source:HGNC Symbol;Acc:HGNC:3690] | 12058 | 0.047 | 0.0126 | No |
| 87 | NKX3-1 | NK3 homeobox 1 [Source:HGNC Symbol;Acc:HGNC:7838] | 12396 | 0.045 | 0.0052 | No |
| 88 | STK36 | serine/threonine kinase 36 [Source:HGNC Symbol;Acc:HGNC:17209] | 12694 | 0.043 | -0.0012 | No |
| 89 | MLH1 | mutL homolog 1 [Source:HGNC Symbol;Acc:HGNC:7127] | 12848 | 0.042 | -0.0040 | No |
| 90 | TCF7 | transcription factor 7 [Source:HGNC Symbol;Acc:HGNC:11639] | 13002 | 0.041 | -0.0068 | No |
| 91 | CDKN1A | cyclin dependent kinase inhibitor 1A [Source:HGNC Symbol;Acc:HGNC:1784] | 13243 | 0.039 | -0.0118 | No |
| 92 | FGF5 | fibroblast growth factor 5 [Source:HGNC Symbol;Acc:HGNC:3683] | 13398 | 0.039 | -0.0147 | No |
| 93 | STK4 | serine/threonine kinase 4 [Source:HGNC Symbol;Acc:HGNC:11408] | 13459 | 0.038 | -0.0152 | No |
| 94 | PLCG1 | phospholipase C gamma 1 [Source:HGNC Symbol;Acc:HGNC:9065] | 13655 | 0.037 | -0.0191 | No |
| 95 | E2F3 | E2F transcription factor 3 [Source:HGNC Symbol;Acc:HGNC:3115] | 14038 | 0.035 | -0.0280 | No |
| 96 | SKP2 | S-phase kinase associated protein 2 [Source:HGNC Symbol;Acc:HGNC:10901] | 14724 | 0.031 | -0.0447 | No |
| 97 | MSH2 | mutS homolog 2 [Source:HGNC Symbol;Acc:HGNC:7325] | 14750 | 0.031 | -0.0445 | No |
| 98 | CHUK | component of inhibitor of nuclear factor kappa B kinase complex [Source:HGNC Symbol;Acc:HGNC:1974] | 15052 | 0.029 | -0.0514 | No |
| 99 | PRKCG | protein kinase C gamma [Source:HGNC Symbol;Acc:HGNC:9402] | 15351 | 0.027 | -0.0583 | No |
| 100 | AXIN1 | axin 1 [Source:HGNC Symbol;Acc:HGNC:903] | 15360 | 0.027 | -0.0577 | No |
| 101 | MAP2K2 | mitogen-activated protein kinase kinase 2 [Source:HGNC Symbol;Acc:HGNC:6842] | 15510 | 0.026 | -0.0608 | No |
| 102 | GSK3B | glycogen synthase kinase 3 beta [Source:HGNC Symbol;Acc:HGNC:4617] | 15772 | 0.025 | -0.0668 | No |
| 103 | RARB | retinoic acid receptor beta [Source:HGNC Symbol;Acc:HGNC:9865] | 15931 | 0.024 | -0.0702 | No |
| 104 | TGFBR2 | transforming growth factor beta receptor 2 [Source:HGNC Symbol;Acc:HGNC:11773] | 15982 | 0.023 | -0.0709 | No |
| 105 | RAF1 | "Raf-1 proto-oncogene, serine/threonine kinase [Source:HGNC Symbol;Acc:HGNC:9829]" | 16140 | 0.022 | -0.0743 | No |
| 106 | CTNNA2 | catenin alpha 2 [Source:HGNC Symbol;Acc:HGNC:2510] | 16312 | 0.022 | -0.0780 | No |
| 107 | LAMC3 | laminin subunit gamma 3 [Source:HGNC Symbol;Acc:HGNC:6494] | 16485 | 0.021 | -0.0819 | No |
| 108 | FGF4 | fibroblast growth factor 4 [Source:HGNC Symbol;Acc:HGNC:3682] | 16737 | 0.019 | -0.0878 | No |
| 109 | CCNE2 | cyclin E2 [Source:HGNC Symbol;Acc:HGNC:1590] | 16835 | 0.019 | -0.0898 | No |
| 110 | LAMB3 | laminin subunit beta 3 [Source:HGNC Symbol;Acc:HGNC:6490] | 16938 | 0.018 | -0.0919 | No |
| 111 | VEGFA | vascular endothelial growth factor A [Source:HGNC Symbol;Acc:HGNC:12680] | 17110 | 0.017 | -0.0958 | No |
| 112 | TGFB1 | transforming growth factor beta 1 [Source:HGNC Symbol;Acc:HGNC:11766] | 17301 | 0.016 | -0.1002 | No |
| 113 | VEGFC | vascular endothelial growth factor C [Source:HGNC Symbol;Acc:HGNC:12682] | 17502 | 0.015 | -0.1049 | No |
| 114 | CTNNA3 | catenin alpha 3 [Source:HGNC Symbol;Acc:HGNC:2511] | 17680 | 0.014 | -0.1091 | No |
| 115 | CDK4 | cyclin dependent kinase 4 [Source:HGNC Symbol;Acc:HGNC:1773] | 17764 | 0.014 | -0.1108 | No |
| 116 | FGF6 | fibroblast growth factor 6 [Source:HGNC Symbol;Acc:HGNC:3684] | 18414 | 0.010 | -0.1272 | No |
| 117 | TFG | trafficking from ER to golgi regulator [Source:HGNC Symbol;Acc:HGNC:11758] | 18467 | 0.010 | -0.1283 | No |
| 118 | APC2 | APC regulator of WNT signaling pathway 2 [Source:HGNC Symbol;Acc:HGNC:24036] | 18523 | 0.009 | -0.1294 | No |
| 119 | BIRC2 | baculoviral IAP repeat containing 2 [Source:HGNC Symbol;Acc:HGNC:590] | 18833 | 0.008 | -0.1371 | No |
| 120 | TGFA | transforming growth factor alpha [Source:HGNC Symbol;Acc:HGNC:11765] | 18900 | 0.007 | -0.1386 | No |
| 121 | JUN | "Jun proto-oncogene, AP-1 transcription factor subunit [Source:HGNC Symbol;Acc:HGNC:6204]" | 18921 | 0.007 | -0.1389 | No |
| 122 | CDKN2B | cyclin dependent kinase inhibitor 2B [Source:HGNC Symbol;Acc:HGNC:1788] | 18975 | 0.007 | -0.1401 | No |
| 123 | CTNNA1 | catenin alpha 1 [Source:HGNC Symbol;Acc:HGNC:2509] | 19036 | 0.006 | -0.1415 | No |
| 124 | SLC2A1 | solute carrier family 2 member 1 [Source:HGNC Symbol;Acc:HGNC:11005] | 19212 | 0.005 | -0.1458 | No |
| 125 | MYC | "MYC proto-oncogene, bHLH transcription factor [Source:HGNC Symbol;Acc:HGNC:7553]" | 19309 | 0.005 | -0.1481 | No |
| 126 | WNT1 | Wnt family member 1 [Source:HGNC Symbol;Acc:HGNC:12774] | 19377 | 0.004 | -0.1497 | No |
| 127 | HGF | hepatocyte growth factor [Source:HGNC Symbol;Acc:HGNC:4893] | 19573 | 0.003 | -0.1547 | No |
| 128 | CDK2 | cyclin dependent kinase 2 [Source:HGNC Symbol;Acc:HGNC:1771] | 19966 | 0.001 | -0.1647 | No |
| 129 | HSP90B1 | heat shock protein 90 beta family member 1 [Source:HGNC Symbol;Acc:HGNC:12028] | 20087 | 0.000 | -0.1678 | No |
| 130 | FZD8 | frizzled class receptor 8 [Source:HGNC Symbol;Acc:HGNC:4046] | 20256 | -0.001 | -0.1720 | No |
| 131 | PIK3CB | "phosphatidylinositol-4,5-bisphosphate 3-kinase catalytic subunit beta [Source:HGNC Symbol;Acc:HGNC:8976]" | 20368 | -0.002 | -0.1748 | No |
| 132 | NTRK1 | neurotrophic receptor tyrosine kinase 1 [Source:HGNC Symbol;Acc:HGNC:8031] | 20383 | -0.002 | -0.1752 | No |
| 133 | NFKB1 | nuclear factor kappa B subunit 1 [Source:HGNC Symbol;Acc:HGNC:7794] | 20504 | -0.002 | -0.1782 | No |
| 134 | HRAS | "HRas proto-oncogene, GTPase [Source:HGNC Symbol;Acc:HGNC:5173]" | 20556 | -0.003 | -0.1794 | No |
| 135 | HSP90AA1 | heat shock protein 90 alpha family class A member 1 [Source:HGNC Symbol;Acc:HGNC:5253] | 20653 | -0.003 | -0.1818 | No |
| 136 | TCF7L1 | transcription factor 7 like 1 [Source:HGNC Symbol;Acc:HGNC:11640] | 20720 | -0.004 | -0.1834 | No |
| 137 | FZD4 | frizzled class receptor 4 [Source:HGNC Symbol;Acc:HGNC:4042] | 20770 | -0.004 | -0.1845 | No |
| 138 | BMP2 | bone morphogenetic protein 2 [Source:HGNC Symbol;Acc:HGNC:1069] | 20791 | -0.004 | -0.1849 | No |
| 139 | PGF | placental growth factor [Source:HGNC Symbol;Acc:HGNC:8893] | 21556 | -0.009 | -0.2043 | No |
| 140 | RET | ret proto-oncogene [Source:HGNC Symbol;Acc:HGNC:9967] | 21563 | -0.009 | -0.2042 | No |
| 141 | MET | "MET proto-oncogene, receptor tyrosine kinase [Source:HGNC Symbol;Acc:HGNC:7029]" | 21734 | -0.010 | -0.2083 | No |
| 142 | PIK3R1 | phosphoinositide-3-kinase regulatory subunit 1 [Source:HGNC Symbol;Acc:HGNC:8979] | 22505 | -0.014 | -0.2276 | No |
| 143 | FN1 | fibronectin 1 [Source:HGNC Symbol;Acc:HGNC:3778] | 22538 | -0.015 | -0.2280 | No |
| 144 | RALBP1 | ralA binding protein 1 [Source:HGNC Symbol;Acc:HGNC:9841] | 22541 | -0.015 | -0.2277 | No |
| 145 | PIAS3 | protein inhibitor of activated STAT 3 [Source:HGNC Symbol;Acc:HGNC:16861] | 22673 | -0.015 | -0.2306 | No |
| 146 | AR | androgen receptor [Source:HGNC Symbol;Acc:HGNC:644] | 22741 | -0.016 | -0.2319 | No |
| 147 | PPARD | peroxisome proliferator activated receptor delta [Source:HGNC Symbol;Acc:HGNC:9235] | 22805 | -0.016 | -0.2331 | No |
| 148 | WNT7B | Wnt family member 7B [Source:HGNC Symbol;Acc:HGNC:12787] | 22905 | -0.017 | -0.2351 | No |
| 149 | IKBKB | inhibitor of nuclear factor kappa B kinase subunit beta [Source:HGNC Symbol;Acc:HGNC:5960] | 22918 | -0.017 | -0.2350 | No |
| 150 | NOS2 | nitric oxide synthase 2 [Source:HGNC Symbol;Acc:HGNC:7873] | 22937 | -0.017 | -0.2350 | No |
| 151 | FGF7 | fibroblast growth factor 7 [Source:HGNC Symbol;Acc:HGNC:3685] | 23088 | -0.018 | -0.2383 | No |
| 152 | PDGFB | platelet derived growth factor subunit B [Source:HGNC Symbol;Acc:HGNC:8800] | 23487 | -0.021 | -0.2480 | No |
| 153 | TGFB3 | transforming growth factor beta 3 [Source:HGNC Symbol;Acc:HGNC:11769] | 23618 | -0.021 | -0.2507 | No |
| 154 | FAS | Fas cell surface death receptor [Source:HGNC Symbol;Acc:HGNC:11920] | 23910 | -0.023 | -0.2575 | No |
| 155 | ZBTB16 | zinc finger and BTB domain containing 16 [Source:HGNC Symbol;Acc:HGNC:12930] | 24001 | -0.024 | -0.2592 | No |
| 156 | PIK3CD | "phosphatidylinositol-4,5-bisphosphate 3-kinase catalytic subunit delta [Source:HGNC Symbol;Acc:HGNC:8977]" | 24002 | -0.024 | -0.2585 | No |
| 157 | CYCS | "cytochrome c, somatic [Source:HGNC Symbol;Acc:HGNC:19986]" | 24187 | -0.025 | -0.2625 | No |
| 158 | LAMB1 | laminin subunit beta 1 [Source:HGNC Symbol;Acc:HGNC:6486] | 24497 | -0.027 | -0.2697 | No |
| 159 | CSF2RA | colony stimulating factor 2 receptor subunit alpha [Source:HGNC Symbol;Acc:HGNC:2435] | 24630 | -0.028 | -0.2723 | No |
| 160 | TRAF3 | TNF receptor associated factor 3 [Source:HGNC Symbol;Acc:HGNC:12033] | 24750 | -0.029 | -0.2745 | No |
| 161 | MMP2 | matrix metallopeptidase 2 [Source:HGNC Symbol;Acc:HGNC:7166] | 24850 | -0.030 | -0.2762 | No |
| 162 | BIRC3 | baculoviral IAP repeat containing 3 [Source:HGNC Symbol;Acc:HGNC:591] | 24900 | -0.031 | -0.2766 | No |
| 163 | FGF9 | fibroblast growth factor 9 [Source:HGNC Symbol;Acc:HGNC:3687] | 25006 | -0.031 | -0.2785 | No |
| 164 | MSH3 | mutS homolog 3 [Source:HGNC Symbol;Acc:HGNC:7326] | 25209 | -0.033 | -0.2827 | No |
| 165 | FGF10 | fibroblast growth factor 10 [Source:HGNC Symbol;Acc:HGNC:3666] | 25431 | -0.034 | -0.2875 | No |
| 166 | WNT9A | Wnt family member 9A [Source:HGNC Symbol;Acc:HGNC:12778] | 25791 | -0.037 | -0.2956 | No |
| 167 | CBLB | Cbl proto-oncogene B [Source:HGNC Symbol;Acc:HGNC:1542] | 25811 | -0.037 | -0.2951 | No |
| 168 | RUNX1 | RUNX family transcription factor 1 [Source:HGNC Symbol;Acc:HGNC:10471] | 25970 | -0.038 | -0.2981 | No |
| 169 | RALA | RAS like proto-oncogene A [Source:HGNC Symbol;Acc:HGNC:9839] | 26058 | -0.038 | -0.2993 | No |
| 170 | DAPK2 | death associated protein kinase 2 [Source:HGNC Symbol;Acc:HGNC:2675] | 26071 | -0.039 | -0.2985 | No |
| 171 | COL4A4 | collagen type IV alpha 4 chain [Source:HGNC Symbol;Acc:HGNC:2206] | 26404 | -0.041 | -0.3059 | No |
| 172 | FZD1 | frizzled class receptor 1 [Source:HGNC Symbol;Acc:HGNC:4038] | 26540 | -0.042 | -0.3082 | No |
| 173 | WNT10B | Wnt family member 10B [Source:HGNC Symbol;Acc:HGNC:12775] | 26603 | -0.042 | -0.3086 | No |
| 174 | FGF1 | fibroblast growth factor 1 [Source:HGNC Symbol;Acc:HGNC:3665] | 26735 | -0.044 | -0.3107 | No |
| 175 | PAX8 | paired box 8 [Source:HGNC Symbol;Acc:HGNC:8622] | 26762 | -0.044 | -0.3102 | No |
| 176 | HSP90AB1 | heat shock protein 90 alpha family class B member 1 [Source:HGNC Symbol;Acc:HGNC:5258] | 27066 | -0.046 | -0.3167 | No |
| 177 | ETS1 | "ETS proto-oncogene 1, transcription factor [Source:HGNC Symbol;Acc:HGNC:3488]" | 27265 | -0.048 | -0.3204 | No |
| 178 | STAT1 | signal transducer and activator of transcription 1 [Source:HGNC Symbol;Acc:HGNC:11362] | 27832 | -0.052 | -0.3335 | No |
| 179 | CASP8 | caspase 8 [Source:HGNC Symbol;Acc:HGNC:1509] | 27944 | -0.053 | -0.3349 | No |
| 180 | ITGA2B | integrin subunit alpha 2b [Source:HGNC Symbol;Acc:HGNC:6138] | 27955 | -0.053 | -0.3337 | No |
| 181 | SPI1 | Spi-1 proto-oncogene [Source:HGNC Symbol;Acc:HGNC:11241] | 28126 | -0.054 | -0.3365 | No |
| 182 | TRAF2 | TNF receptor associated factor 2 [Source:HGNC Symbol;Acc:HGNC:12032] | 28161 | -0.055 | -0.3359 | No |
| 183 | HHIP | hedgehog interacting protein [Source:HGNC Symbol;Acc:HGNC:14866] | 28238 | -0.055 | -0.3363 | No |
| 184 | FZD9 | frizzled class receptor 9 [Source:HGNC Symbol;Acc:HGNC:4047] | 28404 | -0.057 | -0.3390 | No |
| 185 | MAPK9 | mitogen-activated protein kinase 9 [Source:HGNC Symbol;Acc:HGNC:6886] | 28470 | -0.057 | -0.3390 | No |
| 186 | MITF | melanocyte inducing transcription factor [Source:HGNC Symbol;Acc:HGNC:7105] | 28642 | -0.058 | -0.3418 | No |
| 187 | FGF18 | fibroblast growth factor 18 [Source:HGNC Symbol;Acc:HGNC:3674] | 28718 | -0.059 | -0.3421 | No |
| 188 | FGFR1 | fibroblast growth factor receptor 1 [Source:HGNC Symbol;Acc:HGNC:3688] | 28851 | -0.060 | -0.3438 | No |
| 189 | FGF21 | fibroblast growth factor 21 [Source:HGNC Symbol;Acc:HGNC:3678] | 28858 | -0.060 | -0.3423 | No |
| 190 | PML | PML nuclear body scaffold [Source:HGNC Symbol;Acc:HGNC:9113] | 28982 | -0.061 | -0.3437 | No |
| 191 | WNT3A | Wnt family member 3A [Source:HGNC Symbol;Acc:HGNC:15983] | 29041 | -0.062 | -0.3435 | No |
| 192 | MDM2 | MDM2 proto-oncogene [Source:HGNC Symbol;Acc:HGNC:6973] | 29250 | -0.064 | -0.3471 | No |
| 193 | LAMB2 | laminin subunit beta 2 [Source:HGNC Symbol;Acc:HGNC:6487] | 29260 | -0.064 | -0.3455 | No |
| 194 | LAMA2 | laminin subunit alpha 2 [Source:HGNC Symbol;Acc:HGNC:6482] | 29277 | -0.064 | -0.3442 | No |
| 195 | RAD51 | RAD51 recombinase [Source:HGNC Symbol;Acc:HGNC:9817] | 29329 | -0.065 | -0.3437 | No |
| 196 | CDKN1B | cyclin dependent kinase inhibitor 1B [Source:HGNC Symbol;Acc:HGNC:1785] | 29446 | -0.066 | -0.3448 | No |
| 197 | FGF23 | fibroblast growth factor 23 [Source:HGNC Symbol;Acc:HGNC:3680] | 29547 | -0.066 | -0.3456 | No |
| 198 | RXRA | retinoid X receptor alpha [Source:HGNC Symbol;Acc:HGNC:10477] | 29745 | -0.068 | -0.3487 | No |
| 199 | LAMA3 | laminin subunit alpha 3 [Source:HGNC Symbol;Acc:HGNC:6483] | 29779 | -0.069 | -0.3476 | No |
| 200 | RAC2 | Rac family small GTPase 2 [Source:HGNC Symbol;Acc:HGNC:9802] | 29782 | -0.069 | -0.3458 | No |
| 201 | BMP4 | bone morphogenetic protein 4 [Source:HGNC Symbol;Acc:HGNC:1071] | 29894 | -0.070 | -0.3467 | No |
| 202 | FASLG | Fas ligand [Source:HGNC Symbol;Acc:HGNC:11936] | 29910 | -0.070 | -0.3451 | No |
| 203 | PIK3R5 | phosphoinositide-3-kinase regulatory subunit 5 [Source:HGNC Symbol;Acc:HGNC:30035] | 30064 | -0.071 | -0.3471 | No |
| 204 | RUNX1T1 | RUNX1 partner transcriptional co-repressor 1 [Source:HGNC Symbol;Acc:HGNC:1535] | 30072 | -0.071 | -0.3453 | No |
| 205 | ARNT | aryl hydrocarbon receptor nuclear translocator [Source:HGNC Symbol;Acc:HGNC:700] | 30092 | -0.072 | -0.3438 | No |
| 206 | CSF1R | colony stimulating factor 1 receptor [Source:HGNC Symbol;Acc:HGNC:2433] | 30287 | -0.073 | -0.3467 | No |
| 207 | PDGFRA | platelet derived growth factor receptor alpha [Source:HGNC Symbol;Acc:HGNC:8803] | 30377 | -0.074 | -0.3469 | No |
| 208 | STAT5A | signal transducer and activator of transcription 5A [Source:HGNC Symbol;Acc:HGNC:11366] | 30460 | -0.075 | -0.3470 | No |
| 209 | CEBPA | CCAAT enhancer binding protein alpha [Source:HGNC Symbol;Acc:HGNC:1833] | 30565 | -0.076 | -0.3475 | No |
| 210 | TRAF5 | TNF receptor associated factor 5 [Source:HGNC Symbol;Acc:HGNC:12035] | 30629 | -0.077 | -0.3470 | No |
| 211 | PIK3R2 | phosphoinositide-3-kinase regulatory subunit 2 [Source:HGNC Symbol;Acc:HGNC:8980] | 30740 | -0.078 | -0.3477 | No |
| 212 | FLT3 | fms related receptor tyrosine kinase 3 [Source:HGNC Symbol;Acc:HGNC:3765] | 30863 | -0.079 | -0.3486 | No |
| 213 | TP53 | tumor protein p53 [Source:HGNC Symbol;Acc:HGNC:11998] | 30908 | -0.079 | -0.3475 | No |
| 214 | FGF22 | fibroblast growth factor 22 [Source:HGNC Symbol;Acc:HGNC:3679] | 31031 | -0.081 | -0.3484 | No |
| 215 | NFKBIA | NFKB inhibitor alpha [Source:HGNC Symbol;Acc:HGNC:7797] | 31181 | -0.082 | -0.3500 | No |
| 216 | FZD3 | frizzled class receptor 3 [Source:HGNC Symbol;Acc:HGNC:4041] | 31252 | -0.083 | -0.3495 | No |
| 217 | PPARG | peroxisome proliferator activated receptor gamma [Source:HGNC Symbol;Acc:HGNC:9236] | 31491 | -0.085 | -0.3532 | No |
| 218 | DVL2 | dishevelled segment polarity protein 2 [Source:HGNC Symbol;Acc:HGNC:3086] | 31825 | -0.089 | -0.3593 | No |
| 219 | PIAS2 | protein inhibitor of activated STAT 2 [Source:HGNC Symbol;Acc:HGNC:17311] | 31998 | -0.091 | -0.3612 | Yes |
| 220 | MAP2K1 | mitogen-activated protein kinase kinase 1 [Source:HGNC Symbol;Acc:HGNC:6840] | 32048 | -0.091 | -0.3599 | Yes |
| 221 | EGFR | epidermal growth factor receptor [Source:HGNC Symbol;Acc:HGNC:3236] | 32133 | -0.092 | -0.3595 | Yes |
| 222 | PDGFRB | platelet derived growth factor receptor beta [Source:HGNC Symbol;Acc:HGNC:8804] | 32165 | -0.093 | -0.3577 | Yes |
| 223 | MMP9 | matrix metallopeptidase 9 [Source:HGNC Symbol;Acc:HGNC:7176] | 32213 | -0.093 | -0.3563 | Yes |
| 224 | WNT5B | Wnt family member 5B [Source:HGNC Symbol;Acc:HGNC:16265] | 32250 | -0.094 | -0.3546 | Yes |
| 225 | FGF3 | fibroblast growth factor 3 [Source:HGNC Symbol;Acc:HGNC:3681] | 32274 | -0.094 | -0.3526 | Yes |
| 226 | GRB2 | growth factor receptor bound protein 2 [Source:HGNC Symbol;Acc:HGNC:4566] | 32433 | -0.096 | -0.3540 | Yes |
| 227 | EGLN1 | egl-9 family hypoxia inducible factor 1 [Source:HGNC Symbol;Acc:HGNC:1232] | 32449 | -0.096 | -0.3517 | Yes |
| 228 | WNT2 | Wnt family member 2 [Source:HGNC Symbol;Acc:HGNC:12780] | 32471 | -0.096 | -0.3496 | Yes |
| 229 | LAMA4 | laminin subunit alpha 4 [Source:HGNC Symbol;Acc:HGNC:6484] | 32544 | -0.097 | -0.3487 | Yes |
| 230 | VHL | von Hippel-Lindau tumor suppressor [Source:HGNC Symbol;Acc:HGNC:12687] | 32564 | -0.097 | -0.3465 | Yes |
| 231 | SMAD4 | SMAD family member 4 [Source:HGNC Symbol;Acc:HGNC:6770] | 32731 | -0.100 | -0.3480 | Yes |
| 232 | PIAS4 | protein inhibitor of activated STAT 4 [Source:HGNC Symbol;Acc:HGNC:17002] | 32751 | -0.100 | -0.3457 | Yes |
| 233 | HIF1A | hypoxia inducible factor 1 subunit alpha [Source:HGNC Symbol;Acc:HGNC:4910] | 32759 | -0.100 | -0.3431 | Yes |
| 234 | AXIN2 | axin 2 [Source:HGNC Symbol;Acc:HGNC:904] | 32772 | -0.100 | -0.3406 | Yes |
| 235 | CASP3 | caspase 3 [Source:HGNC Symbol;Acc:HGNC:1504] | 32939 | -0.103 | -0.3421 | Yes |
| 236 | STAT3 | signal transducer and activator of transcription 3 [Source:HGNC Symbol;Acc:HGNC:11364] | 32957 | -0.103 | -0.3396 | Yes |
| 237 | WNT8B | Wnt family member 8B [Source:HGNC Symbol;Acc:HGNC:12789] | 33128 | -0.105 | -0.3411 | Yes |
| 238 | PIK3CG | "phosphatidylinositol-4,5-bisphosphate 3-kinase catalytic subunit gamma [Source:HGNC Symbol;Acc:HGNC:8978]" | 33133 | -0.105 | -0.3383 | Yes |
| 239 | ITGAV | integrin subunit alpha V [Source:HGNC Symbol;Acc:HGNC:6150] | 33146 | -0.105 | -0.3356 | Yes |
| 240 | MAPK1 | mitogen-activated protein kinase 1 [Source:HGNC Symbol;Acc:HGNC:6871] | 33350 | -0.108 | -0.3378 | Yes |
| 241 | BRAF | "B-Raf proto-oncogene, serine/threonine kinase [Source:HGNC Symbol;Acc:HGNC:1097]" | 33362 | -0.108 | -0.3351 | Yes |
| 242 | MAX | MYC associated factor X [Source:HGNC Symbol;Acc:HGNC:6913] | 33370 | -0.109 | -0.3323 | Yes |
| 243 | CRK | "CRK proto-oncogene, adaptor protein [Source:HGNC Symbol;Acc:HGNC:2362]" | 33404 | -0.109 | -0.3301 | Yes |
| 244 | IGF1 | insulin like growth factor 1 [Source:HGNC Symbol;Acc:HGNC:5464] | 33450 | -0.110 | -0.3282 | Yes |
| 245 | JUP | junction plakoglobin [Source:HGNC Symbol;Acc:HGNC:6207] | 33566 | -0.111 | -0.3281 | Yes |
| 246 | PIAS1 | protein inhibitor of activated STAT 1 [Source:HGNC Symbol;Acc:HGNC:2752] | 33983 | -0.118 | -0.3355 | Yes |
| 247 | CDH1 | cadherin 1 [Source:HGNC Symbol;Acc:HGNC:1748] | 34025 | -0.118 | -0.3332 | Yes |
| 248 | TPR | "translocated promoter region, nuclear basket protein [Source:HGNC Symbol;Acc:HGNC:12017]" | 34094 | -0.119 | -0.3317 | Yes |
| 249 | CRKL | "CRK like proto-oncogene, adaptor protein [Source:HGNC Symbol;Acc:HGNC:2363]" | 34209 | -0.121 | -0.3312 | Yes |
| 250 | ITGA6 | integrin subunit alpha 6 [Source:HGNC Symbol;Acc:HGNC:6142] | 34278 | -0.122 | -0.3296 | Yes |
| 251 | PRKCB | protein kinase C beta [Source:HGNC Symbol;Acc:HGNC:9395] | 34603 | -0.128 | -0.3344 | Yes |
| 252 | FZD2 | frizzled class receptor 2 [Source:HGNC Symbol;Acc:HGNC:4040] | 34628 | -0.128 | -0.3314 | Yes |
| 253 | KITLG | KIT ligand [Source:HGNC Symbol;Acc:HGNC:6343] | 34776 | -0.131 | -0.3316 | Yes |
| 254 | SMAD2 | SMAD family member 2 [Source:HGNC Symbol;Acc:HGNC:6768] | 34897 | -0.133 | -0.3309 | Yes |
| 255 | FZD6 | frizzled class receptor 6 [Source:HGNC Symbol;Acc:HGNC:4044] | 35028 | -0.136 | -0.3305 | Yes |
| 256 | COL4A6 | collagen type IV alpha 6 chain [Source:HGNC Symbol;Acc:HGNC:2208] | 35078 | -0.137 | -0.3279 | Yes |
| 257 | ITGA2 | integrin subunit alpha 2 [Source:HGNC Symbol;Acc:HGNC:6137] | 35118 | -0.138 | -0.3251 | Yes |
| 258 | PRKCA | protein kinase C alpha [Source:HGNC Symbol;Acc:HGNC:9393] | 35223 | -0.140 | -0.3239 | Yes |
| 259 | FGF16 | fibroblast growth factor 16 [Source:HGNC Symbol;Acc:HGNC:3672] | 35237 | -0.140 | -0.3203 | Yes |
| 260 | RASSF5 | Ras association domain family member 5 [Source:HGNC Symbol;Acc:HGNC:17609] | 35248 | -0.141 | -0.3167 | Yes |
| 261 | XIAP | X-linked inhibitor of apoptosis [Source:HGNC Symbol;Acc:HGNC:592] | 35357 | -0.143 | -0.3155 | Yes |
| 262 | ARNT2 | aryl hydrocarbon receptor nuclear translocator 2 [Source:HGNC Symbol;Acc:HGNC:16876] | 35358 | -0.143 | -0.3115 | Yes |
| 263 | SMAD3 | SMAD family member 3 [Source:HGNC Symbol;Acc:HGNC:6769] | 35374 | -0.144 | -0.3079 | Yes |
| 264 | FGF17 | fibroblast growth factor 17 [Source:HGNC Symbol;Acc:HGNC:3673] | 35557 | -0.148 | -0.3084 | Yes |
| 265 | PLCG2 | phospholipase C gamma 2 [Source:HGNC Symbol;Acc:HGNC:9066] | 35650 | -0.151 | -0.3066 | Yes |
| 266 | PTCH2 | patched 2 [Source:HGNC Symbol;Acc:HGNC:9586] | 35779 | -0.154 | -0.3056 | Yes |
| 267 | RXRG | retinoid X receptor gamma [Source:HGNC Symbol;Acc:HGNC:10479] | 35786 | -0.154 | -0.3015 | Yes |
| 268 | CTNNB1 | catenin beta 1 [Source:HGNC Symbol;Acc:HGNC:2514] | 35880 | -0.157 | -0.2995 | Yes |
| 269 | WNT9B | Wnt family member 9B [Source:HGNC Symbol;Acc:HGNC:12779] | 35896 | -0.157 | -0.2955 | Yes |
| 270 | BCL2 | BCL2 apoptosis regulator [Source:HGNC Symbol;Acc:HGNC:990] | 35981 | -0.160 | -0.2932 | Yes |
| 271 | COL4A2 | collagen type IV alpha 2 chain [Source:HGNC Symbol;Acc:HGNC:2203] | 36002 | -0.160 | -0.2893 | Yes |
| 272 | FGF12 | fibroblast growth factor 12 [Source:HGNC Symbol;Acc:HGNC:3668] | 36028 | -0.161 | -0.2855 | Yes |
| 273 | WNT2B | Wnt family member 2B [Source:HGNC Symbol;Acc:HGNC:12781] | 36087 | -0.163 | -0.2824 | Yes |
| 274 | IGF1R | insulin like growth factor 1 receptor [Source:HGNC Symbol;Acc:HGNC:5465] | 36113 | -0.163 | -0.2785 | Yes |
| 275 | FOS | "Fos proto-oncogene, AP-1 transcription factor subunit [Source:HGNC Symbol;Acc:HGNC:3796]" | 36141 | -0.164 | -0.2747 | Yes |
| 276 | VEGFD | vascular endothelial growth factor D [Source:HGNC Symbol;Acc:HGNC:3708] | 36152 | -0.165 | -0.2703 | Yes |
| 277 | LAMA1 | laminin subunit alpha 1 [Source:HGNC Symbol;Acc:HGNC:6481] | 36168 | -0.166 | -0.2661 | Yes |
| 278 | MAPK10 | mitogen-activated protein kinase 10 [Source:HGNC Symbol;Acc:HGNC:6872] | 36201 | -0.166 | -0.2623 | Yes |
| 279 | DAPK1 | death associated protein kinase 1 [Source:HGNC Symbol;Acc:HGNC:2674] | 36287 | -0.169 | -0.2598 | Yes |
| 280 | EP300 | E1A binding protein p300 [Source:HGNC Symbol;Acc:HGNC:3373] | 36409 | -0.173 | -0.2581 | Yes |
| 281 | SOS1 | SOS Ras/Rac guanine nucleotide exchange factor 1 [Source:HGNC Symbol;Acc:HGNC:11187] | 36506 | -0.177 | -0.2557 | Yes |
| 282 | FGF14 | fibroblast growth factor 14 [Source:HGNC Symbol;Acc:HGNC:3671] | 36577 | -0.179 | -0.2525 | Yes |
| 283 | SHH | sonic hedgehog signaling molecule [Source:HGNC Symbol;Acc:HGNC:10848] | 36592 | -0.179 | -0.2479 | Yes |
| 284 | MSH6 | mutS homolog 6 [Source:HGNC Symbol;Acc:HGNC:7329] | 36629 | -0.181 | -0.2437 | Yes |
| 285 | LAMC1 | laminin subunit gamma 1 [Source:HGNC Symbol;Acc:HGNC:6492] | 36646 | -0.182 | -0.2391 | Yes |
| 286 | CCNA1 | cyclin A1 [Source:HGNC Symbol;Acc:HGNC:1577] | 36697 | -0.184 | -0.2353 | Yes |
| 287 | COL4A1 | collagen type IV alpha 1 chain [Source:HGNC Symbol;Acc:HGNC:2202] | 36874 | -0.192 | -0.2345 | Yes |
| 288 | KRAS | "KRAS proto-oncogene, GTPase [Source:HGNC Symbol;Acc:HGNC:6407]" | 36928 | -0.194 | -0.2304 | Yes |
| 289 | FGF8 | fibroblast growth factor 8 [Source:HGNC Symbol;Acc:HGNC:3686] | 37023 | -0.198 | -0.2273 | Yes |
| 290 | APPL1 | "adaptor protein, phosphotyrosine interacting with PH domain and leucine zipper 1 [Source:HGNC Symbol;Acc:HGNC:24035]" | 37103 | -0.203 | -0.2237 | Yes |
| 291 | RB1 | RB transcriptional corepressor 1 [Source:HGNC Symbol;Acc:HGNC:9884] | 37254 | -0.210 | -0.2218 | Yes |
| 292 | IKBKG | inhibitor of nuclear factor kappa B kinase regulatory subunit gamma [Source:HGNC Symbol;Acc:HGNC:5961] | 37365 | -0.216 | -0.2186 | Yes |
| 293 | IL6 | interleukin 6 [Source:HGNC Symbol;Acc:HGNC:6018] | 37409 | -0.218 | -0.2136 | Yes |
| 294 | CBL | Cbl proto-oncogene [Source:HGNC Symbol;Acc:HGNC:1541] | 37421 | -0.218 | -0.2079 | Yes |
| 295 | CREBBP | CREB binding protein [Source:HGNC Symbol;Acc:HGNC:2348] | 37476 | -0.222 | -0.2031 | Yes |
| 296 | FGFR2 | fibroblast growth factor receptor 2 [Source:HGNC Symbol;Acc:HGNC:3689] | 37585 | -0.230 | -0.1995 | Yes |
| 297 | RALGDS | ral guanine nucleotide dissociation stimulator [Source:HGNC Symbol;Acc:HGNC:9842] | 37589 | -0.231 | -0.1931 | Yes |
| 298 | EGF | epidermal growth factor [Source:HGNC Symbol;Acc:HGNC:3229] | 37597 | -0.231 | -0.1869 | Yes |
| 299 | FGF13 | fibroblast growth factor 13 [Source:HGNC Symbol;Acc:HGNC:3670] | 37611 | -0.232 | -0.1808 | Yes |
| 300 | WNT11 | Wnt family member 11 [Source:HGNC Symbol;Acc:HGNC:12776] | 37666 | -0.236 | -0.1757 | Yes |
| 301 | FGF19 | fibroblast growth factor 19 [Source:HGNC Symbol;Acc:HGNC:3675] | 37781 | -0.243 | -0.1718 | Yes |
| 302 | TRAF1 | TNF receptor associated factor 1 [Source:HGNC Symbol;Acc:HGNC:12031] | 37843 | -0.248 | -0.1665 | Yes |
| 303 | TGFBR1 | transforming growth factor beta receptor 1 [Source:HGNC Symbol;Acc:HGNC:11772] | 37905 | -0.255 | -0.1610 | Yes |
| 304 | ABL1 | "ABL proto-oncogene 1, non-receptor tyrosine kinase [Source:HGNC Symbol;Acc:HGNC:76]" | 37911 | -0.255 | -0.1540 | Yes |
| 305 | DVL3 | dishevelled segment polarity protein 3 [Source:HGNC Symbol;Acc:HGNC:3087] | 37983 | -0.264 | -0.1485 | Yes |
| 306 | FOXO1 | forkhead box O1 [Source:HGNC Symbol;Acc:HGNC:3819] | 37985 | -0.264 | -0.1412 | Yes |
| 307 | WNT3 | Wnt family member 3 [Source:HGNC Symbol;Acc:HGNC:12782] | 38054 | -0.271 | -0.1354 | Yes |
| 308 | TRAF6 | TNF receptor associated factor 6 [Source:HGNC Symbol;Acc:HGNC:12036] | 38104 | -0.278 | -0.1290 | Yes |
| 309 | SMO | "smoothened, frizzled class receptor [Source:HGNC Symbol;Acc:HGNC:11119]" | 38185 | -0.291 | -0.1229 | Yes |
| 310 | FGF2 | fibroblast growth factor 2 [Source:HGNC Symbol;Acc:HGNC:3676] | 38191 | -0.292 | -0.1150 | Yes |
| 311 | STAT5B | signal transducer and activator of transcription 5B [Source:HGNC Symbol;Acc:HGNC:11367] | 38215 | -0.294 | -0.1074 | Yes |
| 312 | PIK3CA | "phosphatidylinositol-4,5-bisphosphate 3-kinase catalytic subunit alpha [Source:HGNC Symbol;Acc:HGNC:8975]" | 38226 | -0.296 | -0.0994 | Yes |
| 313 | PLD1 | phospholipase D1 [Source:HGNC Symbol;Acc:HGNC:9067] | 38276 | -0.304 | -0.0922 | Yes |
| 314 | LEF1 | lymphoid enhancer binding factor 1 [Source:HGNC Symbol;Acc:HGNC:6551] | 38341 | -0.316 | -0.0851 | Yes |
| 315 | AKT3 | AKT serine/threonine kinase 3 [Source:HGNC Symbol;Acc:HGNC:393] | 38358 | -0.320 | -0.0767 | Yes |
| 316 | FZD10 | frizzled class receptor 10 [Source:HGNC Symbol;Acc:HGNC:4039] | 38368 | -0.322 | -0.0679 | Yes |
| 317 | BRCA2 | BRCA2 DNA repair associated [Source:HGNC Symbol;Acc:HGNC:1101] | 38385 | -0.326 | -0.0593 | Yes |
| 318 | APC | APC regulator of WNT signaling pathway [Source:HGNC Symbol;Acc:HGNC:583] | 38413 | -0.336 | -0.0507 | Yes |
| 319 | RALB | RAS like proto-oncogene B [Source:HGNC Symbol;Acc:HGNC:9840] | 38448 | -0.348 | -0.0419 | Yes |
| 320 | DCC | DCC netrin 1 receptor [Source:HGNC Symbol;Acc:HGNC:2701] | 38466 | -0.356 | -0.0325 | Yes |
| 321 | FZD7 | frizzled class receptor 7 [Source:HGNC Symbol;Acc:HGNC:4045] | 38485 | -0.367 | -0.0227 | Yes |
| 322 | WNT5A | Wnt family member 5A [Source:HGNC Symbol;Acc:HGNC:12784] | 38488 | -0.368 | -0.0126 | Yes |
| 323 | GLI1 | GLI family zinc finger 1 [Source:HGNC Symbol;Acc:HGNC:4317] | 38507 | -0.378 | -0.0026 | Yes |
| 324 | PTCH1 | patched 1 [Source:HGNC Symbol;Acc:HGNC:9585] | 38519 | -0.389 | 0.0080 | Yes |
| 325 | GLI2 | GLI family zinc finger 2 [Source:HGNC Symbol;Acc:HGNC:4318] | 38549 | -0.455 | 0.0198 | Yes |
Table: GSEA details [plain text format]

  

Fig 2: KEGG\_PATHWAYS\_IN\_CANCER      
 Blue-Pink O' Gram in the Space of the Analyzed GeneSet

  

Fig 3: KEGG\_PATHWAYS\_IN\_CANCER: Random ES distribution      
 Gene set null distribution of ES for **KEGG\_PATHWAYS\_IN\_CANCER**
