# Supplementary material for: Simultaneous Presentation of Multiple Myeloma and Lung Cancer: Case Report and Gene Bioinformatics Analysis
Source: Front Oncol. 2022 Jun 13;12:859735. doi: 10.3389/fonc.2022.859735 (PMC9235397; doi:10.3389/fonc.2022.859735)
Supplement: Supplementary file 1 [file DataSheet_1.zip › The bioinformatic analysis of MM and lung cancer supplementary materials/Enrichment analysis/MECR/GSEA_4.1.0/LUSC TCGA/KEGG.Gsea.1639042374131/KEGG_PEROXISOME.html]

Details for gene set KEGG\_PEROXISOME[GSEA]

|  || Dataset | ExpData\_collapsed\_to\_symbols.ENSG00000116353\_profile\_in\_ExpData.cls #ENSG00000116353 |
| Phenotype | ENSG00000116353\_profile\_in\_ExpData.cls#ENSG00000116353 |
| Upregulated in class | ENSG00000116353\_pos |
| GeneSet | KEGG\_PEROXISOME |
| Enrichment Score (ES) | 0.55686176 |
| Normalized Enrichment Score (NES) | 2.2653668 |
| Nominal p-value | 0.0 |
| FDR q-value | 0.0 |
| FWER p-Value | 0.0 |
Table: GSEA Results Summary

  

Fig 1: Enrichment plot: KEGG\_PEROXISOME      
 Profile of the Running ES Score & Positions of GeneSet Members on the Rank Ordered List

  

| SYMBOL | TITLE | RANK IN GENE LIST | RANK METRIC SCORE | RUNNING ES | CORE ENRICHMENT || 1 | HMGCL | 3-hydroxy-3-methylglutaryl-CoA lyase [Source:HGNC Symbol;Acc:HGNC:5005] | 18 | 0.508 | 0.0464 | Yes |
| 2 | PEX14 | peroxisomal biogenesis factor 14 [Source:HGNC Symbol;Acc:HGNC:8856] | 32 | 0.477 | 0.0899 | Yes |
| 3 | PEX10 | peroxisomal biogenesis factor 10 [Source:HGNC Symbol;Acc:HGNC:8851] | 64 | 0.430 | 0.1287 | Yes |
| 4 | ACOT8 | acyl-CoA thioesterase 8 [Source:HGNC Symbol;Acc:HGNC:15919] | 156 | 0.357 | 0.1593 | Yes |
| 5 | SCP2 | sterol carrier protein 2 [Source:HGNC Symbol;Acc:HGNC:10606] | 308 | 0.305 | 0.1835 | Yes |
| 6 | MPV17 | mitochondrial inner membrane protein MPV17 [Source:HGNC Symbol;Acc:HGNC:7224] | 314 | 0.304 | 0.2114 | Yes |
| 7 | ACOX3 | "acyl-CoA oxidase 3, pristanoyl [Source:HGNC Symbol;Acc:HGNC:121]" | 331 | 0.300 | 0.2386 | Yes |
| 8 | PRDX5 | peroxiredoxin 5 [Source:HGNC Symbol;Acc:HGNC:9355] | 345 | 0.296 | 0.2655 | Yes |
| 9 | PXMP2 | peroxisomal membrane protein 2 [Source:HGNC Symbol;Acc:HGNC:9716] | 389 | 0.287 | 0.2909 | Yes |
| 10 | AMACR | alpha-methylacyl-CoA racemase [Source:HGNC Symbol;Acc:HGNC:451] | 612 | 0.255 | 0.3088 | Yes |
| 11 | GSTK1 | glutathione S-transferase kappa 1 [Source:HGNC Symbol;Acc:HGNC:16906] | 626 | 0.254 | 0.3318 | Yes |
| 12 | ABCD3 | ATP binding cassette subfamily D member 3 [Source:HGNC Symbol;Acc:HGNC:67] | 636 | 0.253 | 0.3549 | Yes |
| 13 | PEX3 | peroxisomal biogenesis factor 3 [Source:HGNC Symbol;Acc:HGNC:8858] | 641 | 0.252 | 0.3780 | Yes |
| 14 | DECR2 | "2,4-dienoyl-CoA reductase 2 [Source:HGNC Symbol;Acc:HGNC:2754]" | 684 | 0.247 | 0.3996 | Yes |
| 15 | PEX2 | peroxisomal biogenesis factor 2 [Source:HGNC Symbol;Acc:HGNC:9717] | 726 | 0.242 | 0.4208 | Yes |
| 16 | MVK | mevalonate kinase [Source:HGNC Symbol;Acc:HGNC:7530] | 946 | 0.219 | 0.4354 | Yes |
| 17 | PMVK | phosphomevalonate kinase [Source:HGNC Symbol;Acc:HGNC:9141] | 983 | 0.216 | 0.4543 | Yes |
| 18 | IDH2 | isocitrate dehydrogenase (NADP(+)) 2 [Source:HGNC Symbol;Acc:HGNC:5383] | 1041 | 0.212 | 0.4724 | Yes |
| 19 | ECH1 | enoyl-CoA hydratase 1 [Source:HGNC Symbol;Acc:HGNC:3149] | 1140 | 0.206 | 0.4889 | Yes |
| 20 | PEX16 | peroxisomal biogenesis factor 16 [Source:HGNC Symbol;Acc:HGNC:8857] | 1590 | 0.182 | 0.4942 | Yes |
| 21 | ABCD4 | ATP binding cassette subfamily D member 4 [Source:HGNC Symbol;Acc:HGNC:68] | 1665 | 0.180 | 0.5089 | Yes |
| 22 | SLC25A17 | solute carrier family 25 member 17 [Source:HGNC Symbol;Acc:HGNC:10987] | 1864 | 0.171 | 0.5196 | Yes |
| 23 | PEX7 | peroxisomal biogenesis factor 7 [Source:HGNC Symbol;Acc:HGNC:8860] | 1920 | 0.169 | 0.5337 | Yes |
| 24 | AGXT | alanine--glyoxylate and serine--pyruvate aminotransferase [Source:HGNC Symbol;Acc:HGNC:341] | 2525 | 0.149 | 0.5321 | Yes |
| 25 | PEX13 | peroxisomal biogenesis factor 13 [Source:HGNC Symbol;Acc:HGNC:8855] | 2616 | 0.147 | 0.5433 | Yes |
| 26 | CROT | carnitine O-octanoyltransferase [Source:HGNC Symbol;Acc:HGNC:2366] | 3029 | 0.136 | 0.5453 | Yes |
| 27 | HAO2 | hydroxyacid oxidase 2 [Source:HGNC Symbol;Acc:HGNC:4810] | 3063 | 0.135 | 0.5569 | Yes |
| 28 | PEX11G | peroxisomal biogenesis factor 11 gamma [Source:HGNC Symbol;Acc:HGNC:20208] | 4228 | 0.114 | 0.5377 | No |
| 29 | PEX11A | peroxisomal biogenesis factor 11 alpha [Source:HGNC Symbol;Acc:HGNC:8852] | 4356 | 0.112 | 0.5448 | No |
| 30 | XDH | xanthine dehydrogenase [Source:HGNC Symbol;Acc:HGNC:12805] | 4454 | 0.110 | 0.5525 | No |
| 31 | PEX11B | peroxisomal biogenesis factor 11 beta [Source:HGNC Symbol;Acc:HGNC:8853] | 6232 | 0.091 | 0.5156 | No |
| 32 | ACSL5 | acyl-CoA synthetase long chain family member 5 [Source:HGNC Symbol;Acc:HGNC:16526] | 6915 | 0.084 | 0.5060 | No |
| 33 | PEX19 | peroxisomal biogenesis factor 19 [Source:HGNC Symbol;Acc:HGNC:9713] | 7810 | 0.076 | 0.4903 | No |
| 34 | ACAA1 | acetyl-CoA acyltransferase 1 [Source:HGNC Symbol;Acc:HGNC:82] | 8047 | 0.075 | 0.4911 | No |
| 35 | ACOX2 | acyl-CoA oxidase 2 [Source:HGNC Symbol;Acc:HGNC:120] | 8269 | 0.073 | 0.4921 | No |
| 36 | PXMP4 | peroxisomal membrane protein 4 [Source:HGNC Symbol;Acc:HGNC:15920] | 9175 | 0.066 | 0.4752 | No |
| 37 | EPHX2 | epoxide hydrolase 2 [Source:HGNC Symbol;Acc:HGNC:3402] | 9532 | 0.064 | 0.4720 | No |
| 38 | PHYH | phytanoyl-CoA 2-hydroxylase [Source:HGNC Symbol;Acc:HGNC:8940] | 10099 | 0.059 | 0.4630 | No |
| 39 | MPV17L | MPV17 mitochondrial inner membrane protein like [Source:HGNC Symbol;Acc:HGNC:26827] | 11624 | 0.050 | 0.4287 | No |
| 40 | ACOX1 | acyl-CoA oxidase 1 [Source:HGNC Symbol;Acc:HGNC:119] | 11899 | 0.048 | 0.4262 | No |
| 41 | NUDT12 | nudix hydrolase 12 [Source:HGNC Symbol;Acc:HGNC:18826] | 12453 | 0.044 | 0.4162 | No |
| 42 | GNPAT | glyceronephosphate O-acyltransferase [Source:HGNC Symbol;Acc:HGNC:4416] | 14659 | 0.031 | 0.3628 | No |
| 43 | HAO1 | hydroxyacid oxidase 1 [Source:HGNC Symbol;Acc:HGNC:4809] | 15295 | 0.028 | 0.3492 | No |
| 44 | HSD17B4 | hydroxysteroid 17-beta dehydrogenase 4 [Source:HGNC Symbol;Acc:HGNC:5213] | 16072 | 0.023 | 0.3315 | No |
| 45 | ACSL3 | acyl-CoA synthetase long chain family member 3 [Source:HGNC Symbol;Acc:HGNC:3570] | 16191 | 0.022 | 0.3306 | No |
| 46 | DDO | D-aspartate oxidase [Source:HGNC Symbol;Acc:HGNC:2727] | 16400 | 0.021 | 0.3272 | No |
| 47 | PEX6 | peroxisomal biogenesis factor 6 [Source:HGNC Symbol;Acc:HGNC:8859] | 18061 | 0.012 | 0.2860 | No |
| 48 | PRDX1 | peroxiredoxin 1 [Source:HGNC Symbol;Acc:HGNC:9352] | 19714 | 0.002 | 0.2441 | No |
| 49 | SOD1 | superoxide dismutase 1 [Source:HGNC Symbol;Acc:HGNC:11179] | 22374 | -0.014 | 0.1776 | No |
| 50 | NOS2 | nitric oxide synthase 2 [Source:HGNC Symbol;Acc:HGNC:7873] | 22937 | -0.017 | 0.1649 | No |
| 51 | PAOX | polyamine oxidase [Source:HGNC Symbol;Acc:HGNC:20837] | 23070 | -0.018 | 0.1631 | No |
| 52 | PEX26 | peroxisomal biogenesis factor 26 [Source:HGNC Symbol;Acc:HGNC:22965] | 23267 | -0.019 | 0.1599 | No |
| 53 | PEX1 | peroxisomal biogenesis factor 1 [Source:HGNC Symbol;Acc:HGNC:8850] | 23934 | -0.023 | 0.1451 | No |
| 54 | CAT | catalase [Source:HGNC Symbol;Acc:HGNC:1516] | 24549 | -0.028 | 0.1320 | No |
| 55 | ACSL4 | acyl-CoA synthetase long chain family member 4 [Source:HGNC Symbol;Acc:HGNC:3571] | 24666 | -0.029 | 0.1317 | No |
| 56 | ECI2 | enoyl-CoA delta isomerase 2 [Source:HGNC Symbol;Acc:HGNC:14601] | 25126 | -0.032 | 0.1229 | No |
| 57 | ABCD1 | ATP binding cassette subfamily D member 1 [Source:HGNC Symbol;Acc:HGNC:61] | 25280 | -0.033 | 0.1221 | No |
| 58 | BAAT | bile acid-CoA:amino acid N-acyltransferase [Source:HGNC Symbol;Acc:HGNC:932] | 25471 | -0.034 | 0.1204 | No |
| 59 | AGPS | alkylglycerone phosphate synthase [Source:HGNC Symbol;Acc:HGNC:327] | 26379 | -0.041 | 0.1011 | No |
| 60 | FAR1 | fatty acyl-CoA reductase 1 [Source:HGNC Symbol;Acc:HGNC:26222] | 26738 | -0.044 | 0.0960 | No |
| 61 | FAR2 | fatty acyl-CoA reductase 2 [Source:HGNC Symbol;Acc:HGNC:25531] | 27837 | -0.052 | 0.0728 | No |
| 62 | NUDT19 | nudix hydrolase 19 [Source:HGNC Symbol;Acc:HGNC:32036] | 29530 | -0.066 | 0.0358 | No |
| 63 | EHHADH | enoyl-CoA hydratase and 3-hydroxyacyl CoA dehydrogenase [Source:HGNC Symbol;Acc:HGNC:3247] | 29653 | -0.067 | 0.0389 | No |
| 64 | DHRS4 | dehydrogenase/reductase 4 [Source:HGNC Symbol;Acc:HGNC:16985] | 30242 | -0.073 | 0.0306 | No |
| 65 | PEX5 | peroxisomal biogenesis factor 5 [Source:HGNC Symbol;Acc:HGNC:9719] | 31009 | -0.080 | 0.0185 | No |
| 66 | MLYCD | malonyl-CoA decarboxylase [Source:HGNC Symbol;Acc:HGNC:7150] | 31850 | -0.089 | 0.0053 | No |
| 67 | DAO | D-amino acid oxidase [Source:HGNC Symbol;Acc:HGNC:2671] | 32045 | -0.091 | 0.0088 | No |
| 68 | HACL1 | 2-hydroxyacyl-CoA lyase 1 [Source:HGNC Symbol;Acc:HGNC:17856] | 33143 | -0.105 | -0.0095 | No |
| 69 | PECR | peroxisomal trans-2-enoyl-CoA reductase [Source:HGNC Symbol;Acc:HGNC:18281] | 34969 | -0.135 | -0.0436 | No |
| 70 | SOD2 | superoxide dismutase 2 [Source:HGNC Symbol;Acc:HGNC:11180] | 35681 | -0.152 | -0.0477 | No |
| 71 | ACSL1 | acyl-CoA synthetase long chain family member 1 [Source:HGNC Symbol;Acc:HGNC:3569] | 36148 | -0.165 | -0.0445 | No |
| 72 | ABCD2 | ATP binding cassette subfamily D member 2 [Source:HGNC Symbol;Acc:HGNC:66] | 36224 | -0.167 | -0.0310 | No |
| 73 | PIPOX | pipecolic acid and sarcosine oxidase [Source:HGNC Symbol;Acc:HGNC:17804] | 36295 | -0.170 | -0.0171 | No |
| 74 | CRAT | carnitine O-acetyltransferase [Source:HGNC Symbol;Acc:HGNC:2342] | 36718 | -0.185 | -0.0108 | No |
| 75 | IDH1 | isocitrate dehydrogenase (NADP(+)) 1 [Source:HGNC Symbol;Acc:HGNC:5382] | 37020 | -0.198 | -0.0002 | No |
| 76 | ACSL6 | acyl-CoA synthetase long chain family member 6 [Source:HGNC Symbol;Acc:HGNC:16496] | 37189 | -0.207 | 0.0145 | No |
| 77 | SLC27A2 | solute carrier family 27 member 2 [Source:HGNC Symbol;Acc:HGNC:10996] | 37328 | -0.214 | 0.0307 | No |
| 78 | PEX12 | peroxisomal biogenesis factor 12 [Source:HGNC Symbol;Acc:HGNC:8854] | 37429 | -0.219 | 0.0483 | No |
Table: GSEA details [plain text format]

  

Fig 2: KEGG\_PEROXISOME      
 Blue-Pink O' Gram in the Space of the Analyzed GeneSet

  

Fig 3: KEGG\_PEROXISOME: Random ES distribution      
 Gene set null distribution of ES for **KEGG\_PEROXISOME**
