# Supplementary material for: Simultaneous Presentation of Multiple Myeloma and Lung Cancer: Case Report and Gene Bioinformatics Analysis
Source: Front Oncol. 2022 Jun 13;12:859735. doi: 10.3389/fonc.2022.859735 (PMC9235397; doi:10.3389/fonc.2022.859735)
Supplement: Supplementary file 1 [file DataSheet_1.zip › The bioinformatic analysis of MM and lung cancer supplementary materials/Enrichment analysis/MECR/GSEA_4.1.0/LUSC TCGA/KEGG.Gsea.1639042374131/KEGG_PHOSPHATIDYLINOSITOL_SIGNALING_SYSTEM.html]

Details for gene set KEGG\_PHOSPHATIDYLINOSITOL\_SIGNALING\_SYSTEM[GSEA]

|  || Dataset | ExpData\_collapsed\_to\_symbols.ENSG00000116353\_profile\_in\_ExpData.cls #ENSG00000116353 |
| Phenotype | ENSG00000116353\_profile\_in\_ExpData.cls#ENSG00000116353 |
| Upregulated in class | ENSG00000116353\_neg |
| GeneSet | KEGG\_PHOSPHATIDYLINOSITOL\_SIGNALING\_SYSTEM |
| Enrichment Score (ES) | -0.51133484 |
| Normalized Enrichment Score (NES) | -2.0887206 |
| Nominal p-value | 0.0 |
| FDR q-value | 0.001159501 |
| FWER p-Value | 0.002 |
Table: GSEA Results Summary

  

Fig 1: Enrichment plot: KEGG\_PHOSPHATIDYLINOSITOL\_SIGNALING\_SYSTEM      
 Profile of the Running ES Score & Positions of GeneSet Members on the Rank Ordered List

  

| SYMBOL | TITLE | RANK IN GENE LIST | RANK METRIC SCORE | RUNNING ES | CORE ENRICHMENT || 1 | CALM3 | calmodulin 3 [Source:HGNC Symbol;Acc:HGNC:1449] | 850 | 0.227 | 0.0018 | No |
| 2 | INPP5B | inositol polyphosphate-5-phosphatase B [Source:HGNC Symbol;Acc:HGNC:6077] | 1305 | 0.197 | 0.0107 | No |
| 3 | DGKZ | diacylglycerol kinase zeta [Source:HGNC Symbol;Acc:HGNC:2857] | 1862 | 0.171 | 0.0142 | No |
| 4 | PLCB3 | phospholipase C beta 3 [Source:HGNC Symbol;Acc:HGNC:9056] | 1971 | 0.167 | 0.0287 | No |
| 5 | PIK3R3 | phosphoinositide-3-kinase regulatory subunit 3 [Source:HGNC Symbol;Acc:HGNC:8981] | 2364 | 0.153 | 0.0345 | No |
| 6 | INPP4B | inositol polyphosphate-4-phosphatase type II B [Source:HGNC Symbol;Acc:HGNC:6075] | 3228 | 0.132 | 0.0262 | No |
| 7 | PLCD3 | phospholipase C delta 3 [Source:HGNC Symbol;Acc:HGNC:9061] | 3547 | 0.126 | 0.0311 | No |
| 8 | IMPA1 | inositol monophosphatase 1 [Source:HGNC Symbol;Acc:HGNC:6050] | 4067 | 0.116 | 0.0299 | No |
| 9 | INPP5A | inositol polyphosphate-5-phosphatase A [Source:HGNC Symbol;Acc:HGNC:6076] | 4089 | 0.116 | 0.0414 | No |
| 10 | INPP1 | inositol polyphosphate-1-phosphatase [Source:HGNC Symbol;Acc:HGNC:6071] | 4119 | 0.116 | 0.0526 | No |
| 11 | CALM2 | calmodulin 2 [Source:HGNC Symbol;Acc:HGNC:1445] | 6292 | 0.091 | 0.0067 | No |
| 12 | CALML5 | calmodulin like 5 [Source:HGNC Symbol;Acc:HGNC:18180] | 6619 | 0.087 | 0.0074 | No |
| 13 | CDIPT | CDP-diacylglycerol--inositol 3-phosphatidyltransferase [Source:HGNC Symbol;Acc:HGNC:1769] | 7957 | 0.075 | -0.0189 | No |
| 14 | SYNJ2 | synaptojanin 2 [Source:HGNC Symbol;Acc:HGNC:11504] | 8064 | 0.074 | -0.0139 | No |
| 15 | PIP4K2C | phosphatidylinositol-5-phosphate 4-kinase type 2 gamma [Source:HGNC Symbol;Acc:HGNC:23786] | 9671 | 0.063 | -0.0483 | No |
| 16 | PTEN | phosphatase and tensin homolog [Source:HGNC Symbol;Acc:HGNC:9588] | 9897 | 0.061 | -0.0478 | No |
| 17 | CDS1 | CDP-diacylglycerol synthase 1 [Source:HGNC Symbol;Acc:HGNC:1800] | 11640 | 0.049 | -0.0871 | No |
| 18 | PLCG1 | phospholipase C gamma 1 [Source:HGNC Symbol;Acc:HGNC:9065] | 13655 | 0.037 | -0.1345 | No |
| 19 | PRKCG | protein kinase C gamma [Source:HGNC Symbol;Acc:HGNC:9402] | 15351 | 0.027 | -0.1749 | No |
| 20 | PLCE1 | phospholipase C epsilon 1 [Source:HGNC Symbol;Acc:HGNC:17175] | 19244 | 0.005 | -0.2736 | No |
| 21 | PIK3CB | "phosphatidylinositol-4,5-bisphosphate 3-kinase catalytic subunit beta [Source:HGNC Symbol;Acc:HGNC:8976]" | 20368 | -0.002 | -0.3020 | No |
| 22 | CALML6 | calmodulin like 6 [Source:HGNC Symbol;Acc:HGNC:24193] | 20499 | -0.002 | -0.3051 | No |
| 23 | CALM1 | calmodulin 1 [Source:HGNC Symbol;Acc:HGNC:1442] | 21884 | -0.010 | -0.3393 | No |
| 24 | PIK3R1 | phosphoinositide-3-kinase regulatory subunit 1 [Source:HGNC Symbol;Acc:HGNC:8979] | 22505 | -0.014 | -0.3536 | No |
| 25 | ITPK1 | inositol-tetrakisphosphate 1-kinase [Source:HGNC Symbol;Acc:HGNC:6177] | 23438 | -0.020 | -0.3752 | No |
| 26 | INPPL1 | inositol polyphosphate phosphatase like 1 [Source:HGNC Symbol;Acc:HGNC:6080] | 23995 | -0.024 | -0.3869 | No |
| 27 | PIK3CD | "phosphatidylinositol-4,5-bisphosphate 3-kinase catalytic subunit delta [Source:HGNC Symbol;Acc:HGNC:8977]" | 24002 | -0.024 | -0.3846 | No |
| 28 | DGKQ | diacylglycerol kinase theta [Source:HGNC Symbol;Acc:HGNC:2856] | 24188 | -0.025 | -0.3868 | No |
| 29 | SYNJ1 | synaptojanin 1 [Source:HGNC Symbol;Acc:HGNC:11503] | 25444 | -0.034 | -0.4152 | No |
| 30 | INPP5K | inositol polyphosphate-5-phosphatase K [Source:HGNC Symbol;Acc:HGNC:33882] | 25816 | -0.037 | -0.4208 | No |
| 31 | PLCB1 | phospholipase C beta 1 [Source:HGNC Symbol;Acc:HGNC:15917] | 27781 | -0.052 | -0.4655 | No |
| 32 | INPP5J | inositol polyphosphate-5-phosphatase J [Source:HGNC Symbol;Acc:HGNC:8956] | 28392 | -0.056 | -0.4752 | No |
| 33 | IMPA2 | inositol monophosphatase 2 [Source:HGNC Symbol;Acc:HGNC:6051] | 28426 | -0.057 | -0.4702 | No |
| 34 | PI4KB | phosphatidylinositol 4-kinase beta [Source:HGNC Symbol;Acc:HGNC:8984] | 29012 | -0.062 | -0.4787 | No |
| 35 | INPP5D | inositol polyphosphate-5-phosphatase D [Source:HGNC Symbol;Acc:HGNC:6079] | 29285 | -0.064 | -0.4791 | No |
| 36 | ITPR3 | "inositol 1,4,5-trisphosphate receptor type 3 [Source:HGNC Symbol;Acc:HGNC:6182]" | 29471 | -0.066 | -0.4770 | No |
| 37 | PIK3C2G | phosphatidylinositol-4-phosphate 3-kinase catalytic subunit type 2 gamma [Source:HGNC Symbol;Acc:HGNC:8973] | 29981 | -0.071 | -0.4826 | No |
| 38 | PIK3R5 | phosphoinositide-3-kinase regulatory subunit 5 [Source:HGNC Symbol;Acc:HGNC:30035] | 30064 | -0.071 | -0.4774 | No |
| 39 | PIP5K1C | phosphatidylinositol-4-phosphate 5-kinase type 1 gamma [Source:HGNC Symbol;Acc:HGNC:8996] | 30186 | -0.072 | -0.4729 | No |
| 40 | PIK3R2 | phosphoinositide-3-kinase regulatory subunit 2 [Source:HGNC Symbol;Acc:HGNC:8980] | 30740 | -0.078 | -0.4790 | No |
| 41 | PIK3C2B | phosphatidylinositol-4-phosphate 3-kinase catalytic subunit type 2 beta [Source:HGNC Symbol;Acc:HGNC:8972] | 31042 | -0.081 | -0.4783 | No |
| 42 | PLCD4 | phospholipase C delta 4 [Source:HGNC Symbol;Acc:HGNC:9062] | 31989 | -0.091 | -0.4930 | No |
| 43 | PLCB2 | phospholipase C beta 2 [Source:HGNC Symbol;Acc:HGNC:9055] | 32369 | -0.095 | -0.4929 | No |
| 44 | PLCZ1 | phospholipase C zeta 1 [Source:HGNC Symbol;Acc:HGNC:19218] | 33095 | -0.105 | -0.5005 | Yes |
| 45 | PIK3CG | "phosphatidylinositol-4,5-bisphosphate 3-kinase catalytic subunit gamma [Source:HGNC Symbol;Acc:HGNC:8978]" | 33133 | -0.105 | -0.4906 | Yes |
| 46 | PI4KA | phosphatidylinositol 4-kinase alpha [Source:HGNC Symbol;Acc:HGNC:8983] | 33228 | -0.107 | -0.4819 | Yes |
| 47 | CALML3 | calmodulin like 3 [Source:HGNC Symbol;Acc:HGNC:1452] | 33436 | -0.110 | -0.4759 | Yes |
| 48 | ITPR2 | "inositol 1,4,5-trisphosphate receptor type 2 [Source:HGNC Symbol;Acc:HGNC:6181]" | 34058 | -0.119 | -0.4794 | Yes |
| 49 | OCRL | OCRL inositol polyphosphate-5-phosphatase [Source:HGNC Symbol;Acc:HGNC:8108] | 34364 | -0.123 | -0.4744 | Yes |
| 50 | PRKCB | protein kinase C beta [Source:HGNC Symbol;Acc:HGNC:9395] | 34603 | -0.128 | -0.4673 | Yes |
| 51 | PRKCA | protein kinase C alpha [Source:HGNC Symbol;Acc:HGNC:9393] | 35223 | -0.140 | -0.4686 | Yes |
| 52 | CDS2 | CDP-diacylglycerol synthase 2 [Source:HGNC Symbol;Acc:HGNC:1801] | 35314 | -0.142 | -0.4562 | Yes |
| 53 | DGKA | diacylglycerol kinase alpha [Source:HGNC Symbol;Acc:HGNC:2849] | 35454 | -0.146 | -0.4446 | Yes |
| 54 | PLCG2 | phospholipase C gamma 2 [Source:HGNC Symbol;Acc:HGNC:9066] | 35650 | -0.151 | -0.4340 | Yes |
| 55 | DGKD | diacylglycerol kinase delta [Source:HGNC Symbol;Acc:HGNC:2851] | 35683 | -0.152 | -0.4192 | Yes |
| 56 | PIP5K1A | phosphatidylinositol-4-phosphate 5-kinase type 1 alpha [Source:HGNC Symbol;Acc:HGNC:8994] | 35739 | -0.153 | -0.4047 | Yes |
| 57 | PIP4K2A | phosphatidylinositol-5-phosphate 4-kinase type 2 alpha [Source:HGNC Symbol;Acc:HGNC:8997] | 35842 | -0.156 | -0.3912 | Yes |
| 58 | PIK3C3 | phosphatidylinositol 3-kinase catalytic subunit type 3 [Source:HGNC Symbol;Acc:HGNC:8974] | 36105 | -0.163 | -0.3810 | Yes |
| 59 | ITPR1 | "inositol 1,4,5-trisphosphate receptor type 1 [Source:HGNC Symbol;Acc:HGNC:6180]" | 36283 | -0.169 | -0.3680 | Yes |
| 60 | INPP4A | inositol polyphosphate-4-phosphatase type I A [Source:HGNC Symbol;Acc:HGNC:6074] | 36783 | -0.188 | -0.3613 | Yes |
| 61 | DGKH | diacylglycerol kinase eta [Source:HGNC Symbol;Acc:HGNC:2854] | 36880 | -0.192 | -0.3438 | Yes |
| 62 | INPP5E | inositol polyphosphate-5-phosphatase E [Source:HGNC Symbol;Acc:HGNC:21474] | 36989 | -0.197 | -0.3262 | Yes |
| 63 | PIP4K2B | phosphatidylinositol-5-phosphate 4-kinase type 2 beta [Source:HGNC Symbol;Acc:HGNC:8998] | 37226 | -0.209 | -0.3107 | Yes |
| 64 | PIKFYVE | "phosphoinositide kinase, FYVE-type zinc finger containing [Source:HGNC Symbol;Acc:HGNC:23785]" | 37420 | -0.218 | -0.2930 | Yes |
| 65 | PIP5K1B | phosphatidylinositol-4-phosphate 5-kinase type 1 beta [Source:HGNC Symbol;Acc:HGNC:8995] | 37463 | -0.221 | -0.2712 | Yes |
| 66 | DGKB | diacylglycerol kinase beta [Source:HGNC Symbol;Acc:HGNC:2850] | 37641 | -0.234 | -0.2516 | Yes |
| 67 | ITPKA | inositol-trisphosphate 3-kinase A [Source:HGNC Symbol;Acc:HGNC:6178] | 37678 | -0.236 | -0.2281 | Yes |
| 68 | IPPK | inositol-pentakisphosphate 2-kinase [Source:HGNC Symbol;Acc:HGNC:14645] | 37705 | -0.238 | -0.2041 | Yes |
| 69 | DGKI | diacylglycerol kinase iota [Source:HGNC Symbol;Acc:HGNC:2855] | 37768 | -0.242 | -0.1806 | Yes |
| 70 | DGKE | diacylglycerol kinase epsilon [Source:HGNC Symbol;Acc:HGNC:2852] | 37861 | -0.250 | -0.1571 | Yes |
| 71 | PLCD1 | phospholipase C delta 1 [Source:HGNC Symbol;Acc:HGNC:9060] | 38013 | -0.267 | -0.1334 | Yes |
| 72 | DGKG | diacylglycerol kinase gamma [Source:HGNC Symbol;Acc:HGNC:2853] | 38136 | -0.283 | -0.1073 | Yes |
| 73 | PIK3CA | "phosphatidylinositol-4,5-bisphosphate 3-kinase catalytic subunit alpha [Source:HGNC Symbol;Acc:HGNC:8975]" | 38226 | -0.296 | -0.0789 | Yes |
| 74 | PIK3C2A | phosphatidylinositol-4-phosphate 3-kinase catalytic subunit type 2 alpha [Source:HGNC Symbol;Acc:HGNC:8971] | 38233 | -0.297 | -0.0483 | Yes |
| 75 | PLCB4 | phospholipase C beta 4 [Source:HGNC Symbol;Acc:HGNC:9059] | 38308 | -0.309 | -0.0182 | Yes |
| 76 | ITPKB | inositol-trisphosphate 3-kinase B [Source:HGNC Symbol;Acc:HGNC:6179] | 38542 | -0.426 | 0.0199 | Yes |
Table: GSEA details [plain text format]

  

Fig 2: KEGG\_PHOSPHATIDYLINOSITOL\_SIGNALING\_SYSTEM      
 Blue-Pink O' Gram in the Space of the Analyzed GeneSet

  

Fig 3: KEGG\_PHOSPHATIDYLINOSITOL\_SIGNALING\_SYSTEM: Random ES distribution      
 Gene set null distribution of ES for **KEGG\_PHOSPHATIDYLINOSITOL\_SIGNALING\_SYSTEM**
