# Supplementary material for: Simultaneous Presentation of Multiple Myeloma and Lung Cancer: Case Report and Gene Bioinformatics Analysis
Source: Front Oncol. 2022 Jun 13;12:859735. doi: 10.3389/fonc.2022.859735 (PMC9235397; doi:10.3389/fonc.2022.859735)
Supplement: Supplementary file 1 [file DataSheet_1.zip › The bioinformatic analysis of MM and lung cancer supplementary materials/Enrichment analysis/MECR/GSEA_4.1.0/LUSC TCGA/KEGG.Gsea.1639042374131/KEGG_PRIMARY_IMMUNODEFICIENCY.html]

Details for gene set KEGG\_PRIMARY\_IMMUNODEFICIENCY[GSEA]

|  || Dataset | ExpData\_collapsed\_to\_symbols.ENSG00000116353\_profile\_in\_ExpData.cls #ENSG00000116353 |
| Phenotype | ENSG00000116353\_profile\_in\_ExpData.cls#ENSG00000116353 |
| Upregulated in class | ENSG00000116353\_neg |
| GeneSet | KEGG\_PRIMARY\_IMMUNODEFICIENCY |
| Enrichment Score (ES) | -0.544928 |
| Normalized Enrichment Score (NES) | -1.9093022 |
| Nominal p-value | 0.00203666 |
| FDR q-value | 0.0047401497 |
| FWER p-Value | 0.034 |
Table: GSEA Results Summary

  

Fig 1: Enrichment plot: KEGG\_PRIMARY\_IMMUNODEFICIENCY      
 Profile of the Running ES Score & Positions of GeneSet Members on the Rank Ordered List

  

| SYMBOL | TITLE | RANK IN GENE LIST | RANK METRIC SCORE | RUNNING ES | CORE ENRICHMENT || 1 | DCLRE1C | DNA cross-link repair 1C [Source:HGNC Symbol;Acc:HGNC:17642] | 2821 | 0.141 | -0.0304 | No |
| 2 | BLNK | B cell linker [Source:HGNC Symbol;Acc:HGNC:14211] | 3127 | 0.134 | 0.0011 | No |
| 3 | RFXANK | regulatory factor X associated ankyrin containing protein [Source:HGNC Symbol;Acc:HGNC:9987] | 3592 | 0.125 | 0.0258 | No |
| 4 | CD40 | CD40 molecule [Source:HGNC Symbol;Acc:HGNC:11919] | 10160 | 0.059 | -0.1241 | No |
| 5 | UNG | uracil DNA glycosylase [Source:HGNC Symbol;Acc:HGNC:12572] | 19034 | 0.006 | -0.3480 | No |
| 6 | TAP1 | "transporter 1, ATP binding cassette subfamily B member [Source:HGNC Symbol;Acc:HGNC:43]" | 21598 | -0.009 | -0.4107 | No |
| 7 | CD8B | CD8b molecule [Source:HGNC Symbol;Acc:HGNC:1707] | 22034 | -0.011 | -0.4184 | No |
| 8 | TAP2 | "transporter 2, ATP binding cassette subfamily B member [Source:HGNC Symbol;Acc:HGNC:44]" | 25244 | -0.033 | -0.4904 | No |
| 9 | IGLL1 | immunoglobulin lambda like polypeptide 1 [Source:HGNC Symbol;Acc:HGNC:5870] | 26361 | -0.041 | -0.5069 | No |
| 10 | JAK3 | Janus kinase 3 [Source:HGNC Symbol;Acc:HGNC:6193] | 27721 | -0.051 | -0.5265 | No |
| 11 | IL2RG | interleukin 2 receptor subunit gamma [Source:HGNC Symbol;Acc:HGNC:6010] | 28032 | -0.054 | -0.5187 | No |
| 12 | TNFRSF13B | TNF receptor superfamily member 13B [Source:HGNC Symbol;Acc:HGNC:18153] | 29064 | -0.062 | -0.5267 | Yes |
| 13 | CD79A | CD79a molecule [Source:HGNC Symbol;Acc:HGNC:1698] | 29443 | -0.066 | -0.5171 | Yes |
| 14 | RAG2 | recombination activating 2 [Source:HGNC Symbol;Acc:HGNC:9832] | 29659 | -0.067 | -0.5028 | Yes |
| 15 | CD4 | CD4 molecule [Source:HGNC Symbol;Acc:HGNC:1678] | 30573 | -0.076 | -0.5038 | Yes |
| 16 | RAG1 | recombination activating 1 [Source:HGNC Symbol;Acc:HGNC:9831] | 30954 | -0.080 | -0.4900 | Yes |
| 17 | LCK | "LCK proto-oncogene, Src family tyrosine kinase [Source:HGNC Symbol;Acc:HGNC:6524]" | 31086 | -0.081 | -0.4696 | Yes |
| 18 | CD3D | CD3d molecule [Source:HGNC Symbol;Acc:HGNC:1673] | 31455 | -0.085 | -0.4541 | Yes |
| 19 | CIITA | class II major histocompatibility complex transactivator [Source:HGNC Symbol;Acc:HGNC:7067] | 31860 | -0.089 | -0.4382 | Yes |
| 20 | ADA | adenosine deaminase [Source:HGNC Symbol;Acc:HGNC:186] | 32406 | -0.096 | -0.4241 | Yes |
| 21 | AIRE | autoimmune regulator [Source:HGNC Symbol;Acc:HGNC:360] | 33337 | -0.108 | -0.4161 | Yes |
| 22 | AICDA | activation induced cytidine deaminase [Source:HGNC Symbol;Acc:HGNC:13203] | 33374 | -0.109 | -0.3852 | Yes |
| 23 | CD19 | CD19 molecule [Source:HGNC Symbol;Acc:HGNC:1633] | 33399 | -0.109 | -0.3539 | Yes |
| 24 | CD8A | CD8a molecule [Source:HGNC Symbol;Acc:HGNC:1706] | 33518 | -0.111 | -0.3244 | Yes |
| 25 | CD3E | CD3e molecule [Source:HGNC Symbol;Acc:HGNC:1674] | 33847 | -0.116 | -0.2989 | Yes |
| 26 | ZAP70 | zeta chain of T cell receptor associated protein kinase 70 [Source:HGNC Symbol;Acc:HGNC:12858] | 33868 | -0.116 | -0.2654 | Yes |
| 27 | CD40LG | CD40 ligand [Source:HGNC Symbol;Acc:HGNC:11935] | 34481 | -0.125 | -0.2442 | Yes |
| 28 | ICOS | inducible T cell costimulator [Source:HGNC Symbol;Acc:HGNC:5351] | 34857 | -0.132 | -0.2150 | Yes |
| 29 | BTK | Bruton tyrosine kinase [Source:HGNC Symbol;Acc:HGNC:1133] | 34865 | -0.132 | -0.1764 | Yes |
| 30 | RFXAP | regulatory factor X associated protein [Source:HGNC Symbol;Acc:HGNC:9988] | 35244 | -0.141 | -0.1448 | Yes |
| 31 | IL7R | interleukin 7 receptor [Source:HGNC Symbol;Acc:HGNC:6024] | 35643 | -0.151 | -0.1108 | Yes |
| 32 | PTPRC | protein tyrosine phosphatase receptor type C [Source:HGNC Symbol;Acc:HGNC:9666] | 35701 | -0.152 | -0.0676 | Yes |
| 33 | RFX5 | regulatory factor X5 [Source:HGNC Symbol;Acc:HGNC:9986] | 35949 | -0.159 | -0.0274 | Yes |
| 34 | TNFRSF13C | TNF receptor superfamily member 13C [Source:HGNC Symbol;Acc:HGNC:17755] | 36323 | -0.171 | 0.0132 | Yes |
| 35 | IKBKG | inhibitor of nuclear factor kappa B kinase regulatory subunit gamma [Source:HGNC Symbol;Acc:HGNC:5961] | 37365 | -0.216 | 0.0498 | Yes |
Table: GSEA details [plain text format]

  

Fig 2: KEGG\_PRIMARY\_IMMUNODEFICIENCY      
 Blue-Pink O' Gram in the Space of the Analyzed GeneSet

  

Fig 3: KEGG\_PRIMARY\_IMMUNODEFICIENCY: Random ES distribution      
 Gene set null distribution of ES for **KEGG\_PRIMARY\_IMMUNODEFICIENCY**
