# Supplementary material for: Simultaneous Presentation of Multiple Myeloma and Lung Cancer: Case Report and Gene Bioinformatics Analysis
Source: Front Oncol. 2022 Jun 13;12:859735. doi: 10.3389/fonc.2022.859735 (PMC9235397; doi:10.3389/fonc.2022.859735)
Supplement: Supplementary file 1 [file DataSheet_1.zip › The bioinformatic analysis of MM and lung cancer supplementary materials/Enrichment analysis/MECR/GSEA_4.1.0/LUSC TCGA/KEGG.Gsea.1639042374131/KEGG_PROTEASOME.html]

Details for gene set KEGG\_PROTEASOME[GSEA]

|  || Dataset | ExpData\_collapsed\_to\_symbols.ENSG00000116353\_profile\_in\_ExpData.cls #ENSG00000116353 |
| Phenotype | ENSG00000116353\_profile\_in\_ExpData.cls#ENSG00000116353 |
| Upregulated in class | ENSG00000116353\_pos |
| GeneSet | KEGG\_PROTEASOME |
| Enrichment Score (ES) | 0.44577217 |
| Normalized Enrichment Score (NES) | 1.6422889 |
| Nominal p-value | 0.009505703 |
| FDR q-value | 0.028448837 |
| FWER p-Value | 0.524 |
Table: GSEA Results Summary

  

Fig 1: Enrichment plot: KEGG\_PROTEASOME      
 Profile of the Running ES Score & Positions of GeneSet Members on the Rank Ordered List

  

| SYMBOL | TITLE | RANK IN GENE LIST | RANK METRIC SCORE | RUNNING ES | CORE ENRICHMENT || 1 | PSMB2 | proteasome 20S subunit beta 2 [Source:HGNC Symbol;Acc:HGNC:9539] | 22 | 0.502 | 0.1130 | Yes |
| 2 | PSMA5 | proteasome 20S subunit alpha 5 [Source:HGNC Symbol;Acc:HGNC:9534] | 346 | 0.296 | 0.1718 | Yes |
| 3 | PSMA7 | proteasome 20S subunit alpha 7 [Source:HGNC Symbol;Acc:HGNC:9536] | 494 | 0.271 | 0.2295 | Yes |
| 4 | PSMD8 | "proteasome 26S subunit, non-ATPase 8 [Source:HGNC Symbol;Acc:HGNC:9566]" | 2303 | 0.155 | 0.2186 | Yes |
| 5 | PSMD13 | "proteasome 26S subunit, non-ATPase 13 [Source:HGNC Symbol;Acc:HGNC:9558]" | 2620 | 0.147 | 0.2437 | Yes |
| 6 | PSMB1 | proteasome 20S subunit beta 1 [Source:HGNC Symbol;Acc:HGNC:9537] | 2703 | 0.144 | 0.2742 | Yes |
| 7 | PSMC3 | "proteasome 26S subunit, ATPase 3 [Source:HGNC Symbol;Acc:HGNC:9549]" | 3132 | 0.134 | 0.2935 | Yes |
| 8 | PSMB5 | proteasome 20S subunit beta 5 [Source:HGNC Symbol;Acc:HGNC:9542] | 3194 | 0.133 | 0.3220 | Yes |
| 9 | PSMB6 | proteasome 20S subunit beta 6 [Source:HGNC Symbol;Acc:HGNC:9543] | 3917 | 0.119 | 0.3306 | Yes |
| 10 | PSME1 | proteasome activator subunit 1 [Source:HGNC Symbol;Acc:HGNC:9568] | 4019 | 0.117 | 0.3546 | Yes |
| 11 | PSME2 | proteasome activator subunit 2 [Source:HGNC Symbol;Acc:HGNC:9569] | 4312 | 0.113 | 0.3726 | Yes |
| 12 | PSMB10 | proteasome 20S subunit beta 10 [Source:HGNC Symbol;Acc:HGNC:9538] | 4715 | 0.107 | 0.3866 | Yes |
| 13 | PSMF1 | proteasome inhibitor subunit 1 [Source:HGNC Symbol;Acc:HGNC:9571] | 4835 | 0.105 | 0.4073 | Yes |
| 14 | PSMC6 | "proteasome 26S subunit, ATPase 6 [Source:HGNC Symbol;Acc:HGNC:9553]" | 5376 | 0.098 | 0.4158 | Yes |
| 15 | PSMC4 | "proteasome 26S subunit, ATPase 4 [Source:HGNC Symbol;Acc:HGNC:9551]" | 6392 | 0.090 | 0.4102 | Yes |
| 16 | PSMA6 | proteasome 20S subunit alpha 6 [Source:HGNC Symbol;Acc:HGNC:9535] | 6885 | 0.085 | 0.4169 | Yes |
| 17 | SEM1 | SEM1 26S proteasome complex subunit [Source:HGNC Symbol;Acc:HGNC:10845] | 7164 | 0.082 | 0.4284 | Yes |
| 18 | PSMA6P4 | proteasome subunit alpha 6 pseudogene 4 [Source:HGNC Symbol;Acc:HGNC:39609] | 7242 | 0.081 | 0.4449 | Yes |
| 19 | PSMB8 | proteasome 20S subunit beta 8 [Source:HGNC Symbol;Acc:HGNC:9545] | 7922 | 0.075 | 0.4447 | Yes |
| 20 | PSMA3 | proteasome 20S subunit alpha 3 [Source:HGNC Symbol;Acc:HGNC:9532] | 8507 | 0.071 | 0.4458 | Yes |
| 21 | PSMB4 | proteasome 20S subunit beta 4 [Source:HGNC Symbol;Acc:HGNC:9541] | 10941 | 0.054 | 0.3960 | No |
| 22 | PSMB3 | proteasome 20S subunit beta 3 [Source:HGNC Symbol;Acc:HGNC:9540] | 11320 | 0.052 | 0.3981 | No |
| 23 | PSMD4 | "proteasome 26S subunit, non-ATPase 4 [Source:HGNC Symbol;Acc:HGNC:9561]" | 12708 | 0.042 | 0.3724 | No |
| 24 | PSMA2 | proteasome 20S subunit alpha 2 [Source:HGNC Symbol;Acc:HGNC:9531] | 13759 | 0.036 | 0.3539 | No |
| 25 | PSMC2 | "proteasome 26S subunit, ATPase 2 [Source:HGNC Symbol;Acc:HGNC:9548]" | 14364 | 0.033 | 0.3459 | No |
| 26 | POMP | proteasome maturation protein [Source:HGNC Symbol;Acc:HGNC:20330] | 15003 | 0.029 | 0.3363 | No |
| 27 | PSMC1 | "proteasome 26S subunit, ATPase 1 [Source:HGNC Symbol;Acc:HGNC:9547]" | 15290 | 0.028 | 0.3353 | No |
| 28 | PSMD7 | "proteasome 26S subunit, non-ATPase 7 [Source:HGNC Symbol;Acc:HGNC:9565]" | 15412 | 0.027 | 0.3383 | No |
| 29 | PSMB11 | proteasome subunit beta 11 [Source:HGNC Symbol;Acc:HGNC:31963] | 17855 | 0.013 | 0.2790 | No |
| 30 | PSMD14 | "proteasome 26S subunit, non-ATPase 14 [Source:HGNC Symbol;Acc:HGNC:16889]" | 18253 | 0.011 | 0.2714 | No |
| 31 | PSMD1 | "proteasome 26S subunit, non-ATPase 1 [Source:HGNC Symbol;Acc:HGNC:9554]" | 19542 | 0.003 | 0.2393 | No |
| 32 | PSMB9 | proteasome 20S subunit beta 9 [Source:HGNC Symbol;Acc:HGNC:9546] | 19608 | 0.003 | 0.2383 | No |
| 33 | PSMA4 | proteasome 20S subunit alpha 4 [Source:HGNC Symbol;Acc:HGNC:9533] | 20999 | -0.005 | 0.2041 | No |
| 34 | PSMA1 | proteasome 20S subunit alpha 1 [Source:HGNC Symbol;Acc:HGNC:9530] | 21873 | -0.010 | 0.1843 | No |
| 35 | PSMC1P4 | "proteasome 26S subunit, ATPase 1 pseudogene 4 [Source:HGNC Symbol;Acc:HGNC:39779]" | 22720 | -0.016 | 0.1663 | No |
| 36 | PSMC5 | "proteasome 26S subunit, ATPase 5 [Source:HGNC Symbol;Acc:HGNC:9552]" | 23560 | -0.021 | 0.1496 | No |
| 37 | PSME4 | proteasome activator subunit 4 [Source:HGNC Symbol;Acc:HGNC:20635] | 26593 | -0.042 | 0.0820 | No |
| 38 | PSMA8 | proteasome 20S subunit alpha 8 [Source:HGNC Symbol;Acc:HGNC:22985] | 27914 | -0.053 | 0.0604 | No |
| 39 | PSMD12 | "proteasome 26S subunit, non-ATPase 12 [Source:HGNC Symbol;Acc:HGNC:9557]" | 29029 | -0.062 | 0.0460 | No |
| 40 | IFNG | interferon gamma [Source:HGNC Symbol;Acc:HGNC:5438] | 29128 | -0.063 | 0.0577 | No |
| 41 | PSMB7 | proteasome 20S subunit beta 7 [Source:HGNC Symbol;Acc:HGNC:9544] | 29835 | -0.069 | 0.0554 | No |
| 42 | PSMD6 | "proteasome 26S subunit, non-ATPase 6 [Source:HGNC Symbol;Acc:HGNC:9564]" | 30528 | -0.076 | 0.0549 | No |
| 43 | PSMD3 | "proteasome 26S subunit, non-ATPase 3 [Source:HGNC Symbol;Acc:HGNC:9560]" | 30739 | -0.078 | 0.0671 | No |
| 44 | PSME3 | proteasome activator subunit 3 [Source:HGNC Symbol;Acc:HGNC:9570] | 35867 | -0.157 | -0.0280 | No |
| 45 | PSMD2 | "proteasome 26S subunit, non-ATPase 2 [Source:HGNC Symbol;Acc:HGNC:9559]" | 37390 | -0.217 | -0.0176 | No |
| 46 | PSMD11 | "proteasome 26S subunit, non-ATPase 11 [Source:HGNC Symbol;Acc:HGNC:9556]" | 38222 | -0.295 | 0.0280 | No |
Table: GSEA details [plain text format]

  

Fig 2: KEGG\_PROTEASOME      
 Blue-Pink O' Gram in the Space of the Analyzed GeneSet

  

Fig 3: KEGG\_PROTEASOME: Random ES distribution      
 Gene set null distribution of ES for **KEGG\_PROTEASOME**
