# Supplementary material for: Simultaneous Presentation of Multiple Myeloma and Lung Cancer: Case Report and Gene Bioinformatics Analysis
Source: Front Oncol. 2022 Jun 13;12:859735. doi: 10.3389/fonc.2022.859735 (PMC9235397; doi:10.3389/fonc.2022.859735)
Supplement: Supplementary file 1 [file DataSheet_1.zip › The bioinformatic analysis of MM and lung cancer supplementary materials/Enrichment analysis/MECR/GSEA_4.1.0/LUSC TCGA/KEGG.Gsea.1639042374131/KEGG_PURINE_METABOLISM.html]

Details for gene set KEGG\_PURINE\_METABOLISM[GSEA]

|  || Dataset | ExpData\_collapsed\_to\_symbols.ENSG00000116353\_profile\_in\_ExpData.cls #ENSG00000116353 |
| Phenotype | ENSG00000116353\_profile\_in\_ExpData.cls#ENSG00000116353 |
| Upregulated in class | ENSG00000116353\_pos |
| GeneSet | KEGG\_PURINE\_METABOLISM |
| Enrichment Score (ES) | 0.37471187 |
| Normalized Enrichment Score (NES) | 1.7206022 |
| Nominal p-value | 0.0 |
| FDR q-value | 0.016725732 |
| FWER p-Value | 0.313 |
Table: GSEA Results Summary

  

Fig 1: Enrichment plot: KEGG\_PURINE\_METABOLISM      
 Profile of the Running ES Score & Positions of GeneSet Members on the Rank Ordered List

  

| SYMBOL | TITLE | RANK IN GENE LIST | RANK METRIC SCORE | RUNNING ES | CORE ENRICHMENT || 1 | AK2 | adenylate kinase 2 [Source:HGNC Symbol;Acc:HGNC:362] | 10 | 0.528 | 0.0284 | Yes |
| 2 | POLR1H | RNA polymerase I subunit H [Source:HGNC Symbol;Acc:HGNC:13182] | 172 | 0.352 | 0.0434 | Yes |
| 3 | POLR2K | "RNA polymerase II, I and III subunit K [Source:HGNC Symbol;Acc:HGNC:9198]" | 217 | 0.333 | 0.0604 | Yes |
| 4 | POLR2L | "RNA polymerase II, I and III subunit L [Source:HGNC Symbol;Acc:HGNC:9199]" | 221 | 0.331 | 0.0783 | Yes |
| 5 | GUK1 | guanylate kinase 1 [Source:HGNC Symbol;Acc:HGNC:4693] | 364 | 0.293 | 0.0906 | Yes |
| 6 | APRT | adenine phosphoribosyltransferase [Source:HGNC Symbol;Acc:HGNC:626] | 374 | 0.291 | 0.1061 | Yes |
| 7 | POLR2G | RNA polymerase II subunit G [Source:HGNC Symbol;Acc:HGNC:9194] | 461 | 0.275 | 0.1188 | Yes |
| 8 | ITPA | inosine triphosphatase [Source:HGNC Symbol;Acc:HGNC:6176] | 482 | 0.273 | 0.1331 | Yes |
| 9 | NME7 | NME/NM23 family member 7 [Source:HGNC Symbol;Acc:HGNC:20461] | 531 | 0.265 | 0.1463 | Yes |
| 10 | NME3 | NME/NM23 nucleoside diphosphate kinase 3 [Source:HGNC Symbol;Acc:HGNC:7851] | 657 | 0.250 | 0.1567 | Yes |
| 11 | POLE4 | "DNA polymerase epsilon 4, accessory subunit [Source:HGNC Symbol;Acc:HGNC:18755]" | 759 | 0.237 | 0.1670 | Yes |
| 12 | ADK | adenosine kinase [Source:HGNC Symbol;Acc:HGNC:257] | 827 | 0.228 | 0.1777 | Yes |
| 13 | NUDT5 | nudix hydrolase 5 [Source:HGNC Symbol;Acc:HGNC:8052] | 876 | 0.224 | 0.1886 | Yes |
| 14 | IMPDH2 | inosine monophosphate dehydrogenase 2 [Source:HGNC Symbol;Acc:HGNC:6053] | 908 | 0.221 | 0.1998 | Yes |
| 15 | POLR2I | RNA polymerase II subunit I [Source:HGNC Symbol;Acc:HGNC:9196] | 1025 | 0.213 | 0.2084 | Yes |
| 16 | NME1-NME2 | NME1-NME2 readthrough [Source:HGNC Symbol;Acc:HGNC:33531] | 1182 | 0.204 | 0.2155 | Yes |
| 17 | NME4 | NME/NM23 nucleoside diphosphate kinase 4 [Source:HGNC Symbol;Acc:HGNC:7852] | 1206 | 0.203 | 0.2259 | Yes |
| 18 | POLR2J | RNA polymerase II subunit J [Source:HGNC Symbol;Acc:HGNC:9197] | 1233 | 0.201 | 0.2362 | Yes |
| 19 | POLR3A | RNA polymerase III subunit A [Source:HGNC Symbol;Acc:HGNC:30074] | 1258 | 0.200 | 0.2464 | Yes |
| 20 | NME6 | NME/NM23 nucleoside diphosphate kinase 6 [Source:HGNC Symbol;Acc:HGNC:20567] | 1304 | 0.197 | 0.2560 | Yes |
| 21 | POLD1 | "DNA polymerase delta 1, catalytic subunit [Source:HGNC Symbol;Acc:HGNC:9175]" | 1356 | 0.194 | 0.2652 | Yes |
| 22 | POLE2 | "DNA polymerase epsilon 2, accessory subunit [Source:HGNC Symbol;Acc:HGNC:9178]" | 1540 | 0.184 | 0.2705 | Yes |
| 23 | NME1 | NME/NM23 nucleoside diphosphate kinase 1 [Source:HGNC Symbol;Acc:HGNC:7849] | 1592 | 0.182 | 0.2791 | Yes |
| 24 | ADSS1 | adenylosuccinate synthase 1 [Source:HGNC Symbol;Acc:HGNC:20093] | 2098 | 0.162 | 0.2750 | Yes |
| 25 | PDE6D | phosphodiesterase 6D [Source:HGNC Symbol;Acc:HGNC:8788] | 2130 | 0.161 | 0.2829 | Yes |
| 26 | POLR3B | RNA polymerase III subunit B [Source:HGNC Symbol;Acc:HGNC:30348] | 2133 | 0.161 | 0.2916 | Yes |
| 27 | PDE7A | phosphodiesterase 7A [Source:HGNC Symbol;Acc:HGNC:8791] | 2245 | 0.157 | 0.2973 | Yes |
| 28 | GUCY2C | guanylate cyclase 2C [Source:HGNC Symbol;Acc:HGNC:4688] | 2412 | 0.152 | 0.3013 | Yes |
| 29 | ENTPD5 | ectonucleoside triphosphate diphosphohydrolase 5 (inactive) [Source:HGNC Symbol;Acc:HGNC:3367] | 2456 | 0.150 | 0.3084 | Yes |
| 30 | POLR1C | RNA polymerase I and III subunit C [Source:HGNC Symbol;Acc:HGNC:20194] | 2536 | 0.149 | 0.3144 | Yes |
| 31 | POLR3K | RNA polymerase III subunit K [Source:HGNC Symbol;Acc:HGNC:14121] | 2767 | 0.142 | 0.3163 | Yes |
| 32 | POLR2C | RNA polymerase II subunit C [Source:HGNC Symbol;Acc:HGNC:9189] | 2813 | 0.141 | 0.3228 | Yes |
| 33 | POLR3G | RNA polymerase III subunit G [Source:HGNC Symbol;Acc:HGNC:30075] | 3270 | 0.131 | 0.3183 | Yes |
| 34 | PNP | purine nucleoside phosphorylase [Source:HGNC Symbol;Acc:HGNC:7892] | 3344 | 0.129 | 0.3234 | Yes |
| 35 | POLD4 | "DNA polymerase delta 4, accessory subunit [Source:HGNC Symbol;Acc:HGNC:14106]" | 3573 | 0.125 | 0.3244 | Yes |
| 36 | ADSL | adenylosuccinate lyase [Source:HGNC Symbol;Acc:HGNC:291] | 3583 | 0.125 | 0.3309 | Yes |
| 37 | POLR3H | RNA polymerase III subunit H [Source:HGNC Symbol;Acc:HGNC:30349] | 3690 | 0.123 | 0.3349 | Yes |
| 38 | POLR2F | "RNA polymerase II, I and III subunit F [Source:HGNC Symbol;Acc:HGNC:9193]" | 3719 | 0.122 | 0.3408 | Yes |
| 39 | PRPS1 | phosphoribosyl pyrophosphate synthetase 1 [Source:HGNC Symbol;Acc:HGNC:9462] | 3892 | 0.119 | 0.3429 | Yes |
| 40 | GART | "phosphoribosylglycinamide formyltransferase, phosphoribosylglycinamide synthetase, phosphoribosylaminoimidazole synthetase [Source:HGNC Symbol;Acc:HGNC:4163]" | 3951 | 0.119 | 0.3479 | Yes |
| 41 | FHIT | fragile histidine triad diadenosine triphosphatase [Source:HGNC Symbol;Acc:HGNC:3701] | 4050 | 0.117 | 0.3517 | Yes |
| 42 | NT5C | "5', 3'-nucleotidase, cytosolic [Source:HGNC Symbol;Acc:HGNC:17144]" | 4125 | 0.116 | 0.3561 | Yes |
| 43 | PKM | pyruvate kinase M1/2 [Source:HGNC Symbol;Acc:HGNC:9021] | 4388 | 0.111 | 0.3554 | Yes |
| 44 | XDH | xanthine dehydrogenase [Source:HGNC Symbol;Acc:HGNC:12805] | 4454 | 0.110 | 0.3598 | Yes |
| 45 | AK4 | adenylate kinase 4 [Source:HGNC Symbol;Acc:HGNC:363] | 4457 | 0.110 | 0.3657 | Yes |
| 46 | NME2 | NME/NM23 nucleoside diphosphate kinase 2 [Source:HGNC Symbol;Acc:HGNC:7850] | 4705 | 0.107 | 0.3652 | Yes |
| 47 | PDE4C | phosphodiesterase 4C [Source:HGNC Symbol;Acc:HGNC:8782] | 4777 | 0.106 | 0.3691 | Yes |
| 48 | NUDT9 | nudix hydrolase 9 [Source:HGNC Symbol;Acc:HGNC:8056] | 4784 | 0.106 | 0.3747 | Yes |
| 49 | ATIC | 5-aminoimidazole-4-carboxamide ribonucleotide formyltransferase/IMP cyclohydrolase [Source:HGNC Symbol;Acc:HGNC:794] | 5499 | 0.097 | 0.3617 | No |
| 50 | DGUOK | deoxyguanosine kinase [Source:HGNC Symbol;Acc:HGNC:2858] | 6195 | 0.092 | 0.3490 | No |
| 51 | AMPD2 | adenosine monophosphate deaminase 2 [Source:HGNC Symbol;Acc:HGNC:469] | 6294 | 0.091 | 0.3514 | No |
| 52 | ENTPD6 | ectonucleoside triphosphate diphosphohydrolase 6 [Source:HGNC Symbol;Acc:HGNC:3368] | 6358 | 0.090 | 0.3547 | No |
| 53 | PDE6A | phosphodiesterase 6A [Source:HGNC Symbol;Acc:HGNC:8785] | 6562 | 0.088 | 0.3542 | No |
| 54 | PAPSS1 | 3'-phosphoadenosine 5'-phosphosulfate synthase 1 [Source:HGNC Symbol;Acc:HGNC:8603] | 6632 | 0.087 | 0.3572 | No |
| 55 | PRUNE1 | prune exopolyphosphatase 1 [Source:HGNC Symbol;Acc:HGNC:13420] | 6956 | 0.084 | 0.3535 | No |
| 56 | NT5C2 | "5'-nucleotidase, cytosolic II [Source:HGNC Symbol;Acc:HGNC:8022]" | 7091 | 0.083 | 0.3546 | No |
| 57 | POLD2 | "DNA polymerase delta 2, accessory subunit [Source:HGNC Symbol;Acc:HGNC:9176]" | 7505 | 0.079 | 0.3484 | No |
| 58 | NME5 | NME/NM23 family member 5 [Source:HGNC Symbol;Acc:HGNC:7853] | 7564 | 0.079 | 0.3511 | No |
| 59 | POLR2E | "RNA polymerase II, I and III subunit E [Source:HGNC Symbol;Acc:HGNC:9192]" | 7792 | 0.077 | 0.3495 | No |
| 60 | POLR3D | RNA polymerase III subunit D [Source:HGNC Symbol;Acc:HGNC:1080] | 8233 | 0.073 | 0.3422 | No |
| 61 | GMPR2 | guanosine monophosphate reductase 2 [Source:HGNC Symbol;Acc:HGNC:4377] | 8372 | 0.072 | 0.3426 | No |
| 62 | NT5E | 5'-nucleotidase ecto [Source:HGNC Symbol;Acc:HGNC:8021] | 8757 | 0.069 | 0.3365 | No |
| 63 | PAPSS2 | 3'-phosphoadenosine 5'-phosphosulfate synthase 2 [Source:HGNC Symbol;Acc:HGNC:8604] | 9265 | 0.065 | 0.3271 | No |
| 64 | POLR3GL | RNA polymerase III subunit GL [Source:HGNC Symbol;Acc:HGNC:28466] | 9791 | 0.062 | 0.3171 | No |
| 65 | ENTPD2 | ectonucleoside triphosphate diphosphohydrolase 2 [Source:HGNC Symbol;Acc:HGNC:3364] | 9797 | 0.062 | 0.3203 | No |
| 66 | PRPS2 | phosphoribosyl pyrophosphate synthetase 2 [Source:HGNC Symbol;Acc:HGNC:9465] | 9901 | 0.061 | 0.3210 | No |
| 67 | POLR1D | RNA polymerase I and III subunit D [Source:HGNC Symbol;Acc:HGNC:20422] | 10132 | 0.059 | 0.3183 | No |
| 68 | PDE6H | phosphodiesterase 6H [Source:HGNC Symbol;Acc:HGNC:8790] | 10431 | 0.057 | 0.3138 | No |
| 69 | PDE7B | phosphodiesterase 7B [Source:HGNC Symbol;Acc:HGNC:8792] | 10476 | 0.057 | 0.3158 | No |
| 70 | PRIM1 | DNA primase subunit 1 [Source:HGNC Symbol;Acc:HGNC:9369] | 11631 | 0.050 | 0.2890 | No |
| 71 | PDE6C | phosphodiesterase 6C [Source:HGNC Symbol;Acc:HGNC:8787] | 12317 | 0.045 | 0.2740 | No |
| 72 | PRPS1L1 | phosphoribosyl pyrophosphate synthetase 1 like 1 [Source:HGNC Symbol;Acc:HGNC:9463] | 12342 | 0.045 | 0.2758 | No |
| 73 | POLR3C | RNA polymerase III subunit C [Source:HGNC Symbol;Acc:HGNC:30076] | 12617 | 0.043 | 0.2711 | No |
| 74 | PAICS | phosphoribosylaminoimidazole carboxylase and phosphoribosylaminoimidazolesuccinocarboxamide synthase [Source:HGNC Symbol;Acc:HGNC:8587] | 12893 | 0.042 | 0.2664 | No |
| 75 | POLR2B | RNA polymerase II subunit B [Source:HGNC Symbol;Acc:HGNC:9188] | 13423 | 0.038 | 0.2549 | No |
| 76 | POLR1A | RNA polymerase I subunit A [Source:HGNC Symbol;Acc:HGNC:17264] | 13512 | 0.038 | 0.2548 | No |
| 77 | CANT1 | calcium activated nucleotidase 1 [Source:HGNC Symbol;Acc:HGNC:19721] | 13912 | 0.036 | 0.2465 | No |
| 78 | PKLR | pyruvate kinase L/R [Source:HGNC Symbol;Acc:HGNC:9020] | 14013 | 0.035 | 0.2459 | No |
| 79 | URAD | ureidoimidazoline (2-oxo-4-hydroxy-4-carboxy-5-) decarboxylase [Source:HGNC Symbol;Acc:HGNC:17785] | 15030 | 0.029 | 0.2215 | No |
| 80 | AK1 | adenylate kinase 1 [Source:HGNC Symbol;Acc:HGNC:361] | 15167 | 0.028 | 0.2196 | No |
| 81 | POLR1E | RNA polymerase I subunit E [Source:HGNC Symbol;Acc:HGNC:17631] | 15209 | 0.028 | 0.2200 | No |
| 82 | PPAT | phosphoribosyl pyrophosphate amidotransferase [Source:HGNC Symbol;Acc:HGNC:9238] | 16367 | 0.021 | 0.1916 | No |
| 83 | POLD3 | "DNA polymerase delta 3, accessory subunit [Source:HGNC Symbol;Acc:HGNC:20932]" | 16528 | 0.020 | 0.1887 | No |
| 84 | PDE1A | phosphodiesterase 1A [Source:HGNC Symbol;Acc:HGNC:8774] | 16561 | 0.020 | 0.1889 | No |
| 85 | POLR3F | RNA polymerase III subunit F [Source:HGNC Symbol;Acc:HGNC:15763] | 17157 | 0.017 | 0.1747 | No |
| 86 | ADPRM | "ADP-ribose/CDP-alcohol diphosphatase, manganese dependent [Source:HGNC Symbol;Acc:HGNC:30925]" | 17627 | 0.014 | 0.1635 | No |
| 87 | ENPP3 | ectonucleotide pyrophosphatase/phosphodiesterase 3 [Source:HGNC Symbol;Acc:HGNC:3358] | 17636 | 0.014 | 0.1640 | No |
| 88 | NUDT2 | nudix hydrolase 2 [Source:HGNC Symbol;Acc:HGNC:8049] | 17767 | 0.013 | 0.1614 | No |
| 89 | ENTPD8 | ectonucleoside triphosphate diphosphohydrolase 8 [Source:HGNC Symbol;Acc:HGNC:24860] | 17776 | 0.013 | 0.1620 | No |
| 90 | PRIM2 | DNA primase subunit 2 [Source:HGNC Symbol;Acc:HGNC:9370] | 18680 | 0.008 | 0.1394 | No |
| 91 | PDE10A | phosphodiesterase 10A [Source:HGNC Symbol;Acc:HGNC:8772] | 19168 | 0.006 | 0.1272 | No |
| 92 | PFAS | phosphoribosylformylglycinamidine synthase [Source:HGNC Symbol;Acc:HGNC:8863] | 19515 | 0.003 | 0.1186 | No |
| 93 | POLE | "DNA polymerase epsilon, catalytic subunit [Source:HGNC Symbol;Acc:HGNC:9177]" | 19623 | 0.003 | 0.1160 | No |
| 94 | AK5 | adenylate kinase 5 [Source:HGNC Symbol;Acc:HGNC:365] | 19776 | 0.002 | 0.1122 | No |
| 95 | ADCY6 | adenylate cyclase 6 [Source:HGNC Symbol;Acc:HGNC:237] | 20109 | 0.000 | 0.1038 | No |
| 96 | GUCY2F | "guanylate cyclase 2F, retinal [Source:HGNC Symbol;Acc:HGNC:4691]" | 20581 | -0.003 | 0.0919 | No |
| 97 | POLR2J2 | RNA polymerase II subunit J2 [Source:HGNC Symbol;Acc:HGNC:23208] | 20932 | -0.005 | 0.0832 | No |
| 98 | PDE8A | phosphodiesterase 8A [Source:HGNC Symbol;Acc:HGNC:8793] | 21269 | -0.007 | 0.0750 | No |
| 99 | PDE6B | phosphodiesterase 6B [Source:HGNC Symbol;Acc:HGNC:8786] | 22746 | -0.016 | 0.0382 | No |
| 100 | PDE3A | phosphodiesterase 3A [Source:HGNC Symbol;Acc:HGNC:8778] | 22956 | -0.017 | 0.0338 | No |
| 101 | NT5C1A | "5'-nucleotidase, cytosolic IA [Source:HGNC Symbol;Acc:HGNC:17819]" | 23150 | -0.018 | 0.0298 | No |
| 102 | PNPT1 | polyribonucleotide nucleotidyltransferase 1 [Source:HGNC Symbol;Acc:HGNC:23166] | 23526 | -0.021 | 0.0214 | No |
| 103 | PDE11A | phosphodiesterase 11A [Source:HGNC Symbol;Acc:HGNC:8773] | 23848 | -0.023 | 0.0144 | No |
| 104 | RRM2 | ribonucleotide reductase regulatory subunit M2 [Source:HGNC Symbol;Acc:HGNC:10452] | 23886 | -0.023 | 0.0147 | No |
| 105 | POLR2A | RNA polymerase II subunit A [Source:HGNC Symbol;Acc:HGNC:9187] | 24164 | -0.025 | 0.0090 | No |
| 106 | RRM2B | ribonucleotide reductase regulatory TP53 inducible subunit M2B [Source:HGNC Symbol;Acc:HGNC:17296] | 24378 | -0.026 | 0.0050 | No |
| 107 | POLA2 | "DNA polymerase alpha 2, accessory subunit [Source:HGNC Symbol;Acc:HGNC:30073]" | 24419 | -0.027 | 0.0054 | No |
| 108 | HPRT1 | hypoxanthine phosphoribosyltransferase 1 [Source:HGNC Symbol;Acc:HGNC:5157] | 24912 | -0.031 | -0.0055 | No |
| 109 | NPR1 | natriuretic peptide receptor 1 [Source:HGNC Symbol;Acc:HGNC:7943] | 25289 | -0.033 | -0.0133 | No |
| 110 | NT5M | "5',3'-nucleotidase, mitochondrial [Source:HGNC Symbol;Acc:HGNC:15769]" | 25355 | -0.034 | -0.0131 | No |
| 111 | ADCY1 | adenylate cyclase 1 [Source:HGNC Symbol;Acc:HGNC:232] | 25830 | -0.037 | -0.0232 | No |
| 112 | POLR2J3 | RNA polymerase II subunit J3 [Source:HGNC Symbol;Acc:HGNC:33853] | 25994 | -0.038 | -0.0253 | No |
| 113 | PDE2A | phosphodiesterase 2A [Source:HGNC Symbol;Acc:HGNC:8777] | 26475 | -0.042 | -0.0353 | No |
| 114 | POLR2H | "RNA polymerase II, I and III subunit H [Source:HGNC Symbol;Acc:HGNC:9195]" | 26753 | -0.044 | -0.0400 | No |
| 115 | PDE9A | phosphodiesterase 9A [Source:HGNC Symbol;Acc:HGNC:8795] | 27731 | -0.051 | -0.0622 | No |
| 116 | PDE1B | phosphodiesterase 1B [Source:HGNC Symbol;Acc:HGNC:8775] | 27913 | -0.053 | -0.0639 | No |
| 117 | ADCY2 | adenylate cyclase 2 [Source:HGNC Symbol;Acc:HGNC:233] | 27981 | -0.053 | -0.0628 | No |
| 118 | AMPD1 | adenosine monophosphate deaminase 1 [Source:HGNC Symbol;Acc:HGNC:468] | 28108 | -0.054 | -0.0630 | No |
| 119 | IMPDH1 | inosine monophosphate dehydrogenase 1 [Source:HGNC Symbol;Acc:HGNC:6052] | 28627 | -0.058 | -0.0731 | No |
| 120 | NT5C3A | "5'-nucleotidase, cytosolic IIIA [Source:HGNC Symbol;Acc:HGNC:17820]" | 28729 | -0.059 | -0.0725 | No |
| 121 | PDE1C | phosphodiesterase 1C [Source:HGNC Symbol;Acc:HGNC:8776] | 30211 | -0.073 | -0.1063 | No |
| 122 | ENTPD1 | ectonucleoside triphosphate diphosphohydrolase 1 [Source:HGNC Symbol;Acc:HGNC:3363] | 30394 | -0.075 | -0.1069 | No |
| 123 | ENPP1 | ectonucleotide pyrophosphatase/phosphodiesterase 1 [Source:HGNC Symbol;Acc:HGNC:3356] | 30444 | -0.075 | -0.1041 | No |
| 124 | GMPS | guanine monophosphate synthase [Source:HGNC Symbol;Acc:HGNC:4378] | 30756 | -0.078 | -0.1078 | No |
| 125 | GUCY2D | "guanylate cyclase 2D, retinal [Source:HGNC Symbol;Acc:HGNC:4689]" | 30980 | -0.080 | -0.1092 | No |
| 126 | POLR2D | RNA polymerase II subunit D [Source:HGNC Symbol;Acc:HGNC:9191] | 31342 | -0.084 | -0.1139 | No |
| 127 | GUCY1A2 | guanylate cyclase 1 soluble subunit alpha 2 [Source:HGNC Symbol;Acc:HGNC:4684] | 32385 | -0.095 | -0.1353 | No |
| 128 | ADA | adenosine deaminase [Source:HGNC Symbol;Acc:HGNC:186] | 32406 | -0.096 | -0.1306 | No |
| 129 | POLR1B | RNA polymerase I subunit B [Source:HGNC Symbol;Acc:HGNC:20454] | 32596 | -0.098 | -0.1301 | No |
| 130 | PDE6G | phosphodiesterase 6G [Source:HGNC Symbol;Acc:HGNC:8789] | 33043 | -0.104 | -0.1359 | No |
| 131 | AMPD3 | adenosine monophosphate deaminase 3 [Source:HGNC Symbol;Acc:HGNC:470] | 33161 | -0.106 | -0.1331 | No |
| 132 | PDE8B | phosphodiesterase 8B [Source:HGNC Symbol;Acc:HGNC:8794] | 33272 | -0.107 | -0.1301 | No |
| 133 | DCK | deoxycytidine kinase [Source:HGNC Symbol;Acc:HGNC:2704] | 33786 | -0.115 | -0.1370 | No |
| 134 | AK7 | adenylate kinase 7 [Source:HGNC Symbol;Acc:HGNC:20091] | 33921 | -0.117 | -0.1341 | No |
| 135 | POLA1 | "DNA polymerase alpha 1, catalytic subunit [Source:HGNC Symbol;Acc:HGNC:9173]" | 34512 | -0.126 | -0.1423 | No |
| 136 | POLE3 | "DNA polymerase epsilon 3, accessory subunit [Source:HGNC Symbol;Acc:HGNC:13546]" | 34528 | -0.126 | -0.1358 | No |
| 137 | ADCY7 | adenylate cyclase 7 [Source:HGNC Symbol;Acc:HGNC:238] | 34661 | -0.129 | -0.1322 | No |
| 138 | ADCY4 | adenylate cyclase 4 [Source:HGNC Symbol;Acc:HGNC:235] | 34840 | -0.132 | -0.1296 | No |
| 139 | GUCY1A1 | guanylate cyclase 1 soluble subunit alpha 1 [Source:HGNC Symbol;Acc:HGNC:4685] | 35261 | -0.141 | -0.1327 | No |
| 140 | ALLC | allantoicase [Source:HGNC Symbol;Acc:HGNC:17377] | 35360 | -0.143 | -0.1274 | No |
| 141 | ADCY9 | adenylate cyclase 9 [Source:HGNC Symbol;Acc:HGNC:240] | 35385 | -0.144 | -0.1202 | No |
| 142 | ADSS2 | adenylosuccinate synthase 2 [Source:HGNC Symbol;Acc:HGNC:292] | 35734 | -0.153 | -0.1208 | No |
| 143 | PDE4A | phosphodiesterase 4A [Source:HGNC Symbol;Acc:HGNC:8780] | 35869 | -0.157 | -0.1157 | No |
| 144 | GMPR | guanosine monophosphate reductase [Source:HGNC Symbol;Acc:HGNC:4376] | 35874 | -0.157 | -0.1073 | No |
| 145 | GUCY1B1 | guanylate cyclase 1 soluble subunit beta 1 [Source:HGNC Symbol;Acc:HGNC:4687] | 36017 | -0.161 | -0.1022 | No |
| 146 | PDE4D | phosphodiesterase 4D [Source:HGNC Symbol;Acc:HGNC:8783] | 36259 | -0.168 | -0.0992 | No |
| 147 | PDE4B | phosphodiesterase 4B [Source:HGNC Symbol;Acc:HGNC:8781] | 36529 | -0.178 | -0.0964 | No |
| 148 | NT5C1B | "5'-nucleotidase, cytosolic IB [Source:HGNC Symbol;Acc:HGNC:17818]" | 36679 | -0.183 | -0.0903 | No |
| 149 | PDE5A | phosphodiesterase 5A [Source:HGNC Symbol;Acc:HGNC:8784] | 36767 | -0.187 | -0.0824 | No |
| 150 | ENTPD4 | ectonucleoside triphosphate diphosphohydrolase 4 [Source:HGNC Symbol;Acc:HGNC:14573] | 37024 | -0.198 | -0.0781 | No |
| 151 | ENTPD3 | ectonucleoside triphosphate diphosphohydrolase 3 [Source:HGNC Symbol;Acc:HGNC:3365] | 37619 | -0.232 | -0.0807 | No |
| 152 | ADCY5 | adenylate cyclase 5 [Source:HGNC Symbol;Acc:HGNC:236] | 37762 | -0.242 | -0.0712 | No |
| 153 | RRM1 | ribonucleotide reductase catalytic subunit M1 [Source:HGNC Symbol;Acc:HGNC:10451] | 37888 | -0.253 | -0.0606 | No |
| 154 | GDA | guanine deaminase [Source:HGNC Symbol;Acc:HGNC:4212] | 38023 | -0.267 | -0.0495 | No |
| 155 | ADCY10 | adenylate cyclase 10 [Source:HGNC Symbol;Acc:HGNC:21285] | 38118 | -0.280 | -0.0367 | No |
| 156 | NPR2 | natriuretic peptide receptor 2 [Source:HGNC Symbol;Acc:HGNC:7944] | 38193 | -0.292 | -0.0228 | No |
| 157 | ADCY8 | adenylate cyclase 8 [Source:HGNC Symbol;Acc:HGNC:239] | 38268 | -0.302 | -0.0082 | No |
| 158 | PDE3B | phosphodiesterase 3B [Source:HGNC Symbol;Acc:HGNC:8779] | 38314 | -0.310 | 0.0074 | No |
| 159 | ADCY3 | adenylate cyclase 3 [Source:HGNC Symbol;Acc:HGNC:234] | 38423 | -0.337 | 0.0230 | No |
Table: GSEA details [plain text format]

  

Fig 2: KEGG\_PURINE\_METABOLISM      
 Blue-Pink O' Gram in the Space of the Analyzed GeneSet

  

Fig 3: KEGG\_PURINE\_METABOLISM: Random ES distribution      
 Gene set null distribution of ES for **KEGG\_PURINE\_METABOLISM**
