# Supplementary material for: Simultaneous Presentation of Multiple Myeloma and Lung Cancer: Case Report and Gene Bioinformatics Analysis
Source: Front Oncol. 2022 Jun 13;12:859735. doi: 10.3389/fonc.2022.859735 (PMC9235397; doi:10.3389/fonc.2022.859735)
Supplement: Supplementary file 1 [file DataSheet_1.zip › The bioinformatic analysis of MM and lung cancer supplementary materials/Enrichment analysis/MECR/GSEA_4.1.0/LUSC TCGA/KEGG.Gsea.1639042374131/KEGG_PYRIMIDINE_METABOLISM.html]

Details for gene set KEGG\_PYRIMIDINE\_METABOLISM[GSEA]

|  || Dataset | ExpData\_collapsed\_to\_symbols.ENSG00000116353\_profile\_in\_ExpData.cls #ENSG00000116353 |
| Phenotype | ENSG00000116353\_profile\_in\_ExpData.cls#ENSG00000116353 |
| Upregulated in class | ENSG00000116353\_pos |
| GeneSet | KEGG\_PYRIMIDINE\_METABOLISM |
| Enrichment Score (ES) | 0.5162241 |
| Normalized Enrichment Score (NES) | 2.1888797 |
| Nominal p-value | 0.0 |
| FDR q-value | 1.9526716E-4 |
| FWER p-Value | 0.002 |
Table: GSEA Results Summary

  

Fig 1: Enrichment plot: KEGG\_PYRIMIDINE\_METABOLISM      
 Profile of the Running ES Score & Positions of GeneSet Members on the Rank Ordered List

  

| SYMBOL | TITLE | RANK IN GENE LIST | RANK METRIC SCORE | RUNNING ES | CORE ENRICHMENT || 1 | POLR1H | RNA polymerase I subunit H [Source:HGNC Symbol;Acc:HGNC:13182] | 172 | 0.352 | 0.0262 | Yes |
| 2 | POLR2K | "RNA polymerase II, I and III subunit K [Source:HGNC Symbol;Acc:HGNC:9198]" | 217 | 0.333 | 0.0539 | Yes |
| 3 | POLR2L | "RNA polymerase II, I and III subunit L [Source:HGNC Symbol;Acc:HGNC:9199]" | 221 | 0.331 | 0.0826 | Yes |
| 4 | CMPK1 | cytidine/uridine monophosphate kinase 1 [Source:HGNC Symbol;Acc:HGNC:18170] | 372 | 0.291 | 0.1041 | Yes |
| 5 | CTPS1 | CTP synthase 1 [Source:HGNC Symbol;Acc:HGNC:2519] | 409 | 0.284 | 0.1278 | Yes |
| 6 | POLR2G | RNA polymerase II subunit G [Source:HGNC Symbol;Acc:HGNC:9194] | 461 | 0.275 | 0.1504 | Yes |
| 7 | DTYMK | deoxythymidylate kinase [Source:HGNC Symbol;Acc:HGNC:3061] | 463 | 0.275 | 0.1742 | Yes |
| 8 | ITPA | inosine triphosphatase [Source:HGNC Symbol;Acc:HGNC:6176] | 482 | 0.273 | 0.1974 | Yes |
| 9 | NME7 | NME/NM23 family member 7 [Source:HGNC Symbol;Acc:HGNC:20461] | 531 | 0.265 | 0.2191 | Yes |
| 10 | NME3 | NME/NM23 nucleoside diphosphate kinase 3 [Source:HGNC Symbol;Acc:HGNC:7851] | 657 | 0.250 | 0.2376 | Yes |
| 11 | POLE4 | "DNA polymerase epsilon 4, accessory subunit [Source:HGNC Symbol;Acc:HGNC:18755]" | 759 | 0.237 | 0.2556 | Yes |
| 12 | UCKL1 | uridine-cytidine kinase 1 like 1 [Source:HGNC Symbol;Acc:HGNC:15938] | 781 | 0.234 | 0.2754 | Yes |
| 13 | POLR2I | RNA polymerase II subunit I [Source:HGNC Symbol;Acc:HGNC:9196] | 1025 | 0.213 | 0.2877 | Yes |
| 14 | NME1-NME2 | NME1-NME2 readthrough [Source:HGNC Symbol;Acc:HGNC:33531] | 1182 | 0.204 | 0.3014 | Yes |
| 15 | NME4 | NME/NM23 nucleoside diphosphate kinase 4 [Source:HGNC Symbol;Acc:HGNC:7852] | 1206 | 0.203 | 0.3183 | Yes |
| 16 | POLR2J | RNA polymerase II subunit J [Source:HGNC Symbol;Acc:HGNC:9197] | 1233 | 0.201 | 0.3351 | Yes |
| 17 | POLR3A | RNA polymerase III subunit A [Source:HGNC Symbol;Acc:HGNC:30074] | 1258 | 0.200 | 0.3518 | Yes |
| 18 | NME6 | NME/NM23 nucleoside diphosphate kinase 6 [Source:HGNC Symbol;Acc:HGNC:20567] | 1304 | 0.197 | 0.3678 | Yes |
| 19 | POLD1 | "DNA polymerase delta 1, catalytic subunit [Source:HGNC Symbol;Acc:HGNC:9175]" | 1356 | 0.194 | 0.3833 | Yes |
| 20 | POLE2 | "DNA polymerase epsilon 2, accessory subunit [Source:HGNC Symbol;Acc:HGNC:9178]" | 1540 | 0.184 | 0.3946 | Yes |
| 21 | NME1 | NME/NM23 nucleoside diphosphate kinase 1 [Source:HGNC Symbol;Acc:HGNC:7849] | 1592 | 0.182 | 0.4092 | Yes |
| 22 | DHODH | dihydroorotate dehydrogenase (quinone) [Source:HGNC Symbol;Acc:HGNC:2867] | 1658 | 0.180 | 0.4231 | Yes |
| 23 | TXNRD2 | thioredoxin reductase 2 [Source:HGNC Symbol;Acc:HGNC:18155] | 1939 | 0.168 | 0.4305 | Yes |
| 24 | POLR3B | RNA polymerase III subunit B [Source:HGNC Symbol;Acc:HGNC:30348] | 2133 | 0.161 | 0.4396 | Yes |
| 25 | ENTPD5 | ectonucleoside triphosphate diphosphohydrolase 5 (inactive) [Source:HGNC Symbol;Acc:HGNC:3367] | 2456 | 0.150 | 0.4444 | Yes |
| 26 | POLR1C | RNA polymerase I and III subunit C [Source:HGNC Symbol;Acc:HGNC:20194] | 2536 | 0.149 | 0.4553 | Yes |
| 27 | POLR3K | RNA polymerase III subunit K [Source:HGNC Symbol;Acc:HGNC:14121] | 2767 | 0.142 | 0.4618 | Yes |
| 28 | POLR2C | RNA polymerase II subunit C [Source:HGNC Symbol;Acc:HGNC:9189] | 2813 | 0.141 | 0.4729 | Yes |
| 29 | DPYD | dihydropyrimidine dehydrogenase [Source:HGNC Symbol;Acc:HGNC:3012] | 3114 | 0.134 | 0.4768 | Yes |
| 30 | POLR3G | RNA polymerase III subunit G [Source:HGNC Symbol;Acc:HGNC:30075] | 3270 | 0.131 | 0.4842 | Yes |
| 31 | PNP | purine nucleoside phosphorylase [Source:HGNC Symbol;Acc:HGNC:7892] | 3344 | 0.129 | 0.4936 | Yes |
| 32 | POLD4 | "DNA polymerase delta 4, accessory subunit [Source:HGNC Symbol;Acc:HGNC:14106]" | 3573 | 0.125 | 0.4986 | Yes |
| 33 | POLR3H | RNA polymerase III subunit H [Source:HGNC Symbol;Acc:HGNC:30349] | 3690 | 0.123 | 0.5063 | Yes |
| 34 | POLR2F | "RNA polymerase II, I and III subunit F [Source:HGNC Symbol;Acc:HGNC:9193]" | 3719 | 0.122 | 0.5162 | Yes |
| 35 | NT5C | "5', 3'-nucleotidase, cytosolic [Source:HGNC Symbol;Acc:HGNC:17144]" | 4125 | 0.116 | 0.5159 | No |
| 36 | NME2 | NME/NM23 nucleoside diphosphate kinase 2 [Source:HGNC Symbol;Acc:HGNC:7850] | 4705 | 0.107 | 0.5104 | No |
| 37 | CAD | "carbamoyl-phosphate synthetase 2, aspartate transcarbamylase, and dihydroorotase [Source:HGNC Symbol;Acc:HGNC:1424]" | 5372 | 0.098 | 0.5020 | No |
| 38 | ENTPD6 | ectonucleoside triphosphate diphosphohydrolase 6 [Source:HGNC Symbol;Acc:HGNC:3368] | 6358 | 0.090 | 0.4847 | No |
| 39 | TYMS | thymidylate synthetase [Source:HGNC Symbol;Acc:HGNC:12441] | 7032 | 0.083 | 0.4747 | No |
| 40 | NT5C2 | "5'-nucleotidase, cytosolic II [Source:HGNC Symbol;Acc:HGNC:8022]" | 7091 | 0.083 | 0.4805 | No |
| 41 | POLD2 | "DNA polymerase delta 2, accessory subunit [Source:HGNC Symbol;Acc:HGNC:9176]" | 7505 | 0.079 | 0.4768 | No |
| 42 | NME5 | NME/NM23 family member 5 [Source:HGNC Symbol;Acc:HGNC:7853] | 7564 | 0.079 | 0.4821 | No |
| 43 | UCK2 | uridine-cytidine kinase 2 [Source:HGNC Symbol;Acc:HGNC:12562] | 7657 | 0.078 | 0.4865 | No |
| 44 | POLR2E | "RNA polymerase II, I and III subunit E [Source:HGNC Symbol;Acc:HGNC:9192]" | 7792 | 0.077 | 0.4898 | No |
| 45 | TYMP | thymidine phosphorylase [Source:HGNC Symbol;Acc:HGNC:3148] | 8165 | 0.074 | 0.4867 | No |
| 46 | POLR3D | RNA polymerase III subunit D [Source:HGNC Symbol;Acc:HGNC:1080] | 8233 | 0.073 | 0.4913 | No |
| 47 | NT5E | 5'-nucleotidase ecto [Source:HGNC Symbol;Acc:HGNC:8021] | 8757 | 0.069 | 0.4839 | No |
| 48 | UPB1 | beta-ureidopropionase 1 [Source:HGNC Symbol;Acc:HGNC:16297] | 8852 | 0.068 | 0.4874 | No |
| 49 | POLR3GL | RNA polymerase III subunit GL [Source:HGNC Symbol;Acc:HGNC:28466] | 9791 | 0.062 | 0.4689 | No |
| 50 | POLR1D | RNA polymerase I and III subunit D [Source:HGNC Symbol;Acc:HGNC:20422] | 10132 | 0.059 | 0.4653 | No |
| 51 | TK2 | thymidine kinase 2 [Source:HGNC Symbol;Acc:HGNC:11831] | 10200 | 0.059 | 0.4687 | No |
| 52 | PRIM1 | DNA primase subunit 1 [Source:HGNC Symbol;Acc:HGNC:9369] | 11631 | 0.050 | 0.4366 | No |
| 53 | POLR3C | RNA polymerase III subunit C [Source:HGNC Symbol;Acc:HGNC:30076] | 12617 | 0.043 | 0.4152 | No |
| 54 | POLR2B | RNA polymerase II subunit B [Source:HGNC Symbol;Acc:HGNC:9188] | 13423 | 0.038 | 0.3980 | No |
| 55 | POLR1A | RNA polymerase I subunit A [Source:HGNC Symbol;Acc:HGNC:17264] | 13512 | 0.038 | 0.3991 | No |
| 56 | CANT1 | calcium activated nucleotidase 1 [Source:HGNC Symbol;Acc:HGNC:19721] | 13912 | 0.036 | 0.3920 | No |
| 57 | POLR1E | RNA polymerase I subunit E [Source:HGNC Symbol;Acc:HGNC:17631] | 15209 | 0.028 | 0.3614 | No |
| 58 | TK1 | thymidine kinase 1 [Source:HGNC Symbol;Acc:HGNC:11830] | 15745 | 0.025 | 0.3499 | No |
| 59 | POLD3 | "DNA polymerase delta 3, accessory subunit [Source:HGNC Symbol;Acc:HGNC:20932]" | 16528 | 0.020 | 0.3317 | No |
| 60 | DCTD | dCMP deaminase [Source:HGNC Symbol;Acc:HGNC:2710] | 16814 | 0.019 | 0.3261 | No |
| 61 | POLR3F | RNA polymerase III subunit F [Source:HGNC Symbol;Acc:HGNC:15763] | 17157 | 0.017 | 0.3188 | No |
| 62 | NUDT2 | nudix hydrolase 2 [Source:HGNC Symbol;Acc:HGNC:8049] | 17767 | 0.013 | 0.3045 | No |
| 63 | ENTPD8 | ectonucleoside triphosphate diphosphohydrolase 8 [Source:HGNC Symbol;Acc:HGNC:24860] | 17776 | 0.013 | 0.3054 | No |
| 64 | PRIM2 | DNA primase subunit 2 [Source:HGNC Symbol;Acc:HGNC:9370] | 18680 | 0.008 | 0.2831 | No |
| 65 | UPP1 | uridine phosphorylase 1 [Source:HGNC Symbol;Acc:HGNC:12576] | 18816 | 0.008 | 0.2803 | No |
| 66 | POLE | "DNA polymerase epsilon, catalytic subunit [Source:HGNC Symbol;Acc:HGNC:9177]" | 19623 | 0.003 | 0.2600 | No |
| 67 | DPYS | dihydropyrimidinase [Source:HGNC Symbol;Acc:HGNC:3013] | 20185 | -0.000 | 0.2458 | No |
| 68 | POLR2J2 | RNA polymerase II subunit J2 [Source:HGNC Symbol;Acc:HGNC:23208] | 20932 | -0.005 | 0.2272 | No |
| 69 | UMPS | uridine monophosphate synthetase [Source:HGNC Symbol;Acc:HGNC:12563] | 21508 | -0.008 | 0.2132 | No |
| 70 | CDA | cytidine deaminase [Source:HGNC Symbol;Acc:HGNC:1712] | 22423 | -0.014 | 0.1911 | No |
| 71 | NT5C1A | "5'-nucleotidase, cytosolic IA [Source:HGNC Symbol;Acc:HGNC:17819]" | 23150 | -0.018 | 0.1742 | No |
| 72 | PNPT1 | polyribonucleotide nucleotidyltransferase 1 [Source:HGNC Symbol;Acc:HGNC:23166] | 23526 | -0.021 | 0.1665 | No |
| 73 | UPP2 | uridine phosphorylase 2 [Source:HGNC Symbol;Acc:HGNC:23061] | 23846 | -0.023 | 0.1603 | No |
| 74 | RRM2 | ribonucleotide reductase regulatory subunit M2 [Source:HGNC Symbol;Acc:HGNC:10452] | 23886 | -0.023 | 0.1613 | No |
| 75 | POLR2A | RNA polymerase II subunit A [Source:HGNC Symbol;Acc:HGNC:9187] | 24164 | -0.025 | 0.1564 | No |
| 76 | RRM2B | ribonucleotide reductase regulatory TP53 inducible subunit M2B [Source:HGNC Symbol;Acc:HGNC:17296] | 24378 | -0.026 | 0.1533 | No |
| 77 | POLA2 | "DNA polymerase alpha 2, accessory subunit [Source:HGNC Symbol;Acc:HGNC:30073]" | 24419 | -0.027 | 0.1546 | No |
| 78 | NT5M | "5',3'-nucleotidase, mitochondrial [Source:HGNC Symbol;Acc:HGNC:15769]" | 25355 | -0.034 | 0.1336 | No |
| 79 | POLR2J3 | RNA polymerase II subunit J3 [Source:HGNC Symbol;Acc:HGNC:33853] | 25994 | -0.038 | 0.1207 | No |
| 80 | POLR2H | "RNA polymerase II, I and III subunit H [Source:HGNC Symbol;Acc:HGNC:9195]" | 26753 | -0.044 | 0.1051 | No |
| 81 | NT5C3A | "5'-nucleotidase, cytosolic IIIA [Source:HGNC Symbol;Acc:HGNC:17820]" | 28729 | -0.059 | 0.0599 | No |
| 82 | DUT | deoxyuridine triphosphatase [Source:HGNC Symbol;Acc:HGNC:3078] | 29836 | -0.069 | 0.0377 | No |
| 83 | ENTPD1 | ectonucleoside triphosphate diphosphohydrolase 1 [Source:HGNC Symbol;Acc:HGNC:3363] | 30394 | -0.075 | 0.0300 | No |
| 84 | POLR2D | RNA polymerase II subunit D [Source:HGNC Symbol;Acc:HGNC:9191] | 31342 | -0.084 | 0.0131 | No |
| 85 | CMPK2 | cytidine/uridine monophosphate kinase 2 [Source:HGNC Symbol;Acc:HGNC:27015] | 31352 | -0.084 | 0.0202 | No |
| 86 | AK3 | adenylate kinase 3 [Source:HGNC Symbol;Acc:HGNC:17376] | 31382 | -0.084 | 0.0267 | No |
| 87 | CTPS2 | CTP synthase 2 [Source:HGNC Symbol;Acc:HGNC:2520] | 32326 | -0.095 | 0.0109 | No |
| 88 | POLR1B | RNA polymerase I subunit B [Source:HGNC Symbol;Acc:HGNC:20454] | 32596 | -0.098 | 0.0125 | No |
| 89 | UCK1 | uridine-cytidine kinase 1 [Source:HGNC Symbol;Acc:HGNC:14859] | 32598 | -0.098 | 0.0210 | No |
| 90 | DCK | deoxycytidine kinase [Source:HGNC Symbol;Acc:HGNC:2704] | 33786 | -0.115 | 0.0007 | No |
| 91 | POLA1 | "DNA polymerase alpha 1, catalytic subunit [Source:HGNC Symbol;Acc:HGNC:9173]" | 34512 | -0.126 | -0.0069 | No |
| 92 | POLE3 | "DNA polymerase epsilon 3, accessory subunit [Source:HGNC Symbol;Acc:HGNC:13546]" | 34528 | -0.126 | 0.0037 | No |
| 93 | UPRT | uracil phosphoribosyltransferase homolog [Source:HGNC Symbol;Acc:HGNC:28334] | 36442 | -0.175 | -0.0299 | No |
| 94 | NT5C1B | "5'-nucleotidase, cytosolic IB [Source:HGNC Symbol;Acc:HGNC:17818]" | 36679 | -0.183 | -0.0201 | No |
| 95 | ENTPD4 | ectonucleoside triphosphate diphosphohydrolase 4 [Source:HGNC Symbol;Acc:HGNC:14573] | 37024 | -0.198 | -0.0116 | No |
| 96 | ENTPD3 | ectonucleoside triphosphate diphosphohydrolase 3 [Source:HGNC Symbol;Acc:HGNC:3365] | 37619 | -0.232 | -0.0066 | No |
| 97 | RRM1 | ribonucleotide reductase catalytic subunit M1 [Source:HGNC Symbol;Acc:HGNC:10451] | 37888 | -0.253 | 0.0085 | No |
| 98 | TXNRD1 | thioredoxin reductase 1 [Source:HGNC Symbol;Acc:HGNC:12437] | 38375 | -0.323 | 0.0242 | No |
Table: GSEA details [plain text format]

  

Fig 2: KEGG\_PYRIMIDINE\_METABOLISM      
 Blue-Pink O' Gram in the Space of the Analyzed GeneSet

  

Fig 3: KEGG\_PYRIMIDINE\_METABOLISM: Random ES distribution      
 Gene set null distribution of ES for **KEGG\_PYRIMIDINE\_METABOLISM**
