# Supplementary material for: Simultaneous Presentation of Multiple Myeloma and Lung Cancer: Case Report and Gene Bioinformatics Analysis
Source: Front Oncol. 2022 Jun 13;12:859735. doi: 10.3389/fonc.2022.859735 (PMC9235397; doi:10.3389/fonc.2022.859735)
Supplement: Supplementary file 1 [file DataSheet_1.zip › The bioinformatic analysis of MM and lung cancer supplementary materials/Enrichment analysis/MECR/GSEA_4.1.0/LUSC TCGA/KEGG.Gsea.1639042374131/KEGG_RIBOSOME.html]

Details for gene set KEGG\_RIBOSOME[GSEA]

|  || Dataset | ExpData\_collapsed\_to\_symbols.ENSG00000116353\_profile\_in\_ExpData.cls #ENSG00000116353 |
| Phenotype | ENSG00000116353\_profile\_in\_ExpData.cls#ENSG00000116353 |
| Upregulated in class | ENSG00000116353\_pos |
| GeneSet | KEGG\_RIBOSOME |
| Enrichment Score (ES) | 0.75606287 |
| Normalized Enrichment Score (NES) | 3.1539779 |
| Nominal p-value | 0.0 |
| FDR q-value | 0.0 |
| FWER p-Value | 0.0 |
Table: GSEA Results Summary

  

Fig 1: Enrichment plot: KEGG\_RIBOSOME      
 Profile of the Running ES Score & Positions of GeneSet Members on the Rank Ordered List

  

| SYMBOL | TITLE | RANK IN GENE LIST | RANK METRIC SCORE | RUNNING ES | CORE ENRICHMENT || 1 | RPL22 | ribosomal protein L22 [Source:HGNC Symbol;Acc:HGNC:10315] | 83 | 0.410 | 0.0287 | Yes |
| 2 | RPL11 | ribosomal protein L11 [Source:HGNC Symbol;Acc:HGNC:10301] | 105 | 0.392 | 0.0575 | Yes |
| 3 | RPS8 | ribosomal protein S8 [Source:HGNC Symbol;Acc:HGNC:10441] | 168 | 0.353 | 0.0824 | Yes |
| 4 | RPL8 | ribosomal protein L8 [Source:HGNC Symbol;Acc:HGNC:10368] | 256 | 0.321 | 0.1043 | Yes |
| 5 | RPL28 | ribosomal protein L28 [Source:HGNC Symbol;Acc:HGNC:10330] | 320 | 0.302 | 0.1254 | Yes |
| 6 | RPS21 | ribosomal protein S21 [Source:HGNC Symbol;Acc:HGNC:10409] | 445 | 0.278 | 0.1431 | Yes |
| 7 | RPL18 | ribosomal protein L18 [Source:HGNC Symbol;Acc:HGNC:10310] | 485 | 0.273 | 0.1626 | Yes |
| 8 | RPL5 | ribosomal protein L5 [Source:HGNC Symbol;Acc:HGNC:10360] | 490 | 0.272 | 0.1829 | Yes |
| 9 | RPS2 | ribosomal protein S2 [Source:HGNC Symbol;Acc:HGNC:10404] | 553 | 0.262 | 0.2010 | Yes |
| 10 | FAU | FAU ubiquitin like and ribosomal protein S30 fusion [Source:HGNC Symbol;Acc:HGNC:3597] | 568 | 0.261 | 0.2202 | Yes |
| 11 | RPS9 | ribosomal protein S9 [Source:HGNC Symbol;Acc:HGNC:10442] | 639 | 0.252 | 0.2373 | Yes |
| 12 | RPL30 | ribosomal protein L30 [Source:HGNC Symbol;Acc:HGNC:10333] | 678 | 0.247 | 0.2549 | Yes |
| 13 | RPS12 | ribosomal protein S12 [Source:HGNC Symbol;Acc:HGNC:10385] | 699 | 0.245 | 0.2728 | Yes |
| 14 | RPL37 | ribosomal protein L37 [Source:HGNC Symbol;Acc:HGNC:10347] | 753 | 0.238 | 0.2893 | Yes |
| 15 | RPS11 | ribosomal protein S11 [Source:HGNC Symbol;Acc:HGNC:10384] | 783 | 0.233 | 0.3061 | Yes |
| 16 | MRPL13 | mitochondrial ribosomal protein L13 [Source:HGNC Symbol;Acc:HGNC:14278] | 787 | 0.233 | 0.3235 | Yes |
| 17 | RPL13A | ribosomal protein L13a [Source:HGNC Symbol;Acc:HGNC:10304] | 799 | 0.232 | 0.3406 | Yes |
| 18 | RPS19 | ribosomal protein S19 [Source:HGNC Symbol;Acc:HGNC:10402] | 819 | 0.230 | 0.3573 | Yes |
| 19 | RPS20 | ribosomal protein S20 [Source:HGNC Symbol;Acc:HGNC:10405] | 842 | 0.227 | 0.3738 | Yes |
| 20 | RPS29 | ribosomal protein S29 [Source:HGNC Symbol;Acc:HGNC:10419] | 1034 | 0.212 | 0.3849 | Yes |
| 21 | RPS10 | ribosomal protein S10 [Source:HGNC Symbol;Acc:HGNC:10383] | 1159 | 0.205 | 0.3972 | Yes |
| 22 | RPL13 | ribosomal protein L13 [Source:HGNC Symbol;Acc:HGNC:10303] | 1335 | 0.195 | 0.4073 | Yes |
| 23 | RPS18 | ribosomal protein S18 [Source:HGNC Symbol;Acc:HGNC:10401] | 1348 | 0.194 | 0.4216 | Yes |
| 24 | RPS5 | ribosomal protein S5 [Source:HGNC Symbol;Acc:HGNC:10426] | 1371 | 0.193 | 0.4355 | Yes |
| 25 | RPS16 | ribosomal protein S16 [Source:HGNC Symbol;Acc:HGNC:10396] | 1570 | 0.183 | 0.4442 | Yes |
| 26 | RPL10 | ribosomal protein L10 [Source:HGNC Symbol;Acc:HGNC:10298] | 1584 | 0.183 | 0.4576 | Yes |
| 27 | RPL10A | ribosomal protein L10a [Source:HGNC Symbol;Acc:HGNC:10299] | 1652 | 0.180 | 0.4694 | Yes |
| 28 | RPL24 | ribosomal protein L24 [Source:HGNC Symbol;Acc:HGNC:10325] | 1663 | 0.180 | 0.4827 | Yes |
| 29 | RPL34 | ribosomal protein L34 [Source:HGNC Symbol;Acc:HGNC:10340] | 1711 | 0.177 | 0.4948 | Yes |
| 30 | RPL37A | ribosomal protein L37a [Source:HGNC Symbol;Acc:HGNC:10348] | 1769 | 0.174 | 0.5064 | Yes |
| 31 | RPL3 | ribosomal protein L3 [Source:HGNC Symbol;Acc:HGNC:10332] | 1795 | 0.174 | 0.5188 | Yes |
| 32 | RPS3A | ribosomal protein S3A [Source:HGNC Symbol;Acc:HGNC:10421] | 1812 | 0.173 | 0.5314 | Yes |
| 33 | RPLP2 | ribosomal protein lateral stalk subunit P2 [Source:HGNC Symbol;Acc:HGNC:10377] | 2051 | 0.164 | 0.5376 | Yes |
| 34 | RPSA | ribosomal protein SA [Source:HGNC Symbol;Acc:HGNC:6502] | 2077 | 0.163 | 0.5492 | Yes |
| 35 | RPL9 | ribosomal protein L9 [Source:HGNC Symbol;Acc:HGNC:10369] | 2279 | 0.156 | 0.5558 | Yes |
| 36 | RPS26 | ribosomal protein S26 [Source:HGNC Symbol;Acc:HGNC:10414] | 2316 | 0.155 | 0.5665 | Yes |
| 37 | RPL32 | ribosomal protein L32 [Source:HGNC Symbol;Acc:HGNC:10336] | 2333 | 0.154 | 0.5777 | Yes |
| 38 | RPL29 | ribosomal protein L29 [Source:HGNC Symbol;Acc:HGNC:10331] | 2414 | 0.152 | 0.5870 | Yes |
| 39 | RPL36A | ribosomal protein L36a [Source:HGNC Symbol;Acc:HGNC:10359] | 2450 | 0.151 | 0.5975 | Yes |
| 40 | RPLP0 | ribosomal protein lateral stalk subunit P0 [Source:HGNC Symbol;Acc:HGNC:10371] | 2564 | 0.148 | 0.6057 | Yes |
| 41 | RPS24 | ribosomal protein S24 [Source:HGNC Symbol;Acc:HGNC:10411] | 2568 | 0.148 | 0.6167 | Yes |
| 42 | RPS25 | ribosomal protein S25 [Source:HGNC Symbol;Acc:HGNC:10413] | 2672 | 0.145 | 0.6250 | Yes |
| 43 | RPL27A | ribosomal protein L27a [Source:HGNC Symbol;Acc:HGNC:10329] | 2817 | 0.141 | 0.6319 | Yes |
| 44 | RPS28 | ribosomal protein S28 [Source:HGNC Symbol;Acc:HGNC:10418] | 2836 | 0.141 | 0.6420 | Yes |
| 45 | RPL36 | ribosomal protein L36 [Source:HGNC Symbol;Acc:HGNC:13631] | 2881 | 0.140 | 0.6514 | Yes |
| 46 | RPL39 | ribosomal protein L39 [Source:HGNC Symbol;Acc:HGNC:10350] | 2911 | 0.139 | 0.6611 | Yes |
| 47 | RPL7 | ribosomal protein L7 [Source:HGNC Symbol;Acc:HGNC:10363] | 2932 | 0.138 | 0.6710 | Yes |
| 48 | RPS3 | ribosomal protein S3 [Source:HGNC Symbol;Acc:HGNC:10420] | 2935 | 0.138 | 0.6813 | Yes |
| 49 | RPLP1 | ribosomal protein lateral stalk subunit P1 [Source:HGNC Symbol;Acc:HGNC:10372] | 2951 | 0.138 | 0.6913 | Yes |
| 50 | RPS27L | ribosomal protein S27 like [Source:HGNC Symbol;Acc:HGNC:18476] | 3070 | 0.135 | 0.6984 | Yes |
| 51 | RPL14 | ribosomal protein L14 [Source:HGNC Symbol;Acc:HGNC:10305] | 3205 | 0.132 | 0.7049 | Yes |
| 52 | RPL36AL | ribosomal protein L36a like [Source:HGNC Symbol;Acc:HGNC:10346] | 3337 | 0.129 | 0.7113 | Yes |
| 53 | RPS7 | ribosomal protein S7 [Source:HGNC Symbol;Acc:HGNC:10440] | 3710 | 0.123 | 0.7110 | Yes |
| 54 | RPS27A | ribosomal protein S27a [Source:HGNC Symbol;Acc:HGNC:10417] | 3751 | 0.122 | 0.7191 | Yes |
| 55 | RPS23 | ribosomal protein S23 [Source:HGNC Symbol;Acc:HGNC:10410] | 3790 | 0.121 | 0.7273 | Yes |
| 56 | RPL18A | ribosomal protein L18a [Source:HGNC Symbol;Acc:HGNC:10311] | 3928 | 0.119 | 0.7327 | Yes |
| 57 | RPL10L | ribosomal protein L10 like [Source:HGNC Symbol;Acc:HGNC:17976] | 4100 | 0.116 | 0.7370 | Yes |
| 58 | RPL41 | ribosomal protein L41 [Source:HGNC Symbol;Acc:HGNC:10354] | 4136 | 0.115 | 0.7448 | Yes |
| 59 | RPS15A | ribosomal protein S15a [Source:HGNC Symbol;Acc:HGNC:10389] | 4174 | 0.115 | 0.7525 | Yes |
| 60 | RPS13 | ribosomal protein S13 [Source:HGNC Symbol;Acc:HGNC:10386] | 4364 | 0.112 | 0.7561 | Yes |
| 61 | RPL21 | ribosomal protein L21 [Source:HGNC Symbol;Acc:HGNC:10313] | 5050 | 0.102 | 0.7463 | No |
| 62 | RPL26L1 | ribosomal protein L26 like 1 [Source:HGNC Symbol;Acc:HGNC:17050] | 5281 | 0.099 | 0.7479 | No |
| 63 | RPL38 | ribosomal protein L38 [Source:HGNC Symbol;Acc:HGNC:10349] | 5948 | 0.095 | 0.7380 | No |
| 64 | UBA52 | ubiquitin A-52 residue ribosomal protein fusion product 1 [Source:HGNC Symbol;Acc:HGNC:12458] | 6366 | 0.090 | 0.7341 | No |
| 65 | RPS6 | ribosomal protein S6 [Source:HGNC Symbol;Acc:HGNC:10429] | 6848 | 0.085 | 0.7282 | No |
| 66 | RPL6 | ribosomal protein L6 [Source:HGNC Symbol;Acc:HGNC:10362] | 6988 | 0.084 | 0.7310 | No |
| 67 | RPL27 | ribosomal protein L27 [Source:HGNC Symbol;Acc:HGNC:10328] | 7140 | 0.082 | 0.7333 | No |
| 68 | RPL15 | ribosomal protein L15 [Source:HGNC Symbol;Acc:HGNC:10306] | 7371 | 0.080 | 0.7335 | No |
| 69 | RPS15 | ribosomal protein S15 [Source:HGNC Symbol;Acc:HGNC:10388] | 7802 | 0.077 | 0.7283 | No |
| 70 | RPL12 | ribosomal protein L12 [Source:HGNC Symbol;Acc:HGNC:10302] | 7908 | 0.076 | 0.7313 | No |
| 71 | RPS4X | ribosomal protein S4 X-linked [Source:HGNC Symbol;Acc:HGNC:10424] | 7950 | 0.075 | 0.7359 | No |
| 72 | RPL31 | ribosomal protein L31 [Source:HGNC Symbol;Acc:HGNC:10334] | 8198 | 0.073 | 0.7351 | No |
| 73 | RPL3L | ribosomal protein L3 like [Source:HGNC Symbol;Acc:HGNC:10351] | 8765 | 0.069 | 0.7258 | No |
| 74 | RPL4 | ribosomal protein L4 [Source:HGNC Symbol;Acc:HGNC:10353] | 9677 | 0.063 | 0.7073 | No |
| 75 | RPL35 | ribosomal protein L35 [Source:HGNC Symbol;Acc:HGNC:10344] | 10972 | 0.054 | 0.6784 | No |
| 76 | RPS17 | ribosomal protein S17 [Source:HGNC Symbol;Acc:HGNC:10397] | 11799 | 0.048 | 0.6610 | No |
| 77 | RPL35A | ribosomal protein L35a [Source:HGNC Symbol;Acc:HGNC:10345] | 12115 | 0.046 | 0.6564 | No |
| 78 | RPS27 | ribosomal protein S27 [Source:HGNC Symbol;Acc:HGNC:10416] | 12516 | 0.044 | 0.6495 | No |
| 79 | RPL26 | ribosomal protein L26 [Source:HGNC Symbol;Acc:HGNC:10327] | 14718 | 0.031 | 0.5957 | No |
| 80 | RPS4Y1 | ribosomal protein S4 Y-linked 1 [Source:HGNC Symbol;Acc:HGNC:10425] | 14784 | 0.030 | 0.5964 | No |
| 81 | RPL17 | ribosomal protein L17 [Source:HGNC Symbol;Acc:HGNC:10307] | 15465 | 0.026 | 0.5810 | No |
| 82 | RSL24D1 | ribosomal L24 domain containing 1 [Source:HGNC Symbol;Acc:HGNC:18479] | 15600 | 0.026 | 0.5795 | No |
| 83 | RPL23A | ribosomal protein L23a [Source:HGNC Symbol;Acc:HGNC:10317] | 18415 | 0.010 | 0.5086 | No |
| 84 | RPL19 | ribosomal protein L19 [Source:HGNC Symbol;Acc:HGNC:10312] | 21058 | -0.006 | 0.4417 | No |
| 85 | RPL7A | ribosomal protein L7a [Source:HGNC Symbol;Acc:HGNC:10364] | 23552 | -0.021 | 0.3797 | No |
| 86 | RPL23 | ribosomal protein L23 [Source:HGNC Symbol;Acc:HGNC:10316] | 24510 | -0.027 | 0.3574 | No |
| 87 | RSL24D1P11 | ribosomal L24 domain containing 1 pseudogene 11 [Source:HGNC Symbol;Acc:HGNC:37881] | 26956 | -0.045 | 0.2984 | No |
| 88 | RPL22L1 | ribosomal protein L22 like 1 [Source:HGNC Symbol;Acc:HGNC:27610] | 37488 | -0.223 | 0.0468 | No |
Table: GSEA details [plain text format]

  

Fig 2: KEGG\_RIBOSOME      
 Blue-Pink O' Gram in the Space of the Analyzed GeneSet

  

Fig 3: KEGG\_RIBOSOME: Random ES distribution      
 Gene set null distribution of ES for **KEGG\_RIBOSOME**
