# Supplementary material for: Simultaneous Presentation of Multiple Myeloma and Lung Cancer: Case Report and Gene Bioinformatics Analysis
Source: Front Oncol. 2022 Jun 13;12:859735. doi: 10.3389/fonc.2022.859735 (PMC9235397; doi:10.3389/fonc.2022.859735)
Supplement: Supplementary file 1 [file DataSheet_1.zip › The bioinformatic analysis of MM and lung cancer supplementary materials/Enrichment analysis/MECR/GSEA_4.1.0/LUSC TCGA/KEGG.Gsea.1639042374131/KEGG_RNA_POLYMERASE.html]

Details for gene set KEGG\_RNA\_POLYMERASE[GSEA]

|  || Dataset | ExpData\_collapsed\_to\_symbols.ENSG00000116353\_profile\_in\_ExpData.cls #ENSG00000116353 |
| Phenotype | ENSG00000116353\_profile\_in\_ExpData.cls#ENSG00000116353 |
| Upregulated in class | ENSG00000116353\_pos |
| GeneSet | KEGG\_RNA\_POLYMERASE |
| Enrichment Score (ES) | 0.7036377 |
| Normalized Enrichment Score (NES) | 2.3490288 |
| Nominal p-value | 0.0 |
| FDR q-value | 0.0 |
| FWER p-Value | 0.0 |
Table: GSEA Results Summary

  

Fig 1: Enrichment plot: KEGG\_RNA\_POLYMERASE      
 Profile of the Running ES Score & Positions of GeneSet Members on the Rank Ordered List

  

| SYMBOL | TITLE | RANK IN GENE LIST | RANK METRIC SCORE | RUNNING ES | CORE ENRICHMENT || 1 | POLR1H | RNA polymerase I subunit H [Source:HGNC Symbol;Acc:HGNC:13182] | 172 | 0.352 | 0.0934 | Yes |
| 2 | POLR2K | "RNA polymerase II, I and III subunit K [Source:HGNC Symbol;Acc:HGNC:9198]" | 217 | 0.333 | 0.1848 | Yes |
| 3 | POLR2L | "RNA polymerase II, I and III subunit L [Source:HGNC Symbol;Acc:HGNC:9199]" | 221 | 0.331 | 0.2766 | Yes |
| 4 | POLR2G | RNA polymerase II subunit G [Source:HGNC Symbol;Acc:HGNC:9194] | 461 | 0.275 | 0.3470 | Yes |
| 5 | POLR2I | RNA polymerase II subunit I [Source:HGNC Symbol;Acc:HGNC:9196] | 1025 | 0.213 | 0.3917 | Yes |
| 6 | POLR2J | RNA polymerase II subunit J [Source:HGNC Symbol;Acc:HGNC:9197] | 1233 | 0.201 | 0.4423 | Yes |
| 7 | POLR3A | RNA polymerase III subunit A [Source:HGNC Symbol;Acc:HGNC:30074] | 1258 | 0.200 | 0.4971 | Yes |
| 8 | POLR3B | RNA polymerase III subunit B [Source:HGNC Symbol;Acc:HGNC:30348] | 2133 | 0.161 | 0.5195 | Yes |
| 9 | POLR1C | RNA polymerase I and III subunit C [Source:HGNC Symbol;Acc:HGNC:20194] | 2536 | 0.149 | 0.5505 | Yes |
| 10 | POLR3K | RNA polymerase III subunit K [Source:HGNC Symbol;Acc:HGNC:14121] | 2767 | 0.142 | 0.5841 | Yes |
| 11 | POLR2C | RNA polymerase II subunit C [Source:HGNC Symbol;Acc:HGNC:9189] | 2813 | 0.141 | 0.6222 | Yes |
| 12 | POLR3G | RNA polymerase III subunit G [Source:HGNC Symbol;Acc:HGNC:30075] | 3270 | 0.131 | 0.6469 | Yes |
| 13 | POLR3H | RNA polymerase III subunit H [Source:HGNC Symbol;Acc:HGNC:30349] | 3690 | 0.123 | 0.6704 | Yes |
| 14 | POLR2F | "RNA polymerase II, I and III subunit F [Source:HGNC Symbol;Acc:HGNC:9193]" | 3719 | 0.122 | 0.7036 | Yes |
| 15 | POLR2E | "RNA polymerase II, I and III subunit E [Source:HGNC Symbol;Acc:HGNC:9192]" | 7792 | 0.077 | 0.6213 | No |
| 16 | POLR3D | RNA polymerase III subunit D [Source:HGNC Symbol;Acc:HGNC:1080] | 8233 | 0.073 | 0.6303 | No |
| 17 | POLR3GL | RNA polymerase III subunit GL [Source:HGNC Symbol;Acc:HGNC:28466] | 9791 | 0.062 | 0.6078 | No |
| 18 | POLR1D | RNA polymerase I and III subunit D [Source:HGNC Symbol;Acc:HGNC:20422] | 10132 | 0.059 | 0.6156 | No |
| 19 | POLR3C | RNA polymerase III subunit C [Source:HGNC Symbol;Acc:HGNC:30076] | 12617 | 0.043 | 0.5643 | No |
| 20 | POLR2B | RNA polymerase II subunit B [Source:HGNC Symbol;Acc:HGNC:9188] | 13423 | 0.038 | 0.5545 | No |
| 21 | POLR1A | RNA polymerase I subunit A [Source:HGNC Symbol;Acc:HGNC:17264] | 13512 | 0.038 | 0.5628 | No |
| 22 | POLR1E | RNA polymerase I subunit E [Source:HGNC Symbol;Acc:HGNC:17631] | 15209 | 0.028 | 0.5275 | No |
| 23 | POLR3F | RNA polymerase III subunit F [Source:HGNC Symbol;Acc:HGNC:15763] | 17157 | 0.017 | 0.4826 | No |
| 24 | POLR2J2 | RNA polymerase II subunit J2 [Source:HGNC Symbol;Acc:HGNC:23208] | 20932 | -0.005 | 0.3879 | No |
| 25 | POLR2A | RNA polymerase II subunit A [Source:HGNC Symbol;Acc:HGNC:9187] | 24164 | -0.025 | 0.3126 | No |
| 26 | POLR2J3 | RNA polymerase II subunit J3 [Source:HGNC Symbol;Acc:HGNC:33853] | 25994 | -0.038 | 0.2766 | No |
| 27 | POLR2H | "RNA polymerase II, I and III subunit H [Source:HGNC Symbol;Acc:HGNC:9195]" | 26753 | -0.044 | 0.2694 | No |
| 28 | POLR2D | RNA polymerase II subunit D [Source:HGNC Symbol;Acc:HGNC:9191] | 31342 | -0.084 | 0.1759 | No |
| 29 | POLR1B | RNA polymerase I subunit B [Source:HGNC Symbol;Acc:HGNC:20454] | 32596 | -0.098 | 0.1712 | No |
Table: GSEA details [plain text format]

  

Fig 2: KEGG\_RNA\_POLYMERASE      
 Blue-Pink O' Gram in the Space of the Analyzed GeneSet

  

Fig 3: KEGG\_RNA\_POLYMERASE: Random ES distribution      
 Gene set null distribution of ES for **KEGG\_RNA\_POLYMERASE**
