# Supplementary material for: Simultaneous Presentation of Multiple Myeloma and Lung Cancer: Case Report and Gene Bioinformatics Analysis
Source: Front Oncol. 2022 Jun 13;12:859735. doi: 10.3389/fonc.2022.859735 (PMC9235397; doi:10.3389/fonc.2022.859735)
Supplement: Supplementary file 1 [file DataSheet_1.zip › The bioinformatic analysis of MM and lung cancer supplementary materials/Enrichment analysis/MECR/GSEA_4.1.0/LUSC TCGA/KEGG.Gsea.1639042374131/KEGG_SELENOAMINO_ACID_METABOLISM.html]

Details for gene set KEGG\_SELENOAMINO\_ACID\_METABOLISM[GSEA]

|  || Dataset | ExpData\_collapsed\_to\_symbols.ENSG00000116353\_profile\_in\_ExpData.cls #ENSG00000116353 |
| Phenotype | ENSG00000116353\_profile\_in\_ExpData.cls#ENSG00000116353 |
| Upregulated in class | ENSG00000116353\_pos |
| GeneSet | KEGG\_SELENOAMINO\_ACID\_METABOLISM |
| Enrichment Score (ES) | 0.5709385 |
| Normalized Enrichment Score (NES) | 1.8411139 |
| Nominal p-value | 0.00589391 |
| FDR q-value | 0.0061748484 |
| FWER p-Value | 0.104 |
Table: GSEA Results Summary

  

Fig 1: Enrichment plot: KEGG\_SELENOAMINO\_ACID\_METABOLISM      
 Profile of the Running ES Score & Positions of GeneSet Members on the Rank Ordered List

  

| SYMBOL | TITLE | RANK IN GENE LIST | RANK METRIC SCORE | RUNNING ES | CORE ENRICHMENT || 1 | LCMT1 | leucine carboxyl methyltransferase 1 [Source:HGNC Symbol;Acc:HGNC:17557] | 960 | 0.218 | 0.0723 | Yes |
| 2 | BUD23 | BUD23 rRNA methyltransferase and ribosome maturation factor [Source:HGNC Symbol;Acc:HGNC:16405] | 1011 | 0.214 | 0.1662 | Yes |
| 3 | SEPHS1 | selenophosphate synthetase 1 [Source:HGNC Symbol;Acc:HGNC:19685] | 1210 | 0.203 | 0.2512 | Yes |
| 4 | CTH | cystathionine gamma-lyase [Source:HGNC Symbol;Acc:HGNC:2501] | 1547 | 0.184 | 0.3245 | Yes |
| 5 | AHCY | adenosylhomocysteinase [Source:HGNC Symbol;Acc:HGNC:343] | 1880 | 0.171 | 0.3919 | Yes |
| 6 | GGT7 | gamma-glutamyltransferase 7 [Source:HGNC Symbol;Acc:HGNC:4259] | 2534 | 0.149 | 0.4414 | Yes |
| 7 | SEPHS2 | selenophosphate synthetase 2 [Source:HGNC Symbol;Acc:HGNC:19686] | 2621 | 0.147 | 0.5044 | Yes |
| 8 | AHCYL1 | adenosylhomocysteinase like 1 [Source:HGNC Symbol;Acc:HGNC:344] | 2828 | 0.141 | 0.5618 | Yes |
| 9 | PAPSS1 | 3'-phosphoadenosine 5'-phosphosulfate synthase 1 [Source:HGNC Symbol;Acc:HGNC:8603] | 6632 | 0.087 | 0.5039 | Yes |
| 10 | HEMK1 | HemK methyltransferase family member 1 [Source:HGNC Symbol;Acc:HGNC:24923] | 6767 | 0.086 | 0.5386 | Yes |
| 11 | METTL6 | methyltransferase like 6 [Source:HGNC Symbol;Acc:HGNC:28343] | 6966 | 0.084 | 0.5709 | Yes |
| 12 | PAPSS2 | 3'-phosphoadenosine 5'-phosphosulfate synthase 2 [Source:HGNC Symbol;Acc:HGNC:8604] | 9265 | 0.065 | 0.5416 | No |
| 13 | METTL2B | methyltransferase like 2B [Source:HGNC Symbol;Acc:HGNC:18272] | 9365 | 0.065 | 0.5679 | No |
| 14 | GGT1 | gamma-glutamyltransferase 1 [Source:HGNC Symbol;Acc:HGNC:4250] | 11879 | 0.048 | 0.5252 | No |
| 15 | MAT2B | methionine adenosyltransferase 2B [Source:HGNC Symbol;Acc:HGNC:6905] | 12827 | 0.042 | 0.5197 | No |
| 16 | SCLY | selenocysteine lyase [Source:HGNC Symbol;Acc:HGNC:18161] | 15182 | 0.028 | 0.4724 | No |
| 17 | GGT6 | gamma-glutamyltransferase 6 [Source:HGNC Symbol;Acc:HGNC:26891] | 17316 | 0.016 | 0.4251 | No |
| 18 | MAT1A | methionine adenosyltransferase 1A [Source:HGNC Symbol;Acc:HGNC:6903] | 17862 | 0.013 | 0.4170 | No |
| 19 | MAT2A | methionine adenosyltransferase 2A [Source:HGNC Symbol;Acc:HGNC:6904] | 19948 | 0.001 | 0.3644 | No |
| 20 | MARS2 | "methionyl-tRNA synthetase 2, mitochondrial [Source:HGNC Symbol;Acc:HGNC:25133]" | 22652 | -0.015 | 0.3024 | No |
| 21 | CBS | cystathionine beta-synthase [Source:HGNC Symbol;Acc:HGNC:1550] | 22772 | -0.016 | 0.3064 | No |
| 22 | MARS1 | methionyl-tRNA synthetase 1 [Source:HGNC Symbol;Acc:HGNC:6898] | 23032 | -0.018 | 0.3077 | No |
| 23 | AHCYL2 | adenosylhomocysteinase like 2 [Source:HGNC Symbol;Acc:HGNC:22204] | 26094 | -0.039 | 0.2470 | No |
| 24 | TRMT11 | tRNA methyltransferase 11 homolog [Source:HGNC Symbol;Acc:HGNC:21080] | 27376 | -0.048 | 0.2359 | No |
| 25 | GGT5 | gamma-glutamyltransferase 5 [Source:HGNC Symbol;Acc:HGNC:4260] | 29089 | -0.062 | 0.2201 | No |
| 26 | LCMT2 | leucine carboxyl methyltransferase 2 [Source:HGNC Symbol;Acc:HGNC:17558] | 31987 | -0.091 | 0.1867 | No |
Table: GSEA details [plain text format]

  

Fig 2: KEGG\_SELENOAMINO\_ACID\_METABOLISM      
 Blue-Pink O' Gram in the Space of the Analyzed GeneSet

  

Fig 3: KEGG\_SELENOAMINO\_ACID\_METABOLISM: Random ES distribution      
 Gene set null distribution of ES for **KEGG\_SELENOAMINO\_ACID\_METABOLISM**
