# Supplementary material for: Simultaneous Presentation of Multiple Myeloma and Lung Cancer: Case Report and Gene Bioinformatics Analysis
Source: Front Oncol. 2022 Jun 13;12:859735. doi: 10.3389/fonc.2022.859735 (PMC9235397; doi:10.3389/fonc.2022.859735)
Supplement: Supplementary file 1 [file DataSheet_1.zip › The bioinformatic analysis of MM and lung cancer supplementary materials/Enrichment analysis/MECR/GSEA_4.1.0/LUSC TCGA/KEGG.Gsea.1639042374131/KEGG_SPLICEOSOME.html]

Details for gene set KEGG\_SPLICEOSOME[GSEA]

|  || Dataset | ExpData\_collapsed\_to\_symbols.ENSG00000116353\_profile\_in\_ExpData.cls #ENSG00000116353 |
| Phenotype | ENSG00000116353\_profile\_in\_ExpData.cls#ENSG00000116353 |
| Upregulated in class | ENSG00000116353\_pos |
| GeneSet | KEGG\_SPLICEOSOME |
| Enrichment Score (ES) | 0.46353954 |
| Normalized Enrichment Score (NES) | 2.0224547 |
| Nominal p-value | 0.0 |
| FDR q-value | 0.0010199142 |
| FWER p-Value | 0.012 |
Table: GSEA Results Summary

  

Fig 1: Enrichment plot: KEGG\_SPLICEOSOME      
 Profile of the Running ES Score & Positions of GeneSet Members on the Rank Ordered List

  

| SYMBOL | TITLE | RANK IN GENE LIST | RANK METRIC SCORE | RUNNING ES | CORE ENRICHMENT || 1 | PPIH | peptidylprolyl isomerase H [Source:HGNC Symbol;Acc:HGNC:14651] | 79 | 0.413 | 0.0244 | Yes |
| 2 | SF3A3 | splicing factor 3a subunit 3 [Source:HGNC Symbol;Acc:HGNC:10767] | 99 | 0.395 | 0.0491 | Yes |
| 3 | SRSF4 | serine and arginine rich splicing factor 4 [Source:HGNC Symbol;Acc:HGNC:10786] | 109 | 0.389 | 0.0736 | Yes |
| 4 | SNRNP40 | small nuclear ribonucleoprotein U5 subunit 40 [Source:HGNC Symbol;Acc:HGNC:30857] | 113 | 0.385 | 0.0981 | Yes |
| 5 | MAGOH | "mago homolog, exon junction complex subunit [Source:HGNC Symbol;Acc:HGNC:6815]" | 114 | 0.385 | 0.1226 | Yes |
| 6 | SYF2 | SYF2 pre-mRNA splicing factor [Source:HGNC Symbol;Acc:HGNC:19824] | 219 | 0.332 | 0.1412 | Yes |
| 7 | PRPF38A | pre-mRNA processing factor 38A [Source:HGNC Symbol;Acc:HGNC:25930] | 228 | 0.329 | 0.1620 | Yes |
| 8 | PRPF31 | pre-mRNA processing factor 31 [Source:HGNC Symbol;Acc:HGNC:15446] | 259 | 0.320 | 0.1816 | Yes |
| 9 | PPIE | peptidylprolyl isomerase E [Source:HGNC Symbol;Acc:HGNC:9258] | 270 | 0.317 | 0.2016 | Yes |
| 10 | PUF60 | poly(U) binding splicing factor 60 [Source:HGNC Symbol;Acc:HGNC:17042] | 281 | 0.312 | 0.2212 | Yes |
| 11 | SNRPC | small nuclear ribonucleoprotein polypeptide C [Source:HGNC Symbol;Acc:HGNC:11157] | 291 | 0.309 | 0.2407 | Yes |
| 12 | U2AF2 | U2 small nuclear RNA auxiliary factor 2 [Source:HGNC Symbol;Acc:HGNC:23156] | 499 | 0.270 | 0.2527 | Yes |
| 13 | LSM2 | "LSM2 homolog, U6 small nuclear RNA and mRNA degradation associated [Source:HGNC Symbol;Acc:HGNC:13940]" | 737 | 0.240 | 0.2619 | Yes |
| 14 | BCAS2 | BCAS2 pre-mRNA processing factor [Source:HGNC Symbol;Acc:HGNC:975] | 816 | 0.230 | 0.2746 | Yes |
| 15 | HSPA1A | heat shock protein family A (Hsp70) member 1A [Source:HGNC Symbol;Acc:HGNC:5232] | 918 | 0.221 | 0.2861 | Yes |
| 16 | SRSF10 | serine and arginine rich splicing factor 10 [Source:HGNC Symbol;Acc:HGNC:16713] | 950 | 0.218 | 0.2992 | Yes |
| 17 | SNRPD2 | small nuclear ribonucleoprotein D2 polypeptide [Source:HGNC Symbol;Acc:HGNC:11159] | 961 | 0.218 | 0.3128 | Yes |
| 18 | HSPA2 | heat shock protein family A (Hsp70) member 2 [Source:HGNC Symbol;Acc:HGNC:5235] | 1167 | 0.205 | 0.3207 | Yes |
| 19 | SF3B5 | splicing factor 3b subunit 5 [Source:HGNC Symbol;Acc:HGNC:21083] | 1426 | 0.190 | 0.3262 | Yes |
| 20 | CCDC12 | coiled-coil domain containing 12 [Source:HGNC Symbol;Acc:HGNC:28332] | 1524 | 0.185 | 0.3356 | Yes |
| 21 | SMNDC1 | survival motor neuron domain containing 1 [Source:HGNC Symbol;Acc:HGNC:16900] | 1531 | 0.185 | 0.3472 | Yes |
| 22 | BUD31 | BUD31 homolog [Source:HGNC Symbol;Acc:HGNC:29629] | 1641 | 0.181 | 0.3560 | Yes |
| 23 | CTNNBL1 | catenin beta like 1 [Source:HGNC Symbol;Acc:HGNC:15879] | 1677 | 0.179 | 0.3665 | Yes |
| 24 | PRPF6 | pre-mRNA processing factor 6 [Source:HGNC Symbol;Acc:HGNC:15860] | 1739 | 0.176 | 0.3761 | Yes |
| 25 | PQBP1 | polyglutamine binding protein 1 [Source:HGNC Symbol;Acc:HGNC:9330] | 1910 | 0.169 | 0.3826 | Yes |
| 26 | SNU13 | small nuclear ribonucleoprotein 13 [Source:HGNC Symbol;Acc:HGNC:7819] | 2277 | 0.156 | 0.3832 | Yes |
| 27 | SNRPE | small nuclear ribonucleoprotein polypeptide E [Source:HGNC Symbol;Acc:HGNC:11161] | 2346 | 0.154 | 0.3913 | Yes |
| 28 | SNRPB | small nuclear ribonucleoprotein polypeptides B and B1 [Source:HGNC Symbol;Acc:HGNC:11153] | 2374 | 0.153 | 0.4004 | Yes |
| 29 | CWC15 | CWC15 spliceosome associated protein homolog [Source:HGNC Symbol;Acc:HGNC:26939] | 2439 | 0.151 | 0.4084 | Yes |
| 30 | SNRPA | small nuclear ribonucleoprotein polypeptide A [Source:HGNC Symbol;Acc:HGNC:11151] | 2544 | 0.148 | 0.4152 | Yes |
| 31 | LSM3 | "LSM3 homolog, U6 small nuclear RNA and mRNA degradation associated [Source:HGNC Symbol;Acc:HGNC:17874]" | 2593 | 0.147 | 0.4234 | Yes |
| 32 | SNRPD3 | small nuclear ribonucleoprotein D3 polypeptide [Source:HGNC Symbol;Acc:HGNC:11160] | 2631 | 0.146 | 0.4317 | Yes |
| 33 | DHX15 | DEAH-box helicase 15 [Source:HGNC Symbol;Acc:HGNC:2738] | 3240 | 0.132 | 0.4246 | Yes |
| 34 | PHF5A | PHD finger protein 5A [Source:HGNC Symbol;Acc:HGNC:18000] | 3543 | 0.126 | 0.4249 | Yes |
| 35 | ISY1 | ISY1 splicing factor homolog [Source:HGNC Symbol;Acc:HGNC:29201] | 3574 | 0.125 | 0.4321 | Yes |
| 36 | TXNL4A | thioredoxin like 4A [Source:HGNC Symbol;Acc:HGNC:30551] | 3924 | 0.119 | 0.4308 | Yes |
| 37 | SF3B6 | splicing factor 3b subunit 6 [Source:HGNC Symbol;Acc:HGNC:30096] | 4113 | 0.116 | 0.4334 | Yes |
| 38 | PPIL1 | peptidylprolyl isomerase like 1 [Source:HGNC Symbol;Acc:HGNC:9260] | 4471 | 0.110 | 0.4313 | Yes |
| 39 | PRPF18 | pre-mRNA processing factor 18 [Source:HGNC Symbol;Acc:HGNC:17351] | 4474 | 0.110 | 0.4383 | Yes |
| 40 | SRSF3 | serine and arginine rich splicing factor 3 [Source:HGNC Symbol;Acc:HGNC:10785] | 4673 | 0.107 | 0.4401 | Yes |
| 41 | SNRPF | small nuclear ribonucleoprotein polypeptide F [Source:HGNC Symbol;Acc:HGNC:11162] | 4725 | 0.107 | 0.4456 | Yes |
| 42 | LSM4 | "LSM4 homolog, U6 small nuclear RNA and mRNA degradation associated [Source:HGNC Symbol;Acc:HGNC:17259]" | 4744 | 0.106 | 0.4519 | Yes |
| 43 | PRPF19 | pre-mRNA processing factor 19 [Source:HGNC Symbol;Acc:HGNC:17896] | 4894 | 0.104 | 0.4547 | Yes |
| 44 | SNRNP70 | small nuclear ribonucleoprotein U1 subunit 70 [Source:HGNC Symbol;Acc:HGNC:11150] | 4908 | 0.104 | 0.4611 | Yes |
| 45 | SNRPA1 | small nuclear ribonucleoprotein polypeptide A' [Source:HGNC Symbol;Acc:HGNC:11152] | 5066 | 0.102 | 0.4635 | Yes |
| 46 | RBM22 | RNA binding motif protein 22 [Source:HGNC Symbol;Acc:HGNC:25503] | 5404 | 0.098 | 0.4612 | No |
| 47 | SF3B3 | splicing factor 3b subunit 3 [Source:HGNC Symbol;Acc:HGNC:10770] | 5933 | 0.095 | 0.4538 | No |
| 48 | SRSF7 | serine and arginine rich splicing factor 7 [Source:HGNC Symbol;Acc:HGNC:10789] | 6055 | 0.093 | 0.4566 | No |
| 49 | SF3B2 | splicing factor 3b subunit 2 [Source:HGNC Symbol;Acc:HGNC:10769] | 6313 | 0.090 | 0.4558 | No |
| 50 | LSM6 | "LSM6 homolog, U6 small nuclear RNA and mRNA degradation associated [Source:HGNC Symbol;Acc:HGNC:17017]" | 6473 | 0.089 | 0.4574 | No |
| 51 | RBM17 | RNA binding motif protein 17 [Source:HGNC Symbol;Acc:HGNC:16944] | 6971 | 0.084 | 0.4501 | No |
| 52 | SNRPB2 | small nuclear ribonucleoprotein polypeptide B2 [Source:HGNC Symbol;Acc:HGNC:11155] | 7628 | 0.078 | 0.4384 | No |
| 53 | SF3A1 | splicing factor 3a subunit 1 [Source:HGNC Symbol;Acc:HGNC:10765] | 7910 | 0.076 | 0.4360 | No |
| 54 | SNRPG | small nuclear ribonucleoprotein polypeptide G [Source:HGNC Symbol;Acc:HGNC:11163] | 8190 | 0.073 | 0.4336 | No |
| 55 | HNRNPC | heterogeneous nuclear ribonucleoprotein C [Source:HGNC Symbol;Acc:HGNC:5035] | 8472 | 0.071 | 0.4309 | No |
| 56 | DHX16 | DEAH-box helicase 16 [Source:HGNC Symbol;Acc:HGNC:2739] | 9141 | 0.066 | 0.4181 | No |
| 57 | PCBP1 | poly(rC) binding protein 1 [Source:HGNC Symbol;Acc:HGNC:8647] | 9528 | 0.064 | 0.4123 | No |
| 58 | SRSF6 | serine and arginine rich splicing factor 6 [Source:HGNC Symbol;Acc:HGNC:10788] | 9772 | 0.062 | 0.4101 | No |
| 59 | SRSF8 | serine and arginine rich splicing factor 8 [Source:HGNC Symbol;Acc:HGNC:16988] | 9855 | 0.061 | 0.4119 | No |
| 60 | ZMAT2 | zinc finger matrin-type 2 [Source:HGNC Symbol;Acc:HGNC:26433] | 9956 | 0.060 | 0.4132 | No |
| 61 | RBM8A | RNA binding motif protein 8A [Source:HGNC Symbol;Acc:HGNC:9905] | 10862 | 0.054 | 0.3936 | No |
| 62 | MAGOHB | "mago homolog B, exon junction complex subunit [Source:HGNC Symbol;Acc:HGNC:25504]" | 11209 | 0.052 | 0.3881 | No |
| 63 | SRSF5 | serine and arginine rich splicing factor 5 [Source:HGNC Symbol;Acc:HGNC:10787] | 11652 | 0.049 | 0.3799 | No |
| 64 | LSM7 | "LSM7 homolog, U6 small nuclear RNA and mRNA degradation associated [Source:HGNC Symbol;Acc:HGNC:20470]" | 13071 | 0.040 | 0.3463 | No |
| 65 | RBMX | RNA binding motif protein X-linked [Source:HGNC Symbol;Acc:HGNC:9910] | 13110 | 0.040 | 0.3479 | No |
| 66 | SART1 | "spliceosome associated factor 1, recruiter of U4/U6.U5 tri-snRNP [Source:HGNC Symbol;Acc:HGNC:10538]" | 13305 | 0.039 | 0.3455 | No |
| 67 | PRPF38B | pre-mRNA processing factor 38B [Source:HGNC Symbol;Acc:HGNC:25512] | 13620 | 0.037 | 0.3398 | No |
| 68 | DHX38 | DEAH-box helicase 38 [Source:HGNC Symbol;Acc:HGNC:17211] | 13858 | 0.036 | 0.3361 | No |
| 69 | PRPF40B | pre-mRNA processing factor 40 homolog B [Source:HGNC Symbol;Acc:HGNC:25031] | 13923 | 0.036 | 0.3367 | No |
| 70 | SNRPD1 | small nuclear ribonucleoprotein D1 polypeptide [Source:HGNC Symbol;Acc:HGNC:11158] | 14095 | 0.034 | 0.3345 | No |
| 71 | PLRG1 | pleiotropic regulator 1 [Source:HGNC Symbol;Acc:HGNC:9089] | 14483 | 0.032 | 0.3267 | No |
| 72 | SNW1 | SNW domain containing 1 [Source:HGNC Symbol;Acc:HGNC:16696] | 14609 | 0.031 | 0.3255 | No |
| 73 | LSM5 | "LSM5 homolog, U6 small nuclear RNA and mRNA degradation associated [Source:HGNC Symbol;Acc:HGNC:17162]" | 14961 | 0.030 | 0.3185 | No |
| 74 | XAB2 | XPA binding protein 2 [Source:HGNC Symbol;Acc:HGNC:14089] | 14994 | 0.029 | 0.3195 | No |
| 75 | SNRNP27 | small nuclear ribonucleoprotein U4/U6.U5 subunit 27 [Source:HGNC Symbol;Acc:HGNC:30240] | 15140 | 0.028 | 0.3176 | No |
| 76 | HNRNPA1 | heterogeneous nuclear ribonucleoprotein A1 [Source:HGNC Symbol;Acc:HGNC:5031] | 15587 | 0.026 | 0.3079 | No |
| 77 | DDX39B | DExD-box helicase 39B [Source:HGNC Symbol;Acc:HGNC:13917] | 16452 | 0.021 | 0.2872 | No |
| 78 | U2SURP | U2 snRNP associated SURP domain containing [Source:HGNC Symbol;Acc:HGNC:30855] | 16634 | 0.020 | 0.2838 | No |
| 79 | SRSF2 | serine and arginine rich splicing factor 2 [Source:HGNC Symbol;Acc:HGNC:10783] | 17187 | 0.017 | 0.2708 | No |
| 80 | RBM25 | RNA binding motif protein 25 [Source:HGNC Symbol;Acc:HGNC:23244] | 17538 | 0.015 | 0.2628 | No |
| 81 | HSPA8 | heat shock protein family A (Hsp70) member 8 [Source:HGNC Symbol;Acc:HGNC:5241] | 18296 | 0.011 | 0.2442 | No |
| 82 | DDX23 | DEAD-box helicase 23 [Source:HGNC Symbol;Acc:HGNC:17347] | 18806 | 0.008 | 0.2317 | No |
| 83 | ALYREF | Aly/REF export factor [Source:HGNC Symbol;Acc:HGNC:19071] | 19334 | 0.005 | 0.2185 | No |
| 84 | EIF4A3 | eukaryotic translation initiation factor 4A3 [Source:HGNC Symbol;Acc:HGNC:18683] | 19373 | 0.004 | 0.2178 | No |
| 85 | HNRNPA1P60 | heterogeneous nuclear ribonucleoprotein A1 pseudogene 60 [Source:HGNC Symbol;Acc:HGNC:48790] | 19956 | 0.001 | 0.2030 | No |
| 86 | THOC1 | THO complex 1 [Source:HGNC Symbol;Acc:HGNC:19070] | 20989 | -0.005 | 0.1770 | No |
| 87 | ACIN1 | apoptotic chromatin condensation inducer 1 [Source:HGNC Symbol;Acc:HGNC:17066] | 22294 | -0.013 | 0.1446 | No |
| 88 | SF3A2 | splicing factor 3a subunit 2 [Source:HGNC Symbol;Acc:HGNC:10766] | 23040 | -0.018 | 0.1267 | No |
| 89 | USP39 | ubiquitin specific peptidase 39 [Source:HGNC Symbol;Acc:HGNC:20071] | 23411 | -0.020 | 0.1186 | No |
| 90 | TCERG1 | transcription elongation regulator 1 [Source:HGNC Symbol;Acc:HGNC:15630] | 23611 | -0.021 | 0.1148 | No |
| 91 | SRSF9 | serine and arginine rich splicing factor 9 [Source:HGNC Symbol;Acc:HGNC:10791] | 24319 | -0.026 | 0.0985 | No |
| 92 | SF3B4 | splicing factor 3b subunit 4 [Source:HGNC Symbol;Acc:HGNC:10771] | 24747 | -0.029 | 0.0894 | No |
| 93 | WBP11 | WW domain binding protein 11 [Source:HGNC Symbol;Acc:HGNC:16461] | 25098 | -0.032 | 0.0825 | No |
| 94 | U2AF1 | U2 small nuclear RNA auxiliary factor 1 [Source:HGNC Symbol;Acc:HGNC:12453] | 26093 | -0.039 | 0.0597 | No |
| 95 | LSM8 | "LSM8 homolog, U6 small nuclear RNA associated [Source:HGNC Symbol;Acc:HGNC:20471]" | 26366 | -0.041 | 0.0553 | No |
| 96 | TRA2A | transformer 2 alpha homolog [Source:HGNC Symbol;Acc:HGNC:16645] | 27569 | -0.050 | 0.0278 | No |
| 97 | HSPA6 | heat shock protein family A (Hsp70) member 6 [Source:HGNC Symbol;Acc:HGNC:5239] | 27959 | -0.053 | 0.0213 | No |
| 98 | HSPA1L | heat shock protein family A (Hsp70) member 1 like [Source:HGNC Symbol;Acc:HGNC:5234] | 29116 | -0.063 | -0.0042 | No |
| 99 | SF3B1 | splicing factor 3b subunit 1 [Source:HGNC Symbol;Acc:HGNC:10768] | 29268 | -0.064 | -0.0040 | No |
| 100 | SRSF1 | serine and arginine rich splicing factor 1 [Source:HGNC Symbol;Acc:HGNC:10780] | 29741 | -0.068 | -0.0117 | No |
| 101 | SLU7 | "SLU7 homolog, splicing factor [Source:HGNC Symbol;Acc:HGNC:16939]" | 29839 | -0.069 | -0.0097 | No |
| 102 | CDC5L | cell division cycle 5 like [Source:HGNC Symbol;Acc:HGNC:1743] | 30313 | -0.074 | -0.0171 | No |
| 103 | HNRNPA1L2 | heterogeneous nuclear ribonucleoprotein A1 like 2 [Source:HGNC Symbol;Acc:HGNC:27067] | 30461 | -0.075 | -0.0161 | No |
| 104 | NCBP2 | nuclear cap binding protein subunit 2 [Source:HGNC Symbol;Acc:HGNC:7659] | 31809 | -0.089 | -0.0448 | No |
| 105 | EFTUD2 | elongation factor Tu GTP binding domain containing 2 [Source:HGNC Symbol;Acc:HGNC:30858] | 31814 | -0.089 | -0.0392 | No |
| 106 | CRNKL1 | crooked neck pre-mRNA splicing factor 1 [Source:HGNC Symbol;Acc:HGNC:15762] | 32039 | -0.091 | -0.0391 | No |
| 107 | HNRNPM | heterogeneous nuclear ribonucleoprotein M [Source:HGNC Symbol;Acc:HGNC:5046] | 32697 | -0.099 | -0.0495 | No |
| 108 | PRPF40A | pre-mRNA processing factor 40 homolog A [Source:HGNC Symbol;Acc:HGNC:16463] | 33887 | -0.116 | -0.0725 | No |
| 109 | SNRNP200 | small nuclear ribonucleoprotein U5 subunit 200 [Source:HGNC Symbol;Acc:HGNC:30859] | 33978 | -0.117 | -0.0673 | No |
| 110 | HNRNPU | heterogeneous nuclear ribonucleoprotein U [Source:HGNC Symbol;Acc:HGNC:5048] | 34372 | -0.124 | -0.0694 | No |
| 111 | THOC2 | THO complex 2 [Source:HGNC Symbol;Acc:HGNC:19073] | 34375 | -0.124 | -0.0616 | No |
| 112 | DDX5 | DEAD-box helicase 5 [Source:HGNC Symbol;Acc:HGNC:2746] | 34414 | -0.124 | -0.0546 | No |
| 113 | THOC3 | THO complex 3 [Source:HGNC Symbol;Acc:HGNC:19072] | 34726 | -0.130 | -0.0543 | No |
| 114 | HNRNPA3 | heterogeneous nuclear ribonucleoprotein A3 [Source:HGNC Symbol;Acc:HGNC:24941] | 34797 | -0.131 | -0.0477 | No |
| 115 | AQR | aquarius intron-binding spliceosomal factor [Source:HGNC Symbol;Acc:HGNC:29513] | 34859 | -0.132 | -0.0408 | No |
| 116 | DDX46 | DEAD-box helicase 46 [Source:HGNC Symbol;Acc:HGNC:18681] | 35018 | -0.136 | -0.0362 | No |
| 117 | HNRNPK | heterogeneous nuclear ribonucleoprotein K [Source:HGNC Symbol;Acc:HGNC:5044] | 35394 | -0.144 | -0.0365 | No |
| 118 | CHERP | calcium homeostasis endoplasmic reticulum protein [Source:HGNC Symbol;Acc:HGNC:16930] | 35495 | -0.147 | -0.0297 | No |
| 119 | TRA2B | transformer 2 beta homolog [Source:HGNC Symbol;Acc:HGNC:10781] | 36123 | -0.164 | -0.0353 | No |
| 120 | PRPF8 | pre-mRNA processing factor 8 [Source:HGNC Symbol;Acc:HGNC:17340] | 36170 | -0.166 | -0.0259 | No |
| 121 | PRPF3 | pre-mRNA processing factor 3 [Source:HGNC Symbol;Acc:HGNC:17348] | 36448 | -0.175 | -0.0218 | No |
| 122 | HSPA1B | heat shock protein family A (Hsp70) member 1B [Source:HGNC Symbol;Acc:HGNC:5233] | 36628 | -0.181 | -0.0148 | No |
| 123 | DDX42 | DEAD-box helicase 42 [Source:HGNC Symbol;Acc:HGNC:18676] | 36789 | -0.188 | -0.0069 | No |
| 124 | DHX8 | DEAH-box helicase 8 [Source:HGNC Symbol;Acc:HGNC:2749] | 37545 | -0.227 | -0.0117 | No |
| 125 | CDC40 | cell division cycle 40 [Source:HGNC Symbol;Acc:HGNC:17350] | 37557 | -0.228 | 0.0025 | No |
| 126 | NCBP1 | nuclear cap binding protein subunit 1 [Source:HGNC Symbol;Acc:HGNC:7658] | 38378 | -0.324 | 0.0023 | No |
| 127 | PRPF4 | pre-mRNA processing factor 4 [Source:HGNC Symbol;Acc:HGNC:17349] | 38436 | -0.342 | 0.0226 | No |
Table: GSEA details [plain text format]

  

Fig 2: KEGG\_SPLICEOSOME      
 Blue-Pink O' Gram in the Space of the Analyzed GeneSet

  

Fig 3: KEGG\_SPLICEOSOME: Random ES distribution      
 Gene set null distribution of ES for **KEGG\_SPLICEOSOME**
