# Supplementary material for: Simultaneous Presentation of Multiple Myeloma and Lung Cancer: Case Report and Gene Bioinformatics Analysis
Source: Front Oncol. 2022 Jun 13;12:859735. doi: 10.3389/fonc.2022.859735 (PMC9235397; doi:10.3389/fonc.2022.859735)
Supplement: Supplementary file 1 [file DataSheet_1.zip › The bioinformatic analysis of MM and lung cancer supplementary materials/Enrichment analysis/MECR/GSEA_4.1.0/LUSC TCGA/KEGG.Gsea.1639042374131/KEGG_SYSTEMIC_LUPUS_ERYTHEMATOSUS.html]

Details for gene set KEGG\_SYSTEMIC\_LUPUS\_ERYTHEMATOSUS[GSEA]

|  || Dataset | ExpData\_collapsed\_to\_symbols.ENSG00000116353\_profile\_in\_ExpData.cls #ENSG00000116353 |
| Phenotype | ENSG00000116353\_profile\_in\_ExpData.cls#ENSG00000116353 |
| Upregulated in class | ENSG00000116353\_pos |
| GeneSet | KEGG\_SYSTEMIC\_LUPUS\_ERYTHEMATOSUS |
| Enrichment Score (ES) | 0.40933383 |
| Normalized Enrichment Score (NES) | 1.8231521 |
| Nominal p-value | 0.0 |
| FDR q-value | 0.0064967284 |
| FWER p-Value | 0.116 |
Table: GSEA Results Summary

  

Fig 1: Enrichment plot: KEGG\_SYSTEMIC\_LUPUS\_ERYTHEMATOSUS      
 Profile of the Running ES Score & Positions of GeneSet Members on the Rank Ordered List

  

| SYMBOL | TITLE | RANK IN GENE LIST | RANK METRIC SCORE | RUNNING ES | CORE ENRICHMENT || 1 | H3C11 | H3 clustered histone 11 [Source:HGNC Symbol;Acc:HGNC:4771] | 554 | 0.262 | 0.0100 | Yes |
| 2 | MACROH2A1 | macroH2A.1 histone [Source:HGNC Symbol;Acc:HGNC:4740] | 1365 | 0.193 | 0.0072 | Yes |
| 3 | H3C1 | H3 clustered histone 1 [Source:HGNC Symbol;Acc:HGNC:4766] | 1691 | 0.178 | 0.0154 | Yes |
| 4 | H2BC12 | H2B clustered histone 12 [Source:HGNC Symbol;Acc:HGNC:13954] | 1734 | 0.176 | 0.0305 | Yes |
| 5 | H4C9 | H4 clustered histone 9 [Source:HGNC Symbol;Acc:HGNC:4793] | 1748 | 0.175 | 0.0464 | Yes |
| 6 | H2AC7 | H2A clustered histone 7 [Source:HGNC Symbol;Acc:HGNC:4729] | 1814 | 0.173 | 0.0607 | Yes |
| 7 | H2AC16 | H2A clustered histone 16 [Source:HGNC Symbol;Acc:HGNC:4730] | 1842 | 0.172 | 0.0758 | Yes |
| 8 | H4C13 | H4 clustered histone 13 [Source:HGNC Symbol;Acc:HGNC:4791] | 2281 | 0.156 | 0.0791 | Yes |
| 9 | H3-3A | H3.3 histone A [Source:HGNC Symbol;Acc:HGNC:4764] | 2284 | 0.156 | 0.0934 | Yes |
| 10 | SNRPB | small nuclear ribonucleoprotein polypeptides B and B1 [Source:HGNC Symbol;Acc:HGNC:11153] | 2374 | 0.153 | 0.1052 | Yes |
| 11 | H4C3 | H4 clustered histone 3 [Source:HGNC Symbol;Acc:HGNC:4787] | 2393 | 0.152 | 0.1189 | Yes |
| 12 | H2AZ1 | H2A.Z variant histone 1 [Source:HGNC Symbol;Acc:HGNC:4741] | 2421 | 0.152 | 0.1322 | Yes |
| 13 | H2AC6 | H2A clustered histone 6 [Source:HGNC Symbol;Acc:HGNC:4733] | 2546 | 0.148 | 0.1427 | Yes |
| 14 | H3-3B | H3.3 histone B [Source:HGNC Symbol;Acc:HGNC:4765] | 2589 | 0.147 | 0.1552 | Yes |
| 15 | SNRPD3 | small nuclear ribonucleoprotein D3 polypeptide [Source:HGNC Symbol;Acc:HGNC:11160] | 2631 | 0.146 | 0.1677 | Yes |
| 16 | H2BC11 | H2B clustered histone 11 [Source:HGNC Symbol;Acc:HGNC:4761] | 2704 | 0.144 | 0.1791 | Yes |
| 17 | FCGR3B | Fc fragment of IgG receptor IIIb [Source:HGNC Symbol;Acc:HGNC:3620] | 2758 | 0.142 | 0.1909 | Yes |
| 18 | H2BC17 | H2B clustered histone 17 [Source:HGNC Symbol;Acc:HGNC:4758] | 2823 | 0.141 | 0.2023 | Yes |
| 19 | H2AJ | H2A.J histone [Source:HGNC Symbol;Acc:HGNC:14456] | 2924 | 0.139 | 0.2126 | Yes |
| 20 | H4C4 | H4 clustered histone 4 [Source:HGNC Symbol;Acc:HGNC:4782] | 3014 | 0.136 | 0.2229 | Yes |
| 21 | H2BC8 | H2B clustered histone 8 [Source:HGNC Symbol;Acc:HGNC:4746] | 3248 | 0.132 | 0.2290 | Yes |
| 22 | H2AC21 | H2A clustered histone 21 [Source:HGNC Symbol;Acc:HGNC:20508] | 3286 | 0.131 | 0.2402 | Yes |
| 23 | H3C4 | H3 clustered histone 4 [Source:HGNC Symbol;Acc:HGNC:4767] | 3458 | 0.127 | 0.2475 | Yes |
| 24 | H2AC8 | H2A clustered histone 8 [Source:HGNC Symbol;Acc:HGNC:4724] | 3509 | 0.126 | 0.2579 | Yes |
| 25 | H2AC14 | H2A clustered histone 14 [Source:HGNC Symbol;Acc:HGNC:4727] | 4111 | 0.116 | 0.2532 | Yes |
| 26 | H2AC17 | H2A clustered histone 17 [Source:HGNC Symbol;Acc:HGNC:4735] | 4140 | 0.115 | 0.2632 | Yes |
| 27 | ACTN1 | actinin alpha 1 [Source:HGNC Symbol;Acc:HGNC:163] | 4229 | 0.114 | 0.2714 | Yes |
| 28 | H2BC5 | H2B clustered histone 5 [Source:HGNC Symbol;Acc:HGNC:4747] | 4237 | 0.114 | 0.2818 | Yes |
| 29 | ACTN4 | actinin alpha 4 [Source:HGNC Symbol;Acc:HGNC:166] | 4255 | 0.114 | 0.2918 | Yes |
| 30 | H2BC4 | H2B clustered histone 4 [Source:HGNC Symbol;Acc:HGNC:4757] | 4447 | 0.110 | 0.2972 | Yes |
| 31 | H4C6 | H4 clustered histone 6 [Source:HGNC Symbol;Acc:HGNC:4783] | 4514 | 0.109 | 0.3056 | Yes |
| 32 | H4C12 | H4 clustered histone 12 [Source:HGNC Symbol;Acc:HGNC:4784] | 4543 | 0.109 | 0.3149 | Yes |
| 33 | H4C2 | H4 clustered histone 2 [Source:HGNC Symbol;Acc:HGNC:4789] | 4546 | 0.109 | 0.3249 | Yes |
| 34 | H3C12 | H3 clustered histone 12 [Source:HGNC Symbol;Acc:HGNC:4774] | 4687 | 0.107 | 0.3312 | Yes |
| 35 | H3C8 | H3 clustered histone 8 [Source:HGNC Symbol;Acc:HGNC:4772] | 4702 | 0.107 | 0.3408 | Yes |
| 36 | H2BC3 | H2B clustered histone 3 [Source:HGNC Symbol;Acc:HGNC:4751] | 4865 | 0.105 | 0.3463 | Yes |
| 37 | H4C15 | H4 clustered histone 15 [Source:HGNC Symbol;Acc:HGNC:29607] | 4935 | 0.104 | 0.3541 | Yes |
| 38 | H2AW | H2A.W histone [Source:HGNC Symbol;Acc:HGNC:20507] | 4992 | 0.103 | 0.3621 | Yes |
| 39 | H2AC4 | H2A clustered histone 4 [Source:HGNC Symbol;Acc:HGNC:4734] | 5013 | 0.102 | 0.3711 | Yes |
| 40 | H3C6 | H3 clustered histone 6 [Source:HGNC Symbol;Acc:HGNC:4769] | 5031 | 0.102 | 0.3801 | Yes |
| 41 | H3C10 | H3 clustered histone 10 [Source:HGNC Symbol;Acc:HGNC:4775] | 5090 | 0.101 | 0.3880 | Yes |
| 42 | C9 | complement C9 [Source:HGNC Symbol;Acc:HGNC:1358] | 5432 | 0.097 | 0.3883 | Yes |
| 43 | H2BC15 | H2B clustered histone 15 [Source:HGNC Symbol;Acc:HGNC:4749] | 5468 | 0.097 | 0.3963 | Yes |
| 44 | H2AC11 | H2A clustered histone 11 [Source:HGNC Symbol;Acc:HGNC:4737] | 5843 | 0.096 | 0.3957 | Yes |
| 45 | H2AC19 | H2A clustered histone 19 [Source:HGNC Symbol;Acc:HGNC:29668] | 6600 | 0.088 | 0.3844 | Yes |
| 46 | H3C13 | H3 clustered histone 13 [Source:HGNC Symbol;Acc:HGNC:25311] | 6654 | 0.087 | 0.3911 | Yes |
| 47 | H2BC10 | H2B clustered histone 10 [Source:HGNC Symbol;Acc:HGNC:4756] | 6977 | 0.084 | 0.3906 | Yes |
| 48 | H2BC9 | H2B clustered histone 9 [Source:HGNC Symbol;Acc:HGNC:4755] | 7170 | 0.082 | 0.3933 | Yes |
| 49 | TNF | tumor necrosis factor [Source:HGNC Symbol;Acc:HGNC:11892] | 7309 | 0.081 | 0.3973 | Yes |
| 50 | C8B | complement C8 beta chain [Source:HGNC Symbol;Acc:HGNC:1353] | 7414 | 0.080 | 0.4020 | Yes |
| 51 | H4C14 | H4 clustered histone 14 [Source:HGNC Symbol;Acc:HGNC:4794] | 7683 | 0.078 | 0.4023 | Yes |
| 52 | H2BC1 | H2B clustered histone 1 [Source:HGNC Symbol;Acc:HGNC:18730] | 7690 | 0.078 | 0.4093 | Yes |
| 53 | H2ACP1 | H2AC histone family pseudogene 1 [Source:HGNC Symbol;Acc:HGNC:42647] | 8464 | 0.071 | 0.3962 | No |
| 54 | H4C7 | H4 clustered histone 7 [Source:HGNC Symbol;Acc:HGNC:4792] | 8716 | 0.069 | 0.3961 | No |
| 55 | H4C11 | H4 clustered histone 11 [Source:HGNC Symbol;Acc:HGNC:4785] | 8723 | 0.069 | 0.4024 | No |
| 56 | H4C8 | H4 clustered histone 8 [Source:HGNC Symbol;Acc:HGNC:4788] | 9028 | 0.067 | 0.4008 | No |
| 57 | CD40 | CD40 molecule [Source:HGNC Symbol;Acc:HGNC:11919] | 10160 | 0.059 | 0.3774 | No |
| 58 | C8A | complement C8 alpha chain [Source:HGNC Symbol;Acc:HGNC:1352] | 10533 | 0.057 | 0.3731 | No |
| 59 | H2AC20 | H2A clustered histone 20 [Source:HGNC Symbol;Acc:HGNC:4738] | 11065 | 0.053 | 0.3645 | No |
| 60 | H4C1 | H4 clustered histone 1 [Source:HGNC Symbol;Acc:HGNC:4781] | 11460 | 0.051 | 0.3591 | No |
| 61 | H2AC13 | H2A clustered histone 13 [Source:HGNC Symbol;Acc:HGNC:4725] | 12031 | 0.047 | 0.3489 | No |
| 62 | H3Y2 | H3.Y histone 2 [Source:HGNC Symbol;Acc:HGNC:43734] | 12597 | 0.043 | 0.3385 | No |
| 63 | MACROH2A2 | macroH2A.2 histone [Source:HGNC Symbol;Acc:HGNC:14453] | 13827 | 0.036 | 0.3104 | No |
| 64 | SNRPD1 | small nuclear ribonucleoprotein D1 polypeptide [Source:HGNC Symbol;Acc:HGNC:11158] | 14095 | 0.034 | 0.3068 | No |
| 65 | H2BC7 | H2B clustered histone 7 [Source:HGNC Symbol;Acc:HGNC:4752] | 14332 | 0.033 | 0.3038 | No |
| 66 | C6 | complement C6 [Source:HGNC Symbol;Acc:HGNC:1339] | 14903 | 0.030 | 0.2920 | No |
| 67 | H3-5 | H3.5 histone [Source:HGNC Symbol;Acc:HGNC:33164] | 15014 | 0.029 | 0.2919 | No |
| 68 | H2BC14 | H2B clustered histone 14 [Source:HGNC Symbol;Acc:HGNC:4750] | 15225 | 0.028 | 0.2891 | No |
| 69 | H2AC12 | H2A clustered histone 12 [Source:HGNC Symbol;Acc:HGNC:13671] | 15768 | 0.025 | 0.2776 | No |
| 70 | ACTN3 | actinin alpha 3 [Source:HGNC Symbol;Acc:HGNC:165] | 16339 | 0.021 | 0.2650 | No |
| 71 | H2AC18 | H2A clustered histone 18 [Source:HGNC Symbol;Acc:HGNC:4736] | 16525 | 0.020 | 0.2622 | No |
| 72 | H2AB1 | H2A.B variant histone 1 [Source:HGNC Symbol;Acc:HGNC:22516] | 18013 | 0.012 | 0.2253 | No |
| 73 | H3C7 | H3 clustered histone 7 [Source:HGNC Symbol;Acc:HGNC:4773] | 18374 | 0.010 | 0.2171 | No |
| 74 | H3C15 | H3 clustered histone 15 [Source:HGNC Symbol;Acc:HGNC:20505] | 18841 | 0.008 | 0.2059 | No |
| 75 | H3Y1 | H3.Y histone 1 [Source:HGNC Symbol;Acc:HGNC:43735] | 19083 | 0.006 | 0.2003 | No |
| 76 | ACTN2 | actinin alpha 2 [Source:HGNC Symbol;Acc:HGNC:164] | 19220 | 0.005 | 0.1973 | No |
| 77 | H2AX | H2A.X variant histone [Source:HGNC Symbol;Acc:HGNC:4739] | 19464 | 0.004 | 0.1915 | No |
| 78 | H2AZ2 | H2A.Z variant histone 2 [Source:HGNC Symbol;Acc:HGNC:20664] | 19519 | 0.003 | 0.1904 | No |
| 79 | C4A | complement C4A (Rodgers blood group) [Source:HGNC Symbol;Acc:HGNC:1323] | 19827 | 0.002 | 0.1827 | No |
| 80 | H2AB2 | H2A.B variant histone 2 [Source:HGNC Symbol;Acc:HGNC:18298] | 20315 | -0.001 | 0.1704 | No |
| 81 | C4B | complement C4B (Chido blood group) [Source:HGNC Symbol;Acc:HGNC:1324] | 20684 | -0.003 | 0.1613 | No |
| 82 | H4C5 | H4 clustered histone 5 [Source:HGNC Symbol;Acc:HGNC:4790] | 21121 | -0.006 | 0.1508 | No |
| 83 | H2BC13 | H2B clustered histone 13 [Source:HGNC Symbol;Acc:HGNC:4748] | 21294 | -0.007 | 0.1470 | No |
| 84 | H2BU1 | H2B.U histone 1 [Source:HGNC Symbol;Acc:HGNC:20514] | 21337 | -0.007 | 0.1466 | No |
| 85 | ELANE | "elastase, neutrophil expressed [Source:HGNC Symbol;Acc:HGNC:3309]" | 21678 | -0.009 | 0.1388 | No |
| 86 | GRIN2A | glutamate ionotropic receptor NMDA type subunit 2A [Source:HGNC Symbol;Acc:HGNC:4585] | 21887 | -0.010 | 0.1344 | No |
| 87 | H2BC18 | H2B clustered histone 18 [Source:HGNC Symbol;Acc:HGNC:24700] | 21971 | -0.011 | 0.1333 | No |
| 88 | H2BC6 | H2B clustered histone 6 [Source:HGNC Symbol;Acc:HGNC:4753] | 22591 | -0.015 | 0.1189 | No |
| 89 | HLA-DMA | "major histocompatibility complex, class II, DM alpha [Source:HGNC Symbol;Acc:HGNC:4934]" | 22645 | -0.015 | 0.1190 | No |
| 90 | C8G | complement C8 gamma chain [Source:HGNC Symbol;Acc:HGNC:1354] | 25229 | -0.033 | 0.0561 | No |
| 91 | H2AC1 | H2A clustered histone 1 [Source:HGNC Symbol;Acc:HGNC:18729] | 25330 | -0.034 | 0.0566 | No |
| 92 | C3 | complement C3 [Source:HGNC Symbol;Acc:HGNC:1318] | 25379 | -0.034 | 0.0585 | No |
| 93 | SSB | small RNA binding exonuclease protection factor La [Source:HGNC Symbol;Acc:HGNC:11316] | 25987 | -0.038 | 0.0466 | No |
| 94 | H3C14 | H3 clustered histone 14 [Source:HGNC Symbol;Acc:HGNC:20503] | 26105 | -0.039 | 0.0472 | No |
| 95 | HLA-DRA | "major histocompatibility complex, class II, DR alpha [Source:HGNC Symbol;Acc:HGNC:4947]" | 26550 | -0.042 | 0.0397 | No |
| 96 | IL10 | interleukin 10 [Source:HGNC Symbol;Acc:HGNC:5962] | 26585 | -0.042 | 0.0428 | No |
| 97 | H2BW2 | H2B.W histone 2 [Source:HGNC Symbol;Acc:HGNC:27867] | 26680 | -0.043 | 0.0443 | No |
| 98 | HLA-DRB1 | "major histocompatibility complex, class II, DR beta 1 [Source:HGNC Symbol;Acc:HGNC:4948]" | 26773 | -0.044 | 0.0460 | No |
| 99 | FCGR2B | Fc fragment of IgG receptor IIb [Source:HGNC Symbol;Acc:HGNC:3618] | 26922 | -0.045 | 0.0464 | No |
| 100 | FCGR2A | Fc fragment of IgG receptor IIa [Source:HGNC Symbol;Acc:HGNC:3616] | 27275 | -0.048 | 0.0418 | No |
| 101 | C7 | complement C7 [Source:HGNC Symbol;Acc:HGNC:1346] | 27617 | -0.050 | 0.0378 | No |
| 102 | HLA-DQA2 | "major histocompatibility complex, class II, DQ alpha 2 [Source:HGNC Symbol;Acc:HGNC:4943]" | 27660 | -0.051 | 0.0414 | No |
| 103 | HLA-DQB1 | "major histocompatibility complex, class II, DQ beta 1 [Source:HGNC Symbol;Acc:HGNC:4944]" | 27794 | -0.052 | 0.0427 | No |
| 104 | HLA-DPB1 | "major histocompatibility complex, class II, DP beta 1 [Source:HGNC Symbol;Acc:HGNC:4940]" | 28094 | -0.054 | 0.0401 | No |
| 105 | C2 | complement C2 [Source:HGNC Symbol;Acc:HGNC:1248] | 28541 | -0.058 | 0.0340 | No |
| 106 | CD86 | CD86 molecule [Source:HGNC Symbol;Acc:HGNC:1705] | 28574 | -0.058 | 0.0386 | No |
| 107 | FCGR2C | Fc fragment of IgG receptor IIc (gene/pseudogene) [Source:HGNC Symbol;Acc:HGNC:15626] | 28748 | -0.059 | 0.0396 | No |
| 108 | H2BC21 | H2B clustered histone 21 [Source:HGNC Symbol;Acc:HGNC:4760] | 28826 | -0.060 | 0.0432 | No |
| 109 | HLA-DPA1 | "major histocompatibility complex, class II, DP alpha 1 [Source:HGNC Symbol;Acc:HGNC:4938]" | 29080 | -0.062 | 0.0425 | No |
| 110 | IFNG | interferon gamma [Source:HGNC Symbol;Acc:HGNC:5438] | 29128 | -0.063 | 0.0471 | No |
| 111 | HLA-DMB | "major histocompatibility complex, class II, DM beta [Source:HGNC Symbol;Acc:HGNC:4935]" | 29388 | -0.065 | 0.0465 | No |
| 112 | H2AB3 | H2A.B variant histone 3 [Source:HGNC Symbol;Acc:HGNC:14455] | 29416 | -0.065 | 0.0518 | No |
| 113 | CTSG | cathepsin G [Source:HGNC Symbol;Acc:HGNC:2532] | 29467 | -0.066 | 0.0566 | No |
| 114 | HLA-DRB5 | "major histocompatibility complex, class II, DR beta 5 [Source:HGNC Symbol;Acc:HGNC:4953]" | 29919 | -0.070 | 0.0515 | No |
| 115 | C1QA | complement C1q A chain [Source:HGNC Symbol;Acc:HGNC:1241] | 30000 | -0.071 | 0.0560 | No |
| 116 | FCGR3A | Fc fragment of IgG receptor IIIa [Source:HGNC Symbol;Acc:HGNC:3619] | 30111 | -0.072 | 0.0598 | No |
| 117 | HLA-DOA | "major histocompatibility complex, class II, DO alpha [Source:HGNC Symbol;Acc:HGNC:4936]" | 30255 | -0.073 | 0.0630 | No |
| 118 | FCGR1A | Fc fragment of IgG receptor Ia [Source:HGNC Symbol;Acc:HGNC:3613] | 30441 | -0.075 | 0.0652 | No |
| 119 | C1QC | complement C1q C chain [Source:HGNC Symbol;Acc:HGNC:1245] | 30659 | -0.077 | 0.0667 | No |
| 120 | H3-4 | H3.4 histone [Source:HGNC Symbol;Acc:HGNC:4778] | 30931 | -0.080 | 0.0671 | No |
| 121 | C1R | complement C1r [Source:HGNC Symbol;Acc:HGNC:1246] | 31040 | -0.081 | 0.0718 | No |
| 122 | H4-16 | H4 histone 16 [Source:HGNC Symbol;Acc:HGNC:20510] | 31191 | -0.082 | 0.0756 | No |
| 123 | GRIN2B | glutamate ionotropic receptor NMDA type subunit 2B [Source:HGNC Symbol;Acc:HGNC:4586] | 31248 | -0.083 | 0.0818 | No |
| 124 | C1QB | complement C1q B chain [Source:HGNC Symbol;Acc:HGNC:1242] | 31345 | -0.084 | 0.0871 | No |
| 125 | TRIM21 | tripartite motif containing 21 [Source:HGNC Symbol;Acc:HGNC:11312] | 31787 | -0.088 | 0.0840 | No |
| 126 | H2BW1 | H2B.W histone 1 [Source:HGNC Symbol;Acc:HGNC:27252] | 32158 | -0.093 | 0.0831 | No |
| 127 | HLA-DQA1 | "major histocompatibility complex, class II, DQ alpha 1 [Source:HGNC Symbol;Acc:HGNC:4942]" | 32609 | -0.098 | 0.0807 | No |
| 128 | CD80 | CD80 molecule [Source:HGNC Symbol;Acc:HGNC:1700] | 33643 | -0.113 | 0.0647 | No |
| 129 | CD40LG | CD40 ligand [Source:HGNC Symbol;Acc:HGNC:11935] | 34481 | -0.125 | 0.0549 | No |
| 130 | HLA-DOB | "major histocompatibility complex, class II, DO beta [Source:HGNC Symbol;Acc:HGNC:4937]" | 34609 | -0.128 | 0.0635 | No |
| 131 | C1S | complement C1s [Source:HGNC Symbol;Acc:HGNC:1247] | 34775 | -0.131 | 0.0713 | No |
| 132 | C5 | complement C5 [Source:HGNC Symbol;Acc:HGNC:1331] | 35416 | -0.145 | 0.0683 | No |
| 133 | RO60 | "Ro60, Y RNA binding protein [Source:HGNC Symbol;Acc:HGNC:11313]" | 35653 | -0.151 | 0.0762 | No |
| 134 | CD28 | CD28 molecule [Source:HGNC Symbol;Acc:HGNC:1653] | 36550 | -0.178 | 0.0698 | No |
| 135 | H2AC15 | H2A clustered histone 15 [Source:HGNC Symbol;Acc:HGNC:4726] | 38900 | NaN | 0.0108 | No |
Table: GSEA details [plain text format]

  

Fig 2: KEGG\_SYSTEMIC\_LUPUS\_ERYTHEMATOSUS      
 Blue-Pink O' Gram in the Space of the Analyzed GeneSet

  

Fig 3: KEGG\_SYSTEMIC\_LUPUS\_ERYTHEMATOSUS: Random ES distribution      
 Gene set null distribution of ES for **KEGG\_SYSTEMIC\_LUPUS\_ERYTHEMATOSUS**
