# Supplementary material for: Simultaneous Presentation of Multiple Myeloma and Lung Cancer: Case Report and Gene Bioinformatics Analysis
Source: Front Oncol. 2022 Jun 13;12:859735. doi: 10.3389/fonc.2022.859735 (PMC9235397; doi:10.3389/fonc.2022.859735)
Supplement: Supplementary file 1 [file DataSheet_1.zip › The bioinformatic analysis of MM and lung cancer supplementary materials/Enrichment analysis/MECR/GSEA_4.1.0/LUSC TCGA/KEGG.Gsea.1639042374131/KEGG_TERPENOID_BACKBONE_BIOSYNTHESIS.html]

Details for gene set KEGG\_TERPENOID\_BACKBONE\_BIOSYNTHESIS[GSEA]

|  || Dataset | ExpData\_collapsed\_to\_symbols.ENSG00000116353\_profile\_in\_ExpData.cls #ENSG00000116353 |
| Phenotype | ENSG00000116353\_profile\_in\_ExpData.cls#ENSG00000116353 |
| Upregulated in class | ENSG00000116353\_pos |
| GeneSet | KEGG\_TERPENOID\_BACKBONE\_BIOSYNTHESIS |
| Enrichment Score (ES) | 0.60979235 |
| Normalized Enrichment Score (NES) | 1.6614909 |
| Nominal p-value | 0.012552301 |
| FDR q-value | 0.026485696 |
| FWER p-Value | 0.467 |
Table: GSEA Results Summary

  

Fig 1: Enrichment plot: KEGG\_TERPENOID\_BACKBONE\_BIOSYNTHESIS      
 Profile of the Running ES Score & Positions of GeneSet Members on the Rank Ordered List

  

| SYMBOL | TITLE | RANK IN GENE LIST | RANK METRIC SCORE | RUNNING ES | CORE ENRICHMENT || 1 | DHDDS | dehydrodolichyl diphosphate synthase subunit [Source:HGNC Symbol;Acc:HGNC:20603] | 9 | 0.538 | 0.2762 | Yes |
| 2 | MVK | mevalonate kinase [Source:HGNC Symbol;Acc:HGNC:7530] | 946 | 0.219 | 0.3647 | Yes |
| 3 | PMVK | phosphomevalonate kinase [Source:HGNC Symbol;Acc:HGNC:9141] | 983 | 0.216 | 0.4746 | Yes |
| 4 | PDSS2 | decaprenyl diphosphate synthase subunit 2 [Source:HGNC Symbol;Acc:HGNC:23041] | 2210 | 0.158 | 0.5247 | Yes |
| 5 | PDSS1 | decaprenyl diphosphate synthase subunit 1 [Source:HGNC Symbol;Acc:HGNC:17759] | 4554 | 0.109 | 0.5211 | Yes |
| 6 | MVD | mevalonate diphosphate decarboxylase [Source:HGNC Symbol;Acc:HGNC:7529] | 5471 | 0.097 | 0.5477 | Yes |
| 7 | IDI2 | isopentenyl-diphosphate delta isomerase 2 [Source:HGNC Symbol;Acc:HGNC:23487] | 6559 | 0.088 | 0.5652 | Yes |
| 8 | HMGCS2 | 3-hydroxy-3-methylglutaryl-CoA synthase 2 [Source:HGNC Symbol;Acc:HGNC:5008] | 6578 | 0.088 | 0.6098 | Yes |
| 9 | GGPS1 | geranylgeranyl diphosphate synthase 1 [Source:HGNC Symbol;Acc:HGNC:4249] | 10007 | 0.060 | 0.5534 | No |
| 10 | IDI1 | isopentenyl-diphosphate delta isomerase 1 [Source:HGNC Symbol;Acc:HGNC:5387] | 20891 | -0.005 | 0.2789 | No |
| 11 | FDPS | farnesyl diphosphate synthase [Source:HGNC Symbol;Acc:HGNC:3631] | 26235 | -0.040 | 0.1635 | No |
| 12 | ACAT2 | acetyl-CoA acetyltransferase 2 [Source:HGNC Symbol;Acc:HGNC:94] | 28042 | -0.054 | 0.1451 | No |
| 13 | ACAT1 | acetyl-CoA acetyltransferase 1 [Source:HGNC Symbol;Acc:HGNC:93] | 28412 | -0.057 | 0.1648 | No |
| 14 | HMGCS1 | 3-hydroxy-3-methylglutaryl-CoA synthase 1 [Source:HGNC Symbol;Acc:HGNC:5007] | 30398 | -0.075 | 0.1526 | No |
| 15 | HMGCR | 3-hydroxy-3-methylglutaryl-CoA reductase [Source:HGNC Symbol;Acc:HGNC:5006] | 35425 | -0.145 | 0.0992 | No |
Table: GSEA details [plain text format]

  

Fig 2: KEGG\_TERPENOID\_BACKBONE\_BIOSYNTHESIS      
 Blue-Pink O' Gram in the Space of the Analyzed GeneSet

  

Fig 3: KEGG\_TERPENOID\_BACKBONE\_BIOSYNTHESIS: Random ES distribution      
 Gene set null distribution of ES for **KEGG\_TERPENOID\_BACKBONE\_BIOSYNTHESIS**
