# Supplementary material for: Simultaneous Presentation of Multiple Myeloma and Lung Cancer: Case Report and Gene Bioinformatics Analysis
Source: Front Oncol. 2022 Jun 13;12:859735. doi: 10.3389/fonc.2022.859735 (PMC9235397; doi:10.3389/fonc.2022.859735)
Supplement: Supplementary file 1 [file DataSheet_1.zip › The bioinformatic analysis of MM and lung cancer supplementary materials/Enrichment analysis/MECR/GSEA_4.1.0/LUSC TCGA/KEGG.Gsea.1639042374131/KEGG_TGF_BETA_SIGNALING_PATHWAY.html]

Details for gene set KEGG\_TGF\_BETA\_SIGNALING\_PATHWAY[GSEA]

|  || Dataset | ExpData\_collapsed\_to\_symbols.ENSG00000116353\_profile\_in\_ExpData.cls #ENSG00000116353 |
| Phenotype | ENSG00000116353\_profile\_in\_ExpData.cls#ENSG00000116353 |
| Upregulated in class | ENSG00000116353\_neg |
| GeneSet | KEGG\_TGF\_BETA\_SIGNALING\_PATHWAY |
| Enrichment Score (ES) | -0.4128386 |
| Normalized Enrichment Score (NES) | -1.7077909 |
| Nominal p-value | 0.0020661156 |
| FDR q-value | 0.021576408 |
| FWER p-Value | 0.305 |
Table: GSEA Results Summary

  

Fig 1: Enrichment plot: KEGG\_TGF\_BETA\_SIGNALING\_PATHWAY      
 Profile of the Running ES Score & Positions of GeneSet Members on the Rank Ordered List

  

| SYMBOL | TITLE | RANK IN GENE LIST | RANK METRIC SCORE | RUNNING ES | CORE ENRICHMENT || 1 | RPS6KB2 | ribosomal protein S6 kinase B2 [Source:HGNC Symbol;Acc:HGNC:10437] | 145 | 0.365 | 0.0393 | No |
| 2 | MAPK3 | mitogen-activated protein kinase 3 [Source:HGNC Symbol;Acc:HGNC:6877] | 473 | 0.274 | 0.0633 | No |
| 3 | PPP2R1A | protein phosphatase 2 scaffold subunit Aalpha [Source:HGNC Symbol;Acc:HGNC:9302] | 486 | 0.272 | 0.0951 | No |
| 4 | RBX1 | ring-box 1 [Source:HGNC Symbol;Acc:HGNC:9928] | 689 | 0.246 | 0.1190 | No |
| 5 | BMPR1B | bone morphogenetic protein receptor type 1B [Source:HGNC Symbol;Acc:HGNC:1077] | 722 | 0.242 | 0.1467 | No |
| 6 | GDF5 | growth differentiation factor 5 [Source:HGNC Symbol;Acc:HGNC:4220] | 2465 | 0.150 | 0.1200 | No |
| 7 | E2F5 | E2F transcription factor 5 [Source:HGNC Symbol;Acc:HGNC:3119] | 2496 | 0.149 | 0.1368 | No |
| 8 | RHOA | ras homolog family member A [Source:HGNC Symbol;Acc:HGNC:667] | 4969 | 0.103 | 0.0860 | No |
| 9 | TNF | tumor necrosis factor [Source:HGNC Symbol;Acc:HGNC:11892] | 7309 | 0.081 | 0.0359 | No |
| 10 | SKP1 | S-phase kinase associated protein 1 [Source:HGNC Symbol;Acc:HGNC:10899] | 8628 | 0.070 | 0.0105 | No |
| 11 | TGFB2 | transforming growth factor beta 2 [Source:HGNC Symbol;Acc:HGNC:11768] | 8941 | 0.068 | 0.0106 | No |
| 12 | BMP5 | bone morphogenetic protein 5 [Source:HGNC Symbol;Acc:HGNC:1072] | 12002 | 0.047 | -0.0619 | No |
| 13 | NOG | noggin [Source:HGNC Symbol;Acc:HGNC:7866] | 12032 | 0.047 | -0.0571 | No |
| 14 | E2F4 | E2F transcription factor 4 [Source:HGNC Symbol;Acc:HGNC:3118] | 12259 | 0.046 | -0.0574 | No |
| 15 | BMPR1A | bone morphogenetic protein receptor type 1A [Source:HGNC Symbol;Acc:HGNC:1076] | 12749 | 0.042 | -0.0649 | No |
| 16 | PPP2CA | protein phosphatase 2 catalytic subunit alpha [Source:HGNC Symbol;Acc:HGNC:9299] | 13727 | 0.037 | -0.0855 | No |
| 17 | ID1 | "inhibitor of DNA binding 1, HLH protein [Source:HGNC Symbol;Acc:HGNC:5360]" | 13763 | 0.036 | -0.0821 | No |
| 18 | DCN | decorin [Source:HGNC Symbol;Acc:HGNC:2705] | 14309 | 0.033 | -0.0921 | No |
| 19 | LEFTY2 | left-right determination factor 2 [Source:HGNC Symbol;Acc:HGNC:3122] | 14845 | 0.030 | -0.1022 | No |
| 20 | SMAD6 | SMAD family member 6 [Source:HGNC Symbol;Acc:HGNC:6772] | 14940 | 0.030 | -0.1011 | No |
| 21 | SMAD7 | SMAD family member 7 [Source:HGNC Symbol;Acc:HGNC:6773] | 15299 | 0.028 | -0.1070 | No |
| 22 | TGFBR2 | transforming growth factor beta receptor 2 [Source:HGNC Symbol;Acc:HGNC:11773] | 15982 | 0.023 | -0.1216 | No |
| 23 | LEFTY1 | left-right determination factor 1 [Source:HGNC Symbol;Acc:HGNC:6552] | 16128 | 0.023 | -0.1226 | No |
| 24 | LTBP1 | latent transforming growth factor beta binding protein 1 [Source:HGNC Symbol;Acc:HGNC:6714] | 16608 | 0.020 | -0.1325 | No |
| 25 | TGFB1 | transforming growth factor beta 1 [Source:HGNC Symbol;Acc:HGNC:11766] | 17301 | 0.016 | -0.1482 | No |
| 26 | ID3 | "inhibitor of DNA binding 3, HLH protein [Source:HGNC Symbol;Acc:HGNC:5362]" | 17673 | 0.014 | -0.1560 | No |
| 27 | COMP | cartilage oligomeric matrix protein [Source:HGNC Symbol;Acc:HGNC:2227] | 17696 | 0.014 | -0.1550 | No |
| 28 | CDKN2B | cyclin dependent kinase inhibitor 2B [Source:HGNC Symbol;Acc:HGNC:1788] | 18975 | 0.007 | -0.1867 | No |
| 29 | MYC | "MYC proto-oncogene, bHLH transcription factor [Source:HGNC Symbol;Acc:HGNC:7553]" | 19309 | 0.005 | -0.1947 | No |
| 30 | ID4 | "inhibitor of DNA binding 4, HLH protein [Source:HGNC Symbol;Acc:HGNC:5363]" | 20235 | -0.001 | -0.2182 | No |
| 31 | BMP2 | bone morphogenetic protein 2 [Source:HGNC Symbol;Acc:HGNC:1069] | 20791 | -0.004 | -0.2318 | No |
| 32 | TFDP1 | transcription factor Dp-1 [Source:HGNC Symbol;Acc:HGNC:11749] | 20864 | -0.004 | -0.2331 | No |
| 33 | THBS3 | thrombospondin 3 [Source:HGNC Symbol;Acc:HGNC:11787] | 20969 | -0.005 | -0.2352 | No |
| 34 | ACVR1C | activin A receptor type 1C [Source:HGNC Symbol;Acc:HGNC:18123] | 21077 | -0.006 | -0.2372 | No |
| 35 | ZFYVE9 | zinc finger FYVE-type containing 9 [Source:HGNC Symbol;Acc:HGNC:6775] | 21979 | -0.011 | -0.2589 | No |
| 36 | SKP1P2 | S-phase kinase associated protein 1 pseudogene 2 [Source:HGNC Symbol;Acc:HGNC:10900] | 22230 | -0.013 | -0.2638 | No |
| 37 | PITX2 | paired like homeodomain 2 [Source:HGNC Symbol;Acc:HGNC:9005] | 22498 | -0.014 | -0.2689 | No |
| 38 | TGFB3 | transforming growth factor beta 3 [Source:HGNC Symbol;Acc:HGNC:11769] | 23618 | -0.021 | -0.2949 | No |
| 39 | BMP8B | bone morphogenetic protein 8b [Source:HGNC Symbol;Acc:HGNC:1075] | 23652 | -0.022 | -0.2932 | No |
| 40 | PPP2CB | protein phosphatase 2 catalytic subunit beta [Source:HGNC Symbol;Acc:HGNC:9300] | 25064 | -0.032 | -0.3254 | No |
| 41 | INHBA | inhibin subunit beta A [Source:HGNC Symbol;Acc:HGNC:6066] | 25321 | -0.033 | -0.3280 | No |
| 42 | ACVR2A | activin A receptor type 2A [Source:HGNC Symbol;Acc:HGNC:173] | 25447 | -0.034 | -0.3271 | No |
| 43 | THBS1 | thrombospondin 1 [Source:HGNC Symbol;Acc:HGNC:11785] | 27428 | -0.049 | -0.3718 | No |
| 44 | GDF6 | growth differentiation factor 6 [Source:HGNC Symbol;Acc:HGNC:4221] | 28363 | -0.056 | -0.3890 | No |
| 45 | IFNG | interferon gamma [Source:HGNC Symbol;Acc:HGNC:5438] | 29128 | -0.063 | -0.4011 | No |
| 46 | NODAL | nodal growth differentiation factor [Source:HGNC Symbol;Acc:HGNC:7865] | 29436 | -0.066 | -0.4012 | No |
| 47 | BMP4 | bone morphogenetic protein 4 [Source:HGNC Symbol;Acc:HGNC:1071] | 29894 | -0.070 | -0.4046 | Yes |
| 48 | BMP8A | bone morphogenetic protein 8a [Source:HGNC Symbol;Acc:HGNC:21650] | 30182 | -0.072 | -0.4034 | Yes |
| 49 | BMPR2 | bone morphogenetic protein receptor type 2 [Source:HGNC Symbol;Acc:HGNC:1078] | 30422 | -0.075 | -0.4007 | Yes |
| 50 | RBL1 | RB transcriptional corepressor like 1 [Source:HGNC Symbol;Acc:HGNC:9893] | 30686 | -0.077 | -0.3983 | Yes |
| 51 | ACVRL1 | activin A receptor like type 1 [Source:HGNC Symbol;Acc:HGNC:175] | 30966 | -0.080 | -0.3960 | Yes |
| 52 | AMHR2 | anti-Mullerian hormone receptor type 2 [Source:HGNC Symbol;Acc:HGNC:465] | 31506 | -0.085 | -0.3996 | Yes |
| 53 | THBS2 | thrombospondin 2 [Source:HGNC Symbol;Acc:HGNC:11786] | 31757 | -0.088 | -0.3956 | Yes |
| 54 | INHBC | inhibin subunit beta C [Source:HGNC Symbol;Acc:HGNC:6068] | 32033 | -0.091 | -0.3919 | Yes |
| 55 | SMAD1 | SMAD family member 1 [Source:HGNC Symbol;Acc:HGNC:6767] | 32225 | -0.093 | -0.3857 | Yes |
| 56 | ID2 | inhibitor of DNA binding 2 [Source:HGNC Symbol;Acc:HGNC:5361] | 32335 | -0.095 | -0.3773 | Yes |
| 57 | INHBE | inhibin subunit beta E [Source:HGNC Symbol;Acc:HGNC:24029] | 32686 | -0.099 | -0.3746 | Yes |
| 58 | SMAD4 | SMAD family member 4 [Source:HGNC Symbol;Acc:HGNC:6770] | 32731 | -0.100 | -0.3639 | Yes |
| 59 | SMURF1 | SMAD specific E3 ubiquitin protein ligase 1 [Source:HGNC Symbol;Acc:HGNC:16807] | 32837 | -0.101 | -0.3547 | Yes |
| 60 | CHRD | chordin [Source:HGNC Symbol;Acc:HGNC:1949] | 33120 | -0.105 | -0.3495 | Yes |
| 61 | BMP7 | bone morphogenetic protein 7 [Source:HGNC Symbol;Acc:HGNC:1074] | 33291 | -0.107 | -0.3412 | Yes |
| 62 | MAPK1 | mitogen-activated protein kinase 1 [Source:HGNC Symbol;Acc:HGNC:6871] | 33350 | -0.108 | -0.3299 | Yes |
| 63 | ACVR2B | activin A receptor type 2B [Source:HGNC Symbol;Acc:HGNC:174] | 33447 | -0.110 | -0.3194 | Yes |
| 64 | ZFYVE16 | zinc finger FYVE-type containing 16 [Source:HGNC Symbol;Acc:HGNC:20756] | 33809 | -0.115 | -0.3150 | Yes |
| 65 | GDF7 | growth differentiation factor 7 [Source:HGNC Symbol;Acc:HGNC:4222] | 34247 | -0.122 | -0.3119 | Yes |
| 66 | INHBB | inhibin subunit beta B [Source:HGNC Symbol;Acc:HGNC:6067] | 34315 | -0.123 | -0.2991 | Yes |
| 67 | SMURF2 | SMAD specific E3 ubiquitin protein ligase 2 [Source:HGNC Symbol;Acc:HGNC:16809] | 34381 | -0.124 | -0.2862 | Yes |
| 68 | RPS6KB1 | ribosomal protein S6 kinase B1 [Source:HGNC Symbol;Acc:HGNC:10436] | 34832 | -0.132 | -0.2821 | Yes |
| 69 | SMAD2 | SMAD family member 2 [Source:HGNC Symbol;Acc:HGNC:6768] | 34897 | -0.133 | -0.2680 | Yes |
| 70 | PPP2R1B | protein phosphatase 2 scaffold subunit Abeta [Source:HGNC Symbol;Acc:HGNC:9303] | 35047 | -0.137 | -0.2557 | Yes |
| 71 | SMAD9 | SMAD family member 9 [Source:HGNC Symbol;Acc:HGNC:6774] | 35326 | -0.142 | -0.2460 | Yes |
| 72 | RBL2 | RB transcriptional corepressor like 2 [Source:HGNC Symbol;Acc:HGNC:9894] | 35347 | -0.143 | -0.2296 | Yes |
| 73 | SMAD3 | SMAD family member 3 [Source:HGNC Symbol;Acc:HGNC:6769] | 35374 | -0.144 | -0.2133 | Yes |
| 74 | ACVR1 | activin A receptor type 1 [Source:HGNC Symbol;Acc:HGNC:171] | 35400 | -0.144 | -0.1970 | Yes |
| 75 | CUL1 | cullin 1 [Source:HGNC Symbol;Acc:HGNC:2551] | 35662 | -0.151 | -0.1858 | Yes |
| 76 | FST | follistatin [Source:HGNC Symbol;Acc:HGNC:3971] | 35665 | -0.151 | -0.1681 | Yes |
| 77 | EP300 | E1A binding protein p300 [Source:HGNC Symbol;Acc:HGNC:3373] | 36409 | -0.173 | -0.1666 | Yes |
| 78 | BMP6 | bone morphogenetic protein 6 [Source:HGNC Symbol;Acc:HGNC:1073] | 36657 | -0.182 | -0.1514 | Yes |
| 79 | THBS4 | thrombospondin 4 [Source:HGNC Symbol;Acc:HGNC:11788] | 36673 | -0.183 | -0.1302 | Yes |
| 80 | SP1 | Sp1 transcription factor [Source:HGNC Symbol;Acc:HGNC:11205] | 37072 | -0.201 | -0.1167 | Yes |
| 81 | CREBBP | CREB binding protein [Source:HGNC Symbol;Acc:HGNC:2348] | 37476 | -0.222 | -0.1008 | Yes |
| 82 | SMAD5 | SMAD family member 5 [Source:HGNC Symbol;Acc:HGNC:6771] | 37502 | -0.224 | -0.0750 | Yes |
| 83 | AMH | anti-Mullerian hormone [Source:HGNC Symbol;Acc:HGNC:464] | 37614 | -0.232 | -0.0505 | Yes |
| 84 | ROCK1 | Rho associated coiled-coil containing protein kinase 1 [Source:HGNC Symbol;Acc:HGNC:10251] | 37709 | -0.238 | -0.0248 | Yes |
| 85 | TGFBR1 | transforming growth factor beta receptor 1 [Source:HGNC Symbol;Acc:HGNC:11772] | 37905 | -0.255 | 0.0003 | Yes |
| 86 | ROCK2 | Rho associated coiled-coil containing protein kinase 2 [Source:HGNC Symbol;Acc:HGNC:10252] | 38278 | -0.304 | 0.0266 | Yes |
Table: GSEA details [plain text format]

  

Fig 2: KEGG\_TGF\_BETA\_SIGNALING\_PATHWAY      
 Blue-Pink O' Gram in the Space of the Analyzed GeneSet

  

Fig 3: KEGG\_TGF\_BETA\_SIGNALING\_PATHWAY: Random ES distribution      
 Gene set null distribution of ES for **KEGG\_TGF\_BETA\_SIGNALING\_PATHWAY**
