# Supplementary material for: Simultaneous Presentation of Multiple Myeloma and Lung Cancer: Case Report and Gene Bioinformatics Analysis
Source: Front Oncol. 2022 Jun 13;12:859735. doi: 10.3389/fonc.2022.859735 (PMC9235397; doi:10.3389/fonc.2022.859735)
Supplement: Supplementary file 1 [file DataSheet_1.zip › The bioinformatic analysis of MM and lung cancer supplementary materials/Enrichment analysis/MECR/GSEA_4.1.0/LUSC TCGA/KEGG.Gsea.1639042374131/KEGG_VALINE_LEUCINE_AND_ISOLEUCINE_DEGRADATION.html]

Details for gene set KEGG\_VALINE\_LEUCINE\_AND\_ISOLEUCINE\_DEGRADATION[GSEA]

|  || Dataset | ExpData\_collapsed\_to\_symbols.ENSG00000116353\_profile\_in\_ExpData.cls #ENSG00000116353 |
| Phenotype | ENSG00000116353\_profile\_in\_ExpData.cls#ENSG00000116353 |
| Upregulated in class | ENSG00000116353\_pos |
| GeneSet | KEGG\_VALINE\_LEUCINE\_AND\_ISOLEUCINE\_DEGRADATION |
| Enrichment Score (ES) | 0.54799503 |
| Normalized Enrichment Score (NES) | 1.9808251 |
| Nominal p-value | 0.0 |
| FDR q-value | 0.0015462737 |
| FWER p-Value | 0.022 |
Table: GSEA Results Summary

  

Fig 1: Enrichment plot: KEGG\_VALINE\_LEUCINE\_AND\_ISOLEUCINE\_DEGRADATION      
 Profile of the Running ES Score & Positions of GeneSet Members on the Rank Ordered List

  

| SYMBOL | TITLE | RANK IN GENE LIST | RANK METRIC SCORE | RUNNING ES | CORE ENRICHMENT || 1 | HMGCL | 3-hydroxy-3-methylglutaryl-CoA lyase [Source:HGNC Symbol;Acc:HGNC:5005] | 18 | 0.508 | 0.0832 | Yes |
| 2 | ECHS1 | "enoyl-CoA hydratase, short chain 1 [Source:HGNC Symbol;Acc:HGNC:3151]" | 132 | 0.372 | 0.1416 | Yes |
| 3 | BCAT2 | branched chain amino acid transaminase 2 [Source:HGNC Symbol;Acc:HGNC:977] | 231 | 0.329 | 0.1932 | Yes |
| 4 | HADH | hydroxyacyl-CoA dehydrogenase [Source:HGNC Symbol;Acc:HGNC:4799] | 366 | 0.293 | 0.2380 | Yes |
| 5 | ACADM | acyl-CoA dehydrogenase medium chain [Source:HGNC Symbol;Acc:HGNC:89] | 665 | 0.249 | 0.2714 | Yes |
| 6 | OXCT1 | 3-oxoacid CoA-transferase 1 [Source:HGNC Symbol;Acc:HGNC:8527] | 770 | 0.236 | 0.3076 | Yes |
| 7 | BCKDHA | branched chain keto acid dehydrogenase E1 subunit alpha [Source:HGNC Symbol;Acc:HGNC:986] | 962 | 0.218 | 0.3385 | Yes |
| 8 | ACAD8 | acyl-CoA dehydrogenase family member 8 [Source:HGNC Symbol;Acc:HGNC:87] | 1000 | 0.215 | 0.3729 | Yes |
| 9 | DBT | dihydrolipoamide branched chain transacylase E2 [Source:HGNC Symbol;Acc:HGNC:2698] | 1220 | 0.202 | 0.4005 | Yes |
| 10 | ALDH7A1 | aldehyde dehydrogenase 7 family member A1 [Source:HGNC Symbol;Acc:HGNC:877] | 1270 | 0.199 | 0.4320 | Yes |
| 11 | HSD17B10 | hydroxysteroid 17-beta dehydrogenase 10 [Source:HGNC Symbol;Acc:HGNC:4800] | 1578 | 0.183 | 0.4542 | Yes |
| 12 | ACADS | acyl-CoA dehydrogenase short chain [Source:HGNC Symbol;Acc:HGNC:90] | 1766 | 0.175 | 0.4782 | Yes |
| 13 | ACAA2 | acetyl-CoA acyltransferase 2 [Source:HGNC Symbol;Acc:HGNC:83] | 2060 | 0.163 | 0.4976 | Yes |
| 14 | MCEE | methylmalonyl-CoA epimerase [Source:HGNC Symbol;Acc:HGNC:16732] | 2531 | 0.149 | 0.5101 | Yes |
| 15 | OXCT2 | 3-oxoacid CoA-transferase 2 [Source:HGNC Symbol;Acc:HGNC:18606] | 2837 | 0.141 | 0.5255 | Yes |
| 16 | HADHB | hydroxyacyl-CoA dehydrogenase trifunctional multienzyme complex subunit beta [Source:HGNC Symbol;Acc:HGNC:4803] | 3434 | 0.127 | 0.5313 | Yes |
| 17 | HADHA | hydroxyacyl-CoA dehydrogenase trifunctional multienzyme complex subunit alpha [Source:HGNC Symbol;Acc:HGNC:4801] | 3803 | 0.121 | 0.5419 | Yes |
| 18 | ABAT | 4-aminobutyrate aminotransferase [Source:HGNC Symbol;Acc:HGNC:23] | 4397 | 0.111 | 0.5451 | Yes |
| 19 | HIBCH | 3-hydroxyisobutyryl-CoA hydrolase [Source:HGNC Symbol;Acc:HGNC:4908] | 4951 | 0.103 | 0.5480 | Yes |
| 20 | HMGCS2 | 3-hydroxy-3-methylglutaryl-CoA synthase 2 [Source:HGNC Symbol;Acc:HGNC:5008] | 6578 | 0.088 | 0.5210 | No |
| 21 | PCCA | propionyl-CoA carboxylase subunit alpha [Source:HGNC Symbol;Acc:HGNC:8653] | 7009 | 0.084 | 0.5238 | No |
| 22 | PCCB | propionyl-CoA carboxylase subunit beta [Source:HGNC Symbol;Acc:HGNC:8654] | 7261 | 0.081 | 0.5309 | No |
| 23 | BCKDHB | branched chain keto acid dehydrogenase E1 subunit beta [Source:HGNC Symbol;Acc:HGNC:987] | 7978 | 0.075 | 0.5250 | No |
| 24 | ACAA1 | acetyl-CoA acyltransferase 1 [Source:HGNC Symbol;Acc:HGNC:82] | 8047 | 0.075 | 0.5355 | No |
| 25 | HIBADH | 3-hydroxyisobutyrate dehydrogenase [Source:HGNC Symbol;Acc:HGNC:4907] | 9279 | 0.065 | 0.5149 | No |
| 26 | ALDH6A1 | aldehyde dehydrogenase 6 family member A1 [Source:HGNC Symbol;Acc:HGNC:7179] | 9302 | 0.065 | 0.5251 | No |
| 27 | MMUT | methylmalonyl-CoA mutase [Source:HGNC Symbol;Acc:HGNC:7526] | 14034 | 0.035 | 0.4104 | No |
| 28 | ALDH2 | aldehyde dehydrogenase 2 family member [Source:HGNC Symbol;Acc:HGNC:404] | 17769 | 0.013 | 0.3175 | No |
| 29 | ACADSB | acyl-CoA dehydrogenase short/branched chain [Source:HGNC Symbol;Acc:HGNC:91] | 24794 | -0.030 | 0.1436 | No |
| 30 | ACAT2 | acetyl-CoA acetyltransferase 2 [Source:HGNC Symbol;Acc:HGNC:94] | 28042 | -0.054 | 0.0698 | No |
| 31 | ACAT1 | acetyl-CoA acetyltransferase 1 [Source:HGNC Symbol;Acc:HGNC:93] | 28412 | -0.057 | 0.0697 | No |
| 32 | AUH | AU RNA binding methylglutaconyl-CoA hydratase [Source:HGNC Symbol;Acc:HGNC:890] | 29181 | -0.063 | 0.0605 | No |
| 33 | MCCC2 | methylcrotonoyl-CoA carboxylase 2 [Source:HGNC Symbol;Acc:HGNC:6937] | 29497 | -0.066 | 0.0634 | No |
| 34 | EHHADH | enoyl-CoA hydratase and 3-hydroxyacyl CoA dehydrogenase [Source:HGNC Symbol;Acc:HGNC:3247] | 29653 | -0.067 | 0.0705 | No |
| 35 | IL4I1 | interleukin 4 induced 1 [Source:HGNC Symbol;Acc:HGNC:19094] | 29943 | -0.070 | 0.0747 | No |
| 36 | ALDH9A1 | aldehyde dehydrogenase 9 family member A1 [Source:HGNC Symbol;Acc:HGNC:412] | 30215 | -0.073 | 0.0798 | No |
| 37 | HMGCS1 | 3-hydroxy-3-methylglutaryl-CoA synthase 1 [Source:HGNC Symbol;Acc:HGNC:5007] | 30398 | -0.075 | 0.0874 | No |
| 38 | AOX1 | aldehyde oxidase 1 [Source:HGNC Symbol;Acc:HGNC:553] | 30799 | -0.078 | 0.0901 | No |
| 39 | IVD | isovaleryl-CoA dehydrogenase [Source:HGNC Symbol;Acc:HGNC:6186] | 32210 | -0.093 | 0.0695 | No |
| 40 | BCAT1 | branched chain amino acid transaminase 1 [Source:HGNC Symbol;Acc:HGNC:976] | 32467 | -0.096 | 0.0789 | No |
| 41 | ALDH1B1 | aldehyde dehydrogenase 1 family member B1 [Source:HGNC Symbol;Acc:HGNC:407] | 32656 | -0.099 | 0.0903 | No |
| 42 | DLD | dihydrolipoamide dehydrogenase [Source:HGNC Symbol;Acc:HGNC:2898] | 33217 | -0.106 | 0.0936 | No |
| 43 | MCCC1 | methylcrotonoyl-CoA carboxylase 1 [Source:HGNC Symbol;Acc:HGNC:6936] | 35970 | -0.159 | 0.0498 | No |
| 44 | ALDH3A2 | aldehyde dehydrogenase 3 family member A2 [Source:HGNC Symbol;Acc:HGNC:403] | 37378 | -0.216 | 0.0495 | No |
Table: GSEA details [plain text format]

  

Fig 2: KEGG\_VALINE\_LEUCINE\_AND\_ISOLEUCINE\_DEGRADATION      
 Blue-Pink O' Gram in the Space of the Analyzed GeneSet

  

Fig 3: KEGG\_VALINE\_LEUCINE\_AND\_ISOLEUCINE\_DEGRADATION: Random ES distribution      
 Gene set null distribution of ES for **KEGG\_VALINE\_LEUCINE\_AND\_ISOLEUCINE\_DEGRADATION**
