# Supplementary material for: Simultaneous Presentation of Multiple Myeloma and Lung Cancer: Case Report and Gene Bioinformatics Analysis
Source: Front Oncol. 2022 Jun 13;12:859735. doi: 10.3389/fonc.2022.859735 (PMC9235397; doi:10.3389/fonc.2022.859735)
Supplement: Supplementary file 1 [file DataSheet_1.zip › The bioinformatic analysis of MM and lung cancer supplementary materials/Enrichment analysis/MECR/GSEA_4.1.0/LUSC TCGA/KEGG.Gsea.1639042374131/KEGG_VASCULAR_SMOOTH_MUSCLE_CONTRACTION.html]

Details for gene set KEGG\_VASCULAR\_SMOOTH\_MUSCLE\_CONTRACTION[GSEA]

|  || Dataset | ExpData\_collapsed\_to\_symbols.ENSG00000116353\_profile\_in\_ExpData.cls #ENSG00000116353 |
| Phenotype | ENSG00000116353\_profile\_in\_ExpData.cls#ENSG00000116353 |
| Upregulated in class | ENSG00000116353\_neg |
| GeneSet | KEGG\_VASCULAR\_SMOOTH\_MUSCLE\_CONTRACTION |
| Enrichment Score (ES) | -0.44100097 |
| Normalized Enrichment Score (NES) | -1.9337986 |
| Nominal p-value | 0.0 |
| FDR q-value | 0.003610971 |
| FWER p-Value | 0.023 |
Table: GSEA Results Summary

  

Fig 1: Enrichment plot: KEGG\_VASCULAR\_SMOOTH\_MUSCLE\_CONTRACTION      
 Profile of the Running ES Score & Positions of GeneSet Members on the Rank Ordered List

  

| SYMBOL | TITLE | RANK IN GENE LIST | RANK METRIC SCORE | RUNNING ES | CORE ENRICHMENT || 1 | PPP1CA | protein phosphatase 1 catalytic subunit alpha [Source:HGNC Symbol;Acc:HGNC:9281] | 301 | 0.306 | 0.0151 | No |
| 2 | MYL6B | myosin light chain 6B [Source:HGNC Symbol;Acc:HGNC:29823] | 358 | 0.294 | 0.0355 | No |
| 3 | MYL6 | myosin light chain 6 [Source:HGNC Symbol;Acc:HGNC:7587] | 375 | 0.291 | 0.0567 | No |
| 4 | MAPK3 | mitogen-activated protein kinase 3 [Source:HGNC Symbol;Acc:HGNC:6877] | 473 | 0.274 | 0.0746 | No |
| 5 | PLA2G12A | phospholipase A2 group XIIA [Source:HGNC Symbol;Acc:HGNC:18554] | 582 | 0.258 | 0.0910 | No |
| 6 | CALM3 | calmodulin 3 [Source:HGNC Symbol;Acc:HGNC:1449] | 850 | 0.227 | 0.1011 | No |
| 7 | PLCB3 | phospholipase C beta 3 [Source:HGNC Symbol;Acc:HGNC:9056] | 1971 | 0.167 | 0.0849 | No |
| 8 | CACNA1D | calcium voltage-gated channel subunit alpha1 D [Source:HGNC Symbol;Acc:HGNC:1391] | 3402 | 0.128 | 0.0580 | No |
| 9 | CYP4A11 | cytochrome P450 family 4 subfamily A member 11 [Source:HGNC Symbol;Acc:HGNC:2642] | 3622 | 0.124 | 0.0616 | No |
| 10 | KCNMB4 | potassium calcium-activated channel subfamily M regulatory beta subunit 4 [Source:HGNC Symbol;Acc:HGNC:6289] | 3624 | 0.124 | 0.0708 | No |
| 11 | MYLK2 | myosin light chain kinase 2 [Source:HGNC Symbol;Acc:HGNC:16243] | 3703 | 0.123 | 0.0779 | No |
| 12 | GNAS | GNAS complex locus [Source:HGNC Symbol;Acc:HGNC:4392] | 4003 | 0.118 | 0.0790 | No |
| 13 | PLA2G2C | phospholipase A2 group IIC [Source:HGNC Symbol;Acc:HGNC:9032] | 4582 | 0.109 | 0.0724 | No |
| 14 | PLA2G1B | phospholipase A2 group IB [Source:HGNC Symbol;Acc:HGNC:9030] | 4747 | 0.106 | 0.0761 | No |
| 15 | RHOA | ras homolog family member A [Source:HGNC Symbol;Acc:HGNC:667] | 4969 | 0.103 | 0.0781 | No |
| 16 | PPP1R14A | protein phosphatase 1 regulatory inhibitor subunit 14A [Source:HGNC Symbol;Acc:HGNC:14871] | 5018 | 0.102 | 0.0845 | No |
| 17 | ARAF | "A-Raf proto-oncogene, serine/threonine kinase [Source:HGNC Symbol;Acc:HGNC:646]" | 5337 | 0.099 | 0.0837 | No |
| 18 | CALM2 | calmodulin 2 [Source:HGNC Symbol;Acc:HGNC:1445] | 6292 | 0.091 | 0.0661 | No |
| 19 | AGTR1 | angiotensin II receptor type 1 [Source:HGNC Symbol;Acc:HGNC:336] | 6417 | 0.089 | 0.0696 | No |
| 20 | CALML5 | calmodulin like 5 [Source:HGNC Symbol;Acc:HGNC:18180] | 6619 | 0.087 | 0.0710 | No |
| 21 | PPP1R12B | protein phosphatase 1 regulatory subunit 12B [Source:HGNC Symbol;Acc:HGNC:7619] | 6802 | 0.086 | 0.0727 | No |
| 22 | CYP4A22 | cytochrome P450 family 4 subfamily A member 22 [Source:HGNC Symbol;Acc:HGNC:20575] | 7705 | 0.077 | 0.0554 | No |
| 23 | MYL9 | myosin light chain 9 [Source:HGNC Symbol;Acc:HGNC:15754] | 7885 | 0.076 | 0.0565 | No |
| 24 | MYLK3 | myosin light chain kinase 3 [Source:HGNC Symbol;Acc:HGNC:29826] | 8377 | 0.072 | 0.0493 | No |
| 25 | PLA2G12B | phospholipase A2 group XIIB [Source:HGNC Symbol;Acc:HGNC:18555] | 9402 | 0.065 | 0.0280 | No |
| 26 | PLA2G2E | phospholipase A2 group IIE [Source:HGNC Symbol;Acc:HGNC:13414] | 9748 | 0.062 | 0.0238 | No |
| 27 | ADRA1B | adrenoceptor alpha 1B [Source:HGNC Symbol;Acc:HGNC:278] | 10678 | 0.056 | 0.0043 | No |
| 28 | PLA2G2F | phospholipase A2 group IIF [Source:HGNC Symbol;Acc:HGNC:30040] | 10874 | 0.054 | 0.0033 | No |
| 29 | ADORA2B | adenosine A2b receptor [Source:HGNC Symbol;Acc:HGNC:264] | 14239 | 0.033 | -0.0800 | No |
| 30 | RAMP1 | receptor activity modifying protein 1 [Source:HGNC Symbol;Acc:HGNC:9843] | 14320 | 0.033 | -0.0796 | No |
| 31 | PRKCQ | protein kinase C theta [Source:HGNC Symbol;Acc:HGNC:9410] | 14350 | 0.033 | -0.0779 | No |
| 32 | RAMP2 | receptor activity modifying protein 2 [Source:HGNC Symbol;Acc:HGNC:9844] | 14864 | 0.030 | -0.0887 | No |
| 33 | PRKCG | protein kinase C gamma [Source:HGNC Symbol;Acc:HGNC:9402] | 15351 | 0.027 | -0.0991 | No |
| 34 | MAP2K2 | mitogen-activated protein kinase kinase 2 [Source:HGNC Symbol;Acc:HGNC:6842] | 15510 | 0.026 | -0.1012 | No |
| 35 | PLA2G5 | phospholipase A2 group V [Source:HGNC Symbol;Acc:HGNC:9038] | 15541 | 0.026 | -0.1000 | No |
| 36 | ADRA1D | adrenoceptor alpha 1D [Source:HGNC Symbol;Acc:HGNC:280] | 16099 | 0.023 | -0.1125 | No |
| 37 | RAF1 | "Raf-1 proto-oncogene, serine/threonine kinase [Source:HGNC Symbol;Acc:HGNC:9829]" | 16140 | 0.022 | -0.1119 | No |
| 38 | EDNRA | endothelin receptor type A [Source:HGNC Symbol;Acc:HGNC:3179] | 16969 | 0.018 | -0.1317 | No |
| 39 | ARHGEF1 | Rho guanine nucleotide exchange factor 1 [Source:HGNC Symbol;Acc:HGNC:681] | 19147 | 0.006 | -0.1868 | No |
| 40 | ADORA2A | adenosine A2a receptor [Source:HGNC Symbol;Acc:HGNC:263] | 19525 | 0.003 | -0.1961 | No |
| 41 | ADCY6 | adenylate cyclase 6 [Source:HGNC Symbol;Acc:HGNC:237] | 20109 | 0.000 | -0.2110 | No |
| 42 | CALML6 | calmodulin like 6 [Source:HGNC Symbol;Acc:HGNC:24193] | 20499 | -0.002 | -0.2208 | No |
| 43 | ACTG2 | "actin gamma 2, smooth muscle [Source:HGNC Symbol;Acc:HGNC:145]" | 20931 | -0.005 | -0.2314 | No |
| 44 | PRKCE | protein kinase C epsilon [Source:HGNC Symbol;Acc:HGNC:9401] | 21240 | -0.007 | -0.2387 | No |
| 45 | PRKACA | protein kinase cAMP-activated catalytic subunit alpha [Source:HGNC Symbol;Acc:HGNC:9380] | 21332 | -0.007 | -0.2405 | No |
| 46 | CACNA1C | calcium voltage-gated channel subunit alpha1 C [Source:HGNC Symbol;Acc:HGNC:1390] | 21681 | -0.009 | -0.2487 | No |
| 47 | CALM1 | calmodulin 1 [Source:HGNC Symbol;Acc:HGNC:1442] | 21884 | -0.010 | -0.2531 | No |
| 48 | AVPR1B | arginine vasopressin receptor 1B [Source:HGNC Symbol;Acc:HGNC:896] | 21931 | -0.011 | -0.2535 | No |
| 49 | MYLK | myosin light chain kinase [Source:HGNC Symbol;Acc:HGNC:7590] | 23932 | -0.023 | -0.3027 | No |
| 50 | GNA12 | G protein subunit alpha 12 [Source:HGNC Symbol;Acc:HGNC:4380] | 24506 | -0.027 | -0.3153 | No |
| 51 | PPP1CC | protein phosphatase 1 catalytic subunit gamma [Source:HGNC Symbol;Acc:HGNC:9283] | 25009 | -0.031 | -0.3258 | No |
| 52 | KCNMB1 | potassium calcium-activated channel subfamily M regulatory beta subunit 1 [Source:HGNC Symbol;Acc:HGNC:6285] | 25079 | -0.032 | -0.3252 | No |
| 53 | PTGIR | prostaglandin I2 receptor [Source:HGNC Symbol;Acc:HGNC:9602] | 25187 | -0.033 | -0.3255 | No |
| 54 | CALD1 | caldesmon 1 [Source:HGNC Symbol;Acc:HGNC:1441] | 25188 | -0.033 | -0.3231 | No |
| 55 | PLA2G6 | phospholipase A2 group VI [Source:HGNC Symbol;Acc:HGNC:9039] | 25215 | -0.033 | -0.3213 | No |
| 56 | PLA2G4A | phospholipase A2 group IVA [Source:HGNC Symbol;Acc:HGNC:9035] | 25224 | -0.033 | -0.3191 | No |
| 57 | NPR1 | natriuretic peptide receptor 1 [Source:HGNC Symbol;Acc:HGNC:7943] | 25289 | -0.033 | -0.3182 | No |
| 58 | CACNA1S | calcium voltage-gated channel subunit alpha1 S [Source:HGNC Symbol;Acc:HGNC:1397] | 25589 | -0.035 | -0.3232 | No |
| 59 | ADCY1 | adenylate cyclase 1 [Source:HGNC Symbol;Acc:HGNC:232] | 25830 | -0.037 | -0.3266 | No |
| 60 | PRKACB | protein kinase cAMP-activated catalytic subunit beta [Source:HGNC Symbol;Acc:HGNC:9381] | 26088 | -0.039 | -0.3303 | No |
| 61 | PPP1CB | protein phosphatase 1 catalytic subunit beta [Source:HGNC Symbol;Acc:HGNC:9282] | 26978 | -0.045 | -0.3496 | No |
| 62 | ACTA2 | "actin alpha 2, smooth muscle [Source:HGNC Symbol;Acc:HGNC:130]" | 27037 | -0.046 | -0.3477 | No |
| 63 | PLCB1 | phospholipase C beta 1 [Source:HGNC Symbol;Acc:HGNC:15917] | 27781 | -0.052 | -0.3628 | No |
| 64 | ADCY2 | adenylate cyclase 2 [Source:HGNC Symbol;Acc:HGNC:233] | 27981 | -0.053 | -0.3639 | No |
| 65 | GNA11 | G protein subunit alpha 11 [Source:HGNC Symbol;Acc:HGNC:4379] | 28013 | -0.054 | -0.3607 | No |
| 66 | PRKACG | protein kinase cAMP-activated catalytic subunit gamma [Source:HGNC Symbol;Acc:HGNC:9382] | 28777 | -0.059 | -0.3758 | No |
| 67 | PLA2G2A | phospholipase A2 group IIA [Source:HGNC Symbol;Acc:HGNC:9031] | 29183 | -0.063 | -0.3814 | No |
| 68 | ITPR3 | "inositol 1,4,5-trisphosphate receptor type 3 [Source:HGNC Symbol;Acc:HGNC:6182]" | 29471 | -0.066 | -0.3838 | No |
| 69 | PRKCD | protein kinase C delta [Source:HGNC Symbol;Acc:HGNC:9399] | 29983 | -0.071 | -0.3916 | No |
| 70 | RAMP3 | receptor activity modifying protein 3 [Source:HGNC Symbol;Acc:HGNC:9845] | 30425 | -0.075 | -0.3973 | No |
| 71 | MAP2K1 | mitogen-activated protein kinase kinase 1 [Source:HGNC Symbol;Acc:HGNC:6840] | 32048 | -0.091 | -0.4319 | No |
| 72 | PLCB2 | phospholipase C beta 2 [Source:HGNC Symbol;Acc:HGNC:9055] | 32369 | -0.095 | -0.4330 | No |
| 73 | GUCY1A2 | guanylate cyclase 1 soluble subunit alpha 2 [Source:HGNC Symbol;Acc:HGNC:4684] | 32385 | -0.095 | -0.4263 | No |
| 74 | PLA2G10 | phospholipase A2 group X [Source:HGNC Symbol;Acc:HGNC:9029] | 32963 | -0.103 | -0.4333 | Yes |
| 75 | PRKCH | protein kinase C eta [Source:HGNC Symbol;Acc:HGNC:9403] | 33201 | -0.106 | -0.4315 | Yes |
| 76 | MAPK1 | mitogen-activated protein kinase 1 [Source:HGNC Symbol;Acc:HGNC:6871] | 33350 | -0.108 | -0.4272 | Yes |
| 77 | BRAF | "B-Raf proto-oncogene, serine/threonine kinase [Source:HGNC Symbol;Acc:HGNC:1097]" | 33362 | -0.108 | -0.4194 | Yes |
| 78 | CALML3 | calmodulin like 3 [Source:HGNC Symbol;Acc:HGNC:1452] | 33436 | -0.110 | -0.4132 | Yes |
| 79 | IRAG1 | "inositol 1,4,5-triphosphate receptor associated 1 [Source:HGNC Symbol;Acc:HGNC:7237]" | 33907 | -0.116 | -0.4165 | Yes |
| 80 | ITPR2 | "inositol 1,4,5-trisphosphate receptor type 2 [Source:HGNC Symbol;Acc:HGNC:6181]" | 34058 | -0.119 | -0.4115 | Yes |
| 81 | ADRA1A | adrenoceptor alpha 1A [Source:HGNC Symbol;Acc:HGNC:277] | 34203 | -0.121 | -0.4062 | Yes |
| 82 | CACNA1F | calcium voltage-gated channel subunit alpha1 F [Source:HGNC Symbol;Acc:HGNC:1393] | 34492 | -0.126 | -0.4042 | Yes |
| 83 | PRKCB | protein kinase C beta [Source:HGNC Symbol;Acc:HGNC:9395] | 34603 | -0.128 | -0.3975 | Yes |
| 84 | ADCY7 | adenylate cyclase 7 [Source:HGNC Symbol;Acc:HGNC:238] | 34661 | -0.129 | -0.3894 | Yes |
| 85 | ADCY4 | adenylate cyclase 4 [Source:HGNC Symbol;Acc:HGNC:235] | 34840 | -0.132 | -0.3842 | Yes |
| 86 | PLA2G4B | phospholipase A2 group IVB [Source:HGNC Symbol;Acc:HGNC:9036] | 34964 | -0.135 | -0.3773 | Yes |
| 87 | PRKCA | protein kinase C alpha [Source:HGNC Symbol;Acc:HGNC:9393] | 35223 | -0.140 | -0.3735 | Yes |
| 88 | GUCY1A1 | guanylate cyclase 1 soluble subunit alpha 1 [Source:HGNC Symbol;Acc:HGNC:4685] | 35261 | -0.141 | -0.3639 | Yes |
| 89 | ADCY9 | adenylate cyclase 9 [Source:HGNC Symbol;Acc:HGNC:240] | 35385 | -0.144 | -0.3564 | Yes |
| 90 | JMJD7-PLA2G4B | JMJD7-PLA2G4B readthrough [Source:HGNC Symbol;Acc:HGNC:34449] | 36012 | -0.161 | -0.3604 | Yes |
| 91 | GUCY1B1 | guanylate cyclase 1 soluble subunit beta 1 [Source:HGNC Symbol;Acc:HGNC:4687] | 36017 | -0.161 | -0.3485 | Yes |
| 92 | KCNMB2 | potassium calcium-activated channel subfamily M regulatory beta subunit 2 [Source:HGNC Symbol;Acc:HGNC:6286] | 36026 | -0.161 | -0.3368 | Yes |
| 93 | ARHGEF12 | Rho guanine nucleotide exchange factor 12 [Source:HGNC Symbol;Acc:HGNC:14193] | 36195 | -0.166 | -0.3287 | Yes |
| 94 | PLA2G3 | phospholipase A2 group III [Source:HGNC Symbol;Acc:HGNC:17934] | 36216 | -0.167 | -0.3168 | Yes |
| 95 | ITPR1 | "inositol 1,4,5-trisphosphate receptor type 1 [Source:HGNC Symbol;Acc:HGNC:6180]" | 36283 | -0.169 | -0.3059 | Yes |
| 96 | PLA2G4E | phospholipase A2 group IVE [Source:HGNC Symbol;Acc:HGNC:24791] | 36372 | -0.172 | -0.2954 | Yes |
| 97 | KCNMB3 | potassium calcium-activated channel subfamily M regulatory beta subunit 3 [Source:HGNC Symbol;Acc:HGNC:6287] | 36380 | -0.172 | -0.2827 | Yes |
| 98 | GNAQ | G protein subunit alpha q [Source:HGNC Symbol;Acc:HGNC:4390] | 36400 | -0.173 | -0.2704 | Yes |
| 99 | PRKG1 | protein kinase cGMP-dependent 1 [Source:HGNC Symbol;Acc:HGNC:9414] | 36496 | -0.176 | -0.2597 | Yes |
| 100 | PLA2G2D | phospholipase A2 group IID [Source:HGNC Symbol;Acc:HGNC:9033] | 36979 | -0.196 | -0.2574 | Yes |
| 101 | CALCRL | calcitonin receptor like receptor [Source:HGNC Symbol;Acc:HGNC:16709] | 37035 | -0.199 | -0.2440 | Yes |
| 102 | PPP1R12A | protein phosphatase 1 regulatory subunit 12A [Source:HGNC Symbol;Acc:HGNC:7618] | 37241 | -0.209 | -0.2337 | Yes |
| 103 | KCNMA1 | potassium calcium-activated channel subfamily M alpha 1 [Source:HGNC Symbol;Acc:HGNC:6284] | 37288 | -0.212 | -0.2191 | Yes |
| 104 | AVPR1A | arginine vasopressin receptor 1A [Source:HGNC Symbol;Acc:HGNC:895] | 37496 | -0.223 | -0.2078 | Yes |
| 105 | ARHGEF11 | Rho guanine nucleotide exchange factor 11 [Source:HGNC Symbol;Acc:HGNC:14580] | 37695 | -0.237 | -0.1952 | Yes |
| 106 | ROCK1 | Rho associated coiled-coil containing protein kinase 1 [Source:HGNC Symbol;Acc:HGNC:10251] | 37709 | -0.238 | -0.1778 | Yes |
| 107 | ADCY5 | adenylate cyclase 5 [Source:HGNC Symbol;Acc:HGNC:236] | 37762 | -0.242 | -0.1612 | Yes |
| 108 | NPR2 | natriuretic peptide receptor 2 [Source:HGNC Symbol;Acc:HGNC:7944] | 38193 | -0.292 | -0.1504 | Yes |
| 109 | ADCY8 | adenylate cyclase 8 [Source:HGNC Symbol;Acc:HGNC:239] | 38268 | -0.302 | -0.1298 | Yes |
| 110 | ROCK2 | Rho associated coiled-coil containing protein kinase 2 [Source:HGNC Symbol;Acc:HGNC:10252] | 38278 | -0.304 | -0.1075 | Yes |
| 111 | PLCB4 | phospholipase C beta 4 [Source:HGNC Symbol;Acc:HGNC:9059] | 38308 | -0.309 | -0.0852 | Yes |
| 112 | ADCY3 | adenylate cyclase 3 [Source:HGNC Symbol;Acc:HGNC:234] | 38423 | -0.337 | -0.0631 | Yes |
| 113 | MYH11 | myosin heavy chain 11 [Source:HGNC Symbol;Acc:HGNC:7569] | 38506 | -0.378 | -0.0371 | Yes |
| 114 | GNA13 | G protein subunit alpha 13 [Source:HGNC Symbol;Acc:HGNC:4381] | 38514 | -0.383 | -0.0088 | Yes |
| 115 | PRKX | protein kinase X-linked [Source:HGNC Symbol;Acc:HGNC:9441] | 38524 | -0.395 | 0.0204 | Yes |
Table: GSEA details [plain text format]

  

Fig 2: KEGG\_VASCULAR\_SMOOTH\_MUSCLE\_CONTRACTION      
 Blue-Pink O' Gram in the Space of the Analyzed GeneSet

  

Fig 3: KEGG\_VASCULAR\_SMOOTH\_MUSCLE\_CONTRACTION: Random ES distribution      
 Gene set null distribution of ES for **KEGG\_VASCULAR\_SMOOTH\_MUSCLE\_CONTRACTION**
