# Supplementary material for: Simultaneous Presentation of Multiple Myeloma and Lung Cancer: Case Report and Gene Bioinformatics Analysis
Source: Front Oncol. 2022 Jun 13;12:859735. doi: 10.3389/fonc.2022.859735 (PMC9235397; doi:10.3389/fonc.2022.859735)
Supplement: Supplementary file 1 [file DataSheet_1.zip › The bioinformatic analysis of MM and lung cancer supplementary materials/Enrichment analysis/MECR/GSEA_4.1.0/LUSC TCGA/KEGG.Gsea.1639042374131/KEGG_WNT_SIGNALING_PATHWAY.html]

Details for gene set KEGG\_WNT\_SIGNALING\_PATHWAY[GSEA]

|  || Dataset | ExpData\_collapsed\_to\_symbols.ENSG00000116353\_profile\_in\_ExpData.cls #ENSG00000116353 |
| Phenotype | ENSG00000116353\_profile\_in\_ExpData.cls#ENSG00000116353 |
| Upregulated in class | ENSG00000116353\_neg |
| GeneSet | KEGG\_WNT\_SIGNALING\_PATHWAY |
| Enrichment Score (ES) | -0.3788994 |
| Normalized Enrichment Score (NES) | -1.7327719 |
| Nominal p-value | 0.0 |
| FDR q-value | 0.018290427 |
| FWER p-Value | 0.238 |
Table: GSEA Results Summary

  

Fig 1: Enrichment plot: KEGG\_WNT\_SIGNALING\_PATHWAY      
 Profile of the Running ES Score & Positions of GeneSet Members on the Rank Ordered List

  

| SYMBOL | TITLE | RANK IN GENE LIST | RANK METRIC SCORE | RUNNING ES | CORE ENRICHMENT || 1 | DVL1 | dishevelled segment polarity protein 1 [Source:HGNC Symbol;Acc:HGNC:3084] | 49 | 0.451 | 0.0239 | No |
| 2 | CTNNBIP1 | catenin beta interacting protein 1 [Source:HGNC Symbol;Acc:HGNC:16913] | 122 | 0.380 | 0.0433 | No |
| 3 | CSNK2B | casein kinase 2 beta [Source:HGNC Symbol;Acc:HGNC:2460] | 275 | 0.315 | 0.0570 | No |
| 4 | PPP2R1A | protein phosphatase 2 scaffold subunit Aalpha [Source:HGNC Symbol;Acc:HGNC:9302] | 486 | 0.272 | 0.0669 | No |
| 5 | RBX1 | ring-box 1 [Source:HGNC Symbol;Acc:HGNC:9928] | 689 | 0.246 | 0.0755 | No |
| 6 | WNT10A | Wnt family member 10A [Source:HGNC Symbol;Acc:HGNC:13829] | 839 | 0.228 | 0.0844 | No |
| 7 | DAAM1 | dishevelled associated activator of morphogenesis 1 [Source:HGNC Symbol;Acc:HGNC:18142] | 857 | 0.227 | 0.0966 | No |
| 8 | MAPK8 | mitogen-activated protein kinase 8 [Source:HGNC Symbol;Acc:HGNC:6881] | 948 | 0.218 | 0.1065 | No |
| 9 | WNT4 | Wnt family member 4 [Source:HGNC Symbol;Acc:HGNC:12783] | 1086 | 0.210 | 0.1147 | No |
| 10 | PORCN | porcupine O-acyltransferase [Source:HGNC Symbol;Acc:HGNC:17652] | 1215 | 0.202 | 0.1227 | No |
| 11 | CTBP1 | C-terminal binding protein 1 [Source:HGNC Symbol;Acc:HGNC:2494] | 1610 | 0.182 | 0.1228 | No |
| 12 | CAMK2G | calcium/calmodulin dependent protein kinase II gamma [Source:HGNC Symbol;Acc:HGNC:1463] | 1746 | 0.175 | 0.1292 | No |
| 13 | CTBP2 | C-terminal binding protein 2 [Source:HGNC Symbol;Acc:HGNC:2495] | 1773 | 0.174 | 0.1382 | No |
| 14 | RAC3 | Rac family small GTPase 3 [Source:HGNC Symbol;Acc:HGNC:9803] | 1777 | 0.174 | 0.1479 | No |
| 15 | TCF7L2 | transcription factor 7 like 2 [Source:HGNC Symbol;Acc:HGNC:11641] | 1930 | 0.168 | 0.1534 | No |
| 16 | PLCB3 | phospholipase C beta 3 [Source:HGNC Symbol;Acc:HGNC:9056] | 1971 | 0.167 | 0.1617 | No |
| 17 | CSNK1E | casein kinase 1 epsilon [Source:HGNC Symbol;Acc:HGNC:2453] | 2470 | 0.150 | 0.1573 | No |
| 18 | PPP3CA | protein phosphatase 3 catalytic subunit alpha [Source:HGNC Symbol;Acc:HGNC:9314] | 2508 | 0.149 | 0.1647 | No |
| 19 | RUVBL1 | RuvB like AAA ATPase 1 [Source:HGNC Symbol;Acc:HGNC:10474] | 2608 | 0.147 | 0.1704 | No |
| 20 | SIAH1 | siah E3 ubiquitin protein ligase 1 [Source:HGNC Symbol;Acc:HGNC:10857] | 3607 | 0.124 | 0.1519 | No |
| 21 | PPP2R5C | protein phosphatase 2 regulatory subunit B'gamma [Source:HGNC Symbol;Acc:HGNC:9311] | 3748 | 0.122 | 0.1551 | No |
| 22 | RAC1 | Rac family small GTPase 1 [Source:HGNC Symbol;Acc:HGNC:9801] | 4156 | 0.115 | 0.1511 | No |
| 23 | WNT6 | Wnt family member 6 [Source:HGNC Symbol;Acc:HGNC:12785] | 4672 | 0.107 | 0.1440 | No |
| 24 | RHOA | ras homolog family member A [Source:HGNC Symbol;Acc:HGNC:667] | 4969 | 0.103 | 0.1422 | No |
| 25 | WNT16 | Wnt family member 16 [Source:HGNC Symbol;Acc:HGNC:16267] | 6037 | 0.094 | 0.1202 | No |
| 26 | CCND1 | cyclin D1 [Source:HGNC Symbol;Acc:HGNC:1582] | 6470 | 0.089 | 0.1141 | No |
| 27 | VANGL1 | VANGL planar cell polarity protein 1 [Source:HGNC Symbol;Acc:HGNC:15512] | 6949 | 0.084 | 0.1066 | No |
| 28 | CCND3 | cyclin D3 [Source:HGNC Symbol;Acc:HGNC:1585] | 7527 | 0.079 | 0.0963 | No |
| 29 | PPP3CB | protein phosphatase 3 catalytic subunit beta [Source:HGNC Symbol;Acc:HGNC:9315] | 8034 | 0.075 | 0.0875 | No |
| 30 | CAMK2D | calcium/calmodulin dependent protein kinase II delta [Source:HGNC Symbol;Acc:HGNC:1462] | 8249 | 0.073 | 0.0861 | No |
| 31 | WNT8A | Wnt family member 8A [Source:HGNC Symbol;Acc:HGNC:12788] | 8416 | 0.071 | 0.0859 | No |
| 32 | SKP1 | S-phase kinase associated protein 1 [Source:HGNC Symbol;Acc:HGNC:10899] | 8628 | 0.070 | 0.0844 | No |
| 33 | CXXC4 | CXXC finger protein 4 [Source:HGNC Symbol;Acc:HGNC:24593] | 8840 | 0.068 | 0.0828 | No |
| 34 | CAMK2B | calcium/calmodulin dependent protein kinase II beta [Source:HGNC Symbol;Acc:HGNC:1461] | 10058 | 0.060 | 0.0551 | No |
| 35 | TBL1Y | transducin beta like 1 Y-linked [Source:HGNC Symbol;Acc:HGNC:18502] | 10380 | 0.058 | 0.0501 | No |
| 36 | WNT7A | Wnt family member 7A [Source:HGNC Symbol;Acc:HGNC:12786] | 10774 | 0.055 | 0.0431 | No |
| 37 | FZD5 | frizzled class receptor 5 [Source:HGNC Symbol;Acc:HGNC:4043] | 11161 | 0.053 | 0.0362 | No |
| 38 | CSNK1A1L | casein kinase 1 alpha 1 like [Source:HGNC Symbol;Acc:HGNC:20289] | 11977 | 0.047 | 0.0180 | No |
| 39 | CHP1 | calcineurin like EF-hand protein 1 [Source:HGNC Symbol;Acc:HGNC:17433] | 12465 | 0.044 | 0.0081 | No |
| 40 | TCF7 | transcription factor 7 [Source:HGNC Symbol;Acc:HGNC:11639] | 13002 | 0.041 | -0.0033 | No |
| 41 | MMP7 | matrix metallopeptidase 7 [Source:HGNC Symbol;Acc:HGNC:7174] | 13034 | 0.041 | -0.0019 | No |
| 42 | CAMK2A | calcium/calmodulin dependent protein kinase II alpha [Source:HGNC Symbol;Acc:HGNC:1460] | 13120 | 0.040 | -0.0018 | No |
| 43 | SFRP5 | secreted frizzled related protein 5 [Source:HGNC Symbol;Acc:HGNC:10779] | 13630 | 0.037 | -0.0127 | No |
| 44 | PPP2CA | protein phosphatase 2 catalytic subunit alpha [Source:HGNC Symbol;Acc:HGNC:9299] | 13727 | 0.037 | -0.0131 | No |
| 45 | PPP2R5E | protein phosphatase 2 regulatory subunit B'epsilon [Source:HGNC Symbol;Acc:HGNC:9313] | 14166 | 0.034 | -0.0224 | No |
| 46 | PPP2R5D | protein phosphatase 2 regulatory subunit B'delta [Source:HGNC Symbol;Acc:HGNC:9312] | 14931 | 0.030 | -0.0402 | No |
| 47 | NFATC4 | nuclear factor of activated T cells 4 [Source:HGNC Symbol;Acc:HGNC:7778] | 14991 | 0.029 | -0.0401 | No |
| 48 | PRKCG | protein kinase C gamma [Source:HGNC Symbol;Acc:HGNC:9402] | 15351 | 0.027 | -0.0477 | No |
| 49 | AXIN1 | axin 1 [Source:HGNC Symbol;Acc:HGNC:903] | 15360 | 0.027 | -0.0464 | No |
| 50 | GSK3B | glycogen synthase kinase 3 beta [Source:HGNC Symbol;Acc:HGNC:4617] | 15772 | 0.025 | -0.0555 | No |
| 51 | PPP3R2 | "protein phosphatase 3 regulatory subunit B, beta [Source:HGNC Symbol;Acc:HGNC:9318]" | 15798 | 0.024 | -0.0548 | No |
| 52 | LRP5 | LDL receptor related protein 5 [Source:HGNC Symbol;Acc:HGNC:6697] | 16670 | 0.020 | -0.0760 | No |
| 53 | DKK1 | dickkopf WNT signaling pathway inhibitor 1 [Source:HGNC Symbol;Acc:HGNC:2891] | 16877 | 0.018 | -0.0802 | No |
| 54 | NKD1 | NKD inhibitor of WNT signaling pathway 1 [Source:HGNC Symbol;Acc:HGNC:17045] | 17028 | 0.018 | -0.0830 | No |
| 55 | PPP2R5B | protein phosphatase 2 regulatory subunit B'beta [Source:HGNC Symbol;Acc:HGNC:9310] | 17721 | 0.014 | -0.0999 | No |
| 56 | CSNK2A2 | casein kinase 2 alpha 2 [Source:HGNC Symbol;Acc:HGNC:2459] | 18068 | 0.012 | -0.1081 | No |
| 57 | BTRC | beta-transducin repeat containing E3 ubiquitin protein ligase [Source:HGNC Symbol;Acc:HGNC:1144] | 18315 | 0.011 | -0.1138 | No |
| 58 | APC2 | APC regulator of WNT signaling pathway 2 [Source:HGNC Symbol;Acc:HGNC:24036] | 18523 | 0.009 | -0.1186 | No |
| 59 | JUN | "Jun proto-oncogene, AP-1 transcription factor subunit [Source:HGNC Symbol;Acc:HGNC:6204]" | 18921 | 0.007 | -0.1283 | No |
| 60 | MYC | "MYC proto-oncogene, bHLH transcription factor [Source:HGNC Symbol;Acc:HGNC:7553]" | 19309 | 0.005 | -0.1379 | No |
| 61 | WNT1 | Wnt family member 1 [Source:HGNC Symbol;Acc:HGNC:12774] | 19377 | 0.004 | -0.1394 | No |
| 62 | FZD8 | frizzled class receptor 8 [Source:HGNC Symbol;Acc:HGNC:4046] | 20256 | -0.001 | -0.1618 | No |
| 63 | TCF7L1 | transcription factor 7 like 1 [Source:HGNC Symbol;Acc:HGNC:11640] | 20720 | -0.004 | -0.1734 | No |
| 64 | FZD4 | frizzled class receptor 4 [Source:HGNC Symbol;Acc:HGNC:4042] | 20770 | -0.004 | -0.1744 | No |
| 65 | PRKACA | protein kinase cAMP-activated catalytic subunit alpha [Source:HGNC Symbol;Acc:HGNC:9380] | 21332 | -0.007 | -0.1884 | No |
| 66 | CACYBP | calcyclin binding protein [Source:HGNC Symbol;Acc:HGNC:30423] | 21898 | -0.011 | -0.2022 | No |
| 67 | SKP1P2 | S-phase kinase associated protein 1 pseudogene 2 [Source:HGNC Symbol;Acc:HGNC:10900] | 22230 | -0.013 | -0.2099 | No |
| 68 | PPP3R1 | "protein phosphatase 3 regulatory subunit B, alpha [Source:HGNC Symbol;Acc:HGNC:9317]" | 22525 | -0.015 | -0.2166 | No |
| 69 | PPARD | peroxisome proliferator activated receptor delta [Source:HGNC Symbol;Acc:HGNC:9235] | 22805 | -0.016 | -0.2228 | No |
| 70 | WNT7B | Wnt family member 7B [Source:HGNC Symbol;Acc:HGNC:12787] | 22905 | -0.017 | -0.2244 | No |
| 71 | FRAT2 | FRAT regulator of WNT signaling pathway 2 [Source:HGNC Symbol;Acc:HGNC:16048] | 22999 | -0.017 | -0.2258 | No |
| 72 | PSEN1 | presenilin 1 [Source:HGNC Symbol;Acc:HGNC:9508] | 23228 | -0.019 | -0.2306 | No |
| 73 | CSNK2A1 | casein kinase 2 alpha 1 [Source:HGNC Symbol;Acc:HGNC:2457] | 24580 | -0.028 | -0.2635 | No |
| 74 | PPP2CB | protein phosphatase 2 catalytic subunit beta [Source:HGNC Symbol;Acc:HGNC:9300] | 25064 | -0.032 | -0.2741 | No |
| 75 | DAAM2 | dishevelled associated activator of morphogenesis 2 [Source:HGNC Symbol;Acc:HGNC:18143] | 25115 | -0.032 | -0.2736 | No |
| 76 | NFATC3 | nuclear factor of activated T cells 3 [Source:HGNC Symbol;Acc:HGNC:7777] | 25295 | -0.033 | -0.2763 | No |
| 77 | DKK4 | dickkopf WNT signaling pathway inhibitor 4 [Source:HGNC Symbol;Acc:HGNC:2894] | 25575 | -0.035 | -0.2814 | No |
| 78 | WNT9A | Wnt family member 9A [Source:HGNC Symbol;Acc:HGNC:12778] | 25791 | -0.037 | -0.2849 | No |
| 79 | PRKACB | protein kinase cAMP-activated catalytic subunit beta [Source:HGNC Symbol;Acc:HGNC:9381] | 26088 | -0.039 | -0.2903 | No |
| 80 | FZD1 | frizzled class receptor 1 [Source:HGNC Symbol;Acc:HGNC:4038] | 26540 | -0.042 | -0.2995 | No |
| 81 | WNT10B | Wnt family member 10B [Source:HGNC Symbol;Acc:HGNC:12775] | 26603 | -0.042 | -0.2987 | No |
| 82 | PLCB1 | phospholipase C beta 1 [Source:HGNC Symbol;Acc:HGNC:15917] | 27781 | -0.052 | -0.3258 | No |
| 83 | PRICKLE1 | prickle planar cell polarity protein 1 [Source:HGNC Symbol;Acc:HGNC:17019] | 28145 | -0.054 | -0.3321 | No |
| 84 | FZD9 | frizzled class receptor 9 [Source:HGNC Symbol;Acc:HGNC:4047] | 28404 | -0.057 | -0.3355 | No |
| 85 | MAPK9 | mitogen-activated protein kinase 9 [Source:HGNC Symbol;Acc:HGNC:6886] | 28470 | -0.057 | -0.3340 | No |
| 86 | DKK2 | dickkopf WNT signaling pathway inhibitor 2 [Source:HGNC Symbol;Acc:HGNC:2892] | 28538 | -0.058 | -0.3325 | No |
| 87 | MAP3K7 | mitogen-activated protein kinase kinase kinase 7 [Source:HGNC Symbol;Acc:HGNC:6859] | 28753 | -0.059 | -0.3346 | No |
| 88 | PRKACG | protein kinase cAMP-activated catalytic subunit gamma [Source:HGNC Symbol;Acc:HGNC:9382] | 28777 | -0.059 | -0.3319 | No |
| 89 | WNT3A | Wnt family member 3A [Source:HGNC Symbol;Acc:HGNC:15983] | 29041 | -0.062 | -0.3351 | No |
| 90 | RAC2 | Rac family small GTPase 2 [Source:HGNC Symbol;Acc:HGNC:9802] | 29782 | -0.069 | -0.3502 | No |
| 91 | TP53 | tumor protein p53 [Source:HGNC Symbol;Acc:HGNC:11998] | 30908 | -0.079 | -0.3745 | Yes |
| 92 | PPP3CC | protein phosphatase 3 catalytic subunit gamma [Source:HGNC Symbol;Acc:HGNC:9316] | 30963 | -0.080 | -0.3714 | Yes |
| 93 | SFRP1 | secreted frizzled related protein 1 [Source:HGNC Symbol;Acc:HGNC:10776] | 31114 | -0.081 | -0.3707 | Yes |
| 94 | FZD3 | frizzled class receptor 3 [Source:HGNC Symbol;Acc:HGNC:4041] | 31252 | -0.083 | -0.3695 | Yes |
| 95 | SFRP2 | secreted frizzled related protein 2 [Source:HGNC Symbol;Acc:HGNC:10777] | 31255 | -0.083 | -0.3650 | Yes |
| 96 | FRAT1 | FRAT regulator of WNT signaling pathway 1 [Source:HGNC Symbol;Acc:HGNC:3944] | 31265 | -0.083 | -0.3606 | Yes |
| 97 | DVL2 | dishevelled segment polarity protein 2 [Source:HGNC Symbol;Acc:HGNC:3086] | 31825 | -0.089 | -0.3699 | Yes |
| 98 | CHD8 | chromodomain helicase DNA binding protein 8 [Source:HGNC Symbol;Acc:HGNC:20153] | 32161 | -0.093 | -0.3733 | Yes |
| 99 | WNT5B | Wnt family member 5B [Source:HGNC Symbol;Acc:HGNC:16265] | 32250 | -0.094 | -0.3703 | Yes |
| 100 | PLCB2 | phospholipase C beta 2 [Source:HGNC Symbol;Acc:HGNC:9055] | 32369 | -0.095 | -0.3680 | Yes |
| 101 | NFAT5 | nuclear factor of activated T cells 5 [Source:HGNC Symbol;Acc:HGNC:7774] | 32402 | -0.095 | -0.3635 | Yes |
| 102 | PRICKLE2 | prickle planar cell polarity protein 2 [Source:HGNC Symbol;Acc:HGNC:20340] | 32455 | -0.096 | -0.3594 | Yes |
| 103 | WNT2 | Wnt family member 2 [Source:HGNC Symbol;Acc:HGNC:12780] | 32471 | -0.096 | -0.3544 | Yes |
| 104 | SFRP4 | secreted frizzled related protein 4 [Source:HGNC Symbol;Acc:HGNC:10778] | 32591 | -0.098 | -0.3520 | Yes |
| 105 | SOX17 | SRY-box transcription factor 17 [Source:HGNC Symbol;Acc:HGNC:18122] | 32617 | -0.098 | -0.3472 | Yes |
| 106 | NFATC2 | nuclear factor of activated T cells 2 [Source:HGNC Symbol;Acc:HGNC:7776] | 32713 | -0.100 | -0.3440 | Yes |
| 107 | SMAD4 | SMAD family member 4 [Source:HGNC Symbol;Acc:HGNC:6770] | 32731 | -0.100 | -0.3389 | Yes |
| 108 | AXIN2 | axin 2 [Source:HGNC Symbol;Acc:HGNC:904] | 32772 | -0.100 | -0.3343 | Yes |
| 109 | CSNK1A1 | casein kinase 1 alpha 1 [Source:HGNC Symbol;Acc:HGNC:2451] | 33103 | -0.105 | -0.3369 | Yes |
| 110 | WNT8B | Wnt family member 8B [Source:HGNC Symbol;Acc:HGNC:12789] | 33128 | -0.105 | -0.3316 | Yes |
| 111 | FOSL1 | "FOS like 1, AP-1 transcription factor subunit [Source:HGNC Symbol;Acc:HGNC:13718]" | 33429 | -0.109 | -0.3332 | Yes |
| 112 | CER1 | "cerberus 1, DAN family BMP antagonist [Source:HGNC Symbol;Acc:HGNC:1862]" | 33628 | -0.112 | -0.3320 | Yes |
| 113 | CHP2 | calcineurin like EF-hand protein 2 [Source:HGNC Symbol;Acc:HGNC:24927] | 34531 | -0.126 | -0.3479 | Yes |
| 114 | PRKCB | protein kinase C beta [Source:HGNC Symbol;Acc:HGNC:9395] | 34603 | -0.128 | -0.3426 | Yes |
| 115 | FZD2 | frizzled class receptor 2 [Source:HGNC Symbol;Acc:HGNC:4040] | 34628 | -0.128 | -0.3361 | Yes |
| 116 | SMAD2 | SMAD family member 2 [Source:HGNC Symbol;Acc:HGNC:6768] | 34897 | -0.133 | -0.3355 | Yes |
| 117 | FZD6 | frizzled class receptor 6 [Source:HGNC Symbol;Acc:HGNC:4044] | 35028 | -0.136 | -0.3312 | Yes |
| 118 | PPP2R1B | protein phosphatase 2 scaffold subunit Abeta [Source:HGNC Symbol;Acc:HGNC:9303] | 35047 | -0.137 | -0.3240 | Yes |
| 119 | PRKCA | protein kinase C alpha [Source:HGNC Symbol;Acc:HGNC:9393] | 35223 | -0.140 | -0.3207 | Yes |
| 120 | NKD2 | NKD inhibitor of WNT signaling pathway 2 [Source:HGNC Symbol;Acc:HGNC:17046] | 35309 | -0.142 | -0.3149 | Yes |
| 121 | SMAD3 | SMAD family member 3 [Source:HGNC Symbol;Acc:HGNC:6769] | 35374 | -0.144 | -0.3085 | Yes |
| 122 | TBL1XR1 | TBL1X receptor 1 [Source:HGNC Symbol;Acc:HGNC:29529] | 35427 | -0.145 | -0.3018 | Yes |
| 123 | FBXW11 | F-box and WD repeat domain containing 11 [Source:HGNC Symbol;Acc:HGNC:13607] | 35478 | -0.146 | -0.2949 | Yes |
| 124 | NFATC1 | nuclear factor of activated T cells 1 [Source:HGNC Symbol;Acc:HGNC:7775] | 35544 | -0.148 | -0.2883 | Yes |
| 125 | CUL1 | cullin 1 [Source:HGNC Symbol;Acc:HGNC:2551] | 35662 | -0.151 | -0.2828 | Yes |
| 126 | CTNNB1 | catenin beta 1 [Source:HGNC Symbol;Acc:HGNC:2514] | 35880 | -0.157 | -0.2796 | Yes |
| 127 | WNT9B | Wnt family member 9B [Source:HGNC Symbol;Acc:HGNC:12779] | 35896 | -0.157 | -0.2712 | Yes |
| 128 | WNT2B | Wnt family member 2B [Source:HGNC Symbol;Acc:HGNC:12781] | 36087 | -0.163 | -0.2670 | Yes |
| 129 | MAPK10 | mitogen-activated protein kinase 10 [Source:HGNC Symbol;Acc:HGNC:6872] | 36201 | -0.166 | -0.2606 | Yes |
| 130 | EP300 | E1A binding protein p300 [Source:HGNC Symbol;Acc:HGNC:3373] | 36409 | -0.173 | -0.2562 | Yes |
| 131 | WIF1 | WNT inhibitory factor 1 [Source:HGNC Symbol;Acc:HGNC:18081] | 37278 | -0.211 | -0.2665 | Yes |
| 132 | CREBBP | CREB binding protein [Source:HGNC Symbol;Acc:HGNC:2348] | 37476 | -0.222 | -0.2592 | Yes |
| 133 | SENP2 | SUMO specific peptidase 2 [Source:HGNC Symbol;Acc:HGNC:23116] | 37549 | -0.227 | -0.2483 | Yes |
| 134 | VANGL2 | VANGL planar cell polarity protein 2 [Source:HGNC Symbol;Acc:HGNC:15511] | 37574 | -0.229 | -0.2362 | Yes |
| 135 | WNT11 | Wnt family member 11 [Source:HGNC Symbol;Acc:HGNC:12776] | 37666 | -0.236 | -0.2253 | Yes |
| 136 | ROCK1 | Rho associated coiled-coil containing protein kinase 1 [Source:HGNC Symbol;Acc:HGNC:10251] | 37709 | -0.238 | -0.2131 | Yes |
| 137 | CCND2 | cyclin D2 [Source:HGNC Symbol;Acc:HGNC:1583] | 37725 | -0.240 | -0.2001 | Yes |
| 138 | LRP6 | LDL receptor related protein 6 [Source:HGNC Symbol;Acc:HGNC:6698] | 37785 | -0.244 | -0.1880 | Yes |
| 139 | PPP2R5A | protein phosphatase 2 regulatory subunit B'alpha [Source:HGNC Symbol;Acc:HGNC:9309] | 37849 | -0.249 | -0.1758 | Yes |
| 140 | TBL1X | transducin beta like 1 X-linked [Source:HGNC Symbol;Acc:HGNC:11585] | 37897 | -0.254 | -0.1628 | Yes |
| 141 | DVL3 | dishevelled segment polarity protein 3 [Source:HGNC Symbol;Acc:HGNC:3087] | 37983 | -0.264 | -0.1502 | Yes |
| 142 | WNT3 | Wnt family member 3 [Source:HGNC Symbol;Acc:HGNC:12782] | 38054 | -0.271 | -0.1369 | Yes |
| 143 | ROCK2 | Rho associated coiled-coil containing protein kinase 2 [Source:HGNC Symbol;Acc:HGNC:10252] | 38278 | -0.304 | -0.1256 | Yes |
| 144 | PLCB4 | phospholipase C beta 4 [Source:HGNC Symbol;Acc:HGNC:9059] | 38308 | -0.309 | -0.1091 | Yes |
| 145 | NLK | nemo like kinase [Source:HGNC Symbol;Acc:HGNC:29858] | 38317 | -0.310 | -0.0919 | Yes |
| 146 | LEF1 | lymphoid enhancer binding factor 1 [Source:HGNC Symbol;Acc:HGNC:6551] | 38341 | -0.316 | -0.0749 | Yes |
| 147 | FZD10 | frizzled class receptor 10 [Source:HGNC Symbol;Acc:HGNC:4039] | 38368 | -0.322 | -0.0576 | Yes |
| 148 | APC | APC regulator of WNT signaling pathway [Source:HGNC Symbol;Acc:HGNC:583] | 38413 | -0.336 | -0.0400 | Yes |
| 149 | FZD7 | frizzled class receptor 7 [Source:HGNC Symbol;Acc:HGNC:4045] | 38485 | -0.367 | -0.0213 | Yes |
| 150 | WNT5A | Wnt family member 5A [Source:HGNC Symbol;Acc:HGNC:12784] | 38488 | -0.368 | -0.0008 | Yes |
| 151 | PRKX | protein kinase X-linked [Source:HGNC Symbol;Acc:HGNC:9441] | 38524 | -0.395 | 0.0204 | Yes |
Table: GSEA details [plain text format]

  

Fig 2: KEGG\_WNT\_SIGNALING\_PATHWAY      
 Blue-Pink O' Gram in the Space of the Analyzed GeneSet

  

Fig 3: KEGG\_WNT\_SIGNALING\_PATHWAY: Random ES distribution      
 Gene set null distribution of ES for **KEGG\_WNT\_SIGNALING\_PATHWAY**
