# Supplementary material for: Simultaneous Presentation of Multiple Myeloma and Lung Cancer: Case Report and Gene Bioinformatics Analysis
Source: Front Oncol. 2022 Jun 13;12:859735. doi: 10.3389/fonc.2022.859735 (PMC9235397; doi:10.3389/fonc.2022.859735)
Supplement: Supplementary file 1 [file DataSheet_1.zip › The bioinformatic analysis of MM and lung cancer supplementary materials/Enrichment analysis/MECR/GSEA_4.1.0/LUSC TCGA/KEGG.Gsea.1639042374131/gsea_report_for_ENSG00000116353_neg_1639042374131.html]

Report for ENSG00000116353\_neg 1639042374131 [GSEA]

| GS  follow link to MSigDB | GS DETAILS | SIZE | ES | NES | NOM p-val | FDR q-val | FWER p-val | RANK AT MAX | LEADING EDGE || 1 | KEGG\_HEMATOPOIETIC\_CELL\_LINEAGE | Details ... | 85 | -0.51 | -2.10 | 0.000 | 0.002 | 0.002 | 9819 | tags=59%, list=25%, signal=78% |
| 2 | KEGG\_PHOSPHATIDYLINOSITOL\_SIGNALING\_SYSTEM | Details ... | 76 | -0.51 | -2.09 | 0.000 | 0.001 | 0.002 | 6230 | tags=43%, list=16%, signal=51% |
| 3 | KEGG\_CALCIUM\_SIGNALING\_PATHWAY | Details ... | 178 | -0.44 | -2.06 | 0.000 | 0.001 | 0.003 | 7416 | tags=35%, list=19%, signal=43% |
| 4 | KEGG\_CELL\_ADHESION\_MOLECULES\_CAMS | Details ... | 131 | -0.45 | -2.01 | 0.000 | 0.003 | 0.009 | 9128 | tags=53%, list=23%, signal=69% |
| 5 | KEGG\_BASAL\_CELL\_CARCINOMA | Details ... | 55 | -0.52 | -1.98 | 0.000 | 0.003 | 0.012 | 4697 | tags=35%, list=12%, signal=39% |
| 6 | KEGG\_ABC\_TRANSPORTERS | Details ... | 44 | -0.54 | -1.98 | 0.000 | 0.003 | 0.014 | 4131 | tags=36%, list=11%, signal=41% |
| 7 | KEGG\_HEDGEHOG\_SIGNALING\_PATHWAY | Details ... | 56 | -0.50 | -1.94 | 0.000 | 0.004 | 0.022 | 7075 | tags=38%, list=18%, signal=46% |
| 8 | KEGG\_VASCULAR\_SMOOTH\_MUSCLE\_CONTRACTION | Details ... | 115 | -0.44 | -1.93 | 0.000 | 0.004 | 0.023 | 6362 | tags=37%, list=16%, signal=43% |
| 9 | KEGG\_PRIMARY\_IMMUNODEFICIENCY | Details ... | 35 | -0.54 | -1.91 | 0.002 | 0.005 | 0.034 | 10261 | tags=69%, list=26%, signal=93% |
| 10 | KEGG\_NEUROACTIVE\_LIGAND\_RECEPTOR\_INTERACTION | Details ... | 270 | -0.38 | -1.88 | 0.000 | 0.006 | 0.048 | 9921 | tags=38%, list=25%, signal=51% |
| 11 | KEGG\_MAPK\_SIGNALING\_PATHWAY | Details ... | 267 | -0.38 | -1.86 | 0.000 | 0.007 | 0.064 | 7381 | tags=34%, list=19%, signal=42% |
| 12 | KEGG\_PATHWAYS\_IN\_CANCER | Details ... | 325 | -0.36 | -1.84 | 0.000 | 0.009 | 0.085 | 7327 | tags=33%, list=19%, signal=40% |
| 13 | KEGG\_INOSITOL\_PHOSPHATE\_METABOLISM | Details ... | 54 | -0.48 | -1.83 | 0.002 | 0.009 | 0.089 | 7336 | tags=41%, list=19%, signal=50% |
| 14 | KEGG\_GAP\_JUNCTION | Details ... | 90 | -0.43 | -1.83 | 0.000 | 0.008 | 0.089 | 7277 | tags=39%, list=19%, signal=48% |
| 15 | KEGG\_MELANOGENESIS | Details ... | 101 | -0.43 | -1.82 | 0.002 | 0.009 | 0.100 | 4722 | tags=31%, list=12%, signal=35% |
| 16 | KEGG\_METABOLISM\_OF\_XENOBIOTICS\_BY\_CYTOCHROME\_P450 | Details ... | 69 | -0.45 | -1.81 | 0.000 | 0.008 | 0.101 | 4068 | tags=32%, list=10%, signal=36% |
| 17 | KEGG\_LONG\_TERM\_DEPRESSION | Details ... | 70 | -0.43 | -1.73 | 0.000 | 0.019 | 0.236 | 7277 | tags=44%, list=19%, signal=54% |
| 18 | KEGG\_WNT\_SIGNALING\_PATHWAY | Details ... | 151 | -0.38 | -1.73 | 0.000 | 0.018 | 0.238 | 8417 | tags=40%, list=21%, signal=51% |
| 19 | KEGG\_DILATED\_CARDIOMYOPATHY | Details ... | 90 | -0.41 | -1.72 | 0.000 | 0.019 | 0.259 | 11570 | tags=53%, list=29%, signal=75% |
| 20 | KEGG\_TGF\_BETA\_SIGNALING\_PATHWAY | Details ... | 86 | -0.41 | -1.71 | 0.002 | 0.022 | 0.305 | 9431 | tags=47%, list=24%, signal=61% |
| 21 | KEGG\_T\_CELL\_RECEPTOR\_SIGNALING\_PATHWAY |  | 108 | -0.39 | -1.69 | 0.002 | 0.024 | 0.341 | 9654 | tags=47%, list=25%, signal=62% |
| 22 | KEGG\_ARRHYTHMOGENIC\_RIGHT\_VENTRICULAR\_CARDIOMYOPATHY\_ARVC |  | 74 | -0.42 | -1.69 | 0.002 | 0.024 | 0.355 | 10289 | tags=47%, list=26%, signal=64% |
| 23 | KEGG\_INTESTINAL\_IMMUNE\_NETWORK\_FOR\_IGA\_PRODUCTION |  | 46 | -0.46 | -1.68 | 0.002 | 0.024 | 0.364 | 13125 | tags=72%, list=33%, signal=108% |
| 24 | KEGG\_GRAFT\_VERSUS\_HOST\_DISEASE |  | 37 | -0.49 | -1.68 | 0.004 | 0.024 | 0.384 | 12775 | tags=70%, list=32%, signal=104% |
| 25 | KEGG\_AXON\_GUIDANCE |  | 129 | -0.37 | -1.64 | 0.002 | 0.032 | 0.477 | 6923 | tags=36%, list=18%, signal=44% |
| 26 | KEGG\_ECM\_RECEPTOR\_INTERACTION |  | 84 | -0.39 | -1.63 | 0.000 | 0.035 | 0.516 | 10376 | tags=52%, list=26%, signal=71% |
| 27 | KEGG\_GNRH\_SIGNALING\_PATHWAY |  | 101 | -0.38 | -1.62 | 0.000 | 0.038 | 0.566 | 7277 | tags=36%, list=19%, signal=44% |
| 28 | KEGG\_CHEMOKINE\_SIGNALING\_PATHWAY |  | 188 | -0.34 | -1.61 | 0.002 | 0.040 | 0.595 | 8187 | tags=40%, list=21%, signal=50% |
| 29 | KEGG\_PROGESTERONE\_MEDIATED\_OOCYTE\_MATURATION |  | 85 | -0.39 | -1.58 | 0.004 | 0.047 | 0.671 | 6284 | tags=32%, list=16%, signal=38% |
| 30 | KEGG\_ERBB\_SIGNALING\_PATHWAY |  | 87 | -0.37 | -1.57 | 0.004 | 0.049 | 0.710 | 6192 | tags=33%, list=16%, signal=39% |
| 31 | KEGG\_REGULATION\_OF\_ACTIN\_CYTOSKELETON |  | 213 | -0.33 | -1.57 | 0.000 | 0.050 | 0.724 | 7277 | tags=34%, list=19%, signal=42% |
| 32 | KEGG\_MELANOMA |  | 71 | -0.38 | -1.55 | 0.006 | 0.056 | 0.777 | 10683 | tags=48%, list=27%, signal=66% |
| 33 | KEGG\_COLORECTAL\_CANCER |  | 62 | -0.39 | -1.55 | 0.014 | 0.055 | 0.789 | 6594 | tags=35%, list=17%, signal=43% |
| 34 | KEGG\_ADIPOCYTOKINE\_SIGNALING\_PATHWAY |  | 67 | -0.39 | -1.54 | 0.014 | 0.057 | 0.818 | 5344 | tags=30%, list=14%, signal=34% |
| 35 | KEGG\_B\_CELL\_RECEPTOR\_SIGNALING\_PATHWAY |  | 75 | -0.38 | -1.53 | 0.014 | 0.060 | 0.834 | 10040 | tags=51%, list=26%, signal=68% |
| 36 | KEGG\_NEUROTROPHIN\_SIGNALING\_PATHWAY |  | 126 | -0.34 | -1.53 | 0.002 | 0.058 | 0.834 | 6192 | tags=29%, list=16%, signal=35% |
| 37 | KEGG\_VIRAL\_MYOCARDITIS |  | 68 | -0.38 | -1.52 | 0.021 | 0.059 | 0.842 | 12775 | tags=62%, list=32%, signal=91% |
| 38 | KEGG\_HYPERTROPHIC\_CARDIOMYOPATHY\_HCM |  | 83 | -0.37 | -1.52 | 0.015 | 0.058 | 0.842 | 10719 | tags=48%, list=27%, signal=66% |
| 39 | KEGG\_JAK\_STAT\_SIGNALING\_PATHWAY |  | 155 | -0.33 | -1.52 | 0.002 | 0.058 | 0.852 | 7370 | tags=28%, list=19%, signal=35% |
| 40 | KEGG\_ASCORBATE\_AND\_ALDARATE\_METABOLISM |  | 25 | -0.47 | -1.49 | 0.045 | 0.070 | 0.910 | 4068 | tags=36%, list=10%, signal=40% |
| 41 | KEGG\_ADHERENS\_JUNCTION |  | 73 | -0.37 | -1.49 | 0.025 | 0.068 | 0.910 | 7624 | tags=40%, list=19%, signal=49% |
| 42 | KEGG\_DRUG\_METABOLISM\_CYTOCHROME\_P450 |  | 71 | -0.36 | -1.45 | 0.031 | 0.091 | 0.959 | 4068 | tags=24%, list=10%, signal=27% |
| 43 | KEGG\_STARCH\_AND\_SUCROSE\_METABOLISM |  | 52 | -0.39 | -1.45 | 0.044 | 0.091 | 0.961 | 4927 | tags=27%, list=13%, signal=31% |
| 44 | KEGG\_ALDOSTERONE\_REGULATED\_SODIUM\_REABSORPTION |  | 42 | -0.40 | -1.45 | 0.047 | 0.090 | 0.961 | 6433 | tags=38%, list=16%, signal=45% |
| 45 | KEGG\_GLUTATHIONE\_METABOLISM |  | 49 | -0.39 | -1.44 | 0.045 | 0.089 | 0.964 | 3465 | tags=31%, list=9%, signal=34% |
| 46 | KEGG\_DORSO\_VENTRAL\_AXIS\_FORMATION |  | 24 | -0.45 | -1.43 | 0.065 | 0.093 | 0.967 | 7551 | tags=46%, list=19%, signal=57% |
| 47 | KEGG\_ARACHIDONIC\_ACID\_METABOLISM |  | 58 | -0.37 | -1.43 | 0.031 | 0.092 | 0.969 | 4361 | tags=26%, list=11%, signal=29% |
| 48 | KEGG\_TOLL\_LIKE\_RECEPTOR\_SIGNALING\_PATHWAY |  | 102 | -0.33 | -1.43 | 0.029 | 0.091 | 0.972 | 9287 | tags=35%, list=24%, signal=46% |
| 49 | KEGG\_ALLOGRAFT\_REJECTION |  | 35 | -0.40 | -1.43 | 0.078 | 0.092 | 0.974 | 12775 | tags=66%, list=32%, signal=97% |
| 50 | KEGG\_RENAL\_CELL\_CARCINOMA |  | 70 | -0.35 | -1.42 | 0.036 | 0.096 | 0.983 | 7277 | tags=39%, list=19%, signal=47% |
| 51 | KEGG\_FC\_GAMMA\_R\_MEDIATED\_PHAGOCYTOSIS |  | 96 | -0.33 | -1.41 | 0.024 | 0.098 | 0.986 | 9543 | tags=52%, list=24%, signal=69% |
| 52 | KEGG\_PENTOSE\_AND\_GLUCURONATE\_INTERCONVERSIONS |  | 28 | -0.42 | -1.41 | 0.079 | 0.096 | 0.986 | 4068 | tags=32%, list=10%, signal=36% |
| 53 | KEGG\_ASTHMA |  | 28 | -0.44 | -1.41 | 0.074 | 0.097 | 0.987 | 12775 | tags=71%, list=32%, signal=106% |
| 54 | KEGG\_FOCAL\_ADHESION |  | 199 | -0.30 | -1.40 | 0.008 | 0.097 | 0.988 | 10376 | tags=43%, list=26%, signal=58% |
| 55 | KEGG\_SPHINGOLIPID\_METABOLISM |  | 39 | -0.40 | -1.40 | 0.054 | 0.096 | 0.988 | 3325 | tags=26%, list=8%, signal=28% |
| 56 | KEGG\_CYTOKINE\_CYTOKINE\_RECEPTOR\_INTERACTION |  | 264 | -0.28 | -1.39 | 0.010 | 0.100 | 0.990 | 10261 | tags=38%, list=26%, signal=51% |
| 57 | KEGG\_ENDOCYTOSIS |  | 181 | -0.30 | -1.39 | 0.017 | 0.099 | 0.990 | 6488 | tags=31%, list=16%, signal=37% |
| 58 | KEGG\_GLYCEROPHOSPHOLIPID\_METABOLISM |  | 77 | -0.34 | -1.37 | 0.053 | 0.113 | 0.994 | 5502 | tags=31%, list=14%, signal=36% |
| 59 | KEGG\_MATURITY\_ONSET\_DIABETES\_OF\_THE\_YOUNG |  | 25 | -0.43 | -1.36 | 0.081 | 0.119 | 0.996 | 8508 | tags=32%, list=22%, signal=41% |
| 60 | KEGG\_LONG\_TERM\_POTENTIATION |  | 70 | -0.34 | -1.36 | 0.057 | 0.121 | 0.997 | 7277 | tags=33%, list=19%, signal=40% |
| 61 | KEGG\_SMALL\_CELL\_LUNG\_CANCER |  | 84 | -0.32 | -1.35 | 0.068 | 0.123 | 0.997 | 10065 | tags=39%, list=26%, signal=53% |
| 62 | KEGG\_INSULIN\_SIGNALING\_PATHWAY |  | 137 | -0.30 | -1.34 | 0.026 | 0.129 | 0.998 | 7352 | tags=33%, list=19%, signal=40% |
| 63 | KEGG\_TYPE\_II\_DIABETES\_MELLITUS |  | 47 | -0.36 | -1.34 | 0.096 | 0.132 | 0.999 | 6192 | tags=36%, list=16%, signal=43% |
| 64 | KEGG\_RETINOL\_METABOLISM |  | 64 | -0.33 | -1.33 | 0.059 | 0.134 | 0.999 | 5557 | tags=28%, list=14%, signal=33% |
| 65 | KEGG\_LINOLEIC\_ACID\_METABOLISM |  | 29 | -0.40 | -1.32 | 0.107 | 0.142 | 0.999 | 4361 | tags=31%, list=11%, signal=35% |
| 66 | KEGG\_ENDOMETRIAL\_CANCER |  | 52 | -0.35 | -1.32 | 0.090 | 0.143 | 0.999 | 7277 | tags=33%, list=19%, signal=40% |
| 67 | KEGG\_PANCREATIC\_CANCER |  | 70 | -0.33 | -1.31 | 0.086 | 0.148 | 1.000 | 7277 | tags=33%, list=19%, signal=40% |
| 68 | KEGG\_PROSTATE\_CANCER |  | 89 | -0.31 | -1.31 | 0.085 | 0.148 | 1.000 | 9323 | tags=36%, list=24%, signal=47% |
| 69 | KEGG\_FC\_EPSILON\_RI\_SIGNALING\_PATHWAY |  | 79 | -0.31 | -1.29 | 0.087 | 0.162 | 1.000 | 10142 | tags=46%, list=26%, signal=61% |
| 70 | KEGG\_PRION\_DISEASES |  | 35 | -0.37 | -1.28 | 0.147 | 0.166 | 1.000 | 9628 | tags=43%, list=24%, signal=57% |
| 71 | KEGG\_O\_GLYCAN\_BIOSYNTHESIS |  | 30 | -0.38 | -1.28 | 0.132 | 0.166 | 1.000 | 6305 | tags=33%, list=16%, signal=40% |
| 72 | KEGG\_GLYCEROLIPID\_METABOLISM |  | 49 | -0.34 | -1.28 | 0.116 | 0.166 | 1.000 | 6995 | tags=37%, list=18%, signal=45% |
| 73 | KEGG\_STEROID\_HORMONE\_BIOSYNTHESIS |  | 55 | -0.33 | -1.25 | 0.160 | 0.187 | 1.000 | 4216 | tags=22%, list=11%, signal=24% |
| 74 | KEGG\_OOCYTE\_MEIOSIS |  | 113 | -0.28 | -1.25 | 0.090 | 0.191 | 1.000 | 6230 | tags=26%, list=16%, signal=30% |
| 75 | KEGG\_GLYCOSAMINOGLYCAN\_BIOSYNTHESIS\_KERATAN\_SULFATE |  | 15 | -0.42 | -1.20 | 0.237 | 0.245 | 1.000 | 1648 | tags=27%, list=4%, signal=28% |
| 76 | KEGG\_CELL\_CYCLE |  | 125 | -0.27 | -1.20 | 0.131 | 0.243 | 1.000 | 4753 | tags=20%, list=12%, signal=23% |
| 77 | KEGG\_EPITHELIAL\_CELL\_SIGNALING\_IN\_HELICOBACTER\_PYLORI\_INFECTION |  | 68 | -0.30 | -1.20 | 0.164 | 0.248 | 1.000 | 4167 | tags=22%, list=11%, signal=25% |
| 78 | KEGG\_CHRONIC\_MYELOID\_LEUKEMIA |  | 73 | -0.29 | -1.19 | 0.166 | 0.249 | 1.000 | 7277 | tags=30%, list=19%, signal=37% |
| 79 | KEGG\_GLIOMA |  | 65 | -0.30 | -1.16 | 0.220 | 0.291 | 1.000 | 7277 | tags=32%, list=19%, signal=40% |
| 80 | KEGG\_TYPE\_I\_DIABETES\_MELLITUS |  | 41 | -0.32 | -1.16 | 0.234 | 0.290 | 1.000 | 11665 | tags=54%, list=30%, signal=76% |
| 81 | KEGG\_NON\_SMALL\_CELL\_LUNG\_CANCER |  | 54 | -0.30 | -1.15 | 0.230 | 0.291 | 1.000 | 7277 | tags=33%, list=19%, signal=41% |
| 82 | KEGG\_NATURAL\_KILLER\_CELL\_MEDIATED\_CYTOTOXICITY |  | 132 | -0.25 | -1.15 | 0.184 | 0.289 | 1.000 | 9543 | tags=36%, list=24%, signal=48% |
| 83 | KEGG\_TASTE\_TRANSDUCTION |  | 51 | -0.30 | -1.14 | 0.224 | 0.300 | 1.000 | 6027 | tags=20%, list=15%, signal=23% |
| 84 | KEGG\_VASOPRESSIN\_REGULATED\_WATER\_REABSORPTION |  | 44 | -0.31 | -1.14 | 0.243 | 0.304 | 1.000 | 7678 | tags=25%, list=20%, signal=31% |
| 85 | KEGG\_PORPHYRIN\_AND\_CHLOROPHYLL\_METABOLISM |  | 41 | -0.32 | -1.13 | 0.274 | 0.310 | 1.000 | 4675 | tags=27%, list=12%, signal=30% |
| 86 | KEGG\_LEISHMANIA\_INFECTION |  | 70 | -0.28 | -1.10 | 0.294 | 0.356 | 1.000 | 12775 | tags=54%, list=32%, signal=80% |
| 87 | KEGG\_ACUTE\_MYELOID\_LEUKEMIA |  | 57 | -0.28 | -1.09 | 0.286 | 0.364 | 1.000 | 9261 | tags=40%, list=24%, signal=53% |
| 88 | KEGG\_AUTOIMMUNE\_THYROID\_DISEASE |  | 50 | -0.29 | -1.09 | 0.294 | 0.370 | 1.000 | 12775 | tags=50%, list=32%, signal=74% |
| 89 | KEGG\_GLYCOSPHINGOLIPID\_BIOSYNTHESIS\_LACTO\_AND\_NEOLACTO\_SERIES |  | 26 | -0.34 | -1.08 | 0.335 | 0.377 | 1.000 | 4577 | tags=31%, list=12%, signal=35% |
| 90 | KEGG\_GLYCOSPHINGOLIPID\_BIOSYNTHESIS\_GANGLIO\_SERIES |  | 15 | -0.38 | -1.05 | 0.390 | 0.428 | 1.000 | 1648 | tags=13%, list=4%, signal=14% |
| 91 | KEGG\_NOD\_LIKE\_RECEPTOR\_SIGNALING\_PATHWAY |  | 62 | -0.27 | -1.04 | 0.405 | 0.447 | 1.000 | 7339 | tags=24%, list=19%, signal=30% |
| 92 | KEGG\_GLYCOSAMINOGLYCAN\_BIOSYNTHESIS\_CHONDROITIN\_SULFATE |  | 22 | -0.34 | -1.02 | 0.422 | 0.483 | 1.000 | 7391 | tags=36%, list=19%, signal=45% |
| 93 | KEGG\_HOMOLOGOUS\_RECOMBINATION |  | 28 | -0.31 | -1.01 | 0.443 | 0.502 | 1.000 | 3262 | tags=25%, list=8%, signal=27% |
| 94 | KEGG\_PENTOSE\_PHOSPHATE\_PATHWAY |  | 27 | -0.30 | -0.99 | 0.470 | 0.539 | 1.000 | 3665 | tags=22%, list=9%, signal=24% |
| 95 | KEGG\_ALPHA\_LINOLENIC\_ACID\_METABOLISM |  | 19 | -0.32 | -0.97 | 0.487 | 0.569 | 1.000 | 4361 | tags=32%, list=11%, signal=36% |
| 96 | KEGG\_THYROID\_CANCER |  | 29 | -0.30 | -0.96 | 0.519 | 0.579 | 1.000 | 8417 | tags=38%, list=21%, signal=48% |
| 97 | KEGG\_BIOSYNTHESIS\_OF\_UNSATURATED\_FATTY\_ACIDS |  | 22 | -0.31 | -0.96 | 0.499 | 0.573 | 1.000 | 6102 | tags=36%, list=16%, signal=43% |
| 98 | KEGG\_STEROID\_BIOSYNTHESIS |  | 17 | -0.33 | -0.96 | 0.509 | 0.572 | 1.000 | 11753 | tags=53%, list=30%, signal=75% |
| 99 | KEGG\_VIBRIO\_CHOLERAE\_INFECTION |  | 54 | -0.25 | -0.95 | 0.536 | 0.589 | 1.000 | 5473 | tags=20%, list=14%, signal=24% |
| 100 | KEGG\_PYRUVATE\_METABOLISM |  | 40 | -0.26 | -0.93 | 0.590 | 0.626 | 1.000 | 6669 | tags=23%, list=17%, signal=27% |
| 101 | KEGG\_GLYCOSAMINOGLYCAN\_DEGRADATION |  | 21 | -0.30 | -0.92 | 0.584 | 0.648 | 1.000 | 6839 | tags=29%, list=17%, signal=35% |
| 102 | KEGG\_RENIN\_ANGIOTENSIN\_SYSTEM |  | 17 | -0.31 | -0.89 | 0.610 | 0.699 | 1.000 | 10901 | tags=53%, list=28%, signal=73% |
| 103 | KEGG\_ANTIGEN\_PROCESSING\_AND\_PRESENTATION |  | 81 | -0.21 | -0.86 | 0.758 | 0.757 | 1.000 | 13162 | tags=49%, list=33%, signal=74% |
| 104 | KEGG\_BLADDER\_CANCER |  | 42 | -0.22 | -0.82 | 0.751 | 0.817 | 1.000 | 7277 | tags=26%, list=19%, signal=32% |
| 105 | KEGG\_RIBOFLAVIN\_METABOLISM |  | 16 | -0.29 | -0.81 | 0.712 | 0.820 | 1.000 | 5827 | tags=25%, list=15%, signal=29% |
| 106 | KEGG\_PROXIMAL\_TUBULE\_BICARBONATE\_RECLAMATION |  | 23 | -0.23 | -0.73 | 0.863 | 0.925 | 1.000 | 9467 | tags=30%, list=24%, signal=40% |
| 107 | KEGG\_COMPLEMENT\_AND\_COAGULATION\_CASCADES |  | 69 | -0.18 | -0.70 | 0.952 | 0.938 | 1.000 | 8666 | tags=25%, list=22%, signal=32% |
Table: Gene sets enriched in phenotype **negative correlation with profile **[plain text format]****
