# Supplementary material for: Simultaneous Presentation of Multiple Myeloma and Lung Cancer: Case Report and Gene Bioinformatics Analysis
Source: Front Oncol. 2022 Jun 13;12:859735. doi: 10.3389/fonc.2022.859735 (PMC9235397; doi:10.3389/fonc.2022.859735)
Supplement: Supplementary file 1 [file DataSheet_1.zip › The bioinformatic analysis of MM and lung cancer supplementary materials/Enrichment analysis/MECR/GSEA_4.1.0/LUSC TCGA/KEGG.Gsea.1639042374131/gsea_report_for_ENSG00000116353_pos_1639042374131.html]

Report for ENSG00000116353\_pos 1639042374131 [GSEA]

| GS  follow link to MSigDB | GS DETAILS | SIZE | ES | NES | NOM p-val | FDR q-val | FWER p-val | RANK AT MAX | LEADING EDGE || 1 | KEGG\_RIBOSOME | Details ... | 88 | 0.76 | 3.15 | 0.000 | 0.000 | 0.000 | 4364 | tags=68%, list=11%, signal=77% |
| 2 | KEGG\_OXIDATIVE\_PHOSPHORYLATION | Details ... | 132 | 0.65 | 2.89 | 0.000 | 0.000 | 0.000 | 6753 | tags=62%, list=17%, signal=75% |
| 3 | KEGG\_PARKINSONS\_DISEASE | Details ... | 130 | 0.65 | 2.88 | 0.000 | 0.000 | 0.000 | 7734 | tags=62%, list=20%, signal=76% |
| 4 | KEGG\_ALZHEIMERS\_DISEASE | Details ... | 166 | 0.53 | 2.43 | 0.000 | 0.000 | 0.000 | 7734 | tags=54%, list=20%, signal=66% |
| 5 | KEGG\_HUNTINGTONS\_DISEASE | Details ... | 182 | 0.51 | 2.38 | 0.000 | 0.000 | 0.000 | 6106 | tags=46%, list=16%, signal=54% |
| 6 | KEGG\_RNA\_POLYMERASE | Details ... | 29 | 0.70 | 2.35 | 0.000 | 0.000 | 0.000 | 3719 | tags=48%, list=9%, signal=53% |
| 7 | KEGG\_PEROXISOME | Details ... | 78 | 0.56 | 2.27 | 0.000 | 0.000 | 0.000 | 3063 | tags=35%, list=8%, signal=37% |
| 8 | KEGG\_PYRIMIDINE\_METABOLISM | Details ... | 98 | 0.52 | 2.19 | 0.000 | 0.000 | 0.002 | 3719 | tags=35%, list=9%, signal=38% |
| 9 | KEGG\_SPLICEOSOME | Details ... | 127 | 0.46 | 2.02 | 0.000 | 0.001 | 0.012 | 5066 | tags=35%, list=13%, signal=41% |
| 10 | KEGG\_AMINOACYL\_TRNA\_BIOSYNTHESIS | Details ... | 41 | 0.57 | 1.99 | 0.000 | 0.001 | 0.019 | 4685 | tags=41%, list=12%, signal=47% |
| 11 | KEGG\_VALINE\_LEUCINE\_AND\_ISOLEUCINE\_DEGRADATION | Details ... | 44 | 0.55 | 1.98 | 0.000 | 0.002 | 0.022 | 4951 | tags=43%, list=13%, signal=49% |
| 12 | KEGG\_GLYOXYLATE\_AND\_DICARBOXYLATE\_METABOLISM | Details ... | 16 | 0.68 | 1.94 | 0.000 | 0.002 | 0.034 | 6651 | tags=56%, list=17%, signal=68% |
| 13 | KEGG\_BASE\_EXCISION\_REPAIR | Details ... | 35 | 0.56 | 1.93 | 0.000 | 0.002 | 0.035 | 3657 | tags=40%, list=9%, signal=44% |
| 14 | KEGG\_SELENOAMINO\_ACID\_METABOLISM | Details ... | 26 | 0.57 | 1.84 | 0.006 | 0.006 | 0.104 | 6966 | tags=42%, list=18%, signal=51% |
| 15 | KEGG\_SYSTEMIC\_LUPUS\_ERYTHEMATOSUS | Details ... | 135 | 0.41 | 1.82 | 0.000 | 0.006 | 0.116 | 7690 | tags=39%, list=20%, signal=48% |
| 16 | KEGG\_AMINO\_SUGAR\_AND\_NUCLEOTIDE\_SUGAR\_METABOLISM | Details ... | 44 | 0.49 | 1.77 | 0.002 | 0.011 | 0.209 | 3443 | tags=34%, list=9%, signal=37% |
| 17 | KEGG\_PURINE\_METABOLISM | Details ... | 159 | 0.37 | 1.72 | 0.000 | 0.017 | 0.313 | 4784 | tags=30%, list=12%, signal=34% |
| 18 | KEGG\_TERPENOID\_BACKBONE\_BIOSYNTHESIS | Details ... | 15 | 0.61 | 1.66 | 0.013 | 0.026 | 0.467 | 6578 | tags=53%, list=17%, signal=64% |
| 19 | KEGG\_FATTY\_ACID\_METABOLISM | Details ... | 42 | 0.46 | 1.66 | 0.016 | 0.027 | 0.486 | 3803 | tags=31%, list=10%, signal=34% |
| 20 | KEGG\_PROTEASOME | Details ... | 46 | 0.45 | 1.64 | 0.010 | 0.028 | 0.524 | 8507 | tags=43%, list=22%, signal=55% |
| 21 | KEGG\_ALANINE\_ASPARTATE\_AND\_GLUTAMATE\_METABOLISM |  | 32 | 0.48 | 1.64 | 0.020 | 0.028 | 0.532 | 8803 | tags=47%, list=22%, signal=60% |
| 22 | KEGG\_PANTOTHENATE\_AND\_COA\_BIOSYNTHESIS |  | 16 | 0.57 | 1.63 | 0.013 | 0.028 | 0.554 | 3114 | tags=31%, list=8%, signal=34% |
| 23 | KEGG\_BUTANOATE\_METABOLISM |  | 34 | 0.47 | 1.59 | 0.024 | 0.039 | 0.694 | 4688 | tags=35%, list=12%, signal=40% |
| 24 | KEGG\_CITRATE\_CYCLE\_TCA\_CYCLE |  | 31 | 0.48 | 1.59 | 0.021 | 0.038 | 0.702 | 5087 | tags=35%, list=13%, signal=41% |
| 25 | KEGG\_N\_GLYCAN\_BIOSYNTHESIS |  | 46 | 0.42 | 1.58 | 0.015 | 0.038 | 0.716 | 3734 | tags=26%, list=9%, signal=29% |
| 26 | KEGG\_FRUCTOSE\_AND\_MANNOSE\_METABOLISM |  | 34 | 0.46 | 1.57 | 0.016 | 0.040 | 0.747 | 3355 | tags=35%, list=9%, signal=39% |
| 27 | KEGG\_RNA\_DEGRADATION |  | 59 | 0.40 | 1.57 | 0.014 | 0.039 | 0.747 | 5015 | tags=25%, list=13%, signal=29% |
| 28 | KEGG\_NUCLEOTIDE\_EXCISION\_REPAIR |  | 44 | 0.42 | 1.50 | 0.024 | 0.066 | 0.911 | 3573 | tags=32%, list=9%, signal=35% |
| 29 | KEGG\_ARGININE\_AND\_PROLINE\_METABOLISM |  | 54 | 0.39 | 1.49 | 0.028 | 0.066 | 0.923 | 4147 | tags=26%, list=11%, signal=29% |
| 30 | KEGG\_BETA\_ALANINE\_METABOLISM |  | 22 | 0.46 | 1.42 | 0.076 | 0.103 | 0.985 | 4951 | tags=36%, list=13%, signal=42% |
| 31 | KEGG\_SNARE\_INTERACTIONS\_IN\_VESICULAR\_TRANSPORT |  | 38 | 0.40 | 1.42 | 0.052 | 0.103 | 0.989 | 6705 | tags=47%, list=17%, signal=57% |
| 32 | KEGG\_CARDIAC\_MUSCLE\_CONTRACTION |  | 79 | 0.34 | 1.40 | 0.029 | 0.111 | 0.993 | 6345 | tags=38%, list=16%, signal=45% |
| 33 | KEGG\_CYSTEINE\_AND\_METHIONINE\_METABOLISM |  | 34 | 0.41 | 1.38 | 0.069 | 0.122 | 0.998 | 4422 | tags=26%, list=11%, signal=30% |
| 34 | KEGG\_GALACTOSE\_METABOLISM |  | 26 | 0.44 | 1.38 | 0.078 | 0.119 | 0.998 | 4746 | tags=27%, list=12%, signal=31% |
| 35 | KEGG\_PRIMARY\_BILE\_ACID\_BIOSYNTHESIS |  | 16 | 0.48 | 1.36 | 0.125 | 0.134 | 0.998 | 2709 | tags=31%, list=7%, signal=34% |
| 36 | KEGG\_LYSOSOME |  | 121 | 0.30 | 1.30 | 0.059 | 0.186 | 1.000 | 4712 | tags=24%, list=12%, signal=27% |
| 37 | KEGG\_PROTEIN\_EXPORT |  | 24 | 0.41 | 1.29 | 0.165 | 0.194 | 1.000 | 10679 | tags=54%, list=27%, signal=74% |
| 38 | KEGG\_VEGF\_SIGNALING\_PATHWAY |  | 76 | 0.32 | 1.29 | 0.088 | 0.196 | 1.000 | 5065 | tags=26%, list=13%, signal=30% |
| 39 | KEGG\_DNA\_REPLICATION |  | 36 | 0.37 | 1.28 | 0.141 | 0.197 | 1.000 | 4592 | tags=25%, list=12%, signal=28% |
| 40 | KEGG\_GLYCOSYLPHOSPHATIDYLINOSITOL\_GPI\_ANCHOR\_BIOSYNTHESIS |  | 25 | 0.40 | 1.27 | 0.136 | 0.201 | 1.000 | 1966 | tags=28%, list=5%, signal=29% |
| 41 | KEGG\_AMYOTROPHIC\_LATERAL\_SCLEROSIS\_ALS |  | 53 | 0.33 | 1.26 | 0.124 | 0.216 | 1.000 | 7374 | tags=32%, list=19%, signal=39% |
| 42 | KEGG\_GLYCOSAMINOGLYCAN\_BIOSYNTHESIS\_HEPARAN\_SULFATE |  | 26 | 0.39 | 1.26 | 0.161 | 0.212 | 1.000 | 1613 | tags=23%, list=4%, signal=24% |
| 43 | KEGG\_BASAL\_TRANSCRIPTION\_FACTORS |  | 35 | 0.36 | 1.24 | 0.169 | 0.227 | 1.000 | 3084 | tags=20%, list=8%, signal=22% |
| 44 | KEGG\_TYROSINE\_METABOLISM |  | 42 | 0.35 | 1.24 | 0.143 | 0.229 | 1.000 | 6966 | tags=38%, list=18%, signal=46% |
| 45 | KEGG\_TIGHT\_JUNCTION |  | 132 | 0.28 | 1.23 | 0.093 | 0.235 | 1.000 | 4969 | tags=26%, list=13%, signal=29% |
| 46 | KEGG\_MISMATCH\_REPAIR |  | 23 | 0.40 | 1.23 | 0.192 | 0.232 | 1.000 | 4592 | tags=30%, list=12%, signal=34% |
| 47 | KEGG\_UBIQUITIN\_MEDIATED\_PROTEOLYSIS |  | 135 | 0.27 | 1.22 | 0.098 | 0.236 | 1.000 | 4950 | tags=25%, list=13%, signal=29% |
| 48 | KEGG\_GLYCOLYSIS\_GLUCONEOGENESIS |  | 62 | 0.31 | 1.22 | 0.152 | 0.233 | 1.000 | 5472 | tags=26%, list=14%, signal=30% |
| 49 | KEGG\_PATHOGENIC\_ESCHERICHIA\_COLI\_INFECTION |  | 56 | 0.31 | 1.18 | 0.205 | 0.283 | 1.000 | 4983 | tags=27%, list=13%, signal=31% |
| 50 | KEGG\_TRYPTOPHAN\_METABOLISM |  | 40 | 0.33 | 1.17 | 0.214 | 0.291 | 1.000 | 7797 | tags=28%, list=20%, signal=34% |
| 51 | KEGG\_LEUKOCYTE\_TRANSENDOTHELIAL\_MIGRATION |  | 116 | 0.27 | 1.16 | 0.188 | 0.300 | 1.000 | 5936 | tags=25%, list=15%, signal=29% |
| 52 | KEGG\_NOTCH\_SIGNALING\_PATHWAY |  | 47 | 0.30 | 1.12 | 0.274 | 0.352 | 1.000 | 2539 | tags=19%, list=6%, signal=20% |
| 53 | KEGG\_LYSINE\_DEGRADATION |  | 44 | 0.31 | 1.12 | 0.268 | 0.347 | 1.000 | 4370 | tags=27%, list=11%, signal=31% |
| 54 | KEGG\_MTOR\_SIGNALING\_PATHWAY |  | 52 | 0.30 | 1.12 | 0.270 | 0.343 | 1.000 | 4593 | tags=27%, list=12%, signal=30% |
| 55 | KEGG\_ONE\_CARBON\_POOL\_BY\_FOLATE |  | 17 | 0.37 | 1.04 | 0.430 | 0.486 | 1.000 | 7032 | tags=47%, list=18%, signal=57% |
| 56 | KEGG\_REGULATION\_OF\_AUTOPHAGY |  | 35 | 0.29 | 1.03 | 0.386 | 0.495 | 1.000 | 4805 | tags=26%, list=12%, signal=29% |
| 57 | KEGG\_CYTOSOLIC\_DNA\_SENSING\_PATHWAY |  | 55 | 0.27 | 1.02 | 0.439 | 0.526 | 1.000 | 10995 | tags=42%, list=28%, signal=58% |
| 58 | KEGG\_APOPTOSIS |  | 87 | 0.25 | 1.02 | 0.425 | 0.519 | 1.000 | 3889 | tags=17%, list=10%, signal=19% |
| 59 | KEGG\_OLFACTORY\_TRANSDUCTION |  | 386 | 0.20 | 1.01 | 0.446 | 0.515 | 1.000 | 12217 | tags=37%, list=31%, signal=53% |
| 60 | KEGG\_PPAR\_SIGNALING\_PATHWAY |  | 69 | 0.26 | 1.01 | 0.439 | 0.508 | 1.000 | 8269 | tags=32%, list=21%, signal=40% |
| 61 | KEGG\_P53\_SIGNALING\_PATHWAY |  | 68 | 0.25 | 1.00 | 0.459 | 0.530 | 1.000 | 9897 | tags=37%, list=25%, signal=49% |
| 62 | KEGG\_DRUG\_METABOLISM\_OTHER\_ENZYMES |  | 51 | 0.27 | 0.99 | 0.476 | 0.546 | 1.000 | 5453 | tags=25%, list=14%, signal=30% |
| 63 | KEGG\_PROPANOATE\_METABOLISM |  | 33 | 0.29 | 0.99 | 0.446 | 0.539 | 1.000 | 4951 | tags=24%, list=13%, signal=28% |
| 64 | KEGG\_ETHER\_LIPID\_METABOLISM |  | 33 | 0.29 | 0.98 | 0.441 | 0.535 | 1.000 | 4747 | tags=24%, list=12%, signal=28% |
| 65 | KEGG\_NICOTINATE\_AND\_NICOTINAMIDE\_METABOLISM |  | 24 | 0.30 | 0.94 | 0.571 | 0.625 | 1.000 | 4125 | tags=21%, list=10%, signal=23% |
| 66 | KEGG\_PHENYLALANINE\_METABOLISM |  | 18 | 0.31 | 0.91 | 0.603 | 0.682 | 1.000 | 4477 | tags=33%, list=11%, signal=38% |
| 67 | KEGG\_RIG\_I\_LIKE\_RECEPTOR\_SIGNALING\_PATHWAY |  | 71 | 0.22 | 0.90 | 0.641 | 0.691 | 1.000 | 5531 | tags=21%, list=14%, signal=25% |
| 68 | KEGG\_GLYCINE\_SERINE\_AND\_THREONINE\_METABOLISM |  | 31 | 0.26 | 0.86 | 0.678 | 0.758 | 1.000 | 6822 | tags=26%, list=17%, signal=31% |
| 69 | KEGG\_HISTIDINE\_METABOLISM |  | 29 | 0.26 | 0.85 | 0.695 | 0.759 | 1.000 | 6966 | tags=28%, list=18%, signal=34% |
| 70 | KEGG\_OTHER\_GLYCAN\_DEGRADATION |  | 16 | 0.30 | 0.84 | 0.708 | 0.780 | 1.000 | 2835 | tags=19%, list=7%, signal=20% |
| 71 | KEGG\_NITROGEN\_METABOLISM |  | 23 | 0.27 | 0.82 | 0.732 | 0.792 | 1.000 | 3722 | tags=17%, list=9%, signal=19% |
Table: Gene sets enriched in phenotype **positive correlation with profile **[plain text format]****
