# Supplementary material for: Simultaneous Presentation of Multiple Myeloma and Lung Cancer: Case Report and Gene Bioinformatics Analysis
Source: Front Oncol. 2022 Jun 13;12:859735. doi: 10.3389/fonc.2022.859735 (PMC9235397; doi:10.3389/fonc.2022.859735)
Supplement: Supplementary file 1 [file DataSheet_1.zip › The bioinformatic analysis of MM and lung cancer supplementary materials/Enrichment analysis/MECR/GSEA_4.1.0/LUSC TCGA/LUSC-KEGG_pos_bubble.pdf]

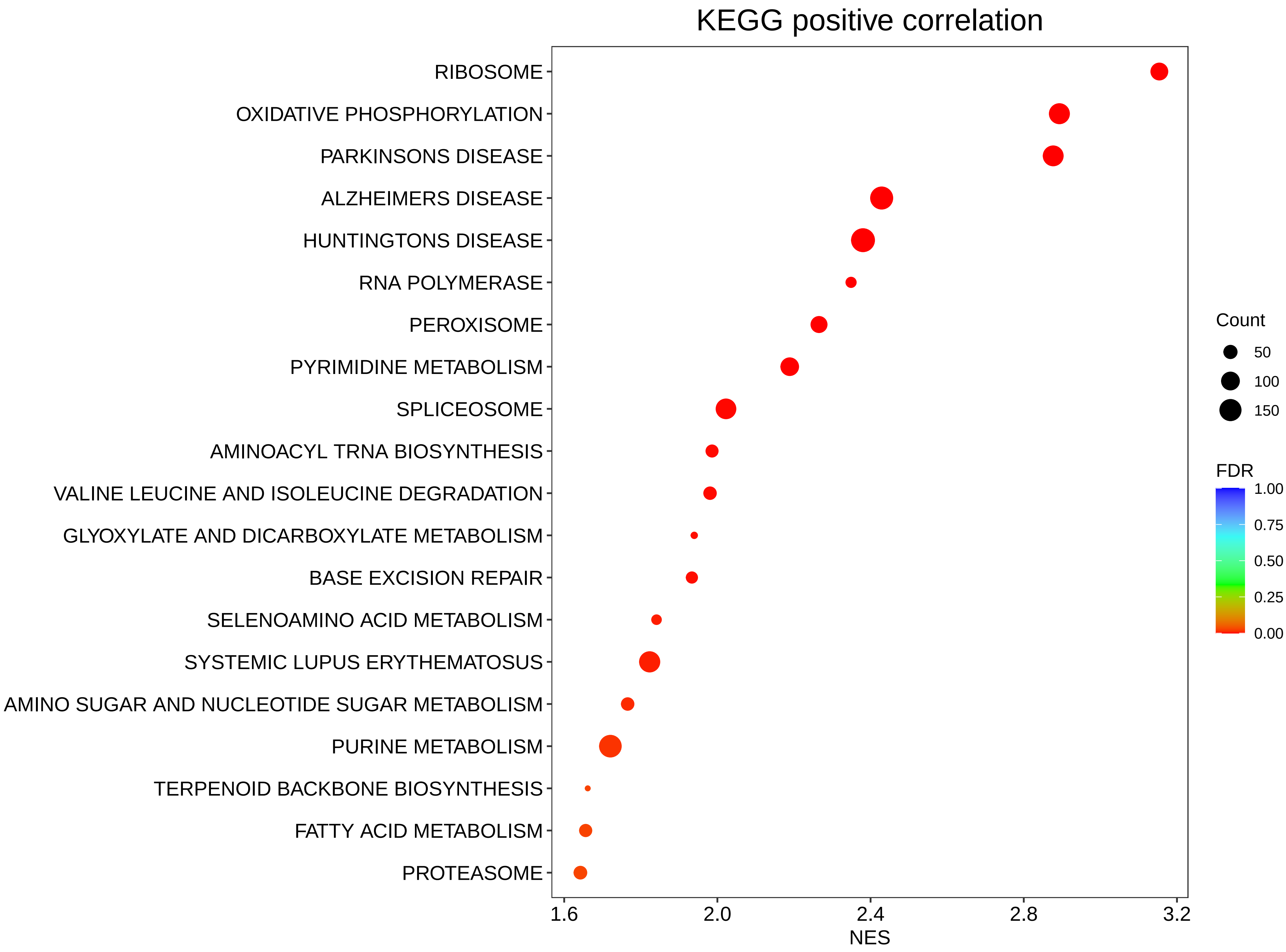
KEGG positive correlation
RIBOSOME
OXIDATIVE PHOSPHORYLATION
PARKINSONS DISEASE
ALZHEIMERS DISEASE
HUNTINGTONS DISEASE
RNA POLYMERASE
PEROXISOME
PYRIMIDINE METABOLISM
SPLICEOSOME
AMINOACYL TRNA BIOSYNTHESIS
VALINE LEUCINE AND ISOLEUCINE DEGRADATION
GLYOXYLATE AND DICARBOXYLATE METABOLISM
BASE EXCISION REPAIR
SELENOAMINO ACID METABOLISM
SYSTEMIC LUPUS ERYTHEMATOSUS
AMINO SUGAR AND NUCLEOTIDE SUGAR METABOLISM
PURINE METABOLISM
TERPENOID BACKBONE BIOSYNTHESIS
FATTY ACID METABOLISM
PROTEASOME
1.6
2.0
2.4
2.8
3.2
NES
Count
50
100
150
FDR
1.00
0.75
0.50
0.25
0.00
