# Supplementary material for: Simultaneous Presentation of Multiple Myeloma and Lung Cancer: Case Report and Gene Bioinformatics Analysis
Source: Front Oncol. 2022 Jun 13;12:859735. doi: 10.3389/fonc.2022.859735 (PMC9235397; doi:10.3389/fonc.2022.859735)
Supplement: Supplementary file 1 [file DataSheet_1.zip › The bioinformatic analysis of MM and lung cancer supplementary materials/Enrichment analysis/MECR/GSEA_4.1.0/MM TCGA/KEGG.Gsea.1639040591792/KEGG_ACUTE_MYELOID_LEUKEMIA.html]

Details for gene set KEGG\_ACUTE\_MYELOID\_LEUKEMIA[GSEA]

|  || Dataset | ExpData\_collapsed\_to\_symbols.ENSG00000116353\_profile\_in\_ExpData.cls #ENSG00000116353 |
| Phenotype | ENSG00000116353\_profile\_in\_ExpData.cls#ENSG00000116353 |
| Upregulated in class | ENSG00000116353\_neg |
| GeneSet | KEGG\_ACUTE\_MYELOID\_LEUKEMIA |
| Enrichment Score (ES) | -0.6288145 |
| Normalized Enrichment Score (NES) | -2.3563108 |
| Nominal p-value | 0.0 |
| FDR q-value | 0.0 |
| FWER p-Value | 0.0 |
Table: GSEA Results Summary

  

Fig 1: Enrichment plot: KEGG\_ACUTE\_MYELOID\_LEUKEMIA      
 Profile of the Running ES Score & Positions of GeneSet Members on the Rank Ordered List

  

| SYMBOL | TITLE | RANK IN GENE LIST | RANK METRIC SCORE | RUNNING ES | CORE ENRICHMENT || 1 | MTOR | mechanistic target of rapamycin kinase [Source:HGNC Symbol;Acc:HGNC:3942] | 1153 | 0.329 | -0.0010 | No |
| 2 | PIK3R2 | phosphoinositide-3-kinase regulatory subunit 2 [Source:HGNC Symbol;Acc:HGNC:8980] | 1248 | 0.320 | 0.0241 | No |
| 3 | PIK3R3 | phosphoinositide-3-kinase regulatory subunit 3 [Source:HGNC Symbol;Acc:HGNC:8981] | 2602 | 0.231 | 0.0095 | No |
| 4 | MYC | "MYC proto-oncogene, bHLH transcription factor [Source:HGNC Symbol;Acc:HGNC:7553]" | 4752 | 0.155 | -0.0319 | No |
| 5 | MAP2K2 | mitogen-activated protein kinase kinase 2 [Source:HGNC Symbol;Acc:HGNC:6842] | 5104 | 0.146 | -0.0283 | No |
| 6 | EIF4EBP1 | eukaryotic translation initiation factor 4E binding protein 1 [Source:HGNC Symbol;Acc:HGNC:3288] | 5294 | 0.142 | -0.0209 | No |
| 7 | JUP | junction plakoglobin [Source:HGNC Symbol;Acc:HGNC:6207] | 6458 | 0.118 | -0.0403 | No |
| 8 | PIK3CB | "phosphatidylinositol-4,5-bisphosphate 3-kinase catalytic subunit beta [Source:HGNC Symbol;Acc:HGNC:8976]" | 7716 | 0.099 | -0.0639 | No |
| 9 | RUNX1T1 | RUNX1 partner transcriptional co-repressor 1 [Source:HGNC Symbol;Acc:HGNC:1535] | 8432 | 0.089 | -0.0744 | No |
| 10 | PIM2 | "Pim-2 proto-oncogene, serine/threonine kinase [Source:HGNC Symbol;Acc:HGNC:8987]" | 8693 | 0.086 | -0.0736 | No |
| 11 | CHUK | component of inhibitor of nuclear factor kappa B kinase complex [Source:HGNC Symbol;Acc:HGNC:1974] | 9925 | 0.071 | -0.0988 | No |
| 12 | RUNX1 | RUNX family transcription factor 1 [Source:HGNC Symbol;Acc:HGNC:10471] | 14916 | 0.032 | -0.2231 | No |
| 13 | SOS1 | SOS Ras/Rac guanine nucleotide exchange factor 1 [Source:HGNC Symbol;Acc:HGNC:11187] | 18532 | 0.012 | -0.3142 | No |
| 14 | IKBKB | inhibitor of nuclear factor kappa B kinase subunit beta [Source:HGNC Symbol;Acc:HGNC:5960] | 20718 | -0.000 | -0.3698 | No |
| 15 | MAPK1 | mitogen-activated protein kinase 1 [Source:HGNC Symbol;Acc:HGNC:6871] | 23167 | -0.015 | -0.4309 | No |
| 16 | HRAS | "HRas proto-oncogene, GTPase [Source:HGNC Symbol;Acc:HGNC:5173]" | 26672 | -0.039 | -0.5168 | No |
| 17 | ARAF | "A-Raf proto-oncogene, serine/threonine kinase [Source:HGNC Symbol;Acc:HGNC:646]" | 26742 | -0.039 | -0.5151 | No |
| 18 | KIT | "KIT proto-oncogene, receptor tyrosine kinase [Source:HGNC Symbol;Acc:HGNC:6342]" | 27060 | -0.042 | -0.5196 | No |
| 19 | RPS6KB2 | ribosomal protein S6 kinase B2 [Source:HGNC Symbol;Acc:HGNC:10437] | 27292 | -0.044 | -0.5218 | No |
| 20 | PML | PML nuclear body scaffold [Source:HGNC Symbol;Acc:HGNC:9113] | 29952 | -0.071 | -0.5833 | No |
| 21 | CCNA1 | cyclin A1 [Source:HGNC Symbol;Acc:HGNC:1577] | 30089 | -0.073 | -0.5805 | No |
| 22 | LEF1 | lymphoid enhancer binding factor 1 [Source:HGNC Symbol;Acc:HGNC:6551] | 31159 | -0.087 | -0.6003 | No |
| 23 | AKT1 | AKT serine/threonine kinase 1 [Source:HGNC Symbol;Acc:HGNC:391] | 31906 | -0.098 | -0.6108 | No |
| 24 | IKBKG | inhibitor of nuclear factor kappa B kinase regulatory subunit gamma [Source:HGNC Symbol;Acc:HGNC:5961] | 32205 | -0.103 | -0.6095 | No |
| 25 | STAT5B | signal transducer and activator of transcription 5B [Source:HGNC Symbol;Acc:HGNC:11367] | 32360 | -0.106 | -0.6043 | No |
| 26 | AKT2 | AKT serine/threonine kinase 2 [Source:HGNC Symbol;Acc:HGNC:392] | 33213 | -0.123 | -0.6154 | No |
| 27 | NRAS | "NRAS proto-oncogene, GTPase [Source:HGNC Symbol;Acc:HGNC:7989]" | 33739 | -0.135 | -0.6172 | Yes |
| 28 | PIK3CD | "phosphatidylinositol-4,5-bisphosphate 3-kinase catalytic subunit delta [Source:HGNC Symbol;Acc:HGNC:8977]" | 33794 | -0.137 | -0.6069 | Yes |
| 29 | AKT3 | AKT serine/threonine kinase 3 [Source:HGNC Symbol;Acc:HGNC:393] | 34501 | -0.156 | -0.6115 | Yes |
| 30 | STAT3 | signal transducer and activator of transcription 3 [Source:HGNC Symbol;Acc:HGNC:11364] | 34682 | -0.161 | -0.6022 | Yes |
| 31 | CEBPA | CCAAT enhancer binding protein alpha [Source:HGNC Symbol;Acc:HGNC:1833] | 34993 | -0.170 | -0.5954 | Yes |
| 32 | MAPK3 | mitogen-activated protein kinase 3 [Source:HGNC Symbol;Acc:HGNC:6877] | 35579 | -0.191 | -0.5939 | Yes |
| 33 | CCND1 | cyclin D1 [Source:HGNC Symbol;Acc:HGNC:1582] | 35880 | -0.203 | -0.5842 | Yes |
| 34 | MAP2K1 | mitogen-activated protein kinase kinase 1 [Source:HGNC Symbol;Acc:HGNC:6840] | 36024 | -0.208 | -0.5699 | Yes |
| 35 | PIM1 | "Pim-1 proto-oncogene, serine/threonine kinase [Source:HGNC Symbol;Acc:HGNC:8986]" | 36038 | -0.209 | -0.5523 | Yes |
| 36 | PIK3CG | "phosphatidylinositol-4,5-bisphosphate 3-kinase catalytic subunit gamma [Source:HGNC Symbol;Acc:HGNC:8978]" | 36243 | -0.217 | -0.5388 | Yes |
| 37 | FLT3 | fms related receptor tyrosine kinase 3 [Source:HGNC Symbol;Acc:HGNC:3765] | 36256 | -0.218 | -0.5203 | Yes |
| 38 | GRB2 | growth factor receptor bound protein 2 [Source:HGNC Symbol;Acc:HGNC:4566] | 36523 | -0.231 | -0.5072 | Yes |
| 39 | TCF7L1 | transcription factor 7 like 1 [Source:HGNC Symbol;Acc:HGNC:11640] | 36849 | -0.251 | -0.4939 | Yes |
| 40 | PIK3R1 | phosphoinositide-3-kinase regulatory subunit 1 [Source:HGNC Symbol;Acc:HGNC:8979] | 37172 | -0.270 | -0.4789 | Yes |
| 41 | TCF7L2 | transcription factor 7 like 2 [Source:HGNC Symbol;Acc:HGNC:11641] | 37205 | -0.273 | -0.4562 | Yes |
| 42 | ZBTB16 | zinc finger and BTB domain containing 16 [Source:HGNC Symbol;Acc:HGNC:12930] | 37455 | -0.293 | -0.4374 | Yes |
| 43 | PIK3R5 | phosphoinositide-3-kinase regulatory subunit 5 [Source:HGNC Symbol;Acc:HGNC:30035] | 37628 | -0.307 | -0.4154 | Yes |
| 44 | BAD | BCL2 associated agonist of cell death [Source:HGNC Symbol;Acc:HGNC:936] | 37742 | -0.318 | -0.3909 | Yes |
| 45 | STAT5A | signal transducer and activator of transcription 5A [Source:HGNC Symbol;Acc:HGNC:11366] | 37773 | -0.321 | -0.3641 | Yes |
| 46 | KRAS | "KRAS proto-oncogene, GTPase [Source:HGNC Symbol;Acc:HGNC:6407]" | 37789 | -0.323 | -0.3368 | Yes |
| 47 | RAF1 | "Raf-1 proto-oncogene, serine/threonine kinase [Source:HGNC Symbol;Acc:HGNC:9829]" | 37807 | -0.325 | -0.3093 | Yes |
| 48 | BRAF | "B-Raf proto-oncogene, serine/threonine kinase [Source:HGNC Symbol;Acc:HGNC:1097]" | 37844 | -0.330 | -0.2818 | Yes |
| 49 | RPS6KB1 | ribosomal protein S6 kinase B1 [Source:HGNC Symbol;Acc:HGNC:10436] | 38015 | -0.352 | -0.2559 | Yes |
| 50 | PPARD | peroxisome proliferator activated receptor delta [Source:HGNC Symbol;Acc:HGNC:9235] | 38050 | -0.356 | -0.2262 | Yes |
| 51 | SPI1 | Spi-1 proto-oncogene [Source:HGNC Symbol;Acc:HGNC:11241] | 38223 | -0.382 | -0.1977 | Yes |
| 52 | PIK3CA | "phosphatidylinositol-4,5-bisphosphate 3-kinase catalytic subunit alpha [Source:HGNC Symbol;Acc:HGNC:8975]" | 38268 | -0.391 | -0.1652 | Yes |
| 53 | RARA | retinoic acid receptor alpha [Source:HGNC Symbol;Acc:HGNC:9864] | 38322 | -0.403 | -0.1319 | Yes |
| 54 | TCF7 | transcription factor 7 [Source:HGNC Symbol;Acc:HGNC:11639] | 38380 | -0.413 | -0.0978 | Yes |
| 55 | NFKB1 | nuclear factor kappa B subunit 1 [Source:HGNC Symbol;Acc:HGNC:7794] | 38470 | -0.443 | -0.0620 | Yes |
| 56 | RELA | "RELA proto-oncogene, NF-kB subunit [Source:HGNC Symbol;Acc:HGNC:9955]" | 38475 | -0.445 | -0.0238 | Yes |
| 57 | SOS2 | SOS Ras/Rho guanine nucleotide exchange factor 2 [Source:HGNC Symbol;Acc:HGNC:11188] | 38581 | -0.528 | 0.0189 | Yes |
Table: GSEA details [plain text format]

  

Fig 2: KEGG\_ACUTE\_MYELOID\_LEUKEMIA      
 Blue-Pink O' Gram in the Space of the Analyzed GeneSet

  

Fig 3: KEGG\_ACUTE\_MYELOID\_LEUKEMIA: Random ES distribution      
 Gene set null distribution of ES for **KEGG\_ACUTE\_MYELOID\_LEUKEMIA**
