# Supplementary material for: Simultaneous Presentation of Multiple Myeloma and Lung Cancer: Case Report and Gene Bioinformatics Analysis
Source: Front Oncol. 2022 Jun 13;12:859735. doi: 10.3389/fonc.2022.859735 (PMC9235397; doi:10.3389/fonc.2022.859735)
Supplement: Supplementary file 1 [file DataSheet_1.zip › The bioinformatic analysis of MM and lung cancer supplementary materials/Enrichment analysis/MECR/GSEA_4.1.0/MM TCGA/KEGG.Gsea.1639040591792/KEGG_ADHERENS_JUNCTION.html]

Details for gene set KEGG\_ADHERENS\_JUNCTION[GSEA]

|  || Dataset | ExpData\_collapsed\_to\_symbols.ENSG00000116353\_profile\_in\_ExpData.cls #ENSG00000116353 |
| Phenotype | ENSG00000116353\_profile\_in\_ExpData.cls#ENSG00000116353 |
| Upregulated in class | ENSG00000116353\_neg |
| GeneSet | KEGG\_ADHERENS\_JUNCTION |
| Enrichment Score (ES) | -0.6360984 |
| Normalized Enrichment Score (NES) | -2.4167502 |
| Nominal p-value | 0.0 |
| FDR q-value | 0.0 |
| FWER p-Value | 0.0 |
Table: GSEA Results Summary

  

Fig 1: Enrichment plot: KEGG\_ADHERENS\_JUNCTION      
 Profile of the Running ES Score & Positions of GeneSet Members on the Rank Ordered List

  

| SYMBOL | TITLE | RANK IN GENE LIST | RANK METRIC SCORE | RUNNING ES | CORE ENRICHMENT || 1 | SSX2IP | SSX family member 2 interacting protein [Source:HGNC Symbol;Acc:HGNC:16509] | 2421 | 0.241 | -0.0423 | No |
| 2 | CTNNA2 | catenin alpha 2 [Source:HGNC Symbol;Acc:HGNC:2510] | 3776 | 0.184 | -0.0620 | No |
| 3 | PTPN6 | protein tyrosine phosphatase non-receptor type 6 [Source:HGNC Symbol;Acc:HGNC:9658] | 4119 | 0.173 | -0.0568 | No |
| 4 | ERBB2 | erb-b2 receptor tyrosine kinase 2 [Source:HGNC Symbol;Acc:HGNC:3430] | 5193 | 0.144 | -0.0726 | No |
| 5 | CTNNA3 | catenin alpha 3 [Source:HGNC Symbol;Acc:HGNC:2511] | 5301 | 0.142 | -0.0639 | No |
| 6 | PTPRB | protein tyrosine phosphatase receptor type B [Source:HGNC Symbol;Acc:HGNC:9665] | 5753 | 0.132 | -0.0648 | No |
| 7 | CSNK2A1 | casein kinase 2 alpha 1 [Source:HGNC Symbol;Acc:HGNC:2457] | 6052 | 0.126 | -0.0623 | No |
| 8 | RAC2 | Rac family small GTPase 2 [Source:HGNC Symbol;Acc:HGNC:9802] | 6127 | 0.124 | -0.0542 | No |
| 9 | MAP3K7 | mitogen-activated protein kinase kinase kinase 7 [Source:HGNC Symbol;Acc:HGNC:6859] | 12527 | 0.048 | -0.2133 | No |
| 10 | PTPRM | protein tyrosine phosphatase receptor type M [Source:HGNC Symbol;Acc:HGNC:9675] | 14066 | 0.038 | -0.2495 | No |
| 11 | WASF1 | WASP family member 1 [Source:HGNC Symbol;Acc:HGNC:12732] | 16267 | 0.024 | -0.3036 | No |
| 12 | IGF1R | insulin like growth factor 1 receptor [Source:HGNC Symbol;Acc:HGNC:5465] | 17189 | 0.019 | -0.3255 | No |
| 13 | WAS | WASP actin nucleation promoting factor [Source:HGNC Symbol;Acc:HGNC:12731] | 17936 | 0.015 | -0.3433 | No |
| 14 | SMAD2 | SMAD family member 2 [Source:HGNC Symbol;Acc:HGNC:6768] | 18855 | 0.010 | -0.3659 | No |
| 15 | FER | FER tyrosine kinase [Source:HGNC Symbol;Acc:HGNC:3655] | 21351 | -0.004 | -0.4291 | No |
| 16 | LMO7 | LIM domain 7 [Source:HGNC Symbol;Acc:HGNC:6646] | 22151 | -0.009 | -0.4488 | No |
| 17 | MAPK1 | mitogen-activated protein kinase 1 [Source:HGNC Symbol;Acc:HGNC:6871] | 23167 | -0.015 | -0.4735 | No |
| 18 | MET | "MET proto-oncogene, receptor tyrosine kinase [Source:HGNC Symbol;Acc:HGNC:7029]" | 25533 | -0.030 | -0.5313 | No |
| 19 | ACTN2 | actinin alpha 2 [Source:HGNC Symbol;Acc:HGNC:164] | 27204 | -0.043 | -0.5704 | No |
| 20 | NLK | nemo like kinase [Source:HGNC Symbol;Acc:HGNC:29858] | 27227 | -0.043 | -0.5675 | No |
| 21 | NECTIN3 | nectin cell adhesion molecule 3 [Source:HGNC Symbol;Acc:HGNC:17664] | 27995 | -0.050 | -0.5830 | No |
| 22 | PTPRF | protein tyrosine phosphatase receptor type F [Source:HGNC Symbol;Acc:HGNC:9670] | 28037 | -0.050 | -0.5800 | No |
| 23 | WASF3 | WASP family member 3 [Source:HGNC Symbol;Acc:HGNC:12734] | 28144 | -0.052 | -0.5786 | No |
| 24 | ACTN4 | actinin alpha 4 [Source:HGNC Symbol;Acc:HGNC:166] | 28351 | -0.054 | -0.5795 | No |
| 25 | NECTIN1 | nectin cell adhesion molecule 1 [Source:HGNC Symbol;Acc:HGNC:9706] | 28761 | -0.058 | -0.5853 | No |
| 26 | NECTIN4 | nectin cell adhesion molecule 4 [Source:HGNC Symbol;Acc:HGNC:19688] | 28767 | -0.058 | -0.5808 | No |
| 27 | ACP1 | acid phosphatase 1 [Source:HGNC Symbol;Acc:HGNC:122] | 29373 | -0.065 | -0.5910 | No |
| 28 | PARD3 | par-3 family cell polarity regulator [Source:HGNC Symbol;Acc:HGNC:16051] | 29993 | -0.072 | -0.6010 | No |
| 29 | TJP1 | tight junction protein 1 [Source:HGNC Symbol;Acc:HGNC:11827] | 30121 | -0.074 | -0.5983 | No |
| 30 | RAC3 | Rac family small GTPase 3 [Source:HGNC Symbol;Acc:HGNC:9803] | 30704 | -0.081 | -0.6066 | No |
| 31 | ACTB | actin beta [Source:HGNC Symbol;Acc:HGNC:132] | 30724 | -0.081 | -0.6006 | No |
| 32 | LEF1 | lymphoid enhancer binding factor 1 [Source:HGNC Symbol;Acc:HGNC:6551] | 31159 | -0.087 | -0.6046 | No |
| 33 | SNAI1 | snail family transcriptional repressor 1 [Source:HGNC Symbol;Acc:HGNC:11128] | 31657 | -0.094 | -0.6098 | No |
| 34 | PTPRJ | protein tyrosine phosphatase receptor type J [Source:HGNC Symbol;Acc:HGNC:9673] | 32692 | -0.113 | -0.6270 | Yes |
| 35 | ACTN3 | actinin alpha 3 [Source:HGNC Symbol;Acc:HGNC:165] | 32823 | -0.115 | -0.6211 | Yes |
| 36 | SNAI2 | snail family transcriptional repressor 2 [Source:HGNC Symbol;Acc:HGNC:11094] | 33252 | -0.124 | -0.6220 | Yes |
| 37 | FYN | "FYN proto-oncogene, Src family tyrosine kinase [Source:HGNC Symbol;Acc:HGNC:4037]" | 33801 | -0.137 | -0.6250 | Yes |
| 38 | TGFBR2 | transforming growth factor beta receptor 2 [Source:HGNC Symbol;Acc:HGNC:11773] | 33892 | -0.139 | -0.6161 | Yes |
| 39 | WASF2 | WASP family member 2 [Source:HGNC Symbol;Acc:HGNC:12733] | 34035 | -0.142 | -0.6083 | Yes |
| 40 | ACTG1 | actin gamma 1 [Source:HGNC Symbol;Acc:HGNC:144] | 34048 | -0.142 | -0.5971 | Yes |
| 41 | SRC | "SRC proto-oncogene, non-receptor tyrosine kinase [Source:HGNC Symbol;Acc:HGNC:11283]" | 34463 | -0.155 | -0.5952 | Yes |
| 42 | SMAD3 | SMAD family member 3 [Source:HGNC Symbol;Acc:HGNC:6769] | 35008 | -0.171 | -0.5954 | Yes |
| 43 | SORBS1 | sorbin and SH3 domain containing 1 [Source:HGNC Symbol;Acc:HGNC:14565] | 35044 | -0.172 | -0.5824 | Yes |
| 44 | CSNK2B | casein kinase 2 beta [Source:HGNC Symbol;Acc:HGNC:2460] | 35114 | -0.175 | -0.5701 | Yes |
| 45 | CTNNA1 | catenin alpha 1 [Source:HGNC Symbol;Acc:HGNC:2509] | 35334 | -0.182 | -0.5610 | Yes |
| 46 | EGFR | epidermal growth factor receptor [Source:HGNC Symbol;Acc:HGNC:3236] | 35417 | -0.186 | -0.5482 | Yes |
| 47 | AFDN | "afadin, adherens junction formation factor [Source:HGNC Symbol;Acc:HGNC:7137]" | 35571 | -0.190 | -0.5368 | Yes |
| 48 | MAPK3 | mitogen-activated protein kinase 3 [Source:HGNC Symbol;Acc:HGNC:6877] | 35579 | -0.191 | -0.5216 | Yes |
| 49 | NECTIN2 | nectin cell adhesion molecule 2 [Source:HGNC Symbol;Acc:HGNC:9707] | 35662 | -0.194 | -0.5081 | Yes |
| 50 | CDH1 | cadherin 1 [Source:HGNC Symbol;Acc:HGNC:1748] | 35798 | -0.199 | -0.4955 | Yes |
| 51 | VCL | vinculin [Source:HGNC Symbol;Acc:HGNC:12665] | 36176 | -0.215 | -0.4878 | Yes |
| 52 | ACTN1 | actinin alpha 1 [Source:HGNC Symbol;Acc:HGNC:163] | 36614 | -0.236 | -0.4799 | Yes |
| 53 | FGFR1 | fibroblast growth factor receptor 1 [Source:HGNC Symbol;Acc:HGNC:3688] | 36687 | -0.241 | -0.4624 | Yes |
| 54 | CSNK2A2 | casein kinase 2 alpha 2 [Source:HGNC Symbol;Acc:HGNC:2459] | 36789 | -0.246 | -0.4452 | Yes |
| 55 | TCF7L1 | transcription factor 7 like 1 [Source:HGNC Symbol;Acc:HGNC:11640] | 36849 | -0.251 | -0.4265 | Yes |
| 56 | BAIAP2 | BAR/IMD domain containing adaptor protein 2 [Source:HGNC Symbol;Acc:HGNC:947] | 36850 | -0.251 | -0.4064 | Yes |
| 57 | YES1 | "YES proto-oncogene 1, Src family tyrosine kinase [Source:HGNC Symbol;Acc:HGNC:12841]" | 36970 | -0.258 | -0.3887 | Yes |
| 58 | CTNND1 | catenin delta 1 [Source:HGNC Symbol;Acc:HGNC:2515] | 37003 | -0.260 | -0.3686 | Yes |
| 59 | TCF7L2 | transcription factor 7 like 2 [Source:HGNC Symbol;Acc:HGNC:11641] | 37205 | -0.273 | -0.3517 | Yes |
| 60 | RAC1 | Rac family small GTPase 1 [Source:HGNC Symbol;Acc:HGNC:9801] | 37263 | -0.277 | -0.3309 | Yes |
| 61 | RHOA | ras homolog family member A [Source:HGNC Symbol;Acc:HGNC:667] | 37417 | -0.290 | -0.3115 | Yes |
| 62 | EP300 | E1A binding protein p300 [Source:HGNC Symbol;Acc:HGNC:3373] | 37644 | -0.308 | -0.2925 | Yes |
| 63 | CDC42 | cell division cycle 42 [Source:HGNC Symbol;Acc:HGNC:1736] | 37805 | -0.324 | -0.2705 | Yes |
| 64 | IQGAP1 | IQ motif containing GTPase activating protein 1 [Source:HGNC Symbol;Acc:HGNC:6110] | 37829 | -0.329 | -0.2446 | Yes |
| 65 | CREBBP | CREB binding protein [Source:HGNC Symbol;Acc:HGNC:2348] | 37926 | -0.340 | -0.2198 | Yes |
| 66 | FARP2 | "FERM, ARH/RhoGEF and pleckstrin domain protein 2 [Source:HGNC Symbol;Acc:HGNC:16460]" | 37981 | -0.347 | -0.1932 | Yes |
| 67 | SMAD4 | SMAD family member 4 [Source:HGNC Symbol;Acc:HGNC:6770] | 37985 | -0.348 | -0.1653 | Yes |
| 68 | INSR | insulin receptor [Source:HGNC Symbol;Acc:HGNC:6091] | 38311 | -0.401 | -0.1413 | Yes |
| 69 | PTPN1 | protein tyrosine phosphatase non-receptor type 1 [Source:HGNC Symbol;Acc:HGNC:9642] | 38316 | -0.402 | -0.1091 | Yes |
| 70 | WASL | WASP like actin nucleation promoting factor [Source:HGNC Symbol;Acc:HGNC:12735] | 38341 | -0.407 | -0.0770 | Yes |
| 71 | TCF7 | transcription factor 7 [Source:HGNC Symbol;Acc:HGNC:11639] | 38380 | -0.413 | -0.0448 | Yes |
| 72 | TGFBR1 | transforming growth factor beta receptor 1 [Source:HGNC Symbol;Acc:HGNC:11772] | 38393 | -0.417 | -0.0116 | Yes |
| 73 | CTNNB1 | catenin beta 1 [Source:HGNC Symbol;Acc:HGNC:2514] | 38456 | -0.438 | 0.0221 | Yes |
Table: GSEA details [plain text format]

  

Fig 2: KEGG\_ADHERENS\_JUNCTION      
 Blue-Pink O' Gram in the Space of the Analyzed GeneSet

  

Fig 3: KEGG\_ADHERENS\_JUNCTION: Random ES distribution      
 Gene set null distribution of ES for **KEGG\_ADHERENS\_JUNCTION**
