# Supplementary material for: Simultaneous Presentation of Multiple Myeloma and Lung Cancer: Case Report and Gene Bioinformatics Analysis
Source: Front Oncol. 2022 Jun 13;12:859735. doi: 10.3389/fonc.2022.859735 (PMC9235397; doi:10.3389/fonc.2022.859735)
Supplement: Supplementary file 1 [file DataSheet_1.zip › The bioinformatic analysis of MM and lung cancer supplementary materials/Enrichment analysis/MECR/GSEA_4.1.0/MM TCGA/KEGG.Gsea.1639040591792/KEGG_AMINOACYL_TRNA_BIOSYNTHESIS.html]

Details for gene set KEGG\_AMINOACYL\_TRNA\_BIOSYNTHESIS[GSEA]

|  || Dataset | ExpData\_collapsed\_to\_symbols.ENSG00000116353\_profile\_in\_ExpData.cls #ENSG00000116353 |
| Phenotype | ENSG00000116353\_profile\_in\_ExpData.cls#ENSG00000116353 |
| Upregulated in class | ENSG00000116353\_pos |
| GeneSet | KEGG\_AMINOACYL\_TRNA\_BIOSYNTHESIS |
| Enrichment Score (ES) | 0.7382101 |
| Normalized Enrichment Score (NES) | 2.275539 |
| Nominal p-value | 0.0 |
| FDR q-value | 0.0 |
| FWER p-Value | 0.0 |
Table: GSEA Results Summary

  

Fig 1: Enrichment plot: KEGG\_AMINOACYL\_TRNA\_BIOSYNTHESIS      
 Profile of the Running ES Score & Positions of GeneSet Members on the Rank Ordered List

  

| SYMBOL | TITLE | RANK IN GENE LIST | RANK METRIC SCORE | RUNNING ES | CORE ENRICHMENT || 1 | LARS1 | leucyl-tRNA synthetase 1 [Source:HGNC Symbol;Acc:HGNC:6512] | 25 | 0.602 | 0.0514 | Yes |
| 2 | FARSB | phenylalanyl-tRNA synthetase subunit beta [Source:HGNC Symbol;Acc:HGNC:17800] | 28 | 0.591 | 0.1023 | Yes |
| 3 | EARS2 | "glutamyl-tRNA synthetase 2, mitochondrial [Source:HGNC Symbol;Acc:HGNC:29419]" | 29 | 0.588 | 0.1532 | Yes |
| 4 | DARS2 | "aspartyl-tRNA synthetase 2, mitochondrial [Source:HGNC Symbol;Acc:HGNC:25538]" | 53 | 0.564 | 0.2013 | Yes |
| 5 | FARS2 | "phenylalanyl-tRNA synthetase 2, mitochondrial [Source:HGNC Symbol;Acc:HGNC:21062]" | 91 | 0.536 | 0.2467 | Yes |
| 6 | FARSA | phenylalanyl-tRNA synthetase subunit alpha [Source:HGNC Symbol;Acc:HGNC:3592] | 152 | 0.503 | 0.2886 | Yes |
| 7 | IARS2 | "isoleucyl-tRNA synthetase 2, mitochondrial [Source:HGNC Symbol;Acc:HGNC:29685]" | 164 | 0.497 | 0.3313 | Yes |
| 8 | AARS2 | "alanyl-tRNA synthetase 2, mitochondrial [Source:HGNC Symbol;Acc:HGNC:21022]" | 208 | 0.482 | 0.3718 | Yes |
| 9 | TARS2 | "threonyl-tRNA synthetase 2, mitochondrial [Source:HGNC Symbol;Acc:HGNC:30740]" | 250 | 0.467 | 0.4112 | Yes |
| 10 | VARS1 | valyl-tRNA synthetase 1 [Source:HGNC Symbol;Acc:HGNC:12651] | 442 | 0.425 | 0.4431 | Yes |
| 11 | NARS2 | "asparaginyl-tRNA synthetase 2, mitochondrial [Source:HGNC Symbol;Acc:HGNC:26274]" | 454 | 0.422 | 0.4793 | Yes |
| 12 | MTFMT | mitochondrial methionyl-tRNA formyltransferase [Source:HGNC Symbol;Acc:HGNC:29666] | 462 | 0.420 | 0.5154 | Yes |
| 13 | MARS2 | "methionyl-tRNA synthetase 2, mitochondrial [Source:HGNC Symbol;Acc:HGNC:25133]" | 703 | 0.382 | 0.5423 | Yes |
| 14 | SARS2 | "seryl-tRNA synthetase 2, mitochondrial [Source:HGNC Symbol;Acc:HGNC:17697]" | 939 | 0.350 | 0.5665 | Yes |
| 15 | LARS2 | "leucyl-tRNA synthetase 2, mitochondrial [Source:HGNC Symbol;Acc:HGNC:17095]" | 1050 | 0.339 | 0.5931 | Yes |
| 16 | VARS2 | "valyl-tRNA synthetase 2, mitochondrial [Source:HGNC Symbol;Acc:HGNC:21642]" | 1087 | 0.336 | 0.6212 | Yes |
| 17 | PARS2 | "prolyl-tRNA synthetase 2, mitochondrial [Source:HGNC Symbol;Acc:HGNC:30563]" | 1174 | 0.327 | 0.6472 | Yes |
| 18 | EPRS1 | glutamyl-prolyl-tRNA synthetase 1 [Source:HGNC Symbol;Acc:HGNC:3418] | 1293 | 0.317 | 0.6716 | Yes |
| 19 | AARS1 | alanyl-tRNA synthetase 1 [Source:HGNC Symbol;Acc:HGNC:20] | 1391 | 0.307 | 0.6957 | Yes |
| 20 | IARS1 | isoleucyl-tRNA synthetase 1 [Source:HGNC Symbol;Acc:HGNC:5330] | 1422 | 0.305 | 0.7212 | Yes |
| 21 | YARS2 | tyrosyl-tRNA synthetase 2 [Source:HGNC Symbol;Acc:HGNC:24249] | 2041 | 0.262 | 0.7281 | Yes |
| 22 | KARS1 | lysyl-tRNA synthetase 1 [Source:HGNC Symbol;Acc:HGNC:6215] | 2798 | 0.222 | 0.7281 | Yes |
| 23 | TARS1 | threonyl-tRNA synthetase 1 [Source:HGNC Symbol;Acc:HGNC:11572] | 3110 | 0.209 | 0.7382 | Yes |
| 24 | CARS2 | "cysteinyl-tRNA synthetase 2, mitochondrial [Source:HGNC Symbol;Acc:HGNC:25695]" | 5065 | 0.147 | 0.7012 | No |
| 25 | NARS1 | asparaginyl-tRNA synthetase 1 [Source:HGNC Symbol;Acc:HGNC:7643] | 5363 | 0.140 | 0.7057 | No |
| 26 | YARS1 | tyrosyl-tRNA synthetase 1 [Source:HGNC Symbol;Acc:HGNC:12840] | 7515 | 0.101 | 0.6597 | No |
| 27 | DARS1 | aspartyl-tRNA synthetase 1 [Source:HGNC Symbol;Acc:HGNC:2678] | 7542 | 0.101 | 0.6678 | No |
| 28 | QARS1 | glutaminyl-tRNA synthetase 1 [Source:HGNC Symbol;Acc:HGNC:9751] | 7911 | 0.096 | 0.6667 | No |
| 29 | WARS2 | "tryptophanyl tRNA synthetase 2, mitochondrial [Source:HGNC Symbol;Acc:HGNC:12730]" | 8448 | 0.089 | 0.6608 | No |
| 30 | MARS1 | methionyl-tRNA synthetase 1 [Source:HGNC Symbol;Acc:HGNC:6898] | 9128 | 0.081 | 0.6505 | No |
| 31 | HARS1 | histidyl-tRNA synthetase 1 [Source:HGNC Symbol;Acc:HGNC:4816] | 10274 | 0.068 | 0.6272 | No |
| 32 | GARS1 | glycyl-tRNA synthetase 1 [Source:HGNC Symbol;Acc:HGNC:4162] | 10574 | 0.065 | 0.6252 | No |
| 33 | PSTK | phosphoseryl-tRNA kinase [Source:HGNC Symbol;Acc:HGNC:28578] | 17988 | 0.015 | 0.4377 | No |
| 34 | WARS1 | tryptophanyl-tRNA synthetase 1 [Source:HGNC Symbol;Acc:HGNC:12729] | 22142 | -0.009 | 0.3327 | No |
| 35 | TARS3 | threonyl-tRNA synthetase 3 [Source:HGNC Symbol;Acc:HGNC:24728] | 29804 | -0.070 | 0.1437 | No |
| 36 | SEPSECS | Sep (O-phosphoserine) tRNA:Sec (selenocysteine) tRNA synthase [Source:HGNC Symbol;Acc:HGNC:30605] | 33426 | -0.127 | 0.0626 | No |
| 37 | RARS2 | "arginyl-tRNA synthetase 2, mitochondrial [Source:HGNC Symbol;Acc:HGNC:21406]" | 33924 | -0.140 | 0.0620 | No |
| 38 | RARS1 | arginyl-tRNA synthetase 1 [Source:HGNC Symbol;Acc:HGNC:9870] | 34408 | -0.153 | 0.0629 | No |
| 39 | HARS2 | "histidyl-tRNA synthetase 2, mitochondrial [Source:HGNC Symbol;Acc:HGNC:4817]" | 34432 | -0.153 | 0.0756 | No |
| 40 | CARS1 | cysteinyl-tRNA synthetase 1 [Source:HGNC Symbol;Acc:HGNC:1493] | 37080 | -0.264 | 0.0310 | No |
| 41 | SARS1 | seryl-tRNA synthetase 1 [Source:HGNC Symbol;Acc:HGNC:10537] | 37558 | -0.301 | 0.0449 | No |
Table: GSEA details [plain text format]

  

Fig 2: KEGG\_AMINOACYL\_TRNA\_BIOSYNTHESIS      
 Blue-Pink O' Gram in the Space of the Analyzed GeneSet

  

Fig 3: KEGG\_AMINOACYL\_TRNA\_BIOSYNTHESIS: Random ES distribution      
 Gene set null distribution of ES for **KEGG\_AMINOACYL\_TRNA\_BIOSYNTHESIS**
