# Supplementary material for: Simultaneous Presentation of Multiple Myeloma and Lung Cancer: Case Report and Gene Bioinformatics Analysis
Source: Front Oncol. 2022 Jun 13;12:859735. doi: 10.3389/fonc.2022.859735 (PMC9235397; doi:10.3389/fonc.2022.859735)
Supplement: Supplementary file 1 [file DataSheet_1.zip › The bioinformatic analysis of MM and lung cancer supplementary materials/Enrichment analysis/MECR/GSEA_4.1.0/MM TCGA/KEGG.Gsea.1639040591792/KEGG_BASE_EXCISION_REPAIR.html]

Details for gene set KEGG\_BASE\_EXCISION\_REPAIR[GSEA]

|  || Dataset | ExpData\_collapsed\_to\_symbols.ENSG00000116353\_profile\_in\_ExpData.cls #ENSG00000116353 |
| Phenotype | ENSG00000116353\_profile\_in\_ExpData.cls#ENSG00000116353 |
| Upregulated in class | ENSG00000116353\_pos |
| GeneSet | KEGG\_BASE\_EXCISION\_REPAIR |
| Enrichment Score (ES) | 0.74010503 |
| Normalized Enrichment Score (NES) | 2.265399 |
| Nominal p-value | 0.0 |
| FDR q-value | 0.0 |
| FWER p-Value | 0.0 |
Table: GSEA Results Summary

  

Fig 1: Enrichment plot: KEGG\_BASE\_EXCISION\_REPAIR      
 Profile of the Running ES Score & Positions of GeneSet Members on the Rank Ordered List

  

| SYMBOL | TITLE | RANK IN GENE LIST | RANK METRIC SCORE | RUNNING ES | CORE ENRICHMENT || 1 | POLD2 | "DNA polymerase delta 2, accessory subunit [Source:HGNC Symbol;Acc:HGNC:9176]" | 15 | 0.617 | 0.0787 | Yes |
| 2 | PARP1 | poly(ADP-ribose) polymerase 1 [Source:HGNC Symbol;Acc:HGNC:270] | 367 | 0.440 | 0.1262 | Yes |
| 3 | SMUG1 | single-strand-selective monofunctional uracil-DNA glycosylase 1 [Source:HGNC Symbol;Acc:HGNC:17148] | 459 | 0.420 | 0.1777 | Yes |
| 4 | POLD1 | "DNA polymerase delta 1, catalytic subunit [Source:HGNC Symbol;Acc:HGNC:9175]" | 541 | 0.405 | 0.2276 | Yes |
| 5 | NTHL1 | nth like DNA glycosylase 1 [Source:HGNC Symbol;Acc:HGNC:8028] | 601 | 0.397 | 0.2770 | Yes |
| 6 | UNG | uracil DNA glycosylase [Source:HGNC Symbol;Acc:HGNC:12572] | 799 | 0.369 | 0.3193 | Yes |
| 7 | APEX2 | apurinic/apyrimidinic endodeoxyribonuclease 2 [Source:HGNC Symbol;Acc:HGNC:17889] | 823 | 0.365 | 0.3655 | Yes |
| 8 | PCNA | proliferating cell nuclear antigen [Source:HGNC Symbol;Acc:HGNC:8729] | 1093 | 0.336 | 0.4016 | Yes |
| 9 | APEX1 | apurinic/apyrimidinic endodeoxyribonuclease 1 [Source:HGNC Symbol;Acc:HGNC:587] | 1255 | 0.320 | 0.4385 | Yes |
| 10 | OGG1 | 8-oxoguanine DNA glycosylase [Source:HGNC Symbol;Acc:HGNC:8125] | 1316 | 0.315 | 0.4773 | Yes |
| 11 | POLD3 | "DNA polymerase delta 3, accessory subunit [Source:HGNC Symbol;Acc:HGNC:20932]" | 1700 | 0.284 | 0.5039 | Yes |
| 12 | FEN1 | flap structure-specific endonuclease 1 [Source:HGNC Symbol;Acc:HGNC:3650] | 1754 | 0.279 | 0.5383 | Yes |
| 13 | LIG3 | DNA ligase 3 [Source:HGNC Symbol;Acc:HGNC:6600] | 1961 | 0.266 | 0.5672 | Yes |
| 14 | NEIL2 | nei like DNA glycosylase 2 [Source:HGNC Symbol;Acc:HGNC:18956] | 2067 | 0.261 | 0.5979 | Yes |
| 15 | NEIL3 | nei like DNA glycosylase 3 [Source:HGNC Symbol;Acc:HGNC:24573] | 2113 | 0.257 | 0.6297 | Yes |
| 16 | MUTYH | mutY DNA glycosylase [Source:HGNC Symbol;Acc:HGNC:7527] | 2243 | 0.250 | 0.6584 | Yes |
| 17 | POLL | DNA polymerase lambda [Source:HGNC Symbol;Acc:HGNC:9184] | 2319 | 0.246 | 0.6880 | Yes |
| 18 | PARP3 | poly(ADP-ribose) polymerase family member 3 [Source:HGNC Symbol;Acc:HGNC:273] | 2733 | 0.226 | 0.7064 | Yes |
| 19 | POLE2 | "DNA polymerase epsilon 2, accessory subunit [Source:HGNC Symbol;Acc:HGNC:9178]" | 3128 | 0.208 | 0.7230 | Yes |
| 20 | POLE4 | "DNA polymerase epsilon 4, accessory subunit [Source:HGNC Symbol;Acc:HGNC:18755]" | 3440 | 0.195 | 0.7401 | Yes |
| 21 | PARP2 | poly(ADP-ribose) polymerase 2 [Source:HGNC Symbol;Acc:HGNC:272] | 5511 | 0.137 | 0.7049 | No |
| 22 | PARP4 | poly(ADP-ribose) polymerase family member 4 [Source:HGNC Symbol;Acc:HGNC:271] | 8888 | 0.084 | 0.6297 | No |
| 23 | POLE | "DNA polymerase epsilon, catalytic subunit [Source:HGNC Symbol;Acc:HGNC:9177]" | 9755 | 0.073 | 0.6171 | No |
| 24 | MPG | N-methylpurine DNA glycosylase [Source:HGNC Symbol;Acc:HGNC:7211] | 10253 | 0.068 | 0.6132 | No |
| 25 | HMGB1P1 | high mobility group box 1 pseudogene 1 [Source:HGNC Symbol;Acc:HGNC:4993] | 13929 | 0.038 | 0.5246 | No |
| 26 | HMGB1P40 | high mobility group box 1 pseudogene 40 [Source:HGNC Symbol;Acc:HGNC:39187] | 14145 | 0.037 | 0.5238 | No |
| 27 | TDG | thymine DNA glycosylase [Source:HGNC Symbol;Acc:HGNC:11700] | 15804 | 0.027 | 0.4851 | No |
| 28 | LIG1 | DNA ligase 1 [Source:HGNC Symbol;Acc:HGNC:6598] | 24175 | -0.021 | 0.2748 | No |
| 29 | XRCC1 | X-ray repair cross complementing 1 [Source:HGNC Symbol;Acc:HGNC:12828] | 24743 | -0.025 | 0.2635 | No |
| 30 | NEIL1 | nei like DNA glycosylase 1 [Source:HGNC Symbol;Acc:HGNC:18448] | 26968 | -0.041 | 0.2121 | No |
| 31 | POLE3 | "DNA polymerase epsilon 3, accessory subunit [Source:HGNC Symbol;Acc:HGNC:13546]" | 27729 | -0.048 | 0.1989 | No |
| 32 | MBD4 | "methyl-CpG binding domain 4, DNA glycosylase [Source:HGNC Symbol;Acc:HGNC:6919]" | 34376 | -0.152 | 0.0492 | No |
| 33 | POLD4 | "DNA polymerase delta 4, accessory subunit [Source:HGNC Symbol;Acc:HGNC:14106]" | 34723 | -0.162 | 0.0612 | No |
| 34 | POLB | DNA polymerase beta [Source:HGNC Symbol;Acc:HGNC:9174] | 35578 | -0.191 | 0.0639 | No |
| 35 | HMGB1 | high mobility group box 1 [Source:HGNC Symbol;Acc:HGNC:4983] | 36774 | -0.245 | 0.0649 | No |
Table: GSEA details [plain text format]

  

Fig 2: KEGG\_BASE\_EXCISION\_REPAIR      
 Blue-Pink O' Gram in the Space of the Analyzed GeneSet

  

Fig 3: KEGG\_BASE\_EXCISION\_REPAIR: Random ES distribution      
 Gene set null distribution of ES for **KEGG\_BASE\_EXCISION\_REPAIR**
