# Supplementary material for: Simultaneous Presentation of Multiple Myeloma and Lung Cancer: Case Report and Gene Bioinformatics Analysis
Source: Front Oncol. 2022 Jun 13;12:859735. doi: 10.3389/fonc.2022.859735 (PMC9235397; doi:10.3389/fonc.2022.859735)
Supplement: Supplementary file 1 [file DataSheet_1.zip › The bioinformatic analysis of MM and lung cancer supplementary materials/Enrichment analysis/MECR/GSEA_4.1.0/MM TCGA/KEGG.Gsea.1639040591792/KEGG_BUTANOATE_METABOLISM.html]

Details for gene set KEGG\_BUTANOATE\_METABOLISM[GSEA]

|  || Dataset | ExpData\_collapsed\_to\_symbols.ENSG00000116353\_profile\_in\_ExpData.cls #ENSG00000116353 |
| Phenotype | ENSG00000116353\_profile\_in\_ExpData.cls#ENSG00000116353 |
| Upregulated in class | ENSG00000116353\_pos |
| GeneSet | KEGG\_BUTANOATE\_METABOLISM |
| Enrichment Score (ES) | 0.664697 |
| Normalized Enrichment Score (NES) | 2.012451 |
| Nominal p-value | 0.0 |
| FDR q-value | 3.2572888E-4 |
| FWER p-Value | 0.007 |
Table: GSEA Results Summary

  

Fig 1: Enrichment plot: KEGG\_BUTANOATE\_METABOLISM      
 Profile of the Running ES Score & Positions of GeneSet Members on the Rank Ordered List

  

| SYMBOL | TITLE | RANK IN GENE LIST | RANK METRIC SCORE | RUNNING ES | CORE ENRICHMENT || 1 | BDH1 | 3-hydroxybutyrate dehydrogenase 1 [Source:HGNC Symbol;Acc:HGNC:1027] | 113 | 0.518 | 0.0719 | Yes |
| 2 | L2HGDH | L-2-hydroxyglutarate dehydrogenase [Source:HGNC Symbol;Acc:HGNC:20499] | 144 | 0.505 | 0.1440 | Yes |
| 3 | PDHA1 | pyruvate dehydrogenase E1 subunit alpha 1 [Source:HGNC Symbol;Acc:HGNC:8806] | 223 | 0.477 | 0.2109 | Yes |
| 4 | ALDH1B1 | aldehyde dehydrogenase 1 family member B1 [Source:HGNC Symbol;Acc:HGNC:407] | 372 | 0.439 | 0.2704 | Yes |
| 5 | OXCT1 | 3-oxoacid CoA-transferase 1 [Source:HGNC Symbol;Acc:HGNC:8527] | 530 | 0.407 | 0.3252 | Yes |
| 6 | ACSM3 | acyl-CoA synthetase medium chain family member 3 [Source:HGNC Symbol;Acc:HGNC:10522] | 871 | 0.359 | 0.3684 | Yes |
| 7 | ECHS1 | "enoyl-CoA hydratase, short chain 1 [Source:HGNC Symbol;Acc:HGNC:3151]" | 890 | 0.356 | 0.4192 | Yes |
| 8 | ALDH3A2 | aldehyde dehydrogenase 3 family member A2 [Source:HGNC Symbol;Acc:HGNC:403] | 902 | 0.354 | 0.4701 | Yes |
| 9 | ALDH9A1 | aldehyde dehydrogenase 9 family member A1 [Source:HGNC Symbol;Acc:HGNC:412] | 1406 | 0.306 | 0.5014 | Yes |
| 10 | ACAT1 | acetyl-CoA acetyltransferase 1 [Source:HGNC Symbol;Acc:HGNC:93] | 1467 | 0.302 | 0.5434 | Yes |
| 11 | HADH | hydroxyacyl-CoA dehydrogenase [Source:HGNC Symbol;Acc:HGNC:4799] | 1802 | 0.276 | 0.5747 | Yes |
| 12 | PDHB | pyruvate dehydrogenase E1 subunit beta [Source:HGNC Symbol;Acc:HGNC:8808] | 2053 | 0.261 | 0.6060 | Yes |
| 13 | EHHADH | enoyl-CoA hydratase and 3-hydroxyacyl CoA dehydrogenase [Source:HGNC Symbol;Acc:HGNC:3247] | 2104 | 0.258 | 0.6420 | Yes |
| 14 | ALDH5A1 | aldehyde dehydrogenase 5 family member A1 [Source:HGNC Symbol;Acc:HGNC:408] | 2541 | 0.235 | 0.6647 | Yes |
| 15 | HMGCL | 3-hydroxy-3-methylglutaryl-CoA lyase [Source:HGNC Symbol;Acc:HGNC:5005] | 4068 | 0.174 | 0.6510 | No |
| 16 | ACSM4 | acyl-CoA synthetase medium chain family member 4 [Source:HGNC Symbol;Acc:HGNC:32016] | 5101 | 0.146 | 0.6458 | No |
| 17 | BDH2 | 3-hydroxybutyrate dehydrogenase 2 [Source:HGNC Symbol;Acc:HGNC:32389] | 6080 | 0.125 | 0.6390 | No |
| 18 | ABAT | 4-aminobutyrate aminotransferase [Source:HGNC Symbol;Acc:HGNC:23] | 6280 | 0.122 | 0.6514 | No |
| 19 | HADHA | hydroxyacyl-CoA dehydrogenase trifunctional multienzyme complex subunit alpha [Source:HGNC Symbol;Acc:HGNC:4801] | 8241 | 0.092 | 0.6148 | No |
| 20 | PDHA2 | pyruvate dehydrogenase E1 subunit alpha 2 [Source:HGNC Symbol;Acc:HGNC:8807] | 8806 | 0.085 | 0.6126 | No |
| 21 | ACADS | acyl-CoA dehydrogenase short chain [Source:HGNC Symbol;Acc:HGNC:90] | 9834 | 0.073 | 0.5970 | No |
| 22 | ACAT2 | acetyl-CoA acetyltransferase 2 [Source:HGNC Symbol;Acc:HGNC:94] | 10235 | 0.068 | 0.5967 | No |
| 23 | GAD2 | glutamate decarboxylase 2 [Source:HGNC Symbol;Acc:HGNC:4093] | 10750 | 0.063 | 0.5927 | No |
| 24 | AACS | acetoacetyl-CoA synthetase [Source:HGNC Symbol;Acc:HGNC:21298] | 11057 | 0.060 | 0.5936 | No |
| 25 | ACSM1 | acyl-CoA synthetase medium chain family member 1 [Source:HGNC Symbol;Acc:HGNC:18049] | 11408 | 0.057 | 0.5930 | No |
| 26 | ALDH7A1 | aldehyde dehydrogenase 7 family member A1 [Source:HGNC Symbol;Acc:HGNC:877] | 14303 | 0.036 | 0.5246 | No |
| 27 | AKR1B10 | aldo-keto reductase family 1 member B10 [Source:HGNC Symbol;Acc:HGNC:382] | 24513 | -0.023 | 0.2681 | No |
| 28 | HMGCS2 | 3-hydroxy-3-methylglutaryl-CoA synthase 2 [Source:HGNC Symbol;Acc:HGNC:5008] | 29182 | -0.063 | 0.1583 | No |
| 29 | ACSM5 | acyl-CoA synthetase medium chain family member 5 [Source:HGNC Symbol;Acc:HGNC:26060] | 30204 | -0.075 | 0.1431 | No |
| 30 | OXCT2 | 3-oxoacid CoA-transferase 2 [Source:HGNC Symbol;Acc:HGNC:18606] | 30341 | -0.076 | 0.1506 | No |
| 31 | GAD1 | glutamate decarboxylase 1 [Source:HGNC Symbol;Acc:HGNC:4092] | 32937 | -0.118 | 0.1016 | No |
| 32 | ACSM2A | acyl-CoA synthetase medium chain family member 2A [Source:HGNC Symbol;Acc:HGNC:32017] | 33165 | -0.122 | 0.1134 | No |
| 33 | HMGCS1 | 3-hydroxy-3-methylglutaryl-CoA synthase 1 [Source:HGNC Symbol;Acc:HGNC:5007] | 34042 | -0.142 | 0.1117 | No |
| 34 | ALDH2 | aldehyde dehydrogenase 2 family member [Source:HGNC Symbol;Acc:HGNC:404] | 34564 | -0.157 | 0.1211 | No |
Table: GSEA details [plain text format]

  

Fig 2: KEGG\_BUTANOATE\_METABOLISM      
 Blue-Pink O' Gram in the Space of the Analyzed GeneSet

  

Fig 3: KEGG\_BUTANOATE\_METABOLISM: Random ES distribution      
 Gene set null distribution of ES for **KEGG\_BUTANOATE\_METABOLISM**
