# Supplementary material for: Simultaneous Presentation of Multiple Myeloma and Lung Cancer: Case Report and Gene Bioinformatics Analysis
Source: Front Oncol. 2022 Jun 13;12:859735. doi: 10.3389/fonc.2022.859735 (PMC9235397; doi:10.3389/fonc.2022.859735)
Supplement: Supplementary file 1 [file DataSheet_1.zip › The bioinformatic analysis of MM and lung cancer supplementary materials/Enrichment analysis/MECR/GSEA_4.1.0/MM TCGA/KEGG.Gsea.1639040591792/KEGG_B_CELL_RECEPTOR_SIGNALING_PATHWAY.html]

Details for gene set KEGG\_B\_CELL\_RECEPTOR\_SIGNALING\_PATHWAY[GSEA]

|  || Dataset | ExpData\_collapsed\_to\_symbols.ENSG00000116353\_profile\_in\_ExpData.cls #ENSG00000116353 |
| Phenotype | ENSG00000116353\_profile\_in\_ExpData.cls#ENSG00000116353 |
| Upregulated in class | ENSG00000116353\_neg |
| GeneSet | KEGG\_B\_CELL\_RECEPTOR\_SIGNALING\_PATHWAY |
| Enrichment Score (ES) | -0.58707374 |
| Normalized Enrichment Score (NES) | -2.195072 |
| Nominal p-value | 0.0 |
| FDR q-value | 0.0 |
| FWER p-Value | 0.0 |
Table: GSEA Results Summary

  

Fig 1: Enrichment plot: KEGG\_B\_CELL\_RECEPTOR\_SIGNALING\_PATHWAY      
 Profile of the Running ES Score & Positions of GeneSet Members on the Rank Ordered List

  

| SYMBOL | TITLE | RANK IN GENE LIST | RANK METRIC SCORE | RUNNING ES | CORE ENRICHMENT || 1 | PPP3CB | protein phosphatase 3 catalytic subunit beta [Source:HGNC Symbol;Acc:HGNC:9315] | 842 | 0.363 | 0.0034 | No |
| 2 | PIK3R2 | phosphoinositide-3-kinase regulatory subunit 2 [Source:HGNC Symbol;Acc:HGNC:8980] | 1248 | 0.320 | 0.0150 | No |
| 3 | PIK3R3 | phosphoinositide-3-kinase regulatory subunit 3 [Source:HGNC Symbol;Acc:HGNC:8981] | 2602 | 0.231 | -0.0037 | No |
| 4 | NFATC3 | nuclear factor of activated T cells 3 [Source:HGNC Symbol;Acc:HGNC:7777] | 2677 | 0.228 | 0.0100 | No |
| 5 | BTK | Bruton tyrosine kinase [Source:HGNC Symbol;Acc:HGNC:1133] | 2874 | 0.219 | 0.0200 | No |
| 6 | FOS | "Fos proto-oncogene, AP-1 transcription factor subunit [Source:HGNC Symbol;Acc:HGNC:3796]" | 3070 | 0.210 | 0.0294 | No |
| 7 | VAV1 | vav guanine nucleotide exchange factor 1 [Source:HGNC Symbol;Acc:HGNC:12657] | 3764 | 0.184 | 0.0243 | No |
| 8 | CR2 | complement C3d receptor 2 [Source:HGNC Symbol;Acc:HGNC:2336] | 4100 | 0.174 | 0.0277 | No |
| 9 | PTPN6 | protein tyrosine phosphatase non-receptor type 6 [Source:HGNC Symbol;Acc:HGNC:9658] | 4119 | 0.173 | 0.0391 | No |
| 10 | JUN | "Jun proto-oncogene, AP-1 transcription factor subunit [Source:HGNC Symbol;Acc:HGNC:6204]" | 5088 | 0.146 | 0.0244 | No |
| 11 | MAP2K2 | mitogen-activated protein kinase kinase 2 [Source:HGNC Symbol;Acc:HGNC:6842] | 5104 | 0.146 | 0.0340 | No |
| 12 | RAC2 | Rac family small GTPase 2 [Source:HGNC Symbol;Acc:HGNC:9802] | 6127 | 0.124 | 0.0165 | No |
| 13 | PIK3CB | "phosphatidylinositol-4,5-bisphosphate 3-kinase catalytic subunit beta [Source:HGNC Symbol;Acc:HGNC:8976]" | 7716 | 0.099 | -0.0172 | No |
| 14 | CHUK | component of inhibitor of nuclear factor kappa B kinase complex [Source:HGNC Symbol;Acc:HGNC:1974] | 9925 | 0.071 | -0.0686 | No |
| 15 | PRKCB | protein kinase C beta [Source:HGNC Symbol;Acc:HGNC:9395] | 11760 | 0.054 | -0.1116 | No |
| 16 | PPP3R2 | "protein phosphatase 3 regulatory subunit B, beta [Source:HGNC Symbol;Acc:HGNC:9318]" | 11820 | 0.054 | -0.1094 | No |
| 17 | GSK3B | glycogen synthase kinase 3 beta [Source:HGNC Symbol;Acc:HGNC:4617] | 12189 | 0.051 | -0.1153 | No |
| 18 | CD79B | CD79b molecule [Source:HGNC Symbol;Acc:HGNC:1699] | 12439 | 0.049 | -0.1183 | No |
| 19 | CARD11 | caspase recruitment domain family member 11 [Source:HGNC Symbol;Acc:HGNC:16393] | 13545 | 0.041 | -0.1437 | No |
| 20 | CHP1 | calcineurin like EF-hand protein 1 [Source:HGNC Symbol;Acc:HGNC:17433] | 15945 | 0.026 | -0.2030 | No |
| 21 | NFATC4 | nuclear factor of activated T cells 4 [Source:HGNC Symbol;Acc:HGNC:7778] | 17587 | 0.017 | -0.2437 | No |
| 22 | SOS1 | SOS Ras/Rac guanine nucleotide exchange factor 1 [Source:HGNC Symbol;Acc:HGNC:11187] | 18532 | 0.012 | -0.2669 | No |
| 23 | IKBKB | inhibitor of nuclear factor kappa B kinase subunit beta [Source:HGNC Symbol;Acc:HGNC:5960] | 20718 | -0.000 | -0.3226 | No |
| 24 | CHP2 | calcineurin like EF-hand protein 2 [Source:HGNC Symbol;Acc:HGNC:24927] | 23148 | -0.015 | -0.3835 | No |
| 25 | MAPK1 | mitogen-activated protein kinase 1 [Source:HGNC Symbol;Acc:HGNC:6871] | 23167 | -0.015 | -0.3829 | No |
| 26 | FCGR2B | Fc fragment of IgG receptor IIb [Source:HGNC Symbol;Acc:HGNC:3618] | 24767 | -0.025 | -0.4219 | No |
| 27 | HRAS | "HRas proto-oncogene, GTPase [Source:HGNC Symbol;Acc:HGNC:5173]" | 26672 | -0.039 | -0.4678 | No |
| 28 | PPP3CA | protein phosphatase 3 catalytic subunit alpha [Source:HGNC Symbol;Acc:HGNC:9314] | 28890 | -0.059 | -0.5202 | No |
| 29 | VAV3 | vav guanine nucleotide exchange factor 3 [Source:HGNC Symbol;Acc:HGNC:12659] | 29349 | -0.065 | -0.5275 | No |
| 30 | RAC3 | Rac family small GTPase 3 [Source:HGNC Symbol;Acc:HGNC:9803] | 30704 | -0.081 | -0.5565 | No |
| 31 | AKT1 | AKT serine/threonine kinase 1 [Source:HGNC Symbol;Acc:HGNC:391] | 31906 | -0.098 | -0.5804 | Yes |
| 32 | PIK3AP1 | phosphoinositide-3-kinase adaptor protein 1 [Source:HGNC Symbol;Acc:HGNC:30034] | 32134 | -0.102 | -0.5792 | Yes |
| 33 | IFITM1 | interferon induced transmembrane protein 1 [Source:HGNC Symbol;Acc:HGNC:5412] | 32197 | -0.103 | -0.5737 | Yes |
| 34 | IKBKG | inhibitor of nuclear factor kappa B kinase regulatory subunit gamma [Source:HGNC Symbol;Acc:HGNC:5961] | 32205 | -0.103 | -0.5668 | Yes |
| 35 | PLCG2 | phospholipase C gamma 2 [Source:HGNC Symbol;Acc:HGNC:9066] | 32294 | -0.105 | -0.5619 | Yes |
| 36 | CD22 | CD22 molecule [Source:HGNC Symbol;Acc:HGNC:1643] | 32416 | -0.107 | -0.5576 | Yes |
| 37 | AKT2 | AKT serine/threonine kinase 2 [Source:HGNC Symbol;Acc:HGNC:392] | 33213 | -0.123 | -0.5695 | Yes |
| 38 | SYK | spleen associated tyrosine kinase [Source:HGNC Symbol;Acc:HGNC:11491] | 33678 | -0.133 | -0.5722 | Yes |
| 39 | NRAS | "NRAS proto-oncogene, GTPase [Source:HGNC Symbol;Acc:HGNC:7989]" | 33739 | -0.135 | -0.5645 | Yes |
| 40 | PIK3CD | "phosphatidylinositol-4,5-bisphosphate 3-kinase catalytic subunit delta [Source:HGNC Symbol;Acc:HGNC:8977]" | 33794 | -0.137 | -0.5566 | Yes |
| 41 | VAV2 | vav guanine nucleotide exchange factor 2 [Source:HGNC Symbol;Acc:HGNC:12658] | 34251 | -0.148 | -0.5580 | Yes |
| 42 | CD81 | CD81 molecule [Source:HGNC Symbol;Acc:HGNC:1701] | 34323 | -0.150 | -0.5496 | Yes |
| 43 | AKT3 | AKT serine/threonine kinase 3 [Source:HGNC Symbol;Acc:HGNC:393] | 34501 | -0.156 | -0.5434 | Yes |
| 44 | CD72 | CD72 molecule [Source:HGNC Symbol;Acc:HGNC:1696] | 35340 | -0.183 | -0.5523 | Yes |
| 45 | LILRB3 | leukocyte immunoglobulin like receptor B3 [Source:HGNC Symbol;Acc:HGNC:6607] | 35548 | -0.190 | -0.5446 | Yes |
| 46 | MAPK3 | mitogen-activated protein kinase 3 [Source:HGNC Symbol;Acc:HGNC:6877] | 35579 | -0.191 | -0.5323 | Yes |
| 47 | CD19 | CD19 molecule [Source:HGNC Symbol;Acc:HGNC:1633] | 35767 | -0.198 | -0.5235 | Yes |
| 48 | MAP2K1 | mitogen-activated protein kinase kinase 1 [Source:HGNC Symbol;Acc:HGNC:6840] | 36024 | -0.208 | -0.5158 | Yes |
| 49 | MALT1 | MALT1 paracaspase [Source:HGNC Symbol;Acc:HGNC:6819] | 36092 | -0.211 | -0.5030 | Yes |
| 50 | PIK3CG | "phosphatidylinositol-4,5-bisphosphate 3-kinase catalytic subunit gamma [Source:HGNC Symbol;Acc:HGNC:8978]" | 36243 | -0.217 | -0.4920 | Yes |
| 51 | INPP5D | inositol polyphosphate-5-phosphatase D [Source:HGNC Symbol;Acc:HGNC:6079] | 36404 | -0.226 | -0.4806 | Yes |
| 52 | GRB2 | growth factor receptor bound protein 2 [Source:HGNC Symbol;Acc:HGNC:4566] | 36523 | -0.231 | -0.4678 | Yes |
| 53 | NFATC2 | nuclear factor of activated T cells 2 [Source:HGNC Symbol;Acc:HGNC:7776] | 36649 | -0.238 | -0.4547 | Yes |
| 54 | CD79A | CD79a molecule [Source:HGNC Symbol;Acc:HGNC:1698] | 36675 | -0.240 | -0.4389 | Yes |
| 55 | BLNK | B cell linker [Source:HGNC Symbol;Acc:HGNC:14211] | 36969 | -0.258 | -0.4288 | Yes |
| 56 | NFAT5 | nuclear factor of activated T cells 5 [Source:HGNC Symbol;Acc:HGNC:7774] | 37139 | -0.268 | -0.4147 | Yes |
| 57 | PIK3R1 | phosphoinositide-3-kinase regulatory subunit 1 [Source:HGNC Symbol;Acc:HGNC:8979] | 37172 | -0.270 | -0.3970 | Yes |
| 58 | NFKBIA | NFKB inhibitor alpha [Source:HGNC Symbol;Acc:HGNC:7797] | 37222 | -0.275 | -0.3795 | Yes |
| 59 | RAC1 | Rac family small GTPase 1 [Source:HGNC Symbol;Acc:HGNC:9801] | 37263 | -0.277 | -0.3615 | Yes |
| 60 | NFATC1 | nuclear factor of activated T cells 1 [Source:HGNC Symbol;Acc:HGNC:7775] | 37512 | -0.297 | -0.3475 | Yes |
| 61 | RASGRP3 | RAS guanyl releasing protein 3 [Source:HGNC Symbol;Acc:HGNC:14545] | 37579 | -0.303 | -0.3285 | Yes |
| 62 | DAPP1 | dual adaptor of phosphotyrosine and 3-phosphoinositides 1 [Source:HGNC Symbol;Acc:HGNC:16500] | 37626 | -0.307 | -0.3086 | Yes |
| 63 | PIK3R5 | phosphoinositide-3-kinase regulatory subunit 5 [Source:HGNC Symbol;Acc:HGNC:30035] | 37628 | -0.307 | -0.2877 | Yes |
| 64 | KRAS | "KRAS proto-oncogene, GTPase [Source:HGNC Symbol;Acc:HGNC:6407]" | 37789 | -0.323 | -0.2697 | Yes |
| 65 | RAF1 | "Raf-1 proto-oncogene, serine/threonine kinase [Source:HGNC Symbol;Acc:HGNC:9829]" | 37807 | -0.325 | -0.2479 | Yes |
| 66 | NFKBIB | NFKB inhibitor beta [Source:HGNC Symbol;Acc:HGNC:7798] | 37987 | -0.348 | -0.2287 | Yes |
| 67 | PPP3R1 | "protein phosphatase 3 regulatory subunit B, alpha [Source:HGNC Symbol;Acc:HGNC:9317]" | 37988 | -0.348 | -0.2048 | Yes |
| 68 | PPP3CC | protein phosphatase 3 catalytic subunit gamma [Source:HGNC Symbol;Acc:HGNC:9316] | 38255 | -0.389 | -0.1850 | Yes |
| 69 | PIK3CA | "phosphatidylinositol-4,5-bisphosphate 3-kinase catalytic subunit alpha [Source:HGNC Symbol;Acc:HGNC:8975]" | 38268 | -0.391 | -0.1586 | Yes |
| 70 | NFKBIE | NFKB inhibitor epsilon [Source:HGNC Symbol;Acc:HGNC:7799] | 38410 | -0.422 | -0.1333 | Yes |
| 71 | LYN | "LYN proto-oncogene, Src family tyrosine kinase [Source:HGNC Symbol;Acc:HGNC:6735]" | 38449 | -0.435 | -0.1045 | Yes |
| 72 | BCL10 | BCL10 immune signaling adaptor [Source:HGNC Symbol;Acc:HGNC:989] | 38451 | -0.436 | -0.0747 | Yes |
| 73 | NFKB1 | nuclear factor kappa B subunit 1 [Source:HGNC Symbol;Acc:HGNC:7794] | 38470 | -0.443 | -0.0449 | Yes |
| 74 | RELA | "RELA proto-oncogene, NF-kB subunit [Source:HGNC Symbol;Acc:HGNC:9955]" | 38475 | -0.445 | -0.0145 | Yes |
| 75 | SOS2 | SOS Ras/Rho guanine nucleotide exchange factor 2 [Source:HGNC Symbol;Acc:HGNC:11188] | 38581 | -0.528 | 0.0189 | Yes |
Table: GSEA details [plain text format]

  

Fig 2: KEGG\_B\_CELL\_RECEPTOR\_SIGNALING\_PATHWAY      
 Blue-Pink O' Gram in the Space of the Analyzed GeneSet

  

Fig 3: KEGG\_B\_CELL\_RECEPTOR\_SIGNALING\_PATHWAY: Random ES distribution      
 Gene set null distribution of ES for **KEGG\_B\_CELL\_RECEPTOR\_SIGNALING\_PATHWAY**
