# Supplementary material for: Simultaneous Presentation of Multiple Myeloma and Lung Cancer: Case Report and Gene Bioinformatics Analysis
Source: Front Oncol. 2022 Jun 13;12:859735. doi: 10.3389/fonc.2022.859735 (PMC9235397; doi:10.3389/fonc.2022.859735)
Supplement: Supplementary file 1 [file DataSheet_1.zip › The bioinformatic analysis of MM and lung cancer supplementary materials/Enrichment analysis/MECR/GSEA_4.1.0/MM TCGA/KEGG.Gsea.1639040591792/KEGG_CHEMOKINE_SIGNALING_PATHWAY.html]

Details for gene set KEGG\_CHEMOKINE\_SIGNALING\_PATHWAY[GSEA]

|  || Dataset | ExpData\_collapsed\_to\_symbols.ENSG00000116353\_profile\_in\_ExpData.cls #ENSG00000116353 |
| Phenotype | ENSG00000116353\_profile\_in\_ExpData.cls#ENSG00000116353 |
| Upregulated in class | ENSG00000116353\_neg |
| GeneSet | KEGG\_CHEMOKINE\_SIGNALING\_PATHWAY |
| Enrichment Score (ES) | -0.5816844 |
| Normalized Enrichment Score (NES) | -2.5415602 |
| Nominal p-value | 0.0 |
| FDR q-value | 0.0 |
| FWER p-Value | 0.0 |
Table: GSEA Results Summary

  

Fig 1: Enrichment plot: KEGG\_CHEMOKINE\_SIGNALING\_PATHWAY      
 Profile of the Running ES Score & Positions of GeneSet Members on the Rank Ordered List

  

| SYMBOL | TITLE | RANK IN GENE LIST | RANK METRIC SCORE | RUNNING ES | CORE ENRICHMENT || 1 | PIK3R2 | phosphoinositide-3-kinase regulatory subunit 2 [Source:HGNC Symbol;Acc:HGNC:8980] | 1248 | 0.320 | -0.0204 | No |
| 2 | CCR2 | C-C motif chemokine receptor 2 [Source:HGNC Symbol;Acc:HGNC:1603] | 1977 | 0.265 | -0.0295 | No |
| 3 | SHC4 | SHC adaptor protein 4 [Source:HGNC Symbol;Acc:HGNC:16743] | 2178 | 0.254 | -0.0256 | No |
| 4 | PIK3R3 | phosphoinositide-3-kinase regulatory subunit 3 [Source:HGNC Symbol;Acc:HGNC:8981] | 2602 | 0.231 | -0.0281 | No |
| 5 | PLCB1 | phospholipase C beta 1 [Source:HGNC Symbol;Acc:HGNC:15917] | 2881 | 0.219 | -0.0274 | No |
| 6 | CSK | C-terminal Src kinase [Source:HGNC Symbol;Acc:HGNC:2444] | 3052 | 0.211 | -0.0242 | No |
| 7 | ADCY5 | adenylate cyclase 5 [Source:HGNC Symbol;Acc:HGNC:236] | 3504 | 0.193 | -0.0288 | No |
| 8 | VAV1 | vav guanine nucleotide exchange factor 1 [Source:HGNC Symbol;Acc:HGNC:12657] | 3764 | 0.184 | -0.0289 | No |
| 9 | PLCB4 | phospholipase C beta 4 [Source:HGNC Symbol;Acc:HGNC:9059] | 3842 | 0.182 | -0.0243 | No |
| 10 | XCR1 | X-C motif chemokine receptor 1 [Source:HGNC Symbol;Acc:HGNC:1625] | 3998 | 0.177 | -0.0219 | No |
| 11 | CX3CR1 | C-X3-C motif chemokine receptor 1 [Source:HGNC Symbol;Acc:HGNC:2558] | 5281 | 0.142 | -0.0496 | No |
| 12 | SHC3 | SHC adaptor protein 3 [Source:HGNC Symbol;Acc:HGNC:18181] | 5437 | 0.138 | -0.0486 | No |
| 13 | GRK6 | G protein-coupled receptor kinase 6 [Source:HGNC Symbol;Acc:HGNC:4545] | 5814 | 0.130 | -0.0536 | No |
| 14 | RAC2 | Rac family small GTPase 2 [Source:HGNC Symbol;Acc:HGNC:9802] | 6127 | 0.124 | -0.0571 | No |
| 15 | GNG13 | G protein subunit gamma 13 [Source:HGNC Symbol;Acc:HGNC:14131] | 6208 | 0.123 | -0.0548 | No |
| 16 | CXCL13 | C-X-C motif chemokine ligand 13 [Source:HGNC Symbol;Acc:HGNC:10639] | 6476 | 0.118 | -0.0573 | No |
| 17 | ADCY3 | adenylate cyclase 3 [Source:HGNC Symbol;Acc:HGNC:234] | 6745 | 0.114 | -0.0601 | No |
| 18 | SHC1 | SHC adaptor protein 1 [Source:HGNC Symbol;Acc:HGNC:10840] | 7103 | 0.108 | -0.0654 | No |
| 19 | PIK3CB | "phosphatidylinositol-4,5-bisphosphate 3-kinase catalytic subunit beta [Source:HGNC Symbol;Acc:HGNC:8976]" | 7716 | 0.099 | -0.0775 | No |
| 20 | GRK1 | G protein-coupled receptor kinase 1 [Source:HGNC Symbol;Acc:HGNC:10013] | 8060 | 0.094 | -0.0829 | No |
| 21 | CXCR6 | C-X-C motif chemokine receptor 6 [Source:HGNC Symbol;Acc:HGNC:16647] | 8198 | 0.092 | -0.0831 | No |
| 22 | ADCY1 | adenylate cyclase 1 [Source:HGNC Symbol;Acc:HGNC:232] | 8649 | 0.087 | -0.0915 | No |
| 23 | CCR3 | C-C motif chemokine receptor 3 [Source:HGNC Symbol;Acc:HGNC:1604] | 8776 | 0.085 | -0.0917 | No |
| 24 | GNGT1 | G protein subunit gamma transducin 1 [Source:HGNC Symbol;Acc:HGNC:4411] | 8957 | 0.083 | -0.0933 | No |
| 25 | CCR10 | C-C motif chemokine receptor 10 [Source:HGNC Symbol;Acc:HGNC:4474] | 8977 | 0.083 | -0.0908 | No |
| 26 | CCL1 | C-C motif chemokine ligand 1 [Source:HGNC Symbol;Acc:HGNC:10609] | 9329 | 0.078 | -0.0970 | No |
| 27 | CHUK | component of inhibitor of nuclear factor kappa B kinase complex [Source:HGNC Symbol;Acc:HGNC:1974] | 9925 | 0.071 | -0.1097 | No |
| 28 | JAK3 | Janus kinase 3 [Source:HGNC Symbol;Acc:HGNC:6193] | 9974 | 0.071 | -0.1084 | No |
| 29 | STAT1 | signal transducer and activator of transcription 1 [Source:HGNC Symbol;Acc:HGNC:11362] | 10873 | 0.062 | -0.1291 | No |
| 30 | PRKCB | protein kinase C beta [Source:HGNC Symbol;Acc:HGNC:9395] | 11760 | 0.054 | -0.1498 | No |
| 31 | GSK3B | glycogen synthase kinase 3 beta [Source:HGNC Symbol;Acc:HGNC:4617] | 12189 | 0.051 | -0.1589 | No |
| 32 | PRKACG | protein kinase cAMP-activated catalytic subunit gamma [Source:HGNC Symbol;Acc:HGNC:9382] | 12813 | 0.046 | -0.1732 | No |
| 33 | PRKCZ | protein kinase C zeta [Source:HGNC Symbol;Acc:HGNC:9412] | 14450 | 0.035 | -0.2137 | No |
| 34 | PRKACA | protein kinase cAMP-activated catalytic subunit alpha [Source:HGNC Symbol;Acc:HGNC:9380] | 14554 | 0.035 | -0.2151 | No |
| 35 | CRK | "CRK proto-oncogene, adaptor protein [Source:HGNC Symbol;Acc:HGNC:2362]" | 14915 | 0.032 | -0.2231 | No |
| 36 | PRKACB | protein kinase cAMP-activated catalytic subunit beta [Source:HGNC Symbol;Acc:HGNC:9381] | 15303 | 0.030 | -0.2319 | No |
| 37 | CCL25 | C-C motif chemokine ligand 25 [Source:HGNC Symbol;Acc:HGNC:10624] | 15877 | 0.026 | -0.2456 | No |
| 38 | XCL1 | X-C motif chemokine ligand 1 [Source:HGNC Symbol;Acc:HGNC:10645] | 15981 | 0.026 | -0.2473 | No |
| 39 | STAT2 | signal transducer and activator of transcription 2 [Source:HGNC Symbol;Acc:HGNC:11363] | 17153 | 0.019 | -0.2766 | No |
| 40 | GNB4 | G protein subunit beta 4 [Source:HGNC Symbol;Acc:HGNC:20731] | 17187 | 0.019 | -0.2767 | No |
| 41 | PRKCD | protein kinase C delta [Source:HGNC Symbol;Acc:HGNC:9399] | 17659 | 0.016 | -0.2882 | No |
| 42 | XCL2 | X-C motif chemokine ligand 2 [Source:HGNC Symbol;Acc:HGNC:10646] | 17789 | 0.016 | -0.2909 | No |
| 43 | WAS | WASP actin nucleation promoting factor [Source:HGNC Symbol;Acc:HGNC:12731] | 17936 | 0.015 | -0.2941 | No |
| 44 | CCR1 | C-C motif chemokine receptor 1 [Source:HGNC Symbol;Acc:HGNC:1602] | 17985 | 0.015 | -0.2948 | No |
| 45 | ELMO1 | engulfment and cell motility 1 [Source:HGNC Symbol;Acc:HGNC:16286] | 18104 | 0.014 | -0.2973 | No |
| 46 | SOS1 | SOS Ras/Rac guanine nucleotide exchange factor 1 [Source:HGNC Symbol;Acc:HGNC:11187] | 18532 | 0.012 | -0.3078 | No |
| 47 | CCR8 | C-C motif chemokine receptor 8 [Source:HGNC Symbol;Acc:HGNC:1609] | 19552 | 0.006 | -0.3337 | No |
| 48 | GNG3 | G protein subunit gamma 3 [Source:HGNC Symbol;Acc:HGNC:4405] | 19942 | 0.004 | -0.3435 | No |
| 49 | PTK2 | protein tyrosine kinase 2 [Source:HGNC Symbol;Acc:HGNC:9611] | 20287 | 0.002 | -0.3522 | No |
| 50 | IKBKB | inhibitor of nuclear factor kappa B kinase subunit beta [Source:HGNC Symbol;Acc:HGNC:5960] | 20718 | -0.000 | -0.3631 | No |
| 51 | GNB3 | G protein subunit beta 3 [Source:HGNC Symbol;Acc:HGNC:4400] | 20844 | -0.001 | -0.3663 | No |
| 52 | GRK4 | G protein-coupled receptor kinase 4 [Source:HGNC Symbol;Acc:HGNC:4543] | 21101 | -0.003 | -0.3727 | No |
| 53 | ADCY2 | adenylate cyclase 2 [Source:HGNC Symbol;Acc:HGNC:233] | 21361 | -0.004 | -0.3792 | No |
| 54 | ADCY7 | adenylate cyclase 7 [Source:HGNC Symbol;Acc:HGNC:238] | 21812 | -0.007 | -0.3905 | No |
| 55 | PRKX | protein kinase X-linked [Source:HGNC Symbol;Acc:HGNC:9441] | 23135 | -0.015 | -0.4237 | No |
| 56 | MAPK1 | mitogen-activated protein kinase 1 [Source:HGNC Symbol;Acc:HGNC:6871] | 23167 | -0.015 | -0.4240 | No |
| 57 | GNG4 | G protein subunit gamma 4 [Source:HGNC Symbol;Acc:HGNC:4407] | 23600 | -0.017 | -0.4344 | No |
| 58 | CCR5 | C-C motif chemokine receptor 5 [Source:HGNC Symbol;Acc:HGNC:1606] | 23927 | -0.020 | -0.4420 | No |
| 59 | ROCK2 | Rho associated coiled-coil containing protein kinase 2 [Source:HGNC Symbol;Acc:HGNC:10252] | 24395 | -0.022 | -0.4532 | No |
| 60 | GNG11 | G protein subunit gamma 11 [Source:HGNC Symbol;Acc:HGNC:4403] | 25149 | -0.027 | -0.4714 | No |
| 61 | CCL21 | C-C motif chemokine ligand 21 [Source:HGNC Symbol;Acc:HGNC:10620] | 25690 | -0.031 | -0.4841 | No |
| 62 | PPBPP1 | pro-platelet basic protein pseudogene 1 [Source:HGNC Symbol;Acc:HGNC:9241] | 26473 | -0.037 | -0.5028 | No |
| 63 | DOCK2 | dedicator of cytokinesis 2 [Source:HGNC Symbol;Acc:HGNC:2988] | 26543 | -0.038 | -0.5032 | No |
| 64 | HRAS | "HRas proto-oncogene, GTPase [Source:HGNC Symbol;Acc:HGNC:5173]" | 26672 | -0.039 | -0.5051 | No |
| 65 | CCL11 | C-C motif chemokine ligand 11 [Source:HGNC Symbol;Acc:HGNC:10610] | 26778 | -0.039 | -0.5063 | No |
| 66 | CCL22 | C-C motif chemokine ligand 22 [Source:HGNC Symbol;Acc:HGNC:10621] | 27090 | -0.042 | -0.5128 | No |
| 67 | ADCY8 | adenylate cyclase 8 [Source:HGNC Symbol;Acc:HGNC:239] | 27844 | -0.049 | -0.5303 | No |
| 68 | PREX1 | "phosphatidylinositol-3,4,5-trisphosphate dependent Rac exchange factor 1 [Source:HGNC Symbol;Acc:HGNC:32594]" | 27961 | -0.050 | -0.5315 | No |
| 69 | GNGT2 | G protein subunit gamma transducin 2 [Source:HGNC Symbol;Acc:HGNC:4412] | 28010 | -0.050 | -0.5309 | No |
| 70 | CCL16 | C-C motif chemokine ligand 16 [Source:HGNC Symbol;Acc:HGNC:10614] | 28287 | -0.053 | -0.5361 | No |
| 71 | ADCY9 | adenylate cyclase 9 [Source:HGNC Symbol;Acc:HGNC:240] | 28450 | -0.054 | -0.5383 | No |
| 72 | GSK3A | glycogen synthase kinase 3 alpha [Source:HGNC Symbol;Acc:HGNC:4616] | 28647 | -0.056 | -0.5412 | No |
| 73 | CCR9 | C-C motif chemokine receptor 9 [Source:HGNC Symbol;Acc:HGNC:1610] | 28783 | -0.058 | -0.5426 | No |
| 74 | FOXO3 | forkhead box O3 [Source:HGNC Symbol;Acc:HGNC:3821] | 29077 | -0.061 | -0.5479 | No |
| 75 | CCL8 | C-C motif chemokine ligand 8 [Source:HGNC Symbol;Acc:HGNC:10635] | 29326 | -0.064 | -0.5519 | No |
| 76 | VAV3 | vav guanine nucleotide exchange factor 3 [Source:HGNC Symbol;Acc:HGNC:12659] | 29349 | -0.065 | -0.5502 | No |
| 77 | CCL13 | C-C motif chemokine ligand 13 [Source:HGNC Symbol;Acc:HGNC:10611] | 29378 | -0.065 | -0.5486 | No |
| 78 | ADCY6 | adenylate cyclase 6 [Source:HGNC Symbol;Acc:HGNC:237] | 29551 | -0.067 | -0.5506 | No |
| 79 | CXCR3 | C-X-C motif chemokine receptor 3 [Source:HGNC Symbol;Acc:HGNC:4540] | 29724 | -0.069 | -0.5525 | No |
| 80 | PARD3 | par-3 family cell polarity regulator [Source:HGNC Symbol;Acc:HGNC:16051] | 29993 | -0.072 | -0.5568 | No |
| 81 | CCL27 | C-C motif chemokine ligand 27 [Source:HGNC Symbol;Acc:HGNC:10626] | 30627 | -0.080 | -0.5701 | No |
| 82 | CCL17 | C-C motif chemokine ligand 17 [Source:HGNC Symbol;Acc:HGNC:10615] | 30976 | -0.084 | -0.5760 | No |
| 83 | TIAM2 | TIAM Rac1 associated GEF 2 [Source:HGNC Symbol;Acc:HGNC:11806] | 31199 | -0.088 | -0.5786 | Yes |
| 84 | GNAI1 | G protein subunit alpha i1 [Source:HGNC Symbol;Acc:HGNC:4384] | 31213 | -0.088 | -0.5757 | Yes |
| 85 | CXCR2 | C-X-C motif chemokine receptor 2 [Source:HGNC Symbol;Acc:HGNC:6027] | 31411 | -0.090 | -0.5775 | Yes |
| 86 | PLCB3 | phospholipase C beta 3 [Source:HGNC Symbol;Acc:HGNC:9056] | 31486 | -0.091 | -0.5762 | Yes |
| 87 | CCL24 | C-C motif chemokine ligand 24 [Source:HGNC Symbol;Acc:HGNC:10623] | 31647 | -0.094 | -0.5769 | Yes |
| 88 | CXCL9 | C-X-C motif chemokine ligand 9 [Source:HGNC Symbol;Acc:HGNC:7098] | 31652 | -0.094 | -0.5736 | Yes |
| 89 | CCL19 | C-C motif chemokine ligand 19 [Source:HGNC Symbol;Acc:HGNC:10617] | 31855 | -0.097 | -0.5753 | Yes |
| 90 | AKT1 | AKT serine/threonine kinase 1 [Source:HGNC Symbol;Acc:HGNC:391] | 31906 | -0.098 | -0.5731 | Yes |
| 91 | CCL15 | C-C motif chemokine ligand 15 [Source:HGNC Symbol;Acc:HGNC:10613] | 31927 | -0.099 | -0.5701 | Yes |
| 92 | CCR6 | C-C motif chemokine receptor 6 [Source:HGNC Symbol;Acc:HGNC:1607] | 32052 | -0.101 | -0.5696 | Yes |
| 93 | IKBKG | inhibitor of nuclear factor kappa B kinase regulatory subunit gamma [Source:HGNC Symbol;Acc:HGNC:5961] | 32205 | -0.103 | -0.5698 | Yes |
| 94 | CCL18 | C-C motif chemokine ligand 18 [Source:HGNC Symbol;Acc:HGNC:10616] | 32213 | -0.103 | -0.5663 | Yes |
| 95 | GNB5 | G protein subunit beta 5 [Source:HGNC Symbol;Acc:HGNC:4401] | 32231 | -0.104 | -0.5631 | Yes |
| 96 | GNAI3 | G protein subunit alpha i3 [Source:HGNC Symbol;Acc:HGNC:4387] | 32286 | -0.105 | -0.5607 | Yes |
| 97 | CCL28 | C-C motif chemokine ligand 28 [Source:HGNC Symbol;Acc:HGNC:17700] | 32346 | -0.106 | -0.5584 | Yes |
| 98 | STAT5B | signal transducer and activator of transcription 5B [Source:HGNC Symbol;Acc:HGNC:11367] | 32360 | -0.106 | -0.5549 | Yes |
| 99 | GNG7 | G protein subunit gamma 7 [Source:HGNC Symbol;Acc:HGNC:4410] | 32399 | -0.107 | -0.5521 | Yes |
| 100 | GRK7 | G protein-coupled receptor kinase 7 [Source:HGNC Symbol;Acc:HGNC:17031] | 32503 | -0.109 | -0.5508 | Yes |
| 101 | CCL4L2 | C-C motif chemokine ligand 4 like 2 [Source:HGNC Symbol;Acc:HGNC:24066] | 32867 | -0.116 | -0.5559 | Yes |
| 102 | PTK2B | protein tyrosine kinase 2 beta [Source:HGNC Symbol;Acc:HGNC:9612] | 32991 | -0.119 | -0.5548 | Yes |
| 103 | AKT2 | AKT serine/threonine kinase 2 [Source:HGNC Symbol;Acc:HGNC:392] | 33213 | -0.123 | -0.5561 | Yes |
| 104 | CXCR5 | C-X-C motif chemokine receptor 5 [Source:HGNC Symbol;Acc:HGNC:1060] | 33366 | -0.126 | -0.5554 | Yes |
| 105 | CXCL10 | C-X-C motif chemokine ligand 10 [Source:HGNC Symbol;Acc:HGNC:10637] | 33451 | -0.128 | -0.5530 | Yes |
| 106 | GNG12 | G protein subunit gamma 12 [Source:HGNC Symbol;Acc:HGNC:19663] | 33587 | -0.131 | -0.5518 | Yes |
| 107 | CXCL11 | C-X-C motif chemokine ligand 11 [Source:HGNC Symbol;Acc:HGNC:10638] | 33604 | -0.131 | -0.5475 | Yes |
| 108 | CCL26 | C-C motif chemokine ligand 26 [Source:HGNC Symbol;Acc:HGNC:10625] | 33627 | -0.132 | -0.5433 | Yes |
| 109 | NRAS | "NRAS proto-oncogene, GTPase [Source:HGNC Symbol;Acc:HGNC:7989]" | 33739 | -0.135 | -0.5413 | Yes |
| 110 | CX3CL1 | C-X3-C motif chemokine ligand 1 [Source:HGNC Symbol;Acc:HGNC:10647] | 33763 | -0.135 | -0.5371 | Yes |
| 111 | CCL3 | C-C motif chemokine ligand 3 [Source:HGNC Symbol;Acc:HGNC:10627] | 33771 | -0.136 | -0.5324 | Yes |
| 112 | PIK3CD | "phosphatidylinositol-4,5-bisphosphate 3-kinase catalytic subunit delta [Source:HGNC Symbol;Acc:HGNC:8977]" | 33794 | -0.137 | -0.5281 | Yes |
| 113 | PAK1 | p21 (RAC1) activated kinase 1 [Source:HGNC Symbol;Acc:HGNC:8590] | 33819 | -0.137 | -0.5238 | Yes |
| 114 | HCK | "HCK proto-oncogene, Src family tyrosine kinase [Source:HGNC Symbol;Acc:HGNC:4840]" | 33886 | -0.139 | -0.5205 | Yes |
| 115 | JAK2 | Janus kinase 2 [Source:HGNC Symbol;Acc:HGNC:6192] | 33960 | -0.141 | -0.5174 | Yes |
| 116 | CCL4 | C-C motif chemokine ligand 4 [Source:HGNC Symbol;Acc:HGNC:10630] | 34162 | -0.145 | -0.5173 | Yes |
| 117 | VAV2 | vav guanine nucleotide exchange factor 2 [Source:HGNC Symbol;Acc:HGNC:12658] | 34251 | -0.148 | -0.5142 | Yes |
| 118 | GNB1 | G protein subunit beta 1 [Source:HGNC Symbol;Acc:HGNC:4396] | 34290 | -0.149 | -0.5099 | Yes |
| 119 | GNG10 | G protein subunit gamma 10 [Source:HGNC Symbol;Acc:HGNC:4402] | 34330 | -0.150 | -0.5055 | Yes |
| 120 | CRKL | "CRK like proto-oncogene, adaptor protein [Source:HGNC Symbol;Acc:HGNC:2363]" | 34457 | -0.154 | -0.5032 | Yes |
| 121 | AKT3 | AKT serine/threonine kinase 3 [Source:HGNC Symbol;Acc:HGNC:393] | 34501 | -0.156 | -0.4987 | Yes |
| 122 | SHC2 | SHC adaptor protein 2 [Source:HGNC Symbol;Acc:HGNC:29869] | 34623 | -0.160 | -0.4961 | Yes |
| 123 | CXCL3 | C-X-C motif chemokine ligand 3 [Source:HGNC Symbol;Acc:HGNC:4604] | 34653 | -0.161 | -0.4911 | Yes |
| 124 | GRK3 | G protein-coupled receptor kinase 3 [Source:HGNC Symbol;Acc:HGNC:290] | 34666 | -0.161 | -0.4857 | Yes |
| 125 | STAT3 | signal transducer and activator of transcription 3 [Source:HGNC Symbol;Acc:HGNC:11364] | 34682 | -0.161 | -0.4803 | Yes |
| 126 | CXCR1 | C-X-C motif chemokine receptor 1 [Source:HGNC Symbol;Acc:HGNC:6026] | 34719 | -0.162 | -0.4754 | Yes |
| 127 | GRK5 | G protein-coupled receptor kinase 5 [Source:HGNC Symbol;Acc:HGNC:4544] | 34721 | -0.162 | -0.4696 | Yes |
| 128 | CCL5 | C-C motif chemokine ligand 5 [Source:HGNC Symbol;Acc:HGNC:10632] | 34763 | -0.164 | -0.4648 | Yes |
| 129 | CXCL6 | C-X-C motif chemokine ligand 6 [Source:HGNC Symbol;Acc:HGNC:10643] | 34787 | -0.164 | -0.4595 | Yes |
| 130 | ITK | IL2 inducible T cell kinase [Source:HGNC Symbol;Acc:HGNC:6171] | 34994 | -0.170 | -0.4587 | Yes |
| 131 | ARRB1 | arrestin beta 1 [Source:HGNC Symbol;Acc:HGNC:711] | 34997 | -0.171 | -0.4526 | Yes |
| 132 | CCL2 | C-C motif chemokine ligand 2 [Source:HGNC Symbol;Acc:HGNC:10618] | 35124 | -0.175 | -0.4496 | Yes |
| 133 | CCL7 | C-C motif chemokine ligand 7 [Source:HGNC Symbol;Acc:HGNC:10634] | 35132 | -0.175 | -0.4435 | Yes |
| 134 | CCL23 | C-C motif chemokine ligand 23 [Source:HGNC Symbol;Acc:HGNC:10622] | 35309 | -0.181 | -0.4415 | Yes |
| 135 | GNG8 | G protein subunit gamma 8 [Source:HGNC Symbol;Acc:HGNC:19664] | 35347 | -0.183 | -0.4359 | Yes |
| 136 | PXN | paxillin [Source:HGNC Symbol;Acc:HGNC:9718] | 35502 | -0.189 | -0.4331 | Yes |
| 137 | ARRB2 | arrestin beta 2 [Source:HGNC Symbol;Acc:HGNC:712] | 35527 | -0.189 | -0.4270 | Yes |
| 138 | MAPK3 | mitogen-activated protein kinase 3 [Source:HGNC Symbol;Acc:HGNC:6877] | 35579 | -0.191 | -0.4215 | Yes |
| 139 | CCL14 | C-C motif chemokine ligand 14 [Source:HGNC Symbol;Acc:HGNC:10612] | 35687 | -0.195 | -0.4172 | Yes |
| 140 | CCR4 | C-C motif chemokine receptor 4 [Source:HGNC Symbol;Acc:HGNC:1605] | 35765 | -0.198 | -0.4121 | Yes |
| 141 | PF4V1 | platelet factor 4 variant 1 [Source:HGNC Symbol;Acc:HGNC:8862] | 35781 | -0.199 | -0.4054 | Yes |
| 142 | GNG5 | G protein subunit gamma 5 [Source:HGNC Symbol;Acc:HGNC:4408] | 35799 | -0.199 | -0.3987 | Yes |
| 143 | CXCL14 | C-X-C motif chemokine ligand 14 [Source:HGNC Symbol;Acc:HGNC:10640] | 35832 | -0.201 | -0.3923 | Yes |
| 144 | BCAR1 | "BCAR1 scaffold protein, Cas family member [Source:HGNC Symbol;Acc:HGNC:971]" | 35834 | -0.201 | -0.3852 | Yes |
| 145 | CCL20 | C-C motif chemokine ligand 20 [Source:HGNC Symbol;Acc:HGNC:10619] | 35867 | -0.202 | -0.3787 | Yes |
| 146 | MAP2K1 | mitogen-activated protein kinase kinase 1 [Source:HGNC Symbol;Acc:HGNC:6840] | 36024 | -0.208 | -0.3753 | Yes |
| 147 | PF4 | platelet factor 4 [Source:HGNC Symbol;Acc:HGNC:8861] | 36067 | -0.210 | -0.3688 | Yes |
| 148 | GNAI2 | G protein subunit alpha i2 [Source:HGNC Symbol;Acc:HGNC:4385] | 36074 | -0.210 | -0.3615 | Yes |
| 149 | CCL3L3 | C-C motif chemokine ligand 3 like 3 [Source:HGNC Symbol;Acc:HGNC:30554] | 36178 | -0.215 | -0.3564 | Yes |
| 150 | PIK3CG | "phosphatidylinositol-4,5-bisphosphate 3-kinase catalytic subunit gamma [Source:HGNC Symbol;Acc:HGNC:8978]" | 36243 | -0.217 | -0.3503 | Yes |
| 151 | NCF1 | neutrophil cytosolic factor 1 [Source:HGNC Symbol;Acc:HGNC:7660] | 36267 | -0.218 | -0.3431 | Yes |
| 152 | GNG2 | G protein subunit gamma 2 [Source:HGNC Symbol;Acc:HGNC:4404] | 36290 | -0.219 | -0.3358 | Yes |
| 153 | CXCL12 | C-X-C motif chemokine ligand 12 [Source:HGNC Symbol;Acc:HGNC:10672] | 36344 | -0.222 | -0.3292 | Yes |
| 154 | TIAM1 | TIAM Rac1 associated GEF 1 [Source:HGNC Symbol;Acc:HGNC:11805] | 36355 | -0.223 | -0.3215 | Yes |
| 155 | PPBP | pro-platelet basic protein [Source:HGNC Symbol;Acc:HGNC:9240] | 36358 | -0.223 | -0.3135 | Yes |
| 156 | CXCL1 | C-X-C motif chemokine ligand 1 [Source:HGNC Symbol;Acc:HGNC:4602] | 36378 | -0.224 | -0.3060 | Yes |
| 157 | GRB2 | growth factor receptor bound protein 2 [Source:HGNC Symbol;Acc:HGNC:4566] | 36523 | -0.231 | -0.3014 | Yes |
| 158 | FGR | "FGR proto-oncogene, Src family tyrosine kinase [Source:HGNC Symbol;Acc:HGNC:3697]" | 36769 | -0.245 | -0.2989 | Yes |
| 159 | CXCR4 | C-X-C motif chemokine receptor 4 [Source:HGNC Symbol;Acc:HGNC:2561] | 36861 | -0.251 | -0.2922 | Yes |
| 160 | CXCL2 | C-X-C motif chemokine ligand 2 [Source:HGNC Symbol;Acc:HGNC:4603] | 36954 | -0.257 | -0.2854 | Yes |
| 161 | PLCB2 | phospholipase C beta 2 [Source:HGNC Symbol;Acc:HGNC:9055] | 36982 | -0.259 | -0.2768 | Yes |
| 162 | RASGRP2 | RAS guanyl releasing protein 2 [Source:HGNC Symbol;Acc:HGNC:9879] | 37170 | -0.270 | -0.2720 | Yes |
| 163 | PIK3R1 | phosphoinositide-3-kinase regulatory subunit 1 [Source:HGNC Symbol;Acc:HGNC:8979] | 37172 | -0.270 | -0.2623 | Yes |
| 164 | GNB2 | G protein subunit beta 2 [Source:HGNC Symbol;Acc:HGNC:4398] | 37173 | -0.270 | -0.2526 | Yes |
| 165 | NFKBIA | NFKB inhibitor alpha [Source:HGNC Symbol;Acc:HGNC:7797] | 37222 | -0.275 | -0.2440 | Yes |
| 166 | RAC1 | Rac family small GTPase 1 [Source:HGNC Symbol;Acc:HGNC:9801] | 37263 | -0.277 | -0.2351 | Yes |
| 167 | RAP1A | "RAP1A, member of RAS oncogene family [Source:HGNC Symbol;Acc:HGNC:9855]" | 37388 | -0.287 | -0.2281 | Yes |
| 168 | CXCL5 | C-X-C motif chemokine ligand 5 [Source:HGNC Symbol;Acc:HGNC:10642] | 37407 | -0.288 | -0.2182 | Yes |
| 169 | RHOA | ras homolog family member A [Source:HGNC Symbol;Acc:HGNC:667] | 37417 | -0.290 | -0.2081 | Yes |
| 170 | ADCY4 | adenylate cyclase 4 [Source:HGNC Symbol;Acc:HGNC:235] | 37426 | -0.290 | -0.1979 | Yes |
| 171 | PIK3R5 | phosphoinositide-3-kinase regulatory subunit 5 [Source:HGNC Symbol;Acc:HGNC:30035] | 37628 | -0.307 | -0.1920 | Yes |
| 172 | CXCL8 | C-X-C motif chemokine ligand 8 [Source:HGNC Symbol;Acc:HGNC:6025] | 37687 | -0.313 | -0.1823 | Yes |
| 173 | CXCL16 | C-X-C motif chemokine ligand 16 [Source:HGNC Symbol;Acc:HGNC:16642] | 37745 | -0.318 | -0.1724 | Yes |
| 174 | KRAS | "KRAS proto-oncogene, GTPase [Source:HGNC Symbol;Acc:HGNC:6407]" | 37789 | -0.323 | -0.1620 | Yes |
| 175 | CDC42 | cell division cycle 42 [Source:HGNC Symbol;Acc:HGNC:1736] | 37805 | -0.324 | -0.1508 | Yes |
| 176 | RAF1 | "Raf-1 proto-oncogene, serine/threonine kinase [Source:HGNC Symbol;Acc:HGNC:9829]" | 37807 | -0.325 | -0.1392 | Yes |
| 177 | BRAF | "B-Raf proto-oncogene, serine/threonine kinase [Source:HGNC Symbol;Acc:HGNC:1097]" | 37844 | -0.330 | -0.1283 | Yes |
| 178 | GRK2 | G protein-coupled receptor kinase 2 [Source:HGNC Symbol;Acc:HGNC:289] | 37914 | -0.338 | -0.1179 | Yes |
| 179 | NFKBIB | NFKB inhibitor beta [Source:HGNC Symbol;Acc:HGNC:7798] | 37987 | -0.348 | -0.1073 | Yes |
| 180 | CCR7 | C-C motif chemokine receptor 7 [Source:HGNC Symbol;Acc:HGNC:1608] | 38087 | -0.360 | -0.0970 | Yes |
| 181 | PIK3CA | "phosphatidylinositol-4,5-bisphosphate 3-kinase catalytic subunit alpha [Source:HGNC Symbol;Acc:HGNC:8975]" | 38268 | -0.391 | -0.0876 | Yes |
| 182 | WASL | WASP like actin nucleation promoting factor [Source:HGNC Symbol;Acc:HGNC:12735] | 38341 | -0.407 | -0.0749 | Yes |
| 183 | RAP1B | "RAP1B, member of RAS oncogene family [Source:HGNC Symbol;Acc:HGNC:9857]" | 38403 | -0.421 | -0.0614 | Yes |
| 184 | LYN | "LYN proto-oncogene, Src family tyrosine kinase [Source:HGNC Symbol;Acc:HGNC:6735]" | 38449 | -0.435 | -0.0470 | Yes |
| 185 | NFKB1 | nuclear factor kappa B subunit 1 [Source:HGNC Symbol;Acc:HGNC:7794] | 38470 | -0.443 | -0.0316 | Yes |
| 186 | RELA | "RELA proto-oncogene, NF-kB subunit [Source:HGNC Symbol;Acc:HGNC:9955]" | 38475 | -0.445 | -0.0158 | Yes |
| 187 | ROCK1 | Rho associated coiled-coil containing protein kinase 1 [Source:HGNC Symbol;Acc:HGNC:10251] | 38575 | -0.519 | 0.0002 | Yes |
| 188 | SOS2 | SOS Ras/Rho guanine nucleotide exchange factor 2 [Source:HGNC Symbol;Acc:HGNC:11188] | 38581 | -0.528 | 0.0190 | Yes |
Table: GSEA details [plain text format]

  

Fig 2: KEGG\_CHEMOKINE\_SIGNALING\_PATHWAY      
 Blue-Pink O' Gram in the Space of the Analyzed GeneSet

  

Fig 3: KEGG\_CHEMOKINE\_SIGNALING\_PATHWAY: Random ES distribution      
 Gene set null distribution of ES for **KEGG\_CHEMOKINE\_SIGNALING\_PATHWAY**
