# Supplementary material for: Simultaneous Presentation of Multiple Myeloma and Lung Cancer: Case Report and Gene Bioinformatics Analysis
Source: Front Oncol. 2022 Jun 13;12:859735. doi: 10.3389/fonc.2022.859735 (PMC9235397; doi:10.3389/fonc.2022.859735)
Supplement: Supplementary file 1 [file DataSheet_1.zip › The bioinformatic analysis of MM and lung cancer supplementary materials/Enrichment analysis/MECR/GSEA_4.1.0/MM TCGA/KEGG.Gsea.1639040591792/KEGG_CITRATE_CYCLE_TCA_CYCLE.html]

Details for gene set KEGG\_CITRATE\_CYCLE\_TCA\_CYCLE[GSEA]

|  || Dataset | ExpData\_collapsed\_to\_symbols.ENSG00000116353\_profile\_in\_ExpData.cls #ENSG00000116353 |
| Phenotype | ENSG00000116353\_profile\_in\_ExpData.cls#ENSG00000116353 |
| Upregulated in class | ENSG00000116353\_pos |
| GeneSet | KEGG\_CITRATE\_CYCLE\_TCA\_CYCLE |
| Enrichment Score (ES) | 0.7420885 |
| Normalized Enrichment Score (NES) | 2.1980138 |
| Nominal p-value | 0.0 |
| FDR q-value | 0.0 |
| FWER p-Value | 0.0 |
Table: GSEA Results Summary

  

Fig 1: Enrichment plot: KEGG\_CITRATE\_CYCLE\_TCA\_CYCLE      
 Profile of the Running ES Score & Positions of GeneSet Members on the Rank Ordered List

  

| SYMBOL | TITLE | RANK IN GENE LIST | RANK METRIC SCORE | RUNNING ES | CORE ENRICHMENT || 1 | FH | fumarate hydratase [Source:HGNC Symbol;Acc:HGNC:3700] | 69 | 0.551 | 0.0729 | Yes |
| 2 | PDHA1 | pyruvate dehydrogenase E1 subunit alpha 1 [Source:HGNC Symbol;Acc:HGNC:8806] | 223 | 0.477 | 0.1337 | Yes |
| 3 | MDH2 | malate dehydrogenase 2 [Source:HGNC Symbol;Acc:HGNC:6971] | 319 | 0.451 | 0.1924 | Yes |
| 4 | OGDH | oxoglutarate dehydrogenase [Source:HGNC Symbol;Acc:HGNC:8124] | 327 | 0.449 | 0.2531 | Yes |
| 5 | SDHC | succinate dehydrogenase complex subunit C [Source:HGNC Symbol;Acc:HGNC:10682] | 333 | 0.448 | 0.3137 | Yes |
| 6 | MDH1 | malate dehydrogenase 1 [Source:HGNC Symbol;Acc:HGNC:6970] | 594 | 0.398 | 0.3611 | Yes |
| 7 | ACO2 | aconitase 2 [Source:HGNC Symbol;Acc:HGNC:118] | 733 | 0.377 | 0.4087 | Yes |
| 8 | DLAT | dihydrolipoamide S-acetyltransferase [Source:HGNC Symbol;Acc:HGNC:2896] | 998 | 0.345 | 0.4487 | Yes |
| 9 | SUCLG2 | succinate-CoA ligase GDP-forming subunit beta [Source:HGNC Symbol;Acc:HGNC:11450] | 1033 | 0.341 | 0.4941 | Yes |
| 10 | ACLY | ATP citrate lyase [Source:HGNC Symbol;Acc:HGNC:115] | 1362 | 0.310 | 0.5278 | Yes |
| 11 | IDH2 | isocitrate dehydrogenase (NADP(+)) 2 [Source:HGNC Symbol;Acc:HGNC:5383] | 1478 | 0.301 | 0.5656 | Yes |
| 12 | IDH3A | isocitrate dehydrogenase (NAD(+)) 3 catalytic subunit alpha [Source:HGNC Symbol;Acc:HGNC:5384] | 1482 | 0.301 | 0.6063 | Yes |
| 13 | ACO1 | aconitase 1 [Source:HGNC Symbol;Acc:HGNC:117] | 1891 | 0.271 | 0.6326 | Yes |
| 14 | PDHB | pyruvate dehydrogenase E1 subunit beta [Source:HGNC Symbol;Acc:HGNC:8808] | 2053 | 0.261 | 0.6639 | Yes |
| 15 | SUCLA2 | succinate-CoA ligase ADP-forming subunit beta [Source:HGNC Symbol;Acc:HGNC:11448] | 2348 | 0.244 | 0.6895 | Yes |
| 16 | SDHB | succinate dehydrogenase complex iron sulfur subunit B [Source:HGNC Symbol;Acc:HGNC:10681] | 2784 | 0.223 | 0.7087 | Yes |
| 17 | IDH1 | isocitrate dehydrogenase (NADP(+)) 1 [Source:HGNC Symbol;Acc:HGNC:5382] | 3088 | 0.210 | 0.7294 | Yes |
| 18 | DLD | dihydrolipoamide dehydrogenase [Source:HGNC Symbol;Acc:HGNC:2898] | 3616 | 0.189 | 0.7416 | Yes |
| 19 | PCK2 | "phosphoenolpyruvate carboxykinase 2, mitochondrial [Source:HGNC Symbol;Acc:HGNC:8725]" | 4933 | 0.150 | 0.7285 | Yes |
| 20 | OGDHL | oxoglutarate dehydrogenase L [Source:HGNC Symbol;Acc:HGNC:25590] | 5169 | 0.145 | 0.7421 | Yes |
| 21 | PDHA2 | pyruvate dehydrogenase E1 subunit alpha 2 [Source:HGNC Symbol;Acc:HGNC:8807] | 8806 | 0.085 | 0.6610 | No |
| 22 | SUCLG1 | succinate-CoA ligase GDP/ADP-forming subunit alpha [Source:HGNC Symbol;Acc:HGNC:11449] | 9415 | 0.077 | 0.6560 | No |
| 23 | PC | pyruvate carboxylase [Source:HGNC Symbol;Acc:HGNC:8636] | 12295 | 0.050 | 0.5895 | No |
| 24 | SDHD | succinate dehydrogenase complex subunit D [Source:HGNC Symbol;Acc:HGNC:10683] | 14412 | 0.036 | 0.5405 | No |
| 25 | IDH3G | isocitrate dehydrogenase (NAD(+)) 3 non-catalytic subunit gamma [Source:HGNC Symbol;Acc:HGNC:5386] | 19627 | 0.006 | 0.4086 | No |
| 26 | SUCLG2P2 | SUCLG2 pseudogene 2 [Source:HGNC Symbol;Acc:HGNC:43997] | 21183 | -0.003 | 0.3694 | No |
| 27 | SDHA | succinate dehydrogenase complex flavoprotein subunit A [Source:HGNC Symbol;Acc:HGNC:10680] | 24822 | -0.025 | 0.2803 | No |
| 28 | IDH3B | isocitrate dehydrogenase (NAD(+)) 3 non-catalytic subunit beta [Source:HGNC Symbol;Acc:HGNC:5385] | 32174 | -0.103 | 0.1071 | No |
| 29 | CS | citrate synthase [Source:HGNC Symbol;Acc:HGNC:2422] | 33775 | -0.136 | 0.0848 | No |
| 30 | PCK1 | phosphoenolpyruvate carboxykinase 1 [Source:HGNC Symbol;Acc:HGNC:8724] | 35185 | -0.177 | 0.0729 | No |
| 31 | DLST | dihydrolipoamide S-succinyltransferase [Source:HGNC Symbol;Acc:HGNC:2911] | 36653 | -0.239 | 0.0680 | No |
Table: GSEA details [plain text format]

  

Fig 2: KEGG\_CITRATE\_CYCLE\_TCA\_CYCLE      
 Blue-Pink O' Gram in the Space of the Analyzed GeneSet

  

Fig 3: KEGG\_CITRATE\_CYCLE\_TCA\_CYCLE: Random ES distribution      
 Gene set null distribution of ES for **KEGG\_CITRATE\_CYCLE\_TCA\_CYCLE**
