# Supplementary material for: Simultaneous Presentation of Multiple Myeloma and Lung Cancer: Case Report and Gene Bioinformatics Analysis
Source: Front Oncol. 2022 Jun 13;12:859735. doi: 10.3389/fonc.2022.859735 (PMC9235397; doi:10.3389/fonc.2022.859735)
Supplement: Supplementary file 1 [file DataSheet_1.zip › The bioinformatic analysis of MM and lung cancer supplementary materials/Enrichment analysis/MECR/GSEA_4.1.0/MM TCGA/KEGG.Gsea.1639040591792/KEGG_CYTOKINE_CYTOKINE_RECEPTOR_INTERACTION.html]

Details for gene set KEGG\_CYTOKINE\_CYTOKINE\_RECEPTOR\_INTERACTION[GSEA]

|  || Dataset | ExpData\_collapsed\_to\_symbols.ENSG00000116353\_profile\_in\_ExpData.cls #ENSG00000116353 |
| Phenotype | ENSG00000116353\_profile\_in\_ExpData.cls#ENSG00000116353 |
| Upregulated in class | ENSG00000116353\_neg |
| GeneSet | KEGG\_CYTOKINE\_CYTOKINE\_RECEPTOR\_INTERACTION |
| Enrichment Score (ES) | -0.54865897 |
| Normalized Enrichment Score (NES) | -2.4667704 |
| Nominal p-value | 0.0 |
| FDR q-value | 0.0 |
| FWER p-Value | 0.0 |
Table: GSEA Results Summary

  

Fig 1: Enrichment plot: KEGG\_CYTOKINE\_CYTOKINE\_RECEPTOR\_INTERACTION      
 Profile of the Running ES Score & Positions of GeneSet Members on the Rank Ordered List

  

| SYMBOL | TITLE | RANK IN GENE LIST | RANK METRIC SCORE | RUNNING ES | CORE ENRICHMENT || 1 | TNFRSF17 | TNF receptor superfamily member 17 [Source:HGNC Symbol;Acc:HGNC:11913] | 1208 | 0.324 | -0.0214 | No |
| 2 | EDA2R | ectodysplasin A2 receptor [Source:HGNC Symbol;Acc:HGNC:17756] | 1750 | 0.280 | -0.0270 | No |
| 3 | CCR2 | C-C motif chemokine receptor 2 [Source:HGNC Symbol;Acc:HGNC:1603] | 1977 | 0.265 | -0.0249 | No |
| 4 | XCR1 | X-C motif chemokine receptor 1 [Source:HGNC Symbol;Acc:HGNC:1625] | 3998 | 0.177 | -0.0714 | No |
| 5 | IFNAR1 | interferon alpha and beta receptor subunit 1 [Source:HGNC Symbol;Acc:HGNC:5432] | 4033 | 0.175 | -0.0671 | No |
| 6 | IL12A | interleukin 12A [Source:HGNC Symbol;Acc:HGNC:5969] | 4178 | 0.171 | -0.0658 | No |
| 7 | VEGFA | vascular endothelial growth factor A [Source:HGNC Symbol;Acc:HGNC:12680] | 4600 | 0.158 | -0.0719 | No |
| 8 | IL6R | interleukin 6 receptor [Source:HGNC Symbol;Acc:HGNC:6019] | 4649 | 0.157 | -0.0684 | No |
| 9 | IFNAR2 | interferon alpha and beta receptor subunit 2 [Source:HGNC Symbol;Acc:HGNC:5433] | 4817 | 0.153 | -0.0682 | No |
| 10 | BMPR1A | bone morphogenetic protein receptor type 1A [Source:HGNC Symbol;Acc:HGNC:1076] | 4953 | 0.150 | -0.0672 | No |
| 11 | IL17RB | interleukin 17 receptor B [Source:HGNC Symbol;Acc:HGNC:18015] | 5092 | 0.146 | -0.0665 | No |
| 12 | CX3CR1 | C-X3-C motif chemokine receptor 1 [Source:HGNC Symbol;Acc:HGNC:2558] | 5281 | 0.142 | -0.0671 | No |
| 13 | IL23R | interleukin 23 receptor [Source:HGNC Symbol;Acc:HGNC:19100] | 5476 | 0.137 | -0.0680 | No |
| 14 | IL26 | interleukin 26 [Source:HGNC Symbol;Acc:HGNC:17119] | 6055 | 0.126 | -0.0791 | No |
| 15 | EPO | erythropoietin [Source:HGNC Symbol;Acc:HGNC:3415] | 6234 | 0.122 | -0.0800 | No |
| 16 | IL11RA | interleukin 11 receptor subunit alpha [Source:HGNC Symbol;Acc:HGNC:5967] | 6283 | 0.122 | -0.0777 | No |
| 17 | IL5 | interleukin 5 [Source:HGNC Symbol;Acc:HGNC:6016] | 6370 | 0.120 | -0.0763 | No |
| 18 | CXCL13 | C-X-C motif chemokine ligand 13 [Source:HGNC Symbol;Acc:HGNC:10639] | 6476 | 0.118 | -0.0755 | No |
| 19 | TSLP | thymic stromal lymphopoietin [Source:HGNC Symbol;Acc:HGNC:30743] | 6571 | 0.117 | -0.0745 | No |
| 20 | IFNW1 | interferon omega 1 [Source:HGNC Symbol;Acc:HGNC:5448] | 7175 | 0.106 | -0.0868 | No |
| 21 | TNFRSF10A | TNF receptor superfamily member 10a [Source:HGNC Symbol;Acc:HGNC:11904] | 7415 | 0.103 | -0.0899 | No |
| 22 | IL20RB | interleukin 20 receptor subunit beta [Source:HGNC Symbol;Acc:HGNC:6004] | 7439 | 0.102 | -0.0875 | No |
| 23 | IL9R | interleukin 9 receptor [Source:HGNC Symbol;Acc:HGNC:6030] | 7944 | 0.096 | -0.0975 | No |
| 24 | IL12RB2 | interleukin 12 receptor subunit beta 2 [Source:HGNC Symbol;Acc:HGNC:5972] | 8071 | 0.094 | -0.0980 | No |
| 25 | CXCR6 | C-X-C motif chemokine receptor 6 [Source:HGNC Symbol;Acc:HGNC:16647] | 8198 | 0.092 | -0.0985 | No |
| 26 | VEGFD | vascular endothelial growth factor D [Source:HGNC Symbol;Acc:HGNC:3708] | 8223 | 0.092 | -0.0964 | No |
| 27 | TNFRSF8 | TNF receptor superfamily member 8 [Source:HGNC Symbol;Acc:HGNC:11923] | 8533 | 0.088 | -0.1017 | No |
| 28 | CCR3 | C-C motif chemokine receptor 3 [Source:HGNC Symbol;Acc:HGNC:1604] | 8776 | 0.085 | -0.1054 | No |
| 29 | IFNA21 | interferon alpha 21 [Source:HGNC Symbol;Acc:HGNC:5424] | 8834 | 0.084 | -0.1044 | No |
| 30 | CCR10 | C-C motif chemokine receptor 10 [Source:HGNC Symbol;Acc:HGNC:4474] | 8977 | 0.083 | -0.1056 | No |
| 31 | TNFSF4 | TNF superfamily member 4 [Source:HGNC Symbol;Acc:HGNC:11934] | 8982 | 0.083 | -0.1032 | No |
| 32 | IL25 | interleukin 25 [Source:HGNC Symbol;Acc:HGNC:13765] | 9220 | 0.080 | -0.1070 | No |
| 33 | CCL1 | C-C motif chemokine ligand 1 [Source:HGNC Symbol;Acc:HGNC:10609] | 9329 | 0.078 | -0.1074 | No |
| 34 | IL17A | interleukin 17A [Source:HGNC Symbol;Acc:HGNC:5981] | 9679 | 0.074 | -0.1142 | No |
| 35 | IL12B | interleukin 12B [Source:HGNC Symbol;Acc:HGNC:5970] | 9896 | 0.072 | -0.1176 | No |
| 36 | AMH | anti-Mullerian hormone [Source:HGNC Symbol;Acc:HGNC:464] | 10056 | 0.070 | -0.1196 | No |
| 37 | TPO | thyroid peroxidase [Source:HGNC Symbol;Acc:HGNC:12015] | 10798 | 0.063 | -0.1367 | No |
| 38 | INHBC | inhibin subunit beta C [Source:HGNC Symbol;Acc:HGNC:6068] | 10974 | 0.061 | -0.1394 | No |
| 39 | TNFRSF21 | TNF receptor superfamily member 21 [Source:HGNC Symbol;Acc:HGNC:13469] | 11319 | 0.058 | -0.1464 | No |
| 40 | TNFSF10 | TNF superfamily member 10 [Source:HGNC Symbol;Acc:HGNC:11925] | 11514 | 0.056 | -0.1497 | No |
| 41 | IL9 | interleukin 9 [Source:HGNC Symbol;Acc:HGNC:6029] | 11541 | 0.056 | -0.1488 | No |
| 42 | INHBE | inhibin subunit beta E [Source:HGNC Symbol;Acc:HGNC:24029] | 12214 | 0.051 | -0.1645 | No |
| 43 | IFNA1 | interferon alpha 1 [Source:HGNC Symbol;Acc:HGNC:5417] | 12354 | 0.050 | -0.1666 | No |
| 44 | TNFRSF10C | TNF receptor superfamily member 10c [Source:HGNC Symbol;Acc:HGNC:11906] | 12603 | 0.048 | -0.1715 | No |
| 45 | IL11 | interleukin 11 [Source:HGNC Symbol;Acc:HGNC:5966] | 12710 | 0.047 | -0.1728 | No |
| 46 | BMPR1B | bone morphogenetic protein receptor type 1B [Source:HGNC Symbol;Acc:HGNC:1077] | 12885 | 0.046 | -0.1759 | No |
| 47 | IL17B | interleukin 17B [Source:HGNC Symbol;Acc:HGNC:5982] | 13639 | 0.040 | -0.1940 | No |
| 48 | EDAR | ectodysplasin A receptor [Source:HGNC Symbol;Acc:HGNC:2895] | 13641 | 0.040 | -0.1929 | No |
| 49 | IFNA5 | interferon alpha 5 [Source:HGNC Symbol;Acc:HGNC:5426] | 14195 | 0.037 | -0.2059 | No |
| 50 | GDF5 | growth differentiation factor 5 [Source:HGNC Symbol;Acc:HGNC:4220] | 14441 | 0.035 | -0.2111 | No |
| 51 | IL21 | interleukin 21 [Source:HGNC Symbol;Acc:HGNC:6005] | 15279 | 0.030 | -0.2317 | No |
| 52 | CD70 | CD70 molecule [Source:HGNC Symbol;Acc:HGNC:11937] | 15294 | 0.030 | -0.2312 | No |
| 53 | IFNA16 | interferon alpha 16 [Source:HGNC Symbol;Acc:HGNC:5421] | 15302 | 0.030 | -0.2304 | No |
| 54 | CSF2RB | colony stimulating factor 2 receptor subunit beta [Source:HGNC Symbol;Acc:HGNC:2436] | 15660 | 0.028 | -0.2388 | No |
| 55 | CSF2 | colony stimulating factor 2 [Source:HGNC Symbol;Acc:HGNC:2434] | 15864 | 0.027 | -0.2432 | No |
| 56 | CCL25 | C-C motif chemokine ligand 25 [Source:HGNC Symbol;Acc:HGNC:10624] | 15877 | 0.026 | -0.2427 | No |
| 57 | XCL1 | X-C motif chemokine ligand 1 [Source:HGNC Symbol;Acc:HGNC:10645] | 15981 | 0.026 | -0.2446 | No |
| 58 | TNFSF15 | TNF superfamily member 15 [Source:HGNC Symbol;Acc:HGNC:11931] | 15998 | 0.026 | -0.2442 | No |
| 59 | FLT1 | fms related receptor tyrosine kinase 1 [Source:HGNC Symbol;Acc:HGNC:3763] | 16066 | 0.025 | -0.2452 | No |
| 60 | IFNA14 | interferon alpha 14 [Source:HGNC Symbol;Acc:HGNC:5420] | 16484 | 0.023 | -0.2552 | No |
| 61 | ACVR1 | activin A receptor type 1 [Source:HGNC Symbol;Acc:HGNC:171] | 17125 | 0.019 | -0.2710 | No |
| 62 | IFNB1 | interferon beta 1 [Source:HGNC Symbol;Acc:HGNC:5434] | 17326 | 0.018 | -0.2756 | No |
| 63 | IL5RA | interleukin 5 receptor subunit alpha [Source:HGNC Symbol;Acc:HGNC:6017] | 17327 | 0.018 | -0.2751 | No |
| 64 | IFNA13 | interferon alpha 13 [Source:HGNC Symbol;Acc:HGNC:5419] | 17705 | 0.016 | -0.2842 | No |
| 65 | XCL2 | X-C motif chemokine ligand 2 [Source:HGNC Symbol;Acc:HGNC:10646] | 17789 | 0.016 | -0.2859 | No |
| 66 | TNFRSF11B | TNF receptor superfamily member 11b [Source:HGNC Symbol;Acc:HGNC:11909] | 17806 | 0.016 | -0.2858 | No |
| 67 | CCR1 | C-C motif chemokine receptor 1 [Source:HGNC Symbol;Acc:HGNC:1602] | 17985 | 0.015 | -0.2900 | No |
| 68 | GH2 | growth hormone 2 [Source:HGNC Symbol;Acc:HGNC:4262] | 18231 | 0.013 | -0.2959 | No |
| 69 | TGFB2 | transforming growth factor beta 2 [Source:HGNC Symbol;Acc:HGNC:11768] | 18348 | 0.013 | -0.2985 | No |
| 70 | TNFRSF13B | TNF receptor superfamily member 13B [Source:HGNC Symbol;Acc:HGNC:18153] | 18352 | 0.012 | -0.2982 | No |
| 71 | ACVR2B | activin A receptor type 2B [Source:HGNC Symbol;Acc:HGNC:174] | 19022 | 0.009 | -0.3150 | No |
| 72 | TNFSF18 | TNF superfamily member 18 [Source:HGNC Symbol;Acc:HGNC:11932] | 19285 | 0.008 | -0.3215 | No |
| 73 | IL3 | interleukin 3 [Source:HGNC Symbol;Acc:HGNC:6011] | 19317 | 0.007 | -0.3221 | No |
| 74 | CCR8 | C-C motif chemokine receptor 8 [Source:HGNC Symbol;Acc:HGNC:1609] | 19552 | 0.006 | -0.3279 | No |
| 75 | IFNK | interferon kappa [Source:HGNC Symbol;Acc:HGNC:21714] | 20280 | 0.002 | -0.3465 | No |
| 76 | VEGFB | vascular endothelial growth factor B [Source:HGNC Symbol;Acc:HGNC:12681] | 20858 | -0.001 | -0.3612 | No |
| 77 | FLT3LG | fms related receptor tyrosine kinase 3 ligand [Source:HGNC Symbol;Acc:HGNC:3766] | 21002 | -0.002 | -0.3648 | No |
| 78 | FASLG | Fas ligand [Source:HGNC Symbol;Acc:HGNC:11936] | 21141 | -0.003 | -0.3682 | No |
| 79 | IFNE | interferon epsilon [Source:HGNC Symbol;Acc:HGNC:18163] | 21677 | -0.006 | -0.3818 | No |
| 80 | IL12RB1 | interleukin 12 receptor subunit beta 1 [Source:HGNC Symbol;Acc:HGNC:5971] | 22440 | -0.010 | -0.4010 | No |
| 81 | HGF | hepatocyte growth factor [Source:HGNC Symbol;Acc:HGNC:4893] | 22763 | -0.012 | -0.4088 | No |
| 82 | LIFR | LIF receptor subunit alpha [Source:HGNC Symbol;Acc:HGNC:6597] | 23192 | -0.015 | -0.4194 | No |
| 83 | CCR5 | C-C motif chemokine receptor 5 [Source:HGNC Symbol;Acc:HGNC:1606] | 23927 | -0.020 | -0.4376 | No |
| 84 | MPL | "MPL proto-oncogene, thrombopoietin receptor [Source:HGNC Symbol;Acc:HGNC:7217]" | 24028 | -0.020 | -0.4395 | No |
| 85 | IL13 | interleukin 13 [Source:HGNC Symbol;Acc:HGNC:5973] | 24699 | -0.025 | -0.4560 | No |
| 86 | IL24 | interleukin 24 [Source:HGNC Symbol;Acc:HGNC:11346] | 24824 | -0.025 | -0.4584 | No |
| 87 | IFNA7 | interferon alpha 7 [Source:HGNC Symbol;Acc:HGNC:5428] | 24884 | -0.026 | -0.4591 | No |
| 88 | IFNL1 | interferon lambda 1 [Source:HGNC Symbol;Acc:HGNC:18363] | 25041 | -0.027 | -0.4623 | No |
| 89 | PRL | prolactin [Source:HGNC Symbol;Acc:HGNC:9445] | 25088 | -0.027 | -0.4627 | No |
| 90 | IL2 | interleukin 2 [Source:HGNC Symbol;Acc:HGNC:6001] | 25091 | -0.027 | -0.4620 | No |
| 91 | IFNA17 | interferon alpha 17 [Source:HGNC Symbol;Acc:HGNC:5422] | 25197 | -0.028 | -0.4639 | No |
| 92 | IL20RA | interleukin 20 receptor subunit alpha [Source:HGNC Symbol;Acc:HGNC:6003] | 25335 | -0.029 | -0.4665 | No |
| 93 | IFNG | interferon gamma [Source:HGNC Symbol;Acc:HGNC:5438] | 25529 | -0.030 | -0.4706 | No |
| 94 | MET | "MET proto-oncogene, receptor tyrosine kinase [Source:HGNC Symbol;Acc:HGNC:7029]" | 25533 | -0.030 | -0.4698 | No |
| 95 | CCL21 | C-C motif chemokine ligand 21 [Source:HGNC Symbol;Acc:HGNC:10620] | 25690 | -0.031 | -0.4728 | No |
| 96 | VEGFC | vascular endothelial growth factor C [Source:HGNC Symbol;Acc:HGNC:12682] | 25847 | -0.033 | -0.4759 | No |
| 97 | CTF1 | cardiotrophin 1 [Source:HGNC Symbol;Acc:HGNC:2499] | 26101 | -0.034 | -0.4813 | No |
| 98 | BMP7 | bone morphogenetic protein 7 [Source:HGNC Symbol;Acc:HGNC:1074] | 26217 | -0.035 | -0.4832 | No |
| 99 | PPBPP1 | pro-platelet basic protein pseudogene 1 [Source:HGNC Symbol;Acc:HGNC:9241] | 26473 | -0.037 | -0.4887 | No |
| 100 | BMPR2 | bone morphogenetic protein receptor type 2 [Source:HGNC Symbol;Acc:HGNC:1078] | 26700 | -0.039 | -0.4933 | No |
| 101 | CCL11 | C-C motif chemokine ligand 11 [Source:HGNC Symbol;Acc:HGNC:10610] | 26778 | -0.039 | -0.4941 | No |
| 102 | IL22RA1 | interleukin 22 receptor subunit alpha 1 [Source:HGNC Symbol;Acc:HGNC:13700] | 27056 | -0.042 | -0.5000 | No |
| 103 | KIT | "KIT proto-oncogene, receptor tyrosine kinase [Source:HGNC Symbol;Acc:HGNC:6342]" | 27060 | -0.042 | -0.4988 | No |
| 104 | TNFSF9 | TNF superfamily member 9 [Source:HGNC Symbol;Acc:HGNC:11939] | 27087 | -0.042 | -0.4982 | No |
| 105 | CCL22 | C-C motif chemokine ligand 22 [Source:HGNC Symbol;Acc:HGNC:10621] | 27090 | -0.042 | -0.4971 | No |
| 106 | IFNA4 | interferon alpha 4 [Source:HGNC Symbol;Acc:HGNC:5425] | 27286 | -0.044 | -0.5008 | No |
| 107 | IFNA6 | interferon alpha 6 [Source:HGNC Symbol;Acc:HGNC:5427] | 28124 | -0.051 | -0.5207 | No |
| 108 | IL4 | interleukin 4 [Source:HGNC Symbol;Acc:HGNC:6014] | 28173 | -0.052 | -0.5204 | No |
| 109 | IFNL2 | interferon lambda 2 [Source:HGNC Symbol;Acc:HGNC:18364] | 28243 | -0.052 | -0.5206 | No |
| 110 | CCL16 | C-C motif chemokine ligand 16 [Source:HGNC Symbol;Acc:HGNC:10614] | 28287 | -0.053 | -0.5201 | No |
| 111 | IL10RB | interleukin 10 receptor subunit beta [Source:HGNC Symbol;Acc:HGNC:5965] | 28590 | -0.056 | -0.5262 | No |
| 112 | CCR9 | C-C motif chemokine receptor 9 [Source:HGNC Symbol;Acc:HGNC:1610] | 28783 | -0.058 | -0.5294 | No |
| 113 | IFNA8 | interferon alpha 8 [Source:HGNC Symbol;Acc:HGNC:5429] | 28823 | -0.058 | -0.5287 | No |
| 114 | TNFRSF19 | TNF receptor superfamily member 19 [Source:HGNC Symbol;Acc:HGNC:11915] | 29070 | -0.061 | -0.5332 | No |
| 115 | CCL8 | C-C motif chemokine ligand 8 [Source:HGNC Symbol;Acc:HGNC:10635] | 29326 | -0.064 | -0.5378 | No |
| 116 | CCL13 | C-C motif chemokine ligand 13 [Source:HGNC Symbol;Acc:HGNC:10611] | 29378 | -0.065 | -0.5372 | No |
| 117 | TNFSF11 | TNF superfamily member 11 [Source:HGNC Symbol;Acc:HGNC:11926] | 29410 | -0.065 | -0.5361 | No |
| 118 | IFNA2 | interferon alpha 2 [Source:HGNC Symbol;Acc:HGNC:5423] | 29488 | -0.066 | -0.5361 | No |
| 119 | LEP | leptin [Source:HGNC Symbol;Acc:HGNC:6553] | 29531 | -0.066 | -0.5352 | No |
| 120 | IL6 | interleukin 6 [Source:HGNC Symbol;Acc:HGNC:6018] | 29703 | -0.068 | -0.5376 | No |
| 121 | CXCR3 | C-X-C motif chemokine receptor 3 [Source:HGNC Symbol;Acc:HGNC:4540] | 29724 | -0.069 | -0.5361 | No |
| 122 | IFNA10 | interferon alpha 10 [Source:HGNC Symbol;Acc:HGNC:5418] | 29865 | -0.070 | -0.5376 | No |
| 123 | LTA | lymphotoxin alpha [Source:HGNC Symbol;Acc:HGNC:6709] | 29962 | -0.072 | -0.5379 | No |
| 124 | IL13RA1 | interleukin 13 receptor subunit alpha 1 [Source:HGNC Symbol;Acc:HGNC:5974] | 30243 | -0.075 | -0.5429 | No |
| 125 | IL2RB | interleukin 2 receptor subunit beta [Source:HGNC Symbol;Acc:HGNC:6009] | 30287 | -0.076 | -0.5417 | No |
| 126 | IL20 | interleukin 20 [Source:HGNC Symbol;Acc:HGNC:6002] | 30306 | -0.076 | -0.5400 | No |
| 127 | PDGFA | platelet derived growth factor subunit A [Source:HGNC Symbol;Acc:HGNC:8799] | 30414 | -0.077 | -0.5404 | No |
| 128 | IL15RA | interleukin 15 receptor subunit alpha [Source:HGNC Symbol;Acc:HGNC:5978] | 30581 | -0.079 | -0.5423 | No |
| 129 | CCL27 | C-C motif chemokine ligand 27 [Source:HGNC Symbol;Acc:HGNC:10626] | 30627 | -0.080 | -0.5411 | No |
| 130 | LTBR | lymphotoxin beta receptor [Source:HGNC Symbol;Acc:HGNC:6718] | 30716 | -0.081 | -0.5410 | No |
| 131 | TGFB3 | transforming growth factor beta 3 [Source:HGNC Symbol;Acc:HGNC:11769] | 30832 | -0.083 | -0.5415 | No |
| 132 | LEPR | leptin receptor [Source:HGNC Symbol;Acc:HGNC:6554] | 30929 | -0.084 | -0.5415 | No |
| 133 | CCL17 | C-C motif chemokine ligand 17 [Source:HGNC Symbol;Acc:HGNC:10615] | 30976 | -0.084 | -0.5402 | No |
| 134 | TNFRSF10B | TNF receptor superfamily member 10b [Source:HGNC Symbol;Acc:HGNC:11905] | 31280 | -0.089 | -0.5453 | No |
| 135 | CXCR2 | C-X-C motif chemokine receptor 2 [Source:HGNC Symbol;Acc:HGNC:6027] | 31411 | -0.090 | -0.5460 | Yes |
| 136 | TNFSF13 | TNF superfamily member 13 [Source:HGNC Symbol;Acc:HGNC:11928] | 31491 | -0.091 | -0.5453 | Yes |
| 137 | CD40LG | CD40 ligand [Source:HGNC Symbol;Acc:HGNC:11935] | 31532 | -0.092 | -0.5436 | Yes |
| 138 | CCL24 | C-C motif chemokine ligand 24 [Source:HGNC Symbol;Acc:HGNC:10623] | 31647 | -0.094 | -0.5438 | Yes |
| 139 | CXCL9 | C-X-C motif chemokine ligand 9 [Source:HGNC Symbol;Acc:HGNC:7098] | 31652 | -0.094 | -0.5411 | Yes |
| 140 | IL19 | interleukin 19 [Source:HGNC Symbol;Acc:HGNC:5990] | 31661 | -0.094 | -0.5385 | Yes |
| 141 | IFNL3 | interferon lambda 3 [Source:HGNC Symbol;Acc:HGNC:18365] | 31673 | -0.094 | -0.5360 | Yes |
| 142 | TNFSF13B | TNF superfamily member 13b [Source:HGNC Symbol;Acc:HGNC:11929] | 31836 | -0.097 | -0.5373 | Yes |
| 143 | CCL19 | C-C motif chemokine ligand 19 [Source:HGNC Symbol;Acc:HGNC:10617] | 31855 | -0.097 | -0.5349 | Yes |
| 144 | CCL15 | C-C motif chemokine ligand 15 [Source:HGNC Symbol;Acc:HGNC:10613] | 31927 | -0.099 | -0.5338 | Yes |
| 145 | PRLR | prolactin receptor [Source:HGNC Symbol;Acc:HGNC:9446] | 32010 | -0.100 | -0.5330 | Yes |
| 146 | CCR6 | C-C motif chemokine receptor 6 [Source:HGNC Symbol;Acc:HGNC:1607] | 32052 | -0.101 | -0.5311 | Yes |
| 147 | IL3RA | interleukin 3 receptor subunit alpha [Source:HGNC Symbol;Acc:HGNC:6012] | 32087 | -0.101 | -0.5289 | Yes |
| 148 | CCL18 | C-C motif chemokine ligand 18 [Source:HGNC Symbol;Acc:HGNC:10616] | 32213 | -0.103 | -0.5291 | Yes |
| 149 | CCL28 | C-C motif chemokine ligand 28 [Source:HGNC Symbol;Acc:HGNC:17700] | 32346 | -0.106 | -0.5293 | Yes |
| 150 | IL22 | interleukin 22 [Source:HGNC Symbol;Acc:HGNC:14900] | 32373 | -0.107 | -0.5269 | Yes |
| 151 | GHR | growth hormone receptor [Source:HGNC Symbol;Acc:HGNC:4263] | 32402 | -0.107 | -0.5244 | Yes |
| 152 | IL1A | interleukin 1 alpha [Source:HGNC Symbol;Acc:HGNC:5991] | 32718 | -0.113 | -0.5291 | Yes |
| 153 | CCL4L2 | C-C motif chemokine ligand 4 like 2 [Source:HGNC Symbol;Acc:HGNC:24066] | 32867 | -0.116 | -0.5295 | Yes |
| 154 | CSF1R | colony stimulating factor 1 receptor [Source:HGNC Symbol;Acc:HGNC:2433] | 33122 | -0.121 | -0.5324 | Yes |
| 155 | EGF | epidermal growth factor [Source:HGNC Symbol;Acc:HGNC:3229] | 33135 | -0.121 | -0.5292 | Yes |
| 156 | IL1RAP | interleukin 1 receptor accessory protein [Source:HGNC Symbol;Acc:HGNC:5995] | 33145 | -0.122 | -0.5258 | Yes |
| 157 | IL21R | interleukin 21 receptor [Source:HGNC Symbol;Acc:HGNC:6006] | 33287 | -0.124 | -0.5257 | Yes |
| 158 | CXCR5 | C-X-C motif chemokine receptor 5 [Source:HGNC Symbol;Acc:HGNC:1060] | 33366 | -0.126 | -0.5240 | Yes |
| 159 | PDGFC | platelet derived growth factor C [Source:HGNC Symbol;Acc:HGNC:8801] | 33384 | -0.127 | -0.5207 | Yes |
| 160 | CXCL10 | C-X-C motif chemokine ligand 10 [Source:HGNC Symbol;Acc:HGNC:10637] | 33451 | -0.128 | -0.5186 | Yes |
| 161 | PDGFB | platelet derived growth factor subunit B [Source:HGNC Symbol;Acc:HGNC:8800] | 33560 | -0.130 | -0.5175 | Yes |
| 162 | CXCL11 | C-X-C motif chemokine ligand 11 [Source:HGNC Symbol;Acc:HGNC:10638] | 33604 | -0.131 | -0.5148 | Yes |
| 163 | CCL26 | C-C motif chemokine ligand 26 [Source:HGNC Symbol;Acc:HGNC:10625] | 33627 | -0.132 | -0.5114 | Yes |
| 164 | ACVRL1 | activin A receptor like type 1 [Source:HGNC Symbol;Acc:HGNC:175] | 33635 | -0.132 | -0.5077 | Yes |
| 165 | INHBA | inhibin subunit beta A [Source:HGNC Symbol;Acc:HGNC:6066] | 33687 | -0.133 | -0.5051 | Yes |
| 166 | CX3CL1 | C-X3-C motif chemokine ligand 1 [Source:HGNC Symbol;Acc:HGNC:10647] | 33763 | -0.135 | -0.5030 | Yes |
| 167 | CCL3 | C-C motif chemokine ligand 3 [Source:HGNC Symbol;Acc:HGNC:10627] | 33771 | -0.136 | -0.4992 | Yes |
| 168 | LTB | lymphotoxin beta [Source:HGNC Symbol;Acc:HGNC:6711] | 33796 | -0.137 | -0.4958 | Yes |
| 169 | IL2RG | interleukin 2 receptor subunit gamma [Source:HGNC Symbol;Acc:HGNC:6010] | 33828 | -0.137 | -0.4925 | Yes |
| 170 | TGFBR2 | transforming growth factor beta receptor 2 [Source:HGNC Symbol;Acc:HGNC:11773] | 33892 | -0.139 | -0.4900 | Yes |
| 171 | EPOR | erythropoietin receptor [Source:HGNC Symbol;Acc:HGNC:3416] | 33949 | -0.140 | -0.4873 | Yes |
| 172 | TNFRSF9 | TNF receptor superfamily member 9 [Source:HGNC Symbol;Acc:HGNC:11924] | 34026 | -0.142 | -0.4851 | Yes |
| 173 | BMP2 | bone morphogenetic protein 2 [Source:HGNC Symbol;Acc:HGNC:1069] | 34040 | -0.142 | -0.4812 | Yes |
| 174 | TNFRSF25 | TNF receptor superfamily member 25 [Source:HGNC Symbol;Acc:HGNC:11910] | 34045 | -0.142 | -0.4771 | Yes |
| 175 | FLT4 | fms related receptor tyrosine kinase 4 [Source:HGNC Symbol;Acc:HGNC:3767] | 34111 | -0.144 | -0.4745 | Yes |
| 176 | CCL4 | C-C motif chemokine ligand 4 [Source:HGNC Symbol;Acc:HGNC:10630] | 34162 | -0.145 | -0.4715 | Yes |
| 177 | TNFRSF1A | TNF receptor superfamily member 1A [Source:HGNC Symbol;Acc:HGNC:11916] | 34204 | -0.147 | -0.4682 | Yes |
| 178 | CD27 | CD27 molecule [Source:HGNC Symbol;Acc:HGNC:11922] | 34333 | -0.151 | -0.4671 | Yes |
| 179 | IL10 | interleukin 10 [Source:HGNC Symbol;Acc:HGNC:5962] | 34351 | -0.151 | -0.4631 | Yes |
| 180 | IL18 | interleukin 18 [Source:HGNC Symbol;Acc:HGNC:5986] | 34385 | -0.152 | -0.4594 | Yes |
| 181 | AMHR2 | anti-Mullerian hormone receptor type 2 [Source:HGNC Symbol;Acc:HGNC:465] | 34469 | -0.155 | -0.4570 | Yes |
| 182 | EDA | ectodysplasin A [Source:HGNC Symbol;Acc:HGNC:3157] | 34484 | -0.155 | -0.4528 | Yes |
| 183 | GH1 | growth hormone 1 [Source:HGNC Symbol;Acc:HGNC:4261] | 34620 | -0.159 | -0.4515 | Yes |
| 184 | CXCL3 | C-X-C motif chemokine ligand 3 [Source:HGNC Symbol;Acc:HGNC:4604] | 34653 | -0.161 | -0.4476 | Yes |
| 185 | KDR | kinase insert domain receptor [Source:HGNC Symbol;Acc:HGNC:6307] | 34689 | -0.161 | -0.4437 | Yes |
| 186 | TNFRSF14 | TNF receptor superfamily member 14 [Source:HGNC Symbol;Acc:HGNC:11912] | 34692 | -0.161 | -0.4390 | Yes |
| 187 | CXCR1 | C-X-C motif chemokine receptor 1 [Source:HGNC Symbol;Acc:HGNC:6026] | 34719 | -0.162 | -0.4349 | Yes |
| 188 | CCL5 | C-C motif chemokine ligand 5 [Source:HGNC Symbol;Acc:HGNC:10632] | 34763 | -0.164 | -0.4312 | Yes |
| 189 | CXCL6 | C-X-C motif chemokine ligand 6 [Source:HGNC Symbol;Acc:HGNC:10643] | 34787 | -0.164 | -0.4269 | Yes |
| 190 | TNFSF8 | TNF superfamily member 8 [Source:HGNC Symbol;Acc:HGNC:11938] | 34808 | -0.165 | -0.4226 | Yes |
| 191 | PDGFRB | platelet derived growth factor receptor beta [Source:HGNC Symbol;Acc:HGNC:8804] | 34887 | -0.167 | -0.4196 | Yes |
| 192 | TNFSF14 | TNF superfamily member 14 [Source:HGNC Symbol;Acc:HGNC:11930] | 34893 | -0.167 | -0.4148 | Yes |
| 193 | IL7 | interleukin 7 [Source:HGNC Symbol;Acc:HGNC:6023] | 34913 | -0.168 | -0.4103 | Yes |
| 194 | IL22RA2 | interleukin 22 receptor subunit alpha 2 [Source:HGNC Symbol;Acc:HGNC:14901] | 34926 | -0.168 | -0.4057 | Yes |
| 195 | TNFRSF11A | TNF receptor superfamily member 11a [Source:HGNC Symbol;Acc:HGNC:11908] | 34959 | -0.169 | -0.4015 | Yes |
| 196 | CCL2 | C-C motif chemokine ligand 2 [Source:HGNC Symbol;Acc:HGNC:10618] | 35124 | -0.175 | -0.4005 | Yes |
| 197 | TNFRSF10D | TNF receptor superfamily member 10d [Source:HGNC Symbol;Acc:HGNC:11907] | 35131 | -0.175 | -0.3955 | Yes |
| 198 | CCL7 | C-C motif chemokine ligand 7 [Source:HGNC Symbol;Acc:HGNC:10634] | 35132 | -0.175 | -0.3904 | Yes |
| 199 | CCL23 | C-C motif chemokine ligand 23 [Source:HGNC Symbol;Acc:HGNC:10622] | 35309 | -0.181 | -0.3895 | Yes |
| 200 | KITLG | KIT ligand [Source:HGNC Symbol;Acc:HGNC:6343] | 35389 | -0.185 | -0.3861 | Yes |
| 201 | EGFR | epidermal growth factor receptor [Source:HGNC Symbol;Acc:HGNC:3236] | 35417 | -0.186 | -0.3813 | Yes |
| 202 | CRLF2 | cytokine receptor like factor 2 [Source:HGNC Symbol;Acc:HGNC:14281] | 35442 | -0.186 | -0.3764 | Yes |
| 203 | IFNLR1 | interferon lambda receptor 1 [Source:HGNC Symbol;Acc:HGNC:18584] | 35590 | -0.191 | -0.3745 | Yes |
| 204 | CCL14 | C-C motif chemokine ligand 14 [Source:HGNC Symbol;Acc:HGNC:10612] | 35687 | -0.195 | -0.3712 | Yes |
| 205 | PLEKHO2 | pleckstrin homology domain containing O2 [Source:HGNC Symbol;Acc:HGNC:30026] | 35698 | -0.196 | -0.3657 | Yes |
| 206 | CNTFR | ciliary neurotrophic factor receptor [Source:HGNC Symbol;Acc:HGNC:2170] | 35744 | -0.197 | -0.3610 | Yes |
| 207 | CCR4 | C-C motif chemokine receptor 4 [Source:HGNC Symbol;Acc:HGNC:1605] | 35765 | -0.198 | -0.3557 | Yes |
| 208 | PF4V1 | platelet factor 4 variant 1 [Source:HGNC Symbol;Acc:HGNC:8862] | 35781 | -0.199 | -0.3502 | Yes |
| 209 | CXCL14 | C-X-C motif chemokine ligand 14 [Source:HGNC Symbol;Acc:HGNC:10640] | 35832 | -0.201 | -0.3456 | Yes |
| 210 | IL18R1 | interleukin 18 receptor 1 [Source:HGNC Symbol;Acc:HGNC:5988] | 35843 | -0.201 | -0.3399 | Yes |
| 211 | IL1B | interleukin 1 beta [Source:HGNC Symbol;Acc:HGNC:5992] | 35858 | -0.202 | -0.3343 | Yes |
| 212 | CCL20 | C-C motif chemokine ligand 20 [Source:HGNC Symbol;Acc:HGNC:10619] | 35867 | -0.202 | -0.3285 | Yes |
| 213 | IL6ST | interleukin 6 signal transducer [Source:HGNC Symbol;Acc:HGNC:6021] | 35874 | -0.202 | -0.3227 | Yes |
| 214 | IL7R | interleukin 7 receptor [Source:HGNC Symbol;Acc:HGNC:6024] | 36033 | -0.209 | -0.3206 | Yes |
| 215 | PF4 | platelet factor 4 [Source:HGNC Symbol;Acc:HGNC:8861] | 36067 | -0.210 | -0.3153 | Yes |
| 216 | IL2RA | interleukin 2 receptor subunit alpha [Source:HGNC Symbol;Acc:HGNC:6008] | 36108 | -0.212 | -0.3100 | Yes |
| 217 | CCL3L3 | C-C motif chemokine ligand 3 like 3 [Source:HGNC Symbol;Acc:HGNC:30554] | 36178 | -0.215 | -0.3055 | Yes |
| 218 | ACVR1B | activin A receptor type 1B [Source:HGNC Symbol;Acc:HGNC:172] | 36194 | -0.215 | -0.2995 | Yes |
| 219 | TGFB1 | transforming growth factor beta 1 [Source:HGNC Symbol;Acc:HGNC:11766] | 36247 | -0.217 | -0.2944 | Yes |
| 220 | FLT3 | fms related receptor tyrosine kinase 3 [Source:HGNC Symbol;Acc:HGNC:3765] | 36256 | -0.218 | -0.2882 | Yes |
| 221 | IL18RAP | interleukin 18 receptor accessory protein [Source:HGNC Symbol;Acc:HGNC:5989] | 36321 | -0.221 | -0.2833 | Yes |
| 222 | CXCL12 | C-X-C motif chemokine ligand 12 [Source:HGNC Symbol;Acc:HGNC:10672] | 36344 | -0.222 | -0.2773 | Yes |
| 223 | PPBP | pro-platelet basic protein [Source:HGNC Symbol;Acc:HGNC:9240] | 36358 | -0.223 | -0.2710 | Yes |
| 224 | CXCL1 | C-X-C motif chemokine ligand 1 [Source:HGNC Symbol;Acc:HGNC:4602] | 36378 | -0.224 | -0.2649 | Yes |
| 225 | CSF3 | colony stimulating factor 3 [Source:HGNC Symbol;Acc:HGNC:2438] | 36399 | -0.225 | -0.2588 | Yes |
| 226 | TNF | tumor necrosis factor [Source:HGNC Symbol;Acc:HGNC:11892] | 36419 | -0.226 | -0.2526 | Yes |
| 227 | CSF2RA | colony stimulating factor 2 receptor subunit alpha [Source:HGNC Symbol;Acc:HGNC:2435] | 36454 | -0.228 | -0.2467 | Yes |
| 228 | CSF1 | colony stimulating factor 1 [Source:HGNC Symbol;Acc:HGNC:2432] | 36518 | -0.231 | -0.2415 | Yes |
| 229 | PDGFRA | platelet derived growth factor receptor alpha [Source:HGNC Symbol;Acc:HGNC:8803] | 36521 | -0.231 | -0.2347 | Yes |
| 230 | INHBB | inhibin subunit beta B [Source:HGNC Symbol;Acc:HGNC:6067] | 36531 | -0.232 | -0.2281 | Yes |
| 231 | OSM | oncostatin M [Source:HGNC Symbol;Acc:HGNC:8506] | 36551 | -0.233 | -0.2217 | Yes |
| 232 | IL15 | interleukin 15 [Source:HGNC Symbol;Acc:HGNC:5977] | 36605 | -0.236 | -0.2161 | Yes |
| 233 | IL4R | interleukin 4 receptor [Source:HGNC Symbol;Acc:HGNC:6015] | 36680 | -0.240 | -0.2109 | Yes |
| 234 | IL1R2 | interleukin 1 receptor type 2 [Source:HGNC Symbol;Acc:HGNC:5994] | 36783 | -0.246 | -0.2063 | Yes |
| 235 | LIF | LIF interleukin 6 family cytokine [Source:HGNC Symbol;Acc:HGNC:6596] | 36799 | -0.247 | -0.1994 | Yes |
| 236 | TNFSF12 | TNF superfamily member 12 [Source:HGNC Symbol;Acc:HGNC:11927] | 36816 | -0.248 | -0.1925 | Yes |
| 237 | FAS | Fas cell surface death receptor [Source:HGNC Symbol;Acc:HGNC:11920] | 36825 | -0.248 | -0.1854 | Yes |
| 238 | CXCR4 | C-X-C motif chemokine receptor 4 [Source:HGNC Symbol;Acc:HGNC:2561] | 36861 | -0.251 | -0.1789 | Yes |
| 239 | TNFRSF1B | TNF receptor superfamily member 1B [Source:HGNC Symbol;Acc:HGNC:11917] | 36950 | -0.256 | -0.1735 | Yes |
| 240 | CXCL2 | C-X-C motif chemokine ligand 2 [Source:HGNC Symbol;Acc:HGNC:4603] | 36954 | -0.257 | -0.1661 | Yes |
| 241 | CSF3R | colony stimulating factor 3 receptor [Source:HGNC Symbol;Acc:HGNC:2439] | 36961 | -0.257 | -0.1586 | Yes |
| 242 | ACVR2A | activin A receptor type 2A [Source:HGNC Symbol;Acc:HGNC:173] | 37002 | -0.260 | -0.1520 | Yes |
| 243 | CD40 | CD40 molecule [Source:HGNC Symbol;Acc:HGNC:11919] | 37041 | -0.262 | -0.1452 | Yes |
| 244 | NGFR | nerve growth factor receptor [Source:HGNC Symbol;Acc:HGNC:7809] | 37268 | -0.277 | -0.1428 | Yes |
| 245 | TNFRSF18 | TNF receptor superfamily member 18 [Source:HGNC Symbol;Acc:HGNC:11914] | 37354 | -0.284 | -0.1366 | Yes |
| 246 | CXCL5 | C-X-C motif chemokine ligand 5 [Source:HGNC Symbol;Acc:HGNC:10642] | 37407 | -0.288 | -0.1294 | Yes |
| 247 | CNTF | ciliary neurotrophic factor [Source:HGNC Symbol;Acc:HGNC:2169] | 37484 | -0.295 | -0.1227 | Yes |
| 248 | IL1R1 | interleukin 1 receptor type 1 [Source:HGNC Symbol;Acc:HGNC:5993] | 37530 | -0.299 | -0.1150 | Yes |
| 249 | TNFRSF4 | TNF receptor superfamily member 4 [Source:HGNC Symbol;Acc:HGNC:11918] | 37681 | -0.312 | -0.1096 | Yes |
| 250 | CXCL8 | C-X-C motif chemokine ligand 8 [Source:HGNC Symbol;Acc:HGNC:6025] | 37687 | -0.313 | -0.1005 | Yes |
| 251 | CXCL16 | C-X-C motif chemokine ligand 16 [Source:HGNC Symbol;Acc:HGNC:16642] | 37745 | -0.318 | -0.0926 | Yes |
| 252 | IL23A | interleukin 23 subunit alpha [Source:HGNC Symbol;Acc:HGNC:15488] | 37758 | -0.319 | -0.0835 | Yes |
| 253 | IFNGR1 | interferon gamma receptor 1 [Source:HGNC Symbol;Acc:HGNC:5439] | 37779 | -0.322 | -0.0745 | Yes |
| 254 | CLCF1 | cardiotrophin like cytokine factor 1 [Source:HGNC Symbol;Acc:HGNC:17412] | 37791 | -0.323 | -0.0653 | Yes |
| 255 | IL10RA | interleukin 10 receptor subunit alpha [Source:HGNC Symbol;Acc:HGNC:5964] | 37928 | -0.340 | -0.0587 | Yes |
| 256 | OSMR | oncostatin M receptor [Source:HGNC Symbol;Acc:HGNC:8507] | 37932 | -0.341 | -0.0487 | Yes |
| 257 | IL17RA | interleukin 17 receptor A [Source:HGNC Symbol;Acc:HGNC:5985] | 37967 | -0.346 | -0.0394 | Yes |
| 258 | CCR7 | C-C motif chemokine receptor 7 [Source:HGNC Symbol;Acc:HGNC:1608] | 38087 | -0.360 | -0.0318 | Yes |
| 259 | TNFRSF12A | TNF receptor superfamily member 12A [Source:HGNC Symbol;Acc:HGNC:18152] | 38203 | -0.379 | -0.0236 | Yes |
| 260 | TNFRSF13C | TNF receptor superfamily member 13C [Source:HGNC Symbol;Acc:HGNC:17755] | 38320 | -0.403 | -0.0147 | Yes |
| 261 | RELT | RELT TNF receptor [Source:HGNC Symbol;Acc:HGNC:13764] | 38340 | -0.407 | -0.0032 | Yes |
| 262 | TGFBR1 | transforming growth factor beta receptor 1 [Source:HGNC Symbol;Acc:HGNC:11772] | 38393 | -0.417 | 0.0078 | Yes |
| 263 | IFNGR2 | interferon gamma receptor 2 [Source:HGNC Symbol;Acc:HGNC:5440] | 38582 | -0.531 | 0.0186 | Yes |
| 264 | TNFRSF6B | TNF receptor superfamily member 6b [Source:HGNC Symbol;Acc:HGNC:11921] | 39208 | NaN | 0.0029 | Yes |
Table: GSEA details [plain text format]

  

Fig 2: KEGG\_CYTOKINE\_CYTOKINE\_RECEPTOR\_INTERACTION      
 Blue-Pink O' Gram in the Space of the Analyzed GeneSet

  

Fig 3: KEGG\_CYTOKINE\_CYTOKINE\_RECEPTOR\_INTERACTION: Random ES distribution      
 Gene set null distribution of ES for **KEGG\_CYTOKINE\_CYTOKINE\_RECEPTOR\_INTERACTION**
