# Supplementary material for: Simultaneous Presentation of Multiple Myeloma and Lung Cancer: Case Report and Gene Bioinformatics Analysis
Source: Front Oncol. 2022 Jun 13;12:859735. doi: 10.3389/fonc.2022.859735 (PMC9235397; doi:10.3389/fonc.2022.859735)
Supplement: Supplementary file 1 [file DataSheet_1.zip › The bioinformatic analysis of MM and lung cancer supplementary materials/Enrichment analysis/MECR/GSEA_4.1.0/MM TCGA/KEGG.Gsea.1639040591792/KEGG_DNA_REPLICATION.html]

Details for gene set KEGG\_DNA\_REPLICATION[GSEA]

|  || Dataset | ExpData\_collapsed\_to\_symbols.ENSG00000116353\_profile\_in\_ExpData.cls #ENSG00000116353 |
| Phenotype | ENSG00000116353\_profile\_in\_ExpData.cls#ENSG00000116353 |
| Upregulated in class | ENSG00000116353\_pos |
| GeneSet | KEGG\_DNA\_REPLICATION |
| Enrichment Score (ES) | 0.7720552 |
| Normalized Enrichment Score (NES) | 2.3225868 |
| Nominal p-value | 0.0 |
| FDR q-value | 0.0 |
| FWER p-Value | 0.0 |
Table: GSEA Results Summary

  

Fig 1: Enrichment plot: KEGG\_DNA\_REPLICATION      
 Profile of the Running ES Score & Positions of GeneSet Members on the Rank Ordered List

  

| SYMBOL | TITLE | RANK IN GENE LIST | RANK METRIC SCORE | RUNNING ES | CORE ENRICHMENT || 1 | POLD2 | "DNA polymerase delta 2, accessory subunit [Source:HGNC Symbol;Acc:HGNC:9176]" | 15 | 0.617 | 0.0748 | Yes |
| 2 | RNASEH2A | ribonuclease H2 subunit A [Source:HGNC Symbol;Acc:HGNC:18518] | 354 | 0.443 | 0.1201 | Yes |
| 3 | RPA1 | replication protein A1 [Source:HGNC Symbol;Acc:HGNC:10289] | 360 | 0.442 | 0.1737 | Yes |
| 4 | MCM3 | minichromosome maintenance complex component 3 [Source:HGNC Symbol;Acc:HGNC:6945] | 439 | 0.426 | 0.2236 | Yes |
| 5 | POLD1 | "DNA polymerase delta 1, catalytic subunit [Source:HGNC Symbol;Acc:HGNC:9175]" | 541 | 0.405 | 0.2704 | Yes |
| 6 | PRIM2 | DNA primase subunit 2 [Source:HGNC Symbol;Acc:HGNC:9370] | 602 | 0.397 | 0.3172 | Yes |
| 7 | POLA1 | "DNA polymerase alpha 1, catalytic subunit [Source:HGNC Symbol;Acc:HGNC:9173]" | 652 | 0.390 | 0.3635 | Yes |
| 8 | PCNA | proliferating cell nuclear antigen [Source:HGNC Symbol;Acc:HGNC:8729] | 1093 | 0.336 | 0.3931 | Yes |
| 9 | PRIM1 | DNA primase subunit 1 [Source:HGNC Symbol;Acc:HGNC:9369] | 1113 | 0.334 | 0.4333 | Yes |
| 10 | MCM2 | minichromosome maintenance complex component 2 [Source:HGNC Symbol;Acc:HGNC:6944] | 1157 | 0.329 | 0.4722 | Yes |
| 11 | POLD3 | "DNA polymerase delta 3, accessory subunit [Source:HGNC Symbol;Acc:HGNC:20932]" | 1700 | 0.284 | 0.4929 | Yes |
| 12 | RFC4 | replication factor C subunit 4 [Source:HGNC Symbol;Acc:HGNC:9972] | 1728 | 0.281 | 0.5265 | Yes |
| 13 | FEN1 | flap structure-specific endonuclease 1 [Source:HGNC Symbol;Acc:HGNC:3650] | 1754 | 0.279 | 0.5599 | Yes |
| 14 | SSBP1 | single stranded DNA binding protein 1 [Source:HGNC Symbol;Acc:HGNC:11317] | 2105 | 0.258 | 0.5823 | Yes |
| 15 | RPA3 | replication protein A3 [Source:HGNC Symbol;Acc:HGNC:10291] | 2705 | 0.227 | 0.5947 | Yes |
| 16 | POLE2 | "DNA polymerase epsilon 2, accessory subunit [Source:HGNC Symbol;Acc:HGNC:9178]" | 3128 | 0.208 | 0.6093 | Yes |
| 17 | MCM4 | minichromosome maintenance complex component 4 [Source:HGNC Symbol;Acc:HGNC:6947] | 3205 | 0.205 | 0.6323 | Yes |
| 18 | RNASEH2C | ribonuclease H2 subunit C [Source:HGNC Symbol;Acc:HGNC:24116] | 3208 | 0.205 | 0.6572 | Yes |
| 19 | MCM6 | minichromosome maintenance complex component 6 [Source:HGNC Symbol;Acc:HGNC:6949] | 3417 | 0.196 | 0.6758 | Yes |
| 20 | POLE4 | "DNA polymerase epsilon 4, accessory subunit [Source:HGNC Symbol;Acc:HGNC:18755]" | 3440 | 0.195 | 0.6990 | Yes |
| 21 | RFC2 | replication factor C subunit 2 [Source:HGNC Symbol;Acc:HGNC:9970] | 3666 | 0.187 | 0.7160 | Yes |
| 22 | POLA2 | "DNA polymerase alpha 2, accessory subunit [Source:HGNC Symbol;Acc:HGNC:30073]" | 3803 | 0.183 | 0.7349 | Yes |
| 23 | RFC3 | replication factor C subunit 3 [Source:HGNC Symbol;Acc:HGNC:9971] | 3869 | 0.181 | 0.7553 | Yes |
| 24 | DNA2 | DNA replication helicase/nuclease 2 [Source:HGNC Symbol;Acc:HGNC:2939] | 4048 | 0.175 | 0.7721 | Yes |
| 25 | RFC5 | replication factor C subunit 5 [Source:HGNC Symbol;Acc:HGNC:9973] | 6469 | 0.118 | 0.7249 | No |
| 26 | RPA2 | replication protein A2 [Source:HGNC Symbol;Acc:HGNC:10290] | 7917 | 0.096 | 0.6997 | No |
| 27 | MCM7 | minichromosome maintenance complex component 7 [Source:HGNC Symbol;Acc:HGNC:6950] | 8021 | 0.095 | 0.7086 | No |
| 28 | MCM5 | minichromosome maintenance complex component 5 [Source:HGNC Symbol;Acc:HGNC:6948] | 8993 | 0.083 | 0.6939 | No |
| 29 | POLE | "DNA polymerase epsilon, catalytic subunit [Source:HGNC Symbol;Acc:HGNC:9177]" | 9755 | 0.073 | 0.6835 | No |
| 30 | RNASEH1 | ribonuclease H1 [Source:HGNC Symbol;Acc:HGNC:18466] | 16235 | 0.024 | 0.5216 | No |
| 31 | RNASEH2B | ribonuclease H2 subunit B [Source:HGNC Symbol;Acc:HGNC:25671] | 22581 | -0.011 | 0.3614 | No |
| 32 | LIG1 | DNA ligase 1 [Source:HGNC Symbol;Acc:HGNC:6598] | 24175 | -0.021 | 0.3235 | No |
| 33 | POLE3 | "DNA polymerase epsilon 3, accessory subunit [Source:HGNC Symbol;Acc:HGNC:13546]" | 27729 | -0.048 | 0.2388 | No |
| 34 | RPA4 | replication protein A4 [Source:HGNC Symbol;Acc:HGNC:30305] | 32283 | -0.105 | 0.1357 | No |
| 35 | POLD4 | "DNA polymerase delta 4, accessory subunit [Source:HGNC Symbol;Acc:HGNC:14106]" | 34723 | -0.162 | 0.0934 | No |
| 36 | RFC1 | replication factor C subunit 1 [Source:HGNC Symbol;Acc:HGNC:9969] | 35666 | -0.195 | 0.0931 | No |
Table: GSEA details [plain text format]

  

Fig 2: KEGG\_DNA\_REPLICATION      
 Blue-Pink O' Gram in the Space of the Analyzed GeneSet

  

Fig 3: KEGG\_DNA\_REPLICATION: Random ES distribution      
 Gene set null distribution of ES for **KEGG\_DNA\_REPLICATION**
