# Supplementary material for: Simultaneous Presentation of Multiple Myeloma and Lung Cancer: Case Report and Gene Bioinformatics Analysis
Source: Front Oncol. 2022 Jun 13;12:859735. doi: 10.3389/fonc.2022.859735 (PMC9235397; doi:10.3389/fonc.2022.859735)
Supplement: Supplementary file 1 [file DataSheet_1.zip › The bioinformatic analysis of MM and lung cancer supplementary materials/Enrichment analysis/MECR/GSEA_4.1.0/MM TCGA/KEGG.Gsea.1639040591792/KEGG_DORSO_VENTRAL_AXIS_FORMATION.html]

Details for gene set KEGG\_DORSO\_VENTRAL\_AXIS\_FORMATION[GSEA]

|  || Dataset | ExpData\_collapsed\_to\_symbols.ENSG00000116353\_profile\_in\_ExpData.cls #ENSG00000116353 |
| Phenotype | ENSG00000116353\_profile\_in\_ExpData.cls#ENSG00000116353 |
| Upregulated in class | ENSG00000116353\_neg |
| GeneSet | KEGG\_DORSO\_VENTRAL\_AXIS\_FORMATION |
| Enrichment Score (ES) | -0.80652946 |
| Normalized Enrichment Score (NES) | -2.4173024 |
| Nominal p-value | 0.0 |
| FDR q-value | 0.0 |
| FWER p-Value | 0.0 |
Table: GSEA Results Summary

  

Fig 1: Enrichment plot: KEGG\_DORSO\_VENTRAL\_AXIS\_FORMATION      
 Profile of the Running ES Score & Positions of GeneSet Members on the Rank Ordered List

  

| SYMBOL | TITLE | RANK IN GENE LIST | RANK METRIC SCORE | RUNNING ES | CORE ENRICHMENT || 1 | CPEB1 | cytoplasmic polyadenylation element binding protein 1 [Source:HGNC Symbol;Acc:HGNC:21744] | 11427 | 0.057 | -0.2767 | No |
| 2 | FMN2 | formin 2 [Source:HGNC Symbol;Acc:HGNC:14074] | 16265 | 0.024 | -0.3938 | No |
| 3 | PIWIL3 | piwi like RNA-mediated gene silencing 3 [Source:HGNC Symbol;Acc:HGNC:18443] | 18221 | 0.013 | -0.4403 | No |
| 4 | SPIRE2 | spire type actin nucleation factor 2 [Source:HGNC Symbol;Acc:HGNC:30623] | 18382 | 0.012 | -0.4413 | No |
| 5 | SOS1 | SOS Ras/Rac guanine nucleotide exchange factor 1 [Source:HGNC Symbol;Acc:HGNC:11187] | 18532 | 0.012 | -0.4423 | No |
| 6 | PIWIL1 | piwi like RNA-mediated gene silencing 1 [Source:HGNC Symbol;Acc:HGNC:9007] | 20437 | 0.001 | -0.4905 | No |
| 7 | MAPK1 | mitogen-activated protein kinase 1 [Source:HGNC Symbol;Acc:HGNC:6871] | 23167 | -0.015 | -0.5563 | No |
| 8 | NOTCH2 | notch receptor 2 [Source:HGNC Symbol;Acc:HGNC:7882] | 27409 | -0.045 | -0.6532 | No |
| 9 | NOTCH4 | notch receptor 4 [Source:HGNC Symbol;Acc:HGNC:7884] | 30604 | -0.080 | -0.7149 | No |
| 10 | ETS1 | "ETS proto-oncogene 1, transcription factor [Source:HGNC Symbol;Acc:HGNC:3488]" | 32607 | -0.111 | -0.7385 | No |
| 11 | PIWIL4 | piwi like RNA-mediated gene silencing 4 [Source:HGNC Symbol;Acc:HGNC:18444] | 35282 | -0.181 | -0.7621 | Yes |
| 12 | EGFR | epidermal growth factor receptor [Source:HGNC Symbol;Acc:HGNC:3236] | 35417 | -0.186 | -0.7198 | Yes |
| 13 | MAPK3 | mitogen-activated protein kinase 3 [Source:HGNC Symbol;Acc:HGNC:6877] | 35579 | -0.191 | -0.6769 | Yes |
| 14 | MAP2K1 | mitogen-activated protein kinase kinase 1 [Source:HGNC Symbol;Acc:HGNC:6840] | 36024 | -0.208 | -0.6369 | Yes |
| 15 | NOTCH3 | notch receptor 3 [Source:HGNC Symbol;Acc:HGNC:7883] | 36126 | -0.212 | -0.5872 | Yes |
| 16 | NOTCH1 | notch receptor 1 [Source:HGNC Symbol;Acc:HGNC:7881] | 36172 | -0.214 | -0.5356 | Yes |
| 17 | PIWIL2 | piwi like RNA-mediated gene silencing 2 [Source:HGNC Symbol;Acc:HGNC:17644] | 36181 | -0.215 | -0.4829 | Yes |
| 18 | GRB2 | growth factor receptor bound protein 2 [Source:HGNC Symbol;Acc:HGNC:4566] | 36523 | -0.231 | -0.4346 | Yes |
| 19 | ETV7 | ETS variant transcription factor 7 [Source:HGNC Symbol;Acc:HGNC:18160] | 36586 | -0.235 | -0.3784 | Yes |
| 20 | SPIRE1 | spire type actin nucleation factor 1 [Source:HGNC Symbol;Acc:HGNC:30622] | 36846 | -0.250 | -0.3235 | Yes |
| 21 | ETS2 | "ETS proto-oncogene 2, transcription factor [Source:HGNC Symbol;Acc:HGNC:3489]" | 37711 | -0.315 | -0.2679 | Yes |
| 22 | KRAS | "KRAS proto-oncogene, GTPase [Source:HGNC Symbol;Acc:HGNC:6407]" | 37789 | -0.323 | -0.1905 | Yes |
| 23 | ETV6 | ETS variant transcription factor 6 [Source:HGNC Symbol;Acc:HGNC:3495] | 38325 | -0.404 | -0.1046 | Yes |
| 24 | SOS2 | SOS Ras/Rho guanine nucleotide exchange factor 2 [Source:HGNC Symbol;Acc:HGNC:11188] | 38581 | -0.528 | 0.0189 | Yes |
Table: GSEA details [plain text format]

  

Fig 2: KEGG\_DORSO\_VENTRAL\_AXIS\_FORMATION      
 Blue-Pink O' Gram in the Space of the Analyzed GeneSet

  

Fig 3: KEGG\_DORSO\_VENTRAL\_AXIS\_FORMATION: Random ES distribution      
 Gene set null distribution of ES for **KEGG\_DORSO\_VENTRAL\_AXIS\_FORMATION**
