# Supplementary material for: Simultaneous Presentation of Multiple Myeloma and Lung Cancer: Case Report and Gene Bioinformatics Analysis
Source: Front Oncol. 2022 Jun 13;12:859735. doi: 10.3389/fonc.2022.859735 (PMC9235397; doi:10.3389/fonc.2022.859735)
Supplement: Supplementary file 1 [file DataSheet_1.zip › The bioinformatic analysis of MM and lung cancer supplementary materials/Enrichment analysis/MECR/GSEA_4.1.0/MM TCGA/KEGG.Gsea.1639040591792/KEGG_ENDOCYTOSIS.html]

Details for gene set KEGG\_ENDOCYTOSIS[GSEA]

|  || Dataset | ExpData\_collapsed\_to\_symbols.ENSG00000116353\_profile\_in\_ExpData.cls #ENSG00000116353 |
| Phenotype | ENSG00000116353\_profile\_in\_ExpData.cls#ENSG00000116353 |
| Upregulated in class | ENSG00000116353\_neg |
| GeneSet | KEGG\_ENDOCYTOSIS |
| Enrichment Score (ES) | -0.49444637 |
| Normalized Enrichment Score (NES) | -2.152375 |
| Nominal p-value | 0.0 |
| FDR q-value | 0.0 |
| FWER p-Value | 0.0 |
Table: GSEA Results Summary

  

Fig 1: Enrichment plot: KEGG\_ENDOCYTOSIS      
 Profile of the Running ES Score & Positions of GeneSet Members on the Rank Ordered List

  

| SYMBOL | TITLE | RANK IN GENE LIST | RANK METRIC SCORE | RUNNING ES | CORE ENRICHMENT || 1 | PIP4K2B | phosphatidylinositol-5-phosphate 4-kinase type 2 beta [Source:HGNC Symbol;Acc:HGNC:8998] | 458 | 0.421 | 0.0028 | No |
| 2 | VPS45 | vacuolar protein sorting 45 homolog [Source:HGNC Symbol;Acc:HGNC:14579] | 534 | 0.406 | 0.0150 | No |
| 3 | LDLRAP1 | low density lipoprotein receptor adaptor protein 1 [Source:HGNC Symbol;Acc:HGNC:18640] | 976 | 0.347 | 0.0157 | No |
| 4 | MVB12A | multivesicular body subunit 12A [Source:HGNC Symbol;Acc:HGNC:25153] | 1138 | 0.331 | 0.0230 | No |
| 5 | AP2M1 | adaptor related protein complex 2 subunit mu 1 [Source:HGNC Symbol;Acc:HGNC:564] | 1804 | 0.276 | 0.0155 | No |
| 6 | PSD4 | pleckstrin and Sec7 domain containing 4 [Source:HGNC Symbol;Acc:HGNC:19096] | 1862 | 0.272 | 0.0235 | No |
| 7 | AGAP2 | "ArfGAP with GTPase domain, ankyrin repeat and PH domain 2 [Source:HGNC Symbol;Acc:HGNC:16921]" | 2126 | 0.257 | 0.0256 | No |
| 8 | VPS25 | vacuolar protein sorting 25 homolog [Source:HGNC Symbol;Acc:HGNC:28122] | 2127 | 0.257 | 0.0345 | No |
| 9 | PARD6A | par-6 family cell polarity regulator alpha [Source:HGNC Symbol;Acc:HGNC:15943] | 2458 | 0.239 | 0.0343 | No |
| 10 | DNM3 | dynamin 3 [Source:HGNC Symbol;Acc:HGNC:29125] | 2878 | 0.219 | 0.0312 | No |
| 11 | CHMP5 | charged multivesicular body protein 5 [Source:HGNC Symbol;Acc:HGNC:26942] | 3122 | 0.208 | 0.0321 | No |
| 12 | HSPA8 | heat shock protein family A (Hsp70) member 8 [Source:HGNC Symbol;Acc:HGNC:5241] | 3186 | 0.205 | 0.0376 | No |
| 13 | EPN3 | epsin 3 [Source:HGNC Symbol;Acc:HGNC:18235] | 3454 | 0.195 | 0.0375 | No |
| 14 | AP2B1 | adaptor related protein complex 2 subunit beta 1 [Source:HGNC Symbol;Acc:HGNC:563] | 3669 | 0.187 | 0.0385 | No |
| 15 | STAM2 | signal transducing adaptor molecule 2 [Source:HGNC Symbol;Acc:HGNC:11358] | 3904 | 0.180 | 0.0387 | No |
| 16 | VPS36 | vacuolar protein sorting 36 homolog [Source:HGNC Symbol;Acc:HGNC:20312] | 3956 | 0.178 | 0.0436 | No |
| 17 | ADRB2 | adrenoceptor beta 2 [Source:HGNC Symbol;Acc:HGNC:286] | 4067 | 0.174 | 0.0468 | No |
| 18 | RUFY1 | RUN and FYVE domain containing 1 [Source:HGNC Symbol;Acc:HGNC:19760] | 4392 | 0.164 | 0.0442 | No |
| 19 | VTA1 | vesicle trafficking 1 [Source:HGNC Symbol;Acc:HGNC:20954] | 4424 | 0.164 | 0.0490 | No |
| 20 | RAB11FIP2 | RAB11 family interacting protein 2 [Source:HGNC Symbol;Acc:HGNC:29152] | 4449 | 0.163 | 0.0541 | No |
| 21 | CHMP6 | charged multivesicular body protein 6 [Source:HGNC Symbol;Acc:HGNC:25675] | 4543 | 0.160 | 0.0572 | No |
| 22 | PIP5K1A | phosphatidylinositol-4-phosphate 5-kinase type 1 alpha [Source:HGNC Symbol;Acc:HGNC:8994] | 5499 | 0.137 | 0.0376 | No |
| 23 | GRK6 | G protein-coupled receptor kinase 6 [Source:HGNC Symbol;Acc:HGNC:4545] | 5814 | 0.130 | 0.0340 | No |
| 24 | SH3GL2 | "SH3 domain containing GRB2 like 2, endophilin A1 [Source:HGNC Symbol;Acc:HGNC:10831]" | 5948 | 0.128 | 0.0351 | No |
| 25 | NTRK1 | neurotrophic receptor tyrosine kinase 1 [Source:HGNC Symbol;Acc:HGNC:8031] | 6517 | 0.118 | 0.0246 | No |
| 26 | FGFR4 | fibroblast growth factor receptor 4 [Source:HGNC Symbol;Acc:HGNC:3691] | 6714 | 0.114 | 0.0235 | No |
| 27 | AP2A1 | adaptor related protein complex 2 subunit alpha 1 [Source:HGNC Symbol;Acc:HGNC:561] | 6823 | 0.112 | 0.0247 | No |
| 28 | CLTA | clathrin light chain A [Source:HGNC Symbol;Acc:HGNC:2090] | 6963 | 0.110 | 0.0249 | No |
| 29 | PIP5K1B | phosphatidylinositol-4-phosphate 5-kinase type 1 beta [Source:HGNC Symbol;Acc:HGNC:8995] | 7022 | 0.109 | 0.0272 | No |
| 30 | STAMBP | STAM binding protein [Source:HGNC Symbol;Acc:HGNC:16950] | 7085 | 0.108 | 0.0293 | No |
| 31 | DNAJC6 | DnaJ heat shock protein family (Hsp40) member C6 [Source:HGNC Symbol;Acc:HGNC:15469] | 7087 | 0.108 | 0.0331 | No |
| 32 | PDCD6IP | programmed cell death 6 interacting protein [Source:HGNC Symbol;Acc:HGNC:8766] | 7221 | 0.106 | 0.0333 | No |
| 33 | MDM2 | MDM2 proto-oncogene [Source:HGNC Symbol;Acc:HGNC:6973] | 7403 | 0.103 | 0.0322 | No |
| 34 | GRK1 | G protein-coupled receptor kinase 1 [Source:HGNC Symbol;Acc:HGNC:10013] | 8060 | 0.094 | 0.0187 | No |
| 35 | PARD6G | par-6 family cell polarity regulator gamma [Source:HGNC Symbol;Acc:HGNC:16076] | 8265 | 0.091 | 0.0167 | No |
| 36 | GIT2 | GIT ArfGAP 2 [Source:HGNC Symbol;Acc:HGNC:4273] | 8792 | 0.085 | 0.0062 | No |
| 37 | PIP5K1C | phosphatidylinositol-4-phosphate 5-kinase type 1 gamma [Source:HGNC Symbol;Acc:HGNC:8996] | 9093 | 0.081 | 0.0013 | No |
| 38 | NEDD4 | NEDD4 E3 ubiquitin protein ligase [Source:HGNC Symbol;Acc:HGNC:7727] | 9508 | 0.076 | -0.0066 | No |
| 39 | VPS37C | VPS37C subunit of ESCRT-I [Source:HGNC Symbol;Acc:HGNC:26097] | 9844 | 0.073 | -0.0127 | No |
| 40 | AP2S1 | adaptor related protein complex 2 subunit sigma 1 [Source:HGNC Symbol;Acc:HGNC:565] | 11295 | 0.058 | -0.0477 | No |
| 41 | FGFR3 | fibroblast growth factor receptor 3 [Source:HGNC Symbol;Acc:HGNC:3690] | 11317 | 0.058 | -0.0463 | No |
| 42 | SH3GLB2 | "SH3 domain containing GRB2 like, endophilin B2 [Source:HGNC Symbol;Acc:HGNC:10834]" | 11519 | 0.056 | -0.0495 | No |
| 43 | AGAP1 | "ArfGAP with GTPase domain, ankyrin repeat and PH domain 1 [Source:HGNC Symbol;Acc:HGNC:16922]" | 11648 | 0.055 | -0.0508 | No |
| 44 | CBL | Cbl proto-oncogene [Source:HGNC Symbol;Acc:HGNC:1541] | 12468 | 0.049 | -0.0701 | No |
| 45 | HLA-C | "major histocompatibility complex, class I, C [Source:HGNC Symbol;Acc:HGNC:4933]" | 12475 | 0.049 | -0.0685 | No |
| 46 | EEA1 | early endosome antigen 1 [Source:HGNC Symbol;Acc:HGNC:3185] | 12502 | 0.049 | -0.0675 | No |
| 47 | RAB11FIP3 | RAB11 family interacting protein 3 [Source:HGNC Symbol;Acc:HGNC:17224] | 13476 | 0.041 | -0.0909 | No |
| 48 | IQSEC3 | IQ motif and Sec7 domain ArfGEF 3 [Source:HGNC Symbol;Acc:HGNC:29193] | 13816 | 0.039 | -0.0982 | No |
| 49 | DAB2 | DAB adaptor protein 2 [Source:HGNC Symbol;Acc:HGNC:2662] | 14026 | 0.038 | -0.1023 | No |
| 50 | ERBB4 | erb-b2 receptor tyrosine kinase 4 [Source:HGNC Symbol;Acc:HGNC:3432] | 14079 | 0.038 | -0.1023 | No |
| 51 | PRKCZ | protein kinase C zeta [Source:HGNC Symbol;Acc:HGNC:9412] | 14450 | 0.035 | -0.1105 | No |
| 52 | AP2A2 | adaptor related protein complex 2 subunit alpha 2 [Source:HGNC Symbol;Acc:HGNC:562] | 14677 | 0.034 | -0.1151 | No |
| 53 | CHMP2A | charged multivesicular body protein 2A [Source:HGNC Symbol;Acc:HGNC:30216] | 14982 | 0.032 | -0.1218 | No |
| 54 | ARAP1 | "ArfGAP with RhoGAP domain, ankyrin repeat and PH domain 1 [Source:HGNC Symbol;Acc:HGNC:16925]" | 15228 | 0.031 | -0.1270 | No |
| 55 | FLT1 | fms related receptor tyrosine kinase 1 [Source:HGNC Symbol;Acc:HGNC:3763] | 16066 | 0.025 | -0.1475 | No |
| 56 | RAB4A | "RAB4A, member RAS oncogene family [Source:HGNC Symbol;Acc:HGNC:9781]" | 16089 | 0.025 | -0.1472 | No |
| 57 | IGF1R | insulin like growth factor 1 receptor [Source:HGNC Symbol;Acc:HGNC:5465] | 17189 | 0.019 | -0.1746 | No |
| 58 | CBLC | Cbl proto-oncogene C [Source:HGNC Symbol;Acc:HGNC:15961] | 18396 | 0.012 | -0.2050 | No |
| 59 | RAB11A | "RAB11A, member RAS oncogene family [Source:HGNC Symbol;Acc:HGNC:9760]" | 19013 | 0.009 | -0.2204 | No |
| 60 | HLA-G | "major histocompatibility complex, class I, G [Source:HGNC Symbol;Acc:HGNC:4964]" | 19374 | 0.007 | -0.2294 | No |
| 61 | DNM1 | dynamin 1 [Source:HGNC Symbol;Acc:HGNC:2972] | 19520 | 0.006 | -0.2329 | No |
| 62 | SNF8 | SNF8 subunit of ESCRT-II [Source:HGNC Symbol;Acc:HGNC:17028] | 20178 | 0.003 | -0.2496 | No |
| 63 | EPN2 | epsin 2 [Source:HGNC Symbol;Acc:HGNC:18639] | 20361 | 0.001 | -0.2542 | No |
| 64 | TFRC | transferrin receptor [Source:HGNC Symbol;Acc:HGNC:11763] | 20952 | -0.002 | -0.2692 | No |
| 65 | GRK4 | G protein-coupled receptor kinase 4 [Source:HGNC Symbol;Acc:HGNC:4543] | 21101 | -0.003 | -0.2729 | No |
| 66 | ADRB3 | adrenoceptor beta 3 [Source:HGNC Symbol;Acc:HGNC:288] | 21890 | -0.007 | -0.2928 | No |
| 67 | RET | ret proto-oncogene [Source:HGNC Symbol;Acc:HGNC:9967] | 23476 | -0.017 | -0.3327 | No |
| 68 | CCR5 | C-C motif chemokine receptor 5 [Source:HGNC Symbol;Acc:HGNC:1606] | 23927 | -0.020 | -0.3435 | No |
| 69 | ADRB1 | adrenoceptor beta 1 [Source:HGNC Symbol;Acc:HGNC:285] | 24251 | -0.022 | -0.3510 | No |
| 70 | EHD3 | EH domain containing 3 [Source:HGNC Symbol;Acc:HGNC:3244] | 24346 | -0.022 | -0.3526 | No |
| 71 | ACAP1 | "ArfGAP with coiled-coil, ankyrin repeat and PH domains 1 [Source:HGNC Symbol;Acc:HGNC:16467]" | 24673 | -0.024 | -0.3601 | No |
| 72 | PRKCI | protein kinase C iota [Source:HGNC Symbol;Acc:HGNC:9404] | 24961 | -0.026 | -0.3666 | No |
| 73 | MET | "MET proto-oncogene, receptor tyrosine kinase [Source:HGNC Symbol;Acc:HGNC:7029]" | 25533 | -0.030 | -0.3801 | No |
| 74 | CLTCL1 | clathrin heavy chain like 1 [Source:HGNC Symbol;Acc:HGNC:2093] | 25545 | -0.030 | -0.3793 | No |
| 75 | PSD3 | pleckstrin and Sec7 domain containing 3 [Source:HGNC Symbol;Acc:HGNC:19093] | 25968 | -0.034 | -0.3890 | No |
| 76 | ACAP3 | "ArfGAP with coiled-coil, ankyrin repeat and PH domains 3 [Source:HGNC Symbol;Acc:HGNC:16754]" | 26076 | -0.034 | -0.3905 | No |
| 77 | HRAS | "HRas proto-oncogene, GTPase [Source:HGNC Symbol;Acc:HGNC:5173]" | 26672 | -0.039 | -0.4044 | No |
| 78 | WWP1 | WW domain containing E3 ubiquitin protein ligase 1 [Source:HGNC Symbol;Acc:HGNC:17004] | 26845 | -0.040 | -0.4074 | No |
| 79 | VPS28 | VPS28 subunit of ESCRT-I [Source:HGNC Symbol;Acc:HGNC:18178] | 26913 | -0.040 | -0.4077 | No |
| 80 | RABEP1 | "rabaptin, RAB GTPase binding effector protein 1 [Source:HGNC Symbol;Acc:HGNC:17677]" | 27031 | -0.041 | -0.4093 | No |
| 81 | KIT | "KIT proto-oncogene, receptor tyrosine kinase [Source:HGNC Symbol;Acc:HGNC:6342]" | 27060 | -0.042 | -0.4085 | No |
| 82 | VPS4A | vacuolar protein sorting 4 homolog A [Source:HGNC Symbol;Acc:HGNC:13488] | 27287 | -0.044 | -0.4128 | No |
| 83 | SH3GL3 | "SH3 domain containing GRB2 like 3, endophilin A3 [Source:HGNC Symbol;Acc:HGNC:10832]" | 27774 | -0.048 | -0.4236 | No |
| 84 | F2R | coagulation factor II thrombin receptor [Source:HGNC Symbol;Acc:HGNC:3537] | 28542 | -0.055 | -0.4412 | No |
| 85 | PLD1 | phospholipase D1 [Source:HGNC Symbol;Acc:HGNC:9067] | 28735 | -0.058 | -0.4442 | No |
| 86 | HLA-B | "major histocompatibility complex, class I, B [Source:HGNC Symbol;Acc:HGNC:4932]" | 28822 | -0.058 | -0.4443 | No |
| 87 | DNM1L | dynamin 1 like [Source:HGNC Symbol;Acc:HGNC:2973] | 28911 | -0.059 | -0.4445 | No |
| 88 | ARFGAP2 | ADP ribosylation factor GTPase activating protein 2 [Source:HGNC Symbol;Acc:HGNC:13504] | 29135 | -0.062 | -0.4481 | No |
| 89 | CLTB | clathrin light chain B [Source:HGNC Symbol;Acc:HGNC:2091] | 29138 | -0.062 | -0.4460 | No |
| 90 | RAB5B | "RAB5B, member RAS oncogene family [Source:HGNC Symbol;Acc:HGNC:9784]" | 29358 | -0.065 | -0.4494 | No |
| 91 | PARD3 | par-3 family cell polarity regulator [Source:HGNC Symbol;Acc:HGNC:16051] | 29993 | -0.072 | -0.4631 | No |
| 92 | VPS37A | VPS37A subunit of ESCRT-I [Source:HGNC Symbol;Acc:HGNC:24928] | 30226 | -0.075 | -0.4664 | No |
| 93 | IL2RB | interleukin 2 receptor subunit beta [Source:HGNC Symbol;Acc:HGNC:6009] | 30287 | -0.076 | -0.4653 | No |
| 94 | ERBB3 | erb-b2 receptor tyrosine kinase 3 [Source:HGNC Symbol;Acc:HGNC:3431] | 30457 | -0.078 | -0.4670 | No |
| 95 | PIKFYVE | "phosphoinositide kinase, FYVE-type zinc finger containing [Source:HGNC Symbol;Acc:HGNC:23785]" | 30696 | -0.081 | -0.4703 | No |
| 96 | CLTC | clathrin heavy chain [Source:HGNC Symbol;Acc:HGNC:2092] | 30796 | -0.082 | -0.4700 | No |
| 97 | HLA-A | "major histocompatibility complex, class I, A [Source:HGNC Symbol;Acc:HGNC:4931]" | 30839 | -0.083 | -0.4682 | No |
| 98 | ASAP3 | "ArfGAP with SH3 domain, ankyrin repeat and PH domain 3 [Source:HGNC Symbol;Acc:HGNC:14987]" | 30935 | -0.084 | -0.4677 | No |
| 99 | RNF41 | ring finger protein 41 [Source:HGNC Symbol;Acc:HGNC:18401] | 31343 | -0.089 | -0.4750 | No |
| 100 | CXCR2 | C-X-C motif chemokine receptor 2 [Source:HGNC Symbol;Acc:HGNC:6027] | 31411 | -0.090 | -0.4736 | No |
| 101 | ASAP2 | "ArfGAP with SH3 domain, ankyrin repeat and PH domain 2 [Source:HGNC Symbol;Acc:HGNC:2721]" | 31994 | -0.100 | -0.4850 | No |
| 102 | VPS37B | VPS37B subunit of ESCRT-I [Source:HGNC Symbol;Acc:HGNC:25754] | 32281 | -0.105 | -0.4887 | No |
| 103 | HLA-F | "major histocompatibility complex, class I, F [Source:HGNC Symbol;Acc:HGNC:4963]" | 32365 | -0.106 | -0.4872 | No |
| 104 | FGFR2 | fibroblast growth factor receptor 2 [Source:HGNC Symbol;Acc:HGNC:3689] | 32385 | -0.107 | -0.4840 | No |
| 105 | GRK7 | G protein-coupled receptor kinase 7 [Source:HGNC Symbol;Acc:HGNC:17031] | 32503 | -0.109 | -0.4832 | No |
| 106 | RAB11FIP1 | RAB11 family interacting protein 1 [Source:HGNC Symbol;Acc:HGNC:30265] | 32614 | -0.111 | -0.4822 | No |
| 107 | PSD | pleckstrin and Sec7 domain containing [Source:HGNC Symbol;Acc:HGNC:9507] | 32654 | -0.112 | -0.4793 | No |
| 108 | GIT1 | GIT ArfGAP 1 [Source:HGNC Symbol;Acc:HGNC:4272] | 32673 | -0.112 | -0.4759 | No |
| 109 | CSF1R | colony stimulating factor 1 receptor [Source:HGNC Symbol;Acc:HGNC:2433] | 33122 | -0.121 | -0.4831 | No |
| 110 | EGF | epidermal growth factor [Source:HGNC Symbol;Acc:HGNC:3229] | 33135 | -0.121 | -0.4792 | No |
| 111 | CHMP1B | charged multivesicular body protein 1B [Source:HGNC Symbol;Acc:HGNC:24287] | 33360 | -0.126 | -0.4806 | No |
| 112 | EPN1 | epsin 1 [Source:HGNC Symbol;Acc:HGNC:21604] | 33628 | -0.132 | -0.4829 | No |
| 113 | IL2RG | interleukin 2 receptor subunit gamma [Source:HGNC Symbol;Acc:HGNC:6010] | 33828 | -0.137 | -0.4832 | No |
| 114 | MVB12B | multivesicular body subunit 12B [Source:HGNC Symbol;Acc:HGNC:23368] | 34082 | -0.143 | -0.4847 | No |
| 115 | SRC | "SRC proto-oncogene, non-receptor tyrosine kinase [Source:HGNC Symbol;Acc:HGNC:11283]" | 34463 | -0.155 | -0.4891 | Yes |
| 116 | NEDD4L | NEDD4 like E3 ubiquitin protein ligase [Source:HGNC Symbol;Acc:HGNC:7728] | 34539 | -0.157 | -0.4856 | Yes |
| 117 | SH3KBP1 | SH3 domain containing kinase binding protein 1 [Source:HGNC Symbol;Acc:HGNC:13867] | 34540 | -0.157 | -0.4802 | Yes |
| 118 | GRK3 | G protein-coupled receptor kinase 3 [Source:HGNC Symbol;Acc:HGNC:290] | 34666 | -0.161 | -0.4778 | Yes |
| 119 | KDR | kinase insert domain receptor [Source:HGNC Symbol;Acc:HGNC:6307] | 34689 | -0.161 | -0.4728 | Yes |
| 120 | CXCR1 | C-X-C motif chemokine receptor 1 [Source:HGNC Symbol;Acc:HGNC:6026] | 34719 | -0.162 | -0.4680 | Yes |
| 121 | GRK5 | G protein-coupled receptor kinase 5 [Source:HGNC Symbol;Acc:HGNC:4544] | 34721 | -0.162 | -0.4624 | Yes |
| 122 | DNM2 | dynamin 2 [Source:HGNC Symbol;Acc:HGNC:2974] | 34825 | -0.165 | -0.4593 | Yes |
| 123 | HSPA2 | heat shock protein family A (Hsp70) member 2 [Source:HGNC Symbol;Acc:HGNC:5235] | 34877 | -0.167 | -0.4548 | Yes |
| 124 | CHMP4C | charged multivesicular body protein 4C [Source:HGNC Symbol;Acc:HGNC:30599] | 34957 | -0.169 | -0.4510 | Yes |
| 125 | ARRB1 | arrestin beta 1 [Source:HGNC Symbol;Acc:HGNC:711] | 34997 | -0.171 | -0.4461 | Yes |
| 126 | HSPA1L | heat shock protein family A (Hsp70) member 1 like [Source:HGNC Symbol;Acc:HGNC:5234] | 35112 | -0.175 | -0.4430 | Yes |
| 127 | ARFGAP3 | ADP ribosylation factor GTPase activating protein 3 [Source:HGNC Symbol;Acc:HGNC:661] | 35211 | -0.178 | -0.4393 | Yes |
| 128 | CHMP4A | charged multivesicular body protein 4A [Source:HGNC Symbol;Acc:HGNC:20274] | 35224 | -0.179 | -0.4335 | Yes |
| 129 | ACAP2 | "ArfGAP with coiled-coil, ankyrin repeat and PH domains 2 [Source:HGNC Symbol;Acc:HGNC:16469]" | 35323 | -0.182 | -0.4297 | Yes |
| 130 | EGFR | epidermal growth factor receptor [Source:HGNC Symbol;Acc:HGNC:3236] | 35417 | -0.186 | -0.4257 | Yes |
| 131 | EHD2 | EH domain containing 2 [Source:HGNC Symbol;Acc:HGNC:3243] | 35492 | -0.188 | -0.4211 | Yes |
| 132 | PSD2 | pleckstrin and Sec7 domain containing 2 [Source:HGNC Symbol;Acc:HGNC:19092] | 35515 | -0.189 | -0.4151 | Yes |
| 133 | ARRB2 | arrestin beta 2 [Source:HGNC Symbol;Acc:HGNC:712] | 35527 | -0.189 | -0.4088 | Yes |
| 134 | HSPA1B | heat shock protein family A (Hsp70) member 1B [Source:HGNC Symbol;Acc:HGNC:5233] | 35575 | -0.191 | -0.4035 | Yes |
| 135 | RAB11FIP5 | RAB11 family interacting protein 5 [Source:HGNC Symbol;Acc:HGNC:24845] | 35713 | -0.196 | -0.4002 | Yes |
| 136 | CBLB | Cbl proto-oncogene B [Source:HGNC Symbol;Acc:HGNC:1542] | 35750 | -0.197 | -0.3943 | Yes |
| 137 | VPS37D | VPS37D subunit of ESCRT-I [Source:HGNC Symbol;Acc:HGNC:18287] | 35784 | -0.199 | -0.3882 | Yes |
| 138 | TSG101 | tumor susceptibility 101 [Source:HGNC Symbol;Acc:HGNC:15971] | 35875 | -0.202 | -0.3836 | Yes |
| 139 | LDLR | low density lipoprotein receptor [Source:HGNC Symbol;Acc:HGNC:6547] | 35939 | -0.205 | -0.3781 | Yes |
| 140 | SMAP1 | small ArfGAP 1 [Source:HGNC Symbol;Acc:HGNC:19651] | 35950 | -0.205 | -0.3712 | Yes |
| 141 | ARAP3 | "ArfGAP with RhoGAP domain, ankyrin repeat and PH domain 3 [Source:HGNC Symbol;Acc:HGNC:24097]" | 36054 | -0.209 | -0.3666 | Yes |
| 142 | ARF6 | ADP ribosylation factor 6 [Source:HGNC Symbol;Acc:HGNC:659] | 36070 | -0.210 | -0.3598 | Yes |
| 143 | IL2RA | interleukin 2 receptor subunit alpha [Source:HGNC Symbol;Acc:HGNC:6008] | 36108 | -0.212 | -0.3534 | Yes |
| 144 | ARAP2 | "ArfGAP with RhoGAP domain, ankyrin repeat and PH domain 2 [Source:HGNC Symbol;Acc:HGNC:16924]" | 36343 | -0.222 | -0.3517 | Yes |
| 145 | CHMP3 | charged multivesicular body protein 3 [Source:HGNC Symbol;Acc:HGNC:29865] | 36455 | -0.228 | -0.3467 | Yes |
| 146 | PDGFRA | platelet derived growth factor receptor alpha [Source:HGNC Symbol;Acc:HGNC:8803] | 36521 | -0.231 | -0.3403 | Yes |
| 147 | IQSEC2 | IQ motif and Sec7 domain ArfGEF 2 [Source:HGNC Symbol;Acc:HGNC:29059] | 36617 | -0.237 | -0.3346 | Yes |
| 148 | SH3GL1 | "SH3 domain containing GRB2 like 1, endophilin A2 [Source:HGNC Symbol;Acc:HGNC:10830]" | 36741 | -0.244 | -0.3293 | Yes |
| 149 | RBSN | "rabenosyn, RAB effector [Source:HGNC Symbol;Acc:HGNC:20759]" | 36752 | -0.244 | -0.3211 | Yes |
| 150 | EHD4 | EH domain containing 4 [Source:HGNC Symbol;Acc:HGNC:3245] | 36782 | -0.245 | -0.3134 | Yes |
| 151 | CXCR4 | C-X-C motif chemokine receptor 4 [Source:HGNC Symbol;Acc:HGNC:2561] | 36861 | -0.251 | -0.3067 | Yes |
| 152 | HSPA6 | heat shock protein family A (Hsp70) member 6 [Source:HGNC Symbol;Acc:HGNC:5239] | 36867 | -0.251 | -0.2982 | Yes |
| 153 | HLA-E | "major histocompatibility complex, class I, E [Source:HGNC Symbol;Acc:HGNC:4962]" | 36918 | -0.254 | -0.2907 | Yes |
| 154 | RAB31 | "RAB31, member RAS oncogene family [Source:HGNC Symbol;Acc:HGNC:9771]" | 36996 | -0.259 | -0.2837 | Yes |
| 155 | VPS4B | vacuolar protein sorting 4 homolog B [Source:HGNC Symbol;Acc:HGNC:10895] | 37007 | -0.260 | -0.2749 | Yes |
| 156 | EPS15 | epidermal growth factor receptor pathway substrate 15 [Source:HGNC Symbol;Acc:HGNC:3419] | 37058 | -0.263 | -0.2671 | Yes |
| 157 | ITCH | itchy E3 ubiquitin protein ligase [Source:HGNC Symbol;Acc:HGNC:13890] | 37181 | -0.271 | -0.2609 | Yes |
| 158 | RAB11B | "RAB11B, member RAS oncogene family [Source:HGNC Symbol;Acc:HGNC:9761]" | 37191 | -0.272 | -0.2517 | Yes |
| 159 | IQSEC1 | IQ motif and Sec7 domain ArfGEF 1 [Source:HGNC Symbol;Acc:HGNC:29112] | 37305 | -0.280 | -0.2449 | Yes |
| 160 | HSPA1A | heat shock protein family A (Hsp70) member 1A [Source:HGNC Symbol;Acc:HGNC:5232] | 37351 | -0.284 | -0.2363 | Yes |
| 161 | CHMP2B | charged multivesicular body protein 2B [Source:HGNC Symbol;Acc:HGNC:24537] | 37398 | -0.288 | -0.2275 | Yes |
| 162 | ARFGAP1 | ADP ribosylation factor GTPase activating protein 1 [Source:HGNC Symbol;Acc:HGNC:15852] | 37422 | -0.290 | -0.2181 | Yes |
| 163 | PARD6B | par-6 family cell polarity regulator beta [Source:HGNC Symbol;Acc:HGNC:16245] | 37481 | -0.295 | -0.2094 | Yes |
| 164 | CDC42 | cell division cycle 42 [Source:HGNC Symbol;Acc:HGNC:1736] | 37805 | -0.324 | -0.2064 | Yes |
| 165 | HGS | hepatocyte growth factor-regulated tyrosine kinase substrate [Source:HGNC Symbol;Acc:HGNC:4897] | 37831 | -0.329 | -0.1957 | Yes |
| 166 | GRK2 | G protein-coupled receptor kinase 2 [Source:HGNC Symbol;Acc:HGNC:289] | 37914 | -0.338 | -0.1861 | Yes |
| 167 | PLD2 | phospholipase D2 [Source:HGNC Symbol;Acc:HGNC:9068] | 38178 | -0.373 | -0.1800 | Yes |
| 168 | TRAF6 | TNF receptor associated factor 6 [Source:HGNC Symbol;Acc:HGNC:12036] | 38202 | -0.379 | -0.1675 | Yes |
| 169 | RAB5C | "RAB5C, member RAS oncogene family [Source:HGNC Symbol;Acc:HGNC:9785]" | 38237 | -0.386 | -0.1550 | Yes |
| 170 | ASAP1 | "ArfGAP with SH3 domain, ankyrin repeat and PH domain 1 [Source:HGNC Symbol;Acc:HGNC:2720]" | 38238 | -0.386 | -0.1417 | Yes |
| 171 | RAB22A | "RAB22A, member RAS oncogene family [Source:HGNC Symbol;Acc:HGNC:9764]" | 38273 | -0.392 | -0.1290 | Yes |
| 172 | STAM | signal transducing adaptor molecule [Source:HGNC Symbol;Acc:HGNC:11357] | 38348 | -0.408 | -0.1168 | Yes |
| 173 | EHD1 | EH domain containing 1 [Source:HGNC Symbol;Acc:HGNC:3242] | 38364 | -0.411 | -0.1030 | Yes |
| 174 | SH3GLB1 | "SH3 domain containing GRB2 like, endophilin B1 [Source:HGNC Symbol;Acc:HGNC:10833]" | 38430 | -0.429 | -0.0898 | Yes |
| 175 | CHMP4B | charged multivesicular body protein 4B [Source:HGNC Symbol;Acc:HGNC:16171] | 38462 | -0.440 | -0.0754 | Yes |
| 176 | USP8 | ubiquitin specific peptidase 8 [Source:HGNC Symbol;Acc:HGNC:12631] | 38463 | -0.441 | -0.0602 | Yes |
| 177 | RAB11FIP4 | RAB11 family interacting protein 4 [Source:HGNC Symbol;Acc:HGNC:30267] | 38469 | -0.443 | -0.0450 | Yes |
| 178 | RAB5A | "RAB5A, member RAS oncogene family [Source:HGNC Symbol;Acc:HGNC:9783]" | 38528 | -0.468 | -0.0304 | Yes |
| 179 | SMURF1 | SMAD specific E3 ubiquitin protein ligase 1 [Source:HGNC Symbol;Acc:HGNC:16807] | 38541 | -0.473 | -0.0143 | Yes |
| 180 | SMURF2 | SMAD specific E3 ubiquitin protein ligase 2 [Source:HGNC Symbol;Acc:HGNC:16809] | 38559 | -0.492 | 0.0022 | Yes |
| 181 | SMAP2 | small ArfGAP2 [Source:HGNC Symbol;Acc:HGNC:25082] | 38567 | -0.500 | 0.0193 | Yes |
Table: GSEA details [plain text format]

  

Fig 2: KEGG\_ENDOCYTOSIS      
 Blue-Pink O' Gram in the Space of the Analyzed GeneSet

  

Fig 3: KEGG\_ENDOCYTOSIS: Random ES distribution      
 Gene set null distribution of ES for **KEGG\_ENDOCYTOSIS**
