# Supplementary material for: Simultaneous Presentation of Multiple Myeloma and Lung Cancer: Case Report and Gene Bioinformatics Analysis
Source: Front Oncol. 2022 Jun 13;12:859735. doi: 10.3389/fonc.2022.859735 (PMC9235397; doi:10.3389/fonc.2022.859735)
Supplement: Supplementary file 1 [file DataSheet_1.zip › The bioinformatic analysis of MM and lung cancer supplementary materials/Enrichment analysis/MECR/GSEA_4.1.0/MM TCGA/KEGG.Gsea.1639040591792/KEGG_FC_GAMMA_R_MEDIATED_PHAGOCYTOSIS.html]

Details for gene set KEGG\_FC\_GAMMA\_R\_MEDIATED\_PHAGOCYTOSIS[GSEA]

|  || Dataset | ExpData\_collapsed\_to\_symbols.ENSG00000116353\_profile\_in\_ExpData.cls #ENSG00000116353 |
| Phenotype | ENSG00000116353\_profile\_in\_ExpData.cls#ENSG00000116353 |
| Upregulated in class | ENSG00000116353\_neg |
| GeneSet | KEGG\_FC\_GAMMA\_R\_MEDIATED\_PHAGOCYTOSIS |
| Enrichment Score (ES) | -0.5361351 |
| Normalized Enrichment Score (NES) | -2.1435275 |
| Nominal p-value | 0.0 |
| FDR q-value | 0.0 |
| FWER p-Value | 0.0 |
Table: GSEA Results Summary

  

Fig 1: Enrichment plot: KEGG\_FC\_GAMMA\_R\_MEDIATED\_PHAGOCYTOSIS      
 Profile of the Running ES Score & Positions of GeneSet Members on the Rank Ordered List

  

| SYMBOL | TITLE | RANK IN GENE LIST | RANK METRIC SCORE | RUNNING ES | CORE ENRICHMENT || 1 | SPHK2 | sphingosine kinase 2 [Source:HGNC Symbol;Acc:HGNC:18859] | 132 | 0.510 | 0.0293 | No |
| 2 | PIP4K2B | phosphatidylinositol-5-phosphate 4-kinase type 2 beta [Source:HGNC Symbol;Acc:HGNC:8998] | 458 | 0.421 | 0.0479 | No |
| 3 | PIK3R2 | phosphoinositide-3-kinase regulatory subunit 2 [Source:HGNC Symbol;Acc:HGNC:8980] | 1248 | 0.320 | 0.0483 | No |
| 4 | PIK3R3 | phosphoinositide-3-kinase regulatory subunit 3 [Source:HGNC Symbol;Acc:HGNC:8981] | 2602 | 0.231 | 0.0286 | No |
| 5 | DNM3 | dynamin 3 [Source:HGNC Symbol;Acc:HGNC:29125] | 2878 | 0.219 | 0.0356 | No |
| 6 | VAV1 | vav guanine nucleotide exchange factor 1 [Source:HGNC Symbol;Acc:HGNC:12657] | 3764 | 0.184 | 0.0248 | No |
| 7 | PLA2G4A | phospholipase A2 group IVA [Source:HGNC Symbol;Acc:HGNC:9035] | 4721 | 0.155 | 0.0104 | No |
| 8 | PLA2G6 | phospholipase A2 group VI [Source:HGNC Symbol;Acc:HGNC:9039] | 5302 | 0.142 | 0.0047 | No |
| 9 | PIP5K1A | phosphatidylinositol-4-phosphate 5-kinase type 1 alpha [Source:HGNC Symbol;Acc:HGNC:8994] | 5499 | 0.137 | 0.0084 | No |
| 10 | RAC2 | Rac family small GTPase 2 [Source:HGNC Symbol;Acc:HGNC:9802] | 6127 | 0.124 | 0.0004 | No |
| 11 | ARPC1A | actin related protein 2/3 complex subunit 1A [Source:HGNC Symbol;Acc:HGNC:703] | 6397 | 0.120 | 0.0012 | No |
| 12 | PIP5K1B | phosphatidylinositol-4-phosphate 5-kinase type 1 beta [Source:HGNC Symbol;Acc:HGNC:8995] | 7022 | 0.109 | -0.0077 | No |
| 13 | PIK3CB | "phosphatidylinositol-4,5-bisphosphate 3-kinase catalytic subunit beta [Source:HGNC Symbol;Acc:HGNC:8976]" | 7716 | 0.099 | -0.0191 | No |
| 14 | ARPC5 | actin related protein 2/3 complex subunit 5 [Source:HGNC Symbol;Acc:HGNC:708] | 8270 | 0.091 | -0.0273 | No |
| 15 | PLPP1 | phospholipid phosphatase 1 [Source:HGNC Symbol;Acc:HGNC:9228] | 8800 | 0.085 | -0.0354 | No |
| 16 | PIP5K1C | phosphatidylinositol-4-phosphate 5-kinase type 1 gamma [Source:HGNC Symbol;Acc:HGNC:8996] | 9093 | 0.081 | -0.0376 | No |
| 17 | ARPC1B | actin related protein 2/3 complex subunit 1B [Source:HGNC Symbol;Acc:HGNC:704] | 10278 | 0.068 | -0.0635 | No |
| 18 | PRKCB | protein kinase C beta [Source:HGNC Symbol;Acc:HGNC:9395] | 11760 | 0.054 | -0.0978 | No |
| 19 | CRK | "CRK proto-oncogene, adaptor protein [Source:HGNC Symbol;Acc:HGNC:2362]" | 14915 | 0.032 | -0.1761 | No |
| 20 | WASF1 | WASP family member 1 [Source:HGNC Symbol;Acc:HGNC:12732] | 16267 | 0.024 | -0.2090 | No |
| 21 | SCIN | scinderin [Source:HGNC Symbol;Acc:HGNC:21695] | 17104 | 0.019 | -0.2290 | No |
| 22 | PRKCD | protein kinase C delta [Source:HGNC Symbol;Acc:HGNC:9399] | 17659 | 0.016 | -0.2421 | No |
| 23 | WAS | WASP actin nucleation promoting factor [Source:HGNC Symbol;Acc:HGNC:12731] | 17936 | 0.015 | -0.2482 | No |
| 24 | DNM1 | dynamin 1 [Source:HGNC Symbol;Acc:HGNC:2972] | 19520 | 0.006 | -0.2881 | No |
| 25 | ARPC3 | actin related protein 2/3 complex subunit 3 [Source:HGNC Symbol;Acc:HGNC:706] | 19525 | 0.006 | -0.2879 | No |
| 26 | AMPH | amphiphysin [Source:HGNC Symbol;Acc:HGNC:471] | 20899 | -0.001 | -0.3228 | No |
| 27 | PLA2G4F | phospholipase A2 group IVF [Source:HGNC Symbol;Acc:HGNC:27396] | 21474 | -0.005 | -0.3371 | No |
| 28 | PLA2G4E | phospholipase A2 group IVE [Source:HGNC Symbol;Acc:HGNC:24791] | 22141 | -0.009 | -0.3535 | No |
| 29 | MARCKSL1 | MARCKS like 1 [Source:HGNC Symbol;Acc:HGNC:7142] | 22171 | -0.009 | -0.3537 | No |
| 30 | LAT | linker for activation of T cells [Source:HGNC Symbol;Acc:HGNC:18874] | 22716 | -0.012 | -0.3668 | No |
| 31 | MAPK1 | mitogen-activated protein kinase 1 [Source:HGNC Symbol;Acc:HGNC:6871] | 23167 | -0.015 | -0.3773 | No |
| 32 | FCGR2B | Fc fragment of IgG receptor IIb [Source:HGNC Symbol;Acc:HGNC:3618] | 24767 | -0.025 | -0.4165 | No |
| 33 | PLA2G4D | phospholipase A2 group IVD [Source:HGNC Symbol;Acc:HGNC:30038] | 25156 | -0.027 | -0.4246 | No |
| 34 | DOCK2 | dedicator of cytokinesis 2 [Source:HGNC Symbol;Acc:HGNC:2988] | 26543 | -0.038 | -0.4575 | No |
| 35 | RPS6KB2 | ribosomal protein S6 kinase B2 [Source:HGNC Symbol;Acc:HGNC:10437] | 27292 | -0.044 | -0.4738 | No |
| 36 | WASF3 | WASP family member 3 [Source:HGNC Symbol;Acc:HGNC:12734] | 28144 | -0.052 | -0.4922 | No |
| 37 | PLD1 | phospholipase D1 [Source:HGNC Symbol;Acc:HGNC:9067] | 28735 | -0.058 | -0.5036 | No |
| 38 | FCGR2C | Fc fragment of IgG receptor IIc (gene/pseudogene) [Source:HGNC Symbol;Acc:HGNC:15626] | 28906 | -0.059 | -0.5041 | No |
| 39 | DNM1L | dynamin 1 like [Source:HGNC Symbol;Acc:HGNC:2973] | 28911 | -0.059 | -0.5004 | No |
| 40 | FCGR3A | Fc fragment of IgG receptor IIIa [Source:HGNC Symbol;Acc:HGNC:3619] | 29324 | -0.064 | -0.5068 | No |
| 41 | VAV3 | vav guanine nucleotide exchange factor 3 [Source:HGNC Symbol;Acc:HGNC:12659] | 29349 | -0.065 | -0.5033 | No |
| 42 | PLPP2 | phospholipid phosphatase 2 [Source:HGNC Symbol;Acc:HGNC:9230] | 29588 | -0.067 | -0.5050 | No |
| 43 | PIKFYVE | "phosphoinositide kinase, FYVE-type zinc finger containing [Source:HGNC Symbol;Acc:HGNC:23785]" | 30696 | -0.081 | -0.5281 | No |
| 44 | ASAP3 | "ArfGAP with SH3 domain, ankyrin repeat and PH domain 3 [Source:HGNC Symbol;Acc:HGNC:14987]" | 30935 | -0.084 | -0.5288 | No |
| 45 | CFL1 | cofilin 1 [Source:HGNC Symbol;Acc:HGNC:1874] | 31054 | -0.086 | -0.5263 | No |
| 46 | PLPP3 | phospholipid phosphatase 3 [Source:HGNC Symbol;Acc:HGNC:9229] | 31439 | -0.091 | -0.5303 | Yes |
| 47 | PLA2G4B | phospholipase A2 group IVB [Source:HGNC Symbol;Acc:HGNC:9036] | 31589 | -0.093 | -0.5282 | Yes |
| 48 | MYO10 | myosin X [Source:HGNC Symbol;Acc:HGNC:7593] | 31756 | -0.096 | -0.5263 | Yes |
| 49 | AKT1 | AKT serine/threonine kinase 1 [Source:HGNC Symbol;Acc:HGNC:391] | 31906 | -0.098 | -0.5238 | Yes |
| 50 | ASAP2 | "ArfGAP with SH3 domain, ankyrin repeat and PH domain 2 [Source:HGNC Symbol;Acc:HGNC:2721]" | 31994 | -0.100 | -0.5196 | Yes |
| 51 | PLCG2 | phospholipase C gamma 2 [Source:HGNC Symbol;Acc:HGNC:9066] | 32294 | -0.105 | -0.5205 | Yes |
| 52 | FCGR1A | Fc fragment of IgG receptor Ia [Source:HGNC Symbol;Acc:HGNC:3613] | 32623 | -0.111 | -0.5218 | Yes |
| 53 | PRKCA | protein kinase C alpha [Source:HGNC Symbol;Acc:HGNC:9393] | 33017 | -0.119 | -0.5242 | Yes |
| 54 | ARPC5L | actin related protein 2/3 complex subunit 5 like [Source:HGNC Symbol;Acc:HGNC:23366] | 33127 | -0.121 | -0.5192 | Yes |
| 55 | AKT2 | AKT serine/threonine kinase 2 [Source:HGNC Symbol;Acc:HGNC:392] | 33213 | -0.123 | -0.5135 | Yes |
| 56 | SYK | spleen associated tyrosine kinase [Source:HGNC Symbol;Acc:HGNC:11491] | 33678 | -0.133 | -0.5168 | Yes |
| 57 | PIK3CD | "phosphatidylinositol-4,5-bisphosphate 3-kinase catalytic subunit delta [Source:HGNC Symbol;Acc:HGNC:8977]" | 33794 | -0.137 | -0.5110 | Yes |
| 58 | PAK1 | p21 (RAC1) activated kinase 1 [Source:HGNC Symbol;Acc:HGNC:8590] | 33819 | -0.137 | -0.5028 | Yes |
| 59 | HCK | "HCK proto-oncogene, Src family tyrosine kinase [Source:HGNC Symbol;Acc:HGNC:4840]" | 33886 | -0.139 | -0.4956 | Yes |
| 60 | WASF2 | WASP family member 2 [Source:HGNC Symbol;Acc:HGNC:12733] | 34035 | -0.142 | -0.4903 | Yes |
| 61 | PRKCG | protein kinase C gamma [Source:HGNC Symbol;Acc:HGNC:9402] | 34056 | -0.143 | -0.4817 | Yes |
| 62 | VAV2 | vav guanine nucleotide exchange factor 2 [Source:HGNC Symbol;Acc:HGNC:12658] | 34251 | -0.148 | -0.4771 | Yes |
| 63 | PRKCE | protein kinase C epsilon [Source:HGNC Symbol;Acc:HGNC:9401] | 34307 | -0.150 | -0.4690 | Yes |
| 64 | CRKL | "CRK like proto-oncogene, adaptor protein [Source:HGNC Symbol;Acc:HGNC:2363]" | 34457 | -0.154 | -0.4629 | Yes |
| 65 | AKT3 | AKT serine/threonine kinase 3 [Source:HGNC Symbol;Acc:HGNC:393] | 34501 | -0.156 | -0.4540 | Yes |
| 66 | DNM2 | dynamin 2 [Source:HGNC Symbol;Acc:HGNC:2974] | 34825 | -0.165 | -0.4517 | Yes |
| 67 | PLCG1 | phospholipase C gamma 1 [Source:HGNC Symbol;Acc:HGNC:9065] | 34842 | -0.166 | -0.4415 | Yes |
| 68 | FCGR2A | Fc fragment of IgG receptor IIa [Source:HGNC Symbol;Acc:HGNC:3616] | 34953 | -0.169 | -0.4335 | Yes |
| 69 | PTPRC | protein tyrosine phosphatase receptor type C [Source:HGNC Symbol;Acc:HGNC:9666] | 35256 | -0.179 | -0.4297 | Yes |
| 70 | MAPK3 | mitogen-activated protein kinase 3 [Source:HGNC Symbol;Acc:HGNC:6877] | 35579 | -0.191 | -0.4257 | Yes |
| 71 | LIMK1 | LIM domain kinase 1 [Source:HGNC Symbol;Acc:HGNC:6613] | 35605 | -0.192 | -0.4140 | Yes |
| 72 | ARPC2 | actin related protein 2/3 complex subunit 2 [Source:HGNC Symbol;Acc:HGNC:705] | 35610 | -0.192 | -0.4018 | Yes |
| 73 | MAP2K1 | mitogen-activated protein kinase kinase 1 [Source:HGNC Symbol;Acc:HGNC:6840] | 36024 | -0.208 | -0.3990 | Yes |
| 74 | ARPC4 | actin related protein 2/3 complex subunit 4 [Source:HGNC Symbol;Acc:HGNC:707] | 36058 | -0.210 | -0.3864 | Yes |
| 75 | ARF6 | ADP ribosylation factor 6 [Source:HGNC Symbol;Acc:HGNC:659] | 36070 | -0.210 | -0.3733 | Yes |
| 76 | PIK3CG | "phosphatidylinositol-4,5-bisphosphate 3-kinase catalytic subunit gamma [Source:HGNC Symbol;Acc:HGNC:8978]" | 36243 | -0.217 | -0.3638 | Yes |
| 77 | NCF1 | neutrophil cytosolic factor 1 [Source:HGNC Symbol;Acc:HGNC:7660] | 36267 | -0.218 | -0.3504 | Yes |
| 78 | INPP5D | inositol polyphosphate-5-phosphatase D [Source:HGNC Symbol;Acc:HGNC:6079] | 36404 | -0.226 | -0.3394 | Yes |
| 79 | MARCKS | myristoylated alanine rich protein kinase C substrate [Source:HGNC Symbol;Acc:HGNC:6759] | 36589 | -0.235 | -0.3291 | Yes |
| 80 | PIK3R1 | phosphoinositide-3-kinase regulatory subunit 1 [Source:HGNC Symbol;Acc:HGNC:8979] | 37172 | -0.270 | -0.3266 | Yes |
| 81 | RAC1 | Rac family small GTPase 1 [Source:HGNC Symbol;Acc:HGNC:9801] | 37263 | -0.277 | -0.3112 | Yes |
| 82 | PIK3R5 | phosphoinositide-3-kinase regulatory subunit 5 [Source:HGNC Symbol;Acc:HGNC:30035] | 37628 | -0.307 | -0.3008 | Yes |
| 83 | CDC42 | cell division cycle 42 [Source:HGNC Symbol;Acc:HGNC:1736] | 37805 | -0.324 | -0.2845 | Yes |
| 84 | RAF1 | "Raf-1 proto-oncogene, serine/threonine kinase [Source:HGNC Symbol;Acc:HGNC:9829]" | 37807 | -0.325 | -0.2638 | Yes |
| 85 | GSN | gelsolin [Source:HGNC Symbol;Acc:HGNC:4620] | 37964 | -0.345 | -0.2457 | Yes |
| 86 | RPS6KB1 | ribosomal protein S6 kinase B1 [Source:HGNC Symbol;Acc:HGNC:10436] | 38015 | -0.352 | -0.2244 | Yes |
| 87 | VASP | vasodilator stimulated phosphoprotein [Source:HGNC Symbol;Acc:HGNC:12652] | 38042 | -0.355 | -0.2024 | Yes |
| 88 | CFL2 | cofilin 2 [Source:HGNC Symbol;Acc:HGNC:1875] | 38086 | -0.360 | -0.1804 | Yes |
| 89 | PLD2 | phospholipase D2 [Source:HGNC Symbol;Acc:HGNC:9068] | 38178 | -0.373 | -0.1589 | Yes |
| 90 | ASAP1 | "ArfGAP with SH3 domain, ankyrin repeat and PH domain 1 [Source:HGNC Symbol;Acc:HGNC:2720]" | 38238 | -0.386 | -0.1357 | Yes |
| 91 | LIMK2 | LIM domain kinase 2 [Source:HGNC Symbol;Acc:HGNC:6614] | 38261 | -0.390 | -0.1113 | Yes |
| 92 | PIK3CA | "phosphatidylinositol-4,5-bisphosphate 3-kinase catalytic subunit alpha [Source:HGNC Symbol;Acc:HGNC:8975]" | 38268 | -0.391 | -0.0865 | Yes |
| 93 | WASL | WASP like actin nucleation promoting factor [Source:HGNC Symbol;Acc:HGNC:12735] | 38341 | -0.407 | -0.0623 | Yes |
| 94 | LYN | "LYN proto-oncogene, Src family tyrosine kinase [Source:HGNC Symbol;Acc:HGNC:6735]" | 38449 | -0.435 | -0.0372 | Yes |
| 95 | SPHK1 | sphingosine kinase 1 [Source:HGNC Symbol;Acc:HGNC:11240] | 38515 | -0.463 | -0.0092 | Yes |
| 96 | GAB2 | GRB2 associated binding protein 2 [Source:HGNC Symbol;Acc:HGNC:14458] | 38520 | -0.465 | 0.0205 | Yes |
Table: GSEA details [plain text format]

  

Fig 2: KEGG\_FC\_GAMMA\_R\_MEDIATED\_PHAGOCYTOSIS      
 Blue-Pink O' Gram in the Space of the Analyzed GeneSet

  

Fig 3: KEGG\_FC\_GAMMA\_R\_MEDIATED\_PHAGOCYTOSIS: Random ES distribution      
 Gene set null distribution of ES for **KEGG\_FC\_GAMMA\_R\_MEDIATED\_PHAGOCYTOSIS**
