# Supplementary material for: Simultaneous Presentation of Multiple Myeloma and Lung Cancer: Case Report and Gene Bioinformatics Analysis
Source: Front Oncol. 2022 Jun 13;12:859735. doi: 10.3389/fonc.2022.859735 (PMC9235397; doi:10.3389/fonc.2022.859735)
Supplement: Supplementary file 1 [file DataSheet_1.zip › The bioinformatic analysis of MM and lung cancer supplementary materials/Enrichment analysis/MECR/GSEA_4.1.0/MM TCGA/KEGG.Gsea.1639040591792/KEGG_FOCAL_ADHESION.html]

Details for gene set KEGG\_FOCAL\_ADHESION[GSEA]

|  || Dataset | ExpData\_collapsed\_to\_symbols.ENSG00000116353\_profile\_in\_ExpData.cls #ENSG00000116353 |
| Phenotype | ENSG00000116353\_profile\_in\_ExpData.cls#ENSG00000116353 |
| Upregulated in class | ENSG00000116353\_neg |
| GeneSet | KEGG\_FOCAL\_ADHESION |
| Enrichment Score (ES) | -0.5684672 |
| Normalized Enrichment Score (NES) | -2.4779954 |
| Nominal p-value | 0.0 |
| FDR q-value | 0.0 |
| FWER p-Value | 0.0 |
Table: GSEA Results Summary

  

Fig 1: Enrichment plot: KEGG\_FOCAL\_ADHESION      
 Profile of the Running ES Score & Positions of GeneSet Members on the Rank Ordered List

  

| SYMBOL | TITLE | RANK IN GENE LIST | RANK METRIC SCORE | RUNNING ES | CORE ENRICHMENT || 1 | TLN2 | talin 2 [Source:HGNC Symbol;Acc:HGNC:15447] | 1239 | 0.321 | -0.0212 | No |
| 2 | PIK3R2 | phosphoinositide-3-kinase regulatory subunit 2 [Source:HGNC Symbol;Acc:HGNC:8980] | 1248 | 0.320 | -0.0110 | No |
| 3 | RELN | reelin [Source:HGNC Symbol;Acc:HGNC:9957] | 1967 | 0.266 | -0.0206 | No |
| 4 | SHC4 | SHC adaptor protein 4 [Source:HGNC Symbol;Acc:HGNC:16743] | 2178 | 0.254 | -0.0177 | No |
| 5 | CAV1 | caveolin 1 [Source:HGNC Symbol;Acc:HGNC:1527] | 2217 | 0.252 | -0.0105 | No |
| 6 | PAK5 | p21 (RAC1) activated kinase 5 [Source:HGNC Symbol;Acc:HGNC:15916] | 2247 | 0.250 | -0.0031 | No |
| 7 | PIK3R3 | phosphoinositide-3-kinase regulatory subunit 3 [Source:HGNC Symbol;Acc:HGNC:8981] | 2602 | 0.231 | -0.0046 | No |
| 8 | VAV1 | vav guanine nucleotide exchange factor 1 [Source:HGNC Symbol;Acc:HGNC:12657] | 3764 | 0.184 | -0.0283 | No |
| 9 | ITGA7 | integrin subunit alpha 7 [Source:HGNC Symbol;Acc:HGNC:6143] | 3910 | 0.179 | -0.0261 | No |
| 10 | TLN1 | talin 1 [Source:HGNC Symbol;Acc:HGNC:11845] | 3999 | 0.177 | -0.0226 | No |
| 11 | VEGFA | vascular endothelial growth factor A [Source:HGNC Symbol;Acc:HGNC:12680] | 4600 | 0.158 | -0.0328 | No |
| 12 | CCND2 | cyclin D2 [Source:HGNC Symbol;Acc:HGNC:1583] | 4849 | 0.152 | -0.0341 | No |
| 13 | TNN | tenascin N [Source:HGNC Symbol;Acc:HGNC:22942] | 5070 | 0.147 | -0.0350 | No |
| 14 | JUN | "Jun proto-oncogene, AP-1 transcription factor subunit [Source:HGNC Symbol;Acc:HGNC:6204]" | 5088 | 0.146 | -0.0306 | No |
| 15 | ERBB2 | erb-b2 receptor tyrosine kinase 2 [Source:HGNC Symbol;Acc:HGNC:3430] | 5193 | 0.144 | -0.0286 | No |
| 16 | ITGA10 | integrin subunit alpha 10 [Source:HGNC Symbol;Acc:HGNC:6135] | 5274 | 0.142 | -0.0260 | No |
| 17 | LAMC1 | laminin subunit gamma 1 [Source:HGNC Symbol;Acc:HGNC:6492] | 5393 | 0.140 | -0.0245 | No |
| 18 | SHC3 | SHC adaptor protein 3 [Source:HGNC Symbol;Acc:HGNC:18181] | 5437 | 0.138 | -0.0211 | No |
| 19 | THBS3 | thrombospondin 3 [Source:HGNC Symbol;Acc:HGNC:11787] | 5606 | 0.135 | -0.0210 | No |
| 20 | CAV3 | caveolin 3 [Source:HGNC Symbol;Acc:HGNC:1529] | 5717 | 0.133 | -0.0195 | No |
| 21 | ITGA3 | integrin subunit alpha 3 [Source:HGNC Symbol;Acc:HGNC:6139] | 5916 | 0.129 | -0.0203 | No |
| 22 | RAC2 | Rac family small GTPase 2 [Source:HGNC Symbol;Acc:HGNC:9802] | 6127 | 0.124 | -0.0216 | No |
| 23 | COL11A1 | collagen type XI alpha 1 chain [Source:HGNC Symbol;Acc:HGNC:2186] | 6403 | 0.120 | -0.0248 | No |
| 24 | SHC1 | SHC adaptor protein 1 [Source:HGNC Symbol;Acc:HGNC:10840] | 7103 | 0.108 | -0.0391 | No |
| 25 | THBS2 | thrombospondin 2 [Source:HGNC Symbol;Acc:HGNC:11786] | 7709 | 0.099 | -0.0513 | No |
| 26 | PIK3CB | "phosphatidylinositol-4,5-bisphosphate 3-kinase catalytic subunit beta [Source:HGNC Symbol;Acc:HGNC:8976]" | 7716 | 0.099 | -0.0483 | No |
| 27 | PAK2 | p21 (RAC1) activated kinase 2 [Source:HGNC Symbol;Acc:HGNC:8591] | 7725 | 0.099 | -0.0453 | No |
| 28 | MYLK2 | myosin light chain kinase 2 [Source:HGNC Symbol;Acc:HGNC:16243] | 7785 | 0.098 | -0.0436 | No |
| 29 | VEGFD | vascular endothelial growth factor D [Source:HGNC Symbol;Acc:HGNC:3708] | 8223 | 0.092 | -0.0518 | No |
| 30 | COL4A6 | collagen type IV alpha 6 chain [Source:HGNC Symbol;Acc:HGNC:2208] | 8336 | 0.090 | -0.0517 | No |
| 31 | ITGB6 | integrin subunit beta 6 [Source:HGNC Symbol;Acc:HGNC:6161] | 8339 | 0.090 | -0.0488 | No |
| 32 | MYLK | myosin light chain kinase [Source:HGNC Symbol;Acc:HGNC:7590] | 8358 | 0.090 | -0.0463 | No |
| 33 | TNR | tenascin R [Source:HGNC Symbol;Acc:HGNC:11953] | 8456 | 0.089 | -0.0459 | No |
| 34 | LAMC3 | laminin subunit gamma 3 [Source:HGNC Symbol;Acc:HGNC:6494] | 8849 | 0.084 | -0.0531 | No |
| 35 | ITGA6 | integrin subunit alpha 6 [Source:HGNC Symbol;Acc:HGNC:6142] | 8958 | 0.083 | -0.0532 | No |
| 36 | PIP5K1C | phosphatidylinositol-4-phosphate 5-kinase type 1 gamma [Source:HGNC Symbol;Acc:HGNC:8996] | 9093 | 0.081 | -0.0540 | No |
| 37 | IBSP | integrin binding sialoprotein [Source:HGNC Symbol;Acc:HGNC:5341] | 10223 | 0.068 | -0.0806 | No |
| 38 | ITGA4 | integrin subunit alpha 4 [Source:HGNC Symbol;Acc:HGNC:6140] | 10741 | 0.063 | -0.0917 | No |
| 39 | COL6A6 | collagen type VI alpha 6 chain [Source:HGNC Symbol;Acc:HGNC:27023] | 10845 | 0.062 | -0.0923 | No |
| 40 | PAK4 | p21 (RAC1) activated kinase 4 [Source:HGNC Symbol;Acc:HGNC:16059] | 11565 | 0.056 | -0.1089 | No |
| 41 | PRKCB | protein kinase C beta [Source:HGNC Symbol;Acc:HGNC:9395] | 11760 | 0.054 | -0.1121 | No |
| 42 | GSK3B | glycogen synthase kinase 3 beta [Source:HGNC Symbol;Acc:HGNC:4617] | 12189 | 0.051 | -0.1214 | No |
| 43 | XIAP | X-linked inhibitor of apoptosis [Source:HGNC Symbol;Acc:HGNC:592] | 13774 | 0.039 | -0.1606 | No |
| 44 | LAMA3 | laminin subunit alpha 3 [Source:HGNC Symbol;Acc:HGNC:6483] | 14726 | 0.034 | -0.1838 | No |
| 45 | CRK | "CRK proto-oncogene, adaptor protein [Source:HGNC Symbol;Acc:HGNC:2362]" | 14915 | 0.032 | -0.1875 | No |
| 46 | MAPK10 | mitogen-activated protein kinase 10 [Source:HGNC Symbol;Acc:HGNC:6872] | 14943 | 0.032 | -0.1872 | No |
| 47 | COL11A2 | collagen type XI alpha 2 chain [Source:HGNC Symbol;Acc:HGNC:2187] | 15922 | 0.026 | -0.2113 | No |
| 48 | FLT1 | fms related receptor tyrosine kinase 1 [Source:HGNC Symbol;Acc:HGNC:3763] | 16066 | 0.025 | -0.2141 | No |
| 49 | ITGA2 | integrin subunit alpha 2 [Source:HGNC Symbol;Acc:HGNC:6137] | 17136 | 0.019 | -0.2408 | No |
| 50 | IGF1R | insulin like growth factor 1 receptor [Source:HGNC Symbol;Acc:HGNC:5465] | 17189 | 0.019 | -0.2415 | No |
| 51 | PDGFD | platelet derived growth factor D [Source:HGNC Symbol;Acc:HGNC:30620] | 18049 | 0.014 | -0.2630 | No |
| 52 | SOS1 | SOS Ras/Rac guanine nucleotide exchange factor 1 [Source:HGNC Symbol;Acc:HGNC:11187] | 18532 | 0.012 | -0.2750 | No |
| 53 | COL5A2 | collagen type V alpha 2 chain [Source:HGNC Symbol;Acc:HGNC:2210] | 18605 | 0.011 | -0.2764 | No |
| 54 | MYL10 | myosin light chain 10 [Source:HGNC Symbol;Acc:HGNC:29825] | 18653 | 0.011 | -0.2773 | No |
| 55 | MYL7 | myosin light chain 7 [Source:HGNC Symbol;Acc:HGNC:21719] | 18720 | 0.011 | -0.2786 | No |
| 56 | PTEN | phosphatase and tensin homolog [Source:HGNC Symbol;Acc:HGNC:9588] | 19053 | 0.009 | -0.2868 | No |
| 57 | ARHGAP5 | Rho GTPase activating protein 5 [Source:HGNC Symbol;Acc:HGNC:675] | 19173 | 0.008 | -0.2896 | No |
| 58 | CAV2 | caveolin 2 [Source:HGNC Symbol;Acc:HGNC:1528] | 19515 | 0.006 | -0.2981 | No |
| 59 | ITGA8 | integrin subunit alpha 8 [Source:HGNC Symbol;Acc:HGNC:6144] | 20064 | 0.003 | -0.3120 | No |
| 60 | ITGA1 | integrin subunit alpha 1 [Source:HGNC Symbol;Acc:HGNC:6134] | 20236 | 0.002 | -0.3163 | No |
| 61 | COL4A4 | collagen type IV alpha 4 chain [Source:HGNC Symbol;Acc:HGNC:2206] | 20276 | 0.002 | -0.3172 | No |
| 62 | PTK2 | protein tyrosine kinase 2 [Source:HGNC Symbol;Acc:HGNC:9611] | 20287 | 0.002 | -0.3174 | No |
| 63 | MAPK9 | mitogen-activated protein kinase 9 [Source:HGNC Symbol;Acc:HGNC:6886] | 20291 | 0.002 | -0.3175 | No |
| 64 | LAMA1 | laminin subunit alpha 1 [Source:HGNC Symbol;Acc:HGNC:6481] | 20448 | 0.001 | -0.3214 | No |
| 65 | VEGFB | vascular endothelial growth factor B [Source:HGNC Symbol;Acc:HGNC:12681] | 20858 | -0.001 | -0.3318 | No |
| 66 | CAPN2 | calpain 2 [Source:HGNC Symbol;Acc:HGNC:1479] | 21039 | -0.002 | -0.3364 | No |
| 67 | RASGRF1 | Ras protein specific guanine nucleotide releasing factor 1 [Source:HGNC Symbol;Acc:HGNC:9875] | 22030 | -0.008 | -0.3614 | No |
| 68 | ITGB1 | integrin subunit beta 1 [Source:HGNC Symbol;Acc:HGNC:6153] | 22081 | -0.008 | -0.3624 | No |
| 69 | ITGA11 | integrin subunit alpha 11 [Source:HGNC Symbol;Acc:HGNC:6136] | 22218 | -0.009 | -0.3656 | No |
| 70 | HGF | hepatocyte growth factor [Source:HGNC Symbol;Acc:HGNC:4893] | 22763 | -0.012 | -0.3791 | No |
| 71 | MAPK1 | mitogen-activated protein kinase 1 [Source:HGNC Symbol;Acc:HGNC:6871] | 23167 | -0.015 | -0.3889 | No |
| 72 | ROCK2 | Rho associated coiled-coil containing protein kinase 2 [Source:HGNC Symbol;Acc:HGNC:10252] | 24395 | -0.022 | -0.4195 | No |
| 73 | MYLK3 | myosin light chain kinase 3 [Source:HGNC Symbol;Acc:HGNC:29826] | 25385 | -0.029 | -0.4439 | No |
| 74 | MET | "MET proto-oncogene, receptor tyrosine kinase [Source:HGNC Symbol;Acc:HGNC:7029]" | 25533 | -0.030 | -0.4466 | No |
| 75 | VEGFC | vascular endothelial growth factor C [Source:HGNC Symbol;Acc:HGNC:12682] | 25847 | -0.033 | -0.4536 | No |
| 76 | HRAS | "HRas proto-oncogene, GTPase [Source:HGNC Symbol;Acc:HGNC:5173]" | 26672 | -0.039 | -0.4734 | No |
| 77 | ACTN2 | actinin alpha 2 [Source:HGNC Symbol;Acc:HGNC:164] | 27204 | -0.043 | -0.4856 | No |
| 78 | ACTN4 | actinin alpha 4 [Source:HGNC Symbol;Acc:HGNC:166] | 28351 | -0.054 | -0.5131 | No |
| 79 | TNXB | tenascin XB [Source:HGNC Symbol;Acc:HGNC:11976] | 28667 | -0.057 | -0.5193 | No |
| 80 | THBS4 | thrombospondin 4 [Source:HGNC Symbol;Acc:HGNC:11788] | 28835 | -0.058 | -0.5217 | No |
| 81 | FN1 | fibronectin 1 [Source:HGNC Symbol;Acc:HGNC:3778] | 29282 | -0.064 | -0.5310 | No |
| 82 | VAV3 | vav guanine nucleotide exchange factor 3 [Source:HGNC Symbol;Acc:HGNC:12659] | 29349 | -0.065 | -0.5306 | No |
| 83 | LAMA5 | laminin subunit alpha 5 [Source:HGNC Symbol;Acc:HGNC:6485] | 29473 | -0.066 | -0.5316 | No |
| 84 | SPP1 | secreted phosphoprotein 1 [Source:HGNC Symbol;Acc:HGNC:11255] | 29539 | -0.067 | -0.5311 | No |
| 85 | LAMC2 | laminin subunit gamma 2 [Source:HGNC Symbol;Acc:HGNC:6493] | 29553 | -0.067 | -0.5292 | No |
| 86 | VTN | vitronectin [Source:HGNC Symbol;Acc:HGNC:12724] | 29988 | -0.072 | -0.5380 | No |
| 87 | COL6A3 | collagen type VI alpha 3 chain [Source:HGNC Symbol;Acc:HGNC:2213] | 30011 | -0.072 | -0.5362 | No |
| 88 | PDGFA | platelet derived growth factor subunit A [Source:HGNC Symbol;Acc:HGNC:8799] | 30414 | -0.077 | -0.5439 | No |
| 89 | DOCK1 | dedicator of cytokinesis 1 [Source:HGNC Symbol;Acc:HGNC:2987] | 30454 | -0.078 | -0.5424 | No |
| 90 | RAC3 | Rac family small GTPase 3 [Source:HGNC Symbol;Acc:HGNC:9803] | 30704 | -0.081 | -0.5461 | No |
| 91 | ACTB | actin beta [Source:HGNC Symbol;Acc:HGNC:132] | 30724 | -0.081 | -0.5440 | No |
| 92 | FLNB | filamin B [Source:HGNC Symbol;Acc:HGNC:3755] | 31302 | -0.089 | -0.5558 | No |
| 93 | ITGB3 | integrin subunit beta 3 [Source:HGNC Symbol;Acc:HGNC:6156] | 31798 | -0.096 | -0.5653 | Yes |
| 94 | AKT1 | AKT serine/threonine kinase 1 [Source:HGNC Symbol;Acc:HGNC:391] | 31906 | -0.098 | -0.5649 | Yes |
| 95 | ARHGAP35 | Rho GTPase activating protein 35 [Source:HGNC Symbol;Acc:HGNC:4591] | 31911 | -0.098 | -0.5618 | Yes |
| 96 | PARVB | parvin beta [Source:HGNC Symbol;Acc:HGNC:14653] | 31973 | -0.099 | -0.5601 | Yes |
| 97 | ITGA9 | integrin subunit alpha 9 [Source:HGNC Symbol;Acc:HGNC:6145] | 32051 | -0.101 | -0.5588 | Yes |
| 98 | CHAD | chondroadherin [Source:HGNC Symbol;Acc:HGNC:1909] | 32057 | -0.101 | -0.5556 | Yes |
| 99 | ITGB5 | integrin subunit beta 5 [Source:HGNC Symbol;Acc:HGNC:6160] | 32063 | -0.101 | -0.5524 | Yes |
| 100 | ITGB7 | integrin subunit beta 7 [Source:HGNC Symbol;Acc:HGNC:6162] | 32073 | -0.101 | -0.5494 | Yes |
| 101 | COL1A2 | collagen type I alpha 2 chain [Source:HGNC Symbol;Acc:HGNC:2198] | 32118 | -0.102 | -0.5472 | Yes |
| 102 | LAMA2 | laminin subunit alpha 2 [Source:HGNC Symbol;Acc:HGNC:6482] | 32367 | -0.106 | -0.5501 | Yes |
| 103 | ACTN3 | actinin alpha 3 [Source:HGNC Symbol;Acc:HGNC:165] | 32823 | -0.115 | -0.5579 | Yes |
| 104 | PRKCA | protein kinase C alpha [Source:HGNC Symbol;Acc:HGNC:9393] | 33017 | -0.119 | -0.5590 | Yes |
| 105 | ELK1 | ETS transcription factor ELK1 [Source:HGNC Symbol;Acc:HGNC:3321] | 33028 | -0.120 | -0.5553 | Yes |
| 106 | FLNC | filamin C [Source:HGNC Symbol;Acc:HGNC:3756] | 33038 | -0.120 | -0.5516 | Yes |
| 107 | MYL2 | myosin light chain 2 [Source:HGNC Symbol;Acc:HGNC:7583] | 33086 | -0.121 | -0.5489 | Yes |
| 108 | EGF | epidermal growth factor [Source:HGNC Symbol;Acc:HGNC:3229] | 33135 | -0.121 | -0.5462 | Yes |
| 109 | PPP1CA | protein phosphatase 1 catalytic subunit alpha [Source:HGNC Symbol;Acc:HGNC:9281] | 33209 | -0.123 | -0.5440 | Yes |
| 110 | AKT2 | AKT serine/threonine kinase 2 [Source:HGNC Symbol;Acc:HGNC:392] | 33213 | -0.123 | -0.5401 | Yes |
| 111 | COMP | cartilage oligomeric matrix protein [Source:HGNC Symbol;Acc:HGNC:2227] | 33307 | -0.125 | -0.5384 | Yes |
| 112 | PDGFC | platelet derived growth factor C [Source:HGNC Symbol;Acc:HGNC:8801] | 33384 | -0.127 | -0.5362 | Yes |
| 113 | PDGFB | platelet derived growth factor subunit B [Source:HGNC Symbol;Acc:HGNC:8800] | 33560 | -0.130 | -0.5364 | Yes |
| 114 | ITGB4 | integrin subunit beta 4 [Source:HGNC Symbol;Acc:HGNC:6158] | 33620 | -0.132 | -0.5336 | Yes |
| 115 | COL5A1 | collagen type V alpha 1 chain [Source:HGNC Symbol;Acc:HGNC:2209] | 33702 | -0.134 | -0.5314 | Yes |
| 116 | PIK3CD | "phosphatidylinositol-4,5-bisphosphate 3-kinase catalytic subunit delta [Source:HGNC Symbol;Acc:HGNC:8977]" | 33794 | -0.137 | -0.5292 | Yes |
| 117 | FYN | "FYN proto-oncogene, Src family tyrosine kinase [Source:HGNC Symbol;Acc:HGNC:4037]" | 33801 | -0.137 | -0.5249 | Yes |
| 118 | PAK1 | p21 (RAC1) activated kinase 1 [Source:HGNC Symbol;Acc:HGNC:8590] | 33819 | -0.137 | -0.5209 | Yes |
| 119 | MYLPF | "myosin light chain, phosphorylatable, fast skeletal muscle [Source:HGNC Symbol;Acc:HGNC:29824]" | 33874 | -0.139 | -0.5177 | Yes |
| 120 | PGF | placental growth factor [Source:HGNC Symbol;Acc:HGNC:8893] | 34015 | -0.142 | -0.5167 | Yes |
| 121 | ACTG1 | actin gamma 1 [Source:HGNC Symbol;Acc:HGNC:144] | 34048 | -0.142 | -0.5129 | Yes |
| 122 | MYL5 | myosin light chain 5 [Source:HGNC Symbol;Acc:HGNC:7586] | 34055 | -0.143 | -0.5084 | Yes |
| 123 | PRKCG | protein kinase C gamma [Source:HGNC Symbol;Acc:HGNC:9402] | 34056 | -0.143 | -0.5037 | Yes |
| 124 | FLT4 | fms related receptor tyrosine kinase 4 [Source:HGNC Symbol;Acc:HGNC:3767] | 34111 | -0.144 | -0.5004 | Yes |
| 125 | PAK6 | p21 (RAC1) activated kinase 6 [Source:HGNC Symbol;Acc:HGNC:16061] | 34232 | -0.148 | -0.4986 | Yes |
| 126 | VAV2 | vav guanine nucleotide exchange factor 2 [Source:HGNC Symbol;Acc:HGNC:12658] | 34251 | -0.148 | -0.4943 | Yes |
| 127 | PAK3 | p21 (RAC1) activated kinase 3 [Source:HGNC Symbol;Acc:HGNC:8592] | 34288 | -0.149 | -0.4903 | Yes |
| 128 | MYL12A | myosin light chain 12A [Source:HGNC Symbol;Acc:HGNC:16701] | 34438 | -0.154 | -0.4891 | Yes |
| 129 | CRKL | "CRK like proto-oncogene, adaptor protein [Source:HGNC Symbol;Acc:HGNC:2363]" | 34457 | -0.154 | -0.4845 | Yes |
| 130 | SRC | "SRC proto-oncogene, non-receptor tyrosine kinase [Source:HGNC Symbol;Acc:HGNC:11283]" | 34463 | -0.155 | -0.4796 | Yes |
| 131 | LAMB4 | laminin subunit beta 4 [Source:HGNC Symbol;Acc:HGNC:6491] | 34499 | -0.156 | -0.4755 | Yes |
| 132 | AKT3 | AKT serine/threonine kinase 3 [Source:HGNC Symbol;Acc:HGNC:393] | 34501 | -0.156 | -0.4704 | Yes |
| 133 | LAMB3 | laminin subunit beta 3 [Source:HGNC Symbol;Acc:HGNC:6490] | 34594 | -0.158 | -0.4676 | Yes |
| 134 | SHC2 | SHC adaptor protein 2 [Source:HGNC Symbol;Acc:HGNC:29869] | 34623 | -0.160 | -0.4631 | Yes |
| 135 | KDR | kinase insert domain receptor [Source:HGNC Symbol;Acc:HGNC:6307] | 34689 | -0.161 | -0.4595 | Yes |
| 136 | PPP1CC | protein phosphatase 1 catalytic subunit gamma [Source:HGNC Symbol;Acc:HGNC:9283] | 34862 | -0.166 | -0.4585 | Yes |
| 137 | PDGFRB | platelet derived growth factor receptor beta [Source:HGNC Symbol;Acc:HGNC:8804] | 34887 | -0.167 | -0.4536 | Yes |
| 138 | COL3A1 | collagen type III alpha 1 chain [Source:HGNC Symbol;Acc:HGNC:2201] | 34939 | -0.169 | -0.4494 | Yes |
| 139 | ITGB8 | integrin subunit beta 8 [Source:HGNC Symbol;Acc:HGNC:6163] | 35094 | -0.174 | -0.4477 | Yes |
| 140 | COL2A1 | collagen type II alpha 1 chain [Source:HGNC Symbol;Acc:HGNC:2200] | 35167 | -0.176 | -0.4438 | Yes |
| 141 | LAMB2 | laminin subunit beta 2 [Source:HGNC Symbol;Acc:HGNC:6487] | 35187 | -0.177 | -0.4385 | Yes |
| 142 | FLNA | filamin A [Source:HGNC Symbol;Acc:HGNC:3754] | 35348 | -0.183 | -0.4366 | Yes |
| 143 | EGFR | epidermal growth factor receptor [Source:HGNC Symbol;Acc:HGNC:3236] | 35417 | -0.186 | -0.4323 | Yes |
| 144 | PXN | paxillin [Source:HGNC Symbol;Acc:HGNC:9718] | 35502 | -0.189 | -0.4283 | Yes |
| 145 | COL1A1 | collagen type I alpha 1 chain [Source:HGNC Symbol;Acc:HGNC:2197] | 35534 | -0.189 | -0.4229 | Yes |
| 146 | MAPK3 | mitogen-activated protein kinase 3 [Source:HGNC Symbol;Acc:HGNC:6877] | 35579 | -0.191 | -0.4178 | Yes |
| 147 | BCAR1 | "BCAR1 scaffold protein, Cas family member [Source:HGNC Symbol;Acc:HGNC:971]" | 35834 | -0.201 | -0.4178 | Yes |
| 148 | CCND1 | cyclin D1 [Source:HGNC Symbol;Acc:HGNC:1582] | 35880 | -0.203 | -0.4123 | Yes |
| 149 | COL6A2 | collagen type VI alpha 2 chain [Source:HGNC Symbol;Acc:HGNC:2212] | 35895 | -0.203 | -0.4060 | Yes |
| 150 | IGF1 | insulin like growth factor 1 [Source:HGNC Symbol;Acc:HGNC:5464] | 35903 | -0.203 | -0.3996 | Yes |
| 151 | MAP2K1 | mitogen-activated protein kinase kinase 1 [Source:HGNC Symbol;Acc:HGNC:6840] | 36024 | -0.208 | -0.3959 | Yes |
| 152 | VWF | von Willebrand factor [Source:HGNC Symbol;Acc:HGNC:12726] | 36101 | -0.212 | -0.3909 | Yes |
| 153 | VCL | vinculin [Source:HGNC Symbol;Acc:HGNC:12665] | 36176 | -0.215 | -0.3858 | Yes |
| 154 | PIK3CG | "phosphatidylinositol-4,5-bisphosphate 3-kinase catalytic subunit gamma [Source:HGNC Symbol;Acc:HGNC:8978]" | 36243 | -0.217 | -0.3804 | Yes |
| 155 | PPP1CB | protein phosphatase 1 catalytic subunit beta [Source:HGNC Symbol;Acc:HGNC:9282] | 36276 | -0.219 | -0.3741 | Yes |
| 156 | ITGA5 | integrin subunit alpha 5 [Source:HGNC Symbol;Acc:HGNC:6141] | 36513 | -0.231 | -0.3726 | Yes |
| 157 | PDGFRA | platelet derived growth factor receptor alpha [Source:HGNC Symbol;Acc:HGNC:8803] | 36521 | -0.231 | -0.3652 | Yes |
| 158 | GRB2 | growth factor receptor bound protein 2 [Source:HGNC Symbol;Acc:HGNC:4566] | 36523 | -0.231 | -0.3577 | Yes |
| 159 | PARVA | parvin alpha [Source:HGNC Symbol;Acc:HGNC:14652] | 36550 | -0.233 | -0.3507 | Yes |
| 160 | ACTN1 | actinin alpha 1 [Source:HGNC Symbol;Acc:HGNC:163] | 36614 | -0.236 | -0.3446 | Yes |
| 161 | MYL9 | myosin light chain 9 [Source:HGNC Symbol;Acc:HGNC:15754] | 36627 | -0.237 | -0.3372 | Yes |
| 162 | COL4A1 | collagen type IV alpha 1 chain [Source:HGNC Symbol;Acc:HGNC:2202] | 36657 | -0.239 | -0.3302 | Yes |
| 163 | COL4A2 | collagen type IV alpha 2 chain [Source:HGNC Symbol;Acc:HGNC:2203] | 36693 | -0.241 | -0.3232 | Yes |
| 164 | LAMA4 | laminin subunit alpha 4 [Source:HGNC Symbol;Acc:HGNC:6484] | 36765 | -0.245 | -0.3170 | Yes |
| 165 | DIAPH1 | diaphanous related formin 1 [Source:HGNC Symbol;Acc:HGNC:2876] | 36840 | -0.249 | -0.3108 | Yes |
| 166 | PDPK1 | 3-phosphoinositide dependent protein kinase 1 [Source:HGNC Symbol;Acc:HGNC:8816] | 36889 | -0.253 | -0.3038 | Yes |
| 167 | COL6A1 | collagen type VI alpha 1 chain [Source:HGNC Symbol;Acc:HGNC:2211] | 37144 | -0.269 | -0.3015 | Yes |
| 168 | COL5A3 | collagen type V alpha 3 chain [Source:HGNC Symbol;Acc:HGNC:14864] | 37155 | -0.269 | -0.2930 | Yes |
| 169 | PIK3R1 | phosphoinositide-3-kinase regulatory subunit 1 [Source:HGNC Symbol;Acc:HGNC:8979] | 37172 | -0.270 | -0.2845 | Yes |
| 170 | RAC1 | Rac family small GTPase 1 [Source:HGNC Symbol;Acc:HGNC:9801] | 37263 | -0.277 | -0.2778 | Yes |
| 171 | ITGA2B | integrin subunit alpha 2b [Source:HGNC Symbol;Acc:HGNC:6138] | 37349 | -0.284 | -0.2707 | Yes |
| 172 | RAP1A | "RAP1A, member of RAS oncogene family [Source:HGNC Symbol;Acc:HGNC:9855]" | 37388 | -0.287 | -0.2624 | Yes |
| 173 | CCND3 | cyclin D3 [Source:HGNC Symbol;Acc:HGNC:1585] | 37400 | -0.288 | -0.2533 | Yes |
| 174 | RHOA | ras homolog family member A [Source:HGNC Symbol;Acc:HGNC:667] | 37417 | -0.290 | -0.2442 | Yes |
| 175 | BIRC2 | baculoviral IAP repeat containing 2 [Source:HGNC Symbol;Acc:HGNC:590] | 37460 | -0.293 | -0.2357 | Yes |
| 176 | BIRC3 | baculoviral IAP repeat containing 3 [Source:HGNC Symbol;Acc:HGNC:591] | 37532 | -0.299 | -0.2278 | Yes |
| 177 | TNC | tenascin C [Source:HGNC Symbol;Acc:HGNC:5318] | 37553 | -0.301 | -0.2185 | Yes |
| 178 | PIK3R5 | phosphoinositide-3-kinase regulatory subunit 5 [Source:HGNC Symbol;Acc:HGNC:30035] | 37628 | -0.307 | -0.2104 | Yes |
| 179 | PARVG | parvin gamma [Source:HGNC Symbol;Acc:HGNC:14654] | 37665 | -0.310 | -0.2012 | Yes |
| 180 | MYL12B | myosin light chain 12B [Source:HGNC Symbol;Acc:HGNC:29827] | 37667 | -0.310 | -0.1911 | Yes |
| 181 | BCL2 | BCL2 apoptosis regulator [Source:HGNC Symbol;Acc:HGNC:990] | 37727 | -0.316 | -0.1823 | Yes |
| 182 | BAD | BCL2 associated agonist of cell death [Source:HGNC Symbol;Acc:HGNC:936] | 37742 | -0.318 | -0.1723 | Yes |
| 183 | PPP1R12A | protein phosphatase 1 regulatory subunit 12A [Source:HGNC Symbol;Acc:HGNC:7618] | 37785 | -0.322 | -0.1628 | Yes |
| 184 | CDC42 | cell division cycle 42 [Source:HGNC Symbol;Acc:HGNC:1736] | 37805 | -0.324 | -0.1527 | Yes |
| 185 | RAF1 | "Raf-1 proto-oncogene, serine/threonine kinase [Source:HGNC Symbol;Acc:HGNC:9829]" | 37807 | -0.325 | -0.1422 | Yes |
| 186 | LAMB1 | laminin subunit beta 1 [Source:HGNC Symbol;Acc:HGNC:6486] | 37810 | -0.326 | -0.1316 | Yes |
| 187 | ITGAV | integrin subunit alpha V [Source:HGNC Symbol;Acc:HGNC:6150] | 37826 | -0.328 | -0.1213 | Yes |
| 188 | BRAF | "B-Raf proto-oncogene, serine/threonine kinase [Source:HGNC Symbol;Acc:HGNC:1097]" | 37844 | -0.330 | -0.1109 | Yes |
| 189 | ZYX | zyxin [Source:HGNC Symbol;Acc:HGNC:13200] | 37931 | -0.341 | -0.1020 | Yes |
| 190 | VASP | vasodilator stimulated phosphoprotein [Source:HGNC Symbol;Acc:HGNC:12652] | 38042 | -0.355 | -0.0933 | Yes |
| 191 | ILK | integrin linked kinase [Source:HGNC Symbol;Acc:HGNC:6040] | 38122 | -0.365 | -0.0834 | Yes |
| 192 | THBS1 | thrombospondin 1 [Source:HGNC Symbol;Acc:HGNC:11785] | 38179 | -0.373 | -0.0726 | Yes |
| 193 | PIK3CA | "phosphatidylinositol-4,5-bisphosphate 3-kinase catalytic subunit alpha [Source:HGNC Symbol;Acc:HGNC:8975]" | 38268 | -0.391 | -0.0621 | Yes |
| 194 | MAPK8 | mitogen-activated protein kinase 8 [Source:HGNC Symbol;Acc:HGNC:6881] | 38339 | -0.406 | -0.0507 | Yes |
| 195 | RAPGEF1 | Rap guanine nucleotide exchange factor 1 [Source:HGNC Symbol;Acc:HGNC:4568] | 38386 | -0.414 | -0.0383 | Yes |
| 196 | RAP1B | "RAP1B, member of RAS oncogene family [Source:HGNC Symbol;Acc:HGNC:9857]" | 38403 | -0.421 | -0.0250 | Yes |
| 197 | CTNNB1 | catenin beta 1 [Source:HGNC Symbol;Acc:HGNC:2514] | 38456 | -0.438 | -0.0120 | Yes |
| 198 | ROCK1 | Rho associated coiled-coil containing protein kinase 1 [Source:HGNC Symbol;Acc:HGNC:10251] | 38575 | -0.519 | 0.0019 | Yes |
| 199 | SOS2 | SOS Ras/Rho guanine nucleotide exchange factor 2 [Source:HGNC Symbol;Acc:HGNC:11188] | 38581 | -0.528 | 0.0190 | Yes |
Table: GSEA details [plain text format]

  

Fig 2: KEGG\_FOCAL\_ADHESION      
 Blue-Pink O' Gram in the Space of the Analyzed GeneSet

  

Fig 3: KEGG\_FOCAL\_ADHESION: Random ES distribution      
 Gene set null distribution of ES for **KEGG\_FOCAL\_ADHESION**
