# Supplementary material for: Simultaneous Presentation of Multiple Myeloma and Lung Cancer: Case Report and Gene Bioinformatics Analysis
Source: Front Oncol. 2022 Jun 13;12:859735. doi: 10.3389/fonc.2022.859735 (PMC9235397; doi:10.3389/fonc.2022.859735)
Supplement: Supplementary file 1 [file DataSheet_1.zip › The bioinformatic analysis of MM and lung cancer supplementary materials/Enrichment analysis/MECR/GSEA_4.1.0/MM TCGA/KEGG.Gsea.1639040591792/KEGG_GLYCOLYSIS_GLUCONEOGENESIS.html]

Details for gene set KEGG\_GLYCOLYSIS\_GLUCONEOGENESIS[GSEA]

|  || Dataset | ExpData\_collapsed\_to\_symbols.ENSG00000116353\_profile\_in\_ExpData.cls #ENSG00000116353 |
| Phenotype | ENSG00000116353\_profile\_in\_ExpData.cls#ENSG00000116353 |
| Upregulated in class | ENSG00000116353\_pos |
| GeneSet | KEGG\_GLYCOLYSIS\_GLUCONEOGENESIS |
| Enrichment Score (ES) | 0.57897 |
| Normalized Enrichment Score (NES) | 1.9546596 |
| Nominal p-value | 0.0 |
| FDR q-value | 4.623265E-4 |
| FWER p-Value | 0.011 |
Table: GSEA Results Summary

  

Fig 1: Enrichment plot: KEGG\_GLYCOLYSIS\_GLUCONEOGENESIS      
 Profile of the Running ES Score & Positions of GeneSet Members on the Rank Ordered List

  

| SYMBOL | TITLE | RANK IN GENE LIST | RANK METRIC SCORE | RUNNING ES | CORE ENRICHMENT || 1 | PDHA1 | pyruvate dehydrogenase E1 subunit alpha 1 [Source:HGNC Symbol;Acc:HGNC:8806] | 223 | 0.477 | 0.0425 | Yes |
| 2 | ALDH1B1 | aldehyde dehydrogenase 1 family member B1 [Source:HGNC Symbol;Acc:HGNC:407] | 372 | 0.439 | 0.0831 | Yes |
| 3 | PFKM | "phosphofructokinase, muscle [Source:HGNC Symbol;Acc:HGNC:8877]" | 381 | 0.438 | 0.1271 | Yes |
| 4 | ENO1 | enolase 1 [Source:HGNC Symbol;Acc:HGNC:3350] | 470 | 0.418 | 0.1671 | Yes |
| 5 | GPI | glucose-6-phosphate isomerase [Source:HGNC Symbol;Acc:HGNC:4458] | 642 | 0.392 | 0.2023 | Yes |
| 6 | ALDH3A2 | aldehyde dehydrogenase 3 family member A2 [Source:HGNC Symbol;Acc:HGNC:403] | 902 | 0.354 | 0.2315 | Yes |
| 7 | DLAT | dihydrolipoamide S-acetyltransferase [Source:HGNC Symbol;Acc:HGNC:2896] | 998 | 0.345 | 0.2639 | Yes |
| 8 | PGM2 | phosphoglucomutase 2 [Source:HGNC Symbol;Acc:HGNC:8906] | 1043 | 0.340 | 0.2971 | Yes |
| 9 | TPI1 | triosephosphate isomerase 1 [Source:HGNC Symbol;Acc:HGNC:12009] | 1094 | 0.335 | 0.3297 | Yes |
| 10 | LDHB | lactate dehydrogenase B [Source:HGNC Symbol;Acc:HGNC:6541] | 1375 | 0.309 | 0.3537 | Yes |
| 11 | ALDH9A1 | aldehyde dehydrogenase 9 family member A1 [Source:HGNC Symbol;Acc:HGNC:412] | 1406 | 0.306 | 0.3839 | Yes |
| 12 | AKR1A1 | aldo-keto reductase family 1 member A1 [Source:HGNC Symbol;Acc:HGNC:380] | 2008 | 0.263 | 0.3951 | Yes |
| 13 | PDHB | pyruvate dehydrogenase E1 subunit beta [Source:HGNC Symbol;Acc:HGNC:8808] | 2053 | 0.261 | 0.4204 | Yes |
| 14 | ADH6 | alcohol dehydrogenase 6 (class V) [Source:HGNC Symbol;Acc:HGNC:255] | 2701 | 0.227 | 0.4268 | Yes |
| 15 | ACSS1 | acyl-CoA synthetase short chain family member 1 [Source:HGNC Symbol;Acc:HGNC:16091] | 2776 | 0.223 | 0.4475 | Yes |
| 16 | ADH5 | "alcohol dehydrogenase 5 (class III), chi polypeptide [Source:HGNC Symbol;Acc:HGNC:253]" | 3136 | 0.207 | 0.4593 | Yes |
| 17 | LDHAL6A | lactate dehydrogenase A like 6A [Source:HGNC Symbol;Acc:HGNC:28335] | 3170 | 0.206 | 0.4793 | Yes |
| 18 | DLD | dihydrolipoamide dehydrogenase [Source:HGNC Symbol;Acc:HGNC:2898] | 3616 | 0.189 | 0.4871 | Yes |
| 19 | LDHA | lactate dehydrogenase A [Source:HGNC Symbol;Acc:HGNC:6535] | 4425 | 0.164 | 0.4830 | Yes |
| 20 | PCK2 | "phosphoenolpyruvate carboxykinase 2, mitochondrial [Source:HGNC Symbol;Acc:HGNC:8725]" | 4933 | 0.150 | 0.4853 | Yes |
| 21 | PGAM1 | phosphoglycerate mutase 1 [Source:HGNC Symbol;Acc:HGNC:8888] | 4977 | 0.149 | 0.4992 | Yes |
| 22 | GALM | galactose mutarotase [Source:HGNC Symbol;Acc:HGNC:24063] | 5076 | 0.147 | 0.5115 | Yes |
| 23 | ADH1A | "alcohol dehydrogenase 1A (class I), alpha polypeptide [Source:HGNC Symbol;Acc:HGNC:249]" | 5185 | 0.144 | 0.5233 | Yes |
| 24 | HK2 | hexokinase 2 [Source:HGNC Symbol;Acc:HGNC:4923] | 5226 | 0.143 | 0.5368 | Yes |
| 25 | PGK1 | phosphoglycerate kinase 1 [Source:HGNC Symbol;Acc:HGNC:8896] | 5390 | 0.140 | 0.5467 | Yes |
| 26 | PGM1 | phosphoglucomutase 1 [Source:HGNC Symbol;Acc:HGNC:8905] | 5640 | 0.134 | 0.5539 | Yes |
| 27 | ALDOA | "aldolase, fructose-bisphosphate A [Source:HGNC Symbol;Acc:HGNC:414]" | 5681 | 0.133 | 0.5663 | Yes |
| 28 | GAPDH | glyceraldehyde-3-phosphate dehydrogenase [Source:HGNC Symbol;Acc:HGNC:4141] | 5712 | 0.133 | 0.5790 | Yes |
| 29 | FBP2 | fructose-bisphosphatase 2 [Source:HGNC Symbol;Acc:HGNC:3607] | 6752 | 0.113 | 0.5640 | No |
| 30 | ACSS2 | acyl-CoA synthetase short chain family member 2 [Source:HGNC Symbol;Acc:HGNC:15814] | 7325 | 0.104 | 0.5599 | No |
| 31 | ALDH3B1 | aldehyde dehydrogenase 3 family member B1 [Source:HGNC Symbol;Acc:HGNC:410] | 7513 | 0.101 | 0.5653 | No |
| 32 | PGK2 | phosphoglycerate kinase 2 [Source:HGNC Symbol;Acc:HGNC:8898] | 8363 | 0.090 | 0.5528 | No |
| 33 | ADH4 | "alcohol dehydrogenase 4 (class II), pi polypeptide [Source:HGNC Symbol;Acc:HGNC:252]" | 8741 | 0.085 | 0.5519 | No |
| 34 | PDHA2 | pyruvate dehydrogenase E1 subunit alpha 2 [Source:HGNC Symbol;Acc:HGNC:8807] | 8806 | 0.085 | 0.5588 | No |
| 35 | PKM | pyruvate kinase M1/2 [Source:HGNC Symbol;Acc:HGNC:9021] | 9357 | 0.078 | 0.5526 | No |
| 36 | ENO3 | enolase 3 [Source:HGNC Symbol;Acc:HGNC:3354] | 10354 | 0.067 | 0.5340 | No |
| 37 | ALDH7A1 | aldehyde dehydrogenase 7 family member A1 [Source:HGNC Symbol;Acc:HGNC:877] | 14303 | 0.036 | 0.4371 | No |
| 38 | PFKL | "phosphofructokinase, liver type [Source:HGNC Symbol;Acc:HGNC:8876]" | 14439 | 0.035 | 0.4373 | No |
| 39 | ALDH1A3 | aldehyde dehydrogenase 1 family member A3 [Source:HGNC Symbol;Acc:HGNC:409] | 14473 | 0.035 | 0.4400 | No |
| 40 | GCK | glucokinase [Source:HGNC Symbol;Acc:HGNC:4195] | 16510 | 0.023 | 0.3904 | No |
| 41 | ALDOC | "aldolase, fructose-bisphosphate C [Source:HGNC Symbol;Acc:HGNC:418]" | 18656 | 0.011 | 0.3369 | No |
| 42 | FBP1 | fructose-bisphosphatase 1 [Source:HGNC Symbol;Acc:HGNC:3606] | 20203 | 0.002 | 0.2977 | No |
| 43 | G6PC2 | glucose-6-phosphatase catalytic subunit 2 [Source:HGNC Symbol;Acc:HGNC:28906] | 20542 | 0.001 | 0.2892 | No |
| 44 | LDHAL6B | lactate dehydrogenase A like 6B [Source:HGNC Symbol;Acc:HGNC:21481] | 20658 | -0.000 | 0.2863 | No |
| 45 | ALDH3B2 | aldehyde dehydrogenase 3 family member B2 [Source:HGNC Symbol;Acc:HGNC:411] | 22825 | -0.013 | 0.2324 | No |
| 46 | G6PC | glucose-6-phosphatase catalytic subunit [Source:HGNC Symbol;Acc:HGNC:4056] | 24013 | -0.020 | 0.2042 | No |
| 47 | ALDH3A1 | aldehyde dehydrogenase 3 family member A1 [Source:HGNC Symbol;Acc:HGNC:405] | 24960 | -0.026 | 0.1827 | No |
| 48 | PGAM4 | phosphoglycerate mutase family member 4 [Source:HGNC Symbol;Acc:HGNC:21731] | 25086 | -0.027 | 0.1823 | No |
| 49 | ADH7 | "alcohol dehydrogenase 7 (class IV), mu or sigma polypeptide [Source:HGNC Symbol;Acc:HGNC:256]" | 26557 | -0.038 | 0.1487 | No |
| 50 | LDHC | lactate dehydrogenase C [Source:HGNC Symbol;Acc:HGNC:6544] | 29290 | -0.064 | 0.0855 | No |
| 51 | PFKP | "phosphofructokinase, platelet [Source:HGNC Symbol;Acc:HGNC:8878]" | 29511 | -0.066 | 0.0866 | No |
| 52 | PKLR | pyruvate kinase L/R [Source:HGNC Symbol;Acc:HGNC:9020] | 31596 | -0.093 | 0.0429 | No |
| 53 | ENO2 | enolase 2 [Source:HGNC Symbol;Acc:HGNC:3353] | 32931 | -0.118 | 0.0208 | No |
| 54 | HK3 | hexokinase 3 [Source:HGNC Symbol;Acc:HGNC:4925] | 34268 | -0.149 | 0.0018 | No |
| 55 | ADH1B | "alcohol dehydrogenase 1B (class I), beta polypeptide [Source:HGNC Symbol;Acc:HGNC:250]" | 34320 | -0.150 | 0.0157 | No |
| 56 | ALDH2 | aldehyde dehydrogenase 2 family member [Source:HGNC Symbol;Acc:HGNC:404] | 34564 | -0.157 | 0.0254 | No |
| 57 | ALDOB | "aldolase, fructose-bisphosphate B [Source:HGNC Symbol;Acc:HGNC:417]" | 34883 | -0.167 | 0.0341 | No |
| 58 | BPGM | bisphosphoglycerate mutase [Source:HGNC Symbol;Acc:HGNC:1093] | 35126 | -0.175 | 0.0456 | No |
| 59 | PCK1 | phosphoenolpyruvate carboxykinase 1 [Source:HGNC Symbol;Acc:HGNC:8724] | 35185 | -0.177 | 0.0620 | No |
| 60 | ADH1C | "alcohol dehydrogenase 1C (class I), gamma polypeptide [Source:HGNC Symbol;Acc:HGNC:251]" | 35727 | -0.197 | 0.0681 | No |
| 61 | HK1 | hexokinase 1 [Source:HGNC Symbol;Acc:HGNC:4922] | 36337 | -0.222 | 0.0750 | No |
| 62 | PGAM2 | phosphoglycerate mutase 2 [Source:HGNC Symbol;Acc:HGNC:8889] | 39065 | NaN | 0.0066 | No |
Table: GSEA details [plain text format]

  

Fig 2: KEGG\_GLYCOLYSIS\_GLUCONEOGENESIS      
 Blue-Pink O' Gram in the Space of the Analyzed GeneSet

  

Fig 3: KEGG\_GLYCOLYSIS\_GLUCONEOGENESIS: Random ES distribution      
 Gene set null distribution of ES for **KEGG\_GLYCOLYSIS\_GLUCONEOGENESIS**
