# Supplementary material for: Simultaneous Presentation of Multiple Myeloma and Lung Cancer: Case Report and Gene Bioinformatics Analysis
Source: Front Oncol. 2022 Jun 13;12:859735. doi: 10.3389/fonc.2022.859735 (PMC9235397; doi:10.3389/fonc.2022.859735)
Supplement: Supplementary file 1 [file DataSheet_1.zip › The bioinformatic analysis of MM and lung cancer supplementary materials/Enrichment analysis/MECR/GSEA_4.1.0/MM TCGA/KEGG.Gsea.1639040591792/KEGG_GLYCOSYLPHOSPHATIDYLINOSITOL_GPI_ANCHOR_BIOSYNTHESIS.html]

Details for gene set KEGG\_GLYCOSYLPHOSPHATIDYLINOSITOL\_GPI\_ANCHOR\_BIOSYNTHESIS[GSEA]

|  || Dataset | ExpData\_collapsed\_to\_symbols.ENSG00000116353\_profile\_in\_ExpData.cls #ENSG00000116353 |
| Phenotype | ENSG00000116353\_profile\_in\_ExpData.cls#ENSG00000116353 |
| Upregulated in class | ENSG00000116353\_pos |
| GeneSet | KEGG\_GLYCOSYLPHOSPHATIDYLINOSITOL\_GPI\_ANCHOR\_BIOSYNTHESIS |
| Enrichment Score (ES) | 0.6896853 |
| Normalized Enrichment Score (NES) | 1.9282337 |
| Nominal p-value | 0.0 |
| FDR q-value | 6.158769E-4 |
| FWER p-Value | 0.016 |
Table: GSEA Results Summary

  

Fig 1: Enrichment plot: KEGG\_GLYCOSYLPHOSPHATIDYLINOSITOL\_GPI\_ANCHOR\_BIOSYNTHESIS      
 Profile of the Running ES Score & Positions of GeneSet Members on the Rank Ordered List

  

| SYMBOL | TITLE | RANK IN GENE LIST | RANK METRIC SCORE | RUNNING ES | CORE ENRICHMENT || 1 | PIGF | phosphatidylinositol glycan anchor biosynthesis class F [Source:HGNC Symbol;Acc:HGNC:8962] | 192 | 0.487 | 0.0856 | Yes |
| 2 | PIGU | phosphatidylinositol glycan anchor biosynthesis class U [Source:HGNC Symbol;Acc:HGNC:15791] | 245 | 0.469 | 0.1714 | Yes |
| 3 | PIGV | phosphatidylinositol glycan anchor biosynthesis class V [Source:HGNC Symbol;Acc:HGNC:26031] | 436 | 0.428 | 0.2460 | Yes |
| 4 | PIGN | phosphatidylinositol glycan anchor biosynthesis class N [Source:HGNC Symbol;Acc:HGNC:8967] | 711 | 0.380 | 0.3097 | Yes |
| 5 | PIGO | phosphatidylinositol glycan anchor biosynthesis class O [Source:HGNC Symbol;Acc:HGNC:23215] | 856 | 0.361 | 0.3731 | Yes |
| 6 | PIGW | phosphatidylinositol glycan anchor biosynthesis class W [Source:HGNC Symbol;Acc:HGNC:23213] | 938 | 0.350 | 0.4361 | Yes |
| 7 | PIGM | phosphatidylinositol glycan anchor biosynthesis class M [Source:HGNC Symbol;Acc:HGNC:18858] | 1193 | 0.325 | 0.4901 | Yes |
| 8 | PIGP | phosphatidylinositol glycan anchor biosynthesis class P [Source:HGNC Symbol;Acc:HGNC:3046] | 1737 | 0.281 | 0.5285 | Yes |
| 9 | PIGX | phosphatidylinositol glycan anchor biosynthesis class X [Source:HGNC Symbol;Acc:HGNC:26046] | 1780 | 0.277 | 0.5789 | Yes |
| 10 | GPAA1 | glycosylphosphatidylinositol anchor attachment 1 [Source:HGNC Symbol;Acc:HGNC:4446] | 1958 | 0.266 | 0.6238 | Yes |
| 11 | PIGK | phosphatidylinositol glycan anchor biosynthesis class K [Source:HGNC Symbol;Acc:HGNC:8965] | 2364 | 0.244 | 0.6588 | Yes |
| 12 | PIGC | phosphatidylinositol glycan anchor biosynthesis class C [Source:HGNC Symbol;Acc:HGNC:8960] | 3140 | 0.207 | 0.6776 | Yes |
| 13 | PIGG | phosphatidylinositol glycan anchor biosynthesis class G [Source:HGNC Symbol;Acc:HGNC:25985] | 3963 | 0.177 | 0.6897 | Yes |
| 14 | PIGT | phosphatidylinositol glycan anchor biosynthesis class T [Source:HGNC Symbol;Acc:HGNC:14938] | 6965 | 0.110 | 0.6338 | No |
| 15 | GPLD1 | glycosylphosphatidylinositol specific phospholipase D1 [Source:HGNC Symbol;Acc:HGNC:4459] | 7294 | 0.105 | 0.6448 | No |
| 16 | PIGB | phosphatidylinositol glycan anchor biosynthesis class B [Source:HGNC Symbol;Acc:HGNC:8959] | 11615 | 0.055 | 0.5452 | No |
| 17 | DPM2 | "dolichyl-phosphate mannosyltransferase subunit 2, regulatory [Source:HGNC Symbol;Acc:HGNC:3006]" | 13794 | 0.039 | 0.4971 | No |
| 18 | PIGZ | phosphatidylinositol glycan anchor biosynthesis class Z [Source:HGNC Symbol;Acc:HGNC:30596] | 18302 | 0.013 | 0.3848 | No |
| 19 | PGAP1 | post-GPI attachment to proteins inositol deacylase 1 [Source:HGNC Symbol;Acc:HGNC:25712] | 21887 | -0.007 | 0.2949 | No |
| 20 | PIGQ | phosphatidylinositol glycan anchor biosynthesis class Q [Source:HGNC Symbol;Acc:HGNC:14135] | 22625 | -0.011 | 0.2783 | No |
| 21 | PIGL | phosphatidylinositol glycan anchor biosynthesis class L [Source:HGNC Symbol;Acc:HGNC:8966] | 33244 | -0.124 | 0.0311 | No |
| 22 | PIGH | phosphatidylinositol glycan anchor biosynthesis class H [Source:HGNC Symbol;Acc:HGNC:8964] | 33875 | -0.139 | 0.0408 | No |
| 23 | PIGS | phosphatidylinositol glycan anchor biosynthesis class S [Source:HGNC Symbol;Acc:HGNC:14937] | 35528 | -0.189 | 0.0339 | No |
| 24 | PIGA | phosphatidylinositol glycan anchor biosynthesis class A [Source:HGNC Symbol;Acc:HGNC:8957] | 37817 | -0.327 | 0.0364 | No |
| 25 | PIGY | phosphatidylinositol glycan anchor biosynthesis class Y [Source:HGNC Symbol;Acc:HGNC:28213] | 38974 | NaN | 0.0089 | No |
Table: GSEA details [plain text format]

  

Fig 2: KEGG\_GLYCOSYLPHOSPHATIDYLINOSITOL\_GPI\_ANCHOR\_BIOSYNTHESIS      
 Blue-Pink O' Gram in the Space of the Analyzed GeneSet

  

Fig 3: KEGG\_GLYCOSYLPHOSPHATIDYLINOSITOL\_GPI\_ANCHOR\_BIOSYNTHESIS: Random ES distribution      
 Gene set null distribution of ES for **KEGG\_GLYCOSYLPHOSPHATIDYLINOSITOL\_GPI\_ANCHOR\_BIOSYNTHESIS**
