# Supplementary material for: Simultaneous Presentation of Multiple Myeloma and Lung Cancer: Case Report and Gene Bioinformatics Analysis
Source: Front Oncol. 2022 Jun 13;12:859735. doi: 10.3389/fonc.2022.859735 (PMC9235397; doi:10.3389/fonc.2022.859735)
Supplement: Supplementary file 1 [file DataSheet_1.zip › The bioinformatic analysis of MM and lung cancer supplementary materials/Enrichment analysis/MECR/GSEA_4.1.0/MM TCGA/KEGG.Gsea.1639040591792/KEGG_GRAFT_VERSUS_HOST_DISEASE.html]

Details for gene set KEGG\_GRAFT\_VERSUS\_HOST\_DISEASE[GSEA]

|  || Dataset | ExpData\_collapsed\_to\_symbols.ENSG00000116353\_profile\_in\_ExpData.cls #ENSG00000116353 |
| Phenotype | ENSG00000116353\_profile\_in\_ExpData.cls#ENSG00000116353 |
| Upregulated in class | ENSG00000116353\_neg |
| GeneSet | KEGG\_GRAFT\_VERSUS\_HOST\_DISEASE |
| Enrichment Score (ES) | -0.64491373 |
| Normalized Enrichment Score (NES) | -2.167387 |
| Nominal p-value | 0.0 |
| FDR q-value | 0.0 |
| FWER p-Value | 0.0 |
Table: GSEA Results Summary

  

Fig 1: Enrichment plot: KEGG\_GRAFT\_VERSUS\_HOST\_DISEASE      
 Profile of the Running ES Score & Positions of GeneSet Members on the Rank Ordered List

  

| SYMBOL | TITLE | RANK IN GENE LIST | RANK METRIC SCORE | RUNNING ES | CORE ENRICHMENT || 1 | KLRC1 | killer cell lectin like receptor C1 [Source:HGNC Symbol;Acc:HGNC:6374] | 8826 | 0.084 | -0.2025 | No |
| 2 | HLA-C | "major histocompatibility complex, class I, C [Source:HGNC Symbol;Acc:HGNC:4933]" | 12475 | 0.049 | -0.2825 | No |
| 3 | KIR3DL2 | "killer cell immunoglobulin like receptor, three Ig domains and long cytoplasmic tail 2 [Source:HGNC Symbol;Acc:HGNC:6339]" | 17576 | 0.017 | -0.4079 | No |
| 4 | CD28 | CD28 molecule [Source:HGNC Symbol;Acc:HGNC:1653] | 18732 | 0.011 | -0.4346 | No |
| 5 | HLA-G | "major histocompatibility complex, class I, G [Source:HGNC Symbol;Acc:HGNC:4964]" | 19374 | 0.007 | -0.4490 | No |
| 6 | FASLG | Fas ligand [Source:HGNC Symbol;Acc:HGNC:11936] | 21141 | -0.003 | -0.4932 | No |
| 7 | KIR3DL1 | "killer cell immunoglobulin like receptor, three Ig domains and long cytoplasmic tail 1 [Source:HGNC Symbol;Acc:HGNC:6338]" | 22356 | -0.010 | -0.5215 | No |
| 8 | IL2 | interleukin 2 [Source:HGNC Symbol;Acc:HGNC:6001] | 25091 | -0.027 | -0.5840 | No |
| 9 | IFNG | interferon gamma [Source:HGNC Symbol;Acc:HGNC:5438] | 25529 | -0.030 | -0.5872 | No |
| 10 | HLA-DQA2 | "major histocompatibility complex, class II, DQ alpha 2 [Source:HGNC Symbol;Acc:HGNC:4943]" | 25697 | -0.031 | -0.5832 | No |
| 11 | KIR2DL1 | "killer cell immunoglobulin like receptor, two Ig domains and long cytoplasmic tail 1 [Source:HGNC Symbol;Acc:HGNC:6329]" | 28123 | -0.051 | -0.6314 | Yes |
| 12 | KIR2DL3 | "killer cell immunoglobulin like receptor, two Ig domains and long cytoplasmic tail 3 [Source:HGNC Symbol;Acc:HGNC:6331]" | 28267 | -0.053 | -0.6212 | Yes |
| 13 | KLRD1 | killer cell lectin like receptor D1 [Source:HGNC Symbol;Acc:HGNC:6378] | 28618 | -0.056 | -0.6153 | Yes |
| 14 | HLA-DOB | "major histocompatibility complex, class II, DO beta [Source:HGNC Symbol;Acc:HGNC:4937]" | 28713 | -0.057 | -0.6026 | Yes |
| 15 | HLA-B | "major histocompatibility complex, class I, B [Source:HGNC Symbol;Acc:HGNC:4932]" | 28822 | -0.058 | -0.5900 | Yes |
| 16 | IL6 | interleukin 6 [Source:HGNC Symbol;Acc:HGNC:6018] | 29703 | -0.068 | -0.5944 | Yes |
| 17 | HLA-A | "major histocompatibility complex, class I, A [Source:HGNC Symbol;Acc:HGNC:4931]" | 30839 | -0.083 | -0.6016 | Yes |
| 18 | PRF1 | perforin 1 [Source:HGNC Symbol;Acc:HGNC:9360] | 31562 | -0.092 | -0.5956 | Yes |
| 19 | HLA-DOA | "major histocompatibility complex, class II, DO alpha [Source:HGNC Symbol;Acc:HGNC:4936]" | 31604 | -0.093 | -0.5722 | Yes |
| 20 | GZMB | granzyme B [Source:HGNC Symbol;Acc:HGNC:4709] | 32016 | -0.100 | -0.5563 | Yes |
| 21 | HLA-F | "major histocompatibility complex, class I, F [Source:HGNC Symbol;Acc:HGNC:4963]" | 32365 | -0.106 | -0.5372 | Yes |
| 22 | IL1A | interleukin 1 alpha [Source:HGNC Symbol;Acc:HGNC:5991] | 32718 | -0.113 | -0.5164 | Yes |
| 23 | HLA-DQA1 | "major histocompatibility complex, class II, DQ alpha 1 [Source:HGNC Symbol;Acc:HGNC:4942]" | 32959 | -0.118 | -0.4913 | Yes |
| 24 | CD80 | CD80 molecule [Source:HGNC Symbol;Acc:HGNC:1700] | 33283 | -0.124 | -0.4668 | Yes |
| 25 | HLA-DRB5 | "major histocompatibility complex, class II, DR beta 5 [Source:HGNC Symbol;Acc:HGNC:4953]" | 33950 | -0.140 | -0.4469 | Yes |
| 26 | CD86 | CD86 molecule [Source:HGNC Symbol;Acc:HGNC:1705] | 34067 | -0.143 | -0.4122 | Yes |
| 27 | HLA-DPA1 | "major histocompatibility complex, class II, DP alpha 1 [Source:HGNC Symbol;Acc:HGNC:4938]" | 34382 | -0.152 | -0.3802 | Yes |
| 28 | HLA-DPB1 | "major histocompatibility complex, class II, DP beta 1 [Source:HGNC Symbol;Acc:HGNC:4940]" | 34547 | -0.157 | -0.3431 | Yes |
| 29 | HLA-DRB1 | "major histocompatibility complex, class II, DR beta 1 [Source:HGNC Symbol;Acc:HGNC:4948]" | 34560 | -0.157 | -0.3020 | Yes |
| 30 | HLA-DMB | "major histocompatibility complex, class II, DM beta [Source:HGNC Symbol;Acc:HGNC:4935]" | 34608 | -0.159 | -0.2614 | Yes |
| 31 | HLA-DMA | "major histocompatibility complex, class II, DM alpha [Source:HGNC Symbol;Acc:HGNC:4934]" | 34644 | -0.160 | -0.2201 | Yes |
| 32 | HLA-DQB1 | "major histocompatibility complex, class II, DQ beta 1 [Source:HGNC Symbol;Acc:HGNC:4944]" | 34874 | -0.167 | -0.1820 | Yes |
| 33 | HLA-DRA | "major histocompatibility complex, class II, DR alpha [Source:HGNC Symbol;Acc:HGNC:4947]" | 35586 | -0.191 | -0.1498 | Yes |
| 34 | IL1B | interleukin 1 beta [Source:HGNC Symbol;Acc:HGNC:5992] | 35858 | -0.202 | -0.1036 | Yes |
| 35 | TNF | tumor necrosis factor [Source:HGNC Symbol;Acc:HGNC:11892] | 36419 | -0.226 | -0.0584 | Yes |
| 36 | FAS | Fas cell surface death receptor [Source:HGNC Symbol;Acc:HGNC:11920] | 36825 | -0.248 | -0.0033 | Yes |
| 37 | HLA-E | "major histocompatibility complex, class I, E [Source:HGNC Symbol;Acc:HGNC:4962]" | 36918 | -0.254 | 0.0612 | Yes |
Table: GSEA details [plain text format]

  

Fig 2: KEGG\_GRAFT\_VERSUS\_HOST\_DISEASE      
 Blue-Pink O' Gram in the Space of the Analyzed GeneSet

  

Fig 3: KEGG\_GRAFT\_VERSUS\_HOST\_DISEASE: Random ES distribution      
 Gene set null distribution of ES for **KEGG\_GRAFT\_VERSUS\_HOST\_DISEASE**
