# Supplementary material for: Simultaneous Presentation of Multiple Myeloma and Lung Cancer: Case Report and Gene Bioinformatics Analysis
Source: Front Oncol. 2022 Jun 13;12:859735. doi: 10.3389/fonc.2022.859735 (PMC9235397; doi:10.3389/fonc.2022.859735)
Supplement: Supplementary file 1 [file DataSheet_1.zip › The bioinformatic analysis of MM and lung cancer supplementary materials/Enrichment analysis/MECR/GSEA_4.1.0/MM TCGA/KEGG.Gsea.1639040591792/KEGG_HEMATOPOIETIC_CELL_LINEAGE.html]

Details for gene set KEGG\_HEMATOPOIETIC\_CELL\_LINEAGE[GSEA]

|  || Dataset | ExpData\_collapsed\_to\_symbols.ENSG00000116353\_profile\_in\_ExpData.cls #ENSG00000116353 |
| Phenotype | ENSG00000116353\_profile\_in\_ExpData.cls#ENSG00000116353 |
| Upregulated in class | ENSG00000116353\_neg |
| GeneSet | KEGG\_HEMATOPOIETIC\_CELL\_LINEAGE |
| Enrichment Score (ES) | -0.55705196 |
| Normalized Enrichment Score (NES) | -2.1773944 |
| Nominal p-value | 0.0 |
| FDR q-value | 0.0 |
| FWER p-Value | 0.0 |
Table: GSEA Results Summary

  

Fig 1: Enrichment plot: KEGG\_HEMATOPOIETIC\_CELL\_LINEAGE      
 Profile of the Running ES Score & Positions of GeneSet Members on the Rank Ordered List

  

| SYMBOL | TITLE | RANK IN GENE LIST | RANK METRIC SCORE | RUNNING ES | CORE ENRICHMENT || 1 | CD38 | CD38 molecule [Source:HGNC Symbol;Acc:HGNC:1667] | 3639 | 0.188 | -0.0748 | No |
| 2 | CR2 | complement C3d receptor 2 [Source:HGNC Symbol;Acc:HGNC:2336] | 4100 | 0.174 | -0.0700 | No |
| 3 | IL6R | interleukin 6 receptor [Source:HGNC Symbol;Acc:HGNC:6019] | 4649 | 0.157 | -0.0690 | No |
| 4 | ITGA3 | integrin subunit alpha 3 [Source:HGNC Symbol;Acc:HGNC:6139] | 5916 | 0.129 | -0.0890 | No |
| 5 | EPO | erythropoietin [Source:HGNC Symbol;Acc:HGNC:3415] | 6234 | 0.122 | -0.0854 | No |
| 6 | IL11RA | interleukin 11 receptor subunit alpha [Source:HGNC Symbol;Acc:HGNC:5967] | 6283 | 0.122 | -0.0751 | No |
| 7 | IL5 | interleukin 5 [Source:HGNC Symbol;Acc:HGNC:6016] | 6370 | 0.120 | -0.0658 | No |
| 8 | CD3E | CD3e molecule [Source:HGNC Symbol;Acc:HGNC:1674] | 7210 | 0.106 | -0.0771 | No |
| 9 | CD33 | CD33 molecule [Source:HGNC Symbol;Acc:HGNC:1659] | 7801 | 0.097 | -0.0829 | No |
| 10 | IL9R | interleukin 9 receptor [Source:HGNC Symbol;Acc:HGNC:6030] | 7944 | 0.096 | -0.0774 | No |
| 11 | ITGA6 | integrin subunit alpha 6 [Source:HGNC Symbol;Acc:HGNC:6142] | 8958 | 0.083 | -0.0953 | No |
| 12 | THPO | thrombopoietin [Source:HGNC Symbol;Acc:HGNC:11795] | 9600 | 0.075 | -0.1045 | No |
| 13 | CD1D | CD1d molecule [Source:HGNC Symbol;Acc:HGNC:1637] | 10416 | 0.066 | -0.1189 | No |
| 14 | ITGA4 | integrin subunit alpha 4 [Source:HGNC Symbol;Acc:HGNC:6140] | 10741 | 0.063 | -0.1211 | No |
| 15 | TPO | thyroid peroxidase [Source:HGNC Symbol;Acc:HGNC:12015] | 10798 | 0.063 | -0.1166 | No |
| 16 | IL11 | interleukin 11 [Source:HGNC Symbol;Acc:HGNC:5966] | 12710 | 0.047 | -0.1608 | No |
| 17 | CSF2 | colony stimulating factor 2 [Source:HGNC Symbol;Acc:HGNC:2434] | 15864 | 0.027 | -0.2386 | No |
| 18 | CD1C | CD1c molecule [Source:HGNC Symbol;Acc:HGNC:1636] | 16118 | 0.025 | -0.2427 | No |
| 19 | ITGA2 | integrin subunit alpha 2 [Source:HGNC Symbol;Acc:HGNC:6137] | 17136 | 0.019 | -0.2668 | No |
| 20 | IL5RA | interleukin 5 receptor subunit alpha [Source:HGNC Symbol;Acc:HGNC:6017] | 17327 | 0.018 | -0.2699 | No |
| 21 | IL3 | interleukin 3 [Source:HGNC Symbol;Acc:HGNC:6011] | 19317 | 0.007 | -0.3199 | No |
| 22 | CD9 | CD9 molecule [Source:HGNC Symbol;Acc:HGNC:1709] | 19609 | 0.006 | -0.3267 | No |
| 23 | ITGA1 | integrin subunit alpha 1 [Source:HGNC Symbol;Acc:HGNC:6134] | 20236 | 0.002 | -0.3425 | No |
| 24 | TFRC | transferrin receptor [Source:HGNC Symbol;Acc:HGNC:11763] | 20952 | -0.002 | -0.3605 | No |
| 25 | FLT3LG | fms related receptor tyrosine kinase 3 ligand [Source:HGNC Symbol;Acc:HGNC:3766] | 21002 | -0.002 | -0.3616 | No |
| 26 | CD1B | CD1b molecule [Source:HGNC Symbol;Acc:HGNC:1635] | 24752 | -0.025 | -0.4547 | No |
| 27 | CD1E | CD1e molecule [Source:HGNC Symbol;Acc:HGNC:1638] | 25594 | -0.031 | -0.4733 | No |
| 28 | CD5 | CD5 molecule [Source:HGNC Symbol;Acc:HGNC:1685] | 26348 | -0.036 | -0.4890 | No |
| 29 | CD8B | CD8b molecule [Source:HGNC Symbol;Acc:HGNC:1707] | 26459 | -0.037 | -0.4883 | No |
| 30 | CD1A | CD1a molecule [Source:HGNC Symbol;Acc:HGNC:1634] | 26571 | -0.038 | -0.4875 | No |
| 31 | DNTT | DNA nucleotidylexotransferase [Source:HGNC Symbol;Acc:HGNC:2983] | 26998 | -0.041 | -0.4944 | No |
| 32 | KIT | "KIT proto-oncogene, receptor tyrosine kinase [Source:HGNC Symbol;Acc:HGNC:6342]" | 27060 | -0.042 | -0.4920 | No |
| 33 | IL4 | interleukin 4 [Source:HGNC Symbol;Acc:HGNC:6014] | 28173 | -0.052 | -0.5154 | No |
| 34 | CD4 | CD4 molecule [Source:HGNC Symbol;Acc:HGNC:1678] | 28372 | -0.054 | -0.5154 | No |
| 35 | CD8A | CD8a molecule [Source:HGNC Symbol;Acc:HGNC:1706] | 29262 | -0.063 | -0.5320 | No |
| 36 | IL6 | interleukin 6 [Source:HGNC Symbol;Acc:HGNC:6018] | 29703 | -0.068 | -0.5367 | No |
| 37 | GP1BA | glycoprotein Ib platelet subunit alpha [Source:HGNC Symbol;Acc:HGNC:4439] | 30504 | -0.078 | -0.5496 | Yes |
| 38 | CD3G | CD3g molecule [Source:HGNC Symbol;Acc:HGNC:1675] | 30583 | -0.079 | -0.5440 | Yes |
| 39 | CD59 | CD59 molecule (CD59 blood group) [Source:HGNC Symbol;Acc:HGNC:1689] | 30625 | -0.080 | -0.5375 | Yes |
| 40 | GP9 | glycoprotein IX platelet [Source:HGNC Symbol;Acc:HGNC:4444] | 30973 | -0.084 | -0.5383 | Yes |
| 41 | CD34 | CD34 molecule [Source:HGNC Symbol;Acc:HGNC:1662] | 31571 | -0.093 | -0.5447 | Yes |
| 42 | CR1 | complement C3b/C4b receptor 1 (Knops blood group) [Source:HGNC Symbol;Acc:HGNC:2334] | 31715 | -0.095 | -0.5393 | Yes |
| 43 | ITGB3 | integrin subunit beta 3 [Source:HGNC Symbol;Acc:HGNC:6156] | 31798 | -0.096 | -0.5322 | Yes |
| 44 | CD2 | CD2 molecule [Source:HGNC Symbol;Acc:HGNC:1639] | 31914 | -0.098 | -0.5257 | Yes |
| 45 | MME | membrane metalloendopeptidase [Source:HGNC Symbol;Acc:HGNC:7154] | 32036 | -0.100 | -0.5193 | Yes |
| 46 | IL3RA | interleukin 3 receptor subunit alpha [Source:HGNC Symbol;Acc:HGNC:6012] | 32087 | -0.101 | -0.5109 | Yes |
| 47 | CD55 | CD55 molecule (Cromer blood group) [Source:HGNC Symbol;Acc:HGNC:2665] | 32187 | -0.103 | -0.5036 | Yes |
| 48 | CD22 | CD22 molecule [Source:HGNC Symbol;Acc:HGNC:1643] | 32416 | -0.107 | -0.4992 | Yes |
| 49 | FCGR1A | Fc fragment of IgG receptor Ia [Source:HGNC Symbol;Acc:HGNC:3613] | 32623 | -0.111 | -0.4938 | Yes |
| 50 | IL1A | interleukin 1 alpha [Source:HGNC Symbol;Acc:HGNC:5991] | 32718 | -0.113 | -0.4854 | Yes |
| 51 | CSF1R | colony stimulating factor 1 receptor [Source:HGNC Symbol;Acc:HGNC:2433] | 33122 | -0.121 | -0.4842 | Yes |
| 52 | CD3D | CD3d molecule [Source:HGNC Symbol;Acc:HGNC:1673] | 33390 | -0.127 | -0.4789 | Yes |
| 53 | GYPA | glycophorin A (MNS blood group) [Source:HGNC Symbol;Acc:HGNC:4702] | 33566 | -0.131 | -0.4709 | Yes |
| 54 | CD37 | CD37 molecule [Source:HGNC Symbol;Acc:HGNC:1666] | 33584 | -0.131 | -0.4589 | Yes |
| 55 | GP5 | glycoprotein V platelet [Source:HGNC Symbol;Acc:HGNC:4443] | 33710 | -0.134 | -0.4493 | Yes |
| 56 | EPOR | erythropoietin receptor [Source:HGNC Symbol;Acc:HGNC:3416] | 33949 | -0.140 | -0.4420 | Yes |
| 57 | HLA-DRB5 | "major histocompatibility complex, class II, DR beta 5 [Source:HGNC Symbol;Acc:HGNC:4953]" | 33950 | -0.140 | -0.4287 | Yes |
| 58 | HLA-DRB1 | "major histocompatibility complex, class II, DR beta 1 [Source:HGNC Symbol;Acc:HGNC:4948]" | 34560 | -0.157 | -0.4292 | Yes |
| 59 | IL7 | interleukin 7 [Source:HGNC Symbol;Acc:HGNC:6023] | 34913 | -0.168 | -0.4221 | Yes |
| 60 | CD7 | CD7 molecule [Source:HGNC Symbol;Acc:HGNC:1695] | 34918 | -0.168 | -0.4062 | Yes |
| 61 | FCER2 | Fc fragment of IgE receptor II [Source:HGNC Symbol;Acc:HGNC:3612] | 35228 | -0.179 | -0.3971 | Yes |
| 62 | MS4A1 | membrane spanning 4-domains A1 [Source:HGNC Symbol;Acc:HGNC:7315] | 35364 | -0.183 | -0.3830 | Yes |
| 63 | KITLG | KIT ligand [Source:HGNC Symbol;Acc:HGNC:6343] | 35389 | -0.185 | -0.3660 | Yes |
| 64 | CD36 | CD36 molecule [Source:HGNC Symbol;Acc:HGNC:1663] | 35440 | -0.186 | -0.3496 | Yes |
| 65 | HLA-DRA | "major histocompatibility complex, class II, DR alpha [Source:HGNC Symbol;Acc:HGNC:4947]" | 35586 | -0.191 | -0.3351 | Yes |
| 66 | CD19 | CD19 molecule [Source:HGNC Symbol;Acc:HGNC:1633] | 35767 | -0.198 | -0.3208 | Yes |
| 67 | IL1B | interleukin 1 beta [Source:HGNC Symbol;Acc:HGNC:5992] | 35858 | -0.202 | -0.3038 | Yes |
| 68 | IL7R | interleukin 7 receptor [Source:HGNC Symbol;Acc:HGNC:6024] | 36033 | -0.209 | -0.2884 | Yes |
| 69 | IL2RA | interleukin 2 receptor subunit alpha [Source:HGNC Symbol;Acc:HGNC:6008] | 36108 | -0.212 | -0.2701 | Yes |
| 70 | FLT3 | fms related receptor tyrosine kinase 3 [Source:HGNC Symbol;Acc:HGNC:3765] | 36256 | -0.218 | -0.2531 | Yes |
| 71 | CD14 | CD14 molecule [Source:HGNC Symbol;Acc:HGNC:1628] | 36298 | -0.220 | -0.2332 | Yes |
| 72 | CSF3 | colony stimulating factor 3 [Source:HGNC Symbol;Acc:HGNC:2438] | 36399 | -0.225 | -0.2143 | Yes |
| 73 | TNF | tumor necrosis factor [Source:HGNC Symbol;Acc:HGNC:11892] | 36419 | -0.226 | -0.1932 | Yes |
| 74 | CSF2RA | colony stimulating factor 2 receptor subunit alpha [Source:HGNC Symbol;Acc:HGNC:2435] | 36454 | -0.228 | -0.1724 | Yes |
| 75 | ITGA5 | integrin subunit alpha 5 [Source:HGNC Symbol;Acc:HGNC:6141] | 36513 | -0.231 | -0.1518 | Yes |
| 76 | CSF1 | colony stimulating factor 1 [Source:HGNC Symbol;Acc:HGNC:2432] | 36518 | -0.231 | -0.1299 | Yes |
| 77 | CD44 | CD44 molecule (Indian blood group) [Source:HGNC Symbol;Acc:HGNC:1681] | 36621 | -0.237 | -0.1099 | Yes |
| 78 | IL4R | interleukin 4 receptor [Source:HGNC Symbol;Acc:HGNC:6015] | 36680 | -0.240 | -0.0885 | Yes |
| 79 | ITGAM | integrin subunit alpha M [Source:HGNC Symbol;Acc:HGNC:6149] | 36727 | -0.243 | -0.0665 | Yes |
| 80 | IL1R2 | interleukin 1 receptor type 2 [Source:HGNC Symbol;Acc:HGNC:5994] | 36783 | -0.246 | -0.0445 | Yes |
| 81 | CSF3R | colony stimulating factor 3 receptor [Source:HGNC Symbol;Acc:HGNC:2439] | 36961 | -0.257 | -0.0246 | Yes |
| 82 | ITGA2B | integrin subunit alpha 2b [Source:HGNC Symbol;Acc:HGNC:6138] | 37349 | -0.284 | -0.0074 | Yes |
| 83 | ANPEP | "alanyl aminopeptidase, membrane [Source:HGNC Symbol;Acc:HGNC:500]" | 37495 | -0.296 | 0.0171 | Yes |
| 84 | IL1R1 | interleukin 1 receptor type 1 [Source:HGNC Symbol;Acc:HGNC:5993] | 37530 | -0.299 | 0.0447 | Yes |
| 85 | GP1BB | glycoprotein Ib platelet subunit beta [Source:HGNC Symbol;Acc:HGNC:4440] | 39098 | NaN | 0.0057 | Yes |
Table: GSEA details [plain text format]

  

Fig 2: KEGG\_HEMATOPOIETIC\_CELL\_LINEAGE      
 Blue-Pink O' Gram in the Space of the Analyzed GeneSet

  

Fig 3: KEGG\_HEMATOPOIETIC\_CELL\_LINEAGE: Random ES distribution      
 Gene set null distribution of ES for **KEGG\_HEMATOPOIETIC\_CELL\_LINEAGE**
