# Supplementary material for: Simultaneous Presentation of Multiple Myeloma and Lung Cancer: Case Report and Gene Bioinformatics Analysis
Source: Front Oncol. 2022 Jun 13;12:859735. doi: 10.3389/fonc.2022.859735 (PMC9235397; doi:10.3389/fonc.2022.859735)
Supplement: Supplementary file 1 [file DataSheet_1.zip › The bioinformatic analysis of MM and lung cancer supplementary materials/Enrichment analysis/MECR/GSEA_4.1.0/MM TCGA/KEGG.Gsea.1639040591792/KEGG_HOMOLOGOUS_RECOMBINATION.html]

Details for gene set KEGG\_HOMOLOGOUS\_RECOMBINATION[GSEA]

|  || Dataset | ExpData\_collapsed\_to\_symbols.ENSG00000116353\_profile\_in\_ExpData.cls #ENSG00000116353 |
| Phenotype | ENSG00000116353\_profile\_in\_ExpData.cls#ENSG00000116353 |
| Upregulated in class | ENSG00000116353\_pos |
| GeneSet | KEGG\_HOMOLOGOUS\_RECOMBINATION |
| Enrichment Score (ES) | 0.6914473 |
| Normalized Enrichment Score (NES) | 2.0154676 |
| Nominal p-value | 0.0 |
| FDR q-value | 3.489952E-4 |
| FWER p-Value | 0.007 |
Table: GSEA Results Summary

  

Fig 1: Enrichment plot: KEGG\_HOMOLOGOUS\_RECOMBINATION      
 Profile of the Running ES Score & Positions of GeneSet Members on the Rank Ordered List

  

| SYMBOL | TITLE | RANK IN GENE LIST | RANK METRIC SCORE | RUNNING ES | CORE ENRICHMENT || 1 | POLD2 | "DNA polymerase delta 2, accessory subunit [Source:HGNC Symbol;Acc:HGNC:9176]" | 15 | 0.617 | 0.0954 | Yes |
| 2 | RPA1 | replication protein A1 [Source:HGNC Symbol;Acc:HGNC:10289] | 360 | 0.442 | 0.1552 | Yes |
| 3 | POLD1 | "DNA polymerase delta 1, catalytic subunit [Source:HGNC Symbol;Acc:HGNC:9175]" | 541 | 0.405 | 0.2135 | Yes |
| 4 | RAD51C | RAD51 paralog C [Source:HGNC Symbol;Acc:HGNC:9820] | 562 | 0.402 | 0.2755 | Yes |
| 5 | RAD51D | RAD51 paralog D [Source:HGNC Symbol;Acc:HGNC:9823] | 619 | 0.395 | 0.3353 | Yes |
| 6 | XRCC2 | X-ray repair cross complementing 2 [Source:HGNC Symbol;Acc:HGNC:12829] | 806 | 0.368 | 0.3877 | Yes |
| 7 | RAD50 | RAD50 double strand break repair protein [Source:HGNC Symbol;Acc:HGNC:9816] | 1264 | 0.319 | 0.4256 | Yes |
| 8 | RAD54B | RAD54 homolog B [Source:HGNC Symbol;Acc:HGNC:17228] | 1386 | 0.308 | 0.4703 | Yes |
| 9 | POLD3 | "DNA polymerase delta 3, accessory subunit [Source:HGNC Symbol;Acc:HGNC:20932]" | 1700 | 0.284 | 0.5064 | Yes |
| 10 | RAD52 | "RAD52 homolog, DNA repair protein [Source:HGNC Symbol;Acc:HGNC:9824]" | 2092 | 0.259 | 0.5366 | Yes |
| 11 | SSBP1 | single stranded DNA binding protein 1 [Source:HGNC Symbol;Acc:HGNC:11317] | 2105 | 0.258 | 0.5762 | Yes |
| 12 | RPA3 | replication protein A3 [Source:HGNC Symbol;Acc:HGNC:10291] | 2705 | 0.227 | 0.5962 | Yes |
| 13 | RAD51 | RAD51 recombinase [Source:HGNC Symbol;Acc:HGNC:9817] | 2946 | 0.216 | 0.6236 | Yes |
| 14 | BLM | BLM RecQ like helicase [Source:HGNC Symbol;Acc:HGNC:1058] | 3593 | 0.190 | 0.6366 | Yes |
| 15 | TOP3A | DNA topoisomerase III alpha [Source:HGNC Symbol;Acc:HGNC:11992] | 3750 | 0.185 | 0.6613 | Yes |
| 16 | MUS81 | MUS81 structure-specific endonuclease subunit [Source:HGNC Symbol;Acc:HGNC:29814] | 3981 | 0.177 | 0.6829 | Yes |
| 17 | RAD54L | RAD54 like [Source:HGNC Symbol;Acc:HGNC:9826] | 4611 | 0.158 | 0.6914 | Yes |
| 18 | EME1 | essential meiotic structure-specific endonuclease 1 [Source:HGNC Symbol;Acc:HGNC:24965] | 6293 | 0.121 | 0.6675 | No |
| 19 | SEM1 | SEM1 26S proteasome complex subunit [Source:HGNC Symbol;Acc:HGNC:10845] | 6557 | 0.117 | 0.6789 | No |
| 20 | RAD51B | RAD51 paralog B [Source:HGNC Symbol;Acc:HGNC:9822] | 7848 | 0.097 | 0.6612 | No |
| 21 | RPA2 | replication protein A2 [Source:HGNC Symbol;Acc:HGNC:10290] | 7917 | 0.096 | 0.6743 | No |
| 22 | TOP3B | DNA topoisomerase III beta [Source:HGNC Symbol;Acc:HGNC:11993] | 10524 | 0.065 | 0.6181 | No |
| 23 | XRCC3 | X-ray repair cross complementing 3 [Source:HGNC Symbol;Acc:HGNC:12830] | 14703 | 0.034 | 0.5171 | No |
| 24 | MRE11 | "MRE11 homolog, double strand break repair nuclease [Source:HGNC Symbol;Acc:HGNC:7230]" | 19031 | 0.009 | 0.4084 | No |
| 25 | RPA4 | replication protein A4 [Source:HGNC Symbol;Acc:HGNC:30305] | 32283 | -0.105 | 0.0874 | No |
| 26 | POLD4 | "DNA polymerase delta 4, accessory subunit [Source:HGNC Symbol;Acc:HGNC:14106]" | 34723 | -0.162 | 0.0505 | No |
| 27 | NBN | nibrin [Source:HGNC Symbol;Acc:HGNC:7652] | 35500 | -0.188 | 0.0600 | No |
| 28 | BRCA2 | BRCA2 DNA repair associated [Source:HGNC Symbol;Acc:HGNC:1101] | 36677 | -0.240 | 0.0673 | No |
Table: GSEA details [plain text format]

  

Fig 2: KEGG\_HOMOLOGOUS\_RECOMBINATION      
 Blue-Pink O' Gram in the Space of the Analyzed GeneSet

  

Fig 3: KEGG\_HOMOLOGOUS\_RECOMBINATION: Random ES distribution      
 Gene set null distribution of ES for **KEGG\_HOMOLOGOUS\_RECOMBINATION**
