# Supplementary material for: Simultaneous Presentation of Multiple Myeloma and Lung Cancer: Case Report and Gene Bioinformatics Analysis
Source: Front Oncol. 2022 Jun 13;12:859735. doi: 10.3389/fonc.2022.859735 (PMC9235397; doi:10.3389/fonc.2022.859735)
Supplement: Supplementary file 1 [file DataSheet_1.zip › The bioinformatic analysis of MM and lung cancer supplementary materials/Enrichment analysis/MECR/GSEA_4.1.0/MM TCGA/KEGG.Gsea.1639040591792/KEGG_HUNTINGTONS_DISEASE.html]

Details for gene set KEGG\_HUNTINGTONS\_DISEASE[GSEA]

|  || Dataset | ExpData\_collapsed\_to\_symbols.ENSG00000116353\_profile\_in\_ExpData.cls #ENSG00000116353 |
| Phenotype | ENSG00000116353\_profile\_in\_ExpData.cls#ENSG00000116353 |
| Upregulated in class | ENSG00000116353\_pos |
| GeneSet | KEGG\_HUNTINGTONS\_DISEASE |
| Enrichment Score (ES) | 0.48083156 |
| Normalized Enrichment Score (NES) | 1.8967718 |
| Nominal p-value | 0.0 |
| FDR q-value | 9.985602E-4 |
| FWER p-Value | 0.028 |
Table: GSEA Results Summary

  

Fig 1: Enrichment plot: KEGG\_HUNTINGTONS\_DISEASE      
 Profile of the Running ES Score & Positions of GeneSet Members on the Rank Ordered List

  

| SYMBOL | TITLE | RANK IN GENE LIST | RANK METRIC SCORE | RUNNING ES | CORE ENRICHMENT || 1 | ATP5MC1 | ATP synthase membrane subunit c locus 1 [Source:HGNC Symbol;Acc:HGNC:841] | 70 | 0.551 | 0.0166 | Yes |
| 2 | NDUFB3 | NADH:ubiquinone oxidoreductase subunit B3 [Source:HGNC Symbol;Acc:HGNC:7698] | 252 | 0.467 | 0.0275 | Yes |
| 3 | SDHC | succinate dehydrogenase complex subunit C [Source:HGNC Symbol;Acc:HGNC:10682] | 333 | 0.448 | 0.0403 | Yes |
| 4 | NDUFA8 | NADH:ubiquinone oxidoreductase subunit A8 [Source:HGNC Symbol;Acc:HGNC:7692] | 358 | 0.442 | 0.0545 | Yes |
| 5 | UQCRC1 | ubiquinol-cytochrome c reductase core protein 1 [Source:HGNC Symbol;Acc:HGNC:12585] | 545 | 0.405 | 0.0632 | Yes |
| 6 | CYCS | "cytochrome c, somatic [Source:HGNC Symbol;Acc:HGNC:19986]" | 580 | 0.401 | 0.0757 | Yes |
| 7 | NDUFAB1 | NADH:ubiquinone oxidoreductase subunit AB1 [Source:HGNC Symbol;Acc:HGNC:7694] | 607 | 0.397 | 0.0882 | Yes |
| 8 | ATP5PD | ATP synthase peripheral stalk subunit d [Source:HGNC Symbol;Acc:HGNC:845] | 669 | 0.387 | 0.0996 | Yes |
| 9 | NDUFV3 | NADH:ubiquinone oxidoreductase subunit V3 [Source:HGNC Symbol;Acc:HGNC:7719] | 776 | 0.372 | 0.1093 | Yes |
| 10 | CREB3L4 | cAMP responsive element binding protein 3 like 4 [Source:HGNC Symbol;Acc:HGNC:18854] | 866 | 0.359 | 0.1189 | Yes |
| 11 | NDUFS2 | NADH:ubiquinone oxidoreductase core subunit S2 [Source:HGNC Symbol;Acc:HGNC:7708] | 973 | 0.348 | 0.1278 | Yes |
| 12 | TAF4 | TATA-box binding protein associated factor 4 [Source:HGNC Symbol;Acc:HGNC:11537] | 1039 | 0.340 | 0.1375 | Yes |
| 13 | CASP9 | caspase 9 [Source:HGNC Symbol;Acc:HGNC:1511] | 1063 | 0.338 | 0.1482 | Yes |
| 14 | NDUFB2 | NADH:ubiquinone oxidoreductase subunit B2 [Source:HGNC Symbol;Acc:HGNC:7697] | 1068 | 0.338 | 0.1593 | Yes |
| 15 | POLR2H | "RNA polymerase II, I and III subunit H [Source:HGNC Symbol;Acc:HGNC:9195]" | 1100 | 0.335 | 0.1697 | Yes |
| 16 | NDUFB6 | NADH:ubiquinone oxidoreductase subunit B6 [Source:HGNC Symbol;Acc:HGNC:7701] | 1121 | 0.333 | 0.1802 | Yes |
| 17 | ATP5F1A | ATP synthase F1 subunit alpha [Source:HGNC Symbol;Acc:HGNC:823] | 1183 | 0.326 | 0.1895 | Yes |
| 18 | CYC1 | cytochrome c1 [Source:HGNC Symbol;Acc:HGNC:2579] | 1273 | 0.318 | 0.1979 | Yes |
| 19 | UQCRC2 | ubiquinol-cytochrome c reductase core protein 2 [Source:HGNC Symbol;Acc:HGNC:12586] | 1346 | 0.312 | 0.2064 | Yes |
| 20 | ATP5PF | ATP synthase peripheral stalk subunit F6 [Source:HGNC Symbol;Acc:HGNC:847] | 1369 | 0.309 | 0.2162 | Yes |
| 21 | NDUFS1 | NADH:ubiquinone oxidoreductase core subunit S1 [Source:HGNC Symbol;Acc:HGNC:7707] | 1387 | 0.307 | 0.2260 | Yes |
| 22 | NDUFS5 | NADH:ubiquinone oxidoreductase subunit S5 [Source:HGNC Symbol;Acc:HGNC:7712] | 1409 | 0.306 | 0.2356 | Yes |
| 23 | SLC25A4 | solute carrier family 25 member 4 [Source:HGNC Symbol;Acc:HGNC:10990] | 1423 | 0.305 | 0.2454 | Yes |
| 24 | ATP5MC3 | ATP synthase membrane subunit c locus 3 [Source:HGNC Symbol;Acc:HGNC:843] | 1436 | 0.304 | 0.2552 | Yes |
| 25 | NDUFB5 | NADH:ubiquinone oxidoreductase subunit B5 [Source:HGNC Symbol;Acc:HGNC:7700] | 1567 | 0.294 | 0.2617 | Yes |
| 26 | HDAC1 | histone deacetylase 1 [Source:HGNC Symbol;Acc:HGNC:4852] | 1569 | 0.294 | 0.2714 | Yes |
| 27 | DNAH3 | dynein axonemal heavy chain 3 [Source:HGNC Symbol;Acc:HGNC:2949] | 1669 | 0.287 | 0.2785 | Yes |
| 28 | NDUFA2 | NADH:ubiquinone oxidoreductase subunit A2 [Source:HGNC Symbol;Acc:HGNC:7685] | 1800 | 0.276 | 0.2843 | Yes |
| 29 | AP2M1 | adaptor related protein complex 2 subunit mu 1 [Source:HGNC Symbol;Acc:HGNC:564] | 1804 | 0.276 | 0.2934 | Yes |
| 30 | POLR2L | "RNA polymerase II, I and III subunit L [Source:HGNC Symbol;Acc:HGNC:9199]" | 1839 | 0.274 | 0.3017 | Yes |
| 31 | NDUFS8 | NADH:ubiquinone oxidoreductase core subunit S8 [Source:HGNC Symbol;Acc:HGNC:7715] | 1900 | 0.270 | 0.3091 | Yes |
| 32 | BAX | "BCL2 associated X, apoptosis regulator [Source:HGNC Symbol;Acc:HGNC:959]" | 2048 | 0.261 | 0.3141 | Yes |
| 33 | NDUFB1 | NADH:ubiquinone oxidoreductase subunit B1 [Source:HGNC Symbol;Acc:HGNC:7695] | 2076 | 0.260 | 0.3220 | Yes |
| 34 | TP53 | tumor protein p53 [Source:HGNC Symbol;Acc:HGNC:11998] | 2121 | 0.257 | 0.3295 | Yes |
| 35 | UQCRQ | ubiquinol-cytochrome c reductase complex III subunit VII [Source:HGNC Symbol;Acc:HGNC:29594] | 2237 | 0.250 | 0.3349 | Yes |
| 36 | COX5A | cytochrome c oxidase subunit 5A [Source:HGNC Symbol;Acc:HGNC:2267] | 2292 | 0.248 | 0.3417 | Yes |
| 37 | NDUFB7 | NADH:ubiquinone oxidoreductase subunit B7 [Source:HGNC Symbol;Acc:HGNC:7702] | 2433 | 0.240 | 0.3462 | Yes |
| 38 | DCTN1 | dynactin subunit 1 [Source:HGNC Symbol;Acc:HGNC:2711] | 2533 | 0.235 | 0.3515 | Yes |
| 39 | NDUFS6 | NADH:ubiquinone oxidoreductase subunit S6 [Source:HGNC Symbol;Acc:HGNC:7713] | 2684 | 0.228 | 0.3552 | Yes |
| 40 | NDUFS7 | NADH:ubiquinone oxidoreductase core subunit S7 [Source:HGNC Symbol;Acc:HGNC:7714] | 2708 | 0.227 | 0.3622 | Yes |
| 41 | SDHB | succinate dehydrogenase complex iron sulfur subunit B [Source:HGNC Symbol;Acc:HGNC:10681] | 2784 | 0.223 | 0.3677 | Yes |
| 42 | DNAL4 | dynein axonemal light chain 4 [Source:HGNC Symbol;Acc:HGNC:2955] | 2808 | 0.222 | 0.3745 | Yes |
| 43 | DNAL1 | dynein axonemal light chain 1 [Source:HGNC Symbol;Acc:HGNC:23247] | 2851 | 0.220 | 0.3807 | Yes |
| 44 | PLCB1 | phospholipase C beta 1 [Source:HGNC Symbol;Acc:HGNC:15917] | 2881 | 0.219 | 0.3873 | Yes |
| 45 | ATP5PB | ATP synthase peripheral stalk-membrane subunit b [Source:HGNC Symbol;Acc:HGNC:840] | 3049 | 0.211 | 0.3900 | Yes |
| 46 | POLR2D | RNA polymerase II subunit D [Source:HGNC Symbol;Acc:HGNC:9191] | 3081 | 0.210 | 0.3962 | Yes |
| 47 | GRM5 | glutamate metabotropic receptor 5 [Source:HGNC Symbol;Acc:HGNC:4597] | 3125 | 0.208 | 0.4020 | Yes |
| 48 | VDAC1 | voltage dependent anion channel 1 [Source:HGNC Symbol;Acc:HGNC:12669] | 3273 | 0.202 | 0.4050 | Yes |
| 49 | UQCR10 | "ubiquinol-cytochrome c reductase, complex III subunit X [Source:HGNC Symbol;Acc:HGNC:30863]" | 3322 | 0.200 | 0.4104 | Yes |
| 50 | HDAC2 | histone deacetylase 2 [Source:HGNC Symbol;Acc:HGNC:4853] | 3509 | 0.193 | 0.4121 | Yes |
| 51 | NDUFA4 | NDUFA4 mitochondrial complex associated [Source:HGNC Symbol;Acc:HGNC:7687] | 3539 | 0.192 | 0.4178 | Yes |
| 52 | NDUFB9 | NADH:ubiquinone oxidoreductase subunit B9 [Source:HGNC Symbol;Acc:HGNC:7704] | 3655 | 0.188 | 0.4211 | Yes |
| 53 | AP2B1 | adaptor related protein complex 2 subunit beta 1 [Source:HGNC Symbol;Acc:HGNC:563] | 3669 | 0.187 | 0.4270 | Yes |
| 54 | NDUFB4 | NADH:ubiquinone oxidoreductase subunit B4 [Source:HGNC Symbol;Acc:HGNC:7699] | 3810 | 0.183 | 0.4295 | Yes |
| 55 | PLCB4 | phospholipase C beta 4 [Source:HGNC Symbol;Acc:HGNC:9059] | 3842 | 0.182 | 0.4347 | Yes |
| 56 | COX7A2 | cytochrome c oxidase subunit 7A2 [Source:HGNC Symbol;Acc:HGNC:2288] | 3863 | 0.181 | 0.4403 | Yes |
| 57 | COX7B | cytochrome c oxidase subunit 7B [Source:HGNC Symbol;Acc:HGNC:2291] | 3992 | 0.177 | 0.4429 | Yes |
| 58 | POLR2F | "RNA polymerase II, I and III subunit F [Source:HGNC Symbol;Acc:HGNC:9193]" | 4041 | 0.175 | 0.4475 | Yes |
| 59 | UQCRHL | ubiquinol-cytochrome c reductase hinge protein like [Source:HGNC Symbol;Acc:HGNC:51714] | 4135 | 0.173 | 0.4509 | Yes |
| 60 | COX6B1 | cytochrome c oxidase subunit 6B1 [Source:HGNC Symbol;Acc:HGNC:2280] | 4181 | 0.171 | 0.4554 | Yes |
| 61 | COX5B | cytochrome c oxidase subunit 5B [Source:HGNC Symbol;Acc:HGNC:2269] | 4402 | 0.164 | 0.4553 | Yes |
| 62 | POLR2E | "RNA polymerase II, I and III subunit E [Source:HGNC Symbol;Acc:HGNC:9192]" | 4463 | 0.162 | 0.4592 | Yes |
| 63 | ATP5F1C | ATP synthase F1 subunit gamma [Source:HGNC Symbol;Acc:HGNC:833] | 4521 | 0.161 | 0.4631 | Yes |
| 64 | NDUFA3 | NADH:ubiquinone oxidoreductase subunit A3 [Source:HGNC Symbol;Acc:HGNC:7686] | 4566 | 0.160 | 0.4673 | Yes |
| 65 | COX7B2 | cytochrome c oxidase subunit 7B2 [Source:HGNC Symbol;Acc:HGNC:24381] | 4647 | 0.157 | 0.4705 | Yes |
| 66 | VDAC3 | voltage dependent anion channel 3 [Source:HGNC Symbol;Acc:HGNC:12674] | 4648 | 0.157 | 0.4757 | Yes |
| 67 | UQCRH | ubiquinol-cytochrome c reductase hinge protein [Source:HGNC Symbol;Acc:HGNC:12590] | 4784 | 0.154 | 0.4774 | Yes |
| 68 | UQCR11 | "ubiquinol-cytochrome c reductase, complex III subunit XI [Source:HGNC Symbol;Acc:HGNC:30862]" | 4848 | 0.152 | 0.4808 | Yes |
| 69 | ATP5F1B | ATP synthase F1 subunit beta [Source:HGNC Symbol;Acc:HGNC:830] | 5137 | 0.145 | 0.4783 | No |
| 70 | NDUFA9 | NADH:ubiquinone oxidoreductase subunit A9 [Source:HGNC Symbol;Acc:HGNC:7693] | 5423 | 0.139 | 0.4756 | No |
| 71 | NDUFS4 | NADH:ubiquinone oxidoreductase subunit S4 [Source:HGNC Symbol;Acc:HGNC:7711] | 5588 | 0.135 | 0.4760 | No |
| 72 | NDUFV1 | NADH:ubiquinone oxidoreductase core subunit V1 [Source:HGNC Symbol;Acc:HGNC:7716] | 6169 | 0.124 | 0.4653 | No |
| 73 | COX7C | cytochrome c oxidase subunit 7C [Source:HGNC Symbol;Acc:HGNC:2292] | 6346 | 0.121 | 0.4648 | No |
| 74 | ATP5F1E | ATP synthase F1 subunit epsilon [Source:HGNC Symbol;Acc:HGNC:838] | 6500 | 0.118 | 0.4648 | No |
| 75 | SLC25A31 | solute carrier family 25 member 31 [Source:HGNC Symbol;Acc:HGNC:25319] | 6795 | 0.113 | 0.4610 | No |
| 76 | AP2A1 | adaptor related protein complex 2 subunit alpha 1 [Source:HGNC Symbol;Acc:HGNC:561] | 6823 | 0.112 | 0.4641 | No |
| 77 | POLR2K | "RNA polymerase II, I and III subunit K [Source:HGNC Symbol;Acc:HGNC:9198]" | 6857 | 0.112 | 0.4670 | No |
| 78 | CLTA | clathrin light chain A [Source:HGNC Symbol;Acc:HGNC:2090] | 6963 | 0.110 | 0.4679 | No |
| 79 | ATP5MC1P5 | ATP synthase membrane subunit c locus 1 pseudogene 5 [Source:HGNC Symbol;Acc:HGNC:39508] | 7332 | 0.104 | 0.4620 | No |
| 80 | ATP5MC2 | ATP synthase membrane subunit c locus 2 [Source:HGNC Symbol;Acc:HGNC:842] | 7382 | 0.103 | 0.4642 | No |
| 81 | BDNF | brain derived neurotrophic factor [Source:HGNC Symbol;Acc:HGNC:1033] | 8184 | 0.092 | 0.4468 | No |
| 82 | COX6A1 | cytochrome c oxidase subunit 6A1 [Source:HGNC Symbol;Acc:HGNC:2277] | 8445 | 0.089 | 0.4431 | No |
| 83 | SLC25A5 | solute carrier family 25 member 5 [Source:HGNC Symbol;Acc:HGNC:10991] | 8607 | 0.087 | 0.4419 | No |
| 84 | NDUFB10 | NADH:ubiquinone oxidoreductase subunit B10 [Source:HGNC Symbol;Acc:HGNC:7696] | 8944 | 0.083 | 0.4361 | No |
| 85 | CREB3L1 | cAMP responsive element binding protein 3 like 1 [Source:HGNC Symbol;Acc:HGNC:18856] | 9079 | 0.081 | 0.4354 | No |
| 86 | MT-CO1 | mitochondrially encoded cytochrome c oxidase I [Source:HGNC Symbol;Acc:HGNC:7419] | 9406 | 0.077 | 0.4296 | No |
| 87 | POLR2G | RNA polymerase II subunit G [Source:HGNC Symbol;Acc:HGNC:9194] | 9521 | 0.076 | 0.4292 | No |
| 88 | GRIN2B | glutamate ionotropic receptor NMDA type subunit 2B [Source:HGNC Symbol;Acc:HGNC:4586] | 9758 | 0.073 | 0.4257 | No |
| 89 | VDAC2P5 | VDAC2 pseudogene 5 [Source:HGNC Symbol;Acc:HGNC:54753] | 10072 | 0.070 | 0.4200 | No |
| 90 | POLR2I | RNA polymerase II subunit I [Source:HGNC Symbol;Acc:HGNC:9196] | 10437 | 0.066 | 0.4129 | No |
| 91 | COX8C | cytochrome c oxidase subunit 8C [Source:HGNC Symbol;Acc:HGNC:24382] | 10480 | 0.066 | 0.4140 | No |
| 92 | TFAM | "transcription factor A, mitochondrial [Source:HGNC Symbol;Acc:HGNC:11741]" | 10567 | 0.065 | 0.4140 | No |
| 93 | COX6C | cytochrome c oxidase subunit 6C [Source:HGNC Symbol;Acc:HGNC:2285] | 11062 | 0.060 | 0.4034 | No |
| 94 | ATP5PO | ATP synthase peripheral stalk subunit OSCP [Source:HGNC Symbol;Acc:HGNC:850] | 11202 | 0.059 | 0.4018 | No |
| 95 | AP2S1 | adaptor related protein complex 2 subunit sigma 1 [Source:HGNC Symbol;Acc:HGNC:565] | 11295 | 0.058 | 0.4014 | No |
| 96 | COX6CP3 | cytochrome c oxidase subunit 6C pseudogene 3 [Source:HGNC Symbol;Acc:HGNC:31721] | 11606 | 0.055 | 0.3953 | No |
| 97 | DNAH2 | dynein axonemal heavy chain 2 [Source:HGNC Symbol;Acc:HGNC:2948] | 12012 | 0.052 | 0.3867 | No |
| 98 | POLR2J3 | RNA polymerase II subunit J3 [Source:HGNC Symbol;Acc:HGNC:33853] | 12042 | 0.052 | 0.3877 | No |
| 99 | DNAI2 | dynein axonemal intermediate chain 2 [Source:HGNC Symbol;Acc:HGNC:18744] | 12317 | 0.050 | 0.3824 | No |
| 100 | SOD1 | superoxide dismutase 1 [Source:HGNC Symbol;Acc:HGNC:11179] | 12392 | 0.049 | 0.3821 | No |
| 101 | NDUFA5 | NADH:ubiquinone oxidoreductase subunit A5 [Source:HGNC Symbol;Acc:HGNC:7688] | 12525 | 0.048 | 0.3803 | No |
| 102 | TBPL2 | TATA-box binding protein like 2 [Source:HGNC Symbol;Acc:HGNC:19841] | 13020 | 0.045 | 0.3692 | No |
| 103 | ATP5F1D | ATP synthase F1 subunit delta [Source:HGNC Symbol;Acc:HGNC:837] | 13360 | 0.042 | 0.3620 | No |
| 104 | NDUFB8 | NADH:ubiquinone oxidoreductase subunit B8 [Source:HGNC Symbol;Acc:HGNC:7703] | 13566 | 0.041 | 0.3581 | No |
| 105 | COX8A | cytochrome c oxidase subunit 8A [Source:HGNC Symbol;Acc:HGNC:2294] | 13791 | 0.039 | 0.3537 | No |
| 106 | SDHD | succinate dehydrogenase complex subunit D [Source:HGNC Symbol;Acc:HGNC:10683] | 14412 | 0.036 | 0.3390 | No |
| 107 | GRIN1 | glutamate ionotropic receptor NMDA type subunit 1 [Source:HGNC Symbol;Acc:HGNC:4584] | 14624 | 0.034 | 0.3348 | No |
| 108 | AP2A2 | adaptor related protein complex 2 subunit alpha 2 [Source:HGNC Symbol;Acc:HGNC:562] | 14677 | 0.034 | 0.3346 | No |
| 109 | POLR2J | RNA polymerase II subunit J [Source:HGNC Symbol;Acc:HGNC:9197] | 14754 | 0.034 | 0.3337 | No |
| 110 | NDUFC1 | NADH:ubiquinone oxidoreductase subunit C1 [Source:HGNC Symbol;Acc:HGNC:7705] | 15304 | 0.030 | 0.3207 | No |
| 111 | UQCR10P1 | UQCR10 pseudogene 1 [Source:HGNC Symbol;Acc:HGNC:54960] | 15591 | 0.028 | 0.3144 | No |
| 112 | POLR2J2 | RNA polymerase II subunit J2 [Source:HGNC Symbol;Acc:HGNC:23208] | 15603 | 0.028 | 0.3150 | No |
| 113 | HAP1 | huntingtin associated protein 1 [Source:HGNC Symbol;Acc:HGNC:4812] | 15619 | 0.028 | 0.3156 | No |
| 114 | NDUFC2 | NADH:ubiquinone oxidoreductase subunit C2 [Source:HGNC Symbol;Acc:HGNC:7706] | 16310 | 0.024 | 0.2987 | No |
| 115 | MT-CO2 | mitochondrially encoded cytochrome c oxidase II [Source:HGNC Symbol;Acc:HGNC:7421] | 16417 | 0.023 | 0.2968 | No |
| 116 | COX6A2 | cytochrome c oxidase subunit 6A2 [Source:HGNC Symbol;Acc:HGNC:2279] | 16431 | 0.023 | 0.2972 | No |
| 117 | COX6B2 | cytochrome c oxidase subunit 6B2 [Source:HGNC Symbol;Acc:HGNC:24380] | 16821 | 0.021 | 0.2880 | No |
| 118 | UQCRB | ubiquinol-cytochrome c reductase binding protein [Source:HGNC Symbol;Acc:HGNC:12582] | 16826 | 0.021 | 0.2886 | No |
| 119 | NDUFA6 | NADH:ubiquinone oxidoreductase subunit A6 [Source:HGNC Symbol;Acc:HGNC:7690] | 19225 | 0.008 | 0.2276 | No |
| 120 | UCP1 | uncoupling protein 1 [Source:HGNC Symbol;Acc:HGNC:12517] | 20462 | 0.001 | 0.1961 | No |
| 121 | UQCRFS1 | "ubiquinol-cytochrome c reductase, Rieske iron-sulfur polypeptide 1 [Source:HGNC Symbol;Acc:HGNC:12587]" | 22359 | -0.010 | 0.1479 | No |
| 122 | GPX1 | glutathione peroxidase 1 [Source:HGNC Symbol;Acc:HGNC:4553] | 22964 | -0.014 | 0.1330 | No |
| 123 | NDUFA1 | NADH:ubiquinone oxidoreductase subunit A1 [Source:HGNC Symbol;Acc:HGNC:7683] | 23269 | -0.015 | 0.1257 | No |
| 124 | MT-ATP8 | mitochondrially encoded ATP synthase membrane subunit 8 [Source:HGNC Symbol;Acc:HGNC:7415] | 23281 | -0.015 | 0.1259 | No |
| 125 | PPARGC1A | PPARG coactivator 1 alpha [Source:HGNC Symbol;Acc:HGNC:9237] | 23490 | -0.017 | 0.1212 | No |
| 126 | NDUFS3 | NADH:ubiquinone oxidoreductase core subunit S3 [Source:HGNC Symbol;Acc:HGNC:7710] | 23954 | -0.020 | 0.1100 | No |
| 127 | POLR2C | RNA polymerase II subunit C [Source:HGNC Symbol;Acc:HGNC:9189] | 24322 | -0.022 | 0.1014 | No |
| 128 | SDHA | succinate dehydrogenase complex flavoprotein subunit A [Source:HGNC Symbol;Acc:HGNC:10680] | 24822 | -0.025 | 0.0895 | No |
| 129 | DNALI1 | dynein axonemal light intermediate chain 1 [Source:HGNC Symbol;Acc:HGNC:14353] | 25482 | -0.030 | 0.0736 | No |
| 130 | CLTCL1 | clathrin heavy chain like 1 [Source:HGNC Symbol;Acc:HGNC:2093] | 25545 | -0.030 | 0.0730 | No |
| 131 | PPID | peptidylprolyl isomerase D [Source:HGNC Symbol;Acc:HGNC:9257] | 25907 | -0.033 | 0.0649 | No |
| 132 | ITPR1 | "inositol 1,4,5-trisphosphate receptor type 1 [Source:HGNC Symbol;Acc:HGNC:6180]" | 26491 | -0.037 | 0.0513 | No |
| 133 | NDUFA7 | NADH:ubiquinone oxidoreductase subunit A7 [Source:HGNC Symbol;Acc:HGNC:7691] | 27225 | -0.043 | 0.0340 | No |
| 134 | SLC25A6 | solute carrier family 25 member 6 [Source:HGNC Symbol;Acc:HGNC:10992] | 27783 | -0.048 | 0.0213 | No |
| 135 | NDUFA4L2 | NDUFA4 mitochondrial complex associated like 2 [Source:HGNC Symbol;Acc:HGNC:29836] | 27785 | -0.048 | 0.0229 | No |
| 136 | MT-ATP6 | mitochondrially encoded ATP synthase membrane subunit 6 [Source:HGNC Symbol;Acc:HGNC:7414] | 28861 | -0.059 | -0.0026 | No |
| 137 | CLTB | clathrin light chain B [Source:HGNC Symbol;Acc:HGNC:2091] | 29138 | -0.062 | -0.0076 | No |
| 138 | DNAI1 | dynein axonemal intermediate chain 1 [Source:HGNC Symbol;Acc:HGNC:2954] | 29771 | -0.069 | -0.0214 | No |
| 139 | CREB3L3 | cAMP responsive element binding protein 3 like 3 [Source:HGNC Symbol;Acc:HGNC:18855] | 29858 | -0.070 | -0.0213 | No |
| 140 | NDUFA10 | NADH:ubiquinone oxidoreductase subunit A10 [Source:HGNC Symbol;Acc:HGNC:7684] | 30079 | -0.073 | -0.0244 | No |
| 141 | COX7A2L | cytochrome c oxidase subunit 7A2 like [Source:HGNC Symbol;Acc:HGNC:2289] | 30323 | -0.076 | -0.0281 | No |
| 142 | POLR2A | RNA polymerase II subunit A [Source:HGNC Symbol;Acc:HGNC:9187] | 30331 | -0.076 | -0.0258 | No |
| 143 | CREB3 | cAMP responsive element binding protein 3 [Source:HGNC Symbol;Acc:HGNC:2347] | 30349 | -0.076 | -0.0237 | No |
| 144 | MT-CYB | mitochondrially encoded cytochrome b [Source:HGNC Symbol;Acc:HGNC:7427] | 30698 | -0.081 | -0.0299 | No |
| 145 | CLTC | clathrin heavy chain [Source:HGNC Symbol;Acc:HGNC:2092] | 30796 | -0.082 | -0.0296 | No |
| 146 | VDAC2 | voltage dependent anion channel 2 [Source:HGNC Symbol;Acc:HGNC:12672] | 30866 | -0.083 | -0.0286 | No |
| 147 | MT-CO3 | mitochondrially encoded cytochrome c oxidase III [Source:HGNC Symbol;Acc:HGNC:7422] | 30889 | -0.083 | -0.0264 | No |
| 148 | DNAH1 | dynein axonemal heavy chain 1 [Source:HGNC Symbol;Acc:HGNC:2940] | 30975 | -0.084 | -0.0258 | No |
| 149 | PLCB3 | phospholipase C beta 3 [Source:HGNC Symbol;Acc:HGNC:9056] | 31486 | -0.091 | -0.0357 | No |
| 150 | DCTN4 | dynactin subunit 4 [Source:HGNC Symbol;Acc:HGNC:15518] | 32356 | -0.106 | -0.0544 | No |
| 151 | HIP1 | huntingtin interacting protein 1 [Source:HGNC Symbol;Acc:HGNC:4913] | 32739 | -0.114 | -0.0604 | No |
| 152 | SP1 | Sp1 transcription factor [Source:HGNC Symbol;Acc:HGNC:11205] | 32829 | -0.116 | -0.0588 | No |
| 153 | COX7A1 | cytochrome c oxidase subunit 7A1 [Source:HGNC Symbol;Acc:HGNC:2287] | 32963 | -0.118 | -0.0583 | No |
| 154 | RCOR1 | REST corepressor 1 [Source:HGNC Symbol;Acc:HGNC:17441] | 33201 | -0.123 | -0.0602 | No |
| 155 | IFT57 | intraflagellar transport 57 [Source:HGNC Symbol;Acc:HGNC:17367] | 33336 | -0.125 | -0.0595 | No |
| 156 | TAF4B | TATA-box binding protein associated factor 4b [Source:HGNC Symbol;Acc:HGNC:11538] | 33513 | -0.129 | -0.0597 | No |
| 157 | COX4I2 | cytochrome c oxidase subunit 4I2 [Source:HGNC Symbol;Acc:HGNC:16232] | 33693 | -0.134 | -0.0598 | No |
| 158 | TBP | TATA-box binding protein [Source:HGNC Symbol;Acc:HGNC:11588] | 34043 | -0.142 | -0.0640 | No |
| 159 | GNAQ | G protein subunit alpha q [Source:HGNC Symbol;Acc:HGNC:4390] | 34211 | -0.147 | -0.0634 | No |
| 160 | PPARG | peroxisome proliferator activated receptor gamma [Source:HGNC Symbol;Acc:HGNC:9236] | 34474 | -0.155 | -0.0649 | No |
| 161 | DLG4 | discs large MAGUK scaffold protein 4 [Source:HGNC Symbol;Acc:HGNC:2903] | 34579 | -0.158 | -0.0623 | No |
| 162 | CASP3 | caspase 3 [Source:HGNC Symbol;Acc:HGNC:1504] | 34816 | -0.165 | -0.0628 | No |
| 163 | TBPL1 | TATA-box binding protein like 1 [Source:HGNC Symbol;Acc:HGNC:11589] | 35345 | -0.183 | -0.0702 | No |
| 164 | COX4I1 | cytochrome c oxidase subunit 4I1 [Source:HGNC Symbol;Acc:HGNC:2265] | 35680 | -0.195 | -0.0723 | No |
| 165 | REST | RE1 silencing transcription factor [Source:HGNC Symbol;Acc:HGNC:9966] | 35689 | -0.195 | -0.0660 | No |
| 166 | CREB3L2 | cAMP responsive element binding protein 3 like 2 [Source:HGNC Symbol;Acc:HGNC:23720] | 35730 | -0.197 | -0.0604 | No |
| 167 | TGM2 | transglutaminase 2 [Source:HGNC Symbol;Acc:HGNC:11778] | 36169 | -0.214 | -0.0645 | No |
| 168 | APAF1 | apoptotic peptidase activating factor 1 [Source:HGNC Symbol;Acc:HGNC:576] | 36307 | -0.220 | -0.0607 | No |
| 169 | NDUFV2 | NADH:ubiquinone oxidoreductase core subunit V2 [Source:HGNC Symbol;Acc:HGNC:7717] | 36361 | -0.223 | -0.0546 | No |
| 170 | BBC3 | BCL2 binding component 3 [Source:HGNC Symbol;Acc:HGNC:17868] | 36564 | -0.234 | -0.0519 | No |
| 171 | CREB5 | cAMP responsive element binding protein 5 [Source:HGNC Symbol;Acc:HGNC:16844] | 36720 | -0.243 | -0.0478 | No |
| 172 | CASP8 | caspase 8 [Source:HGNC Symbol;Acc:HGNC:1509] | 36734 | -0.243 | -0.0401 | No |
| 173 | PLCB2 | phospholipase C beta 2 [Source:HGNC Symbol;Acc:HGNC:9055] | 36982 | -0.259 | -0.0378 | No |
| 174 | DCTN2 | dynactin subunit 2 [Source:HGNC Symbol;Acc:HGNC:2712] | 37064 | -0.263 | -0.0311 | No |
| 175 | POLR2B | RNA polymerase II subunit B [Source:HGNC Symbol;Acc:HGNC:9188] | 37220 | -0.275 | -0.0259 | No |
| 176 | SOD2 | superoxide dismutase 2 [Source:HGNC Symbol;Acc:HGNC:11180] | 37254 | -0.276 | -0.0175 | No |
| 177 | HTT | huntingtin [Source:HGNC Symbol;Acc:HGNC:4851] | 37288 | -0.279 | -0.0090 | No |
| 178 | EP300 | E1A binding protein p300 [Source:HGNC Symbol;Acc:HGNC:3373] | 37644 | -0.308 | -0.0078 | No |
| 179 | CREBBP | CREB binding protein [Source:HGNC Symbol;Acc:HGNC:2348] | 37926 | -0.340 | -0.0037 | No |
| 180 | NRF1 | nuclear respiratory factor 1 [Source:HGNC Symbol;Acc:HGNC:7996] | 38183 | -0.374 | 0.0022 | No |
| 181 | SIN3A | SIN3 transcription regulator family member A [Source:HGNC Symbol;Acc:HGNC:19353] | 38257 | -0.389 | 0.0133 | No |
| 182 | CREB1 | cAMP responsive element binding protein 1 [Source:HGNC Symbol;Acc:HGNC:2345] | 38395 | -0.418 | 0.0237 | No |
Table: GSEA details [plain text format]

  

Fig 2: KEGG\_HUNTINGTONS\_DISEASE      
 Blue-Pink O' Gram in the Space of the Analyzed GeneSet

  

Fig 3: KEGG\_HUNTINGTONS\_DISEASE: Random ES distribution      
 Gene set null distribution of ES for **KEGG\_HUNTINGTONS\_DISEASE**
