# Supplementary material for: Simultaneous Presentation of Multiple Myeloma and Lung Cancer: Case Report and Gene Bioinformatics Analysis
Source: Front Oncol. 2022 Jun 13;12:859735. doi: 10.3389/fonc.2022.859735 (PMC9235397; doi:10.3389/fonc.2022.859735)
Supplement: Supplementary file 1 [file DataSheet_1.zip › The bioinformatic analysis of MM and lung cancer supplementary materials/Enrichment analysis/MECR/GSEA_4.1.0/MM TCGA/KEGG.Gsea.1639040591792/KEGG_LEISHMANIA_INFECTION.html]

Details for gene set KEGG\_LEISHMANIA\_INFECTION[GSEA]

|  || Dataset | ExpData\_collapsed\_to\_symbols.ENSG00000116353\_profile\_in\_ExpData.cls #ENSG00000116353 |
| Phenotype | ENSG00000116353\_profile\_in\_ExpData.cls#ENSG00000116353 |
| Upregulated in class | ENSG00000116353\_neg |
| GeneSet | KEGG\_LEISHMANIA\_INFECTION |
| Enrichment Score (ES) | -0.61243814 |
| Normalized Enrichment Score (NES) | -2.3206797 |
| Nominal p-value | 0.0 |
| FDR q-value | 0.0 |
| FWER p-Value | 0.0 |
Table: GSEA Results Summary

  

Fig 1: Enrichment plot: KEGG\_LEISHMANIA\_INFECTION      
 Profile of the Running ES Score & Positions of GeneSet Members on the Rank Ordered List

  

| SYMBOL | TITLE | RANK IN GENE LIST | RANK METRIC SCORE | RUNNING ES | CORE ENRICHMENT || 1 | FOS | "Fos proto-oncogene, AP-1 transcription factor subunit [Source:HGNC Symbol;Acc:HGNC:3796]" | 3070 | 0.210 | -0.0589 | No |
| 2 | TAB1 | TGF-beta activated kinase 1 (MAP3K7) binding protein 1 [Source:HGNC Symbol;Acc:HGNC:18157] | 3360 | 0.199 | -0.0480 | No |
| 3 | PTPN6 | protein tyrosine phosphatase non-receptor type 6 [Source:HGNC Symbol;Acc:HGNC:9658] | 4119 | 0.173 | -0.0514 | No |
| 4 | IL12A | interleukin 12A [Source:HGNC Symbol;Acc:HGNC:5969] | 4178 | 0.171 | -0.0371 | No |
| 5 | JUN | "Jun proto-oncogene, AP-1 transcription factor subunit [Source:HGNC Symbol;Acc:HGNC:6204]" | 5088 | 0.146 | -0.0468 | No |
| 6 | MYD88 | MYD88 innate immune signal transduction adaptor [Source:HGNC Symbol;Acc:HGNC:7562] | 8652 | 0.087 | -0.1296 | No |
| 7 | IRAK1 | interleukin 1 receptor associated kinase 1 [Source:HGNC Symbol;Acc:HGNC:6112] | 9888 | 0.072 | -0.1544 | No |
| 8 | IL12B | interleukin 12B [Source:HGNC Symbol;Acc:HGNC:5970] | 9896 | 0.072 | -0.1480 | No |
| 9 | ITGA4 | integrin subunit alpha 4 [Source:HGNC Symbol;Acc:HGNC:6140] | 10741 | 0.063 | -0.1637 | No |
| 10 | STAT1 | signal transducer and activator of transcription 1 [Source:HGNC Symbol;Acc:HGNC:11362] | 10873 | 0.062 | -0.1613 | No |
| 11 | PRKCB | protein kinase C beta [Source:HGNC Symbol;Acc:HGNC:9395] | 11760 | 0.054 | -0.1789 | No |
| 12 | MAP3K7 | mitogen-activated protein kinase kinase kinase 7 [Source:HGNC Symbol;Acc:HGNC:6859] | 12527 | 0.048 | -0.1939 | No |
| 13 | TGFB2 | transforming growth factor beta 2 [Source:HGNC Symbol;Acc:HGNC:11768] | 18348 | 0.013 | -0.3410 | No |
| 14 | NCF4 | neutrophil cytosolic factor 4 [Source:HGNC Symbol;Acc:HGNC:7662] | 20742 | -0.001 | -0.4020 | No |
| 15 | ITGB1 | integrin subunit beta 1 [Source:HGNC Symbol;Acc:HGNC:6153] | 22081 | -0.008 | -0.4353 | No |
| 16 | MARCKSL1 | MARCKS like 1 [Source:HGNC Symbol;Acc:HGNC:7142] | 22171 | -0.009 | -0.4367 | No |
| 17 | CYBA | cytochrome b-245 alpha chain [Source:HGNC Symbol;Acc:HGNC:2577] | 22961 | -0.014 | -0.4556 | No |
| 18 | MAPK1 | mitogen-activated protein kinase 1 [Source:HGNC Symbol;Acc:HGNC:6871] | 23167 | -0.015 | -0.4595 | No |
| 19 | PTGS2 | prostaglandin-endoperoxide synthase 2 [Source:HGNC Symbol;Acc:HGNC:9605] | 25111 | -0.027 | -0.5065 | No |
| 20 | MAPK12 | mitogen-activated protein kinase 12 [Source:HGNC Symbol;Acc:HGNC:6874] | 25314 | -0.028 | -0.5090 | No |
| 21 | IFNG | interferon gamma [Source:HGNC Symbol;Acc:HGNC:5438] | 25529 | -0.030 | -0.5117 | No |
| 22 | HLA-DQA2 | "major histocompatibility complex, class II, DQ alpha 2 [Source:HGNC Symbol;Acc:HGNC:4943]" | 25697 | -0.031 | -0.5130 | No |
| 23 | TLR4 | toll like receptor 4 [Source:HGNC Symbol;Acc:HGNC:11850] | 27199 | -0.043 | -0.5473 | No |
| 24 | NOS2 | nitric oxide synthase 2 [Source:HGNC Symbol;Acc:HGNC:7873] | 27899 | -0.049 | -0.5606 | No |
| 25 | IL4 | interleukin 4 [Source:HGNC Symbol;Acc:HGNC:6014] | 28173 | -0.052 | -0.5628 | No |
| 26 | HLA-DOB | "major histocompatibility complex, class II, DO beta [Source:HGNC Symbol;Acc:HGNC:4937]" | 28713 | -0.057 | -0.5713 | No |
| 27 | FCGR2C | Fc fragment of IgG receptor IIc (gene/pseudogene) [Source:HGNC Symbol;Acc:HGNC:15626] | 28906 | -0.059 | -0.5707 | No |
| 28 | FCGR3A | Fc fragment of IgG receptor IIIa [Source:HGNC Symbol;Acc:HGNC:3619] | 29324 | -0.064 | -0.5754 | No |
| 29 | MAPK13 | mitogen-activated protein kinase 13 [Source:HGNC Symbol;Acc:HGNC:6875] | 29402 | -0.065 | -0.5714 | No |
| 30 | IRAK4 | interleukin 1 receptor associated kinase 4 [Source:HGNC Symbol;Acc:HGNC:17967] | 30700 | -0.081 | -0.5970 | No |
| 31 | TGFB3 | transforming growth factor beta 3 [Source:HGNC Symbol;Acc:HGNC:11769] | 30832 | -0.083 | -0.5928 | No |
| 32 | HLA-DOA | "major histocompatibility complex, class II, DO alpha [Source:HGNC Symbol;Acc:HGNC:4936]" | 31604 | -0.093 | -0.6039 | Yes |
| 33 | CR1 | complement C3b/C4b receptor 1 (Knops blood group) [Source:HGNC Symbol;Acc:HGNC:2334] | 31715 | -0.095 | -0.5979 | Yes |
| 34 | FCGR3B | Fc fragment of IgG receptor IIIb [Source:HGNC Symbol;Acc:HGNC:3620] | 32200 | -0.103 | -0.6008 | Yes |
| 35 | FCGR1A | Fc fragment of IgG receptor Ia [Source:HGNC Symbol;Acc:HGNC:3613] | 32623 | -0.111 | -0.6013 | Yes |
| 36 | IL1A | interleukin 1 alpha [Source:HGNC Symbol;Acc:HGNC:5991] | 32718 | -0.113 | -0.5933 | Yes |
| 37 | HLA-DQA1 | "major histocompatibility complex, class II, DQ alpha 1 [Source:HGNC Symbol;Acc:HGNC:4942]" | 32959 | -0.118 | -0.5885 | Yes |
| 38 | ELK1 | ETS transcription factor ELK1 [Source:HGNC Symbol;Acc:HGNC:3321] | 33028 | -0.120 | -0.5793 | Yes |
| 39 | HLA-DRB5 | "major histocompatibility complex, class II, DR beta 5 [Source:HGNC Symbol;Acc:HGNC:4953]" | 33950 | -0.140 | -0.5898 | Yes |
| 40 | JAK2 | Janus kinase 2 [Source:HGNC Symbol;Acc:HGNC:6192] | 33960 | -0.141 | -0.5771 | Yes |
| 41 | IL10 | interleukin 10 [Source:HGNC Symbol;Acc:HGNC:5962] | 34351 | -0.151 | -0.5732 | Yes |
| 42 | HLA-DPA1 | "major histocompatibility complex, class II, DP alpha 1 [Source:HGNC Symbol;Acc:HGNC:4938]" | 34382 | -0.152 | -0.5599 | Yes |
| 43 | HLA-DPB1 | "major histocompatibility complex, class II, DP beta 1 [Source:HGNC Symbol;Acc:HGNC:4940]" | 34547 | -0.157 | -0.5497 | Yes |
| 44 | HLA-DRB1 | "major histocompatibility complex, class II, DR beta 1 [Source:HGNC Symbol;Acc:HGNC:4948]" | 34560 | -0.157 | -0.5355 | Yes |
| 45 | HLA-DMB | "major histocompatibility complex, class II, DM beta [Source:HGNC Symbol;Acc:HGNC:4935]" | 34608 | -0.159 | -0.5221 | Yes |
| 46 | HLA-DMA | "major histocompatibility complex, class II, DM alpha [Source:HGNC Symbol;Acc:HGNC:4934]" | 34644 | -0.160 | -0.5083 | Yes |
| 47 | HLA-DQB1 | "major histocompatibility complex, class II, DQ beta 1 [Source:HGNC Symbol;Acc:HGNC:4944]" | 34874 | -0.167 | -0.4988 | Yes |
| 48 | FCGR2A | Fc fragment of IgG receptor IIa [Source:HGNC Symbol;Acc:HGNC:3616] | 34953 | -0.169 | -0.4852 | Yes |
| 49 | MAPK11 | mitogen-activated protein kinase 11 [Source:HGNC Symbol;Acc:HGNC:6873] | 35006 | -0.171 | -0.4708 | Yes |
| 50 | C3 | complement C3 [Source:HGNC Symbol;Acc:HGNC:1318] | 35497 | -0.188 | -0.4660 | Yes |
| 51 | MAPK3 | mitogen-activated protein kinase 3 [Source:HGNC Symbol;Acc:HGNC:6877] | 35579 | -0.191 | -0.4505 | Yes |
| 52 | HLA-DRA | "major histocompatibility complex, class II, DR alpha [Source:HGNC Symbol;Acc:HGNC:4947]" | 35586 | -0.191 | -0.4331 | Yes |
| 53 | IL1B | interleukin 1 beta [Source:HGNC Symbol;Acc:HGNC:5992] | 35858 | -0.202 | -0.4214 | Yes |
| 54 | TGFB1 | transforming growth factor beta 1 [Source:HGNC Symbol;Acc:HGNC:11766] | 36247 | -0.217 | -0.4113 | Yes |
| 55 | NCF1 | neutrophil cytosolic factor 1 [Source:HGNC Symbol;Acc:HGNC:7660] | 36267 | -0.218 | -0.3917 | Yes |
| 56 | ITGB2 | integrin subunit beta 2 [Source:HGNC Symbol;Acc:HGNC:6155] | 36308 | -0.220 | -0.3725 | Yes |
| 57 | TNF | tumor necrosis factor [Source:HGNC Symbol;Acc:HGNC:11892] | 36419 | -0.226 | -0.3545 | Yes |
| 58 | ITGAM | integrin subunit alpha M [Source:HGNC Symbol;Acc:HGNC:6149] | 36727 | -0.243 | -0.3400 | Yes |
| 59 | NCF2 | neutrophil cytosolic factor 2 [Source:HGNC Symbol;Acc:HGNC:7661] | 36895 | -0.253 | -0.3210 | Yes |
| 60 | NFKBIA | NFKB inhibitor alpha [Source:HGNC Symbol;Acc:HGNC:7797] | 37222 | -0.275 | -0.3040 | Yes |
| 61 | TLR2 | toll like receptor 2 [Source:HGNC Symbol;Acc:HGNC:11848] | 37324 | -0.282 | -0.2806 | Yes |
| 62 | TAB2 | TGF-beta activated kinase 1 (MAP3K7) binding protein 2 [Source:HGNC Symbol;Acc:HGNC:17075] | 37429 | -0.290 | -0.2566 | Yes |
| 63 | IFNGR1 | interferon gamma receptor 1 [Source:HGNC Symbol;Acc:HGNC:5439] | 37779 | -0.322 | -0.2359 | Yes |
| 64 | NFKBIB | NFKB inhibitor beta [Source:HGNC Symbol;Acc:HGNC:7798] | 37987 | -0.348 | -0.2091 | Yes |
| 65 | TRAF6 | TNF receptor associated factor 6 [Source:HGNC Symbol;Acc:HGNC:12036] | 38202 | -0.379 | -0.1798 | Yes |
| 66 | MAPK14 | mitogen-activated protein kinase 14 [Source:HGNC Symbol;Acc:HGNC:6876] | 38287 | -0.396 | -0.1454 | Yes |
| 67 | NFKB1 | nuclear factor kappa B subunit 1 [Source:HGNC Symbol;Acc:HGNC:7794] | 38470 | -0.443 | -0.1093 | Yes |
| 68 | RELA | "RELA proto-oncogene, NF-kB subunit [Source:HGNC Symbol;Acc:HGNC:9955]" | 38475 | -0.445 | -0.0685 | Yes |
| 69 | JAK1 | Janus kinase 1 [Source:HGNC Symbol;Acc:HGNC:6190] | 38488 | -0.448 | -0.0276 | Yes |
| 70 | IFNGR2 | interferon gamma receptor 2 [Source:HGNC Symbol;Acc:HGNC:5440] | 38582 | -0.531 | 0.0189 | Yes |
Table: GSEA details [plain text format]

  

Fig 2: KEGG\_LEISHMANIA\_INFECTION      
 Blue-Pink O' Gram in the Space of the Analyzed GeneSet

  

Fig 3: KEGG\_LEISHMANIA\_INFECTION: Random ES distribution      
 Gene set null distribution of ES for **KEGG\_LEISHMANIA\_INFECTION**
