# Supplementary material for: Simultaneous Presentation of Multiple Myeloma and Lung Cancer: Case Report and Gene Bioinformatics Analysis
Source: Front Oncol. 2022 Jun 13;12:859735. doi: 10.3389/fonc.2022.859735 (PMC9235397; doi:10.3389/fonc.2022.859735)
Supplement: Supplementary file 1 [file DataSheet_1.zip › The bioinformatic analysis of MM and lung cancer supplementary materials/Enrichment analysis/MECR/GSEA_4.1.0/MM TCGA/KEGG.Gsea.1639040591792/KEGG_LEUKOCYTE_TRANSENDOTHELIAL_MIGRATION.html]

Details for gene set KEGG\_LEUKOCYTE\_TRANSENDOTHELIAL\_MIGRATION[GSEA]

|  || Dataset | ExpData\_collapsed\_to\_symbols.ENSG00000116353\_profile\_in\_ExpData.cls #ENSG00000116353 |
| Phenotype | ENSG00000116353\_profile\_in\_ExpData.cls#ENSG00000116353 |
| Upregulated in class | ENSG00000116353\_neg |
| GeneSet | KEGG\_LEUKOCYTE\_TRANSENDOTHELIAL\_MIGRATION |
| Enrichment Score (ES) | -0.59608823 |
| Normalized Enrichment Score (NES) | -2.4461718 |
| Nominal p-value | 0.0 |
| FDR q-value | 0.0 |
| FWER p-Value | 0.0 |
Table: GSEA Results Summary

  

Fig 1: Enrichment plot: KEGG\_LEUKOCYTE\_TRANSENDOTHELIAL\_MIGRATION      
 Profile of the Running ES Score & Positions of GeneSet Members on the Rank Ordered List

  

| SYMBOL | TITLE | RANK IN GENE LIST | RANK METRIC SCORE | RUNNING ES | CORE ENRICHMENT || 1 | CLDN15 | claudin 15 [Source:HGNC Symbol;Acc:HGNC:2036] | 1172 | 0.327 | -0.0118 | No |
| 2 | PIK3R2 | phosphoinositide-3-kinase regulatory subunit 2 [Source:HGNC Symbol;Acc:HGNC:8980] | 1248 | 0.320 | 0.0039 | No |
| 3 | PIK3R3 | phosphoinositide-3-kinase regulatory subunit 3 [Source:HGNC Symbol;Acc:HGNC:8981] | 2602 | 0.231 | -0.0178 | No |
| 4 | CLDN20 | claudin 20 [Source:HGNC Symbol;Acc:HGNC:2042] | 3475 | 0.194 | -0.0293 | No |
| 5 | VAV1 | vav guanine nucleotide exchange factor 1 [Source:HGNC Symbol;Acc:HGNC:12657] | 3764 | 0.184 | -0.0265 | No |
| 6 | CTNNA2 | catenin alpha 2 [Source:HGNC Symbol;Acc:HGNC:2510] | 3776 | 0.184 | -0.0167 | No |
| 7 | RASSF5 | Ras association domain family member 5 [Source:HGNC Symbol;Acc:HGNC:17609] | 4846 | 0.152 | -0.0355 | No |
| 8 | PECAM1 | platelet and endothelial cell adhesion molecule 1 [Source:HGNC Symbol;Acc:HGNC:8823] | 5267 | 0.142 | -0.0384 | No |
| 9 | CTNNA3 | catenin alpha 3 [Source:HGNC Symbol;Acc:HGNC:2511] | 5301 | 0.142 | -0.0314 | No |
| 10 | RAC2 | Rac family small GTPase 2 [Source:HGNC Symbol;Acc:HGNC:9802] | 6127 | 0.124 | -0.0456 | No |
| 11 | JAM3 | junctional adhesion molecule 3 [Source:HGNC Symbol;Acc:HGNC:15532] | 6317 | 0.121 | -0.0437 | No |
| 12 | CLDN14 | claudin 14 [Source:HGNC Symbol;Acc:HGNC:2035] | 7258 | 0.105 | -0.0619 | No |
| 13 | PIK3CB | "phosphatidylinositol-4,5-bisphosphate 3-kinase catalytic subunit beta [Source:HGNC Symbol;Acc:HGNC:8976]" | 7716 | 0.099 | -0.0681 | No |
| 14 | NOX1 | NADPH oxidase 1 [Source:HGNC Symbol;Acc:HGNC:7889] | 8987 | 0.083 | -0.0959 | No |
| 15 | CLDN4 | claudin 4 [Source:HGNC Symbol;Acc:HGNC:2046] | 10091 | 0.070 | -0.1202 | No |
| 16 | ITGA4 | integrin subunit alpha 4 [Source:HGNC Symbol;Acc:HGNC:6140] | 10741 | 0.063 | -0.1332 | No |
| 17 | OCLN | occludin [Source:HGNC Symbol;Acc:HGNC:8104] | 10925 | 0.062 | -0.1345 | No |
| 18 | CLDN11 | claudin 11 [Source:HGNC Symbol;Acc:HGNC:8514] | 11140 | 0.060 | -0.1367 | No |
| 19 | PRKCB | protein kinase C beta [Source:HGNC Symbol;Acc:HGNC:9395] | 11760 | 0.054 | -0.1495 | No |
| 20 | CLDN3 | claudin 3 [Source:HGNC Symbol;Acc:HGNC:2045] | 16060 | 0.025 | -0.2577 | No |
| 21 | CLDN9 | claudin 9 [Source:HGNC Symbol;Acc:HGNC:2051] | 17699 | 0.016 | -0.2986 | No |
| 22 | PTPN11 | protein tyrosine phosphatase non-receptor type 11 [Source:HGNC Symbol;Acc:HGNC:9644] | 18268 | 0.013 | -0.3124 | No |
| 23 | MYL10 | myosin light chain 10 [Source:HGNC Symbol;Acc:HGNC:29825] | 18653 | 0.011 | -0.3215 | No |
| 24 | MYL7 | myosin light chain 7 [Source:HGNC Symbol;Acc:HGNC:21719] | 18720 | 0.011 | -0.3226 | No |
| 25 | ARHGAP5 | Rho GTPase activating protein 5 [Source:HGNC Symbol;Acc:HGNC:675] | 19173 | 0.008 | -0.3337 | No |
| 26 | PTK2 | protein tyrosine kinase 2 [Source:HGNC Symbol;Acc:HGNC:9611] | 20287 | 0.002 | -0.3620 | No |
| 27 | NCF4 | neutrophil cytosolic factor 4 [Source:HGNC Symbol;Acc:HGNC:7662] | 20742 | -0.001 | -0.3736 | No |
| 28 | ITGB1 | integrin subunit beta 1 [Source:HGNC Symbol;Acc:HGNC:6153] | 22081 | -0.008 | -0.4072 | No |
| 29 | CLDN1 | claudin 1 [Source:HGNC Symbol;Acc:HGNC:2032] | 22342 | -0.010 | -0.4133 | No |
| 30 | CLDN17 | claudin 17 [Source:HGNC Symbol;Acc:HGNC:2038] | 22783 | -0.012 | -0.4239 | No |
| 31 | CYBA | cytochrome b-245 alpha chain [Source:HGNC Symbol;Acc:HGNC:2577] | 22961 | -0.014 | -0.4276 | No |
| 32 | ROCK2 | Rho associated coiled-coil containing protein kinase 2 [Source:HGNC Symbol;Acc:HGNC:10252] | 24395 | -0.022 | -0.4629 | No |
| 33 | CLDN8 | claudin 8 [Source:HGNC Symbol;Acc:HGNC:2050] | 25233 | -0.028 | -0.4827 | No |
| 34 | MAPK12 | mitogen-activated protein kinase 12 [Source:HGNC Symbol;Acc:HGNC:6874] | 25314 | -0.028 | -0.4832 | No |
| 35 | CLDN7 | claudin 7 [Source:HGNC Symbol;Acc:HGNC:2049] | 25756 | -0.032 | -0.4927 | No |
| 36 | ESAM | endothelial cell adhesion molecule [Source:HGNC Symbol;Acc:HGNC:17474] | 25881 | -0.033 | -0.4940 | No |
| 37 | ITGAL | integrin subunit alpha L [Source:HGNC Symbol;Acc:HGNC:6148] | 26570 | -0.038 | -0.5095 | No |
| 38 | ACTN2 | actinin alpha 2 [Source:HGNC Symbol;Acc:HGNC:164] | 27204 | -0.043 | -0.5233 | No |
| 39 | JAM2 | junctional adhesion molecule 2 [Source:HGNC Symbol;Acc:HGNC:14686] | 27435 | -0.045 | -0.5267 | No |
| 40 | ACTN4 | actinin alpha 4 [Source:HGNC Symbol;Acc:HGNC:166] | 28351 | -0.054 | -0.5470 | No |
| 41 | CLDN19 | claudin 19 [Source:HGNC Symbol;Acc:HGNC:2040] | 28611 | -0.056 | -0.5506 | No |
| 42 | CLDN16 | claudin 16 [Source:HGNC Symbol;Acc:HGNC:2037] | 28627 | -0.056 | -0.5478 | No |
| 43 | VAV3 | vav guanine nucleotide exchange factor 3 [Source:HGNC Symbol;Acc:HGNC:12659] | 29349 | -0.065 | -0.5627 | No |
| 44 | CLDN6 | claudin 6 [Source:HGNC Symbol;Acc:HGNC:2048] | 29352 | -0.065 | -0.5591 | No |
| 45 | MAPK13 | mitogen-activated protein kinase 13 [Source:HGNC Symbol;Acc:HGNC:6875] | 29402 | -0.065 | -0.5568 | No |
| 46 | ACTB | actin beta [Source:HGNC Symbol;Acc:HGNC:132] | 30724 | -0.081 | -0.5860 | No |
| 47 | CLDN18 | claudin 18 [Source:HGNC Symbol;Acc:HGNC:2039] | 30742 | -0.081 | -0.5820 | No |
| 48 | CLDN23 | claudin 23 [Source:HGNC Symbol;Acc:HGNC:17591] | 31086 | -0.086 | -0.5860 | No |
| 49 | NOX3 | NADPH oxidase 3 [Source:HGNC Symbol;Acc:HGNC:7890] | 31116 | -0.086 | -0.5819 | No |
| 50 | GNAI1 | G protein subunit alpha i1 [Source:HGNC Symbol;Acc:HGNC:4384] | 31213 | -0.088 | -0.5796 | No |
| 51 | CLDN10 | claudin 10 [Source:HGNC Symbol;Acc:HGNC:2033] | 31807 | -0.097 | -0.5894 | No |
| 52 | ARHGAP35 | Rho GTPase activating protein 35 [Source:HGNC Symbol;Acc:HGNC:4591] | 31911 | -0.098 | -0.5865 | No |
| 53 | GNAI3 | G protein subunit alpha i3 [Source:HGNC Symbol;Acc:HGNC:4387] | 32286 | -0.105 | -0.5903 | Yes |
| 54 | PLCG2 | phospholipase C gamma 2 [Source:HGNC Symbol;Acc:HGNC:9066] | 32294 | -0.105 | -0.5847 | Yes |
| 55 | RAPGEF4 | Rap guanine nucleotide exchange factor 4 [Source:HGNC Symbol;Acc:HGNC:16626] | 32455 | -0.108 | -0.5828 | Yes |
| 56 | SIPA1 | signal-induced proliferation-associated 1 [Source:HGNC Symbol;Acc:HGNC:10885] | 32543 | -0.110 | -0.5790 | Yes |
| 57 | VCAM1 | vascular cell adhesion molecule 1 [Source:HGNC Symbol;Acc:HGNC:12663] | 32626 | -0.111 | -0.5749 | Yes |
| 58 | TXK | TXK tyrosine kinase [Source:HGNC Symbol;Acc:HGNC:12434] | 32744 | -0.114 | -0.5716 | Yes |
| 59 | ACTN3 | actinin alpha 3 [Source:HGNC Symbol;Acc:HGNC:165] | 32823 | -0.115 | -0.5672 | Yes |
| 60 | PTK2B | protein tyrosine kinase 2 beta [Source:HGNC Symbol;Acc:HGNC:9612] | 32991 | -0.119 | -0.5649 | Yes |
| 61 | PRKCA | protein kinase C alpha [Source:HGNC Symbol;Acc:HGNC:9393] | 33017 | -0.119 | -0.5590 | Yes |
| 62 | MYL2 | myosin light chain 2 [Source:HGNC Symbol;Acc:HGNC:7583] | 33086 | -0.121 | -0.5540 | Yes |
| 63 | CYBB | cytochrome b-245 beta chain [Source:HGNC Symbol;Acc:HGNC:2578] | 33649 | -0.132 | -0.5611 | Yes |
| 64 | PIK3CD | "phosphatidylinositol-4,5-bisphosphate 3-kinase catalytic subunit delta [Source:HGNC Symbol;Acc:HGNC:8977]" | 33794 | -0.137 | -0.5572 | Yes |
| 65 | MYLPF | "myosin light chain, phosphorylatable, fast skeletal muscle [Source:HGNC Symbol;Acc:HGNC:29824]" | 33874 | -0.139 | -0.5516 | Yes |
| 66 | ACTG1 | actin gamma 1 [Source:HGNC Symbol;Acc:HGNC:144] | 34048 | -0.142 | -0.5481 | Yes |
| 67 | MYL5 | myosin light chain 5 [Source:HGNC Symbol;Acc:HGNC:7586] | 34055 | -0.143 | -0.5404 | Yes |
| 68 | PRKCG | protein kinase C gamma [Source:HGNC Symbol;Acc:HGNC:9402] | 34056 | -0.143 | -0.5325 | Yes |
| 69 | VAV2 | vav guanine nucleotide exchange factor 2 [Source:HGNC Symbol;Acc:HGNC:12658] | 34251 | -0.148 | -0.5293 | Yes |
| 70 | MYL12A | myosin light chain 12A [Source:HGNC Symbol;Acc:HGNC:16701] | 34438 | -0.154 | -0.5255 | Yes |
| 71 | CD99 | CD99 molecule (Xg blood group) [Source:HGNC Symbol;Acc:HGNC:7082] | 34602 | -0.159 | -0.5209 | Yes |
| 72 | THY1 | Thy-1 cell surface antigen [Source:HGNC Symbol;Acc:HGNC:11801] | 34809 | -0.165 | -0.5171 | Yes |
| 73 | PLCG1 | phospholipase C gamma 1 [Source:HGNC Symbol;Acc:HGNC:9065] | 34842 | -0.166 | -0.5088 | Yes |
| 74 | CLDN5 | claudin 5 [Source:HGNC Symbol;Acc:HGNC:2047] | 34875 | -0.167 | -0.5004 | Yes |
| 75 | ITK | IL2 inducible T cell kinase [Source:HGNC Symbol;Acc:HGNC:6171] | 34994 | -0.170 | -0.4940 | Yes |
| 76 | MAPK11 | mitogen-activated protein kinase 11 [Source:HGNC Symbol;Acc:HGNC:6873] | 35006 | -0.171 | -0.4848 | Yes |
| 77 | RHOH | ras homolog family member H [Source:HGNC Symbol;Acc:HGNC:686] | 35071 | -0.173 | -0.4769 | Yes |
| 78 | MSN | moesin [Source:HGNC Symbol;Acc:HGNC:7373] | 35197 | -0.178 | -0.4703 | Yes |
| 79 | CTNNA1 | catenin alpha 1 [Source:HGNC Symbol;Acc:HGNC:2509] | 35334 | -0.182 | -0.4637 | Yes |
| 80 | PXN | paxillin [Source:HGNC Symbol;Acc:HGNC:9718] | 35502 | -0.189 | -0.4575 | Yes |
| 81 | AFDN | "afadin, adherens junction formation factor [Source:HGNC Symbol;Acc:HGNC:7137]" | 35571 | -0.190 | -0.4487 | Yes |
| 82 | BCAR1 | "BCAR1 scaffold protein, Cas family member [Source:HGNC Symbol;Acc:HGNC:971]" | 35834 | -0.201 | -0.4444 | Yes |
| 83 | GNAI2 | G protein subunit alpha i2 [Source:HGNC Symbol;Acc:HGNC:4385] | 36074 | -0.210 | -0.4389 | Yes |
| 84 | VCL | vinculin [Source:HGNC Symbol;Acc:HGNC:12665] | 36176 | -0.215 | -0.4296 | Yes |
| 85 | PIK3CG | "phosphatidylinositol-4,5-bisphosphate 3-kinase catalytic subunit gamma [Source:HGNC Symbol;Acc:HGNC:8978]" | 36243 | -0.217 | -0.4193 | Yes |
| 86 | NCF1 | neutrophil cytosolic factor 1 [Source:HGNC Symbol;Acc:HGNC:7660] | 36267 | -0.218 | -0.4078 | Yes |
| 87 | ITGB2 | integrin subunit beta 2 [Source:HGNC Symbol;Acc:HGNC:6155] | 36308 | -0.220 | -0.3967 | Yes |
| 88 | CXCL12 | C-X-C motif chemokine ligand 12 [Source:HGNC Symbol;Acc:HGNC:10672] | 36344 | -0.222 | -0.3853 | Yes |
| 89 | CDH5 | cadherin 5 [Source:HGNC Symbol;Acc:HGNC:1764] | 36504 | -0.231 | -0.3766 | Yes |
| 90 | ACTN1 | actinin alpha 1 [Source:HGNC Symbol;Acc:HGNC:163] | 36614 | -0.236 | -0.3663 | Yes |
| 91 | MYL9 | myosin light chain 9 [Source:HGNC Symbol;Acc:HGNC:15754] | 36627 | -0.237 | -0.3536 | Yes |
| 92 | F11R | F11 receptor [Source:HGNC Symbol;Acc:HGNC:14685] | 36673 | -0.240 | -0.3415 | Yes |
| 93 | ITGAM | integrin subunit alpha M [Source:HGNC Symbol;Acc:HGNC:6149] | 36727 | -0.243 | -0.3294 | Yes |
| 94 | CXCR4 | C-X-C motif chemokine receptor 4 [Source:HGNC Symbol;Acc:HGNC:2561] | 36861 | -0.251 | -0.3189 | Yes |
| 95 | MMP9 | matrix metallopeptidase 9 [Source:HGNC Symbol;Acc:HGNC:7176] | 36875 | -0.252 | -0.3053 | Yes |
| 96 | NCF2 | neutrophil cytosolic factor 2 [Source:HGNC Symbol;Acc:HGNC:7661] | 36895 | -0.253 | -0.2919 | Yes |
| 97 | CTNND1 | catenin delta 1 [Source:HGNC Symbol;Acc:HGNC:2515] | 37003 | -0.260 | -0.2802 | Yes |
| 98 | PIK3R1 | phosphoinositide-3-kinase regulatory subunit 1 [Source:HGNC Symbol;Acc:HGNC:8979] | 37172 | -0.270 | -0.2696 | Yes |
| 99 | EZR | ezrin [Source:HGNC Symbol;Acc:HGNC:12691] | 37217 | -0.275 | -0.2556 | Yes |
| 100 | MMP2 | matrix metallopeptidase 2 [Source:HGNC Symbol;Acc:HGNC:7166] | 37244 | -0.276 | -0.2410 | Yes |
| 101 | RAC1 | Rac family small GTPase 1 [Source:HGNC Symbol;Acc:HGNC:9801] | 37263 | -0.277 | -0.2262 | Yes |
| 102 | RAP1A | "RAP1A, member of RAS oncogene family [Source:HGNC Symbol;Acc:HGNC:9855]" | 37388 | -0.287 | -0.2135 | Yes |
| 103 | RHOA | ras homolog family member A [Source:HGNC Symbol;Acc:HGNC:667] | 37417 | -0.290 | -0.1982 | Yes |
| 104 | ICAM1 | intercellular adhesion molecule 1 [Source:HGNC Symbol;Acc:HGNC:5344] | 37605 | -0.305 | -0.1861 | Yes |
| 105 | PIK3R5 | phosphoinositide-3-kinase regulatory subunit 5 [Source:HGNC Symbol;Acc:HGNC:30035] | 37628 | -0.307 | -0.1698 | Yes |
| 106 | MYL12B | myosin light chain 12B [Source:HGNC Symbol;Acc:HGNC:29827] | 37667 | -0.310 | -0.1536 | Yes |
| 107 | CLDN2 | claudin 2 [Source:HGNC Symbol;Acc:HGNC:2041] | 37736 | -0.317 | -0.1378 | Yes |
| 108 | CDC42 | cell division cycle 42 [Source:HGNC Symbol;Acc:HGNC:1736] | 37805 | -0.324 | -0.1217 | Yes |
| 109 | VASP | vasodilator stimulated phosphoprotein [Source:HGNC Symbol;Acc:HGNC:12652] | 38042 | -0.355 | -0.1081 | Yes |
| 110 | RAPGEF3 | Rap guanine nucleotide exchange factor 3 [Source:HGNC Symbol;Acc:HGNC:16629] | 38158 | -0.370 | -0.0906 | Yes |
| 111 | PIK3CA | "phosphatidylinositol-4,5-bisphosphate 3-kinase catalytic subunit alpha [Source:HGNC Symbol;Acc:HGNC:8975]" | 38268 | -0.391 | -0.0717 | Yes |
| 112 | MAPK14 | mitogen-activated protein kinase 14 [Source:HGNC Symbol;Acc:HGNC:6876] | 38287 | -0.396 | -0.0503 | Yes |
| 113 | RAP1B | "RAP1B, member of RAS oncogene family [Source:HGNC Symbol;Acc:HGNC:9857]" | 38403 | -0.421 | -0.0300 | Yes |
| 114 | CTNNB1 | catenin beta 1 [Source:HGNC Symbol;Acc:HGNC:2514] | 38456 | -0.438 | -0.0071 | Yes |
| 115 | ROCK1 | Rho associated coiled-coil containing protein kinase 1 [Source:HGNC Symbol;Acc:HGNC:10251] | 38575 | -0.519 | 0.0185 | Yes |
| 116 | CLDN22 | claudin 22 [Source:HGNC Symbol;Acc:HGNC:2044] | 39131 | NaN | 0.0049 | Yes |
Table: GSEA details [plain text format]

  

Fig 2: KEGG\_LEUKOCYTE\_TRANSENDOTHELIAL\_MIGRATION      
 Blue-Pink O' Gram in the Space of the Analyzed GeneSet

  

Fig 3: KEGG\_LEUKOCYTE\_TRANSENDOTHELIAL\_MIGRATION: Random ES distribution      
 Gene set null distribution of ES for **KEGG\_LEUKOCYTE\_TRANSENDOTHELIAL\_MIGRATION**
