# Supplementary material for: Simultaneous Presentation of Multiple Myeloma and Lung Cancer: Case Report and Gene Bioinformatics Analysis
Source: Front Oncol. 2022 Jun 13;12:859735. doi: 10.3389/fonc.2022.859735 (PMC9235397; doi:10.3389/fonc.2022.859735)
Supplement: Supplementary file 1 [file DataSheet_1.zip › The bioinformatic analysis of MM and lung cancer supplementary materials/Enrichment analysis/MECR/GSEA_4.1.0/MM TCGA/KEGG.Gsea.1639040591792/KEGG_MAPK_SIGNALING_PATHWAY.html]

Details for gene set KEGG\_MAPK\_SIGNALING\_PATHWAY[GSEA]

|  || Dataset | ExpData\_collapsed\_to\_symbols.ENSG00000116353\_profile\_in\_ExpData.cls #ENSG00000116353 |
| Phenotype | ENSG00000116353\_profile\_in\_ExpData.cls#ENSG00000116353 |
| Upregulated in class | ENSG00000116353\_neg |
| GeneSet | KEGG\_MAPK\_SIGNALING\_PATHWAY |
| Enrichment Score (ES) | -0.5316889 |
| Normalized Enrichment Score (NES) | -2.4005363 |
| Nominal p-value | 0.0 |
| FDR q-value | 0.0 |
| FWER p-Value | 0.0 |
Table: GSEA Results Summary

  

Fig 1: Enrichment plot: KEGG\_MAPK\_SIGNALING\_PATHWAY      
 Profile of the Running ES Score & Positions of GeneSet Members on the Rank Ordered List

  

| SYMBOL | TITLE | RANK IN GENE LIST | RANK METRIC SCORE | RUNNING ES | CORE ENRICHMENT || 1 | PPP5C | protein phosphatase 5 catalytic subunit [Source:HGNC Symbol;Acc:HGNC:9322] | 188 | 0.489 | 0.0073 | No |
| 2 | PLA2G12A | phospholipase A2 group XIIA [Source:HGNC Symbol;Acc:HGNC:18554] | 217 | 0.479 | 0.0184 | No |
| 3 | CACNB2 | calcium voltage-gated channel auxiliary subunit beta 2 [Source:HGNC Symbol;Acc:HGNC:1402] | 414 | 0.432 | 0.0241 | No |
| 4 | PPP3CB | protein phosphatase 3 catalytic subunit beta [Source:HGNC Symbol;Acc:HGNC:9315] | 842 | 0.363 | 0.0221 | No |
| 5 | STMN1 | stathmin 1 [Source:HGNC Symbol;Acc:HGNC:6510] | 1631 | 0.289 | 0.0091 | No |
| 6 | MAP2K5 | mitogen-activated protein kinase kinase 5 [Source:HGNC Symbol;Acc:HGNC:6845] | 2086 | 0.259 | 0.0039 | No |
| 7 | TP53 | tumor protein p53 [Source:HGNC Symbol;Acc:HGNC:11998] | 2121 | 0.257 | 0.0094 | No |
| 8 | MAP4K1 | mitogen-activated protein kinase kinase kinase kinase 1 [Source:HGNC Symbol;Acc:HGNC:6863] | 2681 | 0.228 | 0.0007 | No |
| 9 | DAXX | death domain associated protein [Source:HGNC Symbol;Acc:HGNC:2681] | 2908 | 0.217 | 0.0003 | No |
| 10 | NTF3 | neurotrophin 3 [Source:HGNC Symbol;Acc:HGNC:8023] | 3006 | 0.213 | 0.0031 | No |
| 11 | FOS | "Fos proto-oncogene, AP-1 transcription factor subunit [Source:HGNC Symbol;Acc:HGNC:3796]" | 3070 | 0.210 | 0.0067 | No |
| 12 | HSPA8 | heat shock protein family A (Hsp70) member 8 [Source:HGNC Symbol;Acc:HGNC:5241] | 3186 | 0.205 | 0.0088 | No |
| 13 | TAB1 | TGF-beta activated kinase 1 (MAP3K7) binding protein 1 [Source:HGNC Symbol;Acc:HGNC:18157] | 3360 | 0.199 | 0.0093 | No |
| 14 | DUSP1 | dual specificity phosphatase 1 [Source:HGNC Symbol;Acc:HGNC:3064] | 3887 | 0.180 | 0.0003 | No |
| 15 | PTPRR | protein tyrosine phosphatase receptor type R [Source:HGNC Symbol;Acc:HGNC:9680] | 4249 | 0.169 | -0.0048 | No |
| 16 | MAPKAPK3 | MAPK activated protein kinase 3 [Source:HGNC Symbol;Acc:HGNC:6888] | 4372 | 0.165 | -0.0038 | No |
| 17 | MAP3K13 | mitogen-activated protein kinase kinase kinase 13 [Source:HGNC Symbol;Acc:HGNC:6852] | 4667 | 0.157 | -0.0074 | No |
| 18 | PLA2G4A | phospholipase A2 group IVA [Source:HGNC Symbol;Acc:HGNC:9035] | 4721 | 0.155 | -0.0050 | No |
| 19 | MYC | "MYC proto-oncogene, bHLH transcription factor [Source:HGNC Symbol;Acc:HGNC:7553]" | 4752 | 0.155 | -0.0019 | No |
| 20 | MAP2K6 | mitogen-activated protein kinase kinase 6 [Source:HGNC Symbol;Acc:HGNC:6846] | 4800 | 0.154 | 0.0007 | No |
| 21 | JUN | "Jun proto-oncogene, AP-1 transcription factor subunit [Source:HGNC Symbol;Acc:HGNC:6204]" | 5088 | 0.146 | -0.0030 | No |
| 22 | MAP2K2 | mitogen-activated protein kinase kinase 2 [Source:HGNC Symbol;Acc:HGNC:6842] | 5104 | 0.146 | 0.0002 | No |
| 23 | CACNB4 | calcium voltage-gated channel auxiliary subunit beta 4 [Source:HGNC Symbol;Acc:HGNC:1404] | 5268 | 0.142 | -0.0005 | No |
| 24 | PLA2G6 | phospholipase A2 group VI [Source:HGNC Symbol;Acc:HGNC:9039] | 5302 | 0.142 | 0.0022 | No |
| 25 | MEF2C | myocyte enhancer factor 2C [Source:HGNC Symbol;Acc:HGNC:6996] | 5562 | 0.136 | -0.0011 | No |
| 26 | FGF14 | fibroblast growth factor 14 [Source:HGNC Symbol;Acc:HGNC:3671] | 5600 | 0.135 | 0.0013 | No |
| 27 | CACNA1C | calcium voltage-gated channel subunit alpha1 C [Source:HGNC Symbol;Acc:HGNC:1390] | 5783 | 0.131 | -0.0001 | No |
| 28 | RAC2 | Rac family small GTPase 2 [Source:HGNC Symbol;Acc:HGNC:9802] | 6127 | 0.124 | -0.0058 | No |
| 29 | RASGRF2 | Ras protein specific guanine nucleotide releasing factor 2 [Source:HGNC Symbol;Acc:HGNC:9876] | 6156 | 0.124 | -0.0034 | No |
| 30 | FGF13 | fibroblast growth factor 13 [Source:HGNC Symbol;Acc:HGNC:3670] | 6443 | 0.119 | -0.0078 | No |
| 31 | MAPT | microtubule associated protein tau [Source:HGNC Symbol;Acc:HGNC:6893] | 6492 | 0.118 | -0.0061 | No |
| 32 | NTRK1 | neurotrophic receptor tyrosine kinase 1 [Source:HGNC Symbol;Acc:HGNC:8031] | 6517 | 0.118 | -0.0038 | No |
| 33 | CACNG1 | calcium voltage-gated channel auxiliary subunit gamma 1 [Source:HGNC Symbol;Acc:HGNC:1405] | 6549 | 0.117 | -0.0017 | No |
| 34 | FGFR4 | fibroblast growth factor receptor 4 [Source:HGNC Symbol;Acc:HGNC:3691] | 6714 | 0.114 | -0.0031 | No |
| 35 | ECSIT | ECSIT signaling integrator [Source:HGNC Symbol;Acc:HGNC:29548] | 7331 | 0.104 | -0.0163 | No |
| 36 | CACNA2D3 | calcium voltage-gated channel auxiliary subunit alpha2delta 3 [Source:HGNC Symbol;Acc:HGNC:15460] | 7704 | 0.099 | -0.0234 | No |
| 37 | PAK2 | p21 (RAC1) activated kinase 2 [Source:HGNC Symbol;Acc:HGNC:8591] | 7725 | 0.099 | -0.0215 | No |
| 38 | BDNF | brain derived neurotrophic factor [Source:HGNC Symbol;Acc:HGNC:1033] | 8184 | 0.092 | -0.0309 | No |
| 39 | MAPK8IP2 | mitogen-activated protein kinase 8 interacting protein 2 [Source:HGNC Symbol;Acc:HGNC:6883] | 9276 | 0.079 | -0.0569 | No |
| 40 | RASA1 | RAS p21 protein activator 1 [Source:HGNC Symbol;Acc:HGNC:9871] | 9649 | 0.075 | -0.0646 | No |
| 41 | DUSP7 | dual specificity phosphatase 7 [Source:HGNC Symbol;Acc:HGNC:3073] | 9887 | 0.072 | -0.0689 | No |
| 42 | CACNG6 | calcium voltage-gated channel auxiliary subunit gamma 6 [Source:HGNC Symbol;Acc:HGNC:13625] | 9913 | 0.072 | -0.0677 | No |
| 43 | CHUK | component of inhibitor of nuclear factor kappa B kinase complex [Source:HGNC Symbol;Acc:HGNC:1974] | 9925 | 0.071 | -0.0662 | No |
| 44 | CACNA2D4 | calcium voltage-gated channel auxiliary subunit alpha2delta 4 [Source:HGNC Symbol;Acc:HGNC:20202] | 10136 | 0.069 | -0.0699 | No |
| 45 | TRAF2 | TNF receptor associated factor 2 [Source:HGNC Symbol;Acc:HGNC:12032] | 10627 | 0.064 | -0.0809 | No |
| 46 | FGF6 | fibroblast growth factor 6 [Source:HGNC Symbol;Acc:HGNC:3684] | 10831 | 0.063 | -0.0845 | No |
| 47 | STK3 | serine/threonine kinase 3 [Source:HGNC Symbol;Acc:HGNC:11406] | 10835 | 0.063 | -0.0830 | No |
| 48 | NTF4 | neurotrophin 4 [Source:HGNC Symbol;Acc:HGNC:8024] | 11291 | 0.058 | -0.0932 | No |
| 49 | FGFR3 | fibroblast growth factor receptor 3 [Source:HGNC Symbol;Acc:HGNC:3690] | 11317 | 0.058 | -0.0924 | No |
| 50 | PLA2G2C | phospholipase A2 group IIC [Source:HGNC Symbol;Acc:HGNC:9032] | 11486 | 0.057 | -0.0953 | No |
| 51 | PRKCB | protein kinase C beta [Source:HGNC Symbol;Acc:HGNC:9395] | 11760 | 0.054 | -0.1010 | No |
| 52 | PPP3R2 | "protein phosphatase 3 regulatory subunit B, beta [Source:HGNC Symbol;Acc:HGNC:9318]" | 11820 | 0.054 | -0.1012 | No |
| 53 | CACNG8 | calcium voltage-gated channel auxiliary subunit gamma 8 [Source:HGNC Symbol;Acc:HGNC:13628] | 11944 | 0.053 | -0.1030 | No |
| 54 | MAPKAPK2 | MAPK activated protein kinase 2 [Source:HGNC Symbol;Acc:HGNC:6887] | 12010 | 0.052 | -0.1034 | No |
| 55 | RPS6KA4 | ribosomal protein S6 kinase A4 [Source:HGNC Symbol;Acc:HGNC:10433] | 12313 | 0.050 | -0.1099 | No |
| 56 | MAP3K7 | mitogen-activated protein kinase kinase kinase 7 [Source:HGNC Symbol;Acc:HGNC:6859] | 12527 | 0.048 | -0.1141 | No |
| 57 | DUSP4 | dual specificity phosphatase 4 [Source:HGNC Symbol;Acc:HGNC:3070] | 12803 | 0.046 | -0.1200 | No |
| 58 | PRKACG | protein kinase cAMP-activated catalytic subunit gamma [Source:HGNC Symbol;Acc:HGNC:9382] | 12813 | 0.046 | -0.1191 | No |
| 59 | MECOM | MDS1 and EVI1 complex locus [Source:HGNC Symbol;Acc:HGNC:3498] | 13654 | 0.040 | -0.1396 | No |
| 60 | CACNA1S | calcium voltage-gated channel subunit alpha1 S [Source:HGNC Symbol;Acc:HGNC:1397] | 14115 | 0.037 | -0.1505 | No |
| 61 | CACNG7 | calcium voltage-gated channel auxiliary subunit gamma 7 [Source:HGNC Symbol;Acc:HGNC:13626] | 14147 | 0.037 | -0.1504 | No |
| 62 | PRKACA | protein kinase cAMP-activated catalytic subunit alpha [Source:HGNC Symbol;Acc:HGNC:9380] | 14554 | 0.035 | -0.1599 | No |
| 63 | TAOK1 | TAO kinase 1 [Source:HGNC Symbol;Acc:HGNC:29259] | 14914 | 0.033 | -0.1683 | No |
| 64 | CRK | "CRK proto-oncogene, adaptor protein [Source:HGNC Symbol;Acc:HGNC:2362]" | 14915 | 0.032 | -0.1675 | No |
| 65 | PLA2G2E | phospholipase A2 group IIE [Source:HGNC Symbol;Acc:HGNC:13414] | 14940 | 0.032 | -0.1673 | No |
| 66 | MAPK10 | mitogen-activated protein kinase 10 [Source:HGNC Symbol;Acc:HGNC:6872] | 14943 | 0.032 | -0.1665 | No |
| 67 | JMJD7-PLA2G4B | JMJD7-PLA2G4B readthrough [Source:HGNC Symbol;Acc:HGNC:34449] | 14979 | 0.032 | -0.1666 | No |
| 68 | PRKACB | protein kinase cAMP-activated catalytic subunit beta [Source:HGNC Symbol;Acc:HGNC:9381] | 15303 | 0.030 | -0.1742 | No |
| 69 | FGF3 | fibroblast growth factor 3 [Source:HGNC Symbol;Acc:HGNC:3681] | 15597 | 0.028 | -0.1810 | No |
| 70 | PLA2G2A | phospholipase A2 group IIA [Source:HGNC Symbol;Acc:HGNC:9031] | 15605 | 0.028 | -0.1804 | No |
| 71 | CHP1 | calcineurin like EF-hand protein 1 [Source:HGNC Symbol;Acc:HGNC:17433] | 15945 | 0.026 | -0.1885 | No |
| 72 | ELK4 | ETS transcription factor ELK4 [Source:HGNC Symbol;Acc:HGNC:3326] | 16013 | 0.026 | -0.1896 | No |
| 73 | FGF10 | fibroblast growth factor 10 [Source:HGNC Symbol;Acc:HGNC:3666] | 16552 | 0.023 | -0.2028 | No |
| 74 | CACNG4 | calcium voltage-gated channel auxiliary subunit gamma 4 [Source:HGNC Symbol;Acc:HGNC:1408] | 17085 | 0.020 | -0.2159 | No |
| 75 | CACNA1E | calcium voltage-gated channel subunit alpha1 E [Source:HGNC Symbol;Acc:HGNC:1392] | 17391 | 0.018 | -0.2233 | No |
| 76 | FGF12 | fibroblast growth factor 12 [Source:HGNC Symbol;Acc:HGNC:3668] | 17515 | 0.017 | -0.2260 | No |
| 77 | NFATC4 | nuclear factor of activated T cells 4 [Source:HGNC Symbol;Acc:HGNC:7778] | 17587 | 0.017 | -0.2274 | No |
| 78 | TGFB2 | transforming growth factor beta 2 [Source:HGNC Symbol;Acc:HGNC:11768] | 18348 | 0.013 | -0.2466 | No |
| 79 | SOS1 | SOS Ras/Rac guanine nucleotide exchange factor 1 [Source:HGNC Symbol;Acc:HGNC:11187] | 18532 | 0.012 | -0.2510 | No |
| 80 | PLA2G5 | phospholipase A2 group V [Source:HGNC Symbol;Acc:HGNC:9038] | 18961 | 0.009 | -0.2617 | No |
| 81 | FGF7 | fibroblast growth factor 7 [Source:HGNC Symbol;Acc:HGNC:3685] | 19331 | 0.007 | -0.2710 | No |
| 82 | FGF20 | fibroblast growth factor 20 [Source:HGNC Symbol;Acc:HGNC:3677] | 19442 | 0.007 | -0.2736 | No |
| 83 | MAPK9 | mitogen-activated protein kinase 9 [Source:HGNC Symbol;Acc:HGNC:6886] | 20291 | 0.002 | -0.2953 | No |
| 84 | IKBKB | inhibitor of nuclear factor kappa B kinase subunit beta [Source:HGNC Symbol;Acc:HGNC:5960] | 20718 | -0.000 | -0.3062 | No |
| 85 | NGF | nerve growth factor [Source:HGNC Symbol;Acc:HGNC:7808] | 20980 | -0.002 | -0.3128 | No |
| 86 | FASLG | Fas ligand [Source:HGNC Symbol;Acc:HGNC:11936] | 21141 | -0.003 | -0.3168 | No |
| 87 | FGF8 | fibroblast growth factor 8 [Source:HGNC Symbol;Acc:HGNC:3686] | 21259 | -0.004 | -0.3197 | No |
| 88 | CACNA1B | calcium voltage-gated channel subunit alpha1 B [Source:HGNC Symbol;Acc:HGNC:1389] | 21612 | -0.005 | -0.3286 | No |
| 89 | RASGRF1 | Ras protein specific guanine nucleotide releasing factor 1 [Source:HGNC Symbol;Acc:HGNC:9875] | 22030 | -0.008 | -0.3391 | No |
| 90 | PLA2G4E | phospholipase A2 group IVE [Source:HGNC Symbol;Acc:HGNC:24791] | 22141 | -0.009 | -0.3417 | No |
| 91 | PLA2G1B | phospholipase A2 group IB [Source:HGNC Symbol;Acc:HGNC:9030] | 22164 | -0.009 | -0.3420 | No |
| 92 | CACNG2 | calcium voltage-gated channel auxiliary subunit gamma 2 [Source:HGNC Symbol;Acc:HGNC:1406] | 22252 | -0.009 | -0.3440 | No |
| 93 | MOS | "MOS proto-oncogene, serine/threonine kinase [Source:HGNC Symbol;Acc:HGNC:7199]" | 22268 | -0.009 | -0.3442 | No |
| 94 | CACNA2D1 | calcium voltage-gated channel auxiliary subunit alpha2delta 1 [Source:HGNC Symbol;Acc:HGNC:1399] | 22531 | -0.011 | -0.3506 | No |
| 95 | CACNA1F | calcium voltage-gated channel subunit alpha1 F [Source:HGNC Symbol;Acc:HGNC:1393] | 22591 | -0.011 | -0.3519 | No |
| 96 | PRKX | protein kinase X-linked [Source:HGNC Symbol;Acc:HGNC:9441] | 23135 | -0.015 | -0.3654 | No |
| 97 | CHP2 | calcineurin like EF-hand protein 2 [Source:HGNC Symbol;Acc:HGNC:24927] | 23148 | -0.015 | -0.3654 | No |
| 98 | MAPK1 | mitogen-activated protein kinase 1 [Source:HGNC Symbol;Acc:HGNC:6871] | 23167 | -0.015 | -0.3655 | No |
| 99 | PLA2G2F | phospholipase A2 group IIF [Source:HGNC Symbol;Acc:HGNC:30040] | 23604 | -0.017 | -0.3762 | No |
| 100 | FGF23 | fibroblast growth factor 23 [Source:HGNC Symbol;Acc:HGNC:3680] | 23636 | -0.018 | -0.3765 | No |
| 101 | PTPN5 | protein tyrosine phosphatase non-receptor type 5 [Source:HGNC Symbol;Acc:HGNC:9657] | 23689 | -0.018 | -0.3774 | No |
| 102 | SRF | serum response factor [Source:HGNC Symbol;Acc:HGNC:11291] | 23807 | -0.019 | -0.3800 | No |
| 103 | MAPKAPK5 | MAPK activated protein kinase 5 [Source:HGNC Symbol;Acc:HGNC:6889] | 24052 | -0.020 | -0.3857 | No |
| 104 | NR4A1 | nuclear receptor subfamily 4 group A member 1 [Source:HGNC Symbol;Acc:HGNC:7980] | 24097 | -0.021 | -0.3863 | No |
| 105 | DUSP6 | dual specificity phosphatase 6 [Source:HGNC Symbol;Acc:HGNC:3072] | 24542 | -0.023 | -0.3971 | No |
| 106 | DUSP9 | dual specificity phosphatase 9 [Source:HGNC Symbol;Acc:HGNC:3076] | 25000 | -0.026 | -0.4082 | No |
| 107 | FGF1 | fibroblast growth factor 1 [Source:HGNC Symbol;Acc:HGNC:3665] | 25015 | -0.027 | -0.4079 | No |
| 108 | MAP3K4 | mitogen-activated protein kinase kinase kinase 4 [Source:HGNC Symbol;Acc:HGNC:6856] | 25165 | -0.027 | -0.4110 | No |
| 109 | MAP3K5 | mitogen-activated protein kinase kinase kinase 5 [Source:HGNC Symbol;Acc:HGNC:6857] | 25180 | -0.028 | -0.4107 | No |
| 110 | PLA2G2D | phospholipase A2 group IID [Source:HGNC Symbol;Acc:HGNC:9033] | 25199 | -0.028 | -0.4104 | No |
| 111 | MAPK12 | mitogen-activated protein kinase 12 [Source:HGNC Symbol;Acc:HGNC:6874] | 25314 | -0.028 | -0.4127 | No |
| 112 | DUSP5 | dual specificity phosphatase 5 [Source:HGNC Symbol;Acc:HGNC:3071] | 25397 | -0.029 | -0.4140 | No |
| 113 | RPS6KA1 | ribosomal protein S6 kinase A1 [Source:HGNC Symbol;Acc:HGNC:10430] | 25575 | -0.031 | -0.4178 | No |
| 114 | CACNG3 | calcium voltage-gated channel auxiliary subunit gamma 3 [Source:HGNC Symbol;Acc:HGNC:1407] | 25935 | -0.033 | -0.4262 | No |
| 115 | FGF21 | fibroblast growth factor 21 [Source:HGNC Symbol;Acc:HGNC:3678] | 26134 | -0.035 | -0.4304 | No |
| 116 | HRAS | "HRas proto-oncogene, GTPase [Source:HGNC Symbol;Acc:HGNC:5173]" | 26672 | -0.039 | -0.4432 | No |
| 117 | DUSP2 | dual specificity phosphatase 2 [Source:HGNC Symbol;Acc:HGNC:3068] | 27071 | -0.042 | -0.4523 | No |
| 118 | NLK | nemo like kinase [Source:HGNC Symbol;Acc:HGNC:29858] | 27227 | -0.043 | -0.4552 | No |
| 119 | CACNG5 | calcium voltage-gated channel auxiliary subunit gamma 5 [Source:HGNC Symbol;Acc:HGNC:1409] | 27880 | -0.049 | -0.4707 | No |
| 120 | RPS6KA2 | ribosomal protein S6 kinase A2 [Source:HGNC Symbol;Acc:HGNC:10431] | 27909 | -0.049 | -0.4702 | No |
| 121 | FGF5 | fibroblast growth factor 5 [Source:HGNC Symbol;Acc:HGNC:3683] | 28433 | -0.054 | -0.4823 | No |
| 122 | TAOK3 | TAO kinase 3 [Source:HGNC Symbol;Acc:HGNC:18133] | 28534 | -0.055 | -0.4835 | No |
| 123 | FGF22 | fibroblast growth factor 22 [Source:HGNC Symbol;Acc:HGNC:3679] | 28604 | -0.056 | -0.4838 | No |
| 124 | TAOK2 | TAO kinase 2 [Source:HGNC Symbol;Acc:HGNC:16835] | 28733 | -0.057 | -0.4857 | No |
| 125 | PPP3CA | protein phosphatase 3 catalytic subunit alpha [Source:HGNC Symbol;Acc:HGNC:9314] | 28890 | -0.059 | -0.4882 | No |
| 126 | CACNA1H | calcium voltage-gated channel subunit alpha1 H [Source:HGNC Symbol;Acc:HGNC:1395] | 28961 | -0.060 | -0.4885 | No |
| 127 | CACNA1A | calcium voltage-gated channel subunit alpha1 A [Source:HGNC Symbol;Acc:HGNC:1388] | 29046 | -0.061 | -0.4892 | No |
| 128 | DUSP10 | dual specificity phosphatase 10 [Source:HGNC Symbol;Acc:HGNC:3065] | 29331 | -0.064 | -0.4949 | No |
| 129 | MAPK13 | mitogen-activated protein kinase 13 [Source:HGNC Symbol;Acc:HGNC:6875] | 29402 | -0.065 | -0.4950 | No |
| 130 | PLA2G3 | phospholipase A2 group III [Source:HGNC Symbol;Acc:HGNC:17934] | 29427 | -0.065 | -0.4940 | No |
| 131 | NTRK2 | neurotrophic receptor tyrosine kinase 2 [Source:HGNC Symbol;Acc:HGNC:8032] | 29448 | -0.066 | -0.4929 | No |
| 132 | DUSP14 | dual specificity phosphatase 14 [Source:HGNC Symbol;Acc:HGNC:17007] | 29656 | -0.068 | -0.4965 | No |
| 133 | FGF4 | fibroblast growth factor 4 [Source:HGNC Symbol;Acc:HGNC:3682] | 29833 | -0.070 | -0.4993 | No |
| 134 | FGF18 | fibroblast growth factor 18 [Source:HGNC Symbol;Acc:HGNC:3674] | 29941 | -0.071 | -0.5003 | No |
| 135 | CACNB1 | calcium voltage-gated channel auxiliary subunit beta 1 [Source:HGNC Symbol;Acc:HGNC:1401] | 30310 | -0.076 | -0.5078 | No |
| 136 | PDGFA | platelet derived growth factor subunit A [Source:HGNC Symbol;Acc:HGNC:8799] | 30414 | -0.077 | -0.5086 | No |
| 137 | RAC3 | Rac family small GTPase 3 [Source:HGNC Symbol;Acc:HGNC:9803] | 30704 | -0.081 | -0.5140 | No |
| 138 | TGFB3 | transforming growth factor beta 3 [Source:HGNC Symbol;Acc:HGNC:11769] | 30832 | -0.083 | -0.5152 | No |
| 139 | CDC25B | cell division cycle 25B [Source:HGNC Symbol;Acc:HGNC:1726] | 30952 | -0.084 | -0.5161 | No |
| 140 | FGF17 | fibroblast growth factor 17 [Source:HGNC Symbol;Acc:HGNC:3673] | 31082 | -0.086 | -0.5173 | No |
| 141 | MAP3K12 | mitogen-activated protein kinase kinase kinase 12 [Source:HGNC Symbol;Acc:HGNC:6851] | 31214 | -0.088 | -0.5185 | No |
| 142 | FLNB | filamin B [Source:HGNC Symbol;Acc:HGNC:3755] | 31302 | -0.089 | -0.5185 | No |
| 143 | PLA2G4B | phospholipase A2 group IVB [Source:HGNC Symbol;Acc:HGNC:9036] | 31589 | -0.093 | -0.5236 | No |
| 144 | MRAS | muscle RAS oncogene homolog [Source:HGNC Symbol;Acc:HGNC:7227] | 31601 | -0.093 | -0.5215 | No |
| 145 | RPS6KA6 | ribosomal protein S6 kinase A6 [Source:HGNC Symbol;Acc:HGNC:10435] | 31671 | -0.094 | -0.5210 | No |
| 146 | AKT1 | AKT serine/threonine kinase 1 [Source:HGNC Symbol;Acc:HGNC:391] | 31906 | -0.098 | -0.5245 | No |
| 147 | MAP3K6 | mitogen-activated protein kinase kinase kinase 6 [Source:HGNC Symbol;Acc:HGNC:6858] | 31995 | -0.100 | -0.5243 | No |
| 148 | IKBKG | inhibitor of nuclear factor kappa B kinase regulatory subunit gamma [Source:HGNC Symbol;Acc:HGNC:5961] | 32205 | -0.103 | -0.5271 | No |
| 149 | HSPB1 | heat shock protein family B (small) member 1 [Source:HGNC Symbol;Acc:HGNC:5246] | 32306 | -0.105 | -0.5271 | No |
| 150 | CACNA1D | calcium voltage-gated channel subunit alpha1 D [Source:HGNC Symbol;Acc:HGNC:1391] | 32332 | -0.106 | -0.5251 | No |
| 151 | FGFR2 | fibroblast growth factor receptor 2 [Source:HGNC Symbol;Acc:HGNC:3689] | 32385 | -0.107 | -0.5238 | No |
| 152 | FGF19 | fibroblast growth factor 19 [Source:HGNC Symbol;Acc:HGNC:3675] | 32426 | -0.108 | -0.5222 | No |
| 153 | GNA12 | G protein subunit alpha 12 [Source:HGNC Symbol;Acc:HGNC:4380] | 32548 | -0.110 | -0.5225 | No |
| 154 | IL1A | interleukin 1 alpha [Source:HGNC Symbol;Acc:HGNC:5991] | 32718 | -0.113 | -0.5241 | No |
| 155 | PRKCA | protein kinase C alpha [Source:HGNC Symbol;Acc:HGNC:9393] | 33017 | -0.119 | -0.5287 | Yes |
| 156 | ELK1 | ETS transcription factor ELK1 [Source:HGNC Symbol;Acc:HGNC:3321] | 33028 | -0.120 | -0.5260 | Yes |
| 157 | PLA2G10 | phospholipase A2 group X [Source:HGNC Symbol;Acc:HGNC:9029] | 33032 | -0.120 | -0.5232 | Yes |
| 158 | FLNC | filamin C [Source:HGNC Symbol;Acc:HGNC:3756] | 33038 | -0.120 | -0.5203 | Yes |
| 159 | EGF | epidermal growth factor [Source:HGNC Symbol;Acc:HGNC:3229] | 33135 | -0.121 | -0.5198 | Yes |
| 160 | GADD45G | growth arrest and DNA damage inducible gamma [Source:HGNC Symbol;Acc:HGNC:4097] | 33212 | -0.123 | -0.5187 | Yes |
| 161 | AKT2 | AKT serine/threonine kinase 2 [Source:HGNC Symbol;Acc:HGNC:392] | 33213 | -0.123 | -0.5156 | Yes |
| 162 | CACNB3 | calcium voltage-gated channel auxiliary subunit beta 3 [Source:HGNC Symbol;Acc:HGNC:1403] | 33255 | -0.124 | -0.5136 | Yes |
| 163 | RPS6KA3 | ribosomal protein S6 kinase A3 [Source:HGNC Symbol;Acc:HGNC:10432] | 33320 | -0.125 | -0.5122 | Yes |
| 164 | FGF11 | fibroblast growth factor 11 [Source:HGNC Symbol;Acc:HGNC:3667] | 33539 | -0.130 | -0.5145 | Yes |
| 165 | PDGFB | platelet derived growth factor subunit B [Source:HGNC Symbol;Acc:HGNC:8800] | 33560 | -0.130 | -0.5118 | Yes |
| 166 | GNG12 | G protein subunit gamma 12 [Source:HGNC Symbol;Acc:HGNC:19663] | 33587 | -0.131 | -0.5092 | Yes |
| 167 | GADD45A | growth arrest and DNA damage inducible alpha [Source:HGNC Symbol;Acc:HGNC:4095] | 33646 | -0.132 | -0.5074 | Yes |
| 168 | RRAS | RAS related [Source:HGNC Symbol;Acc:HGNC:10447] | 33667 | -0.133 | -0.5047 | Yes |
| 169 | NRAS | "NRAS proto-oncogene, GTPase [Source:HGNC Symbol;Acc:HGNC:7989]" | 33739 | -0.135 | -0.5031 | Yes |
| 170 | PTPN7 | protein tyrosine phosphatase non-receptor type 7 [Source:HGNC Symbol;Acc:HGNC:9659] | 33818 | -0.137 | -0.5018 | Yes |
| 171 | PAK1 | p21 (RAC1) activated kinase 1 [Source:HGNC Symbol;Acc:HGNC:8590] | 33819 | -0.137 | -0.4984 | Yes |
| 172 | TGFBR2 | transforming growth factor beta receptor 2 [Source:HGNC Symbol;Acc:HGNC:11773] | 33892 | -0.139 | -0.4968 | Yes |
| 173 | CACNA2D2 | calcium voltage-gated channel auxiliary subunit alpha2delta 2 [Source:HGNC Symbol;Acc:HGNC:1400] | 34027 | -0.142 | -0.4967 | Yes |
| 174 | CACNA1G | calcium voltage-gated channel subunit alpha1 G [Source:HGNC Symbol;Acc:HGNC:1394] | 34032 | -0.142 | -0.4933 | Yes |
| 175 | PRKCG | protein kinase C gamma [Source:HGNC Symbol;Acc:HGNC:9402] | 34056 | -0.143 | -0.4903 | Yes |
| 176 | FGF16 | fibroblast growth factor 16 [Source:HGNC Symbol;Acc:HGNC:3672] | 34121 | -0.144 | -0.4884 | Yes |
| 177 | RASGRP1 | RAS guanyl releasing protein 1 [Source:HGNC Symbol;Acc:HGNC:9878] | 34180 | -0.146 | -0.4863 | Yes |
| 178 | TNFRSF1A | TNF receptor superfamily member 1A [Source:HGNC Symbol;Acc:HGNC:11916] | 34204 | -0.147 | -0.4832 | Yes |
| 179 | CRKL | "CRK like proto-oncogene, adaptor protein [Source:HGNC Symbol;Acc:HGNC:2363]" | 34457 | -0.154 | -0.4858 | Yes |
| 180 | AKT3 | AKT serine/threonine kinase 3 [Source:HGNC Symbol;Acc:HGNC:393] | 34501 | -0.156 | -0.4831 | Yes |
| 181 | PLA2G12B | phospholipase A2 group XIIB [Source:HGNC Symbol;Acc:HGNC:18555] | 34516 | -0.156 | -0.4796 | Yes |
| 182 | CASP3 | caspase 3 [Source:HGNC Symbol;Acc:HGNC:1504] | 34816 | -0.165 | -0.4832 | Yes |
| 183 | HSPA2 | heat shock protein family A (Hsp70) member 2 [Source:HGNC Symbol;Acc:HGNC:5235] | 34877 | -0.167 | -0.4806 | Yes |
| 184 | PDGFRB | platelet derived growth factor receptor beta [Source:HGNC Symbol;Acc:HGNC:8804] | 34887 | -0.167 | -0.4767 | Yes |
| 185 | MAP4K4 | mitogen-activated protein kinase kinase kinase kinase 4 [Source:HGNC Symbol;Acc:HGNC:6866] | 34924 | -0.168 | -0.4734 | Yes |
| 186 | RAPGEF2 | Rap guanine nucleotide exchange factor 2 [Source:HGNC Symbol;Acc:HGNC:16854] | 34936 | -0.169 | -0.4695 | Yes |
| 187 | ARRB1 | arrestin beta 1 [Source:HGNC Symbol;Acc:HGNC:711] | 34997 | -0.171 | -0.4668 | Yes |
| 188 | MAP2K7 | mitogen-activated protein kinase kinase 7 [Source:HGNC Symbol;Acc:HGNC:6847] | 35002 | -0.171 | -0.4627 | Yes |
| 189 | MAPK11 | mitogen-activated protein kinase 11 [Source:HGNC Symbol;Acc:HGNC:6873] | 35006 | -0.171 | -0.4586 | Yes |
| 190 | ATF2 | activating transcription factor 2 [Source:HGNC Symbol;Acc:HGNC:784] | 35029 | -0.172 | -0.4549 | Yes |
| 191 | RASGRP4 | RAS guanyl releasing protein 4 [Source:HGNC Symbol;Acc:HGNC:18958] | 35108 | -0.174 | -0.4526 | Yes |
| 192 | HSPA1L | heat shock protein family A (Hsp70) member 1 like [Source:HGNC Symbol;Acc:HGNC:5234] | 35112 | -0.175 | -0.4483 | Yes |
| 193 | MAX | MYC associated factor X [Source:HGNC Symbol;Acc:HGNC:6913] | 35248 | -0.179 | -0.4473 | Yes |
| 194 | FLNA | filamin A [Source:HGNC Symbol;Acc:HGNC:3754] | 35348 | -0.183 | -0.4454 | Yes |
| 195 | LAMTOR3 | "late endosomal/lysosomal adaptor, MAPK and MTOR activator 3 [Source:HGNC Symbol;Acc:HGNC:15606]" | 35353 | -0.183 | -0.4409 | Yes |
| 196 | EGFR | epidermal growth factor receptor [Source:HGNC Symbol;Acc:HGNC:3236] | 35417 | -0.186 | -0.4379 | Yes |
| 197 | ARRB2 | arrestin beta 2 [Source:HGNC Symbol;Acc:HGNC:712] | 35527 | -0.189 | -0.4361 | Yes |
| 198 | DUSP16 | dual specificity phosphatase 16 [Source:HGNC Symbol;Acc:HGNC:17909] | 35560 | -0.190 | -0.4322 | Yes |
| 199 | HSPA1B | heat shock protein family A (Hsp70) member 1B [Source:HGNC Symbol;Acc:HGNC:5233] | 35575 | -0.191 | -0.4278 | Yes |
| 200 | MAPK3 | mitogen-activated protein kinase 3 [Source:HGNC Symbol;Acc:HGNC:6877] | 35579 | -0.191 | -0.4232 | Yes |
| 201 | CACNA1I | calcium voltage-gated channel subunit alpha1 I [Source:HGNC Symbol;Acc:HGNC:1396] | 35740 | -0.197 | -0.4224 | Yes |
| 202 | FGF9 | fibroblast growth factor 9 [Source:HGNC Symbol;Acc:HGNC:3687] | 35849 | -0.202 | -0.4202 | Yes |
| 203 | IL1B | interleukin 1 beta [Source:HGNC Symbol;Acc:HGNC:5992] | 35858 | -0.202 | -0.4154 | Yes |
| 204 | MAP2K1 | mitogen-activated protein kinase kinase 1 [Source:HGNC Symbol;Acc:HGNC:6840] | 36024 | -0.208 | -0.4144 | Yes |
| 205 | JUND | "JunD proto-oncogene, AP-1 transcription factor subunit [Source:HGNC Symbol;Acc:HGNC:6206]" | 36039 | -0.209 | -0.4096 | Yes |
| 206 | RPS6KA5 | ribosomal protein S6 kinase A5 [Source:HGNC Symbol;Acc:HGNC:10434] | 36069 | -0.210 | -0.4052 | Yes |
| 207 | TGFB1 | transforming growth factor beta 1 [Source:HGNC Symbol;Acc:HGNC:11766] | 36247 | -0.217 | -0.4043 | Yes |
| 208 | CD14 | CD14 molecule [Source:HGNC Symbol;Acc:HGNC:1628] | 36298 | -0.220 | -0.4002 | Yes |
| 209 | MAP3K20 | mitogen-activated protein kinase kinase kinase 20 [Source:HGNC Symbol;Acc:HGNC:17797] | 36340 | -0.222 | -0.3957 | Yes |
| 210 | TNF | tumor necrosis factor [Source:HGNC Symbol;Acc:HGNC:11892] | 36419 | -0.226 | -0.3921 | Yes |
| 211 | DDIT3 | DNA damage inducible transcript 3 [Source:HGNC Symbol;Acc:HGNC:2726] | 36505 | -0.231 | -0.3886 | Yes |
| 212 | PDGFRA | platelet derived growth factor receptor alpha [Source:HGNC Symbol;Acc:HGNC:8803] | 36521 | -0.231 | -0.3833 | Yes |
| 213 | GRB2 | growth factor receptor bound protein 2 [Source:HGNC Symbol;Acc:HGNC:4566] | 36523 | -0.231 | -0.3776 | Yes |
| 214 | MAP4K2 | mitogen-activated protein kinase kinase kinase kinase 2 [Source:HGNC Symbol;Acc:HGNC:6864] | 36571 | -0.234 | -0.3730 | Yes |
| 215 | NFATC2 | nuclear factor of activated T cells 2 [Source:HGNC Symbol;Acc:HGNC:7776] | 36649 | -0.238 | -0.3690 | Yes |
| 216 | FGFR1 | fibroblast growth factor receptor 1 [Source:HGNC Symbol;Acc:HGNC:3688] | 36687 | -0.241 | -0.3640 | Yes |
| 217 | IL1R2 | interleukin 1 receptor type 2 [Source:HGNC Symbol;Acc:HGNC:5994] | 36783 | -0.246 | -0.3604 | Yes |
| 218 | ATF4 | activating transcription factor 4 [Source:HGNC Symbol;Acc:HGNC:786] | 36818 | -0.248 | -0.3551 | Yes |
| 219 | FAS | Fas cell surface death receptor [Source:HGNC Symbol;Acc:HGNC:11920] | 36825 | -0.248 | -0.3491 | Yes |
| 220 | MAP3K14 | mitogen-activated protein kinase kinase kinase 14 [Source:HGNC Symbol;Acc:HGNC:6853] | 36852 | -0.251 | -0.3436 | Yes |
| 221 | MKNK1 | MAPK interacting serine/threonine kinase 1 [Source:HGNC Symbol;Acc:HGNC:7110] | 36858 | -0.251 | -0.3375 | Yes |
| 222 | HSPA6 | heat shock protein family A (Hsp70) member 6 [Source:HGNC Symbol;Acc:HGNC:5239] | 36867 | -0.251 | -0.3315 | Yes |
| 223 | MAPK8IP1 | mitogen-activated protein kinase 8 interacting protein 1 [Source:HGNC Symbol;Acc:HGNC:6882] | 36887 | -0.253 | -0.3257 | Yes |
| 224 | FGF2 | fibroblast growth factor 2 [Source:HGNC Symbol;Acc:HGNC:3676] | 36896 | -0.253 | -0.3197 | Yes |
| 225 | RASA2 | RAS p21 protein activator 2 [Source:HGNC Symbol;Acc:HGNC:9872] | 36932 | -0.255 | -0.3142 | Yes |
| 226 | MAPK7 | mitogen-activated protein kinase 7 [Source:HGNC Symbol;Acc:HGNC:6880] | 37065 | -0.263 | -0.3111 | Yes |
| 227 | GADD45B | growth arrest and DNA damage inducible beta [Source:HGNC Symbol;Acc:HGNC:4096] | 37126 | -0.268 | -0.3060 | Yes |
| 228 | RRAS2 | RAS related 2 [Source:HGNC Symbol;Acc:HGNC:17271] | 37160 | -0.270 | -0.3002 | Yes |
| 229 | RASGRP2 | RAS guanyl releasing protein 2 [Source:HGNC Symbol;Acc:HGNC:9879] | 37170 | -0.270 | -0.2937 | Yes |
| 230 | STK4 | serine/threonine kinase 4 [Source:HGNC Symbol;Acc:HGNC:11408] | 37182 | -0.271 | -0.2873 | Yes |
| 231 | DUSP3 | dual specificity phosphatase 3 [Source:HGNC Symbol;Acc:HGNC:3069] | 37219 | -0.275 | -0.2814 | Yes |
| 232 | RAC1 | Rac family small GTPase 1 [Source:HGNC Symbol;Acc:HGNC:9801] | 37263 | -0.277 | -0.2757 | Yes |
| 233 | NF1 | neurofibromin 1 [Source:HGNC Symbol;Acc:HGNC:7765] | 37284 | -0.279 | -0.2693 | Yes |
| 234 | HSPA1A | heat shock protein family A (Hsp70) member 1A [Source:HGNC Symbol;Acc:HGNC:5232] | 37351 | -0.284 | -0.2639 | Yes |
| 235 | RAP1A | "RAP1A, member of RAS oncogene family [Source:HGNC Symbol;Acc:HGNC:9855]" | 37388 | -0.287 | -0.2578 | Yes |
| 236 | TAB2 | TGF-beta activated kinase 1 (MAP3K7) binding protein 2 [Source:HGNC Symbol;Acc:HGNC:17075] | 37429 | -0.290 | -0.2516 | Yes |
| 237 | IL1R1 | interleukin 1 receptor type 1 [Source:HGNC Symbol;Acc:HGNC:5993] | 37530 | -0.299 | -0.2468 | Yes |
| 238 | RASGRP3 | RAS guanyl releasing protein 3 [Source:HGNC Symbol;Acc:HGNC:14545] | 37579 | -0.303 | -0.2405 | Yes |
| 239 | DUSP8 | dual specificity phosphatase 8 [Source:HGNC Symbol;Acc:HGNC:3074] | 37720 | -0.316 | -0.2362 | Yes |
| 240 | MAP2K4 | mitogen-activated protein kinase kinase 4 [Source:HGNC Symbol;Acc:HGNC:6844] | 37740 | -0.317 | -0.2289 | Yes |
| 241 | KRAS | "KRAS proto-oncogene, GTPase [Source:HGNC Symbol;Acc:HGNC:6407]" | 37789 | -0.323 | -0.2221 | Yes |
| 242 | CDC42 | cell division cycle 42 [Source:HGNC Symbol;Acc:HGNC:1736] | 37805 | -0.324 | -0.2145 | Yes |
| 243 | RAF1 | "Raf-1 proto-oncogene, serine/threonine kinase [Source:HGNC Symbol;Acc:HGNC:9829]" | 37807 | -0.325 | -0.2065 | Yes |
| 244 | BRAF | "B-Raf proto-oncogene, serine/threonine kinase [Source:HGNC Symbol;Acc:HGNC:1097]" | 37844 | -0.330 | -0.1992 | Yes |
| 245 | MAP3K1 | mitogen-activated protein kinase kinase kinase 1 [Source:HGNC Symbol;Acc:HGNC:6848] | 37906 | -0.338 | -0.1924 | Yes |
| 246 | MAP3K11 | mitogen-activated protein kinase kinase kinase 11 [Source:HGNC Symbol;Acc:HGNC:6850] | 37943 | -0.343 | -0.1849 | Yes |
| 247 | PPP3R1 | "protein phosphatase 3 regulatory subunit B, alpha [Source:HGNC Symbol;Acc:HGNC:9317]" | 37988 | -0.348 | -0.1774 | Yes |
| 248 | MAP3K8 | mitogen-activated protein kinase kinase kinase 8 [Source:HGNC Symbol;Acc:HGNC:6860] | 38006 | -0.351 | -0.1691 | Yes |
| 249 | MAPK8IP3 | mitogen-activated protein kinase 8 interacting protein 3 [Source:HGNC Symbol;Acc:HGNC:6884] | 38009 | -0.351 | -0.1605 | Yes |
| 250 | TRAF6 | TNF receptor associated factor 6 [Source:HGNC Symbol;Acc:HGNC:12036] | 38202 | -0.379 | -0.1560 | Yes |
| 251 | NFKB2 | nuclear factor kappa B subunit 2 [Source:HGNC Symbol;Acc:HGNC:7795] | 38221 | -0.382 | -0.1470 | Yes |
| 252 | MAP2K3 | mitogen-activated protein kinase kinase 3 [Source:HGNC Symbol;Acc:HGNC:6843] | 38254 | -0.389 | -0.1382 | Yes |
| 253 | PPP3CC | protein phosphatase 3 catalytic subunit gamma [Source:HGNC Symbol;Acc:HGNC:9316] | 38255 | -0.389 | -0.1286 | Yes |
| 254 | MAPK14 | mitogen-activated protein kinase 14 [Source:HGNC Symbol;Acc:HGNC:6876] | 38287 | -0.396 | -0.1196 | Yes |
| 255 | MKNK2 | MAPK interacting serine/threonine kinase 2 [Source:HGNC Symbol;Acc:HGNC:7111] | 38314 | -0.401 | -0.1103 | Yes |
| 256 | MAPK8 | mitogen-activated protein kinase 8 [Source:HGNC Symbol;Acc:HGNC:6881] | 38339 | -0.406 | -0.1009 | Yes |
| 257 | TGFBR1 | transforming growth factor beta receptor 1 [Source:HGNC Symbol;Acc:HGNC:11772] | 38393 | -0.417 | -0.0920 | Yes |
| 258 | PPM1B | "protein phosphatase, Mg2+/Mn2+ dependent 1B [Source:HGNC Symbol;Acc:HGNC:9276]" | 38401 | -0.421 | -0.0817 | Yes |
| 259 | RAP1B | "RAP1B, member of RAS oncogene family [Source:HGNC Symbol;Acc:HGNC:9857]" | 38403 | -0.421 | -0.0713 | Yes |
| 260 | PPM1A | "protein phosphatase, Mg2+/Mn2+ dependent 1A [Source:HGNC Symbol;Acc:HGNC:9275]" | 38422 | -0.427 | -0.0612 | Yes |
| 261 | NFKB1 | nuclear factor kappa B subunit 1 [Source:HGNC Symbol;Acc:HGNC:7794] | 38470 | -0.443 | -0.0515 | Yes |
| 262 | RELA | "RELA proto-oncogene, NF-kB subunit [Source:HGNC Symbol;Acc:HGNC:9955]" | 38475 | -0.445 | -0.0406 | Yes |
| 263 | MAP3K3 | mitogen-activated protein kinase kinase kinase 3 [Source:HGNC Symbol;Acc:HGNC:6855] | 38504 | -0.456 | -0.0300 | Yes |
| 264 | RELB | "RELB proto-oncogene, NF-kB subunit [Source:HGNC Symbol;Acc:HGNC:9956]" | 38527 | -0.467 | -0.0190 | Yes |
| 265 | MAP4K3 | mitogen-activated protein kinase kinase kinase kinase 3 [Source:HGNC Symbol;Acc:HGNC:6865] | 38577 | -0.521 | -0.0073 | Yes |
| 266 | SOS2 | SOS Ras/Rho guanine nucleotide exchange factor 2 [Source:HGNC Symbol;Acc:HGNC:11188] | 38581 | -0.528 | 0.0057 | Yes |
| 267 | MAP3K2 | mitogen-activated protein kinase kinase kinase 2 [Source:HGNC Symbol;Acc:HGNC:6854] | 38584 | -0.538 | 0.0189 | Yes |
Table: GSEA details [plain text format]

  

Fig 2: KEGG\_MAPK\_SIGNALING\_PATHWAY      
 Blue-Pink O' Gram in the Space of the Analyzed GeneSet

  

Fig 3: KEGG\_MAPK\_SIGNALING\_PATHWAY: Random ES distribution      
 Gene set null distribution of ES for **KEGG\_MAPK\_SIGNALING\_PATHWAY**
