# Supplementary material for: Simultaneous Presentation of Multiple Myeloma and Lung Cancer: Case Report and Gene Bioinformatics Analysis
Source: Front Oncol. 2022 Jun 13;12:859735. doi: 10.3389/fonc.2022.859735 (PMC9235397; doi:10.3389/fonc.2022.859735)
Supplement: Supplementary file 1 [file DataSheet_1.zip › The bioinformatic analysis of MM and lung cancer supplementary materials/Enrichment analysis/MECR/GSEA_4.1.0/MM TCGA/KEGG.Gsea.1639040591792/KEGG_MISMATCH_REPAIR.html]

Details for gene set KEGG\_MISMATCH\_REPAIR[GSEA]

|  || Dataset | ExpData\_collapsed\_to\_symbols.ENSG00000116353\_profile\_in\_ExpData.cls #ENSG00000116353 |
| Phenotype | ENSG00000116353\_profile\_in\_ExpData.cls#ENSG00000116353 |
| Upregulated in class | ENSG00000116353\_pos |
| GeneSet | KEGG\_MISMATCH\_REPAIR |
| Enrichment Score (ES) | 0.7727895 |
| Normalized Enrichment Score (NES) | 2.1329854 |
| Nominal p-value | 0.0 |
| FDR q-value | 0.0 |
| FWER p-Value | 0.0 |
Table: GSEA Results Summary

  

Fig 1: Enrichment plot: KEGG\_MISMATCH\_REPAIR      
 Profile of the Running ES Score & Positions of GeneSet Members on the Rank Ordered List

  

| SYMBOL | TITLE | RANK IN GENE LIST | RANK METRIC SCORE | RUNNING ES | CORE ENRICHMENT || 1 | POLD2 | "DNA polymerase delta 2, accessory subunit [Source:HGNC Symbol;Acc:HGNC:9176]" | 15 | 0.617 | 0.0994 | Yes |
| 2 | RPA1 | replication protein A1 [Source:HGNC Symbol;Acc:HGNC:10289] | 360 | 0.442 | 0.1620 | Yes |
| 3 | MSH2 | mutS homolog 2 [Source:HGNC Symbol;Acc:HGNC:7325] | 369 | 0.440 | 0.2329 | Yes |
| 4 | POLD1 | "DNA polymerase delta 1, catalytic subunit [Source:HGNC Symbol;Acc:HGNC:9175]" | 541 | 0.405 | 0.2941 | Yes |
| 5 | MLH1 | mutL homolog 1 [Source:HGNC Symbol;Acc:HGNC:7127] | 738 | 0.377 | 0.3500 | Yes |
| 6 | MLH3 | mutL homolog 3 [Source:HGNC Symbol;Acc:HGNC:7128] | 889 | 0.356 | 0.4036 | Yes |
| 7 | MSH3 | mutS homolog 3 [Source:HGNC Symbol;Acc:HGNC:7326] | 916 | 0.352 | 0.4599 | Yes |
| 8 | EXO1 | exonuclease 1 [Source:HGNC Symbol;Acc:HGNC:3511] | 1061 | 0.338 | 0.5109 | Yes |
| 9 | PCNA | proliferating cell nuclear antigen [Source:HGNC Symbol;Acc:HGNC:8729] | 1093 | 0.336 | 0.5643 | Yes |
| 10 | PMS2 | "PMS1 homolog 2, mismatch repair system component [Source:HGNC Symbol;Acc:HGNC:9122]" | 1382 | 0.308 | 0.6067 | Yes |
| 11 | POLD3 | "DNA polymerase delta 3, accessory subunit [Source:HGNC Symbol;Acc:HGNC:20932]" | 1700 | 0.284 | 0.6445 | Yes |
| 12 | RFC4 | replication factor C subunit 4 [Source:HGNC Symbol;Acc:HGNC:9972] | 1728 | 0.281 | 0.6894 | Yes |
| 13 | SSBP1 | single stranded DNA binding protein 1 [Source:HGNC Symbol;Acc:HGNC:11317] | 2105 | 0.258 | 0.7214 | Yes |
| 14 | RPA3 | replication protein A3 [Source:HGNC Symbol;Acc:HGNC:10291] | 2705 | 0.227 | 0.7429 | Yes |
| 15 | RFC2 | replication factor C subunit 2 [Source:HGNC Symbol;Acc:HGNC:9970] | 3666 | 0.187 | 0.7487 | Yes |
| 16 | RFC3 | replication factor C subunit 3 [Source:HGNC Symbol;Acc:HGNC:9971] | 3869 | 0.181 | 0.7728 | Yes |
| 17 | RFC5 | replication factor C subunit 5 [Source:HGNC Symbol;Acc:HGNC:9973] | 6469 | 0.118 | 0.7258 | No |
| 18 | RPA2 | replication protein A2 [Source:HGNC Symbol;Acc:HGNC:10290] | 7917 | 0.096 | 0.7045 | No |
| 19 | LIG1 | DNA ligase 1 [Source:HGNC Symbol;Acc:HGNC:6598] | 24175 | -0.021 | 0.2942 | No |
| 20 | MSH6 | mutS homolog 6 [Source:HGNC Symbol;Acc:HGNC:7329] | 32133 | -0.102 | 0.1083 | No |
| 21 | RPA4 | replication protein A4 [Source:HGNC Symbol;Acc:HGNC:30305] | 32283 | -0.105 | 0.1214 | No |
| 22 | POLD4 | "DNA polymerase delta 4, accessory subunit [Source:HGNC Symbol;Acc:HGNC:14106]" | 34723 | -0.162 | 0.0856 | No |
| 23 | RFC1 | replication factor C subunit 1 [Source:HGNC Symbol;Acc:HGNC:9969] | 35666 | -0.195 | 0.0931 | No |
Table: GSEA details [plain text format]

  

Fig 2: KEGG\_MISMATCH\_REPAIR      
 Blue-Pink O' Gram in the Space of the Analyzed GeneSet

  

Fig 3: KEGG\_MISMATCH\_REPAIR: Random ES distribution      
 Gene set null distribution of ES for **KEGG\_MISMATCH\_REPAIR**
