# Supplementary material for: Simultaneous Presentation of Multiple Myeloma and Lung Cancer: Case Report and Gene Bioinformatics Analysis
Source: Front Oncol. 2022 Jun 13;12:859735. doi: 10.3389/fonc.2022.859735 (PMC9235397; doi:10.3389/fonc.2022.859735)
Supplement: Supplementary file 1 [file DataSheet_1.zip › The bioinformatic analysis of MM and lung cancer supplementary materials/Enrichment analysis/MECR/GSEA_4.1.0/MM TCGA/KEGG.Gsea.1639040591792/KEGG_NEUROTROPHIN_SIGNALING_PATHWAY.html]

Details for gene set KEGG\_NEUROTROPHIN\_SIGNALING\_PATHWAY[GSEA]

|  || Dataset | ExpData\_collapsed\_to\_symbols.ENSG00000116353\_profile\_in\_ExpData.cls #ENSG00000116353 |
| Phenotype | ENSG00000116353\_profile\_in\_ExpData.cls#ENSG00000116353 |
| Upregulated in class | ENSG00000116353\_neg |
| GeneSet | KEGG\_NEUROTROPHIN\_SIGNALING\_PATHWAY |
| Enrichment Score (ES) | -0.5949231 |
| Normalized Enrichment Score (NES) | -2.490158 |
| Nominal p-value | 0.0 |
| FDR q-value | 0.0 |
| FWER p-Value | 0.0 |
Table: GSEA Results Summary

  

Fig 1: Enrichment plot: KEGG\_NEUROTROPHIN\_SIGNALING\_PATHWAY      
 Profile of the Running ES Score & Positions of GeneSet Members on the Rank Ordered List

  

| SYMBOL | TITLE | RANK IN GENE LIST | RANK METRIC SCORE | RUNNING ES | CORE ENRICHMENT || 1 | PIK3R2 | phosphoinositide-3-kinase regulatory subunit 2 [Source:HGNC Symbol;Acc:HGNC:8980] | 1248 | 0.320 | -0.0182 | No |
| 2 | YWHAE | tyrosine 3-monooxygenase/tryptophan 5-monooxygenase activation protein epsilon [Source:HGNC Symbol;Acc:HGNC:12851] | 1274 | 0.318 | -0.0054 | No |
| 3 | BAX | "BCL2 associated X, apoptosis regulator [Source:HGNC Symbol;Acc:HGNC:959]" | 2048 | 0.261 | -0.0140 | No |
| 4 | MAP2K5 | mitogen-activated protein kinase kinase 5 [Source:HGNC Symbol;Acc:HGNC:6845] | 2086 | 0.259 | -0.0040 | No |
| 5 | TP53 | tumor protein p53 [Source:HGNC Symbol;Acc:HGNC:11998] | 2121 | 0.257 | 0.0061 | No |
| 6 | SHC4 | SHC adaptor protein 4 [Source:HGNC Symbol;Acc:HGNC:16743] | 2178 | 0.254 | 0.0154 | No |
| 7 | PIK3R3 | phosphoinositide-3-kinase regulatory subunit 3 [Source:HGNC Symbol;Acc:HGNC:8981] | 2602 | 0.231 | 0.0144 | No |
| 8 | NTF3 | neurotrophin 3 [Source:HGNC Symbol;Acc:HGNC:8023] | 3006 | 0.213 | 0.0132 | No |
| 9 | CSK | C-terminal Src kinase [Source:HGNC Symbol;Acc:HGNC:2444] | 3052 | 0.211 | 0.0210 | No |
| 10 | YWHAG | tyrosine 3-monooxygenase/tryptophan 5-monooxygenase activation protein gamma [Source:HGNC Symbol;Acc:HGNC:12852] | 3399 | 0.197 | 0.0205 | No |
| 11 | CALML6 | calmodulin like 6 [Source:HGNC Symbol;Acc:HGNC:24193] | 3642 | 0.188 | 0.0223 | No |
| 12 | NTRK3 | neurotrophic receptor tyrosine kinase 3 [Source:HGNC Symbol;Acc:HGNC:8033] | 4247 | 0.169 | 0.0141 | No |
| 13 | JUN | "Jun proto-oncogene, AP-1 transcription factor subunit [Source:HGNC Symbol;Acc:HGNC:6204]" | 5088 | 0.146 | -0.0011 | No |
| 14 | MAP2K2 | mitogen-activated protein kinase kinase 2 [Source:HGNC Symbol;Acc:HGNC:6842] | 5104 | 0.146 | 0.0047 | No |
| 15 | CALM2 | calmodulin 2 [Source:HGNC Symbol;Acc:HGNC:1445] | 5190 | 0.144 | 0.0086 | No |
| 16 | SHC3 | SHC adaptor protein 3 [Source:HGNC Symbol;Acc:HGNC:18181] | 5437 | 0.138 | 0.0082 | No |
| 17 | SH2B1 | SH2B adaptor protein 1 [Source:HGNC Symbol;Acc:HGNC:30417] | 5647 | 0.134 | 0.0085 | No |
| 18 | SH2B2 | SH2B adaptor protein 2 [Source:HGNC Symbol;Acc:HGNC:17381] | 5734 | 0.132 | 0.0120 | No |
| 19 | NTRK1 | neurotrophic receptor tyrosine kinase 1 [Source:HGNC Symbol;Acc:HGNC:8031] | 6517 | 0.118 | -0.0030 | No |
| 20 | SHC1 | SHC adaptor protein 1 [Source:HGNC Symbol;Acc:HGNC:10840] | 7103 | 0.108 | -0.0133 | No |
| 21 | MAGED1 | MAGE family member D1 [Source:HGNC Symbol;Acc:HGNC:6813] | 7180 | 0.106 | -0.0108 | No |
| 22 | PIK3CB | "phosphatidylinositol-4,5-bisphosphate 3-kinase catalytic subunit beta [Source:HGNC Symbol;Acc:HGNC:8976]" | 7716 | 0.099 | -0.0202 | No |
| 23 | BDNF | brain derived neurotrophic factor [Source:HGNC Symbol;Acc:HGNC:1033] | 8184 | 0.092 | -0.0282 | No |
| 24 | IRAK1 | interleukin 1 receptor associated kinase 1 [Source:HGNC Symbol;Acc:HGNC:6112] | 9888 | 0.072 | -0.0686 | No |
| 25 | CALML3 | calmodulin like 3 [Source:HGNC Symbol;Acc:HGNC:1452] | 10635 | 0.064 | -0.0849 | No |
| 26 | NTF4 | neurotrophin 4 [Source:HGNC Symbol;Acc:HGNC:8024] | 11291 | 0.058 | -0.0992 | No |
| 27 | MAPKAPK2 | MAPK activated protein kinase 2 [Source:HGNC Symbol;Acc:HGNC:6887] | 12010 | 0.052 | -0.1153 | No |
| 28 | GSK3B | glycogen synthase kinase 3 beta [Source:HGNC Symbol;Acc:HGNC:4617] | 12189 | 0.051 | -0.1176 | No |
| 29 | RPS6KA4 | ribosomal protein S6 kinase A4 [Source:HGNC Symbol;Acc:HGNC:10433] | 12313 | 0.050 | -0.1187 | No |
| 30 | BEX3 | brain expressed X-linked 3 [Source:HGNC Symbol;Acc:HGNC:13388] | 12777 | 0.047 | -0.1285 | No |
| 31 | CALM3 | calmodulin 3 [Source:HGNC Symbol;Acc:HGNC:1449] | 12780 | 0.047 | -0.1266 | No |
| 32 | CRK | "CRK proto-oncogene, adaptor protein [Source:HGNC Symbol;Acc:HGNC:2362]" | 14915 | 0.032 | -0.1796 | No |
| 33 | MAPK10 | mitogen-activated protein kinase 10 [Source:HGNC Symbol;Acc:HGNC:6872] | 14943 | 0.032 | -0.1789 | No |
| 34 | IRS1 | insulin receptor substrate 1 [Source:HGNC Symbol;Acc:HGNC:6125] | 15173 | 0.031 | -0.1835 | No |
| 35 | CAMK2B | calcium/calmodulin dependent protein kinase II beta [Source:HGNC Symbol;Acc:HGNC:1461] | 15627 | 0.028 | -0.1938 | No |
| 36 | IRS4 | insulin receptor substrate 4 [Source:HGNC Symbol;Acc:HGNC:6128] | 16117 | 0.025 | -0.2053 | No |
| 37 | KIDINS220 | kinase D interacting substrate 220 [Source:HGNC Symbol;Acc:HGNC:29508] | 16573 | 0.022 | -0.2159 | No |
| 38 | PRKCD | protein kinase C delta [Source:HGNC Symbol;Acc:HGNC:9399] | 17659 | 0.016 | -0.2429 | No |
| 39 | ABL1 | "ABL proto-oncogene 1, non-receptor tyrosine kinase [Source:HGNC Symbol;Acc:HGNC:76]" | 17707 | 0.016 | -0.2434 | No |
| 40 | PTPN11 | protein tyrosine phosphatase non-receptor type 11 [Source:HGNC Symbol;Acc:HGNC:9644] | 18268 | 0.013 | -0.2572 | No |
| 41 | CALML5 | calmodulin like 5 [Source:HGNC Symbol;Acc:HGNC:18180] | 18529 | 0.012 | -0.2633 | No |
| 42 | SOS1 | SOS Ras/Rac guanine nucleotide exchange factor 1 [Source:HGNC Symbol;Acc:HGNC:11187] | 18532 | 0.012 | -0.2629 | No |
| 43 | MAPK9 | mitogen-activated protein kinase 9 [Source:HGNC Symbol;Acc:HGNC:6886] | 20291 | 0.002 | -0.3076 | No |
| 44 | IKBKB | inhibitor of nuclear factor kappa B kinase subunit beta [Source:HGNC Symbol;Acc:HGNC:5960] | 20718 | -0.000 | -0.3185 | No |
| 45 | NGF | nerve growth factor [Source:HGNC Symbol;Acc:HGNC:7808] | 20980 | -0.002 | -0.3251 | No |
| 46 | FASLG | Fas ligand [Source:HGNC Symbol;Acc:HGNC:11936] | 21141 | -0.003 | -0.3290 | No |
| 47 | MAPK1 | mitogen-activated protein kinase 1 [Source:HGNC Symbol;Acc:HGNC:6871] | 23167 | -0.015 | -0.3800 | No |
| 48 | MAP3K5 | mitogen-activated protein kinase kinase kinase 5 [Source:HGNC Symbol;Acc:HGNC:6857] | 25180 | -0.028 | -0.4302 | No |
| 49 | MAPK12 | mitogen-activated protein kinase 12 [Source:HGNC Symbol;Acc:HGNC:6874] | 25314 | -0.028 | -0.4324 | No |
| 50 | RPS6KA1 | ribosomal protein S6 kinase A1 [Source:HGNC Symbol;Acc:HGNC:10430] | 25575 | -0.031 | -0.4377 | No |
| 51 | HRAS | "HRas proto-oncogene, GTPase [Source:HGNC Symbol;Acc:HGNC:5173]" | 26672 | -0.039 | -0.4640 | No |
| 52 | RPS6KA2 | ribosomal protein S6 kinase A2 [Source:HGNC Symbol;Acc:HGNC:10431] | 27909 | -0.049 | -0.4935 | No |
| 53 | YWHAB | tyrosine 3-monooxygenase/tryptophan 5-monooxygenase activation protein beta [Source:HGNC Symbol;Acc:HGNC:12849] | 28304 | -0.053 | -0.5013 | No |
| 54 | ARHGDIB | Rho GDP dissociation inhibitor beta [Source:HGNC Symbol;Acc:HGNC:679] | 28689 | -0.057 | -0.5087 | No |
| 55 | FOXO3 | forkhead box O3 [Source:HGNC Symbol;Acc:HGNC:3821] | 29077 | -0.061 | -0.5159 | No |
| 56 | CAMK2G | calcium/calmodulin dependent protein kinase II gamma [Source:HGNC Symbol;Acc:HGNC:1463] | 29260 | -0.063 | -0.5179 | No |
| 57 | MAPK13 | mitogen-activated protein kinase 13 [Source:HGNC Symbol;Acc:HGNC:6875] | 29402 | -0.065 | -0.5187 | No |
| 58 | NTRK2 | neurotrophic receptor tyrosine kinase 2 [Source:HGNC Symbol;Acc:HGNC:8032] | 29448 | -0.066 | -0.5171 | No |
| 59 | CAMK2D | calcium/calmodulin dependent protein kinase II delta [Source:HGNC Symbol;Acc:HGNC:1462] | 30146 | -0.074 | -0.5317 | No |
| 60 | IRAK4 | interleukin 1 receptor associated kinase 4 [Source:HGNC Symbol;Acc:HGNC:17967] | 30700 | -0.081 | -0.5424 | No |
| 61 | RPS6KA6 | ribosomal protein S6 kinase A6 [Source:HGNC Symbol;Acc:HGNC:10435] | 31671 | -0.094 | -0.5632 | No |
| 62 | AKT1 | AKT serine/threonine kinase 1 [Source:HGNC Symbol;Acc:HGNC:391] | 31906 | -0.098 | -0.5650 | No |
| 63 | PLCG2 | phospholipase C gamma 2 [Source:HGNC Symbol;Acc:HGNC:9066] | 32294 | -0.105 | -0.5704 | No |
| 64 | AKT2 | AKT serine/threonine kinase 2 [Source:HGNC Symbol;Acc:HGNC:392] | 33213 | -0.123 | -0.5886 | No |
| 65 | RPS6KA3 | ribosomal protein S6 kinase A3 [Source:HGNC Symbol;Acc:HGNC:10432] | 33320 | -0.125 | -0.5860 | No |
| 66 | SORT1 | sortilin 1 [Source:HGNC Symbol;Acc:HGNC:11186] | 33671 | -0.133 | -0.5893 | Yes |
| 67 | NRAS | "NRAS proto-oncogene, GTPase [Source:HGNC Symbol;Acc:HGNC:7989]" | 33739 | -0.135 | -0.5853 | Yes |
| 68 | PIK3CD | "phosphatidylinositol-4,5-bisphosphate 3-kinase catalytic subunit delta [Source:HGNC Symbol;Acc:HGNC:8977]" | 33794 | -0.137 | -0.5809 | Yes |
| 69 | CAMK4 | calcium/calmodulin dependent protein kinase IV [Source:HGNC Symbol;Acc:HGNC:1464] | 33910 | -0.139 | -0.5779 | Yes |
| 70 | TP73 | tumor protein p73 [Source:HGNC Symbol;Acc:HGNC:12003] | 34372 | -0.152 | -0.5832 | Yes |
| 71 | CRKL | "CRK like proto-oncogene, adaptor protein [Source:HGNC Symbol;Acc:HGNC:2363]" | 34457 | -0.154 | -0.5788 | Yes |
| 72 | AKT3 | AKT serine/threonine kinase 3 [Source:HGNC Symbol;Acc:HGNC:393] | 34501 | -0.156 | -0.5733 | Yes |
| 73 | SHC2 | SHC adaptor protein 2 [Source:HGNC Symbol;Acc:HGNC:29869] | 34623 | -0.160 | -0.5696 | Yes |
| 74 | PLCG1 | phospholipase C gamma 1 [Source:HGNC Symbol;Acc:HGNC:9065] | 34842 | -0.166 | -0.5681 | Yes |
| 75 | YWHAQ | tyrosine 3-monooxygenase/tryptophan 5-monooxygenase activation protein theta [Source:HGNC Symbol;Acc:HGNC:12854] | 34927 | -0.168 | -0.5631 | Yes |
| 76 | ZNF274 | zinc finger protein 274 [Source:HGNC Symbol;Acc:HGNC:13068] | 34940 | -0.169 | -0.5563 | Yes |
| 77 | MAP2K7 | mitogen-activated protein kinase kinase 7 [Source:HGNC Symbol;Acc:HGNC:6847] | 35002 | -0.171 | -0.5506 | Yes |
| 78 | MAPK11 | mitogen-activated protein kinase 11 [Source:HGNC Symbol;Acc:HGNC:6873] | 35006 | -0.171 | -0.5434 | Yes |
| 79 | CALM1 | calmodulin 1 [Source:HGNC Symbol;Acc:HGNC:1442] | 35210 | -0.178 | -0.5410 | Yes |
| 80 | CAMK2A | calcium/calmodulin dependent protein kinase II alpha [Source:HGNC Symbol;Acc:HGNC:1460] | 35431 | -0.186 | -0.5388 | Yes |
| 81 | MAPK3 | mitogen-activated protein kinase 3 [Source:HGNC Symbol;Acc:HGNC:6877] | 35579 | -0.191 | -0.5344 | Yes |
| 82 | MAP2K1 | mitogen-activated protein kinase kinase 1 [Source:HGNC Symbol;Acc:HGNC:6840] | 36024 | -0.208 | -0.5369 | Yes |
| 83 | IRS2 | insulin receptor substrate 2 [Source:HGNC Symbol;Acc:HGNC:6126] | 36046 | -0.209 | -0.5286 | Yes |
| 84 | RPS6KA5 | ribosomal protein S6 kinase A5 [Source:HGNC Symbol;Acc:HGNC:10434] | 36069 | -0.210 | -0.5202 | Yes |
| 85 | PIK3CG | "phosphatidylinositol-4,5-bisphosphate 3-kinase catalytic subunit gamma [Source:HGNC Symbol;Acc:HGNC:8978]" | 36243 | -0.217 | -0.5154 | Yes |
| 86 | ARHGDIA | Rho GDP dissociation inhibitor alpha [Source:HGNC Symbol;Acc:HGNC:678] | 36369 | -0.224 | -0.5091 | Yes |
| 87 | GRB2 | growth factor receptor bound protein 2 [Source:HGNC Symbol;Acc:HGNC:4566] | 36523 | -0.231 | -0.5032 | Yes |
| 88 | ATF4 | activating transcription factor 4 [Source:HGNC Symbol;Acc:HGNC:786] | 36818 | -0.248 | -0.5002 | Yes |
| 89 | YWHAZ | tyrosine 3-monooxygenase/tryptophan 5-monooxygenase activation protein zeta [Source:HGNC Symbol;Acc:HGNC:12855] | 36853 | -0.251 | -0.4904 | Yes |
| 90 | PDPK1 | 3-phosphoinositide dependent protein kinase 1 [Source:HGNC Symbol;Acc:HGNC:8816] | 36889 | -0.253 | -0.4806 | Yes |
| 91 | MAPK7 | mitogen-activated protein kinase 7 [Source:HGNC Symbol;Acc:HGNC:6880] | 37065 | -0.263 | -0.4739 | Yes |
| 92 | IRAK3 | interleukin 1 receptor associated kinase 3 [Source:HGNC Symbol;Acc:HGNC:17020] | 37171 | -0.270 | -0.4651 | Yes |
| 93 | PIK3R1 | phosphoinositide-3-kinase regulatory subunit 1 [Source:HGNC Symbol;Acc:HGNC:8979] | 37172 | -0.270 | -0.4536 | Yes |
| 94 | NFKBIA | NFKB inhibitor alpha [Source:HGNC Symbol;Acc:HGNC:7797] | 37222 | -0.275 | -0.4432 | Yes |
| 95 | RAC1 | Rac family small GTPase 1 [Source:HGNC Symbol;Acc:HGNC:9801] | 37263 | -0.277 | -0.4324 | Yes |
| 96 | NGFR | nerve growth factor receptor [Source:HGNC Symbol;Acc:HGNC:7809] | 37268 | -0.277 | -0.4208 | Yes |
| 97 | YWHAH | tyrosine 3-monooxygenase/tryptophan 5-monooxygenase activation protein eta [Source:HGNC Symbol;Acc:HGNC:12853] | 37365 | -0.285 | -0.4111 | Yes |
| 98 | RAP1A | "RAP1A, member of RAS oncogene family [Source:HGNC Symbol;Acc:HGNC:9855]" | 37388 | -0.287 | -0.3995 | Yes |
| 99 | PSEN1 | presenilin 1 [Source:HGNC Symbol;Acc:HGNC:9508] | 37403 | -0.288 | -0.3877 | Yes |
| 100 | RHOA | ras homolog family member A [Source:HGNC Symbol;Acc:HGNC:667] | 37417 | -0.290 | -0.3757 | Yes |
| 101 | PIK3R5 | phosphoinositide-3-kinase regulatory subunit 5 [Source:HGNC Symbol;Acc:HGNC:30035] | 37628 | -0.307 | -0.3680 | Yes |
| 102 | BCL2 | BCL2 apoptosis regulator [Source:HGNC Symbol;Acc:HGNC:990] | 37727 | -0.316 | -0.3571 | Yes |
| 103 | BAD | BCL2 associated agonist of cell death [Source:HGNC Symbol;Acc:HGNC:936] | 37742 | -0.318 | -0.3440 | Yes |
| 104 | PRDM4 | PR/SET domain 4 [Source:HGNC Symbol;Acc:HGNC:9348] | 37780 | -0.322 | -0.3313 | Yes |
| 105 | KRAS | "KRAS proto-oncogene, GTPase [Source:HGNC Symbol;Acc:HGNC:6407]" | 37789 | -0.323 | -0.3178 | Yes |
| 106 | CDC42 | cell division cycle 42 [Source:HGNC Symbol;Acc:HGNC:1736] | 37805 | -0.324 | -0.3044 | Yes |
| 107 | RAF1 | "Raf-1 proto-oncogene, serine/threonine kinase [Source:HGNC Symbol;Acc:HGNC:9829]" | 37807 | -0.325 | -0.2907 | Yes |
| 108 | BRAF | "B-Raf proto-oncogene, serine/threonine kinase [Source:HGNC Symbol;Acc:HGNC:1097]" | 37844 | -0.330 | -0.2776 | Yes |
| 109 | MAP3K1 | mitogen-activated protein kinase kinase kinase 1 [Source:HGNC Symbol;Acc:HGNC:6848] | 37906 | -0.338 | -0.2648 | Yes |
| 110 | NFKBIB | NFKB inhibitor beta [Source:HGNC Symbol;Acc:HGNC:7798] | 37987 | -0.348 | -0.2521 | Yes |
| 111 | SH2B3 | SH2B adaptor protein 3 [Source:HGNC Symbol;Acc:HGNC:29605] | 38035 | -0.354 | -0.2383 | Yes |
| 112 | TRAF6 | TNF receptor associated factor 6 [Source:HGNC Symbol;Acc:HGNC:12036] | 38202 | -0.379 | -0.2264 | Yes |
| 113 | PIK3CA | "phosphatidylinositol-4,5-bisphosphate 3-kinase catalytic subunit alpha [Source:HGNC Symbol;Acc:HGNC:8975]" | 38268 | -0.391 | -0.2115 | Yes |
| 114 | MAPK14 | mitogen-activated protein kinase 14 [Source:HGNC Symbol;Acc:HGNC:6876] | 38287 | -0.396 | -0.1951 | Yes |
| 115 | IRAK2 | interleukin 1 receptor associated kinase 2 [Source:HGNC Symbol;Acc:HGNC:6113] | 38318 | -0.402 | -0.1788 | Yes |
| 116 | FRS2 | fibroblast growth factor receptor substrate 2 [Source:HGNC Symbol;Acc:HGNC:16971] | 38323 | -0.403 | -0.1618 | Yes |
| 117 | GAB1 | GRB2 associated binding protein 1 [Source:HGNC Symbol;Acc:HGNC:4066] | 38338 | -0.406 | -0.1449 | Yes |
| 118 | MAPK8 | mitogen-activated protein kinase 8 [Source:HGNC Symbol;Acc:HGNC:6881] | 38339 | -0.406 | -0.1277 | Yes |
| 119 | RAPGEF1 | Rap guanine nucleotide exchange factor 1 [Source:HGNC Symbol;Acc:HGNC:4568] | 38386 | -0.414 | -0.1113 | Yes |
| 120 | RAP1B | "RAP1B, member of RAS oncogene family [Source:HGNC Symbol;Acc:HGNC:9857]" | 38403 | -0.421 | -0.0938 | Yes |
| 121 | NFKBIE | NFKB inhibitor epsilon [Source:HGNC Symbol;Acc:HGNC:7799] | 38410 | -0.422 | -0.0761 | Yes |
| 122 | NFKB1 | nuclear factor kappa B subunit 1 [Source:HGNC Symbol;Acc:HGNC:7794] | 38470 | -0.443 | -0.0588 | Yes |
| 123 | RELA | "RELA proto-oncogene, NF-kB subunit [Source:HGNC Symbol;Acc:HGNC:9955]" | 38475 | -0.445 | -0.0400 | Yes |
| 124 | MAP3K3 | mitogen-activated protein kinase kinase kinase 3 [Source:HGNC Symbol;Acc:HGNC:6855] | 38504 | -0.456 | -0.0214 | Yes |
| 125 | RIPK2 | receptor interacting serine/threonine kinase 2 [Source:HGNC Symbol;Acc:HGNC:10020] | 38525 | -0.467 | -0.0021 | Yes |
| 126 | SOS2 | SOS Ras/Rho guanine nucleotide exchange factor 2 [Source:HGNC Symbol;Acc:HGNC:11188] | 38581 | -0.528 | 0.0189 | Yes |
Table: GSEA details [plain text format]

  

Fig 2: KEGG\_NEUROTROPHIN\_SIGNALING\_PATHWAY      
 Blue-Pink O' Gram in the Space of the Analyzed GeneSet

  

Fig 3: KEGG\_NEUROTROPHIN\_SIGNALING\_PATHWAY: Random ES distribution      
 Gene set null distribution of ES for **KEGG\_NEUROTROPHIN\_SIGNALING\_PATHWAY**
