# Supplementary material for: Simultaneous Presentation of Multiple Myeloma and Lung Cancer: Case Report and Gene Bioinformatics Analysis
Source: Front Oncol. 2022 Jun 13;12:859735. doi: 10.3389/fonc.2022.859735 (PMC9235397; doi:10.3389/fonc.2022.859735)
Supplement: Supplementary file 1 [file DataSheet_1.zip › The bioinformatic analysis of MM and lung cancer supplementary materials/Enrichment analysis/MECR/GSEA_4.1.0/MM TCGA/KEGG.Gsea.1639040591792/KEGG_NOD_LIKE_RECEPTOR_SIGNALING_PATHWAY.html]

Details for gene set KEGG\_NOD\_LIKE\_RECEPTOR\_SIGNALING\_PATHWAY[GSEA]

|  || Dataset | ExpData\_collapsed\_to\_symbols.ENSG00000116353\_profile\_in\_ExpData.cls #ENSG00000116353 |
| Phenotype | ENSG00000116353\_profile\_in\_ExpData.cls#ENSG00000116353 |
| Upregulated in class | ENSG00000116353\_neg |
| GeneSet | KEGG\_NOD\_LIKE\_RECEPTOR\_SIGNALING\_PATHWAY |
| Enrichment Score (ES) | -0.6357234 |
| Normalized Enrichment Score (NES) | -2.3229337 |
| Nominal p-value | 0.0 |
| FDR q-value | 0.0 |
| FWER p-Value | 0.0 |
Table: GSEA Results Summary

  

Fig 1: Enrichment plot: KEGG\_NOD\_LIKE\_RECEPTOR\_SIGNALING\_PATHWAY      
 Profile of the Running ES Score & Positions of GeneSet Members on the Rank Ordered List

  

| SYMBOL | TITLE | RANK IN GENE LIST | RANK METRIC SCORE | RUNNING ES | CORE ENRICHMENT || 1 | CARD8 | caspase recruitment domain family member 8 [Source:HGNC Symbol;Acc:HGNC:17057] | 2108 | 0.257 | -0.0303 | No |
| 2 | TAB1 | TGF-beta activated kinase 1 (MAP3K7) binding protein 1 [Source:HGNC Symbol;Acc:HGNC:18157] | 3360 | 0.199 | -0.0442 | No |
| 3 | CARD18 | caspase recruitment domain family member 18 [Source:HGNC Symbol;Acc:HGNC:28861] | 4226 | 0.170 | -0.0508 | No |
| 4 | CASP1 | caspase 1 [Source:HGNC Symbol;Acc:HGNC:1499] | 5087 | 0.146 | -0.0595 | No |
| 5 | NLRC4 | NLR family CARD domain containing 4 [Source:HGNC Symbol;Acc:HGNC:16412] | 5547 | 0.136 | -0.0588 | No |
| 6 | CARD6 | caspase recruitment domain family member 6 [Source:HGNC Symbol;Acc:HGNC:16394] | 5906 | 0.129 | -0.0563 | No |
| 7 | NOD1 | nucleotide binding oligomerization domain containing 1 [Source:HGNC Symbol;Acc:HGNC:16390] | 6696 | 0.114 | -0.0660 | No |
| 8 | CASP5 | caspase 5 [Source:HGNC Symbol;Acc:HGNC:1506] | 6792 | 0.113 | -0.0582 | No |
| 9 | HSP90B1 | heat shock protein 90 beta family member 1 [Source:HGNC Symbol;Acc:HGNC:12028] | 7140 | 0.107 | -0.0573 | No |
| 10 | CHUK | component of inhibitor of nuclear factor kappa B kinase complex [Source:HGNC Symbol;Acc:HGNC:1974] | 9925 | 0.071 | -0.1218 | No |
| 11 | HSP90AB1 | heat shock protein 90 alpha family class B member 1 [Source:HGNC Symbol;Acc:HGNC:5258] | 10928 | 0.062 | -0.1417 | No |
| 12 | PYCARD | PYD and CARD domain containing [Source:HGNC Symbol;Acc:HGNC:16608] | 11848 | 0.053 | -0.1603 | No |
| 13 | MAP3K7 | mitogen-activated protein kinase kinase kinase 7 [Source:HGNC Symbol;Acc:HGNC:6859] | 12527 | 0.048 | -0.1731 | No |
| 14 | XIAP | X-linked inhibitor of apoptosis [Source:HGNC Symbol;Acc:HGNC:592] | 13774 | 0.039 | -0.2013 | No |
| 15 | MAPK10 | mitogen-activated protein kinase 10 [Source:HGNC Symbol;Acc:HGNC:6872] | 14943 | 0.032 | -0.2281 | No |
| 16 | NAIP | NLR family apoptosis inhibitory protein [Source:HGNC Symbol;Acc:HGNC:7634] | 18076 | 0.014 | -0.3066 | No |
| 17 | MAPK9 | mitogen-activated protein kinase 9 [Source:HGNC Symbol;Acc:HGNC:6886] | 20291 | 0.002 | -0.3628 | No |
| 18 | PYDC1 | pyrin domain containing 1 [Source:HGNC Symbol;Acc:HGNC:30261] | 20514 | 0.001 | -0.3684 | No |
| 19 | IKBKB | inhibitor of nuclear factor kappa B kinase subunit beta [Source:HGNC Symbol;Acc:HGNC:5960] | 20718 | -0.000 | -0.3736 | No |
| 20 | MAPK1 | mitogen-activated protein kinase 1 [Source:HGNC Symbol;Acc:HGNC:6871] | 23167 | -0.015 | -0.4346 | No |
| 21 | MAPK12 | mitogen-activated protein kinase 12 [Source:HGNC Symbol;Acc:HGNC:6874] | 25314 | -0.028 | -0.4866 | No |
| 22 | TRIP6 | thyroid hormone receptor interactor 6 [Source:HGNC Symbol;Acc:HGNC:12311] | 25474 | -0.030 | -0.4880 | No |
| 23 | CCL11 | C-C motif chemokine ligand 11 [Source:HGNC Symbol;Acc:HGNC:10610] | 26778 | -0.039 | -0.5176 | No |
| 24 | CCL8 | C-C motif chemokine ligand 8 [Source:HGNC Symbol;Acc:HGNC:10635] | 29326 | -0.064 | -0.5766 | No |
| 25 | CCL13 | C-C motif chemokine ligand 13 [Source:HGNC Symbol;Acc:HGNC:10611] | 29378 | -0.065 | -0.5721 | No |
| 26 | MAPK13 | mitogen-activated protein kinase 13 [Source:HGNC Symbol;Acc:HGNC:6875] | 29402 | -0.065 | -0.5668 | No |
| 27 | IL6 | interleukin 6 [Source:HGNC Symbol;Acc:HGNC:6018] | 29703 | -0.068 | -0.5682 | No |
| 28 | IKBKG | inhibitor of nuclear factor kappa B kinase regulatory subunit gamma [Source:HGNC Symbol;Acc:HGNC:5961] | 32205 | -0.103 | -0.6225 | No |
| 29 | NLRP1 | NLR family pyrin domain containing 1 [Source:HGNC Symbol;Acc:HGNC:14374] | 32670 | -0.112 | -0.6242 | Yes |
| 30 | MEFV | "MEFV innate immuity regulator, pyrin [Source:HGNC Symbol;Acc:HGNC:6998]" | 33045 | -0.120 | -0.6228 | Yes |
| 31 | SUGT1 | "SGT1 homolog, MIS12 kinetochore complex assembly cochaperone [Source:HGNC Symbol;Acc:HGNC:16987]" | 33552 | -0.130 | -0.6239 | Yes |
| 32 | HSP90AA1 | heat shock protein 90 alpha family class A member 1 [Source:HGNC Symbol;Acc:HGNC:5253] | 33724 | -0.134 | -0.6161 | Yes |
| 33 | PSTPIP1 | proline-serine-threonine phosphatase interacting protein 1 [Source:HGNC Symbol;Acc:HGNC:9580] | 34172 | -0.146 | -0.6142 | Yes |
| 34 | IL18 | interleukin 18 [Source:HGNC Symbol;Acc:HGNC:5986] | 34385 | -0.152 | -0.6059 | Yes |
| 35 | CCL5 | C-C motif chemokine ligand 5 [Source:HGNC Symbol;Acc:HGNC:10632] | 34763 | -0.164 | -0.6006 | Yes |
| 36 | MAPK11 | mitogen-activated protein kinase 11 [Source:HGNC Symbol;Acc:HGNC:6873] | 35006 | -0.171 | -0.5913 | Yes |
| 37 | CCL2 | C-C motif chemokine ligand 2 [Source:HGNC Symbol;Acc:HGNC:10618] | 35124 | -0.175 | -0.5784 | Yes |
| 38 | CCL7 | C-C motif chemokine ligand 7 [Source:HGNC Symbol;Acc:HGNC:10634] | 35132 | -0.175 | -0.5627 | Yes |
| 39 | CARD9 | caspase recruitment domain family member 9 [Source:HGNC Symbol;Acc:HGNC:16391] | 35200 | -0.178 | -0.5483 | Yes |
| 40 | MAPK3 | mitogen-activated protein kinase 3 [Source:HGNC Symbol;Acc:HGNC:6877] | 35579 | -0.191 | -0.5407 | Yes |
| 41 | IL1B | interleukin 1 beta [Source:HGNC Symbol;Acc:HGNC:5992] | 35858 | -0.202 | -0.5294 | Yes |
| 42 | CXCL1 | C-X-C motif chemokine ligand 1 [Source:HGNC Symbol;Acc:HGNC:4602] | 36378 | -0.224 | -0.5223 | Yes |
| 43 | TNF | tumor necrosis factor [Source:HGNC Symbol;Acc:HGNC:11892] | 36419 | -0.226 | -0.5028 | Yes |
| 44 | NLRP3 | NLR family pyrin domain containing 3 [Source:HGNC Symbol;Acc:HGNC:16400] | 36719 | -0.242 | -0.4885 | Yes |
| 45 | CASP8 | caspase 8 [Source:HGNC Symbol;Acc:HGNC:1509] | 36734 | -0.243 | -0.4667 | Yes |
| 46 | TAB3 | TGF-beta activated kinase 1 (MAP3K7) binding protein 3 [Source:HGNC Symbol;Acc:HGNC:30681] | 36893 | -0.253 | -0.4478 | Yes |
| 47 | CXCL2 | C-X-C motif chemokine ligand 2 [Source:HGNC Symbol;Acc:HGNC:4603] | 36954 | -0.257 | -0.4261 | Yes |
| 48 | NFKBIA | NFKB inhibitor alpha [Source:HGNC Symbol;Acc:HGNC:7797] | 37222 | -0.275 | -0.4080 | Yes |
| 49 | ERBIN | erbb2 interacting protein [Source:HGNC Symbol;Acc:HGNC:15842] | 37231 | -0.275 | -0.3832 | Yes |
| 50 | TAB2 | TGF-beta activated kinase 1 (MAP3K7) binding protein 2 [Source:HGNC Symbol;Acc:HGNC:17075] | 37429 | -0.290 | -0.3619 | Yes |
| 51 | BIRC2 | baculoviral IAP repeat containing 2 [Source:HGNC Symbol;Acc:HGNC:590] | 37460 | -0.293 | -0.3360 | Yes |
| 52 | BIRC3 | baculoviral IAP repeat containing 3 [Source:HGNC Symbol;Acc:HGNC:591] | 37532 | -0.299 | -0.3107 | Yes |
| 53 | CXCL8 | C-X-C motif chemokine ligand 8 [Source:HGNC Symbol;Acc:HGNC:6025] | 37687 | -0.313 | -0.2863 | Yes |
| 54 | NFKBIB | NFKB inhibitor beta [Source:HGNC Symbol;Acc:HGNC:7798] | 37987 | -0.348 | -0.2623 | Yes |
| 55 | TNFAIP3 | TNF alpha induced protein 3 [Source:HGNC Symbol;Acc:HGNC:11896] | 38049 | -0.356 | -0.2316 | Yes |
| 56 | NOD2 | nucleotide binding oligomerization domain containing 2 [Source:HGNC Symbol;Acc:HGNC:5331] | 38182 | -0.374 | -0.2011 | Yes |
| 57 | TRAF6 | TNF receptor associated factor 6 [Source:HGNC Symbol;Acc:HGNC:12036] | 38202 | -0.379 | -0.1672 | Yes |
| 58 | MAPK14 | mitogen-activated protein kinase 14 [Source:HGNC Symbol;Acc:HGNC:6876] | 38287 | -0.396 | -0.1334 | Yes |
| 59 | MAPK8 | mitogen-activated protein kinase 8 [Source:HGNC Symbol;Acc:HGNC:6881] | 38339 | -0.406 | -0.0979 | Yes |
| 60 | NFKB1 | nuclear factor kappa B subunit 1 [Source:HGNC Symbol;Acc:HGNC:7794] | 38470 | -0.443 | -0.0610 | Yes |
| 61 | RELA | "RELA proto-oncogene, NF-kB subunit [Source:HGNC Symbol;Acc:HGNC:9955]" | 38475 | -0.445 | -0.0208 | Yes |
| 62 | RIPK2 | receptor interacting serine/threonine kinase 2 [Source:HGNC Symbol;Acc:HGNC:10020] | 38525 | -0.467 | 0.0203 | Yes |
Table: GSEA details [plain text format]

  

Fig 2: KEGG\_NOD\_LIKE\_RECEPTOR\_SIGNALING\_PATHWAY      
 Blue-Pink O' Gram in the Space of the Analyzed GeneSet

  

Fig 3: KEGG\_NOD\_LIKE\_RECEPTOR\_SIGNALING\_PATHWAY: Random ES distribution      
 Gene set null distribution of ES for **KEGG\_NOD\_LIKE\_RECEPTOR\_SIGNALING\_PATHWAY**
