# Supplementary material for: Simultaneous Presentation of Multiple Myeloma and Lung Cancer: Case Report and Gene Bioinformatics Analysis
Source: Front Oncol. 2022 Jun 13;12:859735. doi: 10.3389/fonc.2022.859735 (PMC9235397; doi:10.3389/fonc.2022.859735)
Supplement: Supplementary file 1 [file DataSheet_1.zip › The bioinformatic analysis of MM and lung cancer supplementary materials/Enrichment analysis/MECR/GSEA_4.1.0/MM TCGA/KEGG.Gsea.1639040591792/KEGG_ONE_CARBON_POOL_BY_FOLATE.html]

Details for gene set KEGG\_ONE\_CARBON\_POOL\_BY\_FOLATE[GSEA]

|  || Dataset | ExpData\_collapsed\_to\_symbols.ENSG00000116353\_profile\_in\_ExpData.cls #ENSG00000116353 |
| Phenotype | ENSG00000116353\_profile\_in\_ExpData.cls#ENSG00000116353 |
| Upregulated in class | ENSG00000116353\_pos |
| GeneSet | KEGG\_ONE\_CARBON\_POOL\_BY\_FOLATE |
| Enrichment Score (ES) | 0.80212104 |
| Normalized Enrichment Score (NES) | 2.0390704 |
| Nominal p-value | 0.0 |
| FDR q-value | 3.2324402E-4 |
| FWER p-Value | 0.006 |
Table: GSEA Results Summary

  

Fig 1: Enrichment plot: KEGG\_ONE\_CARBON\_POOL\_BY\_FOLATE      
 Profile of the Running ES Score & Positions of GeneSet Members on the Rank Ordered List

  

| SYMBOL | TITLE | RANK IN GENE LIST | RANK METRIC SCORE | RUNNING ES | CORE ENRICHMENT || 1 | ATIC | 5-aminoimidazole-4-carboxamide ribonucleotide formyltransferase/IMP cyclohydrolase [Source:HGNC Symbol;Acc:HGNC:794] | 16 | 0.613 | 0.1506 | Yes |
| 2 | MTHFD1 | "methylenetetrahydrofolate dehydrogenase, cyclohydrolase and formyltetrahydrofolate synthetase 1 [Source:HGNC Symbol;Acc:HGNC:7432]" | 114 | 0.517 | 0.2756 | Yes |
| 3 | GART | "phosphoribosylglycinamide formyltransferase, phosphoribosylglycinamide synthetase, phosphoribosylaminoimidazole synthetase [Source:HGNC Symbol;Acc:HGNC:4163]" | 166 | 0.496 | 0.3965 | Yes |
| 4 | DHFR | dihydrofolate reductase [Source:HGNC Symbol;Acc:HGNC:2861] | 178 | 0.492 | 0.5175 | Yes |
| 5 | MTFMT | mitochondrial methionyl-tRNA formyltransferase [Source:HGNC Symbol;Acc:HGNC:29666] | 462 | 0.420 | 0.6138 | Yes |
| 6 | TYMS | thymidylate synthetase [Source:HGNC Symbol;Acc:HGNC:12441] | 1461 | 0.302 | 0.6629 | Yes |
| 7 | MTHFD2L | methylenetetrahydrofolate dehydrogenase (NADP+ dependent) 2 like [Source:HGNC Symbol;Acc:HGNC:31865] | 2079 | 0.260 | 0.7111 | Yes |
| 8 | MTHFS | methenyltetrahydrofolate synthetase [Source:HGNC Symbol;Acc:HGNC:7437] | 3535 | 0.192 | 0.7213 | Yes |
| 9 | SHMT1 | serine hydroxymethyltransferase 1 [Source:HGNC Symbol;Acc:HGNC:10850] | 3559 | 0.191 | 0.7678 | Yes |
| 10 | ALDH1L1 | aldehyde dehydrogenase 1 family member L1 [Source:HGNC Symbol;Acc:HGNC:3978] | 3937 | 0.178 | 0.8021 | Yes |
| 11 | MTHFD2 | "methylenetetrahydrofolate dehydrogenase (NADP+ dependent) 2, methenyltetrahydrofolate cyclohydrolase [Source:HGNC Symbol;Acc:HGNC:7434]" | 7400 | 0.103 | 0.7394 | No |
| 12 | MTHFD1L | methylenetetrahydrofolate dehydrogenase (NADP+ dependent) 1 like [Source:HGNC Symbol;Acc:HGNC:21055] | 7748 | 0.098 | 0.7548 | No |
| 13 | SHMT2 | serine hydroxymethyltransferase 2 [Source:HGNC Symbol;Acc:HGNC:10852] | 11394 | 0.057 | 0.6762 | No |
| 14 | MTR | 5-methyltetrahydrofolate-homocysteine methyltransferase [Source:HGNC Symbol;Acc:HGNC:7468] | 12858 | 0.046 | 0.6503 | No |
| 15 | FTCD | formimidoyltransferase cyclodeaminase [Source:HGNC Symbol;Acc:HGNC:3974] | 13873 | 0.039 | 0.6340 | No |
| 16 | AMT | aminomethyltransferase [Source:HGNC Symbol;Acc:HGNC:473] | 15384 | 0.030 | 0.6029 | No |
| 17 | MTHFR | methylenetetrahydrofolate reductase [Source:HGNC Symbol;Acc:HGNC:7436] | 24730 | -0.025 | 0.3713 | No |
Table: GSEA details [plain text format]

  

Fig 2: KEGG\_ONE\_CARBON\_POOL\_BY\_FOLATE      
 Blue-Pink O' Gram in the Space of the Analyzed GeneSet

  

Fig 3: KEGG\_ONE\_CARBON\_POOL\_BY\_FOLATE: Random ES distribution      
 Gene set null distribution of ES for **KEGG\_ONE\_CARBON\_POOL\_BY\_FOLATE**
