# Supplementary material for: Simultaneous Presentation of Multiple Myeloma and Lung Cancer: Case Report and Gene Bioinformatics Analysis
Source: Front Oncol. 2022 Jun 13;12:859735. doi: 10.3389/fonc.2022.859735 (PMC9235397; doi:10.3389/fonc.2022.859735)
Supplement: Supplementary file 1 [file DataSheet_1.zip › The bioinformatic analysis of MM and lung cancer supplementary materials/Enrichment analysis/MECR/GSEA_4.1.0/MM TCGA/KEGG.Gsea.1639040591792/KEGG_OXIDATIVE_PHOSPHORYLATION.html]

Details for gene set KEGG\_OXIDATIVE\_PHOSPHORYLATION[GSEA]

|  || Dataset | ExpData\_collapsed\_to\_symbols.ENSG00000116353\_profile\_in\_ExpData.cls #ENSG00000116353 |
| Phenotype | ENSG00000116353\_profile\_in\_ExpData.cls#ENSG00000116353 |
| Upregulated in class | ENSG00000116353\_pos |
| GeneSet | KEGG\_OXIDATIVE\_PHOSPHORYLATION |
| Enrichment Score (ES) | 0.6079314 |
| Normalized Enrichment Score (NES) | 2.2831643 |
| Nominal p-value | 0.0 |
| FDR q-value | 0.0 |
| FWER p-Value | 0.0 |
Table: GSEA Results Summary

  

Fig 1: Enrichment plot: KEGG\_OXIDATIVE\_PHOSPHORYLATION      
 Profile of the Running ES Score & Positions of GeneSet Members on the Rank Ordered List

  

| SYMBOL | TITLE | RANK IN GENE LIST | RANK METRIC SCORE | RUNNING ES | CORE ENRICHMENT || 1 | ATP5MC1 | ATP synthase membrane subunit c locus 1 [Source:HGNC Symbol;Acc:HGNC:841] | 70 | 0.551 | 0.0256 | Yes |
| 2 | NDUFB3 | NADH:ubiquinone oxidoreductase subunit B3 [Source:HGNC Symbol;Acc:HGNC:7698] | 252 | 0.467 | 0.0441 | Yes |
| 3 | COX15 | cytochrome c oxidase assembly homolog COX15 [Source:HGNC Symbol;Acc:HGNC:2263] | 299 | 0.456 | 0.0656 | Yes |
| 4 | SDHC | succinate dehydrogenase complex subunit C [Source:HGNC Symbol;Acc:HGNC:10682] | 333 | 0.448 | 0.0870 | Yes |
| 5 | NDUFA8 | NADH:ubiquinone oxidoreductase subunit A8 [Source:HGNC Symbol;Acc:HGNC:7692] | 358 | 0.442 | 0.1084 | Yes |
| 6 | UQCRC1 | ubiquinol-cytochrome c reductase core protein 1 [Source:HGNC Symbol;Acc:HGNC:12585] | 545 | 0.405 | 0.1237 | Yes |
| 7 | NDUFAB1 | NADH:ubiquinone oxidoreductase subunit AB1 [Source:HGNC Symbol;Acc:HGNC:7694] | 607 | 0.397 | 0.1419 | Yes |
| 8 | ATP5PD | ATP synthase peripheral stalk subunit d [Source:HGNC Symbol;Acc:HGNC:845] | 669 | 0.387 | 0.1596 | Yes |
| 9 | NDUFV3 | NADH:ubiquinone oxidoreductase subunit V3 [Source:HGNC Symbol;Acc:HGNC:7719] | 776 | 0.372 | 0.1754 | Yes |
| 10 | COX10 | cytochrome c oxidase assembly factor heme A:farnesyltransferase COX10 [Source:HGNC Symbol;Acc:HGNC:2260] | 901 | 0.354 | 0.1898 | Yes |
| 11 | NDUFS2 | NADH:ubiquinone oxidoreductase core subunit S2 [Source:HGNC Symbol;Acc:HGNC:7708] | 973 | 0.348 | 0.2052 | Yes |
| 12 | COX11 | cytochrome c oxidase copper chaperone COX11 [Source:HGNC Symbol;Acc:HGNC:2261] | 1037 | 0.340 | 0.2206 | Yes |
| 13 | NDUFB2 | NADH:ubiquinone oxidoreductase subunit B2 [Source:HGNC Symbol;Acc:HGNC:7697] | 1068 | 0.338 | 0.2366 | Yes |
| 14 | NDUFB6 | NADH:ubiquinone oxidoreductase subunit B6 [Source:HGNC Symbol;Acc:HGNC:7701] | 1121 | 0.333 | 0.2518 | Yes |
| 15 | ATP5MF | ATP synthase membrane subunit f [Source:HGNC Symbol;Acc:HGNC:848] | 1151 | 0.329 | 0.2674 | Yes |
| 16 | ATP5F1A | ATP synthase F1 subunit alpha [Source:HGNC Symbol;Acc:HGNC:823] | 1183 | 0.326 | 0.2828 | Yes |
| 17 | CYC1 | cytochrome c1 [Source:HGNC Symbol;Acc:HGNC:2579] | 1273 | 0.318 | 0.2964 | Yes |
| 18 | UQCRC2 | ubiquinol-cytochrome c reductase core protein 2 [Source:HGNC Symbol;Acc:HGNC:12586] | 1346 | 0.312 | 0.3100 | Yes |
| 19 | ATP5PF | ATP synthase peripheral stalk subunit F6 [Source:HGNC Symbol;Acc:HGNC:847] | 1369 | 0.309 | 0.3248 | Yes |
| 20 | NDUFS1 | NADH:ubiquinone oxidoreductase core subunit S1 [Source:HGNC Symbol;Acc:HGNC:7707] | 1387 | 0.307 | 0.3396 | Yes |
| 21 | NDUFS5 | NADH:ubiquinone oxidoreductase subunit S5 [Source:HGNC Symbol;Acc:HGNC:7712] | 1409 | 0.306 | 0.3543 | Yes |
| 22 | ATP5MC3 | ATP synthase membrane subunit c locus 3 [Source:HGNC Symbol;Acc:HGNC:843] | 1436 | 0.304 | 0.3687 | Yes |
| 23 | NDUFB5 | NADH:ubiquinone oxidoreductase subunit B5 [Source:HGNC Symbol;Acc:HGNC:7700] | 1567 | 0.294 | 0.3800 | Yes |
| 24 | NDUFA2 | NADH:ubiquinone oxidoreductase subunit A2 [Source:HGNC Symbol;Acc:HGNC:7685] | 1800 | 0.276 | 0.3878 | Yes |
| 25 | NDUFS8 | NADH:ubiquinone oxidoreductase core subunit S8 [Source:HGNC Symbol;Acc:HGNC:7715] | 1900 | 0.270 | 0.3987 | Yes |
| 26 | NDUFB1 | NADH:ubiquinone oxidoreductase subunit B1 [Source:HGNC Symbol;Acc:HGNC:7695] | 2076 | 0.260 | 0.4071 | Yes |
| 27 | UQCRQ | ubiquinol-cytochrome c reductase complex III subunit VII [Source:HGNC Symbol;Acc:HGNC:29594] | 2237 | 0.250 | 0.4155 | Yes |
| 28 | COX5A | cytochrome c oxidase subunit 5A [Source:HGNC Symbol;Acc:HGNC:2267] | 2292 | 0.248 | 0.4264 | Yes |
| 29 | NDUFB7 | NADH:ubiquinone oxidoreductase subunit B7 [Source:HGNC Symbol;Acc:HGNC:7702] | 2433 | 0.240 | 0.4348 | Yes |
| 30 | ATP6V1D | ATPase H+ transporting V1 subunit D [Source:HGNC Symbol;Acc:HGNC:13527] | 2437 | 0.240 | 0.4466 | Yes |
| 31 | NDUFS6 | NADH:ubiquinone oxidoreductase subunit S6 [Source:HGNC Symbol;Acc:HGNC:7713] | 2684 | 0.228 | 0.4516 | Yes |
| 32 | NDUFS7 | NADH:ubiquinone oxidoreductase core subunit S7 [Source:HGNC Symbol;Acc:HGNC:7714] | 2708 | 0.227 | 0.4623 | Yes |
| 33 | SDHB | succinate dehydrogenase complex iron sulfur subunit B [Source:HGNC Symbol;Acc:HGNC:10681] | 2784 | 0.223 | 0.4715 | Yes |
| 34 | ATP5PB | ATP synthase peripheral stalk-membrane subunit b [Source:HGNC Symbol;Acc:HGNC:840] | 3049 | 0.211 | 0.4752 | Yes |
| 35 | ATP6V1E2 | ATPase H+ transporting V1 subunit E2 [Source:HGNC Symbol;Acc:HGNC:18125] | 3263 | 0.203 | 0.4799 | Yes |
| 36 | UQCR10 | "ubiquinol-cytochrome c reductase, complex III subunit X [Source:HGNC Symbol;Acc:HGNC:30863]" | 3322 | 0.200 | 0.4883 | Yes |
| 37 | NDUFA4 | NDUFA4 mitochondrial complex associated [Source:HGNC Symbol;Acc:HGNC:7687] | 3539 | 0.192 | 0.4923 | Yes |
| 38 | COX17 | cytochrome c oxidase copper chaperone COX17 [Source:HGNC Symbol;Acc:HGNC:2264] | 3651 | 0.188 | 0.4988 | Yes |
| 39 | NDUFB9 | NADH:ubiquinone oxidoreductase subunit B9 [Source:HGNC Symbol;Acc:HGNC:7704] | 3655 | 0.188 | 0.5081 | Yes |
| 40 | NDUFB4 | NADH:ubiquinone oxidoreductase subunit B4 [Source:HGNC Symbol;Acc:HGNC:7699] | 3810 | 0.183 | 0.5132 | Yes |
| 41 | COX7A2 | cytochrome c oxidase subunit 7A2 [Source:HGNC Symbol;Acc:HGNC:2288] | 3863 | 0.181 | 0.5209 | Yes |
| 42 | COX7B | cytochrome c oxidase subunit 7B [Source:HGNC Symbol;Acc:HGNC:2291] | 3992 | 0.177 | 0.5264 | Yes |
| 43 | ATP6V1C1 | ATPase H+ transporting V1 subunit C1 [Source:HGNC Symbol;Acc:HGNC:856] | 4056 | 0.175 | 0.5335 | Yes |
| 44 | UQCRHL | ubiquinol-cytochrome c reductase hinge protein like [Source:HGNC Symbol;Acc:HGNC:51714] | 4135 | 0.173 | 0.5401 | Yes |
| 45 | COX6B1 | cytochrome c oxidase subunit 6B1 [Source:HGNC Symbol;Acc:HGNC:2280] | 4181 | 0.171 | 0.5475 | Yes |
| 46 | PPA1 | inorganic pyrophosphatase 1 [Source:HGNC Symbol;Acc:HGNC:9226] | 4401 | 0.164 | 0.5500 | Yes |
| 47 | COX5B | cytochrome c oxidase subunit 5B [Source:HGNC Symbol;Acc:HGNC:2269] | 4402 | 0.164 | 0.5582 | Yes |
| 48 | NDUFA11 | NADH:ubiquinone oxidoreductase subunit A11 [Source:HGNC Symbol;Acc:HGNC:20371] | 4483 | 0.162 | 0.5642 | Yes |
| 49 | ATP5F1C | ATP synthase F1 subunit gamma [Source:HGNC Symbol;Acc:HGNC:833] | 4521 | 0.161 | 0.5712 | Yes |
| 50 | NDUFA3 | NADH:ubiquinone oxidoreductase subunit A3 [Source:HGNC Symbol;Acc:HGNC:7686] | 4566 | 0.160 | 0.5780 | Yes |
| 51 | COX7B2 | cytochrome c oxidase subunit 7B2 [Source:HGNC Symbol;Acc:HGNC:24381] | 4647 | 0.157 | 0.5838 | Yes |
| 52 | ATP6V1G2 | ATPase H+ transporting V1 subunit G2 [Source:HGNC Symbol;Acc:HGNC:862] | 4672 | 0.157 | 0.5910 | Yes |
| 53 | UQCRH | ubiquinol-cytochrome c reductase hinge protein [Source:HGNC Symbol;Acc:HGNC:12590] | 4784 | 0.154 | 0.5958 | Yes |
| 54 | UQCR11 | "ubiquinol-cytochrome c reductase, complex III subunit XI [Source:HGNC Symbol;Acc:HGNC:30862]" | 4848 | 0.152 | 0.6018 | Yes |
| 55 | ATP5F1B | ATP synthase F1 subunit beta [Source:HGNC Symbol;Acc:HGNC:830] | 5137 | 0.145 | 0.6016 | Yes |
| 56 | NDUFA9 | NADH:ubiquinone oxidoreductase subunit A9 [Source:HGNC Symbol;Acc:HGNC:7693] | 5423 | 0.139 | 0.6012 | Yes |
| 57 | NDUFS4 | NADH:ubiquinone oxidoreductase subunit S4 [Source:HGNC Symbol;Acc:HGNC:7711] | 5588 | 0.135 | 0.6038 | Yes |
| 58 | LHPP | phospholysine phosphohistidine inorganic pyrophosphate phosphatase [Source:HGNC Symbol;Acc:HGNC:30042] | 5685 | 0.133 | 0.6079 | Yes |
| 59 | NDUFV1 | NADH:ubiquinone oxidoreductase core subunit V1 [Source:HGNC Symbol;Acc:HGNC:7716] | 6169 | 0.124 | 0.6017 | No |
| 60 | COX7C | cytochrome c oxidase subunit 7C [Source:HGNC Symbol;Acc:HGNC:2292] | 6346 | 0.121 | 0.6032 | No |
| 61 | ATP5F1E | ATP synthase F1 subunit epsilon [Source:HGNC Symbol;Acc:HGNC:838] | 6500 | 0.118 | 0.6052 | No |
| 62 | ATP5MC1P5 | ATP synthase membrane subunit c locus 1 pseudogene 5 [Source:HGNC Symbol;Acc:HGNC:39508] | 7332 | 0.104 | 0.5892 | No |
| 63 | ATP5MC2 | ATP synthase membrane subunit c locus 2 [Source:HGNC Symbol;Acc:HGNC:842] | 7382 | 0.103 | 0.5930 | No |
| 64 | MT-ND5 | mitochondrially encoded NADH:ubiquinone oxidoreductase core subunit 5 [Source:HGNC Symbol;Acc:HGNC:7461] | 7668 | 0.099 | 0.5907 | No |
| 65 | PPA2 | inorganic pyrophosphatase 2 [Source:HGNC Symbol;Acc:HGNC:28883] | 7805 | 0.097 | 0.5921 | No |
| 66 | COX6A1 | cytochrome c oxidase subunit 6A1 [Source:HGNC Symbol;Acc:HGNC:2277] | 8445 | 0.089 | 0.5802 | No |
| 67 | NDUFB10 | NADH:ubiquinone oxidoreductase subunit B10 [Source:HGNC Symbol;Acc:HGNC:7696] | 8944 | 0.083 | 0.5716 | No |
| 68 | ATP12A | ATPase H+/K+ transporting non-gastric alpha2 subunit [Source:HGNC Symbol;Acc:HGNC:13816] | 9364 | 0.078 | 0.5648 | No |
| 69 | MT-CO1 | mitochondrially encoded cytochrome c oxidase I [Source:HGNC Symbol;Acc:HGNC:7419] | 9406 | 0.077 | 0.5676 | No |
| 70 | ATP6V0E1 | ATPase H+ transporting V0 subunit e1 [Source:HGNC Symbol;Acc:HGNC:863] | 9678 | 0.074 | 0.5644 | No |
| 71 | ATP6V0A2 | ATPase H+ transporting V0 subunit a2 [Source:HGNC Symbol;Acc:HGNC:18481] | 10323 | 0.067 | 0.5513 | No |
| 72 | COX8C | cytochrome c oxidase subunit 8C [Source:HGNC Symbol;Acc:HGNC:24382] | 10480 | 0.066 | 0.5506 | No |
| 73 | COX6C | cytochrome c oxidase subunit 6C [Source:HGNC Symbol;Acc:HGNC:2285] | 11062 | 0.060 | 0.5387 | No |
| 74 | ATP5PO | ATP synthase peripheral stalk subunit OSCP [Source:HGNC Symbol;Acc:HGNC:850] | 11202 | 0.059 | 0.5381 | No |
| 75 | COX6CP3 | cytochrome c oxidase subunit 6C pseudogene 3 [Source:HGNC Symbol;Acc:HGNC:31721] | 11606 | 0.055 | 0.5306 | No |
| 76 | MT-ND6 | mitochondrially encoded NADH:ubiquinone oxidoreductase core subunit 6 [Source:HGNC Symbol;Acc:HGNC:7462] | 11927 | 0.053 | 0.5251 | No |
| 77 | ATP4B | ATPase H+/K+ transporting subunit beta [Source:HGNC Symbol;Acc:HGNC:820] | 11949 | 0.053 | 0.5271 | No |
| 78 | ATP6V1A | ATPase H+ transporting V1 subunit A [Source:HGNC Symbol;Acc:HGNC:851] | 12272 | 0.050 | 0.5214 | No |
| 79 | NDUFA5 | NADH:ubiquinone oxidoreductase subunit A5 [Source:HGNC Symbol;Acc:HGNC:7688] | 12525 | 0.048 | 0.5174 | No |
| 80 | ATP5F1D | ATP synthase F1 subunit delta [Source:HGNC Symbol;Acc:HGNC:837] | 13360 | 0.042 | 0.4982 | No |
| 81 | ATP5ME | ATP synthase membrane subunit e [Source:HGNC Symbol;Acc:HGNC:846] | 13415 | 0.042 | 0.4989 | No |
| 82 | ATP6AP1 | ATPase H+ transporting accessory protein 1 [Source:HGNC Symbol;Acc:HGNC:868] | 13439 | 0.042 | 0.5004 | No |
| 83 | NDUFB8 | NADH:ubiquinone oxidoreductase subunit B8 [Source:HGNC Symbol;Acc:HGNC:7703] | 13566 | 0.041 | 0.4992 | No |
| 84 | COX8A | cytochrome c oxidase subunit 8A [Source:HGNC Symbol;Acc:HGNC:2294] | 13791 | 0.039 | 0.4955 | No |
| 85 | SDHD | succinate dehydrogenase complex subunit D [Source:HGNC Symbol;Acc:HGNC:10683] | 14412 | 0.036 | 0.4814 | No |
| 86 | NDUFC1 | NADH:ubiquinone oxidoreductase subunit C1 [Source:HGNC Symbol;Acc:HGNC:7705] | 15304 | 0.030 | 0.4602 | No |
| 87 | UQCR10P1 | UQCR10 pseudogene 1 [Source:HGNC Symbol;Acc:HGNC:54960] | 15591 | 0.028 | 0.4543 | No |
| 88 | NDUFC2 | NADH:ubiquinone oxidoreductase subunit C2 [Source:HGNC Symbol;Acc:HGNC:7706] | 16310 | 0.024 | 0.4371 | No |
| 89 | MT-CO2 | mitochondrially encoded cytochrome c oxidase II [Source:HGNC Symbol;Acc:HGNC:7421] | 16417 | 0.023 | 0.4356 | No |
| 90 | COX6A2 | cytochrome c oxidase subunit 6A2 [Source:HGNC Symbol;Acc:HGNC:2279] | 16431 | 0.023 | 0.4364 | No |
| 91 | COX6B2 | cytochrome c oxidase subunit 6B2 [Source:HGNC Symbol;Acc:HGNC:24380] | 16821 | 0.021 | 0.4275 | No |
| 92 | UQCRB | ubiquinol-cytochrome c reductase binding protein [Source:HGNC Symbol;Acc:HGNC:12582] | 16826 | 0.021 | 0.4285 | No |
| 93 | ATP6V1B1 | ATPase H+ transporting V1 subunit B1 [Source:HGNC Symbol;Acc:HGNC:853] | 17536 | 0.017 | 0.4112 | No |
| 94 | NDUFA6 | NADH:ubiquinone oxidoreductase subunit A6 [Source:HGNC Symbol;Acc:HGNC:7690] | 19225 | 0.008 | 0.3685 | No |
| 95 | ATP6V1G3 | ATPase H+ transporting V1 subunit G3 [Source:HGNC Symbol;Acc:HGNC:18265] | 19296 | 0.007 | 0.3671 | No |
| 96 | ATP6V1F | ATPase H+ transporting V1 subunit F [Source:HGNC Symbol;Acc:HGNC:16832] | 21026 | -0.002 | 0.3231 | No |
| 97 | UQCRFS1 | "ubiquinol-cytochrome c reductase, Rieske iron-sulfur polypeptide 1 [Source:HGNC Symbol;Acc:HGNC:12587]" | 22359 | -0.010 | 0.2896 | No |
| 98 | NDUFA1 | NADH:ubiquinone oxidoreductase subunit A1 [Source:HGNC Symbol;Acc:HGNC:7683] | 23269 | -0.015 | 0.2672 | No |
| 99 | MT-ATP8 | mitochondrially encoded ATP synthase membrane subunit 8 [Source:HGNC Symbol;Acc:HGNC:7415] | 23281 | -0.015 | 0.2677 | No |
| 100 | ATP6V1C2 | ATPase H+ transporting V1 subunit C2 [Source:HGNC Symbol;Acc:HGNC:18264] | 23445 | -0.017 | 0.2643 | No |
| 101 | NDUFS3 | NADH:ubiquinone oxidoreductase core subunit S3 [Source:HGNC Symbol;Acc:HGNC:7710] | 23954 | -0.020 | 0.2524 | No |
| 102 | MT-ND4L | mitochondrially encoded NADH:ubiquinone oxidoreductase core subunit 4L [Source:HGNC Symbol;Acc:HGNC:7460] | 24121 | -0.021 | 0.2492 | No |
| 103 | SDHA | succinate dehydrogenase complex flavoprotein subunit A [Source:HGNC Symbol;Acc:HGNC:10680] | 24822 | -0.025 | 0.2326 | No |
| 104 | ATP6V0D2 | ATPase H+ transporting V0 subunit d2 [Source:HGNC Symbol;Acc:HGNC:18266] | 25308 | -0.028 | 0.2216 | No |
| 105 | MT-ND4 | mitochondrially encoded NADH:ubiquinone oxidoreductase core subunit 4 [Source:HGNC Symbol;Acc:HGNC:7459] | 26556 | -0.038 | 0.1917 | No |
| 106 | ATP6V0A4 | ATPase H+ transporting V0 subunit a4 [Source:HGNC Symbol;Acc:HGNC:866] | 26863 | -0.040 | 0.1858 | No |
| 107 | NDUFA7 | NADH:ubiquinone oxidoreductase subunit A7 [Source:HGNC Symbol;Acc:HGNC:7691] | 27225 | -0.043 | 0.1788 | No |
| 108 | ATP6V1E1 | ATPase H+ transporting V1 subunit E1 [Source:HGNC Symbol;Acc:HGNC:857] | 27410 | -0.045 | 0.1763 | No |
| 109 | NDUFA4L2 | NDUFA4 mitochondrial complex associated like 2 [Source:HGNC Symbol;Acc:HGNC:29836] | 27785 | -0.048 | 0.1691 | No |
| 110 | ATP6V1H | ATPase H+ transporting V1 subunit H [Source:HGNC Symbol;Acc:HGNC:18303] | 27855 | -0.049 | 0.1698 | No |
| 111 | ATP6V0E2 | ATPase H+ transporting V0 subunit e2 [Source:HGNC Symbol;Acc:HGNC:21723] | 28277 | -0.053 | 0.1617 | No |
| 112 | MT-ATP6 | mitochondrially encoded ATP synthase membrane subunit 6 [Source:HGNC Symbol;Acc:HGNC:7414] | 28861 | -0.059 | 0.1497 | No |
| 113 | ATP4A | ATPase H+/K+ transporting subunit alpha [Source:HGNC Symbol;Acc:HGNC:819] | 28874 | -0.059 | 0.1523 | No |
| 114 | ATP6V0B | ATPase H+ transporting V0 subunit b [Source:HGNC Symbol;Acc:HGNC:861] | 29995 | -0.072 | 0.1273 | No |
| 115 | NDUFA10 | NADH:ubiquinone oxidoreductase subunit A10 [Source:HGNC Symbol;Acc:HGNC:7684] | 30079 | -0.073 | 0.1289 | No |
| 116 | COX7A2L | cytochrome c oxidase subunit 7A2 like [Source:HGNC Symbol;Acc:HGNC:2289] | 30323 | -0.076 | 0.1265 | No |
| 117 | MT-ND2 | mitochondrially encoded NADH:ubiquinone oxidoreductase core subunit 2 [Source:HGNC Symbol;Acc:HGNC:7456] | 30447 | -0.078 | 0.1272 | No |
| 118 | ATP6V1G1 | ATPase H+ transporting V1 subunit G1 [Source:HGNC Symbol;Acc:HGNC:864] | 30467 | -0.078 | 0.1305 | No |
| 119 | TCIRG1 | "T cell immune regulator 1, ATPase H+ transporting V0 subunit a3 [Source:HGNC Symbol;Acc:HGNC:11647]" | 30556 | -0.079 | 0.1322 | No |
| 120 | ATP5MG | ATP synthase membrane subunit g [Source:HGNC Symbol;Acc:HGNC:14247] | 30642 | -0.080 | 0.1340 | No |
| 121 | MT-CYB | mitochondrially encoded cytochrome b [Source:HGNC Symbol;Acc:HGNC:7427] | 30698 | -0.081 | 0.1366 | No |
| 122 | MT-CO3 | mitochondrially encoded cytochrome c oxidase III [Source:HGNC Symbol;Acc:HGNC:7422] | 30889 | -0.083 | 0.1359 | No |
| 123 | MT-ND1 | mitochondrially encoded NADH:ubiquinone oxidoreductase core subunit 1 [Source:HGNC Symbol;Acc:HGNC:7455] | 31518 | -0.092 | 0.1244 | No |
| 124 | ATP6V1B2 | ATPase H+ transporting V1 subunit B2 [Source:HGNC Symbol;Acc:HGNC:854] | 32531 | -0.110 | 0.1041 | No |
| 125 | COX7A1 | cytochrome c oxidase subunit 7A1 [Source:HGNC Symbol;Acc:HGNC:2287] | 32963 | -0.118 | 0.0990 | No |
| 126 | ATP6V0A1 | ATPase H+ transporting V0 subunit a1 [Source:HGNC Symbol;Acc:HGNC:865] | 33106 | -0.121 | 0.1013 | No |
| 127 | MT-ND3 | mitochondrially encoded NADH:ubiquinone oxidoreductase core subunit 3 [Source:HGNC Symbol;Acc:HGNC:7458] | 33144 | -0.122 | 0.1064 | No |
| 128 | COX4I2 | cytochrome c oxidase subunit 4I2 [Source:HGNC Symbol;Acc:HGNC:16232] | 33693 | -0.134 | 0.0991 | No |
| 129 | COX4I1 | cytochrome c oxidase subunit 4I1 [Source:HGNC Symbol;Acc:HGNC:2265] | 35680 | -0.195 | 0.0581 | No |
| 130 | ATP6V0D1 | ATPase H+ transporting V0 subunit d1 [Source:HGNC Symbol;Acc:HGNC:13724] | 36271 | -0.218 | 0.0539 | No |
| 131 | NDUFV2 | NADH:ubiquinone oxidoreductase core subunit V2 [Source:HGNC Symbol;Acc:HGNC:7717] | 36361 | -0.223 | 0.0627 | No |
| 132 | ATP6V0C | ATPase H+ transporting V0 subunit c [Source:HGNC Symbol;Acc:HGNC:855] | 36977 | -0.258 | 0.0599 | No |
Table: GSEA details [plain text format]

  

Fig 2: KEGG\_OXIDATIVE\_PHOSPHORYLATION      
 Blue-Pink O' Gram in the Space of the Analyzed GeneSet

  

Fig 3: KEGG\_OXIDATIVE\_PHOSPHORYLATION: Random ES distribution      
 Gene set null distribution of ES for **KEGG\_OXIDATIVE\_PHOSPHORYLATION**
