# Supplementary material for: Simultaneous Presentation of Multiple Myeloma and Lung Cancer: Case Report and Gene Bioinformatics Analysis
Source: Front Oncol. 2022 Jun 13;12:859735. doi: 10.3389/fonc.2022.859735 (PMC9235397; doi:10.3389/fonc.2022.859735)
Supplement: Supplementary file 1 [file DataSheet_1.zip › The bioinformatic analysis of MM and lung cancer supplementary materials/Enrichment analysis/MECR/GSEA_4.1.0/MM TCGA/KEGG.Gsea.1639040591792/KEGG_PARKINSONS_DISEASE.html]

Details for gene set KEGG\_PARKINSONS\_DISEASE[GSEA]

|  || Dataset | ExpData\_collapsed\_to\_symbols.ENSG00000116353\_profile\_in\_ExpData.cls #ENSG00000116353 |
| Phenotype | ENSG00000116353\_profile\_in\_ExpData.cls#ENSG00000116353 |
| Upregulated in class | ENSG00000116353\_pos |
| GeneSet | KEGG\_PARKINSONS\_DISEASE |
| Enrichment Score (ES) | 0.58273053 |
| Normalized Enrichment Score (NES) | 2.1743488 |
| Nominal p-value | 0.0 |
| FDR q-value | 0.0 |
| FWER p-Value | 0.0 |
Table: GSEA Results Summary

  

Fig 1: Enrichment plot: KEGG\_PARKINSONS\_DISEASE      
 Profile of the Running ES Score & Positions of GeneSet Members on the Rank Ordered List

  

| SYMBOL | TITLE | RANK IN GENE LIST | RANK METRIC SCORE | RUNNING ES | CORE ENRICHMENT || 1 | ATP5MC1 | ATP synthase membrane subunit c locus 1 [Source:HGNC Symbol;Acc:HGNC:841] | 70 | 0.551 | 0.0250 | Yes |
| 2 | NDUFB3 | NADH:ubiquinone oxidoreductase subunit B3 [Source:HGNC Symbol;Acc:HGNC:7698] | 252 | 0.467 | 0.0430 | Yes |
| 3 | SDHC | succinate dehydrogenase complex subunit C [Source:HGNC Symbol;Acc:HGNC:10682] | 333 | 0.448 | 0.0627 | Yes |
| 4 | NDUFA8 | NADH:ubiquinone oxidoreductase subunit A8 [Source:HGNC Symbol;Acc:HGNC:7692] | 358 | 0.442 | 0.0836 | Yes |
| 5 | PARK7 | Parkinsonism associated deglycase [Source:HGNC Symbol;Acc:HGNC:16369] | 536 | 0.406 | 0.0988 | Yes |
| 6 | UQCRC1 | ubiquinol-cytochrome c reductase core protein 1 [Source:HGNC Symbol;Acc:HGNC:12585] | 545 | 0.405 | 0.1183 | Yes |
| 7 | CYCS | "cytochrome c, somatic [Source:HGNC Symbol;Acc:HGNC:19986]" | 580 | 0.401 | 0.1369 | Yes |
| 8 | NDUFAB1 | NADH:ubiquinone oxidoreductase subunit AB1 [Source:HGNC Symbol;Acc:HGNC:7694] | 607 | 0.397 | 0.1555 | Yes |
| 9 | ATP5PD | ATP synthase peripheral stalk subunit d [Source:HGNC Symbol;Acc:HGNC:845] | 669 | 0.387 | 0.1728 | Yes |
| 10 | NDUFV3 | NADH:ubiquinone oxidoreductase subunit V3 [Source:HGNC Symbol;Acc:HGNC:7719] | 776 | 0.372 | 0.1882 | Yes |
| 11 | NDUFS2 | NADH:ubiquinone oxidoreductase core subunit S2 [Source:HGNC Symbol;Acc:HGNC:7708] | 973 | 0.348 | 0.2000 | Yes |
| 12 | CASP9 | caspase 9 [Source:HGNC Symbol;Acc:HGNC:1511] | 1063 | 0.338 | 0.2142 | Yes |
| 13 | NDUFB2 | NADH:ubiquinone oxidoreductase subunit B2 [Source:HGNC Symbol;Acc:HGNC:7697] | 1068 | 0.338 | 0.2305 | Yes |
| 14 | NDUFB6 | NADH:ubiquinone oxidoreductase subunit B6 [Source:HGNC Symbol;Acc:HGNC:7701] | 1121 | 0.333 | 0.2454 | Yes |
| 15 | ATP5F1A | ATP synthase F1 subunit alpha [Source:HGNC Symbol;Acc:HGNC:823] | 1183 | 0.326 | 0.2596 | Yes |
| 16 | CYC1 | cytochrome c1 [Source:HGNC Symbol;Acc:HGNC:2579] | 1273 | 0.318 | 0.2728 | Yes |
| 17 | UQCRC2 | ubiquinol-cytochrome c reductase core protein 2 [Source:HGNC Symbol;Acc:HGNC:12586] | 1346 | 0.312 | 0.2861 | Yes |
| 18 | ATP5PF | ATP synthase peripheral stalk subunit F6 [Source:HGNC Symbol;Acc:HGNC:847] | 1369 | 0.309 | 0.3006 | Yes |
| 19 | NDUFS1 | NADH:ubiquinone oxidoreductase core subunit S1 [Source:HGNC Symbol;Acc:HGNC:7707] | 1387 | 0.307 | 0.3151 | Yes |
| 20 | NDUFS5 | NADH:ubiquinone oxidoreductase subunit S5 [Source:HGNC Symbol;Acc:HGNC:7712] | 1409 | 0.306 | 0.3294 | Yes |
| 21 | SLC25A4 | solute carrier family 25 member 4 [Source:HGNC Symbol;Acc:HGNC:10990] | 1423 | 0.305 | 0.3439 | Yes |
| 22 | ATP5MC3 | ATP synthase membrane subunit c locus 3 [Source:HGNC Symbol;Acc:HGNC:843] | 1436 | 0.304 | 0.3583 | Yes |
| 23 | NDUFB5 | NADH:ubiquinone oxidoreductase subunit B5 [Source:HGNC Symbol;Acc:HGNC:7700] | 1567 | 0.294 | 0.3693 | Yes |
| 24 | UBA1 | ubiquitin like modifier activating enzyme 1 [Source:HGNC Symbol;Acc:HGNC:12469] | 1767 | 0.278 | 0.3777 | Yes |
| 25 | NDUFA2 | NADH:ubiquinone oxidoreductase subunit A2 [Source:HGNC Symbol;Acc:HGNC:7685] | 1800 | 0.276 | 0.3903 | Yes |
| 26 | NDUFS8 | NADH:ubiquinone oxidoreductase core subunit S8 [Source:HGNC Symbol;Acc:HGNC:7715] | 1900 | 0.270 | 0.4009 | Yes |
| 27 | NDUFB1 | NADH:ubiquinone oxidoreductase subunit B1 [Source:HGNC Symbol;Acc:HGNC:7695] | 2076 | 0.260 | 0.4090 | Yes |
| 28 | UBE2G2 | ubiquitin conjugating enzyme E2 G2 [Source:HGNC Symbol;Acc:HGNC:12483] | 2134 | 0.256 | 0.4200 | Yes |
| 29 | UQCRQ | ubiquinol-cytochrome c reductase complex III subunit VII [Source:HGNC Symbol;Acc:HGNC:29594] | 2237 | 0.250 | 0.4296 | Yes |
| 30 | COX5A | cytochrome c oxidase subunit 5A [Source:HGNC Symbol;Acc:HGNC:2267] | 2292 | 0.248 | 0.4402 | Yes |
| 31 | NDUFB7 | NADH:ubiquinone oxidoreductase subunit B7 [Source:HGNC Symbol;Acc:HGNC:7702] | 2433 | 0.240 | 0.4483 | Yes |
| 32 | NDUFS6 | NADH:ubiquinone oxidoreductase subunit S6 [Source:HGNC Symbol;Acc:HGNC:7713] | 2684 | 0.228 | 0.4530 | Yes |
| 33 | NDUFS7 | NADH:ubiquinone oxidoreductase core subunit S7 [Source:HGNC Symbol;Acc:HGNC:7714] | 2708 | 0.227 | 0.4634 | Yes |
| 34 | SDHB | succinate dehydrogenase complex iron sulfur subunit B [Source:HGNC Symbol;Acc:HGNC:10681] | 2784 | 0.223 | 0.4724 | Yes |
| 35 | ATP5PB | ATP synthase peripheral stalk-membrane subunit b [Source:HGNC Symbol;Acc:HGNC:840] | 3049 | 0.211 | 0.4759 | Yes |
| 36 | VDAC1 | voltage dependent anion channel 1 [Source:HGNC Symbol;Acc:HGNC:12669] | 3273 | 0.202 | 0.4800 | Yes |
| 37 | UQCR10 | "ubiquinol-cytochrome c reductase, complex III subunit X [Source:HGNC Symbol;Acc:HGNC:30863]" | 3322 | 0.200 | 0.4885 | Yes |
| 38 | NDUFA4 | NDUFA4 mitochondrial complex associated [Source:HGNC Symbol;Acc:HGNC:7687] | 3539 | 0.192 | 0.4923 | Yes |
| 39 | NDUFB9 | NADH:ubiquinone oxidoreductase subunit B9 [Source:HGNC Symbol;Acc:HGNC:7704] | 3655 | 0.188 | 0.4985 | Yes |
| 40 | NDUFB4 | NADH:ubiquinone oxidoreductase subunit B4 [Source:HGNC Symbol;Acc:HGNC:7699] | 3810 | 0.183 | 0.5034 | Yes |
| 41 | COX7A2 | cytochrome c oxidase subunit 7A2 [Source:HGNC Symbol;Acc:HGNC:2288] | 3863 | 0.181 | 0.5109 | Yes |
| 42 | COX7B | cytochrome c oxidase subunit 7B [Source:HGNC Symbol;Acc:HGNC:2291] | 3992 | 0.177 | 0.5162 | Yes |
| 43 | SNCAIP | synuclein alpha interacting protein [Source:HGNC Symbol;Acc:HGNC:11139] | 4080 | 0.174 | 0.5225 | Yes |
| 44 | UQCRHL | ubiquinol-cytochrome c reductase hinge protein like [Source:HGNC Symbol;Acc:HGNC:51714] | 4135 | 0.173 | 0.5295 | Yes |
| 45 | COX6B1 | cytochrome c oxidase subunit 6B1 [Source:HGNC Symbol;Acc:HGNC:2280] | 4181 | 0.171 | 0.5367 | Yes |
| 46 | COX5B | cytochrome c oxidase subunit 5B [Source:HGNC Symbol;Acc:HGNC:2269] | 4402 | 0.164 | 0.5390 | Yes |
| 47 | ATP5F1C | ATP synthase F1 subunit gamma [Source:HGNC Symbol;Acc:HGNC:833] | 4521 | 0.161 | 0.5438 | Yes |
| 48 | NDUFA3 | NADH:ubiquinone oxidoreductase subunit A3 [Source:HGNC Symbol;Acc:HGNC:7686] | 4566 | 0.160 | 0.5505 | Yes |
| 49 | COX7B2 | cytochrome c oxidase subunit 7B2 [Source:HGNC Symbol;Acc:HGNC:24381] | 4647 | 0.157 | 0.5561 | Yes |
| 50 | VDAC3 | voltage dependent anion channel 3 [Source:HGNC Symbol;Acc:HGNC:12674] | 4648 | 0.157 | 0.5637 | Yes |
| 51 | UBE2L6 | ubiquitin conjugating enzyme E2 L6 [Source:HGNC Symbol;Acc:HGNC:12490] | 4654 | 0.157 | 0.5712 | Yes |
| 52 | UQCRH | ubiquinol-cytochrome c reductase hinge protein [Source:HGNC Symbol;Acc:HGNC:12590] | 4784 | 0.154 | 0.5754 | Yes |
| 53 | UQCR11 | "ubiquinol-cytochrome c reductase, complex III subunit XI [Source:HGNC Symbol;Acc:HGNC:30862]" | 4848 | 0.152 | 0.5812 | Yes |
| 54 | ATP5F1B | ATP synthase F1 subunit beta [Source:HGNC Symbol;Acc:HGNC:830] | 5137 | 0.145 | 0.5809 | Yes |
| 55 | NDUFA9 | NADH:ubiquinone oxidoreductase subunit A9 [Source:HGNC Symbol;Acc:HGNC:7693] | 5423 | 0.139 | 0.5803 | Yes |
| 56 | NDUFS4 | NADH:ubiquinone oxidoreductase subunit S4 [Source:HGNC Symbol;Acc:HGNC:7711] | 5588 | 0.135 | 0.5827 | Yes |
| 57 | NDUFV1 | NADH:ubiquinone oxidoreductase core subunit V1 [Source:HGNC Symbol;Acc:HGNC:7716] | 6169 | 0.124 | 0.5739 | No |
| 58 | COX7C | cytochrome c oxidase subunit 7C [Source:HGNC Symbol;Acc:HGNC:2292] | 6346 | 0.121 | 0.5753 | No |
| 59 | ATP5F1E | ATP synthase F1 subunit epsilon [Source:HGNC Symbol;Acc:HGNC:838] | 6500 | 0.118 | 0.5771 | No |
| 60 | SLC25A31 | solute carrier family 25 member 31 [Source:HGNC Symbol;Acc:HGNC:25319] | 6795 | 0.113 | 0.5751 | No |
| 61 | ATP5MC1P5 | ATP synthase membrane subunit c locus 1 pseudogene 5 [Source:HGNC Symbol;Acc:HGNC:39508] | 7332 | 0.104 | 0.5665 | No |
| 62 | ATP5MC2 | ATP synthase membrane subunit c locus 2 [Source:HGNC Symbol;Acc:HGNC:842] | 7382 | 0.103 | 0.5702 | No |
| 63 | UBE2J2 | ubiquitin conjugating enzyme E2 J2 [Source:HGNC Symbol;Acc:HGNC:19268] | 7385 | 0.103 | 0.5752 | No |
| 64 | MT-ND5 | mitochondrially encoded NADH:ubiquinone oxidoreductase core subunit 5 [Source:HGNC Symbol;Acc:HGNC:7461] | 7668 | 0.099 | 0.5728 | No |
| 65 | UCHL1 | ubiquitin C-terminal hydrolase L1 [Source:HGNC Symbol;Acc:HGNC:12513] | 8081 | 0.094 | 0.5669 | No |
| 66 | COX6A1 | cytochrome c oxidase subunit 6A1 [Source:HGNC Symbol;Acc:HGNC:2277] | 8445 | 0.089 | 0.5619 | No |
| 67 | SLC25A5 | solute carrier family 25 member 5 [Source:HGNC Symbol;Acc:HGNC:10991] | 8607 | 0.087 | 0.5621 | No |
| 68 | NDUFB10 | NADH:ubiquinone oxidoreductase subunit B10 [Source:HGNC Symbol;Acc:HGNC:7696] | 8944 | 0.083 | 0.5575 | No |
| 69 | MT-CO1 | mitochondrially encoded cytochrome c oxidase I [Source:HGNC Symbol;Acc:HGNC:7419] | 9406 | 0.077 | 0.5495 | No |
| 70 | GPR37 | G protein-coupled receptor 37 [Source:HGNC Symbol;Acc:HGNC:4494] | 9624 | 0.075 | 0.5476 | No |
| 71 | VDAC2P5 | VDAC2 pseudogene 5 [Source:HGNC Symbol;Acc:HGNC:54753] | 10072 | 0.070 | 0.5396 | No |
| 72 | COX8C | cytochrome c oxidase subunit 8C [Source:HGNC Symbol;Acc:HGNC:24382] | 10480 | 0.066 | 0.5324 | No |
| 73 | COX6C | cytochrome c oxidase subunit 6C [Source:HGNC Symbol;Acc:HGNC:2285] | 11062 | 0.060 | 0.5205 | No |
| 74 | ATP5PO | ATP synthase peripheral stalk subunit OSCP [Source:HGNC Symbol;Acc:HGNC:850] | 11202 | 0.059 | 0.5199 | No |
| 75 | COX6CP3 | cytochrome c oxidase subunit 6C pseudogene 3 [Source:HGNC Symbol;Acc:HGNC:31721] | 11606 | 0.055 | 0.5123 | No |
| 76 | UBE2J1 | ubiquitin conjugating enzyme E2 J1 [Source:HGNC Symbol;Acc:HGNC:17598] | 11661 | 0.055 | 0.5136 | No |
| 77 | MT-ND6 | mitochondrially encoded NADH:ubiquinone oxidoreductase core subunit 6 [Source:HGNC Symbol;Acc:HGNC:7462] | 11927 | 0.053 | 0.5094 | No |
| 78 | NDUFA5 | NADH:ubiquinone oxidoreductase subunit A5 [Source:HGNC Symbol;Acc:HGNC:7688] | 12525 | 0.048 | 0.4965 | No |
| 79 | ATP5F1D | ATP synthase F1 subunit delta [Source:HGNC Symbol;Acc:HGNC:837] | 13360 | 0.042 | 0.4773 | No |
| 80 | NDUFB8 | NADH:ubiquinone oxidoreductase subunit B8 [Source:HGNC Symbol;Acc:HGNC:7703] | 13566 | 0.041 | 0.4740 | No |
| 81 | COX8A | cytochrome c oxidase subunit 8A [Source:HGNC Symbol;Acc:HGNC:2294] | 13791 | 0.039 | 0.4702 | No |
| 82 | SDHD | succinate dehydrogenase complex subunit D [Source:HGNC Symbol;Acc:HGNC:10683] | 14412 | 0.036 | 0.4561 | No |
| 83 | SLC6A3 | solute carrier family 6 member 3 [Source:HGNC Symbol;Acc:HGNC:11049] | 15015 | 0.032 | 0.4423 | No |
| 84 | NDUFC1 | NADH:ubiquinone oxidoreductase subunit C1 [Source:HGNC Symbol;Acc:HGNC:7705] | 15304 | 0.030 | 0.4364 | No |
| 85 | UQCR10P1 | UQCR10 pseudogene 1 [Source:HGNC Symbol;Acc:HGNC:54960] | 15591 | 0.028 | 0.4305 | No |
| 86 | NDUFC2 | NADH:ubiquinone oxidoreductase subunit C2 [Source:HGNC Symbol;Acc:HGNC:7706] | 16310 | 0.024 | 0.4134 | No |
| 87 | MT-CO2 | mitochondrially encoded cytochrome c oxidase II [Source:HGNC Symbol;Acc:HGNC:7421] | 16417 | 0.023 | 0.4118 | No |
| 88 | COX6A2 | cytochrome c oxidase subunit 6A2 [Source:HGNC Symbol;Acc:HGNC:2279] | 16431 | 0.023 | 0.4126 | No |
| 89 | COX6B2 | cytochrome c oxidase subunit 6B2 [Source:HGNC Symbol;Acc:HGNC:24380] | 16821 | 0.021 | 0.4037 | No |
| 90 | UQCRB | ubiquinol-cytochrome c reductase binding protein [Source:HGNC Symbol;Acc:HGNC:12582] | 16826 | 0.021 | 0.4046 | No |
| 91 | NDUFA6 | NADH:ubiquinone oxidoreductase subunit A6 [Source:HGNC Symbol;Acc:HGNC:7690] | 19225 | 0.008 | 0.3438 | No |
| 92 | PRKN | parkin RBR E3 ubiquitin protein ligase [Source:HGNC Symbol;Acc:HGNC:8607] | 20533 | 0.001 | 0.3105 | No |
| 93 | UQCRFS1 | "ubiquinol-cytochrome c reductase, Rieske iron-sulfur polypeptide 1 [Source:HGNC Symbol;Acc:HGNC:12587]" | 22359 | -0.010 | 0.2644 | No |
| 94 | NDUFA1 | NADH:ubiquinone oxidoreductase subunit A1 [Source:HGNC Symbol;Acc:HGNC:7683] | 23269 | -0.015 | 0.2419 | No |
| 95 | MT-ATP8 | mitochondrially encoded ATP synthase membrane subunit 8 [Source:HGNC Symbol;Acc:HGNC:7415] | 23281 | -0.015 | 0.2424 | No |
| 96 | TH | tyrosine hydroxylase [Source:HGNC Symbol;Acc:HGNC:11782] | 23631 | -0.018 | 0.2344 | No |
| 97 | SNCA | synuclein alpha [Source:HGNC Symbol;Acc:HGNC:11138] | 23923 | -0.020 | 0.2279 | No |
| 98 | NDUFS3 | NADH:ubiquinone oxidoreductase core subunit S3 [Source:HGNC Symbol;Acc:HGNC:7710] | 23954 | -0.020 | 0.2281 | No |
| 99 | MT-ND4L | mitochondrially encoded NADH:ubiquinone oxidoreductase core subunit 4L [Source:HGNC Symbol;Acc:HGNC:7460] | 24121 | -0.021 | 0.2248 | No |
| 100 | SDHA | succinate dehydrogenase complex flavoprotein subunit A [Source:HGNC Symbol;Acc:HGNC:10680] | 24822 | -0.025 | 0.2082 | No |
| 101 | PPID | peptidylprolyl isomerase D [Source:HGNC Symbol;Acc:HGNC:9257] | 25907 | -0.033 | 0.1822 | No |
| 102 | MT-ND4 | mitochondrially encoded NADH:ubiquinone oxidoreductase core subunit 4 [Source:HGNC Symbol;Acc:HGNC:7459] | 26556 | -0.038 | 0.1675 | No |
| 103 | UBE2L3 | ubiquitin conjugating enzyme E2 L3 [Source:HGNC Symbol;Acc:HGNC:12488] | 26938 | -0.041 | 0.1597 | No |
| 104 | NDUFA7 | NADH:ubiquinone oxidoreductase subunit A7 [Source:HGNC Symbol;Acc:HGNC:7691] | 27225 | -0.043 | 0.1545 | No |
| 105 | SLC18A1 | solute carrier family 18 member A1 [Source:HGNC Symbol;Acc:HGNC:10934] | 27309 | -0.044 | 0.1545 | No |
| 106 | HTRA2 | HtrA serine peptidase 2 [Source:HGNC Symbol;Acc:HGNC:14348] | 27538 | -0.046 | 0.1509 | No |
| 107 | SLC25A6 | solute carrier family 25 member 6 [Source:HGNC Symbol;Acc:HGNC:10992] | 27783 | -0.048 | 0.1470 | No |
| 108 | NDUFA4L2 | NDUFA4 mitochondrial complex associated like 2 [Source:HGNC Symbol;Acc:HGNC:29836] | 27785 | -0.048 | 0.1494 | No |
| 109 | MT-ATP6 | mitochondrially encoded ATP synthase membrane subunit 6 [Source:HGNC Symbol;Acc:HGNC:7414] | 28861 | -0.059 | 0.1248 | No |
| 110 | UBB | ubiquitin B [Source:HGNC Symbol;Acc:HGNC:12463] | 30024 | -0.072 | 0.0987 | No |
| 111 | NDUFA10 | NADH:ubiquinone oxidoreductase subunit A10 [Source:HGNC Symbol;Acc:HGNC:7684] | 30079 | -0.073 | 0.1008 | No |
| 112 | COX7A2L | cytochrome c oxidase subunit 7A2 like [Source:HGNC Symbol;Acc:HGNC:2289] | 30323 | -0.076 | 0.0983 | No |
| 113 | MT-ND2 | mitochondrially encoded NADH:ubiquinone oxidoreductase core subunit 2 [Source:HGNC Symbol;Acc:HGNC:7456] | 30447 | -0.078 | 0.0990 | No |
| 114 | MT-CYB | mitochondrially encoded cytochrome b [Source:HGNC Symbol;Acc:HGNC:7427] | 30698 | -0.081 | 0.0965 | No |
| 115 | VDAC2 | voltage dependent anion channel 2 [Source:HGNC Symbol;Acc:HGNC:12672] | 30866 | -0.083 | 0.0963 | No |
| 116 | MT-CO3 | mitochondrially encoded cytochrome c oxidase III [Source:HGNC Symbol;Acc:HGNC:7422] | 30889 | -0.083 | 0.0998 | No |
| 117 | MT-ND1 | mitochondrially encoded NADH:ubiquinone oxidoreductase core subunit 1 [Source:HGNC Symbol;Acc:HGNC:7455] | 31518 | -0.092 | 0.0882 | No |
| 118 | SEPTIN5 | septin 5 [Source:HGNC Symbol;Acc:HGNC:9164] | 32924 | -0.118 | 0.0581 | No |
| 119 | COX7A1 | cytochrome c oxidase subunit 7A1 [Source:HGNC Symbol;Acc:HGNC:2287] | 32963 | -0.118 | 0.0628 | No |
| 120 | MT-ND3 | mitochondrially encoded NADH:ubiquinone oxidoreductase core subunit 3 [Source:HGNC Symbol;Acc:HGNC:7458] | 33144 | -0.122 | 0.0642 | No |
| 121 | COX4I2 | cytochrome c oxidase subunit 4I2 [Source:HGNC Symbol;Acc:HGNC:16232] | 33693 | -0.134 | 0.0567 | No |
| 122 | UBA7 | ubiquitin like modifier activating enzyme 7 [Source:HGNC Symbol;Acc:HGNC:12471] | 33823 | -0.137 | 0.0600 | No |
| 123 | CASP3 | caspase 3 [Source:HGNC Symbol;Acc:HGNC:1504] | 34816 | -0.165 | 0.0427 | No |
| 124 | SLC18A2 | solute carrier family 18 member A2 [Source:HGNC Symbol;Acc:HGNC:10935] | 35361 | -0.183 | 0.0378 | No |
| 125 | UBE2G1 | ubiquitin conjugating enzyme E2 G1 [Source:HGNC Symbol;Acc:HGNC:12482] | 35493 | -0.188 | 0.0436 | No |
| 126 | COX4I1 | cytochrome c oxidase subunit 4I1 [Source:HGNC Symbol;Acc:HGNC:2265] | 35680 | -0.195 | 0.0483 | No |
| 127 | APAF1 | apoptotic peptidase activating factor 1 [Source:HGNC Symbol;Acc:HGNC:576] | 36307 | -0.220 | 0.0430 | No |
| 128 | NDUFV2 | NADH:ubiquinone oxidoreductase core subunit V2 [Source:HGNC Symbol;Acc:HGNC:7717] | 36361 | -0.223 | 0.0525 | No |
| 129 | PINK1 | PTEN induced kinase 1 [Source:HGNC Symbol;Acc:HGNC:14581] | 36449 | -0.228 | 0.0613 | No |
| 130 | LRRK2 | leucine rich repeat kinase 2 [Source:HGNC Symbol;Acc:HGNC:18618] | 36792 | -0.246 | 0.0646 | No |
Table: GSEA details [plain text format]

  

Fig 2: KEGG\_PARKINSONS\_DISEASE      
 Blue-Pink O' Gram in the Space of the Analyzed GeneSet

  

Fig 3: KEGG\_PARKINSONS\_DISEASE: Random ES distribution      
 Gene set null distribution of ES for **KEGG\_PARKINSONS\_DISEASE**
