# Supplementary material for: Simultaneous Presentation of Multiple Myeloma and Lung Cancer: Case Report and Gene Bioinformatics Analysis
Source: Front Oncol. 2022 Jun 13;12:859735. doi: 10.3389/fonc.2022.859735 (PMC9235397; doi:10.3389/fonc.2022.859735)
Supplement: Supplementary file 1 [file DataSheet_1.zip › The bioinformatic analysis of MM and lung cancer supplementary materials/Enrichment analysis/MECR/GSEA_4.1.0/MM TCGA/KEGG.Gsea.1639040591792/KEGG_PEROXISOME.html]

Details for gene set KEGG\_PEROXISOME[GSEA]

|  || Dataset | ExpData\_collapsed\_to\_symbols.ENSG00000116353\_profile\_in\_ExpData.cls #ENSG00000116353 |
| Phenotype | ENSG00000116353\_profile\_in\_ExpData.cls#ENSG00000116353 |
| Upregulated in class | ENSG00000116353\_pos |
| GeneSet | KEGG\_PEROXISOME |
| Enrichment Score (ES) | 0.5896423 |
| Normalized Enrichment Score (NES) | 2.067666 |
| Nominal p-value | 0.0 |
| FDR q-value | 1.2941819E-4 |
| FWER p-Value | 0.002 |
Table: GSEA Results Summary

  

Fig 1: Enrichment plot: KEGG\_PEROXISOME      
 Profile of the Running ES Score & Positions of GeneSet Members on the Rank Ordered List

  

| SYMBOL | TITLE | RANK IN GENE LIST | RANK METRIC SCORE | RUNNING ES | CORE ENRICHMENT || 1 | MPV17 | mitochondrial inner membrane protein MPV17 [Source:HGNC Symbol;Acc:HGNC:7224] | 110 | 0.521 | 0.0340 | Yes |
| 2 | SLC25A17 | solute carrier family 25 member 17 [Source:HGNC Symbol;Acc:HGNC:10987] | 181 | 0.491 | 0.0668 | Yes |
| 3 | PEX11A | peroxisomal biogenesis factor 11 alpha [Source:HGNC Symbol;Acc:HGNC:8852] | 434 | 0.428 | 0.0905 | Yes |
| 4 | CROT | carnitine O-octanoyltransferase [Source:HGNC Symbol;Acc:HGNC:2366] | 528 | 0.408 | 0.1169 | Yes |
| 5 | PRDX1 | peroxiredoxin 1 [Source:HGNC Symbol;Acc:HGNC:9352] | 590 | 0.399 | 0.1435 | Yes |
| 6 | GNPAT | glyceronephosphate O-acyltransferase [Source:HGNC Symbol;Acc:HGNC:4416] | 618 | 0.395 | 0.1707 | Yes |
| 7 | HSD17B4 | hydroxysteroid 17-beta dehydrogenase 4 [Source:HGNC Symbol;Acc:HGNC:5213] | 639 | 0.392 | 0.1978 | Yes |
| 8 | AMACR | alpha-methylacyl-CoA racemase [Source:HGNC Symbol;Acc:HGNC:451] | 710 | 0.381 | 0.2229 | Yes |
| 9 | PMVK | phosphomevalonate kinase [Source:HGNC Symbol;Acc:HGNC:9141] | 867 | 0.359 | 0.2442 | Yes |
| 10 | PXMP4 | peroxisomal membrane protein 4 [Source:HGNC Symbol;Acc:HGNC:15920] | 984 | 0.347 | 0.2657 | Yes |
| 11 | DECR2 | "2,4-dienoyl-CoA reductase 2 [Source:HGNC Symbol;Acc:HGNC:2754]" | 1020 | 0.343 | 0.2890 | Yes |
| 12 | ECI2 | enoyl-CoA delta isomerase 2 [Source:HGNC Symbol;Acc:HGNC:14601] | 1099 | 0.335 | 0.3106 | Yes |
| 13 | ACSL5 | acyl-CoA synthetase long chain family member 5 [Source:HGNC Symbol;Acc:HGNC:16526] | 1159 | 0.329 | 0.3323 | Yes |
| 14 | PEX19 | peroxisomal biogenesis factor 19 [Source:HGNC Symbol;Acc:HGNC:9713] | 1295 | 0.317 | 0.3512 | Yes |
| 15 | PEX5 | peroxisomal biogenesis factor 5 [Source:HGNC Symbol;Acc:HGNC:9719] | 1457 | 0.302 | 0.3684 | Yes |
| 16 | ABCD2 | ATP binding cassette subfamily D member 2 [Source:HGNC Symbol;Acc:HGNC:66] | 1465 | 0.302 | 0.3895 | Yes |
| 17 | IDH2 | isocitrate dehydrogenase (NADP(+)) 2 [Source:HGNC Symbol;Acc:HGNC:5383] | 1478 | 0.301 | 0.4104 | Yes |
| 18 | PEX10 | peroxisomal biogenesis factor 10 [Source:HGNC Symbol;Acc:HGNC:8851] | 1617 | 0.290 | 0.4274 | Yes |
| 19 | PEX6 | peroxisomal biogenesis factor 6 [Source:HGNC Symbol;Acc:HGNC:8859] | 1713 | 0.283 | 0.4449 | Yes |
| 20 | ACOT8 | acyl-CoA thioesterase 8 [Source:HGNC Symbol;Acc:HGNC:15919] | 1846 | 0.273 | 0.4608 | Yes |
| 21 | DHRS4 | dehydrogenase/reductase 4 [Source:HGNC Symbol;Acc:HGNC:16985] | 2069 | 0.260 | 0.4735 | Yes |
| 22 | EHHADH | enoyl-CoA hydratase and 3-hydroxyacyl CoA dehydrogenase [Source:HGNC Symbol;Acc:HGNC:3247] | 2104 | 0.258 | 0.4908 | Yes |
| 23 | ECH1 | enoyl-CoA hydratase 1 [Source:HGNC Symbol;Acc:HGNC:3149] | 2507 | 0.236 | 0.4972 | Yes |
| 24 | PEX3 | peroxisomal biogenesis factor 3 [Source:HGNC Symbol;Acc:HGNC:8858] | 2678 | 0.228 | 0.5090 | Yes |
| 25 | GSTK1 | glutathione S-transferase kappa 1 [Source:HGNC Symbol;Acc:HGNC:16906] | 2815 | 0.221 | 0.5211 | Yes |
| 26 | PEX7 | peroxisomal biogenesis factor 7 [Source:HGNC Symbol;Acc:HGNC:8860] | 2867 | 0.219 | 0.5352 | Yes |
| 27 | SCP2 | sterol carrier protein 2 [Source:HGNC Symbol;Acc:HGNC:10606] | 2925 | 0.216 | 0.5491 | Yes |
| 28 | PECR | peroxisomal trans-2-enoyl-CoA reductase [Source:HGNC Symbol;Acc:HGNC:18281] | 2936 | 0.216 | 0.5640 | Yes |
| 29 | IDH1 | isocitrate dehydrogenase (NADP(+)) 1 [Source:HGNC Symbol;Acc:HGNC:5382] | 3088 | 0.210 | 0.5750 | Yes |
| 30 | PXMP2 | peroxisomal membrane protein 2 [Source:HGNC Symbol;Acc:HGNC:9716] | 3129 | 0.208 | 0.5886 | Yes |
| 31 | PEX11B | peroxisomal biogenesis factor 11 beta [Source:HGNC Symbol;Acc:HGNC:8853] | 3918 | 0.179 | 0.5811 | Yes |
| 32 | HMGCL | 3-hydroxy-3-methylglutaryl-CoA lyase [Source:HGNC Symbol;Acc:HGNC:5005] | 4068 | 0.174 | 0.5896 | Yes |
| 33 | PEX26 | peroxisomal biogenesis factor 26 [Source:HGNC Symbol;Acc:HGNC:22965] | 4801 | 0.154 | 0.5818 | No |
| 34 | EPHX2 | epoxide hydrolase 2 [Source:HGNC Symbol;Acc:HGNC:3402] | 5018 | 0.148 | 0.5868 | No |
| 35 | MLYCD | malonyl-CoA decarboxylase [Source:HGNC Symbol;Acc:HGNC:7150] | 5493 | 0.137 | 0.5844 | No |
| 36 | ABCD3 | ATP binding cassette subfamily D member 3 [Source:HGNC Symbol;Acc:HGNC:67] | 6235 | 0.122 | 0.5741 | No |
| 37 | NUDT19 | nudix hydrolase 19 [Source:HGNC Symbol;Acc:HGNC:32036] | 7281 | 0.105 | 0.5549 | No |
| 38 | PRDX5 | peroxiredoxin 5 [Source:HGNC Symbol;Acc:HGNC:9355] | 7572 | 0.100 | 0.5545 | No |
| 39 | HAO2 | hydroxyacid oxidase 2 [Source:HGNC Symbol;Acc:HGNC:4810] | 7630 | 0.100 | 0.5601 | No |
| 40 | AGPS | alkylglycerone phosphate synthase [Source:HGNC Symbol;Acc:HGNC:327] | 7863 | 0.097 | 0.5610 | No |
| 41 | DAO | D-amino acid oxidase [Source:HGNC Symbol;Acc:HGNC:2671] | 7961 | 0.095 | 0.5653 | No |
| 42 | ABCD1 | ATP binding cassette subfamily D member 1 [Source:HGNC Symbol;Acc:HGNC:61] | 8215 | 0.092 | 0.5653 | No |
| 43 | HACL1 | 2-hydroxyacyl-CoA lyase 1 [Source:HGNC Symbol;Acc:HGNC:17856] | 8763 | 0.085 | 0.5574 | No |
| 44 | PEX11G | peroxisomal biogenesis factor 11 gamma [Source:HGNC Symbol;Acc:HGNC:20208] | 9643 | 0.075 | 0.5403 | No |
| 45 | CAT | catalase [Source:HGNC Symbol;Acc:HGNC:1516] | 10819 | 0.063 | 0.5147 | No |
| 46 | MVK | mevalonate kinase [Source:HGNC Symbol;Acc:HGNC:7530] | 11938 | 0.053 | 0.4900 | No |
| 47 | SOD1 | superoxide dismutase 1 [Source:HGNC Symbol;Acc:HGNC:11179] | 12392 | 0.049 | 0.4819 | No |
| 48 | ACSL4 | acyl-CoA synthetase long chain family member 4 [Source:HGNC Symbol;Acc:HGNC:3571] | 13366 | 0.042 | 0.4601 | No |
| 49 | ACSL3 | acyl-CoA synthetase long chain family member 3 [Source:HGNC Symbol;Acc:HGNC:3570] | 13933 | 0.038 | 0.4484 | No |
| 50 | PEX2 | peroxisomal biogenesis factor 2 [Source:HGNC Symbol;Acc:HGNC:9717] | 14717 | 0.034 | 0.4308 | No |
| 51 | XDH | xanthine dehydrogenase [Source:HGNC Symbol;Acc:HGNC:12805] | 15854 | 0.027 | 0.4038 | No |
| 52 | AGXT | alanine--glyoxylate and serine--pyruvate aminotransferase [Source:HGNC Symbol;Acc:HGNC:341] | 16916 | 0.021 | 0.3782 | No |
| 53 | DDO | D-aspartate oxidase [Source:HGNC Symbol;Acc:HGNC:2727] | 17408 | 0.018 | 0.3669 | No |
| 54 | PAOX | polyamine oxidase [Source:HGNC Symbol;Acc:HGNC:20837] | 18798 | 0.010 | 0.3322 | No |
| 55 | ACOX3 | "acyl-CoA oxidase 3, pristanoyl [Source:HGNC Symbol;Acc:HGNC:121]" | 18939 | 0.010 | 0.3293 | No |
| 56 | NUDT12 | nudix hydrolase 12 [Source:HGNC Symbol;Acc:HGNC:18826] | 21217 | -0.003 | 0.2715 | No |
| 57 | FAR2 | fatty acyl-CoA reductase 2 [Source:HGNC Symbol;Acc:HGNC:25531] | 23442 | -0.017 | 0.2160 | No |
| 58 | SLC27A2 | solute carrier family 27 member 2 [Source:HGNC Symbol;Acc:HGNC:10996] | 24510 | -0.023 | 0.1905 | No |
| 59 | PHYH | phytanoyl-CoA 2-hydroxylase [Source:HGNC Symbol;Acc:HGNC:8940] | 27290 | -0.044 | 0.1228 | No |
| 60 | NOS2 | nitric oxide synthase 2 [Source:HGNC Symbol;Acc:HGNC:7873] | 27899 | -0.049 | 0.1107 | No |
| 61 | ACOX2 | acyl-CoA oxidase 2 [Source:HGNC Symbol;Acc:HGNC:120] | 29514 | -0.066 | 0.0743 | No |
| 62 | PEX12 | peroxisomal biogenesis factor 12 [Source:HGNC Symbol;Acc:HGNC:8854] | 29814 | -0.070 | 0.0716 | No |
| 63 | PIPOX | pipecolic acid and sarcosine oxidase [Source:HGNC Symbol;Acc:HGNC:17804] | 29866 | -0.070 | 0.0752 | No |
| 64 | CRAT | carnitine O-acetyltransferase [Source:HGNC Symbol;Acc:HGNC:2342] | 30229 | -0.075 | 0.0713 | No |
| 65 | BAAT | bile acid-CoA:amino acid N-acyltransferase [Source:HGNC Symbol;Acc:HGNC:932] | 30770 | -0.082 | 0.0633 | No |
| 66 | MPV17L | MPV17 mitochondrial inner membrane protein like [Source:HGNC Symbol;Acc:HGNC:26827] | 31406 | -0.090 | 0.0535 | No |
| 67 | PEX14 | peroxisomal biogenesis factor 14 [Source:HGNC Symbol;Acc:HGNC:8856] | 31575 | -0.093 | 0.0557 | No |
| 68 | PEX13 | peroxisomal biogenesis factor 13 [Source:HGNC Symbol;Acc:HGNC:8855] | 32117 | -0.102 | 0.0491 | No |
| 69 | PEX1 | peroxisomal biogenesis factor 1 [Source:HGNC Symbol;Acc:HGNC:8850] | 32305 | -0.105 | 0.0518 | No |
| 70 | HAO1 | hydroxyacid oxidase 1 [Source:HGNC Symbol;Acc:HGNC:4809] | 32545 | -0.110 | 0.0534 | No |
| 71 | FAR1 | fatty acyl-CoA reductase 1 [Source:HGNC Symbol;Acc:HGNC:26222] | 33376 | -0.126 | 0.0412 | No |
| 72 | ACOX1 | acyl-CoA oxidase 1 [Source:HGNC Symbol;Acc:HGNC:119] | 33631 | -0.132 | 0.0440 | No |
| 73 | ACSL6 | acyl-CoA synthetase long chain family member 6 [Source:HGNC Symbol;Acc:HGNC:16496] | 35047 | -0.172 | 0.0201 | No |
| 74 | PEX16 | peroxisomal biogenesis factor 16 [Source:HGNC Symbol;Acc:HGNC:8857] | 35051 | -0.172 | 0.0322 | No |
| 75 | ABCD4 | ATP binding cassette subfamily D member 4 [Source:HGNC Symbol;Acc:HGNC:68] | 36103 | -0.212 | 0.0203 | No |
| 76 | ACAA1 | acetyl-CoA acyltransferase 1 [Source:HGNC Symbol;Acc:HGNC:82] | 36185 | -0.215 | 0.0334 | No |
| 77 | SOD2 | superoxide dismutase 2 [Source:HGNC Symbol;Acc:HGNC:11180] | 37254 | -0.276 | 0.0257 | No |
| 78 | ACSL1 | acyl-CoA synthetase long chain family member 1 [Source:HGNC Symbol;Acc:HGNC:3569] | 38226 | -0.383 | 0.0280 | No |
Table: GSEA details [plain text format]

  

Fig 2: KEGG\_PEROXISOME      
 Blue-Pink O' Gram in the Space of the Analyzed GeneSet

  

Fig 3: KEGG\_PEROXISOME: Random ES distribution      
 Gene set null distribution of ES for **KEGG\_PEROXISOME**
