# Supplementary material for: Simultaneous Presentation of Multiple Myeloma and Lung Cancer: Case Report and Gene Bioinformatics Analysis
Source: Front Oncol. 2022 Jun 13;12:859735. doi: 10.3389/fonc.2022.859735 (PMC9235397; doi:10.3389/fonc.2022.859735)
Supplement: Supplementary file 1 [file DataSheet_1.zip › The bioinformatic analysis of MM and lung cancer supplementary materials/Enrichment analysis/MECR/GSEA_4.1.0/MM TCGA/KEGG.Gsea.1639040591792/KEGG_PROPANOATE_METABOLISM.html]

Details for gene set KEGG\_PROPANOATE\_METABOLISM[GSEA]

|  || Dataset | ExpData\_collapsed\_to\_symbols.ENSG00000116353\_profile\_in\_ExpData.cls #ENSG00000116353 |
| Phenotype | ENSG00000116353\_profile\_in\_ExpData.cls#ENSG00000116353 |
| Upregulated in class | ENSG00000116353\_pos |
| GeneSet | KEGG\_PROPANOATE\_METABOLISM |
| Enrichment Score (ES) | 0.7213534 |
| Normalized Enrichment Score (NES) | 2.1575103 |
| Nominal p-value | 0.0 |
| FDR q-value | 0.0 |
| FWER p-Value | 0.0 |
Table: GSEA Results Summary

  

Fig 1: Enrichment plot: KEGG\_PROPANOATE\_METABOLISM      
 Profile of the Running ES Score & Positions of GeneSet Members on the Rank Ordered List

  

| SYMBOL | TITLE | RANK IN GENE LIST | RANK METRIC SCORE | RUNNING ES | CORE ENRICHMENT || 1 | PCCB | propionyl-CoA carboxylase subunit beta [Source:HGNC Symbol;Acc:HGNC:8654] | 40 | 0.581 | 0.0829 | Yes |
| 2 | ALDH1B1 | aldehyde dehydrogenase 1 family member B1 [Source:HGNC Symbol;Acc:HGNC:407] | 372 | 0.439 | 0.1379 | Yes |
| 3 | ACADM | acyl-CoA dehydrogenase medium chain [Source:HGNC Symbol;Acc:HGNC:89] | 803 | 0.369 | 0.1803 | Yes |
| 4 | ECHS1 | "enoyl-CoA hydratase, short chain 1 [Source:HGNC Symbol;Acc:HGNC:3151]" | 890 | 0.356 | 0.2295 | Yes |
| 5 | ALDH3A2 | aldehyde dehydrogenase 3 family member A2 [Source:HGNC Symbol;Acc:HGNC:403] | 902 | 0.354 | 0.2804 | Yes |
| 6 | SUCLG2 | succinate-CoA ligase GDP-forming subunit beta [Source:HGNC Symbol;Acc:HGNC:11450] | 1033 | 0.341 | 0.3264 | Yes |
| 7 | HIBCH | 3-hydroxyisobutyryl-CoA hydrolase [Source:HGNC Symbol;Acc:HGNC:4908] | 1265 | 0.319 | 0.3666 | Yes |
| 8 | LDHB | lactate dehydrogenase B [Source:HGNC Symbol;Acc:HGNC:6541] | 1375 | 0.309 | 0.4085 | Yes |
| 9 | ALDH9A1 | aldehyde dehydrogenase 9 family member A1 [Source:HGNC Symbol;Acc:HGNC:412] | 1406 | 0.306 | 0.4519 | Yes |
| 10 | ACAT1 | acetyl-CoA acetyltransferase 1 [Source:HGNC Symbol;Acc:HGNC:93] | 1467 | 0.302 | 0.4940 | Yes |
| 11 | ALDH6A1 | aldehyde dehydrogenase 6 family member A1 [Source:HGNC Symbol;Acc:HGNC:7179] | 1585 | 0.293 | 0.5334 | Yes |
| 12 | ACACA | acetyl-CoA carboxylase alpha [Source:HGNC Symbol;Acc:HGNC:84] | 1905 | 0.270 | 0.5643 | Yes |
| 13 | EHHADH | enoyl-CoA hydratase and 3-hydroxyacyl CoA dehydrogenase [Source:HGNC Symbol;Acc:HGNC:3247] | 2104 | 0.258 | 0.5965 | Yes |
| 14 | SUCLA2 | succinate-CoA ligase ADP-forming subunit beta [Source:HGNC Symbol;Acc:HGNC:11448] | 2348 | 0.244 | 0.6256 | Yes |
| 15 | ACSS1 | acyl-CoA synthetase short chain family member 1 [Source:HGNC Symbol;Acc:HGNC:16091] | 2776 | 0.223 | 0.6470 | Yes |
| 16 | MMUT | methylmalonyl-CoA mutase [Source:HGNC Symbol;Acc:HGNC:7526] | 3141 | 0.207 | 0.6677 | Yes |
| 17 | LDHAL6A | lactate dehydrogenase A like 6A [Source:HGNC Symbol;Acc:HGNC:28335] | 3170 | 0.206 | 0.6968 | Yes |
| 18 | MCEE | methylmalonyl-CoA epimerase [Source:HGNC Symbol;Acc:HGNC:16732] | 4150 | 0.172 | 0.6968 | Yes |
| 19 | LDHA | lactate dehydrogenase A [Source:HGNC Symbol;Acc:HGNC:6535] | 4425 | 0.164 | 0.7135 | Yes |
| 20 | MLYCD | malonyl-CoA decarboxylase [Source:HGNC Symbol;Acc:HGNC:7150] | 5493 | 0.137 | 0.7062 | Yes |
| 21 | PCCA | propionyl-CoA carboxylase subunit alpha [Source:HGNC Symbol;Acc:HGNC:8653] | 6263 | 0.122 | 0.7042 | Yes |
| 22 | ABAT | 4-aminobutyrate aminotransferase [Source:HGNC Symbol;Acc:HGNC:23] | 6280 | 0.122 | 0.7214 | Yes |
| 23 | ACSS2 | acyl-CoA synthetase short chain family member 2 [Source:HGNC Symbol;Acc:HGNC:15814] | 7325 | 0.104 | 0.7098 | No |
| 24 | HADHA | hydroxyacyl-CoA dehydrogenase trifunctional multienzyme complex subunit alpha [Source:HGNC Symbol;Acc:HGNC:4801] | 8241 | 0.092 | 0.6998 | No |
| 25 | SUCLG1 | succinate-CoA ligase GDP/ADP-forming subunit alpha [Source:HGNC Symbol;Acc:HGNC:11449] | 9415 | 0.077 | 0.6811 | No |
| 26 | ACAT2 | acetyl-CoA acetyltransferase 2 [Source:HGNC Symbol;Acc:HGNC:94] | 10235 | 0.068 | 0.6701 | No |
| 27 | ALDH7A1 | aldehyde dehydrogenase 7 family member A1 [Source:HGNC Symbol;Acc:HGNC:877] | 14303 | 0.036 | 0.5718 | No |
| 28 | LDHAL6B | lactate dehydrogenase A like 6B [Source:HGNC Symbol;Acc:HGNC:21481] | 20658 | -0.000 | 0.4102 | No |
| 29 | SUCLG2P2 | SUCLG2 pseudogene 2 [Source:HGNC Symbol;Acc:HGNC:43997] | 21183 | -0.003 | 0.3973 | No |
| 30 | LDHC | lactate dehydrogenase C [Source:HGNC Symbol;Acc:HGNC:6544] | 29290 | -0.064 | 0.2002 | No |
| 31 | ACSS3 | acyl-CoA synthetase short chain family member 3 [Source:HGNC Symbol;Acc:HGNC:24723] | 31128 | -0.086 | 0.1659 | No |
| 32 | ACACB | acetyl-CoA carboxylase beta [Source:HGNC Symbol;Acc:HGNC:85] | 33834 | -0.137 | 0.1169 | No |
| 33 | ALDH2 | aldehyde dehydrogenase 2 family member [Source:HGNC Symbol;Acc:HGNC:404] | 34564 | -0.157 | 0.1211 | No |
Table: GSEA details [plain text format]

  

Fig 2: KEGG\_PROPANOATE\_METABOLISM      
 Blue-Pink O' Gram in the Space of the Analyzed GeneSet

  

Fig 3: KEGG\_PROPANOATE\_METABOLISM: Random ES distribution      
 Gene set null distribution of ES for **KEGG\_PROPANOATE\_METABOLISM**
