# Supplementary material for: Simultaneous Presentation of Multiple Myeloma and Lung Cancer: Case Report and Gene Bioinformatics Analysis
Source: Front Oncol. 2022 Jun 13;12:859735. doi: 10.3389/fonc.2022.859735 (PMC9235397; doi:10.3389/fonc.2022.859735)
Supplement: Supplementary file 1 [file DataSheet_1.zip › The bioinformatic analysis of MM and lung cancer supplementary materials/Enrichment analysis/MECR/GSEA_4.1.0/MM TCGA/KEGG.Gsea.1639040591792/KEGG_PROSTATE_CANCER.html]

Details for gene set KEGG\_PROSTATE\_CANCER[GSEA]

|  || Dataset | ExpData\_collapsed\_to\_symbols.ENSG00000116353\_profile\_in\_ExpData.cls #ENSG00000116353 |
| Phenotype | ENSG00000116353\_profile\_in\_ExpData.cls#ENSG00000116353 |
| Upregulated in class | ENSG00000116353\_neg |
| GeneSet | KEGG\_PROSTATE\_CANCER |
| Enrichment Score (ES) | -0.57439226 |
| Normalized Enrichment Score (NES) | -2.2055736 |
| Nominal p-value | 0.0 |
| FDR q-value | 0.0 |
| FWER p-Value | 0.0 |
Table: GSEA Results Summary

  

Fig 1: Enrichment plot: KEGG\_PROSTATE\_CANCER      
 Profile of the Running ES Score & Positions of GeneSet Members on the Rank Ordered List

  

| SYMBOL | TITLE | RANK IN GENE LIST | RANK METRIC SCORE | RUNNING ES | CORE ENRICHMENT || 1 | CREB3L4 | cAMP responsive element binding protein 3 like 4 [Source:HGNC Symbol;Acc:HGNC:18854] | 866 | 0.359 | -0.0015 | No |
| 2 | CASP9 | caspase 9 [Source:HGNC Symbol;Acc:HGNC:1511] | 1063 | 0.338 | 0.0129 | No |
| 3 | MTOR | mechanistic target of rapamycin kinase [Source:HGNC Symbol;Acc:HGNC:3942] | 1153 | 0.329 | 0.0295 | No |
| 4 | PIK3R2 | phosphoinositide-3-kinase regulatory subunit 2 [Source:HGNC Symbol;Acc:HGNC:8980] | 1248 | 0.320 | 0.0455 | No |
| 5 | E2F2 | E2F transcription factor 2 [Source:HGNC Symbol;Acc:HGNC:3114] | 1364 | 0.310 | 0.0603 | No |
| 6 | TP53 | tumor protein p53 [Source:HGNC Symbol;Acc:HGNC:11998] | 2121 | 0.257 | 0.0557 | No |
| 7 | PIK3R3 | phosphoinositide-3-kinase regulatory subunit 3 [Source:HGNC Symbol;Acc:HGNC:8981] | 2602 | 0.231 | 0.0568 | No |
| 8 | AR | androgen receptor [Source:HGNC Symbol;Acc:HGNC:644] | 2913 | 0.217 | 0.0613 | No |
| 9 | SRD5A2 | steroid 5 alpha-reductase 2 [Source:HGNC Symbol;Acc:HGNC:11285] | 2962 | 0.215 | 0.0724 | No |
| 10 | MAP2K2 | mitogen-activated protein kinase kinase 2 [Source:HGNC Symbol;Acc:HGNC:6842] | 5104 | 0.146 | 0.0262 | No |
| 11 | ERBB2 | erb-b2 receptor tyrosine kinase 2 [Source:HGNC Symbol;Acc:HGNC:3430] | 5193 | 0.144 | 0.0322 | No |
| 12 | E2F3 | E2F transcription factor 3 [Source:HGNC Symbol;Acc:HGNC:3115] | 5759 | 0.132 | 0.0253 | No |
| 13 | E2F1 | E2F transcription factor 1 [Source:HGNC Symbol;Acc:HGNC:3113] | 6489 | 0.118 | 0.0135 | No |
| 14 | HSP90B1 | heat shock protein 90 beta family member 1 [Source:HGNC Symbol;Acc:HGNC:12028] | 7140 | 0.107 | 0.0031 | No |
| 15 | MDM2 | MDM2 proto-oncogene [Source:HGNC Symbol;Acc:HGNC:6973] | 7403 | 0.103 | 0.0023 | No |
| 16 | PIK3CB | "phosphatidylinositol-4,5-bisphosphate 3-kinase catalytic subunit beta [Source:HGNC Symbol;Acc:HGNC:8976]" | 7716 | 0.099 | -0.0000 | No |
| 17 | CREB3L1 | cAMP responsive element binding protein 3 like 1 [Source:HGNC Symbol;Acc:HGNC:18856] | 9079 | 0.081 | -0.0301 | No |
| 18 | CCNE1 | cyclin E1 [Source:HGNC Symbol;Acc:HGNC:1589] | 9923 | 0.071 | -0.0475 | No |
| 19 | CHUK | component of inhibitor of nuclear factor kappa B kinase complex [Source:HGNC Symbol;Acc:HGNC:1974] | 9925 | 0.071 | -0.0434 | No |
| 20 | HSP90AB1 | heat shock protein 90 alpha family class B member 1 [Source:HGNC Symbol;Acc:HGNC:5258] | 10928 | 0.062 | -0.0654 | No |
| 21 | GSK3B | glycogen synthase kinase 3 beta [Source:HGNC Symbol;Acc:HGNC:4617] | 12189 | 0.051 | -0.0946 | No |
| 22 | TGFA | transforming growth factor alpha [Source:HGNC Symbol;Acc:HGNC:11765] | 12386 | 0.049 | -0.0968 | No |
| 23 | INS | insulin [Source:HGNC Symbol;Acc:HGNC:6081] | 12938 | 0.045 | -0.1082 | No |
| 24 | INSRR | insulin receptor related receptor [Source:HGNC Symbol;Acc:HGNC:6093] | 13274 | 0.043 | -0.1143 | No |
| 25 | IGF1R | insulin like growth factor 1 receptor [Source:HGNC Symbol;Acc:HGNC:5465] | 17189 | 0.019 | -0.2130 | No |
| 26 | RB1 | RB transcriptional corepressor 1 [Source:HGNC Symbol;Acc:HGNC:9884] | 17240 | 0.019 | -0.2132 | No |
| 27 | PDGFD | platelet derived growth factor D [Source:HGNC Symbol;Acc:HGNC:30620] | 18049 | 0.014 | -0.2330 | No |
| 28 | KLK3 | kallikrein related peptidase 3 [Source:HGNC Symbol;Acc:HGNC:6364] | 18403 | 0.012 | -0.2412 | No |
| 29 | SOS1 | SOS Ras/Rac guanine nucleotide exchange factor 1 [Source:HGNC Symbol;Acc:HGNC:11187] | 18532 | 0.012 | -0.2438 | No |
| 30 | PTEN | phosphatase and tensin homolog [Source:HGNC Symbol;Acc:HGNC:9588] | 19053 | 0.009 | -0.2566 | No |
| 31 | IKBKB | inhibitor of nuclear factor kappa B kinase subunit beta [Source:HGNC Symbol;Acc:HGNC:5960] | 20718 | -0.000 | -0.2990 | No |
| 32 | MAPK1 | mitogen-activated protein kinase 1 [Source:HGNC Symbol;Acc:HGNC:6871] | 23167 | -0.015 | -0.3605 | No |
| 33 | HRAS | "HRas proto-oncogene, GTPase [Source:HGNC Symbol;Acc:HGNC:5173]" | 26672 | -0.039 | -0.4476 | No |
| 34 | ARAF | "A-Raf proto-oncogene, serine/threonine kinase [Source:HGNC Symbol;Acc:HGNC:646]" | 26742 | -0.039 | -0.4471 | No |
| 35 | CREB3L3 | cAMP responsive element binding protein 3 like 3 [Source:HGNC Symbol;Acc:HGNC:18855] | 29858 | -0.070 | -0.5225 | No |
| 36 | CREB3 | cAMP responsive element binding protein 3 [Source:HGNC Symbol;Acc:HGNC:2347] | 30349 | -0.076 | -0.5306 | No |
| 37 | PDGFA | platelet derived growth factor subunit A [Source:HGNC Symbol;Acc:HGNC:8799] | 30414 | -0.077 | -0.5278 | No |
| 38 | LEF1 | lymphoid enhancer binding factor 1 [Source:HGNC Symbol;Acc:HGNC:6551] | 31159 | -0.087 | -0.5418 | No |
| 39 | AKT1 | AKT serine/threonine kinase 1 [Source:HGNC Symbol;Acc:HGNC:391] | 31906 | -0.098 | -0.5552 | No |
| 40 | IKBKG | inhibitor of nuclear factor kappa B kinase regulatory subunit gamma [Source:HGNC Symbol;Acc:HGNC:5961] | 32205 | -0.103 | -0.5569 | No |
| 41 | FGFR2 | fibroblast growth factor receptor 2 [Source:HGNC Symbol;Acc:HGNC:3689] | 32385 | -0.107 | -0.5553 | No |
| 42 | EGF | epidermal growth factor [Source:HGNC Symbol;Acc:HGNC:3229] | 33135 | -0.121 | -0.5674 | Yes |
| 43 | AKT2 | AKT serine/threonine kinase 2 [Source:HGNC Symbol;Acc:HGNC:392] | 33213 | -0.123 | -0.5624 | Yes |
| 44 | PDGFC | platelet derived growth factor C [Source:HGNC Symbol;Acc:HGNC:8801] | 33384 | -0.127 | -0.5594 | Yes |
| 45 | GSTP1 | glutathione S-transferase pi 1 [Source:HGNC Symbol;Acc:HGNC:4638] | 33485 | -0.129 | -0.5546 | Yes |
| 46 | PDGFB | platelet derived growth factor subunit B [Source:HGNC Symbol;Acc:HGNC:8800] | 33560 | -0.130 | -0.5490 | Yes |
| 47 | HSP90AA1 | heat shock protein 90 alpha family class A member 1 [Source:HGNC Symbol;Acc:HGNC:5253] | 33724 | -0.134 | -0.5455 | Yes |
| 48 | NRAS | "NRAS proto-oncogene, GTPase [Source:HGNC Symbol;Acc:HGNC:7989]" | 33739 | -0.135 | -0.5381 | Yes |
| 49 | PIK3CD | "phosphatidylinositol-4,5-bisphosphate 3-kinase catalytic subunit delta [Source:HGNC Symbol;Acc:HGNC:8977]" | 33794 | -0.137 | -0.5317 | Yes |
| 50 | AKT3 | AKT serine/threonine kinase 3 [Source:HGNC Symbol;Acc:HGNC:393] | 34501 | -0.156 | -0.5407 | Yes |
| 51 | PDGFRB | platelet derived growth factor receptor beta [Source:HGNC Symbol;Acc:HGNC:8804] | 34887 | -0.167 | -0.5410 | Yes |
| 52 | EGFR | epidermal growth factor receptor [Source:HGNC Symbol;Acc:HGNC:3236] | 35417 | -0.186 | -0.5438 | Yes |
| 53 | MAPK3 | mitogen-activated protein kinase 3 [Source:HGNC Symbol;Acc:HGNC:6877] | 35579 | -0.191 | -0.5370 | Yes |
| 54 | CCNE2 | cyclin E2 [Source:HGNC Symbol;Acc:HGNC:1590] | 35583 | -0.191 | -0.5261 | Yes |
| 55 | CREB3L2 | cAMP responsive element binding protein 3 like 2 [Source:HGNC Symbol;Acc:HGNC:23720] | 35730 | -0.197 | -0.5186 | Yes |
| 56 | CCND1 | cyclin D1 [Source:HGNC Symbol;Acc:HGNC:1582] | 35880 | -0.203 | -0.5108 | Yes |
| 57 | IGF1 | insulin like growth factor 1 [Source:HGNC Symbol;Acc:HGNC:5464] | 35903 | -0.203 | -0.4997 | Yes |
| 58 | NKX3-1 | NK3 homeobox 1 [Source:HGNC Symbol;Acc:HGNC:7838] | 35993 | -0.208 | -0.4900 | Yes |
| 59 | MAP2K1 | mitogen-activated protein kinase kinase 1 [Source:HGNC Symbol;Acc:HGNC:6840] | 36024 | -0.208 | -0.4789 | Yes |
| 60 | PIK3CG | "phosphatidylinositol-4,5-bisphosphate 3-kinase catalytic subunit gamma [Source:HGNC Symbol;Acc:HGNC:8978]" | 36243 | -0.217 | -0.4720 | Yes |
| 61 | PDGFRA | platelet derived growth factor receptor alpha [Source:HGNC Symbol;Acc:HGNC:8803] | 36521 | -0.231 | -0.4658 | Yes |
| 62 | GRB2 | growth factor receptor bound protein 2 [Source:HGNC Symbol;Acc:HGNC:4566] | 36523 | -0.231 | -0.4525 | Yes |
| 63 | FGFR1 | fibroblast growth factor receptor 1 [Source:HGNC Symbol;Acc:HGNC:3688] | 36687 | -0.241 | -0.4429 | Yes |
| 64 | CREB5 | cAMP responsive element binding protein 5 [Source:HGNC Symbol;Acc:HGNC:16844] | 36720 | -0.243 | -0.4298 | Yes |
| 65 | ATF4 | activating transcription factor 4 [Source:HGNC Symbol;Acc:HGNC:786] | 36818 | -0.248 | -0.4181 | Yes |
| 66 | TCF7L1 | transcription factor 7 like 1 [Source:HGNC Symbol;Acc:HGNC:11640] | 36849 | -0.251 | -0.4045 | Yes |
| 67 | PDPK1 | 3-phosphoinositide dependent protein kinase 1 [Source:HGNC Symbol;Acc:HGNC:8816] | 36889 | -0.253 | -0.3910 | Yes |
| 68 | CDKN1B | cyclin dependent kinase inhibitor 1B [Source:HGNC Symbol;Acc:HGNC:1785] | 37087 | -0.265 | -0.3809 | Yes |
| 69 | CDK2 | cyclin dependent kinase 2 [Source:HGNC Symbol;Acc:HGNC:1771] | 37148 | -0.269 | -0.3670 | Yes |
| 70 | PIK3R1 | phosphoinositide-3-kinase regulatory subunit 1 [Source:HGNC Symbol;Acc:HGNC:8979] | 37172 | -0.270 | -0.3521 | Yes |
| 71 | TCF7L2 | transcription factor 7 like 2 [Source:HGNC Symbol;Acc:HGNC:11641] | 37205 | -0.273 | -0.3372 | Yes |
| 72 | NFKBIA | NFKB inhibitor alpha [Source:HGNC Symbol;Acc:HGNC:7797] | 37222 | -0.275 | -0.3219 | Yes |
| 73 | PIK3R5 | phosphoinositide-3-kinase regulatory subunit 5 [Source:HGNC Symbol;Acc:HGNC:30035] | 37628 | -0.307 | -0.3146 | Yes |
| 74 | EP300 | E1A binding protein p300 [Source:HGNC Symbol;Acc:HGNC:3373] | 37644 | -0.308 | -0.2973 | Yes |
| 75 | BCL2 | BCL2 apoptosis regulator [Source:HGNC Symbol;Acc:HGNC:990] | 37727 | -0.316 | -0.2813 | Yes |
| 76 | BAD | BCL2 associated agonist of cell death [Source:HGNC Symbol;Acc:HGNC:936] | 37742 | -0.318 | -0.2634 | Yes |
| 77 | KRAS | "KRAS proto-oncogene, GTPase [Source:HGNC Symbol;Acc:HGNC:6407]" | 37789 | -0.323 | -0.2461 | Yes |
| 78 | RAF1 | "Raf-1 proto-oncogene, serine/threonine kinase [Source:HGNC Symbol;Acc:HGNC:9829]" | 37807 | -0.325 | -0.2280 | Yes |
| 79 | BRAF | "B-Raf proto-oncogene, serine/threonine kinase [Source:HGNC Symbol;Acc:HGNC:1097]" | 37844 | -0.330 | -0.2100 | Yes |
| 80 | CREBBP | CREB binding protein [Source:HGNC Symbol;Acc:HGNC:2348] | 37926 | -0.340 | -0.1926 | Yes |
| 81 | CDKN1A | cyclin dependent kinase inhibitor 1A [Source:HGNC Symbol;Acc:HGNC:1784] | 38207 | -0.380 | -0.1779 | Yes |
| 82 | PIK3CA | "phosphatidylinositol-4,5-bisphosphate 3-kinase catalytic subunit alpha [Source:HGNC Symbol;Acc:HGNC:8975]" | 38268 | -0.391 | -0.1570 | Yes |
| 83 | TCF7 | transcription factor 7 [Source:HGNC Symbol;Acc:HGNC:11639] | 38380 | -0.413 | -0.1362 | Yes |
| 84 | CREB1 | cAMP responsive element binding protein 1 [Source:HGNC Symbol;Acc:HGNC:2345] | 38395 | -0.418 | -0.1126 | Yes |
| 85 | CTNNB1 | catenin beta 1 [Source:HGNC Symbol;Acc:HGNC:2514] | 38456 | -0.438 | -0.0890 | Yes |
| 86 | NFKB1 | nuclear factor kappa B subunit 1 [Source:HGNC Symbol;Acc:HGNC:7794] | 38470 | -0.443 | -0.0640 | Yes |
| 87 | RELA | "RELA proto-oncogene, NF-kB subunit [Source:HGNC Symbol;Acc:HGNC:9955]" | 38475 | -0.445 | -0.0386 | Yes |
| 88 | FOXO1 | forkhead box O1 [Source:HGNC Symbol;Acc:HGNC:3819] | 38578 | -0.522 | -0.0113 | Yes |
| 89 | SOS2 | SOS Ras/Rho guanine nucleotide exchange factor 2 [Source:HGNC Symbol;Acc:HGNC:11188] | 38581 | -0.528 | 0.0189 | Yes |
Table: GSEA details [plain text format]

  

Fig 2: KEGG\_PROSTATE\_CANCER      
 Blue-Pink O' Gram in the Space of the Analyzed GeneSet

  

Fig 3: KEGG\_PROSTATE\_CANCER: Random ES distribution      
 Gene set null distribution of ES for **KEGG\_PROSTATE\_CANCER**
