# Supplementary material for: Simultaneous Presentation of Multiple Myeloma and Lung Cancer: Case Report and Gene Bioinformatics Analysis
Source: Front Oncol. 2022 Jun 13;12:859735. doi: 10.3389/fonc.2022.859735 (PMC9235397; doi:10.3389/fonc.2022.859735)
Supplement: Supplementary file 1 [file DataSheet_1.zip › The bioinformatic analysis of MM and lung cancer supplementary materials/Enrichment analysis/MECR/GSEA_4.1.0/MM TCGA/KEGG.Gsea.1639040591792/KEGG_PURINE_METABOLISM.html]

Details for gene set KEGG\_PURINE\_METABOLISM[GSEA]

|  || Dataset | ExpData\_collapsed\_to\_symbols.ENSG00000116353\_profile\_in\_ExpData.cls #ENSG00000116353 |
| Phenotype | ENSG00000116353\_profile\_in\_ExpData.cls#ENSG00000116353 |
| Upregulated in class | ENSG00000116353\_pos |
| GeneSet | KEGG\_PURINE\_METABOLISM |
| Enrichment Score (ES) | 0.52216667 |
| Normalized Enrichment Score (NES) | 2.0013187 |
| Nominal p-value | 0.0 |
| FDR q-value | 2.874078E-4 |
| FWER p-Value | 0.007 |
Table: GSEA Results Summary

  

Fig 1: Enrichment plot: KEGG\_PURINE\_METABOLISM      
 Profile of the Running ES Score & Positions of GeneSet Members on the Rank Ordered List

  

| SYMBOL | TITLE | RANK IN GENE LIST | RANK METRIC SCORE | RUNNING ES | CORE ENRICHMENT || 1 | AK2 | adenylate kinase 2 [Source:HGNC Symbol;Acc:HGNC:362] | 11 | 0.625 | 0.0220 | Yes |
| 2 | PAICS | phosphoribosylaminoimidazole carboxylase and phosphoribosylaminoimidazolesuccinocarboxamide synthase [Source:HGNC Symbol;Acc:HGNC:8587] | 12 | 0.623 | 0.0443 | Yes |
| 3 | POLD2 | "DNA polymerase delta 2, accessory subunit [Source:HGNC Symbol;Acc:HGNC:9176]" | 15 | 0.617 | 0.0663 | Yes |
| 4 | ATIC | 5-aminoimidazole-4-carboxamide ribonucleotide formyltransferase/IMP cyclohydrolase [Source:HGNC Symbol;Acc:HGNC:794] | 16 | 0.613 | 0.0882 | Yes |
| 5 | PFAS | phosphoribosylformylglycinamidine synthase [Source:HGNC Symbol;Acc:HGNC:8863] | 17 | 0.611 | 0.1100 | Yes |
| 6 | POLR3K | RNA polymerase III subunit K [Source:HGNC Symbol;Acc:HGNC:14121] | 61 | 0.559 | 0.1289 | Yes |
| 7 | ADSL | adenylosuccinate lyase [Source:HGNC Symbol;Acc:HGNC:291] | 84 | 0.542 | 0.1477 | Yes |
| 8 | POLR1A | RNA polymerase I subunit A [Source:HGNC Symbol;Acc:HGNC:17264] | 155 | 0.501 | 0.1638 | Yes |
| 9 | POLR3H | RNA polymerase III subunit H [Source:HGNC Symbol;Acc:HGNC:30349] | 156 | 0.501 | 0.1817 | Yes |
| 10 | GART | "phosphoribosylglycinamide formyltransferase, phosphoribosylglycinamide synthetase, phosphoribosylaminoimidazole synthetase [Source:HGNC Symbol;Acc:HGNC:4163]" | 166 | 0.496 | 0.1992 | Yes |
| 11 | POLR3B | RNA polymerase III subunit B [Source:HGNC Symbol;Acc:HGNC:30348] | 185 | 0.490 | 0.2163 | Yes |
| 12 | ENTPD5 | ectonucleoside triphosphate diphosphohydrolase 5 (inactive) [Source:HGNC Symbol;Acc:HGNC:3367] | 220 | 0.478 | 0.2325 | Yes |
| 13 | NME1 | NME/NM23 nucleoside diphosphate kinase 1 [Source:HGNC Symbol;Acc:HGNC:7849] | 240 | 0.471 | 0.2488 | Yes |
| 14 | NUDT5 | nudix hydrolase 5 [Source:HGNC Symbol;Acc:HGNC:8052] | 283 | 0.459 | 0.2642 | Yes |
| 15 | PNPT1 | polyribonucleotide nucleotidyltransferase 1 [Source:HGNC Symbol;Acc:HGNC:23166] | 385 | 0.437 | 0.2772 | Yes |
| 16 | ITPA | inosine triphosphatase [Source:HGNC Symbol;Acc:HGNC:6176] | 469 | 0.418 | 0.2900 | Yes |
| 17 | POLD1 | "DNA polymerase delta 1, catalytic subunit [Source:HGNC Symbol;Acc:HGNC:9175]" | 541 | 0.405 | 0.3027 | Yes |
| 18 | RRM2 | ribonucleotide reductase regulatory subunit M2 [Source:HGNC Symbol;Acc:HGNC:10452] | 572 | 0.402 | 0.3163 | Yes |
| 19 | PRIM2 | DNA primase subunit 2 [Source:HGNC Symbol;Acc:HGNC:9370] | 602 | 0.397 | 0.3297 | Yes |
| 20 | POLA1 | "DNA polymerase alpha 1, catalytic subunit [Source:HGNC Symbol;Acc:HGNC:9173]" | 652 | 0.390 | 0.3424 | Yes |
| 21 | POLR1B | RNA polymerase I subunit B [Source:HGNC Symbol;Acc:HGNC:20454] | 735 | 0.377 | 0.3538 | Yes |
| 22 | POLR3GL | RNA polymerase III subunit GL [Source:HGNC Symbol;Acc:HGNC:28466] | 745 | 0.376 | 0.3670 | Yes |
| 23 | NME6 | NME/NM23 nucleoside diphosphate kinase 6 [Source:HGNC Symbol;Acc:HGNC:20567] | 900 | 0.354 | 0.3757 | Yes |
| 24 | PPAT | phosphoribosyl pyrophosphate amidotransferase [Source:HGNC Symbol;Acc:HGNC:9238] | 1029 | 0.342 | 0.3847 | Yes |
| 25 | POLR2H | "RNA polymerase II, I and III subunit H [Source:HGNC Symbol;Acc:HGNC:9195]" | 1100 | 0.335 | 0.3949 | Yes |
| 26 | PRIM1 | DNA primase subunit 1 [Source:HGNC Symbol;Acc:HGNC:9369] | 1113 | 0.334 | 0.4065 | Yes |
| 27 | NME7 | NME/NM23 family member 7 [Source:HGNC Symbol;Acc:HGNC:20461] | 1219 | 0.323 | 0.4153 | Yes |
| 28 | POLR3G | RNA polymerase III subunit G [Source:HGNC Symbol;Acc:HGNC:30075] | 1523 | 0.298 | 0.4182 | Yes |
| 29 | POLR3C | RNA polymerase III subunit C [Source:HGNC Symbol;Acc:HGNC:30076] | 1610 | 0.291 | 0.4264 | Yes |
| 30 | POLD3 | "DNA polymerase delta 3, accessory subunit [Source:HGNC Symbol;Acc:HGNC:20932]" | 1700 | 0.284 | 0.4343 | Yes |
| 31 | POLR2L | "RNA polymerase II, I and III subunit L [Source:HGNC Symbol;Acc:HGNC:9199]" | 1839 | 0.274 | 0.4406 | Yes |
| 32 | PDE8B | phosphodiesterase 8B [Source:HGNC Symbol;Acc:HGNC:8794] | 2033 | 0.262 | 0.4450 | Yes |
| 33 | NT5M | "5',3'-nucleotidase, mitochondrial [Source:HGNC Symbol;Acc:HGNC:15769]" | 2250 | 0.250 | 0.4484 | Yes |
| 34 | APRT | adenine phosphoribosyltransferase [Source:HGNC Symbol;Acc:HGNC:626] | 2418 | 0.241 | 0.4527 | Yes |
| 35 | PDE6D | phosphodiesterase 6D [Source:HGNC Symbol;Acc:HGNC:8788] | 2429 | 0.240 | 0.4611 | Yes |
| 36 | NUDT2 | nudix hydrolase 2 [Source:HGNC Symbol;Acc:HGNC:8049] | 2659 | 0.229 | 0.4634 | Yes |
| 37 | NUDT9 | nudix hydrolase 9 [Source:HGNC Symbol;Acc:HGNC:8056] | 2711 | 0.227 | 0.4702 | Yes |
| 38 | FHIT | fragile histidine triad diadenosine triphosphatase [Source:HGNC Symbol;Acc:HGNC:3701] | 2718 | 0.226 | 0.4781 | Yes |
| 39 | PNP | purine nucleoside phosphorylase [Source:HGNC Symbol;Acc:HGNC:7892] | 2797 | 0.222 | 0.4841 | Yes |
| 40 | POLR3A | RNA polymerase III subunit A [Source:HGNC Symbol;Acc:HGNC:30074] | 3031 | 0.212 | 0.4857 | Yes |
| 41 | POLR2D | RNA polymerase II subunit D [Source:HGNC Symbol;Acc:HGNC:9191] | 3081 | 0.210 | 0.4919 | Yes |
| 42 | POLE2 | "DNA polymerase epsilon 2, accessory subunit [Source:HGNC Symbol;Acc:HGNC:9178]" | 3128 | 0.208 | 0.4982 | Yes |
| 43 | PDE6A | phosphodiesterase 6A [Source:HGNC Symbol;Acc:HGNC:8785] | 3411 | 0.196 | 0.4980 | Yes |
| 44 | POLE4 | "DNA polymerase epsilon 4, accessory subunit [Source:HGNC Symbol;Acc:HGNC:18755]" | 3440 | 0.195 | 0.5043 | Yes |
| 45 | ADCY5 | adenylate cyclase 5 [Source:HGNC Symbol;Acc:HGNC:236] | 3504 | 0.193 | 0.5095 | Yes |
| 46 | POLA2 | "DNA polymerase alpha 2, accessory subunit [Source:HGNC Symbol;Acc:HGNC:30073]" | 3803 | 0.183 | 0.5085 | Yes |
| 47 | POLR2F | "RNA polymerase II, I and III subunit F [Source:HGNC Symbol;Acc:HGNC:9193]" | 4041 | 0.175 | 0.5087 | Yes |
| 48 | PRUNE1 | prune exopolyphosphatase 1 [Source:HGNC Symbol;Acc:HGNC:13420] | 4265 | 0.169 | 0.5090 | Yes |
| 49 | PAPSS1 | 3'-phosphoadenosine 5'-phosphosulfate synthase 1 [Source:HGNC Symbol;Acc:HGNC:8603] | 4326 | 0.167 | 0.5134 | Yes |
| 50 | POLR2E | "RNA polymerase II, I and III subunit E [Source:HGNC Symbol;Acc:HGNC:9192]" | 4463 | 0.162 | 0.5158 | Yes |
| 51 | GUCY1A2 | guanylate cyclase 1 soluble subunit alpha 2 [Source:HGNC Symbol;Acc:HGNC:4684] | 4652 | 0.157 | 0.5166 | Yes |
| 52 | ADK | adenosine kinase [Source:HGNC Symbol;Acc:HGNC:257] | 4996 | 0.149 | 0.5131 | Yes |
| 53 | GUCY2F | "guanylate cyclase 2F, retinal [Source:HGNC Symbol;Acc:HGNC:4691]" | 5006 | 0.148 | 0.5182 | Yes |
| 54 | IMPDH2 | inosine monophosphate dehydrogenase 2 [Source:HGNC Symbol;Acc:HGNC:6053] | 5115 | 0.146 | 0.5206 | Yes |
| 55 | ENTPD1 | ectonucleoside triphosphate diphosphohydrolase 1 [Source:HGNC Symbol;Acc:HGNC:3363] | 5885 | 0.129 | 0.5056 | Yes |
| 56 | ADCY10 | adenylate cyclase 10 [Source:HGNC Symbol;Acc:HGNC:21285] | 5972 | 0.127 | 0.5080 | Yes |
| 57 | GUCY2D | "guanylate cyclase 2D, retinal [Source:HGNC Symbol;Acc:HGNC:4689]" | 6229 | 0.123 | 0.5058 | Yes |
| 58 | NME5 | NME/NM23 family member 5 [Source:HGNC Symbol;Acc:HGNC:7853] | 6546 | 0.117 | 0.5019 | Yes |
| 59 | POLR1E | RNA polymerase I subunit E [Source:HGNC Symbol;Acc:HGNC:17631] | 6584 | 0.116 | 0.5052 | Yes |
| 60 | GDA | guanine deaminase [Source:HGNC Symbol;Acc:HGNC:4212] | 6708 | 0.114 | 0.5061 | Yes |
| 61 | NME2 | NME/NM23 nucleoside diphosphate kinase 2 [Source:HGNC Symbol;Acc:HGNC:7850] | 6721 | 0.114 | 0.5099 | Yes |
| 62 | ADCY3 | adenylate cyclase 3 [Source:HGNC Symbol;Acc:HGNC:234] | 6745 | 0.114 | 0.5133 | Yes |
| 63 | POLR2K | "RNA polymerase II, I and III subunit K [Source:HGNC Symbol;Acc:HGNC:9198]" | 6857 | 0.112 | 0.5145 | Yes |
| 64 | GMPR2 | guanosine monophosphate reductase 2 [Source:HGNC Symbol;Acc:HGNC:4377] | 6915 | 0.111 | 0.5170 | Yes |
| 65 | ADA | adenosine deaminase [Source:HGNC Symbol;Acc:HGNC:186] | 7066 | 0.108 | 0.5170 | Yes |
| 66 | POLR1H | RNA polymerase I subunit H [Source:HGNC Symbol;Acc:HGNC:13182] | 7108 | 0.108 | 0.5198 | Yes |
| 67 | RRM1 | ribonucleotide reductase catalytic subunit M1 [Source:HGNC Symbol;Acc:HGNC:10451] | 7422 | 0.103 | 0.5155 | Yes |
| 68 | NT5C3A | "5'-nucleotidase, cytosolic IIIA [Source:HGNC Symbol;Acc:HGNC:17820]" | 7449 | 0.102 | 0.5185 | Yes |
| 69 | DCK | deoxycytidine kinase [Source:HGNC Symbol;Acc:HGNC:2704] | 7523 | 0.101 | 0.5202 | Yes |
| 70 | AMPD2 | adenosine monophosphate deaminase 2 [Source:HGNC Symbol;Acc:HGNC:469] | 7589 | 0.100 | 0.5222 | Yes |
| 71 | POLR3D | RNA polymerase III subunit D [Source:HGNC Symbol;Acc:HGNC:1080] | 8093 | 0.094 | 0.5127 | No |
| 72 | NPR2 | natriuretic peptide receptor 2 [Source:HGNC Symbol;Acc:HGNC:7944] | 8425 | 0.090 | 0.5074 | No |
| 73 | GUCY1A1 | guanylate cyclase 1 soluble subunit alpha 1 [Source:HGNC Symbol;Acc:HGNC:4685] | 8447 | 0.089 | 0.5101 | No |
| 74 | ADCY1 | adenylate cyclase 1 [Source:HGNC Symbol;Acc:HGNC:232] | 8649 | 0.087 | 0.5080 | No |
| 75 | IMPDH1 | inosine monophosphate dehydrogenase 1 [Source:HGNC Symbol;Acc:HGNC:6052] | 8669 | 0.086 | 0.5106 | No |
| 76 | ADSS2 | adenylosuccinate synthase 2 [Source:HGNC Symbol;Acc:HGNC:292] | 9307 | 0.078 | 0.4972 | No |
| 77 | PKM | pyruvate kinase M1/2 [Source:HGNC Symbol;Acc:HGNC:9021] | 9357 | 0.078 | 0.4987 | No |
| 78 | POLR2G | RNA polymerase II subunit G [Source:HGNC Symbol;Acc:HGNC:9194] | 9521 | 0.076 | 0.4972 | No |
| 79 | NME1-NME2 | NME1-NME2 readthrough [Source:HGNC Symbol;Acc:HGNC:33531] | 9526 | 0.076 | 0.4999 | No |
| 80 | GMPR | guanosine monophosphate reductase [Source:HGNC Symbol;Acc:HGNC:4376] | 9554 | 0.076 | 0.5019 | No |
| 81 | POLE | "DNA polymerase epsilon, catalytic subunit [Source:HGNC Symbol;Acc:HGNC:9177]" | 9755 | 0.073 | 0.4994 | No |
| 82 | AK7 | adenylate kinase 7 [Source:HGNC Symbol;Acc:HGNC:20091] | 9866 | 0.072 | 0.4992 | No |
| 83 | PDE6C | phosphodiesterase 6C [Source:HGNC Symbol;Acc:HGNC:8787] | 10159 | 0.069 | 0.4942 | No |
| 84 | POLR2I | RNA polymerase II subunit I [Source:HGNC Symbol;Acc:HGNC:9196] | 10437 | 0.066 | 0.4895 | No |
| 85 | HPRT1 | hypoxanthine phosphoribosyltransferase 1 [Source:HGNC Symbol;Acc:HGNC:5157] | 10776 | 0.063 | 0.4831 | No |
| 86 | PDE11A | phosphodiesterase 11A [Source:HGNC Symbol;Acc:HGNC:8773] | 10968 | 0.061 | 0.4804 | No |
| 87 | ENTPD3 | ectonucleoside triphosphate diphosphohydrolase 3 [Source:HGNC Symbol;Acc:HGNC:3365] | 11400 | 0.057 | 0.4714 | No |
| 88 | AMPD1 | adenosine monophosphate deaminase 1 [Source:HGNC Symbol;Acc:HGNC:468] | 11444 | 0.057 | 0.4724 | No |
| 89 | PAPSS2 | 3'-phosphoadenosine 5'-phosphosulfate synthase 2 [Source:HGNC Symbol;Acc:HGNC:8604] | 11897 | 0.053 | 0.4627 | No |
| 90 | POLR2J3 | RNA polymerase II subunit J3 [Source:HGNC Symbol;Acc:HGNC:33853] | 12042 | 0.052 | 0.4609 | No |
| 91 | NT5C1B | "5'-nucleotidase, cytosolic IB [Source:HGNC Symbol;Acc:HGNC:17818]" | 12436 | 0.049 | 0.4526 | No |
| 92 | PRPS1L1 | phosphoribosyl pyrophosphate synthetase 1 like 1 [Source:HGNC Symbol;Acc:HGNC:9463] | 12798 | 0.046 | 0.4451 | No |
| 93 | POLR1C | RNA polymerase I and III subunit C [Source:HGNC Symbol;Acc:HGNC:20194] | 13431 | 0.042 | 0.4304 | No |
| 94 | PDE1A | phosphodiesterase 1A [Source:HGNC Symbol;Acc:HGNC:8774] | 14183 | 0.037 | 0.4126 | No |
| 95 | POLR2J | RNA polymerase II subunit J [Source:HGNC Symbol;Acc:HGNC:9197] | 14754 | 0.034 | 0.3992 | No |
| 96 | NT5C | "5', 3'-nucleotidase, cytosolic [Source:HGNC Symbol;Acc:HGNC:17144]" | 14778 | 0.033 | 0.3998 | No |
| 97 | PDE10A | phosphodiesterase 10A [Source:HGNC Symbol;Acc:HGNC:8772] | 14959 | 0.032 | 0.3964 | No |
| 98 | NME4 | NME/NM23 nucleoside diphosphate kinase 4 [Source:HGNC Symbol;Acc:HGNC:7852] | 15122 | 0.031 | 0.3934 | No |
| 99 | ALLC | allantoicase [Source:HGNC Symbol;Acc:HGNC:17377] | 15489 | 0.029 | 0.3850 | No |
| 100 | PRPS2 | phosphoribosyl pyrophosphate synthetase 2 [Source:HGNC Symbol;Acc:HGNC:9465] | 15580 | 0.028 | 0.3838 | No |
| 101 | POLR2J2 | RNA polymerase II subunit J2 [Source:HGNC Symbol;Acc:HGNC:23208] | 15603 | 0.028 | 0.3842 | No |
| 102 | PRPS1 | phosphoribosyl pyrophosphate synthetase 1 [Source:HGNC Symbol;Acc:HGNC:9462] | 15712 | 0.027 | 0.3824 | No |
| 103 | ENTPD6 | ectonucleoside triphosphate diphosphohydrolase 6 [Source:HGNC Symbol;Acc:HGNC:3368] | 15740 | 0.027 | 0.3827 | No |
| 104 | XDH | xanthine dehydrogenase [Source:HGNC Symbol;Acc:HGNC:12805] | 15854 | 0.027 | 0.3808 | No |
| 105 | PDE6B | phosphodiesterase 6B [Source:HGNC Symbol;Acc:HGNC:8786] | 16707 | 0.022 | 0.3598 | No |
| 106 | NT5C1A | "5'-nucleotidase, cytosolic IA [Source:HGNC Symbol;Acc:HGNC:17819]" | 18501 | 0.012 | 0.3144 | No |
| 107 | DGUOK | deoxyguanosine kinase [Source:HGNC Symbol;Acc:HGNC:2858] | 20124 | 0.003 | 0.2731 | No |
| 108 | AK5 | adenylate kinase 5 [Source:HGNC Symbol;Acc:HGNC:365] | 20575 | 0.000 | 0.2616 | No |
| 109 | ADCY2 | adenylate cyclase 2 [Source:HGNC Symbol;Acc:HGNC:233] | 21361 | -0.004 | 0.2418 | No |
| 110 | PDE6H | phosphodiesterase 6H [Source:HGNC Symbol;Acc:HGNC:8790] | 21410 | -0.004 | 0.2407 | No |
| 111 | ADCY7 | adenylate cyclase 7 [Source:HGNC Symbol;Acc:HGNC:238] | 21812 | -0.007 | 0.2307 | No |
| 112 | GMPS | guanine monophosphate synthase [Source:HGNC Symbol;Acc:HGNC:4378] | 21870 | -0.007 | 0.2295 | No |
| 113 | PDE3B | phosphodiesterase 3B [Source:HGNC Symbol;Acc:HGNC:8779] | 21875 | -0.007 | 0.2296 | No |
| 114 | ADPRM | "ADP-ribose/CDP-alcohol diphosphatase, manganese dependent [Source:HGNC Symbol;Acc:HGNC:30925]" | 22669 | -0.012 | 0.2098 | No |
| 115 | GUCY1B1 | guanylate cyclase 1 soluble subunit beta 1 [Source:HGNC Symbol;Acc:HGNC:4687] | 22768 | -0.012 | 0.2077 | No |
| 116 | POLR2C | RNA polymerase II subunit C [Source:HGNC Symbol;Acc:HGNC:9189] | 24322 | -0.022 | 0.1689 | No |
| 117 | NME3 | NME/NM23 nucleoside diphosphate kinase 3 [Source:HGNC Symbol;Acc:HGNC:7851] | 24778 | -0.025 | 0.1581 | No |
| 118 | GUK1 | guanylate kinase 1 [Source:HGNC Symbol;Acc:HGNC:4693] | 25510 | -0.030 | 0.1406 | No |
| 119 | PDE3A | phosphodiesterase 3A [Source:HGNC Symbol;Acc:HGNC:8778] | 25625 | -0.031 | 0.1387 | No |
| 120 | ENPP1 | ectonucleotide pyrophosphatase/phosphodiesterase 1 [Source:HGNC Symbol;Acc:HGNC:3356] | 27236 | -0.043 | 0.0992 | No |
| 121 | PDE7A | phosphodiesterase 7A [Source:HGNC Symbol;Acc:HGNC:8791] | 27483 | -0.045 | 0.0945 | No |
| 122 | POLE3 | "DNA polymerase epsilon 3, accessory subunit [Source:HGNC Symbol;Acc:HGNC:13546]" | 27729 | -0.048 | 0.0900 | No |
| 123 | ADCY8 | adenylate cyclase 8 [Source:HGNC Symbol;Acc:HGNC:239] | 27844 | -0.049 | 0.0888 | No |
| 124 | ENPP3 | ectonucleotide pyrophosphatase/phosphodiesterase 3 [Source:HGNC Symbol;Acc:HGNC:3358] | 28015 | -0.050 | 0.0863 | No |
| 125 | ADCY9 | adenylate cyclase 9 [Source:HGNC Symbol;Acc:HGNC:240] | 28450 | -0.054 | 0.0771 | No |
| 126 | PDE7B | phosphodiesterase 7B [Source:HGNC Symbol;Acc:HGNC:8792] | 29509 | -0.066 | 0.0525 | No |
| 127 | ADCY6 | adenylate cyclase 6 [Source:HGNC Symbol;Acc:HGNC:237] | 29551 | -0.067 | 0.0538 | No |
| 128 | POLR2A | RNA polymerase II subunit A [Source:HGNC Symbol;Acc:HGNC:9187] | 30331 | -0.076 | 0.0366 | No |
| 129 | PKLR | pyruvate kinase L/R [Source:HGNC Symbol;Acc:HGNC:9020] | 31596 | -0.093 | 0.0077 | No |
| 130 | PDE6G | phosphodiesterase 6G [Source:HGNC Symbol;Acc:HGNC:8789] | 31981 | -0.099 | 0.0014 | No |
| 131 | PDE5A | phosphodiesterase 5A [Source:HGNC Symbol;Acc:HGNC:8784] | 31988 | -0.100 | 0.0048 | No |
| 132 | GUCY2C | guanylate cyclase 2C [Source:HGNC Symbol;Acc:HGNC:4688] | 32035 | -0.100 | 0.0072 | No |
| 133 | PDE8A | phosphodiesterase 8A [Source:HGNC Symbol;Acc:HGNC:8793] | 32199 | -0.103 | 0.0068 | No |
| 134 | PDE4B | phosphodiesterase 4B [Source:HGNC Symbol;Acc:HGNC:8781] | 32641 | -0.112 | -0.0005 | No |
| 135 | URAD | ureidoimidazoline (2-oxo-4-hydroxy-4-carboxy-5-) decarboxylase [Source:HGNC Symbol;Acc:HGNC:17785] | 32804 | -0.115 | -0.0005 | No |
| 136 | PDE1C | phosphodiesterase 1C [Source:HGNC Symbol;Acc:HGNC:8776] | 32833 | -0.116 | 0.0029 | No |
| 137 | CANT1 | calcium activated nucleotidase 1 [Source:HGNC Symbol;Acc:HGNC:19721] | 32873 | -0.117 | 0.0060 | No |
| 138 | AK4 | adenylate kinase 4 [Source:HGNC Symbol;Acc:HGNC:363] | 32917 | -0.117 | 0.0091 | No |
| 139 | ENTPD2 | ectonucleoside triphosphate diphosphohydrolase 2 [Source:HGNC Symbol;Acc:HGNC:3364] | 33031 | -0.120 | 0.0105 | No |
| 140 | PDE9A | phosphodiesterase 9A [Source:HGNC Symbol;Acc:HGNC:8795] | 33115 | -0.121 | 0.0127 | No |
| 141 | NT5E | 5'-nucleotidase ecto [Source:HGNC Symbol;Acc:HGNC:8021] | 33284 | -0.124 | 0.0129 | No |
| 142 | ADSS1 | adenylosuccinate synthase 1 [Source:HGNC Symbol;Acc:HGNC:20093] | 33446 | -0.128 | 0.0133 | No |
| 143 | NPR1 | natriuretic peptide receptor 1 [Source:HGNC Symbol;Acc:HGNC:7943] | 34285 | -0.149 | -0.0027 | No |
| 144 | PDE4C | phosphodiesterase 4C [Source:HGNC Symbol;Acc:HGNC:8782] | 34458 | -0.154 | -0.0016 | No |
| 145 | AK1 | adenylate kinase 1 [Source:HGNC Symbol;Acc:HGNC:361] | 34533 | -0.157 | 0.0021 | No |
| 146 | POLD4 | "DNA polymerase delta 4, accessory subunit [Source:HGNC Symbol;Acc:HGNC:14106]" | 34723 | -0.162 | 0.0031 | No |
| 147 | ENTPD8 | ectonucleoside triphosphate diphosphohydrolase 8 [Source:HGNC Symbol;Acc:HGNC:24860] | 34784 | -0.164 | 0.0074 | No |
| 148 | NT5C2 | "5'-nucleotidase, cytosolic II [Source:HGNC Symbol;Acc:HGNC:8022]" | 35000 | -0.171 | 0.0080 | No |
| 149 | PDE4D | phosphodiesterase 4D [Source:HGNC Symbol;Acc:HGNC:8783] | 35324 | -0.182 | 0.0063 | No |
| 150 | POLR1D | RNA polymerase I and III subunit D [Source:HGNC Symbol;Acc:HGNC:20422] | 35399 | -0.185 | 0.0110 | No |
| 151 | RRM2B | ribonucleotide reductase regulatory TP53 inducible subunit M2B [Source:HGNC Symbol;Acc:HGNC:17296] | 35518 | -0.189 | 0.0147 | No |
| 152 | POLR3F | RNA polymerase III subunit F [Source:HGNC Symbol;Acc:HGNC:15763] | 36319 | -0.221 | 0.0022 | No |
| 153 | PDE1B | phosphodiesterase 1B [Source:HGNC Symbol;Acc:HGNC:8775] | 36526 | -0.232 | 0.0052 | No |
| 154 | POLR2B | RNA polymerase II subunit B [Source:HGNC Symbol;Acc:HGNC:9188] | 37220 | -0.275 | -0.0027 | No |
| 155 | ADCY4 | adenylate cyclase 4 [Source:HGNC Symbol;Acc:HGNC:235] | 37426 | -0.290 | 0.0025 | No |
| 156 | PDE4A | phosphodiesterase 4A [Source:HGNC Symbol;Acc:HGNC:8780] | 37499 | -0.296 | 0.0112 | No |
| 157 | PDE2A | phosphodiesterase 2A [Source:HGNC Symbol;Acc:HGNC:8777] | 37573 | -0.303 | 0.0202 | No |
| 158 | ENTPD4 | ectonucleoside triphosphate diphosphohydrolase 4 [Source:HGNC Symbol;Acc:HGNC:14573] | 37793 | -0.323 | 0.0261 | No |
| 159 | AMPD3 | adenosine monophosphate deaminase 3 [Source:HGNC Symbol;Acc:HGNC:470] | 38097 | -0.362 | 0.0313 | No |
Table: GSEA details [plain text format]

  

Fig 2: KEGG\_PURINE\_METABOLISM      
 Blue-Pink O' Gram in the Space of the Analyzed GeneSet

  

Fig 3: KEGG\_PURINE\_METABOLISM: Random ES distribution      
 Gene set null distribution of ES for **KEGG\_PURINE\_METABOLISM**
