# Supplementary material for: Simultaneous Presentation of Multiple Myeloma and Lung Cancer: Case Report and Gene Bioinformatics Analysis
Source: Front Oncol. 2022 Jun 13;12:859735. doi: 10.3389/fonc.2022.859735 (PMC9235397; doi:10.3389/fonc.2022.859735)
Supplement: Supplementary file 1 [file DataSheet_1.zip › The bioinformatic analysis of MM and lung cancer supplementary materials/Enrichment analysis/MECR/GSEA_4.1.0/MM TCGA/KEGG.Gsea.1639040591792/KEGG_PYRIMIDINE_METABOLISM.html]

Details for gene set KEGG\_PYRIMIDINE\_METABOLISM[GSEA]

|  || Dataset | ExpData\_collapsed\_to\_symbols.ENSG00000116353\_profile\_in\_ExpData.cls #ENSG00000116353 |
| Phenotype | ENSG00000116353\_profile\_in\_ExpData.cls#ENSG00000116353 |
| Upregulated in class | ENSG00000116353\_pos |
| GeneSet | KEGG\_PYRIMIDINE\_METABOLISM |
| Enrichment Score (ES) | 0.60294807 |
| Normalized Enrichment Score (NES) | 2.1940742 |
| Nominal p-value | 0.0 |
| FDR q-value | 0.0 |
| FWER p-Value | 0.0 |
Table: GSEA Results Summary

  

Fig 1: Enrichment plot: KEGG\_PYRIMIDINE\_METABOLISM      
 Profile of the Running ES Score & Positions of GeneSet Members on the Rank Ordered List

  

| SYMBOL | TITLE | RANK IN GENE LIST | RANK METRIC SCORE | RUNNING ES | CORE ENRICHMENT || 1 | DHODH | dihydroorotate dehydrogenase (quinone) [Source:HGNC Symbol;Acc:HGNC:2867] | 5 | 0.640 | 0.0298 | Yes |
| 2 | POLD2 | "DNA polymerase delta 2, accessory subunit [Source:HGNC Symbol;Acc:HGNC:9176]" | 15 | 0.617 | 0.0585 | Yes |
| 3 | CTPS1 | CTP synthase 1 [Source:HGNC Symbol;Acc:HGNC:2519] | 23 | 0.602 | 0.0865 | Yes |
| 4 | CAD | "carbamoyl-phosphate synthetase 2, aspartate transcarbamylase, and dihydroorotase [Source:HGNC Symbol;Acc:HGNC:1424]" | 36 | 0.584 | 0.1134 | Yes |
| 5 | POLR3K | RNA polymerase III subunit K [Source:HGNC Symbol;Acc:HGNC:14121] | 61 | 0.559 | 0.1390 | Yes |
| 6 | DTYMK | deoxythymidylate kinase [Source:HGNC Symbol;Acc:HGNC:3061] | 72 | 0.549 | 0.1644 | Yes |
| 7 | POLR1A | RNA polymerase I subunit A [Source:HGNC Symbol;Acc:HGNC:17264] | 155 | 0.501 | 0.1857 | Yes |
| 8 | POLR3H | RNA polymerase III subunit H [Source:HGNC Symbol;Acc:HGNC:30349] | 156 | 0.501 | 0.2092 | Yes |
| 9 | POLR3B | RNA polymerase III subunit B [Source:HGNC Symbol;Acc:HGNC:30348] | 185 | 0.490 | 0.2314 | Yes |
| 10 | ENTPD5 | ectonucleoside triphosphate diphosphohydrolase 5 (inactive) [Source:HGNC Symbol;Acc:HGNC:3367] | 220 | 0.478 | 0.2529 | Yes |
| 11 | NME1 | NME/NM23 nucleoside diphosphate kinase 1 [Source:HGNC Symbol;Acc:HGNC:7849] | 240 | 0.471 | 0.2744 | Yes |
| 12 | PNPT1 | polyribonucleotide nucleotidyltransferase 1 [Source:HGNC Symbol;Acc:HGNC:23166] | 385 | 0.437 | 0.2912 | Yes |
| 13 | UMPS | uridine monophosphate synthetase [Source:HGNC Symbol;Acc:HGNC:12563] | 416 | 0.431 | 0.3106 | Yes |
| 14 | ITPA | inosine triphosphatase [Source:HGNC Symbol;Acc:HGNC:6176] | 469 | 0.418 | 0.3288 | Yes |
| 15 | POLD1 | "DNA polymerase delta 1, catalytic subunit [Source:HGNC Symbol;Acc:HGNC:9175]" | 541 | 0.405 | 0.3460 | Yes |
| 16 | RRM2 | ribonucleotide reductase regulatory subunit M2 [Source:HGNC Symbol;Acc:HGNC:10452] | 572 | 0.402 | 0.3640 | Yes |
| 17 | PRIM2 | DNA primase subunit 2 [Source:HGNC Symbol;Acc:HGNC:9370] | 602 | 0.397 | 0.3818 | Yes |
| 18 | POLA1 | "DNA polymerase alpha 1, catalytic subunit [Source:HGNC Symbol;Acc:HGNC:9173]" | 652 | 0.390 | 0.3988 | Yes |
| 19 | POLR1B | RNA polymerase I subunit B [Source:HGNC Symbol;Acc:HGNC:20454] | 735 | 0.377 | 0.4143 | Yes |
| 20 | POLR3GL | RNA polymerase III subunit GL [Source:HGNC Symbol;Acc:HGNC:28466] | 745 | 0.376 | 0.4317 | Yes |
| 21 | NME6 | NME/NM23 nucleoside diphosphate kinase 6 [Source:HGNC Symbol;Acc:HGNC:20567] | 900 | 0.354 | 0.4443 | Yes |
| 22 | UCK2 | uridine-cytidine kinase 2 [Source:HGNC Symbol;Acc:HGNC:12562] | 951 | 0.349 | 0.4594 | Yes |
| 23 | POLR2H | "RNA polymerase II, I and III subunit H [Source:HGNC Symbol;Acc:HGNC:9195]" | 1100 | 0.335 | 0.4713 | Yes |
| 24 | PRIM1 | DNA primase subunit 1 [Source:HGNC Symbol;Acc:HGNC:9369] | 1113 | 0.334 | 0.4866 | Yes |
| 25 | NME7 | NME/NM23 family member 7 [Source:HGNC Symbol;Acc:HGNC:20461] | 1219 | 0.323 | 0.4990 | Yes |
| 26 | DUT | deoxyuridine triphosphatase [Source:HGNC Symbol;Acc:HGNC:3078] | 1278 | 0.318 | 0.5124 | Yes |
| 27 | TYMS | thymidylate synthetase [Source:HGNC Symbol;Acc:HGNC:12441] | 1461 | 0.302 | 0.5219 | Yes |
| 28 | POLR3G | RNA polymerase III subunit G [Source:HGNC Symbol;Acc:HGNC:30075] | 1523 | 0.298 | 0.5343 | Yes |
| 29 | POLR3C | RNA polymerase III subunit C [Source:HGNC Symbol;Acc:HGNC:30076] | 1610 | 0.291 | 0.5457 | Yes |
| 30 | POLD3 | "DNA polymerase delta 3, accessory subunit [Source:HGNC Symbol;Acc:HGNC:20932]" | 1700 | 0.284 | 0.5567 | Yes |
| 31 | POLR2L | "RNA polymerase II, I and III subunit L [Source:HGNC Symbol;Acc:HGNC:9199]" | 1839 | 0.274 | 0.5660 | Yes |
| 32 | NT5M | "5',3'-nucleotidase, mitochondrial [Source:HGNC Symbol;Acc:HGNC:15769]" | 2250 | 0.250 | 0.5672 | Yes |
| 33 | NUDT2 | nudix hydrolase 2 [Source:HGNC Symbol;Acc:HGNC:8049] | 2659 | 0.229 | 0.5675 | Yes |
| 34 | PNP | purine nucleoside phosphorylase [Source:HGNC Symbol;Acc:HGNC:7892] | 2797 | 0.222 | 0.5744 | Yes |
| 35 | POLR3A | RNA polymerase III subunit A [Source:HGNC Symbol;Acc:HGNC:30074] | 3031 | 0.212 | 0.5784 | Yes |
| 36 | POLR2D | RNA polymerase II subunit D [Source:HGNC Symbol;Acc:HGNC:9191] | 3081 | 0.210 | 0.5869 | Yes |
| 37 | POLE2 | "DNA polymerase epsilon 2, accessory subunit [Source:HGNC Symbol;Acc:HGNC:9178]" | 3128 | 0.208 | 0.5955 | Yes |
| 38 | POLE4 | "DNA polymerase epsilon 4, accessory subunit [Source:HGNC Symbol;Acc:HGNC:18755]" | 3440 | 0.195 | 0.5967 | Yes |
| 39 | POLA2 | "DNA polymerase alpha 2, accessory subunit [Source:HGNC Symbol;Acc:HGNC:30073]" | 3803 | 0.183 | 0.5960 | Yes |
| 40 | POLR2F | "RNA polymerase II, I and III subunit F [Source:HGNC Symbol;Acc:HGNC:9193]" | 4041 | 0.175 | 0.5982 | Yes |
| 41 | TXNRD2 | thioredoxin reductase 2 [Source:HGNC Symbol;Acc:HGNC:18155] | 4257 | 0.169 | 0.6006 | Yes |
| 42 | POLR2E | "RNA polymerase II, I and III subunit E [Source:HGNC Symbol;Acc:HGNC:9192]" | 4463 | 0.162 | 0.6029 | Yes |
| 43 | TK2 | thymidine kinase 2 [Source:HGNC Symbol;Acc:HGNC:11831] | 5852 | 0.130 | 0.5736 | No |
| 44 | ENTPD1 | ectonucleoside triphosphate diphosphohydrolase 1 [Source:HGNC Symbol;Acc:HGNC:3363] | 5885 | 0.129 | 0.5789 | No |
| 45 | TXNRD1 | thioredoxin reductase 1 [Source:HGNC Symbol;Acc:HGNC:12437] | 6356 | 0.120 | 0.5725 | No |
| 46 | NME5 | NME/NM23 family member 5 [Source:HGNC Symbol;Acc:HGNC:7853] | 6546 | 0.117 | 0.5732 | No |
| 47 | POLR1E | RNA polymerase I subunit E [Source:HGNC Symbol;Acc:HGNC:17631] | 6584 | 0.116 | 0.5777 | No |
| 48 | NME2 | NME/NM23 nucleoside diphosphate kinase 2 [Source:HGNC Symbol;Acc:HGNC:7850] | 6721 | 0.114 | 0.5795 | No |
| 49 | POLR2K | "RNA polymerase II, I and III subunit K [Source:HGNC Symbol;Acc:HGNC:9198]" | 6857 | 0.112 | 0.5813 | No |
| 50 | POLR1H | RNA polymerase I subunit H [Source:HGNC Symbol;Acc:HGNC:13182] | 7108 | 0.108 | 0.5800 | No |
| 51 | TK1 | thymidine kinase 1 [Source:HGNC Symbol;Acc:HGNC:11830] | 7169 | 0.107 | 0.5834 | No |
| 52 | RRM1 | ribonucleotide reductase catalytic subunit M1 [Source:HGNC Symbol;Acc:HGNC:10451] | 7422 | 0.103 | 0.5818 | No |
| 53 | NT5C3A | "5'-nucleotidase, cytosolic IIIA [Source:HGNC Symbol;Acc:HGNC:17820]" | 7449 | 0.102 | 0.5859 | No |
| 54 | DCK | deoxycytidine kinase [Source:HGNC Symbol;Acc:HGNC:2704] | 7523 | 0.101 | 0.5888 | No |
| 55 | UPP2 | uridine phosphorylase 2 [Source:HGNC Symbol;Acc:HGNC:23061] | 7688 | 0.099 | 0.5892 | No |
| 56 | POLR3D | RNA polymerase III subunit D [Source:HGNC Symbol;Acc:HGNC:1080] | 8093 | 0.094 | 0.5833 | No |
| 57 | POLR2G | RNA polymerase II subunit G [Source:HGNC Symbol;Acc:HGNC:9194] | 9521 | 0.076 | 0.5505 | No |
| 58 | NME1-NME2 | NME1-NME2 readthrough [Source:HGNC Symbol;Acc:HGNC:33531] | 9526 | 0.076 | 0.5539 | No |
| 59 | POLE | "DNA polymerase epsilon, catalytic subunit [Source:HGNC Symbol;Acc:HGNC:9177]" | 9755 | 0.073 | 0.5515 | No |
| 60 | POLR2I | RNA polymerase II subunit I [Source:HGNC Symbol;Acc:HGNC:9196] | 10437 | 0.066 | 0.5373 | No |
| 61 | ENTPD3 | ectonucleoside triphosphate diphosphohydrolase 3 [Source:HGNC Symbol;Acc:HGNC:3365] | 11400 | 0.057 | 0.5154 | No |
| 62 | POLR2J3 | RNA polymerase II subunit J3 [Source:HGNC Symbol;Acc:HGNC:33853] | 12042 | 0.052 | 0.5015 | No |
| 63 | NT5C1B | "5'-nucleotidase, cytosolic IB [Source:HGNC Symbol;Acc:HGNC:17818]" | 12436 | 0.049 | 0.4938 | No |
| 64 | POLR1C | RNA polymerase I and III subunit C [Source:HGNC Symbol;Acc:HGNC:20194] | 13431 | 0.042 | 0.4704 | No |
| 65 | UCKL1 | uridine-cytidine kinase 1 like 1 [Source:HGNC Symbol;Acc:HGNC:15938] | 13854 | 0.039 | 0.4615 | No |
| 66 | AK3 | adenylate kinase 3 [Source:HGNC Symbol;Acc:HGNC:17376] | 13889 | 0.039 | 0.4624 | No |
| 67 | POLR2J | RNA polymerase II subunit J [Source:HGNC Symbol;Acc:HGNC:9197] | 14754 | 0.034 | 0.4419 | No |
| 68 | NT5C | "5', 3'-nucleotidase, cytosolic [Source:HGNC Symbol;Acc:HGNC:17144]" | 14778 | 0.033 | 0.4429 | No |
| 69 | NME4 | NME/NM23 nucleoside diphosphate kinase 4 [Source:HGNC Symbol;Acc:HGNC:7852] | 15122 | 0.031 | 0.4356 | No |
| 70 | POLR2J2 | RNA polymerase II subunit J2 [Source:HGNC Symbol;Acc:HGNC:23208] | 15603 | 0.028 | 0.4247 | No |
| 71 | ENTPD6 | ectonucleoside triphosphate diphosphohydrolase 6 [Source:HGNC Symbol;Acc:HGNC:3368] | 15740 | 0.027 | 0.4225 | No |
| 72 | DPYS | dihydropyrimidinase [Source:HGNC Symbol;Acc:HGNC:3013] | 18395 | 0.012 | 0.3554 | No |
| 73 | NT5C1A | "5'-nucleotidase, cytosolic IA [Source:HGNC Symbol;Acc:HGNC:17819]" | 18501 | 0.012 | 0.3533 | No |
| 74 | POLR2C | RNA polymerase II subunit C [Source:HGNC Symbol;Acc:HGNC:9189] | 24322 | -0.022 | 0.2060 | No |
| 75 | NME3 | NME/NM23 nucleoside diphosphate kinase 3 [Source:HGNC Symbol;Acc:HGNC:7851] | 24778 | -0.025 | 0.1955 | No |
| 76 | CMPK2 | cytidine/uridine monophosphate kinase 2 [Source:HGNC Symbol;Acc:HGNC:27015] | 24945 | -0.026 | 0.1925 | No |
| 77 | CTPS2 | CTP synthase 2 [Source:HGNC Symbol;Acc:HGNC:2520] | 26002 | -0.034 | 0.1672 | No |
| 78 | DPYD | dihydropyrimidine dehydrogenase [Source:HGNC Symbol;Acc:HGNC:3012] | 26860 | -0.040 | 0.1472 | No |
| 79 | CMPK1 | cytidine/uridine monophosphate kinase 1 [Source:HGNC Symbol;Acc:HGNC:18170] | 27252 | -0.043 | 0.1393 | No |
| 80 | POLE3 | "DNA polymerase epsilon 3, accessory subunit [Source:HGNC Symbol;Acc:HGNC:13546]" | 27729 | -0.048 | 0.1294 | No |
| 81 | POLR2A | RNA polymerase II subunit A [Source:HGNC Symbol;Acc:HGNC:9187] | 30331 | -0.076 | 0.0666 | No |
| 82 | CANT1 | calcium activated nucleotidase 1 [Source:HGNC Symbol;Acc:HGNC:19721] | 32873 | -0.117 | 0.0073 | No |
| 83 | TYMP | thymidine phosphorylase [Source:HGNC Symbol;Acc:HGNC:3148] | 32895 | -0.117 | 0.0122 | No |
| 84 | NT5E | 5'-nucleotidase ecto [Source:HGNC Symbol;Acc:HGNC:8021] | 33284 | -0.124 | 0.0082 | No |
| 85 | CDA | cytidine deaminase [Source:HGNC Symbol;Acc:HGNC:1712] | 34110 | -0.144 | -0.0061 | No |
| 86 | POLD4 | "DNA polymerase delta 4, accessory subunit [Source:HGNC Symbol;Acc:HGNC:14106]" | 34723 | -0.162 | -0.0142 | No |
| 87 | ENTPD8 | ectonucleoside triphosphate diphosphohydrolase 8 [Source:HGNC Symbol;Acc:HGNC:24860] | 34784 | -0.164 | -0.0080 | No |
| 88 | NT5C2 | "5'-nucleotidase, cytosolic II [Source:HGNC Symbol;Acc:HGNC:8022]" | 35000 | -0.171 | -0.0055 | No |
| 89 | DCTD | dCMP deaminase [Source:HGNC Symbol;Acc:HGNC:2710] | 35030 | -0.172 | 0.0018 | No |
| 90 | POLR1D | RNA polymerase I and III subunit D [Source:HGNC Symbol;Acc:HGNC:20422] | 35399 | -0.185 | 0.0010 | No |
| 91 | RRM2B | ribonucleotide reductase regulatory TP53 inducible subunit M2B [Source:HGNC Symbol;Acc:HGNC:17296] | 35518 | -0.189 | 0.0069 | No |
| 92 | POLR3F | RNA polymerase III subunit F [Source:HGNC Symbol;Acc:HGNC:15763] | 36319 | -0.221 | -0.0032 | No |
| 93 | UPRT | uracil phosphoribosyltransferase homolog [Source:HGNC Symbol;Acc:HGNC:28334] | 36348 | -0.223 | 0.0065 | No |
| 94 | UPP1 | uridine phosphorylase 1 [Source:HGNC Symbol;Acc:HGNC:12576] | 37037 | -0.262 | 0.0012 | No |
| 95 | POLR2B | RNA polymerase II subunit B [Source:HGNC Symbol;Acc:HGNC:9188] | 37220 | -0.275 | 0.0094 | No |
| 96 | UCK1 | uridine-cytidine kinase 1 [Source:HGNC Symbol;Acc:HGNC:14859] | 37329 | -0.282 | 0.0199 | No |
| 97 | ENTPD4 | ectonucleoside triphosphate diphosphohydrolase 4 [Source:HGNC Symbol;Acc:HGNC:14573] | 37793 | -0.323 | 0.0232 | No |
| 98 | UPB1 | beta-ureidopropionase 1 [Source:HGNC Symbol;Acc:HGNC:16297] | 37908 | -0.338 | 0.0361 | No |
Table: GSEA details [plain text format]

  

Fig 2: KEGG\_PYRIMIDINE\_METABOLISM      
 Blue-Pink O' Gram in the Space of the Analyzed GeneSet

  

Fig 3: KEGG\_PYRIMIDINE\_METABOLISM: Random ES distribution      
 Gene set null distribution of ES for **KEGG\_PYRIMIDINE\_METABOLISM**
