# Supplementary material for: Simultaneous Presentation of Multiple Myeloma and Lung Cancer: Case Report and Gene Bioinformatics Analysis
Source: Front Oncol. 2022 Jun 13;12:859735. doi: 10.3389/fonc.2022.859735 (PMC9235397; doi:10.3389/fonc.2022.859735)
Supplement: Supplementary file 1 [file DataSheet_1.zip › The bioinformatic analysis of MM and lung cancer supplementary materials/Enrichment analysis/MECR/GSEA_4.1.0/MM TCGA/KEGG.Gsea.1639040591792/KEGG_PYRUVATE_METABOLISM.html]

Details for gene set KEGG\_PYRUVATE\_METABOLISM[GSEA]

|  || Dataset | ExpData\_collapsed\_to\_symbols.ENSG00000116353\_profile\_in\_ExpData.cls #ENSG00000116353 |
| Phenotype | ENSG00000116353\_profile\_in\_ExpData.cls#ENSG00000116353 |
| Upregulated in class | ENSG00000116353\_pos |
| GeneSet | KEGG\_PYRUVATE\_METABOLISM |
| Enrichment Score (ES) | 0.65303767 |
| Normalized Enrichment Score (NES) | 2.049095 |
| Nominal p-value | 0.0 |
| FDR q-value | 1.7763038E-4 |
| FWER p-Value | 0.003 |
Table: GSEA Results Summary

  

Fig 1: Enrichment plot: KEGG\_PYRUVATE\_METABOLISM      
 Profile of the Running ES Score & Positions of GeneSet Members on the Rank Ordered List

  

| SYMBOL | TITLE | RANK IN GENE LIST | RANK METRIC SCORE | RUNNING ES | CORE ENRICHMENT || 1 | PDHA1 | pyruvate dehydrogenase E1 subunit alpha 1 [Source:HGNC Symbol;Acc:HGNC:8806] | 223 | 0.477 | 0.0553 | Yes |
| 2 | MDH2 | malate dehydrogenase 2 [Source:HGNC Symbol;Acc:HGNC:6971] | 319 | 0.451 | 0.1104 | Yes |
| 3 | ALDH1B1 | aldehyde dehydrogenase 1 family member B1 [Source:HGNC Symbol;Acc:HGNC:407] | 372 | 0.439 | 0.1651 | Yes |
| 4 | MDH1 | malate dehydrogenase 1 [Source:HGNC Symbol;Acc:HGNC:6970] | 594 | 0.398 | 0.2103 | Yes |
| 5 | ALDH3A2 | aldehyde dehydrogenase 3 family member A2 [Source:HGNC Symbol;Acc:HGNC:403] | 902 | 0.354 | 0.2477 | Yes |
| 6 | DLAT | dihydrolipoamide S-acetyltransferase [Source:HGNC Symbol;Acc:HGNC:2896] | 998 | 0.345 | 0.2893 | Yes |
| 7 | ME2 | malic enzyme 2 [Source:HGNC Symbol;Acc:HGNC:6984] | 1034 | 0.341 | 0.3319 | Yes |
| 8 | ACYP1 | acylphosphatase 1 [Source:HGNC Symbol;Acc:HGNC:179] | 1091 | 0.336 | 0.3733 | Yes |
| 9 | LDHB | lactate dehydrogenase B [Source:HGNC Symbol;Acc:HGNC:6541] | 1375 | 0.309 | 0.4055 | Yes |
| 10 | ALDH9A1 | aldehyde dehydrogenase 9 family member A1 [Source:HGNC Symbol;Acc:HGNC:412] | 1406 | 0.306 | 0.4438 | Yes |
| 11 | ACAT1 | acetyl-CoA acetyltransferase 1 [Source:HGNC Symbol;Acc:HGNC:93] | 1467 | 0.302 | 0.4808 | Yes |
| 12 | HAGH | hydroxyacylglutathione hydrolase [Source:HGNC Symbol;Acc:HGNC:4805] | 1574 | 0.293 | 0.5156 | Yes |
| 13 | GRHPR | glyoxylate and hydroxypyruvate reductase [Source:HGNC Symbol;Acc:HGNC:4570] | 1724 | 0.282 | 0.5477 | Yes |
| 14 | ACACA | acetyl-CoA carboxylase alpha [Source:HGNC Symbol;Acc:HGNC:84] | 1905 | 0.270 | 0.5776 | Yes |
| 15 | PDHB | pyruvate dehydrogenase E1 subunit beta [Source:HGNC Symbol;Acc:HGNC:8808] | 2053 | 0.261 | 0.6072 | Yes |
| 16 | ACSS1 | acyl-CoA synthetase short chain family member 1 [Source:HGNC Symbol;Acc:HGNC:16091] | 2776 | 0.223 | 0.6174 | Yes |
| 17 | LDHAL6A | lactate dehydrogenase A like 6A [Source:HGNC Symbol;Acc:HGNC:28335] | 3170 | 0.206 | 0.6337 | Yes |
| 18 | DLD | dihydrolipoamide dehydrogenase [Source:HGNC Symbol;Acc:HGNC:2898] | 3616 | 0.189 | 0.6465 | Yes |
| 19 | LDHA | lactate dehydrogenase A [Source:HGNC Symbol;Acc:HGNC:6535] | 4425 | 0.164 | 0.6468 | Yes |
| 20 | PCK2 | "phosphoenolpyruvate carboxykinase 2, mitochondrial [Source:HGNC Symbol;Acc:HGNC:8725]" | 4933 | 0.150 | 0.6530 | Yes |
| 21 | ME3 | malic enzyme 3 [Source:HGNC Symbol;Acc:HGNC:6985] | 6893 | 0.111 | 0.6174 | No |
| 22 | LDHD | lactate dehydrogenase D [Source:HGNC Symbol;Acc:HGNC:19708] | 6946 | 0.110 | 0.6301 | No |
| 23 | ACSS2 | acyl-CoA synthetase short chain family member 2 [Source:HGNC Symbol;Acc:HGNC:15814] | 7325 | 0.104 | 0.6338 | No |
| 24 | PDHA2 | pyruvate dehydrogenase E1 subunit alpha 2 [Source:HGNC Symbol;Acc:HGNC:8807] | 8806 | 0.085 | 0.6069 | No |
| 25 | PKM | pyruvate kinase M1/2 [Source:HGNC Symbol;Acc:HGNC:9021] | 9357 | 0.078 | 0.6028 | No |
| 26 | HAGHL | hydroxyacylglutathione hydrolase like [Source:HGNC Symbol;Acc:HGNC:14177] | 10231 | 0.068 | 0.5893 | No |
| 27 | ACAT2 | acetyl-CoA acetyltransferase 2 [Source:HGNC Symbol;Acc:HGNC:94] | 10235 | 0.068 | 0.5980 | No |
| 28 | ACOT12 | acyl-CoA thioesterase 12 [Source:HGNC Symbol;Acc:HGNC:24436] | 10575 | 0.065 | 0.5976 | No |
| 29 | ACYP2 | acylphosphatase 2 [Source:HGNC Symbol;Acc:HGNC:180] | 11931 | 0.053 | 0.5699 | No |
| 30 | PC | pyruvate carboxylase [Source:HGNC Symbol;Acc:HGNC:8636] | 12295 | 0.050 | 0.5670 | No |
| 31 | ALDH7A1 | aldehyde dehydrogenase 7 family member A1 [Source:HGNC Symbol;Acc:HGNC:877] | 14303 | 0.036 | 0.5206 | No |
| 32 | ME1 | malic enzyme 1 [Source:HGNC Symbol;Acc:HGNC:6983] | 19405 | 0.007 | 0.3916 | No |
| 33 | LDHAL6B | lactate dehydrogenase A like 6B [Source:HGNC Symbol;Acc:HGNC:21481] | 20658 | -0.000 | 0.3597 | No |
| 34 | AKR1B1 | aldo-keto reductase family 1 member B [Source:HGNC Symbol;Acc:HGNC:381] | 28964 | -0.060 | 0.1560 | No |
| 35 | LDHC | lactate dehydrogenase C [Source:HGNC Symbol;Acc:HGNC:6544] | 29290 | -0.064 | 0.1559 | No |
| 36 | PKLR | pyruvate kinase L/R [Source:HGNC Symbol;Acc:HGNC:9020] | 31596 | -0.093 | 0.1090 | No |
| 37 | ACACB | acetyl-CoA carboxylase beta [Source:HGNC Symbol;Acc:HGNC:85] | 33834 | -0.137 | 0.0696 | No |
| 38 | ALDH2 | aldehyde dehydrogenase 2 family member [Source:HGNC Symbol;Acc:HGNC:404] | 34564 | -0.157 | 0.0712 | No |
| 39 | PCK1 | phosphoenolpyruvate carboxykinase 1 [Source:HGNC Symbol;Acc:HGNC:8724] | 35185 | -0.177 | 0.0780 | No |
| 40 | GLO1 | glyoxalase I [Source:HGNC Symbol;Acc:HGNC:4323] | 36161 | -0.214 | 0.0805 | No |
Table: GSEA details [plain text format]

  

Fig 2: KEGG\_PYRUVATE\_METABOLISM      
 Blue-Pink O' Gram in the Space of the Analyzed GeneSet

  

Fig 3: KEGG\_PYRUVATE\_METABOLISM: Random ES distribution      
 Gene set null distribution of ES for **KEGG\_PYRUVATE\_METABOLISM**
