# Supplementary material for: Simultaneous Presentation of Multiple Myeloma and Lung Cancer: Case Report and Gene Bioinformatics Analysis
Source: Front Oncol. 2022 Jun 13;12:859735. doi: 10.3389/fonc.2022.859735 (PMC9235397; doi:10.3389/fonc.2022.859735)
Supplement: Supplementary file 1 [file DataSheet_1.zip › The bioinformatic analysis of MM and lung cancer supplementary materials/Enrichment analysis/MECR/GSEA_4.1.0/MM TCGA/KEGG.Gsea.1639040591792/KEGG_REGULATION_OF_ACTIN_CYTOSKELETON.html]

Details for gene set KEGG\_REGULATION\_OF\_ACTIN\_CYTOSKELETON[GSEA]

|  || Dataset | ExpData\_collapsed\_to\_symbols.ENSG00000116353\_profile\_in\_ExpData.cls #ENSG00000116353 |
| Phenotype | ENSG00000116353\_profile\_in\_ExpData.cls#ENSG00000116353 |
| Upregulated in class | ENSG00000116353\_neg |
| GeneSet | KEGG\_REGULATION\_OF\_ACTIN\_CYTOSKELETON |
| Enrichment Score (ES) | -0.53249735 |
| Normalized Enrichment Score (NES) | -2.3765829 |
| Nominal p-value | 0.0 |
| FDR q-value | 0.0 |
| FWER p-Value | 0.0 |
Table: GSEA Results Summary

  

Fig 1: Enrichment plot: KEGG\_REGULATION\_OF\_ACTIN\_CYTOSKELETON      
 Profile of the Running ES Score & Positions of GeneSet Members on the Rank Ordered List

  

| SYMBOL | TITLE | RANK IN GENE LIST | RANK METRIC SCORE | RUNNING ES | CORE ENRICHMENT || 1 | PIP4K2B | phosphatidylinositol-5-phosphate 4-kinase type 2 beta [Source:HGNC Symbol;Acc:HGNC:8998] | 458 | 0.421 | 0.0024 | No |
| 2 | BDKRB1 | bradykinin receptor B1 [Source:HGNC Symbol;Acc:HGNC:1029] | 1120 | 0.333 | -0.0034 | No |
| 3 | PIK3R2 | phosphoinositide-3-kinase regulatory subunit 2 [Source:HGNC Symbol;Acc:HGNC:8980] | 1248 | 0.320 | 0.0041 | No |
| 4 | CHRM3 | cholinergic receptor muscarinic 3 [Source:HGNC Symbol;Acc:HGNC:1952] | 1764 | 0.278 | 0.0002 | No |
| 5 | PAK5 | p21 (RAC1) activated kinase 5 [Source:HGNC Symbol;Acc:HGNC:15916] | 2247 | 0.250 | -0.0038 | No |
| 6 | PIK3R3 | phosphoinositide-3-kinase regulatory subunit 3 [Source:HGNC Symbol;Acc:HGNC:8981] | 2602 | 0.231 | -0.0051 | No |
| 7 | CSK | C-terminal Src kinase [Source:HGNC Symbol;Acc:HGNC:2444] | 3052 | 0.211 | -0.0095 | No |
| 8 | IQGAP3 | IQ motif containing GTPase activating protein 3 [Source:HGNC Symbol;Acc:HGNC:20669] | 3356 | 0.199 | -0.0106 | No |
| 9 | VAV1 | vav guanine nucleotide exchange factor 1 [Source:HGNC Symbol;Acc:HGNC:12657] | 3764 | 0.184 | -0.0149 | No |
| 10 | ITGA7 | integrin subunit alpha 7 [Source:HGNC Symbol;Acc:HGNC:6143] | 3910 | 0.179 | -0.0126 | No |
| 11 | DIAPH3 | diaphanous related formin 3 [Source:HGNC Symbol;Acc:HGNC:15480] | 4049 | 0.175 | -0.0102 | No |
| 12 | MAP2K2 | mitogen-activated protein kinase kinase 2 [Source:HGNC Symbol;Acc:HGNC:6842] | 5104 | 0.146 | -0.0323 | No |
| 13 | ITGA10 | integrin subunit alpha 10 [Source:HGNC Symbol;Acc:HGNC:6135] | 5274 | 0.142 | -0.0319 | No |
| 14 | PIP5K1A | phosphatidylinositol-4-phosphate 5-kinase type 1 alpha [Source:HGNC Symbol;Acc:HGNC:8994] | 5499 | 0.137 | -0.0330 | No |
| 15 | FGF14 | fibroblast growth factor 14 [Source:HGNC Symbol;Acc:HGNC:3671] | 5600 | 0.135 | -0.0311 | No |
| 16 | ITGA3 | integrin subunit alpha 3 [Source:HGNC Symbol;Acc:HGNC:6139] | 5916 | 0.129 | -0.0348 | No |
| 17 | RAC2 | Rac family small GTPase 2 [Source:HGNC Symbol;Acc:HGNC:9802] | 6127 | 0.124 | -0.0360 | No |
| 18 | ARPC1A | actin related protein 2/3 complex subunit 1A [Source:HGNC Symbol;Acc:HGNC:703] | 6397 | 0.120 | -0.0389 | No |
| 19 | FGF13 | fibroblast growth factor 13 [Source:HGNC Symbol;Acc:HGNC:3670] | 6443 | 0.119 | -0.0361 | No |
| 20 | FGFR4 | fibroblast growth factor receptor 4 [Source:HGNC Symbol;Acc:HGNC:3691] | 6714 | 0.114 | -0.0392 | No |
| 21 | PIP5K1B | phosphatidylinositol-4-phosphate 5-kinase type 1 beta [Source:HGNC Symbol;Acc:HGNC:8995] | 7022 | 0.109 | -0.0434 | No |
| 22 | PIK3CB | "phosphatidylinositol-4,5-bisphosphate 3-kinase catalytic subunit beta [Source:HGNC Symbol;Acc:HGNC:8976]" | 7716 | 0.099 | -0.0578 | No |
| 23 | PAK2 | p21 (RAC1) activated kinase 2 [Source:HGNC Symbol;Acc:HGNC:8591] | 7725 | 0.099 | -0.0547 | No |
| 24 | MYLK2 | myosin light chain kinase 2 [Source:HGNC Symbol;Acc:HGNC:16243] | 7785 | 0.098 | -0.0529 | No |
| 25 | PFN4 | profilin family member 4 [Source:HGNC Symbol;Acc:HGNC:31103] | 7879 | 0.097 | -0.0521 | No |
| 26 | ARPC5 | actin related protein 2/3 complex subunit 5 [Source:HGNC Symbol;Acc:HGNC:708] | 8270 | 0.091 | -0.0590 | No |
| 27 | ITGB6 | integrin subunit beta 6 [Source:HGNC Symbol;Acc:HGNC:6161] | 8339 | 0.090 | -0.0577 | No |
| 28 | MYLK | myosin light chain kinase [Source:HGNC Symbol;Acc:HGNC:7590] | 8358 | 0.090 | -0.0552 | No |
| 29 | ITGA6 | integrin subunit alpha 6 [Source:HGNC Symbol;Acc:HGNC:6142] | 8958 | 0.083 | -0.0677 | No |
| 30 | F2 | "coagulation factor II, thrombin [Source:HGNC Symbol;Acc:HGNC:3535]" | 9010 | 0.082 | -0.0663 | No |
| 31 | PIP5K1C | phosphatidylinositol-4-phosphate 5-kinase type 1 gamma [Source:HGNC Symbol;Acc:HGNC:8996] | 9093 | 0.081 | -0.0656 | No |
| 32 | ENAH | ENAH actin regulator [Source:HGNC Symbol;Acc:HGNC:18271] | 10001 | 0.071 | -0.0865 | No |
| 33 | ARPC1B | actin related protein 2/3 complex subunit 1B [Source:HGNC Symbol;Acc:HGNC:704] | 10278 | 0.068 | -0.0913 | No |
| 34 | ABI2 | abl interactor 2 [Source:HGNC Symbol;Acc:HGNC:24011] | 10351 | 0.067 | -0.0909 | No |
| 35 | ITGA4 | integrin subunit alpha 4 [Source:HGNC Symbol;Acc:HGNC:6140] | 10741 | 0.063 | -0.0987 | No |
| 36 | FGF6 | fibroblast growth factor 6 [Source:HGNC Symbol;Acc:HGNC:3684] | 10831 | 0.063 | -0.0989 | No |
| 37 | FGFR3 | fibroblast growth factor receptor 3 [Source:HGNC Symbol;Acc:HGNC:3690] | 11317 | 0.058 | -0.1093 | No |
| 38 | CHRM2 | cholinergic receptor muscarinic 2 [Source:HGNC Symbol;Acc:HGNC:1951] | 11507 | 0.056 | -0.1123 | No |
| 39 | PAK4 | p21 (RAC1) activated kinase 4 [Source:HGNC Symbol;Acc:HGNC:16059] | 11565 | 0.056 | -0.1119 | No |
| 40 | INS | insulin [Source:HGNC Symbol;Acc:HGNC:6081] | 12938 | 0.045 | -0.1454 | No |
| 41 | PFN3 | profilin 3 [Source:HGNC Symbol;Acc:HGNC:18627] | 13110 | 0.044 | -0.1483 | No |
| 42 | APC | APC regulator of WNT signaling pathway [Source:HGNC Symbol;Acc:HGNC:583] | 13119 | 0.044 | -0.1471 | No |
| 43 | INSRR | insulin receptor related receptor [Source:HGNC Symbol;Acc:HGNC:6093] | 13274 | 0.043 | -0.1496 | No |
| 44 | BRK1 | BRICK1 subunit of SCAR/WAVE actin nucleating complex [Source:HGNC Symbol;Acc:HGNC:23057] | 13607 | 0.041 | -0.1567 | No |
| 45 | IQGAP2 | IQ motif containing GTPase activating protein 2 [Source:HGNC Symbol;Acc:HGNC:6111] | 14563 | 0.035 | -0.1800 | No |
| 46 | CRK | "CRK proto-oncogene, adaptor protein [Source:HGNC Symbol;Acc:HGNC:2362]" | 14915 | 0.032 | -0.1878 | No |
| 47 | CYFIP1 | cytoplasmic FMR1 interacting protein 1 [Source:HGNC Symbol;Acc:HGNC:13759] | 15071 | 0.032 | -0.1907 | No |
| 48 | FGF3 | fibroblast growth factor 3 [Source:HGNC Symbol;Acc:HGNC:3681] | 15597 | 0.028 | -0.2032 | No |
| 49 | CHRM5 | cholinergic receptor muscarinic 5 [Source:HGNC Symbol;Acc:HGNC:1954] | 15811 | 0.027 | -0.2078 | No |
| 50 | WASF1 | WASP family member 1 [Source:HGNC Symbol;Acc:HGNC:12732] | 16267 | 0.024 | -0.2186 | No |
| 51 | APC2 | APC regulator of WNT signaling pathway 2 [Source:HGNC Symbol;Acc:HGNC:24036] | 16400 | 0.023 | -0.2212 | No |
| 52 | FGF10 | fibroblast growth factor 10 [Source:HGNC Symbol;Acc:HGNC:3666] | 16552 | 0.023 | -0.2243 | No |
| 53 | SCIN | scinderin [Source:HGNC Symbol;Acc:HGNC:21695] | 17104 | 0.019 | -0.2377 | No |
| 54 | ITGA2 | integrin subunit alpha 2 [Source:HGNC Symbol;Acc:HGNC:6137] | 17136 | 0.019 | -0.2379 | No |
| 55 | FGF12 | fibroblast growth factor 12 [Source:HGNC Symbol;Acc:HGNC:3668] | 17515 | 0.017 | -0.2470 | No |
| 56 | WAS | WASP actin nucleation promoting factor [Source:HGNC Symbol;Acc:HGNC:12731] | 17936 | 0.015 | -0.2572 | No |
| 57 | PDGFD | platelet derived growth factor D [Source:HGNC Symbol;Acc:HGNC:30620] | 18049 | 0.014 | -0.2596 | No |
| 58 | MYH14 | myosin heavy chain 14 [Source:HGNC Symbol;Acc:HGNC:23212] | 18224 | 0.013 | -0.2636 | No |
| 59 | SOS1 | SOS Ras/Rac guanine nucleotide exchange factor 1 [Source:HGNC Symbol;Acc:HGNC:11187] | 18532 | 0.012 | -0.2711 | No |
| 60 | MYL10 | myosin light chain 10 [Source:HGNC Symbol;Acc:HGNC:29825] | 18653 | 0.011 | -0.2738 | No |
| 61 | PIP4K2A | phosphatidylinositol-5-phosphate 4-kinase type 2 alpha [Source:HGNC Symbol;Acc:HGNC:8997] | 18713 | 0.011 | -0.2749 | No |
| 62 | MYL7 | myosin light chain 7 [Source:HGNC Symbol;Acc:HGNC:21719] | 18720 | 0.011 | -0.2747 | No |
| 63 | FGF7 | fibroblast growth factor 7 [Source:HGNC Symbol;Acc:HGNC:3685] | 19331 | 0.007 | -0.2901 | No |
| 64 | FGF20 | fibroblast growth factor 20 [Source:HGNC Symbol;Acc:HGNC:3677] | 19442 | 0.007 | -0.2927 | No |
| 65 | ARPC3 | actin related protein 2/3 complex subunit 3 [Source:HGNC Symbol;Acc:HGNC:706] | 19525 | 0.006 | -0.2946 | No |
| 66 | ITGA8 | integrin subunit alpha 8 [Source:HGNC Symbol;Acc:HGNC:6144] | 20064 | 0.003 | -0.3082 | No |
| 67 | ITGA1 | integrin subunit alpha 1 [Source:HGNC Symbol;Acc:HGNC:6134] | 20236 | 0.002 | -0.3125 | No |
| 68 | PTK2 | protein tyrosine kinase 2 [Source:HGNC Symbol;Acc:HGNC:9611] | 20287 | 0.002 | -0.3137 | No |
| 69 | FGF8 | fibroblast growth factor 8 [Source:HGNC Symbol;Acc:HGNC:3686] | 21259 | -0.004 | -0.3384 | No |
| 70 | SLC9A1 | solute carrier family 9 member A1 [Source:HGNC Symbol;Acc:HGNC:11071] | 21364 | -0.004 | -0.3410 | No |
| 71 | CHRM1 | cholinergic receptor muscarinic 1 [Source:HGNC Symbol;Acc:HGNC:1950] | 21720 | -0.006 | -0.3498 | No |
| 72 | ITGB1 | integrin subunit beta 1 [Source:HGNC Symbol;Acc:HGNC:6153] | 22081 | -0.008 | -0.3588 | No |
| 73 | ITGA11 | integrin subunit alpha 11 [Source:HGNC Symbol;Acc:HGNC:6136] | 22218 | -0.009 | -0.3619 | No |
| 74 | MOS | "MOS proto-oncogene, serine/threonine kinase [Source:HGNC Symbol;Acc:HGNC:7199]" | 22268 | -0.009 | -0.3629 | No |
| 75 | MAPK1 | mitogen-activated protein kinase 1 [Source:HGNC Symbol;Acc:HGNC:6871] | 23167 | -0.015 | -0.3853 | No |
| 76 | FGF23 | fibroblast growth factor 23 [Source:HGNC Symbol;Acc:HGNC:3680] | 23636 | -0.018 | -0.3967 | No |
| 77 | PFN1 | profilin 1 [Source:HGNC Symbol;Acc:HGNC:8881] | 23848 | -0.019 | -0.4015 | No |
| 78 | ROCK2 | Rho associated coiled-coil containing protein kinase 2 [Source:HGNC Symbol;Acc:HGNC:10252] | 24395 | -0.022 | -0.4147 | No |
| 79 | FGF1 | fibroblast growth factor 1 [Source:HGNC Symbol;Acc:HGNC:3665] | 25015 | -0.027 | -0.4296 | No |
| 80 | MYLK3 | myosin light chain kinase 3 [Source:HGNC Symbol;Acc:HGNC:29826] | 25385 | -0.029 | -0.4381 | No |
| 81 | FGD3 | "FYVE, RhoGEF and PH domain containing 3 [Source:HGNC Symbol;Acc:HGNC:16027]" | 25578 | -0.031 | -0.4420 | No |
| 82 | PFN2 | profilin 2 [Source:HGNC Symbol;Acc:HGNC:8882] | 25763 | -0.032 | -0.4456 | No |
| 83 | SSH1 | slingshot protein phosphatase 1 [Source:HGNC Symbol;Acc:HGNC:30579] | 25909 | -0.033 | -0.4482 | No |
| 84 | FGF21 | fibroblast growth factor 21 [Source:HGNC Symbol;Acc:HGNC:3678] | 26134 | -0.035 | -0.4528 | No |
| 85 | ITGAL | integrin subunit alpha L [Source:HGNC Symbol;Acc:HGNC:6148] | 26570 | -0.038 | -0.4626 | No |
| 86 | HRAS | "HRas proto-oncogene, GTPase [Source:HGNC Symbol;Acc:HGNC:5173]" | 26672 | -0.039 | -0.4639 | No |
| 87 | ARAF | "A-Raf proto-oncogene, serine/threonine kinase [Source:HGNC Symbol;Acc:HGNC:646]" | 26742 | -0.039 | -0.4644 | No |
| 88 | BDKRB2 | bradykinin receptor B2 [Source:HGNC Symbol;Acc:HGNC:1030] | 27151 | -0.042 | -0.4734 | No |
| 89 | ACTN2 | actinin alpha 2 [Source:HGNC Symbol;Acc:HGNC:164] | 27204 | -0.043 | -0.4733 | No |
| 90 | ACTN4 | actinin alpha 4 [Source:HGNC Symbol;Acc:HGNC:166] | 28351 | -0.054 | -0.5008 | No |
| 91 | FGF5 | fibroblast growth factor 5 [Source:HGNC Symbol;Acc:HGNC:3683] | 28433 | -0.054 | -0.5010 | No |
| 92 | TMSB4Y | thymosin beta 4 Y-linked [Source:HGNC Symbol;Acc:HGNC:11882] | 28515 | -0.055 | -0.5013 | No |
| 93 | F2R | coagulation factor II thrombin receptor [Source:HGNC Symbol;Acc:HGNC:3537] | 28542 | -0.055 | -0.5001 | No |
| 94 | FGF22 | fibroblast growth factor 22 [Source:HGNC Symbol;Acc:HGNC:3679] | 28604 | -0.056 | -0.4998 | No |
| 95 | ARHGEF1 | Rho guanine nucleotide exchange factor 1 [Source:HGNC Symbol;Acc:HGNC:681] | 28832 | -0.058 | -0.5036 | No |
| 96 | ARHGEF4 | Rho guanine nucleotide exchange factor 4 [Source:HGNC Symbol;Acc:HGNC:684] | 28850 | -0.059 | -0.5021 | No |
| 97 | FN1 | fibronectin 1 [Source:HGNC Symbol;Acc:HGNC:3778] | 29282 | -0.064 | -0.5110 | No |
| 98 | VAV3 | vav guanine nucleotide exchange factor 3 [Source:HGNC Symbol;Acc:HGNC:12659] | 29349 | -0.065 | -0.5105 | No |
| 99 | FGF4 | fibroblast growth factor 4 [Source:HGNC Symbol;Acc:HGNC:3682] | 29833 | -0.070 | -0.5205 | No |
| 100 | FGF18 | fibroblast growth factor 18 [Source:HGNC Symbol;Acc:HGNC:3674] | 29941 | -0.071 | -0.5209 | No |
| 101 | ITGAE | integrin subunit alpha E [Source:HGNC Symbol;Acc:HGNC:6147] | 29981 | -0.072 | -0.5195 | No |
| 102 | PDGFA | platelet derived growth factor subunit A [Source:HGNC Symbol;Acc:HGNC:8799] | 30414 | -0.077 | -0.5279 | No |
| 103 | DOCK1 | dedicator of cytokinesis 1 [Source:HGNC Symbol;Acc:HGNC:2987] | 30454 | -0.078 | -0.5263 | No |
| 104 | PIKFYVE | "phosphoinositide kinase, FYVE-type zinc finger containing [Source:HGNC Symbol;Acc:HGNC:23785]" | 30696 | -0.081 | -0.5298 | Yes |
| 105 | RAC3 | Rac family small GTPase 3 [Source:HGNC Symbol;Acc:HGNC:9803] | 30704 | -0.081 | -0.5273 | Yes |
| 106 | ACTB | actin beta [Source:HGNC Symbol;Acc:HGNC:132] | 30724 | -0.081 | -0.5251 | Yes |
| 107 | ITGAD | integrin subunit alpha D [Source:HGNC Symbol;Acc:HGNC:6146] | 30848 | -0.083 | -0.5254 | Yes |
| 108 | CFL1 | cofilin 1 [Source:HGNC Symbol;Acc:HGNC:1874] | 31054 | -0.086 | -0.5278 | Yes |
| 109 | FGF17 | fibroblast growth factor 17 [Source:HGNC Symbol;Acc:HGNC:3673] | 31082 | -0.086 | -0.5256 | Yes |
| 110 | NCKAP1 | NCK associated protein 1 [Source:HGNC Symbol;Acc:HGNC:7666] | 31131 | -0.086 | -0.5240 | Yes |
| 111 | TIAM2 | TIAM Rac1 associated GEF 2 [Source:HGNC Symbol;Acc:HGNC:11806] | 31199 | -0.088 | -0.5228 | Yes |
| 112 | ARHGEF6 | Rac/Cdc42 guanine nucleotide exchange factor 6 [Source:HGNC Symbol;Acc:HGNC:685] | 31201 | -0.088 | -0.5199 | Yes |
| 113 | MRAS | muscle RAS oncogene homolog [Source:HGNC Symbol;Acc:HGNC:7227] | 31601 | -0.093 | -0.5269 | Yes |
| 114 | ITGB3 | integrin subunit beta 3 [Source:HGNC Symbol;Acc:HGNC:6156] | 31798 | -0.096 | -0.5287 | Yes |
| 115 | ARHGAP35 | Rho GTPase activating protein 35 [Source:HGNC Symbol;Acc:HGNC:4591] | 31911 | -0.098 | -0.5283 | Yes |
| 116 | ITGA9 | integrin subunit alpha 9 [Source:HGNC Symbol;Acc:HGNC:6145] | 32051 | -0.101 | -0.5285 | Yes |
| 117 | ITGB5 | integrin subunit beta 5 [Source:HGNC Symbol;Acc:HGNC:6160] | 32063 | -0.101 | -0.5254 | Yes |
| 118 | ITGB7 | integrin subunit beta 7 [Source:HGNC Symbol;Acc:HGNC:6162] | 32073 | -0.101 | -0.5223 | Yes |
| 119 | ARHGEF12 | Rho guanine nucleotide exchange factor 12 [Source:HGNC Symbol;Acc:HGNC:14193] | 32285 | -0.105 | -0.5242 | Yes |
| 120 | FGFR2 | fibroblast growth factor receptor 2 [Source:HGNC Symbol;Acc:HGNC:3689] | 32385 | -0.107 | -0.5231 | Yes |
| 121 | FGF19 | fibroblast growth factor 19 [Source:HGNC Symbol;Acc:HGNC:3675] | 32426 | -0.108 | -0.5205 | Yes |
| 122 | GNA12 | G protein subunit alpha 12 [Source:HGNC Symbol;Acc:HGNC:4380] | 32548 | -0.110 | -0.5200 | Yes |
| 123 | GIT1 | GIT ArfGAP 1 [Source:HGNC Symbol;Acc:HGNC:4272] | 32673 | -0.112 | -0.5194 | Yes |
| 124 | CYFIP2 | cytoplasmic FMR1 interacting protein 2 [Source:HGNC Symbol;Acc:HGNC:13760] | 32702 | -0.113 | -0.5163 | Yes |
| 125 | ACTN3 | actinin alpha 3 [Source:HGNC Symbol;Acc:HGNC:165] | 32823 | -0.115 | -0.5155 | Yes |
| 126 | MYL2 | myosin light chain 2 [Source:HGNC Symbol;Acc:HGNC:7583] | 33086 | -0.121 | -0.5182 | Yes |
| 127 | ARPC5L | actin related protein 2/3 complex subunit 5 like [Source:HGNC Symbol;Acc:HGNC:23366] | 33127 | -0.121 | -0.5152 | Yes |
| 128 | EGF | epidermal growth factor [Source:HGNC Symbol;Acc:HGNC:3229] | 33135 | -0.121 | -0.5113 | Yes |
| 129 | PPP1CA | protein phosphatase 1 catalytic subunit alpha [Source:HGNC Symbol;Acc:HGNC:9281] | 33209 | -0.123 | -0.5090 | Yes |
| 130 | SSH3 | slingshot protein phosphatase 3 [Source:HGNC Symbol;Acc:HGNC:30581] | 33229 | -0.123 | -0.5054 | Yes |
| 131 | PDGFC | platelet derived growth factor C [Source:HGNC Symbol;Acc:HGNC:8801] | 33384 | -0.127 | -0.5051 | Yes |
| 132 | DIAPH2 | diaphanous related formin 2 [Source:HGNC Symbol;Acc:HGNC:2877] | 33468 | -0.128 | -0.5029 | Yes |
| 133 | FGF11 | fibroblast growth factor 11 [Source:HGNC Symbol;Acc:HGNC:3667] | 33539 | -0.130 | -0.5004 | Yes |
| 134 | PDGFB | platelet derived growth factor subunit B [Source:HGNC Symbol;Acc:HGNC:8800] | 33560 | -0.130 | -0.4965 | Yes |
| 135 | GNG12 | G protein subunit gamma 12 [Source:HGNC Symbol;Acc:HGNC:19663] | 33587 | -0.131 | -0.4928 | Yes |
| 136 | ITGB4 | integrin subunit beta 4 [Source:HGNC Symbol;Acc:HGNC:6158] | 33620 | -0.132 | -0.4892 | Yes |
| 137 | RRAS | RAS related [Source:HGNC Symbol;Acc:HGNC:10447] | 33667 | -0.133 | -0.4860 | Yes |
| 138 | NRAS | "NRAS proto-oncogene, GTPase [Source:HGNC Symbol;Acc:HGNC:7989]" | 33739 | -0.135 | -0.4833 | Yes |
| 139 | PIK3CD | "phosphatidylinositol-4,5-bisphosphate 3-kinase catalytic subunit delta [Source:HGNC Symbol;Acc:HGNC:8977]" | 33794 | -0.137 | -0.4801 | Yes |
| 140 | PAK1 | p21 (RAC1) activated kinase 1 [Source:HGNC Symbol;Acc:HGNC:8590] | 33819 | -0.137 | -0.4761 | Yes |
| 141 | MYLPF | "myosin light chain, phosphorylatable, fast skeletal muscle [Source:HGNC Symbol;Acc:HGNC:29824]" | 33874 | -0.139 | -0.4728 | Yes |
| 142 | WASF2 | WASP family member 2 [Source:HGNC Symbol;Acc:HGNC:12733] | 34035 | -0.142 | -0.4722 | Yes |
| 143 | ACTG1 | actin gamma 1 [Source:HGNC Symbol;Acc:HGNC:144] | 34048 | -0.142 | -0.4677 | Yes |
| 144 | MYL5 | myosin light chain 5 [Source:HGNC Symbol;Acc:HGNC:7586] | 34055 | -0.143 | -0.4631 | Yes |
| 145 | FGF16 | fibroblast growth factor 16 [Source:HGNC Symbol;Acc:HGNC:3672] | 34121 | -0.144 | -0.4599 | Yes |
| 146 | RDX | radixin [Source:HGNC Symbol;Acc:HGNC:9944] | 34205 | -0.147 | -0.4572 | Yes |
| 147 | PAK6 | p21 (RAC1) activated kinase 6 [Source:HGNC Symbol;Acc:HGNC:16061] | 34232 | -0.148 | -0.4529 | Yes |
| 148 | VAV2 | vav guanine nucleotide exchange factor 2 [Source:HGNC Symbol;Acc:HGNC:12658] | 34251 | -0.148 | -0.4484 | Yes |
| 149 | PAK3 | p21 (RAC1) activated kinase 3 [Source:HGNC Symbol;Acc:HGNC:8592] | 34288 | -0.149 | -0.4443 | Yes |
| 150 | MYH10 | myosin heavy chain 10 [Source:HGNC Symbol;Acc:HGNC:7568] | 34375 | -0.152 | -0.4415 | Yes |
| 151 | MYL12A | myosin light chain 12A [Source:HGNC Symbol;Acc:HGNC:16701] | 34438 | -0.154 | -0.4379 | Yes |
| 152 | CRKL | "CRK like proto-oncogene, adaptor protein [Source:HGNC Symbol;Acc:HGNC:2363]" | 34457 | -0.154 | -0.4332 | Yes |
| 153 | PIP4K2C | phosphatidylinositol-5-phosphate 4-kinase type 2 gamma [Source:HGNC Symbol;Acc:HGNC:23786] | 34759 | -0.163 | -0.4354 | Yes |
| 154 | PPP1CC | protein phosphatase 1 catalytic subunit gamma [Source:HGNC Symbol;Acc:HGNC:9283] | 34862 | -0.166 | -0.4325 | Yes |
| 155 | PDGFRB | platelet derived growth factor receptor beta [Source:HGNC Symbol;Acc:HGNC:8804] | 34887 | -0.167 | -0.4275 | Yes |
| 156 | ITGB8 | integrin subunit beta 8 [Source:HGNC Symbol;Acc:HGNC:6163] | 35094 | -0.174 | -0.4269 | Yes |
| 157 | TMSB4XP8 | TMSB4X pseudogene 8 [Source:HGNC Symbol;Acc:HGNC:11885] | 35140 | -0.175 | -0.4222 | Yes |
| 158 | MSN | moesin [Source:HGNC Symbol;Acc:HGNC:7373] | 35197 | -0.178 | -0.4177 | Yes |
| 159 | EGFR | epidermal growth factor receptor [Source:HGNC Symbol;Acc:HGNC:3236] | 35417 | -0.186 | -0.4171 | Yes |
| 160 | PXN | paxillin [Source:HGNC Symbol;Acc:HGNC:9718] | 35502 | -0.189 | -0.4129 | Yes |
| 161 | MAPK3 | mitogen-activated protein kinase 3 [Source:HGNC Symbol;Acc:HGNC:6877] | 35579 | -0.191 | -0.4085 | Yes |
| 162 | LIMK1 | LIM domain kinase 1 [Source:HGNC Symbol;Acc:HGNC:6613] | 35605 | -0.192 | -0.4027 | Yes |
| 163 | ARPC2 | actin related protein 2/3 complex subunit 2 [Source:HGNC Symbol;Acc:HGNC:705] | 35610 | -0.192 | -0.3964 | Yes |
| 164 | NCKAP1L | NCK associated protein 1 like [Source:HGNC Symbol;Acc:HGNC:4862] | 35716 | -0.196 | -0.3925 | Yes |
| 165 | BCAR1 | "BCAR1 scaffold protein, Cas family member [Source:HGNC Symbol;Acc:HGNC:971]" | 35834 | -0.201 | -0.3888 | Yes |
| 166 | FGF9 | fibroblast growth factor 9 [Source:HGNC Symbol;Acc:HGNC:3687] | 35849 | -0.202 | -0.3824 | Yes |
| 167 | MAP2K1 | mitogen-activated protein kinase kinase 1 [Source:HGNC Symbol;Acc:HGNC:6840] | 36024 | -0.208 | -0.3799 | Yes |
| 168 | ARPC4 | actin related protein 2/3 complex subunit 4 [Source:HGNC Symbol;Acc:HGNC:707] | 36058 | -0.210 | -0.3737 | Yes |
| 169 | VCL | vinculin [Source:HGNC Symbol;Acc:HGNC:12665] | 36176 | -0.215 | -0.3695 | Yes |
| 170 | PIK3CG | "phosphatidylinositol-4,5-bisphosphate 3-kinase catalytic subunit gamma [Source:HGNC Symbol;Acc:HGNC:8978]" | 36243 | -0.217 | -0.3640 | Yes |
| 171 | PPP1CB | protein phosphatase 1 catalytic subunit beta [Source:HGNC Symbol;Acc:HGNC:9282] | 36276 | -0.219 | -0.3575 | Yes |
| 172 | CD14 | CD14 molecule [Source:HGNC Symbol;Acc:HGNC:1628] | 36298 | -0.220 | -0.3507 | Yes |
| 173 | ITGB2 | integrin subunit beta 2 [Source:HGNC Symbol;Acc:HGNC:6155] | 36308 | -0.220 | -0.3435 | Yes |
| 174 | TIAM1 | TIAM Rac1 associated GEF 1 [Source:HGNC Symbol;Acc:HGNC:11805] | 36355 | -0.223 | -0.3372 | Yes |
| 175 | TMSB4X | thymosin beta 4 X-linked [Source:HGNC Symbol;Acc:HGNC:11881] | 36396 | -0.225 | -0.3307 | Yes |
| 176 | ITGA5 | integrin subunit alpha 5 [Source:HGNC Symbol;Acc:HGNC:6141] | 36513 | -0.231 | -0.3260 | Yes |
| 177 | PDGFRA | platelet derived growth factor receptor alpha [Source:HGNC Symbol;Acc:HGNC:8803] | 36521 | -0.231 | -0.3184 | Yes |
| 178 | ACTN1 | actinin alpha 1 [Source:HGNC Symbol;Acc:HGNC:163] | 36614 | -0.236 | -0.3129 | Yes |
| 179 | MYL9 | myosin light chain 9 [Source:HGNC Symbol;Acc:HGNC:15754] | 36627 | -0.237 | -0.3052 | Yes |
| 180 | FGFR1 | fibroblast growth factor receptor 1 [Source:HGNC Symbol;Acc:HGNC:3688] | 36687 | -0.241 | -0.2987 | Yes |
| 181 | ITGAM | integrin subunit alpha M [Source:HGNC Symbol;Acc:HGNC:6149] | 36727 | -0.243 | -0.2916 | Yes |
| 182 | DIAPH1 | diaphanous related formin 1 [Source:HGNC Symbol;Acc:HGNC:2876] | 36840 | -0.249 | -0.2861 | Yes |
| 183 | BAIAP2 | BAR/IMD domain containing adaptor protein 2 [Source:HGNC Symbol;Acc:HGNC:947] | 36850 | -0.251 | -0.2779 | Yes |
| 184 | CHRM4 | cholinergic receptor muscarinic 4 [Source:HGNC Symbol;Acc:HGNC:1953] | 36854 | -0.251 | -0.2696 | Yes |
| 185 | FGF2 | fibroblast growth factor 2 [Source:HGNC Symbol;Acc:HGNC:3676] | 36896 | -0.253 | -0.2622 | Yes |
| 186 | RRAS2 | RAS related 2 [Source:HGNC Symbol;Acc:HGNC:17271] | 37160 | -0.270 | -0.2599 | Yes |
| 187 | PIK3R1 | phosphoinositide-3-kinase regulatory subunit 1 [Source:HGNC Symbol;Acc:HGNC:8979] | 37172 | -0.270 | -0.2511 | Yes |
| 188 | EZR | ezrin [Source:HGNC Symbol;Acc:HGNC:12691] | 37217 | -0.275 | -0.2431 | Yes |
| 189 | RAC1 | Rac family small GTPase 1 [Source:HGNC Symbol;Acc:HGNC:9801] | 37263 | -0.277 | -0.2350 | Yes |
| 190 | ITGA2B | integrin subunit alpha 2b [Source:HGNC Symbol;Acc:HGNC:6138] | 37349 | -0.284 | -0.2277 | Yes |
| 191 | RHOA | ras homolog family member A [Source:HGNC Symbol;Acc:HGNC:667] | 37417 | -0.290 | -0.2197 | Yes |
| 192 | ITGAX | integrin subunit alpha X [Source:HGNC Symbol;Acc:HGNC:6152] | 37551 | -0.301 | -0.2130 | Yes |
| 193 | PIK3R5 | phosphoinositide-3-kinase regulatory subunit 5 [Source:HGNC Symbol;Acc:HGNC:30035] | 37628 | -0.307 | -0.2047 | Yes |
| 194 | MYL12B | myosin light chain 12B [Source:HGNC Symbol;Acc:HGNC:29827] | 37667 | -0.310 | -0.1953 | Yes |
| 195 | ARHGEF7 | Rho guanine nucleotide exchange factor 7 [Source:HGNC Symbol;Acc:HGNC:15607] | 37668 | -0.310 | -0.1849 | Yes |
| 196 | PPP1R12A | protein phosphatase 1 regulatory subunit 12A [Source:HGNC Symbol;Acc:HGNC:7618] | 37785 | -0.322 | -0.1771 | Yes |
| 197 | KRAS | "KRAS proto-oncogene, GTPase [Source:HGNC Symbol;Acc:HGNC:6407]" | 37789 | -0.323 | -0.1664 | Yes |
| 198 | CDC42 | cell division cycle 42 [Source:HGNC Symbol;Acc:HGNC:1736] | 37805 | -0.324 | -0.1559 | Yes |
| 199 | RAF1 | "Raf-1 proto-oncogene, serine/threonine kinase [Source:HGNC Symbol;Acc:HGNC:9829]" | 37807 | -0.325 | -0.1451 | Yes |
| 200 | ITGAV | integrin subunit alpha V [Source:HGNC Symbol;Acc:HGNC:6150] | 37826 | -0.328 | -0.1346 | Yes |
| 201 | IQGAP1 | IQ motif containing GTPase activating protein 1 [Source:HGNC Symbol;Acc:HGNC:6110] | 37829 | -0.329 | -0.1236 | Yes |
| 202 | BRAF | "B-Raf proto-oncogene, serine/threonine kinase [Source:HGNC Symbol;Acc:HGNC:1097]" | 37844 | -0.330 | -0.1130 | Yes |
| 203 | FGD1 | "FYVE, RhoGEF and PH domain containing 1 [Source:HGNC Symbol;Acc:HGNC:3663]" | 37921 | -0.339 | -0.1036 | Yes |
| 204 | GSN | gelsolin [Source:HGNC Symbol;Acc:HGNC:4620] | 37964 | -0.345 | -0.0931 | Yes |
| 205 | CFL2 | cofilin 2 [Source:HGNC Symbol;Acc:HGNC:1875] | 38086 | -0.360 | -0.0841 | Yes |
| 206 | MYH9 | myosin heavy chain 9 [Source:HGNC Symbol;Acc:HGNC:7579] | 38206 | -0.380 | -0.0745 | Yes |
| 207 | LIMK2 | LIM domain kinase 2 [Source:HGNC Symbol;Acc:HGNC:6614] | 38261 | -0.390 | -0.0628 | Yes |
| 208 | PIK3CA | "phosphatidylinositol-4,5-bisphosphate 3-kinase catalytic subunit alpha [Source:HGNC Symbol;Acc:HGNC:8975]" | 38268 | -0.391 | -0.0499 | Yes |
| 209 | WASL | WASP like actin nucleation promoting factor [Source:HGNC Symbol;Acc:HGNC:12735] | 38341 | -0.407 | -0.0381 | Yes |
| 210 | GNA13 | G protein subunit alpha 13 [Source:HGNC Symbol;Acc:HGNC:4381] | 38366 | -0.411 | -0.0250 | Yes |
| 211 | SSH2 | slingshot protein phosphatase 2 [Source:HGNC Symbol;Acc:HGNC:30580] | 38434 | -0.430 | -0.0123 | Yes |
| 212 | ROCK1 | Rho associated coiled-coil containing protein kinase 1 [Source:HGNC Symbol;Acc:HGNC:10251] | 38575 | -0.519 | 0.0014 | Yes |
| 213 | SOS2 | SOS Ras/Rho guanine nucleotide exchange factor 2 [Source:HGNC Symbol;Acc:HGNC:11188] | 38581 | -0.528 | 0.0190 | Yes |
Table: GSEA details [plain text format]

  

Fig 2: KEGG\_REGULATION\_OF\_ACTIN\_CYTOSKELETON      
 Blue-Pink O' Gram in the Space of the Analyzed GeneSet

  

Fig 3: KEGG\_REGULATION\_OF\_ACTIN\_CYTOSKELETON: Random ES distribution      
 Gene set null distribution of ES for **KEGG\_REGULATION\_OF\_ACTIN\_CYTOSKELETON**
