# Supplementary material for: Simultaneous Presentation of Multiple Myeloma and Lung Cancer: Case Report and Gene Bioinformatics Analysis
Source: Front Oncol. 2022 Jun 13;12:859735. doi: 10.3389/fonc.2022.859735 (PMC9235397; doi:10.3389/fonc.2022.859735)
Supplement: Supplementary file 1 [file DataSheet_1.zip › The bioinformatic analysis of MM and lung cancer supplementary materials/Enrichment analysis/MECR/GSEA_4.1.0/MM TCGA/KEGG.Gsea.1639040591792/KEGG_SELENOAMINO_ACID_METABOLISM.html]

Details for gene set KEGG\_SELENOAMINO\_ACID\_METABOLISM[GSEA]

|  || Dataset | ExpData\_collapsed\_to\_symbols.ENSG00000116353\_profile\_in\_ExpData.cls #ENSG00000116353 |
| Phenotype | ENSG00000116353\_profile\_in\_ExpData.cls#ENSG00000116353 |
| Upregulated in class | ENSG00000116353\_pos |
| GeneSet | KEGG\_SELENOAMINO\_ACID\_METABOLISM |
| Enrichment Score (ES) | 0.7123228 |
| Normalized Enrichment Score (NES) | 2.012026 |
| Nominal p-value | 0.0 |
| FDR q-value | 3.053708E-4 |
| FWER p-Value | 0.007 |
Table: GSEA Results Summary

  

Fig 1: Enrichment plot: KEGG\_SELENOAMINO\_ACID\_METABOLISM      
 Profile of the Running ES Score & Positions of GeneSet Members on the Rank Ordered List

  

| SYMBOL | TITLE | RANK IN GENE LIST | RANK METRIC SCORE | RUNNING ES | CORE ENRICHMENT || 1 | METTL2B | methyltransferase like 2B [Source:HGNC Symbol;Acc:HGNC:18272] | 321 | 0.450 | 0.0831 | Yes |
| 2 | METTL6 | methyltransferase like 6 [Source:HGNC Symbol;Acc:HGNC:28343] | 322 | 0.450 | 0.1743 | Yes |
| 3 | SCLY | selenocysteine lyase [Source:HGNC Symbol;Acc:HGNC:18161] | 374 | 0.439 | 0.2619 | Yes |
| 4 | MARS2 | "methionyl-tRNA synthetase 2, mitochondrial [Source:HGNC Symbol;Acc:HGNC:25133]" | 703 | 0.382 | 0.3309 | Yes |
| 5 | SEPHS1 | selenophosphate synthetase 1 [Source:HGNC Symbol;Acc:HGNC:19685] | 784 | 0.372 | 0.4043 | Yes |
| 6 | HEMK1 | HemK methyltransferase family member 1 [Source:HGNC Symbol;Acc:HGNC:24923] | 824 | 0.365 | 0.4772 | Yes |
| 7 | LCMT2 | leucine carboxyl methyltransferase 2 [Source:HGNC Symbol;Acc:HGNC:17558] | 872 | 0.359 | 0.5488 | Yes |
| 8 | MAT2A | methionine adenosyltransferase 2A [Source:HGNC Symbol;Acc:HGNC:6904] | 910 | 0.353 | 0.6194 | Yes |
| 9 | AHCY | adenosylhomocysteinase [Source:HGNC Symbol;Acc:HGNC:343] | 1339 | 0.313 | 0.6718 | Yes |
| 10 | AHCYL2 | adenosylhomocysteinase like 2 [Source:HGNC Symbol;Acc:HGNC:22204] | 2054 | 0.261 | 0.7066 | Yes |
| 11 | PAPSS1 | 3'-phosphoadenosine 5'-phosphosulfate synthase 1 [Source:HGNC Symbol;Acc:HGNC:8603] | 4326 | 0.167 | 0.6826 | Yes |
| 12 | GGT1 | gamma-glutamyltransferase 1 [Source:HGNC Symbol;Acc:HGNC:4250] | 4455 | 0.163 | 0.7123 | Yes |
| 13 | LCMT1 | leucine carboxyl methyltransferase 1 [Source:HGNC Symbol;Acc:HGNC:17557] | 5867 | 0.129 | 0.7027 | No |
| 14 | MARS1 | methionyl-tRNA synthetase 1 [Source:HGNC Symbol;Acc:HGNC:6898] | 9128 | 0.081 | 0.6360 | No |
| 15 | BUD23 | BUD23 rRNA methyltransferase and ribosome maturation factor [Source:HGNC Symbol;Acc:HGNC:16405] | 9988 | 0.071 | 0.6286 | No |
| 16 | PAPSS2 | 3'-phosphoadenosine 5'-phosphosulfate synthase 2 [Source:HGNC Symbol;Acc:HGNC:8604] | 11897 | 0.053 | 0.5908 | No |
| 17 | SEPHS2 | selenophosphate synthetase 2 [Source:HGNC Symbol;Acc:HGNC:19686] | 15743 | 0.027 | 0.4985 | No |
| 18 | CBS | cystathionine beta-synthase [Source:HGNC Symbol;Acc:HGNC:1550] | 15795 | 0.027 | 0.5026 | No |
| 19 | GGT6 | gamma-glutamyltransferase 6 [Source:HGNC Symbol;Acc:HGNC:26891] | 16273 | 0.024 | 0.4954 | No |
| 20 | CTH | cystathionine gamma-lyase [Source:HGNC Symbol;Acc:HGNC:2501] | 16651 | 0.022 | 0.4903 | No |
| 21 | TRMT11 | tRNA methyltransferase 11 homolog [Source:HGNC Symbol;Acc:HGNC:21080] | 21372 | -0.004 | 0.3710 | No |
| 22 | AHCYL1 | adenosylhomocysteinase like 1 [Source:HGNC Symbol;Acc:HGNC:344] | 24002 | -0.020 | 0.3082 | No |
| 23 | MAT1A | methionine adenosyltransferase 1A [Source:HGNC Symbol;Acc:HGNC:6903] | 25978 | -0.034 | 0.2647 | No |
| 24 | MAT2B | methionine adenosyltransferase 2B [Source:HGNC Symbol;Acc:HGNC:6905] | 27211 | -0.043 | 0.2420 | No |
| 25 | GGT7 | gamma-glutamyltransferase 7 [Source:HGNC Symbol;Acc:HGNC:4259] | 29746 | -0.069 | 0.1915 | No |
| 26 | GGT5 | gamma-glutamyltransferase 5 [Source:HGNC Symbol;Acc:HGNC:4260] | 36965 | -0.257 | 0.0600 | No |
Table: GSEA details [plain text format]

  

Fig 2: KEGG\_SELENOAMINO\_ACID\_METABOLISM      
 Blue-Pink O' Gram in the Space of the Analyzed GeneSet

  

Fig 3: KEGG\_SELENOAMINO\_ACID\_METABOLISM: Random ES distribution      
 Gene set null distribution of ES for **KEGG\_SELENOAMINO\_ACID\_METABOLISM**
