# Supplementary material for: Simultaneous Presentation of Multiple Myeloma and Lung Cancer: Case Report and Gene Bioinformatics Analysis
Source: Front Oncol. 2022 Jun 13;12:859735. doi: 10.3389/fonc.2022.859735 (PMC9235397; doi:10.3389/fonc.2022.859735)
Supplement: Supplementary file 1 [file DataSheet_1.zip › The bioinformatic analysis of MM and lung cancer supplementary materials/Enrichment analysis/MECR/GSEA_4.1.0/MM TCGA/KEGG.Gsea.1639040591792/KEGG_TOLL_LIKE_RECEPTOR_SIGNALING_PATHWAY.html]

Details for gene set KEGG\_TOLL\_LIKE\_RECEPTOR\_SIGNALING\_PATHWAY[GSEA]

|  || Dataset | ExpData\_collapsed\_to\_symbols.ENSG00000116353\_profile\_in\_ExpData.cls #ENSG00000116353 |
| Phenotype | ENSG00000116353\_profile\_in\_ExpData.cls#ENSG00000116353 |
| Upregulated in class | ENSG00000116353\_neg |
| GeneSet | KEGG\_TOLL\_LIKE\_RECEPTOR\_SIGNALING\_PATHWAY |
| Enrichment Score (ES) | -0.5420173 |
| Normalized Enrichment Score (NES) | -2.1803734 |
| Nominal p-value | 0.0 |
| FDR q-value | 0.0 |
| FWER p-Value | 0.0 |
Table: GSEA Results Summary

  

Fig 1: Enrichment plot: KEGG\_TOLL\_LIKE\_RECEPTOR\_SIGNALING\_PATHWAY      
 Profile of the Running ES Score & Positions of GeneSet Members on the Rank Ordered List

  

| SYMBOL | TITLE | RANK IN GENE LIST | RANK METRIC SCORE | RUNNING ES | CORE ENRICHMENT || 1 | PIK3R2 | phosphoinositide-3-kinase regulatory subunit 2 [Source:HGNC Symbol;Acc:HGNC:8980] | 1248 | 0.320 | -0.0118 | No |
| 2 | TLR5 | toll like receptor 5 [Source:HGNC Symbol;Acc:HGNC:11851] | 2392 | 0.242 | -0.0259 | No |
| 3 | PIK3R3 | phosphoinositide-3-kinase regulatory subunit 3 [Source:HGNC Symbol;Acc:HGNC:8981] | 2602 | 0.231 | -0.0168 | No |
| 4 | FOS | "Fos proto-oncogene, AP-1 transcription factor subunit [Source:HGNC Symbol;Acc:HGNC:3796]" | 3070 | 0.210 | -0.0156 | No |
| 5 | TAB1 | TGF-beta activated kinase 1 (MAP3K7) binding protein 1 [Source:HGNC Symbol;Acc:HGNC:18157] | 3360 | 0.199 | -0.0106 | No |
| 6 | IFNAR1 | interferon alpha and beta receptor subunit 1 [Source:HGNC Symbol;Acc:HGNC:5432] | 4033 | 0.175 | -0.0168 | No |
| 7 | IL12A | interleukin 12A [Source:HGNC Symbol;Acc:HGNC:5969] | 4178 | 0.171 | -0.0098 | No |
| 8 | MAP2K6 | mitogen-activated protein kinase kinase 6 [Source:HGNC Symbol;Acc:HGNC:6846] | 4800 | 0.154 | -0.0160 | No |
| 9 | IFNAR2 | interferon alpha and beta receptor subunit 2 [Source:HGNC Symbol;Acc:HGNC:5433] | 4817 | 0.153 | -0.0069 | No |
| 10 | JUN | "Jun proto-oncogene, AP-1 transcription factor subunit [Source:HGNC Symbol;Acc:HGNC:6204]" | 5088 | 0.146 | -0.0047 | No |
| 11 | MAP2K2 | mitogen-activated protein kinase kinase 2 [Source:HGNC Symbol;Acc:HGNC:6842] | 5104 | 0.146 | 0.0040 | No |
| 12 | TLR9 | toll like receptor 9 [Source:HGNC Symbol;Acc:HGNC:15633] | 5327 | 0.141 | 0.0072 | No |
| 13 | PIK3CB | "phosphatidylinositol-4,5-bisphosphate 3-kinase catalytic subunit beta [Source:HGNC Symbol;Acc:HGNC:8976]" | 7716 | 0.099 | -0.0476 | No |
| 14 | TOLLIP | toll interacting protein [Source:HGNC Symbol;Acc:HGNC:16476] | 8179 | 0.093 | -0.0536 | No |
| 15 | MYD88 | MYD88 innate immune signal transduction adaptor [Source:HGNC Symbol;Acc:HGNC:7562] | 8652 | 0.087 | -0.0602 | No |
| 16 | IFNA21 | interferon alpha 21 [Source:HGNC Symbol;Acc:HGNC:5424] | 8834 | 0.084 | -0.0596 | No |
| 17 | IRAK1 | interleukin 1 receptor associated kinase 1 [Source:HGNC Symbol;Acc:HGNC:6112] | 9888 | 0.072 | -0.0819 | No |
| 18 | IL12B | interleukin 12B [Source:HGNC Symbol;Acc:HGNC:5970] | 9896 | 0.072 | -0.0776 | No |
| 19 | CHUK | component of inhibitor of nuclear factor kappa B kinase complex [Source:HGNC Symbol;Acc:HGNC:1974] | 9925 | 0.071 | -0.0739 | No |
| 20 | STAT1 | signal transducer and activator of transcription 1 [Source:HGNC Symbol;Acc:HGNC:11362] | 10873 | 0.062 | -0.0941 | No |
| 21 | IKBKE | inhibitor of nuclear factor kappa B kinase subunit epsilon [Source:HGNC Symbol;Acc:HGNC:14552] | 11355 | 0.058 | -0.1028 | No |
| 22 | IFNA1 | interferon alpha 1 [Source:HGNC Symbol;Acc:HGNC:5417] | 12354 | 0.050 | -0.1252 | No |
| 23 | MAP3K7 | mitogen-activated protein kinase kinase kinase 7 [Source:HGNC Symbol;Acc:HGNC:6859] | 12527 | 0.048 | -0.1265 | No |
| 24 | IRF3 | interferon regulatory factor 3 [Source:HGNC Symbol;Acc:HGNC:6118] | 13499 | 0.041 | -0.1487 | No |
| 25 | TLR3 | toll like receptor 3 [Source:HGNC Symbol;Acc:HGNC:11849] | 13569 | 0.041 | -0.1479 | No |
| 26 | IFNA5 | interferon alpha 5 [Source:HGNC Symbol;Acc:HGNC:5426] | 14195 | 0.037 | -0.1616 | No |
| 27 | MAPK10 | mitogen-activated protein kinase 10 [Source:HGNC Symbol;Acc:HGNC:6872] | 14943 | 0.032 | -0.1786 | No |
| 28 | IFNA16 | interferon alpha 16 [Source:HGNC Symbol;Acc:HGNC:5421] | 15302 | 0.030 | -0.1858 | No |
| 29 | IFNA14 | interferon alpha 14 [Source:HGNC Symbol;Acc:HGNC:5420] | 16484 | 0.023 | -0.2145 | No |
| 30 | IFNB1 | interferon beta 1 [Source:HGNC Symbol;Acc:HGNC:5434] | 17326 | 0.018 | -0.2348 | No |
| 31 | FADD | Fas associated via death domain [Source:HGNC Symbol;Acc:HGNC:3573] | 17518 | 0.017 | -0.2387 | No |
| 32 | IFNA13 | interferon alpha 13 [Source:HGNC Symbol;Acc:HGNC:5419] | 17705 | 0.016 | -0.2424 | No |
| 33 | MAPK9 | mitogen-activated protein kinase 9 [Source:HGNC Symbol;Acc:HGNC:6886] | 20291 | 0.002 | -0.3082 | No |
| 34 | IKBKB | inhibitor of nuclear factor kappa B kinase subunit beta [Source:HGNC Symbol;Acc:HGNC:5960] | 20718 | -0.000 | -0.3190 | No |
| 35 | MAPK1 | mitogen-activated protein kinase 1 [Source:HGNC Symbol;Acc:HGNC:6871] | 23167 | -0.015 | -0.3805 | No |
| 36 | IFNA7 | interferon alpha 7 [Source:HGNC Symbol;Acc:HGNC:5428] | 24884 | -0.026 | -0.4227 | No |
| 37 | TLR6 | toll like receptor 6 [Source:HGNC Symbol;Acc:HGNC:16711] | 24981 | -0.026 | -0.4235 | No |
| 38 | IFNA17 | interferon alpha 17 [Source:HGNC Symbol;Acc:HGNC:5422] | 25197 | -0.028 | -0.4272 | No |
| 39 | MAPK12 | mitogen-activated protein kinase 12 [Source:HGNC Symbol;Acc:HGNC:6874] | 25314 | -0.028 | -0.4284 | No |
| 40 | TLR4 | toll like receptor 4 [Source:HGNC Symbol;Acc:HGNC:11850] | 27199 | -0.043 | -0.4738 | No |
| 41 | IFNA4 | interferon alpha 4 [Source:HGNC Symbol;Acc:HGNC:5425] | 27286 | -0.044 | -0.4733 | No |
| 42 | IRF7 | interferon regulatory factor 7 [Source:HGNC Symbol;Acc:HGNC:6122] | 27643 | -0.047 | -0.4794 | No |
| 43 | IFNA6 | interferon alpha 6 [Source:HGNC Symbol;Acc:HGNC:5427] | 28124 | -0.051 | -0.4885 | No |
| 44 | TIRAP | TIR domain containing adaptor protein [Source:HGNC Symbol;Acc:HGNC:17192] | 28145 | -0.052 | -0.4857 | No |
| 45 | IFNA8 | interferon alpha 8 [Source:HGNC Symbol;Acc:HGNC:5429] | 28823 | -0.058 | -0.4994 | No |
| 46 | MAPK13 | mitogen-activated protein kinase 13 [Source:HGNC Symbol;Acc:HGNC:6875] | 29402 | -0.065 | -0.5101 | No |
| 47 | LY96 | lymphocyte antigen 96 [Source:HGNC Symbol;Acc:HGNC:17156] | 29405 | -0.065 | -0.5060 | No |
| 48 | IFNA2 | interferon alpha 2 [Source:HGNC Symbol;Acc:HGNC:5423] | 29488 | -0.066 | -0.5040 | No |
| 49 | SPP1 | secreted phosphoprotein 1 [Source:HGNC Symbol;Acc:HGNC:11255] | 29539 | -0.067 | -0.5012 | No |
| 50 | IL6 | interleukin 6 [Source:HGNC Symbol;Acc:HGNC:6018] | 29703 | -0.068 | -0.5010 | No |
| 51 | IFNA10 | interferon alpha 10 [Source:HGNC Symbol;Acc:HGNC:5418] | 29865 | -0.070 | -0.5008 | No |
| 52 | IRAK4 | interleukin 1 receptor associated kinase 4 [Source:HGNC Symbol;Acc:HGNC:17967] | 30700 | -0.081 | -0.5170 | No |
| 53 | CXCL9 | C-X-C motif chemokine ligand 9 [Source:HGNC Symbol;Acc:HGNC:7098] | 31652 | -0.094 | -0.5354 | No |
| 54 | AKT1 | AKT serine/threonine kinase 1 [Source:HGNC Symbol;Acc:HGNC:391] | 31906 | -0.098 | -0.5357 | Yes |
| 55 | IRF5 | interferon regulatory factor 5 [Source:HGNC Symbol;Acc:HGNC:6120] | 32079 | -0.101 | -0.5338 | Yes |
| 56 | IKBKG | inhibitor of nuclear factor kappa B kinase regulatory subunit gamma [Source:HGNC Symbol;Acc:HGNC:5961] | 32205 | -0.103 | -0.5306 | Yes |
| 57 | TLR7 | toll like receptor 7 [Source:HGNC Symbol;Acc:HGNC:15631] | 32573 | -0.110 | -0.5330 | Yes |
| 58 | TRAF3 | TNF receptor associated factor 3 [Source:HGNC Symbol;Acc:HGNC:12033] | 32885 | -0.117 | -0.5337 | Yes |
| 59 | AKT2 | AKT serine/threonine kinase 2 [Source:HGNC Symbol;Acc:HGNC:392] | 33213 | -0.123 | -0.5343 | Yes |
| 60 | CD80 | CD80 molecule [Source:HGNC Symbol;Acc:HGNC:1700] | 33283 | -0.124 | -0.5284 | Yes |
| 61 | CXCL10 | C-X-C motif chemokine ligand 10 [Source:HGNC Symbol;Acc:HGNC:10637] | 33451 | -0.128 | -0.5246 | Yes |
| 62 | CXCL11 | C-X-C motif chemokine ligand 11 [Source:HGNC Symbol;Acc:HGNC:10638] | 33604 | -0.131 | -0.5203 | Yes |
| 63 | CCL3 | C-C motif chemokine ligand 3 [Source:HGNC Symbol;Acc:HGNC:10627] | 33771 | -0.136 | -0.5161 | Yes |
| 64 | PIK3CD | "phosphatidylinositol-4,5-bisphosphate 3-kinase catalytic subunit delta [Source:HGNC Symbol;Acc:HGNC:8977]" | 33794 | -0.137 | -0.5081 | Yes |
| 65 | CD86 | CD86 molecule [Source:HGNC Symbol;Acc:HGNC:1705] | 34067 | -0.143 | -0.5062 | Yes |
| 66 | CCL4 | C-C motif chemokine ligand 4 [Source:HGNC Symbol;Acc:HGNC:10630] | 34162 | -0.145 | -0.4995 | Yes |
| 67 | AKT3 | AKT serine/threonine kinase 3 [Source:HGNC Symbol;Acc:HGNC:393] | 34501 | -0.156 | -0.4984 | Yes |
| 68 | CCL5 | C-C motif chemokine ligand 5 [Source:HGNC Symbol;Acc:HGNC:10632] | 34763 | -0.164 | -0.4949 | Yes |
| 69 | MAP2K7 | mitogen-activated protein kinase kinase 7 [Source:HGNC Symbol;Acc:HGNC:6847] | 35002 | -0.171 | -0.4903 | Yes |
| 70 | MAPK11 | mitogen-activated protein kinase 11 [Source:HGNC Symbol;Acc:HGNC:6873] | 35006 | -0.171 | -0.4797 | Yes |
| 71 | TLR8 | toll like receptor 8 [Source:HGNC Symbol;Acc:HGNC:15632] | 35059 | -0.173 | -0.4703 | Yes |
| 72 | LBP | lipopolysaccharide binding protein [Source:HGNC Symbol;Acc:HGNC:6517] | 35433 | -0.186 | -0.4682 | Yes |
| 73 | MAPK3 | mitogen-activated protein kinase 3 [Source:HGNC Symbol;Acc:HGNC:6877] | 35579 | -0.191 | -0.4600 | Yes |
| 74 | CTSK | cathepsin K [Source:HGNC Symbol;Acc:HGNC:2536] | 35626 | -0.193 | -0.4492 | Yes |
| 75 | IL1B | interleukin 1 beta [Source:HGNC Symbol;Acc:HGNC:5992] | 35858 | -0.202 | -0.4425 | Yes |
| 76 | MAP2K1 | mitogen-activated protein kinase kinase 1 [Source:HGNC Symbol;Acc:HGNC:6840] | 36024 | -0.208 | -0.4337 | Yes |
| 77 | TLR1 | toll like receptor 1 [Source:HGNC Symbol;Acc:HGNC:11847] | 36044 | -0.209 | -0.4211 | Yes |
| 78 | PIK3CG | "phosphatidylinositol-4,5-bisphosphate 3-kinase catalytic subunit gamma [Source:HGNC Symbol;Acc:HGNC:8978]" | 36243 | -0.217 | -0.4126 | Yes |
| 79 | CD14 | CD14 molecule [Source:HGNC Symbol;Acc:HGNC:1628] | 36298 | -0.220 | -0.4003 | Yes |
| 80 | TNF | tumor necrosis factor [Source:HGNC Symbol;Acc:HGNC:11892] | 36419 | -0.226 | -0.3893 | Yes |
| 81 | CASP8 | caspase 8 [Source:HGNC Symbol;Acc:HGNC:1509] | 36734 | -0.243 | -0.3821 | Yes |
| 82 | TICAM2 | toll like receptor adaptor molecule 2 [Source:HGNC Symbol;Acc:HGNC:21354] | 36839 | -0.249 | -0.3692 | Yes |
| 83 | CD40 | CD40 molecule [Source:HGNC Symbol;Acc:HGNC:11919] | 37041 | -0.262 | -0.3580 | Yes |
| 84 | PIK3R1 | phosphoinositide-3-kinase regulatory subunit 1 [Source:HGNC Symbol;Acc:HGNC:8979] | 37172 | -0.270 | -0.3445 | Yes |
| 85 | NFKBIA | NFKB inhibitor alpha [Source:HGNC Symbol;Acc:HGNC:7797] | 37222 | -0.275 | -0.3286 | Yes |
| 86 | TICAM1 | toll like receptor adaptor molecule 1 [Source:HGNC Symbol;Acc:HGNC:18348] | 37258 | -0.277 | -0.3122 | Yes |
| 87 | RAC1 | Rac family small GTPase 1 [Source:HGNC Symbol;Acc:HGNC:9801] | 37263 | -0.277 | -0.2951 | Yes |
| 88 | TLR2 | toll like receptor 2 [Source:HGNC Symbol;Acc:HGNC:11848] | 37324 | -0.282 | -0.2790 | Yes |
| 89 | TAB2 | TGF-beta activated kinase 1 (MAP3K7) binding protein 2 [Source:HGNC Symbol;Acc:HGNC:17075] | 37429 | -0.290 | -0.2635 | Yes |
| 90 | PIK3R5 | phosphoinositide-3-kinase regulatory subunit 5 [Source:HGNC Symbol;Acc:HGNC:30035] | 37628 | -0.307 | -0.2494 | Yes |
| 91 | CXCL8 | C-X-C motif chemokine ligand 8 [Source:HGNC Symbol;Acc:HGNC:6025] | 37687 | -0.313 | -0.2314 | Yes |
| 92 | MAP2K4 | mitogen-activated protein kinase kinase 4 [Source:HGNC Symbol;Acc:HGNC:6844] | 37740 | -0.317 | -0.2130 | Yes |
| 93 | MAP3K8 | mitogen-activated protein kinase kinase kinase 8 [Source:HGNC Symbol;Acc:HGNC:6860] | 38006 | -0.351 | -0.1979 | Yes |
| 94 | TRAF6 | TNF receptor associated factor 6 [Source:HGNC Symbol;Acc:HGNC:12036] | 38202 | -0.379 | -0.1792 | Yes |
| 95 | TBK1 | TANK binding kinase 1 [Source:HGNC Symbol;Acc:HGNC:11584] | 38230 | -0.384 | -0.1560 | Yes |
| 96 | MAP2K3 | mitogen-activated protein kinase kinase 3 [Source:HGNC Symbol;Acc:HGNC:6843] | 38254 | -0.389 | -0.1324 | Yes |
| 97 | PIK3CA | "phosphatidylinositol-4,5-bisphosphate 3-kinase catalytic subunit alpha [Source:HGNC Symbol;Acc:HGNC:8975]" | 38268 | -0.391 | -0.1083 | Yes |
| 98 | MAPK14 | mitogen-activated protein kinase 14 [Source:HGNC Symbol;Acc:HGNC:6876] | 38287 | -0.396 | -0.0840 | Yes |
| 99 | MAPK8 | mitogen-activated protein kinase 8 [Source:HGNC Symbol;Acc:HGNC:6881] | 38339 | -0.406 | -0.0600 | Yes |
| 100 | NFKB1 | nuclear factor kappa B subunit 1 [Source:HGNC Symbol;Acc:HGNC:7794] | 38470 | -0.443 | -0.0357 | Yes |
| 101 | RELA | "RELA proto-oncogene, NF-kB subunit [Source:HGNC Symbol;Acc:HGNC:9955]" | 38475 | -0.445 | -0.0081 | Yes |
| 102 | RIPK1 | receptor interacting serine/threonine kinase 1 [Source:HGNC Symbol;Acc:HGNC:10019] | 38544 | -0.476 | 0.0199 | Yes |
Table: GSEA details [plain text format]

  

Fig 2: KEGG\_TOLL\_LIKE\_RECEPTOR\_SIGNALING\_PATHWAY      
 Blue-Pink O' Gram in the Space of the Analyzed GeneSet

  

Fig 3: KEGG\_TOLL\_LIKE\_RECEPTOR\_SIGNALING\_PATHWAY: Random ES distribution      
 Gene set null distribution of ES for **KEGG\_TOLL\_LIKE\_RECEPTOR\_SIGNALING\_PATHWAY**
