# Supplementary material for: Simultaneous Presentation of Multiple Myeloma and Lung Cancer: Case Report and Gene Bioinformatics Analysis
Source: Front Oncol. 2022 Jun 13;12:859735. doi: 10.3389/fonc.2022.859735 (PMC9235397; doi:10.3389/fonc.2022.859735)
Supplement: Supplementary file 1 [file DataSheet_1.zip › The bioinformatic analysis of MM and lung cancer supplementary materials/Enrichment analysis/MECR/GSEA_4.1.0/MM TCGA/KEGG.Gsea.1639040591792/KEGG_T_CELL_RECEPTOR_SIGNALING_PATHWAY.html]

Details for gene set KEGG\_T\_CELL\_RECEPTOR\_SIGNALING\_PATHWAY[GSEA]

|  || Dataset | ExpData\_collapsed\_to\_symbols.ENSG00000116353\_profile\_in\_ExpData.cls #ENSG00000116353 |
| Phenotype | ENSG00000116353\_profile\_in\_ExpData.cls#ENSG00000116353 |
| Upregulated in class | ENSG00000116353\_neg |
| GeneSet | KEGG\_T\_CELL\_RECEPTOR\_SIGNALING\_PATHWAY |
| Enrichment Score (ES) | -0.5632359 |
| Normalized Enrichment Score (NES) | -2.2548454 |
| Nominal p-value | 0.0 |
| FDR q-value | 0.0 |
| FWER p-Value | 0.0 |
Table: GSEA Results Summary

  

Fig 1: Enrichment plot: KEGG\_T\_CELL\_RECEPTOR\_SIGNALING\_PATHWAY      
 Profile of the Running ES Score & Positions of GeneSet Members on the Rank Ordered List

  

| SYMBOL | TITLE | RANK IN GENE LIST | RANK METRIC SCORE | RUNNING ES | CORE ENRICHMENT || 1 | PPP3CB | protein phosphatase 3 catalytic subunit beta [Source:HGNC Symbol;Acc:HGNC:9315] | 842 | 0.363 | -0.0011 | No |
| 2 | CDK4 | cyclin dependent kinase 4 [Source:HGNC Symbol;Acc:HGNC:1773] | 1141 | 0.331 | 0.0098 | No |
| 3 | PIK3R2 | phosphoinositide-3-kinase regulatory subunit 2 [Source:HGNC Symbol;Acc:HGNC:8980] | 1248 | 0.320 | 0.0251 | No |
| 4 | PAK5 | p21 (RAC1) activated kinase 5 [Source:HGNC Symbol;Acc:HGNC:15916] | 2247 | 0.250 | 0.0136 | No |
| 5 | PIK3R3 | phosphoinositide-3-kinase regulatory subunit 3 [Source:HGNC Symbol;Acc:HGNC:8981] | 2602 | 0.231 | 0.0176 | No |
| 6 | NFATC3 | nuclear factor of activated T cells 3 [Source:HGNC Symbol;Acc:HGNC:7777] | 2677 | 0.228 | 0.0285 | No |
| 7 | FOS | "Fos proto-oncogene, AP-1 transcription factor subunit [Source:HGNC Symbol;Acc:HGNC:3796]" | 3070 | 0.210 | 0.0303 | No |
| 8 | VAV1 | vav guanine nucleotide exchange factor 1 [Source:HGNC Symbol;Acc:HGNC:12657] | 3764 | 0.184 | 0.0229 | No |
| 9 | PTPN6 | protein tyrosine phosphatase non-receptor type 6 [Source:HGNC Symbol;Acc:HGNC:9658] | 4119 | 0.173 | 0.0236 | No |
| 10 | JUN | "Jun proto-oncogene, AP-1 transcription factor subunit [Source:HGNC Symbol;Acc:HGNC:6204]" | 5088 | 0.146 | 0.0072 | No |
| 11 | MAP2K2 | mitogen-activated protein kinase kinase 2 [Source:HGNC Symbol;Acc:HGNC:6842] | 5104 | 0.146 | 0.0149 | No |
| 12 | IL5 | interleukin 5 [Source:HGNC Symbol;Acc:HGNC:6016] | 6370 | 0.120 | -0.0106 | No |
| 13 | DLG1 | discs large MAGUK scaffold protein 1 [Source:HGNC Symbol;Acc:HGNC:2900] | 6650 | 0.115 | -0.0112 | No |
| 14 | CD3E | CD3e molecule [Source:HGNC Symbol;Acc:HGNC:1674] | 7210 | 0.106 | -0.0196 | No |
| 15 | PIK3CB | "phosphatidylinositol-4,5-bisphosphate 3-kinase catalytic subunit beta [Source:HGNC Symbol;Acc:HGNC:8976]" | 7716 | 0.099 | -0.0269 | No |
| 16 | PAK2 | p21 (RAC1) activated kinase 2 [Source:HGNC Symbol;Acc:HGNC:8591] | 7725 | 0.099 | -0.0216 | No |
| 17 | NCK1 | NCK adaptor protein 1 [Source:HGNC Symbol;Acc:HGNC:7664] | 8007 | 0.095 | -0.0234 | No |
| 18 | CHUK | component of inhibitor of nuclear factor kappa B kinase complex [Source:HGNC Symbol;Acc:HGNC:1974] | 9925 | 0.071 | -0.0683 | No |
| 19 | PAK4 | p21 (RAC1) activated kinase 4 [Source:HGNC Symbol;Acc:HGNC:16059] | 11565 | 0.056 | -0.1070 | No |
| 20 | PPP3R2 | "protein phosphatase 3 regulatory subunit B, beta [Source:HGNC Symbol;Acc:HGNC:9318]" | 11820 | 0.054 | -0.1104 | No |
| 21 | GSK3B | glycogen synthase kinase 3 beta [Source:HGNC Symbol;Acc:HGNC:4617] | 12189 | 0.051 | -0.1170 | No |
| 22 | CBL | Cbl proto-oncogene [Source:HGNC Symbol;Acc:HGNC:1541] | 12468 | 0.049 | -0.1213 | No |
| 23 | MAP3K7 | mitogen-activated protein kinase kinase kinase 7 [Source:HGNC Symbol;Acc:HGNC:6859] | 12527 | 0.048 | -0.1201 | No |
| 24 | CARD11 | caspase recruitment domain family member 11 [Source:HGNC Symbol;Acc:HGNC:16393] | 13545 | 0.041 | -0.1437 | No |
| 25 | CSF2 | colony stimulating factor 2 [Source:HGNC Symbol;Acc:HGNC:2434] | 15864 | 0.027 | -0.2013 | No |
| 26 | CHP1 | calcineurin like EF-hand protein 1 [Source:HGNC Symbol;Acc:HGNC:17433] | 15945 | 0.026 | -0.2019 | No |
| 27 | NFATC4 | nuclear factor of activated T cells 4 [Source:HGNC Symbol;Acc:HGNC:7778] | 17587 | 0.017 | -0.2428 | No |
| 28 | CBLC | Cbl proto-oncogene C [Source:HGNC Symbol;Acc:HGNC:15961] | 18396 | 0.012 | -0.2627 | No |
| 29 | SOS1 | SOS Ras/Rac guanine nucleotide exchange factor 1 [Source:HGNC Symbol;Acc:HGNC:11187] | 18532 | 0.012 | -0.2655 | No |
| 30 | CD28 | CD28 molecule [Source:HGNC Symbol;Acc:HGNC:1653] | 18732 | 0.011 | -0.2700 | No |
| 31 | PRKCQ | protein kinase C theta [Source:HGNC Symbol;Acc:HGNC:9410] | 19202 | 0.008 | -0.2815 | No |
| 32 | MAPK9 | mitogen-activated protein kinase 9 [Source:HGNC Symbol;Acc:HGNC:6886] | 20291 | 0.002 | -0.3092 | No |
| 33 | IKBKB | inhibitor of nuclear factor kappa B kinase subunit beta [Source:HGNC Symbol;Acc:HGNC:5960] | 20718 | -0.000 | -0.3200 | No |
| 34 | LAT | linker for activation of T cells [Source:HGNC Symbol;Acc:HGNC:18874] | 22716 | -0.012 | -0.3703 | No |
| 35 | CHP2 | calcineurin like EF-hand protein 2 [Source:HGNC Symbol;Acc:HGNC:24927] | 23148 | -0.015 | -0.3804 | No |
| 36 | MAPK1 | mitogen-activated protein kinase 1 [Source:HGNC Symbol;Acc:HGNC:6871] | 23167 | -0.015 | -0.3800 | No |
| 37 | IL2 | interleukin 2 [Source:HGNC Symbol;Acc:HGNC:6001] | 25091 | -0.027 | -0.4276 | No |
| 38 | MAPK12 | mitogen-activated protein kinase 12 [Source:HGNC Symbol;Acc:HGNC:6874] | 25314 | -0.028 | -0.4316 | No |
| 39 | IFNG | interferon gamma [Source:HGNC Symbol;Acc:HGNC:5438] | 25529 | -0.030 | -0.4354 | No |
| 40 | PDCD1 | programmed cell death 1 [Source:HGNC Symbol;Acc:HGNC:8760] | 26253 | -0.035 | -0.4518 | No |
| 41 | CD8B | CD8b molecule [Source:HGNC Symbol;Acc:HGNC:1707] | 26459 | -0.037 | -0.4550 | No |
| 42 | HRAS | "HRas proto-oncogene, GTPase [Source:HGNC Symbol;Acc:HGNC:5173]" | 26672 | -0.039 | -0.4582 | No |
| 43 | IL4 | interleukin 4 [Source:HGNC Symbol;Acc:HGNC:6014] | 28173 | -0.052 | -0.4936 | No |
| 44 | CD4 | CD4 molecule [Source:HGNC Symbol;Acc:HGNC:1678] | 28372 | -0.054 | -0.4956 | No |
| 45 | PPP3CA | protein phosphatase 3 catalytic subunit alpha [Source:HGNC Symbol;Acc:HGNC:9314] | 28890 | -0.059 | -0.5055 | No |
| 46 | ZAP70 | zeta chain of T cell receptor associated protein kinase 70 [Source:HGNC Symbol;Acc:HGNC:12858] | 28934 | -0.060 | -0.5032 | No |
| 47 | CD8A | CD8a molecule [Source:HGNC Symbol;Acc:HGNC:1706] | 29262 | -0.063 | -0.5080 | No |
| 48 | VAV3 | vav guanine nucleotide exchange factor 3 [Source:HGNC Symbol;Acc:HGNC:12659] | 29349 | -0.065 | -0.5066 | No |
| 49 | MAPK13 | mitogen-activated protein kinase 13 [Source:HGNC Symbol;Acc:HGNC:6875] | 29402 | -0.065 | -0.5043 | No |
| 50 | CD3G | CD3g molecule [Source:HGNC Symbol;Acc:HGNC:1675] | 30583 | -0.079 | -0.5299 | No |
| 51 | ICOS | inducible T cell costimulator [Source:HGNC Symbol;Acc:HGNC:5351] | 31017 | -0.085 | -0.5362 | No |
| 52 | CD40LG | CD40 ligand [Source:HGNC Symbol;Acc:HGNC:11935] | 31532 | -0.092 | -0.5441 | No |
| 53 | AKT1 | AKT serine/threonine kinase 1 [Source:HGNC Symbol;Acc:HGNC:391] | 31906 | -0.098 | -0.5481 | No |
| 54 | IKBKG | inhibitor of nuclear factor kappa B kinase regulatory subunit gamma [Source:HGNC Symbol;Acc:HGNC:5961] | 32205 | -0.103 | -0.5500 | No |
| 55 | LCK | "LCK proto-oncogene, Src family tyrosine kinase [Source:HGNC Symbol;Acc:HGNC:6524]" | 32362 | -0.106 | -0.5480 | No |
| 56 | CTLA4 | cytotoxic T-lymphocyte associated protein 4 [Source:HGNC Symbol;Acc:HGNC:2505] | 32759 | -0.114 | -0.5517 | No |
| 57 | AKT2 | AKT serine/threonine kinase 2 [Source:HGNC Symbol;Acc:HGNC:392] | 33213 | -0.123 | -0.5563 | Yes |
| 58 | CD247 | CD247 molecule [Source:HGNC Symbol;Acc:HGNC:1677] | 33370 | -0.126 | -0.5532 | Yes |
| 59 | CD3D | CD3d molecule [Source:HGNC Symbol;Acc:HGNC:1673] | 33390 | -0.127 | -0.5466 | Yes |
| 60 | NRAS | "NRAS proto-oncogene, GTPase [Source:HGNC Symbol;Acc:HGNC:7989]" | 33739 | -0.135 | -0.5479 | Yes |
| 61 | PIK3CD | "phosphatidylinositol-4,5-bisphosphate 3-kinase catalytic subunit delta [Source:HGNC Symbol;Acc:HGNC:8977]" | 33794 | -0.137 | -0.5417 | Yes |
| 62 | FYN | "FYN proto-oncogene, Src family tyrosine kinase [Source:HGNC Symbol;Acc:HGNC:4037]" | 33801 | -0.137 | -0.5341 | Yes |
| 63 | PAK1 | p21 (RAC1) activated kinase 1 [Source:HGNC Symbol;Acc:HGNC:8590] | 33819 | -0.137 | -0.5269 | Yes |
| 64 | RASGRP1 | RAS guanyl releasing protein 1 [Source:HGNC Symbol;Acc:HGNC:9878] | 34180 | -0.146 | -0.5279 | Yes |
| 65 | NCK2 | NCK adaptor protein 2 [Source:HGNC Symbol;Acc:HGNC:7665] | 34228 | -0.148 | -0.5208 | Yes |
| 66 | PAK6 | p21 (RAC1) activated kinase 6 [Source:HGNC Symbol;Acc:HGNC:16061] | 34232 | -0.148 | -0.5126 | Yes |
| 67 | VAV2 | vav guanine nucleotide exchange factor 2 [Source:HGNC Symbol;Acc:HGNC:12658] | 34251 | -0.148 | -0.5047 | Yes |
| 68 | PAK3 | p21 (RAC1) activated kinase 3 [Source:HGNC Symbol;Acc:HGNC:8592] | 34288 | -0.149 | -0.4973 | Yes |
| 69 | IL10 | interleukin 10 [Source:HGNC Symbol;Acc:HGNC:5962] | 34351 | -0.151 | -0.4904 | Yes |
| 70 | GRAP2 | GRB2 related adaptor protein 2 [Source:HGNC Symbol;Acc:HGNC:4563] | 34407 | -0.153 | -0.4832 | Yes |
| 71 | AKT3 | AKT serine/threonine kinase 3 [Source:HGNC Symbol;Acc:HGNC:393] | 34501 | -0.156 | -0.4769 | Yes |
| 72 | PLCG1 | phospholipase C gamma 1 [Source:HGNC Symbol;Acc:HGNC:9065] | 34842 | -0.166 | -0.4763 | Yes |
| 73 | LCP2 | lymphocyte cytosolic protein 2 [Source:HGNC Symbol;Acc:HGNC:6529] | 34973 | -0.170 | -0.4701 | Yes |
| 74 | ITK | IL2 inducible T cell kinase [Source:HGNC Symbol;Acc:HGNC:6171] | 34994 | -0.170 | -0.4610 | Yes |
| 75 | MAP2K7 | mitogen-activated protein kinase kinase 7 [Source:HGNC Symbol;Acc:HGNC:6847] | 35002 | -0.171 | -0.4516 | Yes |
| 76 | MAPK11 | mitogen-activated protein kinase 11 [Source:HGNC Symbol;Acc:HGNC:6873] | 35006 | -0.171 | -0.4421 | Yes |
| 77 | PTPRC | protein tyrosine phosphatase receptor type C [Source:HGNC Symbol;Acc:HGNC:9666] | 35256 | -0.179 | -0.4384 | Yes |
| 78 | MAPK3 | mitogen-activated protein kinase 3 [Source:HGNC Symbol;Acc:HGNC:6877] | 35579 | -0.191 | -0.4359 | Yes |
| 79 | CBLB | Cbl proto-oncogene B [Source:HGNC Symbol;Acc:HGNC:1542] | 35750 | -0.197 | -0.4292 | Yes |
| 80 | MAP2K1 | mitogen-activated protein kinase kinase 1 [Source:HGNC Symbol;Acc:HGNC:6840] | 36024 | -0.208 | -0.4244 | Yes |
| 81 | MALT1 | MALT1 paracaspase [Source:HGNC Symbol;Acc:HGNC:6819] | 36092 | -0.211 | -0.4143 | Yes |
| 82 | PIK3CG | "phosphatidylinositol-4,5-bisphosphate 3-kinase catalytic subunit gamma [Source:HGNC Symbol;Acc:HGNC:8978]" | 36243 | -0.217 | -0.4059 | Yes |
| 83 | TNF | tumor necrosis factor [Source:HGNC Symbol;Acc:HGNC:11892] | 36419 | -0.226 | -0.3977 | Yes |
| 84 | GRB2 | growth factor receptor bound protein 2 [Source:HGNC Symbol;Acc:HGNC:4566] | 36523 | -0.231 | -0.3874 | Yes |
| 85 | NFATC2 | nuclear factor of activated T cells 2 [Source:HGNC Symbol;Acc:HGNC:7776] | 36649 | -0.238 | -0.3772 | Yes |
| 86 | MAP3K14 | mitogen-activated protein kinase kinase kinase 14 [Source:HGNC Symbol;Acc:HGNC:6853] | 36852 | -0.251 | -0.3683 | Yes |
| 87 | TEC | tec protein tyrosine kinase [Source:HGNC Symbol;Acc:HGNC:11719] | 36879 | -0.252 | -0.3548 | Yes |
| 88 | PDPK1 | 3-phosphoinositide dependent protein kinase 1 [Source:HGNC Symbol;Acc:HGNC:8816] | 36889 | -0.253 | -0.3409 | Yes |
| 89 | NFAT5 | nuclear factor of activated T cells 5 [Source:HGNC Symbol;Acc:HGNC:7774] | 37139 | -0.268 | -0.3322 | Yes |
| 90 | PIK3R1 | phosphoinositide-3-kinase regulatory subunit 1 [Source:HGNC Symbol;Acc:HGNC:8979] | 37172 | -0.270 | -0.3178 | Yes |
| 91 | NFKBIA | NFKB inhibitor alpha [Source:HGNC Symbol;Acc:HGNC:7797] | 37222 | -0.275 | -0.3037 | Yes |
| 92 | RHOA | ras homolog family member A [Source:HGNC Symbol;Acc:HGNC:667] | 37417 | -0.290 | -0.2924 | Yes |
| 93 | NFATC1 | nuclear factor of activated T cells 1 [Source:HGNC Symbol;Acc:HGNC:7775] | 37512 | -0.297 | -0.2781 | Yes |
| 94 | PIK3R5 | phosphoinositide-3-kinase regulatory subunit 5 [Source:HGNC Symbol;Acc:HGNC:30035] | 37628 | -0.307 | -0.2638 | Yes |
| 95 | KRAS | "KRAS proto-oncogene, GTPase [Source:HGNC Symbol;Acc:HGNC:6407]" | 37789 | -0.323 | -0.2498 | Yes |
| 96 | CDC42 | cell division cycle 42 [Source:HGNC Symbol;Acc:HGNC:1736] | 37805 | -0.324 | -0.2320 | Yes |
| 97 | RAF1 | "Raf-1 proto-oncogene, serine/threonine kinase [Source:HGNC Symbol;Acc:HGNC:9829]" | 37807 | -0.325 | -0.2138 | Yes |
| 98 | NFKBIB | NFKB inhibitor beta [Source:HGNC Symbol;Acc:HGNC:7798] | 37987 | -0.348 | -0.1989 | Yes |
| 99 | PPP3R1 | "protein phosphatase 3 regulatory subunit B, alpha [Source:HGNC Symbol;Acc:HGNC:9317]" | 37988 | -0.348 | -0.1793 | Yes |
| 100 | MAP3K8 | mitogen-activated protein kinase kinase kinase 8 [Source:HGNC Symbol;Acc:HGNC:6860] | 38006 | -0.351 | -0.1601 | Yes |
| 101 | PPP3CC | protein phosphatase 3 catalytic subunit gamma [Source:HGNC Symbol;Acc:HGNC:9316] | 38255 | -0.389 | -0.1446 | Yes |
| 102 | PIK3CA | "phosphatidylinositol-4,5-bisphosphate 3-kinase catalytic subunit alpha [Source:HGNC Symbol;Acc:HGNC:8975]" | 38268 | -0.391 | -0.1230 | Yes |
| 103 | MAPK14 | mitogen-activated protein kinase 14 [Source:HGNC Symbol;Acc:HGNC:6876] | 38287 | -0.396 | -0.1012 | Yes |
| 104 | NFKBIE | NFKB inhibitor epsilon [Source:HGNC Symbol;Acc:HGNC:7799] | 38410 | -0.422 | -0.0806 | Yes |
| 105 | BCL10 | BCL10 immune signaling adaptor [Source:HGNC Symbol;Acc:HGNC:989] | 38451 | -0.436 | -0.0572 | Yes |
| 106 | NFKB1 | nuclear factor kappa B subunit 1 [Source:HGNC Symbol;Acc:HGNC:7794] | 38470 | -0.443 | -0.0329 | Yes |
| 107 | RELA | "RELA proto-oncogene, NF-kB subunit [Source:HGNC Symbol;Acc:HGNC:9955]" | 38475 | -0.445 | -0.0080 | Yes |
| 108 | SOS2 | SOS Ras/Rho guanine nucleotide exchange factor 2 [Source:HGNC Symbol;Acc:HGNC:11188] | 38581 | -0.528 | 0.0189 | Yes |
Table: GSEA details [plain text format]

  

Fig 2: KEGG\_T\_CELL\_RECEPTOR\_SIGNALING\_PATHWAY      
 Blue-Pink O' Gram in the Space of the Analyzed GeneSet

  

Fig 3: KEGG\_T\_CELL\_RECEPTOR\_SIGNALING\_PATHWAY: Random ES distribution      
 Gene set null distribution of ES for **KEGG\_T\_CELL\_RECEPTOR\_SIGNALING\_PATHWAY**
