# Supplementary material for: Simultaneous Presentation of Multiple Myeloma and Lung Cancer: Case Report and Gene Bioinformatics Analysis
Source: Front Oncol. 2022 Jun 13;12:859735. doi: 10.3389/fonc.2022.859735 (PMC9235397; doi:10.3389/fonc.2022.859735)
Supplement: Supplementary file 1 [file DataSheet_1.zip › The bioinformatic analysis of MM and lung cancer supplementary materials/Enrichment analysis/MECR/GSEA_4.1.0/MM TCGA/KEGG.Gsea.1639040591792/KEGG_VALINE_LEUCINE_AND_ISOLEUCINE_DEGRADATION.html]

Details for gene set KEGG\_VALINE\_LEUCINE\_AND\_ISOLEUCINE\_DEGRADATION[GSEA]

|  || Dataset | ExpData\_collapsed\_to\_symbols.ENSG00000116353\_profile\_in\_ExpData.cls #ENSG00000116353 |
| Phenotype | ENSG00000116353\_profile\_in\_ExpData.cls#ENSG00000116353 |
| Upregulated in class | ENSG00000116353\_pos |
| GeneSet | KEGG\_VALINE\_LEUCINE\_AND\_ISOLEUCINE\_DEGRADATION |
| Enrichment Score (ES) | 0.7381813 |
| Normalized Enrichment Score (NES) | 2.3164513 |
| Nominal p-value | 0.0 |
| FDR q-value | 0.0 |
| FWER p-Value | 0.0 |
Table: GSEA Results Summary

  

Fig 1: Enrichment plot: KEGG\_VALINE\_LEUCINE\_AND\_ISOLEUCINE\_DEGRADATION      
 Profile of the Running ES Score & Positions of GeneSet Members on the Rank Ordered List

  

| SYMBOL | TITLE | RANK IN GENE LIST | RANK METRIC SCORE | RUNNING ES | CORE ENRICHMENT || 1 | BCKDHB | branched chain keto acid dehydrogenase E1 subunit beta [Source:HGNC Symbol;Acc:HGNC:987] | 39 | 0.582 | 0.0521 | Yes |
| 2 | PCCB | propionyl-CoA carboxylase subunit beta [Source:HGNC Symbol;Acc:HGNC:8654] | 40 | 0.581 | 0.1052 | Yes |
| 3 | MCCC2 | methylcrotonoyl-CoA carboxylase 2 [Source:HGNC Symbol;Acc:HGNC:6937] | 133 | 0.509 | 0.1493 | Yes |
| 4 | BCAT2 | branched chain amino acid transaminase 2 [Source:HGNC Symbol;Acc:HGNC:977] | 195 | 0.486 | 0.1921 | Yes |
| 5 | MCCC1 | methylcrotonoyl-CoA carboxylase 1 [Source:HGNC Symbol;Acc:HGNC:6936] | 289 | 0.457 | 0.2315 | Yes |
| 6 | ALDH1B1 | aldehyde dehydrogenase 1 family member B1 [Source:HGNC Symbol;Acc:HGNC:407] | 372 | 0.439 | 0.2695 | Yes |
| 7 | HADHB | hydroxyacyl-CoA dehydrogenase trifunctional multienzyme complex subunit beta [Source:HGNC Symbol;Acc:HGNC:4803] | 511 | 0.410 | 0.3035 | Yes |
| 8 | OXCT1 | 3-oxoacid CoA-transferase 1 [Source:HGNC Symbol;Acc:HGNC:8527] | 530 | 0.407 | 0.3402 | Yes |
| 9 | BCKDHA | branched chain keto acid dehydrogenase E1 subunit alpha [Source:HGNC Symbol;Acc:HGNC:986] | 791 | 0.371 | 0.3674 | Yes |
| 10 | ACADM | acyl-CoA dehydrogenase medium chain [Source:HGNC Symbol;Acc:HGNC:89] | 803 | 0.369 | 0.4008 | Yes |
| 11 | ECHS1 | "enoyl-CoA hydratase, short chain 1 [Source:HGNC Symbol;Acc:HGNC:3151]" | 890 | 0.356 | 0.4311 | Yes |
| 12 | ALDH3A2 | aldehyde dehydrogenase 3 family member A2 [Source:HGNC Symbol;Acc:HGNC:403] | 902 | 0.354 | 0.4632 | Yes |
| 13 | HSD17B10 | hydroxysteroid 17-beta dehydrogenase 10 [Source:HGNC Symbol;Acc:HGNC:4800] | 1127 | 0.332 | 0.4878 | Yes |
| 14 | HIBADH | 3-hydroxyisobutyrate dehydrogenase [Source:HGNC Symbol;Acc:HGNC:4907] | 1128 | 0.332 | 0.5181 | Yes |
| 15 | HIBCH | 3-hydroxyisobutyryl-CoA hydrolase [Source:HGNC Symbol;Acc:HGNC:4908] | 1265 | 0.319 | 0.5438 | Yes |
| 16 | ALDH9A1 | aldehyde dehydrogenase 9 family member A1 [Source:HGNC Symbol;Acc:HGNC:412] | 1406 | 0.306 | 0.5682 | Yes |
| 17 | ACAT1 | acetyl-CoA acetyltransferase 1 [Source:HGNC Symbol;Acc:HGNC:93] | 1467 | 0.302 | 0.5942 | Yes |
| 18 | IVD | isovaleryl-CoA dehydrogenase [Source:HGNC Symbol;Acc:HGNC:6186] | 1530 | 0.297 | 0.6197 | Yes |
| 19 | ALDH6A1 | aldehyde dehydrogenase 6 family member A1 [Source:HGNC Symbol;Acc:HGNC:7179] | 1585 | 0.293 | 0.6451 | Yes |
| 20 | HADH | hydroxyacyl-CoA dehydrogenase [Source:HGNC Symbol;Acc:HGNC:4799] | 1802 | 0.276 | 0.6648 | Yes |
| 21 | ACADSB | acyl-CoA dehydrogenase short/branched chain [Source:HGNC Symbol;Acc:HGNC:91] | 1889 | 0.271 | 0.6873 | Yes |
| 22 | EHHADH | enoyl-CoA hydratase and 3-hydroxyacyl CoA dehydrogenase [Source:HGNC Symbol;Acc:HGNC:3247] | 2104 | 0.258 | 0.7054 | Yes |
| 23 | MMUT | methylmalonyl-CoA mutase [Source:HGNC Symbol;Acc:HGNC:7526] | 3141 | 0.207 | 0.6980 | Yes |
| 24 | DLD | dihydrolipoamide dehydrogenase [Source:HGNC Symbol;Acc:HGNC:2898] | 3616 | 0.189 | 0.7032 | Yes |
| 25 | AUH | AU RNA binding methylglutaconyl-CoA hydratase [Source:HGNC Symbol;Acc:HGNC:890] | 3753 | 0.185 | 0.7166 | Yes |
| 26 | HMGCL | 3-hydroxy-3-methylglutaryl-CoA lyase [Source:HGNC Symbol;Acc:HGNC:5005] | 4068 | 0.174 | 0.7245 | Yes |
| 27 | MCEE | methylmalonyl-CoA epimerase [Source:HGNC Symbol;Acc:HGNC:16732] | 4150 | 0.172 | 0.7382 | Yes |
| 28 | PCCA | propionyl-CoA carboxylase subunit alpha [Source:HGNC Symbol;Acc:HGNC:8653] | 6263 | 0.122 | 0.6955 | No |
| 29 | ABAT | 4-aminobutyrate aminotransferase [Source:HGNC Symbol;Acc:HGNC:23] | 6280 | 0.122 | 0.7062 | No |
| 30 | HADHA | hydroxyacyl-CoA dehydrogenase trifunctional multienzyme complex subunit alpha [Source:HGNC Symbol;Acc:HGNC:4801] | 8241 | 0.092 | 0.6647 | No |
| 31 | ACADS | acyl-CoA dehydrogenase short chain [Source:HGNC Symbol;Acc:HGNC:90] | 9834 | 0.073 | 0.6308 | No |
| 32 | ACAT2 | acetyl-CoA acetyltransferase 2 [Source:HGNC Symbol;Acc:HGNC:94] | 10235 | 0.068 | 0.6269 | No |
| 33 | BCAT1 | branched chain amino acid transaminase 1 [Source:HGNC Symbol;Acc:HGNC:976] | 11329 | 0.058 | 0.6043 | No |
| 34 | ALDH7A1 | aldehyde dehydrogenase 7 family member A1 [Source:HGNC Symbol;Acc:HGNC:877] | 14303 | 0.036 | 0.5320 | No |
| 35 | DBT | dihydrolipoamide branched chain transacylase E2 [Source:HGNC Symbol;Acc:HGNC:2698] | 18587 | 0.011 | 0.4240 | No |
| 36 | ACAA2 | acetyl-CoA acyltransferase 2 [Source:HGNC Symbol;Acc:HGNC:83] | 22513 | -0.011 | 0.3250 | No |
| 37 | AOX1 | aldehyde oxidase 1 [Source:HGNC Symbol;Acc:HGNC:553] | 24379 | -0.022 | 0.2796 | No |
| 38 | HMGCS2 | 3-hydroxy-3-methylglutaryl-CoA synthase 2 [Source:HGNC Symbol;Acc:HGNC:5008] | 29182 | -0.063 | 0.1630 | No |
| 39 | OXCT2 | 3-oxoacid CoA-transferase 2 [Source:HGNC Symbol;Acc:HGNC:18606] | 30341 | -0.076 | 0.1405 | No |
| 40 | HMGCS1 | 3-hydroxy-3-methylglutaryl-CoA synthase 1 [Source:HGNC Symbol;Acc:HGNC:5007] | 34042 | -0.142 | 0.0593 | No |
| 41 | ALDH2 | aldehyde dehydrogenase 2 family member [Source:HGNC Symbol;Acc:HGNC:404] | 34564 | -0.157 | 0.0604 | No |
| 42 | ACAA1 | acetyl-CoA acyltransferase 1 [Source:HGNC Symbol;Acc:HGNC:82] | 36185 | -0.215 | 0.0388 | No |
| 43 | ACAD8 | acyl-CoA dehydrogenase family member 8 [Source:HGNC Symbol;Acc:HGNC:87] | 36363 | -0.224 | 0.0547 | No |
| 44 | IL4I1 | interleukin 4 induced 1 [Source:HGNC Symbol;Acc:HGNC:19094] | 36405 | -0.226 | 0.0743 | No |
Table: GSEA details [plain text format]

  

Fig 2: KEGG\_VALINE\_LEUCINE\_AND\_ISOLEUCINE\_DEGRADATION      
 Blue-Pink O' Gram in the Space of the Analyzed GeneSet

  

Fig 3: KEGG\_VALINE\_LEUCINE\_AND\_ISOLEUCINE\_DEGRADATION: Random ES distribution      
 Gene set null distribution of ES for **KEGG\_VALINE\_LEUCINE\_AND\_ISOLEUCINE\_DEGRADATION**
