# Supplementary material for: Simultaneous Presentation of Multiple Myeloma and Lung Cancer: Case Report and Gene Bioinformatics Analysis
Source: Front Oncol. 2022 Jun 13;12:859735. doi: 10.3389/fonc.2022.859735 (PMC9235397; doi:10.3389/fonc.2022.859735)
Supplement: Supplementary file 1 [file DataSheet_1.zip › The bioinformatic analysis of MM and lung cancer supplementary materials/Enrichment analysis/MECR/GSEA_4.1.0/MM TCGA/KEGG.Gsea.1639040591792/gsea_report_for_ENSG00000116353_neg_1639040591792.html]

Report for ENSG00000116353\_neg 1639040591792 [GSEA]

| GS  follow link to MSigDB | GS DETAILS | SIZE | ES | NES | NOM p-val | FDR q-val | FWER p-val | RANK AT MAX | LEADING EDGE || 1 | KEGG\_CHEMOKINE\_SIGNALING\_PATHWAY | Details ... | 188 | -0.58 | -2.54 | 0.000 | 0.000 | 0.000 | 8126 | tags=56%, list=21%, signal=71% |
| 2 | KEGG\_NEUROTROPHIN\_SIGNALING\_PATHWAY | Details ... | 126 | -0.59 | -2.49 | 0.000 | 0.000 | 0.000 | 5654 | tags=48%, list=14%, signal=56% |
| 3 | KEGG\_FOCAL\_ADHESION | Details ... | 199 | -0.57 | -2.48 | 0.000 | 0.000 | 0.000 | 7527 | tags=54%, list=19%, signal=66% |
| 4 | KEGG\_CYTOKINE\_CYTOKINE\_RECEPTOR\_INTERACTION | Details ... | 264 | -0.55 | -2.47 | 0.000 | 0.000 | 0.000 | 7914 | tags=49%, list=20%, signal=61% |
| 5 | KEGG\_LEUKOCYTE\_TRANSENDOTHELIAL\_MIGRATION | Details ... | 116 | -0.60 | -2.45 | 0.000 | 0.000 | 0.000 | 7039 | tags=55%, list=18%, signal=67% |
| 6 | KEGG\_DORSO\_VENTRAL\_AXIS\_FORMATION | Details ... | 24 | -0.81 | -2.42 | 0.000 | 0.000 | 0.000 | 4043 | tags=58%, list=10%, signal=65% |
| 7 | KEGG\_ADHERENS\_JUNCTION | Details ... | 73 | -0.64 | -2.42 | 0.000 | 0.000 | 0.000 | 6633 | tags=55%, list=17%, signal=66% |
| 8 | KEGG\_MAPK\_SIGNALING\_PATHWAY | Details ... | 267 | -0.53 | -2.40 | 0.000 | 0.000 | 0.000 | 6308 | tags=42%, list=16%, signal=50% |
| 9 | KEGG\_REGULATION\_OF\_ACTIN\_CYTOSKELETON | Details ... | 213 | -0.53 | -2.38 | 0.000 | 0.000 | 0.000 | 8629 | tags=52%, list=22%, signal=66% |
| 10 | KEGG\_ACUTE\_MYELOID\_LEUKEMIA | Details ... | 57 | -0.63 | -2.36 | 0.000 | 0.000 | 0.000 | 5586 | tags=54%, list=14%, signal=63% |
| 11 | KEGG\_NOD\_LIKE\_RECEPTOR\_SIGNALING\_PATHWAY | Details ... | 62 | -0.64 | -2.32 | 0.000 | 0.000 | 0.000 | 5773 | tags=52%, list=15%, signal=60% |
| 12 | KEGG\_LEISHMANIA\_INFECTION | Details ... | 70 | -0.61 | -2.32 | 0.000 | 0.000 | 0.000 | 7721 | tags=56%, list=20%, signal=69% |
| 13 | KEGG\_T\_CELL\_RECEPTOR\_SIGNALING\_PATHWAY | Details ... | 108 | -0.56 | -2.25 | 0.000 | 0.000 | 0.000 | 6112 | tags=48%, list=16%, signal=57% |
| 14 | KEGG\_PROSTATE\_CANCER | Details ... | 89 | -0.57 | -2.21 | 0.000 | 0.000 | 0.000 | 6190 | tags=54%, list=16%, signal=64% |
| 15 | KEGG\_B\_CELL\_RECEPTOR\_SIGNALING\_PATHWAY | Details ... | 75 | -0.59 | -2.20 | 0.000 | 0.000 | 0.000 | 7419 | tags=60%, list=19%, signal=74% |
| 16 | KEGG\_TOLL\_LIKE\_RECEPTOR\_SIGNALING\_PATHWAY | Details ... | 102 | -0.54 | -2.18 | 0.000 | 0.000 | 0.000 | 6112 | tags=43%, list=16%, signal=51% |
| 17 | KEGG\_HEMATOPOIETIC\_CELL\_LINEAGE | Details ... | 85 | -0.56 | -2.18 | 0.000 | 0.000 | 0.000 | 8821 | tags=58%, list=22%, signal=74% |
| 18 | KEGG\_GRAFT\_VERSUS\_HOST\_DISEASE | Details ... | 37 | -0.64 | -2.17 | 0.000 | 0.000 | 0.000 | 11202 | tags=73%, list=28%, signal=102% |
| 19 | KEGG\_ENDOCYTOSIS | Details ... | 181 | -0.49 | -2.15 | 0.000 | 0.000 | 0.000 | 4862 | tags=37%, list=12%, signal=42% |
| 20 | KEGG\_FC\_GAMMA\_R\_MEDIATED\_PHAGOCYTOSIS | Details ... | 96 | -0.54 | -2.14 | 0.000 | 0.000 | 0.000 | 7886 | tags=53%, list=20%, signal=66% |
| 21 | KEGG\_JAK\_STAT\_SIGNALING\_PATHWAY |  | 155 | -0.49 | -2.12 | 0.000 | 0.000 | 0.000 | 7854 | tags=42%, list=20%, signal=52% |
| 22 | KEGG\_FC\_EPSILON\_RI\_SIGNALING\_PATHWAY |  | 79 | -0.55 | -2.12 | 0.000 | 0.000 | 0.000 | 6308 | tags=46%, list=16%, signal=54% |
| 23 | KEGG\_INTESTINAL\_IMMUNE\_NETWORK\_FOR\_IGA\_PRODUCTION |  | 46 | -0.60 | -2.11 | 0.000 | 0.000 | 0.000 | 8744 | tags=65%, list=22%, signal=84% |
| 24 | KEGG\_ASTHMA |  | 28 | -0.68 | -2.11 | 0.000 | 0.000 | 0.000 | 7793 | tags=64%, list=20%, signal=80% |
| 25 | KEGG\_TGF\_BETA\_SIGNALING\_PATHWAY |  | 86 | -0.54 | -2.11 | 0.000 | 0.000 | 0.000 | 6653 | tags=42%, list=17%, signal=50% |
| 26 | KEGG\_ERBB\_SIGNALING\_PATHWAY |  | 87 | -0.54 | -2.07 | 0.000 | 0.000 | 0.001 | 7419 | tags=52%, list=19%, signal=64% |
| 27 | KEGG\_RENAL\_CELL\_CARCINOMA |  | 70 | -0.56 | -2.06 | 0.000 | 0.000 | 0.001 | 6112 | tags=50%, list=16%, signal=59% |
| 28 | KEGG\_GNRH\_SIGNALING\_PATHWAY |  | 101 | -0.51 | -2.06 | 0.000 | 0.000 | 0.001 | 6334 | tags=37%, list=16%, signal=44% |
| 29 | KEGG\_PATHWAYS\_IN\_CANCER |  | 325 | -0.45 | -2.06 | 0.000 | 0.000 | 0.001 | 7474 | tags=43%, list=19%, signal=53% |
| 30 | KEGG\_ALLOGRAFT\_REJECTION |  | 35 | -0.62 | -2.02 | 0.000 | 0.000 | 0.003 | 8486 | tags=63%, list=22%, signal=80% |
| 31 | KEGG\_ALDOSTERONE\_REGULATED\_SODIUM\_REABSORPTION |  | 42 | -0.58 | -2.01 | 0.000 | 0.000 | 0.003 | 6493 | tags=55%, list=17%, signal=66% |
| 32 | KEGG\_INSULIN\_SIGNALING\_PATHWAY |  | 137 | -0.48 | -1.99 | 0.000 | 0.000 | 0.005 | 6297 | tags=41%, list=16%, signal=48% |
| 33 | KEGG\_EPITHELIAL\_CELL\_SIGNALING\_IN\_HELICOBACTER\_PYLORI\_INFECTION |  | 68 | -0.53 | -1.98 | 0.000 | 0.000 | 0.006 | 7120 | tags=43%, list=18%, signal=52% |
| 34 | KEGG\_CHRONIC\_MYELOID\_LEUKEMIA |  | 73 | -0.53 | -1.98 | 0.000 | 0.000 | 0.006 | 5586 | tags=44%, list=14%, signal=51% |
| 35 | KEGG\_THYROID\_CANCER |  | 29 | -0.63 | -1.95 | 0.000 | 0.000 | 0.009 | 5799 | tags=55%, list=15%, signal=65% |
| 36 | KEGG\_ENDOMETRIAL\_CANCER |  | 52 | -0.55 | -1.94 | 0.003 | 0.000 | 0.009 | 6297 | tags=56%, list=16%, signal=66% |
| 37 | KEGG\_AUTOIMMUNE\_THYROID\_DISEASE |  | 50 | -0.55 | -1.94 | 0.000 | 0.000 | 0.009 | 11201 | tags=62%, list=28%, signal=87% |
| 38 | KEGG\_NATURAL\_KILLER\_CELL\_MEDIATED\_CYTOTOXICITY |  | 132 | -0.47 | -1.93 | 0.000 | 0.000 | 0.013 | 8045 | tags=41%, list=20%, signal=51% |
| 39 | KEGG\_TIGHT\_JUNCTION |  | 132 | -0.46 | -1.92 | 0.000 | 0.001 | 0.014 | 8601 | tags=48%, list=22%, signal=61% |
| 40 | KEGG\_TYPE\_I\_DIABETES\_MELLITUS |  | 41 | -0.55 | -1.92 | 0.000 | 0.001 | 0.015 | 9363 | tags=61%, list=24%, signal=80% |
| 41 | KEGG\_PANCREATIC\_CANCER |  | 70 | -0.51 | -1.92 | 0.000 | 0.001 | 0.015 | 6190 | tags=46%, list=16%, signal=54% |
| 42 | KEGG\_RIG\_I\_LIKE\_RECEPTOR\_SIGNALING\_PATHWAY |  | 71 | -0.51 | -1.90 | 0.000 | 0.001 | 0.016 | 2906 | tags=24%, list=7%, signal=26% |
| 43 | KEGG\_LONG\_TERM\_POTENTIATION |  | 70 | -0.50 | -1.88 | 0.000 | 0.001 | 0.022 | 6308 | tags=41%, list=16%, signal=49% |
| 44 | KEGG\_WNT\_SIGNALING\_PATHWAY |  | 151 | -0.45 | -1.86 | 0.000 | 0.001 | 0.027 | 8219 | tags=46%, list=21%, signal=58% |
| 45 | KEGG\_MELANOMA |  | 71 | -0.49 | -1.83 | 0.000 | 0.002 | 0.044 | 6190 | tags=41%, list=16%, signal=48% |
| 46 | KEGG\_CELL\_ADHESION\_MOLECULES\_CAMS |  | 131 | -0.43 | -1.82 | 0.000 | 0.002 | 0.055 | 8583 | tags=47%, list=22%, signal=59% |
| 47 | KEGG\_ADIPOCYTOKINE\_SIGNALING\_PATHWAY |  | 67 | -0.49 | -1.80 | 0.000 | 0.002 | 0.073 | 6112 | tags=37%, list=16%, signal=44% |
| 48 | KEGG\_MTOR\_SIGNALING\_PATHWAY |  | 52 | -0.50 | -1.80 | 0.003 | 0.002 | 0.074 | 6112 | tags=46%, list=16%, signal=55% |
| 49 | KEGG\_NOTCH\_SIGNALING\_PATHWAY |  | 47 | -0.52 | -1.80 | 0.000 | 0.003 | 0.079 | 5858 | tags=51%, list=15%, signal=60% |
| 50 | KEGG\_VASOPRESSIN\_REGULATED\_WATER\_REABSORPTION |  | 44 | -0.52 | -1.79 | 0.000 | 0.003 | 0.084 | 4437 | tags=34%, list=11%, signal=38% |
| 51 | KEGG\_ECM\_RECEPTOR\_INTERACTION |  | 84 | -0.45 | -1.78 | 0.000 | 0.003 | 0.097 | 8352 | tags=50%, list=21%, signal=63% |
| 52 | KEGG\_GAP\_JUNCTION |  | 90 | -0.45 | -1.77 | 0.000 | 0.003 | 0.105 | 6308 | tags=37%, list=16%, signal=44% |
| 53 | KEGG\_AXON\_GUIDANCE |  | 129 | -0.43 | -1.76 | 0.000 | 0.004 | 0.116 | 7040 | tags=37%, list=18%, signal=45% |
| 54 | KEGG\_TYPE\_II\_DIABETES\_MELLITUS |  | 47 | -0.50 | -1.76 | 0.006 | 0.004 | 0.118 | 7806 | tags=45%, list=20%, signal=56% |
| 55 | KEGG\_COLORECTAL\_CANCER |  | 62 | -0.47 | -1.72 | 0.000 | 0.006 | 0.183 | 5531 | tags=47%, list=14%, signal=54% |
| 56 | KEGG\_VEGF\_SIGNALING\_PATHWAY |  | 76 | -0.45 | -1.71 | 0.000 | 0.007 | 0.220 | 6308 | tags=42%, list=16%, signal=50% |
| 57 | KEGG\_NON\_SMALL\_CELL\_LUNG\_CANCER |  | 54 | -0.47 | -1.70 | 0.008 | 0.007 | 0.225 | 6308 | tags=48%, list=16%, signal=57% |
| 58 | KEGG\_BLADDER\_CANCER |  | 42 | -0.49 | -1.67 | 0.000 | 0.009 | 0.290 | 6430 | tags=45%, list=16%, signal=54% |
| 59 | KEGG\_PATHOGENIC\_ESCHERICHIA\_COLI\_INFECTION |  | 56 | -0.47 | -1.67 | 0.003 | 0.009 | 0.295 | 6308 | tags=39%, list=16%, signal=47% |
| 60 | KEGG\_SMALL\_CELL\_LUNG\_CANCER |  | 84 | -0.43 | -1.66 | 0.003 | 0.009 | 0.315 | 4826 | tags=37%, list=12%, signal=42% |
| 61 | KEGG\_VIRAL\_MYOCARDITIS |  | 68 | -0.44 | -1.65 | 0.000 | 0.011 | 0.350 | 8621 | tags=51%, list=22%, signal=66% |
| 62 | KEGG\_VASCULAR\_SMOOTH\_MUSCLE\_CONTRACTION |  | 115 | -0.41 | -1.65 | 0.000 | 0.011 | 0.352 | 7839 | tags=34%, list=20%, signal=42% |
| 63 | KEGG\_PHOSPHATIDYLINOSITOL\_SIGNALING\_SYSTEM |  | 76 | -0.43 | -1.64 | 0.003 | 0.011 | 0.365 | 6308 | tags=37%, list=16%, signal=44% |
| 64 | KEGG\_MELANOGENESIS |  | 101 | -0.41 | -1.64 | 0.000 | 0.011 | 0.370 | 9179 | tags=44%, list=23%, signal=57% |
| 65 | KEGG\_GLIOMA |  | 65 | -0.45 | -1.64 | 0.006 | 0.011 | 0.375 | 6308 | tags=45%, list=16%, signal=53% |
| 66 | KEGG\_UBIQUITIN\_MEDIATED\_PROTEOLYSIS |  | 135 | -0.39 | -1.61 | 0.000 | 0.014 | 0.449 | 3895 | tags=31%, list=10%, signal=34% |
| 67 | KEGG\_APOPTOSIS |  | 87 | -0.40 | -1.56 | 0.003 | 0.020 | 0.599 | 3467 | tags=30%, list=9%, signal=33% |
| 68 | KEGG\_SPLICEOSOME |  | 127 | -0.37 | -1.55 | 0.003 | 0.022 | 0.632 | 6104 | tags=42%, list=16%, signal=49% |
| 69 | KEGG\_SNARE\_INTERACTIONS\_IN\_VESICULAR\_TRANSPORT |  | 38 | -0.45 | -1.50 | 0.035 | 0.032 | 0.764 | 4537 | tags=37%, list=12%, signal=42% |
| 70 | KEGG\_ARRHYTHMOGENIC\_RIGHT\_VENTRICULAR\_CARDIOMYOPATHY\_ARVC |  | 74 | -0.40 | -1.49 | 0.003 | 0.034 | 0.791 | 8166 | tags=38%, list=21%, signal=48% |
| 71 | KEGG\_LONG\_TERM\_DEPRESSION |  | 70 | -0.39 | -1.46 | 0.024 | 0.041 | 0.845 | 8148 | tags=40%, list=21%, signal=50% |
| 72 | KEGG\_GLYCOSPHINGOLIPID\_BIOSYNTHESIS\_LACTO\_AND\_NEOLACTO\_SERIES |  | 26 | -0.48 | -1.44 | 0.052 | 0.047 | 0.884 | 7223 | tags=54%, list=18%, signal=66% |
| 73 | KEGG\_COMPLEMENT\_AND\_COAGULATION\_CASCADES |  | 69 | -0.39 | -1.43 | 0.020 | 0.048 | 0.891 | 9225 | tags=52%, list=23%, signal=68% |
| 74 | KEGG\_REGULATION\_OF\_AUTOPHAGY |  | 35 | -0.44 | -1.42 | 0.043 | 0.052 | 0.919 | 7504 | tags=37%, list=19%, signal=46% |
| 75 | KEGG\_PPAR\_SIGNALING\_PATHWAY |  | 69 | -0.36 | -1.35 | 0.036 | 0.082 | 0.979 | 5790 | tags=30%, list=15%, signal=36% |
| 76 | KEGG\_DILATED\_CARDIOMYOPATHY |  | 90 | -0.35 | -1.35 | 0.047 | 0.081 | 0.979 | 8636 | tags=33%, list=22%, signal=43% |
| 77 | KEGG\_P53\_SIGNALING\_PATHWAY |  | 68 | -0.36 | -1.34 | 0.038 | 0.088 | 0.988 | 6917 | tags=46%, list=18%, signal=55% |
| 78 | KEGG\_HYPERTROPHIC\_CARDIOMYOPATHY\_HCM |  | 83 | -0.34 | -1.32 | 0.066 | 0.094 | 0.991 | 10573 | tags=45%, list=27%, signal=61% |
| 79 | KEGG\_AMYOTROPHIC\_LATERAL\_SCLEROSIS\_ALS |  | 53 | -0.37 | -1.31 | 0.077 | 0.103 | 0.994 | 5121 | tags=30%, list=13%, signal=35% |
| 80 | KEGG\_INOSITOL\_PHOSPHATE\_METABOLISM |  | 54 | -0.36 | -1.28 | 0.075 | 0.122 | 0.999 | 4566 | tags=30%, list=12%, signal=33% |
| 81 | KEGG\_PRION\_DISEASES |  | 35 | -0.39 | -1.27 | 0.116 | 0.124 | 0.999 | 8542 | tags=46%, list=22%, signal=58% |
| 82 | KEGG\_ANTIGEN\_PROCESSING\_AND\_PRESENTATION |  | 81 | -0.32 | -1.24 | 0.090 | 0.148 | 1.000 | 6366 | tags=30%, list=16%, signal=35% |
| 83 | KEGG\_NITROGEN\_METABOLISM |  | 23 | -0.43 | -1.24 | 0.193 | 0.154 | 1.000 | 5225 | tags=39%, list=13%, signal=45% |
| 84 | KEGG\_LYSOSOME |  | 121 | -0.30 | -1.22 | 0.095 | 0.170 | 1.000 | 6853 | tags=31%, list=17%, signal=38% |
| 85 | KEGG\_BASAL\_CELL\_CARCINOMA |  | 55 | -0.33 | -1.18 | 0.186 | 0.208 | 1.000 | 8980 | tags=40%, list=23%, signal=52% |
| 86 | KEGG\_PROGESTERONE\_MEDIATED\_OOCYTE\_MATURATION |  | 85 | -0.31 | -1.17 | 0.173 | 0.222 | 1.000 | 6112 | tags=31%, list=16%, signal=36% |
| 87 | KEGG\_RENIN\_ANGIOTENSIN\_SYSTEM |  | 17 | -0.40 | -1.10 | 0.320 | 0.311 | 1.000 | 7289 | tags=41%, list=19%, signal=51% |
| 88 | KEGG\_PRIMARY\_IMMUNODEFICIENCY |  | 35 | -0.34 | -1.08 | 0.314 | 0.341 | 1.000 | 8779 | tags=43%, list=22%, signal=55% |
| 89 | KEGG\_CALCIUM\_SIGNALING\_PATHWAY |  | 178 | -0.25 | -1.07 | 0.240 | 0.348 | 1.000 | 7061 | tags=24%, list=18%, signal=29% |
| 90 | KEGG\_NEUROACTIVE\_LIGAND\_RECEPTOR\_INTERACTION |  | 270 | -0.22 | -1.01 | 0.430 | 0.465 | 1.000 | 7515 | tags=23%, list=19%, signal=28% |
| 91 | KEGG\_SPHINGOLIPID\_METABOLISM |  | 39 | -0.30 | -1.00 | 0.457 | 0.491 | 1.000 | 10128 | tags=46%, list=26%, signal=62% |
| 92 | KEGG\_PROXIMAL\_TUBULE\_BICARBONATE\_RECLAMATION |  | 23 | -0.33 | -0.99 | 0.459 | 0.501 | 1.000 | 8064 | tags=43%, list=21%, signal=55% |
| 93 | KEGG\_GLYCOSAMINOGLYCAN\_BIOSYNTHESIS\_KERATAN\_SULFATE |  | 15 | -0.37 | -0.97 | 0.514 | 0.543 | 1.000 | 4954 | tags=40%, list=13%, signal=46% |
| 94 | KEGG\_SYSTEMIC\_LUPUS\_ERYTHEMATOSUS |  | 135 | -0.22 | -0.91 | 0.726 | 0.660 | 1.000 | 6702 | tags=25%, list=17%, signal=30% |
| 95 | KEGG\_HEDGEHOG\_SIGNALING\_PATHWAY |  | 56 | -0.25 | -0.89 | 0.680 | 0.701 | 1.000 | 7593 | tags=25%, list=19%, signal=31% |
| 96 | KEGG\_BIOSYNTHESIS\_OF\_UNSATURATED\_FATTY\_ACIDS |  | 22 | -0.29 | -0.85 | 0.670 | 0.768 | 1.000 | 8555 | tags=45%, list=22%, signal=58% |
| 97 | KEGG\_VIBRIO\_CHOLERAE\_INFECTION |  | 54 | -0.22 | -0.77 | 0.885 | 0.893 | 1.000 | 9757 | tags=33%, list=25%, signal=44% |
| 98 | KEGG\_RIBOSOME |  | 88 | -0.15 | -0.57 | 1.000 | 0.993 | 1.000 | 12862 | tags=33%, list=33%, signal=49% |
Table: Gene sets enriched in phenotype **negative correlation with profile **[plain text format]****
