# Supplementary material for: Simultaneous Presentation of Multiple Myeloma and Lung Cancer: Case Report and Gene Bioinformatics Analysis
Source: Front Oncol. 2022 Jun 13;12:859735. doi: 10.3389/fonc.2022.859735 (PMC9235397; doi:10.3389/fonc.2022.859735)
Supplement: Supplementary file 1 [file DataSheet_1.zip › The bioinformatic analysis of MM and lung cancer supplementary materials/Enrichment analysis/MECR/GSEA_4.1.0/MM TCGA/KEGG.Gsea.1639040591792/gsea_report_for_ENSG00000116353_pos_1639040591792.html]

Report for ENSG00000116353\_pos 1639040591792 [GSEA]

| GS  follow link to MSigDB | GS DETAILS | SIZE | ES | NES | NOM p-val | FDR q-val | FWER p-val | RANK AT MAX | LEADING EDGE || 1 | KEGG\_DNA\_REPLICATION | Details ... | 36 | 0.77 | 2.32 | 0.000 | 0.000 | 0.000 | 4048 | tags=67%, list=10%, signal=74% |
| 2 | KEGG\_VALINE\_LEUCINE\_AND\_ISOLEUCINE\_DEGRADATION | Details ... | 44 | 0.74 | 2.32 | 0.000 | 0.000 | 0.000 | 4150 | tags=61%, list=11%, signal=69% |
| 3 | KEGG\_OXIDATIVE\_PHOSPHORYLATION | Details ... | 132 | 0.61 | 2.28 | 0.000 | 0.000 | 0.000 | 5685 | tags=44%, list=14%, signal=51% |
| 4 | KEGG\_AMINOACYL\_TRNA\_BIOSYNTHESIS | Details ... | 41 | 0.74 | 2.28 | 0.000 | 0.000 | 0.000 | 3110 | tags=56%, list=8%, signal=61% |
| 5 | KEGG\_BASE\_EXCISION\_REPAIR | Details ... | 35 | 0.74 | 2.27 | 0.000 | 0.000 | 0.000 | 3440 | tags=57%, list=9%, signal=63% |
| 6 | KEGG\_CITRATE\_CYCLE\_TCA\_CYCLE | Details ... | 31 | 0.74 | 2.20 | 0.000 | 0.000 | 0.000 | 5169 | tags=65%, list=13%, signal=74% |
| 7 | KEGG\_PYRIMIDINE\_METABOLISM | Details ... | 98 | 0.60 | 2.19 | 0.000 | 0.000 | 0.000 | 4463 | tags=43%, list=11%, signal=48% |
| 8 | KEGG\_PARKINSONS\_DISEASE | Details ... | 130 | 0.58 | 2.17 | 0.000 | 0.000 | 0.000 | 5588 | tags=43%, list=14%, signal=50% |
| 9 | KEGG\_PROPANOATE\_METABOLISM | Details ... | 33 | 0.72 | 2.16 | 0.000 | 0.000 | 0.000 | 6280 | tags=67%, list=16%, signal=79% |
| 10 | KEGG\_MISMATCH\_REPAIR | Details ... | 23 | 0.77 | 2.13 | 0.000 | 0.000 | 0.000 | 3869 | tags=70%, list=10%, signal=77% |
| 11 | KEGG\_PEROXISOME | Details ... | 78 | 0.59 | 2.07 | 0.000 | 0.000 | 0.002 | 4068 | tags=41%, list=10%, signal=46% |
| 12 | KEGG\_PYRUVATE\_METABOLISM | Details ... | 40 | 0.65 | 2.05 | 0.000 | 0.000 | 0.003 | 4933 | tags=50%, list=13%, signal=57% |
| 13 | KEGG\_ONE\_CARBON\_POOL\_BY\_FOLATE | Details ... | 17 | 0.80 | 2.04 | 0.000 | 0.000 | 0.006 | 3937 | tags=59%, list=10%, signal=65% |
| 14 | KEGG\_HOMOLOGOUS\_RECOMBINATION | Details ... | 28 | 0.69 | 2.02 | 0.000 | 0.000 | 0.007 | 4611 | tags=61%, list=12%, signal=69% |
| 15 | KEGG\_BUTANOATE\_METABOLISM | Details ... | 34 | 0.66 | 2.01 | 0.000 | 0.000 | 0.007 | 2541 | tags=41%, list=6%, signal=44% |
| 16 | KEGG\_SELENOAMINO\_ACID\_METABOLISM | Details ... | 26 | 0.71 | 2.01 | 0.000 | 0.000 | 0.007 | 4455 | tags=46%, list=11%, signal=52% |
| 17 | KEGG\_PURINE\_METABOLISM | Details ... | 159 | 0.52 | 2.00 | 0.000 | 0.000 | 0.007 | 7589 | tags=44%, list=19%, signal=54% |
| 18 | KEGG\_GLYCOLYSIS\_GLUCONEOGENESIS | Details ... | 62 | 0.58 | 1.95 | 0.000 | 0.000 | 0.011 | 5712 | tags=45%, list=15%, signal=53% |
| 19 | KEGG\_GLYCOSYLPHOSPHATIDYLINOSITOL\_GPI\_ANCHOR\_BIOSYNTHESIS | Details ... | 25 | 0.69 | 1.93 | 0.000 | 0.001 | 0.016 | 3963 | tags=52%, list=10%, signal=58% |
| 20 | KEGG\_HUNTINGTONS\_DISEASE | Details ... | 182 | 0.48 | 1.90 | 0.000 | 0.001 | 0.028 | 4848 | tags=37%, list=12%, signal=42% |
| 21 | KEGG\_AMINO\_SUGAR\_AND\_NUCLEOTIDE\_SUGAR\_METABOLISM |  | 44 | 0.60 | 1.89 | 0.000 | 0.001 | 0.029 | 5640 | tags=52%, list=14%, signal=61% |
| 22 | KEGG\_FATTY\_ACID\_METABOLISM |  | 42 | 0.60 | 1.88 | 0.000 | 0.001 | 0.032 | 3136 | tags=40%, list=8%, signal=44% |
| 23 | KEGG\_PORPHYRIN\_AND\_CHLOROPHYLL\_METABOLISM |  | 41 | 0.59 | 1.87 | 0.002 | 0.001 | 0.041 | 5013 | tags=34%, list=13%, signal=39% |
| 24 | KEGG\_RNA\_POLYMERASE |  | 29 | 0.65 | 1.85 | 0.002 | 0.001 | 0.050 | 4463 | tags=48%, list=11%, signal=54% |
| 25 | KEGG\_N\_GLYCAN\_BIOSYNTHESIS |  | 46 | 0.59 | 1.84 | 0.000 | 0.002 | 0.056 | 6635 | tags=50%, list=17%, signal=60% |
| 26 | KEGG\_NUCLEOTIDE\_EXCISION\_REPAIR |  | 44 | 0.58 | 1.82 | 0.000 | 0.002 | 0.085 | 6469 | tags=52%, list=16%, signal=62% |
| 27 | KEGG\_GLYOXYLATE\_AND\_DICARBOXYLATE\_METABOLISM |  | 16 | 0.73 | 1.81 | 0.002 | 0.002 | 0.089 | 2079 | tags=50%, list=5%, signal=53% |
| 28 | KEGG\_ALZHEIMERS\_DISEASE |  | 166 | 0.46 | 1.76 | 0.000 | 0.005 | 0.166 | 5783 | tags=38%, list=15%, signal=44% |
| 29 | KEGG\_ARGININE\_AND\_PROLINE\_METABOLISM |  | 54 | 0.53 | 1.75 | 0.003 | 0.005 | 0.188 | 3332 | tags=33%, list=8%, signal=36% |
| 30 | KEGG\_TERPENOID\_BACKBONE\_BIOSYNTHESIS |  | 15 | 0.68 | 1.70 | 0.008 | 0.008 | 0.304 | 2710 | tags=47%, list=7%, signal=50% |
| 31 | KEGG\_PENTOSE\_PHOSPHATE\_PATHWAY |  | 27 | 0.58 | 1.67 | 0.011 | 0.010 | 0.367 | 6752 | tags=44%, list=17%, signal=54% |
| 32 | KEGG\_LYSINE\_DEGRADATION |  | 44 | 0.52 | 1.67 | 0.008 | 0.010 | 0.378 | 2285 | tags=36%, list=6%, signal=39% |
| 33 | KEGG\_PENTOSE\_AND\_GLUCURONATE\_INTERCONVERSIONS |  | 28 | 0.57 | 1.65 | 0.010 | 0.012 | 0.447 | 4484 | tags=29%, list=11%, signal=32% |
| 34 | KEGG\_TRYPTOPHAN\_METABOLISM |  | 40 | 0.52 | 1.64 | 0.006 | 0.013 | 0.471 | 2104 | tags=28%, list=5%, signal=29% |
| 35 | KEGG\_HISTIDINE\_METABOLISM |  | 29 | 0.56 | 1.64 | 0.009 | 0.013 | 0.478 | 2196 | tags=28%, list=6%, signal=29% |
| 36 | KEGG\_CELL\_CYCLE |  | 125 | 0.44 | 1.62 | 0.000 | 0.015 | 0.539 | 4849 | tags=42%, list=12%, signal=48% |
| 37 | KEGG\_CYSTEINE\_AND\_METHIONINE\_METABOLISM |  | 34 | 0.54 | 1.60 | 0.012 | 0.018 | 0.616 | 5012 | tags=38%, list=13%, signal=44% |
| 38 | KEGG\_TYROSINE\_METABOLISM |  | 42 | 0.49 | 1.56 | 0.020 | 0.026 | 0.757 | 5867 | tags=33%, list=15%, signal=39% |
| 39 | KEGG\_ALANINE\_ASPARTATE\_AND\_GLUTAMATE\_METABOLISM |  | 32 | 0.52 | 1.55 | 0.019 | 0.027 | 0.779 | 6973 | tags=44%, list=18%, signal=53% |
| 40 | KEGG\_PROTEASOME |  | 46 | 0.49 | 1.55 | 0.013 | 0.026 | 0.782 | 5741 | tags=43%, list=15%, signal=51% |
| 41 | KEGG\_LINOLEIC\_ACID\_METABOLISM |  | 29 | 0.53 | 1.54 | 0.020 | 0.028 | 0.820 | 6015 | tags=34%, list=15%, signal=41% |
| 42 | KEGG\_BETA\_ALANINE\_METABOLISM |  | 22 | 0.57 | 1.51 | 0.028 | 0.035 | 0.887 | 2104 | tags=36%, list=5%, signal=38% |
| 43 | KEGG\_RNA\_DEGRADATION |  | 59 | 0.43 | 1.46 | 0.034 | 0.055 | 0.973 | 4805 | tags=39%, list=12%, signal=44% |
| 44 | KEGG\_FRUCTOSE\_AND\_MANNOSE\_METABOLISM |  | 34 | 0.48 | 1.43 | 0.045 | 0.070 | 0.993 | 2238 | tags=29%, list=6%, signal=31% |
| 45 | KEGG\_ASCORBATE\_AND\_ALDARATE\_METABOLISM |  | 25 | 0.51 | 1.42 | 0.079 | 0.072 | 0.994 | 7430 | tags=28%, list=19%, signal=35% |
| 46 | KEGG\_STEROID\_HORMONE\_BIOSYNTHESIS |  | 55 | 0.43 | 1.41 | 0.052 | 0.075 | 0.995 | 5164 | tags=25%, list=13%, signal=29% |
| 47 | KEGG\_GLYCEROLIPID\_METABOLISM |  | 49 | 0.43 | 1.41 | 0.040 | 0.077 | 0.997 | 3663 | tags=22%, list=9%, signal=25% |
| 48 | KEGG\_GALACTOSE\_METABOLISM |  | 26 | 0.50 | 1.40 | 0.058 | 0.077 | 0.997 | 6912 | tags=42%, list=18%, signal=51% |
| 49 | KEGG\_GLYCOSAMINOGLYCAN\_BIOSYNTHESIS\_HEPARAN\_SULFATE |  | 26 | 0.50 | 1.40 | 0.073 | 0.079 | 0.998 | 7139 | tags=42%, list=18%, signal=52% |
| 50 | KEGG\_GLUTATHIONE\_METABOLISM |  | 49 | 0.40 | 1.30 | 0.109 | 0.152 | 1.000 | 3088 | tags=20%, list=8%, signal=22% |
| 51 | KEGG\_BASAL\_TRANSCRIPTION\_FACTORS |  | 35 | 0.42 | 1.28 | 0.138 | 0.165 | 1.000 | 5103 | tags=37%, list=13%, signal=43% |
| 52 | KEGG\_OOCYTE\_MEIOSIS |  | 113 | 0.35 | 1.28 | 0.062 | 0.164 | 1.000 | 3642 | tags=26%, list=9%, signal=28% |
| 53 | KEGG\_STEROID\_BIOSYNTHESIS |  | 17 | 0.49 | 1.27 | 0.192 | 0.169 | 1.000 | 6029 | tags=41%, list=15%, signal=49% |
| 54 | KEGG\_DRUG\_METABOLISM\_CYTOCHROME\_P450 |  | 71 | 0.36 | 1.24 | 0.125 | 0.202 | 1.000 | 9802 | tags=32%, list=25%, signal=43% |
| 55 | KEGG\_DRUG\_METABOLISM\_OTHER\_ENZYMES |  | 51 | 0.39 | 1.24 | 0.139 | 0.202 | 1.000 | 9249 | tags=37%, list=24%, signal=49% |
| 56 | KEGG\_STARCH\_AND\_SUCROSE\_METABOLISM |  | 52 | 0.38 | 1.24 | 0.142 | 0.202 | 1.000 | 5640 | tags=23%, list=14%, signal=27% |
| 57 | KEGG\_GLYCOSAMINOGLYCAN\_BIOSYNTHESIS\_CHONDROITIN\_SULFATE |  | 22 | 0.44 | 1.22 | 0.197 | 0.224 | 1.000 | 5285 | tags=36%, list=13%, signal=42% |
| 58 | KEGG\_RETINOL\_METABOLISM |  | 64 | 0.35 | 1.21 | 0.167 | 0.232 | 1.000 | 5256 | tags=20%, list=13%, signal=23% |
| 59 | KEGG\_PANTOTHENATE\_AND\_COA\_BIOSYNTHESIS |  | 16 | 0.47 | 1.16 | 0.272 | 0.293 | 1.000 | 3406 | tags=25%, list=9%, signal=27% |
| 60 | KEGG\_CARDIAC\_MUSCLE\_CONTRACTION |  | 79 | 0.33 | 1.16 | 0.191 | 0.296 | 1.000 | 6549 | tags=28%, list=17%, signal=33% |
| 61 | KEGG\_GLYCEROPHOSPHOLIPID\_METABOLISM |  | 77 | 0.32 | 1.13 | 0.272 | 0.341 | 1.000 | 3525 | tags=21%, list=9%, signal=23% |
| 62 | KEGG\_NICOTINATE\_AND\_NICOTINAMIDE\_METABOLISM |  | 24 | 0.40 | 1.11 | 0.303 | 0.364 | 1.000 | 3639 | tags=25%, list=9%, signal=28% |
| 63 | KEGG\_ABC\_TRANSPORTERS |  | 44 | 0.34 | 1.10 | 0.284 | 0.372 | 1.000 | 3293 | tags=18%, list=8%, signal=20% |
| 64 | KEGG\_GLYCOSAMINOGLYCAN\_DEGRADATION |  | 21 | 0.41 | 1.09 | 0.352 | 0.381 | 1.000 | 6912 | tags=38%, list=18%, signal=46% |
| 65 | KEGG\_RIBOFLAVIN\_METABOLISM |  | 16 | 0.43 | 1.07 | 0.346 | 0.408 | 1.000 | 2427 | tags=25%, list=6%, signal=27% |
| 66 | KEGG\_OTHER\_GLYCAN\_DEGRADATION |  | 16 | 0.43 | 1.07 | 0.368 | 0.418 | 1.000 | 8319 | tags=50%, list=21%, signal=63% |
| 67 | KEGG\_PROTEIN\_EXPORT |  | 24 | 0.37 | 1.06 | 0.363 | 0.426 | 1.000 | 7631 | tags=46%, list=19%, signal=57% |
| 68 | KEGG\_METABOLISM\_OF\_XENOBIOTICS\_BY\_CYTOCHROME\_P450 |  | 69 | 0.29 | 0.98 | 0.471 | 0.571 | 1.000 | 9802 | tags=32%, list=25%, signal=42% |
| 69 | KEGG\_GLYCOSPHINGOLIPID\_BIOSYNTHESIS\_GANGLIO\_SERIES |  | 15 | 0.40 | 0.98 | 0.485 | 0.569 | 1.000 | 6912 | tags=53%, list=18%, signal=65% |
| 70 | KEGG\_ETHER\_LIPID\_METABOLISM |  | 33 | 0.32 | 0.98 | 0.491 | 0.567 | 1.000 | 5302 | tags=24%, list=13%, signal=28% |
| 71 | KEGG\_CYTOSOLIC\_DNA\_SENSING\_PATHWAY |  | 55 | 0.30 | 0.98 | 0.502 | 0.564 | 1.000 | 4004 | tags=18%, list=10%, signal=20% |
| 72 | KEGG\_GLYCINE\_SERINE\_AND\_THREONINE\_METABOLISM |  | 31 | 0.33 | 0.97 | 0.496 | 0.577 | 1.000 | 5931 | tags=26%, list=15%, signal=30% |
| 73 | KEGG\_PRIMARY\_BILE\_ACID\_BIOSYNTHESIS |  | 16 | 0.37 | 0.95 | 0.531 | 0.599 | 1.000 | 5585 | tags=31%, list=14%, signal=36% |
| 74 | KEGG\_ALPHA\_LINOLENIC\_ACID\_METABOLISM |  | 19 | 0.34 | 0.90 | 0.586 | 0.695 | 1.000 | 5302 | tags=16%, list=13%, signal=18% |
| 75 | KEGG\_PHENYLALANINE\_METABOLISM |  | 18 | 0.32 | 0.83 | 0.711 | 0.835 | 1.000 | 4129 | tags=17%, list=10%, signal=19% |
| 76 | KEGG\_MATURITY\_ONSET\_DIABETES\_OF\_THE\_YOUNG |  | 25 | 0.29 | 0.82 | 0.730 | 0.832 | 1.000 | 6464 | tags=20%, list=16%, signal=24% |
| 77 | KEGG\_ARACHIDONIC\_ACID\_METABOLISM |  | 58 | 0.24 | 0.79 | 0.826 | 0.872 | 1.000 | 6015 | tags=19%, list=15%, signal=22% |
| 78 | KEGG\_TASTE\_TRANSDUCTION |  | 51 | 0.24 | 0.78 | 0.835 | 0.875 | 1.000 | 6208 | tags=22%, list=16%, signal=26% |
| 79 | KEGG\_OLFACTORY\_TRANSDUCTION |  | 386 | 0.18 | 0.76 | 0.989 | 0.891 | 1.000 | 15455 | tags=36%, list=39%, signal=59% |
| 80 | KEGG\_O\_GLYCAN\_BIOSYNTHESIS |  | 30 | 0.26 | 0.73 | 0.872 | 0.915 | 1.000 | 8898 | tags=37%, list=23%, signal=47% |
Table: Gene sets enriched in phenotype **positive correlation with profile **[plain text format]****
