# Supplementary material for: Simultaneous Presentation of Multiple Myeloma and Lung Cancer: Case Report and Gene Bioinformatics Analysis
Source: Front Oncol. 2022 Jun 13;12:859735. doi: 10.3389/fonc.2022.859735 (PMC9235397; doi:10.3389/fonc.2022.859735)
Supplement: Supplementary file 1 [file DataSheet_1.zip › The bioinformatic analysis of MM and lung cancer supplementary materials/Enrichment analysis/MECR/GSEA_4.1.0/MM TCGA/MM-KEGG_pos_bubble.pdf]

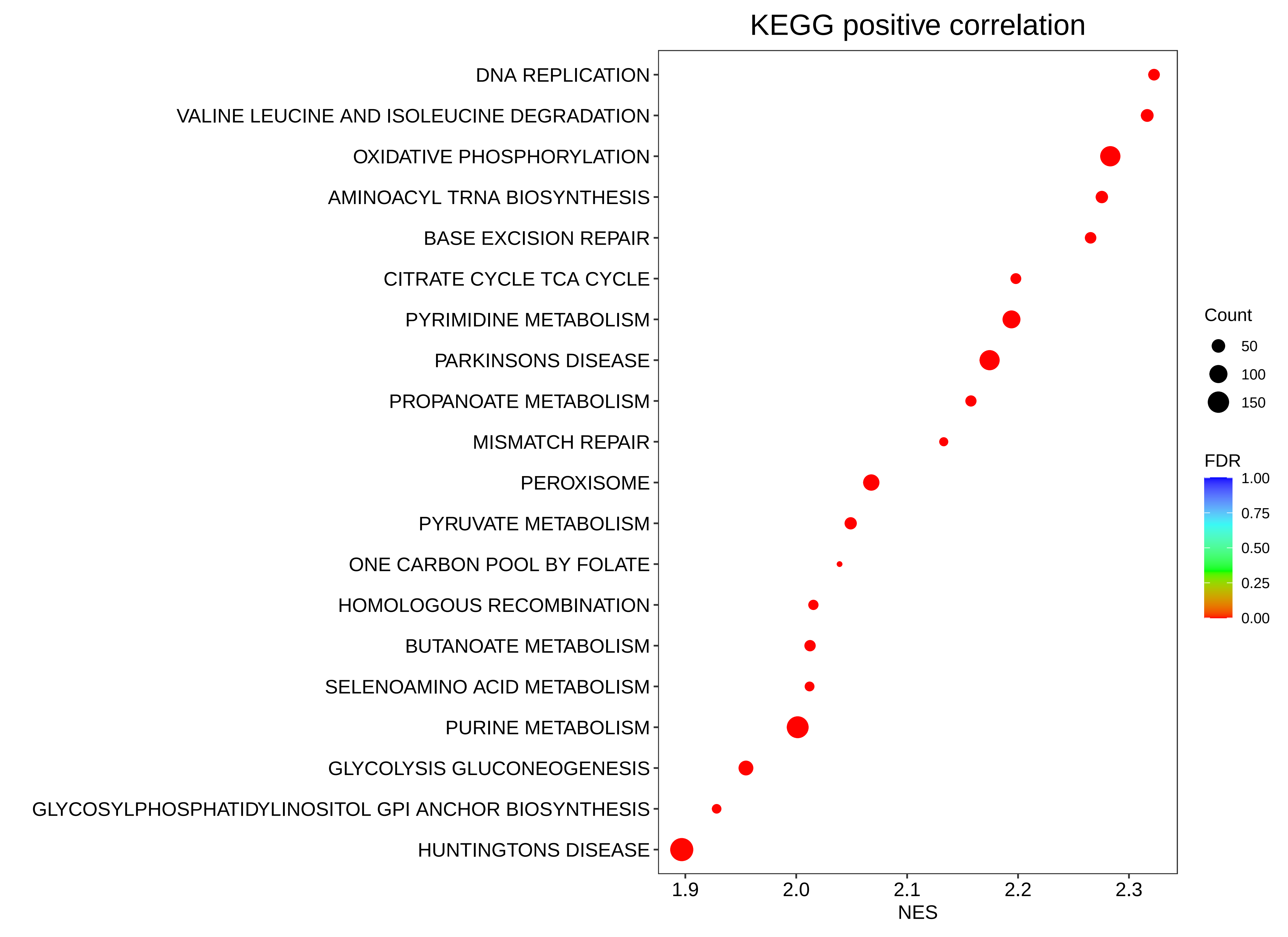
KEGG positive correlation
DNA REPLICATION
VALINE LEUCINE AND ISOLEUCINE DEGRADATION
OXIDATIVE PHOSPHORYLATION
AMINOACYL TRNA BIOSYNTHESIS
BASE EXCISION REPAIR
CITRATE CYCLE TCA CYCLE
PYRIMIDINE METABOLISM
PARKINSONS DISEASE
PROPANOATE METABOLISM
MISMATCH REPAIR
PEROXISOME
PYRUVATE METABOLISM
ONE CARBON POOL BY FOLATE
HOMOLOGOUS RECOMBINATION
BUTANOATE METABOLISM
SELENOAMINO ACID METABOLISM
PURINE METABOLISM
GLYCOLYSIS GLUCONEOGENESIS
GLYCOSYLPHOSPHATIDYLINOSITOL GPI ANCHOR BIOSYNTHESIS
HUNTINGTONS DISEASE
1.9
2.0
2.1
2.2
2.3
NES
Count
50
100
150
FDR
1.00
0.75
0.50
0.25
0.00
