# Supplementary material for: Simultaneous Presentation of Multiple Myeloma and Lung Cancer: Case Report and Gene Bioinformatics Analysis
Source: Front Oncol. 2022 Jun 13;12:859735. doi: 10.3389/fonc.2022.859735 (PMC9235397; doi:10.3389/fonc.2022.859735)
Supplement: Supplementary file 1 [file DataSheet_1.zip › The bioinformatic analysis of MM and lung cancer supplementary materials/Survival analysis/GEO/MECR-surival-GSE24080.pdf]

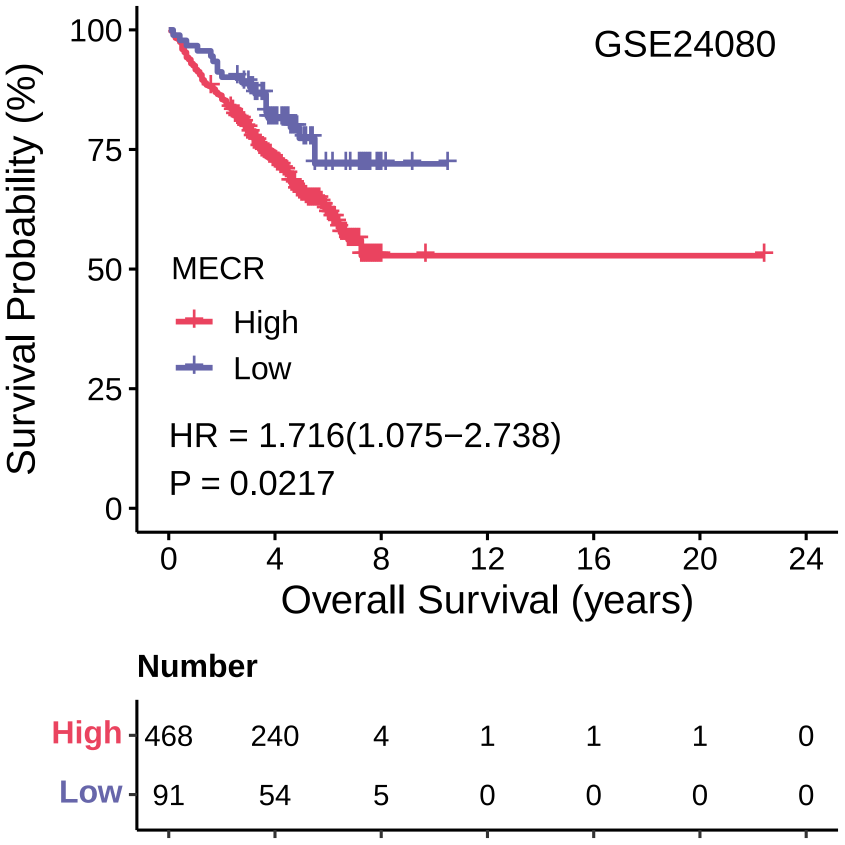
GSE24080
Survival Probability (%)
100
75
50
25
0
MECR
High
Low
HR = 1.716(1.075−2.738)
P = 0.0217
0
4
8
12
16
20
24
Overall Survival (years)
Number
High 468 240 4 1 1 1 0
Low 91 54 5 0 0 0 0
