# Supplementary material for: Simultaneous Presentation of Multiple Myeloma and Lung Cancer: Case Report and Gene Bioinformatics Analysis
Source: Front Oncol. 2022 Jun 13;12:859735. doi: 10.3389/fonc.2022.859735 (PMC9235397; doi:10.3389/fonc.2022.859735)
Supplement: Supplementary file 1 [file DataSheet_1.zip › The bioinformatic analysis of MM and lung cancer supplementary materials/Survival analysis/GEO/MECR-surival-GSE2658.pdf]

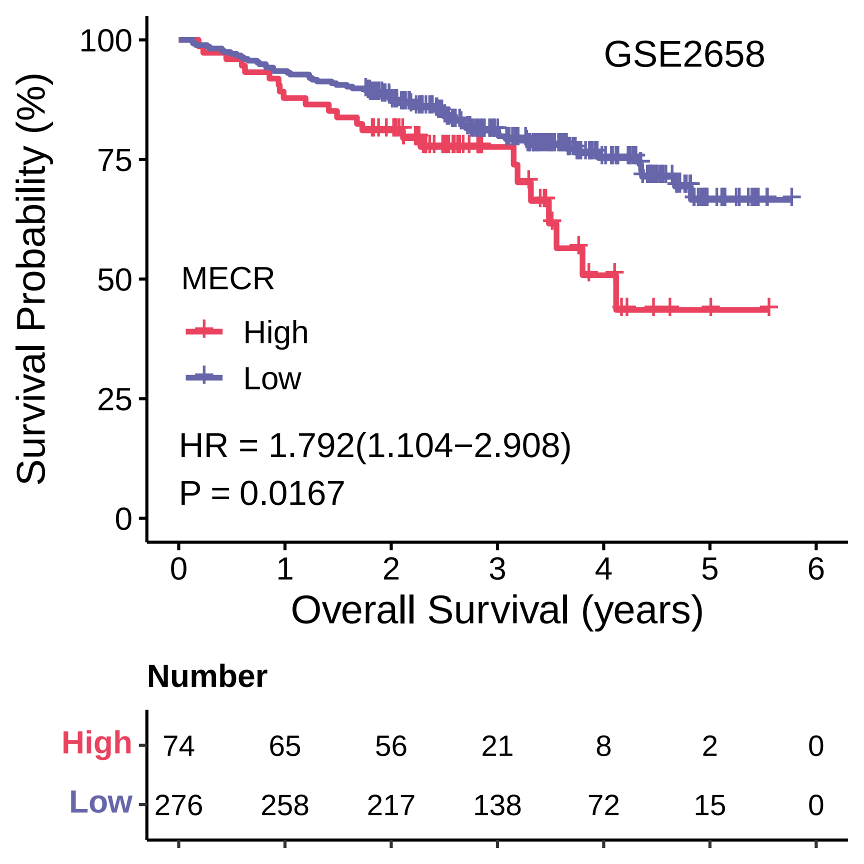

GSE2658
Survival Probability (%)
100
75
50
25
0
MECR
High
Low
HR = 1.792(1.104−2.908)
P = 0.0167
0
1
2
3
4
5
6
Overall Survival (years)
Number
High
74
65
56
21
8
2
0
Low
276
258
217
138
72
15
0
