# Supplementary material for: Simultaneous Presentation of Multiple Myeloma and Lung Cancer: Case Report and Gene Bioinformatics Analysis
Source: Front Oncol. 2022 Jun 13;12:859735. doi: 10.3389/fonc.2022.859735 (PMC9235397; doi:10.3389/fonc.2022.859735)
Supplement: Supplementary file 1 [file DataSheet_1.zip › The bioinformatic analysis of MM and lung cancer supplementary materials/Survival analysis/TCGA/LUAD TCGA.pdf]

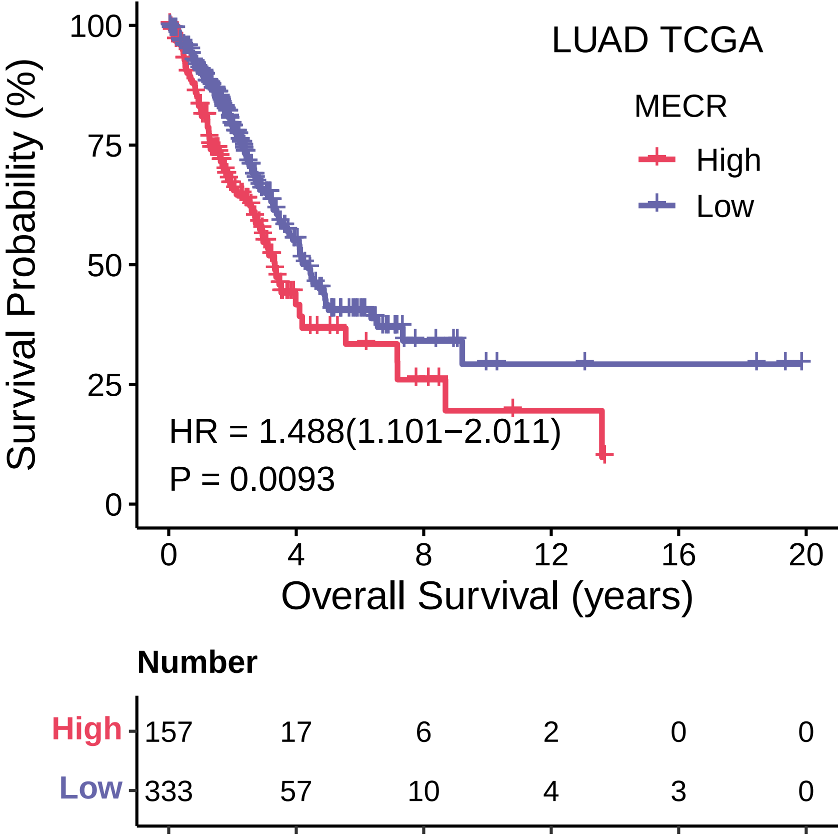
LUAD TCGA
MECR
High
Low
Survival Probability (%)
100
75
50
25
0
HR = 1.488(1.101−2.011)
P = 0.0093
0
4
8
12
16
20
Overall Survival (years)
Number
High 157 17 6 2 0 0
Low 333 57 10 4 3 0
