# Supplementary material for: Simultaneous Presentation of Multiple Myeloma and Lung Cancer: Case Report and Gene Bioinformatics Analysis
Source: Front Oncol. 2022 Jun 13;12:859735. doi: 10.3389/fonc.2022.859735 (PMC9235397; doi:10.3389/fonc.2022.859735)
Supplement: Supplementary file 1 [file DataSheet_1.zip › The bioinformatic analysis of MM and lung cancer supplementary materials/Survival analysis/TCGA/LUSC TCGA.pdf]

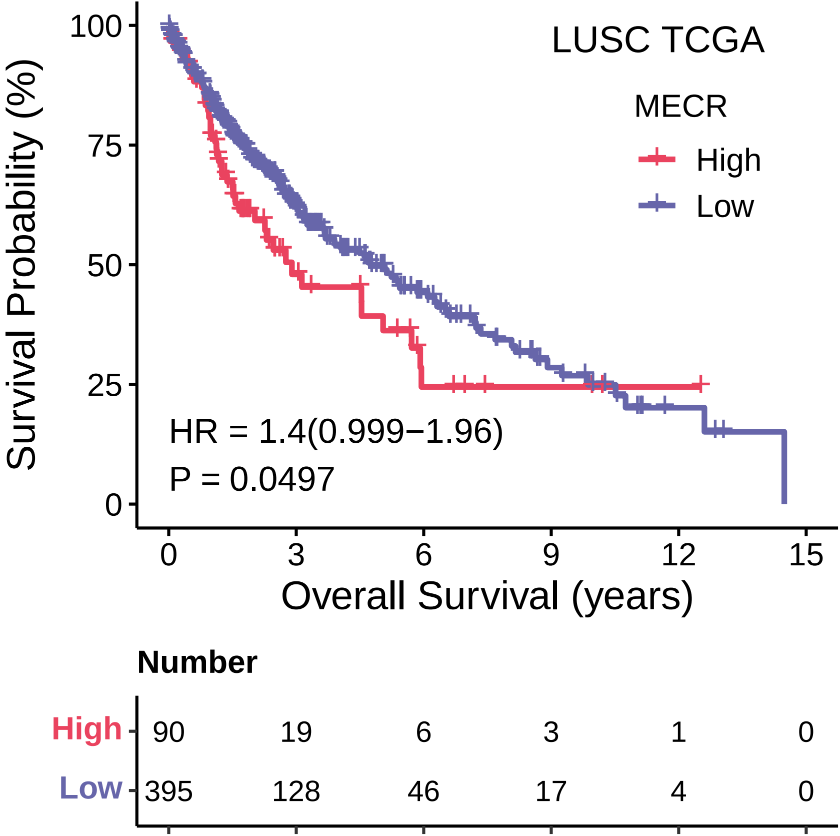
LUSC TCGA
MECR
High
Low
HR = 1.4(0.999−1.96)
P = 0.0497
Survival Probability (%)
100
75
50
25
0
0
3
6
9
12
15
Overall Survival (years)
Number
High 90 19 6 3 1 0
Low 395 128 46 17 4 0
